# Health system performance in Iran: a systematic analysis for the Global Burden of Disease Study 2019

**DOI:** 10.1016/S0140-6736(21)02751-3

**Published:** 2022-04-23

**Authors:** GBD 2019 Iran Collaborators, Farshad Farzadfar, Mohsen Naghavi, Sadaf G Sepanlou, Sahar Saeedi Moghaddam, William James Dangel, Nicole Davis Weaver, Arya Aminorroaya, Sina Azadnajafabad, Sogol Koolaji, Esmaeil Mohammadi, Negar Rezaei, Jaffar Abbas, Behzad Abbasi, Mitra Abbasifard, Mohsen Abbasi-Kangevari, Zeinab Abbasi-Kangevari, Hedayat Abbastabar, Amir Abdoli, Mohammad Abdollahi, Sina Abdollahzade, Hassan Abolhassani, Zahra Abrehdari-Tafreshi, Soodabeh Aghababaei, Bahman Ahadinezhad, Ali Ahmadi, Sepideh Ahmadi, Hamid Ahmadieh, Mohammad Esmaeil Akbari, Yousef Alimohamadi, Vahid Alipour, Hesam Alizade, Saba Alvand, Saeed Amini, Sohrab Amiri, Ali Arash Anoushirvani, Fereshteh Ansari, Jalal Arabloo, Morteza Arab-Zozani, Zahra Aryan, Armin Aryannejad, Mehran Asadi-Aliabadi, Ali A Asadi-Pooya, Zatollah Asemi, Samaneh Asgari, Saeed Asgary, Babak Asghari, Mohammad Asghari Jafarabadi, Elham Ashrafi, Zahra Atafar, Seyyed Shamsadin Athari, Abolfazl Avan, Abbas Azadmehr, Hiva Azami, Mohammadreza Azangou-Khyavy, Samad Azari, Amirhossein Azari Jafari, Ghasem Azarian, Alireza Badirzadeh, Elham Bahrami, Mohammad Amin Bahrami, Nastaran Barati, Mohsen Bayati, Gholamreza Bazmandegan, Masoud Behzadifar, Ali Bijani, Somayeh Bohlouli, Shiva Borzouei, Parnaz Daneshpajouhnejad, Abdollah Dargahi, Ahmad Daryani, Jalal Davoodi Lahijan, Mojtaba Didehdar, Shirin Djalalinia, Saeid Doaei, Fariba Dorostkar, Leila Doshmangir, Mohammadreza Edraki, Amir Emami, Babak Eshrati, Sharareh Eskandarieh, Firooz Esmaeilzadeh, Shahriar Faghani, Mahdi Fakhar, Hamid Reza Farpour, Hossein Farrokhpour, Majid Fasihi Harandi, Mohammad Fereidouni, Masoud Foroutan, Mansour Ghafourifard, Azin Ghamari, Seyyed-Hadi Ghamari, Ahmad Ghashghaee, Fariba Ghassemi, Ali Gholami, Asadollah Gholamian, Abdolmajid Gholizadeh, Salime Goharinezhad, Pouya Goleij, Mostafa Hadei, Nima Hafezi-Nejad, Sanam Hariri, Edris Hasanpoor, Hossein Hassanian-Moghaddam, Soheil Hassanipour, Hadi Hassankhani, Mohammad Heidari, Reza Heidari-Soureshjani, Mohammad Hoseini, Mohammad-Salar Hosseini, Mostafa Hosseini, Seyed Kianoosh Hosseini, Ali Hosseinzadeh, Mehdi Hosseinzadeh, Soodabeh Hoveidamanesh, Pooya Iranpour, Seyed Sina Naghibi Irvani, Jalil Jaafari, Roxana Jabbarinejad, Morteza Jafarinia, Hamed Jafari-Vayghan, Mohammad Ali Jahani, Nader Jahanmehr, Mahsa Jalili, Roksana Janghorban, Fatemeh Javanmardi, Farahnaz Joukar, Ali Kabir, Leila R Kalankesh, Rohollah Kalhor, Zahra Kamiab, Naser Kamyari, Behzad Karami Matin, Amirali Karimi, Salah Eddin Karimi, Ali Kazemi Karyani, Leila Keikavoosi-Arani, Maryam Keramati, Pedram Keshavarz, Mohammad Keykhaei, Ali Khaleghi, Mohammad Khammarnia, Javad Khanali, Maryam Khayamzadeh, Sajad Khosravi, Mina Khosravifar, Omid Khosravizadeh, Neda Kianipour, Ali-Asghar Kolahi, Amirhosein Maali, Mokhtar Mahdavi Mahdavi, Afshin Maleki, Mohammad-Reza Malekpour, Kamyar Mansori, Borhan Mansouri, Mohammad Ali Mansournia, Mohammad Reza Maracy, Abdoljalal Marjani, Sahar Masoudi, Seyedeh Zahra Masoumi, Hossein Masoumi-Asl, Mahsa Mayeli, Entezar Mehrabi Nasab, Fereshteh Mehri, Mohammad Miri, Seyyedmohammadsadeq Mirmoeeni, Hamed Mirzaei, Maryam Mirzaei, Roya Mirzaei, Ashraf Mohamadkhani, Heidar Mohammadi, Seyyede Momeneh Mohammadi, Shadieh Mohammadi, Abdollah Mohammadian-Hafshejani, Noushin Mohammadifard, Reza Mohammadpourhodki, Mohammad Mohseni, Amin Mokari, Sara Momtazmanesh, Abdolvahab Moradi, Masoud Moradi, Yousef Moradi, Mohammad Moradi-Joo, Farhad Moradpour, Maliheh Moradzadeh, Rahmatollah Moradzadeh, Abbas Mosapour, Shandiz Moslehi, Simin Mouodi, Mehdi Naderi, Homa Naderifar, Zhila Najafpour, Javad Nazari, Seyed Aria Nejadghaderi, Leila Nemati-Anaraki, Amin Reza Nikpoor, Marzieh Nojomi, Maryam Noori, Hasti Nouraei, Ali Nowroozi, Morteza Oladnabi, Fatemeh Pashazadeh Kan, Majid Pirestani, Meghdad Pirsaheb, Mohammadreza Pourahmadi, Hadis Pourchamani, Hadi Pourjafar, Akram Pourshams, Mohammad Rabiee, Navid Rabiee, Alireza Rafiei, Sima Rafiei, Fakher Rahim, Amir Masoud Rahmani, Sina Rashedi, Vahid Rashedi, Amirfarzan Rashidi, Mahsa Rashidi, Mohammad-Mahdi Rashidi, Ramin Ravangard, Reza Rawassizadeh, Iman Razeghian-Jahromi, Mohammad Sadegh Razeghinia, Sofia B Redford, Maryam Rezaei, Nazila Rezaei, Nima Rezaei, Saeid Rezaei, Hossein Rezaei Aliabadi, Mohsen Rezaeian, Mohammad Sadegh Rezai, Aziz Rezapour, Hossein Rezazadeh, Sahba Rezazadeh-Khadem, Morteza Rostamian, Ehsan Sadeghi, Erfan Sadeghi, Masoumeh Sadeghi, Reihaneh Sadeghian, Saeid Sadeghian, Hamid Safarpour, Mahdi Safdarian, Sare Safi, Maryam Sahebazzamani, Amirhossein Sahebkar, Mohammad Ali Sahraian, Sarvenaz Salahi, Payman Salamati, Hossein Samadi Kafil, Yaser Sarikhani, Maryam Sarkhosh, Arash Sarveazad, Maryam Seyed-Nezhad, Omid Shafaat, Zahra Shaghaghi, Saeed Shahabi, Sarvenaz Shahin, Elaheh Shaker, Saeed Shakiba, MohammadBagher Shamsi, Erfan Shamsoddin, Kiomars Sharafi, Sakineh Sharifian, Maryam Shaygan, Abbas Sheikhtaheri, Amir Shiani, Kiarash Shirbandi, Reza Shirkoohi, Parnian Shobeiri, Azad Shokri, Soraya Siabani, Ali Reza Sima, Ahmad Sofi-Mahmudi, Amin Soheili, Shahin Soltani, Mohammad Sadegh Soltani-Zangbar, Moslem Soofi, Seidamir Pasha Tabaeian, Mohammadreza Tabary, Alireza Tahamtan, Majid Taheri, Amir Taherkhani, Masih Tajdini, Hamed Tavolinejad, Arash Tehrani-Banihashemi, Amir Tiyuri, Seyed Abolfazl Tohidast, Alireza Vakilian, Sahel Valadan Tahbaz, Bay Vo, Seyed Hossein Yahyazadeh Jabbari, Vahid Yazdi-Feyzabadi, Zabihollah Yousefi, Taraneh Yousefinezhadi, Mazyar Zahir, Telma Zahirian Moghadam, Maryam Zamanian, Hamed Zandian, Alireza Zangeneh, Hadi Zarafshan, Fariba Zare, Ali Zare Dehnavi, Kourosh Zarea, Ahmad Zarei, Zahra Zareshahrabadi, Arash Ziapour, Sina Zoghi, Nizal Sarrafzadegan, Vafa Rahimi-Movaghar, Hamid Reza Jamshidi, Ali H Mokdad, Simon I Hay, Christopher J L Murray, Ardeshir Khosravi, Maziar Moradi-Lakeh, Mohsen Asadi-Lari, Reza Malekzadeh, Bagher Larijani

## Abstract

**Background:**

Better evaluation of existing health programmes, appropriate policy making against emerging health threats, and reducing inequalities in Iran rely on a comprehensive national and subnational breakdown of the burden of diseases, injuries, and risk factors.

**Methods:**

In this systematic analysis, we present the national and subnational estimates of the burden of disease in Iran using the Global Burden of Disease Study 2019. We report trends in demographics, all-cause and cause-specific mortality, as well as years of life lost (YLLs), years lived with disability (YLDs), and disability-adjusted life-years (DALYs) caused by major diseases and risk factors. A multi-intervention segmented-regression model was used to explore the overall impact of health sector changes and sanctions. For this analysis, we used a variety of sources and reports, including vital registration, census, and survey data to provide estimates of mortality and morbidity at the national and subnational level in Iran.

**Findings:**

Iran, which had 84·3 million inhabitants in 2019, had a life expectancy of 79·6 years (95% uncertainty interval 79·2–79·9) in female individuals and 76·1 (75·6–76·5) in male individuals, an increase compared with 1990. The number of DALYs remained stable and reached 19·8 million (17·3–22·6) in 2019, of which 78·1% were caused by non-communicable diseases (NCDs) compared with 43·0% in 1990. During the study period, age-standardised DALY rates and YLL rates decreased considerably; however, YLDs remained nearly constant. The share of age-standardised YLDs contributing to the DALY rate steadily increased to 44·5% by 2019. With regard to the DALY rates of different provinces, inequalities were decreasing. From 1990 to 2019, although the number of DALYs attributed to all risk factors decreased by 16·8%, deaths attributable to all risk factors substantially grew by 43·8%. The regression results revealed a significant negative association between sanctions and health status.

**Interpretation:**

The Iranian health-care system is encountering NCDs as its new challenge, which necessitates a coordinated multisectoral approach. Although the Iranian health-care system has been successful to some extent in controlling mortality, it has overlooked the burden of morbidity and need for rehabilitation. We did not capture alleviation of the burden of diseases in Iran following the 2004 and 2014 health sector reforms; however, the sanctions were associated with deaths of Iranians caused by NCDs.

**Funding:**

Bill & Melinda Gates Foundation.

## Introduction

During the past four decades, Iran has experienced substantial turmoil in its economy and international policy. After the 1979 revolution, Iran was afflicted with the longest war in the 20th century with its neighbour Iraq.^1^ After the end of the war in 1988, Iran entered an era of massive construction and investment in its health and non-health infrastructures, with an increase in gross domestic product (GDP) per person.^2^ The economic and social development included, but was not limited to, improvement in literacy, urbanisation, and investments in the transport and food industries. In the health sector, policies were designed and implemented in the four main domains of primary care, secondary and tertiary care, training health-care professionals, and research to reach universal health coverage (UHC);^3^ nevertheless, international sanctions against Iran in 2011 imposed some restrictions on these efforts.^4^ On the path towards UHC, Iran has undergone several major transformations, including expanding the primary health-care (PHC) system^5^ and integrating the Ministry of Health with Medical Education in the 1980s,^1^ the 2004 Universal Rural Health Insurance,^1^, ^6^, ^7^ and the 2014 Health Transformation Plan;^1^ however, existing evidence indicates unequal improvements in the distribution of specific health indicators across 31 provinces (appendix p 2).^8^, ^9^

Assessment of the performance of the health-care system is necessary for evaluating the success or failure of previous policies, assessing needs, setting priorities, and directing future evidence-based policies; nonetheless, this initiative requires a comprehensive national and subnational breakdown of the burden of diseases, injuries, and risk factors.^10^, ^11^, ^12^, ^13^ In this study, we aimed to provide this breakdown in Iran, using the Global Burden of Diseases, Injuries, and Risk Factors Study (GBD) 2019. This report is the first on the burden of diseases and risk factors that encompasses 30-year trends in all subnational records, to the best of our knowledge.

**Research in context**

**Evidence before this study**

We searched online databases including PubMed and Google Scholar for Farsi and English language articles using keywords including “Iran”, “burden of disease”, “subnational level”, “epidemiological trends”, “mortality”, “morbidity”, “health system performance”, and “sanction”. In 2019, a review paper was published in *The Lancet* entitled *Iran in Transition*, which reported a comprehensive history of Iran and its health-care system and presented the main turning points in the health-care system and infrastructure in Iran. The paper discussed the current health status and future directions on the basis of results and information from various sources. Additionally, several national and subnational studies on the burden of certain diseases and risk factors have been done and reported in Iran at certain points during the past three decades.

**Added value of this study**

To the best of our knowledge, the Global Burden of Diseases, Injuries, and Risk Factors Study 2019 is the most comprehensive, systematic, and concerted effort so far that reports life expectancy, mortality, and disability from 369 causes, and the burden attributable to 87 risk factors at the national and subnational level in Iran from 1990 to 2019. This study includes special consideration of the Iranian health-care system's performance and action plans.

**Implications of all the available evidence**

This study shows a demographic transition leading to population growth and ageing, and an increase in life expectancy, along with an epidemiological transition. Evidence shows that the expanded health-care system has been quite successful in halting communicable, maternal, and neonatal diseases. Economic and social development in Iran is linked to the decreased burden of certain conditions, such as injuries and certain environmental risk factors; however, policies to control non-communicable diseases, such as substance-use disorders and mental disorders, and certain risk factors, including metabolic risk factors, have not been successful. Given the future burden of COVID-19 in the coming years, and the probable continuation of sanctions and their impact on Iran's economy and the function of the health-care system, policies should focus on maintaining the infrastructure and the financing of the health-care system, human resources, and equality and quality of health-care services, and setting priorities favouring fatal causes in vulnerable populations with restricted access to health care.

## Methods

### Overview

GBD 2019 provided estimates of the burden of 369 diseases and injuries and 87 risk factors for 204 countries and territories from 1990 to 2019, with subnational estimates for 21 countries, including Iran. The detailed estimation framework of GBD 2019 has been discussed previously.^10^, ^12^, ^14^, ^15^ All measures are reported in age-standardised rates derived from the GBD standard population structure that were developed as part of the GBD framework. Our article complies with the Guidelines for Accurate and Transparent Health Estimates Reporting (GATHER). Our full GATHER checklist is available in the appendix (p 25). All data sources used in this analysis and related code can be found on the Global Health Data Exchange.^16^, ^17^ Additional results from GBD 2019 can be viewed with our data visualisation tools.

### Subnational estimation

We investigated subnational inequalities through comparing age-standardised rates. GBD 2019 used several databases from Iran, which have been summarised (appendix section 2). The data for national and subnational estimates of health in Iran were retrieved from various sources, including censuses and vital registration. A decomposition analysis was used to identify the cause-specific contributions to changes in life expectancy.^18^ Detailed steps to achieve the decomposition analysis are included in the appendix (p 21). There were sharp declines in life expectancy because of earthquakes in 1990 (Gilan, Iran, and Zanjan, Iran) and 2003 (Kerman, Iran). Therefore, 1991 was chosen as the reference year for life expectancy and all-cause estimates. We reported 95% uncertainty intervals (UIs) for each measure that were generated using the 25th and 975th ordered 1000 draws of the posterior distribution.^10^ To investigate the impact of different health sector reforms (details on the history of major health policies in Iran are provided in the appendix, section 1) and the economic crisis caused by sanctions on the Iranian health-care system, we utilised two methods, the annual percentage change in age-standardised deaths and a multi-intervention segmented regression model to explore the changes in non-communicable diseases (NCDs) and under-5 mortality rate (U5MR).^19^ The U5MR incorporated death rates as the dependent variable and the two major changes (health sector changes and the economic crisis caused by sanctions) as independent variables adjusted to 2021 health expenditure per person (extracted from The World Bank).^20^ The segmented regression model is a method for statistically modelling interrupted time-series data, and to draw more formal inferences regarding the effect of an intervention or event on the response variable. Therefore, by using a multi-intervention segmented regression model, we can specify more than one change point. In the multi-intervention segmented regression model, level (which is the value of the series at the beginning of a given time interval) and trend (which is the rate of change of a measure) in the preintervention segment and changes in level and trend after the interventions were estimated. Additional details on the methods are presented (appendix section 2).

### Role of the funding source

The funder of the study had no role in study design, data collection, data analysis, data interpretation, or writing of the report.

## Results

### Outlines of population structure and health status

**Figure 1:**
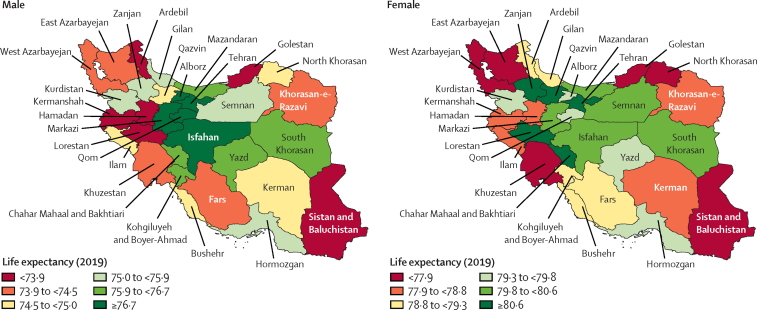
Life expectancy at birth (years) among provinces of Iran in male and female individuals, 2019

Iran is having a late demographic transition.^1^ Iran's total population increased from 58·5 million (95% UI 53·3–63·5) in 1990 to 84·3 million (77·3–91·9) in 2019 because of high fertility rates in the early 1990s (appendix pp 33, 56). From the late 1990s to 2019, the total fertility rate decreased steadily from 4·2 (95% UI 3·7–4·6) to 1·8 (1·4–2·4), which resulted in an apparent ageing of the population in this period (appendix pp 34, 62). In 2019, life expectancy for female individuals in Iran was 79·6 years (95% UI 79·2–79·9) and 76·1 years (75·6–76·5) for male individuals. From 1991 to 2019, life expectancy at birth increased from 68·9 (95% UI 68·2–69·6) to 77·8 (77·5–78·0), with diverse provincial patterns by sex (figure 1; appendix pp 36, 58). The absolute difference between the highest and the lowest life expectancy across provinces was 12·5 years in 1991, which declined to 8·2 years in 2019, indicating a convergence (appendix p 37). The ratio of the highest-to-lowest age-standardised death rates in provinces remained almost constant from 1991 to 2019 (2·0 *vs* 1·9; appendix p 41). More information about the results can be found in the appendix (sections 3, 4).

**Figure 2:**
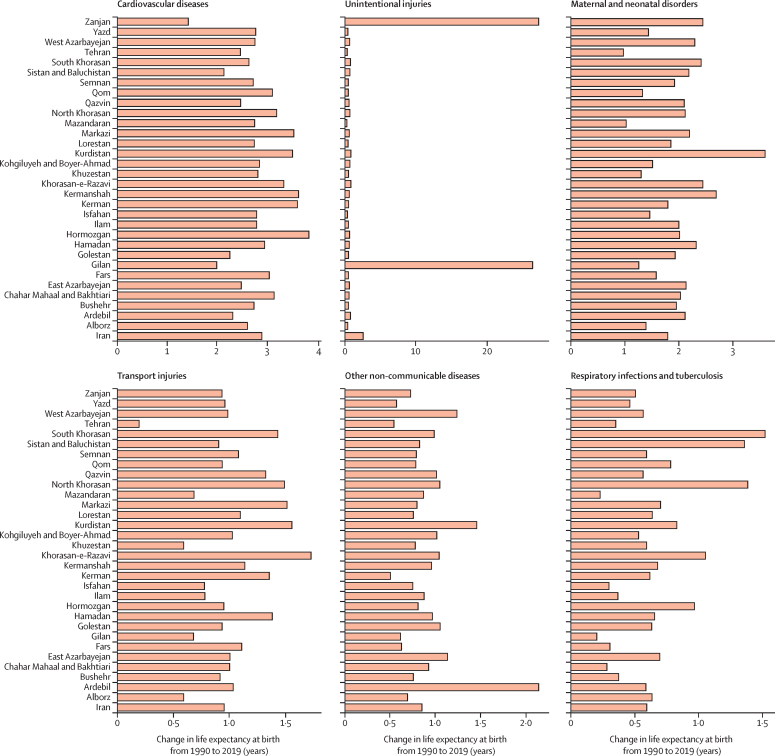

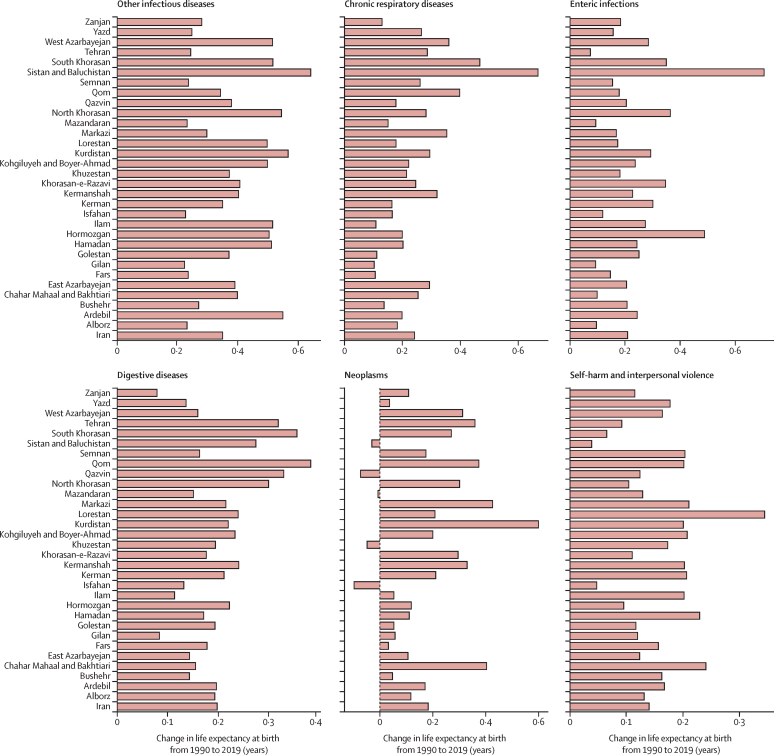

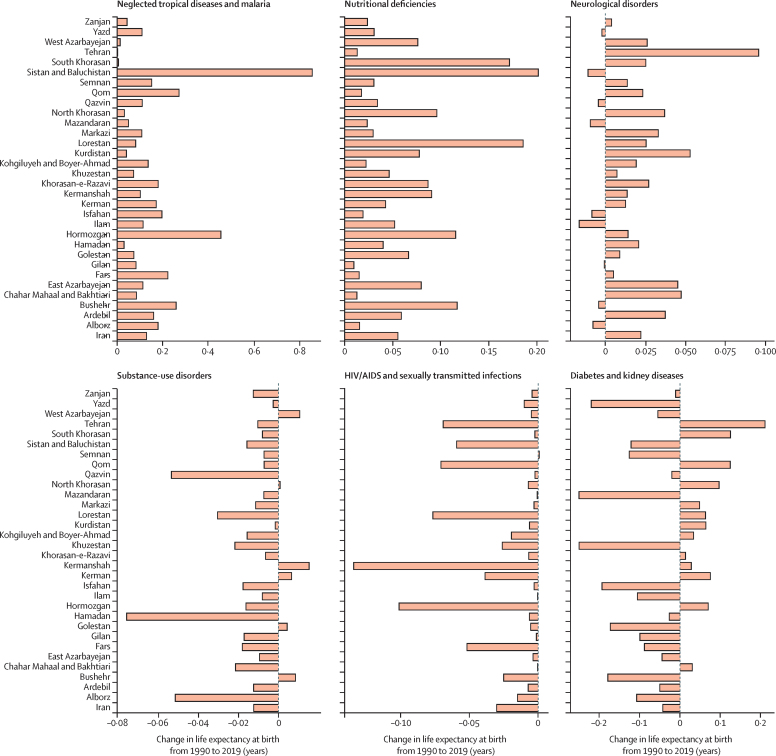
Breakdown of life expectancy in Iran and provinces between 1990 and 2019, for both sexes

All provinces observed increases in life expectancy (appendix p 37). The decline in age-standardised death rates of almost all causes improved life expectancy during the study period. The causes whose reductions made the largest contributions to improvement in life expectancy were cardiovascular diseases (2·9 years), unintentional injuries (2·6 years), maternal and neonatal diseases (1·8 years), and transport injuries (0·9 years). The alleviation of most infectious diseases substantially contributed to the improvement in life expectancy. Conversely, very few conditions quite minimally counteracted the progress in life expectancy, including diabetes and kidney diseases, HIV/AIDS and sexually transmitted infections, substance-use disorders, musculoskeletal disorders, and mental disorders. Improvement in substance-use disorders led to increased life expectancy in six provinces, and neoplasms were associated with life expectancy decrease in five provinces (figure 2; appendix p 63).

In addition to the demographic transition, an epidemiological transition is ongoing, with a shift in burden from injuries and communicable, maternal, neonatal, and nutritional diseases (CMNNDs) towards NCDs. In 2019, the total number of DALYs reached 19·8 million (95% UI 17·3–22·6; appendix p 125). In 2019, 78·1% of disability-adjusted life years (DALYs) were caused by non-communicable diseases compared with 43·0% in 1990. The epidemiological transition level, defined as the ratio of the all-age DALY rate caused by CMNNDs to the DALY rate caused by NCDs and injuries together, ranged between 8·0% and 103·2% in 1990, and from 5·1% to 23·6% in 2019 among provinces (appendix p 38). Over the period studied, the number of DALYs attributed to all risk factors showed a decline of 16·8%, although deaths attributable to risk factors increased substantially by 43·8%. From 1991 to 2019, all-age numbers and age-standardised rates of DALYs and years of life lost (YLLs) due to all causes considerably decreased by more than 35%; however, estimates of years lived with disability (YLDs) were constant, underscoring the crucial role of infrastructure for managing morbidity (appendix p 40). In 2019, the share of age-standardised YLDs contributing to DALYs steadily increased to 44·5%.

### Public health, infectious diseases, and the issue of emerging diseases

During the study period, reductions in major infectious diseases including malaria, measles, and diphtheria were among the major drivers of the nearly 95% of the decrease in the burden of communicable diseases based on the percentage change in the number of all-age DALYs. Within almost every subcategory of communicable diseases, a remarkable decrease in the DALY rate (range 93·4–59·0%) was detected, except for HIV/AIDS and sexually transmitted diseases, which had a substantial 197·6% increase. Among CMNNDs, the HIV/AIDS DALY rate increased by almost 13 times and climbed to the 6th rank (appendix pp 51, 52). Although the burden of tuberculosis had a decreasing pattern (−64·0% in the number of DALYs), this halted in the mid-2000s with the large surge of HIV/AIDS in Iran. The burden of tuberculosis almost reached a steady state in the past two decades, highlighting the importance of the re-emerging condition of tuberculosis as the major comorbidity of HIV/AIDS.

Considering the emergence of HIV/AIDS in Iran, the age-standardised DALY rate of this disease increased in most provinces from 1990 to 2019; however, this rise was more pronounced in Sistan and Baluchistan (23-fold increase), Lorestan (20-fold increase), and Hormozgan (18-fold increase). The highest DALY rates were noted in Kermanshah (299·4 per 100 000 people, 95% UI 229·9–388·8) and the lowest DALY rates were recorded in Semnan (3·5 per 100 000, 2·5–4·8) in 2019. The increasing difference between HIV/AIDS rates of DALYs among provinces in the past three decades was remarkable, with the highest-to-lowest ratio being 17·3 in 1990 compared with 85·8 in 2019 (appendix p 152).

Given the high social stigma of HIV/AIDs in Iran,^21^ its burden has been substantially underestimated and control measures have been neglected, and there is also a pattern of behaviours associated with a higher risk of HIV acquisition coupled with a lower perception of individual risk in the Iranian population.^22^ Injection-drug use and substantially increasing sexual transmission of HIV since the early 1990s are the main drivers of the increasing burden of HIV/AIDS.^23^, ^24^, ^25^ Iran started triangular clinic services in the late 1990s to address the growing epidemic of HIV/AIDS. These clinics provided services for people at high risk of HIV infection and those who were infected in three domains of HIV units, addiction units, and sexually-transmitted-disease units. In the early 2000s, the health-care system integrated these clinics with the PHC system to effectively address the epidemic.^26^ However, shortages in several aspects of this policy resulted in the country's surge in HIV/AIDs cases.^27^

Improved sanitation and nutrition preceded profound improvements in the reduction of communicable diseases in Iran.^28^ Vaccination programmes have been largely successful in reducing the burden of bacterial and viral infections. Hepatitis B vaccination was launched in 1993 in Iran, requiring that all children born after this time receive vaccination for hepatitis B. Increasing vaccination coverage was followed by a sharp decline in cirrhosis caused by chronic hepatitis B since 2001.^29^ Another example is the success of the mumps-measles-rubella mass vaccination that reached more than 96·4% in 2008.^30^ Certain diseases have been systematically controlled with concerted efforts; for example, malaria (*p vivax*) has been largely controlled, with Iran in the WHO pre-elimination phase, with a directly observed therapy method in the PHC network.^1^

### Primary health-care network and improvements in maternal and child health

One of the main drivers of the remarkable reduction in the burden of CMNNDs was improvements in the care of neonatal disorders, the leading cause of DALYs in 1990. By 2019, neonatal disorders had descended to the fourth rank among all causes of DALYs in Iran, decreasing by 81·5%. Furthermore, DALYs caused by maternal disorders substantially decreased by 68·0%. The top cause of DALYs among neonatal disorders was neonatal preterm birth in both 1990 and 2019. Among maternal disorders, the leading causes of DALYs were maternal hypertensive disorders in 1990 and indirect maternal deaths in 2019 (appendix p 130).

The distribution of the burden of maternal and neonatal disorders varied at the subnational level. The decreasing trend of age-standardised deaths caused by neonatal disorders had a converging pattern among provinces in the past three decades, indicating reduced differences between provinces, with the highest age-standardised deaths in Sistan and Baluchistan (19·3, 95% UI 15·6–23·7) and the lowest in Gilan (5·5, 4·1–7·2) in 2019. The decreasing trend in age-standardised death rates caused by maternal disorders had a converging pattern, with the highest rates in female individuals in Sistan and Baluchistan (1·9, 1·4–2·4) and the lowest in Tehran (0·2, 0·1–0·3) in 2019 (appendix pp 818, 871).

Nutritional deficiencies, another major contributor to CMNNDs, showed a 59·4% decrease in the all-age number of DALYs in the study period reaching 123 514 (95% UI 81 350–174 299] in 2019. Dietary iron deficiency and protein-energy malnutrition were the top two causes of nutritional deficiencies in both 1990 and 2019; nevertheless, the DALYs associated with both these nutritional deficiencies declined in their number by 46·7% for dietary iron deficiency and 77·9% for protein-energy malnutrition. Comparing the age-standardised DALY rates at the subnational level showed that provinces had a converging declining pattern, because the highest-to-lowest ratio of age-standardised DALY rates was 3·6 in 1990 against 1·8 in 2019. The Sistan and Baluchistan province had the highest rates of nutritional deficiencies in both 1990 and 2019 (appendix p 152).

The remarkable improvement in maternal, child, and nutritional health status occurred mainly because of improved health literacy, especially among women, urbanisation, and advances in the economic status of the country during specific periods.^1^ Iran implemented many strategies to advance maternal and child health. One of the earliest was the PHC system implemented in the 1980s, which provided health-care services, child nutritional support, better access to clean water and sanitation, and national vaccination programmes in deprived areas of the country.^1^, ^31^, ^32^, ^33^, ^34^ Furthermore, the Ministry of Health took responsibility for educating health-care personnel in 1986, which resulted in a considerable increase in the number of medical students and universities, and the number and density of hospitals, outpatient clinics, and health-care personnel.^1^, ^35^

### Environmental and ecological changes as arising major risks in Iran

Ambient particulate matter pollution, unsafe water, and unsafe sanitation were among the major environmental risk factors with strong impacts on health status. In 2019, ambient particulate matter pollution was the fifth leading contributor to DALYs (137 832, 95% UI 113 167–163 498) and deaths (4913, 3967–5931) in Tehran province, which has a large population. Ambient particulate matter showed a 46·7% increase in attributable DALYs and a 109·6% increase in deaths in this province (appendix p 971). In Tehran province, air pollution is estimated to have contributed to deaths from cardiovascular diseases (6·2%), diabetes and kidney diseases (2·1%), and chronic respiratory diseases (0·6%). The substantial adverse effect of air pollution on health and economics^36^, ^37^ necessitates more rigorous legislative measures, as well as applying novel approaches, such as spatiotemporal screening tools, for the collection and analysis of environmental data.^38^

The number of all-age all-cause DALYs attributable to unsafe water sources declined to 99 237 (95% UI 51 328–151 535) in 2019; the corresponding number for unsafe sanitation was 27 018 (15 392–43 370). With a converging pattern at the subnational level, the highest age-standardised DALY rates attributable to unsafe water sources occurred in Sistan and Baluchistan (208·6, 113·8–305·3) and the lowest in Tehran (94·4, 43·8–152·5) in 2019. The highest DALY rates attributable to unsafe sanitation were seen in Sistan and Baluchistan (89·5, 48·7–140·1) and the lowest in Tehran (15·0, 6·4–29·6) in 2019, with a converging decreasing pattern among all provinces, showing the lessening of inequalities between provinces (Table 1, Table 2; appendix p 971).

**Table 1:** All-age DALYs and deaths attributable to risk factors for all causes, and age-standardised rates (per 100 000 people) in 1990 and 2019, by sex

		**1990**				**2019**			
		DALYs		Deaths		DALYs		Deaths	
		Number	Rate	Number	Rate	Number	Rate	Number	Rate
**All risk factors**									
Both sexes		9 243 048 (8 190 548 to 10 576 260)	19 189·8 (17 788·5 to 20 901·8)	169 011 (156 653 to 184 419)	610·3 (574·5 to 644·2)	7 686 074 (7 031 340 to 8 350 066)	10 286·0 (9481·9 to 11 112·5)	243 052 (231 290 to 254 791)	378·8 (359·0 to 397·0)
Female		3 840 371 (3 369 936 to 4 422 415)	16 154·7 (14 864·1 to 17 724·0)	69 264 (63 613 to 76 225)	529·9 (493·8 to 567·4)	3 139 891 (2 835 769 to 3 450 775)	8730·3 (7945·0 to 9542·4)	104 477 (97 544 to 110 417)	344·1 (320·5 to 363·9)
Male		5 402 677 (4 787 352 to 6 184 535)	21 986·2 (20 298·9 to 24 063·5)	99 747 (92 022 to 109 374)	684·2 (641·8 to 728·4)	4 546 183 (4 169 464 to 4 933 000)	11 839·9 (10 930·0 to 12 807·4)	138 575 (131 522 to 146 256)	414·9 (393·0 to 438·0)
**Environmental and occupational risks**									
Overall									
	Both sexes	2 456 746 (2 149 920 to 2 863 083)	5803·2 (5262·2 to 6431·0)	51 471 (46 733 to 57 175)	200·2 (180·4 to 221·9)	2 228 640 (1 996 190 to 2 470 780)	2943·2 (2652·5 to 3249·9)	75 484 (68 142 to 82 329)	116·6 (104·6 to 128·0)
	Female	941 039 (802 887 to 1 106 149)	4484·1 (4025·4 to 5009·9)	20 072 (17 989 to 22 517)	168·2 (149·9 to 187·8)	812 357 (722 472 to 907 316)	2276·5 (2029·0 to 2527·3)	31 179 (27 624 to 34 423)	102·2 (90·0 to 113·4)
	Male	1 515 707 (1 332 710 to 1 768 490)	7024·7 (6341·4 to 7865·0)	31 399 (28 434 to 35 302)	229·8 (206·2 to 256·6)	1 416 282 (1 269 888 to 1 576 426)	3606·9 (3254·8 to 3992·0)	44 304 (40 099 to 48 555)	131·4 (117·5 to 144·3)
Unsafe water, sanitation, and handwashing									
	Both sexes	471 647 (345 822 to 645 280)	618·5 (460·3 to 830·8)	5164 (3648 to 7147)	9·4 (6·7 to 12·7)	127 854 (82 215 to 179 366)	169·1 (109·4 to 235·3)	1040 (655 to 1485)	1·7 (1·0 to 2·4)
	Female	222 041 (154 394 to 309 403)	595·1 (421·0 to 809·1)	2434 (1614 to 3427)	9·3 (5·7 to 14·5)	60 569 (38 195 to 85 854)	163·4 (104·1 to 229·0)	470 (267 to 762)	1·6 (0·9 to 2·6)
	Male	249 606 (181 133 to 352 778)	639·6 (475·2 to 874·1)	2730 (1912 to 3923)	9·4 (6·7 to 12·8)	67 285 (44 040 to 94 129)	174·7 (114·9 to 242·7)	569 (363 to 838)	1·7 (1·1 to 2·6)
Air pollution									
	Both sexes	1 323 247 (1 143 429 to 1 563 808)	3198·7 (2826·7 to 3625·6)	30 463 (26 960 to 34 732)	116·4 (102·5 to 131·3)	1 190 330 (1 042 443 to 1 349 001)	1603·0 (1404·7 to 1813·8)	43 203 (37 478 to 48 717)	65·5 (56·5 to 74·0)
	Female	540 695 (459 745 to 643 633)	2686·9 (2370·6 to 3058·1)	12 304 (10 831 to 14 017)	100·4 (86·7 to 114·6)	489 525 (425 747 to 556 544)	1364·8 (1186·0 to 1549·4)	18 483 (15 972 to 20 998)	58·7 (50·6 to 67·0)
	Male	782 552 (666 167 to 932 336)	3664·0 (3208·5 to 4195·8)	18 159 (15 903 to 20 825)	130·9 (114·4 to 148·3)	700 805 (610 488 to 789 456)	1841·6 (1607·2 to 2070·8)	24 720 (21 429 to 27 990)	72·4 (62·5 to 82·0)
Non-optimal temperature									
	Both sexes	301 596 (231 651 to 397 680)	931·8 (784·4 to 1104·7)	9641 (8119 to 11 428)	48·3 (40·2 to 56·9)	348 406 (300 862 to 398 741)	510·2 (443·1 to 581·6)	19 997 (17 225 to 22 753)	32·5 (28·0 to 37·0)
	Female	147 601 (116 551 to 190 597)	877·7 (749·9 to 1021·6)	4481 (3833 to 5230)	45·4 (38·1 to 53·4)	159 324 (139 092 to 181 434)	479·7 (417·9 to 546·7)	9445 (8153 to 10 812)	31·9 (27·4 to 36·6)
	Male	153 995 (112 461 to 210 598)	975·4 (800·4 to 1186·5)	5159 (4248 to 6264)	50·7 (41·9 to 59·8)	189 082 (160 424 to 221 661)	543·0 (463·4 to 631·0)	10 553 (9035 to 12 063)	33·2 (28·5 to 37·9)
Other environmental risks									
	Both sexes	266 794 (199 508 to 337 867)	903·5 (665·9 to 1159·9)	8319 (6007 to 10 930)	39·3 (27·7 to 52·6)	331 163 (236 863 to 430 616)	468·5 (334·1 to 610·3)	15 318 (10 786 to 20 591)	24·6 (17·2 to 33·3)
	Female	85 838 (59 562 to 113 860)	626·8 (430·2 to 838·7)	2744 (1818 to 3791)	29·4 (19·2 to 41·5)	114 358 (76 637 to 157 236)	338·1 (229·0 to 462·3)	5697 (3731 to 8098)	19·4 (12·8 to 27·7)
	Male	180 956 (137 123 to 226 850)	1158·0 (870·0 to 1464·9)	5575 (4134 to 7195)	48·6 (35·1 to 64)	216 805 (158 961 to 279 515)	599·9 (444·3 to 768·4)	9620 (7000 to 12 666)	29·8 (21·7 to 39·2)
Occupational risks									
	Both sexes	288 541 (234 081 to 349 374)	695·1 (565·1 to 838·1)	3378 (2760 to 4089)	9·4 (7·7 to 11·2)	412 274 (327 667 to 496 133)	442·7 (353·2 to 530·6)	4398 (3786 to 5106)	5·5 (4·7 to 6·4)
	Female	19 391 (13 902 to 26 020)	105·0 (76·2 to 142·6)	113 (88 to 144)	0·9 (0·7 to 1·3)	50 459 (36 995 to 65 779)	108·3 (80·5 to 139·7)	251 (202 to 313)	0·7 (0·6 to 0·9)
	Male	269 150 (217 370 to 326 600)	1257·9 (1023·7 to 1510·8)	3264 (2652 to 3972)	17·6 (14·5 to 21·2)	361 815 (290 663 to 434 273)	770·7 (621·0 to 918·6)	4147 (3540 to 4871)	10·1 (8·6 to 11·9)
**Behavioural risks**									
Overall									
	Both sexes	7 544 925 (6 544 210 to 8 830 843)	13 448·6 (12 215·6 to 14 960·7)	121 737 (110 103 to 135 956)	360·2 (332·8 to 387·5)	4 418 672 (4 016 468 to 4 823 401)	5822·1 (5310·2 to 6336·0)	125 194 (114 285 to 135 637)	189·5 (172·2 to 205·9)
	Female	3 108 223 (2 655 801 to 3 665 420)	10 746·2 (9598·4 to 12 131·3)	46 731 (41 505 to 52 618)	276·3 (248·1 to 302·8)	1 604 156 (1 412 276 to 1 799 568)	4369·7 (3889·5 to 4862·5)	44 848 (39 353 to 49 440)	144·3 (125·9 to 160·0)
	Male	4 436 702 (3 842 634 to 5 182 530)	15 972·9 (14 507·1 to 17 811·2)	75 006 (67 712 to 84 089)	441·6 (408·2 to 479·6)	2 814 515 (2 581 287 to 3 063 686)	7269·7 (6664·8 to 7909·3)	80 346 (74 301 to 86 800)	235·0 (216·5 to 254·5)
Child and maternal malnutrition									
	Both sexes	5 497 708 (4 494 154 to 6 691 000)	6473·8 (5314·1 to 7849·0)	59 628 (48 241 to 72 978)	69·9 (56·8 to 85·3)	852 138 (712 936 to 1 011 606)	1237·9 (1030·3 to 1478·3)	7199 (5817 to 8869)	11·1 (8·9 to 13·6)
	Female	2 433 922 (1 986 834 to 2 961 190)	5903·1 (4836·5 to 7176·3)	26 223 (21 235 to 32 090)	63 (51·2 to 76·7)	390 403 (327 344 to 461 032)	1151·1 (964·2 to 1362·8)	3124 (2519 to 3817)	9·9 (8·0 to 12·0)
	Male	3 063 786 (2 473 525 to 3 781 655)	7013·7 (5678·2 to 8651·2)	33 405 (26 659 to 41 349)	76·4 (61·1 to 94·4)	461 736 (379 751 to 556 357)	1319·1 (1078·5 to 1594·2)	4075 (3218 to 5054)	12·2 (9·6 to 15·1)
Tobacco use									
	Both sexes	1 052 471 (944 533 to 1 167 768)	3314·5 (3033·5 to 3599·9)	30 711 (27 976 to 33 274)	120·3 (110·8 to 130·1)	1 481 171 (1 360 879 to 1 619 268)	1914·4 (1766·8 to 2083·2)	48 564 (45 319 to 52 061)	70·4 (65·6 to 75·8)
	Female	249 542 (201 341 to 300 925)	1474·0 (1230·1 to 1728·7)	6511 (5432 to 7629)	51·4 (43·2 to 60·2)	323 767 (275 393 to 376 425)	847·4 (718·5 to 980·3)	10 319 (8909 to 11 711)	30·5 (26·2 to 34·7)
	Male	802 928 (723 767 to 882 646)	5044·3 (4601·4 to 5478·1)	24 201 (22 108 to 26 394)	188·9 (173·6 to 205·0)	1 157 404 (1 067 589 to 1 259 569)	2983·7 (2757·0 to 3239·4)	38 245 (35 862 to 41 046)	110·1 (102·9 to 118·3)
Alcohol use									
	Both sexes	50 140 (38 540 to 64 703)	125·1 (96·7 to 159·7)	837 (627 to 1138)	2·8 (2·1 to 3·7)	139 833 (109 766 to 175 225)	158·9 (124·4 to 200·3)	2976 (2244 to 3996)	3·8 (2·9 to 5·2)
	Female	10 373 (8118 to 12 941)	53·3 (42·8 to 65·6)	154 (121 to 193)	1·2 (0·9 to 1·6)	22 610 (17 489 to 28 833)	52·9 (41·0 to 67·9)	416 (302 to 585)	1·2 (0·8 to 1·7)
	Male	39 767 (29 902 to 52 702)	193 (146·1 to 254·5)	683 (492 to 964)	4·3 (3·2 to 5·9)	117 223 (91 263 to 148 028)	263·3 (204·7 to 334·1)	2560 (1922 to 3433)	6·5 (4·8 to 8·8)
Drug use									
	Both sexes	171 635 (141 604 to 206 825)	370·7 (312·8 to 438·1)	1962 (1707 to 2243)	5·5 (4·7 to 6·4)	444 340 (376 720 to 521 935)	475·2 (404·5 to 557·0)	5980 (5470 to 6569)	7·0 (6·4 to 7·8)
	Female	49 753 (39 195 to 62 668)	209·4 (167·9 to 258·3)	434 (371 to 513)	2·4 (2·0 to 2·9)	103 135 (83 667 to 126 514)	224·3 (183·1 to 274·3)	1080 (906 to 1306)	2·6 (2·1 to 3·1)
	Male	121 882 (101 042 to 145 354)	523·3 (441·0 to 611·6)	1528 (1307 to 1768)	8·4 (7·1 to 9·9)	341 205 (291 777 to 396 501)	720·1 (617·1 to 832·9)	4900 (4485 to 5358)	11·5 (10·4 to 12·6)
Dietary risks									
	Both sexes	1 004 233 (840 580 to 1 166 327)	3788·0 (3155·0 to 4390·3)	37 273 (30 928 to 43 438)	182·8 (151·6 to 212·7)	1 473 685 (1 208 551 to 1 739 207)	1986·8 (1627·6 to 2346·4)	64 424 (52 234 to 76 488)	100·0 (80·6 to 118·5)
	Female	361 178 (302 769 to 415 925)	2987·3 (2507·6 to 3434·1)	14 351 (11 947 to 16 553)	155·5 (128·1 to 180·1)	573 055 (473 687 to 663 363)	1624·1 (1333·1 to 1884·7)	27 392 (22 210 to 32 074)	90·5 (73·3 to 106·1)
	Male	643 055 (533 177 to 760 324)	4513·9 (3749·6 to 5353·1)	22 922 (18 893 to 27 346)	207·6 (172·1 to 247·8)	900 631 (728 882 to 1 092 221)	2352·3 (1899·7 to 2848·0)	37 032 (29 787 to 44 723)	110·0 (88·6 to 133·0)
Intimate partner violence									
	Female	59 495 (13 668 to 110 038)	261·8 (57·3 to 493·7)	152 (99 to 207)	0·7 (0·4 to 0·9)	126 718 (30 299 to 237 808)	276·0 (66·6 to 515·7)	342 (241 to 437)	0·8 (0·6 to 1·0)
Childhood sexual abuse and bullying									
	Both sexes	66 060 (26 079 to 125 638)	104·8 (42·7 to 197·9)	17 (2 to 39)	0 (0 to 0·1)	103 460 (40 147 to 202 055)	124·5 (49·6 to 238·7)	27 (4 to 63)	0 (0 to 0·1)
	Female	21 225 (8009 to 43 058)	68·2 (27·8 to 135·7)	2 (0 to 4)	0	34 360 (13 058 to 71 940)	84·6 (32·2 to 174·2)	2 (0 to 4)	0
	Male	44 836 (17 800 to 83 645)	140·0 (57·2 to 258·0)	16 (2 to 36)	0·1 (0 to 0·2)	69 100 (27 055 to 130 477)	162·9 (66·3 to 300·0)	26 (3 to 59)	0·1 (0 to 0·1)
Unsafe sex									
	Both sexes	19 940 (14 986 to 24 076)	57·4 (43·3 to 68·7)	435 (317 to 524)	1·7 (1·2 to 2·1)	50 884 (40 983 to 63 816)	56·2 (45·7 to 69·4)	1154 (914 to 1422)	1·5 (1·1 to 1·8)
	Female	17 693 (13 007 to 21 037)	108·1 (80·1 to 129·3)	425 (304 to 513)	3·3 (2·4 to 4·2)	38 152 (31 239 to 46 660)	87·2 (71·7 to 104·7)	959 (755 to 1127)	2·5 (2·0 to 2·9)
	Male	2247 (1506 to 3270)	9·4 (6·3 to 13·8)	10 (5 to 15)	0·1 (0 to 0·1)	12 732 (8309 to 18 379)	25·3 (17·0 to 36·4)	195 (111 to 304)	0·4 (0·2 to 0·6)
Low physical activity									
	Both sexes	128 650 (59 165 to 242 900)	585·5 (286·3 to 1031·7)	5490 (2548 to 10 053)	34·4 (17·3 to 58)	298 331 (164 409 to 506 086)	433·3 (243·2 to 722·0)	14 446 (7800 to 23 762)	24·0 (13·1 to 38·6)
	Female	55 765 (28 698 to 97 035)	542·6 (283·1 to 909·9)	2543 (1288 to 4314)	33·2 (17·6 to 54·1)	147 116 (85 245 to 233 837)	436·8 (258·7 to 688·1)	7252 (4150 to 11 381)	25·1 (14·4 to 39·2)
	Male	72 884 (29 488 to 149 871)	621·7 (275·5 to 1177·9)	2947 (1214 to 5808)	35·3 (16·3 to 62·0)	151 215 (75 241 to 271 785)	431·4 (221·8 to 748·3)	7194 (3601 to 12 516)	23·0 (11·6 to 39·3)
**Metabolic risks**									
Overall									
	Both sexes	2 264 132 (2 065 676 to 2 470 091)	8570·8 (7795·7 to 9358·4)	79 124 (72 173 to 86 453)	402·9 (364·8 to 442·6)	4 490 268 (4 092 380 to 4 912 190)	6175·4 (5643·6 to 6733·7)	179 522 (165 891 to 193 135)	283·5 (260·8 to 305·9)
	Female	937 318 (853 989 to 1 031 052)	7632·3 (6916·2 to 8393·5)	34 069 (30 915 to 37 400)	375·8 (338·2 to 414·2)	2 013 201 (1 820 664 to 2 216 579)	5727·1 (5202·3 to 6270·4)	84 222 (76 996 to 91 071)	279·6 (254·2 to 302·9)
	Male	1 326 814 (1 198 540 to 1 465 361)	9378·1 (8473·9 to 10 381·0)	45 055 (40 579 to 49 971)	423·6 (377·6 to 469·3)	2 477 067 (2 255 742 to 2 712 522)	6634·7 (6049·3 to 7274·8)	95 300 (87 591 to 103 591)	289·2 (265·0 to 314·5)
High fasting plasma glucose									
	Both sexes	492 031 (400 079 to 607 114)	1988·1 (1601·4 to 2516·0)	16 744 (13 015 to 21 907)	91·4 (68·4 to 127·3)	1 777 998 (1 449 520 to 2 170 035)	2511·2 (2017·4 to 3108·7)	68 121 (51 370 to 91 493)	109·5 (80·8 to 150·7)
	Female	209 639 (171 279 to 258 101)	1808·2 (1450·0 to 2314·3)	7355 (5718 to 9850)	85·4 (63·0 to 120·9)	861 064 (695 980 to 1 052 758)	2472·5 (1979·6 to 3062·7)	33 687 (25 173 to 45 261)	112·2 (81·5 to 156·1)
	Male	282 391 (224 565 to 353 197)	2144·1 (1708·5 to 2720·0)	9390 (7209 to 12 169)	96·2 (71·4 to 133·2)	916 934 (738 525 to 1 128 130)	2555·6 (2028·4 to 3194·5)	34 434 (25 937 to 47 345)	107·6 (79·8 to 148·9)
High LDLcholesterol									
	Both sexes	848 799 (712 609 to 999 261)	3106·6 (2528·4 to 3789·1)	30 291 (24 441 to 37 076)	145·5 (109·3 to 188·8)	1 203 791 (998 464 to 1 437 959)	1574·5 (1268·6 to 1922·4)	52 530 (40 050 to 66 947)	79·9 (58·2 to 105·1)
	Female	314 726 (263 599 to 373 702)	2528·9 (2017·7 to 3120·2)	12 126 (9527 to 15 127)	129·2 (94·5 to 172·3)	460 884 (369 788 to 568 383)	1287·6 (996·7 to 1626·7)	22 882 (16 693 to 30 058)	75·1 (52·4 to 101·7)
	Male	534 072 (445 485 to 635 284)	3620·9 (2960·9 to 4398·9)	18 165 (14 777 to 22 205)	159·2 (120·9 to 202·5)	742 907 (624 494 to 874 498)	1862·3 (1523·2 to 2233·1)	29 648 (23 001 to 36 975)	85·2 (63·9 to 109·5)
High systolic blood pressure									
	Both sexes	1 205 800 (1 054 681 to 1 364 300)	4800·2 (4200·5 to 5423·5)	47 648 (41 787 to 53 977)	244·8 (209·5 to 279·6)	2 127 266 (1 913 281 to 2 347 736)	2973·4 (2652·2 to 3280·2)	99 939 (86 758 to 112 458)	157·8 (135·3 to 179·1)
	Female	487 121 (429 441 to 557 118)	4266·4 (3714·5 to 4858·0)	20 663 (17 866 to 23 588)	231·5 (197·1 to 265·8)	908 895 (806 424 to 1 012 537)	2657·4 (2331·5 to 2961·1)	46 448 (39 687 to 52 480)	154·5 (130·2 to 176·0)
	Male	718 679 (621 097 to 826 401)	5247·5 (4540·8 to 6009·4)	26 986 (23 379 to 30 992)	253·4 (214·8 to 291·8)	1 218 371 (1 091 596 to 1 348 440)	3295·7 (2949·8 to 3650·7)	53 491 (46 734 to 60 346)	162·1 (140·5 to 183·4)
High body-mass index									
	Both sexes	710 771 (443 039 to 990 856)	2419·6 (1497·6 to 3414·7)	21 127 (12 854 to 30 040)	89·2 (52·9 to 131·7)	1 989 457 (1 441 100 to 2 561 406)	2580·9 (1845·1 to 3337·3)	61 415 (43 342 to 81 358)	91·7 (63·9 to 122·1)
	Female	357 549 (236 964 to 480 587)	2589·6 (1695·4 to 3516·8)	10 693 (6968 to 14 654)	96·7 (60·6 to 137·2)	952 359 (710 774 to 1 205 407)	2545·9 (1886·3 to 3238·4)	30 603 (22 263 to 39 710)	96·1 (68·5 to 125·6)
	Male	353 223 (200 530 to 523 534)	2248·7 (1265·1 to 3376·4)	10 433 (5811 to 15 739)	80·7 (43·5 to 125·8)	1 037 098 (712 749 to 1 364 782)	2617·7 (1778·2 to 3468·9)	30 812 (20 622 to 42 064)	88·1 (57·9 to 121·5)
Low bone mineral density									
	Both sexes	71 668 (57 602 to 82 246)	261·0 (211·0 to 300·6)	1828 (1428 to 2073)	7·8 (6·3 to 8·9)	117 725 (96 750 to 137 753)	154·0 (126·9 to 180·5)	3048 (2586 to 3396)	4·4 (3·8 to 5)
	Female	21 117 (17 352 to 24 514)	173·4 (142·0 to 202·0)	506 (413 to 584)	5·1 (4·1 to 6·2)	41 294 (33 393 to 49 855)	114·7 (92·9 to 138·5)	1018 (848 to 1144)	3·2 (2·7 to 3·7)
	Male	50 550 (39 161 to 58 053)	341·3 (265·0 to 391·0)	1322 (967 to 1515)	10·3 (7·9 to 11·7)	76 431 (63 055 to 88 118)	193·0 (159·3 to 224·0)	2030 (1715 to 2333)	5·6 (4·7 to 6·5)
Kidney dysfunction									
	Both sexes	445 178 (387 847 to 502 678)	1596·7 (1377·8 to 1837·6)	15 111 (12 902 to 17 507)	78·4 (65 to 93·1)	790 836 (692 731 to 897 947)	1127·2 (981·1 to 1282·7)	35 987 (30 559 to 41 889)	58·2 (48·8 to 68·1)
	Female	200 663 (176 741 to 226 572)	1502·4 (1297·0 to 1733·1)	6868 (5843 to 7973)	75·1 (61·5 to 89·7)	368 995 (321 289 to 415 886)	1077·5 (933·4 to 1226·1)	17 356 (14 522 to 20 245)	58·2 (48 to 68·7)
	Male	244 514 (208 327 to 281 379)	1673·4 (1419·8 to 1941·1)	8242 (6922 to 9598)	80·8 (66·8 to 96·5)	421 841 (367 827 to 483 023)	1179·4 (1023·7 to 1351·6)	18 631 (15 793 to 21 799)	58·4 (49·0 to 68·8)

Data in parentheses are 95% CIs. DALYs=disability-adjusted life-years.

**Table 2:** All-age percentage change between 1990 and 2019, by sex

		**DALYs**		**Death**	
		Number	Rate	Number	Rate
**All risk factors**					
Both sexes		−16·8% (−28·0 to −5·2)	−46·4% (−51·2 to −42·0)	43·8% (29·8 to 56·4)	−37·9% (−42·0 to −34·8)
Female		−18·2% (−30·5 to −5·5)	−46·0% (−51·6 to −41·0)	50·8% (33·2 to 65·3)	−35·1% (−39·8 to −30·9)
Male		−15·9% (−27·1 to −4·3)	−46·1% (−51·2 to −41·2)	38·9% (25·2 to 52·5)	−39·4% (−43·6 to −35·1)
**Environmental and occupational risks**					
Overall					
	Both sexes	−9·3% (−22·9 to 3·5)	−49·3% (−54·4 to −45·3)	46·7% (29·8 to 59·5)	−41·8% (−46·1 to −38·1)
	Female	−13·7% (−28·2 to 1·0)	−49·2% (−54·8 to −44·3)	55·3% (36·8 to 70·9)	−39·2% (−44·3 to −34·0)
	Male	−6·6% (−19·8 to 5·9)	−48·7% (−54·4 to − 43·6)	41·1% (23·5 to 55·2)	−42·8% (−48·1 to −38·1)
Unsafe water, sanitation, and handwashing					
	Both sexes	−72·9% (−82·4 to −60·4)	−72·7% (−81·7 to − 61·1)	−79·9% (−86·9 to − 70·6)	−82·5% (−87·3 to − 76·9)
	Female	−72·7% (−83·0 to −58·9)	−72·5% (−82·1 to − 59·3)	−80·7% (−88·3 to − 69·5)	−83·1% (−88·4 to − 75·1)
	Male	−73·0% (−82·8 to −59·9)	−72·7% (−82·1 to −60·8)	−79·1% (−86·5 to −67·8)	−81·5% (−86·9 to −75·4)
Air pollution					
	Both sexes	−10·0% (−24·1 to 3·6)	−49·9% (−55·0 to −45·3)	41·8% (26·6 to 54·2)	−43·7% (−48·2 to −39·6)
	Female	−9·5% (−24·5 to 6·5)	−49·2% (−54·8 to −44·0)	50·2% (33·9 to 65·6)	−41·5% (−46·9 to −35·1)
	Male	−10·4% (−24·8 to 3·9)	−49·7% (−55·9 to −44·3)	36·1% (19·3 to 50·9)	−44·7% (−50·4 to −39·6)
Non-optimal temperature					
	Both sexes	15·5% (−13·5 to 44·0)	−45·2% (−52·7 to −39·9)	107·4% (78·4 to 129·2)	−32·7% (−38·6 to −27·3)
	Female	7·9% (−18·4 to 34·3)	−45·3% (−52·9 to −39·6)	110·8% (82·0 to 133·9)	−29·7% (−35·8 to −22·8)
	Male	22·8% (−10·8 to 57·9)	−44·3% (−53·0 to −37·5)	104·5% (73·4 to 133·2)	−34·5% (−41·4 to −28·3)
Other environmental risks					
	Both sexes	24·1% (9·7 to 38·4)	−48·2% (−53·7 to −43·4)	84·1% (57·8 to 108·1)	−37·3% (−44·4 to −30·6)
	Female	33·2% (13·0 to 52·1)	−46·1% (−53·3 to − 39·4)	107·6% (72·1 to 140·8)	−33·9% (−44·2 to − 24·2)
	Male	19·8% (5·9 to 34·2)	−48·2% (−53·9 to − 42·5)	72·5% (48·0 to 96·5)	−38·7% (−45·5 to − 31·4)
Occupational risks					
	Both sexes	42·9% (21·6 to 68·7)	−36·3% (−44·8 to − 26·0)	30·2% (4·6 to 65·2)	−41·6% (−51·9 to − 28·5)
	Female	160·2% (108·8 to 227·7)	3·2% (−17·0 to 28·0)	121·7% (73·6 to 199·6)	−20·3% (−37·7 to 11·3)
	Male	34·4% (13·4 to 60·7)	−38·7% (−47·4 to −28·0)	27·0% (1·5 to 62·3)	−42·8% (−53·0 to −29·3)
**Behavioural risks**					
Overall					
	Both sexes	−41·4% (−50·2 to −31·0)	−56·7% (−61·6 to −51·8)	2·8% (−9·4 to 15·6)	−47·4% (−51·0 to −43·9)
	Female	−48·4% (−57·4 to −38·2)	−59·3% (−64·9 to −53·9)	−4·0% (−17·4 to 9·7)	−47·8% (−52·0 to −43·3)
	Male	−36·6% (−46·2 to −25·7)	−54·5% (−59·7 to −49·3)	7·1% (−5·8 to 19·9)	−46·8% (−51·2 to −42·5)
Child and maternal malnutrition					
	Both sexes	−84·5% (−88·4 to −80·0)	−80·9% (−85·6 to −75·3)	−87·9% (−91·4 to −83·7)	−84·2% (−88·7 to −78·8)
	Female	−84·0% (−87·9 to −79·1)	−80·5% (−85·3 to −74·7)	−88·1% (−91·5 to −83·9)	−84·4% (−88·7 to −78·9)
	Male	−84·9% (−89·0 to −80·2)	−81·2% (−86·3 to −75·5)	−87·8% (−91·5 to −83·5)	−84% (−88·9 to −78·5)
Tobacco use					
	Both sexes	40·7% (27·0 to 55·5)	−42·2% (−46·9 to − 37·1)	58·1% (45·4 to 73·4)	−41·4% (−46·1 to − 36·2)
	Female	29·7% (7·0 to 57·9)	−42·5% (−51·7 to − 31·9)	58·5% (32·4 to 89·8)	−40·6% (−50·3 to − 29·0)
	Male	44·1% (30·8 to 59·9)	−40·9% (−45·6 to − 35·1)	58·0% (43·4 to 75·5)	−41·7% (−46·9 to − 35·6)
Alcohol use					
	Both sexes	178·9% (128·1 to 251·6)	27·0% (3·6 to 59·5)	255·5% (167·7 to 389·3)	36·7% (2·7 to 89·6)
	Female	118·0% (86·3 to 165·4)	−0·7% (−17·9 to 22·6)	169·6% (92·1 to 294·2)	−2·8% (−34·7 to 49·7)
	Male	194·8% (134·7 to 281·1)	36·4% (8·1 to 76·0)	275·0% (176 to 427·9)	49·5% (10·9 to 106·7)
Drug use					
	Both sexes	158·9% (139·6 to 182·4)	28·2% (18·8 to 39·4)	204·8% (166·4 to 255·2)	28·3% (10·7 to 50·1)
	Female	107·3% (87·8 to 131·2)	7·1% (−2·2 to 17·3)	149·1% (107·3 to 204·5)	6·5% (−11·5 to 29·1)
	Male	179·9% (155·3 to 211·3)	37·6% (25·6 to 52·9)	220·7% (175·8 to 282·2)	36·9% (16·5 to 62·7)
Dietary risks					
	Both sexes	46·7% (32·3 to 59·2)	−47·5% (−52·3 to −43·3)	72·8% (56·9 to 87·6)	−45·3% (−49·8 to −40·7)
	Female	58·7% (41·9 to 76·6)	−45·6% (−51·1 to −39·6)	90·9% (71·0 to 114·1)	−41·8% (−47·7 to −33·9)
	Male	40·1% (25·1 to 55·1)	−47·9% (−53·4 to − 42·6)	61·6% (43·3 to 78·1)	−47·0% (−52·6 to −41·8)
Intimate partner violence					
	Female	113·0% (90·3 to 135·0)	5·4% (1·6 to 15·6)	125·4% (84·9 to 186·9)	19·2% (−1·4 to 45·1)
Childhood sexual abuse and bullying					
	Both sexes	56·6% (35·1 to 80·5)	18·8% (8·8 to 27·9)	57·4% (28·4 to 127·1)	−40·0% (−50·9 to −13·5)
	Female	61·9% (38·5 to 88·1)	24·1% (10·7 to 38·8)	4·4% (−11·5 to 22·2)	−51·8% (−58·4 to −43·9)
	Male	54·1% (31·4 to 82·0)	16·3% (5·9 to 28·1)	62·5% (30·2 to 141·8)	−38·0% (−49·9 to −8·8)
Unsafe sex					
	Both sexes	155·2% (112·8 to 220·2)	−2·0% (−18·4 to 23·5)	165·5% (111·6 to 256·5)	−12·3% (−33·3 to 13·8)
	Female	115·6% (82·5 to 173·1)	−19·4% (−32·4 to 2·5)	125·9% (81·5 to 197·2)	−23·5% (−41·5 to −1·2)
	Male	466·7% (273·0 to 759·9)	168·9% (84·3 to 298·8)	1849·5% (1034·1 to 3769·0)	569·4% (323·1 to 1055·1)
Low physical activity					
	Both sexes	131·9% (93·3 to 216·6)	−26·0% (−35·4 to −6·4)	163·1% (122·5 to 232·5)	−30·4% (−36·7 to −19·9)
	Female	163·8% (118·9 to 244·9)	−19·5% (−30·8 to 1·1)	185·2% (141·1 to 252·1)	−24·2% (−32·9 to −9·9)
	Male	107·5% (73·3 to 190·1)	−30·6% (−39·7 to −10·9)	144·1% (103·2 to 232·1)	−34·8% (−41·5 to −24·0)
**Metabolic risks**					
	Both sexes	98·3% (84·1 to 112·1)	−27·9% (−33·3 to − 23·3)	126·9% (108·2 to 142·4)	−29·6% (−34·6 to − 25·2)
	Female	114·8% (96·6 to 132·6)	−25·0% (−31·4 to − 19·1)	147·2% (123·5 to 166·8)	−25·6% (−31·9 to − 19·6)
	Male	86·7% (70·4 to 102·6)	−29·3% (−35·2 to − 23·6)	111·5% (93·1 to 129·7)	−31·7% (−37·2 to − 26·3)
High fasting plasma glucose					
	Both sexes	261·4% (225·3 to 304·3)	26·3% (14·9 to 39·2)	306·8% (248·4 to 385·9)	19·9% (7·9 to 35·2)
	Female	310·7% (264·6 to 359·7)	36·7% (22·5 to 53·4)	358·0% (289·0 to 439·8)	31·3% (14·8 to 52·5)
	Male	224·7% (186·8 to 273·0)	19·2% (7·5 to 33·8)	266·7% (208·5 to 350·9)	11·9% (−1·4 to 28·5)
High LDL cholesterol					
	Both sexes	41·8% (24·0 to 56·8)	−49·3% (−54·7 to −45·1)	73·4% (47·0 to 99·3)	−45·1% (−50·7 to −40·3)
	Female	46·4% (24·8 to 66·2)	−49·1% (−55·1 to −43·1)	88·7% (59·0 to 121·1)	−41·9% (−48·6 to −34)
	Male	39·1% (22·0 to 55·0)	−48·6% (−54·3 to −43·5)	63·2% (38·3 to 89·5)	−46·5% (−53 to −41·2)
High systolic blood pressure					
	Both sexes	76·4% (58·3 to 90·0)	−38·1% (−43·8 to −33·8)	109·7% (85·0 to 127·1)	−35·6% (−42·3 to −31·3)
	Female	86·6% (61·2 to 105·8)	−37·7% (−45·8 to −32·0)	124·8% (90·5 to 148·5)	−33·3% (−43·2 to −26·6)
	Male	69·5% (52·9 to 86)	−37·2% (−42·4 to − 31·8)	98·2% (77·6 to 119·5)	−36% (−41·2 to −30·6)
High body-mass index					
	Both sexes	179·9% (136·4 to 256·3)	6·7% (−9·5 to 35·6)	190·7% (148·0 to 270·2)	2·8% (−13·1 to 32·6)
	Female	166·4% (127·7 to 229·1)	−1·7% (−15·3 to 22·5)	186·2% (141·0 to 261·6)	−0·7% (−16·6 to 28·3)
	Male	193·6% (137·6 to 323·4)	16·4% (−4·9 to 68·8)	195·3% (141·1 to 315·7)	9·1% (−10·8 to 57·2)
Low bone mineral density					
	Both sexes	64·3% (50·6 to 89·1)	−41·0% (−45·7 to −33·2)	66·8% (49·7 to 102·0)	−43·3% (−48·9 to −32·5)
	Female	95·5% (79·3 to 120·0)	−33·8% (−39·7 to −24·3)	101·2% (73·0 to 162·8)	−37·1% (−47·5 to −14·1)
	Male	51·2% (36·3 to 85·8)	−43·4% (−48·7 to −32·1)	53·6% (35·0 to 101·3)	−45·2% (−51·3 to −31·8)
Kidney dysfunction					
	Both sexes	77·6% (64·2 to 91·3)	−29·4% (−33·9 to −24·7)	138·2% (116·9 to 159·1)	−25·8% (−31·7 to −20·5)
	Female	83·9% (69·6 to 100·3)	−28·3% (−33·9 to −22)	152·7% (126·4 to 180·9)	−22·4% (−30·6 to −12·9)
	Male	72·5% (57·0 to 90·0)	−29·5% (−35·0 to −24·0)	126·0% (103·9 to 148·1)	−27·8% (−33·7 to −22)

Data in parentheses are 95% CIs. DALYs=disability-adjusted life-years.

These patterns showed that environmental risk factors are primarily dependent on socioeconomic development. An example is unsafe water and sanitation, with the highest burden in a deprived province—Sistan and Baluchistan—and the lowest burden in the capital, Tehran. Results show a slight peak in the burden attributable to unsafe water and sanitation and mortality caused by enteric infections in 1990, followed by a sharp decline until 2019, which coincides with the expansion of the piped water and sewerage network.^1^ Another example is the Iran Gas Trunk line associated with decreased household air pollution caused by using solid fuels for cooking and heating.^1^ Therefore, supporting development in deprived regions could prevent exposure to harmful environmental risks.

### NCDs as a growing concern

**Figure 3:**
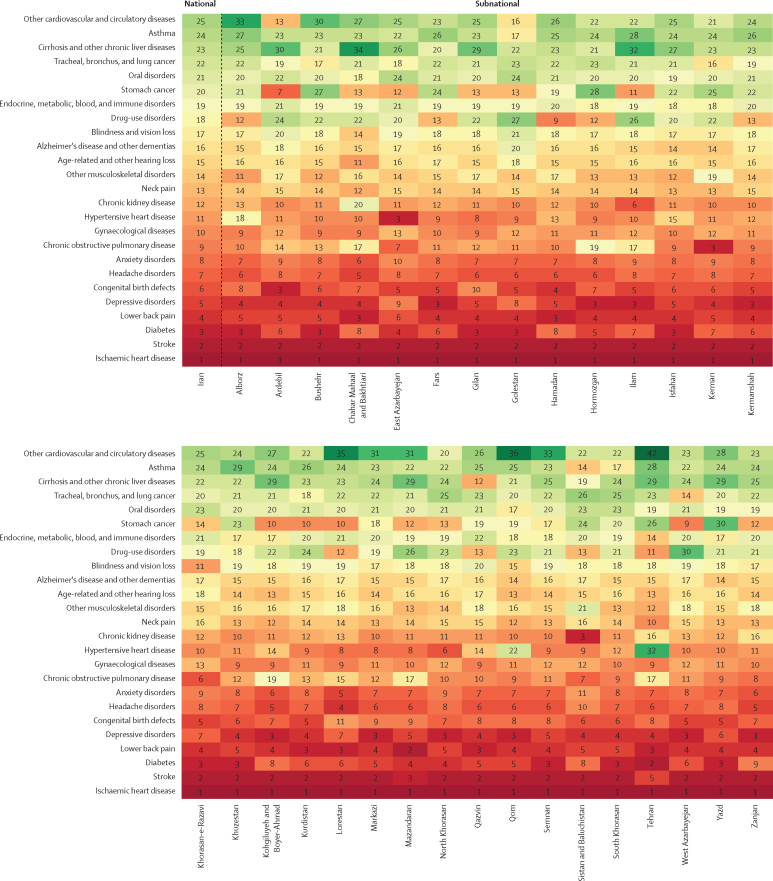
Ranking of age-standardised rates of disability-adjusted life-years caused by level 3 non-communicable diseases at national and subnational levels, 2019

The emergence of NCDs is a new challenge to Iranian health care.^1^ In 2019, 15·5 million (95% UI 13·2–18·1) DALYs were caused by NCDs, 44·2% more than in 1990, whereas the age-standardised DALY rate of NCDs substantially decreased by 25·9% (appendix p 125). Ischaemic heart disease was the leading cause of age-standardised DALY rates in 1990 and 2019 (figure 3; appendix p 45). The next leading causes were stroke, diabetes, lower back pain, and depressive disorders at the national level and in most provinces in 2019, with the largest 30-year change observed for diabetes, a nearly two-fold increase. Chronic obstructive pulmonary disease (age-standardised DALY rate 517·2 per 100 000 people, 471·0–560·8) and asthma (232·3 per 100 000, 185·0–299·3), are main WHO targets for Sustainable Development Goals (SDGs; target 3.4), with percentage changes of −7·0% and −56·1% from 1990 to 2019, respectively. The age-standardised death rate of most neoplasms showed a mixed pattern, with stomach (16·2, 95% UI 14·9–17·4), tracheal, bronchus, and lung (12·9, 11·9–13·9), colorectal (9·3, 8·5–10·1), and prostate (6·5, 4·9–7·4) cancers occupying the leading ranks in 2019 (appendix p 125).

The pattern of all-cause health burden attributable to risk factors changed in favour of metabolic risk factors from 1990 to 2019 (appendix p 53–55). We estimate that 31·7% of total DALYs caused by NCDs (6·3 million, 95% UI 5·7–6·9) were attributable to at least one risk factor in 2019, including 22·0% attributable to metabolic (4·4 million, 4·0–4·8), 17·1% to behavioural (3·4 million, 3·0–3·7), and 9·3% to environmental and occupational (1·9 million, 1·7–2·1) risks. Implementation of the PHC system, Health Transformation Plan, and ease of access to screening programmes for cancer and metabolic disorders are believed to be the lower-stream sources of the changing trend of NCDs at the health-provider level,^39^ which is in line with the upper-stream developments in the economy, literacy, and infrastructure, among other areas. Variability at the subnational level of modified diet, urbanisation, sedentary lifestyle, and ageing of the population can be regarded as the cause of geographical disparities in NCDs.^1^, ^40^

**Figure 4:**
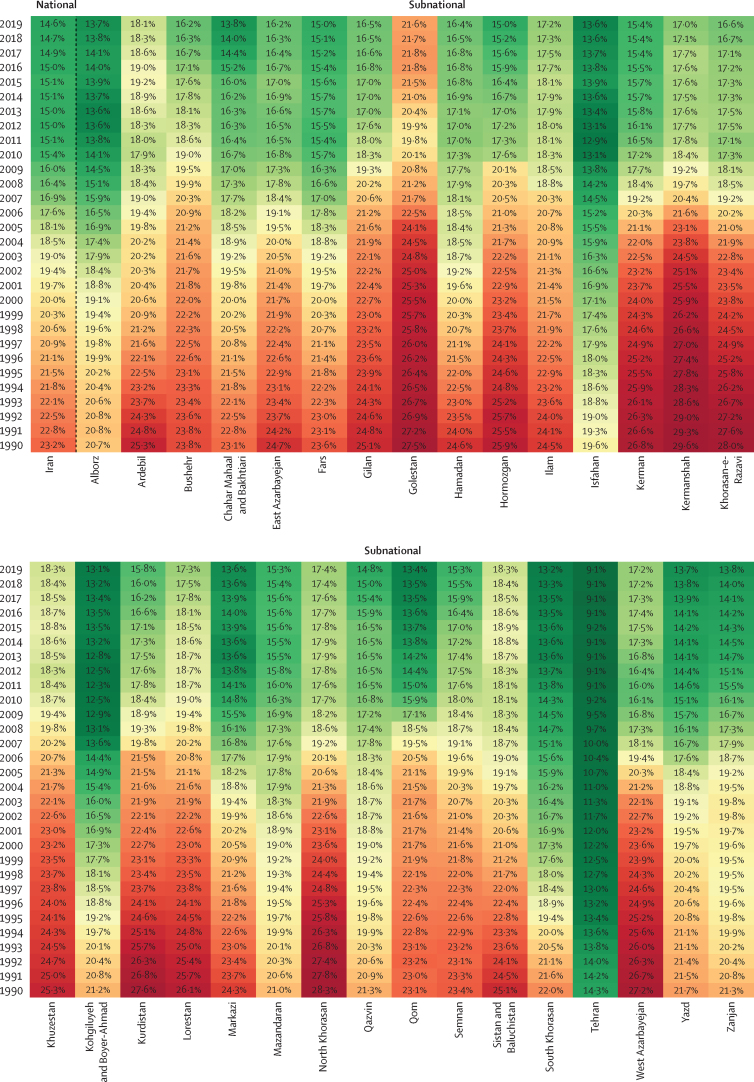
Time trend of the unconditional probability of death at national and subnational levels, 1990–2019

In 2019, 326 508 Iranians (95% UI 318 268–335 734) died from NCDs, 88·0% more than in 1990 (appendix p 125). The age-standardised YLL rate of NCDs significantly declined by 41·3%, whereas the age-standardised YLD rate remained statistically stable with a 1·4% increase. This observation implies that the share of DALYs consisting of YLDs increased compared with the share of YLLs in the past three decades. The number of premature (30–70 years of age) deaths caused by four main NCDs (neoplasms, cardiovascular diseases, chronic respiratory diseases, and diabetes) was 66 818 (52·7% of all-age mortality caused by NCDs) in 1990 and reached 100 893 (37·1%) in 2019. According to SDGs target 3.4, the share of premature deaths should reach 11·6% in 2025 in Iran, which might not be attainable with the current trend. As the top-ranked NCD, cardiovascular diseases caused about 54·6% of premature deaths in 2019. The unconditional probability of death (UPoD) has been estimated to be constantly decreasing in the past three decades and reached 14·6% in 2019 for both sexes (figure 4). Additionally, UPoD was estimated to be 1·5 times higher among male individuals, but both sexes showed a similar decreasing trend. The highest UPoD was estimated in the Golestan province with 21·6% probability, compared with 9·1% in Tehran (the lowest UPoD).

Primary, secondary, and tertiary prevention should be adopted to tackle the challenge of NCDs in Iran. Health policy makers should implement multisectoral approaches to address the high prevalence of modifiable cardiovascular risk factors, including obesity and overweight (60%),^41^ dyslipidaemia (80%),^42^ and hypertension (53%)^37^ in Iranian adults. Since the early 2000s, lifesaving treatments for NCDs, such as primary percutaneous coronary intervention, fibrinolytic therapy, and emergency surgeries, became more extensively available with increasing hospital bed density and adoption of modern facilities.^43^, ^44^ Although the prevention of deaths is an important target, averting deaths can lead to higher rates of chronic conditions compared with previous time periods because of an ageing population, such as heart failure after an acute coronary syndrome, which cause disability and necessitate appropriate rehabilitation centres.

Although the Iranian health-care system appears to be alleviating the risks of NCDs, this does not downgrade the importance of control measures for NCDs.^45^ Subnational geographical dissimilarity indicates uneven distribution of health system goods and urgent need for action. The PHC network, the 2004 Universal Rural Health Insurance, and the 2014 Health Transformation Plan partially contributed to resolving these disparities by easing accessibility and affordability of health-care services in underserved locations.^1^, ^46^ The estimated epidemiological measures indicate that health policies have been effective in modifying the burden of NCDs, but improvements have remained trivial in many sectors and geographical areas.

### The unmet need for mental disorders care

Mental disorders accounted for 2295·8 (95% UI 1702·2–3033·6) of age-standardised DALYs per 100 000 people and substance-use disorders accounted for 399·9 (324·3–481·3) age-standardised DALYs per 100 000 in 2019, with 1·8% and 20·1% increases, respectively, compared with 1990 (appendix pp 46, 125). These disorders are responsible for an increasing percentage of total DALYs, up from 0·7% in 1990 to 1·9% in 2019 (a 193·6% increase). Depressive disorders (890·3, 605·8–1247·8), anxiety disorders (695·8, 483·3–954·7), and bipolar disorder (175·8, 107·9–270·0) were the mental conditions that most contributed to the age-standardised DALY rate in 2019. Depressive disorders had a particularly notable change with a 104·4% increase in burden. At the subnational level, the Fars province always had the greatest age-standardised DALY rate of mental disorders, whereas east Azarbayejan had the lowest rate during most of the study period (appendix p 152). Opioid use was the top cause of substance -use disorders, with an estimated 269 294 DALYs [212 575–333 596] in 2019 and a 146·4% increase. The trend of substance use is increasing all around the country, with no convergence or divergence. Hamadan, Tehran, and Sistan and Baluchistan were found to be the leading provinces by age-standardised DALYs rate; however, Kermanshah recorded an increasing trend from 1990 and reached a peak in 2007, followed by a decrease.

Access to mental health services did not grow sufficiently in response to the growing burden of mental and substance-use disorders. Concerns about psychological issues were highlighted after the end of the Iraq–Iran war as the Iranian Mental Health Survey demonstrated that the prevalence of psychiatric disorders in Iran is relatively high.^1^, ^47^ In addition, mental health awareness and services remain inadequate with two-thirds of Iranians diagnosed with a mental health disorder not receiving any care and treatment options for substance-use disorders.^48^, ^49^ Integration of mental health services into the PHC system by training Behvarzes (community health workers in Iran) from 1990 resulted in modest improvement of attitudes, knowledge, and satisfaction of these practitioners and the general population towards mental health and psychological conditions.^50^ These integrated services were not systematically evaluated for their effectiveness or revisited considering they were made mainstream within community health services since the start of our study period and because of the multifactorial dynamic of mental disorders. Furthermore, actions and efforts should be revisited continuously and weighted on the basis of the burden of mental disorders at national and subnational levels.

### Injuries, an emerging challenge for the health-care system

Road injuries climbed to the second rank by DALY rates from 1990 to 2019 (1302·1 per 100 000, 95% UI 1147·4–1488·3) and caused 21 122 deaths (95% UI 18 110–24 648) in 2019. DALY and death age-standardised rates substantially decreased by around 60% in this period, with a slightly steeper decline between 2002 and 2015. The reduction in the DALY rate might be associated with increased GDP per person, given the reverse-U-shaped association between this index and the burden of transport injuries.^51^, ^52^ Therefore, the minimal reduction in DALYs from road injuries might be related to the impact of sanctions on GDP.^20^ It is noteworthy that, although Iran managed road injury mortality and decreased DALYs, the age-standardised rate of YLDs reduced by 41·0%, and the share of YLDs out of DALYs increased (appendix p 47).

The highest number of new cases of road injuries, 77 143 (95% UI 63 939–91 908), occurred in Tehran province in 2019. Tehran had the lowest (303·8, 95% UI 216·9–667·2) age-standardised DALY rates and Sistan and Baluchistan provinces the highest (2286·8, 1978·1–2627·9) age-standardised DALY rates in 2019. All of the subnational DALY rates converged in this period. Qom, Khorasan-e-Razavi, and Zanjan recorded the greatest declines in age-standardised DALY rates, by 70%, and Tehran had one of the lowest percentage changes in this regard (appendix p 152).

Policies to control road traffic injuries showed many shortcomings in Iran, pointing to the need for more effective measures.^53^ The low prevalence of seatbelt (about 75%) and helmet use (about 14%) in the Iranian population is a major contributor to road traffic injuries.^54^ Despite improvement in road infrastructure, low-quality domestic vehicles along with poor driving behaviours still contribute to this burden.^55^ Although control measures by the traffic police contributed to this,^56^ limitations in the widespread implementation of these measures highlight the importance of novel approaches such as telematics.^53^ Towards this objective, the national action plan for NCD Prevention and Control provided by the Iranian Non-Communicable Diseases Committee (INCDC) was developed in 2015, with a specific target of a 20% relative reduction in the mortality rate caused by traffic injuries by 2025, which needs a multisectoral approach.^57^

### Consequences of sanctions and major health sector reforms on the health of Iranians

**Figure 5:**
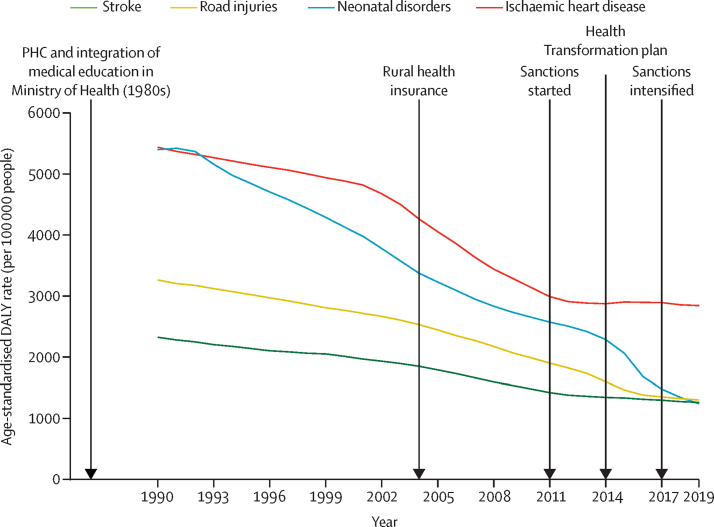
Timeline of major events in Iranian history from 1990 to 2019 DALY=disability-adjusted life-years. PHC=primary health care.

Exploring the impacts of the 2011 sanctions and two major health sector reforms in 2004 (the Universal Rural Health Insurance) and 2014 (the Health Transformation Plan) revealed that NCD deaths had a smaller decreasing slope during the 5 years after the beginning of sanctions in 2011 compared with the previous and following periods. Among NCDs, neoplasms showed the most substantial change in this investigation (appendix p 1176). Concerning NCD deaths, the regression model showed that deaths from NCDs had a significant decreasing effect on temporal changes after 2004 (coefficient −10·58, SE 0·97; p<0·0001) and a significant increasing effect on temporal changes after 2011 (coefficient 11·21, SE 2·25; p=0·0001; appendix p 1177). In the case of U5MR, the model showed a mild increase in mortality after the 2004 health-care transformation (coefficient 0·64, SE 0·21; p=0·013), and the effects of other changes were not statistically significant (appendix p 1178). Overall, among these major changes, the 2011 sanctions had the most remarkable and detrimental impact on the health of Iranians, especially regarding deaths caused by NCDs (figure 5).

The neutral effect of the 2004 reform might be attributed to the fact that this strategy was not planned to address NCDs. Although there were some guidelines for managing NCDs and their risk factors, integrating multifactorial approaches to control NCDs was not done until the 2014 reform. Notably, the 2004 reform aimed to improve children's health; nevertheless, with Behvarzes in health houses, improved vaccination coverage and nutrition messaging had already achieved great strides in improving children's health. Therefore, the 2004 reform was not able to further decrease U5MR. The 2011 sanctions might have affected deaths of Iranians caused by NCDs, especially neoplasms, through decreasing timely diagnosis and reducing accessibility of modern pharmacological interventions.^1^, ^11^ The wide-ranging impacts of sanctions constrained drug supplies and non-pharmaceutical resources necessary for health-care services.^58^, ^59^ The national pursuit of UHC coverage was dampened by the sanctions, leaving some health policy reforms underfunded or ineffective.^60^ We did not capture alleviation in the burden of disease following the 2014 reform, given that it aimed to enhance health infrastructure and improve health-care density, which needs more time to affect the health-care system. It should be noted that the transformation plan expanded hospital and intensive care unit beds, which helped the Iranian health-care system better cope with the COVID-19 pandemic, in addition to other capacities of the health system.^61^ This expansion of health-care-system infrastructures should also be used for prevention. Although the 2014 reform might be a step towards UHC, the absence of a clear and sufficient budget line is an important drawback.^1^

### Call to action

Altogether, the results of this study should be translated into practical actions and strategies. National and international agencies and policy makers should be prompted and informed that sanctions indirectly had major adverse effects on the health of Iranians by diminishing access to quality care. Delayed or inaccessible treatment exacerbated health burdens, especially in those with cancers. Although sanctions do not include health and medical issues, their effects on financial transactions, such as those required for the importation of medicines, have had unfavourable consequences, as represented in this study.

Future policies in the health sector should be prioritised to integrate health-care services to address NCDs, especially cardiovascular diseases, and modifiable risk factors, into the existing health-care infrastructure, such as the Iranian Package of Essential NCD Interventions for primary health care. Because the PHC system is unable to manage NCDs alone, this issue needs an integrated intersectoral collaboration such as the INCDC. Examples in this regard are dietary improvements that require an alliance between the health sector and the food industry, and improvements in ambient air pollution in major cities that require cooperation to shift from using fossil fuels to cleaner sources of energy. Further, strategies should focus on providing integrated care to those with mental health and substance use disorders to combat their increasing and unmet burden. The concurrent HIV/AIDS epidemic and re-emergence of tuberculosis in Iran require continued prevention efforts such as the directly-observed therapy short course.

Regarding the existing health disparities among provinces, closing the health equity gaps in Iran needs a two-part approach of health sector policies and cross-government actions to effectively use all resources to address this concern.^62^ One of the major steps in resolving health disparities is monitoring health inequalities; that includes practical and continuous measurement and reporting of disparities, which is an essential step in low-income and middle-income countries that do not have adequate data sources.^62^ These disparities should encourage multisectoral policy making, collaboration, and resource allocation on the basis of affirmative action to resolve disparities through equal distribution of education, sanitation, nutrition, road infrastructure, and PHC across the country.^63^

Ultimately, the emergence of COVID-19 and its confluence with socioeconomic gaps, economic recession, and the heavy burden of NCDs in Iran could have direct and indirect effects on the care provided for major diseases and expand inequalities in the care provided.^64^ Therefore, careful evidence-based policies should be made to prevent the further adverse effects of COVID-19, which requires integrated collaboration and effort. Limitations of this study are presented in the appendix (section 6).

### Conclusion

A remarkable improvement in life expectancy has happened in the past three decades in Iran. The Iranian health-care system has successfully managed CMNNDs; however, it is encountering NCDs and injuries as its new challenges. In the study period, the Iranian health-care system has been more effective at averting deaths than managing morbidity and mental disorders, indicating an unmet need for rehabilitation centres and integration of mental health services into PHC. Environmental changes as developing risks threaten the population. Besides addressing the current challenges and subnational disparities, the Iranian health-care system must be more prepared for emerging diseases, such as the COVID-19 pandemic.

## Data sharing

This study follows the Guidelines for Accurate and Transparent Health Estimates Reporting. All data sources used in this analysis are found on the Global Health Data Exchange (http://ghdx.healthdata.org/gbd-2019/data-input-sources), and related code is available at http://ghdx.healthdata.org/gbd-2019/code. Additional results from this study and the larger GBD 2019 analysis can be explored using our data visualisation tools at https://vizhub.healthdata.org/gbd-compare.

## Declaration of interests

MA-L reports leadership or fiduciary roles in a board, society, committee, or advocacy groups, paid or unpaid with International Affairs in the Ministry of Health, Iran as Director General, all outside the submitted work. SBor reports support for the present manuscript from medical writing. All other authors declare no competing interests.

## References

[1] Danaei G, Farzadfar F, Kelishadi R (2019). Iran in transition. Lancet.

[2] The World Bank (2021). GDP per capita (current US$)—Iran, Islamic Rep. https://data.worldbank.org/indicator/NY.GDP.PCAP.CD?end=2018&locations=IR&start=1960.

[3] Sajadi HS, Ehsani-Chimeh E, Majdzadeh R (2019). Universal health coverage in Iran: where we stand and how we can move forward. Med J Islamic Rep Iran.

[4] Kokabisaghi F (2018). Assessment of the effects of economic sanctions on Iranians' right to health by using human rights impact assessment tool: a systematic review. Int J Health Policy Manag.

[5] Nasseri K, Sadrizadeh B, Malek-Afzali H (1991). Primary health care and immunisation in Iran. Pub Health.

[6] Takian A, Doshmangir L, Rashidian A (2013). Implementing family physician programme in rural Iran: exploring the role of an existing primary health care network. Fam Pract.

[7] Doshmangir L, Bazyar M, Rashidian A, Gordeev VS (2021). Iran health insurance system in transition: equity concerns and steps to achieve universal health coverage. Int J Equit Health.

[8] Fattahi N, Azadnajafabad S, Mohammadi E (2021). Geographical, gender and age inequalities in non-communicable diseases both at national and provincial levels in Iran. J Diabetes Metab Disord.

[9] Djalalinia S, Modirian M, Sheidaei A (2017). Protocol design for large-scale cross-sectional studies of surveillance of risk factors of non-communicable diseases in Iran: STEPs 2016. Arch Iran Med.

[10] GBD 2019 Diseases and Injuries Collaborators (2020). Global burden of 369 diseases and injuries in 204 countries and territories, 1990–2019: a systematic analysis for the Global Burden of Disease Study 2019. Lancet.

[11] Aminorroaya A, Fattahi N, Azadnajafabad S (2020). Burden of non-communicable diseases in Iran: past, present, and future. J Diabetes Metab Disord.

[12] GBD 2019 Demographics Collaborators (2020). Global age-sex-specific fertility, mortality, healthy life expectancy (HALE), and population estimates in 204 countries and territories, 1950–2019: a comprehensive demographic analysis for the Global Burden of Disease Study 2019. Lancet.

[13] Azadnajafabad S, Mohammadi E, Aminorroaya A (2021). Non-communicable diseases' risk factors in Iran; a review of the present status and action plans. J Diabetes Metab Disord.

[14] GBD 2019 Risk Factors Collaborators (2020). Global burden of 87 risk factors in 204 countries and territories, 1990–2019: a systematic analysis for the Global Burden of Disease Study 2019. Lancet.

[15] GBD 2019 Viewpoint Collaborators (2020). Five insights from the global burden of disease study 2019. Lancet.

[16] Global Health Data Exchange Global Burden of Disease Study 2019 (GBD 2019) data input sources tool. https://ghdx.healthdata.org/gbd-2019/data-input-sources.

[17] Global Health Data Exchange Global Burden of Disease Study 2019 (GBD 2019) code. https://ghdx.healthdata.org/gbd-2019/code.

[18] Andreev EM, Shkolnikov VM, Begun AZ (2002). Algorithm for decomposition of differences between aggregate demographic measures and its application to life expectancies, healthy life expectancies, parity-progression ratios and total fertility rates. Demographic Res.

[19] Wagner AK, Soumerai SB, Zhang F, Ross-Degnan D (2002). Segmented regression analysis of interrupted time series studies in medication use research. J Clin Pharm Therap.

[20] The World Bank (2021). Current health expenditure per capita, PPP (current international $)—Iran, Islamic Rep. https://data.worldbank.org/indicator/SH.XPD.CHEX.PP.CD?locations=IR.

[21] Pourmarzi D, Khoramirad A, Gaeeni M (2017). Perceived stigma in people living with HIV in Qom. J Family Reprod Health.

[22] Baghi HB, Aghazadeh M, Rashedi J, Poor BM (2017). HIV/AIDS in Iran. Clin Infect Dis.

[23] VizHub GBD compare. https://vizhub.healthdata.org/gbd-compare/#.

[24] Joulaei H, Motazedian N (2013). Primary health care strategic key to control HIV/AIDS in Iran. Iran J Public Health.

[25] Heidary M, Nasiri MJ (2016). Why has HIV/AIDS prevalence increased in Iran?. Clin Infect Dis.

[26] Talbot JR, Bohrer M, Rhatigan J (2011).

[27] Khodayari-Zarnaq R, Mosaddeghrad AM, Nadrian H, Kabiri N, Ravaghi H (2019). Comprehensive analysis of the HIV/AIDS policy-making process in Iran. Health Res Pol Syst.

[28] Shadpour K (2000). Primary health care networks in the Islamic Republic of Iran. East Mediterr Health J.

[29] Moghadami M, Dadashpour N, Mokhtari AM, Ebrahimi M, Mirahmadizadeh A (2019). The effectiveness of the national hepatitis B vaccination program 25 years after its introduction in Iran: a historical cohort study. Braz J Infect Dis.

[30] Majdzadeh R, Moradi A, Zeraati H, Sepanlou SG, Zamani G, Zonobi V (2008). Evaluation of the measles-rubella mass vaccination campaign in the population covered by Tehran University of Medical Sciences. East Mediterr Health J.

[31] Azizi MH, Bahadori M (2011). A brief history of tuberculosis in Iran during the 19th and 20th centuries. Arch Iran Med.

[32] Malekafzali H (2009). Primary health care in the rural area of the Islamic Republic of Iran. Iran J Public Health.

[33] Mehrdad R (2009). Health system in Iran. JMAJ.

[34] Moshiri E, Rashidian A, Arab M, Khosravi A (2016). Using an analytical framework to explain the formation of primary health care in rural Iran in the 1980s. Arch Iran Med.

[35] Bastani P, Vatankhah S, Salehi M (2013). Performance ratio analysis: a national study on Iranian hospitals affiliated to Ministry of Health and Medical Education. Iran J Public Health.

[36] Brajer V, Hall J, Rahmatian M (1970). Air pollution, its mortality risk, and economic impacts in Tehran, Iran. Iran J Public Health.

[37] Naddafi K, Hassanvand MS, Yunesian M (2012). Health impact assessment of air pollution in megacity of Tehran, Iran. Iran J Environ Health Sci Eng.

[38] Shamsipour M, Farzadfar F, Gohari K (2014). A framework for exploration and cleaning of environmental data: Tehran air quality data experience. Arch Iran Med.

[39] Mohammadi E, Aminorroaya A, Fattahi N (2021). Epidemiologic pattern of cancers in Iran; current knowledge and future perspective. J Diabet Metab Disord.

[40] UN (2014). https://www.un.org/en/development/desa/publications/2014-revision-world-urbanization-prospects.html#:~:text=The%202014%20revision%20of%20the,population%20between%202014%20and%202050.

[41] Mahdavi M, Parsaeian M, Mohajer B (2020). Insight into blood pressure targets for universal coverage of hypertension services in Iran: the 2017 ACC/AHA versus JNC 8 hypertension guidelines. BMC Public Health.

[42] Aryan Z, Mahmoudi N, Sheidaei A (2018). The prevalence, awareness, and treatment of lipid abnormalities in Iranian adults: surveillance of risk factors of noncommunicable diseases in Iran 2016. J Clin Lipid.

[43] Abdi S, Haji Aghajani M, Janbabaei G (2021). 24/7 primary percutaneous coronary intervention as a national program. Crit Pathw Cardiol.

[44] Rezaei S, Bazyar M, Fallah R, Chavehpour Y, Homaie Rad E (2015). Assessment of need and access to physician and hospital beds: a cross sectional province based study in Iran. Shiraz E-Med J.

[45] Rahbar M, Ahmadi M (2015). Lessons learnt from the model of instructional system for training community health workers in rural health houses of iran. Iran Red Cres Med J.

[46] Farzadfar F, Murray CJL, Gakidou E (2012). Effectiveness of diabetes and hypertension management by rural primary health-care workers (Behvarz workers) in Iran: a nationally representative observational study. Lancet.

[47] Sharifi V, Amin-Esmaeili M, Hajebi A (2015). Twelve-month prevalence and correlates of psychiatric disorders in Iran: the Iranian Mental Health Survey, 2011. Arch Iran Med.

[48] Amin-Esmaeili M, Rahimi-Movaghar A, Sharifi V (2016). Epidemiology of illicit drug use disorders in Iran: prevalence, correlates, comorbidity and service utilization results from the Iranian Mental Health Survey. Addiction.

[49] Danaei G, Farzadfar F, Kelishadi R (2019). Iran in transition. Lancet.

[50] Shariat SV, Mansouri N, Gharraee B, Bolhari J, Yousefi Nourai R, Rahimi Movaghar A (2011). Attitude, knowledge, and satisfaction of health personnel and general population about the program of integration of mental health in PHC in Iran: systematic review. Iran J Psych Clin Psych.

[51] Kopits E, Cropper M (2005). Traffic fatalities and economic growth. Acc Anal Prev.

[52] Iwata K (2010). The relationship between traffic accidents and economic growth in China. Econ Bull.

[53] Azmin M, Jafari A, Rezaei N (2018). An approach towards reducing road traffic injuries and improving public health through big data telematics: a randomised controlled trial protocol. Arch Iran Med.

[54] Fathollahi S, Saeedi Moghaddam S, Rezaei N (2019). Prevalence of behavioural risk factors for road-traffic injuries among the Iranian population: findings from STEPs 2016. Int J Epidemiol.

[55] Mohajer B, Azmin M, Mohebi F, Ahmadi N, Farzadfar F (2020). Low-quality domestic automobiles continue to threaten lives in Iran: economic instability as the potential contributor. Arch Iran Med.

[56] Soori H, Royanian M, Zali AR, Movahedinejad A (2009). Road traffic injuries in Iran: the role of interventions implemented by traffic police. Traffic Inj Prev.

[57] Peykari N, Hashemi H, Dinarvand R (2017). National action plan for non-communicable diseases prevention and control in Iran; a response to emerging epidemic. J Diabet Metab Dis.

[58] Baradaran-Seyed Z, Majdzadeh R (2013). Economic sanctions strangle Iranians' health, not just drug supply. Lancet.

[59] Danaei G, Harirchi I, Sajadi HS, Yahyaei F, Majdzadeh R (2019). The harsh effects of sanctions on Iranian health. Lancet.

[60] Doshmangir L, Bazyar M, Majdzadeh R, Takian A (2019). So near, so far: four decades of health policy reforms in Iran, achievements and challenges. Arch Iran Med.

[61] Azadnajafabad S, Saeedi Moghaddam S, Rezaei N (2021). A report on statistics of an online self-screening platform for COVID-19 and its effectiveness in Iran. Int J Health Policy Manag.

[62] WHO (2013).

[63] Di Cesare M, Khang Y-H, Asaria P (2013). Inequalities in non-communicable diseases and effective responses. Lancet.

[64] Maani N, Abdalla SM, Galea S (2021). Avoiding a legacy of unequal non-communicable disease burden after the COVID-19 pandemic. Lancet Diabetes Endocrinol.

