# Supplementary material for: Health system performance in Iran: a systematic analysis for the Global Burden of Disease Study 2019
Source: Lancet. 2022 Apr 23;399(10335):1625–45. doi: 10.1016/S0140-6736(21)02751-3 (PMC9023870; doi:10.1016/S0140-6736(21)02751-3)
Supplement: Supplementary appendix [file mmc1.pdf]

THE LANCET

Supplementary appendix

This appendix formed part of the original submission and has been peer reviewed. We post it as supplied by the authors.

Supplement to: GBD 2019 Iran Collaborators. Health system performance in Iran: a systematic analysis for the Global Burden of Disease Study 2019. *Lancet* 2022; published online April 6. https://doi.org/10.1016/S0140-6736(21)02751-3.

Supplementary appendix for "Health system performance in Iran: a systematic analysis for the Global Burden of Disease Study 2019"

This appendix provides supplemental background, methods, data resources, results, interpretation, and limitations.

Section 1, History of major health policies in Iran 2

Section 2, Methods 5

Section 3, Supplementary results 27

Section 4, Supplementary figures and tables 30

Section 5, Interpretation 1179

Section 6, Limitations 1180

Section 7, Author contributions..1185

Section 1

History of major health policies in Iran

Regarding universal health coverage (UHC), after improvement of the availability and accessibility of the health-care services, affordability was the next challenge. Despite multiple health insurance organisations in Iran, most of the Iranian population is covered by two public organisations, including the Iran Health Insurance Organization (IHIO) and Social Security Organisation, paying for 70 to 90% of outpatient (not all types) and inpatient services in public sectors.[1] Investigating the accessibility to health shows that there are over 981 hospitals throughout Iran, equalling 150 000 beds (1·9 per 1000 population), among which 62% are public presently.[2] Additionally, there are 51 public medical universities affiliated to Ministry of Health and Medical Education (MOHME) training more than 250 000 (3·1 per 1000 population) medical students throughout the country. There is a very well gender balance in health-care workforce, which has improved in recent decades in favour of females.[3] Finally, in the domain of research, the contribution of Iran to medical science production has increased during last 30 years and presently Iran ranks one of the leading countries in the Eastern Mediterranean region in terms of both quantity and quality of publications.[3]

Universal Rural Health Insurance (2004) was part of the referral system of Iranian health provider. This was amenable to establishment of Ministry of Welfare and Social Security and five-times increase in rural health-care budget. Family physicians, as the cornerstone of the primary health-care (PHC), were ought to primarily evaluate patients in the rural health centres, and if necessary, refer them to upper-level centres. This insurance was free for rural residents or small cities (<20 000 inhabitants) with no other insurance and covers all expenditures after successive referral. This reform improved the access to the hospital services and led to increase in the hospitalisation rate and the utilisation of hospital beds. Although, it makes access to secondary health-care for villagers dependent on access to PHC and family physicians.[1,4]

One of the main strategies to achieve UHC was Health Transformation Plan which was implemented in 2014. This was aimed for ensuring insurance coverage for all especially for those in underserved areas and reducing economic, social and cultural problems, with a dramatic decline in out-of-pocket expenditures. Based on the Ministry of Interior, about 10 million residents inhabit suburban locations. After lunching this act, health centres were created around slums and PHC services were made free of charge. Health Transformation Plan has led to decreased inequality and inaccessibility to health system goods while increasing the financial load on health

provider. Unavailability of financial resources in the last years resulted in shortage, and the act is almost dismissed.[5,6]

It is noteworthy that rural residents met their outpatient services via the PHC system, general practitioners, and family physicians, while urban counterparts mostly utilised private sectors before this transformation. Therefore, the 2014 Health Transformation Plan was planned to address this gap by the empowerment of the PHC services in urban areas as well. In this order, in 2016, the country invested 8·1% of gross domestic product (GDP) with a share of 38·8% out-of-pocket, besides the funds from the government, health insurance organisations, and other sources.[1] Several medical research infrastructures have been established for the first time in Iran including Golestan, Pars, PERSIAN, Tehran Lipid and Glucose Study (TLGS), and Behvarz Health Study, which are the largest prospective population-based cohorts in the region.[7-11] More than 100 chronic diseases registries were established during last 8 years.

To achieve UHC, Iran implemented several plans including PHC system by health houses, integration of the MOHME,[3] increasing the skilled human resources,[3] the National Maternal Mortality Surveillance System,[12] the rural health-care system reform,[3] family physician program,[13] and Health Transformation Plan;[3] however, international sanctions were notable drawbacks in 2011. In the past four decades, Iran successfully controlled the infectious diseases and now confronts the epidemic of non-communicable diseases (NCDs)[3,14-16] because of the aging population, urbanization, and sedentary lifestyle. In this regard, some programs adopted to tackle the transition toward NCDs. Establishment of Iranian Non-Communicable Diseases Committee (INCDC),[17] and the Iranian Package of Essential NCD interventions for primary health-care (IraPEN), and supreme council for health and food security in 2015 to develop the national action plan for combatting NCDs by emphasising the substantial role of the multi-sectoral approach.[18,19] Besides maintaining this functional network and addressing the inequities, this platform provides opportunities for widespread screening, diagnosis, and treatment of NCDs. It is crucial to evaluate the efficacy of the implemented strategies as well,[20] which is being addressed by performing national surveys like the WHO STEPwise approach to risk factor Surveillance (STEPS).[21]

Additionally, the MOHME conducted the National and Subnational Burden of Diseases, Injuries and Risk Factors (NASBOD) project that aimed to estimate levels and trends of the burden of disease at national and subnational levels from 1990 to 2015.[22] In this project, the available data from published and unpublished sources were analysed using statistical models such as Bayesian autoregressive multi-level[23] and spatiotemporal

regression,[24] which were discussed previously.[22] The NASBOD study presented the national and subnational pattern of causes of death from 1990 to 2015.[25]

The apparent outcomes of the constellation of socio-economic development and the health sector transformation were increase in life expectancy, decrease in fertility, and age-standardised mortality and morbidity rates at national level, which led to population growth and ageing that were reported in previous iterations of the Global Burden of Diseases, Injuries, and Risk Factors (GBD);[18,26-36] however, existing evidence implies unequal improvements and disparities in distribution of specific health indicators and risk factors across 31 provinces, e.g., higher child mortality rates in provinces close to the country borders,[37] divergences among subnational child cancer incidence rate,[38] and differences in the prevalence of obesity and overweight.[39] It is, thus, important to obtain geographically disaggregated data in order to disentangle the impact of developments achieved and policies made so far on the actual burden of diseases at local level.

Section 2

Methods

Data sources used in GBD 2019 Iran national and subnational estimation

Data type	Title	Time period covered	Coverage type	Contributors
Administrative data	Control of Epidemic Meningococcal Disease, WHO Practical Guidelines, 2nd Edition	01/1970 to 12/1996	Country	World Health Organization (WHO)
	Iran Communicable Diseases and Vaccination Coverage Surveillance 2006-2010	01/2006 to 12/2010	Country	Ministry of Health and Medical Education (Iran)
	Iran Hospital Data 2001-2010	01/2001 to 12/2010	Country	Ministry of Health and Medical Education (Iran)
	Iran Hospital Injury Sample Registry Tabulations 2005 as provided by the Global Burden of Disease 2010 Injury Expert Group	01/2005 to 12/2005	Country	Ministry of Health and Medical Education (Iran)
	Partially Hydrogenated Vegetable Oil Sales Database	01/1998 to 12/2010	Country	Euromonitor International
	Euromonitor Passport - Alcoholic Drinks Statistics	01/04/21	Global	Euromonitor International
	Euromonitor Passport - Cigarette Statistics	01/01/99	Global	Euromonitor International
	Euromonitor Passport - Dairy Market Statistics	01/02/21	Global	Euromonitor International
	Euromonitor Passport - Processed Meat and Seafood Market Statistics	01/02/21	Global	Euromonitor International
	Euromonitor Passport - Smoking Tobacco Statistics	01/04/21	Global	Euromonitor International
Census	Iran General Census of Population and Housing 2006	10/2006 to 11/2006	Country	Statistical Centre of Iran
	Iran General Census of Population and Housing 2006 - IPUMS	10/2006 to 11/2006	Country	Minnesota Population Center, University of Minnesota, Statistical Centre of Iran
	Iran National Population and Housing Census 2011	10/2011 to 11/2011	Country	Statistical Centre of Iran
	Iran Population and Housing Census 1956	11/01/56	Country	Department of Public Statistics, Ministry of Interior (Iran)
	Iran Population and Housing Census 1966	10/01/66	Country	Statistical Centre of Iran
	Iran Population and Housing Census 1986	10/01/86	Country	Statistical Centre of Iran
	Iran Population and Housing Census 1996	10/1996 to 11/1996	Country	Statistical Centre of Iran

Data type	Title	Time period covered	Coverage type	Contributors
	Iran Population and Housing Census 2011 - IPUMS	10/2011 to 11/2011	Country	Statistical Centre of Iran, Minnesota Population Center, University of Minnesota
	Iran Population and Housing Census 2016	09/2016 to 11/2016	Country	Statistical Centre of Iran
	Iran Population Estimates 2014	01/2014 to 12/2014	Country	
**Disease registry**	Iran National Cancer Registry 2000-2010	01/2000 to 12/2010	Country	Center for Disease Control and Prevention, Ministry of Health and Medical Education (Iran)
	Iran National Cancer Registry Report 2003-2004	03/2003 to 02/2004	Country	Center for Disease Control and Prevention, Ministry of Health and Medical Education (Iran)
	Iran National Cancer Registry Report 2004-2005	03/2004 to 02/2005	Country	Center for Disease Control and Prevention, Ministry of Health and Medical Education (Iran)
	Iran National Cancer Registry Report 2005-2006	03/2005 to 02/2006	Country	Center for Disease Control and Prevention, Ministry of Health and Medical Education (Iran)
	Iran National Cancer Registry Report 2006-2007	03/2006 to 02/2007	Country	Center for Disease Control and Prevention, Ministry of Health and Medical Education (Iran)
	Iran National Cancer Registry Report 2008-2009	03/2008 to 02/2009	Country	Center for Disease Control and Prevention, Ministry of Health and Medical Education (Iran)
	Iran National Cancer Registry Report 2009-2010	03/2009 to 03/2010	Country	Center for Disease Control and Prevention, Ministry of Health and Medical Education (Iran)
	United States Renal Data System Annual Data Report 2015	01/2015 to 12/2015	Country	United States Renal Data System Coordinating Center
	International Clearinghouse for Birth Defects Surveillance and Research Annual Report 2006-2014	01/1974 to 12/2012	Global	International Clearinghouse for Birth Defects Surveillance and Research
	International Incidence of Childhood Cancer Volume III	01/1982 to 12/2015	Global	International Agency for Research on Cancer (IARC), World Health Organization (WHO), International Association of Cancer Registries (IACR)
	Iran - Ardabil Cancer Registry Extracts 1985-2008	01/1985 to 12/2008	Subnational	Digestive Diseases Research Center (Iran), Ardabil University of Medical Sciences, International Agency for Research on Cancer (IARC)
	Iran - Golestan Cancer Registry 2005-2007 - CI5	01/2005 to 12/2007	Subnational	Center for Disease Control and Prevention, Ministry of Health and Medical Education (Iran), Digestive Diseases Research Center (Iran), Golestan University of Medical Sciences (Iran)

Data type	Title	Time period covered	Coverage type	Contributors
	Iran - Golestan Cancer Registry Incidence Data 2006-2008	01/2006 to 12/2008	Subnational	Center for Disease Control and Prevention, Ministry of Health and Medical Education (Iran), Digestive Diseases Research Center (Iran), Golestan University of Medical Sciences (Iran)
	Iran - Tabriz Registry of Congenital Anomalies Data 2006 -2012 - ICBDSR	01/2006 to 12/2012	Subnational	
**Environmental monitoring**	Indoor Radon Level Measurements in Iran Using AEOI Passive Dosimeters	02/1986 to 09/1987	Country	
	Iran Air Quality Monitoring System Particulate Matter Data 2010-2014	01/2010 to 12/2014	Country	Department of the Environment (Iran)
	The Battle Against Solid UFP-emissions from Internal Combustion Engines Without Waiting for Euro VI	03/2014 to 03/2015	Country	Sharif University of Technology (SUT), Air Quality Control Company (Iran)
	Iran - Tehran Air Quality Control Company Particulate Matter Data 2010-2016	01/2010 to 12/2016	Subnational	Air Quality Control Company (Iran)
	Iran - Tehran Air Quality PM2.5 and PM10 Data 2005, 2010, 2012	01/2005 to 12/2012	Subnational	Air Quality Control Company (Iran), Department of the Environment (Iran)
	Iran Air Quality PM2.5 and PM10 Data 2005, 2008-2013	01/2005 to 12/2013		Department of the Environment (Iran)
**Epi surveillance**	Iran Global AIDS Response Progress Reporting (GARPR) System - Antenatal Care Attendees Positive for Syphilis	01/05/21	Country	World Health Organization (WHO), Joint United Nations Program on HIV/AIDS (UNAIDS), United Nations Children's Fund (UNICEF)
	Iran WHO Leishmaniasis Country Profile 1994-2010	01/1994 to 12/2010	Country	World Health Organization (WHO)
	Iran WHO Leishmaniasis Country Profile 2014	01/1998 to 12/2014	Country	World Health Organization (WHO)
	Leishmaniasis in the World Health Organization Eastern Mediterranean Region	01/1989 to 12/2008	Country	
	Progress towards the elimination of leprosy as a public health problem - Weekly Epidemiological Record 1993	01/1989 to 12/1993	Country	World Health Organization (WHO)
	WHO Global Health Observatory - Antenatal Care Attendees Tested for Syphilis Data by Country	01/10/21	Global	World Health Organization (WHO)
	WHO Global Health Observatory Interactive Graph - Number of Cases of Cutaneous Leishmaniasis Reported 1998-2015	01/1998 to 12/2015	Global	World Health Organization (WHO)
	WHO Global Health Observatory Interactive Graph - Number of Cases of Visceral Leishmaniasis Reported 1998-2015	01/1998 to 12/2015	Global	World Health Organization (WHO)
	WHO Tuberculosis Case Notifications	01/01/80	Global	World Health Organization (WHO)

Data type	Title	Time period covered	Coverage type	Contributors
	Infection with coronavirus in camels, Iran. Immediate notification : 29/10/2014	10/2014 to 07/2015	Subnational	World Organisation for Animal Health (France)
	EMRO STI Database, Personal Communication with S. Nabai 2009	01/2009 to 12/2009		
	Iran Sexually Transmitted Disease Prevalence Data 2005, Personal Communication with S. Nabai	01/2005 to 12/2005		
**Estimate**	Low- and Middle-Income Country Child Growth Failure Geospatial Estimates 2000-2017	01/2000 to 12/2017	Country	Institute for Health Metrics and Evaluation (IHME)
	Low- and Middle-Income Country Childhood Overweight and Wasting Geospatial Estimates 2000-2017	01/2000 to 12/2017	Country	Institute for Health Metrics and Evaluation (IHME)
	Low- and Middle-Income Country Educational Attainment Geospatial Estimates 2000-2017	01/2000 to 12/2017	Country	Institute for Health Metrics and Evaluation (IHME)
	Low- and Middle-Income Country Household Air Pollution Geospatial Estimates 2000-2018	01/2000 to 12/2018	Country	Institute for Health Metrics and Evaluation (IHME)
	Low- and Middle-Income Country MCV1 Coverage Geospatial Estimates 2000-2019	01/2000 to 12/2019	Country	Institute for Health Metrics and Evaluation (IHME)
	Low- and Middle-Income Country Neonatal, Infant, and Under-5 Mortality Geospatial Estimates 2000-2017	01/2000 to 12/2017	Country	Institute for Health Metrics and Evaluation (IHME)
	Middle East Respiratory Syndrome Coronavirus Environmental Suitability and Spillover Potential Geospatial Estimates	01/2016 to 12/2016	Country	Institute for Health Metrics and Evaluation (IHME)
	Modell's Haemoglobinopathologist's Almanac	01/1990 to 12/2003	Country	Centre for Health Informatics and Multiprofessional Education, University College London
	Sickle Cell and Thalassemias Prevalence Data, Personal Correspondence with David Weatherall	01/1990 to 12/2009		
**Event data**	2015 Mina Stampede	09/15/21	Country	Wikipedia
	Armed Conflict Location and Event Dataset, Realtime - Robert S. Strauss Center	01/1997 to 02/2020	Country	Climate Change and African Political Stability Project (CCAPS)
	Iran Hazard Profile - Global Assessment Report 2009	01/1986 to 12/2007	Country	United Nations Environment Programme
	Iran Surveys Damage After Cyclone	06/07/21	Country	United Press International (UPI)
	Iran Twelve Percent Increase in Drownings in 2017	01/2017 to 12/2017	Country	National Forensic Medicine Organization (Iran), Iranian Legal Medicine Organization
	Chicago Project on Security and Terrorism Suicide Attack Database	11/1982 to 09/2018	Global	Department of Political Science, University of Chicago
	Iran Aseman Airlines Flight 3704 crash (18 February 2018)	02/12/21	Subnational	Aviation Safety Network

Data type	Title	Time period covered	Coverage type	Contributors
	Iran: death toll from protests crackdown exceeds 200	12/19/21	Subnational	Amnesty International
	Kermanshah Earthquake Death Toll Hit 620 People	11/17/21	Subnational	Iran Online
	Southwest Iran hit hard by flooding, evacuation underway in Ahvaz	03/2019 to 04/2019	Subnational	Reuters
	Are Iranian Fighter Deaths Soaring in Syria?	01/2012 to 11/2016		
Report	Characterizing the HIV/AIDS Epidemic in the Middle East and North Africa: Time for Strategic Action	01/1990 to 12/2009	Country	World Bank
	Iran - National Estimates for Maternal Mortality: An Analysis Based on Reproductive Age Mortality Study (RAMOS) in Iran	01/1996 to 12/1996	Country	
	Iran AIDS Progress Report 2012	01/2010 to 12/2011	Country	Joint United Nations Program on HIV/AIDS (UNAIDS), Ministry of Health and Medical Education (Iran)
	Iran Epidemiological Fact Sheet on HIV/AIDS and STIs 2004	01/1992 to 12/2003	Country	Joint United Nations Program on HIV/AIDS (UNAIDS), United Nations Children's Fund (UNICEF), World Health Organization (WHO)
	Iran Maternal Mortality Report 2012-2013	01/2002 to 12/2012	Country	Ministry of Health and Medical Education (Iran)
	Iran National Health Accounts 1997-1998	01/1988 to 12/1998	Country	World Bank, Statistical Centre of Iran
	Iranian Legal Medicine Organization Annual Report 2014	01/2001 to 12/2014	Country	Iranian Legal Medicine Organization
	Operational Research in Tropical and Other Communicable Diseases: Final Report Summaries 2003-2004	01/2003 to 12/2006	Country	World Health Organization Regional Office for the Eastern Mediterranean (EMRO-WHO)
	Report of the Consultative Meeting on Cutaneous Leishmaniasis	01/2006 to 12/2007	Country	World Health Organization (WHO)
	Situation Analysis of Sexually Transmitted Infections In the Islamic Republic of Iran	01/1998 to 12/2006	Country	Center for Disease Control and Prevention, Ministry of Health and Medical Education (Iran)
	The Work of the WHO in the Eastern Mediterranean 2008	01/2008 to 12/2008	Country	World Health Organization Regional Office for the Eastern Mediterranean (EMRO-WHO)
	Characterizing the HIV/AIDS Epidemic in the Middle East and North Africa 2010	01/1987 to 12/2009	Global	International Bank for Reconstruction and Development, World Bank (WB)
	Global Report on Antimalarial Efficacy and Drug Resistance 2000-2010	01/2000 to 06/2010	Global	World Health Organization (WHO)

Data type	Title	Time period covered	Coverage type	Contributors
	Guidebook on Promotion of Sustainable Energy Consumption: Consumer Organizations and Efficient Energy Use in the Residential Sector	01/1983 to 12/1998	Global	United Nations Economic and Social Commission for Asia and the Pacific (ESCAP)
	Susceptibility of Plasmodium Falciparum to Antimalarial Drugs: Report on Global Monitoring 1996-2004	01/1996 to 12/2004	Global	Roll Back Malaria Partnership, World Health Organization (WHO)
	United Nations Statistical Yearbook for Asia and the Pacific 2014	01/1990 to 12/2014	Global	United Nations Economic and Social Commission for Asia and the Pacific (ESCAP)
	Iran Community-Based Pilot Study for Primary Prevention of the Major Noncommunicable Diseases in Qazvin and Abhar 2001	01/2001 to 12/2001	Subnational	Qazvin University of Medical Sciences, Zanjan University of Medical Sciences
	Iran Multi-center Study on Iron Deficiency Anemia Among 15 to 49 Year Old Women	01/1994 to 12/1995		Ministry of Health and Medical Education (Iran), United Nations Children's Fund (UNICEF)
	Iran Water and Sanitation Data 2001	01/2001 to 12/2001		
	Iran Water and Sanitation Data 2006	01/2006 to 12/2006		
**Scientific literature**	A national study of the prevalence of autism among five-year-old children in Iran	01/2006 to 12/2009	Country	
**Survey**	Epidemiology of Drug Use in Iran	01/1943 to 12/2004	Country	United Nations Office on Drugs and Crime (UNODC)
	Iran Annual Parasite Incidence Data, Personal Communication with M. Naghavi 2017	01/2017 to 12/2017	Country	
	Iran Anthropometric Nutritional Indicators Survey 1998	01/1998 to 12/1998	Country	Ministry of Health and Medical Education (Iran)
	Iran Anthropometric Nutritional Indicators Survey 2004	01/2002 to 12/2004	Country	Ministry of Health and Medical Education (Iran)
	Iran Demographic and Health Survey 2000	01/2000 to 12/2000	Country	Ministry of Health and Medical Education (Iran), Statistical Centre of Iran
	Iran EPI Cluster Survey 1995	01/1995 to 12/1995	Country	Ministry of Health and Medical Education (Iran)
	Iran EPI Coverage Survey 1984	01/1984 to 12/1984	Country	
	Iran Global Youth Tobacco Survey 2003	01/2003 to 12/2003	Country	Centers for Disease Control and Prevention (CDC), World Health Organization (WHO)
	Iran Global Youth Tobacco Survey 2007	01/2007 to 12/2007	Country	Centers for Disease Control and Prevention (CDC), World Health Organization (WHO)
	Iran Health and Disease Survey 1991	01/1991 to 12/1991	Country	Ministry of Health and Medical Education (Iran)

Data type	Title	Time period covered	Coverage type	Contributors
	Iran Health and Disease Survey 1997-1999	01/1997 to 12/1999	Country	Ministry of Health and Medical Education (Iran)
	Iran Knowledge, Attitudes, and Practices Survey 1992	01/1992 to 12/1992	Country	
	Iran Knowledge, Attitudes, and Practices Survey 1997	01/1997 to 12/1997	Country	
	Iran Mortality and Fertility Survey 1984	01/1984 to 12/1984	Country	Ministry of Health and Medical Education (Iran)
	Iran Mortality and Fertility Survey 1996	01/1996 to 12/1996	Country	Ministry of Health and Medical Education (Iran)
	Iran Multiple Indicator Cluster Survey 1995	09/01/95	Country	Ministry of Health and Medical Education (Iran), United Nations Children's Fund (UNICEF), Statistical Centre of Iran
	Iran Multiple Indicator Cluster Survey 1997	01/1997 to 12/1997	Country	United Nations Children's Fund (UNICEF), Ministry of Health and Medical Education (Iran), Statistical Centre of Iran
	Iran Multiple Indicator Cluster Survey 2000	08/2000 to 09/2000	Country	United Nations Children's Fund (UNICEF)
	Iran Multiple Indicator Demographic and Health Survey 2010	01/2010 to 12/2010	Country	Ministry of Health and Medical Education (Iran), Statistical Centre of Iran
	Iran Multi-Round Population Survey 1991	01/1991 to 12/1991	Country	Statistical Centre of Iran
	Iran National Comprehensive Study on Household Food Consumption and Nutritional Status 1991-1995	01/1991 to 12/1995	Country	National Nutrition and Food Technology Research Institute (Iran)
	Iran National Comprehensive Study on Household Food Consumption Pattern and Nutritional Status 2001-2003	01/2001 to 12/2003	Country	Institute of Nutrition and Food Technology, Shaheed Beheshti Medical University (Iran), Ministry of Health and Medical Education (Iran)
	Iran National Integrated Micronutrient Survey 2001	01/2001 to 12/2001	Country	United Nations Children's Fund (UNICEF), Pasteur Institute of Iran, Institute of Nutrition and Food Technology, Shaheed Beheshti Medical University (Iran)
	Iran National Integrated Micronutrient Survey 2012	05/2012 to 05/2013	Country	Tehran University of Medical Sciences, United Nations Children's Fund (UNICEF), Ministry of Health and Medical Education (Iran)
	Iran Plasmodium Falciparum Parasite Rate Data 2002-2015, Personal Communication with M. Naghavi	01/2002 to 12/2015	Country	

**Data type**	**Title**	**Time period covered**	**Coverage type**	**Contributors**
	Iran Population and Health Sample Survey 1974	01/1974 to 12/1974	Country	National Institute of Health Research, Ministry of Health and Medical Education (Iran), Tehran University
	Iran Population Growth Survey 1973-1976	01/1973 to 12/1976	Country	Statistical Centre of Iran
	Iran Population Growth Survey 1998	01/1998 to 12/1998	Country	Statistical Centre of Iran
	Iran Progress in International Reading Literacy Study 2001	04/2001 to 11/2001	Country	International Association for the Evaluation of Educational Achievement (IEA), TIMSS & PIRLS International Study Center, Boston College, Statistics Canada (StatCan), National Foundation for Educational Research (NFER) (UK), Educational Testing Service (ETS)
	Iran Progress in International Reading Literacy Study 2005-2006	10/2005 to 06/2006	Country	International Association for the Evaluation of Educational Achievement (IEA), TIMSS & PIRLS International Study Center, Boston College, Statistics Canada (StatCan), National Foundation for Educational Research (NFER) (UK), Educational Testing Service (ETS)
	Iran Progress in International Reading Literacy Study 2010-2011	10/2010 to 06/2011	Country	International Association for the Evaluation of Educational Achievement (IEA), TIMSS & PIRLS International Study Center, Boston College, Statistics Canada (StatCan), Educational Testing Service (ETS)
	Iran Prospective Urban and Rural Epidemiological Study	01/02/21	Country	Population Health Research Institute (PHRI), McMaster University (Canada), Hamilton Health Sciences
	Iran Routine EPI Reporting 1987	01/1987 to 12/1987	Country	
	Iran Routine EPI Reporting 1989	01/1989 to 12/1989	Country	
	Iran Routine EPI Reporting 1990	01/1990 to 12/1990	Country	
	Iran STEPS Noncommunicable Disease Risk Factors Survey 2005-2009	01/2005 to 12/2009	Country	World Health Organization (WHO), Ministry of Health and Medical Education (Iran)

Data type	Title	Time period covered	Coverage type	Contributors
	Iran STEPS Noncommunicable Disease Risk Factors Survey 2011	05/2011 to 06/2011	Country	Ministry of Health and Medical Education (Iran)
	Iran STEPS Noncommunicable Disease Risk Factors Survey 2016	04/2016 to 11/2016	Country	Ministry of Health and Medical Education (Iran), Tehran University of Medical Sciences, National Institute of Health Research, Ministry of Health and Medical Education (Iran)
	Iran Trends in International Mathematics and Science Study 2010-2011	10/2010 to 06/2011	Country	International Association for the Evaluation of Educational Achievement (IEA), Statistics Canada (StatCan), TIMSS & PIRLS International Study Center, Boston College, Educational Testing Service (ETS)
	Iran WHO Multi-country Survey Study on Health and Health System Responsiveness 2000-2001	01/2000 to 12/2001	Country	World Health Organization (WHO), Ministry of Health and Medical Education (Iran)
	Iran World Malaria Report Foci Data, Personal Communication with M. Naghavi 2017	01/2015 to 12/2017	Country	
	Iran World Poll 2005-2006	01/2005 to 12/2005	Country	Gallup
	Iran World Poll 2007	01/2007 to 12/2007	Country	Gallup
	Iran World Poll 2008	01/2008 to 12/2008	Country	Gallup
	Iran World Poll 2011-2018	01/2011 to 12/2018	Country	Gallup
	Nutritional Status of Rural Iranian Children	01/1980 to 12/1980	Country	
	WHO Global Health Observatory - Population Living in Trachoma Endemic Areas	01/05/21	Global	World Health Organization (WHO)
	Effects of Vaginal Candidiasis and Trichomoniasis on Newborn	01/2001 to 12/2002	Subnational	
	Iran - Golestan Cohort Study 2004-2008 - Teheran University of Medical Sciences	01/2004 to 12/2008	Subnational	Tehran University of Medical Sciences, National Cancer Institute (NCI) (United States), International Agency for Research on Cancer (IARC)
	Iran - Kordestan Rapid Assessment of Avoidable Blindness 2014	01/2014 to 12/2014	Subnational	International Centre for Eye Health (ICEH)
	Iran - Pars Cohort Study	01/13/21	Subnational	Digestive Diseases Research Institute (DDRI) (Iran), Shiraz University of Medical Sciences

Data type	Title	Time period covered	Coverage type	Contributors
	Iran - Tehran Blood Pressure Study 1990-1991	01/1990 to 12/1991	Subnational	Tehran University
	Iran - Tehran International Society of Nephrology Kidney Disease Data Center Chronic Kidney Disease and Cardiovascular Risk Survey 2007	10/2007 to 12/2007	Subnational	International Society of Nephrology (ISN)
	Iran - Tehran Lipid and Glucose Study 1999-2000	02/1999 to 05/2000	Subnational	Shahid Beheshti University of Medical Sciences
	Iran - Varamin Rapid Assessment of Avoidable Blindness 2009	02/2009 to 08/2009	Subnational	International Centre for Eye Health (ICEH)
	Iran Malaria Indicator Survey 2009	08/09/21	Subnational	Ministry of Health and Medical Education (Iran), World Health Organization (WHO), Zahedan University of Medical Sciences (Iran), Hormozgan University of Medical Sciences (Iran), Kerman University of Medical Sciences (Iran), United Nations Development Programme (UNDP)
	Iran Persian Gulf Healthy Heart Study (PGHHS) Phase I 2003-2004	01/2003 to 12/2004	Subnational	Bushehr University of Medical Sciences
	Iran Rapid Nutritional Assessment of 0 to 5 Year Old Kurdish Refugee Children	05/01/91	Subnational	Institute of Nutrition and Food Technology, Shaheed Beheshti Medical University (Iran)
	Association of Trichomonas Vaginalis with Low Birth Weight	01/2009 to 12/2010		
	COVID-19 Health Services Disruption Survey 2020			Institute for Health Metrics and Evaluation (IHME), Bill and Melinda Gates Foundation (BMGF), Premise Data Corporation, Ipsos, ORB International
**Vital registration**	Human Fertility Collection - ASFR, original age scale	01/1751	Country	Max Planck Institute for Demographic Research, Vienna Institute of Demography
	Human Fertility Collection - Total Fertility Rate and Mean Age at Birth	01/1751	Country	Max Planck Institute for Demographic Research, Vienna Institute of Demography
	Iran Civil Registration Recorded Births 1958-2015	03/1958 to 03/2015	Country	National Organization for Civil Registration (Iran)
	Iran Civil Registration Recorded Deaths 2011-2016	01/2011 to 12/2016	Country	National Organization for Civil Registration (Iran)
	Iran Maternal Mortality Surveillance 2010-2014	01/2010 to 12/2014	Country	Ministry of Health and Medical Education (Iran)
	Iran Vital Registration - Deaths 1974 ICD8	01/1974 to 12/1974	Country	World Health Organization (WHO)

Data type	Title	Time period covered	Coverage type	Contributors
	Iran Vital Registration - Deaths 1975 ICD8	01/1975 to 12/1975	Country	World Health Organization (WHO)
	Iran Vital Registration - Deaths 1978 ICD8	01/1978 to 12/1978	Country	World Health Organization (WHO)
	Iran Vital Registration - Deaths 1979 ICD8	01/1979 to 12/1979	Country	World Health Organization (WHO)
	Iran Vital Registration - Deaths 1980 ICD8	01/1980 to 12/1980	Country	World Health Organization (WHO)
	Iran Vital Registration - Deaths 1981 ICD8	01/1981 to 12/1981	Country	World Health Organization (WHO)
	Iran Vital Registration - Deaths 1982 ICD8	01/1982 to 12/1982	Country	World Health Organization (WHO)
	Iran Vital Registration - Deaths 1983 ICD8	01/1983 to 12/1983	Country	World Health Organization (WHO)
	Iran Vital Registration - Deaths 1984 ICD8	01/1984 to 12/1984	Country	World Health Organization (WHO)
	Iran Vital Registration - Deaths 1985 ICD8	01/1985 to 12/1985	Country	World Health Organization (WHO)
	Iran Vital Registration - Deaths 1987 ICD8	01/1987 to 12/1987	Country	World Health Organization (WHO)
	Iran Vital Registration - Deaths 1996-2001	01/1996 to 12/2001	Country	Ministry of Health and Medical Education (Iran)
	Iran Vital Registration - Deaths 2007-2010	01/2007 to 12/2010	Country	Ministry of Health and Medical Education (Iran)
	Iran Vital Registration - Deaths 2011-2012	03/2011 to 03/2012	Country	Ministry of Health and Medical Education (Iran)
	Iran Vital Registration - Deaths 2013 ICD10	01/2013 to 12/2013	Country	World Health Organization (WHO)
	Iran Vital Registration - Deaths 2014 ICD10	01/2014 to 12/2014	Country	World Health Organization (WHO)
	Iran Vital Registration - Deaths 2015 ICD10	01/2015 to 12/2015	Country	World Health Organization (WHO)
	Iran Vital Registration Birth Data 1991	01/1991 to 12/1991	Country	
	Iran Vital Registration Death Data 1983	01/1983 to 12/1983	Country	

Data type	Title	Time period covered	Coverage type	Contributors
	Iran Vital Registration Death Data 1984	01/1984 to 12/1984	Country	
	Iran Vital Registration Death Data 1986	01/1986 to 12/1986	Country	
	Iran Vital Registration Death Data 1991	01/1991 to 12/1991	Country	
	Iran Vital Registration Death Data 2011	01/2011 to 12/2011	Country	
	Iran Vital Registration Death Data 2012	01/2012 to 12/2012	Country	
	Iran Vital Registration Death Data 2013	01/2013 to 12/2013	Country	
	Iran Vital Registration Live Births Data 1950-1959	01/1950 to 12/1959	Country	
	Iran Vital Registration Live Births Data 1961-1990	01/1961 to 12/1990	Country	
	Iran Vital Registration Live Births Data 1991	01/1991 to 12/1991	Country	
	Iran Vital Registration Live Births Data 1994	01/1994 to 12/1994	Country	
	Iran Vital Registration Live Births Data 1996	01/1996 to 12/1996	Country	
	Iran Vital Statistics - Deaths 2000-2006	01/2000 to 12/2006	Country	Ministry of Health and Medical Education (Iran)
	Iran Death Registration System			Ministry of Health and Medical Education (Iran)

Explanation of methods

Geographical location

GBD produced estimates for 204 countries, including numbers and rates of disability-adjusted life-years (DALYs), incidence, prevalence, and years lived with disability (YLDs) from 1990 to 2019, and deaths and years of life lost (YLLs) between 1980 and 2019. GBD 2019 defined standard and non-standard locations, where standard locations are the set of all subnational locations belonging to countries with high-quality data and a population of over 200 million. Therefore, Iran is among the group of non-standard locations.[40]

Cause list

The GBD 2019 presented the estimation at four levels of causes. At Level 1, there are three cause groups: communicable, maternal, neonatal, and nutritional diseases (Group 1); non-communicable diseases (Group 2); and injuries (Group 3), which further disaggregate to subgroups in the following levels. The disaggregation into Levels 3 and 4 contains the most acceptable level of detail for causes captured in GBD 2019. GBD 2019 reported 286 causes of death and 364 non-fatal causes, where the total number of causes is 369. In the present study, we discussed the estimates through the levels one, two, and three of causes. Subnational estimation in GBD 2019 includes Iran at the first level of administrative organisation within the country. All subnational estimates were incorporated into model development and evaluation as part of GBD 2017.[40]

Data input sources and cause of death (CoD) database

GBD 2019 applies a growing number of data input sources, including surveys, censuses, vital statistics, and other health-related data sources. In this study, we extracted and illustrated the data sources for Iran in the data source table in this section. Also, all citations for specific GBD components, causes and risks, and locations are accessible through the Data Input Sources Tool in GHDx: http://ghdx.healthdata.org/gbd-2019/data-input-sources.[40]

Seven types of data sources were used in the CoD database, including vital registration (VR), verbal autopsy (VA), cancer registry, police records, sibling history, surveillance, survey/census, and minimally invasive tissue sample (MITS) diagnoses. In countries with complete VR systems, there is no need to use any other data source. VR data are mainly obtained from the World Health Organization (WHO) Mortality Database and country-specific mortality databases operated by official offices. In countries with less developed VR systems or

inadequate VR, surveys and censuses are often used. All available data on CoD data are standardised and entered into a single database. Then through twelve further steps, the CoD database is prepared for modeling and generating cause-specific mortality estimates by age, sex, year, and geography. We included various known biases in different categories of input data to reach the most precious estimates.[40]

CoD modeling methods

GBD used the cause of death ensemble modeling (CODEm) framework to model most cause-specific death rates in the GBD, which consisted of four components. First, all data were identified, which might have different quality but all contained some signal of the true epidemiological process. They were gathered to be used in the modeling process. Second, four groups of statistical models were incorporated including, linear mixed effects regression (LMER) models of the natural log of the cause-specific death rate, LMER models of the logit of the cause fraction, spatiotemporal Gaussian process regression (ST-GPR) models of the natural logarithm of the cause-specific death rate, and ST-GPR models of the logit of the cause fraction. To use GPR data variance is needed, which is calculated by the sum of sampling variance, non-sampling variance, and garbage code redistribution variance.

Afterward, the out-of-sample predictive validity is applied to assess the performance of all individual models, which are then weighted according to the performance rank. Furthermore, the relative weight of each model is calculated using a parameter ψ, which shows how fast the weight of a model reduces when the rank declines. Afterward, differently weighted combinations of individual models according to their rank and ψ value are evaluated to select the ensemble model with the highest out-of-sample predictive validity.

Once a weighting scheme has been selected, 1000 draws are made that is proportionally contributed to each model based on its weight. The mean of the draws is used as the final point estimate for this model, and a 95% uncertainty interval (UI) is calculated as the 25th and 975th ordered quantiles of these 1000 draws. The validity of the UI is its coverage of the out-of-sample data; the most ideal 95% UI is the one that captures 95% of these data.

Some causes were not compatible with CODEm estimation infrastructure and processes due to the absence of enough cause-specific death records and considerable biases, which resulted in unstable out-of-sample predictive validity testing. Therefore, other techniques including, negative binomial models, natural history models, sub-cause proportion models, and prevalence-based models were applied in such cases. The detail of these models is discussed in previous studies.[40] Since the CoD models are cause-specific, and the sum of results

will not always equal to the all-cause mortality estimates, the GBD applied CoDCorrect, which is an algorithm to provide internal consistency for CoD and all-cause mortality estimates.[40]

YLLs estimation

To estimate YLL, the GBD first estimated the lowest observed age-specific mortality rate by sex in any location in the world with a population greater than 5 million in 2016 to establish a theoretical minimum risk reference life table. Then, YLLs were calculated by multiplying the number of estimated deaths for a specific cause by the standard life expectancy at the age of death.[40]

Non-fatal estimation (incidence, prevalence, and YLDs)

Non-fatal estimation is the process of incidence, prevalence, and YLDs calculation. According to GBD 2019, this estimation is performed through eight steps, including data identification and extraction, data adjustment, estimation of prevalence and incidence by cause and sequelae, impairment estimation, severity distributions, incorporation of disability weights (DWs), comorbidity adjustment, and the estimation of YLDs by sequelae and causes.

The data sources to estimate non-fatal outcomes in GBD 2019 are provided in searchable form through a web tool (http://ghdx.healthdata.org/). Prevalence and incidence were estimated by using DisMod-MR 2.1 or alternative modeling strategies for selected cause groups. The Bayesian meta-regression method DisMod-MR 2.1 also was the most extensively used method for estimating sequelae, but for some causes such as HIV/AIDS or measles, disease-specific natural history models have been used. Some changes were made to DisMod-MR 2.1 in the GBD 2019, which are described in detail elsewhere.[40]

GBD 2019 estimated cause-specific YLDs by multiplying the prevalence of each cause and sequela by the DW for that cause. DW implies the magnitude of health loss associated with the outcome and is measured on a scale from 0 to 1, which are equivalent to full health, and death, respectively. The process of measuring DW is fully discussed previously, as it has improved since 2010. Furthermore, as the final step of YLD estimation, it is adjusted for each comorbidity through a micro-simulation process named “COMO” (for comorbidity correction). The YLDs uncertainty intervals incorporate uncertainty in prevalence and uncertainty in the DW, assuming that there is no correlation between these uncertainties. The 95% UI is then reported as the 25th and 975th quartile of the distribution.[40]

DALYs estimation

DALYs are calculated as the sum of YLLs and YLDs for each age, sex, location, and year. Assuming that the uncertainty in YLLs was independent of uncertainty in YLDs, the 95% UI was calculated as the 25th and 975th ordered draw of the uncertainty distribution.[40] Uncertainty from all of these sources is propagated into the final quantities of interest by taking 1000 draws from the posterior distribution of each component quantity of interest.

Attributable burden of risk factors

The GBD 2019 estimation of attributable burden is based on a general framework established for comparative risk assessment (CRA). CRA consists of six steps; risk–outcome pairs inclusion; modeling relative risk as a function of exposure; calculating exposure levels and distributions; determining the level of exposure with minimum risk called the theoretical minimum risk exposure level (TMREL); estimation of population attributable fractions and attributable burden; and estimation of the burden attributable to various combinations of risk factors. This process is explained in greater detail in the prior publications.[41]

Age-standardised estimations

Age-standardised populations in the GBD were calculated by using the GBD world population age standard. For GBD 2013, GBD 2015, and GBD 2016, the age-specific proportional distributions of all national locations from the UN Population Division World Population Prospects 2012 revision for all years from 2010 to 2035 were used to generate a standard population age structure by using the non-weighted mean across all the aforementioned country-years. For GBD 2017, we used the non-weighted mean of 2017 age-specific proportional distributions from the GBD 2017 population estimates for all national locations with a population greater than 5 million people in 2017 to generate an updated standard population age structure. For GBD 2019, we have continued to use this method using GBD 2019 population estimates.

Socio-demographic index (SDI)

The SDI is an index that incorporates total fertility rate in women younger than 25 years, lag-distributed income per capita, and average years of education for those ages 15 and older. As a composite variable it presents the theoretical level of development relevant to health which ranges from 0 (minimum level) to 1 (theoretical maximum level).[42]

Life expectancy decomposition

To identify the cause-specific contributions to changes in life expectancy, life expectancy decomposition was utilised. To achieve the goal, there are three steps:

1. First the difference in life expectancy must be decomposed by age. The result is labelled $\delta x \delta x$ for each age group x.

2. Then, each age-specific contribution $\delta x \delta x$ is further decomposed into cause-age-specific contributions.

3. Lastly, the cause-age-specific contributions are aggregated across age to produce cause-specific contributions to the difference in life expectancy.

- Decomposition by Age

Andreev, Shkolnikov, and Begun (2002) describe a technique for calculating age specific contributions to differences in life expectancy. They claim that this technique is essentially equivalent to Pollard (1982) and Arriaga (1984), in that they're all just "particular forms of a single general algorithm that includes stepwise replacement of elements from one vector of age-specific mortality rates by respective elements of another vector.".[43,44]

Given two sets of age specific all cause mortality

$$\mu_{1}\rightarrow_{[1]}=(\mu_{[1]1},\ldots,\mu_{[1]\alpha})\mu 1\rightarrow[1]=(\mu 1[1],\ldots,\mu\alpha[1])$$

and

$$\mu_{1}\rightarrow_{[2]}=(\mu_{[2]1},\ldots,\mu_{[2]\alpha})\mu 1\rightarrow[2]=(\mu 1[2],\ldots,\mu\alpha[2]),$$

one would like to know how the difference in all cause mortality for age group xx contributes to the change in life expectancy between the two populations.

To do this, Andreev et al. use a mortality zipper that zips together the age specific all cause mortality at each age group, in order. This mortality zipper is:

$$M_{[x]}\rightarrow:=\{\mu_{[2]y}\mu_{[1]y}\text{ if } y<x \text{ if } y\geq x M[x]\rightarrow:=\{\mu y[2] \text{ if } y<x\mu y[1] \text{ if } y\geq x$$

Then, one may define the difference $\delta^{2-1}_{0|x} = e_0(M_{[x]}) - e^1_0$ as a contribution of ages from $y<x$ to the overall difference $e^2_0 - e^1_0$.

Taking $L_{0|x} = \int_0^x l_t \, dt$, we have

$$\delta^{2-1}_{0|x} = e_0(M_{[x]}) - e^1_0 = (L^2_{0|x} - L^1_{0|x}) + (l^2_x - l^1_x)\, e^1_x \qquad \delta^{2-1}_{0|x+n} = (L^2_{0|x+n} - L^1_{0|x+n}) + (l^2_{x+n} - l^1_{x+n})\, e^1_{x+n}$$

where n is the duration of the age group.

Then Andreev et al. define the contribution of age interval $[x, x+n)$ to the change in life expectancy from mortality 1 to mortality 2 to be

$$\delta^{2-1}_x = \delta^{2-1}_{0|x+n} - \delta^{2-1}_{0|x} = e_0(M_{[x+n]}) - e_0(M_{[x]})$$

One may substitute $L_{0|x} = e_0 - e_x\, l_x$ (and similarly for other L's) into the above equations to arrive at

$$\delta^{2-1}_x = l^2_x\,(e^2_x - e^1_x) - l^2_{x+1}\,(e^2_{x+n} - e^1_{x+n}) \qquad (1)$$

The overall difference between the two life expectancies is hence

$$e^2_0 - e^1_0 = \sum_{x=0}^{\omega} \delta^{2-1}_x$$

where $l_{\omega+1}$ and $e_{\omega+1}$ are assumed to be 0.

One can similarly compute the opposite difference

$$e^1_0 - e^2_0 = \sum_{x=0}^{\omega} \delta^{1-2}_x$$

by swapping the 2's and 1's in the superscripts of δ^{2-1}_x. This should result in a value opposite in sign to that of $e^2_0 - e^1_0$, but not necessarily of the same magnitude, because δ^{2-1}_x is somewhat different from δ^{1-2}_x.

To resolve this asymmetric nature of the mortality zipper, Andreev et al. suggest averaging as a way to obtain symmetrical components:

$$\delta_x = \frac{\delta_x^{2-1} - \delta_x^{1-2}}{2}$$

- Decomposition by Cause

Das Gupta describes a technique for decomposition of additive functions.

Given a mapping $\phi(\vec{x}) = \phi(\sum_c x_c)$ and two vectors $\vec{x}$ and $\vec{y}$, Das Gupta defines the contribution to the difference $\delta := \phi(\vec{y}) - \phi(\vec{x})$ due to the change from $x_c \mapsto y_c$ to be:

$$\delta_c = \delta \cdot \frac{y_c - x_c}{\sum_c y_c - \sum_c x_c}$$

It is important to note that if $y_c - x_c = 0$ then there won't be any contribution δ_c, which is expected. However, if $\sum_c y_c - \sum_c x_c = 0$, then that does not necessarily mean that $\delta_c = 0$; it means that this method cannot be used to determine the decomposition.

If $\vec{u}$ is cause specific mortality, and ϕ is taken to be life expectancy function, then $\phi(\vec{u}) = \phi(\sum_c u_c)$, since $\sum_c u_c$ is all cause mortality.[45,46]

DALYs decomposition

To investigate the three major contributors to the overall change of cause-specific DALYs trend, a cause-specific decomposition analysis was applied. The three significant contributors to the DALYs trend are population growth, population aging, and an increase in age-specific rates of DALYs by cause. Decomposition analysis holds two of these three contributors constant and investigates the third one's contribution to a trend change. Expected DALYs number in 2019 were estimated using two hypothetical demographic scenarios: first, applying the age-specific rates of DALYs in 1990 into 2019 population size; second, applying the age structure and age-specific DALYs rates in 1990 into the 2019 population size and structure. The difference between the two scenarios was

considered as the contribution of population aging. Differences between the second scenario and cause-specific DALYs in 2019 were considered as the contribution of population growth. The remaining increase in DALYs was considered as the changes in the age-specific rates of cause-specific DALYs. Cause-specific contributions of these three factors were reported as the percent of change and the overall impact of them by cause-specific DALYs.[47]

Multi-intervention segmented regression model

To draw more formal inferences regarding the effect of an intervention or event on the response variable, the segmented regression model is a method for statistically modeling the interrupted time series data. Further, by using a multi-intervention segmented regression model, we can specify more than one change point. In the multi-intervention segmented regression model, level (value of the series at the beginning of a given time interval) and trend (rate of change of a measure) in the pre-intervention segment and changes in level and trend after the interventions are estimated.[48]

In this study, to investigate the impact of different health sector reforms (Universal Rural Health Insurance and Health Transformation Plan) and the economic crisis due to sanctions on the Iranian health-care system, two multi-intervention segmented regression models were utilised separately to explore the changes in NCDs and U5MR. In each model, death rate was incorporated as the dependent variable and the three major changes (health sector changes and the economic crisis due to sanctions) were considered as the independent variables, adjusted by the current health expenditure per capita (extracted from the World Bank).[49] The identity function was considered as a link function.

Unconditional probability of death

To calculate the unconditional probability of death, deaths due to four major NCDs (cardiovascular diseases, neoplasms, diabetes, or chronic respiratory diseases) between ages 30 and 70 years were considered. In this regard, a formula developed by the WHO and presented in NCD Global Monitoring Framework: Indicator Definitions and Specifications was used.[50]

All statistical analyses and graphical visualisations were done by R statistical packages v 4.0.3 (http://www.r-project.org/, RRID: SCR_001905).

This study complies with the GATHER recommendations.

GATHER guideline checklist

#	GATHER checklist item	Description of Compliance	Reference
**Objectives and funding**			
**1**	Define the indicator(s), populations (including age, sex, and geographic entities), and time period(s) for which estimates were made.	Narrative provided in paper and methods appendix describing indicators, definitions, and populations	Main text (Methods) and Appendix (Section 2)
**2**	List the funding sources for the work.	Funding sources listed in paper	Main text (Summary)
**Data Inputs**			
For all data inputs from multiple sources that are synthesized as part of the study:			
**3**	Describe how the data were identified and how the data were accessed.	Narrative provided in methods appendix	Appendix (Section 2)
**4**	Specify the inclusion and exclusion criteria. Identify all ad-hoc exclusions.	Narrative provided in methods appendix	Appendix (Section 2)
**5**	Provide information on all included data sources and their main characteristics. For each data source used, report reference information or contact name/institution, population represented, data collection method, year(s) of data collection, sex and age range, diagnostic criteria or measurement method, and sample size, as relevant.	Metadata for data sources by component, geography, cause, risk, or impairment is available through an interactive, online data source tool	Appendix (Section 2) and Online data citation tool: http://ghdx.healthdata.org/gbd-2019
**6**	Identify and describe any categories of input data that have potentially important biases (e.g., based on characteristics listed in item 5).	Summary of known biases included in methods appendix	Appendix (Section 2)
For data inputs that contribute to the analysis but were not synthesized as part of the study:			
**7**	Describe and give sources for any other data inputs.	Included in online data source tool	Online data citation tool: http://ghdx.healthdata.org/gbd-2019
For all data inputs:			
**8**	Provide all data inputs in a file format from which data can be efficiently extracted (e.g., a spreadsheet rather than a PDF), including all relevant meta-data listed in item 5. For any data inputs that cannot be shared because of ethical or legal reasons, such as third-party ownership, provide a contact name or the name of the institution that retains the right to the data.	Downloads of input data are available through online tools, including data visualisation	Online data visualization tools, data query tools, and the Global Health Data Exchange: http://ghdx.healthdata.org
**Data analysis**			
**9**	Provide a conceptual overview of the data analysis method. A diagram may be helpful.	Flow diagrams of the overall methodological processes have been provided	Appendix (Section 2)
**10**	Provide a detailed description of all steps of the analysis, including mathematical formulae. This description should cover, as relevant, data cleaning, data pre-processing, data adjustments and weighting of data sources, and mathematical or statistical model(s).	Flow diagrams and corresponding methodological write-ups have been provided	Appendix (Section 2)

#	GATHER checklist item	Description of Compliance	Reference
**11**	Describe how candidate models were evaluated and how the final model(s) were selected.	Details on model evaluation and finalisation have been provided	Appendix (Section 2)
**12**	Provide the results of an evaluation of model performance, if done, as well as the results of any relevant sensitivity analysis.	Details on evaluation of model performance have been provided elsewhere	See figure S6 on p 1446 of appendix 2 in Vos et al.”[1]
**13**	Describe methods for calculating uncertainty of the estimates. State which sources of uncertainty were, and were not, accounted for in the uncertainty analysis.	Details on uncertainty calculations have been provided	Appendix (Section 2)
**14**	State how analytic or statistical source code used to generate estimates can be accessed.	Code is provided in an online repository	http://ghdx.healthdata.org/gbd-2019/code
**Results and Discussion**			
**15**	Provide published estimates in a file format from which data can be efficiently extracted.	GBD 2019 results are available through online data visualization tools, the Global Health Data Exchange, and the online data query tool	http://ghdx.healthdata.org/gbd-2019
**16**	Report a quantitative measure of the uncertainty of the estimates (e.g. uncertainty intervals).	Uncertainty intervals are provided with all results in main text, methods appendix, and online	Main manuscript (Results), Appendix (Sections 3, 4), and data tool: http://ghdx.healthdata.org/gbd-2019
**17**	Interpret results in light of existing evidence. If updating a previous set of estimates, describe the reasons for changes in estimates.	Discussion has been provided in the main text	Main manuscript (Discussion) and Appendix (Section 5)
**18**	Discuss limitations of the estimates. Include a discussion of any modelling assumptions or data limitations that affect interpretation of the estimates.	Discussion of limitations has been provided	Appendix (Section 6)

Section 3

Supplementary results

The healthy life expectancy (HALE) of both sexes at birth increased from 57·8 (95% uncertainty interval: 55·1–60·1) years in 1990 to 66·8 (63·7–69·7) years in 2019 (appendix p 29). The highest and lowest percent change of HALE across provinces was in Kurdistan (21·6%) and Tehran (7·5%) between 1990 and 2019.

The share of all-cause age-standardised YLD rate from the all-cause age-standardised DALY rate steadily increased from 26·9% in 1991 to 44·5% in 2019 (appendix p 40). In this period, this share increased from 7·8% to 31·3% for communicable, maternal, neonatal, and nutritional diseases (CMNNDs), 36·5% to 49·5% for NCDs, and 16·4% to 22·5% for injuries. The ratio of all-cause age-standardised YLL to YLD ratio was decreased from 2·7 in 1991 to 1·2 in 2019 in Iran. Noticeably, this decrease was consistent in all provinces from 1991 to 2019 with the greatest reduction attributed to Kurdistan province (3·7–1·4) and the smallest reduction belonged to Tehran province (1·5–0·8).

In the study period, the under-5 mortality rate (U5MR) decreased from 71·2 (63·6–79·1) to 11·1 (10·2–12·0) per 1000 live births. The ratio of the highest to the lowest U5MR across provinces was 3·7 in 1990 and 3·3 in 2019; nonetheless, the absolute difference across provinces substantially decreased from 85·8 to 12·4 per 1000 live births, from 1990 to 2019, accordingly, with the greatest decline between 1990 and 2000 (appendix p 15, 31).

In 2019, 78·9 (78·1–79·5) million person-NCDs (>1 NCD in some individuals) were living in Iran, pronouncing the great burden on the health-care system. This number significantly increased by 51·7% from 1990 to 2019. Although the all-ages prevalence rate of NCDs significantly increased by 5·4%, the age-standardised measure was unchanged by 0·5% increase in the study period. Inspecting the age-standardised prevalence rates of level three NCD causes showed that the top five causes were oral disorders, headache disorders, hemoglobinopathies, cirrhosis, and gynaecological diseases both in nation-wide and all provinces in 1990 and 2019 with the same ranking.

The distribution of NCDs in subnational divisions demonstrates that NCDs are more prevalent in eastern and western provinces; nevertheless, most of the Iranian population reside in central areas. The ratio of the highest to the lowest age-standardised DALY rates of provinces due to NCDs remained around 1·4 in the study period. Notably, Tehran province had the lowest rates from 1990 to 2019. Age distribution of prevalent NCDs demonstrates a reverse U-shape structure with the 30–34 age group as the leading group with 8·6 (8·58–8·7)

million person-NCDs. Adjustment for the age indicates a rising in prevalence estimates and stabilising around 30–39 age-group and slowly reaching to 99 997·7 (99 994·7–99 999·4) person-NCDs per 100 000 population in 95+ age group. The trend of age-standardised DALYs in 30 years of GBD in 31 provinces of Iran showed a steady trend with no convergence or divergence; indicative of the need for intensified action plans.

The decomposition analysis revealed that of 44·2% increase in the crude DALYs of NCDs in the period 1990-2019, 44·0% is attributable to population growth, 46·8% to alteration in age structure of community, and 46·6% decreasing change in the burden of NCDs. Moreover, the most attributable factor was the age structure change for both males and females (appendix p 65).

From 1990 to 2019, the number of prevalent cases with mental disorders increased remarkably by 57·2% and reached 15·7 (14·6–17·0) million in 2019; nonetheless, the age-standardised prevalence rate declined not significantly by 1·3%. In the study period, the number of prevalent cases with substance use disorders, by 108·6% increase, reached 0·9 (0·8–1·1) million in 2019, along with a 12·6% not-significant increase in the age-standardised prevalence rate.

Based on the GBD protocol, the fatal outcomes of mental disorders including self-harm and suicide are classified under injuries; however, all deaths due to self-harm and suicide are not attributed to mental disorders. Hence, more than 99% of all burden of mental disorders are related to YLD. In 2019, depressive disorders and anxiety disorders had the highest contribution to the total DALYs of mental disorders, 39·6% and 29·6%, accordingly, followed by bipolar disorders (7·8%) and schizophrenia (7·7%).

According to the decomposition analysis, population growth, changes in the age structure, and alteration in the epidemiology of mental disorders were responsible for 44·0%, 29·6%, and 3·5% of the 77·1% increase in the crude DALYs of these disorders, respectively. This analysis demonstrated that of 132·8% increase in the crude DALYs of substance use disorders, 44·0% and 50·1% were attributed to population growth and alterations in the age structure, accordingly; nonetheless, the DALYs proportion in the decomposition increased by 38·7% in the study period (appendix p 65).

In 1991, 6·3 (5·8–6·9) million incident cases of injuries occurred, which not-significantly increased by 8·7% and reached 6·8 (6·3–7·4) million in 2019; however, the age-standardised incidence rate of injuries considerably decreased by 18·6% in this period. From 1991 to 2019, the greatest reduction of age-standardised incidence rate was for self-harm and interpersonal violence injuries, 58·9%, followed by transport injuries, 29·4%, while it decreased not significantly by 12·0% for unintentional injuries. Furthermore, self-harm that occupied the

12th rank of age-standardised incidence rate among injuries showed a significant decreasing pattern by 23·5% reduction since 1991; 44·9 (35·9–55·7) new cases per 100 000 in 2019. In this time, the decomposition analysis showed that form the overall 34·9% decrease in the DALYs, a 4·5% decrease is attributed to the alteration in age structure, 43·0% to population growth increase, and 73·5% due to the DALYs true reduction (appendix p 65).

In 2019, there were 42·5 (40·3–44·7) million prevalent cases with CMNNDs in Iran, 51·4% greater than 1990; however, the age-standardised prevalence rate remarkably declined by 9·0% in this period. From 1990 to 2019, both all-ages and age-standardised death rates due to CMNNDs considerably decreased by 81·5% and 72·6%, respectively.

According to the decomposition analysis, population growth and changes in the DALYs rate were accountable for 44·0% increase and 63·9% decrease in the 77·1% decrease in the crude DALYs of CMNNDs, accordingly; nevertheless, changes in the age structure contributed to a 57·2% decrease in the number of DALYs. In this regard, within almost each CMNNDs subcategories, a remarkable decreases in the DALYs rate (ranging from 124·4% decrease for neglected tropical diseases and malaria to 51·2% decrease in enteric infections) was detected except for HIV/AIDS and sexually transmitted infections with a substantial 299·1% increment (appendix p 65).

In 2019, the top five causes of DALYs attributed to GBD 2019 risk factor set were cardiovascular diseases, diabetes and kidney diseases, maternal and neonatal disorders, neoplasms, and substance use disorders, with 15·9%, 5·6%, 3·5%, 2·6%, and 1·9% share of total DALYs, respectively. Moreover, cardiovascular diseases, diabetes and kidney diseases, neoplasms, chronic respiratory diseases, and maternal and neonatal disorders, with 39·4%, 7·0%, 5·4%, 2·7%, and 1·6% share of total deaths, accordingly, were the top five sources of deaths attributed to GBD 2019 risk factor set. These findings again highlight the significance of NCDs as the main cause of the burden of diseases in Iran. In 2019, high systolic blood pressure, the chief contributor to DALY and death, led to 1·9 (1·7–2·2) million DALYs and 92 066 (79 208–104 016) deaths due to cardiovascular diseases, and 186 785 (162 003–211 888) DALYs and 7873 (6781–8809) deaths because of diabetes and kidney diseases.

Section 4

Legends of supplementary figures

Appendix Figure 1. The change in age structure of the population (in million) of Iran from 1990 to 2019

Appendix Figure 2. The socio-demographic index among provinces of Iran in 1990 and 2019

Appendix Figure 3. Total fertility rate across provinces of Iran in 2019

Appendix Figure 4. Trend in life expectancy at birth (years) for males and females from 1990 to 2019 in Iran

Appendix Figure 5. Life expectancy at birth (years) across provinces of Iran for both sexes in 1990, 1991 and 2019

Appendix Figure 6. The epidemiological transition level (%) among provinces of Iran in 1990 and 2019

Appendix Figure 7. The trend of all-cause mortality numbers and age-standardised rate (per 100 000) by sex from 1990 to 2019 at national level

Appendix Figure 8. All-cause age-standardised share of YLLs and YLDs rate at national and subnational levels for both sexes in 1991 and 2019

Appendix Figure 9. All-cause age-standardised mortality rate (per 100 000) among provinces of Iran for both sexes in 1990, 1991 and 2019

Appendix Figure 10. All-cause age-standardised mortality rate (per 100 000) among provinces of Iran by sex in 2019

Appendix Figure 11. Under-5 mortality rate (per 1000 live births) among provinces of Iran in 2019

Appendix Figure 12. Ranking of level two causes of age-standardised DALYs rate, 1990 compared to 2019

Appendix Figure 13. Ranking of level three subtypes of NCDs' age-standardised DALYs rate at national and subnational levels for both sexes in 1990

Appendix Figure 14. DALY rates of mental and substance use disorders' subtypes (per 100 000) by age group and sex at national level in 1990 and 2019

Appendix Figure 15. The age-standardised death and DALYs rates (per 100 000) of injuries' subtypes at national level

Appendix Figure 16. Ranking of level three subtypes of injuries' age-standardised DALYs rate at national and subnational levels for both sexes in 1991

Appendix Figure 17. Ranking of level three subtypes of injuries' age-standardised DALYs rate at national and subnational levels for both sexes in 2019

Appendix Figure 18. Time trend of age-standardised DALYs rate (per 100 000) due to CMNNDs among provinces of Iran for both sexes in 1990 and 2019

Appendix Figure 19. Ranking of level three subtypes of CMNNDs' age-standardised DALYs rate at national and subnational levels for both sexes in 1990

Appendix Figure 20. Ranking of level three subtypes of CMNNDs' age-standardised DALYs rate at national and subnational levels for both sexes in 2019

Appendix Figure 21. Age-standardised rate of DALYs attributable to risk factors across provinces of Iran in 1990 and 2019

Appendix Figure 22. Ranking of attributed age-standardised DALYs rate to level three risk factors at national and subnational levels for both sexes in 1990

Appendix Figure 23. Ranking of attributed age-standardised DALYs rate to level three risk factors at national and subnational levels for both sexes in 2019

Legends of supplementary tables

Appendix Table 1. Population (in thousands) by location and sex in 1990, 2000, 2010, and 2019

Appendix Table 2. Life expectancy at birth (years) by location and sex in 1990, 1991, 2000, 2010, and 2019

Appendix Table 3. Healthy life expectancy at birth (years) by location and sex in 1990, 1991, 2000, 2010, and 2019

Appendix Table 4. Total fertility rate and under-5 mortality rate (per 1000 live births) by location in 1990 and 2019

Appendix Table 5. Decomposition of life expectancy at birth (years) from 1990 to 2019 by location

Appendix Table 6. Decomposition analysis of DALYs change by cause, sex, and location, 1990–2019

Appendix Table 7. National DALYs and death due to causes, number for all ages and age-standardised rate (per 100 000) in 1990 and 2019 with percent changes between 1990 and 2019, by sex

Appendix Table 8. Provincial DALYs and death due to causes, number for all ages and age-standardised rate (per 100 000) in 1990 and 2019 with percent changes between 1990 and 2019, by sex

Appendix Table 9. Provincial DALYs and death attributed to risk factors for all-causes, attributed number for all ages and age-standardised rate (per 100 000) in 1990 and 2019 with percent change between 1990 and 2019, by sex

Appendix Table 10. Annual percent change (%) of age-standardised death rate between specific time periods

Appendix Table 11. Multi-intervention segmented regression model of age-standardised NCDs death rate per 100,000

Appendix Table 12. Multi-intervention segmented regression model of under-5 mortality rate per 1000 live births

Appendix figure 1

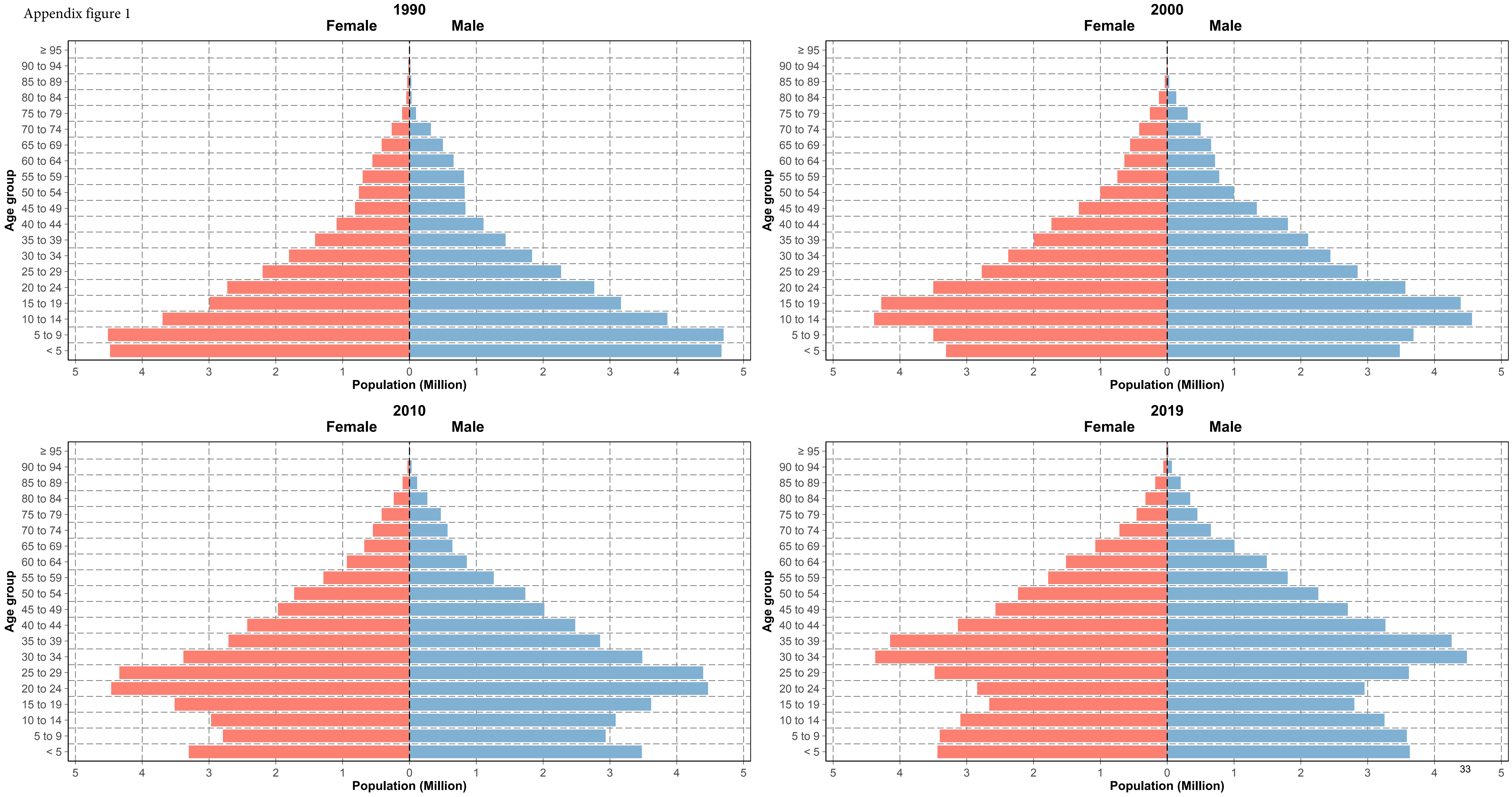

Appendix figure 2

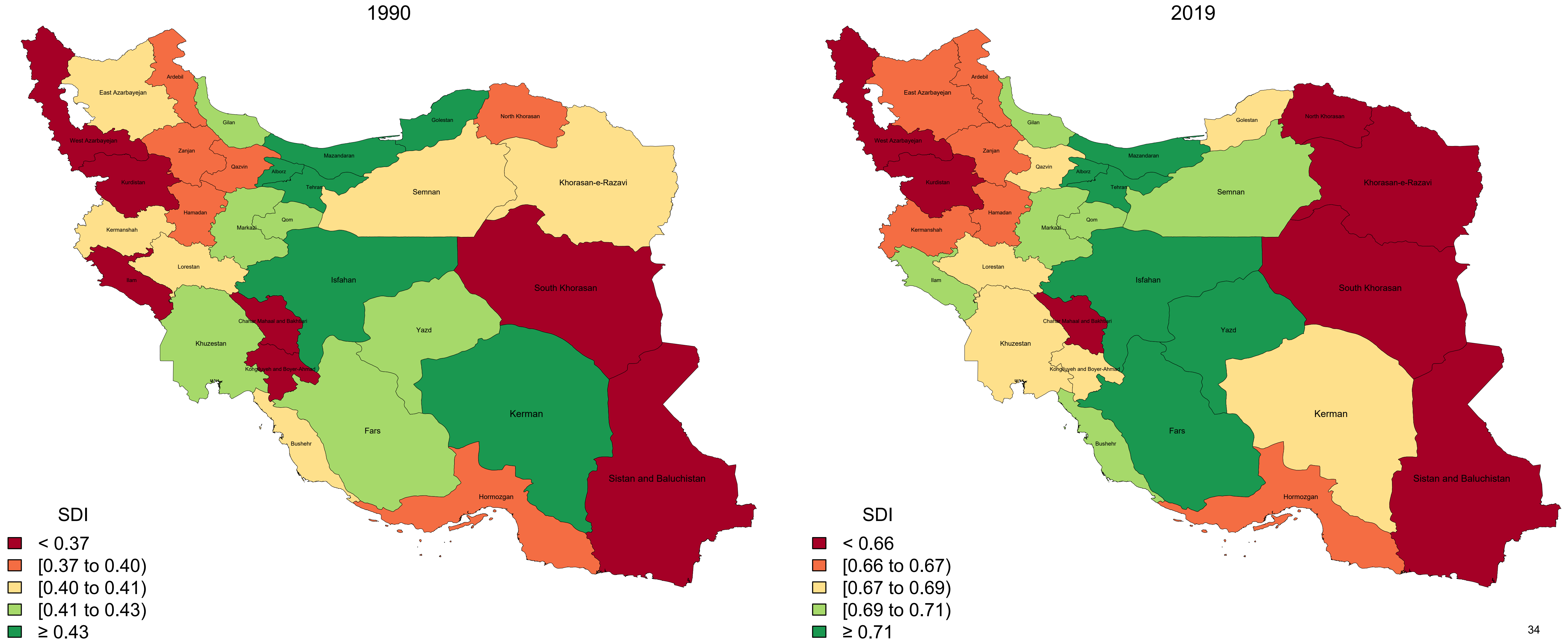

Appendix figure 3

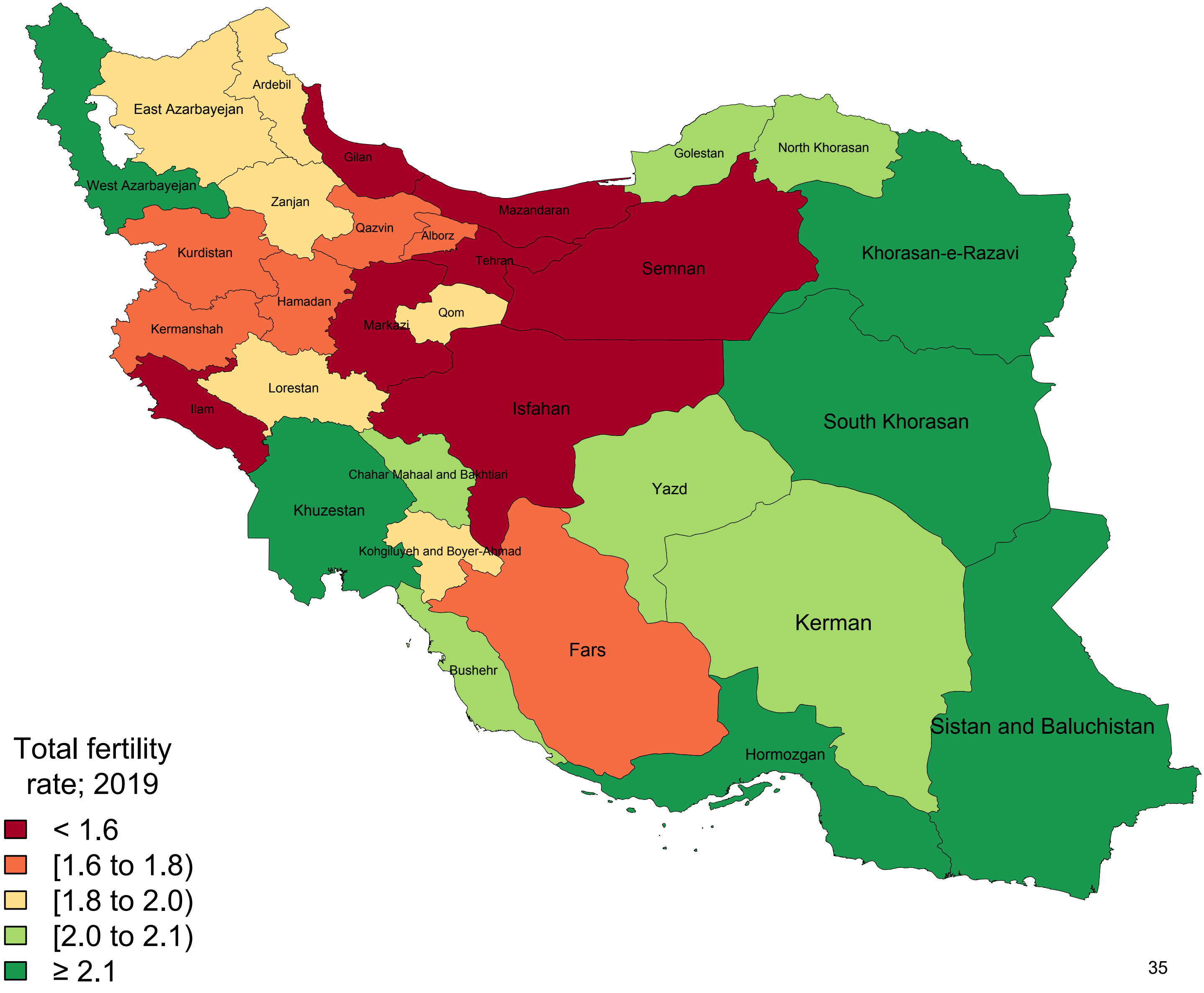

Appendix figure 4

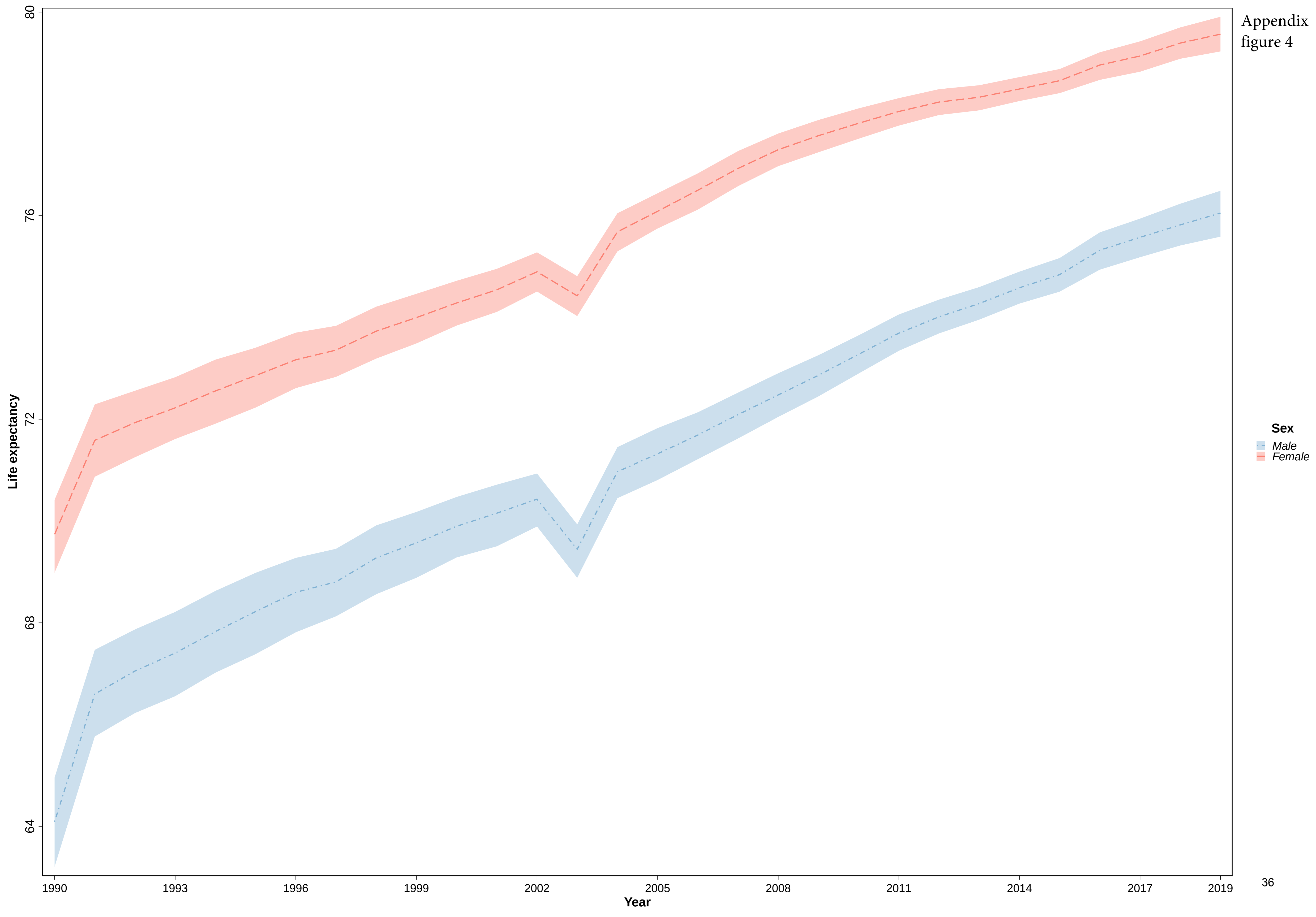

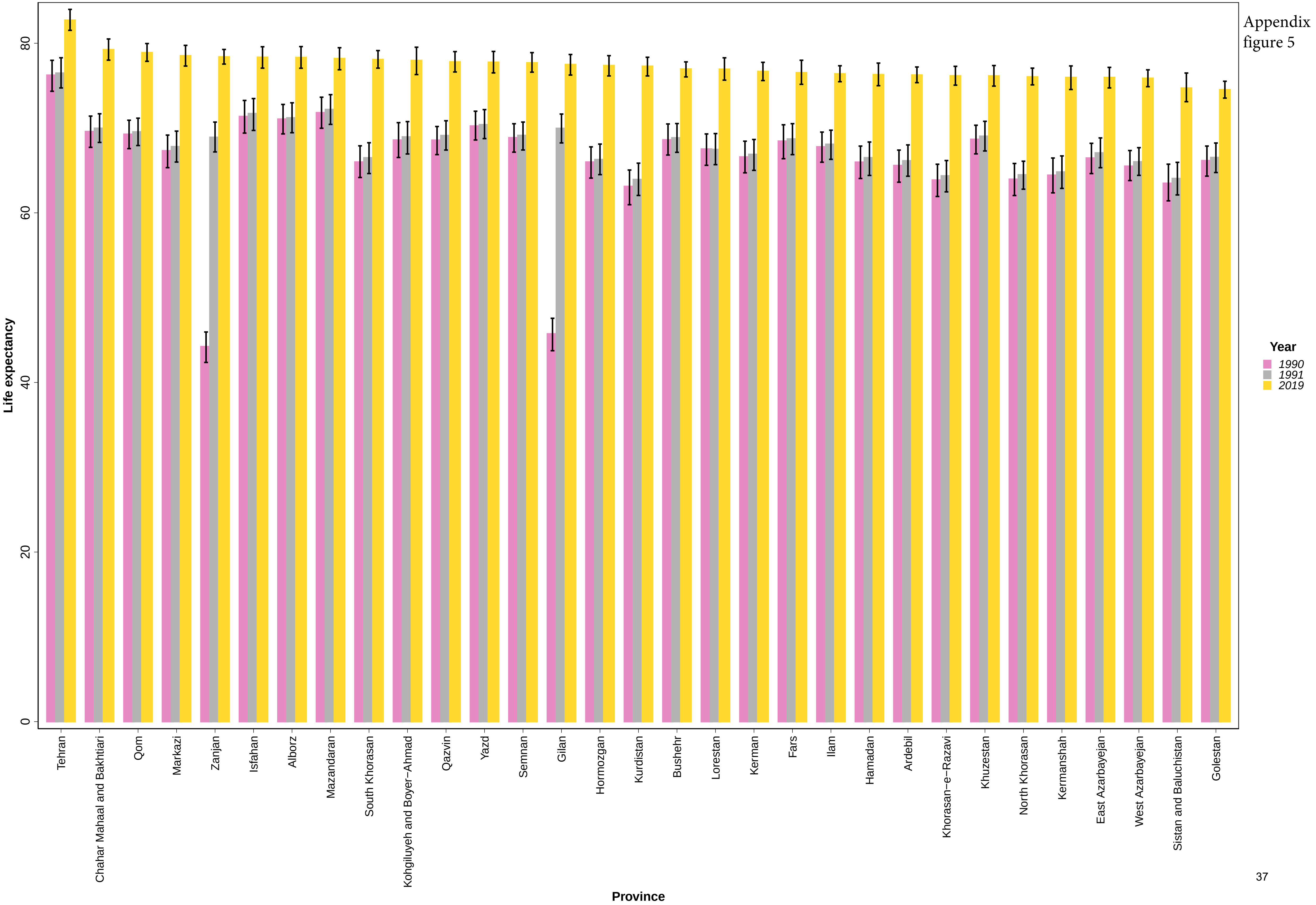
Life expectancy
0
20
40
60
80
Province
Tehran
Chahar Mahaal and Bakhtiari
Qom
Markazi
Zanjan
Isfahan
Alborz
Mazandaran
South Khorasan
Kohgiluyeh and Boyer−Ahmad
Qazvin
Yazd
Semnan
Gilan
Hormozgan
Kurdistan
Bushehr
Lorestan
Kerman
Fars
Ilam
Hamadan
Ardebil
Khorasan−e−Razavi
Khuzestan
North Khorasan
Kermanshah
East Azarbayejan
West Azarbayejan
Sistan and Baluchistan
Golestan
Year
1990
1991
2019

Appendix figure 6

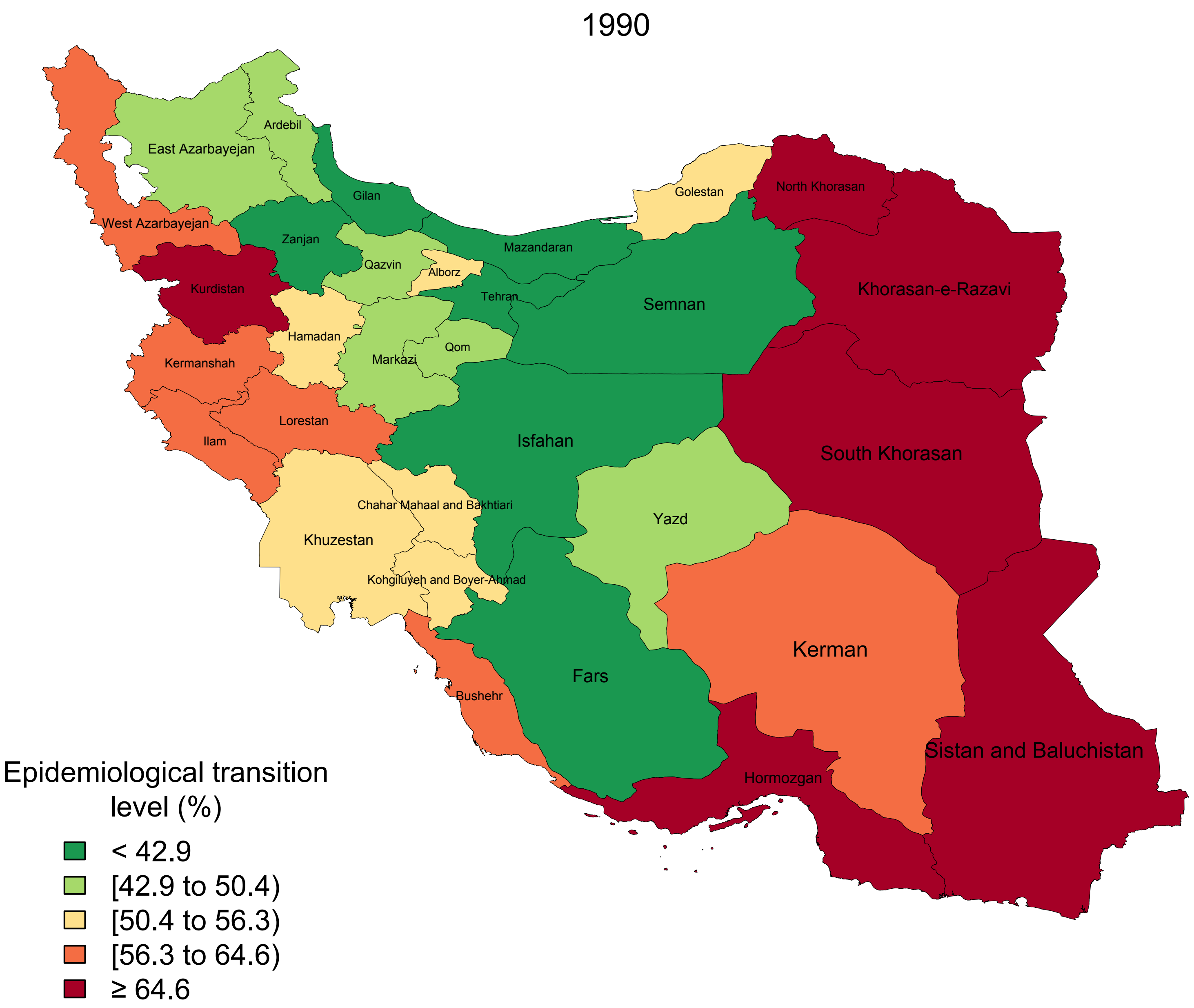

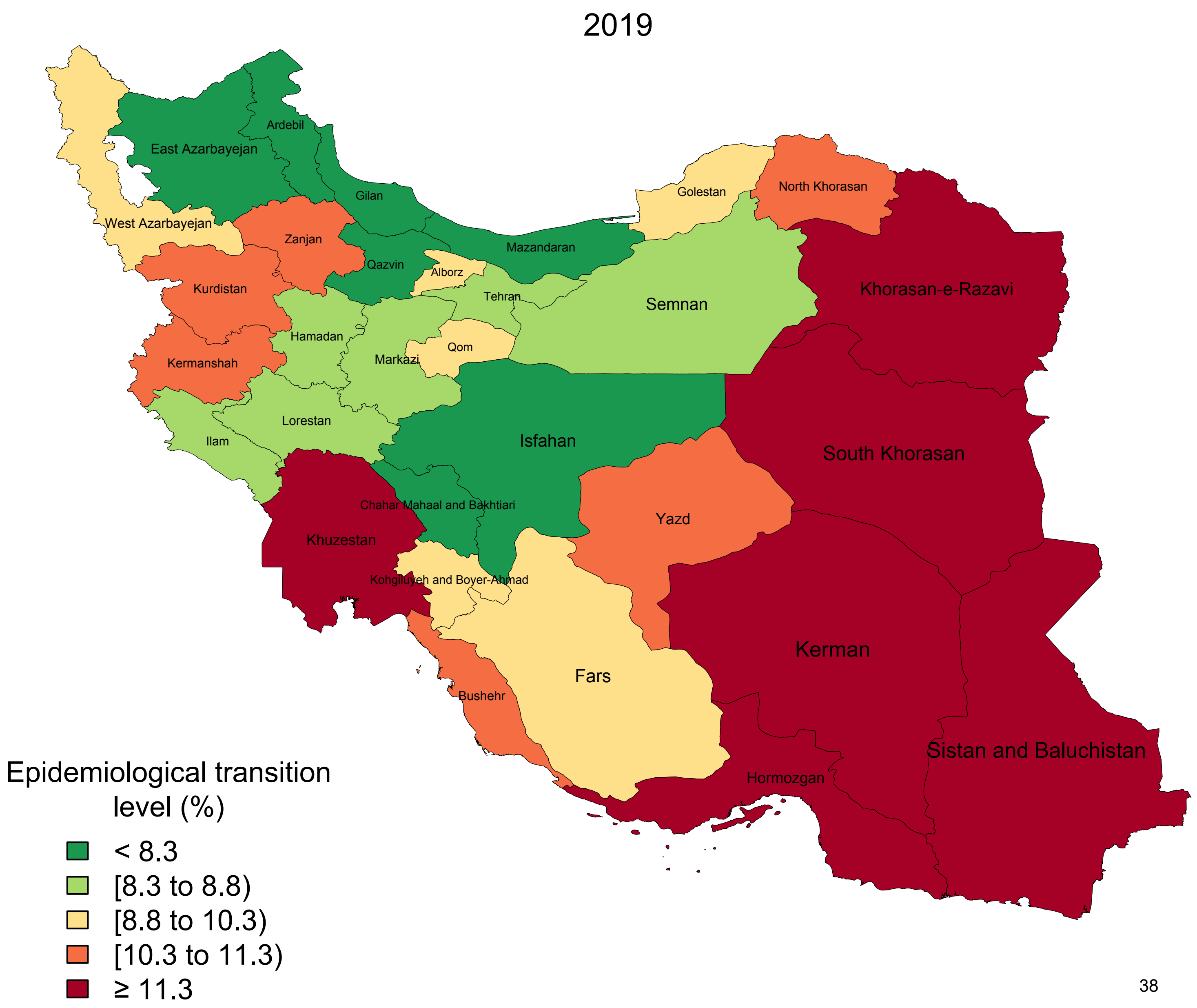

Appendix figure 7

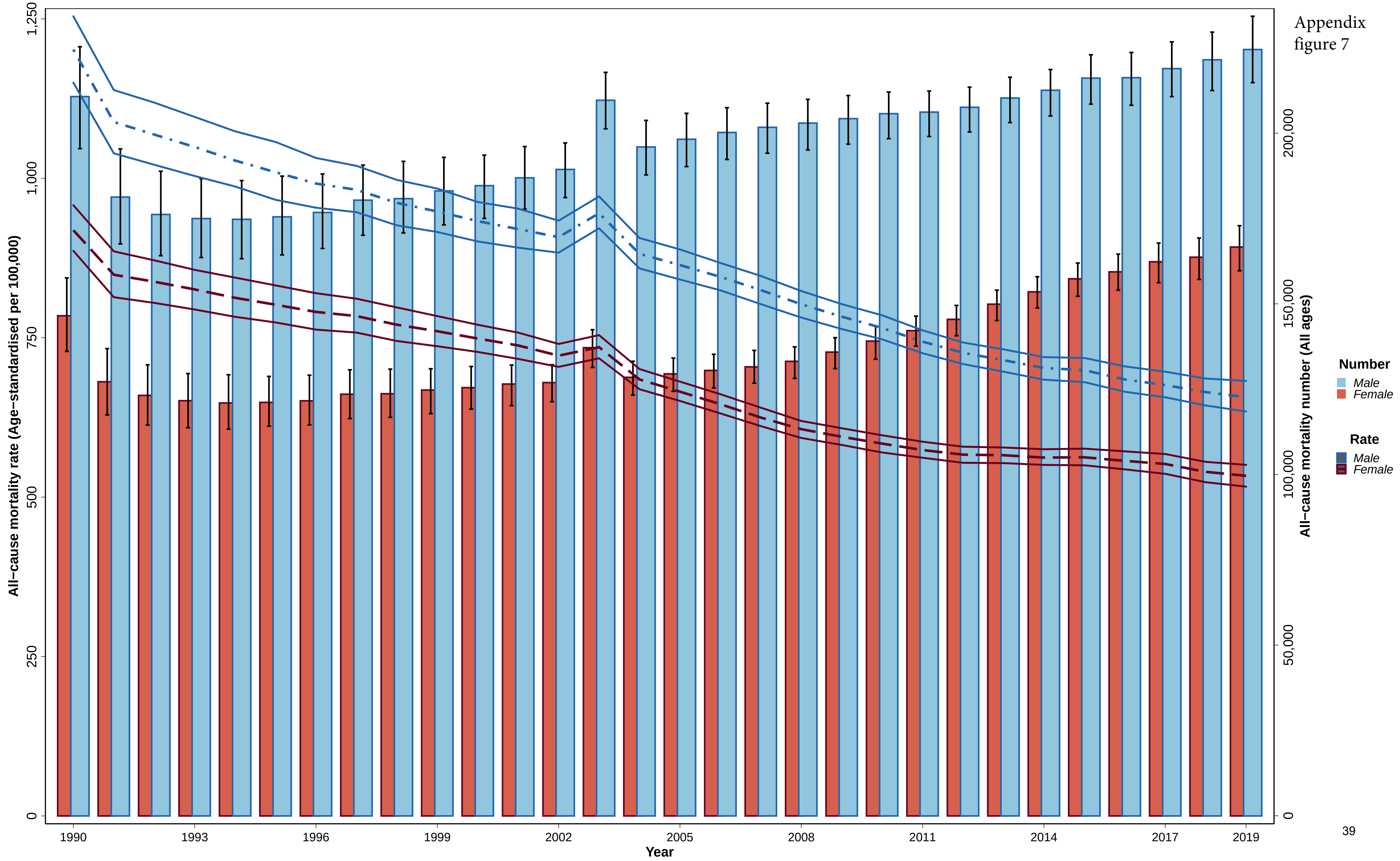

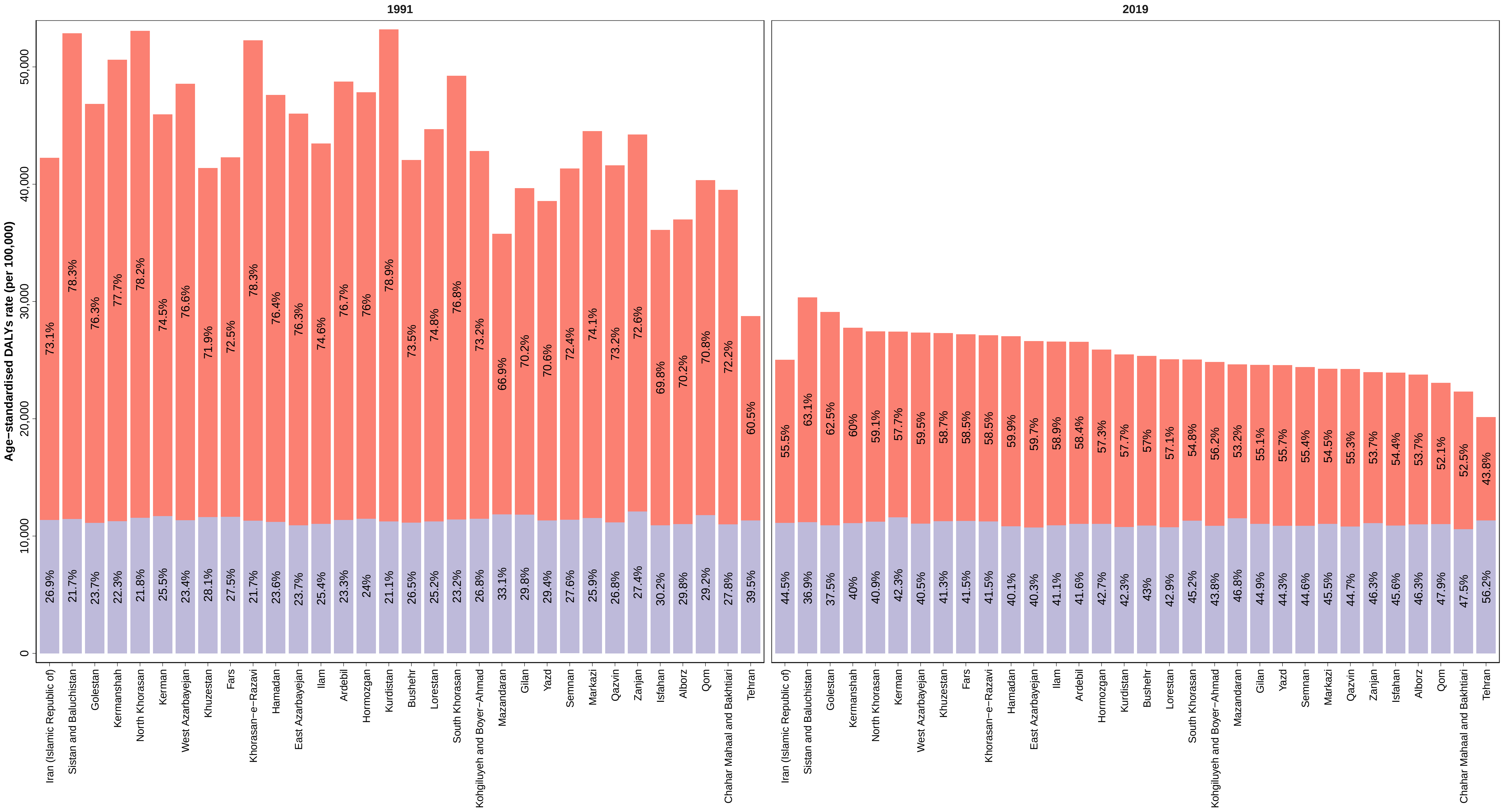

Appendix figure 8

Appendix figure 9

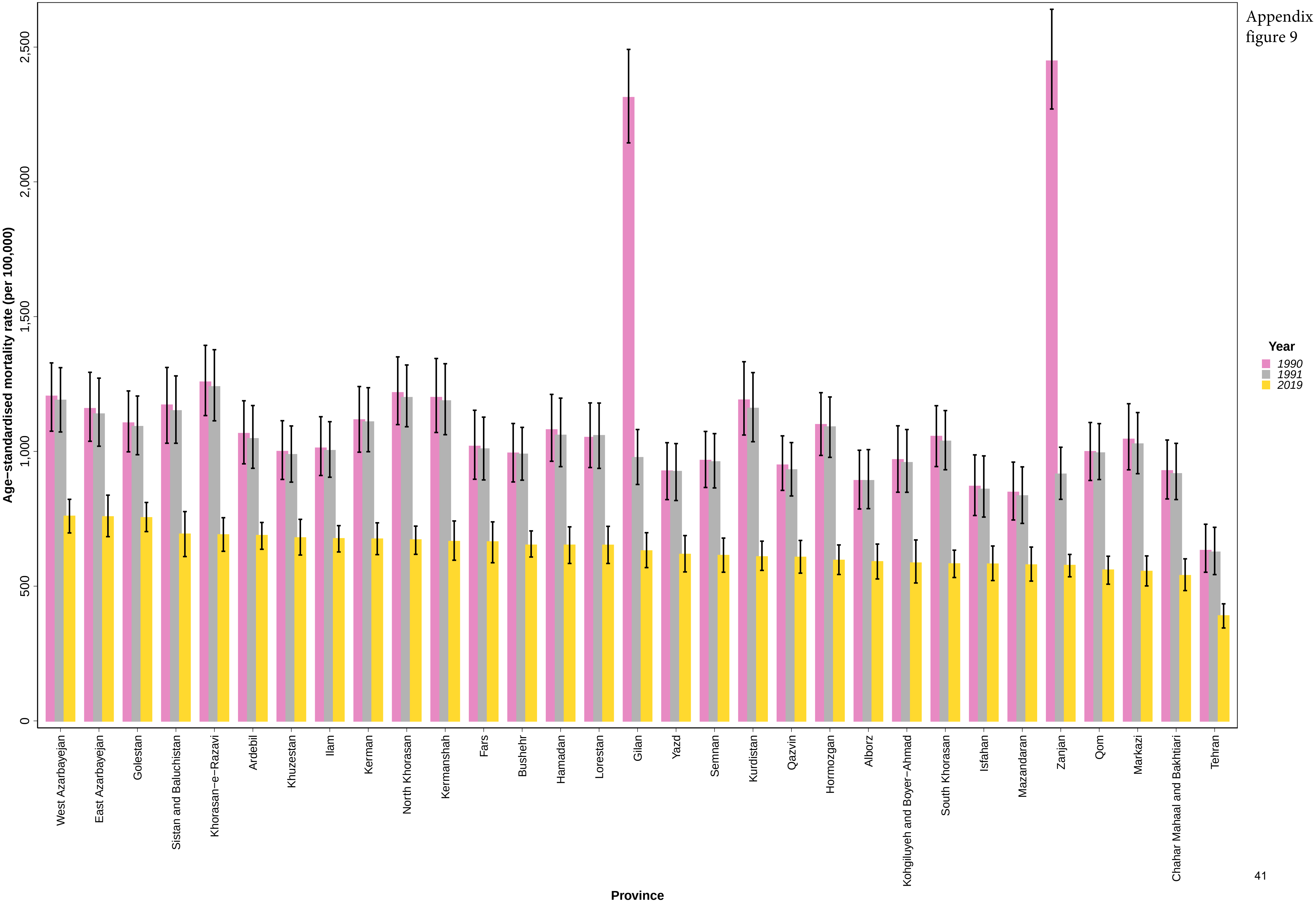

Appendix figure 10

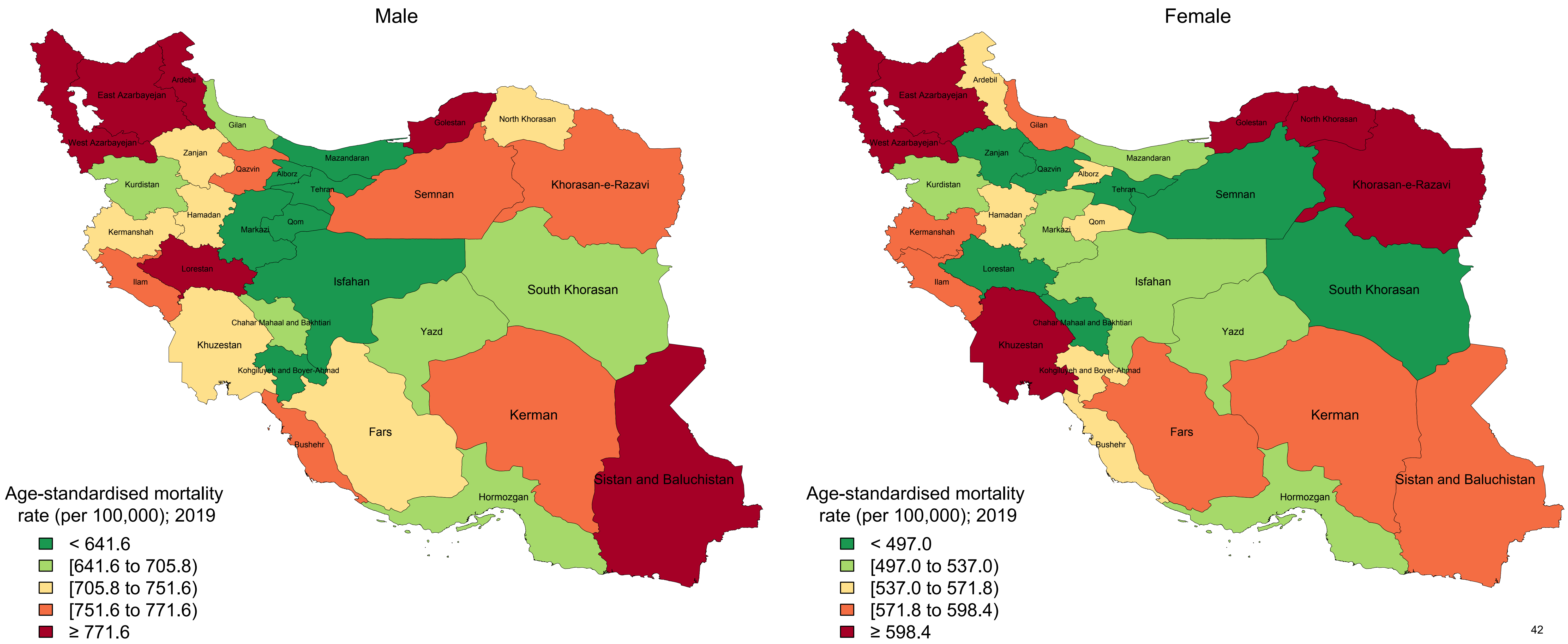

Appendix figure 11

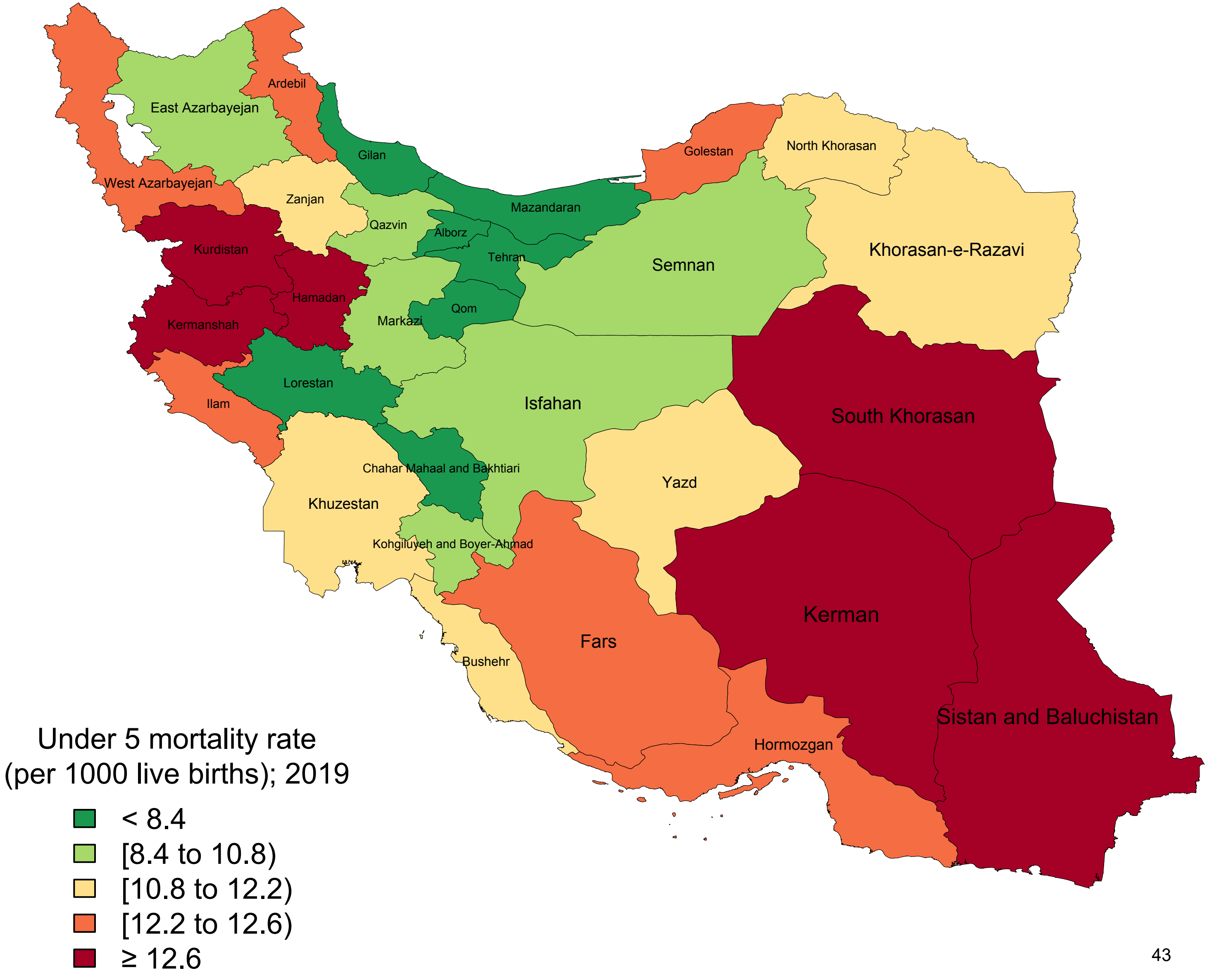

Appendix figure 12

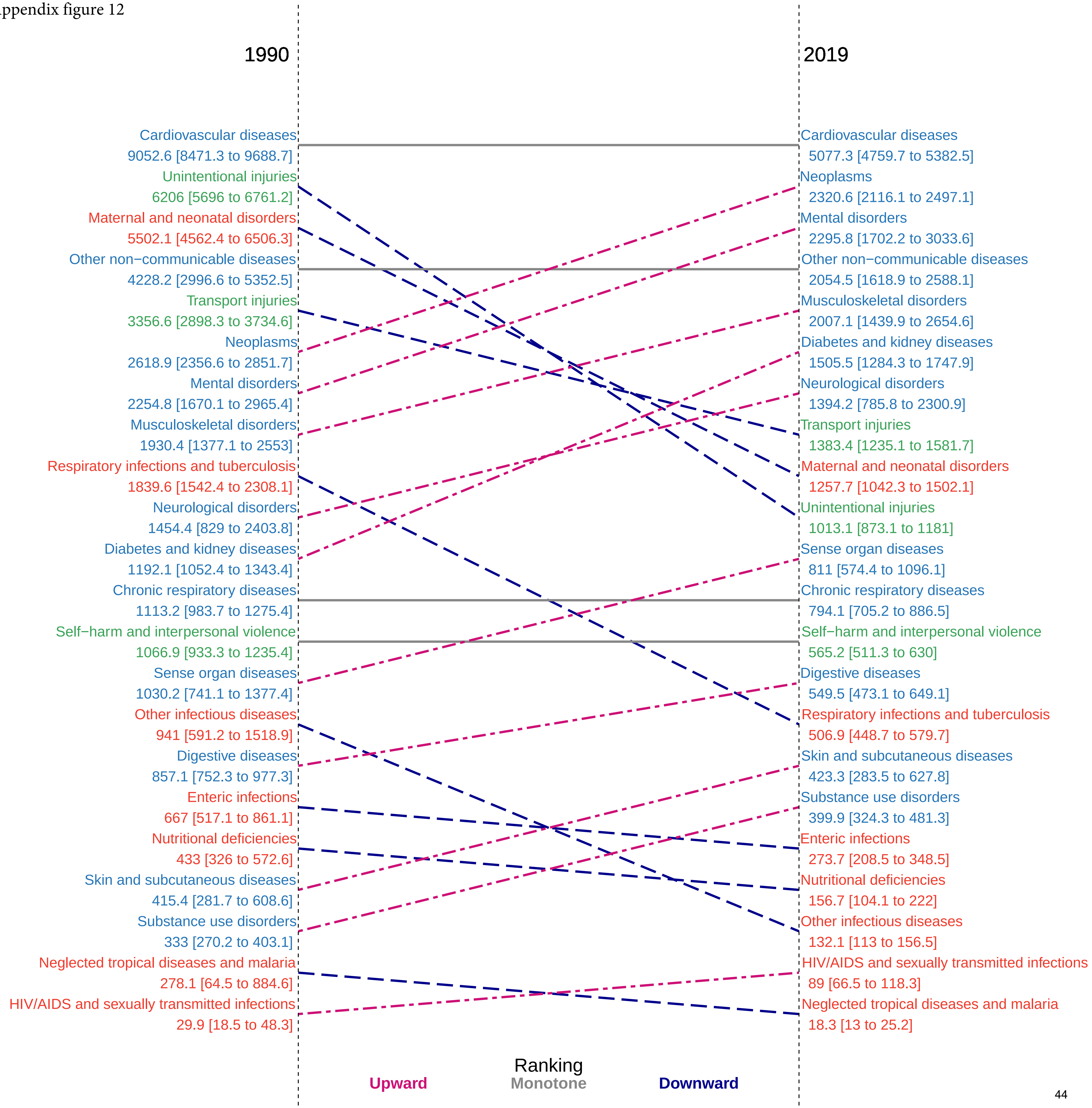

Appendix figure 13

	National	Subnational																														
	Iran (Islamic Republic of)	Alborz	Ardebil	Bushehr	Chahar Mahaal and Bakhtiari	East Azarbayejan	Fars	Gilan	Golestan	Hamadan	Hormozgan	Ilam	Isfahan	Kerman	Kermanshah	Khorasan-e-Razavi	Khuzestan	Kohgiluyeh and Boyer-Ahmad	Kurdistan	Lorestan	Markazi	Mazandaran	North Khorasan	Qazvin	Qom	Semnan	Sistan and Baluchistan	South Khorasan	Tehran	West Azarbayejan	Yazd	Zanjan
Other cardiovascular and circulatory diseases	25	23	6	31	28	30	27	23	21	24	16	22	26	25	26	26	20	15	24	25	29	23	9	20	24	34	21	8	37	26	33	26
Tracheal, bronchus, and lung cancer	24	26	25	19	23	23	25	22	27	26	29	23	24	18	20	21	23	26	20	23	23	26	28	25	23	23	26	24	28	16	22	23
Drug use disorders	23	22	30	27	34	26	23	28	30	23	21	38	27	24	18	24	25	33	32	22	26	33	30	24	31	26	24	26	12	34	27	27
Oral disorders	22	20	23	21	20	24	21	19	23	22	22	18	19	22	23	23	22	22	23	21	21	20	23	21	22	20	23	23	19	23	19	19
Leukemia	21	27	17	20	22	17	20	21	24	18	25	21	21	23	22	18	24	20	19	24	20	21	22	23	20	22	27	22	22	20	18	20
Endocrine, metabolic, blood, and immune disorders	20	18	21	22	21	22	22	20	22	20	20	20	20	21	24	22	21	18	22	20	22	19	21	22	21	21	22	21	15	22	20	18
Alzheimer's disease and other dementias	19	17	20	17	16	19	18	17	18	19	19	15	17	20	19	20	18	17	18	19	19	18	20	18	19	18	19	19	17	18	17	17
Cirrhosis and other chronic liver diseases	18	21	22	18	24	21	13	24	12	16	13	24	22	14	15	16	15	23	16	15	16	22	15	5	11	19	12	14	21	21	23	21
Neck pain	17	16	18	15	15	18	17	15	17	17	17	14	16	17	17	19	16	16	17	17	18	16	19	17	18	17	18	20	10	17	16	16
Age-related and other hearing loss	16	14	15	12	11	15	12	13	15	15	12	13	12	15	16	17	13	12	14	13	15	13	18	16	17	15	15	18	11	15	14	13
Blindness and vision loss	15	13	14	11	12	14	11	14	14	14	11	12	13	13	14	11	14	11	15	12	14	14	17	12	16	16	13	17	16	14	15	12
Asthma	14	19	12	14	13	6	16	12	5	11	14	17	15	8	13	8	17	14	12	18	11	15	8	15	14	10	4	4	23	7	12	14
Gynecological diseases	13	11	13	9	10	13	10	9	13	12	10	11	10	12	12	14	11	10	13	11	13	11	16	11	15	14	14	16	9	13	13	11
Chronic obstructive pulmonary disease	12	8	19	16	14	9	14	16	19	13	18	16	11	6	7	9	12	19	11	14	12	17	14	14	6	12	6	11	14	10	9	9
Stomach cancer	11	15	4	23	7	8	15	8	11	9	24	7	18	16	11	6	19	6	4	6	10	9	7	13	13	11	17	15	20	4	21	7
Diabetes mellitus	10	9	16	13	19	16	19	18	16	21	15	19	14	19	21	15	9	21	21	16	17	8	13	19	12	13	20	10	4	19	11	22
Chronic kidney disease	9	12	9	8	17	7	9	10	8	8	7	6	9	7	8	7	10	13	7	10	7	12	10	10	8	8	5	13	13	12	10	15
Hypertensive heart disease	8	10	8	10	6	5	8	11	10	10	4	10	8	11	6	5	6	7	9	5	6	10	4	6	7	5	8	7	24	8	8	10
Anxiety disorders	7	7	11	7	9	11	7	7	7	7	9	9	7	10	10	13	8	9	10	8	9	7	12	9	10	9	11	12	8	11	6	8
Headache disorders	6	6	10	6	5	10	6	6	6	5	8	8	6	9	9	10	7	8	8	7	8	6	11	8	9	7	10	9	7	9	7	6
Depressive disorders	5	5	7	5	8	12	4	5	9	6	6	5	5	4	5	12	5	5	6	9	5	5	6	7	5	6	9	6	6	6	5	5
Low back pain	4	4	5	4	4	4	5	4	4	4	5	4	4	5	4	4	4	4	5	4	4	4	5	4	4	4	7	5	5	5	4	4
Stroke	3	2	3	2	3	3	3	2	3	3	2	3	3	2	2	3	3	3	3	2	2	3	3	3	2	3	3	3	3	3	2	3
Congenital birth defects	2	3	1	3	2	2	2	3	2	2	3	2	2	3	3	2	2	2	2	3	3	2	2	2	3	2	2	2	2	2	3	2
Ischemic heart disease	1	1	2	1	1	1	1	1	1	1	1	1	1	1	1	1	1	1	1	1	1	1	1	1	1	1	1	1	1	1	1	1

Appendix figure 14

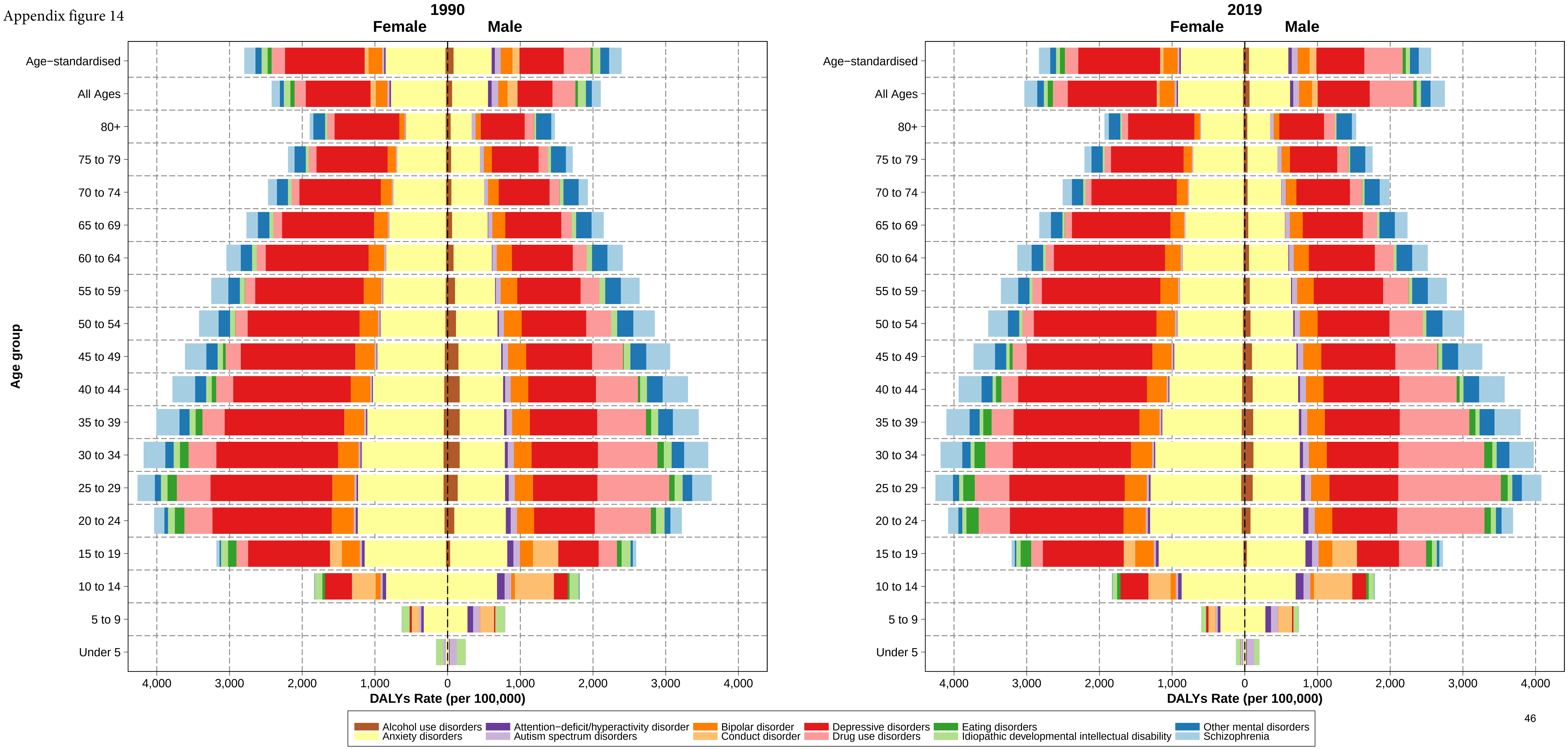

Appendix figure 15

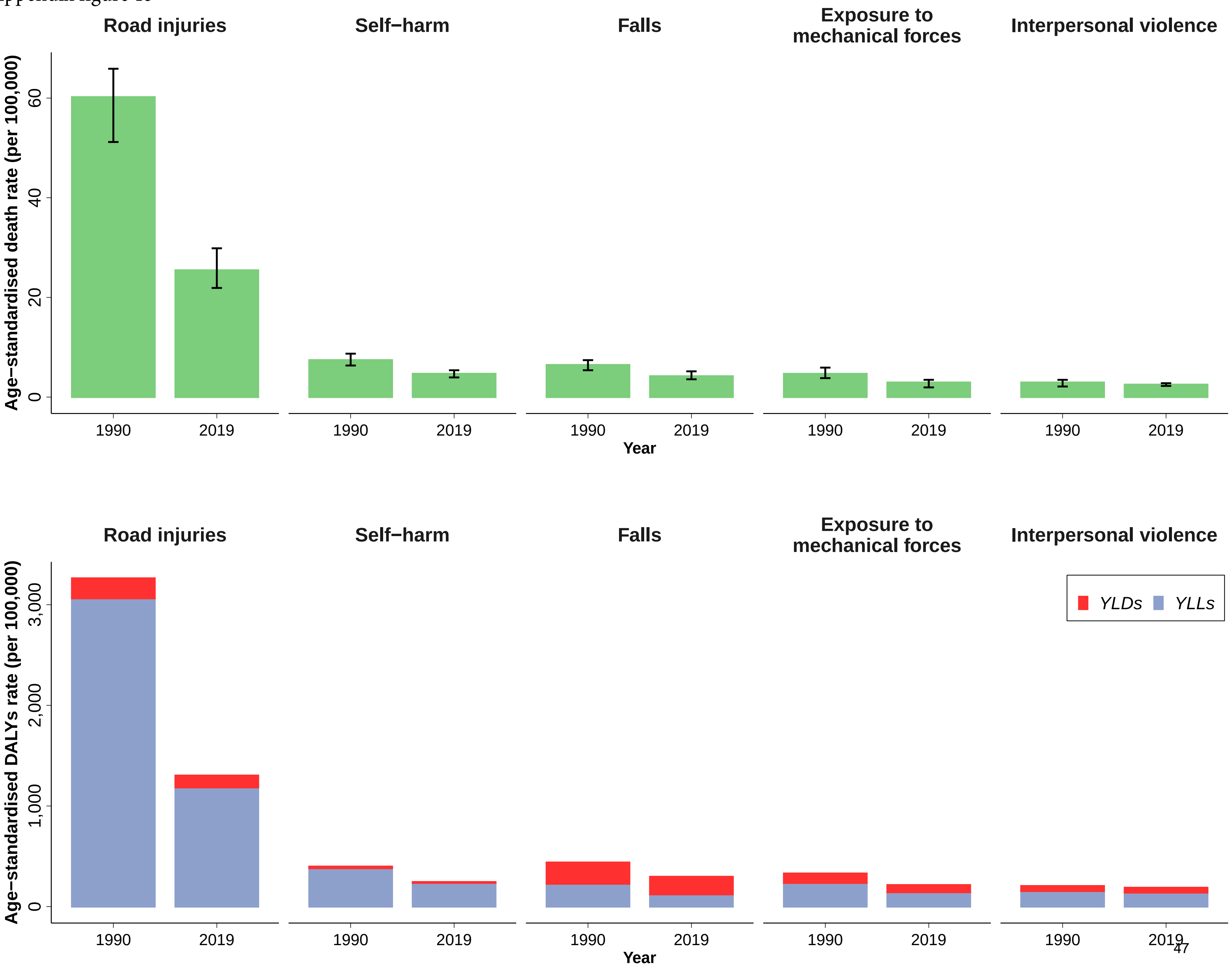

Appendix figure 16 1991

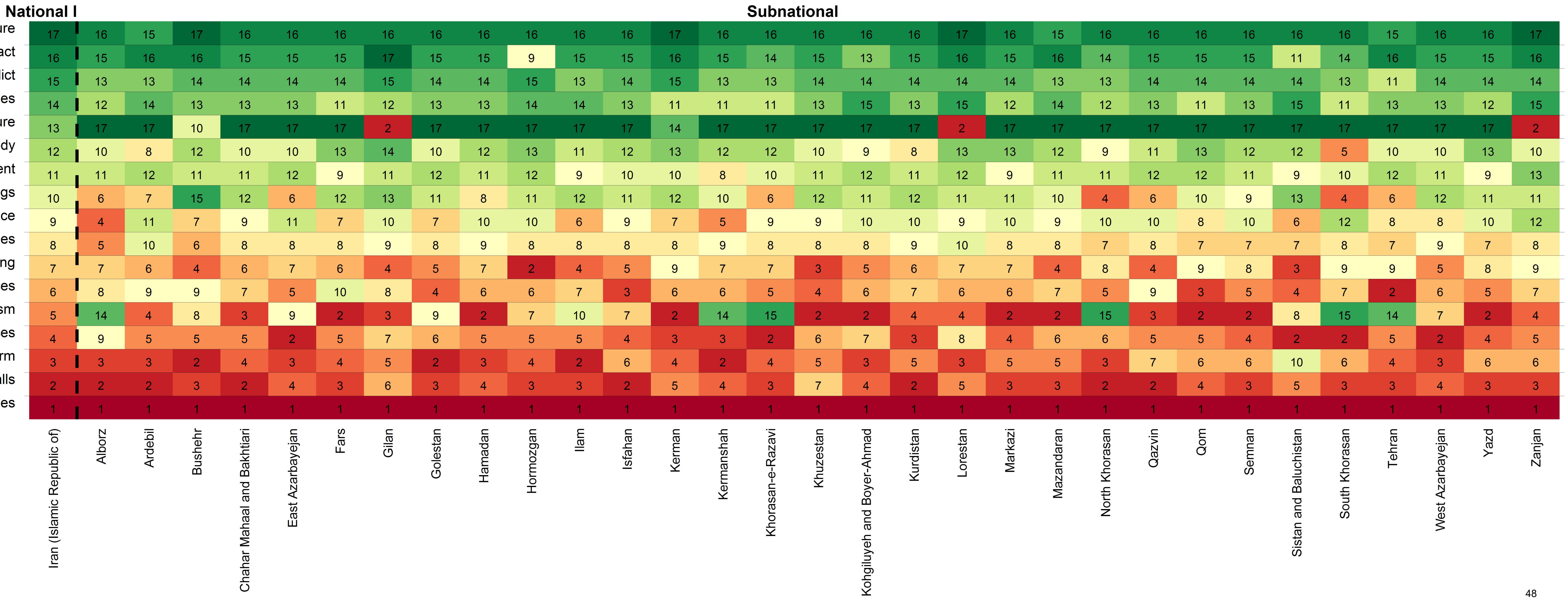

	National	Subnational																													
	Iran (Islamic Republic of)	Alborz	Ardebil	Bushehr	Chahar Mahaal and Bakhtiari	East Azarbayejan	Fars	Gilan	Golestan	Hamadan	Hormozgan	Ilam	Isfahan	Kerman	Kermanshah	Khorasan-e-Razavi	Khuzestan	Kohgiluyeh and Boyer-Ahmad	Kurdistan	Lorestan	Markazi	Mazandaran	North Khorasan	Qazvin	Qom	Semnan	Sistan and Baluchistan	South Khorasan	Tehran	West Azarbayejan	Yazd	Zanjan
Environmental heat and cold exposure	17	16	15	17	16	16	16	16	16	16	16	16	16	17	16	16	16	16	16	17	16	15	16	16	16	16	16	16	15	16	16	17
Animal contact	16	15	16	16	15	15	15	17	15	15	9	15	15	16	15	14	15	13	15	16	15	16	14	15	15	15	11	14	16	15	15	16
Executions and police conflict	15	13	13	14	14	14	14	15	14	14	15	13	14	15	13	13	14	14	14	14	14	13	13	14	14	14	14	13	11	14	14	14
Other transport injuries	14	12	14	13	13	13	11	12	13	13	14	14	13	11	11	11	13	15	13	15	12	14	12	13	11	13	15	11	13	13	12	15
Exposure to forces of nature	13	17	17	10	17	17	17	2	17	17	17	17	17	14	17	17	17	17	17	2	17	17	17	17	17	17	17	17	17	17	17	2
Foreign body	12	10	8	12	10	10	13	14	10	12	13	11	12	13	12	12	10	9	8	13	13	12	9	11	13	12	12	5	10	10	13	10
Adverse effects of medical treatment	11	11	12	11	11	12	9	11	12	11	12	9	10	10	8	10	11	12	11	12	9	11	11	12	12	11	9	10	12	11	9	13
Poisonings	10	6	7	15	12	6	12	13	11	8	11	12	11	12	10	6	12	11	12	11	11	10	4	6	10	9	13	4	6	12	11	11
Interpersonal violence	9	4	11	7	9	11	7	10	7	10	10	6	9	7	5	9	9	10	10	9	10	9	10	10	8	10	6	12	8	8	10	12
Other unintentional injuries	8	5	10	6	8	8	8	9	8	9	8	8	8	8	9	8	8	8	9	10	8	8	7	8	7	7	7	8	7	9	7	8
Drowning	7	7	6	4	6	7	6	4	5	7	2	4	5	9	7	7	3	5	6	7	7	4	8	4	9	8	3	9	9	5	8	9
Exposure to mechanical forces	6	8	9	9	7	5	10	8	4	6	6	7	3	6	6	5	4	6	7	6	6	7	5	9	3	5	4	7	2	6	5	7
Conflict and terrorism	5	14	4	8	3	9	2	3	9	2	7	10	7	2	14	15	2	2	4	4	2	2	15	3	2	2	8	15	14	7	2	4
Fire, heat, and hot substances	4	9	5	5	5	2	5	7	6	5	5	5	4	3	3	2	6	7	3	8	4	6	6	5	5	4	2	2	5	2	4	5
Self-harm	3	3	3	2	4	3	4	5	2	3	4	2	6	4	2	4	5	3	5	3	5	5	3	7	6	6	10	6	4	3	6	6
Falls	2	2	2	3	2	4	3	6	3	4	3	3	2	5	4	3	7	4	2	5	3	3	2	2	4	3	5	3	3	4	3	3
Road injuries	1	1	1	1	1	1	1	1	1	1	1	1	1	1	1	1	1	1	1	1	1	1	1	1	1	1	1	1	1	1	1	1

Appendix figure 17 2019

	National	Subnational																													
	Iran (Islamic Republic of)	Alborz	Ardebil	Bushehr	Chahar Mahaal and Bakhtiari	East Azarbayejan	Fars	Gilan	Golestan	Hamadan	Hormozgan	Ilam	Isfahan	Kerman	Kermanshah	Khorasan-e-Razavi	Khuzestan	Kohgiluyeh and Boyer-Ahmad	Kurdistan	Lorestan	Markazi	Mazandaran	North Khorasan	Qazvin	Qom	Semnan	Sistan and Baluchistan	South Khorasan	Tehran	West Azarbayejan	Yazd	Zanjan
Environmental heat and cold exposure	17	15	16	16	16	15	17	16	16	15	16	16	15	17	17	16	17	16	15	16	15	16	15	15	15	15	16	16	15	15	15	13
Animal contact	16	16	17	15	15	17	16	17	17	16	11	15	16	16	16	17	16	15	16	17	16	17	16	16	16	16	12	15	16	16	16	17
Adverse effects of medical treatment	15	13	11	12	12	13	14	11	14	12	14	11	14	12	10	11	13	14	12	12	11	14	11	13	12	14	11	12	13	13	13	16
Poisonings	14	7	9	14	13	8	13	15	13	10	15	14	11	14	13	10	15	13	13	11	12	13	9	10	14	10	15	10	10	14	12	10
Foreign body	13	12	12	13	9	10	12	14	11	11	13	13	12	13	14	12	11	11	11	14	14	15	10	12	11	11	14	9	9	12	14	11
Exposure to forces of nature	12	17	15	17	17	16	15	2	15	17	17	17	17	2	9	15	14	17	17	13	17	10	17	17	17	17	17	17	17	17	17	3
Executions and police conflict	11	10	14	10	11	11	11	13	10	14	12	9	13	11	11	9	12	12	14	10	10	11	13	14	10	12	8	11	8	10	9	12
Drowning	10	9	10	8	8	14	10	6	9	13	7	8	8	15	12	13	8	8	10	9	13	5	12	9	13	13	10	13	12	11	11	15
Conflict and terrorism	9	14	7	9	7	9	9	10	12	6	8	10	10	8	15	14	6	6	6	5	6	7	14	7	4	3	9	14	14	8	3	8
Other transport injuries	8	11	13	11	14	12	5	12	8	9	10	12	9	10	7	5	10	10	9	15	9	12	7	11	9	8	13	8	11	9	10	14
Fire, heat, and hot substances	7	8	8	7	10	6	8	9	7	8	9	7	7	9	5	7	9	9	8	8	8	9	8	8	8	9	6	7	5	6	8	9
Other unintentional injuries	6	5	6	5	6	7	6	8	6	7	6	6	6	7	8	8	7	7	7	7	7	8	6	6	7	7	7	5	7	7	6	7
Interpersonal violence	5	4	4	3	4	5	4	7	5	5	5	3	5	4	4	4	2	4	4	6	5	4	4	4	6	6	2	6	6	4	7	6
Exposure to mechanical forces	4	6	5	6	5	4	7	5	4	4	4	5	3	6	6	6	3	5	5	4	3	6	5	5	2	5	3	4	1	5	4	4
Self-harm	3	3	2	4	3	3	3	4	2	2	3	2	4	5	2	3	4	2	3	2	4	3	3	3	5	4	5	3	4	2	5	5
Falls	2	2	3	2	2	2	2	3	3	3	2	4	2	3	3	2	5	3	2	3	2	2	2	2	3	2	4	2	3	3	2	2
Road injuries	1	1	1	1	1	1	1	1	1	1	1	1	1	1	1	1	1	1	1	1	1	1	1	1	1	1	1	1	2	1	1	1

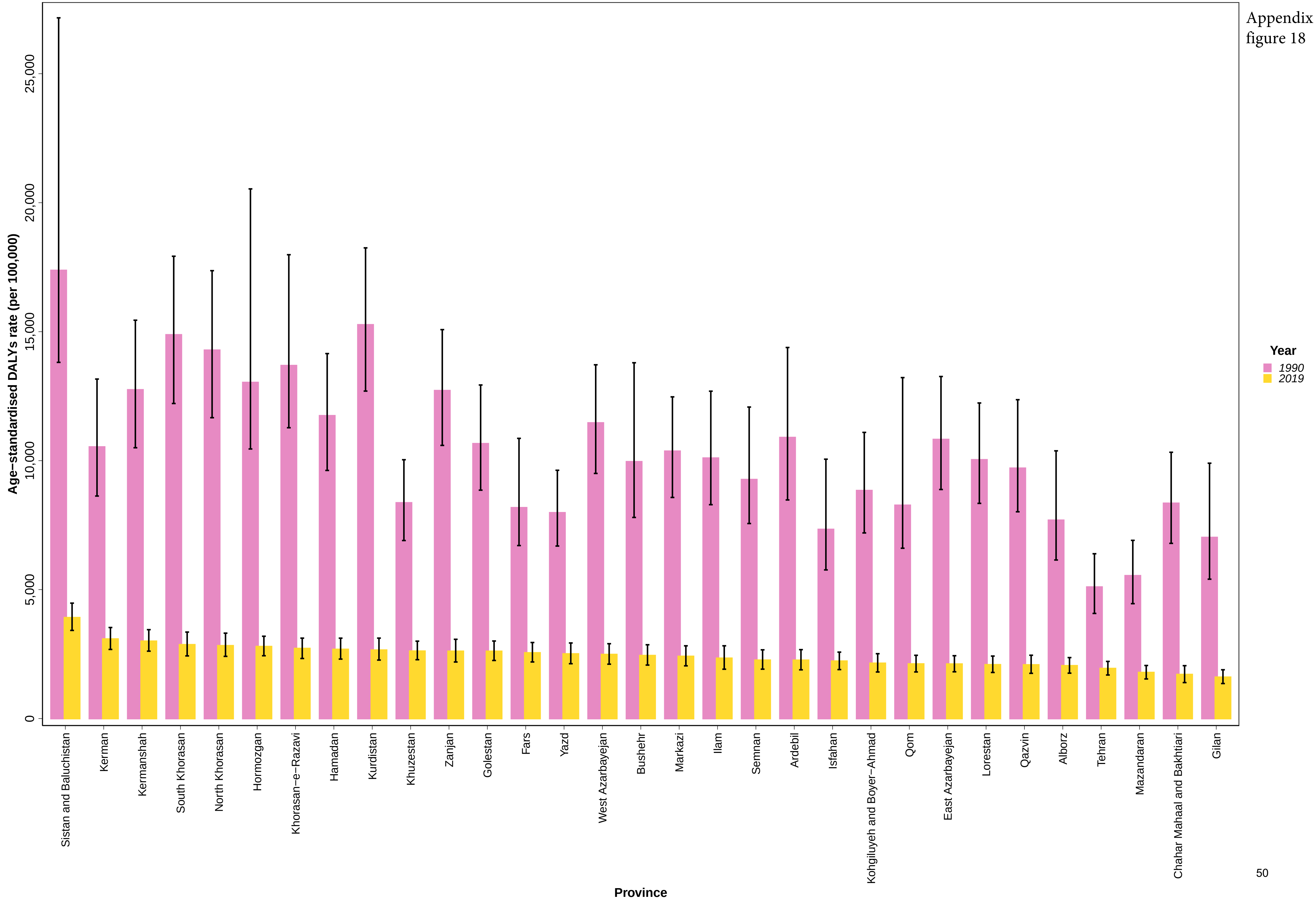

Appendix figure 18

Appendix figure 19

1990

	National	Subnational																														
	Iran (Islamic Republic of)	Alborz	Ardebil	Bushehr	Chahar Mahaal and Bakhtiari	East Azarbayejan	Fars	Gilan	Golestan	Hamadan	Hormozgan	Ilam	Isfahan	Kerman	Kermanshah	Khorasan-e-Razavi	Khuzestan	Kohgiluyeh and Boyer-Ahmad	Kurdistan	Lorestan	Markazi	Mazandaran	North Khorasan	Qazvin	Qom	Semnan	Sistan and Baluchistan	South Khorasan	Tehran	West Azarbayejan	Yazd	Zanjan
Leishmaniasis	25	19	14	26	35	17	15	35	28	29	22	26	28	29	30	26	31	17	35	35	35	28	14	35	16	35	35	33	25	35	34	35
Varicella and herpes zoster	24	24	24	25	25	23	26	24	22	24	21	25	24	22	25	24	24	26	24	23	23	24	22	22	23	23	21	24	23	23	23	22
Invasive Non-typhoidal Salmonella (iNTS)	23	23	22	24	23	22	22	22	24	22	26	24	22	24	24	23	22	23	21	22	21	22	23	24	22	22	27	23	19	22	22	24
Other nutritional deficiencies	22	22	25	20	24	25	23	21	23	23	25	20	21	23	22	22	23	25	25	25	22	20	25	21	26	24	24	21	20	25	21	25
Iodine deficiency	21	21	23	22	19	21	21	19	21	20	23	22	20	21	23	21	21	21	20	21	20	21	21	20	21	20	23	20	22	21	20	20
Tetanus	20	28	21	17	22	24	28	20	17	21	14	13	25	16	20	19	26	24	22	11	27	25	17	25	24	25	14	16	28	17	28	21
Encephalitis	19	20	19	21	21	20	20	23	20	18	20	21	19	20	19	18	20	20	17	20	19	19	20	18	20	19	19	18	16	20	19	19
Other neglected tropical diseases	18	18	20	19	18	19	19	18	18	19	18	19	18	18	18	17	18	19	19	19	18	18	18	19	19	18	17	17	18	18	18	17
Sexually transmitted infections excluding HIV	17	17	18	18	17	18	18	17	19	17	19	18	17	19	17	20	17	18	18	18	17	17	19	17	18	17	20	19	17	16	17	18
Otitis media	16	16	17	16	16	16	17	16	16	16	17	17	15	17	16	16	16	16	16	17	16	15	16	16	17	15	18	15	15	15	15	16
Other unspecified infectious diseases	15	13	15	15	15	15	16	14	15	15	15	16	16	15	15	15	15	13	15	15	15	14	12	15	15	16	15	13	14	14	16	15
Typhoid and paratyphoid	14	15	16	14	13	14	14	13	14	13	16	15	14	14	14	14	14	14	14	16	14	13	15	14	14	14	16	14	10	13	14	14
Upper respiratory infections	13	10	13	13	10	13	9	10	13	12	13	12	9	13	13	13	13	11	11	14	12	9	13	12	12	11	13	12	9	12	12	11
Acute hepatitis	12	14	10	11	8	12	10	15	10	8	12	10	13	11	12	12	11	10	12	9	9	16	11	11	10	12	11	11	13	11	13	13
Maternal disorders	11	11	11	8	11	10	8	11	12	10	11	14	10	10	11	11	12	9	10	13	13	11	10	9	11	8	12	10	11	9	10	12
Tuberculosis	10	9	12	10	14	11	12	9	4	11	9	11	12	8	8	5	7	15	9	12	8	12	9	13	7	7	6	9	6	10	7	9
Protein-energy malnutrition	9	12	9	5	12	6	13	12	9	9	7	9	11	12	6	9	9	12	8	4	11	10	8	10	13	10	7	5	12	7	11	10
Whooping cough	8	7	5	12	7	7	11	7	8	7	8	6	8	7	7	10	6	8	7	7	10	6	5	8	8	13	9	8	8	5	8	7
Meningitis	7	8	8	9	9	8	7	8	7	5	10	8	7	9	9	7	8	5	5	8	7	7	6	6	9	9	8	6	7	8	9	6
Malaria	6	4	7	4	6	9	4	6	11	14	4	7	4	4	10	6	10	7	13	10	6	8	32	7	4	5	4	32	29	19	6	8
Dietary iron deficiency	5	6	6	7	5	5	6	5	6	6	6	5	6	6	5	8	5	6	6	6	5	5	7	5	6	6	10	7	5	6	5	5
Measles	4	5	4	6	3	4	5	4	5	4	5	4	5	5	4	4	4	4	4	5	4	4	4	4	5	4	5	4	3	4	4	4
Diarrheal diseases	3	3	3	3	4	3	3	3	3	3	3	3	3	3	3	3	3	3	3	3	3	3	3	3	3	3	3	3	4	3	3	3
Lower respiratory infections	2	2	2	2	2	2	2	2	2	2	2	2	2	2	2	2	2	2	2	2	2	2	2	2	2	2	2	2	2	2	2	2
Neonatal disorders	1	1	1	1	1	1	1	1	1	1	1	1	1	1	1	1	1	1	1	1	1	1	1	1	1	1	1	1	1	1	1	1

Appendix figure 20 2019

National | **Subnational**

	Iran (Islamic Republic of)	Alborz	Ardebil	Bushehr	Chahar Mahaal and Bakhtiari	East Azarbayejan	Fars	Gilan	Golestan	Hamadan	Hormozgan	Ilam	Isfahan	Kerman	Kermanshah	Khorasan-e-Razavi	Khuzestan	Kohgiluyeh and Boyer-Ahmad	Kurdistan	Lorestan	Markazi	Mazandaran	North Khorasan	Qazvin	Qom	Semnan	Sistan and Baluchistan	South Khorasan	Tehran	West Azarbayejan	Yazd	Zanjan
Tetanus	25	28	29	25	29	29	29	26	25	27	25	25	27	25	28	28	29	29	29	23	28	27	26	28	29	28	25	25	27	26	28	29
Cystic echinococcosis	24	24	25	24	24	24	24	24	24	24	24	24	24	24	24	24	24	24	24	25	23	24	25	24	24	23	24	24	24	24	24	24
Varicella and herpes zoster	23	22	24	22	23	23	22	23	23	23	23	23	23	23	23	23	23	22	23	24	22	23	23	22	22	21	23	23	22	23	22	22
Iodine deficiency	22	19	21	19	18	22	20	19	21	22	21	20	19	21	21	19	22	19	22	21	19	19	22	19	20	18	22	21	23	22	19	19
Invasive Non-typhoidal Salmonella (iNTS)	21	20	22	20	20	20	19	22	22	21	22	22	20	22	22	21	21	20	21	22	20	21	21	21	19	19	21	22	19	21	20	21
Whooping cough	20	23	17	23	22	19	25	20	19	17	18	18	21	19	18	22	17	23	18	19	24	20	18	23	23	27	17	19	20	18	23	23
Measles	19	21	20	21	14	21	21	21	20	19	20	19	22	20	20	20	20	21	19	20	21	22	19	20	21	20	14	20	21	16	21	20
Other nutritional deficiencies	18	17	18	15	16	18	18	17	18	20	19	15	18	18	17	18	18	17	20	18	18	15	20	17	18	16	20	17	17	20	17	18
Other neglected tropical diseases	17	18	19	18	17	17	17	18	17	18	17	17	17	17	19	17	19	16	17	17	17	17	17	18	17	17	15	16	18	19	18	16
Encephalitis	16	15	16	17	15	14	16	15	13	15	15	14	13	16	16	16	16	14	14	16	14	13	14	14	15	15	18	13	15	17	16	13
Maternal disorders	15	16	15	13	13	15	14	16	14	16	12	16	15	13	14	11	13	12	15	14	16	16	15	12	16	11	10	12	16	13	11	17
Typhoid and paratyphoid	14	12	14	16	12	12	13	11	15	12	16	13	11	15	15	14	14	13	13	15	12	12	12	15	13	14	19	15	11	12	15	15
Sexually transmitted infections excluding HIV	13	14	12	14	9	13	12	12	12	11	14	11	10	14	13	15	15	7	12	13	13	11	13	11	14	12	13	14	12	14	13	11
Acute hepatitis	12	13	7	12	10	9	11	13	9	10	13	9	12	12	12	13	10	11	16	8	11	14	11	9	9	13	11	10	13	11	14	14
Otitis media	11	10	8	10	7	8	10	7	11	9	11	8	9	11	11	10	12	10	8	11	10	7	10	8	11	8	16	9	10	9	9	9
Other unspecified infectious diseases	10	9	10	9	8	10	8	9	7	7	10	10	8	8	10	8	11	8	9	9	7	9	7	10	12	10	7	11	14	8	12	8
Tuberculosis	9	11	11	11	19	11	15	10	5	13	7	12	14	10	9	6	7	15	11	12	9	10	9	13	7	7	5	6	8	10	7	12
Protein-energy malnutrition	8	6	6	7	6	6	9	6	10	8	9	6	7	9	8	9	9	9	7	7	8	6	8	7	10	6	12	7	7	6	6	7
Meningitis	7	8	9	8	11	7	7	8	8	6	8	7	6	7	7	7	8	6	6	10	6	8	6	6	8	9	9	8	9	7	8	6
HIV/AIDS	6	7	13	6	21	16	4	14	16	14	4	21	16	5	3	12	6	18	10	4	15	18	16	16	4	22	6	18	4	15	10	10
Upper respiratory infections	5	4	5	5	5	5	6	5	6	5	6	5	5	6	6	5	5	5	5	6	5	4	5	5	6	5	8	5	5	5	5	5
Dietary iron deficiency	4	5	4	4	4	4	5	4	4	4	5	4	4	4	5	4	4	4	4	5	4	5	4	4	5	4	4	4	6	4	4	4
Diarrheal diseases	3	3	2	3	2	3	3	2	3	3	3	2	3	3	4	3	3	2	3	3	3	3	3	3	3	3	3	3	3	3	3	3
Lower respiratory infections	2	2	3	2	3	2	2	3	2	2	2	3	2	2	2	2	2	3	2	2	2	2	2	2	2	2	2	2	2	2	2	2
Neonatal disorders	1	1	1	1	1	1	1	1	1	1	1	1	1	1	1	1	1	1	1	1	1	1	1	1	1	1	1	1	1	1	1	1

Appendix figure 21

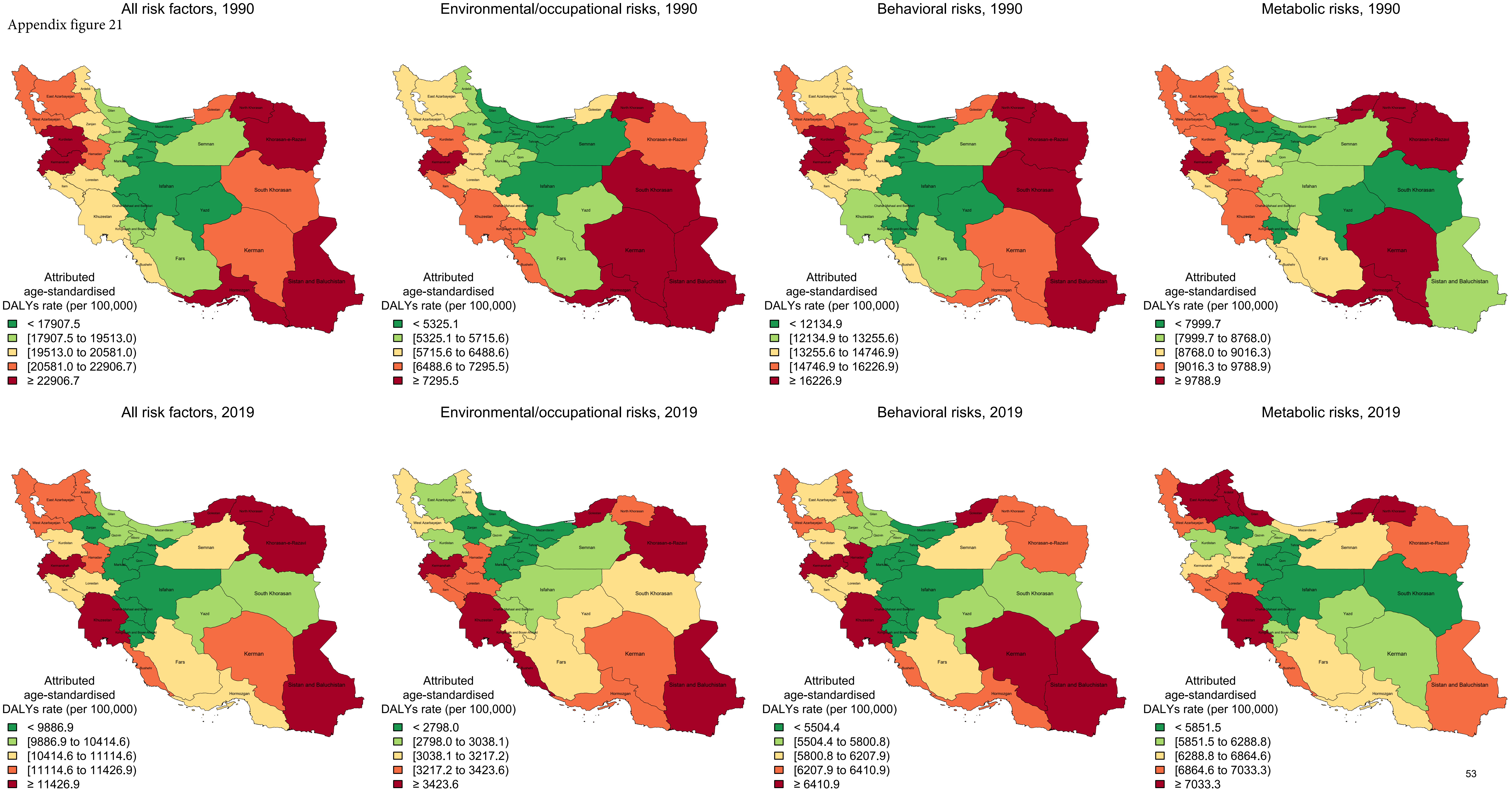

Appendix figure 22 1990

National | Subnational

	Iran (Islamic Republic of)	Alborz	Ardebil	Bushehr	Chahar Mahaal and Bakhtiari	East Azarbayejan	Fars	Gilan	Golestan	Hamadan	Hormozgan	Ilam	Isfahan	Kerman	Kermanshah	Khorasan-e-Razavi	Khuzestan	Kohgiluyeh and Boyer-Ahmad	Kurdistan	Lorestan	Markazi	Mazandaran	North Khorasan	Qazvin	Qom	Semnan	Sistan and Baluchistan	South Khorasan	Tehran	West Azarbayejan	Yazd	Zanjan
Diet low in fiber	25	23	26	27	20	25	24	20	22	23	27	23	25	25	22	24	27	22	22	22	24	24	26	26	26	25	28	28	29	23	26	27
Low bone mineral density	24	24	30	25	21	27	20	23	27	25	29	26	24	24	25	21	29	25	23	24	18	23	25	21	24	23	27	24	23	29	20	22
Diet low in vegetables	23	21	22	23	19	23	19	18	19	20	24	21	21	22	20	20	23	21	20	19	20	20	24	24	23	22	26	26	26	21	24	21
Diet high in red meat	22	19	21	19	22	22	21	19	20	24	25	22	19	23	21	25	19	24	24	23	23	19	28	25	22	20	29	29	19	22	21	25
Unsafe sanitation	21	32	20	21	29	21	28	31	21	21	17	19	28	19	23	19	25	20	19	26	26	29	18	22	25	26	13	16	33	19	25	19
Occupational injuries	20	20	19	20	17	20	22	21	24	22	23	20	18	26	24	26	21	18	21	20	22	18	22	20	17	18	24	21	17	20	18	20
Drug use	19	17	24	22	26	19	18	25	23	19	20	31	20	21	18	22	22	27	29	18	19	22	23	18	20	21	22	22	10	28	22	26
Diet low in nuts and seeds	18	18	18	17	18	17	17	17	17	17	19	18	17	18	17	18	18	19	18	17	17	17	20	19	18	17	23	23	21	18	17	18
Unsafe water source	17	27	17	18	27	18	23	24	18	18	13	17	23	17	19	16	20	17	17	21	21	27	15	17	19	19	12	14	25	17	19	17
Low physical activity	16	15	16	15	16	16	16	15	16	16	18	15	16	16	16	17	15	16	16	16	16	15	17	16	16	16	20	19	12	16	16	16
Diet high in trans fatty acids	15	14	14	13	13	15	13	13	14	15	15	13	14	13	15	15	13	13	15	15	15	13	16	15	15	15	16	18	16	14	15	15
Secondhand smoke	14	13	15	14	15	14	15	16	15	14	14	14	15	14	14	14	14	14	14	14	14	16	13	14	13	14	14	12	13	15	14	14
Low temperature	13	11	13	24	14	11	14	12	13	12	21	16	12	15	12	11	17	15	13	12	11	12	11	12	12	11	19	13	11	13	13	12
Diet low in legumes	12	12	12	12	10	13	10	10	12	13	12	12	10	12	13	13	12	12	12	13	13	10	14	13	14	13	15	15	14	12	12	13
Lead exposure	11	16	11	11	11	12	11	11	11	11	11	11	13	11	11	12	11	11	11	11	12	11	12	11	11	12	11	11	18	11	11	11
Child growth failure	10	10	9	10	12	9	12	14	10	8	7	10	11	10	10	8	10	10	7	9	10	14	5	10	10	10	4	4	15	8	10	7
Diet low in whole grains	9	8	10	8	7	10	7	7	9	7	10	8	9	8	7	10	8	7	9	8	9	8	10	9	9	9	10	10	9	7	9	8
Kidney dysfunction	8	9	8	9	9	7	8	9	7	9	8	7	8	7	8	9	9	9	8	10	7	9	9	8	8	8	8	9	8	10	8	10
High fasting plasma glucose	7	7	7	7	8	8	9	8	8	10	9	9	7	9	9	6	7	8	10	7	8	7	7	7	7	7	9	6	3	9	7	9
High body-mass index	6	6	6	6	6	6	6	6	6	6	6	6	6	6	6	7	6	6	6	6	6	5	8	6	6	6	7	8	4	6	6	6
Smoking	5	5	4	5	5	4	5	5	5	5	5	5	5	5	5	5	5	5	5	5	4	6	6	5	3	4	5	5	5	4	5	5
High LDL cholesterol	4	3	3	4	4	3	3	2	3	3	4	4	3	4	4	4	3	4	4	4	3	2	4	3	5	3	6	7	7	3	4	4
Particulate matter pollution	3	4	5	3	3	5	4	4	4	4	3	3	4	3	3	3	2	2	3	3	5	4	3	4	4	5	3	2	6	5	3	3
Low birth weight and short gestation	2	2	2	1	1	2	2	3	2	1	2	1	2	2	1	1	4	3	1	2	2	3	2	1	2	1	1	1	1	1	2	1
High systolic blood pressure	1	1	1	2	2	1	1	1	1	2	1	2	1	1	2	2	1	1	2	1	1	1	1	2	1	2	2	3	2	2	1	2

Appendix figure 23 2019

National | **Subnational**

Risk factor	Iran (Islamic Republic of)	Alborz	Ardebil	Bushehr	Chahar Mahaal and Bakhtiari	East Azarbayejan	Fars	Gilan	Golestan	Hamadan	Hormozgan	Ilam	Isfahan	Kerman	Kermanshah	Khorasan-e-Razavi
Diet low in seafood omega-3 fatty acids	25	23	22	25	23	24	24	21	19	21	25	23	25	27	24	25
Diet high in sodium	24	24	19	23	22	20	22	20	20	22	24	22	26	28	22	23
Unsafe water source	23	25	25	26	20	23	25	24	26	24	22	25	23	23	25	22
Intimate partner violence	22	19	23	21	28	25	21	25	28	27	26	17	22	22	21	26
Occupational injuries	21	22	20	22	17	21	23	23	25	23	23	24	21	25	23	21
Low bone mineral density	20	21	24	19	18	19	18	22	22	18	20	20	18	20	19	17
Alcohol use	19	18	21	20	24	17	17	19	18	17	18	21	19	17	17	18
Diet high in red meat	18	17	18	18	21	18	19	17	17	19	19	19	17	24	18	20
Occupational ergonomic factors	17	20	17	24	19	22	20	18	21	20	21	18	20	18	20	19
Diet high in trans fatty acids	16	15	14	14	14	16	16	15	15	15	15	14	16	16	16	16
Diet low in legumes	15	13	11	12	11	15	14	13	12	13	12	11	13	12	13	15
Secondhand smoke	14	14	15	13	15	14	15	14	14	16	14	15	12	11	15	13
Low physical activity	13	12	13	11	12	12	12	10	13	14	13	12	10	15	14	14
Low temperature	12	11	12	17	13	11	13	12	11	12	17	13	11	14	12	10
Lead exposure	11	16	10	10	10	10	11	11	10	11	10	10	15	10	11	11
Drug use	10	10	16	15	16	13	10	16	16	10	11	16	14	13	10	12
Diet low in whole grains	9	8	9	9	7	8	9	8	8	9	9	9	9	9	9	9
Low birth weight and short gestation	8	9	8	8	9	9	8	9	9	8	8	8	8	7	8	8
Kidney dysfunction	7	7	7	7	8	6	7	7	7	7	7	6	7	8	7	7
Particulate matter pollution	6	6	6	4	5	7	4	6	6	6	5	5	4	5	5	5
High LDL cholesterol	5	4	4	5	3	5	5	4	4	4	4	4	5	6	4	6
Smoking	4	5	5	6	6	4	6	5	5	5	6	7	6	4	6	4
High fasting plasma glucose	3	2	3	2	4	3	3	3	3	3	3	3	2	3	3	2
High body-mass index	2	3	2	3	2	2	2	2	2	2	2	2	3	2	2	3
High systolic blood pressure	1	1	1	1	1	1	1	1	1	1	1	1	1	1	1	1

Risk factor	Khuzestan	Kohgiluyeh and Boyer-Ahmad	Kurdistan	Lorestan	Markazi	Mazandaran	North Khorasan	Qazvin	Qom	Semnan	Sistan and Baluchistan	South Khorasan	Tehran	West Azarbayejan	Yazd	Zanjan
Diet low in seafood omega-3 fatty acids	20	23	25	22	27	24	26	24	26	24	29	30	30	25	29	25
Diet high in sodium	22	24	22	21	24	22	21	22	25	22	25	26	28	23	23	24
Unsafe water source	27	25	23	25	23	26	22	23	22	23	19	21	24	24	22	22
Intimate partner violence	21	21	24	29	22	21	24	25	20	26	26	24	18	18	25	23
Occupational injuries	23	20	19	23	20	23	23	21	17	21	21	20	16	19	21	19
Low bone mineral density	26	17	18	19	17	19	18	17	21	17	22	18	22	22	18	18
Alcohol use	19	22	20	20	19	20	19	18	19	19	18	19	21	21	20	21
Diet high in red meat	18	18	21	18	21	18	20	19	18	18	23	23	20	20	17	20
Occupational ergonomic factors	24	19	17	17	18	17	17	20	23	20	27	17	17	17	19	17
Diet high in trans fatty acids	15	13	15	15	16	15	16	16	16	16	14	16	15	15	16	16
Diet low in legumes	13	11	12	13	15	13	13	13	15	14	12	15	14	13	15	12
Secondhand smoke	12	15	14	16	13	12	12	15	14	13	13	12	12	14	11	15
Low physical activity	11	12	13	14	14	10	14	14	13	11	15	14	11	12	10	13
Low temperature	16	14	11	12	10	11	11	12	11	10	16	13	13	11	13	11
Lead exposure	10	10	10	9	11	14	10	11	10	12	10	10	19	10	12	10
Drug use	14	16	16	11	12	16	15	10	12	15	11	11	9	16	14	14
Diet low in whole grains	8	8	9	8	9	8	9	8	8	9	9	9	10	9	9	9
Low birth weight and short gestation	9	9	7	10	8	9	8	9	9	8	8	7	7	8	7	7
Kidney dysfunction	7	7	8	7	7	7	7	7	7	7	6	8	8	7	8	8
Particulate matter pollution	4	4	6	6	6	6	6	6	6	6	4	4	5	6	4	6
High LDL cholesterol	5	3	5	4	5	4	4	5	5	5	5	6	6	4	6	5
Smoking	6	6	4	5	4	5	5	4	4	4	7	5	4	5	5	4
High fasting plasma glucose	2	5	3	3	3	3	3	3	2	3	3	1	1	2	1	3
High body-mass index	3	2	2	2	2	2	2	2	3	2	2	3	2	3	3	2
High systolic blood pressure	1	1	1	1	1	1	1	1	1	1	1	2	3	1	2	1

Appendix Table 1. Population (in thousands) by location and sex in 1990, 2000, 2010, and 2019

Location	Sex	Population (in thousands)			
		1990	2000	2010	2019
Iran (Islamic Republic of)	Both	58540 (53285 to 63545)	67312 (61417 to 73395)	76581 (71120 to 82068)	84298 (77331 to 91936)
	Female	28618 (26049 to 31064)	32971 (30083 to 35950)	37830 (35133 to 40541)	41480 (38052 to 45239)
	Male	29922 (27236 to 32481)	34341 (31334 to 37444)	38750 (35987 to 41527)	42817 (39279 to 46697)
Alborz	Both	1525 (1346 to 1729)	2039 (1795 to 2320)	2467 (2234 to 2735)	2979 (2637 to 3360)
	Female	735 (649 to 834)	980 (862 to 1114)	1210 (1096 to 1341)	1462 (1294 to 1649)
	Male	790 (697 to 896)	1060 (933 to 1205)	1257 (1139 to 1394)	1517 (1343 to 1711)
Ardebil	Both	1172 (1033 to 1320)	1250 (1098 to 1408)	1294 (1166 to 1429)	1300 (1148 to 1461)
	Female	571 (504 to 643)	620 (545 to 699)	640 (576 to 706)	634 (560 to 712)
	Male	601 (530 to 677)	630 (553 to 710)	655 (590 to 723)	666 (588 to 749)
Bushehr	Both	725 (638 to 815)	815 (716 to 920)	1033 (929 to 1139)	1243 (1101 to 1394)
	Female	357 (314 to 401)	396 (348 to 447)	473 (426 to 522)	587 (520 to 659)
	Male	368 (324 to 414)	419 (368 to 473)	560 (504 to 617)	656 (581 to 735)
Chahar Mahaal and Bakhtiari	Both	743 (653 to 838)	843 (738 to 956)	927 (834 to 1025)	1005 (886 to 1133)
	Female	362 (319 to 409)	418 (366 to 474)	460 (414 to 508)	493 (434 to 555)
	Male	381 (335 to 429)	425 (373 to 482)	467 (420 to 516)	513 (452 to 578)
East Azarbayejan	Both	3477 (3041 to 3892)	3551 (3092 to 3980)	3800 (3399 to 4165)	4108 (3608 to 4581)
	Female	1704 (1491 to 1908)	1743 (1518 to 1954)	1873 (1675 to 2053)	2013 (1768 to 2245)
	Male	1772 (1550 to 1984)	1807 (1574 to 2026)	1927 (1724 to 2113)	2095 (1840 to 2336)
Fars	Both	3724 (3244 to 4190)	4169 (3635 to 4670)	4680 (4175 to 5153)	5063 (4444 to 5658)
	Female	1819 (1584 to 2046)	2042 (1782 to 2288)	2315 (2065 to 2549)	2483 (2179 to 2774)
	Male	1906 (1660 to 2144)	2126 (1854 to 2382)	2365 (2110 to 2604)	2580 (2265 to 2884)
Gilan	Both	2358 (2071 to 2669)	2401 (2104 to 2718)	2538 (2277 to 2812)	2631 (2318 to 2961)
	Female	1169 (1027 to 1323)	1203 (1054 to 1362)	1275 (1144 to 1412)	1306 (1151 to 1470)
	Male	1189 (1044 to 1346)	1198 (1050 to 1356)	1263 (1133 to 1400)	1325 (1167 to 1492)
Golestan	Both	1357 (1193 to 1543)	1550 (1368 to 1758)	1807 (1626 to 2006)	1953 (1729 to 2202)
	Female	673 (591 to 765)	779 (688 to 884)	905 (814 to 1004)	971 (859 to 1094)
	Male	685 (601 to 778)	770 (680 to 874)	903 (812 to 1002)	983 (870 to 1108)
Hamadan	Both	1695 (1483 to 1919)	1753 (1526 to 1982)	1817 (1627 to 2009)	1753 (1540 to 1977)
	Female	820 (717 to 928)	868 (756 to 982)	901 (807 to 996)	862 (757 to 972)
	Male	875 (766 to 991)	885 (770 to 1000)	916 (820 to 1013)	891 (783 to 1005)
Hormozgan	Both	932 (811 to 1065)	1236 (1081 to 1409)	1586 (1420 to 1770)	1911 (1682 to 2167)
	Female	454 (395 to 519)	594 (519 to 677)	776 (695 to 866)	931 (820 to 1056)
	Male	478 (416 to 546)	642 (561 to 732)	810 (725 to 904)	980 (862 to 1111)
Ilam	Both	453 (397 to 511)	537 (470 to 609)	572 (514 to 631)	598 (527 to 670)
	Female	219 (192 to 247)	262 (229 to 297)	282 (253 to 311)	293 (258 to 328)
	Male	234 (205 to 264)	274 (240 to 311)	290 (261 to 320)	305 (269 to 342)
Isfahan	Both	3881 (3422 to 4390)	4298 (3773 to 4885)	4921 (4451 to 5440)	5319 (4720 to 6005)
	Female	1873 (1651 to 2119)	2086 (1831 to 2370)	2414 (2183 to 2668)	2617 (2322 to 2954)
	Male	2008 (1771 to 2271)	2212 (1942 to 2514)	2507 (2268 to 2772)	2703 (2398 to 3051)
Kerman	Both	1893 (1669 to 2131)	2327 (2046 to 2616)	2971 (2689 to 3269)	3375 (2996 to 3771)
	Female	930 (820 to 1047)	1136 (999 to 1277)	1468 (1328 to 1615)	1637 (1452 to 1828)
	Male	963 (849 to 1084)	1191 (1048 to 1339)	1503 (1360 to 1654)	1739 (1543 to 1942)
Kermanshah	Both	1721 (1510 to 1939)	1903 (1675 to 2148)	2009 (1812 to 2221)	2002 (1771 to 2251)
	Female	832 (730 to 937)	924 (813 to 1043)	992 (894 to 1096)	987 (873 to 1110)
	Male	889 (780 to 1002)	979 (862 to 1105)	1017 (917 to 1125)	1015 (897 to 1141)
Khorasan-e-Razavi	Both	4814 (4223 to 5430)	5277 (4622 to 5943)	6131 (5529 to 6746)	6807 (6032 to 7634)
	Female	2374 (2083 to 2678)	2624 (2299 to 2955)	3059 (2759 to 3365)	3364 (2981 to 3773)
	Male	2440 (2140 to 2752)	2653 (2324 to 2988)	3072 (2770 to 3381)	3443 (3051 to 3861)
Khuzestan	Both	3268 (2867 to 3684)	4175 (3677 to 4691)	4655 (4202 to 5132)	4974 (4398 to 5580)
	Female	1597 (1402 to 1800)	2032 (1790 to 2283)	2297 (2074 to 2533)	2447 (2164 to 2745)
	Male	1671 (1466 to 1883)	2143 (1888 to 2408)	2358 (2128 to 2599)	2527 (2234 to 2835)

Location	Sex	Population (in thousands)			
		1990	2000	2010	2019
Kohgiluyeh and Boyer-Ahmad	Both	496 (438 to 566)	612 (540 to 697)	680 (615 to 755)	754 (669 to 854)
	Female	242 (214 to 276)	301 (266 to 343)	339 (307 to 377)	368 (327 to 417)
	Male	254 (224 to 289)	311 (274 to 354)	341 (308 to 378)	386 (342 to 437)
Kurdistan	Both	1262 (1111 to 1428)	1448 (1280 to 1644)	1536 (1384 to 1701)	1688 (1493 to 1915)
	Female	617 (543 to 698)	711 (628 to 807)	763 (687 to 845)	828 (732 to 939)
	Male	645 (568 to 730)	737 (652 to 837)	773 (697 to 857)	861 (761 to 976)
Lorestan	Both	1568 (1374 to 1778)	1721 (1511 to 1950)	1821 (1637 to 2023)	1792 (1582 to 2029)
	Female	764 (670 to 866)	841 (739 to 953)	902 (810 to 1001)	883 (779 to 999)
	Male	804 (704 to 911)	880 (772 to 997)	920 (827 to 1021)	910 (803 to 1030)
Markazi	Both	1223 (1070 to 1383)	1320 (1156 to 1492)	1442 (1296 to 1597)	1458 (1282 to 1643)
	Female	605 (529 to 684)	656 (575 to 741)	711 (639 to 787)	717 (630 to 808)
	Male	619 (541 to 699)	664 (581 to 751)	731 (657 to 810)	742 (652 to 835)
Mazandaran	Both	2654 (2321 to 2985)	2855 (2475 to 3228)	3132 (2810 to 3446)	3490 (3074 to 3905)
	Female	1319 (1153 to 1483)	1425 (1236 to 1612)	1558 (1398 to 1714)	1732 (1525 to 1938)
	Male	1335 (1168 to 1502)	1430 (1239 to 1616)	1574 (1413 to 1732)	1758 (1548 to 1967)
North Khorasan	Both	636 (556 to 718)	764 (670 to 861)	895 (805 to 989)	874 (774 to 982)
	Female	315 (275 to 355)	386 (338 to 435)	450 (404 to 496)	433 (383 to 486)
	Male	321 (281 to 363)	378 (331 to 426)	446 (401 to 492)	441 (391 to 495)
Qazvin	Both	953 (835 to 1081)	1115 (970 to 1273)	1226 (1105 to 1358)	1333 (1177 to 1512)
	Female	464 (407 to 526)	543 (472 to 620)	603 (543 to 668)	652 (575 to 739)
	Male	489 (428 to 555)	572 (498 to 654)	623 (562 to 690)	682 (602 to 773)
Qom	Both	753 (657 to 856)	963 (845 to 1091)	1157 (1037 to 1285)	1386 (1218 to 1566)
	Female	365 (319 to 415)	468 (410 to 530)	565 (506 to 627)	678 (596 to 766)
	Male	388 (339 to 441)	496 (435 to 561)	592 (531 to 657)	707 (622 to 799)
Semnan	Both	489 (421 to 555)	555 (477 to 629)	637 (564 to 705)	758 (656 to 855)
	Female	237 (204 to 269)	268 (230 to 304)	314 (278 to 347)	372 (322 to 420)
	Male	252 (217 to 286)	287 (247 to 326)	323 (286 to 358)	386 (334 to 435)
Sistan and Baluchistan	Both	1486 (1299 to 1673)	2095 (1828 to 2359)	2610 (2337 to 2887)	3001 (2636 to 3364)
	Female	732 (640 to 824)	1023 (893 to 1152)	1299 (1163 to 1437)	1479 (1299 to 1658)
	Male	754 (659 to 849)	1072 (935 to 1207)	1311 (1173 to 1450)	1522 (1337 to 1707)
South Khorasan	Both	679 (599 to 772)	653 (574 to 744)	680 (613 to 755)	844 (747 to 955)
	Female	334 (294 to 379)	321 (282 to 365)	338 (304 to 375)	414 (367 to 469)
	Male	346 (305 to 393)	332 (292 to 379)	342 (309 to 380)	430 (380 to 486)
Tehran	Both	8634 (7577 to 9755)	10517 (9323 to 11836)	12301 (11137 to 13588)	14146 (12597 to 15825)
	Female	4195 (3680 to 4740)	5080 (4503 to 5719)	6063 (5489 to 6698)	7019 (6251 to 7852)
	Male	4440 (3896 to 5015)	5437 (4820 to 6117)	6238 (5648 to 6891)	7127 (6346 to 7973)
West Azarbayejan	Both	2373 (2074 to 2710)	2723 (2383 to 3097)	3127 (2806 to 3472)	3480 (3067 to 3944)
	Female	1165 (1018 to 1330)	1336 (1169 to 1519)	1546 (1387 to 1716)	1710 (1507 to 1937)
	Male	1208 (1056 to 1380)	1387 (1214 to 1577)	1581 (1419 to 1756)	1770 (1561 to 2006)
Yazd	Both	699 (614 to 794)	877 (773 to 994)	1085 (980 to 1202)	1182 (1047 to 1333)
	Female	337 (296 to 383)	422 (372 to 478)	524 (473 to 580)	573 (508 to 646)
	Male	362 (318 to 411)	455 (401 to 516)	562 (507 to 622)	609 (540 to 686)
Zanjan	Both	896 (785 to 1001)	972 (842 to 1099)	1042 (934 to 1144)	1089 (956 to 1218)
	Female	440 (385 to 491)	484 (419 to 547)	520 (466 to 571)	537 (471 to 600)
	Male	456 (400 to 510)	488 (423 to 552)	522 (468 to 573)	552 (485 to 618)

Data in parentheses are 95% uncertainty intervals

Appendix Table 2. Life expectancy at birth (years) by location and sex in 1990, 1991, 2000, 2010, and 2019

Location	Sex	Life expectancy at birth (years)				
		1990	1991	2000	2010	2019
Iran (Islamic Republic of)	Both	66.7 (65.9 to 67.4)	68.9 (68.2 to 69.6)	71.9 (71.5 to 72.4)	75.5 (75.2 to 75.7)	77.8 (77.5 to 78)
	Female	69.7 (69 to 70.4)	71.6 (70.9 to 72.3)	74.3 (73.8 to 74.7)	77.8 (77.5 to 78.1)	79.6 (79.2 to 79.9)
	Male	64.1 (63.2 to 65)	66.6 (65.8 to 67.5)	69.9 (69.3 to 70.5)	73.3 (72.9 to 73.6)	76.1 (75.6 to 76.5)
Alborz	Both	71.1 (69.3 to 72.8)	71.2 (69.4 to 73)	73.3 (72.3 to 74.5)	77.1 (76.2 to 77.9)	78.3 (77.1 to 79.6)
	Female	73.2 (71.2 to 75.3)	73.3 (71.3 to 75.3)	74.9 (73.7 to 76.3)	78.4 (77.6 to 79.4)	79.8 (78.4 to 81.1)
	Male	69.2 (66.5 to 71.8)	69.4 (66.8 to 72)	72.1 (70.4 to 73.9)	75.8 (74.5 to 77.2)	76.9 (74.8 to 78.9)
Ardebil	Both	65.6 (63.6 to 67.4)	66.1 (64.3 to 68)	71.2 (70.2 to 72.2)	74.9 (74.3 to 75.5)	76.2 (75.4 to 77.2)
	Female	68.5 (66.4 to 70.5)	69 (67 to 71)	73.4 (72.3 to 74.4)	78.1 (77.3 to 78.8)	79.3 (78.4 to 80.3)
	Male	63.2 (60.6 to 65.6)	63.8 (61.2 to 66.3)	69.2 (67.9 to 70.8)	72.2 (71.3 to 73.1)	73.6 (72.2 to 75)
Bushehr	Both	68.6 (66.8 to 70.5)	68.8 (67.1 to 70.5)	71.5 (70.8 to 72.2)	73.8 (73.3 to 74.3)	77 (76 to 77.8)
	Female	71.2 (69.2 to 73.2)	71.5 (69.5 to 73.3)	73.9 (73.1 to 74.7)	76.3 (75.7 to 76.8)	79.2 (78.3 to 80.1)
	Male	66.4 (63.9 to 69)	66.6 (64.2 to 69)	69.4 (68.5 to 70.4)	71.8 (71.1 to 72.3)	75 (73.7 to 76.2)
Chahar Mahaal and Bakhtiari	Both	69.6 (67.7 to 71.4)	70 (68.3 to 71.7)	73.2 (72 to 74.5)	76.1 (75.3 to 77.1)	79.2 (78 to 80.5)
	Female	72.8 (70.6 to 74.9)	73.1 (71.1 to 75.1)	76.1 (74.7 to 77.7)	79.4 (78.4 to 80.5)	82.7 (80.9 to 84.1)
	Male	67 (64.1 to 69.5)	67.4 (64.8 to 69.8)	70.8 (69 to 72.7)	73.4 (72.2 to 74.7)	76.3 (74.7 to 77.9)
East Azarbayejan	Both	66.5 (64.6 to 68.2)	67.1 (65.3 to 68.8)	70.3 (69.1 to 71.6)	73.5 (72.7 to 74.4)	76 (74.7 to 77.2)
	Female	69.2 (67 to 71.3)	69.7 (67.6 to 71.7)	72.6 (71.2 to 74)	76.1 (75.2 to 77.1)	77.9 (76.5 to 79.2)
	Male	64.1 (61.6 to 66.9)	64.8 (62.4 to 67.5)	68.3 (66.5 to 70.2)	71.3 (70 to 72.5)	74.2 (72.3 to 76)
Fars	Both	68.5 (66.4 to 70.4)	68.7 (66.9 to 70.5)	71.2 (69.9 to 72.5)	74 (72.9 to 75)	76.5 (75.2 to 78)
	Female	72 (69.7 to 74.1)	72.2 (70.1 to 74.3)	74.4 (73.1 to 75.9)	76.9 (75.9 to 78)	79 (77.5 to 80.5)
	Male	65.5 (62.8 to 68.4)	65.8 (63.2 to 68.6)	68.5 (66.6 to 70.6)	71.3 (69.8 to 72.9)	74.3 (72.1 to 76.4)
Gilan	Both	45.7 (43.7 to 47.6)	70 (68.2 to 71.7)	72.4 (71.3 to 73.4)	75.6 (74.8 to 76.5)	77.5 (76.2 to 78.7)
	Female	51.9 (49.9 to 53.8)	72.7 (70.6 to 74.8)	74.9 (73.7 to 76.1)	77.9 (77.1 to 78.8)	79.2 (77.8 to 80.6)
	Male	40.9 (38.8 to 42.8)	67.4 (64.7 to 70)	70.1 (68.5 to 71.8)	73.4 (72.2 to 74.8)	75.9 (73.9 to 77.8)
Golestan	Both	66.2 (64.3 to 67.9)	66.5 (64.8 to 68.2)	69.3 (68.4 to 70.1)	73.1 (72.6 to 73.7)	74.5 (73.5 to 75.5)
	Female	69.3 (67.2 to 71.3)	69.6 (67.6 to 71.6)	72 (71.1 to 72.9)	76.1 (75.5 to 76.6)	77.3 (76.1 to 78.4)
	Male	63.5 (61 to 66.1)	63.9 (61.5 to 66.5)	66.8 (65.6 to 68.1)	70.4 (69.5 to 71.2)	72 (70.4 to 73.6)
Hamadan	Both	66 (64.1 to 67.9)	66.5 (64.4 to 68.4)	70.8 (69.5 to 72.1)	74 (73.1 to 75.1)	76.3 (75 to 77.7)
	Female	69.2 (66.8 to 71.2)	69.6 (67.4 to 71.6)	73.3 (72 to 74.7)	77.1 (76.2 to 78)	78.8 (77.5 to 80.2)
	Male	63.3 (60.5 to 66.2)	63.9 (61.1 to 66.7)	68.5 (66.6 to 70.5)	71.1 (69.7 to 72.7)	73.9 (71.8 to 76)
Hormozgan	Both	66 (64.1 to 67.8)	66.3 (64.5 to 68.1)	69.9 (68.8 to 70.9)	74.1 (73.4 to 74.8)	77.4 (76.1 to 78.5)
	Female	69.3 (67.2 to 71.3)	69.5 (67.5 to 71.5)	72.8 (71.7 to 74)	76.6 (75.9 to 77.5)	79.6 (78.6 to 80.7)
	Male	63.4 (60.7 to 65.9)	63.7 (61.3 to 66.3)	67.5 (66 to 69)	71.7 (70.6 to 72.9)	75.2 (73.2 to 77.3)
Ilam	Both	67.8 (66 to 69.5)	68.1 (66.3 to 69.7)	71.1 (70.2 to 72)	74.1 (73.4 to 74.7)	76.4 (75.5 to 77.3)
	Female	70.6 (68.7 to 72.7)	70.9 (69 to 72.8)	73.6 (72.6 to 74.6)	76.5 (75.8 to 77.2)	78.5 (77.4 to 79.6)
	Male	65.7 (63.2 to 68)	66 (63.5 to 68.4)	69.1 (67.8 to 70.3)	71.9 (70.9 to 72.8)	74.6 (73.1 to 76.1)
Isfahan	Both	71.4 (69.4 to 73.3)	71.7 (69.7 to 73.5)	74.3 (73.1 to 75.5)	76.9 (76.1 to 77.7)	78.4 (77.1 to 79.6)
	Female	73.9 (71.9 to 76)	74.2 (72 to 76.2)	76.5 (75.2 to 77.8)	79.1 (78.2 to 80)	80 (78.6 to 81.3)
	Male	69.1 (66.3 to 71.8)	69.5 (66.8 to 72.1)	72.4 (70.6 to 74.2)	75 (73.7 to 76.3)	76.8 (74.9 to 78.8)
Kerman	Both	66.6 (64.7 to 68.4)	66.9 (65 to 68.7)	69.6 (68.5 to 70.7)	73.9 (73.3 to 74.6)	76.7 (75.6 to 77.8)
	Female	70.4 (68.2 to 72.5)	70.6 (68.6 to 72.7)	73.1 (71.9 to 74.3)	76.9 (76.1 to 77.6)	78.8 (77.6 to 80.1)
	Male	63.5 (61 to 66.1)	63.8 (61.4 to 66.5)	66.7 (65.1 to 68.3)	71.2 (70.3 to 72.2)	74.7 (73 to 76.4)
Kermanshah	Both	64.4 (62.4 to 66.5)	64.8 (62.9 to 66.7)	68.2 (66.9 to 69.4)	72.9 (71.9 to 73.8)	76 (74.5 to 77.3)
	Female	68.2 (66 to 70.3)	68.5 (66.4 to 70.7)	71.4 (70 to 72.9)	75.5 (74.6 to 76.5)	78.1 (76.6 to 79.6)
	Male	61.6 (58.8 to 64.3)	62 (59.2 to 64.5)	65.5 (63.7 to 67.4)	70.4 (68.9 to 71.9)	73.9 (71.8 to 76.1)
Khorasan-e-Razavi	Both	63.9 (61.9 to 65.7)	64.4 (62.5 to 66.2)	68.3 (67.2 to 69.4)	73 (72.3 to 73.7)	76.2 (75.1 to 77.3)
	Female	66.9 (64.8 to 69)	67.4 (65.2 to 69.6)	71 (69.6 to 72.5)	75.8 (75 to 76.6)	78.2 (76.9 to 79.5)
	Male	61.3 (58.6 to 64)	61.8 (59.3 to 64.6)	65.9 (64.4 to 67.6)	70.5 (69.4 to 71.4)	74.3 (72.6 to 76)
Khuzestan	Both	68.7 (67 to 70.3)	69 (67.3 to 70.8)	71.1 (70 to 72.2)	74.1 (73.3 to 74.9)	76.1 (74.9 to 77.4)
	Female	71.7 (69.7 to 73.8)	71.9 (70 to 74)	73.5 (72.4 to 74.7)	76.8 (75.9 to 77.6)	77.9 (76.5 to 79.3)
	Male	66.1 (63.4 to 68.6)	66.6 (64 to 69.1)	69 (67.4 to 70.7)	71.7 (70.6 to 72.8)	74.5 (72.6 to 76.4)

Location	Sex	Life expectancy at birth (years)				
		1990	1991	2000	2010	2019
Kohgiluyeh and Boyer-Ahmad	Both	68.6 (66.5 to 70.6)	69 (66.9 to 70.8)	72.4 (70.8 to 74)	76.1 (74.9 to 77.6)	78 (76.3 to 79.5)
	Female	71 (68.8 to 73.3)	71.3 (69.1 to 73.5)	74.2 (72.4 to 76)	78 (76.4 to 79.6)	79.3 (77.4 to 81.1)
	Male	66.6 (63.4 to 69.6)	67 (64 to 69.9)	70.8 (68.5 to 73.4)	74.4 (72.4 to 76.6)	76.7 (74.1 to 79.2)
Kurdistan	Both	63.1 (61 to 65)	63.9 (62.1 to 65.9)	69.3 (68.1 to 70.5)	73.7 (72.9 to 74.6)	77.3 (76.1 to 78.3)
	Female	66.7 (64.4 to 68.7)	67.4 (65.3 to 69.5)	72.4 (71.1 to 73.8)	76.6 (75.7 to 77.5)	79.4 (78.4 to 80.6)
	Male	60.4 (57.7 to 63.1)	61.3 (58.7 to 63.8)	66.9 (65.1 to 68.7)	71.1 (69.9 to 72.5)	75.3 (73.3 to 77.3)
Lorestan	Both	67.5 (65.6 to 69.3)	67.5 (65.7 to 69.3)	71.3 (70.2 to 72.5)	74.6 (73.7 to 75.5)	76.9 (75.6 to 78.3)
	Female	71 (68.8 to 73.1)	71 (69 to 73.1)	74.4 (73.2 to 75.7)	78.3 (77.3 to 79.3)	80.7 (79.3 to 82.5)
	Male	64.8 (62.1 to 67.4)	64.7 (62.1 to 67.1)	68.9 (67.1 to 70.7)	71.6 (70.2 to 72.9)	73.8 (72 to 75.4)
Markazi	Both	67.3 (65.3 to 69.2)	67.8 (66 to 69.6)	71.2 (70 to 72.4)	75.9 (75.1 to 76.8)	78.5 (77.3 to 79.8)
	Female	70.7 (68.5 to 72.7)	71.1 (69 to 73)	74.1 (72.9 to 75.4)	78.4 (77.5 to 79.4)	80.3 (78.9 to 81.6)
	Male	64.4 (61.6 to 67.1)	64.9 (62.1 to 67.8)	68.5 (66.7 to 70.4)	73.5 (72.2 to 74.9)	76.9 (74.9 to 78.9)
Mazandaran	Both	71.8 (70 to 73.6)	72.2 (70.4 to 73.9)	74.1 (72.9 to 75.2)	76.5 (75.6 to 77.4)	78.2 (76.9 to 79.5)
	Female	74.1 (72 to 76.3)	74.4 (72.3 to 76.5)	75.9 (74.6 to 77.3)	78.8 (77.9 to 79.9)	79.9 (78.4 to 81.4)
	Male	69.7 (67.1 to 72.4)	70.2 (67.5 to 72.9)	72.3 (70.6 to 74.2)	74.3 (73.1 to 75.7)	76.6 (74.6 to 78.6)
North Khorasan	Both	64 (62 to 65.8)	64.5 (62.8 to 66.1)	69 (68 to 70)	74 (73.2 to 74.7)	76 (75.1 to 77.1)
	Female	66.8 (64.6 to 68.8)	67.2 (65.2 to 69.2)	71.2 (70.1 to 72.2)	76.4 (75.6 to 77.1)	77.3 (76.2 to 78.5)
	Male	61.6 (59.1 to 64)	62.1 (59.8 to 64.5)	67 (65.6 to 68.4)	71.7 (70.6 to 72.6)	74.7 (73.2 to 76.2)
Qazvin	Both	68.6 (66.9 to 70.2)	69.1 (67.4 to 70.9)	72.3 (71.3 to 73.4)	75.1 (74.4 to 75.9)	77.8 (76.6 to 79)
	Female	71.4 (69.3 to 73.3)	71.8 (69.9 to 73.8)	74.8 (73.6 to 76.1)	78.3 (77.6 to 79.2)	81.1 (79.7 to 82.6)
	Male	66.1 (63.7 to 68.7)	66.7 (64.3 to 69.4)	70.2 (68.7 to 71.7)	72.3 (71.3 to 73.4)	74.9 (73.5 to 76.5)
Qom	Both	69.3 (67.6 to 70.9)	69.5 (67.9 to 71.2)	71.9 (71 to 72.9)	75.7 (75 to 76.4)	78.9 (77.9 to 80)
	Female	71.7 (69.9 to 73.8)	71.9 (70.2 to 73.8)	73.9 (72.9 to 74.9)	77 (76.3 to 77.8)	79.7 (78.6 to 80.8)
	Male	67.2 (64.7 to 69.9)	67.5 (65.1 to 70.1)	70.2 (68.7 to 71.6)	74.4 (73.3 to 75.4)	78 (76.3 to 79.7)
Semnan	Both	68.9 (67.2 to 70.5)	69.1 (67.4 to 70.7)	71.7 (70.8 to 72.7)	74.8 (74.1 to 75.5)	77.7 (76.6 to 78.9)
	Female	72.3 (70.2 to 74.5)	72.6 (70.5 to 74.6)	75 (73.8 to 76.3)	77.8 (76.9 to 78.7)	80.6 (79.3 to 82.2)
	Male	65.8 (63.2 to 68.1)	66.2 (63.8 to 68.5)	68.9 (67.6 to 70.4)	72.1 (71.2 to 73.2)	75.1 (73.8 to 76.6)
Sistan and Baluchistan	Both	63.5 (61.4 to 65.7)	64 (62.1 to 66)	68.8 (67.2 to 70.5)	72.5 (71.3 to 73.6)	74.7 (73.1 to 76.5)
	Female	67.3 (65 to 69.8)	67.8 (65.5 to 70.2)	71.8 (70.1 to 73.6)	75.5 (74.2 to 76.8)	77.1 (75 to 79.2)
	Male	60.5 (57.6 to 63.3)	61.1 (58.3 to 63.9)	66.3 (64.1 to 68.7)	69.8 (68.1 to 71.5)	72.6 (70.2 to 75.1)
South Khorasan	Both	66 (64.2 to 67.9)	66.5 (64.6 to 68.3)	71.6 (70.5 to 72.6)	75.4 (74.6 to 76.2)	78.1 (77.1 to 79.1)
	Female	69.2 (67.2 to 71.4)	69.6 (67.6 to 71.7)	74 (72.9 to 75.2)	77.9 (77 to 78.7)	80.1 (78.9 to 81.3)
	Male	63.3 (60.7 to 65.8)	63.8 (61.3 to 66.2)	69.3 (67.9 to 70.8)	73.1 (72 to 74.1)	76.2 (74.6 to 77.7)
Tehran	Both	76.2 (74.3 to 78)	76.5 (74.7 to 78.3)	78.5 (77.5 to 79.7)	81.4 (80.7 to 82.2)	82.7 (81.5 to 84)
	Female	77.4 (75.2 to 79.4)	77.6 (75.5 to 79.6)	79.3 (78 to 80.6)	82 (81.1 to 83)	82.8 (81.4 to 84.3)
	Male	75.3 (72.5 to 77.9)	75.6 (72.8 to 78.2)	78 (76.3 to 79.8)	80.8 (79.7 to 82.1)	82.6 (80.7 to 84.5)
West Azarbayejan	Both	65.5 (63.8 to 67.3)	66 (64.4 to 67.7)	69.4 (68.2 to 70.4)	73.9 (73.3 to 74.5)	75.9 (74.9 to 76.9)
	Female	68.5 (66.3 to 70.6)	69 (67 to 70.9)	71.9 (70.8 to 73.1)	76.5 (75.8 to 77.2)	77.9 (76.7 to 79)
	Male	63 (60.5 to 65.6)	63.6 (61.3 to 66)	67.2 (65.7 to 68.7)	71.5 (70.7 to 72.4)	74 (72.4 to 75.6)
Yazd	Both	70.2 (68.6 to 72)	70.4 (68.8 to 72.2)	72.4 (71.2 to 73.8)	74.9 (73.9 to 75.8)	77.8 (76.5 to 79)
	Female	73 (71 to 75.2)	73.2 (71.1 to 75.2)	75 (73.6 to 76.5)	77.6 (76.6 to 78.7)	79.7 (78.3 to 81.1)
	Male	67.7 (65.3 to 70.5)	67.9 (65.5 to 70.5)	70.2 (68.4 to 72.1)	72.5 (71.1 to 73.9)	76 (74.2 to 77.9)
Zanjan	Both	44.2 (42.4 to 45.9)	68.9 (67.2 to 70.7)	72.1 (71.2 to 73)	75.6 (75.1 to 76.1)	78.4 (77.5 to 79.3)
	Female	48.8 (47 to 50.6)	72.2 (70.3 to 74.2)	75.4 (74.4 to 76.3)	79.2 (78.6 to 79.8)	81.3 (80.2 to 82.4)
	Male	41 (39.1 to 42.7)	66.1 (63.6 to 68.6)	69.4 (68.1 to 70.5)	72.5 (71.8 to 73.2)	75.8 (74.5 to 77.1)

Data in parentheses are 95% uncertainty intervals
Due to the major impact of the 1990’s Rudbar earthquake on the life expectancy of Gilan and Zanjan provinces, 1991 is reported as well.

Appendix Table 3. Healthy life expectancy at birth (years) by location and sex in 1990, 1991, 2000, 2010, and 2019

**Location**	**Sex**	**Healthy life expectancy at birth (years)**				
		**1990**	**1991**	**2000**	**2010**	**2019**
Iran (Islamic Republic of)	Both	57.8 (55.1 to 60.1)	59.6 (56.9 to 62.1)	62.3 (59.5 to 64.8)	65 (62 to 67.6)	66.8 (63.7 to 69.7)
	Female	59.3 (56.3 to 62.1)	60.9 (57.8 to 63.6)	63.2 (59.9 to 66.1)	65.8 (62.3 to 68.9)	67.2 (63.6 to 70.4)
	Male	56.5 (54.2 to 58.5)	58.6 (56.2 to 60.8)	61.5 (59.2 to 63.7)	64.2 (61.5 to 66.5)	66.4 (63.7 to 68.9)
Alborz	Both	61.7 (58.6 to 64.6)	61.8 (58.9 to 64.6)	63.7 (60.8 to 66.3)	66.4 (63.2 to 69.2)	67.4 (64.2 to 70.5)
	Female	62.5 (59.1 to 65.5)	62.6 (59.2 to 65.9)	63.9 (60.6 to 67)	66.6 (63.1 to 69.7)	67.7 (63.9 to 71)
	Male	61 (57.9 to 63.9)	61.2 (58 to 64)	63.5 (60.7 to 66.1)	66.3 (63.3 to 69)	67.2 (64.1 to 70.1)
Ardebil	Both	56.8 (53.9 to 59.4)	57.3 (54.4 to 60.1)	61.7 (58.8 to 64.2)	64.7 (61.6 to 67.4)	65.9 (62.7 to 68.7)
	Female	58.3 (54.9 to 61.3)	58.7 (55.5 to 61.6)	62.4 (59.1 to 65.4)	66.1 (62.6 to 69.2)	67.2 (63.5 to 70.4)
	Male	55.7 (52.7 to 58.3)	56.2 (53.2 to 59.1)	61 (58.5 to 63.5)	63.6 (61 to 65.9)	64.7 (62 to 67.3)
Bushehr	Both	59.6 (56.7 to 62.2)	59.8 (57.1 to 62.6)	62.1 (59.5 to 64.6)	64 (61.1 to 66.5)	66.5 (63.5 to 69.2)
	Female	60.8 (57.3 to 63.9)	61 (57.6 to 64.4)	63.1 (59.8 to 66)	64.8 (61.4 to 67.8)	67.1 (63.6 to 70.3)
	Male	58.6 (55.7 to 61.5)	58.9 (56.1 to 61.7)	61.4 (58.9 to 63.5)	63.3 (60.8 to 65.5)	66 (63.3 to 68.5)
Chahar Mahaal and Bakhtiari	Both	60.5 (57.5 to 63.2)	60.8 (57.9 to 63.5)	63.7 (60.8 to 66.3)	66.1 (63 to 68.9)	68.5 (65.2 to 71.4)
	Female	62.2 (58.8 to 65.3)	62.5 (59 to 65.5)	65 (61.8 to 68.2)	67.6 (64.1 to 70.9)	70.1 (66.3 to 73.6)
	Male	59.2 (56.3 to 62)	59.5 (56.6 to 62.4)	62.6 (59.9 to 65.2)	64.9 (62.1 to 67.4)	67.2 (64.2 to 69.8)
East Azarbayejan	Both	58 (55.2 to 60.7)	58.6 (55.8 to 61.1)	61.4 (58.8 to 63.8)	63.9 (61.1 to 66.4)	65.9 (63 to 68.6)
	Female	59.5 (56.4 to 62.5)	59.9 (56.8 to 62.8)	62.4 (59.2 to 65.2)	65 (61.8 to 67.9)	66.6 (63.3 to 69.7)
	Male	56.9 (54 to 59.7)	57.4 (54.7 to 60.1)	60.5 (58 to 62.9)	62.9 (60.4 to 65.4)	65.4 (62.6 to 68.1)
Fars	Both	59.1 (56 to 62)	59.4 (56.4 to 62.3)	61.6 (58.8 to 64.2)	63.7 (60.6 to 66.3)	65.8 (62.7 to 68.8)
	Female	61.1 (57.5 to 64.5)	61.3 (58.1 to 64.6)	63.2 (60 to 66.1)	65.1 (61.7 to 68.2)	66.9 (63.3 to 70.2)
	Male	57.5 (54.3 to 60.3)	57.7 (54.5 to 60.8)	60.2 (57.5 to 62.8)	62.4 (59.4 to 64.8)	64.8 (61.8 to 67.9)
Gilan	Both	40.1 (37.8 to 42.4)	60.3 (57.4 to 63.2)	62.7 (60 to 65.2)	65.3 (62.4 to 68.1)	66.8 (63.8 to 69.6)
	Female	44.7 (41.9 to 47.2)	61.8 (58.3 to 65.2)	63.9 (60.5 to 66.8)	66.2 (62.8 to 69.3)	67.2 (63.7 to 70.6)
	Male	36.5 (34.5 to 38.7)	59.1 (55.9 to 62.1)	61.7 (59.2 to 64.2)	64.4 (61.8 to 66.9)	66.4 (63.3 to 69.2)
Golestan	Both	57.6 (54.8 to 60.3)	58 (55.1 to 60.6)	60.4 (57.7 to 62.9)	63.4 (60.5 to 65.9)	64.6 (61.8 to 67.2)
	Female	59.3 (56.1 to 62.3)	59.6 (56.3 to 62.5)	61.7 (58.7 to 64.5)	64.8 (61.5 to 67.8)	65.9 (62.4 to 69.1)
	Male	56.3 (53.3 to 59.1)	56.7 (53.9 to 59.6)	59.3 (56.8 to 61.6)	62 (59.5 to 64.4)	63.5 (60.8 to 66.1)
Hamadan	Both	57.3 (54.4 to 59.9)	57.8 (54.9 to 60.6)	61.5 (58.8 to 64.1)	64.1 (61.4 to 66.8)	66 (63 to 68.8)
	Female	59 (55.8 to 62.1)	59.4 (56.2 to 62.7)	62.7 (59.5 to 65.6)	65.6 (62.3 to 68.6)	67.1 (63.6 to 70.2)
	Male	55.9 (52.7 to 58.9)	56.4 (53.3 to 59.4)	60.5 (57.8 to 63.1)	62.6 (60.1 to 65.1)	65 (61.9 to 67.8)
Hormozgan	Both	57.2 (54.4 to 59.7)	57.5 (54.5 to 60.2)	60.7 (58.1 to 63.4)	64 (61.2 to 66.6)	66.6 (63.5 to 69.6)
	Female	59 (55.6 to 62.1)	59.3 (55.8 to 62.4)	62.1 (58.9 to 65)	65.2 (62 to 68)	67.5 (64 to 70.7)
	Male	55.9 (53 to 58.6)	56.2 (53.2 to 59)	59.6 (57.2 to 62.3)	62.9 (60.4 to 65.4)	65.8 (62.7 to 68.9)
Ilam	Both	59 (56.2 to 61.8)	59.3 (56.5 to 61.9)	61.9 (59.3 to 64.4)	64.2 (61.3 to 66.8)	66.1 (63 to 68.9)
	Female	60.3 (56.9 to 63.4)	60.5 (57.3 to 63.5)	62.8 (59.7 to 65.7)	65.1 (61.6 to 68)	66.7 (63.1 to 69.7)
	Male	58.2 (55.1 to 61)	58.5 (55.6 to 61.2)	61.3 (58.9 to 63.7)	63.4 (60.8 to 65.8)	65.6 (62.7 to 68.4)
Isfahan	Both	61.9 (58.9 to 64.8)	62.3 (59.2 to 65.3)	64.4 (61.5 to 67.2)	66.3 (63.2 to 69.1)	67.5 (64.2 to 70.5)
	Female	63.1 (59.7 to 66.4)	63.4 (59.8 to 66.9)	65.2 (61.7 to 68.4)	66.9 (63.3 to 70.2)	67.8 (64 to 71.1)
	Male	61 (58 to 64.1)	61.4 (58 to 64.6)	63.8 (60.9 to 66.5)	65.7 (62.9 to 68.2)	67.3 (64.1 to 70.1)
Kerman	Both	57.6 (54.7 to 60.5)	57.9 (54.8 to 60.4)	60.3 (57.5 to 62.9)	63.2 (60.3 to 66)	65.6 (62.4 to 68.5)
	Female	59.7 (56.2 to 63.1)	60 (56.5 to 63.1)	62.1 (58.9 to 65.3)	64.6 (61.2 to 67.7)	66.3 (62.6 to 69.5)
	Male	55.9 (52.9 to 58.9)	56.2 (53.3 to 59.2)	58.8 (56.3 to 61.2)	62 (59.3 to 64.5)	64.9 (62.1 to 67.7)
Kermanshah	Both	56.2 (53.5 to 59)	56.5 (53.7 to 59.3)	59.3 (56.6 to 61.7)	63 (60 to 65.6)	65.5 (62.4 to 68.5)
	Female	58.4 (55.1 to 61.7)	58.6 (55.5 to 61.8)	61.1 (57.8 to 63.8)	64.2 (60.8 to 67)	66.3 (62.7 to 69.6)
	Male	54.5 (52 to 57.5)	54.9 (52 to 57.9)	57.9 (55.4 to 60.4)	61.8 (58.9 to 64.4)	64.7 (61.6 to 68)
Khorasan-e-Razavi	Both	55.6 (52.8 to 58.1)	56 (53.1 to 58.9)	59.4 (56.7 to 61.9)	62.9 (60 to 65.6)	65.4 (62.3 to 68.4)
	Female	57.2 (53.9 to 60.2)	57.6 (54.3 to 60.6)	60.6 (57.5 to 63.5)	64.1 (60.8 to 67.1)	66.1 (62.6 to 69.5)
	Male	54.3 (51.4 to 57.4)	54.8 (51.9 to 57.8)	58.3 (55.9 to 60.7)	61.8 (59.2 to 64.2)	64.8 (61.8 to 67.6)
Khuzestan	Both	59.3 (56.4 to 62.1)	59.6 (56.6 to 62.4)	61.6 (58.8 to 64.1)	63.8 (60.9 to 66.5)	65.5 (62.4 to 68.4)
	Female	60.8 (57.5 to 64)	61.1 (57.7 to 64.1)	62.6 (59.3 to 65.5)	64.9 (61.5 to 68)	66 (62.6 to 69.2)
	Male	58 (55 to 60.8)	58.4 (55.4 to 61.4)	60.7 (58.2 to 63.3)	62.8 (60.2 to 65.1)	65.1 (62.2 to 67.9)

Location	Sex	Healthy life expectancy at birth (years)				
		1990	1991	2000	2010	2019
Kohgiluyeh and Boyer-Ahmad	Both	59.2 (56.1 to 62)	59.5 (56.8 to 62.4)	62.7 (59.6 to 65.5)	65.7 (62.6 to 68.6)	67.3 (63.9 to 70.4)
	Female	60.3 (57 to 63.6)	60.6 (57.2 to 63.8)	63.2 (59.7 to 66.3)	66.2 (62.9 to 69.3)	67.4 (63.7 to 70.9)
	Male	58.3 (55.1 to 61.5)	58.7 (55.3 to 61.9)	62.2 (59.1 to 65.2)	65.2 (62.2 to 68.2)	67.2 (64 to 70.3)
Kurdistan	Both	55 (52.2 to 57.7)	55.7 (53 to 58.2)	60.3 (57.8 to 62.8)	63.9 (61 to 66.5)	66.8 (63.8 to 69.7)
	Female	57 (53.7 to 59.9)	57.7 (54.7 to 60.7)	61.8 (58.8 to 65)	65.2 (62 to 68.1)	67.6 (64.1 to 70.7)
	Male	53.4 (50.5 to 56.5)	54.2 (51.2 to 57.1)	59.2 (56.6 to 61.5)	62.6 (60 to 65)	66.1 (63 to 69.1)
Lorestan	Both	58.7 (55.8 to 61.3)	58.7 (55.8 to 61.4)	62.1 (59.4 to 64.7)	64.8 (61.9 to 67.5)	66.7 (63.6 to 69.7)
	Female	60.6 (57.3 to 63.9)	60.6 (57.5 to 63.7)	63.6 (60.2 to 66.7)	66.7 (63.3 to 69.6)	68.6 (65.1 to 71.9)
	Male	57.2 (54.2 to 59.9)	57.2 (54.3 to 60)	61 (58.5 to 63.5)	63.3 (60.5 to 65.8)	65.2 (62.3 to 67.9)
Markazi	Both	58.2 (55.1 to 61)	58.6 (55.6 to 61.4)	61.6 (58.8 to 64.3)	65.3 (62.1 to 68)	67.5 (64.2 to 70.4)
	Female	60.1 (56.8 to 63.2)	60.4 (57 to 63.6)	63 (59.6 to 66.1)	66.3 (62.7 to 69.5)	67.9 (64.4 to 71.2)
	Male	56.6 (53.5 to 59.6)	57.1 (54.1 to 60)	60.3 (57.6 to 63)	64.3 (61.4 to 66.8)	67.1 (63.9 to 70)
Mazandaran	Both	61.5 (58.6 to 64.4)	61.9 (58.6 to 64.9)	63.6 (60.6 to 66.5)	65.5 (62.3 to 68.5)	66.8 (63.3 to 69.8)
	Female	61.9 (58.2 to 65.3)	62.1 (58.4 to 65.7)	63.6 (60 to 66.8)	65.7 (61.9 to 69.2)	66.5 (62.5 to 70.1)
	Male	61.3 (58.3 to 64.3)	61.7 (58.4 to 64.6)	63.7 (60.8 to 66.4)	65.3 (62.5 to 68)	67.1 (63.9 to 69.8)
North Khorasan	Both	55.5 (52.6 to 58.2)	55.9 (53.1 to 58.5)	59.9 (57.1 to 62.4)	63.8 (60.6 to 66.3)	65.4 (62.3 to 68.3)
	Female	56.9 (53.7 to 60.1)	57.3 (53.9 to 60.3)	60.7 (57.4 to 63.5)	64.7 (61.3 to 67.7)	65.6 (62 to 68.7)
	Male	54.4 (51.5 to 57.3)	54.8 (52 to 57.5)	59.1 (56.6 to 61.5)	62.8 (60 to 65.1)	65.3 (62.4 to 68.2)
Qazvin	Both	59.4 (56.7 to 62.3)	59.9 (57 to 62.6)	62.8 (60 to 65.3)	65.1 (62.1 to 67.7)	67.2 (64.1 to 70.1)
	Female	60.7 (57.4 to 63.9)	61.2 (58.1 to 64.2)	63.8 (60.4 to 66.5)	66.6 (63.1 to 69.7)	68.7 (65.1 to 72)
	Male	58.4 (55.5 to 61.2)	58.9 (55.7 to 61.8)	62.1 (59.5 to 64.5)	63.8 (61.3 to 66.1)	66 (63.2 to 68.5)
Qom	Both	59.6 (56.6 to 62.4)	59.9 (57.1 to 62.8)	62.2 (59.2 to 64.7)	65.2 (62.3 to 67.8)	67.8 (64.6 to 70.7)
	Female	60.8 (57.2 to 64.1)	61 (57.6 to 64.1)	62.9 (59.7 to 65.7)	65.3 (62 to 68.4)	67.6 (64.1 to 70.8)
	Male	58.7 (55.6 to 61.5)	59 (56.2 to 62)	61.6 (58.8 to 64.1)	65 (62.4 to 67.5)	68 (64.8 to 70.8)
Semnan	Both	59.5 (56.6 to 62.2)	59.8 (57 to 62.5)	62.2 (59.4 to 64.7)	64.7 (61.7 to 67.3)	67.1 (63.7 to 70)
	Female	61.5 (58 to 64.6)	61.7 (58.3 to 64.9)	64 (60.8 to 66.9)	66.1 (62.7 to 69.2)	68.3 (64.7 to 71.7)
	Male	57.8 (54.9 to 60.7)	58.1 (55.3 to 60.9)	60.8 (58.2 to 63.2)	63.5 (60.9 to 65.8)	66 (63.2 to 68.7)
Sistan and Baluchistan	Both	55.1 (52.2 to 58)	55.6 (52.7 to 58.3)	59.7 (56.8 to 62.4)	62.5 (59.6 to 65.2)	64.5 (61.3 to 67.4)
	Female	57.4 (53.9 to 60.4)	57.8 (54.8 to 61)	61.2 (57.9 to 64.3)	63.9 (60.5 to 67)	65.4 (61.5 to 68.8)
	Male	53.4 (50.5 to 56.4)	53.9 (51 to 56.8)	58.4 (55.5 to 61.3)	61.3 (58.4 to 64)	63.7 (60.5 to 66.8)
South Khorasan	Both	57.1 (54.1 to 59.9)	57.5 (54.6 to 60.1)	61.7 (58.9 to 64.5)	64.7 (61.5 to 67.6)	66.8 (63.6 to 69.9)
	Female	58.7 (55.4 to 61.9)	59.1 (55.7 to 62.3)	62.7 (59.3 to 65.7)	65.6 (61.9 to 68.7)	67.3 (63.6 to 70.8)
	Male	55.7 (52.7 to 58.6)	56.2 (53.3 to 59)	60.9 (58.4 to 63.4)	63.9 (61.2 to 66.5)	66.4 (63.5 to 69.3)
Tehran	Both	65.3 (61.8 to 68.4)	65.6 (62.1 to 68.5)	67.3 (64.2 to 70.4)	69.3 (65.9 to 72.4)	70.2 (66.7 to 73.6)
	Female	65.3 (61.5 to 68.8)	65.5 (61.8 to 69)	66.9 (63.2 to 70.2)	68.8 (65 to 72.3)	69.4 (65.2 to 73)
	Male	65.4 (61.9 to 68.8)	65.7 (62.3 to 68.9)	67.8 (64.8 to 70.7)	69.8 (66.7 to 72.7)	71.1 (67.6 to 74.6)
West Azarbayejan	Both	56.9 (54.3 to 59.5)	57.4 (54.8 to 60.1)	60.3 (57.6 to 62.8)	63.8 (60.9 to 66.4)	65.6 (62.3 to 68.3)
	Female	58.3 (55 to 61.4)	58.7 (55.7 to 61.8)	61.2 (58 to 64.2)	64.7 (61.3 to 67.8)	66 (62.4 to 69.1)
	Male	55.8 (53.1 to 58.5)	56.3 (53.7 to 59)	59.6 (57.3 to 61.9)	63.1 (60.7 to 65.3)	65.2 (62.5 to 67.7)
Yazd	Both	60.7 (57.8 to 63.6)	60.9 (57.9 to 63.7)	62.8 (60 to 65.5)	64.7 (61.5 to 67.5)	67 (63.6 to 69.9)
	Female	62.1 (58.9 to 65.4)	62.3 (58.9 to 65.6)	64 (60.8 to 67)	65.9 (62.3 to 69)	67.7 (64.1 to 71)
	Male	59.5 (56.6 to 62.6)	59.7 (56.8 to 62.6)	61.8 (59.3 to 64.7)	63.6 (60.7 to 66.3)	66.5 (63.2 to 69.2)
Zanjan	Both	38.7 (36.6 to 40.8)	58.9 (56 to 61.8)	62 (59.1 to 64.8)	65 (61.9 to 67.7)	67.4 (64.3 to 70.3)
	Female	41.8 (39.2 to 44.1)	60.7 (57.2 to 64)	63.6 (60.2 to 66.7)	66.8 (63.2 to 69.9)	68.6 (65.1 to 72)
	Male	36.5 (34.5 to 38.5)	57.5 (54.6 to 60.4)	60.7 (58.1 to 63.2)	63.6 (60.9 to 65.9)	66.4 (63.5 to 69)

Data in parentheses are 95% uncertainty intervals

Due to the major impact of the 1990's Rudbar earthquake on the healthy life expectancy of Gilan and Zanjan provinces, 1991 is reported as well.

Appendix Table 4. Total fertility rate and under-5 mortality rate per 1,000 live births by location in 1990 and 2019

Location	Total fertility rate		Under-5 mortality rate (per 1,000 live births)	
	1990	2019	1990	2019
Iran (Islamic Republic of)	4.2 (3.7 to 4.6)	1.8 (1.4 to 2.4)	71.2 (63.6 to 79.1)	11.1 (10.2 to 12)
Alborz	4.2 (3.3 to 5.3)	1.6 (1.2 to 2.2)	49.5 (42.7 to 56.9)	7.2 (6.6 to 7.9)
Ardebil	4.5 (4 to 4.9)	1.8 (1.4 to 2.3)	102.6 (90.5 to 115.6)	12.6 (11.5 to 13.7)
Bushehr	4.6 (4.1 to 5)	2 (1.6 to 2.6)	62.5 (51.6 to 75.4)	12.1 (10.4 to 14)
Chahar Mahaal and Bakhtiari	4.7 (4.3 to 5.2)	2 (1.6 to 2.6)	60.8 (53.3 to 68.7)	7.8 (6.7 to 9.1)
East Azarbayejan	4 (3.6 to 4.4)	1.8 (1.4 to 2.3)	79 (69.4 to 89.4)	9.6 (8.8 to 10.4)
Fars	4.1 (3.7 to 4.6)	1.7 (1.3 to 2.2)	55.3 (48.4 to 62.4)	12.4 (11.3 to 13.4)
Gilan	3.7 (3.3 to 4.1)	1.4 (1.1 to 1.8)	99.4 (88.6 to 111.9)	5.5 (4.5 to 6.5)
Golestan	4.5 (4 to 4.9)	2.1 (1.6 to 2.7)	77.2 (68.2 to 87.1)	12.4 (11.3 to 13.4)
Hamadan	4.3 (3.8 to 4.8)	1.7 (1.4 to 2.2)	85 (75 to 95.8)	13 (11.9 to 14.1)
Hormozgan	5 (4.5 to 5.5)	2.1 (1.7 to 2.8)	82.5 (72.6 to 92.9)	12.4 (11.4 to 13.5)
Ilam	4.8 (4.3 to 5.3)	1.5 (1.2 to 1.9)	73 (64 to 82.8)	12.4 (10.2 to 14.7)
Isfahan	3.9 (3.4 to 4.4)	1.4 (1.1 to 1.8)	50.6 (42.5 to 60.5)	10.8 (9.9 to 11.7)
Kerman	4.5 (4.1 to 5)	2.1 (1.6 to 2.7)	66 (57.7 to 75.1)	14.8 (13.6 to 16)
Kermanshah	4.1 (3.7 to 4.5)	1.7 (1.3 to 2.2)	84.7 (74.4 to 95.7)	13.1 (12 to 14.2)
Khorasan-e-Razavi	4.6 (4.1 to 5.1)	2.1 (1.7 to 2.7)	92.5 (80.8 to 105.3)	12.2 (11.2 to 13.2)
Khuzestan	4.6 (4.1 to 5.1)	2.1 (1.7 to 2.8)	57.6 (50.7 to 65.2)	11.9 (10.9 to 12.9)
Kohgiluyeh and Boyer-Ahmad	5.3 (4.8 to 5.8)	2 (1.6 to 2.6)	67.3 (58.5 to 76.5)	9.4 (8.1 to 11)
Kurdistan	4.4 (3.8 to 4.9)	1.8 (1.4 to 2.3)	117.9 (103.6 to 132.6)	13 (11.9 to 14.1)
Lorestan	4.7 (4.2 to 5.1)	1.9 (1.5 to 2.4)	65.3 (56.7 to 74.1)	7.1 (6 to 8.2)
Markazi	4 (3.5 to 4.5)	1.6 (1.2 to 2)	68.5 (60 to 77.7)	10.8 (9.2 to 12.4)
Mazandaran	3.9 (3.5 to 4.2)	1.4 (1.1 to 1.8)	42.4 (35.8 to 49.1)	7.6 (6.9 to 8.2)
North Khorasan	5.3 (4.7 to 5.9)	2 (1.6 to 2.6)	96 (84.1 to 109.2)	12.2 (10.5 to 14.2)
Qazvin	4.1 (3.7 to 4.5)	1.7 (1.4 to 2.3)	74 (64.9 to 83.8)	9.5 (8.1 to 11)
Qom	4.2 (3.8 to 4.7)	1.9 (1.5 to 2.4)	53.6 (45.6 to 62)	8.4 (7.2 to 9.7)
Semnan	3.9 (3.5 to 4.3)	1.6 (1.2 to 2)	61.3 (54 to 69.2)	10.1 (8.4 to 12)
Sistan and Baluchistan	5.8 (5.2 to 6.5)	3.3 (2.6 to 4.3)	108.5 (95.2 to 122.3)	17.9 (16.4 to 19.4)
South Khorasan	5 (4.4 to 5.5)	2.2 (1.7 to 2.9)	101.9 (90 to 115.3)	13.3 (11.4 to 15.4)
Tehran	3.5 (3.1 to 3.9)	1.5 (1.2 to 1.9)	32.1 (26.1 to 38.7)	7.6 (6.9 to 8.2)
West Azarbayejan	4.2 (3.7 to 4.7)	2.2 (1.7 to 2.8)	85.7 (75.1 to 97.2)	12.3 (11.3 to 13.3)
Yazd	4.4 (3.9 to 5)	2 (1.6 to 2.6)	50.8 (43.9 to 58.5)	12 (10.3 to 13.9)
Zanjan	4.3 (3.9 to 4.7)	1.8 (1.4 to 2.3)	247.4 (231 to 265.2)	12 (10.3 to 13.9)

Data in parentheses are 95% uncertainty intervals

Appendix Table 5. Decomposition of life expectancy at birth (years) from 1990 to 2019 by location

Location	Communicable, maternal, neonatal, and nutritional diseases							Non-communicable diseases											Injuries		
	HIV/AIDS and sexually transmitted infections	Respiratory infections and tuberculosis	Enteric infections	Neglected tropical diseases and malaria	Other infectious diseases	Maternal and neonatal disorders	Nutritional deficiencies	Neoplasms	Cardiovascular diseases	Chronic respiratory diseases	Digestive diseases	Neurological disorders	Mental disorders	Substance use disorders	Diabetes and kidney diseases	Skin and subcutaneous diseases	Musculoskeletal disorders	Other non-communicable diseases	Transport injuries	Unintentional injuries	Self-harm and interpersonal violence
Iran (Islamic Republic of)	-0.0302	0.5922	0.2081	0.1302	0.3493	1.7943	0.0548	0.1827	2.8953	0.2414	0.1982	0.0219	-0.000009	-0.0124	-0.0430	0.0004	-0.0053	0.8500	0.9506	2.5661	0.1400
Alborz	-0.0150	0.6336	0.0946	0.1800	0.2315	1.3913	0.0150	0.1174	2.6061	0.1820	0.1936	-0.0080	-0.000003	-0.0517	-0.1079	-0.0011	-0.0037	0.6909	0.5904	0.3952	0.1314
Ardebil	-0.0073	0.5857	0.2425	0.1611	0.5489	2.1154	0.0585	0.1709	2.3121	0.1984	0.1967	0.0371	-0.000001	-0.0124	-0.0506	-0.0004	-0.0039	2.1386	1.0311	0.7794	0.1670
East Azarbayejan	-0.0037	0.6945	0.2041	0.1143	0.3900	2.1329	0.0791	0.1064	2.4836	0.2928	0.1433	0.0449	-0.000032	-0.0094	-0.0448	-0.0005	-0.0063	1.1322	1.0018	0.6230	0.1234
West Azarbayejan	-0.0050	0.5649	0.2833	0.0153	0.5148	2.2936	0.0756	0.3129	2.7555	0.3598	0.1598	0.0258	-0.000001	0.0107	-0.0554	0.0002	-0.0052	1.2369	0.9832	0.6739	0.1634
Bushehr	-0.0251	0.3729	0.2058	0.2588	0.2700	1.9540	0.1165	0.0481	2.7379	0.1366	0.1432	-0.0044	-0.000002	0.0086	-0.1794	0.0002	-0.0060	0.7548	0.9138	0.4848	0.1624
Chahar Mahaal and Bakhtiari	-0.0003	0.2818	0.0976	0.0858	0.3981	2.0290	0.0122	0.4033	3.1408	0.2537	0.1553	0.0471	-0.000002	-0.0215	0.0300	0.0008	0.0002	0.9244	0.9995	0.5746	0.2402
Fars	-0.0518	0.3048	0.1454	0.2228	0.2354	1.5806	0.0145	0.0327	3.0431	0.1060	0.1783	0.0049	-0.000023	-0.0181	-0.0886	-0.0002	-0.0073	0.6249	1.1066	0.4866	0.1564
Gilan	-0.0013	0.2028	0.0921	0.084	0.2234	1.2578	0.0091	0.0580	1.9921	0.1024	0.0837	-0.0010	-0.000001	-0.0172	-0.0998	-0.0003	-0.0039	0.6138	0.6775	26.3675	0.1198
Golestan	-0.0055	0.6311	0.2493	0.0748	0.3705	1.9319	0.0659	0.0536	2.2542	0.1112	0.1941	0.0088	-0.000001	0.0044	-0.1733	-0.0013	-0.006	1.0518	0.9322	0.5035	0.1168
Hamadan	-0.0064	0.6541	0.2418	0.0322	0.5113	2.3195	0.0396	0.1114	2.9515	0.2017	0.1715	0.0206	-0.000002	-0.0761	-0.0270	-0.0005	-0.0066	0.9654	1.3787	0.6131	0.2291
Hormozgan	-0.1011	0.9645	0.4869	0.4540	0.5033	2.0123	0.1150	0.1193	3.8371	0.1990	0.2226	0.0139	-0.000002	-0.0162	0.0695	-0.0009	-0.0088	0.8077	0.9476	0.6570	0.0954
Ilam	-0.0004	0.3669	0.2722	0.1148	0.5152	1.9999	0.0515	0.0535	2.7914	0.1082	0.1139	-0.0165	-0.000003	-0.0080	-0.1061	-0.0001	-0.0071	0.8747	0.7792	0.4992	0.2016
Isfahan	-0.0030	0.2974	0.1179	0.1968	0.2265	1.4607	0.0186	-0.0970	2.7911	0.1648	0.1321	-0.0086	-0.000002	-0.0177	-0.1938	-0.0013	-0.0103	0.7497	0.775	0.3485	0.0477
Kerman	-0.0386	0.6167	0.2996	0.172	0.3495	1.7952	0.0421	0.2112	3.6058	0.1634	0.2120	0.0124	-0.000002	0.0066	0.0748	0.0005	-0.0095	0.5024	1.3518	0.5152	0.2061
Kermanshah	-0.1342	0.6775	0.2261	0.1029	0.4021	2.6905	0.0900	0.3298	3.6287	0.3189	0.2411	0.0134	-0.000002	0.0154	0.0278	0.0004	-0.0088	0.9559	1.1343	0.6176	0.2022
North Khorasan	-0.0072	1.3803	0.3627	0.0333	0.5441	2.1168	0.0954	0.3013	3.1929	0.2811	0.3003	0.0367	-0.000151	0.0008	0.0966	-0.0030	-0.0047	1.0472	1.4866	0.7087	0.1043

Location	Communicable, maternal, neonatal, and nutritional diseases							Non-communicable diseases											Injuries		
	HIV/AIDS and sexually transmitted infections	Respiratory infections and tuberculosis	Enteric infections	Neglected tropical diseases and malaria	Other infectious diseases	Maternal and neonatal disorders	Nutritional deficiencies	Neoplasms	Cardiovascular diseases	Chronic respiratory diseases	Digestive diseases	Neurological disorders	Mental disorders	Substance use disorders	Diabetes and kidney diseases	Skin and subcutaneous diseases	Musculoskeletal disorders	Other non-communicable diseases	Transport injuries	Unintentional injuries	Self-harm and interpersonal violence
Khorasan-e-Razavi	-0.0069	1.0499	0.3458	0.1805	0.4062	2.4443	0.0863	0.2959	3.3345	0.2455	0.1768	0.0268	-0.000002	-0.0065	0.0134	0.0002	-0.0062	1.0409	1.7236	0.8392	0.1104
South Khorasan	-0.0024	1.5159	0.3484	0.0077	0.5159	2.4108	0.1710	0.2701	2.6366	0.4664	0.3566	0.0250	-0.000003	-0.0080	0.1248	-0.0023	-0.0029	0.9860	1.4272	0.7807	0.0654
Khuzestan	-0.0261	0.5915	0.1802	0.0737	0.3716	1.3016	0.0458	-0.0476	2.8156	0.2141	0.1948	0.0070	-0.000031	-0.0218	-0.2506	-0.0037	-0.0055	0.7772	0.5907	0.5001	0.1729
Kohgiluyeh and Boyer-Ahmad	-0.0195	0.5281	0.2356	0.1369	0.4977	1.5124	0.0218	0.2002	2.8469	0.2212	0.2338	0.0190	-0.000004	-0.0156	0.0332	-0.0018	-0.0069	1.0149	1.0233	0.7053	0.2072
Kurdistan	-0.0063	0.8270	0.2918	0.0425	0.5662	3.5958	0.0772	0.5989	3.5092	0.2941	0.2204	0.0526	-0.000001	-0.0016	0.0638	0.0003	-0.0078	1.4557	1.5529	0.8431	0.2004
Lorestan	-0.0765	0.6355	0.1722	0.0827	0.4971	1.8514	0.1852	0.2086	2.7441	0.1774	0.2399	0.0252	-0.000002	-0.0305	0.0630	0.0005	-0.0007	0.7558	1.0945	0.4397	0.3435
Markazi	-0.0030	0.6999	0.1668	0.1094	0.2972	2.1968	0.0291	0.4262	3.5336	0.3526	0.2155	0.0327	-0.000002	-0.0114	0.0481	0.0003	-0.0053	0.7958	1.5100	0.6082	0.2102
Mazandaran	-0.0007	0.2282	0.0925	0.0512	0.2319	1.0253	0.0229	-0.0067	2.7500	0.1501	0.1511	-0.0096	-0.000002	-0.0073	-0.2511	-0.0024	-0.0067	0.8687	0.6828	0.2846	0.1287
Qazvin	-0.0023	0.564	0.2031	0.1113	0.3788	2.0994	0.0336	-0.0726	2.4694	0.1774	0.3305	-0.0046	-0.000002	-0.0536	-0.0205	-0.0023	-0.0043	1.0103	1.3203	0.5763	0.1239
Qom	-0.0707	0.7787	0.1775	0.2714	0.3422	1.3285	0.0170	0.3743	3.1051	0.3972	0.3839	0.0231	-0.000003	-0.0071	0.1244	-0.0090	-0.0016	0.7821	0.9336	0.4762	0.2014
Semnan	0.0008	0.5909	0.1535	0.1526	0.2360	1.9198	0.0299	0.1743	2.7216	0.2605	0.1635	0.0134	-0.000002	-0.0072	-0.1264	-0.0001	-0.0038	0.7885	1.0783	0.4792	0.2031
Sistan and Baluchistan	-0.0593	1.3545	0.7029	0.8515	0.6410	2.1849	0.2010	-0.0299	2.1362	0.6668	0.2751	-0.0111	-0.000002	-0.0158	-0.1217	-0.0016	-0.0083	0.8238	0.9020	0.7025	0.0388
Tehran	-0.0691	0.3505	0.0729	0.0045	0.2436	0.9721	0.0126	0.3596	2.4662	0.2854	0.3194	0.0954	-0.000002	-0.0103	0.2105	0.0128	0.0033	0.5409	0.1938	0.3310	0.0919
Yazd	-0.0101	0.4600	0.1558	0.1103	0.2476	1.4373	0.0301	0.0370	2.7725	0.2653	0.1366	-0.0024	-0.000064	-0.0026	-0.2206	-0.0005	-0.0108	0.5705	0.9575	0.4146	0.1770
Zanjan	-0.0045	0.5039	0.1825	0.0454	0.2805	2.4434	0.0232	0.1089	1.4230	0.1294	0.0790	0.0036	-0.000037	-0.0126	-0.0112	-0.0001	-0.0034	0.7255	0.9317	27.2074	0.1149

Appendix Table 6. Decomposition analysis of DALYs change by cause, sex, and location, 1990–2019

Location	Cause	Sex	Expected DALYs in 2019		% 1990 - 2019 DALYs change cause			% 1990 - 2019 DALYs overall change
			Population growth	Population growth + Aging	Population growth	Age structure change	DALYs rate change	
**Iran (Islamic republic of)**	**All causes**	Both	36011208	36030559	44.0%	0.1%	-64.8%	-20.7%
		Female	15920655	15933836	44.9%	0.1%	-60.8%	-15.8%
		Male	20067557	20001698	43.1%	-0.5%	-67.2%	-24.6%
	**Communicable, maternal, neonatal, and nutritional diseases**	Both	11157223	6724415	44.0%	-57.2%	-63.9%	-77.1%
		Female	5143089	3104235	44.9%	-57.5%	-64.7%	-77.2%
		Male	6009699	3619046	43.1%	-56.9%	-63.1%	-77.0%
	HIV/AIDS and sexually transmitted infections	Both	26693	25271	44.0%	-7.7%	299.1%	335.5%
		Female	13035	13037	44.9%	0%	229.5%	274.4%
		Male	13657	12242	43.1%	-14.8%	364.7%	393.0%
	Respiratory infections and tuberculosis	Both	1891367	1304780	44.0%	-44.7%	-70.6%	-71.2%
		Female	918354	609427	44.9%	-48.8%	-70.5%	-74.3%
		Male	972855	694222	43.1%	-41.0%	-70.5%	-68.4%
	Enteric infections	Both	734909	469574	44.0%	-52.0%	-51.2%	-59.2%
		Female	347352	220696	44.9%	-52.9%	-50.7%	-58.6%
		Male	387375	248379	43.1%	-51.3%	-51.5%	-59.7%
	Neglected tropical diseases and malaria	Both	242976	224342	44.0%	-11.0%	-124.4%	-91.4%
		Female	111698	102207	44.9%	-12.3%	-123.3%	-90.7%
		Male	131178	121772	43.1%	-10.3%	-124.8%	-92.0%
	Other infectious diseases	Both	1109708	678584	44.0%	-55.9%	-74.8%	-86.7%
		Female	581072	344628	44.9%	-59.0%	-74.7%	-88.7%
		Male	529084	332855	43.1%	-53.1%	-74.7%	-84.6%
	Maternal and neonatal disorders	Both	6713936	3691647	44.0%	-64.8%	-60.5%	-81.4%
		Female	2940422	1634580	44.9%	-64.4%	-61.2%	-80.6%
		Male	3768870	2059251	43.1%	-64.9%	-60.1%	-81.9%
	Nutritional deficiencies	Both	437634	330218	44.0%	-35.3%	-68.0%	-59.4%
		Female	231156	179660	44.9%	-32.3%	-71.1%	-58.4%
		Male	206679	150325	43.1%	-39.0%	-64.5%	-60.4%
	**Non-communicable diseases**	Both	15470986	20503990	44.0%	46.8%	-46.6%	44.2%
		Female	7429263	9932222	44.9%	48.8%	-42.4%	51.4%
		Male	8039378	10499053	43.1%	43.8%	-49.2%	37.7%
	Neoplasms	Both	1339769	2047571	44.0%	76.1%	-26.4%	93.7%
		Female	583736	894643	44.9%	77.2%	-23.6%	98.6%
		Male	755068	1140810	43.1%	73.1%	-26.3%	89.9%
	Cardiovascular diseases	Both	3484557	6591369	44.0%	128.4%	-123.5%	48.9%
		Female	1413279	2773716	44.9%	139.5%	-128.7%	55.8%
		Male	2067426	3757217	43.1%	117%	-115.8%	44.3%
	Chronic respiratory diseases	Both	526218	826396	44.0%	82.1%	-65.3%	60.9%
		Female	231028	363959	44.9%	83.4%	-69.6%	58.7%
		Male	294833	459004	43.1%	79.7%	-60.2%	62.6%
	Digestive diseases	Both	481889	672580	44.0%	57.0%	-69.9%	31.1%
		Female	216720	298367	44.9%	54.6%	-68.9%	30.7%
		Male	264909	369803	43.1%	56.7%	-68.3%	31.5%

Location	Cause	Sex	Expected DALYs in 2019		% 1990 - 2019 DALYs change cause			% 1990 - 2019 DALYs overall change
			Population growth	Population growth + Aging	Population growth	Age structure change	DALYs rate change	
	Neurological disorders	Both	880763	1160003	44.0%	45.7%	-9.2%	80.4%
		Female	477618	632590	44.9%	47.0%	-5.3%	86.7%
		Male	403710	529256	43.1%	44.5%	-14.5%	73.1%
	Mental disorders	Both	1669553	2012804	44.0%	29.6%	3.5%	77.1%
		Female	929349	1142190	44.9%	33.2%	3.1%	81.2%
		Male	741582	872625	43.1%	25.3%	3.8%	72.1%
	Substance use disorders	Both	236237	318481	44.0%	50.1%	38.7%	132.8%
		Female	74898	98144	44.9%	45.0%	-4.0%	86.0%
		Male	160810	220308	43.1%	52.9%	58.3%	154.3%
	Diabetes and kidney diseases	Both	548811	892712	44.0%	90.2%	60.9%	195.1%
		Female	266662	443683	44.9%	96.2%	68.0%	209.1%
		Male	282105	447695	43.1%	84.0%	54.9%	182.0%
	Skin and subcutaneous diseases	Both	359700	339357	44.0%	-8.1%	1.7%	37.6%
		Female	188493	177438	44.9%	-8.5%	1.1%	37.5%
		Male	171354	161907	43.1%	-7.9%	2.4%	37.6%
	Sense organ diseases	Both	460768	765980	44.0%	95.4%	-52.0%	87.4%
		Female	224265	380999	44.9%	101.3%	-53.4%	92.8%
		Male	236471	384729	43.1%	89.7%	-50.4%	82.4%
	Musculoskeletal disorders	Both	1046500	1710663	44.0%	91.4%	8.4%	143.7%
		Female	557915	926519	44.9%	95.8%	12.7%	153.4%
		Male	489134	787187	43.1%	87.2%	2.6%	132.9%
	Other non-communicable diseases	Both	4436221	3166074	44.0%	-41.2%	-48.1%	-45.4%
		Female	2265300	1799975	44.9%	-29.8%	-44.9%	-29.7%
		Male	2171974	1368511	43.1%	-52.9%	-51.6%	-61.5%
	**Injuries***	Both	5620426	5445309	43.0%	-4.5%	-73.5%	-34.9%
		Female	1848199	1650000	43.6%	-15.4%	-75.2%	-47.0%
		Male	3764897	3786488	42.5%	0.8%	-72.3%	-29.0%
	Transport Injuries	Both	2947148	2802476	44.0%	-7.1%	-79.1%	-42.2%
		Female	926932	784154	44.9%	-22.3%	-79.8%	-57.2%
		Male	2013528	2009341	43.1%	-0.3%	-78.2%	-35.4%
	Unintentional Injuries*	Both	1963659	1812196	43.0%	-11.0%	-69.2%	-37.2%
		Female	702869	607679	43.6%	-19.4%	-69.1%	-45.0%
		Male	1258685	1201639	42.5%	-6.5%	-68.9%	-32.9%
	Self-harm and interpersonal violence	Both	825157	916679	44.0%	16.0%	-70.5%	-10.6%
		Female	256915	278735	44.9%	12.3%	-78.7%	-21.4%
		Male	566336	636608	43.1%	17.8%	-66.6%	-5.7%
**Alborz**	**All causes**	Both	964753	1019090	95.4%	11%	-72.2%	34.2%
		Female	426352	461316	99%	16.3%	-73.6%	41.7%
		Male	536745	554959	92%	6.5%	-70.2%	28.4%
	**Communicable, maternal, neonatal, and nutritional diseases**	Both	336921	201812	95.4%	-78.4%	-85.1%	-68.1%
		Female	147003	87815	99%	-80.1%	-86.6%	-67.7%
		Male	189273	113972	92%	-76.4%	-84%	-68.4%

Location	Cause	Sex	Expected DALYs in 2019		% 1990 - 2019 DALYs change cause			% 1990 - 2019 DALYs overall change
			Population growth	Population growth + Aging	Population growth	Age structure change	DALYs rate change	
	HIV/AIDS and sexually transmitted infections	Both	773	769	95.4%	-1%	250.4%	344.9%
		Female	381	408	99%	14.1%	106.7%	219.8%
		Male	392	364	92%	-13.6%	383.8%	462.2%
	Respiratory infections and tuberculosis	Both	74053	51189	95.4%	-60.3%	-94.3%	-59.2%
		Female	30755	20599	99%	-65.7%	-95.5%	-62.2%
		Male	43102	30451	92%	-56.4%	-92.8%	-57.1%
	Enteric infections	Both	14843	9634	95.4%	-68.6%	-37.5%	-10.6%
		Female	7175	4603	99%	-71.3%	-37.6%	-9.9%
		Male	7664	5019	92%	-66.3%	-37%	-11.3%
	Neglected tropical diseases and malaria	Both	8511	9730	95.4%	28%	-213.6%	-90.2%
		Female	3839	4439	99%	31.1%	-219.4%	-89.3%
		Male	4659	5241	92%	24%	-206.9%	-90.9%
	Other infectious diseases	Both	27770	16493	95.4%	-79.4%	-95%	-79%
		Female	14191	8134	99%	-85%	-96.2%	-82.2%
		Male	13599	8337	92%	-74.3%	-93.5%	-75.7%
	Maternal and neonatal disorders	Both	200875	106017	95.4%	-92.3%	-79.8%	-76.6%
		Female	85542	45359	99%	-93.5%	-80.5%	-74.9%
		Male	114875	60841	92%	-90.3%	-79.6%	-77.8%
	Nutritional deficiencies	Both	10095	7979	95.4%	-41%	-84.9%	-30.4%
		Female	5120	4273	99%	-32.9%	-92.1%	-26%
		Male	4982	3718	92%	-48.7%	-78.1%	-34.8%
	**Non-communicable diseases**	Both	477355	669658	95.4%	78.7%	-57.3%	116.8%
		Female	229097	327492	99%	85.5%	-59.1%	125.4%
		Male	248075	341113	92%	72%	-54.8%	109.2%
	Neoplasms	Both	37166	65027	95.4%	146.5%	-31.3%	210.5%
		Female	17296	31014	99%	157.9%	-57%	199.9%
		Male	19837	34044	92%	137.5%	-10.1%	219.5%
	Cardiovascular diseases	Both	99347	208191	95.4%	214.1%	-172.2%	137.3%
		Female	40969	87167	99%	224.4%	-195.9%	127.6%
		Male	58105	118558	92%	199.8%	-147.9%	144%
	Chronic respiratory diseases	Both	14711	24596	95.4%	131.3%	-97.4%	129.3%
		Female	6497	10828	99%	132.7%	-115.1%	116.7%
		Male	8188	13674	92%	128.7%	-81.7%	139%
	Digestive diseases	Both	15103	21805	95.4%	86.7%	-99.1%	83%
		Female	6779	9831	99%	89.6%	-106.7%	81.9%
		Male	8302	11801	92%	80.9%	-89.1%	83.9%
	Neurological disorders	Both	28116	38250	95.4%	70.4%	-5.1%	160.8%
		Female	15327	21074	99%	74.6%	-2.5%	171.1%
		Male	12843	17425	92%	68.5%	-11.7%	148.8%
	Mental disorders	Both	58891	72977	95.4%	46.7%	8.5%	150.6%
		Female	32345	41236	99%	54.7%	6.8%	160.6%
		Male	26666	32155	92%	39.5%	7.5%	139%

Location	Cause	Sex	Expected DALYs in 2019		% 1990 - 2019 DALYs change cause			% 1990 - 2019 DALYs overall change
			Population growth	Population growth + Aging	Population growth	Age structure change	DALYs rate change	
	Substance use disorders	Both	9428	12620	95.4%	66.2%	81.3%	242.9%
		Female	2924	3907	99%	66.9%	-41.4%	124.5%
		Male	6444	8552	92%	62.8%	139.9%	294.8%
	Diabetes and kidney diseases	Both	14585	27152	95.4%	168.4%	141.5%	405.3%
		Female	7446	14164	99%	179.5%	131.8%	410.4%
		Male	7149	13010	92%	157.4%	150.7%	400.2%
	Skin and subcutaneous diseases	Both	12636	11793	95.4%	-13%	3.1%	85.5%
		Female	6627	6158	99%	-14.1%	2.3%	87.2%
		Male	6024	5652	92%	-11.9%	3.5%	83.7%
	Sense organ diseases	Both	14044	25295	95.4%	156.6%	-76.1%	175.8%
		Female	6808	12299	99%	160.5%	-77.9%	181.6%
		Male	7233	12986	92%	152.8%	-74.2%	170.6%
	Musculoskeletal disorders	Both	34884	62030	95.4%	152.1%	10.2%	257.7%
		Female	18059	32916	99%	163.7%	18.7%	281.4%
		Male	16858	29317	92%	141.9%	-0.9%	233.1%
	Other non-communicable diseases	Both	138445	99921	95.4%	-54.4%	-62.6%	-21.6%
		Female	68021	56900	99%	-32.5%	-57.8%	8.7%
		Male	70427	43939	92%	-72.2%	-69.6%	-49.8%
	**Injuries***	Both	149464	148035	90.1%	-1.8%	-89.5%	-1.3%
		Female	49696	46143	93.2%	-13.8%	-100.4%	-21%
		Male	99020	100192	87.1%	2.2%	-81%	8.3%
	Transport Injuries	Both	66975	63515	95.4%	-10.1%	-103.4%	-18%
		Female	22868	20706	99%	-18.8%	-114.6%	-34.4%
		Male	43756	42153	92%	-7%	-94.8%	-9.8%
	Unintentional Injuries*	Both	57298	55022	90.1%	-7.5%	-85.4%	-2.9%
		Female	19740	17609	93.2%	-20.9%	-92.3%	-19.9%
		Male	37293	36814	87.1%	-2.4%	-78.9%	5.8%
	Self-harm and interpersonal violence	Both	25206	29010	95.4%	29.5%	-67.6%	57.3%
		Female	7245	7706	99%	12.6%	-85.2%	26.4%
		Male	17781	20837	92%	33%	-55.6%	69.5%
**Ardebil**	**All causes**	Both	621198	575643	10.9%	-8.1%	-44.8%	-42.1%
		Female	279051	261541	10.9%	-7%	-45.7%	-41.7%
		Male	342127	312102	10.8%	-9.7%	-43.5%	-42.4%
	**Communicable, maternal, neonatal, and nutritional diseases**	Both	200280	113085	10.9%	-48.3%	-49%	-86.4%
		Female	93548	53233	10.9%	-47.8%	-49.7%	-86.6%
		Male	106729	59766	10.8%	-48.8%	-48.3%	-86.2%
	HIV/AIDS and sexually transmitted infections	Both	422	358	10.9%	-16.7%	48.6%	42.8%
		Female	214	205	10.9%	-5%	80.2%	86.1%
		Male	207	151	10.8%	-30.1%	17.3%	-2%
	Respiratory infections and tuberculosis	Both	28865	17654	10.9%	-43.1%	-53.4%	-85.6%
		Female	13869	8338	10.9%	-44.2%	-53.8%	-87.1%
		Male	14995	9298	10.8%	-42.1%	-52.9%	-84.2%

Location	Cause	Sex	Expected DALYs in 2019		% 1990 - 2019 DALYs change cause			% 1990 - 2019 DALYs overall change
			Population growth	Population growth + Aging	Population growth	Age structure change	DALYs rate change	
	Enteric infections	Both	12550	7777	10.9%	-42.2%	-41.1%	-72.4%
		Female	5773	3625	10.9%	-41.3%	-40.8%	-71.2%
		Male	6777	4152	10.8%	-42.9%	-41.2%	-73.4%
	Neglected tropical diseases and malaria	Both	5150	4497	10.9%	-14.1%	-91.1%	-94.3%
		Female	2387	2050	10.9%	-15.7%	-89.5%	-94.3%
		Male	2762	2437	10.8%	-13.1%	-92.1%	-94.3%
	Other infectious diseases	Both	25820	14588	10.9%	-48.2%	-56.3%	-93.6%
		Female	13943	7737	10.9%	-49.4%	-56.3%	-94.7%
		Male	11878	6827	10.8%	-47.1%	-56%	-92.3%
	Maternal and neonatal disorders	Both	120178	62917	10.9%	-52.8%	-45.6%	-87.5%
		Female	53683	28458	10.9%	-52.1%	-46.3%	-87.5%
		Male	66491	34417	10.8%	-53.5%	-44.9%	-87.5%
	Nutritional deficiencies	Both	7297	5294	10.9%	-30.4%	-52.4%	-71.9%
		Female	3678	2820	10.9%	-25.9%	-55.2%	-70.1%
		Male	3619	2485	10.8%	-34.7%	-49.9%	-73.8%
	**Non-communicable diseases**	Both	316206	371183	10.9%	19.3%	-37.5%	-7.3%
		Female	147646	178843	10.9%	23.4%	-39.5%	-5.2%
		Male	168554	189913	10.8%	14%	-34.1%	-9.2%
	Neoplasms	Both	27516	42658	10.9%	61%	-19.8%	52.1%
		Female	12009	18926	10.9%	63.9%	-37.5%	37.3%
		Male	15506	22962	10.8%	53.3%	-0.7%	63.5%
	Cardiovascular diseases	Both	60920	113684	10.9%	96%	-76.7%	30.2%
		Female	24172	48589	10.9%	112%	-94.4%	28.6%
		Male	36743	63961	10.8%	82.1%	-61.6%	31.3%
	Chronic respiratory diseases	Both	7784	11957	10.9%	59.5%	-50.7%	19.6%
		Female	3499	5602	10.9%	66.7%	-65.1%	12.5%
		Male	4284	6244	10.8%	50.7%	-36%	25.5%
	Digestive diseases	Both	8014	9968	10.9%	27%	-57.1%	-19.2%
		Female	3811	4714	10.9%	26.3%	-62.3%	-25.1%
		Male	4203	5204	10.8%	26.4%	-50.9%	-13.7%
	Neurological disorders	Both	14826	19076	10.9%	31.8%	-12.2%	30.5%
		Female	7750	10356	10.9%	37.3%	-9.8%	38.4%
		Male	7077	8665	10.8%	24.9%	-13.9%	21.8%
	Mental disorders	Both	25568	31889	10.9%	27.4%	2.7%	41%
		Female	13908	17644	10.9%	29.8%	2.9%	43.7%
		Male	11660	14164	10.8%	23.8%	3.2%	37.9%
	Substance use disorders	Both	2352	3528	10.9%	55.4%	52.5%	118.8%
		Female	824	1182	10.9%	48.2%	2.9%	62%
		Male	1528	2384	10.8%	62.1%	76.4%	149.4%
	Diabetes and kidney diseases	Both	9359	14152	10.9%	56.8%	33.9%	101.6%
		Female	4627	7287	10.9%	63.8%	28%	102.6%
		Male	4732	6851	10.8%	49.6%	40.1%	100.6%

Location	Cause	Sex	Expected DALYs in 2019		% 1990 - 2019 DALYs change cause			% 1990 - 2019 DALYs overall change
			Population growth	Population growth + Aging	Population growth	Age structure change	DALYs rate change	
	Skin and subcutaneous diseases	Both	5600	5208	10.9%	-7.8%	1.1%	4.3%
		Female	2902	2697	10.9%	-7.8%	0.9%	4%
		Male	2698	2507	10.8%	-7.9%	1.6%	4.5%
	Sense organ diseases	Both	6983	12319	10.9%	84.7%	-45.7%	49.9%
		Female	3280	6230	10.9%	99.7%	-49.2%	61.5%
		Male	3703	6091	10.8%	71.5%	-42.7%	39.6%
	Musculoskeletal disorders	Both	14694	25852	10.9%	84.2%	7.1%	102.2%
		Female	7663	13810	10.9%	89%	11.5%	111.4%
		Male	7031	12098	10.8%	79.9%	1.5%	92.2%
	Other non-communicable diseases	Both	132590	80893	10.9%	-43.2%	-44.6%	-76.9%
		Female	63200	41808	10.9%	-37.5%	-42.2%	-68.8%
		Male	69388	38783	10.8%	-48.9%	-46.2%	-84.3%
	**Injuries***	Both	100742	89197	11%	-12.7%	-58.9%	-60.7%
		Female	36450	29002	10.5%	-22.6%	-60.1%	-72.1%
		Male	64389	60811	11.4%	-6.2%	-59.3%	-54.1%
	Transport Injuries	Both	49192	42531	10.9%	-15%	-62.2%	-66.3%
		Female	17253	12792	10.9%	-28.7%	-61.9%	-79.6%
		Male	31933	30021	10.8%	-6.6%	-63.3%	-59.1%
	Unintentional Injuries*	Both	39319	32057	11%	-20.5%	-56.8%	-66.3%
		Female	14835	11060	10.5%	-28.1%	-56.9%	-74.5%
		Male	24517	21149	11.4%	-15.3%	-57.4%	-61.3%
	Self-harm and interpersonal violence	Both	14607	16196	10.9%	12.1%	-56%	-33.1%
		Female	5079	5438	10.9%	7.8%	-62.2%	-43.4%
		Male	9526	10866	10.8%	15.6%	-54%	-27.6%
**Bushehr**	**All causes**	Both	474509	452740	71.4%	-7.9%	-68.8%	-5.2%
		Female	209733	201769	64.7%	-6.3%	-63.8%	-5.4%
		Male	265939	251128	78%	-9.9%	-73.1%	-5%
	**Communicable, maternal, neonatal, and nutritional diseases**	Both	186150	110650	71.4%	-69.5%	-77.6%	-75.7%
		Female	83856	51901	64.7%	-62.8%	-78.6%	-76.7%
		Male	102624	58568	78%	-76.4%	-76.4%	-74.8%
	HIV/AIDS and sexually transmitted infections	Both	411	377	71.4%	-14%	264.7%	322.1%
		Female	188	179	64.7%	-7.8%	184.6%	241.5%
		Male	223	197	78%	-20.8%	338.7%	395.9%
	Respiratory infections and tuberculosis	Both	20679	14172	71.4%	-53.9%	-79.7%	-62.2%
		Female	10029	6774	64.7%	-53.5%	-79.7%	-68.4%
		Male	10630	7410	78%	-53.9%	-79.9%	-55.8%
	Enteric infections	Both	11735	7302	71.4%	-64.8%	-62.7%	-56%
		Female	5416	3451	64.7%	-59.8%	-62.5%	-57.5%
		Male	6330	3852	78%	-69.6%	-62.9%	-54.6%
	Neglected tropical diseases and malaria	Both	6387	6064	71.4%	-8.7%	-157.3%	-94.5%
		Female	3032	2660	64.7%	-20.2%	-139.3%	-94.7%
		Male	3355	3410	78%	3%	-175.2%	-94.3%

Location	Cause	Sex	Expected DALYs in 2019		% 1990 - 2019 DALYs change cause			% 1990 - 2019 DALYs overall change
			Population growth	Population growth + Aging	Population growth	Age structure change	DALYs rate change	
	Other infectious diseases	Both	13388	8337	71.4%	-64.7%	-90.8%	-84.1%
		Female	7078	4464	64.7%	-60.8%	-90.7%	-86.8%
		Male	6250	3865	78%	-67.9%	-90.8%	-80.8%
	Maternal and neonatal disorders	Both	123764	67550	71.4%	-77.9%	-73.6%	-80%
		Female	52961	30642	64.7%	-69.4%	-75%	-79.7%
		Male	71247	36735	78%	-86.2%	-72%	-80.3%
	Nutritional deficiencies	Both	9786	6848	71.4%	-51.5%	-85.7%	-65.7%
		Female	5152	3730	64.7%	-45.5%	-87.3%	-68.1%
		Male	4591	3098	78%	-57.9%	-82.9%	-62.8%
	**Non-communicable diseases**	Both	211650	263851	71.4%	42.3%	-50.5%	63.2%
		Female	100333	126529	64.7%	43%	-44.8%	62.9%
		Male	111290	136045	78%	39.6%	-54%	63.5%
	Neoplasms	Both	15428	23065	71.4%	84.9%	-22%	134.3%
		Female	7070	10643	64.7%	83.2%	-20.4%	127.5%
		Male	8375	12246	78%	82.2%	-19.7%	140.5%
	Cardiovascular diseases	Both	46258	83318	71.4%	137.4%	-132.3%	76.5%
		Female	17911	33991	64.7%	147.9%	-138.1%	74.5%
		Male	28664	49238	78%	127.7%	-127.9%	77.8%
	Chronic respiratory diseases	Both	5440	7920	71.4%	78.2%	-59.1%	90.5%
		Female	2485	3714	64.7%	81.5%	-68.5%	77.7%
		Male	2962	4177	78%	73%	-48.9%	102.1%
	Digestive diseases	Both	5742	8007	71.4%	67.6%	-70.7%	68.3%
		Female	2574	3558	64.7%	62.9%	-70.3%	57.4%
		Male	3179	4450	78%	71.1%	-71.2%	77.9%
	Neurological disorders	Both	11713	14823	71.4%	45.5%	-9.6%	107.3%
		Female	6201	7922	64.7%	45.7%	-3.3%	107.1%
		Male	5459	6786	78%	43.2%	-13.6%	107.6%
	Mental disorders	Both	24534	29719	71.4%	36.2%	3%	110.7%
		Female	13001	15762	64.7%	35%	4.2%	103.9%
		Male	11419	13709	78%	35.7%	5.4%	119.1%
	Substance use disorders	Both	2617	3662	71.4%	68.5%	15.5%	155.4%
		Female	856	1120	64.7%	50.6%	-25.4%	89.9%
		Male	1791	2627	78%	83%	28.3%	189.2%
	Diabetes and kidney diseases	Both	6103	9330	71.4%	90.7%	117.2%	279.4%
		Female	2862	4508	64.7%	94.7%	130.8%	290.2%
		Male	3242	4800	78%	85.5%	105.6%	269%
	Skin and subcutaneous diseases	Both	5316	5053	71.4%	-8.5%	2.9%	65.9%
		Female	2674	2547	64.7%	-7.9%	2.2%	59%
		Male	2628	2495	78%	-9%	4.5%	73.5%
	Sense organ diseases	Both	6173	9495	71.4%	92.2%	-61.7%	101.9%
		Female	2898	4642	64.7%	99.1%	-62.3%	101.5%
		Male	3276	4853	78%	85.6%	-61.2%	102.4%

Location	Cause	Sex	Expected DALYs in 2019		% 1990 - 2019 DALYs change cause			% 1990 - 2019 DALYs overall change
			Population growth	Population growth + Aging	Population growth	Age structure change	DALYs rate change	
	Musculoskeletal disorders	Both	13658	21888	71.4%	103.3%	10.6%	185.3%
		Female	6980	11226	64.7%	100.2%	17%	181.9%
		Male	6636	10601	78%	106.3%	5%	189.3%
	Other non-communicable diseases	Both	68668	47570	71.4%	-52.7%	-58.2%	-39.4%
		Female	34819	26898	64.7%	-37.5%	-53.4%	-26.1%
		Male	33659	20063	78%	-71.9%	-60.4%	-54.4%
	**Injuries***	Both	78096	79958	70.4%	4.1%	-99.2%	-24.7%
		Female	25987	24060	63.4%	-12.1%	-92.4%	-41.1%
		Male	53029	57606	77.2%	15.3%	-108.5%	-16%
	Transport Injuries	Both	40613	42053	71.4%	6.1%	-101.7%	-24.2%
		Female	11859	10706	64.7%	-16%	-93.3%	-44.6%
		Male	29343	32399	78%	18.5%	-111.8%	-15.3%
	Unintentional Injuries*	Both	26804	25273	70.4%	-9.7%	-99.1%	-38.4%
		Female	10019	8645	63.4%	-22.4%	-90.9%	-50%
		Male	17008	17045	77.2%	0.4%	-108.6%	-31.1%
	Self-harm and interpersonal violence	Both	11165	13006	71.4%	28.3%	-94.4%	5.3%
		Female	4288	4745	64.7%	17.5%	-93.6%	-11.3%
		Male	6956	8458	78%	38.4%	-100%	16.4%
**Chahar Mahaal and Bakhtiari**	**All causes**	Both	362475	366567	35.3%	1.5%	-60.1%	-23.3%
		Female	161562	163294	36%	1.5%	-59.3%	-21.8%
		Male	200770	201895	34.8%	0.8%	-59.9%	-24.4%
	**Communicable, maternal, neonatal, and nutritional diseases**	Both	126900	74830	35.3%	-55.5%	-63.2%	-83.4%
		Female	59427	35151	36%	-55.5%	-63.8%	-83.4%
		Male	67448	39654	34.8%	-55.5%	-62.6%	-83.4%
	HIV/AIDS and sexually transmitted infections	Both	294	260	35.3%	-15.6%	1%	20.7%
		Female	150	148	36%	-1.8%	-8.1%	26.1%
		Male	144	111	34.8%	-30.7%	11%	15%
	Respiratory infections and tuberculosis	Both	11477	7870	35.3%	-42.5%	-64.4%	-71.6%
		Female	5519	3668	36%	-45.6%	-65.1%	-74.7%
		Male	5957	4195	34.8%	-39.9%	-63.6%	-68.7%
	Enteric infections	Both	4486	3258	35.3%	-37.1%	-22.9%	-24.6%
		Female	2248	1649	36%	-36.2%	-26.4%	-26.7%
		Male	2239	1604	34.8%	-38.2%	-19.1%	-22.6%
	Neglected tropical diseases and malaria	Both	1705	1757	35.3%	4.1%	-127.7%	-88.3%
		Female	850	818	36%	-5.1%	-119.5%	-88.6%
		Male	855	931	34.8%	12%	-134.6%	-87.9%
	Other infectious diseases	Both	14839	8840	35.3%	-54.7%	-72.8%	-92.2%
		Female	7899	4601	36%	-56.8%	-72.5%	-93.3%
		Male	6945	4226	34.8%	-52.8%	-72.9%	-90.9%
	Maternal and neonatal disorders	Both	90178	49701	35.3%	-60.7%	-62.6%	-88%
		Female	40733	22569	36%	-60.6%	-63.1%	-87.7%
		Male	49414	27141	34.8%	-60.7%	-62.2%	-88.2%

Location	Cause	Sex	Expected DALYs in 2019		% 1990 - 2019 DALYs change cause			% 1990 - 2019 DALYs overall change
			Population growth	Population growth + Aging	Population growth	Age structure change	DALYs rate change	
	Nutritional deficiencies	Both	3920	3145	35.3%	-26.8%	-60.3%	-51.7%
		Female	2028	1699	36%	-22.1%	-64.9%	-51%
		Male	1893	1446	34.8%	-31.8%	-55.4%	-52.5%
	**Non-communicable diseases**	Both	169677	225328	35.3%	44.4%	-49.8%	29.9%
		Female	80552	108711	36%	47.5%	-51.1%	32.4%
		Male	89102	115368	34.8%	39.7%	-46.8%	27.7%
	Neoplasms	Both	13877	22115	35.3%	80.3%	-50.4%	65.3%
		Female	5690	9229	36%	84.5%	-60%	60.5%
		Male	8176	12603	34.8%	72.9%	-39.1%	68.6%
	Cardiovascular diseases	Both	38567	75875	35.3%	130.9%	-115.2%	51%
		Female	15065	31793	36%	151%	-143.2%	43.8%
		Male	23468	43239	34.8%	113.5%	-92.6%	55.7%
	Chronic respiratory diseases	Both	4815	7586	35.3%	77.9%	-68.5%	44.7%
		Female	2092	3374	36%	83.4%	-76.1%	43.2%
		Male	2721	4157	34.8%	71.1%	-60.1%	45.8%
	Digestive diseases	Both	3459	5354	35.3%	74.1%	-71.6%	37.8%
		Female	1550	2423	36%	76.6%	-76%	36.6%
		Male	1908	2902	34.8%	70.2%	-66.1%	38.9%
	Neurological disorders	Both	10084	13449	35.3%	45.2%	-13.5%	67%
		Female	5427	7373	36%	48.8%	-9.3%	75.4%
		Male	4662	6078	34.8%	40.9%	-18.3%	57.4%
	Mental disorders	Both	17837	21843	35.3%	30.4%	2%	67.7%
		Female	9682	12125	36%	34.3%	1.6%	71.9%
		Male	8163	9724	34.8%	25.8%	2.3%	62.8%
	Substance use disorders	Both	1595	2315	35.3%	61.1%	81.1%	177.6%
		Female	531	744	36%	54.6%	15.8%	106.3%
		Male	1062	1576	34.8%	65.3%	112.8%	212.9%
	Diabetes and kidney diseases	Both	3796	6237	35.3%	87%	48.9%	171.3%
		Female	1766	2980	36%	93.5%	43.5%	173%
		Male	2029	3227	34.8%	79.5%	55.5%	169.8%
	Skin and subcutaneous diseases	Both	4314	4047	35.3%	-8.4%	1.5%	28.4%
		Female	2254	2111	36%	-8.6%	1.3%	28.6%
		Male	2062	1936	34.8%	-8.2%	1.7%	28.3%
	Sense organ diseases	Both	5360	9047	35.3%	93.1%	-48.8%	79.7%
		Female	2564	4561	36%	105.9%	-51.8%	90%
		Male	2795	4482	34.8%	81.3%	-45.9%	70.2%
	Musculoskeletal disorders	Both	10770	18468	35.3%	96.8%	8.3%	140.4%
		Female	5628	9884	36%	102.8%	12.4%	151.2%
		Male	5145	8626	34.8%	91.2%	2.7%	128.6%
	Other non-communicable diseases	Both	55204	38992	35.3%	-39.7%	-50.4%	-54.8%
		Female	28304	22112	36%	-29.7%	-47.1%	-40.9%
		Male	26911	16819	34.8%	-50.5%	-53.4%	-69.2%

Location	Cause	Sex	Expected DALYs in 2019		% 1990 - 2019 DALYs change cause			% 1990 - 2019 DALYs overall change
			Population growth	Population growth + Aging	Population growth	Age structure change	DALYs rate change	
	**Injuries***	Both	63535	64787	34.4%	2.6%	-79.8%	-42.7%
		Female	20772	19037	34.6%	-11.2%	-76.6%	-53.3%
		Male	42747	45752	34.3%	9.4%	-81.3%	-37.5%
	Transport Injuries	Both	32809	33386	35.3%	2.4%	-81%	-43.3%
		Female	10048	8720	36%	-18%	-75.4%	-57.4%
		Male	22707	24596	34.8%	11.2%	-83.1%	-37.2%
	Unintentional Injuries*	Both	21701	20418	34.4%	-8%	-75.8%	-49.3%
		Female	7446	6344	34.6%	-19.9%	-73.7%	-59.1%
		Male	14249	14073	34.3%	-1.7%	-76.8%	-44.1%
	Self-harm and interpersonal violence	Both	10639	12292	35.3%	21%	-90.8%	-34.4%
		Female	3741	4252	36%	18.6%	-89.2%	-34.7%
		Male	6885	8033	34.8%	22.5%	-91.5%	-34.2%
**East Azarbay ejan**	**All causes**	Both	1725395	1745002	18.2%	1.3%	-48.3%	-28.8%
		Female	766063	783225	18.1%	2.6%	-46.9%	-26.1%
		Male	959381	956808	18.2%	-0.3%	-48.8%	-30.9%
	**Communicable, maternal, neonatal, and nutritional diseases**	Both	578249	341021	18.2%	-48.5%	-54.8%	-85.1%
		Female	268981	159697	18.1%	-48%	-55.2%	-85%
		Male	309277	181163	18.2%	-49%	-54.4%	-85.2%
	HIV/AIDS and sexually transmitted infections	Both	1174	1056	18.2%	-11.8%	35.1%	41.5%
		Female	597	592	18.1%	-1%	39.3%	56.5%
		Male	577	462	18.2%	-23.4%	31.1%	25.9%
	Respiratory infections and tuberculosis	Both	106874	70299	18.2%	-40.4%	-57.4%	-79.7%
		Female	53117	34030	18.1%	-42.4%	-57.5%	-81.8%
		Male	53757	36174	18.2%	-38.7%	-57.2%	-77.7%
	Enteric infections	Both	34172	22010	18.2%	-42.1%	-41.4%	-65.3%
		Female	16426	10614	18.1%	-41.8%	-41.7%	-65.3%
		Male	17746	11379	18.2%	-42.4%	-41%	-65.2%
	Neglected tropical diseases and malaria	Both	11825	9978	18.2%	-18.5%	-92.6%	-92.9%
		Female	5290	4378	18.1%	-20.4%	-89.9%	-92.1%
		Male	6535	5578	18.2%	-17.3%	-94.3%	-93.4%
	Other infectious diseases	Both	56961	34342	18.2%	-46.9%	-62%	-90.7%
		Female	29904	17562	18.1%	-48.8%	-61.6%	-92.2%
		Male	27056	16722	18.2%	-45.1%	-62.1%	-89.1%
	Maternal and neonatal disorders	Both	342481	184989	18.2%	-54.3%	-52.9%	-89%
		Female	150516	82453	18.1%	-53.4%	-53.2%	-88.5%
		Male	191976	102560	18.2%	-55.1%	-52.6%	-89.5%
	Nutritional deficiencies	Both	24763	18348	18.2%	-30.6%	-58.6%	-71.1%
		Female	13132	10068	18.1%	-27.6%	-61.1%	-70.5%
		Male	11630	8289	18.2%	-34%	-55.9%	-71.7%
	**Non-communicable diseases**	Both	838226	1123687	18.2%	40.2%	-40.2%	18.2%
		Female	396033	540743	18.1%	43.2%	-38.2%	23.1%
		Male	442202	577469	18.2%	36.2%	-40.5%	13.9%

Location	Cause	Sex	Expected DALYs in 2019		% 1990 - 2019 DALYs change cause			% 1990 - 2019 DALYs overall change
			Population growth	Population growth + Aging	Population growth	Age structure change	DALYs rate change	
	Neoplasms	Both	84025	129236	18.2%	63.6%	-12.5%	69.2%
		Female	36452	55974	18.1%	63.3%	-13.7%	67.7%
		Male	47575	71737	18.2%	60%	-7.8%	70.4%
	Cardiovascular diseases	Both	190563	378147	18.2%	116.3%	-100.7%	33.7%
		Female	76313	163253	18.1%	134.6%	-109.9%	42.8%
		Male	114261	211814	18.2%	100.9%	-91.4%	27.7%
	Chronic respiratory diseases	Both	34545	58968	18.2%	83.5%	-64%	37.7%
		Female	15621	27546	18.1%	90.2%	-73.5%	34.8%
		Male	18925	31073	18.2%	75.9%	-54%	40.1%
	Digestive diseases	Both	24026	34174	18.2%	49.9%	-50.1%	18%
		Female	10967	15593	18.1%	49.8%	-52%	15.9%
		Male	13059	18392	18.2%	48.3%	-46.8%	19.7%
	Neurological disorders	Both	51198	63096	18.2%	27.5%	-14.8%	30.8%
		Female	26960	33916	18.1%	30.5%	-10.7%	37.9%
		Male	24237	29137	18.2%	23.9%	-19.2%	22.9%
	Mental disorders	Both	74919	89632	18.2%	23.2%	3.3%	44.7%
		Female	40727	49543	18.1%	25.6%	3.3%	47%
		Male	34190	40032	18.2%	20.2%	3.5%	41.9%
	Substance use disorders	Both	11042	14839	18.2%	40.6%	27.7%	86.5%
		Female	3335	4283	18.1%	33.6%	-8.1%	43.6%
		Male	7708	10641	18.2%	45%	42%	105.2%
	Diabetes and kidney diseases	Both	29416	47868	18.2%	74.1%	56.5%	148.8%
		Female	14072	23615	18.1%	80.1%	62.4%	160.7%
		Male	15344	24144	18.2%	67.8%	51.9%	137.9%
	Skin and subcutaneous diseases	Both	17636	16630	18.2%	-6.7%	1.2%	12.6%
		Female	9189	8644	18.1%	-7%	0.8%	11.9%
		Male	8447	7980	18.2%	-6.5%	1.7%	13.4%
	Sense organ diseases	Both	23433	40146	18.2%	84.3%	-46.4%	56%
		Female	11159	20110	18.1%	94.7%	-49.2%	63.7%
		Male	12273	20041	18.2%	74.8%	-44%	49%
	Musculoskeletal disorders	Both	47721	79418	18.2%	78.5%	3.7%	100.4%
		Female	24767	42134	18.1%	82.8%	11.7%	112.7%
		Male	22953	37393	18.2%	74.4%	-5.4%	87.1%
	Other non-communicable diseases	Both	249702	171534	18.2%	-37%	-42.3%	-61.2%
		Female	126470	96132	18.1%	-28.3%	-39.2%	-49.4%
		Male	123230	75085	18.2%	-46.2%	-45.2%	-73.2%
	**Injuries***	Both	296101	273391	18.9%	-9.1%	-58.3%	-48.5%
		Female	95828	80634	18.5%	-18.8%	-59.9%	-60.1%
		Male	200590	193745	19.3%	-4.1%	-58.1%	-42.8%
	Transport Injuries	Both	165566	150735	18.2%	-10.6%	-60.4%	-52.8%
		Female	51665	41271	18.1%	-23.8%	-60.4%	-66%
		Male	113920	109818	18.2%	-4.3%	-60.8%	-46.9%

Location	Cause	Sex	Expected DALYs in 2019		% 1990 - 2019 DALYs change cause			% 1990 - 2019 DALYs overall change
			Population growth	Population growth + Aging	Population growth	Age structure change	DALYs rate change	
	Unintentional Injuries*	Both	102585	89910	18.9%	-14.7%	-58.7%	-54.5%
		Female	37375	30776	18.5%	-20.9%	-59.4%	-61.8%
		Male	65293	59279	19.3%	-11%	-58.6%	-50.3%
	Self-harm and interpersonal violence	Both	35522	37404	18.2%	6.3%	-47.9%	-23.5%
		Female	9799	9984	18.1%	2.2%	-56%	-35.7%
		Male	25727	27654	18.2%	8.9%	-45.9%	-18.8%
**Fars**	**All causes**	Both	1812519	1953012	35.9%	10.5%	-48.6%	-2.1%
		Female	780421	837655	36.5%	10%	-45.5%	1%
		Male	1031187	1110240	35.4%	10.4%	-50.2%	-4.4%
	**Communicable, maternal, neonatal, and nutritional diseases**	Both	537424	315558	35.9%	-56.1%	-52.6%	-72.8%
		Female	244717	144953	36.5%	-55.7%	-54.4%	-73.6%
		Male	292549	170613	35.4%	-56.4%	-51.2%	-72.2%
	HIV/AIDS and sexually transmitted infections	Both	1725	1803	35.9%	6.2%	458.9%	501%
		Female	775	803	36.5%	4.8%	303%	344.3%
		Male	949	1000	35.4%	7.4%	585.2%	627.9%
	Respiratory infections and tuberculosis	Both	68149	49593	35.9%	-37%	-51.3%	-52.4%
		Female	32135	22041	36.5%	-42.9%	-51.8%	-58.2%
		Male	36004	27598	35.4%	-31.6%	-51%	-47.2%
	Enteric infections	Both	32267	20250	35.9%	-50.6%	-35.3%	-50%
		Female	15134	9478	36.5%	-51%	-34.3%	-48.8%
		Male	17127	10746	35.4%	-50.4%	-36%	-51%
	Neglected tropical diseases and malaria	Both	21105	21880	35.9%	5%	-134.9%	-94%
		Female	9056	9586	36.5%	8%	-137.7%	-93.2%
		Male	12038	12300	35.4%	2.9%	-132.9%	-94.6%
	Other infectious diseases	Both	43820	26688	35.9%	-53.1%	-64.6%	-81.8%
		Female	22720	13209	36.5%	-57.1%	-64.5%	-85.1%
		Male	21110	13427	35.4%	-49.3%	-64.4%	-78.3%
	Maternal and neonatal disorders	Both	351392	180185	35.9%	-66.2%	-50.3%	-80.6%
		Female	154851	81338	36.5%	-64.8%	-51.8%	-80.1%
		Male	196395	98889	35.4%	-67.2%	-49.1%	-80.9%
	Nutritional deficiencies	Both	18966	15158	35.9%	-27.3%	-58.3%	-49.7%
		Female	10046	8498	36.5%	-21%	-63.4%	-47.9%
		Male	8927	6653	35.4%	-34.5%	-52.5%	-51.6%
	**Non-communicable diseases**	Both	867084	1224016	35.9%	56%	-38.5%	53.4%
		Female	409629	577855	36.5%	56.1%	-33.9%	58.7%
		Male	457325	641496	35.4%	54.5%	-41.2%	48.7%
	Neoplasms	Both	67895	106889	35.9%	78.1%	-2.3%	111.7%
		Female	28334	44419	36.5%	77.5%	5.8%	119.9%
		Male	39520	61989	35.4%	77%	-6.4%	105.9%
	Cardiovascular diseases	Both	211492	436100	35.9%	144.4%	-121.6%	58.7%
		Female	83045	177417	36.5%	155.1%	-125.5%	66.1%
		Male	128274	254575	35.4%	133.3%	-114.7%	53.9%

Location	Cause	Sex	Expected DALYs in 2019		% 1990 - 2019 DALYs change cause			% 1990 - 2019 DALYs overall change
			Population growth	Population growth + Aging	Population growth	Age structure change	DALYs rate change	
	Chronic respiratory diseases	Both	24597	38722	35.9%	78.1%	-29.2%	84.8%
		Female	11069	17260	36.5%	76.4%	-37.7%	75.1%
		Male	13519	21443	35.4%	79.3%	-22%	92.7%
	Digestive diseases	Both	28275	42432	35.9%	68.1%	-56.5%	47.5%
		Female	12040	17595	36.5%	63%	-57.3%	42.2%
		Male	16220	24628	35.4%	70.2%	-54.2%	51.3%
	Neurological disorders	Both	52039	70481	35.9%	48.2%	-2.7%	81.4%
		Female	28822	39261	36.5%	49.4%	0.4%	86.3%
		Male	23245	31315	35.4%	47%	-7%	75.3%
	Mental disorders	Both	110328	137825	35.9%	33.9%	1.9%	71.7%
		Female	57526	72797	36.5%	36.2%	1%	73.8%
		Male	52831	65038	35.4%	31.3%	2.8%	69.4%
	Substance use disorders	Both	14876	21650	35.9%	61.9%	52.3%	150.1%
		Female	4298	5986	36.5%	53.6%	18.9%	109%
		Male	10552	15693	35.4%	66%	65.4%	166.7%
	Diabetes and kidney diseases	Both	25057	41900	35.9%	91.4%	100.2%	227.5%
		Female	11744	19851	36.5%	94.3%	116.9%	247.7%
		Male	13309	21981	35.4%	88.2%	86.3%	209.9%
	Skin and subcutaneous diseases	Both	21542	20060	35.9%	-9.4%	2%	28.6%
		Female	11261	10443	36.5%	-9.9%	1.4%	28%
		Male	10287	9618	35.4%	-8.8%	2.6%	29.1%
	Sense organ diseases	Both	26673	45693	35.9%	96.9%	-51.2%	81.7%
		Female	13009	22669	36.5%	101.4%	-52.6%	85.3%
		Male	13664	23001	35.4%	92.5%	-49.6%	78.2%
	Musculoskeletal disorders	Both	57232	98487	35.9%	98%	13.1%	147%
		Female	30394	53113	36.5%	102%	17.8%	156.3%
		Male	26858	45511	35.4%	94%	7.2%	136.6%
	Other non-communicable diseases	Both	227079	163778	35.9%	-37.9%	-35.2%	-37.2%
		Female	118088	97043	36.5%	-24.3%	-33.3%	-21.1%
		Male	109047	66703	35.4%	-52.6%	-37.2%	-54.4%
	**Injuries***	Both	395860	402436	35.4%	2.2%	-62.6%	-25%
		Female	122443	112382	35.6%	-11.1%	-64.8%	-40.3%
		Male	273185	290078	35.1%	8.4%	-61.6%	-18.1%
	Transport Injuries	Both	223777	225817	35.9%	1.2%	-66%	-28.9%
		Female	65970	58169	36.5%	-16.1%	-66.7%	-46.3%
		Male	157440	167275	35.4%	8.5%	-65.4%	-21.6%
	Unintentional Injuries*	Both	108691	103114	35.4%	-6.9%	-59.7%	-31.3%
		Female	37084	31759	35.6%	-19.5%	-58.9%	-42.8%
		Male	71555	71327	35.1%	-0.4%	-60.1%	-25.4%
	Self-harm and interpersonal violence	Both	71397	81684	35.9%	19.6%	-65.7%	-10.2%
		Female	21202	23960	36.5%	17.8%	-75%	-20.7%
		Male	50080	57755	35.4%	20.7%	-61.9%	-5.8%

Location	Cause	Sex	Expected DALYs in 2019		% 1990 - 2019 DALYs change cause			% 1990 - 2019 DALYs overall change
			Population growth	Population growth + Aging	Population growth	Age structure change	DALYs rate change	
**Gilan**	**All causes**	Both	2883097	2784284	11.6%	-3.8%	-80.2%	-72.5%
		Female	1162307	1096095	11.7%	-6.4%	-73.2%	-67.8%
		Male	1720119	1693927	11.4%	-1.7%	-85.4%	-75.6%
	**Communicable, maternal, neonatal, and nutritional diseases**	Both	216332	115282	11.6%	-52.1%	-41.7%	-82.2%
		Female	99082	53303	11.7%	-51.6%	-41.6%	-81.5%
		Male	117229	61995	11.4%	-52.5%	-41.7%	-82.8%
	HIV/AIDS and sexually transmitted infections	Both	730	619	11.6%	-17%	26.8%	21.4%
		Female	383	366	11.7%	-4.9%	30.2%	37%
		Male	347	252	11.4%	-30.6%	23.5%	4.3%
	Respiratory infections and tuberculosis	Both	25320	19089	11.6%	-27.5%	-44%	-59.9%
		Female	11917	8459	11.7%	-32.4%	-42.4%	-63.1%
		Male	13402	10723	11.4%	-22.3%	-46.2%	-57%
	Enteric infections	Both	12977	8385	11.6%	-39.5%	-20%	-47.9%
		Female	6094	3951	11.7%	-39.3%	-19.1%	-46.6%
		Male	6882	4408	11.4%	-40.1%	-20.4%	-49%
	Neglected tropical diseases and malaria	Both	6308	6277	11.6%	-0.6%	-104.8%	-93.8%
		Female	2943	2693	11.7%	-9.5%	-95.4%	-93.2%
		Male	3364	3608	11.4%	8.1%	-113.8%	-94.3%
	Other infectious diseases	Both	24810	12737	11.6%	-54.3%	-47.1%	-89.8%
		Female	13044	6454	11.7%	-56.4%	-46.7%	-91.4%
		Male	11768	6263	11.4%	-52.1%	-47.4%	-88.1%
	Maternal and neonatal disorders	Both	137694	61199	11.6%	-62%	-39.4%	-89.8%
		Female	60135	27404	11.7%	-60.8%	-39.7%	-88.8%
		Male	77538	33795	11.4%	-62.9%	-39.1%	-90.6%
	Nutritional deficiencies	Both	8493	6977	11.6%	-19.9%	-47.8%	-56.1%
		Female	4566	3976	11.7%	-14.4%	-50.9%	-53.6%
		Male	3928	2944	11.4%	-27.9%	-42.5%	-59%
	**Non-communicable diseases**	Both	473947	751969	11.6%	65.5%	-38.6%	38.4%
		Female	231085	362268	11.7%	63.4%	-33%	42.1%
		Male	242851	388041	11.4%	66.6%	-43.2%	34.8%
	Neoplasms	Both	43795	79592	11.6%	91.2%	-9.9%	92.9%
		Female	18464	32960	11.7%	87.7%	-2.5%	96.9%
		Male	25323	46944	11.4%	95.1%	-16.6%	90%
	Cardiovascular diseases	Both	142160	318940	11.6%	138.8%	-108%	42.3%
		Female	61291	139581	11.7%	142.7%	-107%	47.5%
		Male	80845	177482	11.4%	133.2%	-106.2%	38.4%
	Chronic respiratory diseases	Both	14973	27118	11.6%	90.5%	-43.7%	58.4%
		Female	6450	11425	11.7%	86.2%	-49.2%	48.7%
		Male	8521	15877	11.4%	96.2%	-42%	65.6%
	Digestive diseases	Both	11670	19719	11.6%	77%	-55.6%	33%
		Female	5246	8606	11.7%	71.6%	-48.3%	35%
		Male	6422	11080	11.4%	80.8%	-61%	31.3%

Location	Cause	Sex	Expected DALYs in 2019		% 1990 - 2019 DALYs change cause			% 1990 - 2019 DALYs overall change
			Population growth	Population growth + Aging	Population growth	Age structure change	DALYs rate change	
	Neurological disorders	Both	26731	39112	11.6%	51.7%	-1.6%	61.7%
		Female	14974	21527	11.7%	48.9%	0.3%	60.9%
		Male	11761	17589	11.4%	55.2%	-4.1%	62.6%
	Mental disorders	Both	51033	60956	11.6%	21.7%	2.9%	36.2%
		Female	28685	34666	11.7%	23.3%	2.3%	37.3%
		Male	22357	26211	11.4%	19.2%	4%	34.7%
	Substance use disorders	Both	5139	6778	11.6%	35.6%	64.6%	111.7%
		Female	1806	2314	11.7%	31.5%	4.2%	47.4%
		Male	3332	4483	11.4%	38.5%	96.5%	146.5%
	Diabetes and kidney diseases	Both	13820	26537	11.6%	102.7%	142.3%	256.6%
		Female	6856	13109	11.7%	101.9%	167.6%	281.2%
		Male	6964	13367	11.4%	102.5%	118.5%	232.4%
	Skin and subcutaneous diseases	Both	11185	10469	11.6%	-7.1%	0.8%	5.3%
		Female	5898	5498	11.7%	-7.6%	0.5%	4.7%
		Male	5288	4968	11.4%	-6.7%	1.2%	5.9%
	Sense organ diseases	Both	15271	29316	11.6%	102.6%	-51.3%	62.9%
		Female	7695	14756	11.7%	102.5%	-52%	62.2%
		Male	7576	14519	11.4%	102.1%	-50.1%	63.5%
	Musculoskeletal disorders	Both	34230	58953	11.6%	80.6%	7.3%	99.4%
		Female	18466	32112	11.7%	82.6%	11.7%	106%
		Male	15768	26854	11.4%	78.4%	2%	91.8%
	Other non-communicable diseases	Both	103941	74478	11.6%	-31.6%	-29.8%	-49.9%
		Female	55254	45713	11.7%	-19.3%	-27.1%	-34.7%
		Male	48696	28666	11.4%	-45.8%	-32.7%	-67.1%
	**Injuries***	Both	179039	186940	13%	5%	-62.1%	-44.1%
		Female	56251	56537	12.6%	0.6%	-64.7%	-51.5%
		Male	123023	131269	13.4%	7.6%	-61.6%	-40.6%
	Transport Injuries	Both	85374	87810	11.6%	3.2%	-64.2%	-49.4%
		Female	23868	22858	11.7%	-4.7%	-68.6%	-61.6%
		Male	61460	65231	11.4%	6.8%	-63%	-44.7%
	Unintentional Injuries*	Both	69074	70925	13%	3%	-58.5%	-42.5%
		Female	24784	24668	12.6%	-0.5%	-58.4%	-46.3%
		Male	44358	46476	13.4%	5.4%	-59.1%	-40.3%
	Self-harm and interpersonal violence	Both	26773	30349	11.6%	14.9%	-65%	-38.6%
		Female	8009	9426	11.7%	19.8%	-73%	-41.5%
		Male	18751	21049	11.4%	13.7%	-62.4%	-37.3%
**Golestan**	**All causes**	Both	850559	834687	43.9%	-2.7%	-56.8%	-15.5%
		Female	381125	375881	44.3%	-2%	-56.4%	-14.1%
		Male	469211	456378	43.5%	-3.9%	-56.3%	-16.7%
	**Communicable, maternal, neonatal, and nutritional diseases**	Both	306202	192799	43.9%	-53.3%	-69.3%	-78.7%
		Female	138583	87473	44.3%	-53.2%	-69.7%	-78.6%
		Male	167546	105255	43.5%	-53.4%	-68.9%	-78.7%

Location	Cause	Sex	Expected DALYs in 2019		% 1990 - 2019 DALYs change cause			% 1990 - 2019 DALYs overall change
			Population growth	Population growth + Aging	Population growth	Age structure change	DALYs rate change	
	HIV/AIDS and sexually transmitted infections	Both	619	571	43.9%	-11.2%	48%	80.7%
		Female	310	309	44.3%	-0.4%	51.1%	95%
		Male	309	262	43.5%	-22%	44.8%	66.4%
	Respiratory infections and tuberculosis	Both	50407	36054	43.9%	-41%	-76.6%	-73.6%
		Female	23491	16341	44.3%	-43.9%	-76.6%	-76.2%
		Male	26907	19661	43.5%	-38.7%	-76.2%	-71.4%
	Enteric infections	Both	21467	13984	43.9%	-50.2%	-60.5%	-66.7%
		Female	10368	6714	44.3%	-50.9%	-60.7%	-67.2%
		Male	11097	7257	43.5%	-49.7%	-60.1%	-66.2%
	Neglected tropical diseases and malaria	Both	3281	3331	43.9%	2.2%	-130.6%	-84.4%
		Female	1561	1566	44.3%	0.5%	-128.4%	-83.6%
		Male	1719	1761	43.5%	3.4%	-132.2%	-85.2%
	Other infectious diseases	Both	29634	19686	43.9%	-48.3%	-78.9%	-83.3%
		Female	15568	10043	44.3%	-51.2%	-79.6%	-86.5%
		Male	14070	9597	43.5%	-45.6%	-77.7%	-79.8%
	Maternal and neonatal disorders	Both	188411	109797	43.9%	-60.1%	-66%	-82.1%
		Female	80865	47490	44.3%	-59.6%	-66%	-81.2%
		Male	107478	62353	43.5%	-60.3%	-66.1%	-82.8%
	Nutritional deficiencies	Both	12383	9376	43.9%	-35%	-72.1%	-63.1%
		Female	6419	5010	44.3%	-31.7%	-74.2%	-61.6%
		Male	5965	4365	43.5%	-38.5%	-69.7%	-64.7%
	**Non-communicable diseases**	Both	393760	498153	43.9%	38.2%	-42%	40.1%
		Female	192087	244087	44.3%	39.1%	-41.3%	42.1%
		Male	201654	251585	43.5%	35.5%	-40.8%	38.3%
	Neoplasms	Both	30566	47526	43.9%	79.9%	-9.5%	114.3%
		Female	13425	21181	44.3%	83.4%	-14.4%	113.3%
		Male	17132	25841	43.5%	73%	-1.4%	115.1%
	Cardiovascular diseases	Both	93375	169857	43.9%	117.9%	-90.3%	71.5%
		Female	38738	72800	44.3%	126.9%	-97.3%	73.9%
		Male	54595	95383	43.5%	107.2%	-80.9%	69.9%
	Chronic respiratory diseases	Both	12175	17893	43.9%	67.6%	-32.2%	79.3%
		Female	5470	8174	44.3%	71.4%	-45.1%	70.5%
		Male	6702	9613	43.5%	62.4%	-19.4%	86.5%
	Digestive diseases	Both	13723	18503	43.9%	50.1%	-61.5%	32.6%
		Female	6245	8243	44.3%	46.2%	-66.2%	24.3%
		Male	7474	10133	43.5%	51.1%	-55.2%	39.4%
	Neurological disorders	Both	21442	26116	43.9%	31.4%	-11.1%	64.2%
		Female	11777	14506	44.3%	33.4%	-9.7%	68.1%
		Male	9671	11602	43.5%	28.7%	-12.8%	59.4%
	Mental disorders	Both	34625	41572	43.9%	28.9%	3.5%	76.3%
		Female	19017	23262	44.3%	32.2%	3.6%	80.1%
		Male	15617	18296	43.5%	24.6%	3.5%	71.7%

Location	Cause	Sex	Expected DALYs in 2019		% 1990 - 2019 DALYs change cause			% 1990 - 2019 DALYs overall change
			Population growth	Population growth + Aging	Population growth	Age structure change	DALYs rate change	
	Substance use disorders	Both	3907	5468	43.9%	57.5%	18.4%	119.9%
		Female	1446	1939	44.3%	49.1%	-16.7%	76.8%
		Male	2458	3540	43.5%	63.2%	38.3%	145.1%
	Diabetes and kidney diseases	Both	11340	17651	43.9%	80.1%	105.4%	229.5%
		Female	5594	9004	44.3%	88%	111.6%	243.9%
		Male	5745	8615	43.5%	71.7%	100.3%	215.5%
	Skin and subcutaneous diseases	Both	8388	7898	43.9%	-8.4%	2.5%	38.1%
		Female	4439	4171	44.3%	-8.7%	1.9%	37.5%
		Male	3950	3726	43.5%	-8.1%	3.3%	38.7%
	Sense organ diseases	Both	10016	16015	43.9%	86.2%	-51.8%	78.3%
		Female	4902	8134	44.3%	95.1%	-54%	85.4%
		Male	5113	7873	43.5%	77.5%	-49.4%	71.6%
	Musculoskeletal disorders	Both	22250	36644	43.9%	93.1%	8%	145%
		Female	11934	20067	44.3%	98.4%	12.7%	155.4%
		Male	10320	16640	43.5%	87.9%	1.6%	133.1%
	Other non-communicable diseases	Both	131955	93012	43.9%	-42.5%	-56.1%	-54.6%
		Female	69098	52605	44.3%	-34.4%	-54.5%	-44.6%
		Male	62876	40325	43.5%	-51.5%	-57.6%	-65.5%
	**Injuries***	Both	146712	141921	42.9%	-4.7%	-69.6%	-31.4%
		Female	49032	43870	42.8%	-15%	-77.2%	-49.5%
		Male	97730	98378	43.1%	0.9%	-66.3%	-22.2%
	Transport Injuries	Both	82125	78206	43.9%	-6.9%	-73.2%	-36.2%
		Female	25019	21585	44.3%	-19.8%	-81.8%	-57.3%
		Male	57021	56695	43.5%	-0.8%	-69.7%	-27%
	Unintentional Injuries*	Both	48471	43867	42.9%	-13.6%	-68.8%	-39.4%
		Female	17300	14604	42.8%	-22.3%	-72%	-51.4%
		Male	31185	29305	43.1%	-8.6%	-67.2%	-32.7%
	Self-harm and interpersonal violence	Both	18368	20994	43.9%	20.6%	-58.8%	5.7%
		Female	7466	7942	44.3%	9.2%	-75.1%	-21.6%
		Male	10893	13106	43.5%	29.2%	-48.4%	24.3%
**Hamadan**	**All causes**	Both	796451	795146	3.4%	-0.2%	-41.3%	-38%
		Female	354727	351512	5.1%	-1%	-41.7%	-37.5%
		Male	440566	440408	1.8%	0%	-40.3%	-38.5%
	**Communicable, maternal, neonatal, and nutritional diseases**	Both	280792	159320	3.4%	-44.7%	-44.5%	-85.8%
		Female	133615	74987	5.1%	-46.1%	-45.3%	-86.3%
		Male	147037	84258	1.8%	-43.5%	-43.7%	-85.3%
	HIV/AIDS and sexually transmitted infections	Both	538	482	3.4%	-10.8%	50.6%	43.3%
		Female	271	262	5.1%	-3.7%	11.9%	13.3%
		Male	267	219	1.8%	-18.1%	89.1%	72.8%
	Respiratory infections and tuberculosis	Both	45128	28995	3.4%	-37%	-47.8%	-81.3%
		Female	22348	13688	5.1%	-40.7%	-48.7%	-84.3%
		Male	22786	15248	1.8%	-33.7%	-46.7%	-78.5%

Location	Cause	Sex	Expected DALYs in 2019		% 1990 - 2019 DALYs change cause			% 1990 - 2019 DALYs overall change
			Population growth	Population growth + Aging	Population growth	Age structure change	DALYs rate change	
	Enteric infections	Both	17167	10744	3.4%	-38.7%	-37.5%	-72.8%
		Female	7947	4936	5.1%	-39.8%	-37.1%	-71.8%
		Male	9204	5794	1.8%	-37.7%	-37.8%	-73.6%
	Neglected tropical diseases and malaria	Both	1941	1554	3.4%	-20.6%	-67.1%	-84.2%
		Female	898	753	5.1%	-17%	-71.1%	-82.9%
		Male	1041	799	1.8%	-23.6%	-63.6%	-85.4%
	Other infectious diseases	Both	32415	19154	3.4%	-42.3%	-52.3%	-91.2%
		Female	16778	9511	5.1%	-45.5%	-52.4%	-92.8%
		Male	15664	9619	1.8%	-39.3%	-52.1%	-89.5%
	Maternal and neonatal disorders	Both	175291	91952	3.4%	-49.2%	-42.7%	-88.5%
		Female	81120	42365	5.1%	-50.2%	-43.4%	-88.5%
		Male	94012	49603	1.8%	-48.1%	-42.2%	-88.5%
	Nutritional deficiencies	Both	8312	6439	3.4%	-23.3%	-47.6%	-67.4%
		Female	4253	3472	5.1%	-19.3%	-50.3%	-64.5%
		Male	4064	2976	1.8%	-27.3%	-45%	-70.4%
	**Non-communicable diseases**	Both	349203	482337	3.4%	39.4%	-33.4%	9.4%
		Female	166566	231183	5.1%	40.8%	-34.6%	11.4%
		Male	182476	248418	1.8%	36.8%	-30.9%	7.8%
	Neoplasms	Both	29998	45985	3.4%	55.1%	-11.2%	47.4%
		Female	12955	19604	5.1%	54%	-16.2%	42.9%
		Male	16986	25891	1.8%	53.4%	-4.5%	50.7%
	Cardiovascular diseases	Both	91028	183112	3.4%	104.6%	-84.6%	23.5%
		Female	36873	78806	5.1%	119.6%	-96.8%	27.9%
		Male	53907	102407	1.8%	91.6%	-72.9%	20.5%
	Chronic respiratory diseases	Both	11787	19247	3.4%	65.5%	-41.1%	27.8%
		Female	4926	8192	5.1%	69.7%	-52.3%	22.5%
		Male	6833	10839	1.8%	59.7%	-30.1%	31.5%
	Digestive diseases	Both	11547	16292	3.4%	42.5%	-42.9%	3%
		Female	5257	7313	5.1%	41.1%	-50.1%	-3.8%
		Male	6277	8908	1.8%	42.7%	-35.9%	8.6%
	Neurological disorders	Both	19422	26320	3.4%	36.7%	-5.6%	34.5%
		Female	10599	14241	5.1%	36.1%	-4.3%	37%
		Male	8856	12088	1.8%	37.2%	-7.3%	31.7%
	Mental disorders	Both	32640	39007	3.4%	20.2%	1.4%	25%
		Female	17955	21902	5.1%	23.1%	1.1%	29.4%
		Male	14745	17129	1.8%	16.5%	1.5%	19.8%
	Substance use disorders	Both	5145	6955	3.4%	36.4%	103%	142.8%
		Female	1702	2231	5.1%	32.6%	15.7%	53.5%
		Male	3417	4739	1.8%	39.4%	144.7%	185.9%
	Diabetes and kidney diseases	Both	10018	16309	3.4%	64.9%	49.7%	118.1%
		Female	4828	7948	5.1%	67.9%	57.3%	130.4%
		Male	5187	8294	1.8%	61%	44.1%	106.9%

Location	Cause	Sex	Expected DALYs in 2019		% 1990 - 2019 DALYs change cause			% 1990 - 2019 DALYs overall change
			Population growth	Population growth + Aging	Population growth	Age structure change	DALYs rate change	
	Skin and subcutaneous diseases	Both	7479	7006	3.4%	-6.5%	1.2%	-1.9%
		Female	3904	3650	5.1%	-6.8%	0.8%	-0.8%
		Male	3582	3357	1.8%	-6.4%	1.6%	-2.9%
	Sense organ diseases	Both	10123	18262	3.4%	83.2%	-40.7%	45.9%
		Female	4912	9131	5.1%	90.3%	-43.4%	52%
		Male	5209	9135	1.8%	76.7%	-38.3%	40.3%
	Musculoskeletal disorders	Both	21045	35541	3.4%	71.2%	9.4%	84.1%
		Female	11243	19189	5.1%	74.3%	13.3%	92.8%
		Male	9830	16403	1.8%	68.1%	4.6%	74.5%
	Other non-communicable diseases	Both	98970	68302	3.4%	-32.1%	-34.5%	-63.1%
		Female	51411	38976	5.1%	-25.4%	-32.6%	-52.9%
		Male	47646	29229	1.8%	-39.4%	-36.2%	-73.8%
	**Injuries***	Both	159730	148708	3.7%	-7.2%	-51.7%	-55.2%
		Female	52222	44090	4.8%	-16.3%	-54.2%	-65.7%
		Male	106943	104488	2.6%	-2.4%	-50.4%	-50.2%
	Transport Injuries	Both	91111	82057	3.4%	-10.3%	-54.3%	-61.2%
		Female	30144	23661	5.1%	-22.6%	-55.5%	-73%
		Male	60506	58133	1.8%	-4%	-53.3%	-55.5%
	Unintentional Injuries*	Both	45155	39941	3.7%	-12%	-48%	-56.3%
		Female	15590	12799	4.8%	-18.8%	-50.3%	-64.2%
		Male	29423	27122	2.6%	-8%	-46.8%	-52.2%
	Self-harm and interpersonal violence	Both	28392	30558	3.4%	7.9%	-53.9%	-42.5%
		Female	8047	8569	5.1%	6.8%	-59.2%	-47.2%
		Male	20159	21909	1.8%	8.8%	-51.4%	-40.7%
**Hormozgan**	**All causes**	Both	910649	813796	105%	-21.8%	-89.7%	-6.5%
		Female	404014	360895	105.1%	-21.9%	-90.5%	-7.3%
		Male	506584	450612	104.9%	-22.6%	-88.1%	-5.8%
	**Communicable, maternal, neonatal, and nutritional diseases**	Both	394195	253000	105%	-73.4%	-105%	-73.5%
		Female	180944	116525	105.1%	-73%	-106.3%	-74.2%
		Male	213236	136399	104.9%	-73.8%	-103.9%	-72.9%
	HIV/AIDS and sexually transmitted infections	Both	805	769	105%	-9.2%	1072.1%	1167.9%
		Female	369	352	105.1%	-9.4%	1030.7%	1126.5%
		Male	436	417	104.9%	-8.7%	1106.8%	1203%
	Respiratory infections and tuberculosis	Both	75743	50443	105%	-68.5%	-112.9%	-76.4%
		Female	36905	23976	105.1%	-71.9%	-112.8%	-79.5%
		Male	38837	26440	104.9%	-65.4%	-112.9%	-73.4%
	Enteric infections	Both	39209	25049	105%	-74%	-101.6%	-70.7%
		Female	16459	10411	105.1%	-75.4%	-99%	-69.2%
		Male	22747	14647	104.9%	-73%	-103.6%	-71.7%
	Neglected tropical diseases and malaria	Both	18144	16562	105%	-17.9%	-182.7%	-95.6%
		Female	8970	7755	105.1%	-27.8%	-173.1%	-95.7%
		Male	9174	8725	104.9%	-10%	-190.3%	-95.5%

Location	Cause	Sex	Expected DALYs in 2019		% 1990 - 2019 DALYs change cause			% 1990 - 2019 DALYs overall change
			Population growth	Population growth + Aging	Population growth	Age structure change	DALYs rate change	
	Other infectious diseases	Both	39667	25228	105%	-74.6%	-117.4%	-87%
		Female	20954	13108	105.1%	-76.8%	-117.4%	-89%
		Male	18716	12085	104.9%	-72.6%	-117.1%	-84.8%
	Maternal and neonatal disorders	Both	204075	122855	105%	-81.6%	-97.6%	-74.2%
		Female	88478	54395	105.1%	-79%	-99.5%	-73.4%
		Male	115584	68529	104.9%	-83.4%	-96.3%	-74.8%
	Nutritional deficiencies	Both	16552	12093	105%	-55.2%	-110.9%	-61.1%
		Female	8810	6528	105.1%	-53.1%	-113.9%	-62%
		Male	7743	5557	104.9%	-57.8%	-107.1%	-60.1%
	**Non-communicable diseases**	Both	368887	417988	105%	27.3%	-66.7%	65.6%
		Female	171890	200332	105.1%	33.9%	-64.3%	74.7%
		Male	196987	215601	104.9%	19.4%	-66.6%	57.6%
	Neoplasms	Both	20800	26202	105%	53.2%	-32.6%	125.7%
		Female	9010	11987	105.1%	67.8%	-36.5%	136.4%
		Male	11788	13988	104.9%	38.2%	-25.6%	117.5%
	Cardiovascular diseases	Both	96270	136051	105%	84.7%	-139.6%	50.1%
		Female	37235	55304	105.1%	99.5%	-151.7%	52.9%
		Male	59022	79017	104.9%	69.4%	-126%	48.3%
	Chronic respiratory diseases	Both	9660	11971	105%	49%	-77.8%	76.2%
		Female	4239	5398	105.1%	56%	-87.6%	73.6%
		Male	5420	6509	104.9%	41.2%	-67.7%	78.3%
	Digestive diseases	Both	12995	14971	105%	31.2%	-86.9%	49.3%
		Female	5848	6654	105.1%	28.3%	-95%	38.4%
		Male	7147	8191	104.9%	29.9%	-76.6%	58.2%
	Neurological disorders	Both	19739	23614	105%	40.2%	-14.2%	131.1%
		Female	10524	12778	105.1%	43.9%	-10.3%	138.7%
		Male	9216	10850	104.9%	36.3%	-18.8%	122.4%
	Mental disorders	Both	37038	44788	105%	42.9%	5.1%	153%
		Female	20141	24844	105.1%	47.9%	4.6%	157.6%
		Male	16900	19966	104.9%	37.2%	5.5%	147.6%
	Substance use disorders	Both	6261	8393	105%	69.8%	60.9%	235.7%
		Female	1961	2552	105.1%	61.8%	-3.9%	163%
		Male	4298	5852	104.9%	74.1%	89.9%	268.9%
	Diabetes and kidney diseases	Both	13640	17042	105%	51.1%	18.3%	174.4%
		Female	6365	8170	105.1%	58.2%	20.8%	184.1%
		Male	7275	8831	104.9%	43.8%	17.2%	165.9%
	Skin and subcutaneous diseases	Both	8081	7649	105%	-11%	4.7%	98.7%
		Female	4205	3970	105.1%	-11.5%	3.7%	97.4%
		Male	3876	3680	104.9%	-10.4%	5.7%	100.1%
	Sense organ diseases	Both	10512	14036	105%	68.7%	-65.2%	108.5%
		Female	4992	6922	105.1%	79.3%	-68.6%	115.8%
		Male	5520	7108	104.9%	58.9%	-61.9%	101.9%

Location	Cause	Sex	Expected DALYs in 2019		% 1990 - 2019 DALYs change cause			% 1990 - 2019 DALYs overall change
			Population growth	Population growth + Aging	Population growth	Age structure change	DALYs rate change	
	Musculoskeletal disorders	Both	21240	31601	105%	100%	16%	221%
		Female	10978	16831	105.1%	109.4%	22.4%	236.8%
		Male	10262	14840	104.9%	91.4%	7.8%	204%
	Other non-communicable diseases	Both	112652	81670	105%	-56.4%	-78.4%	-29.8%
		Female	56392	44925	105.1%	-41.7%	-74.1%	-10.7%
		Male	56262	36770	104.9%	-71%	-82.9%	-49%
	**Injuries***	Both	142966	139444	101%	-5%	-102.5%	-6.5%
		Female	49443	43131	101.1%	-25.7%	-120.6%	-45.2%
		Male	93511	96191	100.9%	5.8%	-92.7%	14%
	Transport Injuries	Both	75293	74901	105%	-1.1%	-103.4%	0.5%
		Female	25583	22463	105.1%	-25%	-128.4%	-48.3%
		Male	49696	52303	104.9%	10.7%	-90%	25.6%
	Unintentional Injuries*	Both	54318	47786	101%	-24.2%	-109.8%	-33%
		Female	20797	16741	101.1%	-39.2%	-115.5%	-53.6%
		Male	33518	30978	100.9%	-15.2%	-105.9%	-20.1%
	Self-harm and interpersonal violence	Both	16413	19351	105%	36.7%	-98%	43.7%
		Female	4102	4579	105.1%	23.9%	-117.4%	11.6%
		Male	12306	14833	104.9%	42.1%	-92.6%	54.3%
**Ilam**	**All causes**	Both	241540	226756	32%	-8.1%	-45.6%	-21.7%
		Female	110271	102585	33.7%	-9.3%	-44.9%	-20.5%
		Male	131070	122710	30.4%	-8.3%	-44.8%	-22.7%
	**Communicable, maternal, neonatal, and nutritional diseases**	Both	93968	46596	32%	-66.5%	-49.8%	-84.3%
		Female	44685	22028	33.7%	-67.8%	-50.7%	-84.8%
		Male	49250	24545	30.4%	-65.4%	-49%	-84%
	HIV/AIDS and sexually transmitted infections	Both	177	149	32%	-20.5%	2%	13.5%
		Female	91	89	33.7%	-2.8%	-1.1%	29.8%
		Male	86	61	30.4%	-38.4%	4.7%	-3.3%
	Respiratory infections and tuberculosis	Both	9959	5789	32%	-55.3%	-51.4%	-74.7%
		Female	5414	2983	33.7%	-60%	-52.2%	-78.5%
		Male	4559	2794	30.4%	-50.5%	-50.2%	-70.3%
	Enteric infections	Both	7189	3803	32%	-62.2%	-44%	-74.2%
		Female	3478	1797	33.7%	-64.6%	-43.9%	-74.8%
		Male	3710	2000	30.4%	-60.1%	-43.9%	-73.6%
	Neglected tropical diseases and malaria	Both	1309	1463	32%	15.5%	-138.1%	-90.6%
		Female	639	702	33.7%	13.2%	-138.3%	-91.5%
		Male	670	765	30.4%	18.7%	-138.9%	-89.8%
	Other infectious diseases	Both	11920	6027	32%	-65.3%	-59.5%	-92.8%
		Female	6542	3146	33.7%	-69.4%	-58.8%	-94.5%
		Male	5395	2857	30.4%	-61.4%	-59.8%	-90.7%
	Maternal and neonatal disorders	Both	60118	27128	32%	-72.4%	-46.1%	-86.6%
		Female	26960	12178	33.7%	-73.3%	-46.8%	-86.4%
		Male	33096	14955	30.4%	-71.5%	-45.6%	-86.7%

Location	Cause	Sex	Expected DALYs in 2019		% 1990 - 2019 DALYs change cause			% 1990 - 2019 DALYs overall change
			Population growth	Population growth + Aging	Population growth	Age structure change	DALYs rate change	
	Nutritional deficiencies	Both	3296	2239	32%	-42.4%	-56.6%	-66.9%
		Female	1561	1135	33.7%	-36.5%	-59.6%	-62.5%
		Male	1734	1112	30.4%	-46.8%	-54.5%	-70.8%
	**Non-communicable diseases**	Both	105586	138909	32%	41.7%	-37.1%	36.5%
		Female	49988	67018	33.7%	45.5%	-33%	46.2%
		Male	55555	70707	30.4%	35.6%	-38%	28%
	Neoplasms	Both	8411	14275	32%	92%	-6.3%	117.8%
		Female	3424	6167	33.7%	107.1%	-1%	139.8%
		Male	4969	7821	30.4%	74.9%	-2.3%	103%
	Cardiovascular diseases	Both	22849	45996	32%	133.7%	-110.8%	54.9%
		Female	8549	18475	33.7%	155.2%	-117.1%	71.9%
		Male	14236	26604	30.4%	113.3%	-98.8%	44.9%
	Chronic respiratory diseases	Both	2430	3690	32%	68.5%	-38.8%	61.6%
		Female	1093	1705	33.7%	74.8%	-44.9%	63.6%
		Male	1334	1951	30.4%	60.3%	-30.6%	60.1%
	Digestive diseases	Both	2488	3581	32%	58%	-50.5%	39.5%
		Female	1158	1650	33.7%	56.8%	-51.8%	38.7%
		Male	1328	1899	30.4%	56%	-46.3%	40.1%
	Neurological disorders	Both	5590	7489	32%	44.8%	-1.7%	75.2%
		Female	2980	4087	33.7%	49.6%	1.2%	84.5%
		Male	2615	3419	30.4%	40.1%	-5.7%	64.8%
	Mental disorders	Both	11935	15409	32%	38.4%	3.7%	74.1%
		Female	6746	8974	33.7%	44.2%	3.4%	81.3%
		Male	5210	6485	30.4%	31.9%	2.8%	65.1%
	Substance use disorders	Both	872	1359	32%	73.7%	48.6%	154.3%
		Female	294	440	33.7%	66.7%	-6.6%	93.7%
		Male	575	915	30.4%	77.1%	76.9%	184.5%
	Diabetes and kidney diseases	Both	3677	5693	32%	72.4%	66.8%	171.2%
		Female	1575	2511	33.7%	79.4%	91.1%	204.2%
		Male	2096	3104	30.4%	62.7%	53.9%	147%
	Skin and subcutaneous diseases	Both	2569	2375	32%	-10%	1.1%	23.2%
		Female	1343	1237	33.7%	-10.6%	0.5%	23.6%
		Male	1228	1140	30.4%	-9.3%	1.6%	22.7%
	Sense organ diseases	Both	3024	5201	32%	95%	-57.7%	69.3%
		Female	1394	2546	33.7%	110.5%	-62%	82.2%
		Male	1628	2652	30.4%	82%	-53.9%	58.6%
	Musculoskeletal disorders	Both	6289	11714	32%	113.9%	11.5%	157.4%
		Female	3233	6290	33.7%	126.4%	16.4%	176.5%
		Male	3060	5483	30.4%	103.3%	4%	137.7%
	Other non-communicable diseases	Both	35453	22128	32%	-49.6%	-37.8%	-55.4%
		Female	18198	12935	33.7%	-38.7%	-36.9%	-41.9%
		Male	17274	9233	30.4%	-60.7%	-38.9%	-69.2%

Location	Cause	Sex	Expected DALYs in 2019		% 1990 - 2019 DALYs change cause			% 1990 - 2019 DALYs overall change
			Population growth	Population growth + Aging	Population growth	Age structure change	DALYs rate change	
	**Injuries***	Both	41595	40960	30.4%	-2%	-56.7%	-28.2%
		Female	15430	13499	31.7%	-16.5%	-65.9%	-50.7%
		Male	26073	27251	29.2%	5.8%	-50.3%	-15.2%
	Transport Injuries	Both	19648	18994	32%	-4.4%	-61.3%	-33.7%
		Female	6663	5178	33.7%	-29.8%	-64.2%	-60.3%
		Male	12913	13659	30.4%	7.5%	-58.2%	-20.2%
	Unintentional Injuries*	Both	13761	11868	30.4%	-17.9%	-56.2%	-43.7%
		Female	4958	3767	31.7%	-31.6%	-57%	-56.9%
		Male	8770	8041	29.2%	-10.8%	-54.8%	-36.3%
	Self-harm and interpersonal violence	Both	8360	10285	32%	30.4%	-50.8%	11.6%
		Female	3884	4581	33.7%	24%	-82.7%	-25%
		Male	4470	5680	30.4%	35.3%	-23.1%	42.7%
**Isfahan**	**All causes**	Both	1591980	1758595	37.1%	14.3%	-44.2%	7.2%
		Female	710658	788924	39.7%	15.4%	-40.7%	14.4%
		Male	878678	965171	34.6%	13.3%	-46.2%	1.6%
	**Communicable, maternal, neonatal, and nutritional diseases**	Both	447588	255984	37.1%	-58.7%	-50.7%	-72.3%
		Female	204899	117977	39.7%	-59.3%	-51.9%	-71.4%
		Male	242132	138177	34.6%	-57.8%	-49.7%	-72.9%
	HIV/AIDS and sexually transmitted infections	Both	1379	1237	37.1%	-14.2%	39.5%	62.4%
		Female	722	725	39.7%	0.6%	37.4%	77.7%
		Male	658	517	34.6%	-28.9%	40.5%	46.3%
	Respiratory infections and tuberculosis	Both	60763	43267	37.1%	-39.5%	-49.4%	-51.8%
		Female	28821	19431	39.7%	-45.5%	-49.7%	-55.5%
		Male	31903	23872	34.6%	-33.9%	-49.3%	-48.6%
	Enteric infections	Both	25872	16650	37.1%	-48.9%	-23.8%	-35.6%
		Female	12344	7906	39.7%	-50.2%	-23.4%	-33.9%
		Male	13514	8701	34.6%	-47.9%	-23.8%	-37.1%
	Neglected tropical diseases and malaria	Both	17379	18585	37.1%	9.5%	-140.2%	-93.6%
		Female	7413	8481	39.7%	20.1%	-152.3%	-92.4%
		Male	9925	10130	34.6%	2.8%	-131.8%	-94.4%
	Other infectious diseases	Both	39474	22721	37.1%	-58.2%	-60.5%	-81.6%
		Female	20640	11377	39.7%	-62.7%	-60.6%	-83.6%
		Male	18879	11309	34.6%	-54%	-60%	-79.4%
	Maternal and neonatal disorders	Both	283721	137309	37.1%	-70.7%	-46.4%	-80.1%
		Female	125056	61455	39.7%	-71.1%	-47.6%	-79%
		Male	158135	76089	34.6%	-69.8%	-45.7%	-80.9%
	Nutritional deficiencies	Both	19000	16213	37.1%	-20.1%	-58.7%	-41.8%
		Female	9902	8601	39.7%	-18.4%	-60.9%	-39.5%
		Male	9118	7558	34.6%	-23%	-55.7%	-44.1%
	**Non-communicable diseases**	Both	870454	1235604	37.1%	57.5%	-35.6%	59%
		Female	429359	601795	39.7%	56.1%	-31.8%	64%
		Male	441139	631954	34.6%	58.2%	-38.5%	54.3%

Location	Cause	Sex	Expected DALYs in 2019		% 1990 - 2019 DALYs change cause			% 1990 - 2019 DALYs overall change
			Population growth	Population growth + Aging	Population growth	Age structure change	DALYs rate change	
	Neoplasms	Both	69617	111649	37.1%	82.8%	11.6%	131.4%
		Female	31232	49163	39.7%	80.2%	12.4%	132.4%
		Male	38276	62954	34.6%	86.8%	9.2%	130.7%
	Cardiovascular diseases	Both	202469	422301	37.1%	148.8%	-123.7%	62.2%
		Female	85211	176353	39.7%	149.4%	-126.3%	62.8%
		Male	116733	241840	34.6%	144.3%	-117.1%	61.7%
	Chronic respiratory diseases	Both	29268	49211	37.1%	93.4%	-44.5%	86%
		Female	13028	21299	39.7%	88.7%	-50.7%	77.7%
		Male	16190	28121	34.6%	99.2%	-41.3%	92.5%
	Digestive diseases	Both	23568	36743	37.1%	76.6%	-54.6%	59.1%
		Female	10747	16224	39.7%	71.2%	-49.4%	61.5%
		Male	12790	20374	34.6%	79.8%	-57.2%	57.2%
	Neurological disorders	Both	54603	75011	37.1%	51.2%	-0.8%	87.5%
		Female	29939	40966	39.7%	51.5%	1.9%	93.1%
		Male	24777	34367	34.6%	52.1%	-5.7%	81%
	Mental disorders	Both	105819	127668	37.1%	28.3%	3.3%	68.7%
		Female	61028	75474	39.7%	33.1%	1.5%	74.3%
		Male	45119	52799	34.6%	22.9%	3.7%	61.3%
	Substance use disorders	Both	11482	15216	37.1%	44.6%	59.2%	140.9%
		Female	3110	4065	39.7%	42.9%	16.6%	99.2%
		Male	8280	10971	34.6%	43.8%	77.6%	156%
	Diabetes and kidney diseases	Both	28385	50735	37.1%	107.9%	128.7%	273.7%
		Female	13938	24588	39.7%	106.8%	132.9%	279.4%
		Male	14446	25983	34.6%	107.5%	126.2%	268.3%
	Skin and subcutaneous diseases	Both	22671	21213	37.1%	-8.8%	2%	30.2%
		Female	11884	11068	39.7%	-9.6%	1.3%	31.4%
		Male	10814	10165	34.6%	-8.1%	2.4%	29%
	Sense organ diseases	Both	29865	52332	37.1%	103.1%	-52.4%	87.8%
		Female	14870	25682	39.7%	101.6%	-52.9%	88.4%
		Male	15001	26623	34.6%	104.3%	-51.6%	87.3%
	Musculoskeletal disorders	Both	66459	112796	37.1%	95.6%	8.7%	141.3%
		Female	35349	60240	39.7%	98.4%	14.6%	152.7%
		Male	31207	52646	34.6%	92.5%	1.8%	128.9%
	Other non-communicable diseases	Both	226247	160730	37.1%	-39.7%	-34.6%	-37.2%
		Female	119023	96673	39.7%	-26.2%	-32.7%	-19.2%
		Male	107507	65110	34.6%	-53.1%	-37.9%	-56.4%
	**Injuries***	Both	267669	263211	36.6%	-2.3%	-60.3%	-26%
		Female	74207	68187	38.8%	-11.3%	-60.3%	-32.7%
		Male	191663	192543	34.5%	0.6%	-58.6%	-23.5%
	Transport Injuries	Both	150052	144638	37.1%	-4.9%	-70.7%	-38.5%
		Female	40269	35419	39.7%	-16.8%	-69.2%	-46.3%
		Male	108558	107648	34.6%	-1.1%	-69.2%	-35.8%

Location	Cause	Sex	Expected DALYs in 2019		% 1990 - 2019 DALYs change cause			% 1990 - 2019 DALYs overall change
			Population growth	Population growth + Aging	Population growth	Age structure change	DALYs rate change	
	Unintentional Injuries*	Both	90974	87883	36.6%	-4.6%	-51.4%	-19.5%
		Female	28233	25858	38.8%	-11.7%	-52.7%	-25.5%
		Male	62224	61342	34.5%	-1.9%	-49.4%	-16.8%
	Self-harm and interpersonal violence	Both	30219	33194	37.1%	13.5%	-41.5%	9.1%
		Female	6867	7541	39.7%	13.7%	-44.8%	8.6%
		Male	23060	25178	34.6%	12.4%	-37.7%	9.3%
**Kerman**	**All causes**	Both	1380985	1381542	78.3%	0.1%	-73.5%	4.9%
		Female	590673	590857	76%	0.1%	-66.5%	9.5%
		Male	792349	788580	80.5%	-0.9%	-78.4%	1.3%
	**Communicable, maternal, neonatal, and nutritional diseases**	Both	501074	317746	78.3%	-65.2%	-80.3%	-67.2%
		Female	228600	147023	76%	-62.8%	-81%	-67.8%
		Male	272845	170483	80.5%	-67.7%	-79.5%	-66.7%
	HIV/AIDS and sexually transmitted infections	Both	1088	1066	78.3%	-3.6%	457.9%	532.5%
		Female	515	521	76%	1.9%	412.2%	490%
		Male	573	545	80.5%	-9%	500.2%	571.7%
	Respiratory infections and tuberculosis	Both	82616	59512	78.3%	-49.9%	-87.7%	-59.2%
		Female	38019	26775	76%	-52%	-85.8%	-61.8%
		Male	44650	32711	80.5%	-48.3%	-89.2%	-56.9%
	Enteric infections	Both	43453	27697	78.3%	-64.6%	-74.2%	-60.5%
		Female	20095	12808	76%	-63.8%	-72.6%	-60.5%
		Male	23383	14884	80.5%	-65.6%	-75.5%	-60.6%
	Neglected tropical diseases and malaria	Both	12657	11822	78.3%	-11.8%	-156.7%	-90.1%
		Female	6242	5430	76%	-22.9%	-143.5%	-90.4%
		Male	6411	6378	80.5%	-0.9%	-169.4%	-89.8%
	Other infectious diseases	Both	44633	29170	78.3%	-61.8%	-97.6%	-81.1%
		Female	22800	14672	76%	-62.7%	-96.9%	-83.7%
		Male	21804	14457	80.5%	-60.8%	-98%	-78.3%
	Maternal and neonatal disorders	Both	299374	175054	78.3%	-74%	-75.1%	-70.8%
		Female	131566	79330	76%	-69.9%	-76.7%	-70.6%
		Male	168158	95610	80.5%	-77.9%	-73.7%	-71%
	Nutritional deficiencies	Both	17253	13423	78.3%	-39.6%	-83.2%	-44.5%
		Female	9362	7488	76%	-35.2%	-86.5%	-45.7%
		Male	7865	5898	80.5%	-45.1%	-78.3%	-42.9%
	**Non-communicable diseases**	Both	606492	786914	78.3%	53%	-60.7%	70.6%
		Female	276681	364238	76%	55.7%	-50%	81.7%
		Male	330260	418969	80.5%	48.5%	-67.9%	61.1%
	Neoplasms	Both	49791	69843	78.3%	71.8%	-33.6%	116.5%
		Female	20040	28668	76%	75.8%	-22%	129.7%
		Male	29856	40738	80.5%	65.8%	-38.9%	107.4%
	Cardiovascular diseases	Both	160503	266658	78.3%	117.9%	-151%	45.3%
		Female	59994	104951	76%	131.9%	-150.7%	57.2%
		Male	100970	159690	80.5%	105%	-147.5%	38%

Location	Cause	Sex	Expected DALYs in 2019		% 1990 - 2019 DALYs change cause			% 1990 - 2019 DALYs overall change
			Population growth	Population growth + Aging	Population growth	Age structure change	DALYs rate change	
	Chronic respiratory diseases	Both	28905	41692	78.3%	78.9%	-42%	115.1%
		Female	11612	17056	76%	82.5%	-43.5%	115%
		Male	17355	24484	80.5%	74.2%	-39.4%	115.2%
	Digestive diseases	Both	20003	26791	78.3%	60.5%	-83.4%	55.4%
		Female	8149	10750	76%	56.2%	-73.2%	59%
		Male	11894	15899	80.5%	60.8%	-88.5%	52.8%
	Neurological disorders	Both	33769	43332	78.3%	50.5%	-10.1%	118.7%
		Female	17495	22825	76%	53.6%	-1.6%	128%
		Male	16245	20422	80.5%	46.4%	-18.6%	108.4%
	Mental disorders	Both	69256	84435	78.3%	39.1%	0.3%	117.7%
		Female	38043	47014	76%	41.5%	0.5%	118%
		Male	31097	37138	80.5%	35.1%	1.7%	117.3%
	Substance use disorders	Both	8963	12627	78.3%	72.9%	5.4%	156.5%
		Female	2680	3632	76%	62.5%	-1.3%	137.2%
		Male	6326	9147	80.5%	80.5%	4%	165%
	Diabetes and kidney diseases	Both	22569	32004	78.3%	74.5%	24.9%	177.7%
		Female	10295	14858	76%	78%	44.1%	198.1%
		Male	12291	17031	80.5%	69.6%	10.1%	160.2%
	Skin and subcutaneous diseases	Both	14402	13556	78.3%	-10.5%	2.6%	70.4%
		Female	7435	6990	76%	-10.5%	2.2%	67.6%
		Male	6955	6553	80.5%	-10.4%	3.4%	73.5%
	Sense organ diseases	Both	18121	26994	78.3%	87.3%	-56.8%	108.8%
		Female	8660	13281	76%	93.9%	-58.2%	111.7%
		Male	9465	13702	80.5%	80.8%	-55.2%	106.1%
	Musculoskeletal disorders	Both	38123	59505	78.3%	100%	1%	179.3%
		Female	19908	31513	76%	102.6%	9.6%	188.2%
		Male	18178	27998	80.5%	97.5%	-8.9%	169.2%
	Other non-communicable diseases	Both	142087	109476	78.3%	-40.9%	-51.9%	-14.5%
		Female	72369	62700	76%	-23.5%	-47%	5.4%
		Male	69628	46166	80.5%	-60.8%	-55.5%	-35.8%
	**Injuries***	Both	266172	272184	77.1%	4%	-88.2%	-7.1%
		Female	82930	78526	74.5%	-9.3%	-80.6%	-15.3%
		Male	184567	195649	79.6%	10.8%	-93.6%	-3.3%
	Transport Injuries	Both	156263	157301	78.3%	1.2%	-103.7%	-24.2%
		Female	47231	43567	76%	-13.7%	-99.4%	-37.1%
		Male	109768	114700	80.5%	8.1%	-107.2%	-18.5%
	Unintentional Injuries*	Both	73316	71273	77.1%	-4.9%	-50.9%	21.2%
		Female	25878	23121	74.5%	-18.6%	-40.2%	15.8%
		Male	47716	48562	79.6%	3.2%	-58.5%	24.3%
	Self-harm and interpersonal violence	Both	42067	47749	78.3%	24.1%	-104.6%	-2.2%
		Female	11455	12711	76%	19.3%	-100%	-4.7%
		Male	30842	35543	80.5%	27.5%	-109.3%	-1.2%

Location	Cause	Sex	Expected DALYs in 2019		% 1990 - 2019 DALYs change cause			% 1990 - 2019 DALYs overall change
			Population growth	Population growth + Aging	Population growth	Age structure change	DALYs rate change	
**Kermanshah**	**All causes**	Both	923843	942981	16.3%	2.4%	-50.5%	-31.7%
		Female	408295	407136	18.7%	-0.3%	-47.4%	-28.9%
		Male	513693	527719	14.1%	3.1%	-51.1%	-33.9%
	**Communicable, maternal, neonatal, and nutritional diseases**	Both	354798	199247	16.3%	-51%	-48.3%	-83%
		Female	168544	93106	18.7%	-53.2%	-50%	-84.4%
		Male	186002	105932	14.1%	-49.1%	-46.7%	-81.7%
	HIV/AIDS and sexually transmitted infections	Both	911	941	16.3%	3.9%	823.7%	843.9%
		Female	383	366	18.7%	-5.4%	513%	526.3%
		Male	526	576	14.1%	11%	1041.3%	1066.3%
	Respiratory infections and tuberculosis	Both	52147	34562	16.3%	-39.2%	-55.2%	-78.1%
		Female	25940	16016	18.7%	-45.4%	-54.7%	-81.4%
		Male	26216	18410	14.1%	-34%	-55.1%	-75%
	Enteric infections	Both	18509	11462	16.3%	-44.3%	-41.1%	-69%
		Female	9034	5464	18.7%	-46.9%	-40.9%	-69.1%
		Male	9471	5977	14.1%	-42.1%	-41%	-69%
	Neglected tropical diseases and malaria	Both	4212	4545	16.3%	9.2%	-116.3%	-90.8%
		Female	1760	1956	18.7%	13.2%	-121.3%	-89.4%
		Male	2439	2565	14.1%	5.9%	-111.7%	-91.8%
	Other infectious diseases	Both	29772	17596	16.3%	-47.6%	-58.7%	-89.9%
		Female	15923	8896	18.7%	-52.4%	-58.1%	-91.7%
		Male	13898	8640	14.1%	-43.2%	-58.9%	-87.9%
	Maternal and neonatal disorders	Both	235585	120396	16.3%	-56.9%	-47.7%	-88.3%
		Female	108065	54993	18.7%	-58.3%	-48.6%	-88.2%
		Male	127202	65412	14.1%	-55.4%	-47%	-88.3%
	Nutritional deficiencies	Both	13662	9745	16.3%	-33.4%	-56.2%	-73.2%
		Female	7438	5416	18.7%	-32.3%	-58.9%	-72.4%
		Male	6251	4352	14.1%	-34.7%	-53.6%	-74.1%
	**Non-communicable diseases**	Both	407155	579632	16.3%	49.3%	-48.6%	17%
		Female	186620	265323	18.7%	50.1%	-41.1%	27.7%
		Male	219979	307566	14.1%	45.4%	-51.3%	8.3%
	Neoplasms	Both	35261	59197	16.3%	79%	-34.4%	60.9%
		Female	14143	23933	18.7%	82.2%	-23.9%	77.1%
		Male	20991	33823	14.1%	69.8%	-33.4%	50.5%
	Cardiovascular diseases	Both	105542	219361	16.3%	125.5%	-118.6%	23.2%
		Female	38308	86243	18.7%	148.6%	-121%	46.4%
		Male	66696	128329	14.1%	105.4%	-109.2%	10.4%
	Chronic respiratory diseases	Both	14442	25064	16.3%	85.6%	-75.1%	26.8%
		Female	5916	10568	18.7%	93.4%	-76.9%	35.2%
		Male	8478	14072	14.1%	75.3%	-68.1%	21.2%
	Digestive diseases	Both	12895	19695	16.3%	61.4%	-68.6%	9.1%
		Female	5363	8065	18.7%	59.8%	-64.4%	14.2%
		Male	7493	11326	14.1%	58.4%	-66.9%	5.6%

Location	Cause	Sex	Expected DALYs in 2019		% 1990 - 2019 DALYs change cause			% 1990 - 2019 DALYs overall change
			Population growth	Population growth + Aging	Population growth	Age structure change	DALYs rate change	
	Neurological disorders	Both	19701	27612	16.3%	46.7%	-4%	59%
		Female	10703	14960	18.7%	47.2%	-0.9%	65.1%
		Male	9036	12680	14.1%	46%	-8%	52.1%
	Mental disorders	Both	40389	49638	16.3%	26.6%	1.9%	44.9%
		Female	21576	27035	18.7%	30%	2.4%	51.2%
		Male	18878	22681	14.1%	23%	0.8%	37.9%
	Substance use disorders	Both	7662	10994	16.3%	50.6%	12.9%	79.8%
		Female	2192	3011	18.7%	44.4%	3.5%	66.6%
		Male	5408	7987	14.1%	54.4%	16.4%	85%
	Diabetes and kidney diseases	Both	12349	20777	16.3%	79.4%	39.2%	134.9%
		Female	5681	9804	18.7%	86.2%	62.6%	167.6%
		Male	6651	10816	14.1%	71.4%	22.6%	108.1%
	Skin and subcutaneous diseases	Both	8550	7993	16.3%	-7.6%	1%	9.8%
		Female	4479	4165	18.7%	-8.3%	0.6%	11.1%
		Male	4082	3832	14.1%	-7%	1.3%	8.4%
	Sense organ diseases	Both	10920	19582	16.3%	92.3%	-47.4%	61.2%
		Female	5157	9771	18.7%	106.2%	-51.9%	73.1%
		Male	5754	9809	14.1%	80.4%	-43.6%	50.9%
	Musculoskeletal disorders	Both	23657	41364	16.3%	87.1%	7.8%	111.2%
		Female	12469	22505	18.7%	95.6%	12.3%	126.6%
		Male	11219	19036	14.1%	79.5%	1.2%	94.8%
	Other non-communicable diseases	Both	115786	78357	16.3%	-37.6%	-37.5%	-58.8%
		Female	60634	45264	18.7%	-30.1%	-36.2%	-47.5%
		Male	55291	33175	14.1%	-45.6%	-39.2%	-70.7%
	**Injuries***	Both	159613	163290	15.6%	2.7%	-59.8%	-41.5%
		Female	51962	48458	17.9%	-7.9%	-61.3%	-51.4%
		Male	106677	113788	13.6%	7.6%	-58.1%	-36.9%
	Transport Injuries	Both	83516	84595	16.3%	1.5%	-64.9%	-47.1%
		Female	23244	20541	18.7%	-13.8%	-64.3%	-59.3%
		Male	59568	63259	14.1%	7.1%	-63.7%	-42.5%
	Unintentional Injuries*	Both	51036	49090	15.6%	-4.4%	-60.1%	-48.9%
		Female	18668	16482	17.9%	-13.8%	-58.1%	-54.1%
		Male	32131	32345	13.6%	0.8%	-60.3%	-45.9%
	Self-harm and interpersonal violence	Both	26041	30028	16.3%	17.8%	-44.1%	-9.9%
		Female	10609	11568	18.7%	10.7%	-60%	-30.5%
		Male	15345	18375	14.1%	22.5%	-32.9%	3.7%
**Khorasan-e-Razavi**	**All causes**	Both	3480894	3302333	41.4%	-7.3%	-66.8%	-32.7%
		Female	1572272	1491414	41.7%	-7.3%	-64.9%	-30.5%
		Male	1908033	1801246	41.1%	-7.9%	-67.7%	-34.5%
	**Communicable, maternal, neonatal, and nutritional diseases**	Both	1369873	874469	41.4%	-51.1%	-72.9%	-82.6%
		Female	650424	416004	41.7%	-51.1%	-73.7%	-83.1%
		Male	719344	458026	41.1%	-51.3%	-72.1%	-82.2%

Location	Cause	Sex	Expected DALYs in 2019		% 1990 - 2019 DALYs change cause			% 1990 - 2019 DALYs overall change
			Population growth	Population growth + Aging	Population growth	Age structure change	DALYs rate change	
	HIV/AIDS and sexually transmitted infections	Both	2040	1903	41.4%	-9.5%	68.1%	100%
		Female	1036	1042	41.7%	0.9%	91.9%	134.5%
		Male	1005	859	41.1%	-20.5%	43.9%	64.5%
	Respiratory infections and tuberculosis	Both	290059	201059	41.4%	-43.4%	-77.9%	-79.9%
		Female	150177	101077	41.7%	-46.3%	-78.1%	-82.8%
		Male	139909	99585	41.1%	-40.7%	-77.3%	-76.8%
	Enteric infections	Both	102990	66472	41.4%	-50.1%	-65.7%	-74.5%
		Female	47865	30733	41.7%	-50.7%	-64.9%	-73.9%
		Male	55114	35713	41.1%	-49.7%	-66.4%	-75%
	Neglected tropical diseases and malaria	Both	25100	26138	41.4%	5.8%	-139.9%	-92.7%
		Female	11123	12218	41.7%	13.9%	-147.4%	-91.8%
		Male	13972	13959	41.1%	-0.1%	-134.4%	-93.4%
	Other infectious diseases	Both	110854	71782	41.4%	-49.8%	-80.4%	-88.9%
		Female	58027	36762	41.7%	-51.9%	-80%	-90.2%
		Male	52840	34894	41.1%	-47.9%	-80.6%	-87.4%
	Maternal and neonatal disorders	Both	791242	471591	41.4%	-57.1%	-69.1%	-84.8%
		Female	355646	214074	41.7%	-56.4%	-69.9%	-84.7%
		Male	435455	257599	41.1%	-57.6%	-68.4%	-84.9%
	Nutritional deficiencies	Both	47587	35525	41.4%	-35.8%	-74.6%	-69%
		Female	26550	20099	41.7%	-34.4%	-77.7%	-70.4%
		Male	21049	15418	41.1%	-37.8%	-70.6%	-67.3%
	**Non-communicable diseases**	Both	1452992	1812685	41.4%	35%	-52.6%	23.8%
		Female	690078	878099	41.7%	38.6%	-49.9%	30.4%
		Male	762803	925629	41.1%	30.1%	-53.3%	17.9%
	Neoplasms	Both	138401	191935	41.4%	54.7%	-34.3%	61.7%
		Female	58153	81162	41.7%	56.1%	-28.6%	69.1%
		Male	80207	108500	41.1%	49.8%	-34.5%	56.4%
	Cardiovascular diseases	Both	339108	592759	41.4%	105.8%	-123.5%	23.6%
		Female	136810	257707	41.7%	125.2%	-133.8%	33.1%
		Male	202175	329727	41.1%	89%	-112.9%	17.3%
	Chronic respiratory diseases	Both	55477	82376	41.4%	68.6%	-55.2%	54.7%
		Female	24896	38491	41.7%	77.4%	-63.9%	55.1%
		Male	30571	43392	41.1%	59.2%	-45.9%	54.4%
	Digestive diseases	Both	44990	58348	41.4%	42%	-61.3%	22.1%
		Female	20259	26215	41.7%	41.6%	-61.3%	22%
		Male	24723	31743	41.1%	40.1%	-59%	22.1%
	Neurological disorders	Both	71892	91418	41.4%	38.4%	-11.3%	68.5%
		Female	38746	50062	41.7%	41.4%	-6.9%	76.2%
		Male	33159	41292	41.1%	34.6%	-16.1%	59.6%
	Mental disorders	Both	124636	149002	41.4%	27.6%	0.2%	69.3%
		Female	68726	83750	41.7%	31%	0.5%	73.2%
		Male	55939	65202	41.1%	23.4%	0%	64.5%

Location	Cause	Sex	Expected DALYs in 2019		% 1990 - 2019 DALYs change cause			% 1990 - 2019 DALYs overall change
			Population growth	Population growth + Aging	Population growth	Age structure change	DALYs rate change	
	Substance use disorders	Both	18023	25601	41.4%	59.4%	41.7%	142.5%
		Female	5955	8081	41.7%	50.6%	-11.9%	80.3%
		Male	12056	17598	41.1%	64.9%	67.1%	173.1%
	Diabetes and kidney diseases	Both	53470	76517	41.4%	60.9%	40.4%	142.7%
		Female	24659	36432	41.7%	67.6%	44.1%	153.4%
		Male	28804	39827	41.1%	54%	38.4%	133.5%
	Skin and subcutaneous diseases	Both	29063	27468	41.4%	-7.8%	2.3%	35.9%
		Female	15288	14413	41.7%	-8.1%	1.9%	35.5%
		Male	13779	13049	41.1%	-7.5%	2.8%	36.4%
	Sense organ diseases	Both	44870	68838	41.4%	75.5%	-34.9%	82%
		Female	21821	35019	41.7%	85.7%	-36%	91.4%
		Male	23048	33844	41.1%	66.1%	-33.9%	73.3%
	Musculoskeletal disorders	Both	80484	126646	41.4%	81.1%	7.4%	129.8%
		Female	42526	68280	41.7%	85.8%	12.5%	140%
		Male	37968	58564	41.1%	76.5%	0.8%	118.5%
	Other non-communicable diseases	Both	452579	321776	41.4%	-40.9%	-55.6%	-55.1%
		Female	232239	178489	41.7%	-32.8%	-52.6%	-43.7%
		Male	220374	142892	41.1%	-49.6%	-58.5%	-67%
	**Injuries***	Both	641687	607875	41.3%	-7.4%	-86.1%	-52.3%
		Female	224016	194849	41.2%	-18.4%	-85.9%	-63.1%
		Male	417867	413509	41.5%	-1.5%	-86.4%	-46.4%
	Transport Injuries	Both	385312	361853	41.4%	-8.6%	-91.7%	-59%
		Female	131561	111121	41.7%	-22%	-88.7%	-69%
		Male	253517	250360	41.1%	-1.8%	-93.1%	-53.8%
	Unintentional Injuries*	Both	217934	195078	41.3%	-14.8%	-83%	-56.5%
		Female	80156	67077	41.2%	-23%	-81.6%	-63.5%
		Male	137835	128174	41.5%	-9.9%	-84%	-52.4%
	Self-harm and interpersonal violence	Both	47640	55530	41.4%	23.4%	-52.7%	12.1%
		Female	17025	18316	41.7%	10.7%	-77.7%	-25.3%
		Male	30589	37306	41.1%	31%	-39.3%	32.8%
**Khuzestan**	**All causes**	Both	1827974	1846370	52.2%	1.5%	-55.8%	-2%
		Female	799252	816790	53.2%	3.4%	-51.8%	4.8%
		Male	1027450	1026381	51.3%	-0.2%	-58.4%	-7.3%
	**Communicable, maternal, neonatal, and nutritional diseases**	Both	618406	392048	52.2%	-55.7%	-67.1%	-70.6%
		Female	276117	176072	53.2%	-55.5%	-67.7%	-70%
		Male	341931	216112	51.3%	-55.7%	-66.7%	-71.1%
	HIV/AIDS and sexually transmitted infections	Both	1605	1535	52.2%	-6.6%	260.2%	305.8%
		Female	786	798	53.2%	2.3%	185.8%	241.3%
		Male	819	738	51.3%	-14.9%	330.6%	366.9%
	Respiratory infections and tuberculosis	Both	123618	87390	52.2%	-44.6%	-74.4%	-66.8%
		Female	54530	37507	53.2%	-47.8%	-73.2%	-67.9%
		Male	69008	49948	51.3%	-41.8%	-75.4%	-65.9%

Location	Cause	Sex	Expected DALYs in 2019		% 1990 - 2019 DALYs change cause			% 1990 - 2019 DALYs overall change
			Population growth	Population growth + Aging	Population growth	Age structure change	DALYs rate change	
	Enteric infections	Both	40796	27099	52.2%	-51.1%	-53.8%	-52.7%
		Female	18886	12521	53.2%	-51.6%	-52.1%	-50.5%
		Male	21895	14562	51.3%	-50.7%	-55.2%	-54.6%
	Neglected tropical diseases and malaria	Both	8284	8640	52.2%	6.5%	-141.1%	-82.4%
		Female	3923	4265	53.2%	13.4%	-148.8%	-82.2%
		Male	4359	4375	51.3%	0.6%	-134.4%	-82.5%
	Other infectious diseases	Both	71804	46002	52.2%	-54.7%	-82.9%	-85.3%
		Female	36669	22882	53.2%	-57.6%	-82.7%	-87.1%
		Male	35152	23062	51.3%	-52%	-82.8%	-83.5%
	Maternal and neonatal disorders	Both	345485	200596	52.2%	-63.8%	-62.2%	-73.8%
		Female	147481	87012	53.2%	-62.8%	-62.4%	-72%
		Male	197718	113748	51.3%	-64.2%	-62.2%	-75.1%
	Nutritional deficiencies	Both	26813	20784	52.2%	-34.2%	-71.7%	-53.7%
		Female	13842	11088	53.2%	-30.5%	-74.9%	-52.1%
		Male	12980	9679	51.3%	-38.5%	-68.2%	-55.4%
	**Non-communicable diseases**	Both	880867	1125134	52.2%	42.2%	-40.8%	53.6%
		Female	422223	544907	53.2%	44.5%	-35.9%	61.8%
		Male	458502	577501	51.3%	39.3%	-44.3%	46.2%
	Neoplasms	Both	55668	84150	52.2%	77.9%	6.3%	136.4%
		Female	24394	37637	53.2%	83.2%	14.4%	150.7%
		Male	31236	46196	51.3%	72.4%	1.7%	125.4%
	Cardiovascular diseases	Both	211808	380465	52.2%	121.2%	-109.6%	63.8%
		Female	88677	160439	53.2%	124%	-109%	68.2%
		Male	122933	217356	51.3%	116.2%	-106.8%	60.7%
	Chronic respiratory diseases	Both	25101	37209	52.2%	73.4%	-64.5%	61.2%
		Female	11019	16380	53.2%	74.5%	-67.2%	60.6%
		Male	14065	20753	51.3%	71.9%	-61.6%	61.6%
	Digestive diseases	Both	25780	35612	52.2%	58%	-72.1%	38.1%
		Female	11584	15783	53.2%	55.5%	-71.1%	37.6%
		Male	14182	19669	51.3%	58.5%	-71.2%	38.5%
	Neurological disorders	Both	47513	61163	52.2%	43.7%	-6.4%	89.6%
		Female	25891	33593	53.2%	45.6%	-2.3%	96.5%
		Male	21654	27714	51.3%	42.3%	-12.1%	81.4%
	Mental disorders	Both	94925	116574	52.2%	34.7%	4.8%	91.8%
		Female	52255	65445	53.2%	38.7%	4.1%	96%
		Male	42741	51230	51.3%	30%	5.4%	86.7%
	Substance use disorders	Both	11030	15745	52.2%	65.1%	74.5%	191.7%
		Female	3471	4823	53.2%	59.6%	7.4%	120.3%
		Male	7534	10910	51.3%	67.8%	105.2%	224.3%
	Diabetes and kidney diseases	Both	30758	49114	52.2%	90.8%	133.8%	276.8%
		Female	14955	24457	53.2%	97.3%	142.5%	293%
		Male	15801	24640	51.3%	84.6%	125.8%	261.6%

Location	Cause	Sex	Expected DALYs in 2019		% 1990 - 2019 DALYs change cause			% 1990 - 2019 DALYs overall change
			Population growth	Population growth + Aging	Population growth	Age structure change	DALYs rate change	
	Skin and subcutaneous diseases	Both	21211	19932	52.2%	-9.2%	3.8%	46.8%
		Female	11131	10432	53.2%	-9.6%	3%	46.6%
		Male	10089	9506	51.3%	-8.7%	4.5%	47%
	Sense organ diseases	Both	24095	37559	52.2%	85.1%	-51.3%	86%
		Female	11923	18678	53.2%	86.8%	-52.2%	87.8%
		Male	12173	18869	51.3%	83.2%	-50.2%	84.2%
	Musculoskeletal disorders	Both	51558	85039	52.2%	98.8%	14.6%	165.7%
		Female	27501	45909	53.2%	102.5%	19.4%	175.2%
		Male	24084	39240	51.3%	95.2%	8.5%	155%
	Other non-communicable diseases	Both	281420	202571	52.2%	-42.6%	-50.7%	-41.2%
		Female	139422	111332	53.2%	-30.9%	-46.8%	-24.5%
		Male	142011	91416	51.3%	-53.9%	-54.7%	-57.3%
	**Injuries***	Both	317879	320563	49%	1.3%	-71.5%	-21.3%
		Female	98707	94502	49.7%	-6.4%	-72.7%	-29.4%
		Male	218649	225698	48.3%	4.8%	-70.7%	-17.6%
	Transport Injuries	Both	131303	133748	52.2%	2.8%	-72.2%	-17.2%
		Female	34975	32799	53.2%	-9.5%	-73.4%	-29.8%
		Male	95951	100634	51.3%	7.4%	-71.3%	-12.7%
	Unintentional Injuries*	Both	122888	113720	49%	-11.1%	-68.3%	-30.4%
		Female	41817	36667	49.7%	-18.4%	-69.8%	-38.6%
		Male	80902	76926	48.3%	-7.3%	-67.2%	-26.2%
	Self-harm and interpersonal violence	Both	71506	80270	52.2%	18.7%	-87.4%	-16.6%
		Female	23034	26025	53.2%	19.9%	-84.4%	-11.3%
		Male	48318	54146	51.3%	18.2%	-88.6%	-19.1%
**Kohgiluyeh and Boyer-Ahmad**	**All causes**	Both	318625	293071	51.9%	-12.2%	-61.4%	-21.7%
		Female	142914	130680	51.9%	-13%	-59%	-20.2%
		Male	175725	161696	52%	-12.1%	-62.8%	-22.9%
	**Communicable, maternal, neonatal, and nutritional diseases**	Both	111754	65630	51.9%	-62.7%	-68.5%	-79.2%
		Female	50998	30413	51.9%	-61.3%	-69%	-78.5%
		Male	60760	35191	52%	-64%	-67.9%	-79.8%
	HIV/AIDS and sexually transmitted infections	Both	279	236	51.9%	-23.2%	77.1%	105.9%
		Female	139	131	51.9%	-8.9%	41.6%	84.6%
		Male	140	106	52%	-36.9%	111.9%	127%
	Respiratory infections and tuberculosis	Both	17860	11115	51.9%	-57.4%	-73.7%	-79.2%
		Female	8979	5434	51.9%	-59.9%	-73.4%	-81.5%
		Male	8881	5668	52%	-55%	-73.8%	-76.8%
	Enteric infections	Both	8139	5036	51.9%	-57.9%	-59%	-65%
		Female	3940	2407	51.9%	-59.1%	-58.4%	-65.6%
		Male	4199	2622	52%	-57.1%	-59.3%	-64.4%
	Neglected tropical diseases and malaria	Both	2518	2163	51.9%	-21.4%	-121.8%	-91.3%
		Female	1091	996	51.9%	-13.2%	-128.7%	-90%
		Male	1427	1174	52%	-26.9%	-117.3%	-92.3%

Location	Cause	Sex	Expected DALYs in 2019		% 1990 - 2019 DALYs change cause			% 1990 - 2019 DALYs overall change
			Population growth	Population growth + Aging	Population growth	Age structure change	DALYs rate change	
	Other infectious diseases	Both	15784	9344	51.9%	-62%	-79%	-89%
		Female	8175	4767	51.9%	-63.3%	-79.3%	-90.7%
		Male	7608	4565	52%	-60.8%	-78.4%	-87.2%
	Maternal and neonatal disorders	Both	63556	34985	51.9%	-68.3%	-64.1%	-80.4%
		Female	26872	15243	51.9%	-65.7%	-63.9%	-77.8%
		Male	36688	19744	52%	-70.2%	-64.2%	-82.4%
	Nutritional deficiencies	Both	3618	2752	51.9%	-36.4%	-69.1%	-53.5%
		Female	1802	1434	51.9%	-31%	-72.6%	-51.8%
		Male	1817	1312	52%	-42.2%	-65%	-55.2%
	**Non-communicable diseases**	Both	137335	163423	51.9%	28.9%	-49.1%	31.7%
		Female	66479	79218	51.9%	29.1%	-45%	36%
		Male	70856	83507	52%	27.1%	-51.5%	27.7%
	Neoplasms	Both	10597	14726	51.9%	59.2%	-27.6%	83.6%
		Female	4437	6104	51.9%	57%	-22.1%	86.8%
		Male	6161	8589	52%	59.9%	-30.7%	81.2%
	Cardiovascular diseases	Both	27300	48400	51.9%	117.4%	-112.9%	56.5%
		Female	11609	20239	51.9%	112.9%	-109.2%	55.6%
		Male	15693	27444	52%	113.8%	-108.7%	57.2%
	Chronic respiratory diseases	Both	3315	4746	51.9%	65.6%	-66.6%	50.9%
		Female	1452	2032	51.9%	60.6%	-63.8%	48.6%
		Male	1863	2708	52%	69%	-68.3%	52.7%
	Digestive diseases	Both	4219	5186	51.9%	34.8%	-79.8%	7%
		Female	1925	2269	51.9%	27.2%	-72.5%	6.6%
		Male	2294	2871	52%	38.2%	-82.8%	7.4%
	Neurological disorders	Both	7192	9404	51.9%	46.7%	-6.9%	91.8%
		Female	3779	4992	51.9%	48.8%	-2.4%	98.2%
		Male	3414	4434	52%	45.4%	-12.8%	84.6%
	Mental disorders	Both	14360	18127	51.9%	39.9%	4.1%	95.9%
		Female	7807	10123	51.9%	45%	3.6%	100.5%
		Male	6552	8015	52%	34%	4.4%	90.3%
	Substance use disorders	Both	1198	1788	51.9%	74.9%	86.7%	213.6%
		Female	426	620	51.9%	69.3%	26.4%	147.6%
		Male	772	1169	52%	78.2%	119.8%	250%
	Diabetes and kidney diseases	Both	3457	4988	51.9%	67.3%	29.8%	149%
		Female	1658	2387	51.9%	66.7%	33.9%	152.5%
		Male	1799	2583	52%	66.3%	27.5%	145.8%
	Skin and subcutaneous diseases	Both	3221	2998	51.9%	-10.5%	2.5%	44%
		Female	1681	1557	51.9%	-11.2%	2.4%	43%
		Male	1540	1441	52%	-9.8%	2.7%	45%
	Sense organ diseases	Both	3577	5820	51.9%	95.3%	-59.5%	87.7%
		Female	1711	2766	51.9%	93.7%	-59.5%	86.1%
		Male	1866	3046	52%	96.1%	-58.9%	89.2%

Location	Cause	Sex	Expected DALYs in 2019		% 1990 - 2019 DALYs change cause			% 1990 - 2019 DALYs overall change
			Population growth	Population growth + Aging	Population growth	Age structure change	DALYs rate change	
	Musculoskeletal disorders	Both	7395	12734	51.9%	109.7%	17.9%	179.5%
		Female	3827	6781	51.9%	117.2%	23.1%	192.2%
		Male	3568	5993	52%	103.4%	10.6%	166%
	Other non-communicable diseases	Both	51505	34507	51.9%	-50.1%	-57.3%	-55.5%
		Female	26168	19348	51.9%	-39.6%	-56.1%	-43.8%
		Male	25336	15216	52%	-60.7%	-58.9%	-67.6%
	**Injuries***	Both	67953	62791	49.6%	-11.4%	-72.3%	-34.1%
		Female	24996	20882	48.9%	-24.5%	-74.5%	-50.1%
		Male	43026	42051	50.2%	-3.4%	-71.6%	-24.8%
	Transport Injuries	Both	32700	29578	51.9%	-14.5%	-73.9%	-36.4%
		Female	10888	8644	51.9%	-31.3%	-76%	-55.4%
		Male	21818	20968	52%	-5.9%	-73%	-26.9%
	Unintentional Injuries*	Both	24376	20567	49.6%	-23.4%	-70.5%	-44.3%
		Female	9879	7455	48.9%	-36.6%	-71.7%	-59.3%
		Male	14514	13118	50.2%	-14.4%	-69.8%	-34%
	Self-harm and interpersonal violence	Both	11989	13657	51.9%	21.1%	-82.2%	-9.1%
		Female	4434	4899	51.9%	15.9%	-82.8%	-15%
		Male	7557	8780	52%	24.6%	-82.3%	-5.7%
**Kurdistan**	**All causes**	Both	914296	820547	33.8%	-13.7%	-61.3%	-41.2%
		Female	411837	361817	34.2%	-16.3%	-58.7%	-40.8%
		Male	502264	454814	33.5%	-12.6%	-62.4%	-41.5%
	**Communicable, maternal, neonatal, and nutritional diseases**	Both	376467	208097	33.8%	-59.9%	-60.6%	-86.6%
		Female	175661	97478	34.2%	-59.7%	-61.1%	-86.6%
		Male	200757	110525	33.5%	-60%	-60.1%	-86.6%
	HIV/AIDS and sexually transmitted infections	Both	544	484	33.8%	-14.6%	62.4%	81.6%
		Female	269	259	34.2%	-4.6%	44.5%	74.1%
		Male	275	224	33.5%	-24.9%	80.2%	88.8%
	Respiratory infections and tuberculosis	Both	51556	31495	33.8%	-52.1%	-66.5%	-84.8%
		Female	26066	15460	34.2%	-54.6%	-66.6%	-87%
		Male	25494	15982	33.5%	-49.8%	-66.2%	-82.5%
	Enteric infections	Both	19269	11705	33.8%	-52.5%	-52.5%	-71.2%
		Female	9997	6004	34.2%	-53.6%	-54.2%	-73.6%
		Male	9275	5690	33.5%	-51.6%	-50.5%	-68.6%
	Neglected tropical diseases and malaria	Both	2120	1890	33.8%	-14.5%	-101.7%	-82.4%
		Female	1046	938	34.2%	-13.8%	-103%	-82.6%
		Male	1074	945	33.5%	-16%	-99.7%	-82.2%
	Other infectious diseases	Both	35192	19681	33.8%	-59%	-67.9%	-93%
		Female	18808	10279	34.2%	-60.9%	-67.5%	-94.2%
		Male	16392	9372	33.5%	-57.2%	-68%	-91.7%
	Maternal and neonatal disorders	Both	256909	135140	33.8%	-63.4%	-58.8%	-88.4%
		Female	113418	60152	34.2%	-63%	-58.9%	-87.7%
		Male	143424	74981	33.5%	-63.7%	-58.8%	-89%

Location	Cause	Sex	Expected DALYs in 2019		% 1990 - 2019 DALYs change cause			% 1990 - 2019 DALYs overall change
			Population growth	Population growth + Aging	Population growth	Age structure change	DALYs rate change	
	Nutritional deficiencies	Both	10877	7703	33.8%	-39.1%	-63.6%	-68.8%
		Female	6058	4385	34.2%	-37.1%	-66.8%	-69.7%
		Male	4823	3330	33.5%	-41.3%	-59.9%	-67.7%
	**Non-communicable diseases**	Both	368105	458411	33.8%	32.8%	-55.4%	11.3%
		Female	171701	214921	34.2%	33.8%	-50.1%	17.9%
		Male	196357	239436	33.5%	29.3%	-57.2%	5.6%
	Neoplasms	Both	36094	55795	33.8%	73%	-54.8%	52%
		Female	14386	21867	34.2%	69.8%	-49%	54.9%
		Male	21691	32623	33.5%	67.3%	-50.6%	50.1%
	Cardiovascular diseases	Both	82574	150897	33.8%	110.7%	-123.3%	21.3%
		Female	31958	61692	34.2%	124.9%	-128.2%	30.8%
		Male	50569	87020	33.5%	96.2%	-114.4%	15.3%
	Chronic respiratory diseases	Both	11322	17141	33.8%	68.8%	-73.2%	29.5%
		Female	4885	7334	34.2%	67.3%	-71.2%	30.2%
		Male	6433	9587	33.5%	65.5%	-70.1%	28.9%
	Digestive diseases	Both	10977	14244	33.8%	39.8%	-68.2%	5.4%
		Female	5130	6385	34.2%	32.8%	-65.6%	1.4%
		Male	5845	7738	33.5%	43.2%	-67.7%	8.9%
	Neurological disorders	Both	19198	24418	33.8%	36.4%	-14.1%	56.1%
		Female	9954	12952	34.2%	40.4%	-9.1%	65.5%
		Male	9247	11428	33.5%	31.5%	-18.9%	46%
	Mental disorders	Both	32079	39427	33.8%	30.7%	-0.2%	64.3%
		Female	17648	22165	34.2%	34.3%	-0.3%	68.2%
		Male	14441	17232	33.5%	25.8%	0.2%	59.4%
	Substance use disorders	Both	3014	4464	33.8%	64.4%	35.2%	133.5%
		Female	980	1383	34.2%	55.2%	-8.2%	81.2%
		Male	2032	3107	33.5%	70.7%	54.4%	158.5%
	Diabetes and kidney diseases	Both	11170	16280	33.8%	61.2%	22.7%	117.7%
		Female	5220	7733	34.2%	64.6%	34.9%	133.7%
		Male	5948	8473	33.5%	56.7%	13.7%	103.8%
	Skin and subcutaneous diseases	Both	7254	6778	33.8%	-8.8%	1.1%	26.1%
		Female	3780	3525	34.2%	-9.1%	0.7%	25.9%
		Male	3475	3252	33.5%	-8.6%	1.5%	26.4%
	Sense organ diseases	Both	8935	14880	33.8%	89%	-48.6%	74.3%
		Female	4181	7345	34.2%	101.5%	-52.2%	83.5%
		Male	4752	7532	33.5%	78.1%	-45.3%	66.2%
	Musculoskeletal disorders	Both	19333	32539	33.8%	91.4%	11%	136.2%
		Female	10224	17659	34.2%	97.6%	14.9%	146.7%
		Male	9113	14962	33.5%	85.7%	5.4%	124.5%
	Other non-communicable diseases	Both	126156	81549	33.8%	-47.3%	-51.6%	-65.1%
		Female	63353	44881	34.2%	-39.1%	-49.4%	-54.3%
		Male	62811	36481	33.5%	-56%	-53.4%	-75.8%

Location	Cause	Sex	Expected DALYs in 2019		% 1990 - 2019 DALYs change cause			% 1990 - 2019 DALYs overall change
			Population growth	Population growth + Aging	Population growth	Age structure change	DALYs rate change	
	**Injuries***	Both	158769	147210	32.6%	-9.7%	-74.5%	-51.6%
		Female	59463	47020	32.6%	-27.7%	-75%	-70.1%
		Male	99295	100549	32.5%	1.7%	-74.7%	-40.5%
	Transport Injuries	Both	92558	83570	33.8%	-13%	-76.5%	-55.7%
		Female	35405	25980	34.2%	-35.7%	-75.2%	-76.8%
		Male	57099	57651	33.5%	1.3%	-77.5%	-42.7%
	Unintentional Injuries*	Both	52496	44143	32.6%	-21.1%	-71.5%	-60.1%
		Female	20717	15692	32.6%	-32.2%	-72.1%	-71.6%
		Male	31776	28463	32.5%	-13.8%	-71.2%	-52.5%
	Self-harm and interpersonal violence	Both	20931	24500	33.8%	22.8%	-83.3%	-26.6%
		Female	6500	6982	34.2%	10%	-85.6%	-41.5%
		Male	14411	17706	33.5%	30.5%	-83.9%	-19.9%
**Lorestan**	**All causes**	Both	717099	730520	14.3%	2.1%	-49.7%	-33.2%
		Female	315131	321171	15.5%	2.2%	-50.2%	-32.4%
		Male	401191	404611	13.2%	1%	-47.9%	-33.8%
	**Communicable, maternal, neonatal, and nutritional diseases**	Both	270703	157987	14.3%	-47.6%	-52.5%	-85.7%
		Female	123963	71853	15.5%	-48.6%	-53.1%	-86.2%
		Male	146550	85976	13.2%	-46.8%	-51.8%	-85.4%
	HIV/AIDS and sexually transmitted infections	Both	662	619	14.3%	-7.3%	535.1%	542.1%
		Female	314	297	15.5%	-6.1%	419.2%	428.6%
		Male	348	323	13.2%	-8%	637.2%	642.4%
	Respiratory infections and tuberculosis	Both	45176	29487	14.3%	-39.7%	-57.6%	-83%
		Female	21335	13289	15.5%	-43.6%	-57.6%	-85.6%
		Male	23822	16117	13.2%	-36.6%	-57.3%	-80.7%
	Enteric infections	Both	13541	8814	14.3%	-39.9%	-38.3%	-63.9%
		Female	5866	3844	15.5%	-39.8%	-36.3%	-60.6%
		Male	7658	4964	13.2%	-39.8%	-39.8%	-66.4%
	Neglected tropical diseases and malaria	Both	3150	3227	14.3%	2.8%	-107%	-89.9%
		Female	1500	1454	15.5%	-3.5%	-101.3%	-89.3%
		Male	1649	1757	13.2%	7.4%	-111%	-90.4%
	Other infectious diseases	Both	33354	20367	14.3%	-44.5%	-61.5%	-91.7%
		Female	16869	9914	15.5%	-47.6%	-61.5%	-93.6%
		Male	16494	10389	13.2%	-41.9%	-61.1%	-89.8%
	Maternal and neonatal disorders	Both	156339	82905	14.3%	-53.7%	-50.8%	-90.2%
		Female	69113	36797	15.5%	-54%	-51.3%	-89.7%
		Male	87065	46104	13.2%	-53.2%	-50.5%	-90.5%
	Nutritional deficiencies	Both	18481	12566	14.3%	-36.6%	-59.5%	-81.8%
		Female	8965	6257	15.5%	-34.9%	-60.9%	-80.3%
		Male	9513	6323	13.2%	-37.9%	-58.4%	-83.2%
	**Non-communicable diseases**	Both	312453	438010	14.3%	45.9%	-39.9%	20.3%
		Female	146112	207487	15.5%	48.5%	-42.5%	21.6%
		Male	166182	226260	13.2%	40.9%	-34.8%	19.3%

Location	Cause	Sex	Expected DALYs in 2019		% 1990 - 2019 DALYs change cause			% 1990 - 2019 DALYs overall change
			Population growth	Population growth + Aging	Population growth	Age structure change	DALYs rate change	
	Neoplasms	Both	26986	45258	14.3%	77.4%	-25.3%	66.4%
		Female	10907	19058	15.5%	86.3%	-39.9%	62%
		Male	16031	25292	13.2%	65.4%	-9.2%	69.3%
	Cardiovascular diseases	Both	78932	157420	14.3%	113.7%	-92.1%	35.9%
		Female	29503	64092	15.5%	135.4%	-118.2%	32.7%
		Male	49237	90330	13.2%	94.4%	-69.8%	37.8%
	Chronic respiratory diseases	Both	8750	13901	14.3%	67.3%	-50.4%	31.2%
		Female	3790	6206	15.5%	73.6%	-60.3%	28.8%
		Male	4949	7537	13.2%	59.2%	-39.3%	33.1%
	Digestive diseases	Both	10512	14982	14.3%	48.6%	-68.9%	-6%
		Female	4399	6221	15.5%	47.9%	-70.3%	-6.9%
		Male	6098	8532	13.2%	45.2%	-63.7%	-5.4%
	Neurological disorders	Both	17327	23826	14.3%	42.9%	-8%	49.2%
		Female	9353	12993	15.5%	45%	-4.4%	56.1%
		Male	7991	10845	13.2%	40.4%	-12.3%	41.3%
	Mental disorders	Both	31986	39123	14.3%	25.5%	2.3%	42.1%
		Female	17415	21791	15.5%	29%	2.4%	47%
		Male	14605	17346	13.2%	21.2%	1.9%	36.3%
	Substance use disorders	Both	5155	7424	14.3%	50.3%	61.1%	125.7%
		Female	1466	2047	15.5%	45.8%	39%	100.3%
		Male	3667	5428	13.2%	54.3%	68.2%	135.7%
	Diabetes and kidney diseases	Both	9776	16525	14.3%	78.9%	17.8%	111%
		Female	4316	7768	15.5%	92.4%	17.7%	125.6%
		Male	5449	8657	13.2%	66.6%	20%	99.7%
	Skin and subcutaneous diseases	Both	7657	7133	14.3%	-7.8%	1.5%	8%
		Female	4014	3730	15.5%	-8.2%	1.1%	8.5%
		Male	3648	3403	13.2%	-7.6%	1.9%	7.5%
	Sense organ diseases	Both	9235	16135	14.3%	85.4%	-45.1%	54.6%
		Female	4385	8144	15.5%	99%	-48.5%	66.1%
		Male	4846	7988	13.2%	73.4%	-42.1%	44.4%
	Musculoskeletal disorders	Both	19458	33884	14.3%	84.8%	10.8%	109.8%
		Female	10222	18447	15.5%	93%	14.3%	122.8%
		Male	9249	15582	13.2%	77.5%	5.2%	95.8%
	Other non-communicable diseases	Both	86680	62400	14.3%	-32%	-41.3%	-59%
		Female	46343	36990	15.5%	-23.3%	-39.3%	-47%
		Male	40412	25319	13.2%	-42.3%	-43.4%	-72.5%
	**Injuries***	Both	151291	151911	14.2%	0.5%	-72.1%	-57.5%
		Female	52177	48687	15.1%	-7.7%	-72.7%	-65.3%
		Male	98768	103101	13.3%	5%	-71.7%	-53.4%
	Transport Injuries	Both	69086	67671	14.3%	-2.3%	-67.4%	-55.4%
		Female	22117	18954	15.5%	-16.5%	-67.8%	-68.8%
		Male	46726	48477	13.2%	4.2%	-66.6%	-49.2%

Location	Cause	Sex	Expected DALYs in 2019		% 1990 - 2019 DALYs change cause			% 1990 - 2019 DALYs overall change
			Population growth	Population growth + Aging	Population growth	Age structure change	DALYs rate change	
	Unintentional Injuries*	Both	56031	52747	14.2%	-6.7%	-77.3%	-69.9%
		Female	19677	17475	15.1%	-12.9%	-76.4%	-74.2%
		Male	36231	35323	13.3%	-2.8%	-78%	-67.6%
	Self-harm and interpersonal violence	Both	29946	34876	14.3%	18.8%	-77.5%	-44.3%
		Female	11427	13027	15.5%	16.2%	-77.7%	-46%
		Male	18452	21835	13.2%	20.8%	-77.2%	-43.3%
**Markazi**	**All causes**	Both	569807	616925	19.2%	9.9%	-53.3%	-24.2%
		Female	247481	270531	18.5%	11%	-48.5%	-18.9%
		Male	322687	345837	19.9%	8.6%	-56.9%	-28.4%
	**Communicable, maternal, neonatal, and nutritional diseases**	Both	179962	104417	19.2%	-50%	-50.5%	-81.3%
		Female	81176	47848	18.5%	-48.7%	-50.8%	-80.9%
		Male	98867	56519	19.9%	-51.3%	-50.1%	-81.6%
	HIV/AIDS and sexually transmitted infections	Both	415	385	19.2%	-8.8%	33.3%	43.7%
		Female	207	209	18.5%	1.3%	15.3%	35.1%
		Male	208	173	19.9%	-20.3%	52.6%	52.2%
	Respiratory infections and tuberculosis	Both	31131	22756	19.2%	-32.1%	-59.1%	-72%
		Female	15573	10723	18.5%	-36.9%	-57.3%	-75.6%
		Male	15555	12023	19.9%	-27.2%	-60.9%	-68.3%
	Enteric infections	Both	9092	5994	19.2%	-40.6%	-32.9%	-54.4%
		Female	4652	3066	18.5%	-40.4%	-34.8%	-56.6%
		Male	4438	2919	19.9%	-41%	-30.8%	-51.9%
	Neglected tropical diseases and malaria	Both	3362	3258	19.2%	-3.7%	-107.9%	-92.4%
		Female	1412	1561	18.5%	12.6%	-122.1%	-91%
		Male	1953	1689	19.9%	-16.2%	-97%	-93.3%
	Other infectious diseases	Both	13800	8593	19.2%	-45%	-60.1%	-85.9%
		Female	7436	4471	18.5%	-47.3%	-59.6%	-88.3%
		Male	6357	4114	19.9%	-42.3%	-60.5%	-83%
	Maternal and neonatal disorders	Both	116007	58526	19.2%	-59.1%	-46.8%	-86.7%
		Female	48516	24996	18.5%	-57.5%	-46.6%	-85.6%
		Male	67586	33530	19.9%	-60.4%	-46.9%	-87.5%
	Nutritional deficiencies	Both	6155	4906	19.2%	-24.2%	-54.4%	-59.4%
		Female	3381	2821	18.5%	-19.6%	-57.8%	-58.8%
		Male	2770	2071	19.9%	-30.3%	-49.6%	-60%
	**Non-communicable diseases**	Both	271220	394126	19.2%	54%	-46.8%	26.4%
		Female	129230	189111	18.5%	54.9%	-41.5%	31.9%
		Male	142034	203416	19.9%	51.8%	-50.3%	21.3%
	Neoplasms	Both	27738	43867	19.2%	69.3%	-39.1%	49.5%
		Female	11698	18380	18.5%	67.7%	-30.1%	56.2%
		Male	16062	25177	19.9%	68%	-43.4%	44.5%
	Cardiovascular diseases	Both	68633	140395	19.2%	124.6%	-120.2%	23.6%
		Female	27506	60314	18.5%	141.4%	-127.8%	32.1%
		Male	41196	79465	19.9%	111.3%	-113.3%	17.9%

Location	Cause	Sex	Expected DALYs in 2019		% 1990 - 2019 DALYs change cause			% 1990 - 2019 DALYs overall change
			Population growth	Population growth + Aging	Population growth	Age structure change	DALYs rate change	
	Chronic respiratory diseases	Both	10758	18637	19.2%	87.3%	-76.1%	30.4%
		Female	4443	7843	18.5%	90.7%	-78.7%	30.5%
		Male	6324	10704	19.9%	83%	-72.6%	30.3%
	Digestive diseases	Both	8444	12846	19.2%	62.1%	-65.8%	15.6%
		Female	3767	5699	18.5%	60.8%	-63.1%	16.3%
		Male	4681	7147	19.9%	63.1%	-67.9%	15.1%
	Neurological disorders	Both	15828	22655	19.2%	51.4%	-8.8%	61.8%
		Female	8479	12086	18.5%	50.4%	-3.8%	65.2%
		Male	7342	10515	19.9%	51.8%	-13.8%	57.9%
	Mental disorders	Both	29907	36217	19.2%	25.2%	3%	47.4%
		Female	16520	20235	18.5%	26.7%	3.2%	48.4%
		Male	13366	15867	19.9%	22.4%	3.8%	46.1%
	Substance use disorders	Both	3420	4708	19.2%	44.9%	50.6%	114.7%
		Female	1130	1515	18.5%	40.3%	-7.2%	51.7%
		Male	2295	3244	19.9%	49.5%	76.7%	146.1%
	Diabetes and kidney diseases	Both	10248	17308	19.2%	82.1%	35.3%	136.6%
		Female	4970	8514	18.5%	84.5%	45.9%	149%
		Male	5278	8777	19.9%	79.4%	25.5%	124.8%
	Skin and subcutaneous diseases	Both	6245	5844	19.2%	-7.6%	1.3%	12.9%
		Female	3254	3041	18.5%	-7.7%	1%	11.8%
		Male	2989	2797	19.9%	-7.7%	1.9%	14.1%
	Sense organ diseases	Both	8565	15373	19.2%	94.8%	-44.8%	69.2%
		Female	4168	7708	18.5%	100.7%	-46.5%	72.7%
		Male	4398	7665	19.9%	89.1%	-43.1%	65.8%
	Musculoskeletal disorders	Both	17753	29800	19.2%	80.9%	10.8%	110.9%
		Female	9544	16065	18.5%	81%	14.7%	114.2%
		Male	8200	13699	19.9%	80.4%	6.7%	106.9%
	Other non-communicable diseases	Both	63682	46475	19.2%	-32.2%	-36%	-49%
		Female	33751	27711	18.5%	-21.2%	-32.7%	-35.4%
		Male	29904	18360	19.9%	-46.3%	-38.1%	-64.5%
	**Injuries***	Both	114058	114853	19.3%	0.8%	-71.8%	-51.6%
		Female	35596	32676	18.3%	-9.7%	-67.3%	-58.7%
		Male	78829	83394	20.4%	7%	-75.7%	-48.4%
	Transport Injuries	Both	66318	65528	19.2%	-1.4%	-75.9%	-58.1%
		Female	20470	17831	18.5%	-15.3%	-69.6%	-66.3%
		Male	45982	48326	19.9%	6.1%	-80.4%	-54.4%
	Unintentional Injuries*	Both	32587	31311	19.3%	-4.7%	-62.7%	-48%
		Female	11481	10179	18.3%	-13.4%	-60.4%	-55.5%
		Male	21187	21397	20.4%	1.2%	-65.5%	-43.9%
	Self-harm and interpersonal violence	Both	18522	20913	19.2%	15.4%	-78.3%	-43.7%
		Female	4579	5343	18.5%	19.8%	-78.9%	-40.6%
		Male	13993	15792	19.9%	15.4%	-80%	-44.7%

Location	Cause	Sex	Expected DALYs in 2019		% 1990 - 2019 DALYs change cause			% 1990 - 2019 DALYs overall change
			Population growth	Population growth + Aging	Population growth	Age structure change	DALYs rate change	
**Mazandaran**	**All causes**	Both	1038723	1209551	31.5%	21.6%	-40.8%	12.3%
		Female	471359	574110	31.3%	28.6%	-38.4%	21.5%
		Male	567481	633477	31.6%	15.3%	-42.4%	4.6%
	**Communicable, maternal, neonatal, and nutritional diseases**	Both	243308	129502	31.5%	-61.5%	-43%	-73%
		Female	109980	60300	31.3%	-59.3%	-43.7%	-71.7%
		Male	133357	69141	31.6%	-63.4%	-42.4%	-74.1%
	HIV/AIDS and sexually transmitted infections	Both	967	813	31.5%	-20.9%	14%	24.6%
		Female	504	484	31.3%	-5.4%	11.9%	37.8%
		Male	463	327	31.6%	-38.5%	17.1%	10.2%
	Respiratory infections and tuberculosis	Both	33998	24010	31.5%	-38.6%	-45.3%	-52.4%
		Female	14499	10263	31.3%	-38.4%	-45.6%	-52.7%
		Male	19505	13773	31.6%	-38.7%	-45.2%	-52.3%
	Enteric infections	Both	14835	9614	31.5%	-46.3%	-17.6%	-32.3%
		Female	6689	4475	31.3%	-43.5%	-15.2%	-27.3%
		Male	8148	5123	31.6%	-48.9%	-19.2%	-36.5%
	Neglected tropical diseases and malaria	Both	4147	3862	31.5%	-9%	-107.3%	-84.8%
		Female	1888	1998	31.3%	7.7%	-121.7%	-82.7%
		Male	2260	1865	31.6%	-23%	-95.2%	-86.5%
	Other infectious diseases	Both	28862	15042	31.5%	-63%	-54.9%	-86.3%
		Female	14846	7608	31.3%	-64%	-55%	-87.7%
		Male	14014	7419	31.6%	-61.9%	-54.5%	-84.8%
	Maternal and neonatal disorders	Both	148992	67188	31.5%	-72.2%	-40.5%	-81.2%
		Female	65530	30509	31.3%	-70.2%	-40.7%	-79.5%
		Male	83484	36661	31.6%	-73.8%	-40.4%	-82.6%
	Nutritional deficiencies	Both	11508	8972	31.5%	-29%	-53.4%	-50.9%
		Female	6024	4964	31.3%	-23.1%	-55.9%	-47.7%
		Male	5484	3973	31.6%	-36.3%	-49.8%	-54.4%
	**Non-communicable diseases**	Both	603265	887809	31.5%	62%	-34.9%	58.6%
		Female	303951	457348	31.3%	66.3%	-32%	65.6%
		Male	299302	427923	31.6%	56.6%	-36.7%	51.5%
	Neoplasms	Both	49391	89064	31.5%	105.6%	0.2%	137.3%
		Female	21698	39006	31.3%	104.7%	0.4%	136.4%
		Male	27701	49804	31.6%	105%	1.3%	138%
	Cardiovascular diseases	Both	133478	303013	31.5%	167%	-118.1%	80.3%
		Female	58507	138861	31.3%	180.3%	-131.3%	80.3%
		Male	74991	162376	31.6%	153.4%	-104.7%	80.3%
	Chronic respiratory diseases	Both	16424	28606	31.5%	97.5%	-51.5%	77.5%
		Female	7485	12994	31.3%	96.6%	-57.6%	70.3%
		Male	8941	15648	31.6%	98.7%	-46.9%	83.5%
	Digestive diseases	Both	15186	24190	31.5%	78%	-64.9%	44.6%
		Female	6840	10738	31.3%	74.8%	-59%	47.1%
		Male	8348	13339	31.6%	78.7%	-67.9%	42.5%

Location	Cause	Sex	Expected DALYs in 2019		% 1990 - 2019 DALYs change cause			% 1990 - 2019 DALYs overall change
			Population growth	Population growth + Aging	Population growth	Age structure change	DALYs rate change	
	Neurological disorders	Both	34004	48924	31.5%	57.7%	-1%	88.1%
		Female	18853	27059	31.3%	57.1%	1.4%	89.9%
		Male	15145	21848	31.6%	58.3%	-3.9%	86%
	Mental disorders	Both	71173	86987	31.5%	29.2%	2.3%	63%
		Female	41483	51302	31.3%	31.1%	2.3%	64.6%
		Male	29674	35541	31.6%	26%	3%	60.7%
	Substance use disorders	Both	6304	8235	31.5%	40.3%	22.7%	94.4%
		Female	2205	2838	31.3%	37.7%	-8.6%	60.5%
		Male	4102	5436	31.6%	42.8%	38.3%	112.7%
	Diabetes and kidney diseases	Both	20797	41086	31.5%	128.3%	135.9%	295.6%
		Female	10896	22150	31.3%	135.6%	138.4%	305.3%
		Male	9900	18984	31.6%	120.8%	132.6%	285%
	Skin and subcutaneous diseases	Both	14853	13843	31.5%	-8.9%	1.6%	24.2%
		Female	7819	7252	31.3%	-9.5%	1.2%	23%
		Male	7033	6587	31.6%	-8.4%	2.2%	25.5%
	Sense organ diseases	Both	18837	35722	31.5%	117.8%	-55%	94.3%
		Female	9274	17932	31.3%	122.6%	-56.1%	97.8%
		Male	9564	17773	31.6%	113%	-53.7%	90.9%
	Musculoskeletal disorders	Both	57746	98193	31.5%	92.1%	7.1%	130.7%
		Female	37961	63681	31.3%	89%	7.5%	127.8%
		Male	19761	34496	31.6%	98.2%	6.5%	136.3%
	Other non-communicable diseases	Both	165070	109946	31.5%	-43.9%	-36%	-48.5%
		Female	80931	63534	31.3%	-28.2%	-32.2%	-29.1%
		Male	84141	46091	31.6%	-59.5%	-39.3%	-67.1%
	**Injuries***	Both	186297	187535	31.5%	0.9%	-55.3%	-22.9%
		Female	56231	55518	31%	-1.7%	-61.4%	-32%
		Male	130368	132880	32.1%	2.5%	-53.5%	-18.9%
	Transport Injuries	Both	98899	98533	31.5%	-0.5%	-59.2%	-28.2%
		Female	29848	28221	31.3%	-7.2%	-65.9%	-41.8%
		Male	69102	70650	31.6%	2.9%	-56.9%	-22.4%
	Unintentional Injuries*	Both	55533	52509	31.5%	-7.2%	-41.7%	-17.3%
		Female	16969	15900	31%	-8.3%	-45.5%	-22.8%
		Male	38653	36783	32.1%	-6.4%	-40.6%	-14.9%
	Self-harm and interpersonal violence	Both	36092	40362	31.5%	15.6%	-72.9%	-25.9%
		Female	10085	12135	31.3%	26.7%	-80.8%	-22.8%
		Male	26027	28407	31.6%	12%	-70.8%	-27.1%
**North Khorasan**	**All causes**	Both	498669	447609	37.5%	-14.1%	-63%	-39.6%
		Female	226292	202500	37.6%	-14.5%	-60%	-36.8%
		Male	272334	243601	37.3%	-14.5%	-64.8%	-41.9%
	**Communicable, maternal, neonatal, and nutritional diseases**	Both	211588	126334	37.5%	-55.4%	-67.7%	-85.6%
		Female	98624	58620	37.6%	-55.8%	-67.5%	-85.7%
		Male	112952	67672	37.3%	-55.1%	-67.7%	-85.5%

Location	Cause	Sex	Expected DALYs in 2019		% 1990 - 2019 DALYs change cause			% 1990 - 2019 DALYs overall change
			Population growth	Population growth + Aging	Population growth	Age structure change	DALYs rate change	
	HIV/AIDS and sexually transmitted infections	Both	302	251	37.5%	-23.1%	48.5%	62.9%
		Female	154	140	37.6%	-12.3%	75.1%	100.4%
		Male	148	111	37.3%	-34.9%	21.5%	24%
	Respiratory infections and tuberculosis	Both	57660	36215	37.5%	-51.1%	-72.8%	-86.4%
		Female	28726	17605	37.6%	-53.3%	-71.9%	-87.6%
		Male	28935	18555	37.3%	-49.3%	-73.4%	-85.3%
	Enteric infections	Both	15277	9578	37.5%	-51.3%	-63.7%	-77.5%
		Female	7321	4519	37.6%	-52.7%	-62.9%	-77.9%
		Male	7955	5052	37.3%	-50.1%	-64.3%	-77.1%
	Neglected tropical diseases and malaria	Both	1291	1017	37.5%	-29.2%	-86%	-77.7%
		Female	530	424	37.6%	-27.6%	-84.7%	-74.7%
		Male	761	596	37.3%	-29.8%	-87.3%	-79.7%
	Other infectious diseases	Both	21334	13110	37.5%	-53%	-75.7%	-91.2%
		Female	11133	6644	37.6%	-55.5%	-74.8%	-92.7%
		Male	10202	6437	37.3%	-50.7%	-76.3%	-89.6%
	Maternal and neonatal disorders	Both	108447	60862	37.5%	-60.3%	-63.7%	-86.6%
		Female	46883	26410	37.6%	-60.1%	-63.6%	-86%
		Male	61550	34503	37.3%	-60.4%	-63.9%	-86.9%
	Nutritional deficiencies	Both	7277	5301	37.5%	-37.3%	-72.4%	-72.2%
		Female	3876	2878	37.6%	-35.4%	-73.8%	-71.5%
		Male	3401	2419	37.3%	-39.7%	-70.6%	-73%
	**Non-communicable diseases**	Both	202123	245189	37.5%	29.3%	-52.9%	13.8%
		Female	96263	118458	37.6%	31.7%	-47.2%	22.1%
		Male	105852	125193	37.3%	25.1%	-56.1%	6.3%
	Neoplasms	Both	17118	24822	37.5%	61.9%	-34.8%	64.6%
		Female	7224	10449	37.6%	61.4%	-25.8%	73.3%
		Male	9891	14012	37.3%	57.2%	-36.3%	58.2%
	Cardiovascular diseases	Both	46296	80744	37.5%	102.3%	-111.5%	28.3%
		Female	19459	35652	37.6%	114.5%	-109.9%	42.2%
		Male	26830	44173	37.3%	88.8%	-108%	18.1%
	Chronic respiratory diseases	Both	6883	10307	37.5%	68.4%	-68.1%	37.8%
		Female	3144	4769	37.6%	71.1%	-69.3%	39.5%
		Male	3739	5483	37.3%	64.1%	-65.1%	36.3%
	Digestive diseases	Both	8219	9549	37.5%	22.3%	-76%	-16.2%
		Female	3803	4342	37.6%	19.5%	-73.4%	-16.2%
		Male	4415	5127	37.3%	22.2%	-75.7%	-16.2%
	Neurological disorders	Both	10057	12391	37.5%	31.9%	-13.7%	55.6%
		Female	5690	6839	37.6%	27.8%	-11.9%	53.5%
		Male	4369	5539	37.3%	36.8%	-15.8%	58.4%
	Mental disorders	Both	17827	20918	37.5%	23.8%	1.6%	62.9%
		Female	9862	11732	37.6%	26.1%	2.1%	65.8%
		Male	7967	9167	37.3%	20.7%	1.3%	59.4%

Location	Cause	Sex	Expected DALYs in 2019		% 1990 - 2019 DALYs change cause			% 1990 - 2019 DALYs overall change
			Population growth	Population growth + Aging	Population growth	Age structure change	DALYs rate change	
	Substance use disorders	Both	2127	2761	37.5%	41%	29.7%	108.2%
		Female	728	924	37.6%	37.1%	-4.1%	70.7%
		Male	1398	1853	37.3%	44.7%	45.6%	127.7%
	Diabetes and kidney diseases	Both	7899	11261	37.5%	58.5%	3.8%	99.8%
		Female	3653	5337	37.6%	63.4%	14.9%	115.9%
		Male	4246	5866	37.3%	52.4%	-3.9%	85.9%
	Skin and subcutaneous diseases	Both	3687	3519	37.5%	-6.3%	3.6%	34.8%
		Female	1941	1846	37.6%	-6.7%	3.1%	34%
		Male	1746	1671	37.3%	-5.9%	4.2%	35.7%
	Sense organ diseases	Both	4793	7873	37.5%	88.3%	-52.9%	72.9%
		Female	2298	3909	37.6%	96.5%	-55.5%	78.7%
		Male	2495	3961	37.3%	80.7%	-50.4%	67.7%
	Musculoskeletal disorders	Both	10594	16371	37.5%	75%	7.7%	120.1%
		Female	5584	8843	37.6%	80.3%	12.6%	130.6%
		Male	5011	7556	37.3%	69.7%	1.5%	108.6%
	Other non-communicable diseases	Both	66622	44672	37.5%	-45.3%	-55.1%	-62.9%
		Female	32876	23814	37.6%	-37.9%	-51%	-51.3%
		Male	33746	20784	37.3%	-52.8%	-58.8%	-74.2%
	**Injuries***	Both	83340	75043	36.3%	-13.6%	-74.4%	-51.7%
		Female	30619	25091	35.7%	-24.5%	-74.7%	-63.5%
		Male	52805	50154	36.8%	-6.9%	-74.8%	-44.9%
	Transport Injuries	Both	48970	44250	37.5%	-13.3%	-81.1%	-56.9%
		Female	16891	13588	37.6%	-26.9%	-79.7%	-69%
		Male	32064	30715	37.3%	-5.8%	-82.1%	-50.5%
	Unintentional Injuries*	Both	28602	24143	36.3%	-21.2%	-71.7%	-56.7%
		Female	11351	8784	35.7%	-30.7%	-71.8%	-66.8%
		Male	17273	15399	36.8%	-14.8%	-72%	-50%
	Self-harm and interpersonal violence	Both	6667	7311	37.5%	13.3%	-46%	4.8%
		Female	2806	2921	37.6%	5.6%	-62.2%	-18.9%
		Male	3860	4398	37.3%	19.2%	-34.5%	22%
**Qazvin**	**All causes**	Both	496807	500337	39.9%	1%	-55.8%	-14.9%
		Female	225545	227918	40.4%	1.5%	-57.1%	-15.2%
		Male	271127	271549	39.3%	0.2%	-54.2%	-14.7%
	**Communicable, maternal, neonatal, and nutritional diseases**	Both	164774	98301	39.9%	-56.4%	-63.7%	-80.3%
		Female	74984	45351	40.4%	-55.5%	-65.1%	-80.2%
		Male	89747	52922	39.3%	-57.2%	-62.5%	-80.4%
	HIV/AIDS and sexually transmitted infections	Both	379	355	39.9%	-9.2%	29.6%	60.3%
		Female	190	192	40.4%	2.1%	11.6%	54.1%
		Male	190	161	39.3%	-21.4%	48.5%	66.4%
	Respiratory infections and tuberculosis	Both	26050	17223	39.9%	-47.4%	-67.3%	-74.8%
		Female	12886	8233	40.4%	-50.7%	-69%	-79.3%
		Male	13166	8991	39.3%	-44.2%	-65.7%	-70.5%

Location	Cause	Sex	Expected DALYs in 2019		% 1990 - 2019 DALYs change cause			% 1990 - 2019 DALYs overall change
			Population growth	Population growth + Aging	Population growth	Age structure change	DALYs rate change	
	Enteric infections	Both	9553	6271	39.9%	-48.1%	-46.9%	-55.1%
		Female	4676	3071	40.4%	-48.2%	-47.9%	-55.7%
		Male	4877	3191	39.3%	-48.2%	-45.6%	-54.5%
	Neglected tropical diseases and malaria	Both	2521	2902	39.9%	21.1%	-150.8%	-89.8%
		Female	1171	1389	40.4%	26.2%	-155.8%	-89.1%
		Male	1350	1510	39.3%	16.5%	-146.1%	-90.3%
	Other infectious diseases	Both	16384	9944	39.9%	-55%	-73.7%	-88.9%
		Female	8889	5273	40.4%	-57.1%	-74.1%	-90.8%
		Male	7503	4660	39.3%	-52.8%	-73.1%	-86.6%
	Maternal and neonatal disorders	Both	103759	56779	39.9%	-63.3%	-60.8%	-84.3%
		Female	43661	24366	40.4%	-62.1%	-61.3%	-83%
		Male	60043	32419	39.3%	-64.1%	-60.5%	-85.2%
	Nutritional deficiencies	Both	6126	4827	39.9%	-29.7%	-67.7%	-57.5%
		Female	3512	2828	40.4%	-27.4%	-72.4%	-59.4%
		Male	2618	1991	39.3%	-33.4%	-60.8%	-54.9%
	**Non-communicable diseases**	Both	235394	308531	39.9%	43.5%	-40%	43.4%
		Female	114945	152256	40.4%	45.6%	-45%	41%
		Male	120447	155213	39.3%	40.2%	-34%	45.6%
	Neoplasms	Both	18877	28907	39.9%	74.3%	2.7%	116.9%
		Female	8277	12750	40.4%	75.9%	-16.9%	99.4%
		Male	10593	15923	39.3%	70.1%	21%	130.5%
	Cardiovascular diseases	Both	51527	95265	39.9%	118.7%	-104.7%	53.9%
		Female	21277	40992	40.4%	130.1%	-129.2%	41.4%
		Male	30219	53852	39.3%	109%	-85.6%	62.7%
	Chronic respiratory diseases	Both	7871	11987	39.9%	73.2%	-54%	59%
		Female	3611	5611	40.4%	77.7%	-78.6%	39.6%
		Male	4257	6340	39.3%	68.1%	-32.1%	75.4%
	Digestive diseases	Both	11193	15395	39.9%	52.5%	-71.9%	20.4%
		Female	5348	7182	40.4%	48.2%	-82.4%	6.2%
		Male	5844	8125	39.3%	54.4%	-60.3%	33.4%
	Neurological disorders	Both	13501	17969	39.9%	46.3%	-5.1%	81%
		Female	7430	10039	40.4%	49.3%	-4.1%	85.7%
		Male	6077	7895	39.3%	41.7%	-5.7%	75.4%
	Mental disorders	Both	24818	30268	39.9%	30.7%	1.1%	71.6%
		Female	13664	16943	40.4%	33.7%	0.6%	74.7%
		Male	11166	13277	39.3%	26.3%	2.2%	67.9%
	Substance use disorders	Both	3150	4455	39.9%	58%	126.7%	224.5%
		Female	1121	1527	40.4%	50.9%	-1.7%	89.6%
		Male	2026	2952	39.3%	63.7%	195.5%	298.5%
	Diabetes and kidney diseases	Both	7135	11256	39.9%	80.8%	59.6%	180.2%
		Female	3560	5741	40.4%	86%	52.5%	178.9%
		Male	3575	5505	39.3%	75.2%	67.1%	181.6%

Location	Cause	Sex	Expected DALYs in 2019		% 1990 - 2019 DALYs change cause			% 1990 - 2019 DALYs overall change
			Population growth	Population growth + Aging	Population growth	Age structure change	DALYs rate change	
	Skin and subcutaneous diseases	Both	5710	5316	39.9%	-9.7%	2.2%	32.4%
		Female	2966	2762	40.4%	-9.7%	1.6%	32.3%
		Male	2745	2552	39.3%	-9.8%	3%	32.6%
	Sense organ diseases	Both	7456	12217	39.9%	89.3%	-52.9%	76.2%
		Female	3631	6190	40.4%	99%	-55.6%	83.8%
		Male	3826	6022	39.3%	80%	-50.3%	69.1%
	Musculoskeletal disorders	Both	15358	26138	39.9%	98.2%	11%	149%
		Female	8111	13893	40.4%	100.1%	17.1%	157.6%
		Male	7252	12254	39.3%	96.1%	4%	139.5%
	Other non-communicable diseases	Both	68798	49358	39.9%	-39.5%	-49.5%	-49.1%
		Female	35949	28626	40.4%	-28.6%	-46.2%	-34.4%
		Male	32868	20516	39.3%	-52.4%	-52.1%	-65.1%
	**Injuries***	Both	92463	90887	38.3%	-2.4%	-79.5%	-43.6%
		Female	33896	29595	38.6%	-17.6%	-79.6%	-58.6%
		Male	58508	61537	37.9%	7.1%	-80%	-34.9%
	Transport Injuries	Both	53945	52964	39.9%	-2.5%	-88.9%	-51.6%
		Female	19723	16501	40.4%	-22.9%	-85.6%	-68.1%
		Male	34170	36597	39.3%	9.9%	-91.4%	-42.2%
	Unintentional Injuries*	Both	29021	26508	38.3%	-12%	-67.3%	-41%
		Female	11461	9564	38.6%	-23%	-71%	-55.3%
		Male	17545	17001	37.9%	-4.3%	-65.4%	-31.8%
	Self-harm and interpersonal violence	Both	12298	13443	39.9%	13%	-76.6%	-23.8%
		Female	3775	4039	40.4%	9.8%	-77.3%	-27.1%
		Male	8506	9439	39.3%	15.3%	-76.9%	-22.3%
**Qom**	**All causes**	Both	468030	492876	84.1%	9.8%	-82.3%	11.5%
		Female	208116	218203	85.8%	9%	-75.3%	19.5%
		Male	259524	273512	82.4%	9.8%	-87%	5.3%
	**Communicable, maternal, neonatal, and nutritional diseases**	Both	147253	96176	84.1%	-63.8%	-87.8%	-67.5%
		Female	66441	43498	85.8%	-64.2%	-89.1%	-67.5%
		Male	80708	52693	82.4%	-63.3%	-86.7%	-67.6%
	HIV/AIDS and sexually transmitted infections	Both	531	517	84.1%	-4.7%	694%	773.4%
		Female	243	240	85.8%	-2.9%	545.6%	628.6%
		Male	287	277	82.4%	-6.3%	817.7%	893.8%
	Respiratory infections and tuberculosis	Both	33498	24134	84.1%	-51.5%	-100.7%	-68.1%
		Female	16677	11449	85.8%	-58.3%	-99.6%	-72%
		Male	16827	12666	82.4%	-45.1%	-101.5%	-64.2%
	Enteric infections	Both	9139	6323	84.1%	-56.7%	-61.4%	-34%
		Female	4327	2960	85.8%	-58.7%	-60.5%	-33.4%
		Male	4809	3354	82.4%	-55.2%	-61.8%	-34.6%
	Neglected tropical diseases and malaria	Both	6636	6564	84.1%	-2%	-175.7%	-93.7%
		Female	2803	2769	85.8%	-2.3%	-176.2%	-92.6%
		Male	3825	3784	82.4%	-1.9%	-174.9%	-94.4%

Location	Cause	Sex	Expected DALYs in 2019		% 1990 - 2019 DALYs change cause			% 1990 - 2019 DALYs overall change
			Population growth	Population growth + Aging	Population growth	Age structure change	DALYs rate change	
	Other infectious diseases	Both	15145	10035	84.1%	-62.1%	-105.4%	-83.4%
		Female	8102	5185	85.8%	-66.9%	-105.4%	-86.5%
		Male	7056	4838	82.4%	-57.4%	-105%	-80%
	Maternal and neonatal disorders	Both	76403	43765	84.1%	-78.6%	-79.5%	-74.1%
		Female	31088	18190	85.8%	-77.1%	-79.8%	-71.1%
		Male	45198	25648	82.4%	-78.9%	-79.6%	-76.1%
	Nutritional deficiencies	Both	5901	4839	84.1%	-33.1%	-88.1%	-37.2%
		Female	3201	2705	85.8%	-28.8%	-94.7%	-37.6%
		Male	2705	2126	82.4%	-39.1%	-80.1%	-36.7%
	**Non-communicable diseases**	Both	236109	310302	84.1%	57.8%	-66.9%	75%
		Female	116279	150891	85.8%	55.3%	-59.2%	82%
		Male	119843	158948	82.4%	59.5%	-73.5%	68.4%
	Neoplasms	Both	23559	35294	84.1%	91.7%	-56.2%	119.6%
		Female	10380	15346	85.8%	88.9%	-51.3%	123.5%
		Male	13158	19962	82.4%	94.3%	-60.2%	116.5%
	Cardiovascular diseases	Both	48757	88846	84.1%	151.3%	-170.9%	64.5%
		Female	20968	37399	85.8%	145.6%	-162.3%	69.2%
		Male	27735	50716	82.4%	151.1%	-172.5%	61%
	Chronic respiratory diseases	Both	9495	14799	84.1%	102.8%	-115.1%	71.7%
		Female	4103	6232	85.8%	96.4%	-110.9%	71.4%
		Male	5382	8540	82.4%	107%	-117.4%	72%
	Digestive diseases	Both	9780	13510	84.1%	70.2%	-123.8%	30.4%
		Female	4480	5939	85.8%	60.5%	-119.8%	26.6%
		Male	5295	7505	82.4%	76.2%	-125%	33.6%
	Neurological disorders	Both	13648	17697	84.1%	54.6%	-10.3%	128.4%
		Female	7477	9626	85.8%	53.4%	-7%	132.2%
		Male	6185	8136	82.4%	57.5%	-16.1%	123.9%
	Mental disorders	Both	27529	33195	84.1%	37.9%	7.2%	129.2%
		Female	15076	18612	85.8%	43.6%	6.2%	135.6%
		Male	12482	14687	82.4%	32.2%	6.9%	121.5%
	Substance use disorders	Both	2638	3558	84.1%	64.2%	73%	221.2%
		Female	825	1107	85.8%	63.7%	-7.9%	141.6%
		Male	1805	2420	82.4%	62.2%	112.4%	256.9%
	Diabetes and kidney diseases	Both	9333	14933	84.1%	110.5%	8.6%	203.2%
		Female	4645	7302	85.8%	106.3%	18.4%	210.5%
		Male	4689	7595	82.4%	113%	0.5%	196%
	Skin and subcutaneous diseases	Both	5885	5527	84.1%	-11.2%	6.8%	79.7%
		Female	3075	2878	85.8%	-11.9%	5.9%	79.9%
		Male	2814	2654	82.4%	-10.4%	7.5%	79.6%
	Sense organ diseases	Both	7213	11379	84.1%	106.3%	-63.5%	126.9%
		Female	3534	5483	85.8%	102.5%	-64.2%	124.1%
		Male	3679	5893	82.4%	109.8%	-62.6%	129.5%

Location	Cause	Sex	Expected DALYs in 2019		% 1990 - 2019 DALYs change cause			% 1990 - 2019 DALYs overall change
			Population growth	Population growth + Aging	Population growth	Age structure change	DALYs rate change	
	Musculoskeletal disorders	Both	14985	24304	84.1%	114.5%	19%	217.6%
		Female	7953	13006	85.8%	118.1%	23.2%	227.1%
		Male	7044	11327	82.4%	110.9%	13.6%	207%
	Other non-communicable diseases	Both	63286	47260	84.1%	-46.6%	-63.6%	-26.2%
		Female	33763	27962	85.8%	-31.9%	-60.7%	-6.8%
		Male	29575	19513	82.4%	-62.1%	-68.3%	-47.9%
	**Injuries***	Both	81291	83741	80.3%	5.4%	-112.3%	-26.5%
		Female	24554	23335	81.6%	-9%	-110.7%	-38.1%
		Male	56530	59823	79.1%	10.4%	-111.1%	-21.5%
	Transport Injuries	Both	35851	36094	84.1%	1.3%	-133.7%	-48.4%
		Female	10719	9750	85.8%	-16.8%	-129.1%	-60%
		Male	25005	26035	82.4%	7.5%	-133.4%	-43.5%
	Unintentional Injuries*	Both	27443	27563	80.3%	0.8%	-89.2%	-8%
		Female	9123	8422	81.6%	-14%	-94.1%	-26.5%
		Male	18261	18974	79.1%	7%	-85.1%	1%
	Self-harm and interpersonal violence	Both	20558	22296	84.1%	15.6%	-118.3%	-18.6%
		Female	5182	5495	85.8%	11.2%	-112.3%	-15.2%
		Male	15286	16590	82.4%	15.6%	-117.7%	-19.8%
**Semnan**	**All causes**	Both	272340	282565	54.9%	5.8%	-60.8%	-0.1%
		Female	119648	123822	57.1%	5.5%	-57.1%	5.5%
		Male	152317	158635	52.9%	6.3%	-63.6%	-4.3%
	**Communicable, maternal, neonatal, and nutritional diseases**	Both	81728	50885	54.9%	-58.5%	-69.6%	-73.2%
		Female	37807	23873	57.1%	-57.9%	-71.9%	-72.7%
		Male	43859	26998	52.9%	-58.8%	-67.7%	-73.6%
	HIV/AIDS and sexually transmitted infections	Both	194	176	54.9%	-13.9%	-7.4%	33.7%
		Female	104	105	57.1%	2.2%	-8.7%	50.6%
		Male	90	71	52.9%	-33%	-5.1%	14.8%
	Respiratory infections and tuberculosis	Both	15003	10927	54.9%	-42.1%	-73.3%	-60.5%
		Female	6931	4899	57.1%	-46.1%	-74.2%	-63.2%
		Male	8060	6043	52.9%	-38.3%	-72.9%	-58.3%
	Enteric infections	Both	4289	2908	54.9%	-49.9%	-44%	-39%
		Female	1968	1359	57.1%	-48.6%	-43.8%	-35.3%
		Male	2317	1543	52.9%	-51.1%	-43.8%	-42%
	Neglected tropical diseases and malaria	Both	1997	2242	54.9%	19%	-165.7%	-91.8%
		Female	945	1051	57.1%	17.6%	-166%	-91.3%
		Male	1051	1190	52.9%	20.3%	-165.4%	-92.2%
	Other infectious diseases	Both	5667	3615	54.9%	-56.1%	-81.8%	-82.9%
		Female	2866	1787	57.1%	-59.1%	-83%	-85%
		Male	2803	1827	52.9%	-53.2%	-80.5%	-80.9%
	Maternal and neonatal disorders	Both	51506	28568	54.9%	-69%	-65.6%	-79.7%
		Female	23306	13284	57.1%	-67.5%	-68.2%	-78.7%
		Male	28148	15270	52.9%	-69.9%	-63.5%	-80.5%

Location	Cause	Sex	Expected DALYs in 2019		% 1990 - 2019 DALYs change cause			% 1990 - 2019 DALYs overall change
			Population growth	Population growth + Aging	Population growth	Age structure change	DALYs rate change	
	Nutritional deficiencies	Both	3072	2448	54.9%	-31.5%	-73.4%	-49.9%
		Female	1688	1389	57.1%	-27.8%	-78.5%	-49.2%
		Male	1389	1053	52.9%	-37.1%	-66.6%	-50.7%
	**Non-communicable diseases**	Both	140863	180293	54.9%	43.4%	-43.9%	54.4%
		Female	66997	85867	57.1%	44.2%	-41.9%	59.4%
		Male	73808	94362	52.9%	42.6%	-45.6%	49.9%
	Neoplasms	Both	13334	18956	54.9%	65.3%	-24.8%	95.4%
		Female	5497	7862	57.1%	67.6%	-21.5%	103.2%
		Male	7809	11089	52.9%	64.2%	-27%	90.1%
	Cardiovascular diseases	Both	36611	59389	54.9%	96.4%	-108.9%	42.4%
		Female	14657	24336	57.1%	103.7%	-121.8%	39%
		Male	21865	35039	52.9%	92.1%	-100.3%	44.7%
	Chronic respiratory diseases	Both	5597	8078	54.9%	68.7%	-68.4%	55.1%
		Female	2361	3435	57.1%	71.4%	-76.1%	52.5%
		Male	3226	4653	52.9%	67.7%	-63.5%	57.1%
	Digestive diseases	Both	4109	5621	54.9%	57%	-64.6%	47.4%
		Female	1797	2448	57.1%	56.8%	-70.1%	43.8%
		Male	2306	3171	52.9%	57.4%	-60.2%	50.1%
	Neurological disorders	Both	8526	10811	54.9%	41.5%	-10.6%	85.9%
		Female	4621	5951	57.1%	45.2%	-6.2%	96.1%
		Male	3916	4857	52.9%	36.7%	-15.5%	74.1%
	Mental disorders	Both	14808	17671	54.9%	30%	1.6%	86.5%
		Female	7845	9493	57.1%	33%	3%	93.1%
		Male	6978	8177	52.9%	26.3%	0.1%	79.3%
	Substance use disorders	Both	1787	2458	54.9%	58.1%	48.7%	161.7%
		Female	563	752	57.1%	52.9%	-4.7%	105.2%
		Male	1216	1709	52.9%	62%	72.3%	187.2%
	Diabetes and kidney diseases	Both	5780	8461	54.9%	71.9%	76%	202.7%
		Female	2791	4124	57.1%	75%	85.6%	217.7%
		Male	2987	4340	52.9%	69.2%	67.1%	189.2%
	Skin and subcutaneous diseases	Both	3227	3045	54.9%	-8.7%	1.8%	48%
		Female	1684	1588	57.1%	-9%	0.9%	49%
		Male	1545	1457	52.9%	-8.7%	2.7%	46.9%
	Sense organ diseases	Both	4524	6694	54.9%	74.3%	-52.9%	76.3%
		Female	2260	3391	57.1%	78.6%	-55.2%	80.5%
		Male	2264	3298	52.9%	69.8%	-50.3%	72.3%
	Musculoskeletal disorders	Both	9710	14647	54.9%	78.8%	13.2%	146.9%
		Female	5255	7879	57.1%	78.4%	17.1%	152.7%
		Male	4468	6762	52.9%	78.5%	9%	140.4%
	Other non-communicable diseases	Both	32851	24461	54.9%	-39.6%	-48%	-32.6%
		Female	17665	14609	57.1%	-27.2%	-45.1%	-15.2%
		Male	15226	9809	52.9%	-54.4%	-50.8%	-52.3%

Location	Cause	Sex	Expected DALYs in 2019		% 1990 - 2019 DALYs change cause			% 1990 - 2019 DALYs overall change
			Population growth	Population growth + Aging	Population growth	Age structure change	DALYs rate change	
	**Injuries***	Both	48028	50095	54.3%	6.6%	-93%	-32.1%
		Female	14362	13817	56.3%	-5.9%	-87.5%	-37.1%
		Male	33429	36263	52.3%	12.9%	-95.2%	-30%
	Transport Injuries	Both	26100	27044	54.9%	5.6%	-97.4%	-36.8%
		Female	7895	7285	57.1%	-12.1%	-92.1%	-47.1%
		Male	18073	19749	52.9%	14.2%	-99.5%	-32.4%
	Unintentional Injuries*	Both	13682	13570	54.3%	-1.3%	-81.3%	-28.3%
		Female	4297	3976	56.3%	-11.7%	-74.9%	-30.2%
		Male	9323	9597	52.3%	4.5%	-84.2%	-27.4%
	Self-harm and interpersonal violence	Both	9519	10559	54.9%	16.9%	-104.5%	-32.6%
		Female	2463	2747	57.1%	18.1%	-98.6%	-23.4%
		Male	6997	7791	52.9%	17.3%	-106%	-35.8%
**Sistan and Baluchistan**	**All causes**	Both	1738960	1580648	101.9%	-18.4%	-99.4%	-15.9%
		Female	748341	686478	102%	-16.7%	-97.1%	-11.8%
		Male	990524	892003	101.9%	-20.1%	-100.7%	-19%
	**Communicable, maternal, neonatal, and nutritional diseases**	Both	883052	704723	101.9%	-40.8%	-129.5%	-68.4%
		Female	399920	316748	102%	-42%	-128.4%	-68.3%
		Male	483101	388132	101.9%	-39.7%	-130.6%	-68.4%
	HIV/AIDS and sexually transmitted infections	Both	1185	1109	101.9%	-12.9%	676.7%	765.7%
		Female	596	587	102%	-3.1%	866.6%	965.5%
		Male	588	527	101.9%	-21.2%	482.8%	563.5%
	Respiratory infections and tuberculosis	Both	176407	142698	101.9%	-38.6%	-140.6%	-77.2%
		Female	80787	64535	102%	-40.6%	-139.6%	-78.2%
		Male	95615	78141	101.9%	-36.9%	-141.3%	-76.4%
	Enteric infections	Both	96081	76011	101.9%	-42.2%	-134.9%	-75.1%
		Female	42689	33413	102%	-43.9%	-132.5%	-74.4%
		Male	53388	42651	101.9%	-40.6%	-136.9%	-75.7%
	Neglected tropical diseases and malaria	Both	71029	62592	101.9%	-24%	-174.1%	-96.1%
		Female	33187	28391	102%	-29.2%	-168.6%	-95.8%
		Male	37840	33933	101.9%	-20.8%	-177.4%	-96.4%
	Other infectious diseases	Both	90522	71991	101.9%	-41.3%	-137%	-76.4%
		Female	45222	35438	102%	-43.7%	-136.8%	-78.5%
		Male	45300	36460	101.9%	-39.4%	-136.7%	-74.2%
	Maternal and neonatal disorders	Both	407875	317514	101.9%	-44.7%	-116.5%	-59.3%
		Female	177263	137800	102%	-45%	-115.8%	-58.7%
		Male	230591	180183	101.9%	-44.1%	-117.4%	-59.7%
	Nutritional deficiencies	Both	39953	32809	101.9%	-36.1%	-129.5%	-63.6%
		Female	20175	16585	102%	-35.9%	-129.5%	-63.4%
		Male	19779	16237	101.9%	-36.1%	-129.6%	-63.9%
	**Non-communicable diseases**	Both	585000	615904	101.9%	10.7%	-53.7%	59%
		Female	262323	291828	102%	22.7%	-46.8%	77.9%
		Male	322654	321710	101.9%	-0.6%	-57.7%	43.5%

Location	Cause	Sex	Expected DALYs in 2019		% 1990 - 2019 DALYs change cause			% 1990 - 2019 DALYs overall change
			Population growth	Population growth + Aging	Population growth	Age structure change	DALYs rate change	
	Neoplasms	Both	35564	39532	101.9%	22.5%	5.9%	130.4%
		Female	14332	17316	102%	42.1%	23.6%	167.7%
		Male	21229	21777	101.9%	5.2%	-1.9%	105.2%
	Cardiovascular diseases	Both	114917	139829	101.9%	43.8%	-76.9%	68.8%
		Female	43807	58911	102%	69.7%	-81.9%	89.8%
		Male	71098	78963	101.9%	22.3%	-68.3%	55.9%
	Chronic respiratory diseases	Both	33217	37798	101.9%	27.9%	-90.4%	39.4%
		Female	13980	17315	102%	48.2%	-97.3%	53%
		Male	19235	20223	101.9%	10.4%	-82.7%	29.5%
	Digestive diseases	Both	25176	27072	101.9%	15.2%	-70%	47.2%
		Female	10441	11743	102%	25.2%	-64.7%	62.5%
		Male	14734	15076	101.9%	4.7%	-70.2%	36.3%
	Neurological disorders	Both	29388	33567	101.9%	28.7%	-9.1%	121.5%
		Female	15434	18144	102%	35.5%	-1.8%	135.7%
		Male	13954	15456	101.9%	21.7%	-17.7%	105.8%
	Mental disorders	Both	56815	64625	101.9%	27.8%	2.7%	132.5%
		Female	31027	35962	102%	32.1%	2.6%	136.7%
		Male	25790	28703	101.9%	22.8%	2.7%	127.3%
	Substance use disorders	Both	7976	10346	101.9%	60%	122.4%	284.4%
		Female	2749	3463	102%	52.4%	104%	258.5%
		Male	5225	6865	101.9%	63.4%	132.8%	298.1%
	Diabetes and kidney diseases	Both	22780	25204	101.9%	21.5%	43.5%	167%
		Female	9426	11267	102%	39.4%	70.3%	211.8%
		Male	13352	13725	101.9%	5.6%	27.9%	135.4%
	Skin and subcutaneous diseases	Both	12768	12380	101.9%	-6.1%	5.7%	101.5%
		Female	6715	6489	102%	-6.8%	4.8%	100%
		Male	6054	5887	101.9%	-5.6%	6.9%	103.2%
	Sense organ diseases	Both	15415	18417	101.9%	39.3%	-52.4%	88.9%
		Female	7121	9113	102%	56.5%	-56.3%	102.3%
		Male	8293	9296	101.9%	24.4%	-48.8%	77.4%
	Musculoskeletal disorders	Both	30769	39533	101.9%	57.5%	10.3%	169.8%
		Female	15813	21424	102%	71.7%	16.2%	189.9%
		Male	14956	18273	101.9%	44.8%	1.9%	148.5%
	Other non-communicable diseases	Both	200217	167599	101.9%	-32.9%	-97.1%	-28%
		Female	91477	80680	102%	-23.8%	-86.2%	-8%
		Male	108733	87467	101.9%	-39.5%	-107.3%	-44.9%
	**Injuries***	Both	260890	252703	97.3%	-6.2%	-96%	-4.9%
		Female	82717	75891	97.3%	-16.3%	-102.2%	-21.2%
		Male	178199	176917	97.4%	-1.4%	-93.3%	2.7%
	Transport Injuries	Both	140283	134929	101.9%	-7.7%	-95.8%	-1.6%
		Female	44608	40459	102%	-18.8%	-104%	-20.8%
		Male	95654	94371	101.9%	-2.7%	-91.8%	7.4%

Location	Cause	Sex	Expected DALYs in 2019		% 1990 - 2019 DALYs change cause			% 1990 - 2019 DALYs overall change
			Population growth	Population growth + Aging	Population growth	Age structure change	DALYs rate change	
	Unintentional Injuries*	Both	102878	95208	97.3%	-14.7%	-113.5%	-30.9%
		Female	35665	31881	97.3%	-20.9%	-113.6%	-37.3%
		Male	67221	63326	97.4%	-11.4%	-113.4%	-27.5%
	Self-harm and interpersonal violence	Both	23663	27298	101.9%	31%	-50.9%	82.1%
		Female	4399	4800	102%	18.4%	-26.7%	93.8%
		Male	19257	22661	101.9%	35.7%	-58.1%	79.4%
**South Khorasan**	**All causes**	Both	451924	396633	24.2%	-15.2%	-55%	-45.9%
		Female	200538	179634	24.1%	-12.9%	-53.5%	-42.4%
		Male	251433	215945	24.4%	-17.6%	-55.6%	-48.8%
	**Communicable, maternal, neonatal, and nutritional diseases**	Both	195899	120476	24.2%	-47.8%	-62.1%	-85.7%
		Female	90682	56339	24.1%	-47%	-62.9%	-85.8%
		Male	105230	64007	24.4%	-48.7%	-61.3%	-85.6%
	HIV/AIDS and sexually transmitted infections	Both	241	211	24.2%	-15.5%	15.9%	24.6%
		Female	123	119	24.1%	-4.1%	20.6%	40.6%
		Male	118	91	24.4%	-27.7%	11.1%	7.8%
	Respiratory infections and tuberculosis	Both	53036	34557	24.2%	-43.3%	-67.9%	-87%
		Female	25900	16702	24.1%	-44.1%	-68%	-88%
		Male	27136	17812	24.4%	-42.7%	-67.6%	-86%
	Enteric infections	Both	12873	8298	24.2%	-44.2%	-57%	-76.9%
		Female	5944	3860	24.1%	-43.5%	-56.8%	-76.2%
		Male	6930	4433	24.4%	-44.8%	-57.2%	-77.6%
	Neglected tropical diseases and malaria	Both	538	425	24.2%	-26%	-59.3%	-61.2%
		Female	277	229	24.1%	-21.7%	-60.9%	-58.4%
		Male	260	196	24.4%	-30.5%	-57.9%	-64.1%
	Other infectious diseases	Both	17780	11112	24.2%	-46.6%	-70%	-92.3%
		Female	8735	5431	24.1%	-46.9%	-70%	-92.8%
		Male	9044	5666	24.4%	-46.5%	-69.7%	-91.8%
	Maternal and neonatal disorders	Both	102618	59733	24.2%	-51.9%	-58.2%	-85.9%
		Female	45078	26687	24.1%	-50.6%	-59.1%	-85.7%
		Male	57552	32976	24.4%	-53.1%	-57.4%	-86.1%
	Nutritional deficiencies	Both	8813	6141	24.2%	-37.7%	-66.4%	-79.8%
		Female	4623	3309	24.1%	-35.3%	-68%	-79.2%
		Male	4189	2832	24.4%	-40.3%	-64.6%	-80.5%
	**Non-communicable diseases**	Both	180300	209966	24.2%	20.4%	-41.5%	3.1%
		Female	83074	101613	24.1%	27.7%	-38.1%	13.7%
		Male	97239	107142	24.4%	12.7%	-42.9%	-5.9%
	Neoplasms	Both	16025	20613	24.2%	35.6%	-26.2%	33.6%
		Female	6384	8619	24.1%	43.4%	-17.6%	50%
		Male	9644	11702	24.4%	26.5%	-28.1%	22.8%
	Cardiovascular diseases	Both	36970	57259	24.2%	68.2%	-86.5%	5.9%
		Female	14765	25190	24.1%	87.6%	-93%	18.7%
		Male	22213	31461	24.4%	51.8%	-78.8%	-2.7%

Location	Cause	Sex	Expected DALYs in 2019		% 1990 - 2019 DALYs change cause			% 1990 - 2019 DALYs overall change
			Population growth	Population growth + Aging	Population growth	Age structure change	DALYs rate change	
	Chronic respiratory diseases	Both	9197	12876	24.2%	49.7%	-63.6%	10.3%
		Female	3724	5538	24.1%	60.5%	-65.5%	19.1%
		Male	5475	7224	24.4%	39.7%	-59.7%	4.4%
	Digestive diseases	Both	8473	9410	24.2%	13.7%	-67%	-29%
		Female	3806	4363	24.1%	18.2%	-68.3%	-26.1%
		Male	4667	4982	24.4%	8.4%	-64.2%	-31.5%
	Neurological disorders	Both	8944	11709	24.2%	38.4%	-9.1%	53.5%
		Female	4658	6268	24.1%	42.9%	-2.6%	64.4%
		Male	4285	5423	24.4%	33%	-15.7%	41.7%
	Mental disorders	Both	16901	20076	24.2%	23.3%	0.6%	48.1%
		Female	9264	11099	24.1%	24.6%	1.3%	50%
		Male	7635	8920	24.4%	20.9%	0.6%	45.9%
	Substance use disorders	Both	1937	2661	24.2%	46.4%	38.9%	109.5%
		Female	733	970	24.1%	40.2%	-16.7%	47.7%
		Male	1205	1711	24.4%	52.2%	70.6%	147.3%
	Diabetes and kidney diseases	Both	7999	10598	24.2%	40.4%	10.3%	74.9%
		Female	3717	5276	24.1%	52%	22.1%	98.3%
		Male	4282	5299	24.4%	29.5%	0.7%	54.6%
	Skin and subcutaneous diseases	Both	3579	3420	24.2%	-5.5%	3%	21.7%
		Female	1868	1777	24.1%	-6.1%	2.6%	20.6%
		Male	1711	1642	24.4%	-5%	3.7%	23%
	Sense organ diseases	Both	5181	7812	24.2%	63.1%	-42.6%	44.7%
		Female	2475	3974	24.1%	75.1%	-45.6%	53.6%
		Male	2705	3836	24.4%	52%	-39.8%	36.5%
	Musculoskeletal disorders	Both	10497	15362	24.2%	57.6%	6.1%	87.9%
		Female	5503	8333	24.1%	63.8%	10.1%	98%
		Male	4993	7072	24.4%	51.8%	0.7%	76.8%
	Other non-communicable diseases	Both	54597	38170	24.2%	-37.4%	-48.7%	-61.8%
		Female	26177	20204	24.1%	-28.3%	-45.5%	-49.8%
		Male	28421	17869	24.4%	-46.2%	-51.2%	-73%
	**Injuries***	Both	73630	65299	25.6%	-14.2%	-69.7%	-58.2%
		Female	25903	21405	25.3%	-21.8%	-71.4%	-67.8%
		Male	47781	44266	26%	-9.3%	-69.7%	-53%
	Transport Injuries	Both	44241	38929	24.2%	-14.9%	-71.4%	-62.1%
		Female	15166	12165	24.1%	-24.6%	-72.1%	-72.6%
		Male	29090	26974	24.4%	-9%	-71.9%	-56.6%
	Unintentional Injuries*	Both	26701	22290	25.6%	-20.8%	-69.1%	-64.2%
		Female	9864	7881	25.3%	-25.2%	-69.3%	-69.2%
		Male	16853	14465	26%	-17.9%	-69.4%	-61.3%
	Self-harm and interpersonal violence	Both	3851	4634	24.2%	25.2%	-41.2%	8.2%
		Female	1372	1531	24.1%	14.3%	-59.8%	-21.3%
		Male	2480	3142	24.4%	33.2%	-32.9%	24.7%

Location	Cause	Sex	Expected DALYs in 2019		% 1990 - 2019 DALYs change cause			% 1990 - 2019 DALYs overall change
			Population growth	Population growth + Aging	Population growth	Age structure change	DALYs rate change	
**Tehran**	**All causes**	Both	3417111	3912354	63.8%	23.7%	-50%	37.5%
		Female	1604722	1889123	67.3%	29.7%	-46.2%	50.8%
		Male	1808698	2016797	60.5%	18.5%	-52.7%	26.3%
	**Communicable, maternal, neonatal, and nutritional diseases**	Both	871509	530009	63.8%	-64.2%	-55.8%	-56.2%
		Female	385585	237656	67.3%	-64.2%	-57.3%	-54.2%
		Male	484020	292356	60.5%	-63.6%	-54.7%	-57.8%
	HIV/AIDS and sexually transmitted infections	Both	4713	4801	63.8%	3.1%	640.2%	707%
		Female	2184	2301	67.3%	9%	472%	548.3%
		Male	2523	2489	60.5%	-2.2%	780.5%	838.9%
	Respiratory infections and tuberculosis	Both	117390	108792	63.8%	-12%	-83.4%	-31.5%
		Female	59042	51378	67.3%	-21.7%	-82.4%	-36.8%
		Male	58380	57082	60.5%	-3.6%	-83.4%	-26.4%
	Enteric infections	Both	44317	32677	63.8%	-43%	-12.5%	8.3%
		Female	21851	16161	67.3%	-43.6%	-14.4%	9.3%
		Male	22460	16389	60.5%	-43.4%	-9.8%	7.4%
	Neglected tropical diseases and malaria	Both	4628	3663	63.8%	-34.2%	-71.5%	-41.8%
		Female	2306	1914	67.3%	-28.5%	-78%	-39.2%
		Male	2322	1753	60.5%	-39.4%	-65.5%	-44.3%
	Other infectious diseases	Both	105006	63240	63.8%	-65.2%	-79.5%	-80.9%
		Female	56483	32182	67.3%	-72%	-79.4%	-84.1%
		Male	48701	30926	60.5%	-58.6%	-79.3%	-77.3%
	Maternal and neonatal disorders	Both	558387	286221	63.8%	-79.9%	-54.4%	-70.4%
		Female	224278	116899	67.3%	-80.1%	-53.5%	-66.3%
		Male	331964	169988	60.5%	-78.3%	-55.3%	-73.1%
	Nutritional deficiencies	Both	37069	30616	63.8%	-28.5%	-61.4%	-26.1%
		Female	19440	16821	67.3%	-22.5%	-65.9%	-21.2%
		Male	17671	13729	60.5%	-35.8%	-56%	-31.3%
	**Non-communicable diseases**	Both	2169356	3002455	63.8%	62.9%	-44.4%	82.4%
		Female	1095594	1532765	67.3%	66.8%	-40%	94.1%
		Male	1074541	1467888	60.5%	58.8%	-48.4%	70.9%
	Neoplasms	Both	209659	327397	63.8%	92%	-49.5%	106.4%
		Female	105102	165403	67.3%	96%	-51.6%	111.7%
		Male	104601	163379	60.5%	90.2%	-49.5%	101.2%
	Cardiovascular diseases	Both	333423	654068	63.8%	157.6%	-150.2%	71.2%
		Female	142703	279945	67.3%	160.9%	-145.9%	82.4%
		Male	189796	365695	60.5%	148.8%	-146.1%	63.2%
	Chronic respiratory diseases	Both	63988	104047	63.8%	102.6%	-74.4%	92%
		Female	29343	45980	67.3%	94.9%	-73.8%	88.4%
		Male	34548	57798	60.5%	108%	-73.6%	95%
	Digestive diseases	Both	72617	107324	63.8%	78.3%	-97.2%	44.9%
		Female	33990	49986	67.3%	78.7%	-96.7%	49.4%
		Male	38544	56192	60.5%	73.5%	-92.9%	41.2%

Location	Cause	Sex	Expected DALYs in 2019		% 1990 - 2019 DALYs change cause			% 1990 - 2019 DALYs overall change
			Population growth	Population growth + Aging	Population growth	Age structure change	DALYs rate change	
	Neurological disorders	Both	150378	207641	63.8%	62.4%	-15.5%	110.7%
		Female	83274	113301	67.3%	60.3%	-10.7%	117%
		Male	67456	95658	60.5%	67.1%	-24.4%	103.2%
	Mental disorders	Both	306200	362719	63.8%	30.2%	4.3%	98.4%
		Female	178265	215992	67.3%	35.4%	2%	104.7%
		Male	129005	149097	60.5%	25%	4.4%	89.9%
	Substance use disorders	Both	71181	83277	63.8%	27.8%	4.8%	96.4%
		Female	22914	26900	67.3%	29.1%	-29.7%	66.7%
		Male	47763	55367	60.5%	25.6%	24%	110.1%
	Diabetes and kidney diseases	Both	117323	222741	63.8%	147.2%	-3.6%	207.4%
		Female	61958	117124	67.3%	149%	-4.7%	211.6%
		Male	55517	104922	60.5%	142.9%	-0.4%	203%
	Skin and subcutaneous diseases	Both	60247	57997	63.8%	-6.1%	-1.3%	56.5%
		Female	31926	30716	67.3%	-6.3%	-2.7%	58.2%
		Male	28403	27239	60.5%	-6.6%	0.6%	54.5%
	Sense organ diseases	Both	73616	130361	63.8%	126.3%	-58.3%	131.8%
		Female	36787	64117	67.3%	124.3%	-58.1%	133.6%
		Male	36840	66212	60.5%	128%	-58.3%	130.2%
	Musculoskeletal disorders	Both	223675	360132	63.8%	99.9%	0.7%	164.5%
		Female	115402	189671	67.3%	107.7%	3.2%	178.2%
		Male	108453	171025	60.5%	92.6%	-2.7%	150.5%
	Other non-communicable diseases	Both	487049	384753	63.8%	-34.4%	-38.4%	-9%
		Female	253931	233630	67.3%	-13.4%	-34.5%	19.4%
		Male	233616	155304	60.5%	-53.8%	-45.3%	-38.6%
	**Injuries***	Both	370406	375958	61.2%	2.4%	-67.4%	-3.8%
		Female	120713	117484	64.5%	-4.4%	-65.2%	-5.1%
		Male	247294	254099	58.2%	4.4%	-65.7%	-3.1%
	Transport Injuries	Both	104753	99052	63.8%	-8.9%	-81.3%	-26.4%
		Female	31480	26723	67.3%	-25.3%	-85%	-42.9%
		Male	72440	71112	60.5%	-2.9%	-77.1%	-19.5%
	Unintentional Injuries*	Both	205018	208745	61.2%	2.9%	-61.1%	3.1%
		Female	75063	74561	64.5%	-1.1%	-55.7%	7.6%
		Male	128941	132046	58.2%	3.8%	-61.4%	0.6%
	Self-harm and interpersonal violence	Both	61589	68835	63.8%	19.3%	-69%	14.1%
		Female	14890	16439	67.3%	17.4%	-74.6%	10.1%
		Male	46062	51229	60.5%	18%	-63.2%	15.3%
**West Azarbayejan**	**All causes**	Both	1590228	1540213	46.6%	-4.6%	-65.1%	-23.1%
		Female	713597	698091	46.8%	-3.2%	-62.3%	-18.7%
		Male	876516	837603	46.5%	-6.5%	-66.7%	-26.7%
	**Communicable, maternal, neonatal, and nutritional diseases**	Both	575447	363236	46.6%	-54.1%	-72.8%	-80.2%
		Female	268919	170108	46.8%	-53.9%	-73.2%	-80.4%
		Male	306505	193109	46.5%	-54.2%	-72.4%	-80.1%

Location	Cause	Sex	Expected DALYs in 2019		% 1990 - 2019 DALYs change cause			% 1990 - 2019 DALYs overall change
			Population growth	Population growth + Aging	Population growth	Age structure change	DALYs rate change	
	HIV/AIDS and sexually transmitted infections	Both	1073	975	46.6%	-13.4%	50.1%	83.3%
		Female	542	531	46.8%	-2.9%	55%	98.9%
		Male	531	442	46.5%	-24.6%	45.5%	67.4%
	Respiratory infections and tuberculosis	Both	81061	56795	46.6%	-43.9%	-76.8%	-74.1%
		Female	39646	27150	46.8%	-46.3%	-76.6%	-76.1%
		Male	41415	29556	46.5%	-42%	-76.7%	-72.1%
	Enteric infections	Both	41383	27503	46.6%	-49.2%	-64.3%	-66.8%
		Female	21545	14192	46.8%	-50.1%	-65.7%	-69%
		Male	19839	13286	46.5%	-48.4%	-62.6%	-64.5%
	Neglected tropical diseases and malaria	Both	2541	2138	46.6%	-23.3%	-90%	-66.6%
		Female	1259	1100	46.8%	-18.6%	-94.5%	-66.3%
		Male	1282	1038	46.5%	-27.9%	-85.6%	-66.9%
	Other infectious diseases	Both	68624	43428	46.6%	-53.8%	-83.2%	-90.4%
		Female	36341	22508	46.8%	-55.9%	-82.4%	-91.5%
		Male	32287	20854	46.5%	-51.9%	-83.9%	-89.2%
	Maternal and neonatal disorders	Both	358464	215293	46.6%	-58.6%	-70.9%	-82.8%
		Female	157921	95422	46.8%	-58.1%	-71.1%	-82.5%
		Male	200513	120016	46.5%	-58.8%	-70.8%	-83.1%
	Nutritional deficiencies	Both	22302	17103	46.6%	-34.2%	-76.2%	-63.8%
		Female	11665	9205	46.8%	-31%	-79.1%	-63.3%
		Male	10637	7915	46.5%	-37.5%	-73.2%	-64.2%
	**Non-communicable diseases**	Both	733263	919210	46.6%	37.2%	-55%	28.8%
		Female	350145	447524	46.8%	40.8%	-49.9%	37.7%
		Male	383101	466880	46.5%	32%	-57.8%	20.7%
	Neoplasms	Both	71056	107043	46.6%	74.3%	-37%	83.9%
		Female	29751	44967	46.8%	75.1%	-33.5%	88.3%
		Male	41296	60429	46.5%	67.9%	-33.7%	80.7%
	Cardiovascular diseases	Both	159407	290895	46.6%	121%	-123.5%	44.1%
		Female	63707	125654	46.8%	142.7%	-131.5%	58%
		Male	95675	162841	46.5%	102.9%	-114.4%	34.9%
	Chronic respiratory diseases	Both	27087	42358	46.6%	82.7%	-89.5%	39.8%
		Female	11611	18832	46.8%	91.3%	-94.9%	43.1%
		Male	15472	23087	46.5%	72.1%	-81.4%	37.2%
	Digestive diseases	Both	19180	25833	46.6%	50.9%	-68.1%	29.4%
		Female	8810	11825	46.8%	50.2%	-65.1%	31.9%
		Male	10370	13869	46.5%	49.5%	-68.7%	27.3%
	Neurological disorders	Both	37759	47035	46.6%	36%	-14.2%	68.5%
		Female	20159	25726	46.8%	40.5%	-8.6%	78.7%
		Male	17602	21267	46.5%	30.5%	-20.2%	56.8%
	Mental disorders	Both	72600	86066	46.6%	27.2%	1.7%	75.5%
		Female	42593	51562	46.8%	30.9%	1.3%	79%
		Male	30018	34501	46.5%	21.9%	2.2%	70.6%

Location	Cause	Sex	Expected DALYs in 2019		% 1990 - 2019 DALYs change cause			% 1990 - 2019 DALYs overall change
			Population growth	Population growth + Aging	Population growth	Age structure change	DALYs rate change	
	Substance use disorders	Both	6437	8579	46.6%	48.8%	-2.7%	92.7%
		Female	2129	2742	46.8%	42.2%	-19.8%	69.2%
		Male	4306	5860	46.5%	52.9%	5%	104.4%
	Diabetes and kidney diseases	Both	19302	28551	46.6%	70.3%	74%	190.9%
		Female	9461	14523	46.8%	78.5%	88.9%	214.2%
		Male	9841	13992	46.5%	61.8%	60.3%	168.6%
	Skin and subcutaneous diseases	Both	14892	14105	46.6%	-7.8%	1.5%	40.4%
		Female	7790	7366	46.8%	-8%	1%	39.8%
		Male	7103	6733	46.5%	-7.6%	2.2%	41.1%
	Sense organ diseases	Both	18695	29832	46.6%	87.4%	-52.7%	81.3%
		Female	8928	15113	46.8%	101.7%	-56%	92.5%
		Male	9766	14720	46.5%	74.3%	-49.7%	71.1%
	Musculoskeletal disorders	Both	40654	64479	46.6%	85.9%	9%	141.5%
		Female	21408	34737	46.8%	91.4%	13.2%	151.4%
		Male	19249	29856	46.5%	80.7%	3.4%	130.6%
	Other non-communicable diseases	Both	246195	174435	46.6%	-42.7%	-59.7%	-55.8%
		Female	123797	94475	46.8%	-34.8%	-55.8%	-43.8%
		Male	122403	79726	46.5%	-51.1%	-63.4%	-68%
	**Injuries***	Both	270905	251448	45.4%	-10.4%	-74.7%	-39.8%
		Female	90680	78686	45.3%	-19.2%	-76.7%	-50.6%
		Male	180285	173194	45.5%	-5.7%	-74.1%	-34.3%
	Transport Injuries	Both	139551	127219	46.6%	-13%	-78.3%	-44.6%
		Female	41155	33801	46.8%	-26.2%	-78.9%	-58.4%
		Male	98350	93653	46.5%	-7%	-78.4%	-38.9%
	Unintentional Injuries*	Both	94218	82514	45.4%	-18.1%	-78.7%	-51.4%
		Female	35042	29044	45.3%	-24.9%	-79.1%	-58.7%
		Male	59192	53545	45.5%	-13.9%	-78.7%	-47.1%
	Self-harm and interpersonal violence	Both	43695	46201	46.6%	8.4%	-61.1%	-6.1%
		Female	16654	17010	46.8%	3.1%	-68.9%	-19%
		Male	27033	29217	46.5%	11.8%	-56.5%	1.9%
**Yazd**	**All causes**	Both	394058	410153	69.2%	6.9%	-63.9%	12.2%
		Female	176898	182209	70.3%	5.1%	-58.9%	16.4%
		Male	216983	228622	68.1%	9%	-68.4%	8.7%
	**Communicable, maternal, neonatal, and nutritional diseases**	Both	123053	81813	69.2%	-56.7%	-76.9%	-64.5%
		Female	57130	38229	70.3%	-56.3%	-77.9%	-64%
		Male	65892	43668	68.1%	-56.7%	-76.3%	-64.9%
	HIV/AIDS and sexually transmitted infections	Both	326	315	69.2%	-5.8%	123.7%	187%
		Female	163	168	70.3%	6.1%	123.5%	199.9%
		Male	163	147	68.1%	-17.3%	123.6%	174.4%
	Respiratory infections and tuberculosis	Both	19551	15631	69.2%	-33.9%	-84.6%	-49.4%
		Female	9574	7367	70.3%	-39.3%	-85.3%	-54.3%
		Male	9978	8303	68.1%	-28.2%	-84.7%	-44.7%

Location	Cause	Sex	Expected DALYs in 2019		% 1990 - 2019 DALYs change cause			% 1990 - 2019 DALYs overall change
			Population growth	Population growth + Aging	Population growth	Age structure change	DALYs rate change	
	Enteric infections	Both	7659	5400	69.2%	-49.9%	-55%	-35.7%
		Female	3562	2509	70.3%	-50.3%	-54.3%	-34.4%
		Male	4095	2889	68.1%	-49.5%	-55.4%	-36.8%
	Neglected tropical diseases and malaria	Both	2963	2618	69.2%	-19.7%	-140%	-90.5%
		Female	1505	1263	70.3%	-27.3%	-133.8%	-90.9%
		Male	1459	1356	68.1%	-11.9%	-146.4%	-90.2%
	Other infectious diseases	Both	10242	6900	69.2%	-55.2%	-96.1%	-82.2%
		Female	5478	3594	70.3%	-58.6%	-96.6%	-84.9%
		Male	4770	3305	68.1%	-51.7%	-95.6%	-79.2%
	Maternal and neonatal disorders	Both	77195	46748	69.2%	-66.7%	-72.5%	-70%
		Female	34225	21115	70.3%	-65.2%	-73%	-67.9%
		Male	42930	25697	68.1%	-67.5%	-72.3%	-71.7%
	Nutritional deficiencies	Both	5118	4202	69.2%	-30.3%	-85.5%	-46.7%
		Female	2624	2213	70.3%	-26.7%	-89.7%	-46.1%
		Male	2496	1972	68.1%	-35.3%	-80.1%	-47.2%
	**Non-communicable diseases**	Both	199251	253390	69.2%	46%	-42.7%	72.4%
		Female	97594	122729	70.3%	43.8%	-39.4%	74.7%
		Male	101669	131541	68.1%	49.4%	-47.2%	70.3%
	Neoplasms	Both	18241	25126	69.2%	63.8%	-8.3%	124.7%
		Female	8389	11325	70.3%	59.6%	-9.5%	120.3%
		Male	9847	14084	68.1%	72.4%	-12.1%	128.4%
	Cardiovascular diseases	Both	47154	78206	69.2%	111.4%	-125.4%	55.2%
		Female	20239	32916	70.3%	106.6%	-124.6%	52.3%
		Male	26882	45497	68.1%	116.4%	-127.2%	57.3%
	Chronic respiratory diseases	Both	8336	12060	69.2%	75.6%	-68.3%	76.5%
		Female	3441	4848	70.3%	69.6%	-69.1%	70.8%
		Male	4887	7427	68.1%	87.4%	-75.1%	80.4%
	Digestive diseases	Both	5260	7236	69.2%	63.5%	-66.3%	66.4%
		Female	2400	3201	70.3%	56.8%	-65.2%	61.8%
		Male	2858	4059	68.1%	70.6%	-68.6%	70.1%
	Neurological disorders	Both	12039	15490	69.2%	48.5%	-6.1%	111.6%
		Female	6489	8393	70.3%	50%	-3.5%	116.7%
		Male	5558	7124	68.1%	47.4%	-9.9%	105.7%
	Mental disorders	Both	21694	25658	69.2%	30.9%	4.1%	104.2%
		Female	11758	14224	70.3%	35.7%	1.6%	107.6%
		Male	9952	11483	68.1%	25.9%	6.1%	100.1%
	Substance use disorders	Both	2518	3409	69.2%	59.9%	47.5%	176.5%
		Female	731	979	70.3%	57.8%	1.8%	129.8%
		Male	1781	2410	68.1%	59.5%	67.8%	195.4%
	Diabetes and kidney diseases	Both	7510	11103	69.2%	80.9%	138.4%	288.5%
		Female	3657	5271	70.3%	75.2%	131.9%	277.3%
		Male	3854	5864	68.1%	87.7%	143.2%	299%

Location	Cause	Sex	Expected DALYs in 2019		% 1990 - 2019 DALYs change cause			% 1990 - 2019 DALYs overall change
			Population growth	Population growth + Aging	Population growth	Age structure change	DALYs rate change	
	Skin and subcutaneous diseases	Both	5042	4793	69.2%	-8.4%	3.6%	64.4%
		Female	2610	2476	70.3%	-8.8%	2.6%	64.1%
		Male	2434	2319	68.1%	-7.9%	4.5%	64.7%
	Sense organ diseases	Both	6605	9742	69.2%	80.3%	-57.1%	92.4%
		Female	3329	4809	70.3%	75.6%	-56.7%	89.2%
		Male	3277	4921	68.1%	84.3%	-56.8%	95.7%
	Musculoskeletal disorders	Both	14076	21185	69.2%	85.4%	15.7%	170.3%
		Female	7502	11197	70.3%	83.9%	20.4%	174.5%
		Male	6582	9957	68.1%	86.2%	11.2%	165.6%
	Other non-communicable diseases	Both	50776	39380	69.2%	-38%	-50.4%	-19.2%
		Female	27049	23089	70.3%	-24.9%	-48.1%	-2.8%
		Male	23757	16395	68.1%	-52.1%	-53.7%	-37.6%
	**Injuries***	Both	69073	72631	66.7%	8.6%	-97.4%	-22%
		Female	21545	20859	67.3%	-5.3%	-94%	-32%
		Male	47441	51579	66.1%	14.5%	-98.2%	-17.6%
	Transport Injuries	Both	35826	37408	69.2%	7.5%	-104.2%	-27.6%
		Female	10478	9753	70.3%	-11.8%	-97.7%	-39.2%
		Male	25262	27543	68.1%	15.2%	-106.1%	-22.8%
	Unintentional Injuries*	Both	20584	21000	66.7%	3.4%	-85.4%	-15.3%
		Female	7395	6945	67.3%	-10.2%	-86.6%	-29.5%
		Male	13169	14024	66.1%	10.8%	-84.3%	-7.4%
	Self-harm and interpersonal violence	Both	14830	16212	69.2%	15.8%	-110.8%	-25.9%
		Female	4072	4466	70.3%	16.5%	-107.5%	-20.8%
		Male	10719	11614	68.1%	14%	-110%	-27.8%
**Zanjan**	**All causes**	Both	1320475	1178642	21.6%	-13.1%	-85.5%	-77%
		Female	579912	493397	22.1%	-18.2%	-79.6%	-75.7%
		Male	739912	681157	21%	-9.6%	-89.5%	-78.1%
	**Communicable, maternal, neonatal, and nutritional diseases**	Both	187715	107967	21.6%	-51.6%	-54.6%	-84.7%
		Female	85580	49024	22.1%	-52.2%	-54.9%	-84.9%
		Male	102072	58957	21%	-51.1%	-54.4%	-84.5%
	HIV/AIDS and sexually transmitted infections	Both	336	310	21.6%	-9.4%	54.6%	66.7%
		Female	171	172	22.1%	0.6%	73.9%	96.6%
		Male	165	136	21%	-21.2%	36.3%	36.1%
	Respiratory infections and tuberculosis	Both	26675	17180	21.6%	-43.3%	-57.8%	-79.5%
		Female	13260	8166	22.1%	-46.9%	-58.2%	-83%
		Male	13416	9012	21%	-39.7%	-57.3%	-76%
	Enteric infections	Both	9943	6323	21.6%	-44.3%	-44.9%	-67.6%
		Female	4859	3067	22.1%	-45%	-45.3%	-68.3%
		Male	5083	3245	21%	-43.8%	-44.3%	-67%
	Neglected tropical diseases and malaria	Both	1762	1585	21.6%	-12.2%	-96.9%	-87.5%
		Female	867	775	22.1%	-13.1%	-97.4%	-88.3%
		Male	895	806	21%	-12%	-95.8%	-86.8%

Location	Cause	Sex	Expected DALYs in 2019		% 1990 - 2019 DALYs change cause			% 1990 - 2019 DALYs overall change
			Population growth	Population growth + Aging	Population growth	Age structure change	DALYs rate change	
	Other infectious diseases	Both	14319	8453	21.6%	-49.8%	-62.1%	-90.3%
		Female	7596	4363	22.1%	-52%	-62%	-91.9%
		Male	6728	4078	21%	-47.7%	-62%	-88.6%
	Maternal and neonatal disorders	Both	129717	70209	21.6%	-55.8%	-53.5%	-87.7%
		Female	56176	30302	22.1%	-56.2%	-53.4%	-87.5%
		Male	73470	39961	21%	-55.2%	-53.6%	-87.8%
	Nutritional deficiencies	Both	4963	3908	21.6%	-25.8%	-57.5%	-61.8%
		Female	2650	2179	22.1%	-21.7%	-61.2%	-60.8%
		Male	2315	1720	21%	-31.1%	-52.9%	-63%
	**Non-communicable diseases**	Both	187550	253960	21.6%	43%	-38.1%	26.4%
		Female	91226	123858	22.1%	43.7%	-37.2%	28.6%
		Male	96313	128550	21%	40.5%	-37.1%	24.4%
	Neoplasms	Both	16161	25045	21.6%	66.8%	-25.5%	62.9%
		Female	6641	10249	22.1%	66.4%	-25.9%	62.6%
		Male	9508	14484	21%	63.3%	-21.3%	63.1%
	Cardiovascular diseases	Both	41924	82231	21.6%	116.9%	-95.6%	42.8%
		Female	16281	34414	22.1%	136%	-108.7%	49.4%
		Male	25603	47045	21%	101.3%	-83.7%	38.6%
	Chronic respiratory diseases	Both	7004	11330	21.6%	75.1%	-52.3%	44.3%
		Female	3058	5112	22.1%	82%	-61.4%	42.7%
		Male	3942	6148	21%	67.7%	-43.2%	45.5%
	Digestive diseases	Both	5417	7568	21.6%	48.3%	-46.8%	23%
		Female	2510	3469	22.1%	46.7%	-52.2%	16.6%
		Male	2906	4061	21%	48.1%	-40.7%	28.4%
	Neurological disorders	Both	11000	15133	21.6%	45.7%	-8%	59.2%
		Female	5969	8333	22.1%	48.4%	-4.4%	66.1%
		Male	5037	6745	21%	41.1%	-11%	51.1%
	Mental disorders	Both	21393	26501	21.6%	29%	3.8%	54.4%
		Female	11815	14876	22.1%	31.6%	3.6%	57.4%
		Male	9589	11567	21%	25%	4.7%	50.7%
	Substance use disorders	Both	2050	3000	21.6%	56.3%	66.2%	144.1%
		Female	652	915	22.1%	49.1%	5.1%	76.3%
		Male	1395	2118	21%	62.8%	91.7%	175.5%
	Diabetes and kidney diseases	Both	4806	7530	21.6%	68.9%	56.8%	147.2%
		Female	2301	3691	22.1%	73.8%	60.8%	156.7%
		Male	2504	3825	21%	63.8%	53.7%	138.5%
	Skin and subcutaneous diseases	Both	4683	4368	21.6%	-8.2%	0.8%	14.2%
		Female	2452	2285	22.1%	-8.3%	0.5%	14.3%
		Male	2232	2080	21%	-8.3%	1.3%	14%
	Sense organ diseases	Both	6145	10662	21.6%	89.3%	-48.9%	62%
		Female	2982	5465	22.1%	101.7%	-52.2%	71.6%
		Male	3163	5184	21%	77.3%	-45.3%	53.1%

Location	Cause	Sex	Expected DALYs in 2019		% 1990 - 2019 DALYs change cause			% 1990 - 2019 DALYs overall change
			Population growth	Population growth + Aging	Population growth	Age structure change	DALYs rate change	
	Musculoskeletal disorders	Both	12287	20957	21.6%	85.8%	10.9%	118.2%
		Female	6529	11331	22.1%	89.8%	15%	126.9%
		Male	5762	9636	21%	81.4%	6.1%	108.5%
	Other non-communicable diseases	Both	54679	39636	21.6%	-33.4%	-43%	-54.9%
		Female	30036	23717	22.1%	-25.7%	-41.2%	-44.8%
		Male	24671	15655	21%	-44.2%	-43.9%	-67.1%
	**Injuries***	Both	83575	83034	21.7%	-0.8%	-75.8%	-54.8%
		Female	30154	26874	21.8%	-13.3%	-72.5%	-64%
		Male	53404	56696	21.7%	7.5%	-78.9%	-49.7%
	Transport Injuries	Both	44380	42437	21.6%	-5.3%	-78.4%	-62.1%
		Female	15790	12706	22.1%	-23.9%	-72%	-73.7%
		Male	28535	29944	21%	6%	-82.8%	-55.8%
	Unintentional Injuries*	Both	31384	31229	21.7%	-0.6%	-73.7%	-52.5%
		Female	12058	11275	21.8%	-7.9%	-73.8%	-59.9%
		Male	19321	20140	21.7%	5.2%	-74.7%	-47.9%
	Self-harm and interpersonal violence	Both	9851	10708	21.6%	10.6%	-71.2%	-39.1%
		Female	4072	3978	22.1%	-2.8%	-72.6%	-53.3%
		Male	5773	6743	21%	20.3%	-70.5%	-29.1%

DALYs= disability-adjusted life-years

*Analysis for these categories are provided for the 1991 – 2019 period. (Due to the major impact of the 1990's Rudbar earthquake on DALY rates)

Appendix Table 7. National DALYs and death due to causes, number for all ages and age-standardised rate (per 100,000) in 1990 and 2019 with percent changes between 1990 and 2019, by sex

Cause	Sex	1990				2019				1990-2019 percent change (%)			
		DALYs		Death		DALYs		Death		DALYs		Death	
		Number	Rate	Number	Rate	Number	Rate	Number	Rate	Number	Rate	Number	Rate
**All causes**	Both	25007732 (22720649 to 27393140)	46800.5 (43063.5 to 50769.4)	357195 (335408 to 380401)	1065.2 (1029.2 to 1102.5)	19828721 (17282464 to 22648468)	25029.1 (22059.7 to 28319)	391113 (381513 to 402132)	594.4 (580.3 to 610.3)	-20.7 (-29 to -12.3)	-46.5 (-50.5 to -42.6)	9.5 (2.6 to 16.9)	-44.2 (-46.5 to -41.8)
	Female	10983762 (9898766 to 12100707)	42059.7 (38261.2 to 46293.3)	146493 (137227 to 157039)	918.7 (886.3 to 957.8)	9250748 (7864001 to 10840161)	23586.3 (20383.2 to 27306.8)	166629 (161052 to 172274)	533.4 (516.5 to 550.6)	-15.8 (-25.2 to -6)	-43.9 (-48.4 to -39.5)	13.7 (5.7 to 22.1)	-41.9 (-44.8 to -39.1)
	Male	14023970 (12815516 to 15272283)	51205.5 (47518.5 to 55210.8)	210702 (197115 to 224517)	1202 (1150.1 to 1254.2)	10577972 (9421430 to 11813136)	26472.6 (23767.4 to 29387.2)	224485 (216637 to 233407)	656.6 (634.3 to 682.2)	-24.6 (-31.9 to -17)	-48.3 (-51.9 to -44.7)	6.5 (-0.9 to 14.1)	-45.4 (-48.3 to -42.3)
**Communicable, maternal, neonatal, and nutritional diseases**	Both	7748056 (6441086 to 9286747)	9690.7 (8175.4 to 11484.5)	88010 (73390 to 105909)	130 (112.3 to 152.6)	1775950 (1546039 to 2025327)	2434.5 (2114.8 to 2782.5)	23415 (21191 to 25721)	35.7 (32.1 to 39.3)	-77.1 (-81.8 to -71.3)	-74.9 (-79.9 to -69.1)	-73.4 (-78.6 to -67.1)	-72.6 (-77.2 to -67.2)
	Female	3548250 (2947918 to 4248557)	9113.5 (7689.2 to 10830.6)	39957 (33271 to 48066)	120.2 (103.5 to 140.5)	807999 (700852 to 926775)	2259.1 (1965.8 to 2585.3)	10162 (9046 to 11294)	32.2 (28.5 to 36.1)	-77.2 (-82 to -71.4)	-75.2 (-80.2 to -69.4)	-74.6 (-79.8 to -68.3)	-73.2 (-78 to -68)
	Male	4199805 (3452838 to 5119172)	10231.9 (8489.6 to 12411.5)	48054 (39468 to 58828)	139.4 (118.2 to 165.6)	967952 (842987 to 1105445)	2602.9 (2255.3 to 2983.4)	13253 (11864 to 14624)	39.1 (35 to 43.2)	-77 (-82.1 to -70.9)	-74.6 (-80.2 to -68)	-72.4 (-78.2 to -65.2)	-72 (-77.2 to -66)
HIV/AIDS and sexually transmitted infections	Both	18537 (10238 to 33201)	29.9 (18.5 to 48.3)	167 (80 to 337)	0.3 (0.2 to 0.5)	80724 (61406 to 108040)	89 (66.5 to 118.3)	1285 (956 to 1829)	1.4 (1.1 to 2)	335.5 (138.2 to 704)	197.6 (87.4 to 368.7)	667.4 (261.8 to 1666.6)	417.9 (185.1 to 866.2)
	Female	8993 (4970 to 16125)	30.9 (18.7 to 50.3)	69 (31 to 146)	0.2 (0.1 to 0.4)	33672 (23272 to 46862)	78.2 (53 to 108.4)	487 (306 to 751)	1.2 (0.7 to 1.8)	274.4 (109 to 577.6)	153.1 (60.7 to 328.8)	602.1 (204.1 to 1742.9)	404.2 (149.6 to 952.7)
	Male	9544 (5093 to 17805)	28.9 (17.9 to 47.4)	98 (47 to 195)	0.3 (0.2 to 0.5)	47052 (37962 to 63009)	99.4 (79.1 to 130)	798 (624 to 1137)	1.7 (1.3 to 2.4)	393 (166.7 to 834.8)	243.6 (106.8 to 454.6)	713.5 (308.7 to 1637.1)	432.6 (202 to 917.8)
HIV/AIDS	Both	2573 (1250 to 3630)	5.4 (2.5 to 7.6)	38 (13 to 58)	0.1 (0 to 0.1)	64050 (47949 to 88445)	69 (51 to 94.6)	1177 (852 to 1714)	1.3 (0.9 to 1.8)	2389.4 (1377 to 5101.7)	1182 (653.4 to 2761.4)	2959.3 (1558.9 to 8817.2)	1301.9 (654.7 to 4176.8)
	Female	668 (337 to 959)	2.5 (1.3 to 3.6)	9 (3 to 14)	0 (0 to 0.1)	23723 (14635 to 35007)	55 (33.8 to 80.3)	432 (255 to 694)	1 (0.6 to 1.6)	3449.7 (1762.9 to 7375.1)	2076.8 (1041.2 to 4505.7)	4525.3 (2020.4 to 12450)	2325.2 (1027.9 to 6980)
	Male	1905 (926 to 2690)	8.2 (3.6 to 11.7)	29 (9 to 44)	0.1 (0 to 0.2)	40328 (31923 to 55453)	82.6 (65.1 to 111.3)	744 (579 to 1083)	1.5 (1.2 to 2.2)	2017.4 (1231.1 to 4580.3)	912.1 (534.4 to 2218.8)	2456.3 (1375.9 to 8118.4)	1004.4 (540.3 to 3456.7)
Sexually transmitted infections excluding HIV	Both	15964 (7797 to 30678)	24.5 (13.6 to 43.4)	129 (45 to 300)	0.2 (0.1 to 0.4)	16674 (10405 to 26401)	20 (12 to 32.6)	109 (53 to 186)	0.2 (0.1 to 0.3)	4.4 (-30.9 to 59.3)	-18.5 (-36.2 to -1.7)	-15.9 (-45.5 to 60.8)	-11.2 (-38.9 to 32.1)
	Female	8324 (4331 to 15561)	28.4 (16.3 to 47.6)	60 (22 to 136)	0.2 (0.1 to 0.4)	9949 (6010 to 15773)	23.2 (14 to 37.6)	55 (31 to 89)	0.2 (0.1 to 0.3)	19.5 (-19.8 to 71.9)	-18.1 (-33.9 to -4.3)	-9 (-43 to 74.4)	-9.9 (-38.8 to 30.6)
	Male	7640 (3344 to 16110)	20.8 (10.5 to 39.9)	69 (23 to 164)	0.2 (0.1 to 0.4)	6725 (4052 to 10896)	16.8 (9.6 to 28)	54 (20 to 96)	0.2 (0.1 to 0.3)	-12 (-42.5 to 41.4)	-19.1 (-42.6 to 4.3)	-21.9 (-54.7 to 59.3)	-10.5 (-46.8 to 53.8)
Respiratory infections and tuberculosis	Both	1313446 (1055226 to 1730395)	1839.6 (1542.4 to 2308.1)	17097 (14244 to 21606)	37.8 (34 to 42.7)	377693 (331630 to 436634)	506.9 (448.7 to 579.7)	11054 (9932 to 11861)	17.6 (15.7 to 19)	-71.2 (-78.8 to -63.2)	-72.4 (-78.7 to -66.3)	-35.3 (-49.1 to -21.4)	-53.4 (-59.7 to -48)
	Female	633578 (497082 to 842176)	1767.6 (1444 to 2237.1)	8066 (6534 to 10335)	34.7 (30.4 to 40)	163040 (141189 to 191985)	452 (394.9 to 524.2)	4795 (4037 to 5283)	16.1 (13.4 to 17.8)	-74.3 (-81.5 to -65.9)	-74.4 (-80.6 to -67.6)	-40.6 (-54.6 to -25.7)	-53.7 (-61.6 to -46.8)

Cause	Sex	1990				2019				1990-2019 percent change (%)			
		DALYs		Death		DALYs		Death		DALYs		Death	
		Number	Rate	Number	Rate	Number	Rate	Number	Rate	Number	Rate	Number	Rate
	Male	679868 (540409 to 904508)	1908.7 (1587.1 to 2391.3)	9031 (7488 to 11504)	41.1 (36.6 to 47)	214653 (190116 to 247354)	561.2 (498.8 to 642.1)	6259 (5634 to 6765)	19.2 (17.1 to 20.8)	-68.4 (-76.8 to -59.5)	-70.6 (-77.3 to -64)	-30.7 (-46.3 to -15.1)	-53.3 (-59.8 to -47.1)
Tuberculosis	Both	75460 (61470 to 86349)	156.9 (127 to 178.1)	1484 (1178 to 1694)	4.8 (3.7 to 5.7)	27155 (25111 to 29853)	33.7 (31.3 to 37.3)	818 (756 to 917)	1.2 (1.1 to 1.3)	-64 (-69.1 to -52.3)	-78.5 (-81.4 to -70.9)	-44.9 (-52.8 to -22.7)	-75.6 (-79.7 to -64.8)
	Female	33966 (19924 to 42205)	137.1 (77.8 to 175.8)	621 (330 to 813)	4 (2.1 to 5.6)	11016 (9813 to 13649)	28.4 (25.3 to 35.2)	334 (297 to 429)	1 (0.9 to 1.3)	-67.6 (-75 to -33.4)	-79.3 (-84.3 to -55.1)	-46.2 (-60.8 to 31.3)	-75.2 (-82.8 to -39.1)
	Male	41495 (36764 to 48467)	175.8 (159.8 to 197.6)	864 (780 to 976)	5.6 (4.9 to 6.4)	16139 (14952 to 17450)	39 (36 to 42.2)	484 (448 to 523)	1.3 (1.2 to 1.5)	-61.1 (-66.7 to -55.6)	-77.8 (-80.4 to -75.2)	-43.9 (-50.6 to -36.4)	-76 (-78.8 to -71.8)
Lower respiratory infections	Both	1161357 (912538 to 1571576)	1563.8 (1280.6 to 2009.3)	15595 (12752 to 20079)	33 (29.4 to 37.7)	255439 (232950 to 277268)	359 (326.5 to 390.5)	10219 (9150 to 10998)	16.4 (14.5 to 17.8)	-78 (-84.1 to -71.1)	-77 (-82.8 to -71)	-34.5 (-49.5 to -18.6)	-50.2 (-57.6 to -43.8)
	Female	563605 (432596 to 766960)	1516.3 (1212.9 to 1984.2)	7437 (5953 to 9712)	30.6 (26.3 to 35.7)	107342 (94521 to 118783)	314.4 (275 to 349.2)	4455 (3723 to 4913)	15 (12.4 to 16.7)	-81 (-86.6 to -74.1)	-79.3 (-84.8 to -72.9)	-40.1 (-55.5 to -23.6)	-50.8 (-59.8 to -42.7)
	Male	597752 (463852 to 815184)	1609.5 (1312.3 to 2091.7)	8158 (6646 to 10605)	35.4 (31.2 to 41.1)	148097 (134082 to 160787)	403.3 (365.6 to 438.5)	5764 (5161 to 6240)	17.8 (15.8 to 19.3)	-75.2 (-82.5 to -67.2)	-74.9 (-81.3 to -68.2)	-29.3 (-46.4 to -12)	-49.8 (-57.4 to -42.5)
Upper respiratory infections	Both	54947 (33324 to 86601)	86 (52.6 to 133.8)	18 (2 to 37)	0 (0 to 0.1)	71512 (43154 to 112357)	85.5 (51.8 to 134.2)	17 (1 to 33)	0 (0 to 0.1)	30.1 (21.7 to 40.1)	-0.6 (-3.4 to 2.6)	-8.2 (-90.2 to 534)	-42.2 (-90.9 to 191)
	Female	26431 (15922 to 41887)	84.2 (51.5 to 131.6)	9 (1 to 20)	0 (0 to 0.1)	34169 (20516 to 53714)	83.3 (50.1 to 130.9)	6 (0 to 12)	0 (0 to 0)	29.3 (20.1 to 39.4)	-1 (-4.8 to 2.2)	-33 (-92.9 to 479.1)	-53.4 (-92.5 to 181.1)
	Male	28516 (17338 to 45031)	87.8 (53.7 to 136.6)	10 (1 to 19)	0 (0 to 0.1)	37343 (22637 to 58643)	87.7 (53.3 to 137.5)	11 (1 to 22)	0 (0 to 0.1)	31 (22.4 to 41.2)	-0.1 (-3.3 to 3.5)	14.1 (-88.5 to 653.8)	-34.7 (-90 to 227.3)
Otitis media	Both	21682 (12415 to 35510)	32.9 (19 to 54.2)	0 (0 to 0)	0 (0 to 0)	23587 (13369 to 39224)	28.7 (16 to 47.4)	0 (0 to 0)	0 (0 to 0)	8.8 (0.2 to 19)	-12.7 (-16.1 to -9.7)	-41.9 (-77.5 to 31.5)	-75.6 (-89.5 to -31.1)
	Female	9577 (5527 to 15601)	30.1 (17.1 to 49.4)	0 (0 to 0)	0 (0 to 0)	10513 (5871 to 17426)	25.9 (14.7 to 43.1)	0 (0 to 0)	0 (0 to 0)	9.8 (0.6 to 20.5)	-13.7 (-18 to -9.5)	-18 (-70.3 to 172.2)	-74.1 (-90.2 to -12.7)
	Male	12105 (6800 to 19931)	35.6 (20.2 to 58)	0 (0 to 0)	0 (0 to 0)	13074 (7385 to 21845)	31.3 (17.4 to 52.3)	0 (0 to 0)	0 (0 to 0)	8 (-1.1 to 18.3)	-11.9 (-16 to -8.2)	-78.6 (-86 to -57)	-80.9 (-86.8 to -61.4)
Enteric infections	Both	510353 (390319 to 675430)	667 (517.1 to 861.1)	5497 (4095 to 7385)	9.5 (6.9 to 13)	208261 (158547 to 265627)	273.7 (208.5 to 348.5)	1260 (932 to 1819)	1.9 (1.4 to 2.9)	-59.2 (-72 to -43.3)	-59 (-70.9 to -44.7)	-77.1 (-84.8 to -65.4)	-79.9 (-85 to -72.2)
	Female	239640 (174407 to 317370)	642.3 (477.3 to 842.2)	2592 (1800 to 3569)	9.6 (5.8 to 15.6)	99270 (74941 to 128473)	265.8 (202.1 to 343.1)	585 (358 to 1027)	1.9 (1.1 to 3.4)	-58.6 (-71.7 to -39.8)	-58.6 (-70.9 to -41.3)	-77.4 (-87.1 to -61.6)	-80.5 (-87.1 to -69.1)
	Male	270712 (204979 to 369212)	689 (532.2 to 915)	2905 (2146 to 4059)	9.3 (6.9 to 12.8)	108991 (81686 to 138997)	281.4 (211.6 to 357.7)	675 (503 to 1083)	2 (1.5 to 3.3)	-59.7 (-72.8 to -44)	-59.2 (-71.3 to -44.8)	-76.8 (-85.2 to -62.2)	-78.8 (-85.1 to -70.1)
Diarrheal diseases	Both	458635 (345077 to 606146)	600.4 (461.4 to 776.5)	4802 (3502 to 6460)	8.5 (6 to 12)	188226 (138449 to 244021)	247.3 (184.3 to 320.4)	962 (662 to 1525)	1.5 (1 to 2.5)	-59 (-72.6 to -41.6)	-58.8 (-71.3 to -43.4)	-80 (-87.4 to -67.7)	-82.1 (-87.2 to -74.2)
	Female	214629 (153810 to 288135)	576.8 (417.2 to 763.2)	2257 (1512 to 3132)	8.6 (4.9 to 14.4)	90223 (66382 to 118593)	241.5 (176.8 to 317.4)	449 (225 to 878)	1.5 (0.7 to 3)	-58 (-72 to -36.9)	-58.1 (-71.4 to -39.3)	-80.1 (-90.2 to -62.4)	-82.6 (-89.5 to -70.9)
	Male	244006 (180633 to 336695)	621.4 (467.2 to 829.1)	2545 (1786 to 3564)	8.3 (5.8 to 11.8)	98003 (72733 to 126817)	253 (187.1 to 326.7)	513 (346 to 909)	1.6 (1.1 to 2.9)	-59.8 (-73.7 to -42.3)	-59.3 (-72.1 to -43.6)	-79.9 (-87.8 to -64.6)	-81.2 (-87.2 to -72.2)
Typhoid and paratyphoid	Both	40140 (16440 to 80329)	51.8 (21.6 to 103.3)	541 (218 to 1085)	0.7 (0.3 to 1.5)	14793 (6023 to 28846)	19.6 (8 to 37.9)	212 (88 to 408)	0.3 (0.1 to 0.5)	-63.1 (-71.6 to -55.2)	-62.2 (-70.6 to -55)	-60.7 (-69 to -52.6)	-62.8 (-69.9 to -56.5)
	Female	18222 (7355 to 36248)	48.1 (19.7 to 95.5)	245 (99 to 493)	0.7 (0.3 to 1.4)	6703 (2743 to 13012)	18.1 (7.5 to 35.6)	96 (40 to 185)	0.3 (0.1 to 0.5)	-63.2 (-72 to -54.4)	-62.2 (-70.9 to -53.9)	-60.7 (-69.6 to -51.6)	-63 (-70.5 to -55.7)
	Male	21918 (8925 to 43414)	55.4 (23.1 to 110.5)	295 (121 to 592)	0.8 (0.3 to 1.5)	8090 (3316 to 15780)	21 (8.5 to 40.5)	116 (49 to 223)	0.3 (0.1 to 0.6)	-63.1 (-71.6 to -55.2)	-62.2 (-70.4 to -54.6)	-60.7 (-69.2 to -52.5)	-62.7 (-69.8 to -56.1)

Cause	Sex	1990				2019				1990-2019 percent change (%)			
		DALYs		Death		DALYs		Death		DALYs		Death	
		Number	Rate	Number	Rate	Number	Rate	Number	Rate	Number	Rate	Number	Rate
Invasive Non-typhoidal Salmonella (iNTS)	Both	9718 (5164 to 16543)	12.6 (6.8 to 20.9)	133 (72 to 222)	0.2 (0.1 to 0.3)	4907 (2497 to 8685)	6.4 (3.2 to 11.3)	82 (43 to 140)	0.1 (0.1 to 0.2)	-49.5 (-67.4 to -31.4)	-48.9 (-65.1 to -33.3)	-38.6 (-56.9 to -19.7)	-47.8 (-60.4 to -36.5)
	Female	5728 (2951 to 9797)	14.9 (7.9 to 24.8)	77 (41 to 130)	0.2 (0.1 to 0.4)	2180 (1103 to 3896)	5.8 (2.9 to 10.3)	37 (19 to 64)	0.1 (0.1 to 0.2)	-61.9 (-76 to -46.1)	-61.1 (-74.4 to -47.4)	-51.5 (-67 to -33.9)	-58.2 (-68.7 to -47.5)
	Male	3990 (2123 to 6847)	10.3 (5.6 to 17.2)	56 (30 to 93)	0.2 (0.1 to 0.3)	2727 (1371 to 4839)	7 (3.5 to 12.3)	44 (23 to 77)	0.1 (0.1 to 0.2)	-31.6 (-55.5 to -7.6)	-32 (-54.5 to -10.9)	-20.8 (-43.6 to 2.4)	-33.9 (-50.1 to -18.3)
Other intestinal infectious diseases	Both	1861 (470 to 3724)	2.2 (0.6 to 4.3)	21 (5 to 43)	0 (0 to 0.1)	335 (220 to 482)	0.4 (0.3 to 0.6)	3 (2 to 5)	0 (0 to 0)	-82 (-92.2 to -26.2)	-80.1 (-91.3 to -27)	-83.8 (-93.7 to -28.2)	-82.8 (-93 to -45.6)
	Female	1062 (120 to 2602)	2.5 (0.4 to 6.1)	12 (1 to 30)	0 (0 to 0.1)	163 (89 to 257)	0.4 (0.2 to 0.7)	2 (1 to 3)	0 (0 to 0)	-84.6 (-94.9 to 45.8)	-82.6 (-94.3 to 26.4)	-85.8 (-96.2 to 78.7)	-84.2 (-95.5 to -5.1)
	Male	799 (129 to 1924)	1.9 (0.4 to 4.3)	9 (1 to 22)	0 (0 to 0.1)	171 (99 to 258)	0.4 (0.2 to 0.7)	2 (1 to 3)	0 (0 to 0)	-78.6 (-92.3 to 40.7)	-76.8 (-91.7 to 14.5)	-81.1 (-94.8 to 51.5)	-80.8 (-94.1 to -13.6)
Neglected tropical diseases and malaria	Both	168733 (42037 to 483563)	278.1 (64.5 to 884.6)	2530 (364 to 8682)	5.3 (0.7 to 19.9)	14518 (10295 to 19966)	18.3 (13 to 25.2)	89 (54 to 150)	0.1 (0.1 to 0.2)	-91.4 (-97.1 to -66.3)	-93.4 (-97.9 to -72.2)	-96.5 (-99 to -76.9)	-97.7 (-99.4 to -82.6)
	Female	77061 (21065 to 208395)	254.5 (66.3 to 767.3)	1079 (162 to 3592)	4.4 (0.6 to 16.3)	7169 (5030 to 9620)	18.3 (12.8 to 24.8)	37 (19 to 58)	0.1 (0.1 to 0.2)	-90.7 (-96.8 to -66.7)	-92.8 (-97.6 to -73.5)	-96.6 (-99 to -78.6)	-97.6 (-99.3 to -83.4)
	Male	91672 (20910 to 281974)	300.8 (60.8 to 1000.6)	1451 (194 to 5106)	6.2 (0.7 to 24.1)	7349 (5050 to 10691)	18.2 (12.5 to 26.7)	51 (27 to 94)	0.1 (0.1 to 0.2)	-92 (-97.5 to -64.9)	-93.9 (-98.2 to -70.1)	-96.5 (-99.1 to -75.3)	-97.8 (-99.5 to -82.5)
Malaria	Both	134019 (14144 to 447965)	228.2 (21.8 to 840.1)	2268 (182 to 8463)	4.8 (0.3 to 19.5)	971 (718 to 1285)	1.1 (0.8 to 1.5)	0 (0 to 0)	0 (0 to 0)	-99.3 (-99.8 to -93)	-99.5 (-99.9 to -94.8)	-100 (-100 to -99.9)	-100 (-100 to -100)
	Female	61161 (7489 to 186402)	206 (23.2 to 723.4)	980 (90 to 3527)	4 (0.3 to 16)	493 (365 to 648)	1.2 (0.9 to 1.5)	0 (0 to 0)	0 (0 to 0)	-99.2 (-99.8 to -93.2)	-99.4 (-99.8 to -94.9)	-100 (-100 to -99.9)	-100 (-100 to -100)
	Male	72858 (6816 to 263450)	249.6 (20.6 to 966.8)	1289 (93 to 5008)	5.7 (0.3 to 23.8)	478 (352 to 638)	1.1 (0.8 to 1.5)	0 (0 to 0)	0 (0 to 0)	-99.3 (-99.8 to -93)	-99.6 (-99.9 to -94.8)	-100 (-100 to -99.9)	-100 (-100 to -100)
Leishmaniasis	Both	8100 (80 to 85082)	10.7 (0.1 to 106.7)	109 (0 to 1126)	0.2 (0 to 1.5)	558 (49 to 4533)	0.7 (0.1 to 5.8)	7 (0 to 67)	0 (0 to 0.1)	-93.1 (-94.9 to 35.1)	-93.5 (-95.1 to 9.7)	-93.2 (-94.1 to -89.5)	-94.5 (-95.4 to -93.6)
	Female	2755 (40 to 27815)	7.4 (0.1 to 70.5)	36 (0 to 365)	0.1 (0 to 1)	210 (30 to 1392)	0.5 (0.1 to 3.6)	2 (0 to 21)	0 (0 to 0.1)	-92.4 (-95.1 to 85.7)	-92.9 (-95.3 to 40)	-93.5 (-94.5 to -89.2)	-94.7 (-95.7 to -93.5)
	Male	5345 (39 to 56806)	13.9 (0.1 to 141.6)	73 (0 to 761)	0.2 (0 to 2.1)	348 (20 to 3116)	0.8 (0 to 7.7)	5 (0 to 47)	0 (0 to 0.1)	-93.5 (-94.8 to -3.4)	-93.9 (-95.2 to -11.1)	-93.1 (-94 to -89.3)	-94.4 (-95.3 to -93.4)
Schistosomiasis	Both	525 (315 to 875)	1.1 (0.7 to 1.8)	4 (2 to 6)	0 (0 to 0)	127 (74 to 195)	0.1 (0.1 to 0.2)	3 (1 to 5)	0 (0 to 0)	-75.8 (-86.7 to -55.4)	-86.4 (-92.5 to -75.5)	-25.7 (-71.6 to 90.1)	-73.4 (-90.5 to -26.1)
	Female	281 (159 to 473)	1.2 (0.7 to 2)	2 (0 to 3)	0 (0 to 0)	53 (27 to 94)	0.1 (0.1 to 0.2)	1 (0 to 3)	0 (0 to 0)	-81.1 (-90.5 to -62.2)	-89.1 (-94.8 to -77.6)	-17.1 (-82.4 to 232.5)	-71.4 (-94 to 36)
	Male	244 (128 to 416)	1 (0.5 to 1.7)	2 (1 to 5)	0 (0 to 0)	74 (36 to 126)	0.2 (0.1 to 0.3)	2 (0 to 3)	0 (0 to 0)	-69.7 (-85.8 to -33)	-83.2 (-92.4 to -62.6)	-31.3 (-83.8 to 176.8)	-74.7 (-94.1 to 13)
Cystic echinococcosis	Both	3868 (2251 to 5846)	6.8 (4.4 to 9.2)	50 (27 to 77)	0.1 (0.1 to 0.2)	2346 (1701 to 3194)	2.7 (2 to 3.6)	16 (9 to 24)	0 (0 to 0)	-39.3 (-63.3 to 6.5)	-60.2 (-72.8 to -38.4)	-68.1 (-84.1 to -34)	-80.6 (-90.2 to -64.2)
	Female	1698 (787 to 2942)	6.1 (3.5 to 9.1)	20 (7 to 36)	0.1 (0 to 0.2)	1253 (886 to 1773)	2.9 (2 to 4)	7 (3 to 12)	0 (0 to 0)	-26.2 (-60.6 to 54.8)	-52.8 (-70.8 to -19.4)	-64.8 (-88 to 13.4)	-77.8 (-92.7 to -38.6)
	Male	2170 (1004 to 3649)	7.4 (4.3 to 11)	30 (13 to 50)	0.1 (0.1 to 0.2)	1094 (764 to 1475)	2.5 (1.8 to 3.4)	9 (4 to 15)	0 (0 to 0)	-49.6 (-73.2 to 11.7)	-66 (-79.2 to -38)	-70.2 (-89.8 to -19.6)	-82.4 (-93.5 to -57.5)
Trachoma	Both	494 (277 to 824)	2.1 (1.2 to 3.5)	--	--	274 (153 to 456)	0.4 (0.2 to 0.7)	--	--	-44.4 (-51.4 to -36.5)	-80.9 (-82.9 to -79)	--	--
	Female	371 (216 to 618)	3.3 (1.9 to 5.3)	--	--	191 (108 to 315)	0.6 (0.3 to 0.9)	--	--	-48.6 (-55.4 to -41.5)	-82.9 (-85 to -80.9)	--	--

Cause	Sex	1990				2019				1990-2019 percent change (%)			
		DALYs		Death		DALYs		Death		DALYs		Death	
		Number	Rate	Number	Rate	Number	Rate	Number	Rate	Number	Rate	Number	Rate
	Male	122 (62 to 216)	1 (0.5 to 1.7)	--	--	83 (43 to 146)	0.2 (0.1 to 0.4)	--	--	-31.9 (-42.5 to -17.4)	-74.9 (-78.2 to -71.5)	--	--
Rabies	Both	290 (67 to 485)	0.5 (0.1 to 0.9)	5 (1 to 9)	0 (0 to 0)	145 (32 to 181)	0.2 (0 to 0.2)	3 (1 to 4)	0 (0 to 0)	-50 (-72.8 to -18.5)	-67.1 (-81.5 to -48.2)	-39.5 (-65.5 to -0.5)	-66.7 (-80.5 to -42.2)
	Female	36 (8 to 50)	0.2 (0 to 0.3)	1 (0 to 1)	0 (0 to 0)	22 (4 to 29)	0.1 (0 to 0.1)	1 (0 to 1)	0 (0 to 0)	-38.2 (-70.1 to -6.4)	-70.8 (-79.3 to -47.5)	-37.9 (-55.5 to 12.4)	-74.3 (-81.4 to -35.2)
	Male	254 (42 to 449)	0.9 (0.1 to 1.5)	4 (1 to 8)	0 (0 to 0)	123 (24 to 154)	0.3 (0.1 to 0.4)	3 (1 to 3)	0 (0 to 0)	-51.6 (-75.5 to -16.5)	-66.1 (-82.6 to -43.6)	-39.8 (-69 to 3.6)	-64.1 (-81 to -42.2)
Intestinal nematode infections	Both	4700 (3418 to 6422)	6.3 (4.6 to 8.6)	34 (25 to 48)	0 (0 to 0.1)	860 (521 to 1392)	1.1 (0.7 to 1.7)	3 (2 to 3)	0 (0 to 0)	-81.7 (-88.4 to -73)	-83.1 (-88.7 to -75.8)	-92.4 (-95.1 to -88.5)	-91.1 (-94 to -87.3)
	Female	2583 (1801 to 3568)	7.5 (5.2 to 10.3)	16 (11 to 24)	0 (0 to 0.1)	525 (310 to 856)	1.3 (0.8 to 2.1)	1 (1 to 2)	0 (0 to 0)	-79.7 (-87.3 to -69.6)	-82.5 (-88.5 to -74.5)	-92.6 (-95.5 to -88)	-91 (-94.3 to -86)
	Male	2118 (1519 to 2980)	5.2 (3.7 to 7.2)	19 (13 to 27)	0 (0 to 0.1)	335 (206 to 535)	0.8 (0.5 to 1.3)	1 (1 to 2)	0 (0 to 0)	-84.2 (-90.6 to -75.1)	-84 (-90 to -75.6)	-92.2 (-95.2 to -87.7)	-91.1 (-94.3 to -86.7)
Food-borne trematodiases	Both	202 (48 to 416)	0.5 (0.1 to 1)	--		882 (214 to 1826)	1 (0.2 to 2)	--	--	337.3 (280.8 to 398.5)	108.4 (88.2 to 131.8)	--	--
	Female	82 (20 to 171)	0.4 (0.1 to 0.8)	--	--	348 (83 to 723)	0.8 (0.2 to 1.7)	--	--	326.8 (269 to 398.1)	100.3 (78.1 to 128.6)	--	--
	Male	120 (29 to 249)	0.6 (0.1 to 1.1)	--	--	535 (124 to 1098)	1.2 (0.3 to 2.5)	--	--	344.4 (277.9 to 413.7)	115 (89.3 to 145.2)	--	--
Leprosy	Both	16 (10 to 25)	0 (0 to 0.1)	--	--	28 (17 to 43)	0 (0 to 0.1)	--	--	73 (64.2 to 81.6)	-30.3 (-33.4 to -27.6)	--	--
	Female	6 (3 to 9)	0 (0 to 0.1)	--	--	9 (6 to 14)	0 (0 to 0)	--	--	63.8 (51.7 to 76.6)	-35.5 (-39.8 to -31.2)	--	--
	Male	11 (7 to 16)	0.1 (0 to 0.1)	--	--	19 (12 to 29)	0 (0 to 0.1)	--	--	77.8 (68.8 to 86.1)	-26.9 (-30.4 to -23.9)	--	--
Other neglected tropical diseases	Both	16519 (11149 to 23147)	21.9 (15.1 to 30.2)	59 (36 to 98)	0.1 (0.1 to 0.2)	8325 (5531 to 11826)	10.9 (7.3 to 15.6)	57 (27 to 71)	0.1 (0 to 0.1)	-49.6 (-63.5 to -31.7)	-49.9 (-63 to -34.5)	-4.3 (-53.3 to 42.2)	-29.8 (-59.7 to -0.1)
	Female	8089 (5248 to 11718)	22.4 (14.9 to 31.3)	25 (11 to 40)	0.1 (0 to 0.2)	4065 (2590 to 5800)	10.9 (7 to 15.6)	25 (9 to 32)	0.1 (0 to 0.1)	-49.7 (-64.4 to -30)	-51.6 (-64.2 to -35.1)	-0.2 (-47.4 to 78.3)	-27.9 (-57.3 to 23.5)
	Male	8430 (5264 to 12707)	21.3 (13.8 to 31.4)	34 (16 to 68)	0.1 (0.1 to 0.2)	4260 (2626 to 6440)	11 (6.8 to 16.6)	32 (12 to 45)	0.1 (0 to 0.1)	-49.5 (-68.7 to -20.8)	-48.2 (-66.8 to -21.9)	-7.3 (-59.2 to 61.5)	-31 (-62.3 to 15.9)
Other infectious diseases	Both	770629 (455781 to 1296564)	941 (591.2 to 1518.9)	9371 (5683 to 15424)	13.6 (9.3 to 20.5)	102118 (87959 to 119769)	132.1 (113 to 156.5)	1936 (1678 to 2163)	2.7 (2.3 to 3.1)	-86.7 (-91.8 to -77.2)	-86 (-91.1 to -77.2)	-79.3 (-87.1 to -65.1)	-80 (-86.4 to -70.3)
	Female	400885 (229526 to 678821)	984.4 (598.7 to 1610.1)	4787 (2823 to 7957)	13.7 (9 to 21.1)	45280 (38399 to 54336)	120.5 (101.4 to 146.3)	769 (665 to 879)	2.2 (1.9 to 2.6)	-88.7 (-93.3 to -80.6)	-87.8 (-92.5 to -79.8)	-83.9 (-90.1 to -72.8)	-83.6 (-89.3 to -75)
	Male	369744 (219187 to 617915)	897.8 (570.7 to 1449.9)	4583 (2820 to 7575)	13.4 (9.1 to 20.2)	56838 (48417 to 66300)	143.7 (122.2 to 169.6)	1166 (966 to 1306)	3.2 (2.6 to 3.6)	-84.6 (-90.6 to -73.8)	-84 (-89.8 to -74.2)	-74.6 (-84.3 to -57.7)	-76.2 (-84.1 to -64.2)
Meningitis	Both	143100 (114166 to 179805)	186.1 (151.9 to 229.4)	1784 (1432 to 2222)	2.9 (2.4 to 3.5)	30092 (26169 to 34399)	38.6 (33.5 to 44.3)	577 (515 to 647)	0.8 (0.7 to 0.9)	-79 (-84.1 to -71.6)	-79.2 (-84.1 to -72.5)	-67.7 (-75 to -57.1)	-72.3 (-78 to -64.3)
	Female	70546 (54659 to 90802)	186.3 (150.1 to 235)	864 (680 to 1105)	2.8 (2.4 to 3.6)	14293 (12235 to 16477)	37.4 (31.8 to 43.2)	249 (221 to 280)	0.7 (0.6 to 0.8)	-79.7 (-85 to -72.3)	-79.9 (-84.9 to -73.4)	-71.1 (-78.6 to -61.5)	-74.5 (-81 to -67.8)
	Male	72554 (55998 to 94149)	185.6 (146 to 235.1)	920 (713 to 1176)	3 (2.3 to 3.6)	15799 (13541 to 18574)	39.8 (33.9 to 46.9)	328 (286 to 387)	0.9 (0.8 to 1.1)	-78.2 (-84.5 to -68.5)	-78.6 (-84.4 to -69.3)	-64.4 (-73.8 to -48.4)	-70 (-76.9 to -56.6)

Cause	Sex	1990				2019				1990-2019 percent change (%)			
		DALYs		Death		DALYs		Death		DALYs		Death	
		Number	Rate	Number	Rate	Number	Rate	Number	Rate	Number	Rate	Number	Rate
Encephalitis	Both	14646 (11309 to 19037)	21 (17.1 to 25.8)	184 (146 to 232)	0.4 (0.3 to 0.4)	12690 (10236 to 14589)	16.3 (12.8 to 19)	217 (157 to 244)	0.3 (0.2 to 0.3)	-13.4 (-37.5 to 14.3)	-22.1 (-41.5 to -0.8)	18 (-13.1 to 52.2)	-15.6 (-37.9 to 4.8)
	Female	7664 (5225 to 10734)	22.1 (16.5 to 29)	93 (64 to 127)	0.4 (0.3 to 0.4)	5970 (4687 to 7027)	15.7 (11.9 to 18.7)	96 (67 to 111)	0.3 (0.2 to 0.3)	-22.1 (-53 to 9.4)	-28.9 (-55.2 to -4.6)	3.1 (-33.6 to 41.2)	-23.7 (-43.4 to -1.7)
	Male	6982 (4936 to 9463)	19.9 (14.8 to 25.8)	91 (65 to 121)	0.4 (0.3 to 0.4)	6720 (5186 to 7859)	16.9 (12.7 to 20)	121 (86 to 138)	0.3 (0.2 to 0.4)	-3.8 (-35 to 43.1)	-14.8 (-42.9 to 21.4)	33.3 (-10.1 to 96.7)	-7 (-37 to 26.2)
Diphtheria	Both	2570 (1781 to 3816)	3 (2.1 to 4.3)	31 (22 to 45)	0 (0 to 0.1)	58 (46 to 72)	0.1 (0.1 to 0.1)	1 (1 to 1)	0 (0 to 0)	-97.8 (-98.7 to -96.5)	-97.5 (-98.5 to -96.1)	-97.2 (-98.3 to -95.8)	-97.1 (-98.2 to -95.6)
	Female	1186 (746 to 1833)	2.8 (1.8 to 4.2)	14 (9 to 22)	0 (0 to 0.1)	22 (17 to 29)	0.1 (0 to 0.1)	0 (0 to 0)	0 (0 to 0)	-98.1 (-99 to -96.7)	-97.8 (-98.8 to -96.2)	-97.7 (-98.7 to -96)	-97.5 (-98.6 to -95.8)
	Male	1385 (934 to 2048)	3.1 (2.2 to 4.6)	17 (12 to 24)	0 (0 to 0.1)	35 (28 to 45)	0.1 (0.1 to 0.1)	1 (0 to 1)	0 (0 to 0)	-97.4 (-98.5 to -95.9)	-97.2 (-98.4 to -95.5)	-96.8 (-98.1 to -95)	-96.7 (-98 to -95)
Whooping cough	Both	157472 (18985 to 465215)	175.9 (21.1 to 519.1)	1788 (185 to 5348)	2 (0.2 to 6)	5807 (739 to 16841)	8.3 (1.1 to 24.1)	65 (6 to 194)	0.1 (0 to 0.3)	-96.3 (-99.5 to -66)	-95.3 (-99.4 to -56.8)	-96.4 (-99.7 to -64)	-95.4 (-99.6 to -54.2)
	Female	90948 (10817 to 266623)	207.4 (24.5 to 604.6)	1033 (109 to 3065)	2.4 (0.2 to 7)	3225 (405 to 9278)	9.5 (1.2 to 27.3)	36 (3 to 107)	0.1 (0 to 0.3)	-96.5 (-99.6 to -69)	-95.4 (-99.5 to -60.1)	-96.5 (-99.7 to -66.3)	-95.6 (-99.6 to -57.1)
	Male	66523 (8032 to 201623)	145.8 (17.6 to 442.8)	755 (78 to 2320)	1.7 (0.2 to 5.1)	2582 (332 to 7587)	7.2 (0.9 to 21.1)	29 (2 to 87)	0.1 (0 to 0.2)	-96.1 (-99.5 to -64.5)	-95.1 (-99.4 to -54.9)	-96.2 (-99.6 to -60.4)	-95.2 (-99.6 to -50)
Tetanus	Both	16153 (6064 to 32628)	20.3 (8.5 to 40)	199 (84 to 394)	0.3 (0.2 to 0.5)	911 (693 to 1254)	1.2 (0.9 to 1.7)	17 (12 to 24)	0 (0 to 0)	-94.4 (-97.6 to -82.1)	-94.1 (-97.4 to -82.3)	-91.5 (-96.4 to -75.4)	-92.3 (-96.6 to -81.3)
	Female	6935 (2104 to 14717)	17.4 (6 to 36.3)	84 (29 to 174)	0.2 (0.1 to 0.5)	382 (315 to 468)	1 (0.8 to 1.3)	7 (6 to 8)	0 (0 to 0)	-94.5 (-97.5 to -79.2)	-94.2 (-97.3 to -80.7)	-91.7 (-96.2 to -73.6)	-92.3 (-96.3 to -79.7)
	Male	9218 (3382 to 20335)	23 (8.8 to 48.1)	115 (44 to 240)	0.3 (0.1 to 0.6)	529 (360 to 812)	1.4 (0.9 to 2.1)	10 (6 to 17)	0 (0 to 0)	-94.3 (-97.8 to -79.9)	-94 (-97.7 to -78.7)	-91.3 (-96.8 to -69.9)	-92.3 (-96.9 to -74.4)
Measles	Both	352875 (115323 to 759943)	387.6 (126.2 to 835.4)	4101 (1344 to 8842)	4.5 (1.5 to 9.7)	6254 (1895 to 14419)	8.8 (2.7 to 20.2)	71 (21 to 161)	0.1 (0 to 0.2)	-98.2 (-99.2 to -96.7)	-97.7 (-99 to -95.7)	-98.3 (-99.2 to -96.7)	-97.8 (-99 to -95.8)
	Female	183878 (60980 to 409713)	412.6 (136.4 to 917.8)	2138 (711 to 4753)	4.8 (1.6 to 10.7)	3091 (920 to 7307)	8.9 (2.7 to 21.1)	35 (10 to 84)	0.1 (0 to 0.2)	-98.3 (-99.3 to -96.8)	-97.8 (-99 to -95.8)	-98.3 (-99.3 to -96.8)	-97.9 (-99.1 to -95.9)
	Male	168997 (54848 to 363711)	363.6 (117.9 to 781.9)	1963 (636 to 4226)	4.2 (1.4 to 9.1)	3163 (963 to 7185)	8.7 (2.6 to 19.7)	36 (11 to 82)	0.1 (0 to 0.2)	-98.1 (-99.1 to -96.5)	-97.6 (-98.9 to -95.6)	-98.2 (-99.2 to -96.6)	-97.7 (-99 to -95.7)
Varicella and herpes zoster	Both	7227 (5670 to 9734)	11.3 (9.4 to 14.1)	92 (75 to 119)	0.3 (0.2 to 0.3)	3670 (2736 to 4888)	4.7 (3.6 to 6.2)	44 (38 to 50)	0.1 (0.1 to 0.1)	-49.2 (-66.3 to -29.8)	-58.1 (-69.4 to -46.4)	-52.5 (-65 to -37.9)	-72.3 (-76.5 to -67.5)
	Female	3678 (2781 to 4986)	11.6 (9.3 to 14.6)	47 (38 to 61)	0.3 (0.2 to 0.3)	1821 (1362 to 2445)	4.8 (3.6 to 6.3)	21 (18 to 25)	0.1 (0.1 to 0.1)	-50.5 (-67.6 to -29.3)	-58.9 (-70.5 to -46.3)	-54.9 (-67.5 to -39.3)	-72.3 (-77.4 to -65.8)
	Male	3549 (2740 to 4847)	11 (9 to 13.9)	45 (35 to 59)	0.3 (0.2 to 0.3)	1849 (1354 to 2476)	4.7 (3.5 to 6.2)	22 (19 to 26)	0.1 (0.1 to 0.1)	-47.9 (-65.9 to -27)	-57.3 (-69.2 to -45)	-49.8 (-63.7 to -32.6)	-72.3 (-77.4 to -65.9)
Acute hepatitis	Both	49518 (32444 to 67635)	94.7 (61.7 to 130.4)	915 (583 to 1280)	2.5 (1.6 to 3.6)	18628 (16320 to 21417)	22.6 (19.9 to 26)	466 (403 to 534)	0.6 (0.5 to 0.7)	-62.4 (-74 to -38.6)	-76.1 (-83.4 to -60.9)	-49.1 (-66.2 to -12.9)	-75.1 (-84.4 to -57.2)
	Female	23964 (12695 to 32063)	85.5 (43.6 to 112.7)	397 (190 to 535)	2.1 (0.9 to 3.1)	5623 (4723 to 6625)	14.1 (11.9 to 16.6)	121 (96 to 139)	0.3 (0.3 to 0.4)	-76.5 (-83.6 to -51.6)	-83.5 (-88.1 to -64.5)	-69.6 (-79 to -27.1)	-84.1 (-89.9 to -60.1)
	Male	25554 (17132 to 40834)	102.3 (69.8 to 165.2)	518 (344 to 859)	2.8 (1.9 to 4.7)	13005 (11367 to 15277)	31.2 (27.2 to 36.6)	345 (295 to 406)	0.9 (0.8 to 1.1)	-49.1 (-69.8 to -19.6)	-69.5 (-82.1 to -52.8)	-33.4 (-63 to 7.9)	-67.9 (-82.6 to -48.8)
Other unspecified infectious diseases	Both	27068 (18296 to 50661)	41.2 (28.8 to 71.6)	277 (169 to 579)	0.7 (0.4 to 1.2)	24008 (16576 to 29115)	31.5 (21.3 to 38.5)	478 (280 to 565)	0.7 (0.4 to 0.8)	-11.3 (-62.9 to 40.3)	-23.7 (-65.2 to 18)	72.9 (-43.7 to 224.9)	0.6 (-56.9 to 86.2)
	Female	12087 (8221 to 23508)	38.7 (26.9 to 66.2)	117 (70 to 250)	0.6 (0.4 to 1.3)	10853 (7360 to 13502)	29.1 (19.8 to 36.2)	203 (119 to 243)	0.6 (0.4 to 0.7)	-10.2 (-63.7 to 36.8)	-24.8 (-66.1 to 12.5)	74.2 (-42.3 to 230.9)	-5.6 (-57.1 to 92.2)

Cause	Sex	1990				2019				1990-2019 percent change (%)			
		DALYs		Death		DALYs		Death		DALYs		Death	
		Number	Rate	Number	Rate	Number	Rate	Number	Rate	Number	Rate	Number	Rate
	Male	14981 (9389 to 30441)	43.5 (28.9 to 82.4)	160 (93 to 354)	0.7 (0.5 to 1.4)	13155 (8474 to 16768)	33.8 (21.5 to 43.5)	275 (139 to 335)	0.8 (0.4 to 1)	-12.2 (-65.2 to 54.8)	-22.3 (-67 to 30.7)	71.9 (-47.2 to 245.4)	7.1 (-59.4 to 90.2)
Maternal and neonatal disorders	Both	4662446 (3860549 to 5524287)	5502.1 (4562.4 to 6506.3)	51992 (42920 to 61648)	61.3 (50.7 to 72.5)	869123 (726851 to 1034588)	1257.7 (1042.3 to 1502.1)	7562 (6048 to 9260)	11.5 (9.2 to 14.1)	-81.4 (-85.5 to -76.2)	-77.1 (-82.5 to -70.8)	-85.5 (-89.2 to -80.9)	-81.2 (-86 to -75.4)
	Female	2028616 (1686545 to 2387496)	4953.2 (4129.4 to 5807.3)	22649 (18795 to 26651)	55.4 (46.2 to 65)	393289 (329344 to 463854)	1153.1 (959.3 to 1373.5)	3374 (2727 to 4089)	10.4 (8.4 to 12.7)	-80.6 (-84.8 to -75.3)	-76.7 (-82 to -70.2)	-85.1 (-88.9 to -80.5)	-81.2 (-86.1 to -75.4)
	Male	2633829 (2128999 to 3161654)	6021 (4864.9 to 7224)	29343 (23697 to 35271)	66.9 (54 to 80.4)	475834 (392392 to 572225)	1356.3 (1106.2 to 1639)	4188 (3271 to 5197)	12.5 (9.8 to 15.5)	-81.9 (-86.4 to -76.5)	-77.5 (-83.1 to -70.6)	-85.7 (-89.7 to -80.8)	-81.3 (-86.5 to -74.8)
Maternal disorders	Female	55572 (48579 to 63072)	210.4 (184.6 to 237.3)	776 (674 to 875)	3.1 (2.7 to 3.5)	17789 (15308 to 20766)	36.9 (31.7 to 43.3)	214 (198 to 234)	0.4 (0.4 to 0.5)	-68 (-72.4 to -63.1)	-82.5 (-84.9 to -79.7)	-72.4 (-76.1 to -67)	-86.1 (-87.9 to -83.5)
Neonatal disorders	Both	4606874 (3805707 to 5466937)	5398.1 (4461.2 to 6404)	51216 (42199 to 60913)	59.8 (49.3 to 71.2)	851334 (709846 to 1015120)	1239.6 (1023.7 to 1482.7)	7347 (5839 to 9031)	11.3 (9 to 13.9)	-81.5 (-85.7 to -76.3)	-77 (-82.5 to -70.5)	-85.7 (-89.4 to -81)	-81.1 (-86 to -75)
	Female	1973045 (1637454 to 2332188)	4742.8 (3942.4 to 5599)	21873 (18060 to 25873)	52.3 (43.2 to 61.9)	375500 (310937 to 445184)	1116.2 (923.2 to 1332.5)	3159 (2512 to 3871)	10 (7.9 to 12.2)	-81 (-85.3 to -75.6)	-76.5 (-82 to -69.7)	-85.6 (-89.5 to -80.8)	-80.9 (-86.1 to -74.7)
	Male	2633829 (2128999 to 3161654)	6021 (4864.9 to 7224)	29343 (23697 to 35271)	66.9 (54 to 80.4)	475834 (392392 to 572225)	1356.3 (1106.2 to 1639)	4188 (3271 to 5197)	12.5 (9.8 to 15.5)	-81.9 (-86.4 to -76.5)	-77.5 (-83.1 to -70.6)	-85.7 (-89.7 to -80.8)	-81.3 (-86.5 to -74.8)
Nutritional deficiencies	Both	303912 (229022 to 403936)	433 (326 to 572.6)	1356 (911 to 1863)	2.2 (1.5 to 2.9)	123514 (81350 to 174299)	156.7 (104.1 to 222)	230 (207 to 248)	0.4 (0.3 to 0.4)	-59.4 (-68.4 to -48.5)	-63.8 (-71.2 to -55.2)	-83 (-87.8 to -74.5)	-83.2 (-87.1 to -75.5)
	Female	159476 (115563 to 215442)	480.7 (349.4 to 642.2)	714 (471 to 1094)	2.3 (1.6 to 3.5)	66279 (43620 to 96761)	171.1 (112.8 to 245.2)	115 (102 to 126)	0.4 (0.3 to 0.4)	-58.4 (-69.7 to -44.8)	-64.4 (-73 to -54.8)	-84 (-89.5 to -75.7)	-83 (-88.7 to -75.4)
	Male	144435 (104828 to 198077)	385.6 (283.3 to 522.2)	641 (365 to 888)	2.2 (1.3 to 2.7)	57235 (36408 to 85048)	142.6 (90.7 to 211.9)	115 (104 to 126)	0.4 (0.3 to 0.4)	-60.4 (-71.7 to -46.1)	-63 (-73.1 to -50.4)	-82 (-87.3 to -67.6)	-83.3 (-87.1 to -70.5)
Protein-energy malnutrition	Both	126045 (90110 to 170610)	157.6 (115 to 209.9)	1309 (882 to 1807)	2.1 (1.5 to 2.8)	27886 (19391 to 39034)	35.7 (25.1 to 49.2)	173 (156 to 189)	0.3 (0.3 to 0.3)	-77.9 (-85.8 to -66.5)	-77.3 (-85.2 to -66.6)	-86.7 (-90.5 to -79.9)	-86.5 (-89.7 to -80.2)
	Female	66181 (46385 to 97363)	167.1 (118.8 to 242.4)	694 (458 to 1068)	2.2 (1.5 to 3.4)	13304 (9031 to 18705)	34.9 (24.1 to 48.8)	87 (77 to 97)	0.3 (0.3 to 0.3)	-79.9 (-87.7 to -67.6)	-79.1 (-87 to -67)	-87.4 (-91.7 to -80.9)	-86.3 (-90.9 to -79.7)
	Male	59864 (39291 to 82107)	148.4 (100.2 to 198.9)	615 (351 to 855)	2 (1.2 to 2.6)	14582 (10065 to 20488)	36.6 (25.6 to 50.9)	86 (77 to 95)	0.3 (0.2 to 0.3)	-75.6 (-84 to -59.1)	-75.4 (-83.4 to -60.7)	-86 (-90.2 to -75.2)	-86.6 (-89.8 to -76.7)
Iodine deficiency	Both	7700 (4839 to 11636)	14.8 (9.5 to 21.7)	--	--	5158 (2961 to 8514)	5.9 (3.4 to 9.6)	--	--	-33 (-48.1 to -16.6)	-59.9 (-68.5 to -52.4)	--	--
	Female	4661 (2897 to 7045)	18.2 (11.7 to 26.9)	--	--	3221 (1876 to 5310)	7.5 (4.3 to 12.3)	--	--	-30.9 (-47.6 to -14)	-58.8 (-68.1 to -50.6)	--	--
	Male	3040 (1882 to 4524)	11.5 (7.4 to 16.8)	--	--	1937 (1102 to 3178)	4.4 (2.5 to 7.2)	--	--	-36.3 (-51 to -21.7)	-61.6 (-69.9 to -54)	--	--
Vitamin A deficiency	Both	9549 (5821 to 14855)	10.3 (6.3 to 16)	--	--	540 (284 to 986)	0.8 (0.4 to 1.4)	--	--	-94.3 (-96.7 to -90.2)	-92.7 (-95.7 to -87.3)	--	--
	Female	3525 (1967 to 5597)	7.8 (4.4 to 12.4)	--	--	235 (110 to 428)	0.7 (0.3 to 1.2)	--	--	-93.3 (-96.4 to -88.2)	-91.3 (-95.2 to -84.7)	--	--
	Male	6024 (3309 to 10109)	12.7 (7 to 21.3)	--	--	306 (128 to 647)	0.8 (0.3 to 1.7)	--	--	-94.9 (-97.7 to -88.8)	-93.4 (-97.1 to -85.7)	--	--

Cause	Sex	1990				2019				1990-2019 percent change (%)			
		DALYs		Death		DALYs		Death		DALYs		Death	
		Number	Rate	Number	Rate	Number	Rate	Number	Rate	Number	Rate	Number	Rate
Dietary iron deficiency	Both	152985 (93564 to 228688)	237.3 (152.9 to 347.8)	--	--	81576 (49682 to 127680)	104.3 (63.8 to 163.5)	--	--	-46.7 (-60.2 to -28.8)	-56.1 (-66.2 to -43.6)	--	--
	Female	81686 (49409 to 121773)	275.7 (173.3 to 408.1)	--	--	45528 (27019 to 72272)	118.2 (70.4 to 184.4)	--	--	-44.3 (-60.4 to -22.1)	-57.1 (-68.2 to -42.6)	--	--
	Male	71299 (40387 to 114804)	198.7 (118.4 to 313.8)	--	--	36048 (19976 to 60950)	90.5 (50 to 154)	--	--	-49.4 (-69.1 to -21.5)	-54.5 (-71.4 to -31.5)	--	--
Other nutritional deficiencies	Both	7633 (5523 to 10231)	13.1 (9.4 to 17.6)	47 (29 to 64)	0.1 (0.1 to 0.2)	8354 (5692 to 11911)	10 (6.9 to 14.1)	57 (50 to 62)	0.1 (0.1 to 0.1)	9.4 (-11.2 to 36.5)	-23.3 (-36.2 to -7.7)	20.1 (-13.7 to 106.6)	-25.7 (-46 to 26.2)
	Female	3424 (2362 to 4775)	11.8 (8.1 to 16.3)	21 (13 to 35)	0.1 (0.1 to 0.2)	3991 (2638 to 5768)	9.8 (6.6 to 14)	27 (24 to 30)	0.1 (0.1 to 0.1)	16.6 (-13.2 to 51.9)	-17.4 (-37.1 to 5.6)	32.3 (-25 to 116.7)	-15.7 (-53.7 to 51.6)
	Male	4209 (2981 to 5921)	14.3 (10.3 to 20.3)	27 (12 to 37)	0.1 (0.1 to 0.2)	4363 (2910 to 6329)	10.3 (7 to 14.8)	29 (26 to 33)	0.1 (0.1 to 0.1)	3.6 (-23 to 38.1)	-28 (-44.3 to -7.8)	10.7 (-24.6 to 151.9)	-33.6 (-52.5 to 36.3)
**Non-communicable diseases**	Both	10743718 (9053266 to 12338036)	26480.2 (23371.6 to 29475.7)	173710 (159460 to 187266)	747.9 (718.9 to 775.1)	15494548 (13229363 to 18078885)	19632.9 (17000.1 to 22614.3)	326508 (318268 to 335734)	508.9 (496.5 to 522.7)	44.2 (28.8 to 62.1)	-25.9 (-31.4 to -20)	88 (73.1 to 106.6)	-31.9 (-35.1 to -28.4)
	Female	5125496 (4264108 to 5931059)	25644.1 (22329.7 to 29019.8)	74522 (68098 to 81355)	674.9 (646.2 to 705.4)	7760484 (6496609 to 9218300)	19685.5 (16706.5 to 23100.9)	146503 (141464 to 151659)	475.4 (459.6 to 491.6)	51.4 (34.4 to 69.2)	-23.2 (-29 to -17.6)	96.6 (78.6 to 118.1)	-29.6 (-33.5 to -25.4)
	Male	5618222 (4735970 to 6413179)	27169.6 (24164.6 to 29934.6)	99188 (90009 to 108142)	814.6 (772 to 856.9)	7734064 (6744951 to 8846192)	19625.6 (17282.4 to 22198.7)	180005 (173339 to 187658)	544.3 (524.8 to 566.4)	37.7 (21.9 to 58)	-27.8 (-33.8 to -21.1)	81.5 (65.5 to 103.4)	-33.2 (-37.3 to -28.4)
Neoplasms	Both	930393 (826200 to 1041493)	2618.9 (2356.6 to 2851.7)	26647 (23901 to 29005)	102.3 (91.3 to 110.8)	1801835 (1644788 to 1941131)	2320.6 (2116.1 to 2497.1)	66792 (61592 to 71949)	95.8 (88.2 to 103.1)	93.7 (72.4 to 125)	-11.4 (-18.9 to 3.8)	150.7 (129.1 to 196.1)	-6.4 (-13.7 to 11.5)
	Female	402723 (336615 to 459986)	2236.9 (1977.4 to 2479.1)	10736 (9480 to 11937)	81.8 (72.2 to 93.1)	799680 (734095 to 855770)	2013.8 (1849.4 to 2160)	27815 (25668 to 29951)	78.2 (71.5 to 84.4)	98.6 (70.9 to 133.4)	-10 (-20.4 to 2.8)	159.1 (128.4 to 199.1)	-4.4 (-16.7 to 10.1)
	Male	527670 (446468 to 605518)	2983.8 (2590.1 to 3312.4)	15911 (13786 to 17680)	123.7 (105.3 to 135.9)	1002155 (891125 to 1100991)	2632.5 (2353.6 to 2885.9)	38978 (35413 to 42375)	113.2 (102.8 to 122.8)	89.9 (64.2 to 130.4)	-11.8 (-21.5 to 9.1)	145 (117.8 to 202.6)	-8.5 (-17.3 to 15.2)
Lip and oral cavity cancer	Both	5085 (4264 to 5863)	16.9 (13.9 to 19.5)	171 (140 to 199)	0.7 (0.6 to 0.8)	12433 (11507 to 13523)	16 (14.7 to 17.4)	486 (446 to 534)	0.7 (0.6 to 0.8)	144.5 (99.9 to 209.8)	-5.2 (-22.7 to 21.9)	185 (131.4 to 270)	-1.3 (-19.5 to 30.5)
	Female	1741 (1534 to 1947)	11.8 (10 to 13.4)	57 (47 to 64)	0.5 (0.4 to 0.6)	5299 (4898 to 5731)	13.7 (12.6 to 14.9)	208 (188 to 229)	0.6 (0.5 to 0.7)	204.4 (163.2 to 257.8)	15.7 (-1.4 to 42.1)	266.2 (208 to 357.7)	22 (0.5 to 59)
	Male	3344 (2690 to 4069)	21.5 (17.1 to 26.1)	114 (91 to 138)	0.9 (0.7 to 1.1)	7134 (6430 to 7979)	18.3 (16.5 to 20.4)	278 (251 to 312)	0.8 (0.7 to 0.9)	113.3 (62.6 to 188.8)	-15 (-35 to 15.7)	144.5 (87.6 to 234.1)	-13.5 (-33 to 19.5)
Nasopharynx cancer	Both	2851 (2493 to 3205)	7.9 (6.9 to 8.7)	78 (69 to 87)	0.3 (0.2 to 0.3)	4110 (3839 to 4438)	5 (4.7 to 5.4)	129 (120 to 139)	0.2 (0.2 to 0.2)	44.2 (25.4 to 72.7)	-36.4 (-43.8 to -24)	64.5 (45.2 to 98.6)	-36.6 (-45 to -23.2)
	Female	1053 (823 to 1188)	6 (4.7 to 7)	29 (22 to 34)	0.2 (0.2 to 0.3)	1385 (1256 to 1558)	3.5 (3.2 to 3.9)	44 (39 to 49)	0.1 (0.1 to 0.1)	31.6 (12.1 to 86.2)	-42 (-52.8 to -18.3)	53 (21.2 to 116.6)	-42.8 (-57.9 to -20.4)
	Male	1798 (1494 to 2084)	9.6 (8.1 to 10.9)	50 (42 to 57)	0.3 (0.3 to 0.4)	2724 (2484 to 2970)	6.5 (6 to 7.1)	85 (78 to 93)	0.2 (0.2 to 0.2)	51.5 (25.7 to 93.6)	-31.8 (-42.7 to -15)	71.1 (44.3 to 111.4)	-31.4 (-41.2 to -16.5)
Other pharynx cancer	Both	1859 (1622 to 2113)	6.3 (5.4 to 7.1)	65 (56 to 73)	0.3 (0.2 to 0.3)	3695 (3410 to 4027)	4.8 (4.4 to 5.2)	145 (133 to 159)	0.2 (0.2 to 0.2)	98.8 (65.7 to 142.1)	-24.4 (-36.5 to -7.2)	123.4 (88 to 176.9)	-21.7 (-33.9 to -2.3)

Cause	Sex	1990				2019				1990-2019 percent change (%)			
		DALYs		Death		DALYs		Death		DALYs		Death	
		Number	Rate	Number	Rate	Number	Rate	Number	Rate	Number	Rate	Number	Rate
	Female	794 (669 to 916)	5.5 (4.6 to 6.4)	27 (22 to 32)	0.2 (0.2 to 0.3)	1404 (1285 to 1562)	3.6 (3.3 to 4)	55 (49 to 61)	0.2 (0.1 to 0.2)	76.7 (44.9 to 129.6)	-35 (-47.1 to -14)	103.2 (62.5 to 168.9)	-31.7 (-48.3 to -8.5)
	Male	1064 (892 to 1268)	6.9 (5.8 to 8.2)	38 (32 to 45)	0.3 (0.2 to 0.3)	2291 (2054 to 2521)	5.9 (5.3 to 6.5)	90 (81 to 100)	0.3 (0.2 to 0.3)	115.3 (68.4 to 172.4)	-14.5 (-33 to 7.8)	137.8 (87.3 to 201)	-12.3 (-30.5 to 11.1)
Esophageal cancer	Both	36344 (26071 to 42262)	130.6 (93.5 to 154.6)	1355 (968 to 1617)	5.9 (4.2 to 7.3)	70307 (57445 to 77012)	94.3 (75.3 to 103.3)	3069 (2420 to 3361)	4.5 (3.5 to 5)	93.4 (59.5 to 140.6)	-27.8 (-40.8 to -11)	126.4 (83.9 to 178.2)	-23 (-38.2 to -6.5)
	Female	14920 (10785 to 18485)	112.9 (81.4 to 147.7)	560 (404 to 746)	5.1 (3.7 to 7.2)	25929 (20597 to 29163)	69.9 (55.3 to 78.6)	1148 (900 to 1294)	3.4 (2.7 to 3.9)	73.8 (43.9 to 128.5)	-38 (-50.8 to -19.2)	104.8 (58.5 to 166.1)	-33.2 (-51.6 to -13.4)
	Male	21424 (14132 to 25431)	147 (97 to 174.6)	795 (524 to 947)	6.6 (4.3 to 7.9)	44378 (33378 to 48951)	118.7 (88 to 131)	1921 (1403 to 2130)	5.6 (4.1 to 6.3)	107.1 (60.2 to 175.8)	-19.3 (-37 to 7.2)	141.7 (89 to 217.8)	-15.1 (-33.7 to 12.5)
Stomach cancer	Both	160499 (141363 to 175116)	565 (500.4 to 621)	5861 (5170 to 6449)	25.3 (22.5 to 28.5)	254282 (240136 to 272100)	338.7 (318.5 to 363.1)	10951 (10144 to 11752)	16.2 (14.9 to 17.4)	58.4 (41.4 to 88.9)	-40.1 (-47 to -29.1)	86.9 (64.9 to 121.4)	-36 (-43.9 to -24.9)
	Female	56709 (50147 to 65623)	408.6 (360.4 to 483.8)	2003 (1757 to 2426)	18.1 (15.7 to 23.1)	89132 (82024 to 97833)	235.6 (217.2 to 260)	3820 (3486 to 4212)	11.4 (10.3 to 12.5)	57.2 (28.6 to 84.1)	-42.3 (-53 to -32.3)	90.8 (55.1 to 124.2)	-37.1 (-50.7 to -26.1)
	Male	103790 (87336 to 114929)	710.3 (589.6 to 784.4)	3858 (3169 to 4272)	32.4 (26 to 35.9)	165149 (154883 to 178540)	443.2 (415.2 to 479.6)	7131 (6602 to 7711)	21 (19.4 to 22.7)	59.1 (39.7 to 97)	-37.6 (-45.1 to -21.1)	84.8 (62.3 to 134.4)	-35.1 (-42.7 to -15.1)
Colon and rectum cancer	Both	48385 (39872 to 58027)	165.6 (136.6 to 198.7)	1665 (1369 to 1997)	7.2 (5.9 to 8.6)	159445 (148776 to 171672)	206.7 (192.2 to 222.1)	6413 (5903 to 6908)	9.3 (8.5 to 10.1)	229.5 (162.7 to 315.8)	24.8 (-0.4 to 57.7)	285.2 (206.4 to 392.6)	29.9 (3.8 to 66.7)
	Female	21570 (17870 to 27028)	153.6 (125.7 to 193.2)	740 (603 to 933)	6.6 (5.3 to 8.4)	70378 (64159 to 76353)	183.2 (167.1 to 198.9)	2870 (2593 to 3131)	8.4 (7.5 to 9.2)	226.3 (147.1 to 310.9)	19.2 (-9.3 to 52.7)	287.6 (194.8 to 404.6)	26.7 (-3.6 to 69.4)
	Male	26815 (20833 to 32706)	176.3 (138 to 214.2)	925 (719 to 1127)	7.6 (5.9 to 9.3)	89067 (81535 to 97659)	230.7 (211 to 253.1)	3543 (3233 to 3898)	10.2 (9.3 to 11.3)	232.2 (158.8 to 348.4)	30.8 (2.4 to 77)	283.2 (198.9 to 416.6)	33.6 (5.2 to 80.2)
Liver cancer	Both	30563 (25874 to 35118)	102.7 (84.9 to 120.1)	1076 (889 to 1258)	4.6 (3.7 to 5.5)	61489 (56204 to 67247)	81.3 (74.2 to 88.8)	2501 (2272 to 2750)	3.6 (3.3 to 4)	101.2 (65.9 to 150.7)	-20.9 (-36.3 to 0.2)	132.5 (85.7 to 196)	-20.4 (-38.4 to 4.4)
	Female	12360 (9568 to 16853)	86 (64.3 to 123.1)	429 (318 to 619)	3.9 (2.8 to 5.8)	23365 (20776 to 25674)	61.6 (54.7 to 67.8)	948 (833 to 1047)	2.8 (2.4 to 3.1)	89 (28.5 to 162.9)	-28.3 (-53.9 to 3.1)	121 (40 to 221.8)	-28.6 (-56.3 to 6.3)
	Male	18203 (15815 to 21594)	117.5 (101.8 to 138.7)	647 (559 to 768)	5.2 (4.4 to 6.1)	38123 (33517 to 43448)	101.2 (88.9 to 115.7)	1554 (1363 to 1778)	4.5 (3.9 to 5.2)	109.4 (75 to 149.6)	-13.8 (-28.9 to 3.7)	140.1 (96.9 to 190.5)	-13.2 (-29.4 to 6.6)
Gallbladder and biliary tract cancer	Both	7904 (6525 to 9101)	29.2 (24.4 to 34.3)	303 (253 to 360)	1.4 (1.2 to 1.7)	18392 (14210 to 20736)	24.6 (19.3 to 27.8)	802 (638 to 904)	1.2 (1 to 1.3)	132.7 (85 to 208.1)	-15.7 (-32.3 to 9.7)	164.8 (116.8 to 243.2)	-13 (-28.5 to 10.7)
	Female	4048 (3243 to 5131)	31.7 (25.7 to 40.6)	159 (129 to 208)	1.5 (1.2 to 2)	9333 (6861 to 10800)	25.3 (18.8 to 29.3)	418 (316 to 496)	1.3 (1 to 1.5)	130.6 (84.1 to 208.6)	-20.3 (-35.8 to 3.7)	163.6 (114.4 to 242.2)	-16 (-32.8 to 6.8)
	Male	3856 (3076 to 4427)	26.7 (21.2 to 30.8)	144 (115 to 167)	1.2 (1 to 1.4)	9059 (6200 to 10352)	23.9 (16.7 to 27.2)	383 (275 to 436)	1.1 (0.8 to 1.3)	134.9 (77.6 to 215.4)	-10.5 (-31.7 to 20.7)	166.1 (106.9 to 255.5)	-8.7 (-28.9 to 22.7)
Pancreatic cancer	Both	14392 (11728 to 17286)	51 (41.4 to 61.5)	534 (434 to 646)	2.3 (1.8 to 2.7)	79881 (74924 to 85848)	105.9 (99.1 to 114.1)	3361 (3124 to 3643)	4.9 (4.5 to 5.3)	455 (346.7 to 607)	107.5 (66.2 to 165.1)	528.9 (402.7 to 704.1)	117 (72.3 to 179)
	Female	5126 (4256 to 6664)	38.7 (32 to 50.9)	193 (159 to 256)	1.7 (1.4 to 2.3)	31566 (28442 to 34598)	84.5 (75.8 to 92.8)	1365 (1214 to 1502)	4 (3.6 to 4.4)	515.8 (337.5 to 678)	118.5 (54 to 176.7)	606.6 (394.5 to 799.3)	132.5 (59.9 to 198.9)
	Male	9266 (7180 to 11904)	62.6 (48.5 to 80)	341 (264 to 437)	2.8 (2.1 to 3.5)	48315 (44702 to 52597)	127.4 (117.9 to 138.8)	1995 (1842 to 2173)	5.8 (5.3 to 6.3)	421.4 (292 to 595.4)	103.6 (53.5 to 171.9)	485 (341 to 680.7)	107.7 (57.8 to 179.8)
Larynx cancer	Both	21890 (18024 to 23885)	74.6 (61.8 to 81.2)	762 (633 to 830)	3 (2.5 to 3.3)	37051 (34051 to 41125)	48.1 (44.2 to 53.3)	1451 (1322 to 1614)	2.1 (1.9 to 2.3)	69.3 (51 to 122.1)	-35.5 (-42.4 to -14.9)	90.3 (69.8 to 151.3)	-31.8 (-38.7 to -9.8)

Cause	Sex	1990				2019				1990-2019 percent change (%)			
		DALYs		Death		DALYs		Death		DALYs		Death	
		Number	Rate	Number	Rate	Number	Rate	Number	Rate	Number	Rate	Number	Rate
	Female	5113 (4355 to 5623)	35.6 (29.9 to 39)	169 (140 to 186)	1.4 (1.1 to 1.6)	8236 (7646 to 8951)	21.3 (19.8 to 23.3)	322 (296 to 352)	0.9 (0.8 to 1)	61.1 (42.8 to 95.4)	-40 (-46.6 to -25.8)	90.3 (68.2 to 139.6)	-33.9 (-41.1 to -13.8)
	Male	16777 (13472 to 18635)	111 (89.1 to 122.7)	593 (476 to 658)	4.7 (3.7 to 5.1)	28816 (26009 to 32526)	75.1 (68 to 84.5)	1129 (1019 to 1268)	3.2 (2.9 to 3.6)	71.8 (49.8 to 134)	-32.3 (-40.8 to -8.1)	90.3 (66.4 to 157.2)	-30.7 (-39.1 to -5.9)
Tracheal, bronchus, and lung cancer	Both	80596 (67976 to 97081)	275.8 (230.9 to 333.4)	2889 (2412 to 3501)	11.8 (9.7 to 14.4)	218990 (203461 to 234523)	286.8 (266.1 to 307.6)	8923 (8247 to 9595)	12.9 (11.9 to 13.9)	171.7 (113.4 to 241.5)	4 (-18.2 to 32.5)	208.9 (141.8 to 296.5)	8.9 (-14.4 to 40.8)
	Female	17355 (14966 to 24193)	120.1 (102.5 to 167.9)	584 (495 to 816)	5 (4.2 to 7.1)	68653 (62555 to 74042)	178.2 (161.4 to 192.4)	2768 (2488 to 3018)	8 (7.2 to 8.8)	295.6 (159.8 to 374.1)	48.3 (-3.3 to 80.9)	373.7 (208.4 to 484.7)	59 (3.7 to 99.2)
	Male	63240 (51519 to 77197)	420.7 (340.3 to 512)	2304 (1857 to 2815)	18.5 (14.8 to 22.6)	150337 (137471 to 164431)	396.9 (364.1 to 434.8)	6155 (5636 to 6725)	17.8 (16.2 to 19.4)	137.7 (81.1 to 212.8)	-5.7 (-27.7 to 24.6)	167.1 (104.4 to 253.7)	-4 (-26.2 to 27.9)
Malignant skin melanoma	Both	6010 (3229 to 8074)	18.9 (10.1 to 25.2)	189 (100 to 251)	0.8 (0.4 to 1.1)	8882 (6336 to 11544)	11.2 (7.8 to 14.1)	328 (216 to 396)	0.5 (0.3 to 0.6)	47.8 (1.3 to 153.7)	-41.1 (-58.4 to -1.2)	73.7 (26.9 to 178.7)	-42.9 (-56.6 to -7.7)
	Female	2534 (1213 to 3554)	16.1 (7.9 to 21.6)	78 (38 to 102)	0.7 (0.3 to 0.9)	3759 (2464 to 5205)	9.4 (6.1 to 12.9)	135 (85 to 179)	0.4 (0.2 to 0.5)	48.3 (0.2 to 156.8)	-41.6 (-58.2 to -6)	73.4 (28.8 to 168.6)	-43.4 (-55.1 to -17.6)
	Male	3476 (1708 to 4664)	21.6 (10 to 29.4)	111 (51 to 151)	1 (0.4 to 1.4)	5123 (3301 to 7578)	12.9 (7.9 to 18.4)	193 (110 to 262)	0.6 (0.3 to 0.7)	47.4 (0.3 to 158)	-40.3 (-59.2 to 7.2)	73.9 (22.8 to 195.4)	-42.9 (-59.9 to 4.3)
Non-melanoma skin cancer	Both	1848 (1571 to 2080)	7.1 (5.9 to 8.1)	75 (61 to 85)	0.4 (0.3 to 0.4)	6431 (5956 to 6881)	8.8 (8.1 to 9.4)	313 (282 to 338)	0.5 (0.4 to 0.5)	248.1 (198.6 to 320.2)	23.1 (5.4 to 51.6)	319.2 (258.2 to 422.7)	28.5 (8.6 to 64.8)
	Female	393 (293 to 498)	3.1 (2.2 to 4.1)	16 (11 to 21)	0.2 (0.1 to 0.2)	1599 (1376 to 1810)	4.3 (3.7 to 4.9)	75 (62 to 86)	0.2 (0.2 to 0.3)	306.8 (185.1 to 483.7)	38.1 (-0.1 to 111.7)	378.1 (245.1 to 643.1)	39.5 (-3.4 to 131.5)
	Male	1455 (1256 to 1644)	11.1 (9.4 to 12.6)	59 (50 to 67)	0.6 (0.5 to 0.7)	4832 (4488 to 5201)	13.2 (12.3 to 14.2)	238 (216 to 258)	0.7 (0.7 to 0.8)	232.2 (185.3 to 294)	19.5 (2.1 to 43.2)	303.5 (242 to 388.2)	20.6 (2.9 to 48.2)
Breast cancer	Both	50647 (43014 to 62892)	157 (130.7 to 198.1)	1436 (1186 to 1823)	5.1 (4.1 to 6.7)	163091 (148806 to 179221)	185.6 (169.7 to 203.4)	4760 (4362 to 5250)	6 (5.5 to 6.6)	222 (150.1 to 290.7)	18.2 (-9 to 45.9)	231.5 (151.4 to 314.3)	16.5 (-14.2 to 49.9)
	Female	49919 (42208 to 62170)	320.2 (265.4 to 405.4)	1413 (1160 to 1796)	10.3 (8.2 to 13.6)	161486 (147227 to 177500)	368.7 (336.7 to 404.3)	4704 (4306 to 5192)	11.9 (10.8 to 13.1)	223.5 (150.8 to 293.2)	15.2 (-11.7 to 42.5)	232.9 (151.2 to 318)	14.9 (-15.1 to 47.7)
	Male	728 (574 to 926)	4.5 (3.5 to 5.8)	23 (18 to 30)	0.2 (0.1 to 0.2)	1605 (1379 to 1790)	4 (3.5 to 4.5)	56 (48 to 63)	0.2 (0.1 to 0.2)	120.4 (58.2 to 197.1)	-10.7 (-36.1 to 20.7)	142.8 (72.5 to 232.2)	-11.4 (-37 to 22.6)
Cervical cancer	Female	13987 (9830 to 16221)	90.4 (63.9 to 107.5)	416 (297 to 501)	3.2 (2.3 to 4.1)	22367 (16199 to 25705)	54.1 (38.9 to 62.2)	761 (552 to 874)	2.1 (1.5 to 2.4)	59.9 (39.1 to 129.9)	-40.2 (-49.4 to -16.4)	82.9 (51.9 to 154.2)	-36 (-49.9 to -14)
Uterine cancer	Female	3072 (2021 to 3852)	21.7 (14.9 to 27.3)	102 (73 to 128)	0.9 (0.6 to 1.1)	9894 (4775 to 11778)	25 (12.6 to 29.7)	349 (186 to 409)	1 (0.5 to 1.1)	222.1 (107.1 to 347.1)	15.2 (-26.6 to 59.5)	242.7 (122.5 to 374.8)	14.8 (-25.2 to 58.9)
Ovarian cancer	Female	7744 (5050 to 14528)	48.1 (32.4 to 89.7)	223 (152 to 412)	1.6 (1.1 to 3)	35213 (24068 to 40121)	85.4 (59.5 to 97)	1148 (818 to 1302)	3 (2.2 to 3.4)	354.7 (113.2 to 568.5)	77.5 (-16.2 to 161.5)	415.4 (146.1 to 659.7)	87 (-10 to 177.2)
Prostate cancer	Male	19290 (15182 to 23620)	181.9 (142.8 to 227.3)	917 (725 to 1150)	11.4 (9 to 14.4)	67728 (50160 to 76614)	208.1 (155.2 to 235.7)	3916 (2912 to 4439)	12.8 (9.6 to 14.6)	251.1 (189.6 to 333.1)	14.4 (-4.4 to 41.6)	326.9 (249.1 to 435.7)	12.2 (-6.7 to 40.8)

Cause	Sex	1990				2019				1990-2019 percent change (%)			
		DALYs		Death		DALYs		Death		DALYs		Death	
		Number	Rate	Number	Rate	Number	Rate	Number	Rate	Number	Rate	Number	Rate
Testicular cancer	Male	1210 (873 to 1709)	4 (3 to 5.6)	19 (14 to 26)	0.1 (0.1 to 0.1)	4940 (4114 to 5763)	11.2 (9.2 to 13.1)	91 (81 to 103)	0.2 (0.2 to 0.3)	308.2 (174.1 to 497.2)	176.3 (91.9 to 294.1)	389.8 (241.5 to 591.1)	171.5 (94.7 to 273.3)
Kidney cancer	Both	11442 (8864 to 14583)	26.8 (19.5 to 32.2)	274 (197 to 328)	1 (0.6 to 1.1)	26586 (24228 to 28540)	34.4 (31.1 to 37)	934 (837 to 1018)	1.3 (1.2 to 1.5)	132.3 (71.7 to 212.7)	28.1 (0.3 to 83.9)	241.4 (170.4 to 402.9)	39.8 (12.8 to 121.7)
	Female	4816 (4042 to 6481)	22.4 (17.1 to 26.9)	109 (82 to 131)	0.8 (0.5 to 1)	9543 (8715 to 10439)	24.8 (22.6 to 27.1)	311 (282 to 346)	0.9 (0.8 to 1)	98.1 (36.8 to 145.5)	10.6 (-13.6 to 48.3)	185.3 (127.1 to 302.6)	13.5 (-10.4 to 87.9)
	Male	6626 (4639 to 8758)	30.9 (21.5 to 38.6)	165 (115 to 206)	1.1 (0.7 to 1.4)	17043 (14676 to 18783)	44 (38.1 to 48.5)	623 (541 to 694)	1.8 (1.5 to 2)	157.2 (80.3 to 287.7)	42.6 (4.4 to 118.9)	278.6 (178.4 to 483.5)	59.5 (18.8 to 151.2)
Bladder cancer	Both	12576 (10087 to 14634)	49.8 (39.5 to 58.3)	510 (403 to 596)	2.5 (2 to 3)	35675 (33013 to 38856)	50.2 (46.2 to 54.7)	1672 (1523 to 1828)	2.6 (2.3 to 2.8)	183.7 (135.2 to 271.4)	0.7 (-16.3 to 33.2)	227.9 (172.2 to 336.5)	1.9 (-15.9 to 37.2)
	Female	2559 (1974 to 3153)	21.9 (16.5 to 28.3)	108 (81 to 141)	1.2 (0.9 to 1.6)	7239 (6455 to 8043)	20.9 (18.5 to 23.2)	368 (323 to 412)	1.2 (1 to 1.3)	182.8 (113.8 to 288.6)	-4.7 (-29.2 to 33.6)	241 (147.9 to 382.3)	0.1 (-31.3 to 43.9)
	Male	10017 (7846 to 11966)	76.9 (60.3 to 91.1)	402 (314 to 477)	4 (3.1 to 4.7)	28436 (25772 to 31178)	79.7 (72.3 to 87.6)	1304 (1172 to 1436)	4 (3.6 to 4.4)	183.9 (125.8 to 283.9)	3.7 (-17 to 39.7)	224.3 (159.7 to 337.6)	0.1 (-19.3 to 35.1)
Brain and central nervous system cancer	Both	91481 (49514 to 123036)	175 (102 to 223.7)	1769 (1038 to 2267)	4.6 (2.8 to 5.9)	128547 (67682 to 153606)	156.4 (82 to 187)	3494 (1751 to 4173)	4.6 (2.3 to 5.5)	40.5 (-27.9 to 81.4)	-10.7 (-50.1 to 8.5)	97.5 (5 to 138.5)	-1.1 (-43.8 to 22.9)
	Female	39063 (16172 to 67161)	150.6 (70.4 to 246.8)	733 (343 to 1199)	3.9 (2 to 6.1)	59400 (30653 to 74451)	147 (76 to 184.9)	1620 (824 to 1988)	4.3 (2.2 to 5.3)	52.1 (-29 to 190)	-2.4 (-51.1 to 67.9)	120.8 (6.4 to 275)	10.3 (-41.3 to 75)
	Male	52418 (25373 to 75851)	197.6 (104 to 275.6)	1035 (549 to 1441)	5.3 (2.9 to 7.2)	69147 (30758 to 88577)	165.7 (72.9 to 213.1)	1874 (793 to 2391)	4.8 (2 to 6.2)	31.9 (-32.2 to 89.9)	-16.1 (-52 to 14.1)	81 (-2 to 143.6)	-8.7 (-45.2 to 22.3)
Thyroid cancer	Both	3172 (2747 to 3697)	10.4 (9 to 12.3)	99 (86 to 118)	0.4 (0.4 to 0.5)	10469 (6928 to 11742)	13.2 (8.9 to 14.6)	341 (245 to 375)	0.5 (0.4 to 0.5)	230 (109.7 to 311.3)	26.4 (-16.4 to 57.7)	243.5 (138.5 to 327.8)	15.1 (-19.6 to 44.8)
	Female	1934 (1630 to 2311)	13.5 (11.3 to 17.1)	62 (51 to 79)	0.5 (0.4 to 0.8)	5995 (3638 to 6924)	15.2 (9.5 to 17.4)	195 (131 to 220)	0.6 (0.4 to 0.6)	210 (86.2 to 300.9)	12.7 (-30.3 to 43.4)	216.6 (104.3 to 305.5)	2.4 (-34.2 to 32.9)
	Male	1239 (1013 to 1598)	7.4 (6 to 9.8)	38 (30 to 50)	0.3 (0.2 to 0.4)	4474 (3132 to 5029)	11.1 (8 to 12.4)	147 (112 to 163)	0.4 (0.3 to 0.5)	261.2 (133.9 to 367.7)	49.6 (-2.9 to 95.6)	287.3 (155 to 405.5)	41.8 (-6 to 86.1)
Mesothelioma	Both	383 (263 to 576)	1.2 (0.9 to 1.8)	11 (8 to 17)	0 (0 to 0.1)	1292 (1147 to 1443)	1.5 (1.3 to 1.8)	41 (37 to 50)	0.1 (0 to 0.1)	237.4 (120.3 to 406.1)	28.3 (-16.8 to 89.9)	269.6 (136.6 to 444.1)	31.3 (-14.4 to 88.5)
	Female	208 (122 to 291)	1.3 (0.8 to 1.8)	6 (4 to 8)	0 (0 to 0.1)	412 (281 to 486)	0.9 (0.7 to 1.1)	13 (9 to 15)	0 (0 to 0)	97.7 (33.9 to 214.7)	-27.8 (-49.4 to 9.9)	110.4 (50.3 to 214.3)	-27.7 (-45.4 to 3)
	Male	175 (118 to 373)	1 (0.7 to 2.2)	5 (4 to 11)	0 (0 to 0.1)	880 (767 to 1049)	2.1 (1.8 to 2.6)	29 (25 to 38)	0.1 (0.1 to 0.1)	403.8 (134 to 693.3)	97.2 (-9.4 to 205.8)	455.2 (148.3 to 746.9)	99.7 (-9.7 to 199.4)
Hodgkin lymphoma	Both	7468 (5917 to 11514)	16.1 (12.8 to 24.7)	159 (126 to 244)	0.5 (0.4 to 0.7)	12813 (8447 to 14749)	15.1 (9.9 to 17.4)	307 (203 to 353)	0.4 (0.3 to 0.4)	71.6 (2.2 to 140.9)	-6.5 (-44.1 to 32)	92.8 (16.3 to 170.9)	-14.2 (-48.6 to 22.9)
	Female	2582 (1984 to 4148)	12 (9.2 to 19.8)	57 (44 to 94)	0.4 (0.3 to 0.6)	4870 (2982 to 5771)	11.7 (7.2 to 13.9)	115 (71 to 136)	0.3 (0.2 to 0.3)	88.6 (13.9 to 176.3)	-2.1 (-42.6 to 44.7)	102.2 (21.6 to 197.3)	-17.5 (-51 to 28.7)
	Male	4886 (3804 to 8206)	20 (15.6 to 33.8)	102 (80 to 172)	0.5 (0.4 to 0.9)	7943 (4639 to 9229)	18.4 (10.7 to 21.4)	191 (112 to 222)	0.5 (0.3 to 0.6)	62.6 (-8.5 to 131.5)	-7.9 (-47 to 30.4)	87.6 (7.9 to 166.8)	-10.7 (-48 to 28)
Non-Hodgkin lymphoma	Both	26086 (21178 to 31260)	60.5 (51.3 to 69.6)	612 (518 to 703)	1.9 (1.6 to 2.2)	53063 (49253 to 56838)	65.4 (60.5 to 70.1)	1581 (1467 to 1694)	2.1 (2 to 2.3)	103.4 (62.7 to 161.6)	8 (-9.9 to 33.6)	158.3 (116.7 to 218.8)	10.2 (-4.2 to 36.8)

Cause	Sex	1990				2019				1990-2019 percent change (%)			
		DALYs		Death		DALYs		Death		DALYs		Death	
		Number	Rate	Number	Rate	Number	Rate	Number	Rate	Number	Rate	Number	Rate
	Female	9340 (6619 to 12341)	45.1 (31 to 56.3)	220 (150 to 273)	1.5 (1 to 1.8)	20102 (18022 to 22081)	50.4 (45.2 to 55.3)	607 (549 to 675)	1.7 (1.5 to 1.9)	115.2 (51.9 to 219.7)	11.6 (-16.7 to 72)	175.5 (108.6 to 333.5)	12.4 (-9.9 to 86.5)
	Male	16746 (13617 to 19515)	75 (63.8 to 84.7)	392 (333 to 441)	2.4 (2 to 2.6)	32961 (30249 to 35627)	80.3 (73.8 to 86.7)	973 (899 to 1047)	2.6 (2.4 to 2.8)	96.8 (63.5 to 152)	7.1 (-8.8 to 31.6)	148.5 (112.5 to 203.4)	9.5 (-4.1 to 33)
Multiple myeloma	Both	6531 (5511 to 8193)	22.8 (19.1 to 28.5)	237 (196 to 297)	1 (0.8 to 1.2)	17392 (12220 to 19716)	22.7 (16 to 26)	677 (483 to 790)	1 (0.7 to 1.1)	166.3 (65.8 to 227.9)	-0.7 (-37 to 23.5)	185.6 (84.5 to 255.6)	-0.3 (-34.1 to 25.8)
	Female	2851 (2247 to 3472)	21.5 (16.6 to 25.8)	107 (82 to 130)	0.9 (0.7 to 1.2)	6979 (4712 to 8043)	18.2 (12.5 to 21)	273 (191 to 315)	0.8 (0.6 to 0.9)	144.8 (75.1 to 228.8)	-15 (-37.2 to 14.5)	156 (95.3 to 246.1)	-16.3 (-33.5 to 13.5)
	Male	3680 (2928 to 5155)	24.1 (19.2 to 34)	130 (104 to 184)	1 (0.8 to 1.4)	10413 (6974 to 12709)	27.1 (18.4 to 33.5)	404 (276 to 505)	1.1 (0.8 to 1.5)	183 (56.7 to 273.4)	12.6 (-37.8 to 48.8)	209.8 (73.6 to 305.4)	14.3 (-35.6 to 48.8)
Leukemia	Both	185562 (120969 to 249702)	324.3 (224.8 to 413.4)	3287 (2284 to 4201)	8.3 (6.1 to 9.8)	162850 (110681 to 188806)	205.3 (140.3 to 237.8)	4424 (3137 to 5030)	6 (4.3 to 6.8)	-12.2 (-34.6 to 19)	-36.7 (-50.4 to -17.4)	34.6 (6.6 to 73.7)	-27.1 (-37.9 to -6.3)
	Female	90176 (43279 to 121267)	308 (181.1 to 389.9)	1509 (893 to 1903)	7.3 (5.1 to 8.5)	67397 (43013 to 76696)	171 (110 to 195)	1775 (1195 to 1972)	4.8 (3.3 to 5.4)	-25.3 (-46.1 to 32.1)	-44.5 (-57.9 to -21)	17.7 (-8.3 to 66.4)	-33.8 (-44.1 to -16.4)
	Male	95386 (45836 to 132954)	339.8 (193.2 to 446.3)	1779 (1010 to 2340)	9.3 (6.2 to 11.5)	95453 (55945 to 114529)	239.1 (140.6 to 286.5)	2649 (1622 to 3129)	7.2 (4.5 to 8.5)	0.1 (-28.1 to 70.6)	-29.6 (-47.5 to 4.5)	48.9 (14.1 to 129.5)	-22.3 (-38.5 to 5.9)
Other malignant neoplasms	Both	69693 (58973 to 79643)	149.2 (125.9 to 168)	1505 (1259 to 1701)	4.7 (3.8 to 5.4)	111061 (87420 to 123895)	142.2 (111.5 to 158.8)	3310 (2587 to 3742)	4.6 (3.6 to 5.3)	59.4 (17.5 to 96.8)	-4.7 (-24.6 to 20.2)	119.9 (70.7 to 183.4)	-2.1 (-18.5 to 35.9)
	Female	29962 (26120 to 34523)	125.6 (110.6 to 140.9)	609 (534 to 683)	3.8 (3.1 to 4.4)	47258 (43193 to 51533)	122.3 (111.1 to 133.6)	1354 (1230 to 1494)	3.8 (3.4 to 4.2)	57.7 (32.6 to 91.3)	-2.6 (-17.3 to 18.6)	122.4 (88.7 to 173.2)	1 (-17.2 to 36.1)
	Male	39731 (30715 to 48651)	171.6 (135.4 to 199.5)	896 (703 to 1045)	5.7 (4.4 to 6.7)	63803 (43769 to 74536)	162.2 (109.4 to 189.4)	1956 (1262 to 2299)	5.5 (3.5 to 6.4)	60.6 (-2.8 to 117.3)	-5.5 (-36.8 to 28)	118.2 (40.2 to 200.4)	-4.4 (-32.5 to 38.1)
Other neoplasms	Both	1824 (1327 to 2163)	5.4 (3.6 to 6.4)	48 (29 to 57)	0.2 (0.1 to 0.2)	3467 (3009 to 4334)	4.6 (4 to 5.8)	113 (98 to 153)	0.2 (0.1 to 0.2)	90.1 (60.9 to 159.8)	-13.9 (-28.5 to 23.8)	136 (84.8 to 283.3)	-18.3 (-36.8 to 36.6)
	Female	794 (558 to 997)	4.7 (2.9 to 5.9)	19 (10 to 23)	0.2 (0.1 to 0.2)	1485 (1284 to 1798)	3.9 (3.4 to 4.7)	44 (39 to 52)	0.1 (0.1 to 0.2)	86.9 (44 to 173.6)	-15.7 (-33.4 to 40.9)	137.9 (76.6 to 380.9)	-21.2 (-42.4 to 81.6)
	Male	1030 (707 to 1238)	6.1 (4.1 to 7.3)	29 (18 to 36)	0.2 (0.1 to 0.3)	1983 (1581 to 2767)	5.4 (4.2 to 7.5)	68 (51 to 106)	0.2 (0.2 to 0.3)	92.5 (57 to 163.7)	-11.6 (-27.8 to 18.6)	134.8 (79.8 to 251.1)	-16.2 (-35.8 to 20.3)
Cardiovascular diseases	Both	2419826 (2283426 to 2599478)	9052.6 (8471.3 to 9688.7)	89211 (83564 to 95793)	464.8 (426.1 to 498.2)	3603912 (3393353 to 3847274)	5077.3 (4759.7 to 5382.5)	173601 (159445 to 183901)	277.7 (252.8 to 294.8)	48.9 (35.3 to 59.1)	-43.9 (-49.3 to -40.3)	94.6 (73.6 to 108.5)	-40.2 (-46.4 to -36.2)
	Female	975030 (907245 to 1052151)	7826.6 (7203.9 to 8452.8)	37852 (34656 to 41084)	426 (383.6 to 462.1)	1519187 (1403020 to 1623567)	4483.1 (4116.3 to 4796.4)	79485 (71331 to 85148)	268.7 (239 to 288.5)	55.8 (40 to 68.8)	-42.7 (-48.7 to -37.4)	110 (85.4 to 130.9)	-36.9 (-44.4 to -30.3)
	Male	1444796 (1345517 to 1576557)	10128.3 (9409.1 to 10980.8)	51358 (47760 to 55784)	497 (455 to 536.5)	2084724 (1965318 to 2229440)	5683.6 (5331.3 to 6069.6)	94116 (86700 to 100156)	288.6 (264.2 to 306.6)	44.3 (31.5 to 56.5)	-43.9 (-49 to -39.4)	83.3 (64.7 to 98.8)	-41.9 (-47.7 to -37.2)
Rheumatic heart disease	Both	65069 (53428 to 80344)	135.1 (112.6 to 169.7)	1136 (932 to 1486)	3.6 (2.9 to 5.2)	51152 (42003 to 62953)	61.3 (51.3 to 74.5)	1030 (886 to 1165)	1.5 (1.3 to 1.7)	-21.4 (-39.9 to -3.5)	-54.6 (-66.9 to -44.5)	-9.3 (-39.6 to 16.9)	-59.4 (-75.7 to -46.1)
	Female	36068 (26744 to 42954)	151.9 (114.6 to 187.2)	629 (456 to 791)	4.1 (2.9 to 6)	26307 (21222 to 32775)	64.3 (52.8 to 79.2)	554 (466 to 655)	1.6 (1.4 to 1.9)	-27.1 (-43 to 3.6)	-57.7 (-68.2 to -40.3)	-12 (-40 to 38.2)	-60.1 (-76.4 to -37.1)
	Male	29001 (20763 to 38823)	118.3 (85.7 to 163.4)	507 (345 to 753)	3.1 (2.2 to 5.1)	24845 (19965 to 31020)	58.3 (47.3 to 71.8)	476 (405 to 574)	1.3 (1.1 to 1.6)	-14.3 (-40.5 to 23.9)	-50.7 (-66.3 to -28.9)	-6 (-43.4 to 52.1)	-57.6 (-76.7 to -32.4)
Ischemic heart disease	Both	1392859 (1303028 to 1515545)	5437.6 (5060.9 to 5851)	55668 (51802 to 60039)	285.7 (260.8 to 307.1)	2025424 (1898318 to 2228261)	2842.7 (2657 to 3103.1)	102799 (94455 to 111215)	163.6 (149 to 176.2)	45.4 (30.2 to 57.9)	-47.7 (-52.9 to -43.6)	84.7 (65.3 to 100)	-42.7 (-47.9 to -38.1)

Cause	Sex	1990				2019				1990-2019 percent change (%)			
		DALYs		Death		DALYs		Death		DALYs		Death	
		Number	Rate	Number	Rate	Number	Rate	Number	Rate	Number	Rate	Number	Rate
	Female	491487 (451951 to 537892)	4260.8 (3841.2 to 4634.4)	21630 (19335 to 23582)	245.6 (213.6 to 269.1)	768826 (709374 to 825790)	2298.4 (2100.5 to 2484)	44479 (39745 to 48195)	150.9 (133.8 to 164)	56.4 (37.7 to 73.1)	-46.1 (-51.7 to -40)	105.6 (82 to 130.9)	-38.5 (-44.7 to -30.4)
	Male	901372 (830023 to 995658)	6502 (6020.9 to 7142.4)	34038 (31468 to 37505)	322.1 (295.4 to 352.9)	1256599 (1175477 to 1399982)	3393.6 (3160.6 to 3734)	58320 (53654 to 63323)	177.2 (161.5 to 192.5)	39.4 (23.7 to 53.2)	-47.8 (-53.8 to -43)	71.3 (50.1 to 87.9)	-45 (-51.8 to -39.9)
Stroke	Both	597142 (547356 to 651017)	2324.3 (2051.8 to 2547)	21698 (18964 to 23908)	120.7 (102.8 to 133.5)	884768 (812248 to 943655)	1262.2 (1153.5 to 1346.3)	40912 (36741 to 43849)	66.2 (58.7 to 71.3)	48.2 (33.4 to 63.4)	-45.7 (-51 to -38.3)	88.6 (67.7 to 118.5)	-45.1 (-50.6 to -35.4)
	Female	278177 (252616 to 307433)	2273.2 (1968 to 2521.3)	10320 (8721 to 11496)	119.3 (97.9 to 132.8)	428596 (387323 to 465444)	1252.8 (1126.4 to 1360.2)	20239 (17684 to 22040)	68.2 (59 to 74.4)	54.1 (38.2 to 74)	-44.9 (-50.5 to -34.8)	96.1 (73.7 to 139.3)	-42.8 (-49 to -28.2)
	Male	318965 (283986 to 361137)	2353.7 (2039.8 to 2680)	11378 (9864 to 13008)	120.5 (99.8 to 136.2)	456172 (420688 to 490032)	1274.9 (1173.4 to 1371.5)	20673 (18463 to 22410)	64.8 (57.3 to 70.3)	43 (23.7 to 62.9)	-45.8 (-52.2 to -38.4)	81.7 (55.5 to 109.5)	-46.3 (-53.9 to -37.9)
Hypertensive heart disease	Both	147285 (121914 to 188151)	645.8 (531.2 to 785.7)	6380 (5185 to 7919)	37.2 (30.2 to 47.5)	340486 (302456 to 368968)	498.6 (436.9 to 540.6)	18477 (15571 to 20027)	30.2 (25.1 to 32.8)	131.2 (71.3 to 184.6)	-22.8 (-41.5 to -5.7)	189.6 (111.3 to 262.6)	-19 (-41.9 to 1.3)
	Female	70435 (55676 to 100956)	660.5 (519.6 to 984.5)	3284 (2572 to 5012)	39.9 (31.1 to 62.3)	161839 (134194 to 180747)	491.3 (402.4 to 549.1)	9443 (7424 to 10624)	32.3 (25.1 to 36.4)	129.8 (37.4 to 201)	-25.6 (-57.7 to -2.6)	187.5 (57.6 to 283.8)	-19.2 (-57.2 to 8.2)
	Male	76850 (58370 to 101299)	621.3 (472.1 to 788.9)	3096 (2315 to 4031)	33.6 (25.1 to 43.2)	178647 (151316 to 193693)	508 (429.9 to 551.8)	9035 (7404 to 9867)	28.4 (23.2 to 31.1)	132.5 (62.2 to 213.2)	-18.2 (-40.2 to 8.6)	191.9 (104.1 to 292.1)	-15.5 (-38.3 to 11.4)
Non-rheumatic valvular heart disease	Both	12828 (10640 to 15249)	41 (35.1 to 46.7)	409 (351 to 466)	1.8 (1.6 to 2.1)	25047 (23114 to 28517)	32.3 (29.8 to 36.9)	997 (911 to 1171)	1.5 (1.3 to 1.7)	95.3 (57.7 to 144.3)	-21.2 (-32.8 to -4.7)	143.9 (108.7 to 193.3)	-20.3 (-32.1 to -6.2)
	Female	6463 (5144 to 7922)	44.4 (36.9 to 51.6)	218 (181 to 254)	2.1 (1.8 to 2.6)	12079 (11143 to 13366)	32.1 (29.4 to 35.5)	516 (466 to 572)	1.6 (1.4 to 1.8)	86.9 (47.9 to 147.7)	-27.7 (-39.9 to -8.1)	137 (95.2 to 198.7)	-24.4 (-41 to -9)
	Male	6364 (5174 to 8130)	37.2 (31.1 to 46.2)	191 (160 to 238)	1.5 (1.3 to 1.9)	12968 (11376 to 15382)	32.6 (28.6 to 39.4)	481 (421 to 606)	1.4 (1.2 to 1.8)	103.8 (47.4 to 164.1)	-12.4 (-34.9 to 9.3)	151.8 (87 to 219.4)	-11.1 (-32.2 to 7.9)
Cardiomyopathy and myocarditis	Both	38743 (26335 to 60877)	77.8 (59.5 to 104.7)	775 (586 to 1046)	2.7 (2 to 3.5)	37984 (28350 to 47302)	50.5 (37.2 to 62.7)	1271 (884 to 1590)	1.9 (1.3 to 2.4)	-2 (-44 to 53.9)	-35.2 (-57.1 to -10.1)	64.1 (3 to 127)	-29.1 (-49.6 to -7.3)
	Female	16748 (10543 to 28109)	67.7 (48.6 to 102)	335 (240 to 502)	2.5 (1.6 to 3.7)	13755 (9709 to 17107)	38 (26.4 to 46.5)	487 (312 to 583)	1.5 (1 to 1.8)	-17.9 (-55.6 to 42.8)	-43.9 (-61.8 to -16.7)	45.7 (-4.9 to 111.8)	-38 (-62 to -8.9)
	Male	21995 (13585 to 34818)	86.9 (59.3 to 122.3)	440 (298 to 621)	2.8 (2 to 3.6)	24229 (16640 to 32675)	62.8 (43 to 84.8)	784 (507 to 1074)	2.3 (1.4 to 3.1)	10.2 (-38.6 to 71.4)	-27.7 (-54.4 to 1.1)	78.1 (7.6 to 149)	-20.4 (-42.4 to 3.1)
Atrial fibrillation and flutter	Both	13784 (10574 to 17796)	75.3 (57.3 to 95.6)	372 (263 to 449)	3.1 (2.1 to 3.8)	47997 (37710 to 60548)	75.3 (59.9 to 94.6)	1766 (1510 to 2003)	3.2 (2.7 to 3.6)	248.2 (216.7 to 301.7)	0 (-10.4 to 19.1)	375.4 (283.3 to 587.7)	1.9 (-18.7 to 51.5)
	Female	7629 (5678 to 9929)	87 (63.1 to 111.4)	248 (158 to 305)	3.9 (2.4 to 4.9)	26357 (21003 to 33260)	84.5 (67.7 to 106.3)	1020 (864 to 1219)	3.8 (3.2 to 4.5)	245.5 (209.2 to 338.2)	-2.9 (-14.9 to 26.8)	310.9 (222.5 to 607.6)	-3.3 (-25.2 to 70.6)
	Male	6154 (4531 to 8137)	61.5 (47 to 79.5)	123 (88 to 160)	2.1 (1.4 to 2.8)	21640 (16722 to 27615)	66.5 (51.3 to 84.2)	747 (517 to 854)	2.6 (1.8 to 3)	251.6 (194.6 to 302.9)	8 (-8.7 to 21.6)	505.3 (299.3 to 691.8)	23.5 (-14.7 to 59.1)
Aortic aneurysm	Both	6096 (4725 to 7559)	22.2 (17.5 to 26.9)	233 (183 to 282)	1.1 (0.9 to 1.3)	14815 (13371 to 16363)	20 (18 to 22)	671 (598 to 740)	1 (0.9 to 1.1)	143 (86.4 to 229.6)	-10.1 (-30 to 19.9)	188.1 (123.7 to 286.5)	-8.2 (-27.8 to 21)

Cause	Sex	1990				2019				1990-2019 percent change (%)			
		DALYs		Death		DALYs		Death		DALYs		Death	
		Number	Rate	Number	Rate	Number	Rate	Number	Rate	Number	Rate	Number	Rate
	Female	1485 (1314 to 1764)	12 (10.6 to 14.1)	62 (54 to 72)	0.7 (0.6 to 0.8)	3773 (3447 to 4120)	10.2 (9.3 to 11)	170 (154 to 186)	0.5 (0.5 to 0.6)	154.2 (109.5 to 194.3)	-15.7 (-30.1 to -2.7)	175.8 (128.1 to 219.9)	-20.8 (-34.7 to -5.4)
	Male	4612 (3297 to 5996)	31.9 (22.7 to 41)	171 (122 to 219)	1.6 (1.1 to 2)	11042 (9693 to 12438)	29.8 (26.1 to 33.4)	501 (437 to 560)	1.5 (1.3 to 1.7)	139.4 (70 to 258.5)	-6.6 (-32.8 to 39.5)	192.6 (110.8 to 338)	-3.9 (-30.1 to 44.1)
Peripheral artery disease	Both	1391 (781 to 2290)	7.1 (4.1 to 11.4)	16 (12 to 23)	0.1 (0.1 to 0.2)	5932 (4078 to 8689)	9.2 (6.3 to 13.6)	171 (123 to 197)	0.3 (0.2 to 0.3)	326.4 (254 to 443.3)	30.3 (9.2 to 64)	949.7 (540.7 to 1414.6)	153.3 (45 to 280.4)
	Female	766 (400 to 1310)	8.1 (4.4 to 13.6)	7 (4 to 14)	0.1 (0.1 to 0.2)	2986 (1764 to 4677)	9.4 (5.6 to 14.8)	73 (28 to 90)	0.3 (0.1 to 0.3)	289.8 (200.9 to 410)	16.9 (-11.3 to 51.1)	888.9 (242.3 to 2199.8)	138.5 (-16.8 to 482.5)
	Male	625 (381 to 994)	6 (3.7 to 9.4)	9 (6 to 12)	0.1 (0.1 to 0.2)	2945 (2225 to 4088)	9 (6.7 to 12.5)	99 (86 to 111)	0.3 (0.3 to 0.4)	371.3 (287 to 507.4)	49.8 (26.9 to 88.7)	999.4 (721.3 to 1506.3)	170.1 (101.7 to 296.8)
Endocarditis	Both	10692 (7615 to 14294)	21.4 (17.7 to 29.2)	214 (176 to 292)	0.7 (0.5 to 1.1)	11411 (9593 to 16793)	14.4 (12 to 21.1)	384 (322 to 596)	0.6 (0.5 to 0.9)	6.7 (-26.6 to 85.3)	-32.8 (-46.9 to -17.4)	79.9 (43.5 to 131.4)	-20.8 (-35.2 to -5.2)
	Female	5266 (2877 to 7665)	20.5 (15 to 29.6)	101 (73 to 148)	0.7 (0.5 to 1.3)	4784 (3832 to 8156)	12.7 (10 to 21.3)	179 (139 to 315)	0.5 (0.4 to 0.9)	-9.2 (-45.1 to 100.4)	-37.9 (-55.5 to -18.4)	77 (27.8 to 138.4)	-19.4 (-35.2 to 3.2)
	Male	5425 (3944 to 7818)	22.2 (17.4 to 33)	112 (88 to 166)	0.7 (0.5 to 1.1)	6627 (5329 to 9588)	16.1 (12.6 to 23.1)	205 (159 to 314)	0.6 (0.4 to 0.9)	22.1 (-18.9 to 86.1)	-27.7 (-46.3 to -8.7)	82.5 (36.4 to 136)	-20.2 (-38.9 to -4.1)
Other cardiovascular and circulatory diseases	Both	133937 (112618 to 158625)	264.9 (228.7 to 320.1)	2311 (1998 to 2839)	8 (6.9 to 10.9)	158895 (141293 to 179448)	210.8 (188.5 to 235.6)	5121 (4525 to 5574)	7.9 (6.9 to 8.6)	18.6 (-3.1 to 41.4)	-20.4 (-37.1 to -7.8)	121.6 (65.1 to 165.6)	-1.7 (-35.4 to 16.2)
	Female	60506 (48794 to 73508)	240.5 (203.3 to 303.7)	1018 (854 to 1346)	7.2 (6.1 to 11.2)	69886 (60916 to 81032)	189.4 (166 to 216.9)	2325 (1953 to 2590)	7.5 (6.3 to 8.4)	15.5 (-10.2 to 46.8)	-21.3 (-41.9 to -6.2)	128.4 (52.4 to 180.5)	4.2 (-41.7 to 27)
	Male	73432 (60364 to 90241)	287.3 (243.6 to 359.6)	1293 (1095 to 1635)	8.8 (7.5 to 11.6)	89010 (79513 to 99807)	232.1 (208.4 to 259.5)	2796 (2492 to 3085)	8.3 (7.3 to 9.2)	21.2 (-2.1 to 46.7)	-19.2 (-37.4 to -4.2)	116.3 (62.7 to 165.9)	-5.5 (-32 to 13.9)
Chronic respiratory diseases	Both	365429 (315783 to 425585)	1113.2 (983.7 to 1275.4)	8207 (7169 to 9874)	42.6 (36.1 to 52.6)	587911 (521418 to 661392)	794.1 (705.2 to 886.5)	16835 (14588 to 18193)	26.9 (23.2 to 29.1)	60.9 (42.1 to 77)	-28.7 (-38.1 to -21.2)	105.1 (67.2 to 137.4)	-36.8 (-49 to -26.6)
	Female	159388 (129921 to 190367)	979.1 (792.3 to 1180.9)	3362 (2545 to 4272)	36.2 (27.2 to 47.8)	252949 (219784 to 289416)	690.4 (600.6 to 784.4)	6764 (5356 to 7883)	22.4 (17.5 to 26.1)	58.7 (37.3 to 102.7)	-29.5 (-40.4 to -9.6)	101.2 (54.6 to 202)	-38.1 (-55.1 to -10.1)
	Male	206041 (178519 to 241938)	1241.6 (1100.8 to 1472.1)	4845 (4272 to 6048)	49.4 (42.9 to 62.7)	334963 (296527 to 374789)	898.6 (801.8 to 997.7)	10071 (8775 to 10865)	31.4 (27.6 to 34)	62.6 (38.4 to 81.1)	-27.6 (-39.9 to -20.1)	107.9 (60.7 to 140.2)	-36.4 (-50.7 to -27.3)
Chronic obstructive pulmonary disease	Both	153371 (138121 to 176277)	555.9 (495 to 646.6)	4230 (3709 to 5228)	23.4 (20.1 to 29.4)	372569 (338458 to 404911)	517.2 (471 to 560.8)	12557 (10993 to 13621)	20.3 (17.7 to 22.1)	142.9 (109 to 168)	-7 (-20.6 to 3.2)	196.9 (139.3 to 246.1)	-13 (-30.5 to 1.7)
	Female	63198 (49406 to 77006)	466.8 (365.9 to 593)	1641 (1075 to 2305)	19 (12.8 to 27.4)	151368 (131933 to 170196)	427.5 (371.6 to 480.7)	4855 (3932 to 5781)	16.4 (12.9 to 19.6)	139.5 (101.2 to 250.6)	-8.4 (-26.5 to 34.4)	195.9 (111.8 to 437.2)	-13.9 (-38.3 to 49.6)
	Male	90173 (79421 to 109525)	641.6 (565.9 to 776.5)	2588 (2216 to 3296)	28 (23.5 to 35.3)	221200 (199050 to 240391)	607.8 (549.4 to 662)	7701 (6760 to 8327)	24.2 (21.3 to 26.3)	145.3 (97.6 to 179.1)	-5.3 (-23.3 to 7.8)	197.5 (126 to 253.6)	-13.4 (-32.9 to 2.9)
Pneumoconiosis	Both	443 (342 to 546)	1.4 (1.1 to 1.8)	12 (9 to 16)	0.1 (0 to 0.1)	2422 (2010 to 2890)	3.1 (2.6 to 3.7)	68 (59 to 77)	0.1 (0.1 to 0.1)	446.6 (320.8 to 635.9)	117.3 (68.1 to 191.5)	452 (310.1 to 725)	89.6 (41.9 to 174.8)
	Female	141 (104 to 189)	0.9 (0.6 to 1.1)	2 (1 to 3)	0 (0 to 0)	1176 (875 to 1575)	2.9 (2.2 to 3.8)	17 (13 to 20)	0.1 (0 to 0.1)	736.4 (551.6 to 970.1)	237.9 (166.8 to 331.6)	631.7 (392.4 to 1106.6)	128.3 (46.9 to 265.4)

Cause	Sex	1990				2019				1990-2019 percent change (%)			
		DALYs		Death		DALYs		Death		DALYs		Death	
		Number	Rate	Number	Rate	Number	Rate	Number	Rate	Number	Rate	Number	Rate
	Male	303 (208 to 400)	2 (1.4 to 2.6)	10 (7 to 13)	0.1 (0.1 to 0.1)	1246 (1056 to 1429)	3.4 (2.9 to 3.9)	51 (43 to 59)	0.2 (0.1 to 0.2)	311.9 (189.8 to 556.4)	69.8 (21.3 to 171)	410.8 (256.3 to 774.4)	77.6 (24.5 to 207.6)
Asthma	Both	200080 (159763 to 248232)	529.3 (431 to 652)	3806 (3075 to 4777)	18.5 (14 to 24.9)	178733 (141139 to 230621)	232.3 (185 to 299.3)	3606 (3095 to 4030)	5.6 (4.8 to 6.2)	-10.7 (-21.8 to 0.7)	-56.1 (-64.9 to -47.5)	-5.3 (-29.6 to 21.9)	-69.7 (-78.9 to -59.7)
	Female	90398 (69201 to 116418)	485.3 (363.6 to 654.1)	1646 (1112 to 2374)	16.5 (9.8 to 26.1)	84388 (64903 to 108840)	220.8 (169.9 to 284.9)	1645 (1209 to 1935)	5.2 (3.8 to 6.1)	-6.6 (-22.3 to 8.2)	-54.5 (-67.1 to -42.2)	-0.1 (-36.7 to 37.1)	-68.2 (-81.8 to -54.6)
	Male	109682 (89124 to 136252)	571 (477.6 to 690.7)	2160 (1755 to 2738)	20.6 (16.5 to 27.8)	94344 (74917 to 121556)	243.7 (194.8 to 311.8)	1961 (1728 to 2212)	6 (5.3 to 6.8)	-14 (-25.1 to -0.2)	-57.3 (-65.1 to -47.4)	-9.2 (-32 to 22)	-70.9 (-79.7 to -60.8)
Interstitial lung disease and pulmonary sarcoidosis	Both	3977 (3121 to 4908)	13.1 (10.1 to 16.3)	106 (78 to 137)	0.5 (0.4 to 0.7)	11389 (7941 to 13899)	14.7 (10.3 to 17.7)	371 (233 to 441)	0.6 (0.4 to 0.7)	186.4 (113.7 to 262)	12.1 (-15.4 to 46.1)	251.7 (139.8 to 389.6)	5.2 (-26.8 to 52.6)
	Female	1829 (1277 to 2418)	12.7 (8.6 to 17.4)	49 (29 to 72)	0.5 (0.3 to 0.8)	5195 (3486 to 6506)	13.4 (8.8 to 16.5)	158 (84 to 199)	0.5 (0.3 to 0.6)	184.1 (124.6 to 288.8)	5.7 (-20.9 to 57.7)	219.7 (124.2 to 481.2)	-7.6 (-37.7 to 75.6)
	Male	2148 (1621 to 2839)	13.6 (10.2 to 17.7)	56 (39 to 77)	0.6 (0.4 to 0.8)	6194 (3665 to 7717)	16.1 (9.7 to 19.6)	214 (114 to 263)	0.6 (0.4 to 0.8)	188.3 (75.7 to 288.3)	18.4 (-24.5 to 66.1)	279.7 (118.1 to 480.5)	17.9 (-30 to 89.6)
Other chronic respiratory diseases	Both	7557 (5760 to 11412)	13.4 (10.8 to 18.2)	53 (35 to 102)	0.2 (0.1 to 0.2)	22800 (16969 to 27606)	26.8 (19.6 to 32.5)	233 (78 to 329)	0.3 (0.1 to 0.5)	201.7 (66.1 to 302.4)	98.9 (21.4 to 156.2)	338.6 (-8.3 to 775.1)	100.5 (-40.8 to 249.1)
	Female	3822 (2866 to 6091)	13.5 (10.7 to 19.2)	23 (13 to 52)	0.1 (0.1 to 0.2)	10822 (8414 to 12955)	25.9 (19.7 to 31.1)	89 (31 to 124)	0.3 (0.1 to 0.4)	183.1 (58.1 to 276.1)	91.9 (19.2 to 149)	288 (-22.5 to 799.2)	77.2 (-47.2 to 285.6)
	Male	3735 (2850 to 5532)	13.4 (10.5 to 18.1)	30 (20 to 55)	0.2 (0.1 to 0.3)	11978 (8365 to 14880)	27.6 (18.9 to 34.6)	144 (43 to 212)	0.4 (0.1 to 0.6)	220.7 (73.3 to 341.9)	106.4 (22.5 to 175.9)	376.8 (-1.6 to 887.9)	116.4 (-40.5 to 323.9)
Digestive diseases	Both	334644 (284323 to 384811)	857.1 (752.3 to 977.3)	6936 (6201 to 7698)	28.9 (24.6 to 34.7)	438714 (374268 to 522113)	549.5 (473.1 to 649.1)	11482 (10418 to 12803)	17.3 (15.5 to 19.3)	31.1 (10.6 to 58.8)	-35.9 (-42.8 to -26)	65.5 (42.6 to 100.1)	-40.3 (-50.8 to -28.2)
	Female	149516 (127232 to 173218)	775.4 (659.9 to 920.7)	2893 (2492 to 3401)	26 (20.2 to 34.2)	195353 (161474 to 240589)	497.9 (418 to 602.2)	4777 (4280 to 5377)	15.2 (13.4 to 17.1)	30.7 (9.6 to 54.5)	-35.8 (-42.2 to -26.3)	65.1 (41.3 to 102.7)	-41.5 (-55.2 to -26.3)
	Male	185128 (157492 to 212481)	927.5 (816.3 to 1033.1)	4043 (3497 to 4466)	31.3 (27.5 to 35.1)	243361 (209662 to 285236)	601.7 (523.5 to 698.8)	6705 (5968 to 7561)	19.4 (17.3 to 21.9)	31.5 (9.8 to 65.5)	-35.1 (-44.1 to -20.1)	65.9 (38.7 to 113.3)	-38.1 (-46.3 to -23.1)
Cirrhosis and other chronic liver diseases	Both	148682 (126412 to 169275)	392.8 (346.5 to 445)	3999 (3518 to 4520)	15.6 (13 to 18.8)	184174 (166611 to 204894)	235.5 (213.1 to 261.9)	6832 (6143 to 7638)	9.9 (8.8 to 11.1)	23.9 (1.6 to 56.8)	-40 (-50.5 to -26.1)	70.8 (40.4 to 110.2)	-36.6 (-48.9 to -21.6)
	Female	61165 (49625 to 72012)	318.9 (266.9 to 387.5)	1591 (1325 to 1952)	13.2 (9.9 to 18.2)	65528 (58248 to 73192)	174.9 (155.9 to 195.7)	2593 (2301 to 2994)	7.9 (6.9 to 9.2)	7.1 (-14.8 to 43.2)	-45.2 (-55.1 to -30.6)	63 (28.1 to 112.5)	-40.3 (-58.4 to -17.7)
	Male	87517 (72865 to 101773)	458.5 (391.8 to 524.8)	2408 (2046 to 2745)	17.6 (15.2 to 20.2)	118647 (103739 to 133666)	296.2 (260.5 to 333.4)	4238 (3713 to 4791)	11.9 (10.4 to 13.4)	35.6 (5.6 to 76)	-35.4 (-49.2 to -17.2)	76 (38.4 to 124.3)	-32.7 (-44.7 to -14.6)
Upper digestive system diseases	Both	89344 (67768 to 121701)	246.3 (190.3 to 324.8)	1373 (1167 to 1647)	6.7 (5.3 to 8.4)	129964 (86183 to 199426)	151.7 (103.4 to 229)	1486 (1257 to 1663)	2.4 (2 to 2.7)	45.5 (19.5 to 67.4)	-38.4 (-49.3 to -28.3)	8.2 (-10.9 to 29.1)	-64.6 (-71.7 to -55.6)
	Female	41136 (29727 to 58040)	230.2 (167.6 to 317.6)	541 (418 to 702)	5.8 (4.1 to 8.3)	66304 (42485 to 103922)	156.2 (103.2 to 239.7)	659 (548 to 774)	2.2 (1.9 to 2.6)	61.2 (33.8 to 84.3)	-32.1 (-44.7 to -21)	21.9 (-8.7 to 75.6)	-61.2 (-73.6 to -41.8)
	Male	48208 (37337 to 64737)	261.1 (206.5 to 337.3)	832 (703 to 1004)	7.6 (6.2 to 9.5)	63660 (42832 to 95370)	147.6 (102.4 to 216.3)	826 (670 to 935)	2.5 (2 to 2.9)	32.1 (5.3 to 56)	-43.5 (-54 to -32.4)	-0.7 (-21.3 to 19.5)	-66.7 (-72.8 to -59.7)
Appendicitis	Both	12725 (7640 to 18521)	23.8 (16.9 to 30.5)	225 (153 to 296)	0.7 (0.6 to 1)	7536 (6120 to 9475)	9.4 (7.7 to 11.8)	161 (136 to 211)	0.2 (0.2 to 0.3)	-40.8 (-62.1 to 19.9)	-60.4 (-71.7 to -33)	-28.2 (-49.8 to 34.8)	-66.9 (-78.3 to -49.3)

Cause	Sex	1990				2019				1990-2019 percent change (%)			
		DALYs		Death		DALYs		Death		DALYs		Death	
		Number	Rate	Number	Rate	Number	Rate	Number	Rate	Number	Rate	Number	Rate
	Female	5401 (3168 to 9141)	20.7 (14.2 to 31.8)	95 (63 to 153)	0.7 (0.5 to 1.1)	3369 (2639 to 4324)	8.7 (6.9 to 11.1)	72 (57 to 104)	0.2 (0.2 to 0.3)	-37.6 (-63 to 28.5)	-57.8 (-74.2 to -27.1)	-24.9 (-59.2 to 37.6)	-65.7 (-81.9 to -44.8)
	Male	7324 (4046 to 11678)	26.7 (16.5 to 36.5)	129 (76 to 185)	0.8 (0.5 to 1)	4166 (3371 to 5410)	10.1 (8.1 to 13.2)	90 (69 to 122)	0.3 (0.2 to 0.3)	-43.1 (-67.5 to 28)	-62.1 (-74.5 to -24.5)	-30.6 (-59.9 to 52.1)	-67.4 (-75.7 to -38.8)
Paralytic ileus and intestinal obstruction	Both	35779 (24507 to 48726)	59.2 (46.1 to 76.5)	567 (438 to 739)	1.9 (1.5 to 2.5)	21504 (15690 to 26526)	31.1 (22.9 to 38.2)	744 (536 to 902)	1.2 (0.9 to 1.5)	-39.9 (-65.1 to 2.5)	-47.5 (-67.3 to -21.9)	31.1 (-17.8 to 86)	-36.3 (-56.5 to -13.2)
	Female	17002 (11858 to 22964)	58.8 (46 to 75.2)	280 (217 to 359)	2 (1.5 to 2.6)	9358 (7139 to 11345)	27.8 (21.3 to 33.6)	323 (248 to 400)	1.1 (0.8 to 1.3)	-45 (-64.1 to -8.4)	-52.8 (-66.6 to -30.3)	15.1 (-18.8 to 66.5)	-46.1 (-60.2 to -21.1)
	Male	18777 (11672 to 27623)	59 (41.5 to 84)	287 (198 to 414)	1.7 (1.3 to 2.5)	12146 (7855 to 15803)	34.3 (22.4 to 44.6)	421 (266 to 521)	1.3 (0.8 to 1.6)	-35.3 (-69.4 to 27.3)	-41.9 (-70.9 to 3.5)	46.9 (-27.2 to 147.6)	-23.4 (-55.1 to 6.1)
Inguinal, femoral, and abdominal hernia	Both	10950 (7267 to 16026)	20.1 (14.4 to 27)	64 (41 to 102)	0.3 (0.2 to 0.4)	14066 (9860 to 19548)	17.4 (12.3 to 23.9)	102 (60 to 129)	0.2 (0.1 to 0.2)	28.5 (-11.4 to 66.9)	-13.2 (-30.6 to 3.5)	60.8 (-20.1 to 201.4)	-33.9 (-53.8 to 3.5)
	Female	3902 (2495 to 6125)	14 (9.8 to 19.6)	22 (12 to 42)	0.2 (0.1 to 0.3)	4867 (3356 to 6729)	12.2 (8.4 to 16.9)	41 (17 to 53)	0.1 (0.1 to 0.2)	24.7 (-26.7 to 67.9)	-12.6 (-39.2 to 7.6)	83.3 (-34.9 to 230.9)	-29.5 (-54 to 6.6)
	Male	7048 (4691 to 10368)	25.8 (18 to 35.1)	41 (24 to 69)	0.3 (0.2 to 0.5)	9200 (6456 to 12779)	22.5 (16 to 30.9)	62 (33 to 78)	0.2 (0.1 to 0.2)	30.5 (-8.3 to 70.8)	-12.8 (-30 to 4.5)	48.7 (-19.7 to 193.7)	-38.3 (-61.1 to 3.5)
Inflammatory bowel disease	Both	4022 (3121 to 5103)	11.1 (8.3 to 14.2)	51 (33 to 68)	0.2 (0.1 to 0.3)	9481 (6679 to 12439)	11 (7.6 to 14.2)	132 (55 to 164)	0.2 (0.1 to 0.2)	135.7 (84.2 to 177)	-0.6 (-15.8 to 13.6)	158 (43.7 to 247.6)	-11.5 (-42.4 to 25.1)
	Female	2062 (1505 to 2683)	11.5 (8 to 15.4)	25 (13 to 40)	0.2 (0.1 to 0.4)	4612 (3184 to 6078)	10.9 (7.3 to 14)	63 (24 to 80)	0.2 (0.1 to 0.2)	123.7 (77.8 to 177.7)	-5.2 (-22.4 to 14.6)	147.8 (48.3 to 287.1)	-15 (-56.9 to 45.1)
	Male	1961 (1493 to 2541)	10.7 (8.1 to 13.8)	26 (17 to 34)	0.2 (0.1 to 0.3)	4870 (3313 to 6373)	11.2 (7.6 to 14.5)	69 (28 to 87)	0.2 (0.1 to 0.2)	148.3 (81.9 to 194)	5.1 (-17.2 to 21.3)	168 (33.5 to 272.2)	-3.7 (-44.2 to 32.3)
Vascular intestinal disorders	Both	2526 (2050 to 3161)	10.9 (8 to 14.5)	109 (79 to 147)	0.6 (0.4 to 0.9)	6274 (5188 to 7062)	9 (7.5 to 10.2)	323 (268 to 366)	0.5 (0.4 to 0.6)	148.4 (92.3 to 198.3)	-16.8 (-39.3 to 5.8)	195.4 (113.7 to 285.3)	-17.5 (-42.6 to 13.8)
	Female	1194 (827 to 1667)	11.3 (6.7 to 17.1)	57 (32 to 88)	0.7 (0.4 to 1.1)	2840 (2301 to 3225)	8.4 (6.8 to 9.5)	151 (125 to 174)	0.5 (0.4 to 0.6)	137.9 (73.2 to 226.7)	-25.8 (-49.9 to 15.4)	165.7 (73.8 to 335.1)	-27.8 (-55 to 28.1)
	Male	1332 (1073 to 1601)	10.2 (8.2 to 12.5)	52 (41 to 65)	0.5 (0.4 to 0.7)	3433 (2729 to 3951)	9.7 (7.7 to 11.2)	171 (134 to 199)	0.5 (0.4 to 0.6)	157.9 (99.6 to 232.2)	-5.1 (-24 to 21)	227.7 (159.6 to 318.4)	-2.2 (-22.7 to 23.5)
Gallbladder and biliary diseases	Both	18632 (13399 to 25284)	57 (42.2 to 74.7)	224 (179 to 294)	1.3 (1 to 1.8)	40972 (29746 to 55431)	51.6 (37.6 to 68.2)	785 (498 to 941)	1.3 (0.8 to 1.6)	119.9 (80.6 to 156.6)	-9.5 (-23.9 to 3.7)	250.9 (123.1 to 391.5)	-2.6 (-32.2 to 30)
	Female	12240 (8611 to 16801)	75.1 (54.2 to 100.7)	124 (90 to 168)	1.5 (1 to 2.2)	27392 (19371 to 38247)	68.4 (48.9 to 92.1)	440 (260 to 538)	1.5 (0.9 to 1.9)	123.8 (87.4 to 153.3)	-9 (-21.8 to 1.8)	255.9 (122.3 to 388.3)	1.2 (-30.6 to 40.9)
	Male	6392 (4754 to 8591)	39.4 (29.7 to 51.7)	100 (75 to 147)	1.1 (0.9 to 1.6)	13579 (10099 to 17481)	35.2 (26.2 to 44.3)	345 (216 to 419)	1.1 (0.7 to 1.3)	112.4 (63.5 to 165)	-10.7 (-31.1 to 11)	244.8 (107.6 to 434.5)	-2.3 (-38.5 to 47.8)
Pancreatitis	Both	5401 (4183 to 7567)	18.2 (13.6 to 26.6)	171 (125 to 258)	0.8 (0.6 to 1.3)	10144 (8531 to 12698)	13.2 (10.9 to 16.2)	398 (308 to 480)	0.6 (0.5 to 0.7)	87.8 (46 to 139.3)	-27.8 (-43 to -3.9)	133.5 (76.5 to 226.3)	-27 (-48.6 to 7.1)
	Female	2206 (1678 to 3115)	16.4 (11.1 to 25.2)	77 (49 to 121)	0.8 (0.5 to 1.4)	4395 (3247 to 5305)	12.2 (8.9 to 14.5)	197 (135 to 239)	0.6 (0.4 to 0.8)	99.3 (49.1 to 158.7)	-25.8 (-48.5 to 4.8)	156.2 (68.6 to 284.1)	-23.8 (-53.7 to 24.1)
	Male	3195 (2420 to 4776)	19.6 (14.9 to 29.2)	94 (70 to 143)	0.8 (0.6 to 1.1)	5750 (4703 to 7897)	14.2 (11.6 to 18.9)	201 (156 to 267)	0.6 (0.4 to 0.7)	80 (42.9 to 135.6)	-27.6 (-42.7 to -5.2)	114.8 (65.8 to 191.8)	-26.1 (-45.4 to -1)
Other digestive diseases	Both	6584 (4817 to 9036)	17.8 (14.6 to 23.6)	154 (121 to 213)	0.8 (0.6 to 1.3)	14599 (9892 to 16953)	19.6 (13.5 to 22.7)	519 (354 to 594)	0.8 (0.6 to 0.9)	121.7 (24.3 to 230.4)	10.2 (-29.7 to 43.9)	237 (105.5 to 362.8)	1.8 (-37.1 to 46.1)
	Female	3209 (2282 to 5004)	18.5 (13.8 to 27.2)	80 (55 to 126)	0.9 (0.5 to 1.7)	6688 (4382 to 7939)	18.4 (12.1 to 21.7)	237 (148 to 280)	0.8 (0.5 to 0.9)	108.4 (9 to 217)	-1 (-34.3 to 42.2)	195.4 (85.4 to 357)	-13.7 (-52.7 to 46.1)
	Male	3375 (2357 to 4709)	16.6 (12.6 to 20.7)	74 (54 to 95)	0.7 (0.5 to 0.9)	7911 (5362 to 9226)	20.8 (14.4 to 24.3)	281 (190 to 326)	0.9 (0.6 to 1)	134.4 (24.4 to 269.1)	25.4 (-25 to 78)	282.5 (129.2 to 462)	29.1 (-18.4 to 79.6)

Cause	Sex	1990				2019				1990-2019 percent change (%)			
		DALYs		Death		DALYs		Death		DALYs		Death	
		Number	Rate	Number	Rate	Number	Rate	Number	Rate	Number	Rate	Number	Rate
Neurological disorders	Both	611640 (324538 to 1050361)	1454.4 (829 to 2403.8)	4874 (2648 to 9808)	32.6 (13.3 to 76.4)	1103459 (584174 to 1853014)	1394.2 (785.8 to 2300.9)	18338 (7975 to 41415)	32.1 (13.2 to 73.9)	80.4 (60.7 to 111.9)	-4.1 (-10.3 to 4.7)	276.2 (178.8 to 397.5)	-1.4 (-8.6 to 27.8)
	Female	329512 (158785 to 591408)	1581.4 (846.4 to 2702.7)	2420 (1209 to 5124)	31.6 (12.1 to 76.2)	615198 (293805 to 1084664)	1550.8 (797 to 2652.9)	8828 (3583 to 20333)	32.2 (12.3 to 76.3)	86.7 (65.9 to 116.3)	-1.9 (-8.6 to 8.7)	264.8 (170.3 to 409.9)	2 (-7.9 to 43.7)
	Male	282128 (162256 to 455584)	1335 (812.9 to 2098.4)	2455 (1429 to 4729)	33.7 (14.4 to 76.6)	488261 (286031 to 781489)	1242.3 (750.9 to 1967.3)	9511 (4372 to 20987)	32 (14.1 to 71.7)	73.1 (51.6 to 108.7)	-6.9 (-13 to 1.2)	287.5 (181.1 to 383.5)	-5 (-13.3 to 18.8)
Alzheimer's disease and other dementias	Both	55067 (23815 to 124608)	386.1 (169.3 to 879)	2886 (673 to 7855)	25.5 (6 to 70.2)	228128 (100792 to 497824)	386 (170.3 to 844)	13910 (3444 to 37233)	25.3 (6.4 to 67.6)	314.3 (277 to 401.2)	0 (-5.4 to 21.3)	382 (336.4 to 552.3)	-0.6 (-7.1 to 34.5)
	Female	28898 (12538 to 65752)	393.6 (174.1 to 889.1)	1566 (365 to 4267)	25.7 (6.1 to 70.7)	117436 (51647 to 258457)	405.8 (178.8 to 896.1)	7015 (1755 to 18668)	26.6 (6.6 to 71.2)	306.4 (268.4 to 420.8)	3.1 (-4.3 to 32.6)	348 (299.3 to 559.5)	3.5 (-6.1 to 53.2)
	Male	26168 (11339 to 60036)	378.5 (164.6 to 861.5)	1320 (302 to 3585)	25.3 (6 to 68.9)	110692 (49166 to 242691)	366.8 (164 to 815.6)	6895 (1707 to 18561)	24.1 (6 to 64.6)	323 (278.3 to 392.8)	-3.1 (-9.4 to 11.7)	422.4 (358.6 to 555.3)	-4.4 (-11.7 to 20)
Parkinson's disease	Both	13806 (12015 to 15388)	74.5 (64.7 to 83.5)	643 (554 to 724)	4.5 (3.8 to 5.3)	48779 (42378 to 53932)	76.8 (66.9 to 84.9)	2848 (2432 to 3155)	4.8 (4.1 to 5.3)	253.3 (207.9 to 313.5)	3.1 (-12.9 to 20.5)	343.1 (276.8 to 429.9)	6.7 (-12.6 to 28.4)
	Female	5436 (4616 to 6371)	59 (49.4 to 73.3)	256 (212 to 335)	3.5 (2.8 to 4.9)	19784 (17547 to 22026)	62.3 (54.8 to 69.8)	1120 (968 to 1338)	3.8 (3.3 to 4.7)	263.9 (209.1 to 335.9)	5.7 (-13.5 to 28.1)	336.7 (259.9 to 450.4)	9.6 (-12.6 to 39.2)
	Male	8370 (6863 to 9464)	92.1 (76.6 to 104.3)	386 (315 to 440)	5.7 (4.6 to 6.5)	28995 (22124 to 32655)	91 (69.6 to 102.5)	1728 (1287 to 1954)	5.7 (4.2 to 6.4)	246.4 (184.8 to 324.1)	-1.1 (-20.1 to 21.2)	347.4 (260.3 to 469.9)	0.3 (-21.6 to 27.3)
Idiopathic epilepsy	Both	146662 (103886 to 189513)	215 (158.5 to 275.8)	998 (675 to 1224)	1.7 (1.3 to 2)	113267 (78371 to 155611)	140.2 (96.1 to 192.5)	662 (605 to 776)	0.8 (0.7 to 1)	-22.8 (-43.4 to 6.4)	-34.8 (-51.4 to -13)	-33.7 (-49.3 to 7.6)	-51.1 (-60.7 to -26.1)
	Female	66618 (44930 to 87414)	198.5 (142.5 to 259.9)	449 (254 to 586)	1.5 (1 to 1.8)	50566 (34681 to 69739)	128 (86.5 to 177.9)	273 (243 to 345)	0.7 (0.6 to 0.9)	-24.1 (-47.3 to 9.7)	-35.5 (-53.5 to -10.3)	-39.3 (-57.5 to 18.5)	-54.4 (-65.4 to -24.4)
	Male	80044 (55298 to 105042)	230.8 (167.8 to 299.8)	550 (358 to 699)	1.8 (1.3 to 2.2)	62701 (43423 to 85677)	152 (104.6 to 207.6)	389 (353 to 459)	0.9 (0.8 to 1.1)	-21.7 (-42.3 to 10.4)	-34.1 (-50.7 to -10.8)	-29.2 (-47.5 to 21.3)	-48.3 (-59.4 to -17.6)
Multiple sclerosis	Both	11056 (8407 to 14846)	29 (21.9 to 40.2)	144 (96 to 239)	0.4 (0.3 to 0.8)	26915 (21772 to 34918)	29.2 (23.7 to 37.4)	396 (328 to 587)	0.5 (0.4 to 0.7)	143.4 (103.1 to 191.7)	0.5 (-18.6 to 20.3)	175.2 (96.6 to 288.4)	4.8 (-28.2 to 55.7)
	Female	6323 (4733 to 8616)	33.6 (24.8 to 45.1)	73 (47 to 118)	0.5 (0.3 to 0.8)	15806 (12967 to 18985)	34.5 (28.3 to 41.4)	204 (171 to 271)	0.5 (0.4 to 0.6)	150 (78 to 209.6)	2.9 (-26 to 28.5)	177.8 (54 to 345.7)	4.4 (-40.7 to 80.2)
	Male	4734 (3403 to 7560)	24.6 (17.4 to 40.1)	71 (45 to 139)	0.4 (0.3 to 0.8)	11109 (8345 to 17558)	23.9 (18.2 to 37.3)	192 (138 to 361)	0.5 (0.3 to 0.8)	134.7 (67.5 to 189.6)	-2.9 (-33.7 to 15.5)	172.4 (59 to 266.8)	6.3 (-39.5 to 45.5)
Motor neuron disease	Both	1419 (1120 to 1936)	3.3 (2.6 to 4.4)	28 (21 to 40)	0.1 (0.1 to 0.1)	3832 (3359 to 4363)	4.7 (4.1 to 5.4)	103 (89 to 119)	0.1 (0.1 to 0.2)	170.1 (88.7 to 257.6)	43.7 (1 to 90.3)	268.2 (140 to 424.8)	61.9 (4 to 134)
	Female	758 (582 to 1109)	3.4 (2.6 to 4.9)	14 (10 to 22)	0.1 (0.1 to 0.1)	1778 (1538 to 2079)	4.4 (3.8 to 5.2)	48 (41 to 57)	0.1 (0.1 to 0.2)	134.5 (52.5 to 220.7)	32.5 (-14 to 78.4)	245.8 (104.8 to 402.8)	53.8 (-15.2 to 130.6)
	Male	661 (497 to 998)	3.2 (2.3 to 4.6)	14 (10 to 22)	0.1 (0.1 to 0.1)	2054 (1763 to 2397)	5 (4.3 to 5.8)	55 (46 to 65)	0.1 (0.1 to 0.2)	210.9 (87.8 to 336.2)	56.8 (-3.9 to 117.7)	290.4 (122.2 to 491.6)	73.5 (0.2 to 167.3)
Headache disorders	Both	356229 (76460 to 786902)	703.6 (169.9 to 1500.6)	--	--	641837 (160733 to 1355805)	705.8 (173.2 to 1503.1)	--	--	80.2 (66.5 to 117.2)	0.3 (-3.3 to 5.8)	--	--
	Female	210168 (41754 to 468537)	856.4 (188.8 to 1880.9)	--	--	391800 (88685 to 845694)	869.5 (193.5 to 1882)	--	--	86.4 (72.4 to 115.9)	1.5 (-3.1 to 6.5)	--	--

Cause	Sex	1990				2019				1990-2019 percent change (%)			
		DALYs		Death		DALYs		Death		DALYs		Death	
		Number	Rate	Number	Rate	Number	Rate	Number	Rate	Number	Rate	Number	Rate
	Male	146061 (34269 to 315951)	557.4 (151.8 to 1166.9)	--	--	250037 (73224 to 519991)	547 (154.8 to 1145.5)	--	--	71.2 (54.6 to 114.9)	-1.9 (-6.6 to 6.4)	--	--
Other neurological disorders	Both	27402 (20616 to 36454)	42.8 (32.7 to 55.5)	175 (132 to 220)	0.4 (0.3 to 0.5)	40701 (31189 to 51857)	51.5 (39.2 to 66.3)	419 (390 to 449)	0.6 (0.5 to 0.6)	48.5 (19.8 to 84.1)	20.2 (-2.5 to 47.4)	138.6 (82.4 to 224.6)	39.2 (8.1 to 95.9)
	Female	11311 (8161 to 15433)	37 (27.8 to 48.2)	61 (49 to 75)	0.3 (0.2 to 0.4)	18029 (13728 to 23321)	46.2 (34.8 to 60.1)	168 (152 to 185)	0.4 (0.4 to 0.5)	59.4 (27.1 to 97.1)	25 (1.2 to 51.5)	173.8 (113 to 251.3)	41.6 (3 to 103.5)
	Male	16091 (11800 to 21192)	48.5 (36.5 to 62.4)	114 (80 to 146)	0.5 (0.3 to 0.6)	22673 (17381 to 28523)	56.6 (43.7 to 71.6)	251 (230 to 272)	0.7 (0.6 to 0.7)	40.9 (12.1 to 78.8)	16.9 (-6.3 to 47.2)	119.7 (61.7 to 217.8)	38.6 (5.8 to 95.4)
Mental disorders	Both	1159410 (851688 to 1530200)	2254.8 (1670.1 to 2965.4)	0 (0 to 0)	0 (0 to 0)	2053871 (1516179 to 2714847)	2295.8 (1702.2 to 3033.6)	0 (0 to 0)	0 (0 to 0)	77.1 (69.8 to 84.8)	1.8 (0.7 to 2.9)	665.4 (285.7 to 1243.7)	288.7 (94.2 to 582.7)
	Female	641164 (470193 to 853922)	2583.4 (1910.9 to 3416.9)	0 (0 to 0)	0 (0 to 0)	1161796 (859248 to 1535077)	2623.6 (1927.5 to 3470.3)	0 (0 to 0)	0 (0 to 0)	81.2 (73.5 to 89.4)	1.6 (0.3 to 2.8)	718 (281.9 to 1541.3)	313.3 (92.6 to 792.4)
	Male	518245 (376570 to 680356)	1939.9 (1432.2 to 2542.8)	0 (0 to 0)	0 (0 to 0)	892076 (659461 to 1176297)	1976.3 (1455.5 to 2599.3)	0 (0 to 0)	0 (0 to 0)	72.1 (64.4 to 80.2)	1.9 (0.4 to 3.4)	427 (74.1 to 1243.1)	178.8 (-6.3 to 648.7)
Schizophrenia	Both	69046 (50674 to 88190)	160.6 (118.1 to 203.4)	--	--	158115 (114789 to 202150)	162 (118.3 to 205.9)	--	--	129 (118.2 to 140)	0.9 (-1.7 to 3.2)	--	--
	Female	31962 (23332 to 40858)	152.3 (111.8 to 193.6)	--	--	73662 (53756 to 94312)	153.1 (112.3 to 194.7)	--	--	130.5 (118.1 to 143)	0.6 (-2.8 to 3.9)	--	--
	Male	37084 (27241 to 47401)	168.5 (123.4 to 213)	--	--	84453 (61036 to 108052)	170.7 (124.7 to 216.5)	--	--	127.7 (116.1 to 139.4)	1.3 (-2 to 4.6)	--	--
Depressive disorders	Both	398047 (265359 to 558440)	848.1 (576 to 1182)	--	--	813441 (553621 to 1143612)	890.3 (605.8 to 1247.8)	--	--	104.4 (89.1 to 119.9)	5 (3.4 to 6.6)	--	--
	Female	254259 (169439 to 355505)	1098 (748.4 to 1519.5)	--	--	507146 (344690 to 709373)	1126.7 (768.4 to 1576.1)	--	--	99.5 (84.5 to 114.6)	2.6 (0.8 to 4.6)	--	--
	Male	143788 (95393 to 205725)	609.3 (413.7 to 860.1)	--	--	306295 (205722 to 437083)	660.1 (444.9 to 933.6)	--	--	113 (96.6 to 129.8)	8.3 (6.2 to 10.5)	--	--
Bipolar disorder	Both	83936 (51139 to 130234)	173.6 (107 to 267.1)	--	--	160227 (98666 to 248099)	175.8 (107.9 to 270)	--	--	90.9 (79.7 to 102.3)	1.3 (-0.4 to 3.2)	--	--
	Female	44940 (27298 to 69303)	187.2 (115.2 to 286)	--	--	84942 (51853 to 131818)	189.6 (115.6 to 291.6)	--	--	89 (77 to 101.4)	1.3 (-1.2 to 4)	--	--
	Male	38997 (23616 to 60482)	160.5 (98.7 to 246.5)	--	--	75285 (46457 to 115993)	162.6 (99.8 to 252.8)	--	--	93.1 (81.5 to 105.4)	1.3 (-1.3 to 4)	--	--
Anxiety disorders	Both	363831 (253144 to 501830)	672.1 (470.2 to 922)	--	--	608057 (422918 to 835643)	695.8 (483.3 to 954.7)	--	--	67.1 (55.5 to 80.1)	3.5 (2 to 5.1)	--	--
	Female	215823 (149896 to 295630)	825.1 (575.2 to 1129.4)	--	--	370121 (256898 to 504762)	854.2 (595.2 to 1170.7)	--	--	71.5 (60 to 84.3)	3.5 (1.2 to 5.8)	--	--
	Male	148008 (102602 to 205110)	525 (365.7 to 721.6)	--	--	237936 (164956 to 328789)	541.8 (379.5 to 747.4)	--	--	60.8 (49.4 to 74.4)	3.2 (0.8 to 5.5)	--	--
Eating disorders	Both	25251 (15750 to 37480)	42.9 (26.7 to 63.8)	0 (0 to 0)	0 (0 to 0)	50001 (31280 to 74071)	53.9 (33.5 to 80.2)	0 (0 to 0)	0 (0 to 0)	98 (79.4 to 120.8)	25.8 (21.5 to 30.2)	665.4 (285.7 to 1243.7)	288.7 (94.2 to 582.7)

Cause	Sex	1990				2019				1990-2019 percent change (%)			
		DALYs		Death		DALYs		Death		DALYs		Death	
		Number	Rate	Number	Rate	Number	Rate	Number	Rate	Number	Rate	Number	Rate
	Female	15480 (9558 to 23215)	53.4 (33.2 to 78.5)	0 (0 to 0)	0 (0 to 0)	30436 (19134 to 45141)	67.6 (42.1 to 100.3)	0 (0 to 0)	0 (0 to 0)	96.6 (77.8 to 118.7)	26.7 (21.3 to 32.5)	718 (281.9 to 1541.3)	313.3 (92.6 to 792.4)
	Male	9772 (6021 to 14695)	32.7 (19.8 to 49.9)	0 (0 to 0)	0 (0 to 0)	19564 (11877 to 29872)	40.8 (25 to 62.1)	0 (0 to 0)	0 (0 to 0)	100.2 (78.2 to 126.1)	24.7 (18.6 to 31.2)	427 (74.1 to 1243.1)	178.8 (-6.3 to 648.7)
Autism spectrum disorders	Both	34583 (22489 to 50672)	55.4 (36.2 to 80.6)	--	--	47262 (31078 to 68539)	56.4 (37 to 81.7)	--	--	36.7 (32.1 to 41.7)	1.8 (-1.4 to 5.1)	--	--
	Female	8444 (5487 to 12366)	27.5 (17.9 to 40)	--	--	11523 (7535 to 16801)	28 (18.3 to 41)	--	--	36.5 (29.9 to 43.2)	1.8 (-2.5 to 6.3)	--	--
	Male	26139 (16910 to 37974)	82.2 (53.5 to 119.9)	--	--	35739 (23526 to 51773)	83.9 (55.1 to 121.4)	--	--	36.7 (31.2 to 43)	2.1 (-1.7 to 6.4)	--	--
Attention-deficit/hyperactivity disorder	Both	22243 (12586 to 38642)	31.5 (17.9 to 54.3)	--	--	26331 (14933 to 44603)	32.4 (18.1 to 55.5)	0 (0 to 0)	0 (0 to 0)	18.4 (3.1 to 36.7)	2.8 (-9.1 to 14.1)	--	--
	Female	6634 (3729 to 11742)	19.3 (10.9 to 33.8)	--	--	7946 (4421 to 13402)	19.9 (11.1 to 33.7)	0 (0 to 0)	0 (0 to 0)	19.8 (1.9 to 41.8)	3.3 (-11.2 to 20.3)	--	--
	Male	15609 (8752 to 27065)	43.3 (24.4 to 74)	--	--	18385 (10391 to 31245)	44.4 (25.3 to 75.9)	0 (0 to 0)	0 (0 to 0)	17.8 (-1.4 to 40.2)	2.5 (-12.6 to 17.5)	--	--
Conduct disorder	Both	62327 (35601 to 98603)	74.8 (42.2 to 118.1)	--	--	50475 (28501 to 80429)	72.6 (41 to 116.1)	0 (0 to 0)	0 (0 to 0)	-19 (-24.1 to -14.4)	-2.9 (-8.9 to 2.6)	--	--
	Female	21414 (11395 to 34999)	52.5 (27.9 to 85.8)	--	--	16946 (8881 to 28374)	49.9 (26.1 to 83)	0 (0 to 0)	0 (0 to 0)	-20.9 (-27.2 to -13.7)	-4.9 (-12.7 to 3.3)	--	--
	Male	40914 (23401 to 63643)	96 (55.5 to 149)	--	--	33529 (19538 to 53349)	94.1 (54.9 to 149.1)	0 (0 to 0)	0 (0 to 0)	-18 (-24.2 to -12.5)	-2 (-9.3 to 4.7)	--	--
Idiopathic developmental intellectual disability	Both	60884 (33590 to 97087)	95.3 (52.9 to 151.2)	--	--	46923 (23606 to 79095)	55.9 (28.1 to 94.4)	0 (0 to 0)	0 (0 to 0)	-22.9 (-34.9 to -16.3)	-41.3 (-50.5 to -36.2)	--	--
	Female	26710 (14741 to 42972)	85.8 (47.4 to 138)	--	--	21417 (10858 to 35950)	52 (26.2 to 87.6)	0 (0 to 0)	0 (0 to 0)	-19.8 (-31.1 to -13.6)	-39.4 (-47.9 to -34.7)	--	--
	Male	34174 (18801 to 54046)	104.3 (57.5 to 164.5)	--	--	25506 (12677 to 43089)	59.7 (29.5 to 100.8)	0 (0 to 0)	0 (0 to 0)	-25.4 (-37.9 to -17.9)	-42.8 (-52.8 to -37)	--	--
Other mental disorders	Both	39261 (25100 to 60030)	100.6 (64.6 to 152.9)	--	--	93040 (59193 to 142670)	100.6 (64.5 to 153.4)	0 (0 to 0)	0 (0 to 0)	137 (125.7 to 149.1)	-0.1 (-1.6 to 1.5)	--	--
	Female	15501 (9833 to 23562)	82.5 (53.3 to 125.1)	--	--	37656 (23988 to 57792)	82.6 (53 to 125.5)	0 (0 to 0)	0 (0 to 0)	142.9 (130.5 to 156.9)	0.1 (-2.3 to 2.6)	--	--
	Male	23761 (15284 to 36017)	118 (76.3 to 178.7)	--	--	55384 (35451 to 84130)	118.2 (76.2 to 179.5)	0 (0 to 0)	0 (0 to 0)	133.1 (121.5 to 145)	0.2 (-1.7 to 2.3)	--	--
Substance use disorders	Both	164053 (131947 to 201960)	333 (270.2 to 403.1)	1208 (1086 to 1347)	3 (2.7 to 3.3)	381901 (311284 to 458741)	399.9 (324.3 to 481.3)	3168 (2937 to 3381)	3.4 (3.2 to 3.6)	132.8 (116.5 to 149.4)	20.1 (12.6 to 27.6)	162.3 (128.4 to 199.9)	13.2 (-0.3 to 28.6)
	Female	51673 (40630 to 65061)	211.6 (169.2 to 261.6)	347 (304 to 400)	1.8 (1.6 to 2.1)	96090 (74939 to 119144)	207.4 (161.2 to 255.5)	604 (545 to 673)	1.4 (1.3 to 1.5)	86 (70.4 to 102.6)	-2 (-9.8 to 4.9)	74.2 (44.2 to 109.4)	-23.2 (-34.9 to -9)
	Male	112380 (89720 to 137302)	450.3 (368 to 541.2)	861 (753 to 984)	4.2 (3.7 to 4.7)	285811 (235316 to 339962)	586.6 (481.2 to 700.2)	2565 (2316 to 2766)	5.4 (4.9 to 5.8)	154.3 (133.2 to 178.2)	30.2 (20.4 to 41)	197.8 (153.5 to 252.1)	29.1 (9.8 to 50.4)
Alcohol use disorders	Both	25115 (18742 to 32734)	55.5 (42 to 71.2)	168 (133 to 194)	0.5 (0.4 to 0.5)	40064 (29908 to 52926)	41.8 (31.4 to 54.9)	250 (225 to 291)	0.3 (0.3 to 0.3)	59.5 (47.4 to 76.6)	-24.6 (-30.5 to -17.2)	49 (25 to 107.7)	-39.7 (-49.2 to -17.8)

Cause	Sex	1990				2019				1990-2019 percent change (%)			
		DALYs		Death		DALYs		Death		DALYs		Death	
		Number	Rate	Number	Rate	Number	Rate	Number	Rate	Number	Rate	Number	Rate
	Female	6682 (4890 to 8732)	28.6 (21.3 to 37.2)	34 (30 to 38)	0.2 (0.2 to 0.2)	11567 (8133 to 15821)	24.9 (17.5 to 33.9)	39 (36 to 42)	0.1 (0.1 to 0.1)	73.1 (58.1 to 88.1)	-13 (-19.7 to -7.6)	14.9 (0.9 to 31.4)	-48.7 (-54.4 to -40.5)
	Male	18433 (13640 to 23861)	81.2 (61.2 to 104.3)	134 (100 to 159)	0.7 (0.6 to 0.9)	28498 (21667 to 37309)	58.3 (44.5 to 75.7)	211 (186 to 249)	0.5 (0.4 to 0.6)	54.6 (41.1 to 75.5)	-28.2 (-34.6 to -18.1)	57.6 (27.4 to 135.5)	-36.5 (-48.4 to -7.9)
Drug use disorders	Both	138938 (110015 to 173534)	277.5 (224.3 to 339.2)	1040 (932 to 1171)	2.5 (2.3 to 2.8)	341837 (278547 to 411189)	358.1 (291.5 to 431.8)	2918 (2682 to 3121)	3.1 (2.9 to 3.3)	146 (126.8 to 164.7)	29.1 (20.8 to 37.7)	180.6 (144.1 to 220.1)	23.1 (7.8 to 39.8)
	Female	44991 (34882 to 57139)	183 (145.1 to 229.3)	313 (271 to 365)	1.6 (1.4 to 1.9)	84523 (65434 to 105956)	182.5 (141.3 to 227.4)	565 (507 to 633)	1.3 (1.2 to 1.4)	87.9 (70.7 to 106.9)	-0.3 (-8.9 to 7.5)	80.6 (47.8 to 119.3)	-20.2 (-32.9 to -4.9)
	Male	93947 (74598 to 116016)	369.2 (299.5 to 450.5)	727 (637 to 842)	3.4 (3 to 4)	257313 (211710 to 307654)	528.2 (433.3 to 631.3)	2353 (2117 to 2542)	4.9 (4.4 to 5.3)	173.9 (149.5 to 200.2)	43.1 (31.6 to 54.8)	223.6 (172.8 to 282)	43.3 (21.3 to 66.5)
Diabetes and kidney diseases	Both	381118 (335423 to 432432)	1192.1 (1052.4 to 1343.4)	8093 (7433 to 8798)	37.9 (34.4 to 42.8)	1124710 (952403 to 1315159)	1505.5 (1284.3 to 1747.9)	27504 (24668 to 29237)	42.8 (38.3 to 45.6)	195.1 (167.1 to 222.5)	26.3 (15.8 to 35.3)	239.8 (197.7 to 276.3)	13 (-4.5 to 23.8)
	Female	183972 (159174 to 210253)	1190.4 (1043.7 to 1358.9)	3913 (3512 to 4457)	37.8 (33.4 to 46.5)	568738 (474651 to 671149)	1536.1 (1290.4 to 1804.8)	14019 (11656 to 15181)	44.6 (37.2 to 48.5)	209.1 (173.2 to 245.7)	29 (13.4 to 40.5)	258.3 (185.8 to 309.1)	18.1 (-9.4 to 34.6)
	Male	197146 (171777 to 222610)	1190 (1056.1 to 1341.3)	4180 (3801 to 4596)	37.8 (33.7 to 42.4)	555972 (472872 to 648319)	1475.4 (1265.6 to 1712.2)	13485 (12278 to 14567)	41.1 (37.2 to 44.5)	182 (154.2 to 213)	24 (13.7 to 34.5)	222.6 (185.5 to 262.6)	8.6 (-5.7 to 23)
Diabetes mellitus	Both	160376 (131623 to 194501)	565.9 (466.3 to 681.8)	2765 (2454 to 3140)	13.6 (11.8 to 15.6)	760543 (613584 to 932943)	1009.8 (821.6 to 1231.8)	14738 (12479 to 15876)	22.7 (19.1 to 24.5)	374.2 (331.8 to 405)	78.4 (60.6 to 91.4)	433.1 (326.6 to 519.4)	67 (33.5 to 97.5)
	Female	78652 (64396 to 94967)	583.3 (482.5 to 699.8)	1473 (1210 to 1763)	15 (11.9 to 18.1)	393388 (313632 to 484473)	1052.3 (845.5 to 1286.4)	7922 (5502 to 8793)	24.9 (17.8 to 27.8)	400.2 (333.9 to 447.9)	80.4 (52.5 to 102.6)	438 (243.5 to 599.4)	66.2 (6.3 to 125.1)
	Male	81724 (66086 to 100417)	547.4 (442.8 to 667.4)	1292 (1111 to 1518)	12 (10.2 to 14.3)	367154 (296416 to 447859)	967.5 (785.9 to 1176.5)	6816 (6162 to 7488)	20.6 (18.5 to 22.7)	349.3 (308.4 to 386.1)	76.8 (60 to 92)	427.5 (333.5 to 535.5)	71 (38.8 to 105.6)
Chronic kidney disease	Both	217425 (190844 to 243775)	620.3 (569.1 to 676.9)	5268 (4815 to 5771)	24.1 (21.8 to 28.8)	357020 (324206 to 390695)	486.7 (443.9 to 530.5)	12546 (11381 to 13406)	19.8 (17.8 to 21.2)	64.2 (46.2 to 84.1)	-21.5 (-28.8 to -15.4)	138.1 (109.3 to 159.8)	-18 (-35.4 to -10.8)
	Female	103873 (89373 to 118360)	602.1 (537.9 to 694.9)	2415 (2136 to 2963)	22.6 (19.6 to 31.5)	172300 (153997 to 189928)	475.6 (427.2 to 522.4)	5997 (5419 to 6440)	19.4 (17.3 to 20.9)	65.9 (45.5 to 88.3)	-21 (-33.2 to -13.5)	148.3 (91.7 to 179)	-14.3 (-42.8 to -3.3)
	Male	113552 (95514 to 127686)	636 (564.4 to 696)	2853 (2502 to 3125)	25.6 (22.5 to 28.4)	184720 (168372 to 202851)	498 (455.2 to 545.9)	6549 (5894 to 7135)	20.2 (18 to 22)	62.7 (42.7 to 91.5)	-21.7 (-28.6 to -11.4)	129.5 (105.8 to 166.8)	-21.2 (-31.2 to -9)
Acute glomerulonephritis	Both	3317 (2243 to 5255)	5.8 (4.4 to 7.9)	60 (46 to 82)	0.2 (0.1 to 0.2)	7148 (1527 to 8840)	9.1 (2.2 to 11.2)	220 (44 to 268)	0.3 (0.1 to 0.4)	115.5 (-69.8 to 279.3)	54.9 (-69.3 to 137.3)	267 (-40.6 to 461.8)	80.5 (-59.2 to 153.8)
	Female	1447 (839 to 2135)	5 (3.2 to 6.9)	25 (16 to 35)	0.1 (0.1 to 0.2)	3050 (683 to 3845)	8.2 (1.9 to 10.2)	100 (22 to 125)	0.3 (0.1 to 0.4)	110.7 (-54.9 to 322.3)	62.3 (-55.4 to 201.6)	296.2 (-12.5 to 621.8)	107.7 (-40.2 to 276.1)
	Male	1870 (1100 to 3538)	6.6 (4.4 to 9.8)	35 (23 to 53)	0.2 (0.1 to 0.3)	4098 (576 to 5613)	9.9 (1.6 to 13.7)	120 (16 to 167)	0.3 (0 to 0.5)	119.2 (-82 to 403.5)	50.3 (-81.5 to 211.1)	245.7 (-67.4 to 619.9)	61.3 (-76.6 to 202.8)
Skin and subcutaneous diseases	Both	249791 (166552 to 364085)	415.4 (281.7 to 608.6)	79 (59 to 98)	0.5 (0.3 to 0.7)	343632 (229918 to 509178)	423.3 (283.5 to 627.8)	272 (158 to 314)	0.5 (0.3 to 0.5)	37.6 (32.9 to 42)	1.9 (-0.3 to 3.8)	245.8 (112.9 to 388.5)	-9.2 (-39.7 to 39)

Cause	Sex	1990				2019				1990-2019 percent change (%)			
		DALYs		Death		DALYs		Death		DALYs		Death	
		Number	Rate	Number	Rate	Number	Rate	Number	Rate	Number	Rate	Number	Rate
	Female	130043 (86463 to 187879)	441 (298.9 to 645.3)	45 (31 to 63)	0.6 (0.4 to 0.9)	178820 (118811 to 265279)	446.9 (298.7 to 662.9)	137 (65 to 164)	0.5 (0.2 to 0.6)	37.5 (32.5 to 42.4)	1.4 (-1.1 to 3.3)	203.3 (60.4 to 384.4)	-20.2 (-56.9 to 43.5)
	Male	119749 (79693 to 176179)	390.6 (263.1 to 573.1)	34 (24 to 44)	0.4 (0.3 to 0.5)	164812 (109447 to 243895)	400.6 (267.5 to 593.9)	135 (78 to 157)	0.4 (0.3 to 0.5)	37.6 (33.1 to 42)	2.6 (0.1 to 4.6)	302.7 (157.9 to 466.3)	17.6 (-27.9 to 73.9)
Dermatitis	Both	57387 (34013 to 90641)	93.4 (56.5 to 144)	--	--	78743 (47930 to 119963)	94 (57.2 to 145.2)	--	--	37.2 (28.1 to 48.4)	0.6 (-1.7 to 2.8)	--	--
	Female	31225 (18384 to 49372)	103.7 (62.1 to 159.3)	--	--	43055 (25869 to 65803)	104.4 (62.9 to 160.7)	--	--	37.9 (28.6 to 48.7)	0.7 (-2.5 to 3.9)	--	--
	Male	26162 (15386 to 40967)	83.6 (50.6 to 128.3)	--	--	35688 (21839 to 54504)	83.9 (51 to 128.9)	--	--	36.4 (27 to 48.4)	0.4 (-2.6 to 3.6)	--	--
Psoriasis	Both	22029 (15466 to 29216)	46.6 (33.1 to 61.7)	--	--	29643 (21186 to 39323)	33.5 (23.9 to 44.5)	--	--	34.6 (29.9 to 39.5)	-28 (-30.1 to -26)	--	--
	Female	11320 (7928 to 15030)	48.3 (34.2 to 63.7)	--	--	15120 (10838 to 20100)	34.7 (25 to 46.1)	--	--	33.6 (27.4 to 39.9)	-28 (-30.7 to -25.1)	--	--
	Male	10709 (7510 to 14185)	44.9 (31.7 to 59.6)	--	--	14523 (10305 to 19322)	32.3 (22.9 to 43)	--	--	35.6 (29.3 to 42)	-28 (-31 to -25.2)	--	--
Bacterial skin diseases	Both	4036 (2792 to 6317)	8.9 (6.5 to 12.6)	50 (35 to 65)	0.3 (0.2 to 0.4)	6329 (4589 to 9337)	8.1 (5.9 to 11.7)	133 (106 to 183)	0.2 (0.2 to 0.3)	56.8 (36.4 to 79.5)	-8.9 (-22.1 to 10.4)	165 (101.2 to 290.2)	-22.8 (-44.3 to 27.8)
	Female	1867 (1287 to 2941)	8.8 (6.2 to 12.6)	27 (18 to 40)	0.3 (0.2 to 0.5)	2759 (1873 to 4224)	7.2 (5 to 11)	60 (42 to 105)	0.2 (0.1 to 0.4)	47.8 (29.3 to 81.3)	-17.9 (-36.5 to 9.1)	126.5 (40.1 to 325.9)	-35.1 (-63 to 37.1)
	Male	2169 (1472 to 3346)	8.8 (6.3 to 12.7)	23 (17 to 34)	0.2 (0.1 to 0.4)	3570 (2497 to 5143)	8.9 (6.4 to 12.6)	72 (52 to 84)	0.2 (0.2 to 0.3)	64.6 (35.5 to 96.7)	1.5 (-20.3 to 22.8)	209 (134.1 to 349.2)	-1 (-27.6 to 62.9)
Scabies	Both	15862 (8638 to 25580)	25.2 (13.8 to 40.3)	--	--	20665 (11418 to 33415)	25.1 (13.8 to 40.3)	--	--	30.3 (22.9 to 39.4)	-0.4 (-1.5 to 0.8)	--	--
	Female	7807 (4209 to 12574)	25.2 (13.8 to 40.5)	--	--	10166 (5587 to 16408)	25.1 (13.8 to 40.4)	--	--	30.2 (22.4 to 39.5)	-0.3 (-2 to 1.4)	--	--
	Male	8055 (4429 to 13005)	25.1 (13.8 to 40.4)	--	--	10499 (5792 to 16969)	25 (13.8 to 40.2)	--	--	30.3 (23.2 to 39.4)	-0.4 (-2 to 1.3)	--	--
Fungal skin diseases	Both	7914 (3157 to 16935)	18.9 (7.6 to 40.2)	--	--	14792 (5948 to 31898)	18.5 (7.5 to 39.6)	--	--	86.9 (75.3 to 97.9)	-1.9 (-2.8 to -1.2)	--	--
	Female	3569 (1434 to 7600)	17.6 (7.1 to 38.2)	--	--	6837 (2770 to 14800)	17.3 (6.9 to 37.7)	--	--	91.5 (80.2 to 103.2)	-1.9 (-2.9 to -1)	--	--
	Male	4345 (1736 to 9343)	20.1 (8.1 to 42.9)	--	--	7955 (3201 to 17123)	19.7 (7.9 to 42.2)	--	--	83.1 (72.1 to 94.6)	-2 (-3 to -1.2)	--	--
Viral skin diseases	Both	39178 (24839 to 58907)	51.2 (32.6 to 76.7)	--	--	39978 (25539 to 59680)	51.4 (32.8 to 76.8)	--	--	2 (0.2 to 4)	0.3 (-0.8 to 1.4)	--	--
	Female	19009 (12036 to 28985)	50.8 (32.4 to 76.9)	--	--	19429 (12413 to 29162)	51 (32.5 to 76.4)	--	--	2.2 (0 to 4.8)	0.3 (-1.2 to 1.7)	--	--
	Male	20169 (12699 to 30020)	51.6 (33 to 76.8)	--	--	20549 (13115 to 30646)	51.8 (33 to 77.4)	--	--	1.9 (-0.4 to 4.2)	0.3 (-1.3 to 1.8)	--	--
Acne vulgaris	Both	37002 (22450 to 58886)	51.8 (31.5 to 82.1)	--	--	52254 (31584 to 83081)	70.4 (42.5 to 112.1)	--	--	41.2 (36.9 to 45.6)	36 (33 to 39.2)	--	--
	Female	17522 (10620 to 27748)	50.3 (30.6 to 79.7)	--	--	25017 (15225 to 39687)	68.3 (41.5 to 109.2)	--	--	42.8 (37.9 to 47.9)	35.9 (32 to 39.8)	--	--
	Male	19480 (11809 to 30897)	53.2 (32.2 to 84.1)	--	--	27236 (16353 to 43279)	72.3 (43.3 to 115.3)	--	--	39.8 (34.3 to 45.5)	36 (31.6 to 41.1)	--	--

Cause	Sex	1990				2019				1990-2019 percent change (%)			
		DALYs		Death		DALYs		Death		DALYs		Death	
		Number	Rate	Number	Rate	Number	Rate	Number	Rate	Number	Rate	Number	Rate
Alopecia areata	Both	3239 (2027 to 4841)	6.3 (4 to 9.4)	--	--	5867 (3705 to 8685)	6.4 (4 to 9.4)	--	--	81.1 (74.9 to 87)	0.4 (-2.3 to 3.1)	--	--
	Female	2073 (1297 to 3086)	8.3 (5.2 to 12.3)	--	--	3778 (2387 to 5574)	8.3 (5.2 to 12.3)	--	--	82.2 (75.1 to 89.6)	0.2 (-3.1 to 3.7)	--	--
	Male	1166 (730 to 1750)	4.5 (2.8 to 6.7)	--	--	2089 (1324 to 3120)	4.5 (2.8 to 6.7)	--	--	79.1 (69.1 to 89.5)	0.2 (-4.8 to 4.8)	--	--
Pruritus	Both	4826 (2269 to 8647)	10 (4.8 to 18)	--	--	9017 (4302 to 16356)	10.6 (5.1 to 19.1)	--	--	86.9 (74.6 to 99.7)	5.3 (4 to 6.5)	--	--
	Female	2578 (1215 to 4607)	11 (5.2 to 19.7)	--	--	4865 (2327 to 8818)	11.6 (5.5 to 20.8)	--	--	88.7 (76.3 to 101.8)	5 (3.1 to 6.9)	--	--
	Male	2247 (1055 to 4015)	9.1 (4.4 to 16.4)	--	--	4151 (1979 to 7526)	9.6 (4.6 to 17.4)	--	--	84.7 (73.4 to 98.1)	5.4 (3.3 to 7.3)	--	--
Urticaria	Both	38969 (25230 to 57169)	58.1 (38 to 84)	--	--	47757 (31139 to 68212)	58.2 (38.3 to 84.1)	--	--	22.6 (14.7 to 31.2)	0.3 (-1.2 to 1.8)	--	--
	Female	23544 (15375 to 34508)	70.9 (46.6 to 102)	--	--	28445 (18750 to 40497)	71.1 (46.9 to 102)	--	--	20.8 (13.1 to 29.8)	0.3 (-1.6 to 2.2)	--	--
	Male	15425 (9969 to 22732)	45.8 (30 to 66.3)	--	--	19312 (12452 to 27719)	45.9 (29.9 to 66.7)	--	--	25.2 (16.7 to 35.4)	0.2 (-2.1 to 2.6)	--	--
Decubitus ulcer	Both	505 (261 to 697)	2.7 (1.3 to 3.9)	22 (9 to 35)	0.2 (0.1 to 0.3)	2050 (758 to 2630)	3.2 (1.2 to 4.1)	119 (31 to 157)	0.2 (0.1 to 0.3)	306.3 (125.9 to 433.1)	19.9 (-31.7 to 72.5)	431.5 (120.2 to 748.5)	10.9 (-51.2 to 94.2)
	Female	261 (91 to 416)	3.1 (0.9 to 5.2)	15 (3 to 26)	0.2 (0 to 0.4)	1005 (322 to 1395)	3.3 (1.1 to 4.6)	64 (12 to 92)	0.2 (0 to 0.3)	285 (95.8 to 465.8)	5.9 (-44 to 69.7)	333.9 (53 to 731.2)	-2.2 (-63 to 104.3)
	Male	243 (135 to 332)	2.1 (1.3 to 2.8)	8 (4 to 11)	0.1 (0.1 to 0.2)	1045 (381 to 1299)	3.1 (1.2 to 3.9)	55 (13 to 70)	0.2 (0 to 0.2)	329.1 (150.1 to 482.1)	49.4 (-25.1 to 101.6)	619.8 (195 to 967)	52.9 (-40 to 131.9)
Other skin and subcutaneous diseases	Both	18846 (9001 to 34326)	42.4 (20.4 to 76.6)	6 (4 to 10)	0 (0 to 0.1)	36538 (17561 to 66123)	44.1 (21.2 to 79.6)	21 (13 to 36)	0 (0 to 0.1)	93.9 (90.8 to 96.9)	4 (3 to 4.8)	229.1 (119.4 to 400.9)	-13.7 (-46.1 to 38.4)
	Female	9266 (4435 to 16893)	43 (20.8 to 77.5)	4 (2 to 7)	0 (0 to 0.1)	18343 (8828 to 33290)	44.7 (21.8 to 80.9)	12 (6 to 27)	0 (0 to 0.1)	98 (94.4 to 102.2)	4 (2.5 to 5.4)	238 (75.6 to 540)	-16.4 (-59.6 to 69.1)
	Male	9579 (4566 to 17433)	41.8 (20.1 to 75.7)	3 (2 to 4)	0 (0 to 0)	18194 (8701 to 32833)	43.5 (20.9 to 78.5)	8 (6 to 11)	0 (0 to 0)	89.9 (86.4 to 93.3)	4.1 (2.9 to 5.3)	216.9 (105.3 to 382.5)	6.5 (-27.7 to 70.7)
Sense organ diseases	Both	319977 (228506 to 431049)	1030.2 (741.1 to 1377.4)	--	--	599733 (422462 to 814940)	811 (574.4 to 1096.1)	--	--	87.4 (78.6 to 96.2)	-21.3 (-23.8 to -18.3)	--	--
	Female	154722 (110747 to 207763)	1031.8 (742.1 to 1373.1)	--	--	298360 (211087 to 403674)	812.7 (579.4 to 1093.7)	--	--	92.8 (83.7 to 101.9)	-21.2 (-23.8 to -18.4)	--	--
	Male	165255 (117435 to 223359)	1027.6 (735 to 1377.9)	--	--	301373 (212521 to 411330)	809.4 (570.1 to 1099.1)	--	--	82.4 (73.2 to 91.3)	-21.2 (-24 to -18.2)	--	--
Blindness and vision loss	Both	148263 (105176 to 200835)	500.5 (357.7 to 670)	--	--	270459 (191734 to 365108)	376.1 (267.1 to 505.8)	--	--	82.4 (77.1 to 88.2)	-24.9 (-27.2 to -22.2)	--	--
	Female	75511 (53849 to 101786)	526.9 (377.2 to 702.2)	--	--	142596 (101091 to 192515)	397.7 (282.7 to 535.5)	--	--	88.8 (83.4 to 94.6)	-24.5 (-26.9 to -21.9)	--	--
	Male	72752 (51332 to 99049)	475.2 (339.1 to 636.5)	--	--	127863 (90434 to 173325)	354.3 (251.3 to 477.1)	--	--	75.8 (70.4 to 81.7)	-25.4 (-27.8 to -22.7)	--	--
Age-related and other hearing loss	Both	155504 (107706 to 215571)	488.3 (348.4 to 679.6)	--	--	294348 (204654 to 413665)	391.6 (274.4 to 549.2)	--	--	89.3 (71.7 to 106.2)	-19.8 (-23.1 to -16.4)	--	--

Cause	Sex	1990				2019				1990-2019 percent change (%)			
		DALYs		Death		DALYs		Death		DALYs		Death	
		Number	Rate	Number	Rate	Number	Rate	Number	Rate	Number	Rate	Number	Rate
	Female	70705 (48759 to 97262)	459.8 (328 to 635.7)	--	--	137010 (95607 to 192292)	368.2 (258.8 to 513.3)	--	--	93.8 (75.5 to 111.7)	-19.9 (-23.2 to -16.3)	--	--
	Male	84800 (58784 to 118911)	514.4 (367 to 714.6)	--	--	157338 (109121 to 222142)	415.3 (289.7 to 580.6)	--	--	85.5 (68.1 to 102)	-19.3 (-22.8 to -15.6)	--	--
Other sense organ diseases	Both	16210 (9877 to 24697)	41.5 (24.7 to 64.4)	--	--	34926 (20647 to 54388)	43.3 (25.7 to 67)	--	--	115.5 (100.4 to 129.5)	4.4 (2.9 to 5.9)	--	--
	Female	8506 (5199 to 12983)	45 (27 to 70)	--	--	18754 (11050 to 29023)	46.8 (27.7 to 72.4)	--	--	120.5 (104.2 to 136)	3.9 (1.8 to 6.2)	--	--
	Male	7704 (4719 to 11735)	38 (22.7 to 58.9)	--	--	16172 (9583 to 24896)	39.8 (23.7 to 61.7)	--	--	109.9 (94.7 to 123.4)	4.6 (2.5 to 6.8)	--	--
Musculoskeletal disorders	Both	726735 (517710 to 971163)	1930.4 (1377.1 to 2553)	158 (130 to 205)	0.6 (0.5 to 0.8)	1771348 (1267185 to 2346806)	2007.1 (1439.9 to 2654.6)	632 (428 to 715)	0.9 (0.6 to 1)	143.7 (134.9 to 153.1)	4 (2 to 6.1)	300.8 (171.9 to 417.1)	49.1 (3.6 to 96.7)
	Female	384910 (275472 to 513469)	2112.2 (1513.8 to 2791.8)	103 (78 to 147)	0.8 (0.5 to 1.1)	975343 (702766 to 1292630)	2233.1 (1611.1 to 2951.6)	433 (239 to 507)	1.2 (0.6 to 1.4)	153.4 (144.3 to 163)	5.7 (3.8 to 7.8)	321.2 (140.5 to 497.5)	59.1 (-13.7 to 139.1)
	Male	341825 (240144 to 457848)	1758.3 (1249.2 to 2335.7)	55 (47 to 70)	0.4 (0.4 to 0.5)	796006 (565257 to 1059951)	1784.5 (1271.6 to 2366)	199 (145 to 219)	0.6 (0.4 to 0.6)	132.9 (123.8 to 143.1)	1.5 (-1 to 4)	262.6 (103.9 to 340.6)	35.3 (-16.1 to 67.3)
Rheumatoid arthritis	Both	5863 (4191 to 7734)	15.8 (11.4 to 20.6)	25 (14 to 37)	0.1 (0.1 to 0.2)	15366 (10877 to 20209)	17.3 (12.4 to 22.7)	83 (36 to 107)	0.1 (0.1 to 0.2)	162.1 (135.3 to 185.5)	9.8 (-3 to 20.5)	238.6 (45.4 to 458)	9.5 (-54.6 to 88.6)
	Female	4448 (3152 to 5869)	24.4 (17.3 to 32.3)	19 (9 to 30)	0.2 (0.1 to 0.3)	11709 (8311 to 15536)	26.4 (18.7 to 34.7)	61 (15 to 85)	0.2 (0 to 0.3)	163.2 (128.7 to 193.4)	8.4 (-7.9 to 22)	225.4 (-13.1 to 553.6)	5 (-73.1 to 123.1)
	Male	1415 (992 to 1874)	7.6 (5.4 to 10)	6 (4 to 8)	0 (0 to 0.1)	3657 (2627 to 4833)	8.3 (6 to 10.9)	22 (18 to 27)	0.1 (0.1 to 0.1)	158.4 (140.4 to 178.7)	10 (2.6 to 19.1)	281.3 (176.4 to 484.7)	34.1 (-0.5 to 97)
Osteoarthritis	Both	49531 (25023 to 97062)	181 (91.8 to 355)	--	--	149354 (75490 to 293270)	195.3 (98.7 to 381)	--	--	201.5 (197.8 to 206)	7.9 (6.9 to 9.1)	--	--
	Female	26852 (13574 to 52638)	206.5 (104.9 to 405.2)	--	--	85439 (43376 to 167896)	223.3 (113.5 to 439.6)	--	--	218.2 (213.3 to 223.8)	8.1 (6.7 to 9.8)	--	--
	Male	22679 (11378 to 44498)	157 (78.9 to 310.7)	--	--	63915 (31778 to 126546)	167.1 (83.7 to 332)	--	--	181.8 (176.9 to 187.2)	6.5 (5 to 7.9)	--	--
Low back pain	Both	418229 (292449 to 558428)	1051.4 (738.5 to 1404.5)	--	--	837779 (583888 to 1128834)	951.9 (668.9 to 1275.2)	--	--	100.3 (90.9 to 108.9)	-9.5 (-10.7 to -8.2)	--	--
	Female	195476 (137740 to 262881)	1007.7 (712.7 to 1340.1)	--	--	408084 (285313 to 547130)	944.1 (665 to 1258.7)	--	--	108.8 (98.1 to 118.8)	-6.3 (-7.7 to -4.7)	--	--
	Male	222753 (155680 to 298277)	1091.4 (762.3 to 1464.1)	--	--	429694 (299265 to 579099)	960.1 (674.7 to 1289.7)	--	--	92.9 (83.8 to 101.3)	-12 (-13.7 to -10.4)	--	--

Cause	Sex	1990				2019				1990-2019 percent change (%)			
		DALYs		Death		DALYs		Death		DALYs		Death	
		Number	Rate	Number	Rate	Number	Rate	Number	Rate	Number	Rate	Number	Rate
Neck pain	Both	154692 (100656 to 224784)	418.8 (277.1 to 604.8)	--	--	385175 (253143 to 558607)	423.5 (280.3 to 609.8)	--	--	149 (134.9 to 160.2)	1.1 (0.4 to 1.8)	--	--
	Female	92581 (60690 to 135006)	518 (345.5 to 751)	--	--	235692 (156315 to 341588)	521.7 (347.5 to 754.2)	--	--	154.6 (139.9 to 166.1)	0.7 (0 to 1.5)	--	--
	Male	62111 (40091 to 90325)	325.5 (211.8 to 472.8)	--	--	149483 (96885 to 219633)	326.9 (212.1 to 474.9)	--	--	140.7 (127 to 152.8)	0.4 (-0.5 to 1.4)	--	--
Gout	Both	4172 (2591 to 6028)	14.1 (8.7 to 20.4)	--	--	12230 (7692 to 17716)	15.2 (9.5 to 22)	--	--	193.2 (179.2 to 209.1)	7.5 (3.9 to 11.5)	--	--
	Female	862 (538 to 1247)	6.1 (3.9 to 8.8)	--	--	2766 (1715 to 4018)	6.9 (4.4 to 10)	--	--	220.8 (198.9 to 245.8)	12.5 (5.6 to 19.4)	--	--
	Male	3310 (2059 to 4797)	21.7 (13.4 to 31.3)	--	--	9464 (5926 to 13664)	23.3 (14.6 to 34)	--	--	186 (170.7 to 203.7)	7.6 (3.4 to 12.3)	--	--
Other musculoskeletal disorders	Both	94248 (62857 to 133654)	249.3 (164.8 to 357.1)	133 (112 to 176)	0.5 (0.4 to 0.6)	371445 (254898 to 516639)	403.9 (277.9 to 555.6)	549 (390 to 644)	0.8 (0.5 to 0.9)	294.1 (267.4 to 327.8)	62 (50.9 to 77.1)	312.3 (179.2 to 431.8)	58.4 (10.4 to 107.5)
	Female	64690 (44179 to 89908)	349.5 (237.3 to 485.8)	84 (65 to 125)	0.6 (0.4 to 0.8)	231653 (161353 to 316801)	510.6 (359 to 693.9)	372 (222 to 456)	1 (0.6 to 1.2)	258.1 (238.7 to 281.6)	46.1 (38 to 56.2)	342.6 (171 to 521.9)	75.2 (2.7 to 155.6)
	Male	29558 (17498 to 44737)	155.2 (92.5 to 235)	49 (42 to 65)	0.4 (0.3 to 0.4)	139792 (91991 to 198769)	298.8 (198.2 to 418.5)	177 (125 to 195)	0.5 (0.3 to 0.6)	372.9 (314.9 to 461.4)	92.5 (69.6 to 128)	260.4 (91.6 to 338.9)	35.4 (-20.4 to 66)
Other non-communicable diseases	Both	3080703 (2107034 to 4018213)	4228.2 (2996.6 to 5352.5)	28298 (17889 to 39394)	34.6 (22.8 to 47.4)	1683522 (1309767 to 2144098)	2054.5 (1618.9 to 2588.1)	7883 (6527 to 9689)	11.6 (9.6 to 14.3)	-45.4 (-62.1 to -15.8)	-51.4 (-64.3 to -30.8)	-72.1 (-81.6 to -49.7)	-66.6 (-77.7 to -42.2)
	Female	1562845 (1094819 to 2048977)	4674.2 (3376.8 to 5944.9)	12852 (8013 to 18118)	32.5 (21.3 to 44.9)	1098972 (831529 to 1433770)	2589.7 (1990.9 to 3356.2)	3643 (2991 to 4437)	11 (9 to 13.4)	-29.7 (-50.9 to 4.8)	-44.6 (-58.2 to -25.5)	-71.7 (-82 to -49)	-66.2 (-78.3 to -41.3)
	Male	1517858 (918475 to 2077776)	3796.6 (2420.2 to 5061.2)	15446 (8652 to 21549)	36.6 (21.4 to 50.5)	584550 (458877 to 740679)	1534 (1213.6 to 1931.7)	4240 (3379 to 5370)	12.1 (9.7 to 15.4)	-61.5 (-74.5 to -28.9)	-59.6 (-72.2 to -30.3)	-72.5 (-82.3 to -44.6)	-66.9 (-78.4 to -35)
Congenital birth defects	Both	2332694 (1421946 to 3241865)	2718.8 (1669.2 to 3766.6)	25811 (15497 to 36103)	30 (18.1 to 41.9)	517026 (409706 to 660853)	745.8 (586.3 to 961)	5192 (3991 to 6822)	7.6 (5.8 to 10)	-77.8 (-86.1 to -56.9)	-72.6 (-82.8 to -46.3)	-79.9 (-87.5 to -59.1)	-74.7 (-84.4 to -48.4)
	Female	1051533 (626163 to 1499621)	2506.1 (1499.9 to 3560)	11679 (6815 to 16840)	27.8 (16.3 to 39.9)	230357 (179219 to 295896)	680.8 (525.1 to 880.6)	2341 (1786 to 3080)	7 (5.3 to 9.3)	-78.1 (-86.7 to -56.4)	-72.8 (-83.5 to -46.2)	-80 (-88 to -58.6)	-74.8 (-85 to -48.3)
	Male	1281161 (683051 to 1805716)	2921.2 (1574.8 to 4110.3)	14132 (7304 to 20157)	32.2 (16.8 to 45.7)	286669 (226138 to 385128)	807.6 (632.7 to 1092.1)	2851 (2178 to 3916)	8.1 (6.2 to 11.3)	-77.6 (-85.9 to -48.5)	-72.4 (-82.6 to -36.5)	-79.8 (-87.5 to -50.7)	-74.7 (-84.3 to -38.5)
Urinary diseases and male infertility	Both	13236 (9952 to 17813)	33.6 (25.4 to 44.6)	120 (100 to 146)	0.6 (0.5 to 0.9)	29118 (21263 to 38239)	37.4 (27.3 to 48.3)	535 (285 to 617)	0.9 (0.5 to 1)	120 (91 to 140.4)	11.4 (-9.2 to 23.9)	344.5 (160 to 458)	42.3 (-21.2 to 90)
	Female	4857 (3839 to 6079)	22.4 (17.9 to 28.5)	69 (53 to 94)	0.7 (0.5 to 1.1)	9559 (6408 to 11701)	25.4 (16.6 to 30.6)	284 (151 to 341)	1 (0.5 to 1.2)	96.8 (58.5 to 127.5)	13.2 (-18 to 37.3)	310 (132.2 to 462)	36.1 (-27 to 103.3)

Cause	Sex	1990				2019				1990-2019 percent change (%)			
		DALYs		Death		DALYs		Death		DALYs		Death	
		Number	Rate	Number	Rate	Number	Rate	Number	Rate	Number	Rate	Number	Rate
	Male	8379 (5865 to 12112)	44.1 (30.5 to 63.2)	51 (41 to 64)	0.5 (0.4 to 0.7)	19559 (13580 to 27465)	49.5 (34.5 to 68.4)	251 (123 to 294)	0.8 (0.4 to 1)	133.4 (104.4 to 156.4)	12.5 (-6.4 to 22.7)	391.3 (144.3 to 524.4)	56.4 (-24.9 to 109.8)
Gynecological diseases	Female	237140 (162837 to 330426)	1094.9 (747.8 to 1521.6)	4 (2 to 7)	0 (0 to 0.1)	512250 (348497 to 709893)	1045.1 (715.7 to 1450.1)	9 (2 to 12)	0 (0 to 0)	116 (105.2 to 126)	-4.6 (-6.4 to -2.9)	103.8 (-36.7 to 367.7)	-19.8 (-70.9 to 77.2)
Hemoglobinopathies and hemolytic anemias	Both	119881 (90364 to 157657)	181.9 (137 to 241.2)	626 (513 to 763)	1 (0.9 to 1.1)	68561 (50195 to 94497)	84.2 (62.7 to 115.3)	404 (379 to 430)	0.5 (0.5 to 0.6)	-42.8 (-52.2 to -32.3)	-53.7 (-60.4 to -46)	-35.5 (-48.5 to -18.9)	-45.3 (-53.6 to -35.9)
	Female	70092 (50346 to 94440)	228.1 (162.9 to 311.3)	298 (224 to 389)	0.9 (0.8 to 1.1)	41345 (28696 to 58660)	100.8 (71.8 to 141.4)	186 (169 to 206)	0.5 (0.5 to 0.6)	-41 (-53.7 to -25.5)	-55.8 (-64 to -46)	-37.5 (-54.4 to -10.1)	-47.1 (-58.4 to -30.7)
	Male	49789 (38358 to 64169)	136.5 (105.7 to 176.3)	329 (261 to 395)	1 (0.9 to 1.1)	27216 (20797 to 36446)	68 (52 to 91.1)	218 (202 to 237)	0.6 (0.5 to 0.6)	-45.3 (-55.6 to -30.8)	-50.2 (-58.7 to -38.4)	-33.7 (-46.5 to -12.2)	-43.5 (-52.3 to -30.4)
Endocrine, metabolic, blood, and immune disorders	Both	180388 (135397 to 240083)	345.4 (253.7 to 456.3)	824 (650 to 1262)	2 (1.6 to 2.7)	284591 (205635 to 377450)	343.5 (251.7 to 456.2)	1618 (957 to 1887)	2.4 (1.4 to 2.8)	57.8 (19.7 to 80.4)	-0.5 (-18 to 9.2)	96.4 (-9.5 to 172.9)	21.7 (-30.6 to 63.7)
	Female	106675 (75860 to 145799)	427.3 (302.5 to 574.8)	387 (272 to 608)	2 (1.5 to 3.1)	174102 (123543 to 235257)	419.8 (301.1 to 563.2)	765 (396 to 896)	2.3 (1.2 to 2.7)	63.2 (26.1 to 84.8)	-1.7 (-18 to 7.5)	97.7 (-18.6 to 209.9)	13.6 (-39.1 to 76.8)
	Male	73713 (54315 to 101903)	267.5 (200.9 to 354.3)	437 (309 to 729)	1.8 (1.4 to 2.7)	110489 (79129 to 144461)	269.6 (192.5 to 348.2)	853 (452 to 1033)	2.4 (1.3 to 3)	49.9 (6.1 to 80.9)	0.8 (-21.2 to 16)	95.2 (-5.9 to 183.7)	32.5 (-21.4 to 82.8)
Oral disorders	Both	116664 (68709 to 183710)	317.6 (189.3 to 496.9)	--	--	260892 (154358 to 414961)	311.8 (185.9 to 488.1)	--	--	123.6 (115.7 to 131.4)	-1.8 (-3 to -0.8)	--	--
	Female	55804 (32684 to 88304)	307.9 (183.1 to 483.8)	--	--	126214 (74456 to 202426)	302 (179.7 to 477.3)	--	--	126.2 (117.5 to 135)	-1.9 (-3.2 to -0.8)	--	--
	Male	60859 (36091 to 95412)	327.1 (194.4 to 509.6)	--	--	134678 (79452 to 212502)	321.8 (189.6 to 502.3)	--	--	121.3 (113.8 to 128.8)	-1.6 (-2.9 to -0.5)	--	--
Sudden infant death syndrome	Both	80702 (16250 to 235112)	94.1 (19 to 274.1)	913 (184 to 2659)	1.1 (0.2 to 3.1)	11083 (6649 to 19636)	16.6 (10 to 29.5)	125 (75 to 222)	0.2 (0.1 to 0.3)	-86.3 (-94.5 to -29.5)	-82.3 (-92.9 to -9.3)	-86.3 (-94.5 to -29.5)	-82.3 (-92.9 to -9.3)
	Female	36744 (8232 to 113855)	87.6 (19.6 to 271.5)	416 (93 to 1288)	1 (0.2 to 3.1)	5144 (2714 to 9959)	15.9 (8.4 to 30.7)	58 (31 to 113)	0.2 (0.1 to 0.3)	-86 (-94.6 to -30.6)	-81.9 (-93 to -10.3)	-86 (-94.6 to -30.6)	-81.9 (-93 to -10.3)
	Male	43958 (6909 to 130781)	100.3 (15.8 to 298.3)	497 (78 to 1479)	1.1 (0.2 to 3.4)	5939 (3247 to 11162)	17.4 (9.5 to 32.6)	67 (37 to 126)	0.2 (0.1 to 0.4)	-86.5 (-94.7 to -23.8)	-82.7 (-93.2 to -2.4)	-86.5 (-94.7 to -23.9)	-82.7 (-93.2 to -2.4)
**Injuries**	Both	6515958 (5944606 to 7136159)	10629.6 (9791.9 to 11510.5)	95475 (87772 to 103390)	187.3 (173 to 201.2)	2558222 (2357921 to 2776557)	2961.7 (2737.1 to 3211.7)	41191 (38984 to 44240)	49.8 (47.1 to 53.6)	-60.7 (-64.6 to -56.6)	-72.1 (-74.6 to -69.6)	-56.9 (-61.1 to -51.8)	-73.4 (-75.8 to -70.4)
	Female	2310016 (2081576 to 2582828)	7302.1 (6672.8 to 8036.3)	32014 (29084 to 35402)	123.6 (113.9 to 134.1)	682266 (613856 to 756233)	1641.8 (1479.7 to 1816.6)	9964 (9288 to 10623)	25.8 (23.9 to 27.6)	-70.5 (-74 to -66.2)	-77.5 (-80 to -74.7)	-68.9 (-72.8 to -65)	-79.1 (-81.4 to -76.8)
	Male	4205942 (3854389 to 4584867)	13804 (12694 to 14936.3)	63461 (58144 to 68606)	248 (226.4 to 266.3)	1875956 (1735295 to 2028688)	4244.1 (3929.5 to 4588.8)	31226 (29410 to 34048)	73.3 (68.8 to 80.1)	-55.4 (-59.6 to -50.8)	-69.3 (-71.9 to -66.2)	-50.8 (-55.6 to -44.3)	-70.5 (-73.1 to -66.6)
Transport injuries	Both	2046626 (1749825 to 2358659)	3356.6 (2898.3 to 3734.6)	31501 (26937 to 35293)	61.9 (52.5 to 67.7)	1183076 (1051431 to 1347766)	1383.4 (1235.1 to 1581.7)	22425 (19477 to 26145)	27 (23.5 to 31.7)	-42.2 (-50.5 to -29.9)	-58.8 (-63.9 to -50.5)	-28.8 (-37.9 to -13.3)	-56.4 (-61.1 to -46.4)
	Female	639496 (521492 to 774492)	1966.5 (1664.3 to 2276.6)	8965 (7428 to 10559)	32.8 (28.2 to 36.6)	273534 (241062 to 297866)	661.6 (587.9 to 722.3)	5032 (4276 to 5418)	12.6 (10.8 to 13.5)	-57.2 (-65.5 to -47.4)	-66.4 (-71.9 to -60.3)	-43.9 (-53.5 to -33.3)	-61.6 (-66.6 to -56.2)

Cause	Sex	1990				2019				1990-2019 percent change (%)			
		DALYs		Death		DALYs		Death		DALYs		Death	
		Number	Rate	Number	Rate	Number	Rate	Number	Rate	Number	Rate	Number	Rate
	Male	1407130 (1200925 to 1583207)	4683.9 (3934.5 to 5173.9)	22536 (18807 to 25035)	89.8 (73.2 to 98.6)	909542 (804777 to 1061244)	2083.8 (1849.2 to 2435.8)	17393 (14982 to 20865)	41.1 (35.5 to 50)	-35.4 (-43.7 to -20.6)	-55.5 (-60.8 to -45.2)	-22.8 (-32.3 to -2.9)	-54.2 (-59.6 to -41.8)
Road injuries	Both	1993124 (1702319 to 2290934)	3262.5 (2815.5 to 3635.4)	30693 (26110 to 34458)	60.2 (51.2 to 65.9)	1112765 (978584 to 1267172)	1302.1 (1147.4 to 1488.3)	21122 (18110 to 24648)	25.4 (21.9 to 29.9)	-44.2 (-52.5 to -32.5)	-60.1 (-65.2 to -51.7)	-31.2 (-40.1 to -15.8)	-57.7 (-62.3 to -48.1)
	Female	627081 (509911 to 761209)	1926.1 (1625.3 to 2230.4)	8807 (7298 to 10369)	32.2 (27.7 to 36)	260756 (229218 to 284315)	630.8 (557.5 to 689.5)	4820 (4076 to 5190)	12 (10.3 to 13)	-58.4 (-66.6 to -48.8)	-67.3 (-72.7 to -61.3)	-45.3 (-54.7 to -35.1)	-62.6 (-67.4 to -57.3)
	Male	1366043 (1163748 to 1539097)	4538.7 (3813.6 to 5026.1)	21885 (18318 to 24351)	87 (71 to 95.7)	852009 (744737 to 988937)	1953.3 (1712.5 to 2284.3)	16302 (13849 to 19529)	38.5 (32.8 to 46.8)	-37.6 (-45.8 to -23.4)	-57 (-62.1 to -46.8)	-25.5 (-34.9 to -6.2)	-55.7 (-61 to -43.6)
Other transport injuries	Both	53502 (42642 to 66677)	94.1 (76.7 to 114.3)	809 (619 to 1000)	1.7 (1.3 to 2.1)	70311 (61526 to 86130)	81.3 (71.2 to 99.2)	1303 (1108 to 1644)	1.6 (1.3 to 2)	31.4 (4.9 to 63.6)	-13.6 (-29.2 to 5.5)	61.2 (27.8 to 103.4)	-8.1 (-27.2 to 15.5)
	Female	12415 (9534 to 16359)	40.4 (32.7 to 51.4)	158 (122 to 211)	0.6 (0.5 to 0.8)	12777 (11179 to 14583)	30.9 (27.1 to 35.2)	212 (179 to 242)	0.5 (0.5 to 0.6)	2.9 (-18.1 to 31.5)	-23.6 (-36.8 to -7.7)	34.3 (6.5 to 71.5)	-9.9 (-26.5 to 12.9)
	Male	41087 (31490 to 54335)	145.2 (113.4 to 183.5)	651 (479 to 842)	2.7 (2.1 to 3.5)	57534 (49032 to 72664)	130.4 (111.7 to 163)	1092 (890 to 1409)	2.6 (2.1 to 3.3)	40 (2.5 to 86.3)	-10.2 (-32.8 to 16.1)	67.7 (19.5 to 124.9)	-6.8 (-33.4 to 23.7)
Unintentional injuries	Both	3896308 (3561419 to 4265245)	6206 (5696 to 6761.2)	57250 (52557 to 62424)	112.3 (104 to 121.8)	862679 (741588 to 1011355)	1013.1 (873.1 to 1181)	11509 (10175 to 12680)	14.7 (13 to 16.4)	-77.9 (-80.9 to -74.4)	-83.7 (-85.8 to -81.3)	-79.9 (-82.2 to -76.8)	-86.9 (-88.5 to -85)
	Female	1493272 (1340690 to 1665766)	4664.1 (4237 to 5149.2)	21183 (19081 to 23421)	83.6 (75.9 to 91.6)	269425 (224015 to 326931)	663.2 (553.3 to 799.2)	3300 (2912 to 3708)	9.3 (8.2 to 10.5)	-82 (-85 to -78.1)	-85.8 (-88 to -83.1)	-84.4 (-86.7 to -81.5)	-88.8 (-90.4 to -86.8)
	Male	2403035 (2193726 to 2637409)	7673.8 (7036 to 8359.1)	36067 (33039 to 39428)	139.5 (129.1 to 151.4)	593255 (513125 to 687265)	1353.9 (1172.8 to 1562.7)	8210 (7103 to 9109)	20 (17.1 to 22.4)	-75.3 (-78.6 to -71.4)	-82.4 (-84.7 to -79.9)	-77.2 (-80.4 to -73.7)	-85.7 (-87.7 to -83.5)
Falls	Both	214772 (175484 to 263777)	438.5 (363.8 to 530.9)	2296 (1901 to 2756)	6.4 (5.4 to 7.4)	249029 (197963 to 311677)	296.6 (237.5 to 368)	2941 (2570 to 3655)	4.2 (3.6 to 5.2)	16 (-6.2 to 37.8)	-32.4 (-40.8 to -23)	28.1 (2.7 to 67.5)	-35 (-44.8 to -17)
	Female	65346 (44692 to 88274)	272.5 (212.7 to 336.4)	657 (473 to 861)	4.4 (3.4 to 5.4)	72126 (56087 to 93882)	185.1 (145.7 to 236.7)	848 (698 to 979)	2.8 (2.3 to 3.3)	10.4 (-20.6 to 59.9)	-32.1 (-43.7 to -19.2)	29.2 (-4.7 to 83.4)	-35.5 (-45.9 to -8.8)
	Male	149426 (115572 to 184654)	594.4 (479.4 to 723.9)	1639 (1263 to 2017)	8.3 (6.8 to 9.8)	176902 (140643 to 219161)	406.2 (325.2 to 501.9)	2092 (1792 to 2814)	5.5 (4.6 to 7.4)	18.4 (-5.4 to 61.4)	-31.7 (-41.5 to -15.2)	27.6 (0 to 96.1)	-33.4 (-44.2 to -10.6)
Drowning	Both	229730 (163940 to 290053)	286.7 (205.8 to 357)	2986 (2132 to 3742)	4 (2.9 to 5)	51442 (46206 to 58493)	63.6 (57.2 to 72.9)	866 (774 to 985)	1.1 (1 to 1.2)	-77.6 (-83 to -68.5)	-77.8 (-82.8 to -68.9)	-71 (-77.5 to -59.9)	-73.7 (-79.3 to -64.3)
	Female	71711 (53323 to 92632)	170.9 (130.5 to 216.7)	878 (662 to 1122)	2.2 (1.7 to 2.8)	8561 (7639 to 9718)	22.3 (19.7 to 25.4)	132 (121 to 147)	0.3 (0.3 to 0.4)	-88.1 (-91.3 to -83.1)	-87 (-90.4 to -81.8)	-85 (-88.8 to -79.2)	-84.4 (-88.1 to -79.1)
	Male	158019 (91008 to 208024)	397.9 (236.1 to 514.5)	2108 (1241 to 2736)	5.8 (3.5 to 7.4)	42881 (37413 to 49268)	103.3 (90.7 to 119.6)	734 (638 to 841)	1.8 (1.5 to 2)	-72.9 (-80 to -55.8)	-74 (-80.5 to -58.6)	-65.2 (-74 to -44.5)	-69.8 (-76.8 to -52.9)
Fire, heat, and hot substances	Both	263304 (138418 to 397143)	399.5 (230.6 to 573.8)	3704 (1962 to 5488)	6.9 (4.2 to 9.5)	91768 (76127 to 112754)	107.6 (89.9 to 133.9)	1763 (1454 to 2240)	2.2 (1.9 to 2.9)	-65.1 (-78.7 to -29.6)	-73.1 (-82.9 to -49.8)	-52.4 (-70.7 to -1.7)	-67.8 (-79 to -41.9)
	Female	116169 (29975 to 209034)	361.5 (123.8 to 610.9)	1607 (441 to 2835)	6.2 (2.7 to 9.6)	43367 (33993 to 58347)	105.5 (82.8 to 142.8)	811 (627 to 1211)	2.2 (1.7 to 3.3)	-62.7 (-81.8 to 50.4)	-70.8 (-85 to -10.5)	-49.6 (-75.7 to 101.4)	-65 (-81.6 to -12.6)
	Male	147136 (59050 to 198057)	435.5 (196.5 to 563.9)	2097 (884 to 2778)	7.6 (3.7 to 9.6)	48402 (37600 to 63167)	109.8 (86.2 to 147.6)	953 (731 to 1306)	2.3 (1.8 to 3.2)	-67.1 (-76.7 to 9.4)	-74.8 (-81.7 to -26.5)	-54.6 (-67.3 to 45.2)	-69.8 (-77.6 to -22)
Poisonings	Both	121403 (87090 to 151038)	169 (123.3 to 208)	1658 (1201 to 2056)	2.7 (2 to 3.4)	40388 (36730 to 44608)	46 (42.4 to 51.8)	778 (667 to 844)	0.9 (0.8 to 1)	-66.7 (-73.6 to -52.6)	-72.8 (-78.1 to -61.8)	-53.1 (-62.8 to -36.3)	-66.4 (-73.6 to -56.6)

Cause	Sex	1990				2019				1990-2019 percent change (%)			
		DALYs		Death		DALYs		Death		DALYs		Death	
		Number	Rate	Number	Rate	Number	Rate	Number	Rate	Number	Rate	Number	Rate
	Female	47774 (33315 to 62611)	127.1 (93.9 to 160.9)	617 (447 to 789)	1.9 (1.5 to 2.3)	11933 (10821 to 13350)	29 (26.1 to 32.5)	216 (199 to 240)	0.5 (0.5 to 0.6)	-75 (-81.6 to -58.8)	-77.2 (-82.9 to -63.8)	-64.9 (-73.5 to -46.3)	-71.3 (-77.2 to -57.9)
	Male	73629 (50401 to 99301)	209.2 (145.4 to 287.5)	1041 (721 to 1441)	3.5 (2.4 to 4.8)	28455 (23854 to 32057)	62.6 (54 to 71.4)	561 (440 to 623)	1.3 (1 to 1.4)	-61.4 (-72.1 to -42.7)	-70.1 (-78.4 to -57.1)	-46.1 (-61.1 to -24)	-63.8 (-74.5 to -51.2)
Exposure to mechanical forces	Both	183554 (145856 to 225867)	329.1 (266.4 to 402.2)	2327 (1827 to 2923)	4.7 (3.8 to 5.9)	185734 (136882 to 224769)	214.4 (155.1 to 261.1)	2468 (1671 to 2913)	2.9 (1.9 to 3.5)	1.2 (-23.8 to 36.3)	-34.9 (-52.2 to -14.6)	6.1 (-28.1 to 55.3)	-37.3 (-59.1 to -10.3)
	Female	46240 (26652 to 64010)	158.8 (106.3 to 207)	508 (264 to 724)	2 (1.3 to 2.7)	43566 (29599 to 54422)	105.5 (71.5 to 131.6)	482 (264 to 574)	1.2 (0.7 to 1.5)	-5.8 (-34.3 to 36.6)	-33.6 (-49.3 to -11.8)	-5 (-38.8 to 46.9)	-37.6 (-56.8 to -13.3)
	Male	137315 (102699 to 175212)	492.1 (389.2 to 622.9)	1819 (1336 to 2380)	7.2 (5.6 to 9.7)	142169 (103726 to 172787)	320.2 (229 to 392.1)	1986 (1332 to 2384)	4.6 (3 to 5.5)	3.5 (-24.6 to 49.8)	-34.9 (-54.7 to -9.1)	9.2 (-30.2 to 70)	-36.8 (-63.3 to -4.3)
Adverse effects of medical treatment	Both	78404 (49273 to 103141)	139.2 (87.4 to 189.9)	1348 (847 to 1833)	3.4 (2.2 to 4.8)	33338 (29038 to 39437)	40.9 (36.1 to 49.1)	879 (762 to 1088)	1.2 (1 to 1.5)	-57.5 (-68.8 to -32.6)	-70.6 (-79.3 to -53.5)	-34.8 (-56.4 to 4.6)	-64.5 (-77.6 to -42.9)
	Female	29546 (18864 to 37047)	103.3 (69.9 to 135.4)	487 (330 to 643)	2.5 (1.6 to 4.4)	13028 (10959 to 16145)	33 (28.1 to 41.6)	345 (286 to 444)	1 (0.8 to 1.3)	-55.9 (-65.9 to -27.6)	-68 (-76.4 to -53.8)	-29.2 (-51 to 3.6)	-60.9 (-76.2 to -41)
	Male	48859 (27278 to 69614)	173.3 (93.8 to 252.9)	861 (470 to 1248)	4.2 (2.3 to 6.1)	20310 (16405 to 24912)	48.6 (39.8 to 60.7)	533 (442 to 688)	1.4 (1.2 to 1.9)	-58.4 (-71.8 to -27.3)	-72 (-81.7 to -49.1)	-38 (-61 to 15)	-66.5 (-79.5 to -39.6)
Animal contact	Both	28649 (16045 to 38577)	39 (23.6 to 50.5)	336 (175 to 452)	0.5 (0.3 to 0.7)	12055 (9398 to 14655)	14.9 (11.4 to 17.9)	136 (98 to 160)	0.2 (0.1 to 0.2)	-57.9 (-70.1 to -31.8)	-61.9 (-71.7 to -42.4)	-59.6 (-71.1 to -30.7)	-65.8 (-74.4 to -45.9)
	Female	12212 (4891 to 17705)	33.7 (16.5 to 46.6)	135 (47 to 199)	0.4 (0.2 to 0.6)	5707 (4023 to 7198)	14.4 (10 to 18.1)	53 (31 to 67)	0.1 (0.1 to 0.2)	-53.3 (-70.1 to -3.2)	-57.4 (-71 to -26.6)	-60.7 (-74.8 to -14.4)	-64.4 (-75.7 to -34.9)
	Male	16437 (8146 to 22616)	44.1 (24 to 58.6)	200 (93 to 280)	0.6 (0.3 to 0.9)	6348 (5060 to 7688)	15.4 (12.2 to 18.5)	82 (63 to 97)	0.2 (0.2 to 0.3)	-61.4 (-73.5 to -27)	-65.1 (-75.2 to -39.4)	-58.9 (-72.2 to -17.8)	-66.5 (-76.9 to -37.3)
Foreign body	Both	99439 (74523 to 128540)	128.5 (100.2 to 164)	1098 (831 to 1426)	1.5 (1.2 to 1.9)	37703 (31184 to 45400)	47 (39.3 to 56)	426 (383 to 472)	0.6 (0.5 to 0.6)	-62.1 (-73.2 to -48)	-63.4 (-73.5 to -52.2)	-61.2 (-72.3 to -46.8)	-61.8 (-71.8 to -50.1)
	Female	48076 (35632 to 67395)	124.1 (94.9 to 168.6)	529 (387 to 741)	1.4 (1.1 to 1.9)	14337 (11645 to 17386)	37.1 (30.4 to 44.2)	152 (134 to 171)	0.4 (0.4 to 0.5)	-70.2 (-80.3 to -56.7)	-70.1 (-79.8 to -58.1)	-71.2 (-81 to -58.4)	-69.3 (-78.9 to -58)
	Male	51362 (34478 to 68810)	132.7 (95.5 to 172.8)	569 (392 to 763)	1.6 (1.2 to 2)	23366 (19370 to 28185)	56.6 (47.1 to 68)	274 (245 to 309)	0.7 (0.6 to 0.8)	-54.5 (-68.3 to -33.5)	-57.4 (-69.3 to -41.6)	-51.8 (-66.3 to -26.1)	-55.3 (-67.4 to -38.5)
Environmental heat and cold exposure	Both	8617 (6480 to 12154)	17.5 (13.2 to 24.8)	58 (43 to 120)	0.2 (0.1 to 0.3)	11846 (8629 to 15746)	13.6 (9.9 to 18.1)	71 (39 to 87)	0.1 (0.1 to 0.1)	37.5 (-1.7 to 57.5)	-22.3 (-41.5 to -12.1)	21.7 (-34 to 74.5)	-42.9 (-66.7 to -16.5)
	Female	3683 (2652 to 5234)	15.4 (11.2 to 21.3)	15 (10 to 37)	0.1 (0.1 to 0.2)	5508 (3895 to 7546)	12.8 (9.1 to 17.5)	20 (10 to 27)	0.1 (0 to 0.1)	49.5 (9.6 to 70.9)	-16.8 (-35.1 to -7)	35.5 (-34.4 to 115.7)	-32.5 (-69 to 15.5)
	Male	4934 (3740 to 7496)	19.5 (14.7 to 29.9)	43 (32 to 91)	0.2 (0.2 to 0.5)	6338 (4658 to 8272)	14.3 (10.6 to 18.7)	50 (24 to 66)	0.1 (0.1 to 0.2)	28.5 (-12.8 to 49.5)	-26.5 (-48.2 to -14.8)	16.9 (-34.6 to 65.1)	-47 (-70 to -24.7)
Exposure to forces of nature	Both	2541947 (2307397 to 2802597)	4047.8 (3673.3 to 4459.4)	40042 (36358 to 44058)	79.6 (72.3 to 87.6)	44303 (35543 to 55539)	48.7 (39.2 to 60.5)	126 (114 to 138)	0.2 (0.1 to 0.2)	-98.3 (-98.6 to -97.8)	-98.8 (-99 to -98.5)	-99.7 (-99.7 to -99.7)	-99.8 (-99.8 to -99.8)
	Female	1011071 (917686 to 1114226)	3160.4 (2869.9 to 3485)	15363 (13950 to 16904)	61.3 (55.7 to 67.4)	18031 (13739 to 23589)	40.4 (30.9 to 52.4)	46 (42 to 51)	0.1 (0.1 to 0.1)	-98.2 (-98.6 to -97.7)	-98.7 (-99 to -98.4)	-99.7 (-99.7 to -99.7)	-99.8 (-99.8 to -99.8)
	Male	1530876 (1390327 to 1688410)	4892 (4441.9 to 5393.1)	24679 (22408 to 27153)	96.9 (88 to 106.6)	26272 (21672 to 32084)	56.9 (47.4 to 68.8)	80 (72 to 87)	0.2 (0.2 to 0.2)	-98.3 (-98.6 to -97.9)	-98.8 (-99 to -98.6)	-99.7 (-99.7 to -99.7)	-99.8 (-99.8 to -99.8)
Other unintentional injuries	Both	126489 (102564 to 162262)	211.2 (170.7 to 270.4)	1396 (1109 to 1862)	2.4 (1.9 to 3.3)	105074 (85012 to 130176)	119.8 (97.2 to 148.2)	1056 (909 to 1158)	1.2 (1 to 1.3)	-16.9 (-38.5 to 4.8)	-43.3 (-57 to -31.5)	-24.3 (-48.5 to 0.9)	-49.2 (-65.7 to -33.6)

Cause	Sex	1990				2019				1990-2019 percent change (%)			
		DALYs		Death		DALYs		Death		DALYs		Death	
		Number	Rate	Number	Rate	Number	Rate	Number	Rate	Number	Rate	Number	Rate
	Female	41445 (30631 to 53343)	136.4 (102.4 to 172)	386 (263 to 523)	1.2 (0.9 to 1.6)	33261 (24277 to 44816)	78.2 (57.4 to 105.2)	193 (168 to 224)	0.5 (0.4 to 0.6)	-19.7 (-43.9 to 13.6)	-42.7 (-57 to -25.2)	-50 (-66.8 to -18.7)	-60.8 (-72.9 to -39.4)
	Male	85044 (66953 to 118128)	283.1 (223.1 to 386.7)	1010 (780 to 1518)	3.5 (2.7 to 5.2)	71813 (58534 to 86072)	160 (130.1 to 191.6)	864 (697 to 962)	1.9 (1.5 to 2.1)	-15.6 (-45.2 to 8.8)	-43.5 (-62.7 to -29.4)	-14.5 (-49.2 to 19.2)	-45 (-67.2 to -24.3)
Self-harm and interpersonal violence	Both	573025 (504356 to 660048)	1066.9 (933.3 to 1235.4)	6724 (6056 to 7374)	13.1 (11.6 to 14.5)	512467 (462154 to 573870)	565.2 (511.3 to 630)	7256 (6553 to 8016)	8.1 (7.3 to 8.9)	-10.6 (-17.9 to -1.6)	-47 (-52.1 to -41.2)	7.9 (-2.2 to 24.2)	-38.2 (-44.1 to -27.7)
	Female	177247 (152426 to 213326)	671.5 (575.7 to 809.5)	1866 (1666 to 2064)	7.2 (6.5 to 7.9)	139307 (122707 to 159280)	316.9 (281.5 to 357.1)	1632 (1487 to 1764)	3.8 (3.5 to 4.1)	-21.4 (-28.3 to -13.5)	-52.8 (-57.8 to -47.6)	-12.5 (-20.9 to -1.6)	-46.5 (-51.4 to -40.2)
	Male	395777 (347603 to 455523)	1446.4 (1267.9 to 1677.4)	4858 (4103 to 5426)	18.8 (15.5 to 21.2)	373159 (330120 to 419580)	806.5 (708.4 to 903.9)	5624 (4852 to 6352)	12.2 (10.5 to 13.8)	-5.7 (-15.4 to 8)	-44.2 (-50.3 to -35.7)	15.8 (1.8 to 42.1)	-34.8 (-42.6 to -19.3)
Self-harm	Both	216466 (184877 to 242896)	398.2 (343.7 to 455.7)	3640 (3105 to 4154)	7.4 (6.3 to 8.7)	221208 (189829 to 254451)	244.4 (207.8 to 278.4)	4172 (3546 to 4840)	4.7 (3.9 to 5.4)	2.2 (-8.9 to 24.9)	-38.6 (-44.9 to -25.4)	14.6 (2.2 to 42.8)	-37 (-43.8 to -21.1)
	Female	91298 (81504 to 101267)	328.6 (295.4 to 361.8)	1445 (1292 to 1599)	5.6 (5.1 to 6.2)	59057 (52349 to 64768)	137.6 (122.2 to 150.9)	1036 (917 to 1137)	2.4 (2.2 to 2.7)	-35.3 (-43 to -24.9)	-58.1 (-63.1 to -52)	-28.3 (-37.2 to -16.8)	-56.7 (-61.8 to -50.3)
	Male	125168 (94588 to 149501)	464.4 (353.1 to 570)	2195 (1663 to 2657)	9.1 (6.9 to 11.5)	162151 (126204 to 190303)	348.1 (266.9 to 404.7)	3136 (2438 to 3739)	6.9 (5.2 to 8.1)	29.5 (11.5 to 82.7)	-25 (-35.6 to 4.7)	42.8 (23.2 to 101.8)	-25 (-36.1 to 6.1)
Interpersonal violence	Both	113080 (89515 to 132896)	204.7 (163.8 to 238.2)	1491 (1092 to 1781)	2.9 (2.1 to 3.5)	165721 (148967 to 184727)	187.7 (168.2 to 208.6)	2209 (1978 to 2469)	2.5 (2.2 to 2.8)	46.6 (27.5 to 79.8)	-8.3 (-19.1 to 11.6)	48.1 (27.3 to 94.6)	-14.5 (-27.2 to 13.1)
	Female	34282 (27182 to 42109)	119.1 (97.1 to 142.5)	342 (255 to 426)	1.2 (1 to 1.5)	50674 (43831 to 58289)	118.1 (102.8 to 134.9)	513 (456 to 562)	1.2 (1.1 to 1.3)	47.8 (22.9 to 79.8)	-0.8 (-13.5 to 15.8)	49.9 (20.5 to 94.8)	-1.9 (-16.7 to 22.1)
	Male	78798 (56246 to 96565)	286.6 (206.4 to 345.7)	1149 (751 to 1436)	4.5 (2.9 to 5.6)	115047 (102478 to 130106)	255.1 (226.3 to 288.7)	1697 (1492 to 1962)	3.7 (3.3 to 4.3)	46 (23.4 to 96.8)	-11 (-24.5 to 17.4)	47.6 (21.2 to 111.6)	-17.2 (-32.4 to 20.3)
Conflict and terrorism	Both	212126 (161904 to 288966)	405.1 (303.6 to 561.7)	1106 (1004 to 1217)	1.8 (1.6 to 1.9)	74973 (49464 to 115615)	79.6 (53.1 to 121.7)	38 (35 to 42)	0 (0 to 0)	-64.7 (-70.4 to -59)	-80.3 (-83.6 to -77)	-96.6 (-96.6 to -96.6)	-97.5 (-97.5 to -97.5)
	Female	47050 (29153 to 77760)	206.6 (131 to 332.2)	16 (15 to 18)	0.1 (0.1 to 0.1)	24415 (14857 to 41914)	49.5 (30.4 to 83.9)	10 (9 to 11)	0 (0 to 0)	-48.1 (-55.6 to -41.2)	-76 (-80.5 to -71.4)	-37.4 (-37.4 to -37.4)	-61.6 (-61.6 to -61.6)
	Male	165076 (131127 to 213253)	596.1 (469.4 to 774.9)	1090 (989 to 1199)	3.4 (3.1 to 3.7)	50558 (33448 to 75689)	109.5 (73.1 to 162.2)	28 (25 to 31)	0.1 (0.1 to 0.1)	-69.4 (-74.8 to -63.9)	-81.6 (-84.8 to -78.3)	-97.5 (-97.5 to -97.5)	-98.2 (-98.2 to -98.2)
Executions and police conflict	Both	31353 (24107 to 35497)	59 (44.5 to 66.5)	486 (356 to 548)	1 (0.7 to 1.1)	50565 (30256 to 60398)	53.4 (33.5 to 63.4)	837 (457 to 1013)	0.9 (0.5 to 1.1)	61.3 (19.5 to 91.9)	-9.4 (-28.4 to 7.9)	72.1 (22.1 to 111.1)	-11.6 (-34.1 to 7.9)
	Female	4617 (4049 to 5285)	17.3 (14.9 to 20)	63 (55 to 72)	0.3 (0.2 to 0.3)	5162 (4340 to 6191)	11.8 (10 to 14)	73 (61 to 86)	0.2 (0.1 to 0.2)	11.8 (2.6 to 25)	-31.9 (-37.4 to -25.3)	16.3 (6.9 to 31.3)	-36.9 (-42.3 to -30.4)
	Male	26736 (19738 to 30515)	99.3 (71.2 to 112.6)	423 (296 to 482)	1.7 (1.2 to 1.9)	45403 (25603 to 54979)	93.8 (55.6 to 112.9)	763 (390 to 934)	1.6 (0.9 to 1.9)	69.8 (21.1 to 105.7)	-5.5 (-27.9 to 15.2)	80.4 (23.4 to 124.6)	-7.4 (-33.1 to 15.7)

Data in parentheses are 95% uncertainty intervals. DALY=disability-adjusted life-year

Appendix Table 8. Provincial DALYs and death due to causes, number for all ages and age-standardised rate (per 100,000) in 1990 and 2019 with percent changes between 1990 and 2019, by sex

Province	Cause	Sex	1990				2019				1990-2019 percent change (%)			
			DALYs		Death		DALYs		Death		DALYs		Death	
			Number	Rate	Number	Rate	Number	Rate	Number	Rate	Number	Rate	Number	Rate
**Alborz**	**All causes**	Both	493711 (435060 to 556123)	37103.3 (32824.8 to 41531.8)	6610 (5873 to 7427)	891.1 (786.3 to 1004.3)	662396 (562009 to 769444)	23766.5 (20384.7 to 27225.8)	12078 (10678 to 13563)	590.1 (526.7 to 656.1)	34.2 (17.5 to 52.6)	-35.9 (-43.1 to -28.4)	82.7 (53.3 to 116.8)	-33.8 (-44.1 to -22)
		Female	214218 (188095 to 244243)	34402.4 (30166.2 to 39431.5)	2711 (2360 to 3117)	817.5 (690.2 to 949.3)	303615 (251502 to 365359)	22657.3 (19031.9 to 26798.5)	4873 (4187 to 5657)	571.8 (498.3 to 653.5)	41.7 (20.9 to 62.4)	-34.1 (-42.5 to -25.3)	79.7 (46.9 to 122.7)	-30 (-42.5 to -12.7)
		Male	279493 (246343 to 316454)	39538.9 (34460.6 to 45374.7)	3899 (3355 to 4529)	956 (799.2 to 1149.1)	358781 (305980 to 415740)	25028.8 (21506.2 to 28836.7)	7205 (6064 to 8510)	629.2 (533.3 to 737.5)	28.4 (9.8 to 49.5)	-36.7 (-46.4 to -26.1)	84.8 (46.4 to 130.7)	-34.2 (-48 to -16)
	**Communicable, maternal, neonatal, and nutritional diseases**	Both	172419 (135716 to 225593)	7691.3 (6148.9 to 10380.4)	1963 (1533 to 2608)	110.7 (87.9 to 180.7)	55058 (47301 to 63597)	2050.6 (1761.8 to 2365.5)	744 (629 to 843)	34.6 (29.6 to 39.1)	-68.1 (-76.6 to -58)	-73.3 (-80.8 to -65.3)	-62.1 (-73 to -49.8)	-68.7 (-80.5 to -59.5)
		Female	73861 (58149 to 94636)	6867.8 (5471.4 to 9110.7)	831 (647 to 1085)	98.9 (76.8 to 159.8)	23865 (20103 to 27831)	1856.6 (1572.1 to 2141.9)	294 (236 to 343)	32.1 (25.5 to 37.7)	-67.7 (-76.6 to -57.2)	-73 (-80.5 to -64.9)	-64.7 (-75 to -52.2)	-67.6 (-80.2 to -56.7)
		Male	98558 (75588 to 131376)	8462.2 (6544.1 to 11590.1)	1132 (867 to 1541)	121.5 (94.4 to 192.1)	31193 (26716 to 35916)	2243.5 (1932.4 to 2592.5)	450 (374 to 523)	37.9 (31.7 to 44.3)	-68.4 (-77.4 to -56.9)	-73.5 (-81.4 to -64.3)	-60.2 (-72.3 to -44.7)	-68.8 (-80.1 to -57.6)
	HIV/AIDS and sexually transmitted infections	Both	396 (220 to 711)	23.7 (14.7 to 38.4)	3 (2 to 7)	0.2 (0.1 to 0.3)	1760 (1268 to 2448)	51.3 (36.5 to 71.9)	25 (18 to 38)	0.8 (0.5 to 1.1)	344.9 (151.3 to 687.5)	116.7 (38.9 to 232.9)	663.8 (243.4 to 1739.1)	297.7 (106.4 to 675)
		Female	192 (105 to 356)	25 (14.9 to 41.6)	1 (1 to 3)	0.1 (0.1 to 0.3)	613 (371 to 928)	38.6 (23.3 to 58.8)	6 (3 to 11)	0.4 (0.2 to 0.8)	219.8 (76.9 to 463.9)	54.1 (-0.7 to 149.3)	379.8 (69.3 to 1323.7)	218.5 (34.1 to 641.2)
		Male	204 (110 to 376)	22.6 (14.4 to 36.5)	2 (1 to 4)	0.2 (0.1 to 0.4)	1147 (871 to 1640)	63.6 (48.1 to 89)	19 (14 to 29)	1.1 (0.8 to 1.6)	462.2 (203.6 to 961.5)	182.1 (74.4 to 355.5)	848.8 (358.5 to 2083.4)	348.6 (145.9 to 763.6)
	HIV/AIDS	Both	51 (25 to 72)	4.1 (1.9 to 5.8)	1 (0 to 1)	0.1 (0 to 0.1)	1185 (820 to 1825)	33.1 (22.3 to 50.8)	22 (15 to 35)	0.6 (0.4 to 1)	2241 (1206 to 5148.7)	712.3 (354 to 1719.6)	2806.4 (1382.1 to 8621.4)	779.5 (349.2 to 2700.5)
		Female	11 (5 to 15)	1.5 (0.8 to 2.1)	0 (0 to 0)	0 (0 to 0)	270 (116 to 488)	17.4 (7.3 to 31)	5 (2 to 9)	0.3 (0.1 to 0.6)	2453.9 (862.4 to 6212.5)	1055.4 (326.1 to 2661.3)	3090 (875.6 to 9810.6)	1089.9 (249.5 to 3675.1)
		Male	40 (19 to 57)	6.4 (2.8 to 9.1)	1 (0 to 1)	0.1 (0 to 0.2)	915 (693 to 1383)	48.3 (35.9 to 72.4)	17 (13 to 27)	0.9 (0.7 to 1.4)	2184.8 (1275 to 5075.2)	652.8 (356.8 to 1669.2)	2737.9 (1474.6 to 8995.3)	729.5 (360.7 to 2672.6)
	Sexually transmitted infections excluding HIV	Both	345 (173 to 665)	19.6 (11.1 to 34.2)	3 (1 to 6)	0.1 (0.1 to 0.3)	575 (352 to 911)	18.2 (11 to 29.8)	3 (2 to 6)	0.1 (0.1 to 0.2)	66.6 (5.7 to 140.5)	-7.1 (-29.9 to 16.3)	33.6 (-20.5 to 157.3)	21.5 (-26 to 103.6)
		Female	181 (97 to 345)	23.5 (13.4 to 39.9)	1 (0 to 3)	0.1 (0.1 to 0.2)	342 (203 to 552)	21.2 (12.8 to 34)	2 (1 to 3)	0.2 (0.1 to 0.2)	89.2 (20 to 161.9)	-9.9 (-28.3 to 8.6)	37.5 (-19.8 to 171.6)	30.5 (-24.7 to 124.8)
		Male	164 (74 to 338)	16.1 (8.4 to 30)	1 (0 to 3)	0.1 (0 to 0.3)	232 (138 to 380)	15.4 (8.9 to 25.3)	2 (1 to 3)	0.1 (0 to 0.3)	41.6 (-13.9 to 121.5)	-4.8 (-35.2 to 29.7)	30.3 (-29.8 to 174)	16.9 (-36.2 to 122.2)
	Respiratory infections and tuberculosis	Both	37897 (27631 to 53805)	1952.8 (1503 to 2630.5)	494 (364 to 682)	42.8 (32.8 to 51.3)	15468 (12443 to 18052)	596.8 (484.3 to 695.3)	429 (324 to 500)	22 (17.2 to 25.8)	-59.2 (-72.3 to -42.6)	-69.4 (-77.8 to -59.2)	-13.2 (-38.6 to 20.1)	-48.5 (-59 to -34.5)
		Female	15452 (10503 to 22916)	1638.6 (1165.4 to 2260.1)	199 (137 to 287)	36.9 (26.4 to 46.6)	5846 (4571 to 7123)	501.2 (390.5 to 599.5)	164 (114 to 201)	20.9 (14.6 to 25.6)	-62.2 (-75.5 to -42.5)	-69.4 (-78.8 to -56.9)	-17.8 (-43.8 to 20.3)	-43.4 (-57 to -23.6)

Province	Cause	Sex	1990				2019				1990-2019 percent change (%)			
			DALYs		Death		DALYs		Death		DALYs		Death	
			Number	Rate	Number	Rate	Number	Rate	Number	Rate	Number	Rate	Number	Rate
		Male	22444 (15702 to 33358)	2244.2 (1663.4 to 3123.2)	295 (210 to 422)	48.2 (36.3 to 60.6)	9622 (7299 to 11478)	695.7 (541.2 to 826.4)	265 (195 to 324)	23.8 (18.3 to 29.2)	-57.1 (-72.7 to -35.8)	-69 (-78.5 to -56.4)	-10.1 (-40.6 to 31.3)	-50.6 (-63.1 to -32.8)
	Tuberculosis	Both	1110 (778 to 1391)	99.3 (68.7 to 124.1)	22 (15 to 28)	3.3 (2.2 to 4.1)	754 (640 to 882)	26.9 (23.1 to 31.3)	22 (18 to 26)	1 (0.8 to 1.2)	-32 (-47.9 to 3.8)	-72.9 (-79.3 to -57.7)	-0.3 (-26.3 to 65.2)	-70.1 (-78 to -50.5)
		Female	506 (253 to 694)	92.5 (44.9 to 127)	10 (4 to 13)	3 (1.2 to 4.3)	320 (257 to 395)	24.9 (20.3 to 30.8)	9 (7 to 12)	1 (0.8 to 1.2)	-36.8 (-56.7 to 42.8)	-73.1 (-81.4 to -36.4)	-2 (-35.5 to 179.5)	-67.8 (-79.3 to -4.5)
		Male	604 (448 to 763)	105.7 (77.5 to 135.1)	12 (9 to 16)	3.5 (2.5 to 4.7)	434 (350 to 535)	29.1 (23.6 to 35.5)	13 (10 to 16)	1 (0.8 to 1.3)	-28 (-47 to 3.2)	-72.4 (-79.9 to -59.8)	1.1 (-28.1 to 51.5)	-71.3 (-79.7 to -56.3)
	Lower respiratory infections	Both	34824 (24733 to 50703)	1737.8 (1290.5 to 2401.2)	472 (347 to 659)	39.5 (30.2 to 47.9)	11406 (8271 to 13134)	456.8 (348.5 to 522.4)	406 (302 to 475)	21 (16.3 to 24.6)	-67.2 (-78.6 to -51.9)	-73.7 (-81.8 to -63.6)	-13.8 (-39.9 to 19.8)	-46.8 (-58.1 to -32.2)
		Female	14037 (9133 to 21430)	1435.2 (970.2 to 2061.9)	190 (127 to 277)	34 (23.5 to 43.1)	3972 (2862 to 4746)	368.1 (266.5 to 438.3)	154 (105 to 189)	20 (13.8 to 24.6)	-71.7 (-82.8 to -55.4)	-74.4 (-82.8 to -62.6)	-18.6 (-45.2 to 20.6)	-41.2 (-55.8 to -20.1)
		Male	20788 (14173 to 31513)	2018.5 (1450.6 to 2875.6)	282 (201 to 408)	44.6 (33.5 to 56.6)	7434 (5040 to 8966)	548.8 (398.3 to 656.1)	252 (181 to 309)	22.8 (17.4 to 28)	-64.2 (-78.2 to -44.7)	-72.8 (-82.1 to -60.3)	-10.6 (-41.8 to 31.8)	-48.9 (-62.1 to -30)
	Upper respiratory infections	Both	1428 (865 to 2288)	85.2 (51.9 to 132.8)	0 (0 to 0)	0 (0 to 0)	2513 (1505 to 3947)	85.4 (51.3 to 133.9)	0 (0 to 1)	0 (0 to 0)	76 (62.3 to 92.7)	0.3 (-4.4 to 5.3)	91.1 (-81.5 to 1227.8)	17.8 (-87 to 518.6)
		Female	677 (407 to 1075)	83.1 (50.1 to 130.8)	0 (0 to 0)	0 (0 to 0)	1201 (720 to 1874)	83.2 (49.9 to 131.2)	0 (0 to 0)	0 (0 to 0)	77.3 (60.2 to 98.4)	0.1 (-6.2 to 7.2)	60.6 (-86.5 to 1496.4)	22.6 (-88 to 665.7)
		Male	751 (455 to 1176)	87 (53.4 to 135.9)	0 (0 to 0)	0 (0 to 0)	1313 (789 to 2055)	87.5 (53.2 to 136.5)	0 (0 to 1)	0 (0 to 0)	74.8 (57.9 to 93.8)	0.5 (-6.6 to 8.4)	106.3 (-79.9 to 1345.5)	15.7 (-86.5 to 523.1)
	Otitis media	Both	534 (302 to 884)	30.5 (17.5 to 50.8)	0 (0 to 0)	0 (0 to 0)	794 (439 to 1329)	27.7 (15.4 to 45.9)	0 (0 to 0)	0 (0 to 0)	48.5 (33.6 to 67.5)	-9.2 (-15.1 to -3.3)	-50.5 (-81 to 55.1)	-71.2 (-86.9 to -31)
		Female	232 (135 to 386)	27.8 (15.8 to 46.1)	0 (0 to 0)	0 (0 to 0)	353 (199 to 591)	25 (14 to 41.5)	0 (0 to 0)	0 (0 to 0)	52 (34.2 to 76.5)	-10 (-18.2 to -1.4)	8.7 (-60.3 to 296.9)	-54.1 (-81.4 to 53.7)
		Male	302 (166 to 499)	33.1 (18.7 to 54.8)	0 (0 to 0)	0 (0 to 0)	441 (244 to 737)	30.3 (16.8 to 50.3)	0 (0 to 0)	0 (0 to 0)	45.9 (29.4 to 66.8)	-8.4 (-16.8 to 0.1)	-70.8 (-91.2 to 15.2)	-79.7 (-92.8 to -33.9)
	Enteric infections	Both	7596 (5131 to 11081)	376.8 (268.5 to 522.8)	75 (48 to 116)	5.1 (3.3 to 7.6)	6788 (5037 to 8814)	257.7 (193.1 to 335.3)	36 (25 to 53)	1.7 (1.2 to 2.7)	-10.6 (-45.4 to 30.8)	-31.6 (-54.5 to -7.5)	-52 (-71.9 to -23.1)	-66.7 (-77.3 to -52)
		Female	3605 (2258 to 5912)	372 (247.4 to 572.7)	36 (19 to 63)	5.5 (2.8 to 9.9)	3247 (2397 to 4247)	253.8 (187.7 to 330.4)	16 (10 to 28)	1.8 (1 to 3.3)	-9.9 (-46.9 to 44.7)	-31.8 (-56.1 to 2.1)	-54.1 (-77.7 to -10.8)	-68.3 (-81.9 to -44.3)
		Male	3991 (2573 to 6391)	380.6 (258.2 to 566.4)	39 (23 to 66)	4.7 (2.9 to 7.9)	3541 (2606 to 4658)	261.9 (195 to 342.2)	19 (13 to 33)	1.7 (1.2 to 3.1)	-11.3 (-49.2 to 39.4)	-31.2 (-57.9 to -1.9)	-50 (-73.5 to -7.6)	-63.6 (-77.2 to -43.8)
	Diarrheal diseases	Both	6428 (4176 to 9822)	318.9 (222.2 to 457.6)	59 (34 to 97)	4.3 (2.5 to 6.8)	6129 (4468 to 8117)	231.6 (168.1 to 305.9)	26 (17 to 42)	1.3 (0.9 to 2.3)	-4.7 (-44.3 to 44.3)	-27.4 (-53.7 to 1.6)	-55.9 (-75.9 to -21.8)	-69 (-80.1 to -52.9)
		Female	3057 (1783 to 5335)	316.5 (199.1 to 508.4)	28 (14 to 54)	4.7 (2.1 to 8.9)	2941 (2106 to 3932)	229.1 (164.7 to 306.2)	12 (6 to 23)	1.4 (0.6 to 3)	-3.8 (-46.7 to 62.6)	-27.6 (-55.4 to 11.8)	-58.5 (-83.1 to -5.8)	-70.5 (-84.5 to -43.8)
		Male	3371 (2041 to 5665)	320.6 (208.6 to 500.6)	31 (16 to 57)	3.8 (2.2 to 7.1)	3187 (2323 to 4263)	234.4 (172.1 to 311.1)	14 (9 to 27)	1.3 (0.8 to 2.6)	-5.4 (-49.6 to 56.9)	-26.9 (-57.2 to 10)	-53.6 (-78.2 to -2.9)	-65.7 (-80.1 to -42.7)
	Typhoid and paratyphoid	Both	938 (348 to 1840)	46.8 (17.8 to 91.9)	13 (5 to 25)	0.7 (0.3 to 1.3)	496 (203 to 967)	19.9 (8 to 38.9)	7 (3 to 14)	0.3 (0.1 to 0.5)	-47.2 (-61.6 to -22.5)	-57.6 (-68.5 to -37.5)	-43.1 (-58 to -16.1)	-58.5 (-69 to -39.8)
		Female	416 (156 to 847)	42.6 (15.9 to 86)	6 (2 to 12)	0.6 (0.2 to 1.2)	229 (91 to 448)	18.7 (7.4 to 37)	3 (1 to 6)	0.3 (0.1 to 0.5)	-44.9 (-61.9 to -15.7)	-56 (-69 to -33.1)	-40.6 (-59.1 to -6.4)	-57.4 (-69.9 to -34)
		Male	522 (198 to 1051)	50.7 (20.1 to 101.3)	7 (3 to 14)	0.7 (0.3 to 1.4)	267 (107 to 515)	20.9 (8.4 to 40.5)	4 (2 to 7)	0.3 (0.1 to 0.6)	-49 (-64.2 to -20.6)	-58.7 (-71 to -38.6)	-45 (-61.5 to -15.5)	-59.3 (-71 to -39)
		Both	199 (102 to 340)	9.7 (5 to 16.3)	3 (1 to 5)	0.2 (0.1 to 0.3)	153 (75 to 272)	5.9 (2.9 to 10.6)	3 (1 to 5)	0.1 (0 to 0.2)	-22.9 (-52.2 to 15.8)	-39.5 (-59.9 to -10.4)	-4.3 (-35.9 to 42.4)	-39.1 (-55.9 to -14.2)

Province	Cause	Sex	1990				2019				1990-2019 percent change (%)			
			DALYs		Death		DALYs		Death		DALYs		Death	
			Number	Rate	Number	Rate	Number	Rate	Number	Rate	Number	Rate	Number	Rate
	Invasive Non-typhoidal Salmonella (iNTS)	Female	116 (58 to 200)	11.5 (5.7 to 19.5)	2 (1 to 3)	0.2 (0.1 to 0.3)	72 (35 to 130)	5.6 (2.7 to 10.1)	1 (1 to 2)	0.1 (0 to 0.2)	-38.4 (-61.6 to -0.8)	-51.3 (-68.5 to -24)	-21.4 (-49 to 23.4)	-49.2 (-64.7 to -24)
		Male	83 (41 to 145)	8.1 (4.1 to 13.9)	1 (1 to 2)	0.1 (0.1 to 0.2)	82 (39 to 149)	6.2 (2.9 to 11.5)	1 (1 to 2)	0.1 (0.1 to 0.2)	-1.2 (-39.3 to 50.2)	-23.4 (-51 to 14.6)	18.8 (-23.1 to 74.8)	-25.6 (-49 to 4.9)
	Other intestinal infectious diseases	Both	31 (8 to 64)	1.3 (0.4 to 2.6)	0 (0 to 1)	0 (0 to 0)	10 (7 to 14)	0.4 (0.2 to 0.5)	0 (0 to 0)	0 (0 to 0)	-68 (-86.3 to 32.8)	-71.9 (-87.6 to -8.3)	-73.2 (-89.8 to 23.7)	-77 (-90.6 to -31.1)
		Female	16 (2 to 42)	1.4 (0.3 to 3.5)	0 (0 to 0)	0 (0 to 0)	5 (3 to 7)	0.4 (0.2 to 0.6)	0 (0 to 0)	0 (0 to 0)	-70.2 (-90 to 132.7)	-73.3 (-90.8 to 48.3)	-75.1 (-93.4 to 202.3)	-76.1 (-93.5 to 13.2)
		Male	15 (2 to 37)	1.3 (0.3 to 3)	0 (0 to 0)	0 (0 to 0)	5 (3 to 8)	0.4 (0.2 to 0.6)	0 (0 to 0)	0 (0 to 0)	-65.6 (-87.9 to 129.2)	-70.3 (-89 to 46.2)	-71.1 (-92.5 to 217.1)	-76.6 (-93.2 to 11.1)
	Neglected tropical diseases and malaria	Both	4355 (445 to 45223)	323.7 (26 to 3599.3)	74 (2 to 873)	7.4 (0.2 to 85.9)	427 (288 to 637)	15.2 (10.2 to 22.9)	3 (1 to 5)	0.1 (0.1 to 0.2)	-90.2 (-99.1 to -12.7)	-95.3 (-99.6 to -47.2)	-96.4 (-99.7 to 13.2)	-98.5 (-99.9 to -47.8)
		Female	1929 (199 to 18396)	301.7 (25.5 to 3216.8)	32 (1 to 366)	7 (0.1 to 80)	206 (138 to 296)	15 (9.9 to 21.6)	1 (0 to 2)	0.1 (0 to 0.2)	-89.3 (-98.9 to -7.7)	-95 (-99.5 to -48.1)	-96.7 (-99.7 to 27.3)	-98.6 (-99.9 to -39.7)
		Male	2426 (214 to 26368)	341.8 (23.5 to 3847.6)	42 (1 to 495)	7.6 (0.2 to 86.6)	221 (143 to 371)	15.5 (9.9 to 26.4)	2 (1 to 4)	0.1 (0.1 to 0.3)	-90.9 (-99.2 to -2.8)	-95.5 (-99.6 to -37.1)	-96.2 (-99.7 to 22.2)	-98.4 (-99.9 to -43.2)
	Malaria	Both	3453 (30 to 44356)	275.6 (1.7 to 3551.2)	67 (0 to 865)	6.9 (0 to 85.3)	33 (24 to 45)	1.1 (0.8 to 1.5)	0 (0 to 0)	0 (0 to 0)	-99 (-99.9 to 11.4)	-99.6 (-100 to -38.7)	-100 (-100 to 5.6)	-100 (-100 to -54)
		Female	1553 (16 to 18181)	258.3 (2 to 3190)	30 (0 to 365)	6.6 (0 to 79.8)	17 (12 to 23)	1.1 (0.8 to 1.5)	0 (0 to 0)	0 (0 to 0)	-98.9 (-99.9 to 4.4)	-99.6 (-100 to -45.6)	-100 (-100 to 1.2)	-100 (-100 to -53.8)
		Male	1900 (13 to 26136)	289.2 (1.4 to 3824.5)	37 (0 to 494)	7 (0 to 86.4)	16 (12 to 22)	1.1 (0.8 to 1.4)	0 (0 to 0)	0 (0 to 0)	-99.1 (-99.9 to 27.6)	-99.6 (-100 to -26.2)	-100 (-100 to 7.4)	-100 (-100 to -54.8)
	Leishmaniasis	Both	369 (2 to 3888)	18.5 (0.1 to 179.8)	5 (0 to 51)	0.3 (0 to 2.6)	23 (1 to 206)	0.8 (0 to 7.7)	0 (0 to 3)	0 (0 to 0.1)	-93.6 (-95.1 to 14.7)	-95.5 (-96.4 to -27.2)	-93 (-94.2 to -86.1)	-95.8 (-96.8 to -94)
		Female	120 (1 to 1203)	12.3 (0.1 to 115.2)	2 (0 to 16)	0.2 (0 to 1.7)	8 (1 to 64)	0.6 (0.1 to 4.8)	0 (0 to 1)	0 (0 to 0.1)	-93.1 (-95.1 to 67.9)	-95.2 (-96.7 to -1.8)	-93 (-94.4 to -85.1)	-95.9 (-97.1 to -93.9)
		Male	249 (1 to 2778)	24.3 (0.1 to 253)	3 (0 to 37)	0.4 (0 to 3.6)	15 (0 to 139)	1.1 (0 to 10.3)	0 (0 to 2)	0 (0 to 0.1)	-93.9 (-95.1 to -21.8)	-95.6 (-96.6 to -44.4)	-93 (-94.2 to -86.2)	-95.7 (-96.8 to -93.7)
	Schistosomiasis	Both	0 (0 to 0)	0 (0 to 0)	0 (0 to 0)	0 (0 to 0)	0 (0 to 0)	0 (0 to 0)	0 (0 to 0)	0 (0 to 0)	0 (0 to 0)	0 (0 to 0)	0 (0 to 0)	0 (0 to 0)
		Female	0 (0 to 0)	0 (0 to 0)	0 (0 to 0)	0 (0 to 0)	0 (0 to 0)	0 (0 to 0)	0 (0 to 0)	0 (0 to 0)	0 (0 to 0)	0 (0 to 0)	0 (0 to 0)	0 (0 to 0)
		Male	0 (0 to 0)	0 (0 to 0)	0 (0 to 0)	0 (0 to 0)	0 (0 to 0)	0 (0 to 0)	0 (0 to 0)	0 (0 to 0)	0 (0 to 0)	0 (0 to 0)	0 (0 to 0)	0 (0 to 0)
	Cystic echinococcosis	Both	70 (40 to 109)	5.1 (3.3 to 7.2)	1 (0 to 1)	0.1 (0 to 0.1)	79 (51 to 117)	2.5 (1.7 to 3.7)	0 (0 to 1)	0 (0 to 0)	13.1 (-34.2 to 102.1)	-50.5 (-67.3 to -21.4)	-48.5 (-75.7 to 22.5)	-78.2 (-89.3 to -54.1)
		Female	30 (16 to 48)	4.8 (2.8 to 7.1)	0 (0 to 1)	0.1 (0 to 0.1)	43 (26 to 64)	2.7 (1.8 to 4.1)	0 (0 to 0)	0 (0 to 0)	42.6 (-24.9 to 179.9)	-42.4 (-65.3 to -2.9)	-45.6 (-82.5 to 95.2)	-73.3 (-92.2 to -9.3)
		Male	40 (17 to 72)	5.5 (2.9 to 8.4)	0 (0 to 1)	0.1 (0 to 0.2)	37 (23 to 55)	2.4 (1.5 to 3.4)	0 (0 to 0)	0 (0 to 0)	-8.8 (-56.1 to 113)	-57 (-74.9 to -19)	-50.4 (-85 to 54.9)	-80.1 (-92.9 to -44.5)
	Trachoma	Both	6 (3 to 10)	1.2 (0.6 to 2.1)	--	--	6 (3 to 10)	0.3 (0.1 to 0.5)	--	--	3.1 (-21.5 to 36.4)	-78.3 (-83.3 to -71)	--	--
		Female	4 (2 to 7)	1.8 (1 to 3.1)	--	--	4 (2 to 7)	0.4 (0.2 to 0.6)	--	--	-5.8 (-31 to 28.1)	-80.3 (-85.6 to -72.8)	--	--
		Male	2 (1 to 3)	0.6 (0.3 to 1.1)	--	--	2 (1 to 4)	0.2 (0.1 to 0.3)	--	--	27.7 (-16.2 to 99.9)	-71.2 (-81.1 to -56.1)	--	--

Province	Cause	Sex	1990				2019				1990-2019 percent change (%)			
			DALYs		Death		DALYs		Death		DALYs		Death	
			Number	Rate	Number	Rate	Number	Rate	Number	Rate	Number	Rate	Number	Rate
	Rabies	Both	2 (0 to 5)	0.1 (0 to 0.3)	0 (0 to 0)	0 (0 to 0)	1 (1 to 4)	0 (0 to 0.1)	0 (0 to 0)	0 (0 to 0)	-22.5 (-69.3 to 258.1)	-59 (-82.5 to 69)	8.9 (-53.6 to 342.9)	-54.5 (-80.6 to 71.9)
		Female	1 (0 to 3)	0.1 (0 to 0.3)	0 (0 to 0)	0 (0 to 0)	1 (0 to 1)	0 (0 to 0.1)	0 (0 to 0)	0 (0 to 0)	-40.4 (-81.1 to 297.5)	-69.1 (-89 to 76.5)	-15 (-69.4 to 392.1)	-65.1 (-86.5 to 72.7)
		Male	1 (0 to 3)	0.1 (0 to 0.4)	0 (0 to 0)	0 (0 to 0)	1 (0 to 3)	0.1 (0 to 0.2)	0 (0 to 0)	0 (0 to 0)	-2.4 (-65.9 to 431.9)	-47.4 (-81 to 169.8)	36.1 (-52.2 to 579)	-40.6 (-78 to 176.8)
	Intestinal nematode infections	Both	63 (31 to 110)	3.5 (1.9 to 5.9)	0 (0 to 1)	0 (0 to 0)	21 (11 to 38)	0.7 (0.4 to 1.3)	0 (0 to 0)	0 (0 to 0)	-67 (-83.4 to -35.6)	-79.1 (-88.5 to -62.3)	-87.3 (-96 to -33)	-87.9 (-95.6 to -61)
		Female	36 (17 to 67)	4.6 (2.4 to 8.4)	0 (0 to 0)	0 (0 to 0)	13 (6 to 24)	0.9 (0.4 to 1.7)	0 (0 to 0)	0 (0 to 0)	-64.1 (-82.5 to -29.2)	-79.9 (-89.6 to -61.2)	-85.8 (-97.1 to 120.3)	-83.3 (-96.8 to 0.3)
		Male	27 (9 to 59)	2.6 (1 to 5.1)	0 (0 to 1)	0 (0 to 0)	8 (4 to 14)	0.6 (0.3 to 1)	0 (0 to 0)	0 (0 to 0)	-70.8 (-89.2 to -15)	-78.2 (-91.2 to -47.5)	-88.2 (-97.5 to 64.3)	-89.7 (-97.7 to -41.3)
	Food-borne trematodiases	Both	5 (1 to 11)	0.5 (0.1 to 1)	--	--	32 (7 to 66)	1 (0.2 to 2.1)	--	--	526.3 (364.7 to 725.5)	107.7 (60.9 to 162.2)	--	--
		Female	2 (0 to 4)	0.4 (0.1 to 0.8)	--	--	12 (3 to 26)	0.8 (0.2 to 1.7)	--	--	523.8 (369.5 to 722.8)	100.8 (48.8 to 163.4)	--	--
		Male	3 (1 to 6)	0.6 (0.1 to 1.2)	--	--	19 (4 to 41)	1.2 (0.3 to 2.5)	--	--	527.9 (290.1 to 816.6)	114.9 (48.3 to 195.8)	--	--
	Leprosy	Both	0 (0 to 0)	0 (0 to 0)	--	--	1 (0 to 1)	0 (0 to 0)	--	--	230.6 (204.9 to 262.2)	-11.6 (-17.9 to -3.9)	--	--
		Female	0 (0 to 0)	0 (0 to 0)	--	--	0 (0 to 0)	0 (0 to 0)	--	--	213.7 (174.3 to 252.1)	-19.4 (-28.6 to -10.6)	--	--
		Male	0 (0 to 0)	0 (0 to 0.1)	--	--	1 (0 to 1)	0 (0 to 0.1)	--	--	239.3 (202.7 to 283.9)	-6.5 (-15.4 to 4.6)	--	--
	Other neglected tropical diseases	Both	387 (236 to 588)	19.2 (12.4 to 28.4)	2 (1 to 2)	0.1 (0.1 to 0.2)	230 (142 to 355)	8.7 (5.4 to 13.4)	2 (1 to 2)	0.1 (0 to 0.1)	-40.5 (-61.1 to -7.6)	-54.8 (-68.9 to -32.1)	19.1 (-35.2 to 107.7)	-38 (-57.3 to 0.9)
		Female	183 (103 to 298)	19.4 (11.7 to 29.6)	1 (0 to 1)	0.1 (0 to 0.2)	108 (64 to 174)	8.4 (5 to 13.4)	1 (0 to 1)	0.1 (0 to 0.1)	-40.8 (-63.7 to 2.5)	-56.9 (-72.2 to -29.9)	24.2 (-31.5 to 171.2)	-37.4 (-60 to 17.4)
		Male	204 (105 to 341)	19 (10.6 to 30.5)	1 (0 to 2)	0.1 (0.1 to 0.2)	122 (68 to 205)	9 (4.9 to 15.3)	1 (0 to 2)	0.1 (0 to 0.1)	-40.1 (-68.5 to 14.5)	-52.6 (-74.1 to -10.9)	15.8 (-44.6 to 143.3)	-37.8 (-61.4 to 14.8)
	Other infectious diseases	Both	14211 (7493 to 28082)	629.9 (358.8 to 1167.7)	169 (90 to 327)	9.3 (5.8 to 15.3)	2990 (2442 to 3569)	108.4 (88.3 to 129.6)	58 (45 to 70)	2.5 (1.9 to 3)	-79 (-89.3 to -60.1)	-82.8 (-90.8 to -69.6)	-65.5 (-82.5 to -36.2)	-73.3 (-84.6 to -56.9)
		Female	7130 (3523 to 14024)	641.3 (348.2 to 1196.7)	84 (42 to 164)	9.2 (5.6 to 15.8)	1272 (1042 to 1568)	96.5 (79 to 118)	22 (17 to 26)	2 (1.6 to 2.5)	-82.2 (-91.3 to -63.7)	-85 (-92.2 to -72.1)	-74.2 (-87.2 to -48.8)	-77.8 (-87.6 to -61.7)
		Male	7081 (3822 to 13426)	617.9 (358.8 to 1110.2)	85 (46 to 159)	9.3 (5.8 to 15.1)	1718 (1336 to 2073)	120.2 (92.6 to 144.6)	37 (26 to 46)	2.9 (2 to 3.7)	-75.7 (-87.2 to -54.1)	-80.5 (-89.4 to -66)	-56.9 (-77.7 to -19.4)	-68.6 (-81.4 to -48.3)
	Meningitis	Both	2503 (1765 to 3479)	121.2 (88.1 to 160.5)	31 (21 to 42)	2 (1.5 to 2.6)	846 (713 to 1006)	30.4 (25.7 to 36)	16 (13 to 19)	0.7 (0.6 to 0.8)	-66.2 (-77 to -48.8)	-74.9 (-82.2 to -63.2)	-48.2 (-64.1 to -19.7)	-66 (-75.5 to -50.6)
		Female	1097 (722 to 1638)	111.6 (77.4 to 156.5)	13 (9 to 20)	1.9 (1.3 to 2.6)	394 (318 to 487)	29.4 (24 to 35.9)	6 (5 to 8)	0.6 (0.5 to 0.8)	-64.1 (-78.1 to -41.6)	-73.7 (-82.7 to -60)	-52.1 (-70.2 to -21.4)	-66.9 (-78.3 to -50.3)

Province	Cause	Sex	1990				2019				1990-2019 percent change (%)			
			DALYs		Death		DALYs		Death		DALYs		Death	
			Number	Rate	Number	Rate	Number	Rate	Number	Rate	Number	Rate	Number	Rate
		Male	1406 (917 to 2149)	130.2 (89.7 to 188.4)	17 (12 to 26)	2.1 (1.4 to 2.9)	453 (367 to 562)	31.5 (25.6 to 38.9)	10 (7 to 12)	0.8 (0.6 to 1)	-67.8 (-80.2 to -45.1)	-75.8 (-84.7 to -60.1)	-45.2 (-66.4 to -4)	-64.6 (-77.5 to -41.4)
	Encephalitis	Both	326 (231 to 471)	18.1 (13.6 to 24)	4 (3 to 6)	0.3 (0.2 to 0.4)	440 (336 to 539)	16 (11.8 to 19.8)	8 (5 to 9)	0.3 (0.2 to 0.4)	34.8 (-14 to 102.3)	-11.9 (-39.7 to 27.7)	95.1 (26.3 to 199.9)	-0.3 (-35.1 to 47.6)
		Female	158 (99 to 262)	18 (12.3 to 26.8)	2 (1 to 3)	0.3 (0.2 to 0.4)	207 (154 to 265)	15.5 (11 to 20.2)	3 (2 to 4)	0.3 (0.2 to 0.4)	30.5 (-34.1 to 117.2)	-14 (-51.3 to 33.8)	81.8 (-2.8 to 227.3)	-1.5 (-39.8 to 64.8)
		Male	168 (108 to 264)	18.2 (12.5 to 25.9)	2 (1 to 3)	0.3 (0.2 to 0.5)	233 (168 to 301)	16.5 (11.7 to 21.5)	4 (3 to 6)	0.3 (0.2 to 0.4)	38.9 (-23.6 to 132.2)	-9.5 (-44.9 to 40.8)	106.7 (21 to 256.3)	2.7 (-36.1 to 67.4)
	Diphtheria	Both	63 (26 to 129)	2.7 (1.2 to 5.2)	1 (0 to 2)	0 (0 to 0.1)	2 (1 to 3)	0.1 (0 to 0.1)	0 (0 to 0)	0 (0 to 0)	-97.4 (-99 to -92.6)	-97.7 (-99.1 to -93.8)	-96.8 (-98.6 to -91.3)	-97.4 (-98.9 to -93.4)
		Female	27 (7 to 67)	2.3 (0.7 to 5.5)	0 (0 to 1)	0 (0 to 0.1)	1 (0 to 1)	0 (0 to 0.1)	0 (0 to 0)	0 (0 to 0)	-97.7 (-99.3 to -90.5)	-97.9 (-99.4 to -91.6)	-97.2 (-99.1 to -89.3)	-97.7 (-99.2 to -92)
		Male	36 (11 to 90)	3 (1 to 7)	0 (0 to 1)	0 (0 to 0.1)	1 (1 to 2)	0.1 (0 to 0.1)	0 (0 to 0)	0 (0 to 0)	-97.2 (-99.1 to -88.7)	-97.6 (-99.2 to -90.9)	-96.4 (-98.7 to -86.3)	-97.2 (-99 to -90.7)
	Whooping cough	Both	3883 (232 to 16077)	152.3 (9.1 to 632)	44 (2 to 185)	1.7 (0.1 to 7.3)	97 (10 to 433)	3.8 (0.4 to 16.7)	1 (0 to 5)	0 (0 to 0.2)	-97.5 (-99.8 to -55.9)	-97.5 (-99.8 to -56.5)	-97.6 (-99.9 to -40.5)	-97.6 (-99.9 to -41.5)
		Female	2195 (132 to 8866)	176.3 (10.6 to 714.3)	25 (1 to 102)	2 (0.1 to 8.2)	56 (5 to 248)	4.4 (0.4 to 19.8)	1 (0 to 3)	0 (0 to 0.2)	-97.5 (-99.8 to -55.4)	-97.5 (-99.8 to -55.7)	-97.6 (-99.9 to -42.6)	-97.6 (-99.9 to -43.2)
		Male	1688 (101 to 7393)	129.5 (7.7 to 568.1)	19 (1 to 85)	1.5 (0.1 to 6.5)	42 (4 to 192)	3.1 (0.3 to 14.5)	0 (0 to 2)	0 (0 to 0.2)	-97.5 (-99.8 to -54.5)	-97.6 (-99.8 to -55.4)	-97.6 (-99.9 to -36)	-97.7 (-99.9 to -37.6)
	Tetanus	Both	80 (25 to 211)	3.7 (1.5 to 9.1)	1 (0 to 3)	0.1 (0 to 0.2)	23 (19 to 33)	0.8 (0.7 to 1.2)	0 (0 to 1)	0 (0 to 0)	-71.1 (-90 to -3.9)	-77.6 (-91.3 to -41.3)	-59.1 (-84.7 to 9.6)	-76.5 (-89.7 to -47.6)
		Female	42 (11 to 125)	3.9 (1.5 to 10.9)	1 (0 to 1)	0.1 (0 to 0.1)	12 (9 to 14)	0.9 (0.7 to 1.1)	0 (0 to 0)	0 (0 to 0)	-72.6 (-90.8 to 8.6)	-78.3 (-92 to -36.6)	-60.4 (-86.1 to 20.2)	-76.2 (-89.7 to -45.7)
		Male	38 (11 to 114)	3.5 (1.2 to 10.7)	0 (0 to 2)	0.1 (0 to 0.2)	12 (9 to 16)	0.8 (0.6 to 1.1)	0 (0 to 0)	0 (0 to 0)	-69.6 (-90.1 to 6.7)	-76.8 (-91.9 to -32.6)	-57.7 (-85.1 to 23.2)	-76.2 (-90.3 to -30.1)
	Measles	Both	5799 (1804 to 13105)	227.9 (71.1 to 513.9)	67 (21 to 151)	2.6 (0.8 to 6)	112 (31 to 267)	4.4 (1.2 to 10.5)	1 (0 to 3)	0 (0 to 0.1)	-98.1 (-99.2 to -96.2)	-98.1 (-99.2 to -96.2)	-98.1 (-99.2 to -96.2)	-98.1 (-99.2 to -96.2)
		Female	2954 (916 to 6733)	237.8 (73.8 to 540.8)	34 (11 to 78)	2.8 (0.9 to 6.3)	58 (16 to 141)	4.7 (1.3 to 11.4)	1 (0 to 2)	0.1 (0 to 0.1)	-98 (-99.1 to -96.2)	-98 (-99.1 to -96.2)	-98.1 (-99.2 to -96.3)	-98.1 (-99.2 to -96.3)
		Male	2845 (949 to 6426)	218.4 (72.7 to 492.3)	33 (11 to 75)	2.5 (0.8 to 5.7)	55 (16 to 129)	4.2 (1.2 to 9.8)	1 (0 to 1)	0 (0 to 0.1)	-98.1 (-99.2 to -96.2)	-98.1 (-99.2 to -96.2)	-98.1 (-99.3 to -96.2)	-98.2 (-99.3 to -96.3)
	Varicella and herpes zoster	Both	136 (80 to 221)	8.3 (5.8 to 12)	2 (1 to 3)	0.2 (0.1 to 0.2)	110 (75 to 156)	4 (2.7 to 5.6)	1 (1 to 1)	0 (0 to 0.1)	-18.8 (-56.4 to 40.2)	-52 (-69.8 to -29.2)	-44.5 (-71 to -0.9)	-72.3 (-83.9 to -52.9)
		Female	67 (34 to 124)	8.4 (5.2 to 13.1)	1 (0 to 1)	0.2 (0.1 to 0.3)	54 (35 to 81)	4 (2.6 to 5.8)	0 (0 to 1)	0 (0 to 0.1)	-19.2 (-61.4 to 71.2)	-52.2 (-72.6 to -21.3)	-49 (-77.3 to 16.2)	-72.1 (-86.6 to -42.3)
		Male	69 (36 to 124)	8.1 (5.3 to 12.7)	1 (0 to 1)	0.2 (0.1 to 0.3)	56 (37 to 80)	3.9 (2.7 to 5.6)	0 (0 to 1)	0 (0 to 0.1)	-18.5 (-60 to 60.6)	-51.8 (-71.7 to -21.2)	-40.1 (-72.7 to 29.7)	-72.4 (-86.5 to -43.7)
	Acute hepatitis	Both	573 (368 to 822)	47.4 (30.6 to 71.3)	10 (6 to 16)	1.3 (0.8 to 2.2)	546 (436 to 676)	18.7 (15.1 to 23.2)	13 (10 to 17)	0.5 (0.4 to 0.7)	-4.8 (-36.8 to 50.8)	-60.6 (-75.2 to -38)	28.5 (-23.6 to 115.7)	-61.2 (-78.2 to -35.1)
		Female	255 (148 to 384)	41.2 (22.7 to 64.7)	4 (2 to 7)	1.1 (0.5 to 2)	163 (126 to 206)	12 (9.4 to 15.2)	3 (2 to 5)	0.3 (0.2 to 0.4)	-36.1 (-61.5 to 16.7)	-70.9 (-83.1 to -45.4)	-21.4 (-58.5 to 66.9)	-74.1 (-88 to -42.3)
		Male	318 (196 to 520)	52.5 (32.4 to 87.5)	6 (3 to 11)	1.5 (0.9 to 2.7)	383 (292 to 493)	25.2 (19.3 to 32.6)	10 (7 to 14)	0.7 (0.5 to 1)	20.4 (-29.2 to 108.3)	-52 (-73.1 to -16.6)	63.8 (-15.9 to 212.5)	-50.8 (-75.6 to -7.1)
	Other unspecified infectious diseases	Both	848 (533 to 1320)	48.4 (33 to 68.8)	10 (6 to 15)	1 (0.6 to 1.3)	814 (502 to 1037)	30.3 (18.3 to 38.8)	18 (8 to 23)	0.8 (0.4 to 1.1)	-4 (-48.3 to 60.4)	-37.4 (-62.3 to -4.4)	83.7 (-13.1 to 229.4)	-17.3 (-50.4 to 38.5)

Province	Cause	Sex	1990				2019				1990-2019 percent change (%)			
			DALYs		Death		DALYs		Death		DALYs		Death	
			Number	Rate	Number	Rate	Number	Rate	Number	Rate	Number	Rate	Number	Rate
		Female	334 (207 to 556)	41.8 (28.4 to 61.8)	4 (2 to 6)	0.9 (0.5 to 1.3)	329 (213 to 445)	25.6 (16.3 to 34.2)	7 (4 to 9)	0.7 (0.3 to 0.9)	-1.5 (-49.4 to 67.3)	-38.9 (-63.9 to -4)	81.6 (-13.9 to 242.6)	-24.8 (-54.3 to 38.1)
		Male	514 (276 to 913)	54.4 (33.6 to 86.2)	6 (3 to 11)	1.1 (0.6 to 1.5)	485 (255 to 656)	34.9 (18 to 47.7)	11 (4 to 16)	0.9 (0.3 to 1.4)	-5.7 (-54.8 to 93.1)	-35.9 (-66.3 to 12.6)	84.9 (-21.3 to 282)	-10.6 (-53.7 to 58.7)
	Maternal and neonatal disorders	Both	102798 (81225 to 127148)	4094.8 (3246.3 to 5044.3)	1138 (894 to 1416)	45.1 (35.5 to 56)	24029 (19892 to 28869)	892.2 (736.2 to 1076.3)	188 (148 to 239)	7.3 (5.7 to 9.2)	-76.6 (-83 to -68)	-78.2 (-84.2 to -70.1)	-83.4 (-88.6 to -76.5)	-83.9 (-88.9 to -77.1)
		Female	42980 (33455 to 53953)	3571.2 (2805.8 to 4452.3)	474 (365 to 597)	39.2 (30.4 to 49.3)	10778 (8877 to 12848)	811.5 (662.9 to 974.3)	82 (64 to 103)	6.5 (5 to 8.1)	-74.9 (-82.1 to -65.4)	-77.3 (-83.8 to -68.7)	-82.6 (-88.2 to -75.3)	-83.6 (-88.9 to -76.8)
		Male	59818 (46008 to 76268)	4592.5 (3535.4 to 5842)	663 (508 to 851)	50.6 (38.8 to 65)	13251 (10667 to 16090)	967.8 (778.6 to 1178.8)	106 (80 to 137)	8 (6.1 to 10.3)	-77.8 (-84.7 to -68.8)	-78.9 (-85.5 to -70.4)	-84 (-89.6 to -76.4)	-84.1 (-89.6 to -76.6)
	Maternal disorders	Female	1098 (543 to 1559)	156.8 (79.5 to 223.5)	14 (5 to 22)	2.2 (0.7 to 3.3)	517 (394 to 671)	29.6 (22.5 to 38.4)	6 (4 to 8)	0.3 (0.2 to 0.4)	-52.9 (-68.4 to 0.5)	-81.1 (-87.5 to -61.2)	-58.1 (-75.4 to 34)	-84.6 (-91.1 to -56.1)
	Neonatal disorders	Both	101700 (80342 to 126220)	4019.4 (3181.6 to 4974.9)	1123 (882 to 1404)	44 (34.6 to 55)	23512 (19425 to 28335)	877.5 (722.4 to 1059.3)	182 (142 to 232)	7.1 (5.5 to 9)	-76.9 (-83.3 to -68.3)	-78.2 (-84.2 to -69.9)	-83.8 (-88.9 to -77)	-83.9 (-89 to -77.1)
		Female	41882 (32508 to 52732)	3414.3 (2659.5 to 4284.3)	460 (352 to 582)	37.1 (28.4 to 46.9)	10261 (8355 to 12304)	781.9 (633.1 to 941.6)	76 (58 to 97)	6.1 (4.6 to 7.8)	-75.5 (-82.7 to -65.9)	-77.1 (-83.9 to -68)	-83.4 (-89 to -76)	-83.5 (-89 to -76.1)
		Male	59818 (46008 to 76268)	4592.5 (3535.4 to 5842)	663 (508 to 851)	50.6 (38.8 to 65)	13251 (10667 to 16090)	967.8 (778.6 to 1178.8)	106 (80 to 137)	8 (6.1 to 10.3)	-77.8 (-84.7 to -68.8)	-78.9 (-85.5 to -70.4)	-84 (-89.6 to -76.4)	-84.1 (-89.6 to -76.6)
	Nutritional deficiencies	Both	5166 (3318 to 7606)	289.6 (189.4 to 413.3)	10 (6 to 17)	0.8 (0.6 to 1.2)	3596 (2238 to 5355)	128.9 (80.6 to 197.5)	5 (4 to 6)	0.3 (0.2 to 0.3)	-30.4 (-49.8 to -0.9)	-55.5 (-66.4 to -39.7)	-52.9 (-71.5 to -15.9)	-65.8 (-76.3 to -49)
		Female	2572 (1617 to 3909)	318 (207.2 to 464.6)	4 (2 to 8)	0.8 (0.5 to 1.2)	1904 (1146 to 2902)	140.1 (85 to 216.4)	2 (2 to 3)	0.3 (0.3 to 0.4)	-26 (-51.4 to 10.8)	-55.9 (-69.2 to -37.2)	-46.8 (-73.8 to 8.6)	-56.7 (-75.2 to -26.8)
		Male	2594 (1537 to 4199)	262.6 (166.2 to 401.3)	6 (3 to 11)	0.9 (0.6 to 1.3)	1692 (979 to 2705)	118.8 (67.9 to 195)	3 (2 to 3)	0.3 (0.2 to 0.3)	-34.8 (-58.8 to 3.4)	-54.8 (-70.8 to -28.2)	-57.2 (-76.4 to -15.7)	-70.8 (-80.5 to -52.4)
	Protein-energy malnutrition	Both	1300 (872 to 1927)	62.8 (44.6 to 88.2)	10 (6 to 16)	0.8 (0.6 to 1.1)	932 (613 to 1355)	33.3 (22.2 to 47.6)	4 (3 to 5)	0.2 (0.2 to 0.3)	-28.3 (-54.6 to 7)	-47.1 (-64.7 to -24.9)	-59.5 (-75.8 to -27.8)	-69.5 (-78.9 to -54.2)
		Female	564 (356 to 916)	56.5 (36.8 to 84.9)	4 (2 to 8)	0.7 (0.4 to 1.2)	443 (287 to 659)	32.7 (21.4 to 47.9)	2 (2 to 2)	0.3 (0.2 to 0.4)	-21.6 (-56.8 to 27.4)	-42.2 (-65.8 to -13.5)	-53.4 (-77.3 to -4.1)	-60.6 (-77.4 to -33.1)
		Male	735 (471 to 1169)	68.8 (46.8 to 103.8)	6 (3 to 10)	0.8 (0.5 to 1.2)	489 (319 to 713)	33.9 (22.3 to 49.1)	2 (2 to 3)	0.2 (0.2 to 0.3)	-33.5 (-61.7 to 7)	-50.6 (-69.9 to -26.7)	-63.9 (-80.6 to -27.9)	-74.5 (-83.3 to -59)
	Iodine deficiency	Both	168 (98 to 263)	12.7 (7.8 to 19.6)	--	--	187 (107 to 304)	6 (3.4 to 9.7)	--	--	11.3 (-22.2 to 56.5)	-52.9 (-65.5 to -36)	--	--
		Female	92 (52 to 149)	14.7 (8.7 to 23.1)	--	--	116 (67 to 191)	7.5 (4.3 to 12.3)	--	--	25 (-21.5 to 90.3)	-48.6 (-65.9 to -24.2)	--	--
		Male	75 (42 to 117)	10.9 (6.4 to 16.7)	--	--	71 (40 to 114)	4.5 (2.6 to 7.2)	--	--	-5.6 (-33.2 to 36.4)	-58.8 (-70.2 to -44.4)	--	--
	Vitamin A deficiency	Both	138 (65 to 253)	5.5 (2.6 to 10)	--	--	10 (4 to 22)	0.4 (0.2 to 0.9)	--	--	-92.7 (-97.2 to -82.8)	-92.4 (-97.1 to -82.1)	--	--
		Female	52 (23 to 102)	4.2 (1.9 to 8.4)	--	--	4 (1 to 11)	0.4 (0.1 to 0.9)	--	--	-91.5 (-97.3 to -77.4)	-91.2 (-97.2 to -76.6)	--	--
		Male	86 (29 to 186)	6.7 (2.2 to 14.2)	--	--	6 (1 to 16)	0.5 (0.1 to 1.3)	--	--	-93.4 (-98.4 to -76.7)	-93.2 (-98.4 to -75.4)	--	--
	Dietary iron deficiency	Both	3409 (1825 to 5583)	197.8 (114.4 to 311.2)	--	--	2194 (1178 to 3674)	80.2 (41.6 to 136.5)	--	--	-35.6 (-59 to 4.7)	-59.5 (-73 to -37)	--	--
		Female	1799 (929 to 2994)	233.1 (134.7 to 362.6)	--	--	1211 (604 to 2080)	90.7 (45.9 to 156.1)	--	--	-32.7 (-62.1 to 17.5)	-61.1 (-76.6 to -37.1)	--	--

Province	Cause	Sex	1990				2019				1990-2019 percent change (%)			
			DALYs		Death		DALYs		Death		DALYs		Death	
			Number	Rate	Number	Rate	Number	Rate	Number	Rate	Number	Rate	Number	Rate
		Male	1610 (718 to 2989)	164.2 (81.3 to 288.4)	--	--	983 (424 to 1900)	70.5 (30.5 to 142.2)	--	--	-38.9 (-69.4 to 31.3)	-57.1 (-78.1 to -16.9)	--	--
	Other nutritional deficiencies	Both	152 (98 to 222)	10.8 (7 to 15.8)	0 (0 to 1)	0.1 (0 to 0.1)	273 (170 to 412)	9.1 (5.8 to 13.6)	1 (1 to 1)	0 (0 to 0.1)	79.9 (38.4 to 131.9)	-15.4 (-33.4 to 7.9)	96.6 (26.6 to 252.5)	-10.9 (-41.9 to 56.8)
		Female	65 (39 to 98)	9.4 (5.6 to 13.9)	0 (0 to 0)	0 (0 to 0.1)	130 (78 to 205)	8.8 (5.4 to 13.7)	0 (0 to 0)	0 (0 to 0.1)	100.6 (42.9 to 184.9)	-6.3 (-33.5 to 31.5)	131 (16.8 to 363.5)	19.5 (-45.1 to 161)
		Male	87 (54 to 132)	12 (7.6 to 18.3)	0 (0 to 0)	0.1 (0 to 0.1)	143 (87 to 222)	9.4 (5.8 to 14.4)	1 (0 to 1)	0.1 (0 to 0.1)	64.5 (12.5 to 133.7)	-21.8 (-45.7 to 10.6)	78.3 (2.9 to 249.7)	-26.5 (-55.1 to 42.6)
	**Non-communicable diseases**	Both	244286 (196143 to 286991)	24508.7 (20547.9 to 28282.4)	3581 (2868 to 4183)	699 (585.7 to 798)	529690 (439585 to 626858)	19188.5 (16206.4 to 22305.1)	10081 (8909 to 11352)	512 (456.8 to 571.5)	116.8 (86.3 to 159.3)	-21.7 (-31.1 to -9.2)	181.6 (128.7 to 265.1)	-26.8 (-38.8 to -9.6)
		Female	115108 (91670 to 138220)	24342.9 (20056 to 28763.1)	1548 (1183 to 1871)	666.9 (545.7 to 783.4)	259423 (210900 to 315764)	19380.6 (16045.4 to 23283.7)	4277 (3652 to 4971)	515.9 (447.7 to 591.3)	125.4 (90.8 to 172)	-20.4 (-30.7 to -5.9)	176.3 (116.7 to 281.8)	-22.7 (-37.4 to 0.6)
		Male	129178 (101595 to 154576)	24613.3 (20086.1 to 28668.6)	2033 (1568 to 2487)	725.8 (585 to 891.2)	270266 (227088 to 317355)	19178.1 (16269.9 to 22366.9)	5804 (4851 to 6865)	528.8 (446.9 to 621.1)	109.2 (72.3 to 162.3)	-22.1 (-35 to -4.5)	185.5 (116.3 to 300.2)	-27.2 (-43.3 to -3.6)
	Neoplasms	Both	19020 (14060 to 23225)	2356 (1756.1 to 2865.7)	535 (399 to 652)	95.3 (71.3 to 115)	59065 (50156 to 67836)	2181 (1859.7 to 2496.6)	2126 (1829 to 2446)	92.7 (80.1 to 106.3)	210.5 (141.5 to 334.4)	-7.4 (-27.1 to 33.4)	297.6 (212.4 to 475.7)	-2.8 (-23 to 37.5)
		Female	8690 (5785 to 11464)	2196 (1539.4 to 2865.2)	234 (163 to 305)	83.4 (58.1 to 105.8)	26061 (21340 to 31453)	1914.1 (1590.4 to 2283.9)	883 (728 to 1058)	78.4 (65.1 to 92.9)	199.9 (115.4 to 379.5)	-12.8 (-36.9 to 35.9)	278 (172.3 to 487.9)	-6 (-31.1 to 38.5)
		Male	10330 (7273 to 13596)	2521.8 (1758.3 to 3280)	301 (209 to 396)	107.7 (77.3 to 138.7)	33004 (26798 to 39762)	2439.4 (1994.5 to 2929.6)	1244 (1018 to 1499)	105.9 (87.3 to 127.4)	219.5 (128.3 to 386.4)	-3.3 (-30.1 to 48.1)	312.8 (197.4 to 537)	-1.7 (-28.3 to 43.6)
	Lip and oral cavity cancer	Both	118 (79 to 155)	17.6 (11.7 to 23.2)	4 (3 to 5)	0.8 (0.5 to 1)	482 (405 to 568)	17.8 (15.1 to 20.8)	18 (15 to 21)	0.8 (0.7 to 0.9)	309.6 (187.5 to 568.7)	0.9 (-28.7 to 60.7)	360.1 (224.2 to 641.6)	4.5 (-25.1 to 61.8)
		Female	46 (29 to 61)	14.2 (9.2 to 18.7)	1 (1 to 2)	0.6 (0.4 to 0.8)	213 (172 to 263)	16.6 (13.6 to 20.4)	8 (7 to 10)	0.8 (0.6 to 1)	367.8 (225.3 to 713.1)	17.4 (-17 to 96.3)	445.9 (284.8 to 823.7)	29 (-7.8 to 100.7)
		Male	72 (43 to 106)	20.7 (12.6 to 30.1)	2 (1 to 4)	0.9 (0.6 to 1.3)	269 (207 to 344)	19.1 (14.8 to 24.1)	10 (8 to 13)	0.8 (0.6 to 1)	272.8 (123.6 to 601.2)	-8 (-44.5 to 63.2)	308.1 (146.7 to 617.1)	-9.3 (-43.5 to 55.2)
	Nasopharynx cancer	Both	58 (40 to 77)	6.9 (4.9 to 9.1)	2 (1 to 2)	0.2 (0.2 to 0.3)	153 (122 to 197)	5.2 (4.2 to 6.8)	5 (4 to 6)	0.2 (0.1 to 0.2)	165 (97.5 to 312.7)	-24.7 (-44 to 16.4)	198.5 (120.2 to 355.6)	-24.9 (-44.4 to 12.9)
		Female	19 (11 to 28)	4.9 (2.9 to 7.1)	1 (0 to 1)	0.2 (0.1 to 0.3)	43 (32 to 57)	3.2 (2.5 to 4.2)	1 (1 to 2)	0.1 (0.1 to 0.2)	122 (48.6 to 309.1)	-35.3 (-55.9 to 17.6)	152.5 (66.3 to 352.4)	-34.5 (-57.6 to 12.9)
		Male	38 (25 to 53)	8.7 (5.8 to 11.8)	1 (1 to 1)	0.3 (0.2 to 0.4)	110 (83 to 147)	7.2 (5.5 to 9.6)	3 (3 to 5)	0.2 (0.2 to 0.3)	186.4 (94 to 380.7)	-16.7 (-42.7 to 33.2)	221.2 (119 to 410.8)	-17.7 (-43.7 to 23.4)
	Other pharynx cancer	Both	34 (24 to 46)	5.3 (3.7 to 7)	1 (1 to 2)	0.2 (0.2 to 0.3)	116 (95 to 144)	4.3 (3.5 to 5.3)	4 (4 to 5)	0.2 (0.2 to 0.2)	236 (139 to 451.5)	-19.8 (-42.3 to 25.9)	269.1 (162.6 to 472.6)	-16 (-38.7 to 27.1)
		Female	13 (8 to 19)	4.3 (2.6 to 5.9)	0 (0 to 1)	0.2 (0.1 to 0.2)	37 (29 to 48)	2.8 (2.2 to 3.5)	1 (1 to 2)	0.1 (0.1 to 0.2)	177.5 (80.2 to 421.6)	-34 (-56.5 to 18)	209.1 (104.6 to 465)	-27.2 (-52.2 to 24.6)

Province	Cause	Sex	1990				2019				1990-2019 percent change (%)			
			DALYs		Death		DALYs		Death		DALYs		Death	
			Number	Rate	Number	Rate	Number	Rate	Number	Rate	Number	Rate	Number	Rate
		Male	21 (13 to 30)	6.3 (4.1 to 8.9)	1 (0 to 1)	0.3 (0.2 to 0.4)	78 (60 to 103)	5.7 (4.3 to 7.3)	3 (2 to 4)	0.2 (0.2 to 0.3)	273.7 (141.1 to 546.4)	-9.5 (-41.3 to 49.1)	305.2 (161.9 to 574)	-9 (-39.4 to 47.1)
	Esophageal cancer	Both	530 (359 to 689)	90.1 (62.6 to 117.7)	20 (14 to 26)	4.3 (3.1 to 5.7)	1872 (1541 to 2472)	72.7 (60.3 to 93.9)	79 (66 to 101)	3.6 (3 to 4.5)	253.3 (151 to 444.5)	-19.3 (-42.6 to 22.5)	294.9 (181.7 to 493.7)	-15.7 (-40.6 to 22.7)
		Female	234 (134 to 333)	85.2 (51.7 to 121.4)	9 (5 to 13)	4.1 (2.7 to 6.2)	681 (530 to 883)	55.7 (43.9 to 71.4)	29 (23 to 38)	2.9 (2.3 to 3.7)	191.5 (87.4 to 465.1)	-34.6 (-56.6 to 16.6)	226.9 (116.7 to 482.1)	-29 (-54.5 to 14.5)
		Male	296 (184 to 415)	94.5 (62 to 131.4)	11 (7 to 15)	4.5 (3.1 to 6.2)	1191 (915 to 1709)	89.3 (68.8 to 127)	50 (39 to 70)	4.3 (3.3 to 5.9)	302.1 (157.9 to 588.6)	-5.5 (-38.4 to 55.2)	350.2 (193 to 636.3)	-4.1 (-36.9 to 50.2)
	Stomach cancer	Both	2588 (1747 to 3252)	420.9 (295.7 to 523.8)	94 (66 to 118)	19.5 (14.4 to 24.3)	7115 (5960 to 8393)	275.9 (232.6 to 324.9)	299 (251 to 351)	13.6 (11.5 to 16)	174.9 (99.9 to 323.8)	-34.5 (-51.5 to -1.4)	216.3 (132 to 382.4)	-30.3 (-47.5 to 0.1)
		Female	997 (605 to 1378)	330.4 (214 to 444.8)	35 (23 to 47)	15.1 (10.2 to 20.1)	2268 (1786 to 2820)	183.2 (145.9 to 226.3)	96 (76 to 118)	9.5 (7.7 to 11.7)	127.4 (44.4 to 328.9)	-44.6 (-62.8 to -6.1)	173.2 (82.8 to 368.3)	-36.8 (-57.2 to -2)
		Male	1591 (1006 to 2146)	504.6 (342.5 to 674.6)	59 (40 to 80)	23.9 (16.4 to 31.3)	4847 (3830 to 5980)	366.3 (293.7 to 452)	203 (162 to 251)	17.5 (14.2 to 21.7)	204.6 (107.2 to 409.6)	-27.4 (-49.6 to 16.7)	241.8 (138.4 to 453.5)	-26.6 (-48.4 to 13.4)
	Colon and rectum cancer	Both	1355 (905 to 1846)	207.6 (142.9 to 281)	46 (31 to 62)	9.1 (6.4 to 12.2)	6246 (5242 to 7349)	231.5 (195.2 to 270.7)	239 (202 to 280)	10.6 (8.9 to 12.3)	361 (222.1 to 656.8)	11.5 (-21.7 to 80.2)	421.8 (264.9 to 742.5)	15.9 (-17.9 to 76.4)
		Female	628 (385 to 919)	203 (133.8 to 292.5)	21 (14 to 31)	8.9 (6 to 12.5)	2495 (1993 to 3105)	194.4 (156 to 239.4)	98 (78 to 121)	9.5 (7.6 to 11.6)	297.5 (153.8 to 590.6)	-4.3 (-38 to 60.1)	364.1 (199.8 to 678.9)	6.6 (-28.2 to 74.2)
		Male	727 (446 to 1067)	211.8 (134.1 to 312.3)	25 (15 to 37)	9.3 (6.2 to 13.4)	3750 (2974 to 4631)	269.3 (213.8 to 330.7)	141 (112 to 174)	11.8 (9.3 to 14.5)	415.8 (220.2 to 831.8)	27.1 (-20.1 to 116.8)	471 (259.9 to 885.2)	26.1 (-20.6 to 103.7)
	Liver cancer	Both	430 (301 to 557)	69 (46.4 to 90.9)	16 (10 to 21)	3.2 (2.2 to 4.3)	1625 (1323 to 1983)	62.6 (51.2 to 76.3)	66 (54 to 81)	2.9 (2.4 to 3.6)	278.2 (161.2 to 502.5)	-9.3 (-36.7 to 44.9)	322.6 (188.3 to 583.1)	-9 (-37.3 to 44.9)
		Female	169 (107 to 242)	56.6 (35.2 to 86.1)	6 (4 to 9)	2.7 (1.7 to 4.3)	537 (413 to 688)	42.8 (33.2 to 54)	21 (17 to 27)	2.1 (1.6 to 2.6)	217 (94.8 to 455.1)	-24.4 (-56.4 to 35.4)	252.6 (100.1 to 528.1)	-23.8 (-58 to 32.8)
		Male	260 (169 to 365)	79.9 (53.3 to 112.1)	10 (6 to 14)	3.7 (2.6 to 5.1)	1088 (822 to 1414)	82.1 (62.4 to 107)	45 (34 to 58)	3.8 (2.9 to 4.9)	318 (159.9 to 601.9)	2.8 (-35.9 to 69.3)	367.3 (190.2 to 688.6)	2.5 (-35.7 to 64.1)
	Gallbladder and biliary tract cancer	Both	228 (121 to 303)	38 (22.8 to 49.2)	8 (5 to 11)	1.8 (1.2 to 2.3)	671 (468 to 801)	26 (18.5 to 30.9)	28 (20 to 34)	1.3 (0.9 to 1.5)	194.4 (111.4 to 396.8)	-31.7 (-49.7 to 10.3)	232.7 (145.5 to 437.6)	-27.9 (-46.2 to 8.7)
		Female	133 (60 to 188)	47.6 (24.7 to 65.6)	5 (3 to 7)	2.2 (1.3 to 3)	353 (215 to 451)	29 (18.1 to 36.7)	15 (10 to 19)	1.5 (1 to 1.9)	165.8 (72.5 to 387.7)	-39.1 (-58.8 to 7)	204.7 (105.9 to 435.6)	-31.8 (-52.7 to 8.1)
		Male	95 (51 to 138)	29.1 (17.4 to 40.7)	3 (2 to 5)	1.3 (0.9 to 1.8)	318 (214 to 399)	23.4 (16.1 to 29.1)	13 (9 to 16)	1.1 (0.8 to 1.4)	234.3 (121.1 to 465.5)	-19.5 (-45.4 to 30.6)	273.7 (152.7 to 507.8)	-17.6 (-43.1 to 27.6)
	Pancreatic cancer	Both	418 (288 to 566)	67.5 (47.9 to 90.3)	15 (11 to 20)	3 (2.2 to 4)	3265 (2729 to 3886)	123.3 (103.3 to 146.4)	132 (110 to 157)	5.8 (4.8 to 6.8)	681.3 (445.3 to 1139.3)	82.7 (28.5 to 180.7)	770.4 (511.9 to 1249.2)	92.2 (36.8 to 187.8)
		Female	153 (93 to 215)	54 (35.1 to 76.1)	6 (4 to 8)	2.5 (1.7 to 3.5)	1208 (969 to 1507)	96.2 (77.6 to 120.1)	51 (41 to 63)	4.8 (3.9 to 5.9)	691.2 (410.8 to 1271.3)	78.2 (16.4 to 191.9)	785.2 (476 to 1330.3)	94 (24.8 to 204.5)

Province	Cause	Sex	1990				2019				1990-2019 percent change (%)			
			DALYs		Death		DALYs		Death		DALYs		Death	
			Number	Rate	Number	Rate	Number	Rate	Number	Rate	Number	Rate	Number	Rate
		Male	265 (163 to 400)	79.6 (50.6 to 119.6)	9 (6 to 14)	3.5 (2.3 to 5.2)	2058 (1622 to 2587)	150.1 (118.7 to 187.9)	82 (64 to 102)	6.8 (5.3 to 8.4)	675.6 (376.3 to 1241.4)	88.6 (18.1 to 216)	761.4 (435.8 to 1358.9)	92.6 (21.8 to 213.4)
	Larynx cancer	Both	312 (198 to 421)	48.7 (31.6 to 64.9)	11 (7 to 14)	2 (1.4 to 2.7)	927 (745 to 1152)	34.3 (27.8 to 42.3)	35 (28 to 43)	1.5 (1.2 to 1.9)	197.3 (110.9 to 398.5)	-29.5 (-49.2 to 13.6)	227.4 (134.1 to 425.1)	-25.9 (-45.8 to 13)
		Female	58 (34 to 80)	18.4 (11.7 to 25)	2 (1 to 3)	0.7 (0.5 to 1)	164 (129 to 206)	12.8 (10.1 to 16)	6 (5 to 8)	0.6 (0.5 to 0.7)	184 (85.5 to 439.4)	-30.4 (-53.4 to 25.3)	235.4 (120.4 to 500.8)	-19.7 (-46 to 36.5)
		Male	254 (151 to 358)	76 (48.2 to 105)	9 (5 to 12)	3.3 (2.1 to 4.5)	762 (590 to 977)	55 (42.8 to 69.7)	29 (22 to 37)	2.4 (1.8 to 3)	200.3 (99.8 to 416.8)	-27.6 (-50.4 to 19.7)	225.7 (120.3 to 442.9)	-28 (-50.1 to 16.6)
	Tracheal, bronchus, and lung cancer	Both	1500 (993 to 2037)	233.2 (154.1 to 315.3)	53 (35 to 71)	10.2 (7 to 13.7)	6534 (5532 to 7710)	246.5 (208.9 to 290.9)	262 (221 to 309)	11.5 (9.7 to 13.5)	335.6 (202.9 to 604.1)	5.7 (-25.5 to 68.2)	398.1 (252.8 to 697.9)	12.7 (-19.8 to 75)
		Female	355 (210 to 550)	110.2 (66.9 to 168.1)	12 (7 to 18)	4.7 (3.1 to 6.9)	2000 (1592 to 2478)	153.9 (123.7 to 189)	78 (63 to 96)	7.3 (5.8 to 9)	464 (236.5 to 899.6)	39.7 (-15.3 to 139.1)	575.5 (314.8 to 1036.9)	57.3 (-3.2 to 157.7)
		Male	1146 (716 to 1629)	346 (219.2 to 489.4)	41 (26 to 58)	15.6 (10.3 to 21.7)	4535 (3645 to 5686)	336.5 (273.5 to 418.7)	183 (148 to 227)	15.5 (12.5 to 19)	295.8 (155.1 to 597.4)	-2.7 (-36.6 to 62.3)	347.9 (193.3 to 647.3)	-0.7 (-33.4 to 58.5)
	Malignant skin melanoma	Both	109 (62 to 160)	14.9 (8.6 to 21.1)	3 (2 to 5)	0.7 (0.4 to 0.9)	296 (208 to 428)	10.6 (7.3 to 14.9)	10 (7 to 14)	0.5 (0.3 to 0.6)	171.4 (69.6 to 406.7)	-28.8 (-54 to 18.8)	204 (98.8 to 393.5)	-32 (-53.3 to 5.7)
		Female	44 (23 to 71)	12.4 (6.7 to 18.8)	1 (1 to 2)	0.6 (0.3 to 0.8)	111 (76 to 187)	8.3 (5.7 to 13.5)	4 (2 to 6)	0.4 (0.2 to 0.5)	155.2 (37.2 to 429.7)	-33.2 (-60.7 to 20.4)	180 (66.6 to 400.2)	-34.4 (-56.7 to 3.3)
		Male	65 (34 to 99)	17.2 (8.4 to 26.4)	2 (1 to 3)	0.8 (0.3 to 1.2)	185 (112 to 279)	12.9 (7.5 to 18.7)	7 (4 to 9)	0.5 (0.3 to 0.7)	182.2 (71.9 to 415.5)	-25 (-54.7 to 35.1)	219.6 (96.8 to 456.5)	-30.2 (-57.1 to 25.7)
	Non-melanoma skin cancer	Both	43 (30 to 57)	7.8 (5.5 to 10.3)	2 (1 to 2)	0.4 (0.3 to 0.6)	225 (188 to 265)	9.1 (7.7 to 10.7)	10 (9 to 12)	0.5 (0.4 to 0.6)	425 (276.1 to 705.2)	17.1 (-15.5 to 76.1)	467.8 (306.1 to 758.4)	19 (-14.9 to 77.6)
		Female	10 (5 to 14)	3.7 (2.2 to 5.4)	0 (0 to 1)	0.2 (0.1 to 0.3)	62 (46 to 80)	5.3 (3.9 to 6.7)	3 (2 to 3)	0.3 (0.2 to 0.4)	551.5 (277.8 to 1078.7)	42.5 (-15.8 to 158.9)	567.9 (295.6 to 1124.9)	44.4 (-17.6 to 171.2)
		Male	33 (22 to 47)	11.5 (8.1 to 16)	1 (1 to 2)	0.6 (0.5 to 0.9)	162 (129 to 201)	12.7 (10.1 to 15.7)	7 (6 to 9)	0.7 (0.5 to 0.9)	388.5 (228.1 to 671.3)	9.6 (-27.1 to 72.3)	438.1 (260.6 to 740.2)	7.3 (-28.8 to 65.2)
	Breast cancer	Both	1498 (943 to 2154)	195.6 (127.9 to 280.4)	41 (27 to 60)	6.5 (4.2 to 9.6)	6187 (5024 to 7598)	195.7 (159 to 238.1)	180 (146 to 220)	6.5 (5.3 to 7.9)	313.1 (173.4 to 584.2)	0 (-35 to 68.8)	335.4 (180.2 to 654.5)	0.2 (-36.4 to 66.8)
		Female	1472 (912 to 2124)	411.2 (260.3 to 594)	41 (26 to 59)	13.4 (8.6 to 19.8)	6097 (4942 to 7508)	394.2 (319.8 to 480.4)	177 (143 to 217)	13.4 (10.9 to 16.2)	314.1 (173.7 to 597.6)	-4.1 (-38 to 65.1)	336.5 (179.8 to 675.9)	-0.4 (-36.9 to 65.9)
		Male	25 (14 to 39)	6.8 (3.9 to 10.2)	1 (0 to 1)	0.3 (0.2 to 0.4)	90 (64 to 121)	6.2 (4.4 to 8.3)	3 (2 to 4)	0.2 (0.2 to 0.3)	255.4 (101.4 to 583.6)	-8.6 (-47.2 to 72.9)	280 (114.3 to 615.9)	-11.5 (-49.6 to 62.8)
	Cervical cancer	Female	354 (172 to 503)	103.5 (51.9 to 143.6)	11 (5 to 15)	3.8 (2 to 5.3)	838 (486 to 1096)	59 (34.4 to 76.1)	29 (17 to 37)	2.4 (1.4 to 3.1)	136.7 (58.4 to 393.8)	-43 (-61.3 to 11.1)	171 (81.9 to 428.1)	-37 (-57 to 9)
	Uterine cancer	Female	78 (38 to 114)	24.9 (13.6 to 34.7)	2 (1 to 4)	1 (0.6 to 1.3)	476 (155 to 648)	34.6 (12.1 to 46.5)	16 (6 to 21)	1.4 (0.5 to 1.8)	506.9 (195.3 to 1046.4)	39.3 (-33.1 to 160.7)	540.3 (215.1 to 1096.8)	44.2 (-29 to 154)

Province	Cause	Sex	1990				2019				1990-2019 percent change (%)			
			DALYs		Death		DALYs		Death		DALYs		Death	
			Number	Rate	Number	Rate	Number	Rate	Number	Rate	Number	Rate	Number	Rate
	Ovarian cancer	Female	243 (97 to 516)	65.7 (29.1 to 137.6)	7 (3 to 14)	2.3 (1.1 to 4.5)	1501 (850 to 1981)	102.5 (59.1 to 134.3)	48 (28 to 63)	3.7 (2.2 to 4.8)	518.7 (145.2 to 1176.6)	56 (-36.4 to 202.4)	606 (190.5 to 1271)	65 (-29.1 to 216)
	Prostate cancer	Male	423 (273 to 589)	192 (128.7 to 264.6)	21 (14 to 29)	12.1 (8.3 to 16.5)	2372 (1706 to 2982)	215.9 (156.7 to 270.9)	126 (93 to 159)	13 (9.7 to 16.3)	460.4 (289.2 to 768.2)	12.4 (-20.5 to 68.9)	504.6 (319.7 to 824.5)	7.8 (-22.9 to 60.3)
	Testicular cancer	Male	68 (40 to 100)	8.4 (5 to 12.1)	1 (1 to 1)	0.2 (0.1 to 0.3)	230 (165 to 308)	14.5 (10.4 to 19.3)	4 (3 to 5)	0.3 (0.2 to 0.4)	239.7 (104.2 to 518)	71.9 (6.8 to 213)	288.2 (137.2 to 597.7)	53.5 (-4.9 to 158.7)
	Kidney cancer	Both	273 (185 to 383)	27.5 (18.3 to 37.1)	6 (4 to 9)	1 (0.6 to 1.4)	962 (806 to 1141)	35.4 (29.7 to 42)	33 (27 to 40)	1.4 (1.2 to 1.7)	252.5 (137.9 to 446)	28.7 (-9.4 to 105.5)	416.8 (257.2 to 739.5)	39 (-2.3 to 138.1)
		Female	103 (68 to 156)	21.6 (13.8 to 30.1)	2 (2 to 3)	0.8 (0.5 to 1.1)	298 (242 to 362)	22.7 (18.7 to 27.4)	10 (8 to 12)	0.9 (0.7 to 1.1)	188.1 (78.1 to 369.5)	5 (-29.7 to 70.9)	302.3 (164.2 to 573.1)	7.5 (-30.6 to 100.6)
		Male	170 (103 to 253)	32.7 (19.9 to 48)	4 (2 to 6)	1.2 (0.7 to 1.8)	664 (521 to 844)	47.7 (37.6 to 59.9)	24 (18 to 30)	1.9 (1.5 to 2.4)	291.8 (130.1 to 577)	45.9 (-9.8 to 161.3)	483.7 (259.9 to 940.8)	59.5 (0.2 to 192.1)
	Bladder cancer	Both	285 (193 to 375)	53.2 (37.5 to 68.9)	12 (8 to 15)	2.8 (2 to 3.6)	1405 (1164 to 1692)	58.7 (48.9 to 70.5)	62 (52 to 75)	3.1 (2.6 to 3.7)	393.4 (248.1 to 699.1)	10.3 (-21 to 70)	435.9 (282.9 to 732.9)	11.5 (-21 to 71)
		Female	73 (43 to 105)	29.6 (18.7 to 42.4)	3 (2 to 4)	1.6 (1 to 2.4)	286 (230 to 355)	26.6 (21.7 to 32.5)	14 (11 to 17)	1.6 (1.3 to 2)	293.4 (143.1 to 633.2)	-9.9 (-42.7 to 56.5)	353 (182.4 to 696.5)	-0.3 (-38 to 70.7)
		Male	212 (139 to 296)	75.2 (50.9 to 103)	9 (6 to 12)	3.9 (2.7 to 5.3)	1118 (883 to 1392)	89.5 (71 to 110.6)	48 (38 to 60)	4.4 (3.5 to 5.5)	427.7 (244.1 to 784.3)	18.9 (-21.8 to 95.5)	465.9 (269.8 to 844.4)	14 (-24.4 to 82.7)
	Brain and central nervous system cancer	Both	2125 (1132 to 3015)	165.5 (88.5 to 235.8)	40 (22 to 58)	4.6 (2.5 to 6.4)	4551 (2267 to 5860)	156.1 (76.8 to 199.8)	123 (59 to 161)	4.7 (2.2 to 6.1)	114.2 (19.7 to 213)	-5.7 (-43.1 to 37.8)	205.9 (78 to 346.4)	2.6 (-37.1 to 56.2)
		Female	849 (398 to 1598)	141.4 (66.8 to 268)	16 (8 to 31)	4 (2 to 7.3)	2002 (950 to 2621)	142.3 (67.4 to 186.9)	54 (25 to 71)	4.3 (2 to 5.7)	135.8 (5.7 to 391.1)	0.7 (-52.5 to 104.8)	233.5 (58 to 566.5)	9.5 (-44.7 to 108.3)
		Male	1276 (614 to 2116)	187.3 (88.9 to 303.4)	24 (12 to 39)	5.1 (2.5 to 8.2)	2550 (973 to 3596)	169.8 (64.6 to 238.7)	69 (25 to 99)	5 (1.8 to 7.2)	99.8 (6.2 to 234.7)	-9.4 (-47.9 to 49.4)	187.3 (58.3 to 375.8)	-1.2 (-41.4 to 65)
	Thyroid cancer	Both	102 (60 to 134)	14.4 (9 to 18.4)	3 (2 to 4)	0.6 (0.4 to 0.7)	428 (246 to 523)	15.2 (9.2 to 18.3)	13 (8 to 16)	0.6 (0.4 to 0.7)	322.1 (204.4 to 569.3)	5.4 (-22.1 to 63.7)	338 (217.6 to 555.8)	-2.3 (-30.4 to 42.6)
		Female	66 (33 to 93)	20.1 (10.4 to 27.3)	2 (1 to 3)	0.8 (0.5 to 1.1)	232 (121 to 306)	17.1 (9.5 to 22.1)	7 (4 to 9)	0.6 (0.4 to 0.8)	253.8 (132.7 to 574.6)	-14.9 (-41.4 to 52.4)	260.1 (147.8 to 515.9)	-18.5 (-43.8 to 31.4)
		Male	36 (22 to 52)	9.1 (5.7 to 13)	1 (1 to 2)	0.3 (0.2 to 0.5)	197 (118 to 254)	13.4 (8.4 to 17.1)	6 (4 to 8)	0.5 (0.3 to 0.6)	446.3 (243.8 to 806.5)	47.2 (-7.8 to 140.2)	482.8 (258.2 to 885.8)	39.2 (-13.6 to 130.2)
	Mesothelioma	Both	13 (8 to 22)	1.7 (1.1 to 2.7)	0 (0 to 1)	0.1 (0 to 0.1)	45 (34 to 57)	1.4 (1.1 to 1.8)	1 (1 to 2)	0.1 (0 to 0.1)	231.4 (96.8 to 501.9)	-18.4 (-48.5 to 44.1)	260.4 (127.3 to 537.9)	-14.9 (-42.8 to 40.7)
		Female	6 (2 to 12)	1.6 (0.7 to 3.2)	0 (0 to 0)	0.1 (0 to 0.1)	17 (10 to 26)	1.1 (0.7 to 1.6)	1 (0 to 1)	0 (0 to 0.1)	190.3 (26.8 to 654.6)	-31.8 (-67.7 to 57.8)	212.2 (51.7 to 611.8)	-29.3 (-62.7 to 44.2)

Province	Cause	Sex	1990				2019				1990-2019 percent change (%)			
			DALYs		Death		DALYs		Death		DALYs		Death	
			Number	Rate	Number	Rate	Number	Rate	Number	Rate	Number	Rate	Number	Rate
		Male	8 (5 to 12)	1.8 (1.1 to 2.9)	0 (0 to 0)	0.1 (0 to 0.1)	27 (21 to 36)	1.7 (1.3 to 2.3)	1 (1 to 1)	0.1 (0 to 0.1)	263.8 (119.3 to 564)	-6.6 (-41.6 to 67.6)	297.7 (148.3 to 608.5)	-3.3 (-36.9 to 64.1)
	Hodgkin lymphoma	Both	204 (116 to 271)	17.4 (11 to 22.5)	4 (3 to 5)	0.5 (0.3 to 0.6)	407 (268 to 515)	13.6 (8.8 to 17.2)	10 (6 to 12)	0.3 (0.2 to 0.4)	99.8 (44.9 to 217.7)	-22.1 (-45.6 to 19.5)	130.9 (63.5 to 261.6)	-29.8 (-52.5 to 4.5)
		Female	80 (38 to 120)	15 (7.9 to 21.8)	2 (1 to 2)	0.5 (0.3 to 0.6)	160 (93 to 224)	11.1 (6.3 to 15.5)	4 (2 to 5)	0.3 (0.2 to 0.4)	101.8 (35.8 to 293.6)	-26.1 (-50.2 to 34)	118.3 (51 to 292.5)	-38.8 (-58.3 to -1.1)
		Male	124 (70 to 173)	19.4 (11.5 to 27.6)	2 (1 to 3)	0.5 (0.3 to 0.8)	247 (149 to 325)	15.9 (9.6 to 20.8)	6 (4 to 8)	0.4 (0.2 to 0.5)	98.5 (35.1 to 217.9)	-17.8 (-47 to 36.1)	139.7 (56.9 to 300.4)	-20.5 (-51.3 to 34.8)
	Non-Hodgkin lymphoma	Both	523 (351 to 699)	54.8 (38.7 to 70.5)	13 (9 to 16)	1.9 (1.3 to 2.3)	1803 (1501 to 2104)	63 (52.7 to 73.3)	54 (45 to 63)	2.1 (1.8 to 2.5)	244.9 (144.4 to 459.9)	14.8 (-16.3 to 83.5)	320.9 (207.1 to 571.7)	12.9 (-15.9 to 72.5)
		Female	183 (105 to 284)	43 (25.1 to 62.7)	5 (3 to 7)	1.5 (0.9 to 2.1)	600 (472 to 742)	43.8 (34.7 to 53.7)	18 (15 to 23)	1.5 (1.2 to 1.9)	227.2 (92 to 520.6)	1.8 (-37.4 to 86.4)	288.9 (139.2 to 617)	0.1 (-34 to 88)
		Male	339 (227 to 450)	65.4 (43.9 to 86)	8 (5 to 11)	2.2 (1.5 to 2.9)	1203 (968 to 1483)	81.4 (65.6 to 100.1)	35 (28 to 44)	2.7 (2.1 to 3.3)	254.5 (149 to 508.1)	24.5 (-11.2 to 105.9)	339.7 (212.7 to 630.5)	21.8 (-12.8 to 89.9)
	Multiple myeloma	Both	144 (98 to 197)	22.9 (15.8 to 31.1)	5 (3 to 7)	1 (0.7 to 1.3)	660 (412 to 833)	24.2 (15.5 to 30.5)	25 (16 to 31)	1 (0.7 to 1.3)	358.1 (190.8 to 644.8)	5.6 (-32.6 to 66.1)	384.7 (212.9 to 662.8)	6.2 (-30.2 to 61.6)
		Female	66 (38 to 99)	22.9 (14.2 to 31.8)	2 (1 to 3)	1 (0.6 to 1.3)	256 (152 to 339)	19.2 (11.9 to 25.3)	10 (6 to 13)	0.8 (0.6 to 1.1)	286.2 (131.8 to 592.5)	-15.8 (-46.2 to 44.6)	301.8 (158.7 to 599)	-14.8 (-41.8 to 44.4)
		Male	78 (49 to 120)	22.9 (15 to 34.3)	3 (2 to 4)	1 (0.6 to 1.5)	404 (236 to 547)	29 (17.3 to 39.6)	15 (9 to 21)	1.2 (0.8 to 1.7)	419.3 (189.4 to 818.2)	26.7 (-29.7 to 116.8)	458.2 (209.9 to 859)	26.6 (-28.7 to 107.6)
	Leukemia	Both	3136 (2089 to 4489)	224.8 (153.2 to 297.2)	56 (38 to 75)	6.3 (4.4 to 7.9)	4057 (3286 to 4837)	147.9 (119.1 to 176.5)	111 (90 to 135)	4.6 (3.7 to 5.6)	29.4 (-14.5 to 105.2)	-34.2 (-53.1 to 3.1)	99.6 (40.7 to 213.5)	-27.1 (-45.1 to 9.9)
		Female	1496 (808 to 2226)	217.9 (126.6 to 306.3)	26 (15 to 37)	5.8 (3.6 to 7.8)	1598 (1236 to 1997)	119.5 (93.7 to 146.9)	42 (34 to 53)	3.6 (2.9 to 4.5)	6.8 (-32.6 to 121.2)	-45.2 (-63.1 to 2.5)	63.9 (9.4 to 208.6)	-37.8 (-55.5 to 0.9)
		Male	1640 (1006 to 2479)	231.8 (155.8 to 318.1)	30 (20 to 42)	6.8 (4.7 to 9.2)	2459 (1793 to 3147)	175.2 (128.2 to 223.7)	69 (51 to 90)	5.5 (4 to 7.3)	49.9 (-4.8 to 172.5)	-24.4 (-49.2 to 23.8)	130.5 (53.6 to 292.6)	-19.2 (-44.6 to 24)
	Other malignant neoplasms	Both	1788 (1256 to 2268)	153.6 (106.6 to 196.6)	37 (25 to 47)	5 (3.5 to 6.5)	3486 (2776 to 4104)	128.1 (101.6 to 150.1)	100 (79 to 118)	4.2 (3.3 to 5)	95 (48.7 to 183.7)	-16.6 (-36 to 29.5)	171.8 (107.6 to 316.3)	-16 (-36.5 to 33.4)
		Female	745 (503 to 959)	127.9 (88.7 to 164)	15 (10 to 19)	4 (2.7 to 5.2)	1468 (1192 to 1767)	111.6 (92 to 132.9)	40 (33 to 48)	3.6 (2.9 to 4.3)	97.2 (42.5 to 217.2)	-12.7 (-36 to 37.6)	173.7 (96.5 to 336.1)	-11.6 (-37 to 41.9)
		Male	1043 (655 to 1488)	177.3 (110.6 to 251.2)	22 (14 to 31)	6 (3.7 to 8.4)	2018 (1432 to 2516)	144.2 (102.4 to 179.8)	60 (41 to 76)	4.8 (3.3 to 6.2)	93.5 (34.3 to 208.1)	-18.7 (-43.6 to 33)	170.6 (87.4 to 344.3)	-18.9 (-44 to 36.6)
	Other neoplasms	Both	42 (30 to 53)	5.4 (3.6 to 6.9)	1 (1 to 1)	0.2 (0.1 to 0.3)	130 (106 to 169)	5 (4.1 to 6.6)	4 (3 to 6)	0.2 (0.1 to 0.3)	210.2 (137.8 to 352.5)	-6.2 (-29 to 40.2)	309.4 (186.9 to 585.9)	-7.8 (-36 to 56.2)
		Female	19 (12 to 26)	5 (2.9 to 7)	0 (0 to 1)	0.2 (0.1 to 0.3)	59 (47 to 74)	4.6 (3.7 to 5.8)	2 (1 to 2)	0.2 (0.1 to 0.2)	208.8 (110.6 to 402.4)	-7.6 (-37.7 to 70.2)	311.9 (150.2 to 821.1)	-8.9 (-45.7 to 138)
		Male	23 (16 to 31)	5.7 (3.8 to 7.9)	1 (0 to 1)	0.2 (0.1 to 0.3)	71 (54 to 104)	5.4 (4.1 to 8)	2 (2 to 4)	0.2 (0.1 to 0.3)	211.4 (117.2 to 371.7)	-4 (-32 to 37)	307.6 (161.1 to 533.8)	-6.1 (-38.7 to 40.4)

Province	Cause	Sex	1990				2019				1990-2019 percent change (%)			
			DALYs		Death		DALYs		Death		DALYs		Death	
			Number	Rate	Number	Rate	Number	Rate	Number	Rate	Number	Rate	Number	Rate
	Cardiovascular diseases	Both	50841 (40658 to 60013)	8488.7 (6887.6 to 9965)	1840 (1466 to 2179)	449.7 (374.1 to 523.2)	120662 (105570 to 138592)	5151.3 (4514.9 to 5829.3)	5463 (4752 to 6220)	292.9 (254.7 to 330)	137.3 (86.6 to 210.8)	-39.3 (-50.9 to -20.6)	197 (136.3 to 294.2)	-34.9 (-46.3 to -18.8)
		Female	20584 (15386 to 25361)	7687.7 (6076.6 to 9280)	796 (611 to 968)	437.2 (348.5 to 521.7)	46844 (39846 to 54675)	4689.3 (4000.6 to 5433.9)	2341 (1982 to 2742)	312.6 (264 to 360.9)	127.6 (77.3 to 220.2)	-39 (-51.4 to -16.6)	194.1 (130.8 to 311.3)	-28.5 (-42.3 to -5.2)
		Male	30256 (22733 to 37959)	9130.1 (6877.5 to 11461.1)	1044 (770 to 1320)	457.5 (358.5 to 563)	73818 (60732 to 88720)	5741.1 (4774.4 to 6844.9)	3122 (2572 to 3747)	291.9 (242.3 to 347)	144 (81.7 to 245.3)	-37.1 (-52.8 to -13.4)	199.2 (122.7 to 317.5)	-36.2 (-51.5 to -14.7)
	Rheumatic heart disease	Both	912 (662 to 1216)	73 (53.9 to 97)	12 (8 to 17)	1.6 (1.1 to 2.5)	1508 (1143 to 1962)	50.3 (39.1 to 64.3)	23 (19 to 27)	1 (0.8 to 1.2)	65.3 (24.2 to 116.9)	-31.1 (-49.9 to -10.5)	94.5 (17.5 to 205.3)	-37.1 (-65.7 to -2.8)
		Female	476 (306 to 661)	81.2 (52.8 to 107.4)	6 (3 to 9)	1.9 (1.1 to 3)	755 (556 to 1005)	52.5 (39.6 to 68.3)	11 (9 to 15)	1.2 (0.9 to 1.5)	58.8 (11.1 to 143.6)	-35.4 (-53.3 to -0.4)	79.6 (10.6 to 278.5)	-38.1 (-66.7 to 21.7)
		Male	437 (281 to 645)	65.4 (42.7 to 94.8)	5 (3 to 9)	1.4 (0.8 to 2.3)	753 (566 to 978)	48.7 (37 to 62.6)	11 (9 to 15)	0.9 (0.7 to 1.2)	72.4 (13.3 to 156.1)	-25.5 (-50.4 to 9.2)	112.3 (9 to 313.8)	-31.2 (-65.5 to 31.5)
	Ischemic heart disease	Both	27889 (20726 to 34493)	5023.4 (4005.2 to 6057.1)	1127 (895 to 1363)	269.9 (218.8 to 320.7)	76864 (66089 to 89228)	3255.5 (2814.8 to 3731.5)	3677 (3177 to 4203)	194.4 (168.6 to 220.6)	175.6 (113.6 to 285.1)	-35.2 (-48.3 to -13.3)	226.4 (158.3 to 338.9)	-28 (-41.4 to -8.9)
		Female	9820 (6867 to 12578)	4081.4 (3025.4 to 5092.8)	440 (323 to 550)	242.5 (184 to 299)	26297 (22109 to 31181)	2715.5 (2294.2 to 3184.1)	1477 (1246 to 1735)	198.6 (167 to 231.3)	167.8 (100.4 to 311.5)	-33.5 (-48.8 to -5.2)	235.4 (159.5 to 377.5)	-18.1 (-35.7 to 12.4)
		Male	18069 (12636 to 23670)	5823.3 (4352.2 to 7465.2)	686 (505 to 881)	293.5 (227 to 367.2)	50567 (41165 to 61171)	3868 (3171.2 to 4651.2)	2200 (1803 to 2654)	201.8 (166.6 to 240.8)	179.9 (99.2 to 330.4)	-33.6 (-51.3 to -5.2)	220.6 (132.8 to 362.2)	-31.2 (-48.2 to -6.7)
	Stroke	Both	13078 (10311 to 15940)	2323.8 (1859.8 to 2866.3)	474 (370 to 596)	128.6 (100.6 to 157.1)	27171 (23708 to 30794)	1192.6 (1047.5 to 1356)	1163 (1004 to 1339)	64.6 (55.4 to 74.3)	107.8 (63.2 to 171.8)	-48.7 (-59.9 to -31.7)	145.1 (87.5 to 236.5)	-49.8 (-61.2 to -33.8)
		Female	6402 (4833 to 8094)	2467.5 (1872.7 to 3087.3)	241 (172 to 310)	139.5 (104.3 to 174.1)	13063 (11039 to 15174)	1266.3 (1071.5 to 1465.5)	564 (468 to 667)	74.5 (61.4 to 88.2)	104.1 (54.9 to 185.4)	-48.7 (-61.5 to -28.6)	134.3 (70.8 to 241.7)	-46.6 (-60.2 to -23.8)
		Male	6677 (4823 to 8674)	2172.3 (1572.9 to 2853.6)	234 (163 to 313)	117.2 (83.8 to 156.2)	14107 (11678 to 16837)	1154.8 (954.4 to 1379.7)	598 (483 to 729)	59.2 (47.4 to 71.9)	111.3 (52.1 to 204.2)	-46.8 (-61.8 to -25.4)	156.2 (78.7 to 273)	-49.5 (-64 to -28.3)
	Hypertensive heart disease	Both	2619 (1747 to 3581)	537.5 (376 to 722.8)	114 (78 to 158)	31.2 (21.7 to 43.5)	7555 (6303 to 10239)	343 (285.9 to 448)	375 (309 to 493)	20.9 (17.1 to 26.5)	188.5 (113.2 to 352)	-36.2 (-53.4 to -4.2)	227.8 (136.3 to 401.8)	-33 (-54.5 to 0.9)
		Female	1255 (779 to 1916)	563.3 (355.4 to 901.1)	59 (36 to 96)	34.4 (21.5 to 57.1)	3479 (2719 to 4624)	362 (284.1 to 462.5)	188 (146 to 248)	25.4 (19.5 to 32.5)	177.1 (67.3 to 371.6)	-35.7 (-64.4 to 6.9)	220.8 (72.4 to 457)	-26.3 (-62.1 to 22.2)
		Male	1363 (852 to 1944)	507.8 (333.5 to 718.8)	56 (34 to 80)	27.9 (17.7 to 39.8)	4076 (3185 to 5555)	337.4 (266.2 to 447.9)	187 (144 to 255)	18.3 (14.2 to 25)	199 (106 to 394.2)	-33.6 (-53.6 to 3.4)	235.2 (126.8 to 444.8)	-34.2 (-54.9 to 3.6)
	Non-rheumatic valvular heart disease	Both	177 (116 to 264)	25.4 (18.2 to 34.5)	6 (4 to 8)	1.2 (0.9 to 1.6)	556 (459 to 769)	21 (17.4 to 29.1)	21 (18 to 30)	1 (0.8 to 1.4)	214.3 (124.2 to 402.9)	-17.5 (-39.3 to 21.5)	275.4 (174.1 to 458.9)	-16.4 (-39 to 14.2)
		Female	81 (48 to 119)	26.5 (17.7 to 35.5)	3 (2 to 4)	1.3 (0.9 to 1.9)	243 (192 to 302)	20.3 (16.2 to 25.4)	10 (8 to 13)	1.1 (0.9 to 1.4)	199.7 (91.1 to 458.8)	-23.3 (-47.1 to 25.3)	258.4 (145.1 to 482.2)	-17 (-45.7 to 25.3)
		Male	96 (58 to 157)	24 (15.4 to 39.1)	3 (2 to 5)	1 (0.7 to 1.7)	314 (241 to 515)	22.1 (16.9 to 37.2)	11 (8 to 20)	0.9 (0.7 to 1.7)	226.5 (102.6 to 470.7)	-8.2 (-41.8 to 49)	292.2 (145.6 to 549.5)	-8.6 (-40 to 39.2)

Province	Cause	Sex	1990				2019				1990-2019 percent change (%)			
			DALYs		Death		DALYs		Death		DALYs		Death	
			Number	Rate	Number	Rate	Number	Rate	Number	Rate	Number	Rate	Number	Rate
	Cardiomyopathy and myocarditis	Both	1093 (659 to 1721)	76 (52.9 to 105.3)	19 (13 to 27)	2.5 (1.8 to 3.4)	1028 (750 to 1758)	39.5 (29.3 to 66.8)	31 (23 to 55)	1.5 (1.1 to 2.5)	-5.9 (-50.7 to 106.7)	-48 (-68.4 to -5.8)	64.4 (-3.1 to 216)	-41.9 (-62.9 to -3.6)
		Female	479 (249 to 881)	70.4 (43.8 to 106.7)	9 (5 to 13)	2.6 (1.5 to 4)	365 (265 to 596)	30.6 (22.6 to 48.1)	12 (8 to 19)	1.3 (0.9 to 1.9)	-23.9 (-62.3 to 87.1)	-56.5 (-73.7 to -12.2)	35.8 (-22.6 to 194.4)	-50.5 (-72.9 to -11.1)
		Male	613 (340 to 1106)	80.4 (53.2 to 124)	10 (7 to 16)	2.4 (1.7 to 3.6)	663 (443 to 1254)	48.4 (32.3 to 92.8)	19 (13 to 39)	1.6 (1.1 to 3.4)	8.1 (-51.6 to 143.9)	-39.8 (-68.1 to 10.3)	88.2 (-4.7 to 272.9)	-31.1 (-59.7 to 14.4)
	Atrial fibrillation and flutter	Both	308 (232 to 397)	79.4 (59.8 to 101)	9 (6 to 12)	3.4 (2.2 to 4.5)	1539 (1178 to 1944)	77.5 (60.6 to 97)	50 (42 to 59)	3.4 (2.7 to 3.9)	399.9 (333 to 481.8)	-2.5 (-17 to 18.1)	439.9 (300.9 to 713.5)	-1.7 (-27.2 to 54.2)
		Female	169 (122 to 220)	94.7 (66.4 to 123.2)	6 (4 to 8)	4.7 (2.6 to 6.4)	812 (630 to 1041)	91.4 (73.4 to 114.5)	29 (23 to 35)	4.6 (3.7 to 5.6)	379.6 (294.7 to 521.7)	-3.5 (-22.6 to 33.8)	367.5 (224.1 to 783.6)	-0.4 (-32.8 to 100.8)
		Male	139 (100 to 185)	63.4 (47.1 to 83.4)	3 (2 to 4)	2.2 (1.4 to 3.1)	727 (538 to 946)	67.2 (50.3 to 86.5)	21 (14 to 27)	2.5 (1.6 to 3.2)	424.8 (336.2 to 529.1)	6.1 (-12.7 to 26.7)	582.5 (351.8 to 901.3)	16.5 (-20.9 to 69)
	Aortic aneurysm	Both	177 (107 to 246)	27.6 (18.6 to 36.8)	6 (4 to 8)	1.4 (1 to 1.8)	585 (481 to 700)	22.8 (19 to 27.2)	25 (21 to 29)	1.2 (1 to 1.4)	231.2 (122.9 to 491.9)	-17.4 (-43.3 to 32.1)	289.2 (167.7 to 521)	-15.1 (-40 to 26.9)
		Female	48 (30 to 70)	17.2 (11.8 to 23.6)	2 (1 to 3)	1 (0.7 to 1.3)	148 (118 to 182)	12.4 (10.1 to 15)	6 (5 to 8)	0.7 (0.6 to 0.8)	209.2 (97.9 to 429.4)	-28 (-49.9 to 10.4)	240.1 (136.3 to 427.2)	-27.9 (-49.8 to 5.5)
		Male	129 (71 to 195)	37 (22 to 54.5)	4 (3 to 7)	1.8 (1.1 to 2.5)	436 (342 to 545)	32.7 (25.6 to 40.7)	18 (14 to 23)	1.6 (1.3 to 2)	239.4 (99.2 to 562.9)	-11.5 (-46.3 to 61.8)	310.3 (150.2 to 657.2)	-9.7 (-43.7 to 62.2)
	Peripheral artery disease	Both	29 (16 to 47)	7.1 (4.2 to 11.5)	0 (0 to 1)	0.1 (0.1 to 0.2)	162 (106 to 247)	7.8 (5.1 to 11.8)	4 (3 to 4)	0.2 (0.2 to 0.3)	466 (386.8 to 602.2)	10.2 (-4.2 to 34)	799.3 (514.7 to 1287.3)	59.7 (8.5 to 148.5)
		Female	16 (9 to 27)	8.2 (4.6 to 13.6)	0 (0 to 0)	0.1 (0.1 to 0.2)	79 (47 to 127)	8.3 (5 to 13.3)	1 (1 to 2)	0.2 (0.1 to 0.3)	401.3 (324.6 to 509.7)	0.9 (-15 to 25.1)	627.6 (297.3 to 1549.9)	46.6 (-19.6 to 253.5)
		Male	13 (8 to 21)	6 (3.7 to 9.4)	0 (0 to 0)	0.1 (0.1 to 0.2)	83 (58 to 119)	7.6 (5.3 to 11)	2 (2 to 3)	0.2 (0.2 to 0.3)	545 (407.2 to 782.9)	25.4 (1.4 to 65.5)	947.1 (550.5 to 1704.5)	78.8 (10.4 to 206.3)
	Endocarditis	Both	236 (151 to 351)	18.1 (12.1 to 27.1)	4 (3 to 7)	0.6 (0.4 to 1.1)	310 (216 to 586)	11.3 (8 to 20.8)	10 (7 to 20)	0.5 (0.3 to 0.8)	31.7 (-29.2 to 173.7)	-37.9 (-58.1 to -4.3)	128 (46.2 to 268.1)	-25.9 (-44.4 to 0.1)
		Female	117 (60 to 203)	18.2 (11.4 to 28.9)	2 (1 to 3)	0.6 (0.4 to 1.3)	130 (87 to 273)	10.7 (7.4 to 21.6)	5 (3 to 10)	0.5 (0.3 to 1)	11.7 (-50.5 to 216.2)	-41.1 (-64.7 to -4.7)	114.9 (18.1 to 286.9)	-21.9 (-46.8 to 17.2)
		Male	119 (66 to 200)	18 (10.9 to 30.3)	2 (1 to 4)	0.6 (0.3 to 1)	180 (113 to 338)	12.1 (7.8 to 22.6)	5 (3 to 11)	0.4 (0.3 to 0.8)	51.1 (-26.5 to 207.4)	-32.9 (-59.2 to 11.4)	140.6 (40.6 to 318.6)	-24.4 (-47.5 to 9.5)
	Other cardiovascular and circulatory diseases	Both	4324 (3150 to 5665)	297.3 (229.8 to 379.2)	67 (51 to 88)	9.1 (7 to 13.4)	3383 (2745 to 4426)	130 (107.6 to 165.6)	85 (70 to 118)	4.3 (3.5 to 5.8)	-21.8 (-43.5 to 23.7)	-56.3 (-68.3 to -38.3)	26 (-13.7 to 104.9)	-53.1 (-72 to -31.1)
		Female	1722 (1132 to 2525)	259 (192.8 to 355.5)	28 (20 to 40)	8.6 (6.2 to 14.7)	1472 (1171 to 1868)	119.2 (98.1 to 147)	37 (30 to 47)	4.5 (3.6 to 5.5)	-14.5 (-45.5 to 37.4)	-54 (-69.3 to -35.1)	33.3 (-16.7 to 102.3)	-47.8 (-73 to -22.1)
		Male	2602 (1775 to 3617)	332.4 (248.1 to 435.1)	39 (28 to 53)	9.5 (7.1 to 13.2)	1912 (1520 to 2765)	142.1 (114 to 200.9)	47 (37 to 79)	4.3 (3.3 to 7)	-26.5 (-51.3 to 37)	-57.2 (-70.1 to -32.5)	20.8 (-22.9 to 131.6)	-55.3 (-72 to -27.1)
	Chronic respiratory diseases	Both	7528 (6030 to 9108)	954 (779.1 to 1141.1)	140 (105 to 181)	33.6 (25.5 to 44)	17262 (14826 to 19896)	691.3 (600 to 793)	429 (364 to 506)	23.1 (19.5 to 27.4)	129.3 (94.7 to 179.2)	-27.5 (-40.9 to -11)	206.3 (125.9 to 336.1)	-31.4 (-49.1 to -7)
		Female	3264 (2320 to 4127)	857.1 (612.6 to 1075.4)	57 (33 to 78)	29 (17.8 to 41.1)	7073 (5956 to 8441)	597.3 (506.2 to 713)	156 (124 to 206)	19.6 (15.5 to 25.2)	116.7 (77 to 199.9)	-30.3 (-45.1 to -0.8)	175.9 (82.7 to 433.2)	-32.2 (-56.1 to 19)
		Male	4264 (3296 to 5345)	1043 (818.7 to 1312.5)	83 (59 to 115)	38.1 (28.2 to 52.7)	10189 (8553 to 11974)	785.4 (660.1 to 918.3)	273 (216 to 337)	26.4 (21.1 to 32.7)	139 (88.4 to 203.1)	-24.7 (-43.4 to -3.9)	226.9 (113.5 to 383.1)	-30.8 (-53.7 to -1.9)

Province	Cause	Sex	1990				2019				1990-2019 percent change (%)			
			DALYs		Death		DALYs		Death		DALYs		Death	
			Number	Rate	Number	Rate	Number	Rate	Number	Rate	Number	Rate	Number	Rate
	Chronic obstructive pulmonary disease	Both	3815 (3066 to 4557)	600.1 (487.5 to 723.3)	96 (72 to 123)	23.9 (18.5 to 30.6)	11321 (9861 to 12808)	471 (411 to 536.8)	345 (292 to 412)	18.9 (15.9 to 22.6)	196.8 (139.2 to 276.8)	-21.5 (-37.1 to -1.2)	260.1 (164.3 to 408.6)	-21 (-41.1 to 7)
		Female	1526 (1066 to 1928)	501.8 (353 to 641.8)	35 (19 to 50)	19.1 (11.2 to 27.4)	4288 (3651 to 5139)	385.4 (329.2 to 463)	121 (95 to 161)	15.6 (12.3 to 20.3)	180.9 (115.6 to 326.5)	-23.2 (-40.6 to 11.6)	240.4 (127.7 to 558.3)	-17.9 (-44.8 to 49.9)
		Male	2288 (1752 to 2980)	690.5 (529.8 to 907.4)	60 (42 to 87)	28.7 (21.2 to 41)	7033 (5905 to 8214)	556.1 (468.2 to 651)	224 (177 to 280)	22 (17.4 to 27.5)	207.3 (120.6 to 318.4)	-19.5 (-42.9 to 7.2)	271.6 (133.5 to 457.4)	-23.2 (-50.3 to 9.5)
	Pneumoconiosis	Both	25 (15 to 36)	3.4 (2.2 to 4.9)	1 (0 to 1)	0.1 (0.1 to 0.2)	81 (63 to 102)	3.1 (2.4 to 3.9)	2 (2 to 3)	0.1 (0.1 to 0.1)	229.9 (111 to 483.1)	-9.8 (-42.3 to 51.9)	236.5 (98.2 to 559.5)	-17.4 (-51.3 to 52.9)
		Female	6 (4 to 8)	1.3 (0.9 to 1.8)	0 (0 to 0)	0 (0 to 0)	29 (20 to 41)	2.1 (1.5 to 2.8)	0 (0 to 0)	0 (0 to 0)	417.1 (305.7 to 555.6)	53.3 (21.5 to 91.4)	398.9 (187.1 to 989.5)	37.5 (-23.4 to 164)
		Male	19 (10 to 29)	5.3 (3 to 8.1)	1 (0 to 1)	0.2 (0.1 to 0.4)	52 (39 to 69)	4 (3 to 5.3)	2 (1 to 3)	0.2 (0.1 to 0.2)	174.3 (55 to 496.7)	-24.9 (-56.5 to 46.5)	222.4 (79.9 to 553.2)	-26.1 (-59.7 to 51.2)
	Asthma	Both	3399 (2479 to 4642)	324 (240.5 to 418.4)	40 (27 to 55)	8.9 (5.8 to 13.6)	4744 (3504 to 6459)	178.6 (132.8 to 242.5)	63 (51 to 78)	3.2 (2.6 to 4)	39.6 (20.7 to 64.2)	-44.9 (-57.1 to -31.4)	57.9 (4.2 to 147.6)	-64 (-78.5 to -44)
		Female	1594 (1103 to 2177)	328.1 (229.1 to 462.5)	20 (11 to 32)	9.3 (5 to 16.9)	2266 (1635 to 3113)	175.3 (127.1 to 239.3)	29 (20 to 40)	3.3 (2.3 to 4.5)	42.2 (15.1 to 76.7)	-46.6 (-62.4 to -29.7)	46.9 (-19.1 to 184.6)	-64.2 (-82.6 to -36.6)
		Male	1805 (1271 to 2497)	320 (235.5 to 422.8)	21 (13 to 29)	8.5 (5.5 to 12.2)	2477 (1815 to 3400)	182.9 (134.7 to 248.8)	35 (26 to 45)	3.2 (2.4 to 4.2)	37.3 (14.4 to 67.9)	-42.9 (-57.1 to -26.6)	68.4 (3.1 to 191.8)	-62.5 (-77.8 to -36.8)
	Interstitial lung disease and pulmonary sarcoidosis	Both	93 (67 to 122)	13.1 (9.3 to 17.3)	2 (1 to 3)	0.5 (0.3 to 0.7)	404 (272 to 519)	14.9 (10.3 to 19)	12 (7 to 16)	0.6 (0.3 to 0.7)	332.8 (219.5 to 512.9)	14.4 (-18.2 to 70.5)	428.4 (243.9 to 799.1)	12.5 (-30 to 98.1)
		Female	42 (28 to 60)	12.5 (7.9 to 18.8)	1 (1 to 2)	0.5 (0.2 to 0.8)	166 (116 to 224)	12.4 (8.4 to 16.6)	4 (3 to 6)	0.4 (0.2 to 0.6)	292.9 (179.4 to 491)	-0.6 (-32.4 to 62.4)	328.9 (164.2 to 790)	-8.4 (-45.7 to 96.8)
		Male	51 (34 to 71)	13.6 (9.1 to 19)	1 (1 to 2)	0.5 (0.3 to 0.9)	238 (129 to 326)	17.3 (9.6 to 23.4)	8 (3 to 11)	0.7 (0.3 to 1)	365.8 (187.6 to 650.4)	27.4 (-22.4 to 113.1)	509.7 (239.2 to 1063.2)	28.6 (-29.2 to 157.6)
	Other chronic respiratory diseases	Both	197 (148 to 289)	13.5 (10.5 to 17.8)	1 (1 to 2)	0.1 (0.1 to 0.2)	712 (552 to 875)	23.7 (18 to 29.3)	6 (2 to 9)	0.3 (0.1 to 0.4)	262 (120.7 to 385.8)	75.2 (16.9 to 134.4)	478.7 (44.3 to 1434.4)	106.2 (-33.2 to 380.4)
		Female	96 (69 to 152)	13.4 (10 to 19.3)	0 (0 to 1)	0.1 (0.1 to 0.2)	324 (257 to 395)	22.2 (17.3 to 27.3)	2 (1 to 3)	0.2 (0.1 to 0.3)	237 (110.1 to 359.2)	65.3 (12.6 to 122.2)	363.1 (16.3 to 1332.5)	67.8 (-42.7 to 364.2)
		Male	101 (74 to 139)	13.5 (10.4 to 17.8)	1 (0 to 1)	0.1 (0.1 to 0.3)	388 (274 to 498)	25.1 (17.3 to 32.4)	4 (1 to 7)	0.3 (0.1 to 0.5)	285.8 (129 to 460.8)	85.4 (19.6 to 163.3)	556.7 (59.6 to 1749.7)	133 (-32.5 to 529.8)
	Digestive diseases	Both	7729 (5742 to 9662)	791.2 (603.9 to 964.2)	145 (102 to 181)	26.8 (19.9 to 35.5)	14147 (11699 to 17695)	505.2 (425.6 to 614.9)	336 (283 to 396)	16.1 (13.6 to 18.9)	83 (40.9 to 150.1)	-36.2 (-48.9 to -14.5)	131.2 (70.9 to 244.4)	-39.9 (-57.4 to -13.6)
		Female	3406 (2488 to 4440)	745.1 (568.3 to 931)	62 (42 to 80)	26.2 (17.8 to 37.7)	6197 (4921 to 7924)	466.5 (377.1 to 582.8)	132 (109 to 160)	15.3 (12.7 to 18.5)	81.9 (34.5 to 154.9)	-37.4 (-50.4 to -15.5)	113 (54.2 to 232.1)	-41.5 (-61.4 to -8.7)
		Male	4323 (3054 to 5673)	826.2 (589.4 to 1043.5)	83 (55 to 110)	27.1 (19.1 to 35.9)	7950 (6461 to 9878)	548.2 (446.9 to 669.7)	204 (162 to 254)	17.5 (14 to 21.8)	83.9 (27.9 to 170.2)	-33.7 (-51.7 to -3.1)	144.6 (62.3 to 293.3)	-35.2 (-56 to 1.2)
	Cirrhosis and other chronic liver diseases	Both	2891 (1932 to 3833)	313.7 (214.8 to 403.3)	73 (50 to 94)	12.5 (8.9 to 16.4)	5261 (4357 to 6291)	193.5 (160.9 to 232.9)	189 (155 to 232)	8.3 (6.8 to 10.3)	82 (23.5 to 187.5)	-38.3 (-55.6 to -0.6)	158.2 (84 to 315.9)	-33.4 (-54.5 to 0.3)

Province	Cause	Sex	1990				2019				1990-2019 percent change (%)			
			DALYs		Death		DALYs		Death		DALYs		Death	
			Number	Rate	Number	Rate	Number	Rate	Number	Rate	Number	Rate	Number	Rate
		Female	1155 (717 to 1618)	258.8 (165.7 to 349.9)	29 (18 to 39)	11.1 (7.1 to 16.4)	1731 (1382 to 2201)	140.3 (113.1 to 177)	65 (52 to 85)	6.8 (5.4 to 9)	49.8 (-3.8 to 166.6)	-45.8 (-62.7 to -8.5)	124.2 (48.4 to 286.2)	-39 (-62.3 to 0.8)
		Male	1736 (1110 to 2483)	359.4 (227.5 to 498.5)	44 (27 to 61)	13.7 (8.9 to 18.8)	3530 (2737 to 4427)	246.5 (193.4 to 311.1)	124 (96 to 157)	10 (7.8 to 12.7)	103.3 (24.2 to 246)	-31.4 (-57.3 to 18.1)	180.6 (72.1 to 381.3)	-26.7 (-54 to 20.4)
	Upper digestive system diseases	Both	2319 (1689 to 3205)	265.2 (199.9 to 349)	36 (25 to 46)	7.9 (5.7 to 11)	4897 (3263 to 7458)	163.1 (114.5 to 237.1)	59 (45 to 71)	3.2 (2.4 to 3.8)	111.1 (64.9 to 158.7)	-38.5 (-51.2 to -23.4)	66.2 (18.8 to 151)	-60 (-72.1 to -41.4)
		Female	1067 (748 to 1512)	260.4 (182.3 to 361.7)	15 (10 to 22)	7.9 (5 to 12.2)	2454 (1578 to 3830)	170.1 (115.5 to 255.9)	26 (19 to 34)	3.5 (2.7 to 4.5)	130 (78.9 to 183.5)	-34.7 (-49.7 to -16.6)	69.9 (9.7 to 193.4)	-55.5 (-73.6 to -20.9)
		Male	1253 (887 to 1703)	268.4 (196.9 to 360.9)	20 (14 to 28)	7.9 (5.6 to 10.6)	2443 (1675 to 3631)	158.4 (111.1 to 226.1)	33 (24 to 42)	3.1 (2.2 to 3.9)	95.1 (38.7 to 153.5)	-41 (-56 to -23.5)	63.4 (4.8 to 159.2)	-60.8 (-73.9 to -40.2)
	Appendicitis	Both	106 (59 to 252)	7.5 (4.7 to 17.2)	1 (1 to 4)	0.2 (0.1 to 0.5)	142 (97 to 215)	5 (3.4 to 7.9)	2 (1 to 4)	0.1 (0.1 to 0.2)	33.8 (-23.4 to 144.5)	-34 (-58.9 to 9.1)	9.5 (-29.6 to 132)	-62.8 (-77.6 to -42.8)
		Female	44 (24 to 118)	6.8 (4 to 17.4)	1 (0 to 2)	0.2 (0.1 to 0.6)	67 (43 to 103)	4.9 (3.1 to 8.1)	1 (0 to 2)	0.1 (0 to 0.3)	51.2 (-24 to 194.7)	-27.5 (-58.4 to 23.5)	13.7 (-42.8 to 127.3)	-60.1 (-82.8 to -32.7)
		Male	62 (31 to 138)	8.2 (4.7 to 18.1)	1 (0 to 2)	0.2 (0.1 to 0.5)	75 (50 to 118)	5 (3.3 to 8.1)	1 (1 to 2)	0.1 (0 to 0.2)	21.2 (-32.9 to 161.5)	-38.4 (-62.4 to 19.9)	6.6 (-42.9 to 191.4)	-62.9 (-77 to -20.9)
	Paralytic ileus and intestinal obstruction	Both	1210 (605 to 2145)	68.2 (44.5 to 102.4)	17 (11 to 27)	2.1 (1.5 to 2.9)	671 (432 to 886)	27.6 (17.9 to 35.9)	22 (13 to 28)	1.2 (0.7 to 1.5)	-44.6 (-76.3 to 29)	-59.6 (-78.8 to -29.3)	26.3 (-38.1 to 128.8)	-45.1 (-63.4 to -18.5)
		Female	533 (245 to 1047)	65.4 (40.5 to 105.8)	8 (5 to 14)	2.3 (1.4 to 3.5)	282 (186 to 386)	24.8 (16.4 to 33.3)	9 (6 to 12)	1.1 (0.7 to 1.5)	-47 (-77.7 to 29)	-62.1 (-80.2 to -27.7)	13.5 (-47.5 to 125.2)	-52 (-70.4 to -17.8)
		Male	678 (281 to 1298)	70.2 (39.4 to 117.2)	9 (5 to 16)	1.9 (1.3 to 2.7)	388 (230 to 551)	30.4 (18.3 to 42.4)	13 (7 to 17)	1.2 (0.7 to 1.7)	-42.7 (-78.2 to 67)	-56.7 (-80.8 to -5.4)	37.4 (-44.9 to 208.9)	-35 (-63.7 to 5.2)
	Inguinal, femoral, and abdominal hernia	Both	291 (170 to 503)	19.9 (13.6 to 28.7)	2 (1 to 4)	0.3 (0.2 to 0.4)	493 (339 to 681)	17.2 (12 to 23.4)	3 (1 to 4)	0.2 (0.1 to 0.2)	69.6 (-5.1 to 158.8)	-13.5 (-39.3 to 15)	89.2 (-32.7 to 399.7)	-30.7 (-59 to 22.1)
		Female	95 (53 to 167)	12.9 (8.5 to 19.3)	0 (0 to 1)	0.2 (0.1 to 0.3)	157 (101 to 229)	11.2 (7.3 to 16.2)	1 (0 to 1)	0.1 (0.1 to 0.2)	65.8 (-15.8 to 163.8)	-13.6 (-45.2 to 17.5)	100.3 (-47.8 to 397.1)	-32.1 (-61 to 19.1)
		Male	196 (112 to 366)	26.2 (17.3 to 39.6)	1 (1 to 3)	0.3 (0.2 to 0.6)	336 (230 to 469)	23 (15.9 to 31.7)	2 (1 to 3)	0.2 (0.1 to 0.3)	71.4 (-10.3 to 178.4)	-12.4 (-41.7 to 21.4)	85.1 (-34.9 to 459.3)	-31.5 (-64.9 to 35.1)
	Inflammatory bowel disease	Both	92 (67 to 122)	10.5 (7.7 to 14.1)	1 (1 to 1)	0.2 (0.1 to 0.3)	333 (231 to 449)	10.7 (7.4 to 14.2)	4 (1 to 5)	0.2 (0.1 to 0.2)	262.6 (167.5 to 358)	1.9 (-19.9 to 26.6)	323.2 (98.4 to 629)	3.5 (-41.6 to 79)
		Female	46 (32 to 63)	11 (7.6 to 15.2)	0 (0 to 1)	0.2 (0.1 to 0.3)	176 (112 to 241)	11.8 (7.1 to 16)	2 (1 to 3)	0.2 (0.1 to 0.3)	278 (162 to 419.6)	7.2 (-23.8 to 42.5)	428.1 (104.2 to 941.3)	38.3 (-39.8 to 187.5)
		Male	45 (31 to 63)	9.9 (7 to 13.6)	0 (0 to 1)	0.1 (0.1 to 0.2)	157 (107 to 216)	9.7 (6.8 to 13.1)	1 (1 to 2)	0.1 (0.1 to 0.2)	246.8 (160.6 to 358.4)	-2.7 (-25.4 to 26.4)	218.4 (66.5 to 493.4)	-23.9 (-59.9 to 38.9)
	Vascular intestinal disorders	Both	56 (39 to 76)	11.4 (7.5 to 16.9)	2 (2 to 4)	0.7 (0.4 to 1.1)	212 (160 to 256)	9.2 (7.1 to 11.1)	10 (8 to 12)	0.5 (0.4 to 0.6)	276.9 (162 to 468.2)	-19 (-47.3 to 21.9)	317.6 (169.2 to 539.9)	-20.6 (-51.4 to 26.3)
		Female	29 (17 to 42)	13 (7.2 to 20.9)	1 (1 to 2)	0.8 (0.4 to 1.4)	106 (73 to 137)	10.1 (7.3 to 12.8)	5 (4 to 7)	0.6 (0.5 to 0.8)	269.5 (139.5 to 527.3)	-22.6 (-54.3 to 35.6)	283.4 (123.4 to 592)	-23.6 (-57.7 to 52)
		Male	27 (18 to 39)	9.5 (6.5 to 13.4)	1 (1 to 2)	0.5 (0.3 to 0.7)	106 (77 to 137)	8.7 (6.3 to 11.1)	5 (4 to 6)	0.5 (0.3 to 0.6)	284.7 (140.6 to 524)	-9.2 (-42.5 to 46.9)	361.7 (185.9 to 657.8)	-7.8 (-41.9 to 46.1)

Province	Cause	Sex	1990				2019				1990-2019 percent change (%)			
			DALYs		Death		DALYs		Death		DALYs		Death	
			Number	Rate	Number	Rate	Number	Rate	Number	Rate	Number	Rate	Number	Rate
	Gallbladder and biliary diseases	Both	477 (336 to 654)	59.7 (43 to 78.4)	5 (4 to 8)	1.4 (0.9 to 2)	1379 (968 to 1890)	49.2 (35.7 to 65.9)	21 (13 to 27)	1.2 (0.7 to 1.6)	189.2 (144.3 to 254.5)	-17.5 (-30.8 to -0.8)	288.4 (157.2 to 510.5)	-14.3 (-43.8 to 29)
		Female	308 (212 to 431)	80.5 (56.5 to 108.9)	3 (2 to 4)	1.7 (0.9 to 2.6)	903 (610 to 1289)	65.3 (46 to 89.7)	11 (6 to 15)	1.5 (0.8 to 2)	192.9 (142.1 to 259.4)	-18.8 (-33.5 to -2)	260.9 (127.6 to 485.2)	-11 (-46 to 43)
		Male	169 (120 to 229)	41 (29.9 to 54.6)	2 (1 to 4)	1.1 (0.7 to 1.7)	476 (337 to 650)	34.7 (24.5 to 45.8)	10 (6 to 14)	1 (0.6 to 1.4)	182.4 (118.5 to 276.1)	-15.5 (-36.5 to 14.7)	321.9 (156.8 to 678.5)	-8.6 (-43.6 to 63.7)
	Pancreatitis	Both	118 (82 to 160)	17.1 (11.3 to 24.7)	4 (2 to 5)	0.8 (0.5 to 1.3)	298 (235 to 426)	11.2 (9 to 15.4)	11 (8 to 15)	0.5 (0.4 to 0.7)	151.8 (76.8 to 270.3)	-34.3 (-55.7 to -2.8)	196.2 (90.8 to 358)	-37.4 (-63.7 to 4.7)
		Female	54 (32 to 76)	17.9 (9.7 to 28.7)	2 (1 to 3)	1 (0.4 to 1.8)	127 (92 to 166)	11.2 (8.1 to 14.6)	5 (4 to 7)	0.6 (0.4 to 0.9)	136.8 (55.6 to 267.4)	-37.2 (-61.3 to 1.4)	182.7 (64.7 to 379.4)	-36.3 (-67.5 to 26.6)
		Male	64 (40 to 97)	15.8 (9.9 to 24.1)	2 (1 to 3)	0.6 (0.4 to 1)	171 (121 to 275)	11.6 (8.3 to 18.7)	5 (4 to 9)	0.4 (0.3 to 0.7)	164.4 (64.1 to 321.9)	-26.8 (-53.7 to 15.7)	210.4 (87.7 to 414.8)	-28.6 (-57.5 to 15.9)
	Other digestive diseases	Both	168 (110 to 253)	18.1 (12.8 to 25)	4 (2 to 5)	0.8 (0.5 to 1.3)	462 (301 to 561)	18.5 (12.3 to 22.3)	15 (10 to 19)	0.8 (0.5 to 1)	175.2 (53.7 to 357.6)	2.4 (-32.6 to 47.4)	322.1 (156.1 to 565.9)	-0.6 (-44.9 to 63.1)
		Female	75 (47 to 116)	18.4 (12 to 27.3)	2 (1 to 3)	0.9 (0.4 to 1.7)	194 (123 to 253)	16.8 (10.9 to 21.6)	6 (4 to 8)	0.8 (0.5 to 1)	158.6 (30.6 to 347.2)	-8.6 (-40.3 to 46.5)	259.4 (107 to 544.2)	-14.2 (-57.9 to 81.1)
		Male	93 (54 to 154)	17.4 (11.8 to 24.2)	2 (1 to 3)	0.7 (0.4 to 1)	268 (167 to 344)	20.3 (12.9 to 26.1)	9 (6 to 12)	0.8 (0.5 to 1.1)	188.5 (46.4 to 429.5)	16.6 (-30.7 to 88.9)	381 (166.1 to 743.2)	23.1 (-27.2 to 107.9)
	Neurological disorders	Both	14388 (6529 to 25413)	1385.7 (744.4 to 2302.7)	101 (48 to 212)	31.1 (12 to 74.7)	37519 (18649 to 64940)	1385.8 (753.6 to 2301.3)	505 (225 to 1151)	32.1 (13.2 to 74.1)	160.8 (115.6 to 242.3)	0 (-13.1 to 17.7)	399.3 (291.5 to 588.3)	3.4 (-13.2 to 37.7)
		Female	7701 (3182 to 14222)	1527.2 (754.4 to 2618.4)	47 (21 to 105)	30.3 (10.9 to 74.8)	20879 (9264 to 37927)	1551.1 (771.4 to 2674.3)	221 (91 to 494)	32.9 (12.1 to 76.2)	171.1 (128.2 to 244.7)	1.6 (-11.2 to 20.3)	367.2 (243.8 to 582.9)	8.4 (-14.5 to 56.7)
		Male	6688 (3331 to 11304)	1261.2 (720.5 to 2044.5)	54 (27 to 110)	31.9 (12.8 to 72.8)	16639 (9393 to 27158)	1229.2 (717.2 to 1966.6)	284 (131 to 652)	31.9 (13.8 to 73.7)	148.8 (97.2 to 238.6)	-2.5 (-17.6 to 18.5)	427.5 (293.6 to 638.1)	0 (-21.4 to 34.6)
	Alzheimer's disease and other dementias	Both	1187 (520 to 2682)	375.9 (166.2 to 836)	66 (15 to 184)	24.5 (5.7 to 68.6)	6667 (2927 to 14651)	388.9 (174.4 to 857.6)	367 (89 to 1008)	25.2 (6.2 to 67.4)	461.8 (383.6 to 606.1)	3.5 (-9.4 to 26.3)	458.4 (363.3 to 681.2)	2.9 (-13.2 to 43.5)
		Female	590 (257 to 1349)	381.4 (169.1 to 858.1)	32 (7 to 91)	24.6 (5.6 to 69.2)	3201 (1392 to 7010)	410.9 (179.2 to 894.9)	166 (39 to 442)	26.9 (6.5 to 70.9)	442.2 (347.3 to 614.5)	7.7 (-9.7 to 39.9)	412.7 (295.8 to 663.1)	9.2 (-13.4 to 65.6)
		Male	596 (257 to 1372)	370.8 (163.3 to 840.2)	33 (8 to 91)	24.4 (5.8 to 65.8)	3466 (1521 to 7877)	371.7 (166.8 to 845.5)	201 (48 to 566)	24.2 (5.9 to 66.2)	481.1 (371.8 to 632.8)	0.2 (-16.7 to 25.8)	502.6 (370.1 to 733.4)	-1 (-21.6 to 36.1)
	Parkinson's disease	Both	308 (239 to 370)	79.3 (62.9 to 94.2)	15 (11 to 18)	4.8 (3.8 to 5.8)	1565 (1315 to 1811)	79 (67.1 to 90.9)	85 (71 to 99)	5 (4.1 to 5.8)	407.5 (312.2 to 559.9)	-0.4 (-19 to 27)	478.1 (355.9 to 672.7)	3.4 (-18.3 to 34.5)
		Female	129 (94 to 163)	67.2 (50.4 to 85.7)	6 (4 to 8)	4 (3 to 5.5)	606 (505 to 726)	65.4 (54.5 to 79.2)	32 (26 to 39)	4.3 (3.5 to 5.5)	367.8 (251.6 to 568.6)	-2.7 (-25.3 to 35.1)	425.7 (286.2 to 670.4)	6.1 (-20.4 to 51)

Province	Cause	Sex	1990				2019				1990-2019 percent change (%)			
			DALYs		Death		DALYs		Death		DALYs		Death	
			Number	Rate	Number	Rate	Number	Rate	Number	Rate	Number	Rate	Number	Rate
		Male	179 (127 to 230)	92.1 (68 to 115.7)	9 (6 to 11)	5.7 (4 to 7.2)	959 (716 to 1149)	91.5 (68.3 to 109.3)	53 (38 to 64)	5.6 (4.1 to 6.8)	436.2 (304.5 to 639.3)	-0.6 (-24.4 to 35.7)	515.5 (346.1 to 785.1)	-0.7 (-27.1 to 40.6)
	Idiopathic epilepsy	Both	2743 (1231 to 4679)	159.8 (69.8 to 272.5)	13 (8 to 19)	0.9 (0.6 to 1.4)	3365 (1389 to 6245)	120.3 (48.6 to 228.9)	15 (12 to 25)	0.5 (0.4 to 0.9)	22.7 (-55.3 to 207.3)	-24.7 (-72.9 to 89.9)	17.9 (-17.6 to 106.1)	-41 (-57.2 to 0.7)
		Female	1186 (517 to 2130)	144.7 (62 to 256.6)	5 (3 to 9)	0.8 (0.4 to 1.3)	1511 (591 to 2885)	111.1 (42.9 to 213.9)	6 (4 to 11)	0.4 (0.3 to 0.8)	27.5 (-56.2 to 256.5)	-23.2 (-73.1 to 109.5)	16.3 (-26 to 154)	-43.6 (-62.4 to 13.1)
		Male	1557 (717 to 2614)	174.1 (77.4 to 296.4)	8 (5 to 11)	1 (0.6 to 1.5)	1853 (792 to 3427)	129.2 (53.3 to 240.4)	9 (7 to 15)	0.6 (0.5 to 1)	19 (-54.7 to 189.9)	-25.8 (-72.7 to 85.9)	18.9 (-22.1 to 108.7)	-38.8 (-57.5 to 5.1)
	Multiple sclerosis	Both	277 (188 to 384)	28.8 (19.9 to 40.5)	3 (2 to 6)	0.4 (0.2 to 0.8)	924 (678 to 1347)	27.1 (20.2 to 38.8)	13 (9 to 23)	0.4 (0.3 to 0.7)	234 (131.1 to 359.9)	-5.8 (-28.1 to 24.5)	283 (121.5 to 566.3)	-2 (-35.3 to 75.8)
		Female	176 (121 to 275)	37.9 (26.7 to 54.4)	2 (1 to 4)	0.5 (0.3 to 0.9)	568 (432 to 729)	34 (26.1 to 43.3)	7 (5 to 10)	0.5 (0.4 to 0.7)	222.2 (95.1 to 362.4)	-10.4 (-41.2 to 23.6)	245.3 (61 to 617.5)	-11.4 (-53.8 to 77.7)
		Male	101 (60 to 177)	20.5 (12.2 to 37)	1 (1 to 3)	0.3 (0.1 to 0.8)	357 (222 to 683)	20.5 (13 to 38.3)	6 (3 to 14)	0.4 (0.2 to 0.9)	254.5 (132.8 to 397.6)	-0.3 (-32.1 to 35.4)	342.3 (136.8 to 712.1)	13.3 (-37.2 to 100.1)
	Motor neuron disease	Both	21 (16 to 27)	2 (1.4 to 2.6)	0 (0 to 0)	0 (0 to 0.1)	133 (107 to 167)	4.5 (3.6 to 5.6)	3 (3 to 5)	0.1 (0.1 to 0.2)	524.9 (339 to 810.9)	126.5 (52.9 to 250.3)	973.1 (537.3 to 1830.7)	203.9 (77.5 to 444.9)
		Female	12 (9 to 16)	2.1 (1.5 to 3)	0 (0 to 0)	0 (0 to 0.1)	60 (48 to 75)	4.2 (3.3 to 5.3)	2 (1 to 2)	0.1 (0.1 to 0.2)	402.5 (247.5 to 648.7)	95.6 (29.2 to 208.2)	782.1 (416.8 to 1513.4)	172.4 (48.1 to 414.7)
		Male	9 (6 to 13)	1.8 (1.2 to 2.6)	0 (0 to 0)	0 (0 to 0.1)	73 (56 to 97)	4.7 (3.6 to 6.3)	2 (1 to 3)	0.1 (0.1 to 0.2)	681.5 (389.6 to 1132.8)	161.9 (58.2 to 327.1)	1202.1 (550.7 to 2608.9)	239.3 (71.9 to 598.8)
	Headache disorders	Both	9170 (1971 to 20125)	698.9 (169.9 to 1492.6)	--	--	23202 (5791 to 49364)	706 (172.2 to 1510.2)	--	--	153 (129.9 to 210.5)	1 (-3.9 to 7.3)	--	--
		Female	5325 (1057 to 11913)	857.4 (192.5 to 1882.2)	--	--	14208 (3210 to 30718)	871.8 (192.9 to 1897.2)	--	--	166.8 (143.5 to 217.8)	1.7 (-4.8 to 8)	--	--
		Male	3846 (894 to 8328)	556.5 (148.6 to 1177.3)	--	--	8994 (2672 to 18767)	545.2 (153.1 to 1144.6)	--	--	133.9 (107.3 to 207)	-2 (-8.5 to 8.1)	--	--
	Other neurological disorders	Both	682 (365 to 1111)	41.1 (24.2 to 62.7)	4 (3 to 6)	0.4 (0.3 to 0.5)	1663 (1161 to 2405)	60.1 (41.2 to 88.3)	22 (18 to 25)	0.9 (0.7 to 1)	143.9 (37.2 to 370.6)	46.4 (-15.9 to 164.4)	410.9 (258.2 to 686.4)	119.8 (55.8 to 240.6)
		Female	283 (142 to 473)	36.4 (20.9 to 56.8)	2 (1 to 2)	0.3 (0.2 to 0.5)	726 (488 to 1063)	53.7 (35.1 to 79.8)	9 (7 to 10)	0.7 (0.6 to 0.8)	156.9 (37.3 to 409.4)	47.5 (-17.1 to 167)	456.8 (281 to 779.2)	116.9 (40 to 260.7)
		Male	399 (210 to 641)	45.4 (26.2 to 69.3)	3 (2 to 4)	0.4 (0.3 to 0.6)	937 (649 to 1356)	66.4 (44.9 to 97.4)	13 (11 to 16)	1 (0.8 to 1.2)	134.6 (33.6 to 356)	46.2 (-15.8 to 165.4)	385.4 (205.5 to 711.3)	124.6 (46.5 to 262.1)
	Mental disorders	Both	30137 (22009 to 39965)	2276.7 (1678.7 to 2996.6)	0 (0 to 0)	0 (0 to 0)	75538 (56022 to 99728)	2346.9 (1739.8 to 3099.8)	0 (0 to 0)	0 (0 to 0)	150.6 (137.9 to 164)	3.1 (0.3 to 6)	452.7 (149.8 to 1302.4)	98.5 (-9.5 to 422.5)
		Female	16252 (11789 to 21539)	2595.9 (1915.6 to 3414.9)	0 (0 to 0)	0 (0 to 0)	42347 (31026 to 56050)	2658.2 (1958.8 to 3528.9)	0 (0 to 0)	0 (0 to 0)	160.6 (145.7 to 176)	2.4 (-1.4 to 6.2)	415.2 (106 to 1314.5)	77 (-30.5 to 388.7)

Province	Cause	Sex	1990				2019				1990-2019 percent change (%)			
			DALYs		Death		DALYs		Death		DALYs		Death	
			Number	Rate	Number	Rate	Number	Rate	Number	Rate	Number	Rate	Number	Rate
		Male	13886 (10153 to 18415)	1988.9 (1467.8 to 2624.7)	0 (0 to 0)	0 (0 to 0)	33191 (24478 to 43532)	2043.7 (1494.3 to 2678.9)	0 (0 to 0)	0 (0 to 0)	139 (125.4 to 153.8)	2.8 (-1 to 6.4)	586.4 (86.8 to 2914.7)	167.5 (-25 to 1114.5)
	Schizophrenia	Both	1816 (1294 to 2375)	161.9 (116.2 to 209)	--	--	5888 (4254 to 7500)	162.9 (119.7 to 206.9)	--	--	224.3 (194.4 to 257.1)	0.6 (-7 to 9.2)	--	--
		Female	813 (570 to 1063)	153.2 (108.8 to 198)	--	--	2738 (1961 to 3512)	154.2 (112.1 to 195.6)	--	--	236.9 (194.7 to 287.7)	0.7 (-10.9 to 13.3)	--	--
		Male	1003 (717 to 1310)	169.7 (122.7 to 218)	--	--	3150 (2202 to 4008)	171.4 (122.6 to 215.3)	--	--	214 (176.9 to 259.8)	1 (-9.9 to 13.3)	--	--
	Depressive disorders	Both	10814 (7226 to 15064)	890.5 (607.4 to 1231.5)	--	--	31431 (21219 to 44383)	947.5 (648.4 to 1320.6)	--	--	190.7 (165.7 to 216.1)	6.4 (2.2 to 10.8)	--	--
		Female	6544 (4363 to 9058)	1121 (762.9 to 1555.9)	--	--	18918 (12712 to 26618)	1160 (790.1 to 1622)	--	--	189.1 (160.9 to 217.7)	3.5 (-2.1 to 9.3)	--	--
		Male	4270 (2864 to 6000)	682.8 (465.9 to 954)	--	--	12513 (8544 to 17544)	741.5 (505.3 to 1035.1)	--	--	193.1 (166.3 to 224.2)	8.6 (2.7 to 15.6)	--	--
	Bipolar disorder	Both	2179 (1310 to 3360)	174.5 (107.4 to 266.5)	--	--	5845 (3608 to 9059)	176.8 (108 to 270.7)	--	--	168.2 (141.8 to 197)	1.3 (-5.3 to 8.3)	--	--
		Female	1139 (672 to 1746)	188 (114.9 to 285.5)	--	--	3086 (1888 to 4750)	190.1 (116 to 291.4)	--	--	171 (136.9 to 209.2)	1.1 (-8.1 to 11.4)	--	--
		Male	1040 (625 to 1602)	162 (98.6 to 244.9)	--	--	2759 (1726 to 4286)	163.7 (101.7 to 250.2)	--	--	165.2 (135 to 200)	1.1 (-8.1 to 11.5)	--	--
	Anxiety disorders	Both	9375 (6446 to 12897)	670.7 (467.1 to 919.4)	--	--	21701 (15129 to 29851)	698.2 (489.7 to 956.6)	--	--	131.5 (107.7 to 157.8)	4.1 (-1.8 to 10.8)	--	--
		Female	5484 (3823 to 7575)	828.2 (578.7 to 1142.7)	--	--	13253 (9177 to 18397)	858.8 (595.3 to 1179)	--	--	141.7 (114.5 to 173.8)	3.7 (-3.9 to 12.4)	--	--
		Male	3891 (2641 to 5412)	527.2 (364.5 to 729.9)	--	--	8448 (5854 to 11610)	542.2 (378 to 748)	--	--	117.1 (91.3 to 148.6)	2.8 (-6.5 to 13.3)	--	--
	Eating disorders	Both	747 (449 to 1121)	47.8 (29.2 to 71.3)	0 (0 to 0)	0 (0 to 0)	1888 (1172 to 2862)	57.1 (35.3 to 85.6)	0 (0 to 0)	0 (0 to 0)	152.6 (118.7 to 194)	19.5 (7.6 to 33.3)	452.7 (149.8 to 1302.4)	98.5 (-9.5 to 422.5)
		Female	451 (276 to 678)	60 (37.1 to 89.9)	0 (0 to 0)	0 (0 to 0)	1153 (721 to 1715)	71.4 (44.8 to 107.2)	0 (0 to 0)	0 (0 to 0)	155.6 (116.7 to 205.8)	19 (3.7 to 37.1)	415.2 (106 to 1314.5)	77 (-30.5 to 388.7)
		Male	296 (174 to 448)	36.6 (20.9 to 55.7)	0 (0 to 0)	0 (0 to 0)	734 (435 to 1146)	43.1 (26 to 65.9)	0 (0 to 0)	0 (0 to 0)	148 (103.1 to 204.1)	18 (0.7 to 38.1)	586.4 (86.8 to 2914.7)	167.5 (-25 to 1114.5)
	Autism spectrum disorders	Both	746 (484 to 1105)	45.5 (29.7 to 66.9)	--	--	1333 (863 to 1927)	45.4 (29.3 to 65.5)	--	--	78.8 (61.3 to 97.1)	-0.2 (-10.1 to 10.4)	--	--

Province	Cause	Sex	1990				2019				1990-2019 percent change (%)			
			DALYs		Death		DALYs		Death		DALYs		Death	
			Number	Rate	Number	Rate	Number	Rate	Number	Rate	Number	Rate	Number	Rate
		Female	179 (117 to 264)	22.5 (14.6 to 33.1)	--	--	327 (212 to 483)	22.7 (14.7 to 33.4)	--	--	82.6 (56.9 to 115.4)	0.7 (-13.1 to 17.3)	--	--
		Male	567 (366 to 850)	66.6 (43.3 to 98.7)	--	--	1006 (646 to 1471)	67.2 (43.2 to 98.4)	--	--	77.6 (56.6 to 102.1)	0.8 (-11.5 to 14.1)	--	--
	Attention-deficit/hyperactivity disorder	Both	597 (337 to 1030)	32.7 (18.6 to 55.8)	--	--	925 (523 to 1555)	33.5 (18.9 to 56.5)	--	--	55.1 (32.2 to 84.4)	2.5 (-10.8 to 16.3)	--	--
		Female	177 (98 to 306)	20.1 (11.3 to 34.6)	--	--	284 (159 to 472)	20.9 (11.7 to 34.6)	--	--	60.8 (32.8 to 95.2)	4 (-12.6 to 24.3)	--	--
		Male	420 (234 to 724)	44.5 (25.1 to 75.3)	--	--	641 (366 to 1093)	45.7 (26.2 to 78.3)	--	--	52.7 (25.4 to 86.4)	2.7 (-14.3 to 21.7)	--	--
	Conduct disorder	Both	1610 (923 to 2544)	76 (43.4 to 119.8)	--	--	1618 (918 to 2558)	74.4 (42.2 to 117.7)	--	--	0.5 (-8.6 to 10.5)	-2 (-11 to 7.6)	--	--
		Female	549 (290 to 886)	53 (27.8 to 85.5)	--	--	538 (285 to 906)	50.9 (26.9 to 85.8)	--	--	-1.9 (-15.5 to 14)	-3.8 (-17.3 to 11.9)	--	--
		Male	1062 (621 to 1667)	97.8 (57.3 to 152.7)	--	--	1080 (625 to 1709)	96.7 (56 to 153.1)	--	--	1.7 (-10.6 to 14.4)	-1.1 (-13.3 to 11)	--	--
	Idiopathic developmental intellectual disability	Both	1248 (659 to 2015)	75.6 (40.4 to 121.5)	--	--	1485 (725 to 2521)	50.2 (24.6 to 85.4)	--	--	19 (0.1 to 30.5)	-33.5 (-44.7 to -27)	--	--
		Female	535 (283 to 872)	67.2 (36.1 to 109.4)	--	--	676 (338 to 1141)	46.6 (23 to 78.3)	--	--	26.5 (8.5 to 38.8)	-30.7 (-40.7 to -24.2)	--	--
		Male	714 (375 to 1154)	83.3 (43.9 to 134.1)	--	--	809 (388 to 1375)	53.8 (25.4 to 91.2)	--	--	13.4 (-7.4 to 27.9)	-35.5 (-47.6 to -27.1)	--	--
	Other mental disorders	Both	1005 (639 to 1548)	101.5 (65.1 to 154.9)	--	--	3424 (2187 to 5279)	100.8 (64.9 to 153.5)	--	--	240.6 (214.4 to 271.3)	-0.8 (-6 to 4.9)	--	--
		Female	382 (242 to 591)	82.8 (53.3 to 126.7)	--	--	1374 (864 to 2139)	82.6 (52.5 to 127.5)	--	--	259.3 (220.9 to 304.6)	-0.2 (-8.5 to 8.9)	--	--
		Male	623 (391 to 942)	118.3 (75.9 to 180.5)	--	--	2049 (1297 to 3108)	118.2 (75.7 to 178.5)	--	--	229 (198.7 to 262)	-0.1 (-7.1 to 7.4)	--	--
	Substance use disorders	Both	4825 (3510 to 6074)	366.3 (272.5 to 457.1)	32 (17 to 42)	3.2 (2 to 4)	16543 (13376 to 20036)	478.2 (385.3 to 577.7)	167 (136 to 203)	5 (4.1 to 6.1)	242.9 (190.9 to 326.3)	30.5 (13.1 to 59.3)	421.3 (272.9 to 841)	58.2 (16.7 to 155.6)
		Female	1469 (1052 to 1943)	233.6 (170 to 304.1)	10 (4 to 14)	2.1 (1.2 to 2.9)	3298 (2553 to 4157)	199 (153.8 to 251.4)	23 (18 to 30)	1.6 (1.3 to 2)	124.5 (81.3 to 180.8)	-14.8 (-30 to 7.2)	139.9 (48.5 to 476.2)	-24 (-49.1 to 35.4)
		Male	3355 (2483 to 4320)	486.9 (358.7 to 617.7)	22 (13 to 31)	4.1 (2.6 to 5.5)	13245 (10589 to 16139)	751.3 (603.7 to 916.6)	144 (113 to 181)	8.3 (6.6 to 10.3)	294.8 (226.2 to 395.5)	54.3 (29.5 to 92.6)	541.4 (333.9 to 1087.5)	101.9 (40 to 251.1)
	Alcohol use disorders	Both	673 (472 to 879)	56 (39.3 to 72.5)	5 (3 to 6)	0.5 (0.3 to 0.7)	1696 (1251 to 2260)	48.2 (35.8 to 63.7)	14 (9 to 17)	0.4 (0.3 to 0.5)	151.8 (112.8 to 213.8)	-14 (-27.4 to 6.1)	191.9 (102.8 to 416)	-17.2 (-41.6 to 44.3)
		Female	185 (131 to 248)	31.3 (22.7 to 41.3)	1 (1 to 2)	0.3 (0.2 to 0.4)	445 (311 to 617)	26.7 (19.1 to 36.8)	2 (2 to 3)	0.2 (0.1 to 0.2)	140.4 (91.8 to 205.8)	-14.9 (-29.7 to 6.3)	78 (16.9 to 271.4)	-39.6 (-58.1 to 2.8)
		Male	488 (332 to 644)	78.1 (54 to 102.2)	4 (2 to 5)	0.7 (0.4 to 1)	1250 (906 to 1656)	69 (50.5 to 90.6)	12 (6 to 15)	0.7 (0.4 to 0.9)	156.2 (109.6 to 231.7)	-11.6 (-27.9 to 14.2)	228.2 (113.4 to 555.8)	-6.6 (-38.9 to 72.6)

Province	Cause	Sex	1990				2019				1990-2019 percent change (%)			
			DALYs		Death		DALYs		Death		DALYs		Death	
			Number	Rate	Number	Rate	Number	Rate	Number	Rate	Number	Rate	Number	Rate
	Drug use disorders	Both	4151 (3019 to 5347)	310.3 (230.7 to 390.8)	27 (14 to 36)	2.7 (1.6 to 3.4)	14848 (12003 to 18153)	430 (346.2 to 525)	153 (123 to 189)	4.6 (3.7 to 5.6)	257.7 (200.3 to 354.1)	38.6 (18.9 to 74.4)	461 (294.7 to 937.4)	72.9 (24.8 to 181.6)
		Female	1284 (915 to 1718)	202.3 (147.2 to 266)	8 (4 to 13)	1.8 (1 to 2.6)	2853 (2168 to 3647)	172.3 (131.7 to 221.1)	21 (16 to 27)	1.4 (1.1 to 1.8)	122.2 (75.9 to 179.4)	-14.8 (-31.5 to 8.6)	148.3 (51.5 to 504.8)	-21.7 (-48.9 to 38.3)
		Male	2867 (2091 to 3714)	408.8 (302.5 to 520.1)	19 (10 to 26)	3.4 (2.1 to 4.6)	11995 (9559 to 14740)	682.3 (541.8 to 840.8)	132 (103 to 167)	7.6 (6 to 9.5)	318.3 (239.8 to 440.3)	66.9 (39 to 111.5)	600.9 (368.6 to 1247.5)	125.5 (52.7 to 296.2)
	Diabetes and kidney diseases	Both	7464 (6098 to 8844)	1031.7 (840 to 1227.3)	146 (113 to 180)	31.1 (24.7 to 38.6)	37716 (30702 to 45247)	1453.4 (1183 to 1722.1)	810 (687 to 924)	39.9 (34 to 45.4)	405.3 (331.1 to 508.3)	40.9 (19.7 to 68.7)	454.9 (329.1 to 652.9)	28.4 (-0.9 to 67.3)
		Female	3741 (2925 to 4551)	1098.2 (867.5 to 1350.6)	74 (52 to 95)	33.4 (24.7 to 44.2)	19095 (15188 to 23292)	1538.1 (1245.6 to 1854.7)	406 (317 to 489)	45 (35.6 to 54)	410.4 (307.2 to 542.4)	40.1 (12.5 to 75.5)	449.5 (289.7 to 709.9)	34.7 (-6.9 to 90.3)
		Male	3723 (2983 to 4514)	967.7 (759.6 to 1191.9)	72 (53 to 94)	28.6 (21.3 to 37.1)	18621 (15030 to 22482)	1385.7 (1129.9 to 1660.2)	404 (325 to 486)	36.6 (29.9 to 44)	400.2 (312.8 to 511.2)	43.2 (16.5 to 76.6)	460.4 (294.2 to 703.5)	28 (-9.9 to 80.2)
	Diabetes mellitus	Both	3592 (2817 to 4433)	548.9 (433.4 to 672.4)	58 (43 to 74)	12.2 (9.3 to 15.4)	27599 (21837 to 34368)	1045.9 (836.1 to 1285.6)	495 (405 to 575)	23.7 (19.4 to 27.5)	668.3 (560.5 to 803.6)	90.5 (63.5 to 123.1)	753.9 (523.9 to 1102.4)	93.7 (40.7 to 171.8)
		Female	1827 (1365 to 2276)	598.2 (458.8 to 748.5)	32 (22 to 44)	14.5 (9.9 to 19.5)	14254 (10823 to 17953)	1125.6 (859.7 to 1393.9)	260 (184 to 320)	27.9 (20.6 to 34)	680.2 (534.3 to 879.7)	88.1 (49.1 to 135.2)	710.6 (403.4 to 1221.9)	92.5 (20.3 to 196.5)
		Male	1765 (1330 to 2233)	502.7 (385.5 to 636)	26 (18 to 35)	10 (7.2 to 13.3)	13345 (10458 to 16604)	978 (773.4 to 1208.2)	235 (187 to 290)	20.8 (16.6 to 25.5)	656.1 (533.9 to 813.1)	94.6 (63.8 to 133.5)	807.5 (518.7 to 1291.1)	108.1 (45.8 to 208.7)
	Chronic kidney disease	Both	3858 (3098 to 4563)	481.9 (393.3 to 579.4)	88 (68 to 109)	18.8 (14.8 to 24.8)	10100 (8608 to 11703)	406.9 (349.7 to 470.8)	314 (268 to 365)	16.2 (13.7 to 18.7)	161.8 (115.3 to 232.5)	-15.6 (-31.1 to 8.9)	258.4 (173.1 to 394.6)	-14 (-38.5 to 13.1)
		Female	1907 (1470 to 2346)	498.9 (386.6 to 646.4)	42 (30 to 58)	18.9 (13.8 to 29.2)	4832 (3988 to 5770)	411.7 (343.1 to 487.6)	146 (118 to 177)	17.1 (13.9 to 20.6)	153.3 (97.4 to 251.1)	-17.5 (-35.9 to 14.5)	250.1 (141.2 to 443.2)	-9.5 (-42.2 to 30.4)
		Male	1951 (1540 to 2377)	464.2 (365.1 to 584.3)	46 (34 to 60)	18.6 (13.8 to 24.5)	5268 (4368 to 6296)	407.1 (339.3 to 483.4)	168 (134 to 207)	15.8 (12.6 to 19.5)	170 (107.3 to 254)	-12.3 (-33.4 to 16.6)	265.9 (158 to 423.2)	-15 (-40.6 to 20.2)
	Acute glomerulonephritis	Both	14 (6 to 73)	0.9 (0.5 to 4.2)	0 (0 to 1)	0 (0 to 0.1)	18 (14 to 27)	0.7 (0.5 to 1.2)	1 (0 to 1)	0 (0 to 0)	29.2 (-63.9 to 193.4)	-28.7 (-71.9 to 41.2)	126.9 (-25.8 to 380.5)	-14.5 (-63.6 to 65.6)
		Female	7 (3 to 22)	1 (0.5 to 2.8)	0 (0 to 0)	0 (0 to 0.1)	10 (7 to 13)	0.8 (0.6 to 1.1)	0 (0 to 0)	0 (0 to 0.1)	40.5 (-36.1 to 253)	-21.7 (-61.4 to 76.4)	155.3 (14.8 to 504.4)	4.6 (-54.1 to 138.3)
		Male	7 (2 to 52)	0.8 (0.3 to 5.6)	0 (0 to 1)	0 (0 to 0.2)	8 (6 to 16)	0.6 (0.4 to 1.3)	0 (0 to 0)	0 (0 to 0)	17.5 (-73.9 to 324.8)	-34.8 (-78.7 to 88)	95.3 (-48.9 to 528.6)	-28.6 (-74.2 to 100.6)
	Skin and subcutaneous diseases	Both	6466 (4296 to 9452)	413.4 (277.8 to 609.8)	1 (1 to 2)	0.3 (0.2 to 0.5)	11997 (7947 to 17658)	424.7 (282.1 to 627.4)	8 (5 to 10)	0.4 (0.3 to 0.5)	85.5 (77.2 to 92.7)	2.7 (-0.1 to 5.2)	488.3 (213.3 to 831.2)	27.6 (-30.6 to 121.6)
		Female	3330 (2196 to 4815)	439.1 (295.3 to 638.1)	1 (0 to 1)	0.4 (0.2 to 0.6)	6234 (4121 to 9204)	449.1 (298.1 to 663.2)	4 (2 to 5)	0.5 (0.2 to 0.7)	87.2 (77.5 to 95.7)	2.3 (-1.4 to 5.5)	431.8 (119.7 to 895.5)	28.3 (-54.6 to 170.4)
		Male	3137 (2078 to 4622)	389.5 (261.3 to 581.7)	1 (0 to 1)	0.3 (0.2 to 0.5)	5763 (3807 to 8451)	401.5 (266.5 to 593)	4 (2 to 5)	0.4 (0.2 to 0.5)	83.7 (74.3 to 92.3)	3.1 (-0.7 to 6.6)	558.4 (263.6 to 1042.3)	42.6 (-25.5 to 164.3)

Province	Cause	Sex	1990				2019				1990-2019 percent change (%)			
			DALYs		Death		DALYs		Death		DALYs		Death	
			Number	Rate	Number	Rate	Number	Rate	Number	Rate	Number	Rate	Number	Rate
	Dermatitis	Both	1496 (884 to 2379)	93.4 (56.6 to 144.6)	--	--	2763 (1683 to 4233)	94 (57.3 to 146)	--	--	84.7 (68.6 to 104.1)	0.6 (-3.9 to 5.3)	--	--
		Female	805 (472 to 1260)	103.9 (63 to 158.6)	--	--	1509 (910 to 2329)	104.4 (62.8 to 162.8)	--	--	87.4 (67.7 to 109.3)	0.5 (-6.1 to 7.1)	--	--
		Male	690 (401 to 1115)	83.8 (50.3 to 128.6)	--	--	1254 (769 to 1914)	83.9 (50.9 to 128.5)	--	--	81.6 (63.4 to 103.9)	0.2 (-6.4 to 7.6)	--	--
	Psoriasis	Both	522 (369 to 701)	43.3 (31.1 to 57.8)	--	--	1037 (720 to 1402)	32.4 (22.7 to 43.7)	--	--	98.5 (76 to 123.8)	-25.3 (-32.4 to -17.4)	--	--
		Female	263 (182 to 360)	44.9 (31.5 to 60.4)	--	--	526 (364 to 710)	33.6 (23.2 to 44.9)	--	--	100 (69.1 to 136.2)	-25.2 (-35.4 to -14.1)	--	--
		Male	259 (183 to 349)	41.9 (29.8 to 56.3)	--	--	511 (351 to 698)	31.2 (21.7 to 42.6)	--	--	97 (66.3 to 132.7)	-25.5 (-35.6 to -13.8)	--	--
	Bacterial skin diseases	Both	98 (65 to 159)	8.8 (6.2 to 12.6)	1 (1 to 2)	0.3 (0.2 to 0.4)	226 (161 to 335)	8.5 (6.2 to 12.2)	5 (4 to 6)	0.2 (0.2 to 0.3)	129.4 (92.5 to 182.9)	-4.2 (-23.1 to 25.7)	309.4 (172.5 to 590.1)	-13.1 (-46.3 to 67.4)
		Female	46 (30 to 74)	9.3 (6.2 to 13.7)	1 (0 to 1)	0.3 (0.2 to 0.5)	107 (73 to 162)	8.8 (6 to 12.8)	3 (2 to 4)	0.3 (0.2 to 0.5)	132.9 (84 to 222.3)	-5.6 (-35.1 to 51.7)	302.7 (107.4 to 792.9)	-6.4 (-55.3 to 163)
		Male	52 (34 to 84)	8.3 (5.6 to 12.4)	1 (0 to 1)	0.2 (0.1 to 0.4)	119 (82 to 173)	8.3 (5.9 to 12.1)	2 (1 to 3)	0.2 (0.1 to 0.3)	126.4 (85.7 to 187.8)	0.8 (-22 to 33.4)	317.9 (165.9 to 601.2)	-9.1 (-44.4 to 67.3)
	Scabies	Both	413 (223 to 662)	25.1 (13.8 to 39.9)	--	--	720 (396 to 1168)	25.1 (13.8 to 40.5)	--	--	74.2 (61 to 90)	-0.1 (-4.5 to 4.8)	--	--
		Female	201 (108 to 323)	25.2 (14 to 40.2)	--	--	354 (193 to 569)	25.1 (13.8 to 40.5)	--	--	76.5 (59.7 to 96.7)	-0.1 (-6.1 to 6.8)	--	--
		Male	213 (116 to 341)	25 (13.6 to 40)	--	--	366 (202 to 600)	25 (13.8 to 40.5)	--	--	72 (56.8 to 90.4)	-0.2 (-6 to 6.9)	--	--
	Fungal skin diseases	Both	199 (79 to 420)	18.9 (7.6 to 40.3)	--	--	513 (206 to 1107)	18.4 (7.4 to 39.8)	--	--	158.2 (140.2 to 176.5)	-2.3 (-4.1 to -0.5)	--	--
		Female	88 (35 to 186)	17.6 (7.1 to 37.8)	--	--	234 (94 to 507)	17.2 (6.9 to 37.5)	--	--	166.9 (146.8 to 187.1)	-2.1 (-4.6 to 0.3)	--	--
		Male	111 (44 to 234)	20.1 (8 to 43.4)	--	--	279 (112 to 606)	19.6 (7.8 to 41.9)	--	--	151.2 (132.9 to 170.8)	-2.5 (-5 to 0)	--	--
	Viral skin diseases	Both	1028 (652 to 1550)	51.3 (32.7 to 77.2)	--	--	1351 (860 to 2027)	51.5 (32.7 to 77.4)	--	--	31.4 (25.1 to 37.8)	0.3 (-4 to 4.7)	--	--
		Female	496 (314 to 745)	50.9 (32.3 to 76.4)	--	--	656 (417 to 997)	51 (32.3 to 77.8)	--	--	32.3 (24.3 to 41.2)	0.3 (-5.7 to 6.2)	--	--
		Male	532 (336 to 803)	51.7 (32.7 to 77.7)	--	--	695 (438 to 1030)	51.9 (32.8 to 77.1)	--	--	30.6 (22.2 to 39.2)	0.3 (-5.4 to 6)	--	--
	Acne vulgaris	Both	989 (590 to 1576)	54.3 (32.5 to 86.7)	--	--	1811 (1088 to 2865)	72.7 (44 to 115.3)	--	--	83.1 (68.9 to 99.2)	33.9 (24.2 to 44.6)	--	--
		Female	462 (275 to 732)	52.6 (31.4 to 83.3)	--	--	872 (513 to 1373)	70.6 (41.5 to 111.6)	--	--	88.9 (69.5 to 111.7)	34.2 (20.8 to 49.4)	--	--
		Male	527 (311 to 837)	55.8 (33 to 88.3)	--	--	939 (568 to 1475)	74.8 (45 to 117.5)	--	--	78.1 (59.3 to 99.5)	33.9 (20.5 to 49.1)	--	--

Province	Cause	Sex	1990				2019				1990-2019 percent change (%)			
			DALYs		Death		DALYs		Death		DALYs		Death	
			Number	Rate	Number	Rate	Number	Rate	Number	Rate	Number	Rate	Number	Rate
	Alopecia areata	Both	84 (52 to 127)	6.3 (3.9 to 9.3)	--	--	212 (131 to 320)	6.4 (3.9 to 9.6)	--	--	151.3 (123.1 to 184.3)	0.9 (-9.1 to 12.6)	--	--
		Female	53 (33 to 80)	8.3 (5.2 to 12.3)	--	--	137 (86 to 206)	8.3 (5.2 to 12.6)	--	--	157.5 (123.9 to 200.3)	0.1 (-11.4 to 14.3)	--	--
		Male	31 (19 to 48)	4.5 (2.7 to 6.7)	--	--	75 (46 to 115)	4.4 (2.7 to 6.8)	--	--	140.9 (96.5 to 202.3)	-0.7 (-17.7 to 20)	--	--
	Pruritus	Both	125 (59 to 227)	10.2 (4.9 to 18.4)	--	--	324 (153 to 581)	10.7 (5.1 to 19)	--	--	159.6 (138.4 to 190)	4.4 (-0.6 to 10.1)	--	--
		Female	66 (31 to 119)	11.2 (5.4 to 20)	--	--	174 (82 to 316)	11.7 (5.6 to 21.2)	--	--	165.7 (138.5 to 198.6)	4.2 (-2.8 to 11.4)	--	--
		Male	59 (28 to 109)	9.3 (4.4 to 16.8)	--	--	150 (71 to 267)	9.7 (4.6 to 17.4)	--	--	152.9 (129.6 to 184.4)	4.1 (-3.1 to 12.5)	--	--
	Urticaria	Both	1030 (665 to 1518)	58.1 (37.7 to 84.4)	--	--	1675 (1101 to 2403)	58.2 (38.2 to 83.5)	--	--	62.7 (47.4 to 79.7)	0.3 (-5 to 5.7)	--	--
		Female	617 (399 to 915)	71.1 (46.9 to 102.6)	--	--	994 (649 to 1417)	71.1 (46.8 to 103.6)	--	--	61.1 (44.5 to 79.3)	0 (-6.8 to 7.2)	--	--
		Male	412 (259 to 614)	45.9 (29.3 to 67.2)	--	--	681 (444 to 978)	45.9 (30.1 to 66.1)	--	--	65.2 (45 to 87.4)	0 (-8.4 to 9.3)	--	--
	Decubitus ulcer	Both	5 (4 to 7)	1.1 (0.8 to 1.6)	0 (0 to 0)	0.1 (0 to 0.1)	60 (21 to 81)	3 (1.1 to 4)	3 (1 to 4)	0.2 (0.1 to 0.3)	1050.9 (258 to 1620.8)	169 (-15.7 to 322)	1831.2 (210.3 to 3811.2)	246.8 (-33.6 to 618.2)
		Female	2 (2 to 4)	1.1 (0.7 to 1.9)	0 (0 to 0)	0.1 (0 to 0.1)	24 (8 to 36)	2.8 (0.9 to 4.3)	1 (0 to 2)	0.2 (0 to 0.3)	922.6 (204.6 to 1769.2)	158.7 (-29.3 to 409.4)	1506.1 (103.2 to 4325.5)	247.1 (-59.1 to 902.3)
		Male	3 (2 to 4)	1.1 (0.8 to 1.9)	0 (0 to 0)	0 (0 to 0.1)	36 (12 to 49)	3.2 (1.2 to 4.3)	2 (0 to 2)	0.2 (0 to 0.3)	1154.3 (274 to 1825.8)	187.4 (-16.3 to 367.6)	2193.3 (263.2 to 5369.4)	268.6 (-36.1 to 790.2)
	Other skin and subcutaneous diseases	Both	477 (229 to 886)	42.6 (20.6 to 78.6)	0 (0 to 0)	0 (0 to 0)	1304 (624 to 2391)	44 (21.2 to 80.2)	0 (0 to 0)	0 (0 to 0)	173.6 (165.5 to 182.2)	3.3 (1 to 5.7)	579.3 (270.8 to 1193.6)	4.1 (-44.2 to 115.5)
		Female	231 (111 to 430)	43.1 (20.9 to 79.9)	0 (0 to 0)	0 (0 to 0)	647 (311 to 1195)	44.4 (21.4 to 81.3)	0 (0 to 0)	0 (0 to 0)	180.1 (168.9 to 191.3)	2.9 (-0.3 to 6.1)	302.8 (40.3 to 1157.4)	-25.1 (-77.6 to 168.4)
		Male	246 (118 to 455)	42.2 (20.3 to 78.1)	0 (0 to 0)	0 (0 to 0)	658 (311 to 1198)	43.6 (20.8 to 79.3)	0 (0 to 0)	0 (0 to 0)	167.6 (157.6 to 178.6)	3.5 (0.4 to 7)	841.8 (341.9 to 1808.7)	39.4 (-33 to 208.6)
	Sense organ diseases	Both	7187 (5079 to 9788)	989.9 (716.2 to 1324.9)	--	--	19823 (13907 to 27167)	782.7 (553 to 1066.5)	--	--	175.8 (160.7 to 190.8)	-20.9 (-23.8 to -17.8)	--	--
		Female	3421 (2402 to 4628)	991.6 (713.8 to 1320.7)	--	--	9633 (6770 to 13258)	783.9 (558.3 to 1058.5)	--	--	181.6 (164.5 to 197.8)	-20.9 (-24.2 to -17.7)	--	--

Province	Cause	Sex	1990				2019				1990-2019 percent change (%)			
			DALYs		Death		DALYs		Death		DALYs		Death	
			Number	Rate	Number	Rate	Number	Rate	Number	Rate	Number	Rate	Number	Rate
		Male	3766 (2651 to 5167)	987.2 (711 to 1328.9)	--	--	10190 (7103 to 13956)	782 (548.4 to 1066.6)	--	--	170.6 (153.9 to 186.8)	-20.8 (-24 to -17)	--	--
	Blindness and vision loss	Both	3237 (2272 to 4408)	474.1 (339.7 to 635.3)	--	--	8638 (6081 to 11739)	353.9 (252 to 477.4)	--	--	166.8 (156.7 to 178.1)	-25.4 (-28.4 to -22.2)	--	--
		Female	1629 (1144 to 2198)	500.2 (361.6 to 669.4)	--	--	4454 (3156 to 6034)	374.1 (266.4 to 502.2)	--	--	173.5 (160.5 to 188.3)	-25.2 (-28.7 to -21.6)	--	--
		Male	1609 (1137 to 2195)	449.3 (320.6 to 601.6)	--	--	4184 (2942 to 5700)	334.6 (237.3 to 454.9)	--	--	160.1 (146.8 to 173.2)	-25.5 (-28.9 to -21.6)	--	--
	Age-related and other hearing loss	Both	3546 (2457 to 4939)	473.8 (338 to 653.2)	--	--	9935 (6802 to 14083)	385.2 (267.2 to 537.7)	--	--	180.2 (153.1 to 208)	-18.7 (-22.8 to -14.7)	--	--
		Female	1584 (1092 to 2188)	445.7 (315.2 to 608.4)	--	--	4515 (3107 to 6383)	362.6 (255.4 to 507.4)	--	--	185.1 (152.7 to 218.3)	-18.6 (-23.4 to -14.1)	--	--
		Male	1962 (1357 to 2731)	499.3 (353.1 to 688.8)	--	--	5420 (3698 to 7699)	407.3 (282.6 to 573.6)	--	--	176.2 (147.1 to 204)	-18.4 (-22.9 to -13.8)	--	--
	Other sense organ diseases	Both	403 (245 to 620)	42 (25.2 to 65.3)	--	--	1250 (742 to 1944)	43.6 (26 to 67.5)	--	--	209.8 (179.8 to 241.5)	3.8 (-1.5 to 9.1)	--	--
		Female	208 (125 to 318)	45.6 (27.5 to 70.5)	--	--	663 (390 to 1032)	47.1 (28 to 73.3)	--	--	218.5 (184.2 to 257.7)	3.3 (-3.8 to 10.4)	--	--
		Male	195 (117 to 305)	38.6 (23.1 to 59.9)	--	--	587 (342 to 916)	40.2 (23.7 to 62.1)	--	--	200.4 (169.1 to 236.9)	4 (-3.6 to 11.9)	--	--
	Musculoskeletal disorders	Both	17852 (12701 to 23741)	1921.9 (1383.2 to 2563.6)	4 (3 to 6)	0.7 (0.5 to 1.1)	63847 (46106 to 84730)	1980 (1428.9 to 2610.8)	21 (14 to 26)	0.9 (0.6 to 1.1)	257.7 (239.7 to 276.3)	3 (-0.1 to 6.3)	380.7 (236.3 to 670.1)	24.6 (-14.8 to 81.7)
		Female	9073 (6499 to 11935)	2082.1 (1502.5 to 2750.5)	3 (2 to 5)	1 (0.6 to 1.6)	34608 (25157 to 45739)	2191.8 (1589.7 to 2892.5)	15 (8 to 19)	1.4 (0.7 to 1.8)	281.4 (260.2 to 302.4)	5.3 (1.6 to 9.4)	382.2 (203.3 to 766.4)	31.4 (-20.9 to 117.5)
		Male	8778 (6200 to 11769)	1779.2 (1262.7 to 2381.7)	1 (1 to 2)	0.5 (0.3 to 0.6)	29239 (20909 to 38955)	1778.2 (1271.3 to 2352.9)	7 (5 to 8)	0.6 (0.4 to 0.7)	233.1 (213.1 to 253.7)	-0.1 (-4.2 to 4.2)	377.4 (212.4 to 669.1)	21.3 (-17.2 to 80)
	Rheumatoid arthritis	Both	123 (85 to 164)	13.8 (9.7 to 18.3)	1 (0 to 1)	0.1 (0.1 to 0.2)	500 (346 to 674)	15.5 (11.1 to 20.6)	3 (1 to 4)	0.1 (0.1 to 0.2)	307.8 (245.7 to 375.7)	12.2 (-4.5 to 30.3)	427.9 (192 to 844.1)	5.4 (-41.9 to 89.2)
		Female	85 (58 to 116)	20.1 (13.9 to 26.9)	0 (0 to 1)	0.2 (0.1 to 0.3)	353 (245 to 481)	22.2 (15.4 to 30)	2 (0 to 3)	0.2 (0 to 0.3)	316.8 (237.9 to 420.1)	10.6 (-10.5 to 35)	410 (83.5 to 1029)	4.6 (-63.9 to 137.6)

Province	Cause	Sex	1990				2019				1990-2019 percent change (%)			
			DALYs		Death		DALYs		Death		DALYs		Death	
			Number	Rate	Number	Rate	Number	Rate	Number	Rate	Number	Rate	Number	Rate
		Male	38 (26 to 52)	8.3 (5.8 to 11.1)	0 (0 to 0)	0.1 (0 to 0.1)	148 (104 to 199)	9.1 (6.5 to 12.2)	1 (1 to 1)	0.1 (0.1 to 0.1)	287.8 (227.4 to 361.7)	10.1 (-6.3 to 30.4)	469.1 (238.8 to 946)	20.1 (-26.7 to 111.8)
	Osteoarthritis	Both	1092 (548 to 2138)	181.5 (92 to 355.5)	--	--	5246 (2646 to 10411)	192.6 (97.2 to 377.2)	--	--	380.2 (362.3 to 400.8)	6.1 (2.7 to 10.2)	--	--
		Female	573 (289 to 1120)	205.9 (103.6 to 401.7)	--	--	2927 (1476 to 5827)	219.5 (111.5 to 431)	--	--	410.9 (387.4 to 438.7)	6.6 (2.3 to 11.7)	--	--
		Male	519 (259 to 1018)	159.7 (79.9 to 317.2)	--	--	2318 (1174 to 4529)	167 (84 to 329.2)	--	--	346.4 (322.4 to 373)	4.5 (-0.5 to 9.9)	--	--
	Low back pain	Both	10074 (6966 to 13548)	1013.8 (714.2 to 1368.6)	--	--	29020 (20209 to 39320)	907.9 (637.4 to 1212.6)	--	--	188.1 (167.7 to 208.1)	-10.4 (-14.4 to -6.3)	--	--
		Female	4516 (3116 to 6093)	960.7 (673.9 to 1294.2)	--	--	13979 (9751 to 18707)	900.6 (632.3 to 1201.5)	--	--	209.6 (182.5 to 238.7)	-6.3 (-12 to -0.2)	--	--
		Male	5558 (3848 to 7530)	1059.7 (746.3 to 1433.5)	--	--	15041 (10464 to 20387)	916.7 (644.3 to 1230.4)	--	--	170.6 (150.3 to 193.7)	-13.5 (-18.3 to -8)	--	--
	Neck pain	Both	3685 (2408 to 5379)	400.6 (263.8 to 579.8)	--	--	13589 (8910 to 19880)	404.1 (266 to 585.3)	--	--	268.8 (241.7 to 290.9)	0.9 (-1 to 2.9)	--	--
		Female	2129 (1403 to 3115)	493.6 (326.8 to 719.8)	--	--	8165 (5379 to 12002)	493.3 (328.8 to 719.1)	--	--	283.5 (253 to 311.1)	-0.1 (-2.6 to 2.4)	--	--
		Male	1555 (1016 to 2275)	318.9 (210.1 to 461.7)	--	--	5423 (3479 to 7974)	318.3 (208.5 to 461.8)	--	--	248.7 (220.9 to 273.1)	-0.2 (-3.3 to 3.1)	--	--
	Gout	Both	104 (64 to 151)	15.2 (9.3 to 21.9)	--	--	457 (286 to 672)	15.8 (9.9 to 23.2)	--	--	339.5 (274.2 to 408.5)	4.5 (-8.3 to 18.4)	--	--
		Female	20 (12 to 31)	6.5 (3.9 to 9.5)	--	--	100 (61 to 147)	7.1 (4.5 to 10.5)	--	--	387.7 (287.2 to 517)	9.6 (-10.5 to 36.1)	--	--
		Male	84 (51 to 122)	23 (14.1 to 33.4)	--	--	357 (223 to 532)	24.1 (14.8 to 35.3)	--	--	327.7 (254.7 to 407.5)	4.7 (-10.4 to 20.9)	--	--
	Other musculoskeletal disorders	Both	2774 (1908 to 3863)	297.1 (204.6 to 414.1)	4 (2 to 5)	0.6 (0.4 to 0.9)	15036 (10334 to 20588)	444.1 (306.9 to 607.8)	19 (13 to 23)	0.8 (0.5 to 1)	442 (392.1 to 496.8)	49.5 (38 to 62.8)	374.3 (232.1 to 685.7)	28.2 (-11.5 to 90.4)
		Female	1750 (1210 to 2406)	395.4 (275.4 to 539.5)	3 (1 to 4)	0.9 (0.5 to 1.3)	9084 (6261 to 12469)	549.2 (386.9 to 747.6)	13 (8 to 17)	1.2 (0.6 to 1.5)	419 (364.8 to 483.3)	38.9 (26 to 53.9)	378.4 (204.4 to 771.1)	36.7 (-15.3 to 129.6)
		Male	1024 (659 to 1469)	209.6 (136.1 to 300.8)	1 (1 to 2)	0.4 (0.3 to 0.5)	5952 (4029 to 8378)	343 (231.4 to 477.2)	6 (4 to 7)	0.5 (0.3 to 0.6)	481.5 (405.1 to 580.3)	63.7 (44.1 to 88.2)	365.2 (194.6 to 664.7)	21.5 (-18.2 to 81.4)

Province	Cause	Sex	1990				2019				1990-2019 percent change (%)			
			DALYs		Death		DALYs		Death		DALYs		Death	
			Number	Rate	Number	Rate	Number	Rate	Number	Rate	Number	Rate	Number	Rate
	Other non-communicable diseases	Both	70849 (43655 to 97194)	3533.1 (2375.7 to 4638.1)	636 (336 to 923)	27 (15.2 to 38.5)	55571 (42486 to 72001)	1808.2 (1394.6 to 2308.6)	215 (173 to 267)	8.8 (7.1 to 10.7)	-21.6 (-48.2 to 33.3)	-48.8 (-63.4 to -23.4)	-66.2 (-79.4 to -26.5)	-67.5 (-79.6 to -34.1)
		Female	34176 (21980 to 47126)	3889.4 (2721.2 to 5105.4)	265 (135 to 409)	23.7 (13.2 to 35)	37154 (27477 to 49193)	2342.4 (1763.9 to 3076.1)	96 (77 to 122)	8.4 (6.6 to 10.5)	8.7 (-27.9 to 68.7)	-39.8 (-55.3 to -18.2)	-63.7 (-78.5 to -20.9)	-64.4 (-78.4 to -29.1)
		Male	36672 (19908 to 53488)	3231.5 (1868.5 to 4517.6)	371 (179 to 564)	30 (15.2 to 44.7)	18417 (14129 to 23982)	1292.4 (994.7 to 1669.2)	118 (91 to 159)	9.2 (7.1 to 12.2)	-49.8 (-69.1 to 3.5)	-60 (-73.8 to -26.1)	-68 (-81 to -23.2)	-69.4 (-81.6 to -30.4)
	Congenital birth defects	Both	53018 (26353 to 77130)	2124.4 (1071.8 to 3073)	584 (280 to 858)	23.3 (11.3 to 34.1)	14276 (10999 to 19091)	543.8 (417.5 to 729)	140 (105 to 195)	5.4 (4 to 7.5)	-73.1 (-84.1 to -37.2)	-74.4 (-84.8 to -41)	-76.1 (-86.2 to -41.1)	-77 (-86.8 to -43.6)
		Female	21937 (10926 to 34627)	1809.9 (919.3 to 2841.7)	242 (117 to 387)	19.9 (9.6 to 31.6)	6124 (4585 to 8360)	478.7 (358.3 to 657.2)	60 (44 to 86)	4.8 (3.5 to 6.8)	-72.1 (-84.2 to -33)	-73.5 (-85 to -37.5)	-75.1 (-86.5 to -37)	-76.1 (-87.1 to -39.8)
		Male	31081 (14231 to 46996)	2422.3 (1130.7 to 3643.8)	342 (150 to 520)	26.5 (11.8 to 40.2)	8152 (6201 to 11848)	605.4 (459.1 to 884.4)	79 (58 to 122)	5.9 (4.3 to 9.1)	-73.8 (-84.9 to -29.5)	-75 (-85.6 to -33.4)	-76.8 (-87 to -32.5)	-77.7 (-87.5 to -36)
	Urinary diseases and male infertility	Both	308 (221 to 425)	29.5 (20.9 to 41)	2 (1 to 3)	0.3 (0.2 to 0.7)	956 (692 to 1287)	34.5 (25.8 to 45.8)	12 (8 to 14)	0.6 (0.5 to 0.8)	210.2 (172.5 to 254.7)	16.9 (-7.2 to 35.4)	537.5 (243.3 to 869.9)	127.9 (-21.6 to 289.4)
		Female	115 (83 to 150)	18.5 (13.7 to 25.1)	1 (1 to 2)	0.4 (0.2 to 1)	315 (215 to 402)	24.4 (16.6 to 30.5)	7 (4 to 9)	0.9 (0.6 to 1.1)	174.2 (117.8 to 254.1)	31.7 (-20.6 to 79.5)	522.6 (195.1 to 952.9)	129.1 (-19.9 to 341.8)
		Male	193 (128 to 293)	39.6 (26 to 58.3)	1 (1 to 1)	0.2 (0.1 to 0.5)	641 (436 to 921)	44.6 (31 to 63.8)	5 (4 to 6)	0.5 (0.3 to 0.6)	231.5 (187.3 to 280.9)	12.6 (-3.7 to 26.6)	561 (254.7 to 913.8)	162 (-19 to 359.2)
	Gynecological diseases	Female	5995 (4107 to 8394)	1086.3 (743.8 to 1510.7)	0 (0 to 0)	0 (0 to 0)	18921 (12858 to 26260)	1040.6 (709.1 to 1446)	0 (0 to 0)	0 (0 to 0)	215.6 (195.6 to 235)	-4.2 (-7.2 to -1.1)	84.7 (-22.2 to 1476.9)	-35.6 (-72 to 345.7)
	Hemoglobinopathies and hemolytic anemias	Both	2112 (1420 to 2998)	126.5 (86.6 to 180.5)	7 (5 to 9)	0.5 (0.4 to 0.6)	1628 (1091 to 2415)	56.6 (38.4 to 83.4)	7 (6 to 8)	0.3 (0.3 to 0.3)	-22.9 (-41.5 to 1)	-55.2 (-64.3 to -43.1)	6.3 (-23 to 60.1)	-38.7 (-51.6 to -15.7)
		Female	1251 (798 to 1803)	165.7 (106.1 to 243.2)	3 (2 to 4)	0.5 (0.3 to 0.6)	997 (621 to 1553)	68.7 (44.8 to 105.1)	3 (3 to 4)	0.3 (0.2 to 0.3)	-20.3 (-44 to 14.7)	-58.5 (-69.7 to -42.6)	8.7 (-31.2 to 89)	-38.9 (-55.8 to -5)
		Male	861 (571 to 1265)	90.2 (61.4 to 130.3)	4 (3 to 5)	0.5 (0.4 to 0.6)	631 (424 to 949)	45 (29.9 to 68.7)	4 (3 to 5)	0.3 (0.3 to 0.4)	-26.7 (-48.8 to 6.1)	-50.1 (-64.8 to -28.2)	4.6 (-27.8 to 64.6)	-37.7 (-54.5 to -10.4)
	Endocrine, metabolic, blood, and immune disorders	Both	4759 (3491 to 6353)	352.4 (257.6 to 471.9)	22 (15 to 31)	2.1 (1.5 to 2.7)	10077 (7257 to 13498)	337.8 (246.3 to 447.9)	52 (33 to 64)	2.4 (1.5 to 2.9)	111.7 (69.2 to 152.4)	-4.1 (-16.7 to 6.4)	132.8 (31.2 to 257.8)	11.7 (-27.2 to 63)
		Female	2772 (1970 to 3746)	441 (309.5 to 591.9)	10 (6 to 15)	2.3 (1.5 to 3.3)	6166 (4322 to 8503)	417 (298.3 to 569)	24 (13 to 31)	2.4 (1.3 to 3.1)	122.4 (79 to 164.7)	-5.4 (-17.2 to 6.4)	131.1 (19.3 to 315.2)	4.7 (-39.9 to 77.5)
		Male	1986 (1413 to 2750)	274 (202.2 to 368.1)	12 (7 to 19)	1.9 (1.4 to 2.7)	3910 (2833 to 5162)	262.4 (191.3 to 341.2)	28 (15 to 37)	2.4 (1.3 to 3.1)	96.8 (44.1 to 151.4)	-4.2 (-21.3 to 11.3)	134.2 (29.2 to 269.7)	23.3 (-20.2 to 85.5)
	Oral disorders	Both	2840 (1656 to 4568)	315.9 (189.1 to 498.5)	--	--	9373 (5525 to 15063)	311.4 (184.2 to 491.1)	--	--	230 (214.7 to 246.9)	-1.4 (-3.5 to 0.6)	--	--
		Female	1333 (777 to 2117)	305.9 (181.7 to 484.4)	--	--	4473 (2581 to 7273)	300.5 (177.1 to 482)	--	--	235.5 (217.9 to 253.1)	-1.8 (-4.8 to 1.2)	--	--
		Male	1507 (884 to 2431)	325.4 (196.2 to 511.2)	--	--	4900 (2913 to 7840)	321.5 (192.6 to 504.5)	--	--	225.2 (209.3 to 243.3)	-1.2 (-4 to 1.6)	--	--

Province	Cause	Sex	1990				2019				1990-2019 percent change (%)			
			DALYs		Death		DALYs		Death		DALYs		Death	
			Number	Rate	Number	Rate	Number	Rate	Number	Rate	Number	Rate	Number	Rate
	Sudden infant death syndrome	Both	1816 (443 to 5632)	71.2 (17.4 to 220.9)	21 (5 to 64)	0.8 (0.2 to 2.5)	341 (163 to 638)	13 (6.2 to 24.3)	4 (2 to 7)	0.1 (0.1 to 0.3)	-81.2 (-94.2 to 1.6)	-81.8 (-94.4 to -1.5)	-81.2 (-94.2 to 1.7)	-81.8 (-94.4 to -1.4)
		Female	772 (210 to 2432)	62.1 (16.9 to 195.5)	9 (2 to 28)	0.7 (0.2 to 2.2)	159 (68 to 329)	12.4 (5.4 to 25.7)	2 (1 to 4)	0.1 (0.1 to 0.3)	-79.5 (-94.1 to 2.1)	-80 (-94.3 to -0.6)	-79.5 (-94.1 to 2)	-80 (-94.3 to -0.7)
		Male	1044 (206 to 3474)	79.9 (15.8 to 265.9)	12 (2 to 39)	0.9 (0.2 to 3)	182 (81 to 360)	13.5 (6 to 26.7)	2 (1 to 4)	0.2 (0.1 to 0.3)	-82.6 (-94.9 to 5.4)	-83.1 (-95 to 2)	-82.6 (-94.9 to 5.5)	-83.1 (-95 to 2)
	**Injuries**	Both	77006 (56080 to 91410)	4903.3 (3626.4 to 5779.8)	1066 (714 to 1283)	81.5 (57.8 to 98.3)	77649 (67659 to 88821)	2527.3 (2212.3 to 2876.1)	1253 (1076 to 1455)	43.5 (37.6 to 50.5)	0.8 (-18.6 to 44)	-48.5 (-57.8 to -27.3)	17.5 (-8.4 to 78.7)	-46.6 (-58 to -22)
		Female	25249 (18808 to 31353)	3191.7 (2337.3 to 3939.7)	333 (230 to 422)	51.7 (35.6 to 66.2)	20327 (17058 to 23877)	1420.1 (1206.1 to 1654.6)	302 (247 to 360)	23.9 (19.8 to 28.4)	-19.5 (-38.3 to 14.6)	-55.5 (-65.2 to -38)	-9.2 (-33.4 to 42)	-53.7 (-65.8 to -30.7)
		Male	51757 (36723 to 62567)	6463.4 (4729.2 to 7829.4)	734 (485 to 903)	108.6 (75.7 to 136.2)	57321 (49002 to 66612)	3607.2 (3094.3 to 4173.1)	951 (783 to 1148)	62.5 (51.8 to 75.5)	10.8 (-12.9 to 63.9)	-44.2 (-55.9 to -19.8)	29.6 (-2.2 to 102.2)	-42.4 (-56.9 to -15.2)
	Transport injuries	Both	34275 (23690 to 42996)	2143.2 (1501.7 to 2688.9)	504 (345 to 645)	38.3 (26.8 to 50.3)	28090 (22206 to 41853)	934.3 (741.7 to 1376.8)	521 (389 to 822)	18.2 (13.6 to 28.3)	-18 (-38.1 to 21.8)	-56.4 (-66.2 to -36.1)	3.3 (-23.4 to 59.4)	-52.5 (-64.9 to -29.3)
		Female	11490 (7459 to 15173)	1431.6 (952.3 to 1843.7)	161 (101 to 212)	24.3 (15.1 to 31.9)	7543 (6085 to 9082)	523.4 (422.1 to 626.4)	134 (103 to 165)	9.9 (7.6 to 12.1)	-34.4 (-53 to 0.8)	-63.4 (-73 to -44.2)	-16.8 (-42.1 to 34.3)	-59.2 (-71.1 to -33.4)
		Male	22785 (14954 to 30807)	2794.8 (1867.1 to 3814)	343 (223 to 478)	51.2 (32.8 to 74)	20548 (15311 to 34070)	1333.9 (1002.9 to 2179.6)	387 (273 to 682)	26.1 (18.3 to 45.6)	-9.8 (-34.3 to 37.8)	-52.3 (-65 to -28.5)	12.8 (-21.2 to 80)	-48.9 (-64.5 to -20.6)
	Road injuries	Both	33190 (22996 to 41920)	2063.5 (1442.8 to 2590.2)	487 (333 to 623)	36.8 (25.9 to 48.4)	26749 (20901 to 40093)	890.8 (698.8 to 1315.6)	497 (367 to 786)	17.4 (12.9 to 27.1)	-19.4 (-39.3 to 19.9)	-56.8 (-66.6 to -36.5)	2.1 (-24.8 to 58.1)	-52.8 (-65 to -29.4)
		Female	11220 (7266 to 14826)	1393.8 (927.1 to 1799)	157 (99 to 207)	23.7 (14.8 to 31.2)	7234 (5838 to 8710)	502.1 (404.5 to 600.9)	129 (99 to 159)	9.6 (7.3 to 11.7)	-35.5 (-54.1 to -0.9)	-64 (-73.6 to -45.3)	-17.8 (-43.1 to 31.8)	-59.6 (-71.5 to -34.4)
		Male	21970 (14427 to 29817)	2677.3 (1789 to 3671.7)	330 (214 to 462)	48.9 (31.3 to 71.3)	19515 (14471 to 32329)	1268.9 (946.8 to 2073.4)	368 (255 to 650)	24.9 (17.3 to 43.4)	-11.2 (-35.9 to 35.6)	-52.6 (-65.3 to -28.9)	11.6 (-22.4 to 77.6)	-49.1 (-64.5 to -21.3)
	Other transport injuries	Both	1085 (637 to 1708)	79.7 (46.4 to 123.6)	17 (9 to 28)	1.5 (0.8 to 2.4)	1341 (1052 to 1896)	43.5 (34.1 to 60.7)	23 (17 to 35)	0.8 (0.6 to 1.2)	23.6 (-17.4 to 106.7)	-45.5 (-63.6 to -9.6)	39.8 (-11.4 to 161.6)	-45.9 (-65.8 to 0.3)
		Female	270 (153 to 442)	37.9 (22.3 to 60.8)	4 (2 to 6)	0.6 (0.3 to 1)	308 (242 to 395)	21.3 (16.8 to 27.4)	5 (3 to 6)	0.3 (0.3 to 0.5)	14.2 (-24.8 to 96.8)	-43.8 (-61.9 to -3.4)	27.6 (-23.8 to 172.5)	-41.8 (-64.5 to 17.4)
		Male	815 (419 to 1442)	117.5 (61.1 to 201.5)	13 (6 to 24)	2.3 (1 to 4.1)	1033 (764 to 1568)	65 (48.6 to 97.1)	19 (13 to 30)	1.2 (0.9 to 2)	26.8 (-24.3 to 148.5)	-44.6 (-66.3 to 7.2)	43.1 (-17.3 to 222.1)	-45.6 (-68.8 to 14.5)
	Unintentional injuries	Both	29832 (21914 to 36291)	1866 (1389.7 to 2261.9)	368 (250 to 453)	28.1 (20.2 to 34.3)	29264 (23136 to 35074)	966.5 (769.6 to 1147.6)	396 (297 to 468)	14.9 (11.4 to 17.5)	-1.9 (-21.5 to 33.2)	-48.2 (-57 to -31.8)	7.7 (-15.8 to 59.6)	-47 (-58.1 to -24.6)
		Female	10119 (7429 to 12785)	1260.1 (956.9 to 1561)	121 (83 to 157)	19.6 (13.6 to 24.7)	8182 (6559 to 10142)	589 (479.2 to 721.6)	103 (82 to 130)	9.5 (7.7 to 11.9)	-19.1 (-40.4 to 14.9)	-53.3 (-63.8 to -37.5)	-14.7 (-39.5 to 29.2)	-51.5 (-64.1 to -30.7)
		Male	19714 (13890 to 25110)	2416.8 (1716.7 to 3051.2)	247 (157 to 320)	35.8 (23.1 to 46.6)	21082 (15996 to 25699)	1334.6 (1008 to 1615.6)	293 (200 to 360)	20.2 (13.9 to 24.8)	6.9 (-15.9 to 46.4)	-44.8 (-55.7 to -26)	18.7 (-11.9 to 79.5)	-43.6 (-58.1 to -17.7)
	Falls	Both	6245 (4216 to 8589)	493.6 (348.5 to 654.2)	68 (40 to 99)	7.4 (4.7 to 10.1)	10483 (7728 to 13549)	348.8 (258.3 to 444.9)	123 (78 to 155)	5.3 (3.4 to 6.6)	67.9 (25.8 to 118.1)	-29.3 (-41.9 to -11.9)	80.7 (28.7 to 176)	-29.3 (-45.4 to 2)
		Female	1569 (973 to 2292)	266.6 (193.7 to 341.8)	16 (9 to 24)	4.9 (3.1 to 6.6)	2322 (1761 to 3020)	178.4 (138.4 to 226.8)	27 (19 to 33)	3.3 (2.3 to 4.1)	48 (-0.1 to 123.3)	-33.1 (-46.3 to -16.8)	63 (6.5 to 171.1)	-32.9 (-52.4 to -2.1)
		Male	4676 (2870 to 6697)	697.3 (462.8 to 956.6)	51 (26 to 80)	9.7 (5.5 to 14)	8161 (5854 to 10600)	515.3 (366.2 to 665.6)	96 (56 to 128)	7.2 (4.1 to 9.5)	74.5 (25.8 to 153.9)	-26.1 (-42.4 to -3.7)	86.4 (22.6 to 229.7)	-25.7 (-46.9 to 14)

Province	Cause	Sex	1990				2019				1990-2019 percent change (%)			
			DALYs		Death		DALYs		Death		DALYs		Death	
			Number	Rate	Number	Rate	Number	Rate	Number	Rate	Number	Rate	Number	Rate
	Drowning	Both	4829 (3192 to 6809)	225.3 (146.5 to 306.5)	62 (40 to 86)	3.2 (2.1 to 4.3)	1963 (1550 to 2326)	71.4 (56.1 to 85.1)	33 (26 to 39)	1.2 (0.9 to 1.4)	-59.3 (-72.6 to -35.6)	-68.3 (-78 to -48.8)	-47.5 (-63.5 to -14.3)	-63.4 (-73.5 to -39.7)
		Female	1798 (1032 to 2737)	165.3 (96.4 to 242.6)	22 (13 to 34)	2.3 (1.3 to 3.3)	517 (414 to 648)	40.1 (32.1 to 50.7)	8 (7 to 10)	0.6 (0.5 to 0.8)	-71.3 (-82.8 to -45.9)	-75.7 (-84.9 to -53.1)	-63.8 (-77.4 to -29.1)	-71.8 (-81.5 to -46.9)
		Male	3031 (1697 to 4584)	281.3 (160.2 to 414.5)	40 (22 to 59)	4.1 (2.4 to 5.9)	1446 (1050 to 1775)	101.8 (73 to 125.6)	25 (18 to 30)	1.7 (1.3 to 2.1)	-52.3 (-70.1 to -18.7)	-63.8 (-76.6 to -39.2)	-38.2 (-60.3 to 5.1)	-58.4 (-72.2 to -29.4)
	Fire, heat, and hot substances	Both	3194 (1743 to 4449)	191.8 (108.8 to 260.4)	45 (24 to 63)	3.6 (1.9 to 5)	2399 (1811 to 3505)	80 (60.2 to 118.3)	47 (34 to 73)	1.8 (1.3 to 2.8)	-24.9 (-51.8 to 49.4)	-58.3 (-72.2 to -22.8)	4.8 (-34.3 to 117.2)	-50.6 (-67.2 to -8.9)
		Female	1159 (503 to 2042)	151.7 (73.4 to 260.3)	16 (6 to 30)	2.9 (1.3 to 5)	1025 (629 to 1936)	72.8 (44.1 to 138.8)	20 (11 to 40)	1.7 (0.9 to 3.4)	-11.6 (-58.7 to 144.2)	-52 (-75.6 to 8.3)	20.4 (-47.6 to 227.6)	-43 (-70.4 to 7.8)
		Male	2035 (1074 to 2890)	228.9 (131.4 to 312.9)	29 (15 to 41)	4.2 (2.4 to 5.7)	1374 (1038 to 2029)	87.3 (66.6 to 132.9)	28 (20 to 42)	1.9 (1.4 to 3)	-32.4 (-56.3 to 65.3)	-61.8 (-74.8 to -14.7)	-4 (-38.8 to 129.9)	-54.5 (-69.9 to -4.7)
	Poisonings	Both	4250 (1885 to 6069)	231.4 (103.5 to 320.5)	60 (26 to 84)	4 (1.7 to 5.6)	2617 (1341 to 3293)	84.9 (44.5 to 106)	51 (24 to 65)	1.7 (0.9 to 2.1)	-38.4 (-57.8 to 3.2)	-63.3 (-73.9 to -38.3)	-14.9 (-40.4 to 47.5)	-57.3 (-69.5 to -31.2)
		Female	1829 (758 to 2819)	197.5 (77.6 to 291)	25 (10 to 38)	3.3 (1.3 to 4.7)	881 (647 to 1124)	62.6 (46.8 to 79.7)	17 (12 to 21)	1.3 (0.9 to 1.6)	-51.8 (-70.7 to -7.6)	-68.3 (-79.7 to -43.1)	-33.6 (-57.8 to 20.4)	-62.5 (-74.3 to -34.5)
		Male	2422 (833 to 3925)	261.9 (88.3 to 421.3)	35 (11 to 57)	4.6 (1.4 to 7.4)	1736 (546 to 2380)	106.7 (34.5 to 144.7)	34 (10 to 47)	2.2 (0.6 to 2.9)	-28.3 (-54.7 to 25.9)	-59.3 (-74 to -27.4)	-1.4 (-37.3 to 80.3)	-53.1 (-70.5 to -18.9)
	Exposure to mechanical forces	Both	2866 (1859 to 4182)	207.8 (138.5 to 296.6)	32 (18 to 53)	2.5 (1.4 to 4.3)	4462 (3563 to 5713)	142.3 (114.1 to 181.4)	51 (40 to 68)	1.7 (1.3 to 2.2)	55.7 (8.5 to 134.2)	-31.5 (-49.8 to -2)	59.2 (2.9 to 201.8)	-33.6 (-57.4 to 21.5)
		Female	704 (422 to 1221)	100.6 (68.5 to 147.9)	6 (3 to 12)	1 (0.6 to 1.7)	1115 (741 to 1518)	75.6 (50.4 to 102.5)	10 (4 to 14)	0.7 (0.4 to 1)	58.4 (-6.9 to 139)	-24.8 (-47 to 0.7)	49.2 (-27.8 to 187.3)	-27.8 (-58.3 to 22.6)
		Male	2162 (1303 to 3310)	304.8 (190.3 to 461.8)	25 (12 to 44)	3.9 (2 to 7.1)	3347 (2608 to 4348)	207.1 (161.9 to 265.8)	41 (30 to 59)	2.6 (1.9 to 3.6)	54.8 (7.4 to 152.5)	-32 (-52.4 to 4.5)	61.7 (-2.4 to 254.8)	-33.4 (-60.4 to 36.8)
	Adverse effects of medical treatment	Both	1188 (842 to 1515)	83.4 (57.6 to 110.7)	20 (14 to 26)	2.2 (1.5 to 3.3)	929 (749 to 1248)	32.4 (26.2 to 44.1)	24 (19 to 33)	1 (0.8 to 1.4)	-21.8 (-43.9 to 19.4)	-61.2 (-73.8 to -40)	19.9 (-21.8 to 89.2)	-55.6 (-73.4 to -29.1)
		Female	505 (312 to 704)	73.6 (46.3 to 101.3)	8 (5 to 12)	2 (1.2 to 3.5)	344 (268 to 476)	25.7 (20.1 to 35.7)	9 (7 to 13)	0.8 (0.6 to 1.2)	-31.8 (-56.4 to 21.9)	-65.1 (-77.7 to -40.4)	4.9 (-34.5 to 81.6)	-58.9 (-78.2 to -25.8)
		Male	683 (460 to 930)	92.2 (60.6 to 130.9)	12 (7 to 16)	2.4 (1.6 to 4.1)	585 (440 to 824)	38.9 (29.6 to 55.9)	15 (11 to 23)	1.2 (0.8 to 1.8)	-14.4 (-44.4 to 33.7)	-57.8 (-73.7 to -32.7)	30.9 (-22.9 to 113.2)	-52.5 (-73.3 to -18.9)
	Animal contact	Both	285 (170 to 423)	16.4 (10.6 to 22.8)	3 (1 to 5)	0.2 (0.1 to 0.3)	260 (189 to 350)	8.7 (6.4 to 11.7)	2 (1 to 2)	0.1 (0 to 0.1)	-8.9 (-39.2 to 41)	-46.7 (-61.4 to -26.6)	-36.1 (-58.7 to 15.3)	-60.6 (-73 to -36.3)
		Female	86 (52 to 129)	11.3 (7.4 to 16.6)	1 (0 to 1)	0.1 (0 to 0.2)	118 (84 to 167)	8 (5.7 to 11.3)	0 (0 to 1)	0 (0 to 0.1)	37.7 (-7.8 to 110.7)	-29.4 (-46.4 to -6.1)	-29.9 (-60.4 to 69.1)	-54.8 (-72.2 to -9.1)
		Male	199 (92 to 335)	21.1 (11.5 to 33.1)	2 (1 to 4)	0.3 (0.1 to 0.5)	142 (100 to 188)	9.4 (6.7 to 12.5)	1 (1 to 2)	0.1 (0 to 0.2)	-28.9 (-56 to 27.8)	-55.4 (-70.2 to -29.2)	-37.9 (-61.9 to 15.8)	-62.4 (-75.3 to -36.4)
	Foreign body	Both	1952 (1333 to 2839)	93.4 (67.2 to 128.9)	21 (14 to 31)	1.1 (0.8 to 1.5)	1094 (864 to 1382)	37.6 (29.9 to 46.7)	11 (10 to 14)	0.5 (0.4 to 0.6)	-43.9 (-64.3 to -14.4)	-59.8 (-72.9 to -42.7)	-46 (-65.6 to -12.8)	-58.5 (-71.3 to -37.8)
		Female	910 (540 to 1516)	87.8 (57.2 to 134.9)	10 (6 to 17)	1 (0.7 to 1.6)	431 (335 to 543)	30.9 (24.2 to 38.4)	4 (4 to 5)	0.4 (0.3 to 0.5)	-52.6 (-74.4 to -17.4)	-64.8 (-79.6 to -43.9)	-56 (-76.8 to -19.2)	-62.5 (-77.7 to -39.7)
		Male	1042 (669 to 1597)	98.6 (68 to 142)	11 (7 to 17)	1.1 (0.8 to 1.6)	663 (516 to 853)	44 (34.5 to 56.2)	7 (6 to 9)	0.5 (0.4 to 0.7)	-36.4 (-61.5 to 2.6)	-55.3 (-71.1 to -34.6)	-37.2 (-62.7 to 14.9)	-54.6 (-70.9 to -26.9)
	Environmental heat and cold exposure	Both	191 (138 to 267)	15.4 (11.1 to 21.3)	1 (1 to 2)	0.1 (0.1 to 0.3)	360 (251 to 502)	11.5 (8 to 15.9)	1 (1 to 2)	0.1 (0 to 0.1)	88.3 (54.1 to 116.9)	-25.4 (-37.6 to -15.5)	35.9 (-3.8 to 127.9)	-55.4 (-69.3 to -29.9)
		Female	84 (59 to 115)	14.2 (10.1 to 19.5)	0 (0 to 1)	0.1 (0 to 0.2)	175 (121 to 246)	11.4 (7.9 to 15.9)	0 (0 to 1)	0 (0 to 0.1)	109 (69.6 to 137.8)	-20.3 (-33 to -11.5)	39.1 (-9.2 to 145.1)	-49.1 (-70.8 to -5.7)

Province	Cause	Sex	1990				2019				1990-2019 percent change (%)			
			DALYs		Death		DALYs		Death		DALYs		Death	
			Number	Rate	Number	Rate	Number	Rate	Number	Rate	Number	Rate	Number	Rate
		Male	107 (77 to 158)	16.4 (11.9 to 24.3)	1 (0 to 2)	0.2 (0.1 to 0.4)	185 (130 to 259)	11.6 (8.2 to 16.2)	1 (1 to 2)	0.1 (0 to 0.1)	72.3 (36.6 to 104.3)	-29.5 (-43.8 to -17.6)	34.6 (-11.6 to 134.1)	-58 (-73.2 to -30.2)
	Exposure to forces of nature	Both	55 (46 to 66)	3.8 (3.1 to 4.5)	1 (0 to 1)	0 (0 to 0)	44 (33 to 61)	1.4 (1 to 1.9)	0 (0 to 0)	0 (0 to 0)	-19.1 (-31.1 to -5.6)	-63.2 (-68.2 to -57.7)	-66.9 (-66.9 to -66.9)	-85.4 (-85.4 to -85.4)
		Female	20 (17 to 24)	2.8 (2.3 to 3.4)	0 (0 to 0)	0 (0 to 0)	16 (11 to 22)	1 (0.8 to 1.4)	0 (0 to 0)	0 (0 to 0)	-21.3 (-34.3 to -6)	-63.9 (-69 to -57.9)	-67.8 (-67.8 to -67.8)	-85.4 (-85.4 to -85.4)
		Male	35 (29 to 42)	4.6 (3.9 to 5.6)	0 (0 to 0)	0.1 (0 to 0.1)	29 (22 to 39)	1.7 (1.3 to 2.3)	0 (0 to 0)	0 (0 to 0)	-17.9 (-29.5 to -4.5)	-62.4 (-67.4 to -56.7)	-66.4 (-66.4 to -66.4)	-85.1 (-85.1 to -85.1)
	Other unintentional injuries	Both	4779 (2551 to 7336)	303.8 (171.2 to 455.1)	55 (24 to 92)	3.6 (1.6 to 6)	4653 (2870 to 5990)	147.5 (91.2 to 189.4)	53 (25 to 70)	1.7 (0.8 to 2.2)	-2.6 (-37.8 to 45.6)	-51.5 (-67.3 to -29.7)	-4 (-45.8 to 69.3)	-53.2 (-73.2 to -17.3)
		Female	1456 (901 to 2167)	188.6 (123 to 267.1)	15 (7 to 24)	1.9 (1 to 3)	1238 (896 to 1691)	82.6 (60.2 to 111.8)	8 (6 to 11)	0.6 (0.5 to 0.8)	-15 (-46.9 to 37.7)	-56.2 (-70 to -36.1)	-44.5 (-69.1 to 17.1)	-67.4 (-81.3 to -34.6)
		Male	3323 (1516 to 5731)	409.5 (207.9 to 680.9)	40 (14 to 78)	5.2 (1.8 to 10)	3414 (1792 to 4487)	210.7 (110.7 to 274.4)	45 (16 to 61)	2.8 (1 to 3.8)	2.8 (-42.3 to 65.4)	-48.6 (-69.8 to -20.1)	10.7 (-46.1 to 118.7)	-47.1 (-74.4 to 2.4)
	Self-harm and interpersonal violence	Both	12899 (7234 to 16318)	894 (528.5 to 1134.8)	195 (104 to 253)	15.1 (8.8 to 19.4)	20294 (14911 to 24118)	626.6 (463.4 to 739)	336 (237 to 407)	10.4 (7.4 to 12.6)	57.3 (22.1 to 190.7)	-29.9 (-45.5 to 20.5)	72.5 (30 to 236.4)	-30.8 (-48 to 21)
		Female	3640 (1949 to 4841)	500 (274.8 to 659.6)	51 (24 to 71)	7.8 (4.1 to 10.7)	4603 (3775 to 5616)	307.7 (253 to 373.4)	65 (51 to 82)	4.5 (3.6 to 5.6)	26.4 (-10.6 to 132.2)	-38.5 (-56.1 to 10)	27.4 (-13.3 to 165.9)	-42.3 (-60.5 to 12.6)
		Male	9259 (4837 to 12346)	1251.8 (699.1 to 1676.6)	144 (71 to 198)	21.6 (11.2 to 29.9)	15692 (10080 to 19338)	938.7 (602.1 to 1152.2)	271 (165 to 342)	16.2 (10 to 20.3)	69.5 (23.7 to 209.7)	-25 (-45 to 28.1)	88.6 (33.1 to 265.2)	-25.1 (-46.7 to 29.5)
	Self-harm	Both	6448 (2925 to 8757)	462.3 (220.1 to 622.9)	110 (50 to 149)	8.8 (4.2 to 12)	11111 (6206 to 14198)	337.8 (192.5 to 426.9)	209 (116 to 269)	6.5 (3.6 to 8.2)	72.3 (26.7 to 276.8)	-26.9 (-45.8 to 39.8)	90.5 (38.7 to 293)	-26.8 (-46.3 to 30)
		Female	1829 (744 to 2601)	261.5 (116.1 to 365.1)	29 (11 to 42)	4.6 (2.1 to 6.4)	2146 (1628 to 2720)	144.1 (110 to 184)	37 (28 to 48)	2.6 (2 to 3.3)	17.3 (-24.8 to 174.6)	-44.9 (-64 to 18.6)	28.1 (-18.4 to 210.3)	-43.9 (-63.3 to 17.3)
		Male	4619 (1433 to 6797)	644.5 (216.5 to 945.2)	81 (26 to 119)	12.7 (4.5 to 18.5)	8965 (4013 to 12106)	527.6 (234.5 to 706.7)	172 (77 to 232)	10.2 (4.6 to 13.7)	94.1 (34.5 to 326.8)	-18.1 (-42.5 to 58.1)	113.1 (47.2 to 343.2)	-19.2 (-43.9 to 59)
	Interpersonal violence	Both	5243 (2986 to 6734)	350.3 (209.4 to 443.4)	73 (38 to 96)	5.3 (2.9 to 7)	7243 (5962 to 8621)	230 (188.2 to 275.6)	102 (80 to 125)	3.2 (2.5 to 3.9)	38.1 (3.2 to 140.4)	-34.3 (-50.4 to 11.3)	38.7 (-1.5 to 173.8)	-39.8 (-57.7 to 13.2)
		Female	1701 (1038 to 2451)	222.9 (139 to 309.4)	21 (11 to 32)	2.9 (1.6 to 4.3)	2253 (1778 to 2831)	150.1 (118.4 to 189.1)	25 (18 to 34)	1.7 (1.3 to 2.3)	32.5 (-13.2 to 127.9)	-32.7 (-53.8 to 13)	21.9 (-28.2 to 155.7)	-41.2 (-63.2 to 20.4)
		Male	3543 (1806 to 4774)	465.6 (249.5 to 620.3)	52 (25 to 73)	7.5 (3.8 to 10.4)	4990 (3935 to 6152)	308.1 (241.5 to 382.6)	76 (57 to 97)	4.6 (3.5 to 5.9)	40.9 (-4 to 161.5)	-33.8 (-55 to 17.7)	45.3 (-6.1 to 192.6)	-37.7 (-60.6 to 21.7)
	Conflict and terrorism	Both	540 (375 to 786)	34.2 (23.8 to 50.1)	2 (2 to 2)	0.1 (0.1 to 0.1)	453 (295 to 701)	14.4 (9.4 to 22.4)	1 (1 to 1)	0 (0 to 0)	-16.1 (-27.3 to -1.2)	-57.9 (-63.5 to -50.1)	-44.5 (-44.5 to -44.5)	-69.2 (-69.2 to -69.2)
		Female	15 (10 to 25)	2.2 (1.4 to 3.5)	0 (0 to 0)	0 (0 to 0)	44 (30 to 76)	3.1 (2.1 to 5.4)	0 (0 to 0)	0 (0 to 0)	185 (117 to 310.3)	42.3 (7.2 to 110.2)	606.8 (606.8 to 606.8)	275.9 (275.9 to 275.9)
		Male	524 (364 to 763)	63.8 (44.5 to 93.1)	2 (2 to 2)	0.2 (0.2 to 0.3)	409 (264 to 638)	25.4 (16.5 to 39.5)	1 (1 to 1)	0.1 (0 to 0.1)	-22 (-32.5 to -10.3)	-60.2 (-65.6 to -54.2)	-58.5 (-58.5 to -58.5)	-77 (-77 to -77)
	Executions and police conflict	Both	668 (502 to 900)	47.2 (34.8 to 63.6)	10 (7 to 14)	0.8 (0.6 to 1.1)	1487 (1044 to 2145)	44.3 (32.2 to 62.9)	24 (16 to 37)	0.7 (0.5 to 1.1)	122.6 (49.4 to 240.9)	-6.1 (-35.7 to 42.3)	146.8 (56.3 to 303.8)	-7.8 (-39.5 to 49.5)
		Female	95 (84 to 108)	13.5 (11.9 to 15.3)	1 (1 to 1)	0.2 (0.2 to 0.2)	159 (138 to 185)	10.4 (9.1 to 12.1)	2 (2 to 3)	0.2 (0.1 to 0.2)	67.6 (58.2 to 77.9)	-22.7 (-26.7 to -18.3)	81.2 (74 to 88.9)	-30.2 (-33 to -27.5)
		Male	573 (410 to 803)	77.9 (54.7 to 108.4)	9 (6 to 13)	1.3 (0.9 to 1.9)	1328 (883 to 1989)	77.6 (53.6 to 114.9)	22 (14 to 35)	1.3 (0.8 to 1.9)	131.7 (47.1 to 271.2)	-0.5 (-35.8 to 59.2)	156.3 (53.7 to 341.8)	-1.1 (-38.5 to 70.5)

Province	Cause	Sex	1990				2019				1990-2019 percent change (%)			
			DALYs		Death		DALYs		Death		DALYs		Death	
			Number	Rate	Number	Rate	Number	Rate	Number	Rate	Number	Rate	Number	Rate
**Ardebil**	**All causes**	Both	560246 (497375 to 623945)	49552.9 (44680.6 to 54845.3)	7552 (6741 to 8471)	1065.7 (953.8 to 1187.8)	324501 (282712 to 372598)	26569.9 (23324.7 to 30203.7)	7142 (6585 to 7671)	687.3 (636.8 to 736.3)	-42.1 (-49.7 to -33.7)	-46.4 (-52.1 to -40.5)	-5.4 (-17.6 to 8.5)	-35.5 (-43.4 to -27.1)
		Female	251565 (222986 to 280821)	45593.1 (40416.5 to 51015.8)	3150 (2769 to 3529)	920.2 (806.6 to 1045.9)	146681 (124824 to 172806)	23896.5 (20537.7 to 27890.6)	2852 (2601 to 3092)	542 (496.5 to 584)	-41.7 (-49.8 to -32.2)	-47.6 (-53.9 to -41.2)	-9.5 (-22.1 to 6.3)	-41.1 (-49.2 to -31.1)
		Male	308681 (273114 to 348267)	53104 (47232.9 to 60121.6)	4402 (3832 to 5076)	1199.2 (1027.4 to 1400.6)	177819 (155831 to 201360)	29367.5 (25935.8 to 32960.8)	4290 (3828 to 4755)	841 (756.2 to 922.8)	-42.4 (-50.7 to -33.4)	-44.7 (-52.3 to -36.8)	-2.5 (-18.4 to 15.5)	-29.9 (-41.4 to -15.4)
	**Communicable, maternal, neonatal, and nutritional diseases**	Both	180629 (139435 to 239251)	10897.3 (8477 to 14386.9)	2022 (1536 to 2733)	135.4 (105.3 to 189.6)	24561 (20797 to 28841)	2264.2 (1891.1 to 2673.3)	273 (231 to 370)	27.4 (23.1 to 36.8)	-86.4 (-90.1 to -81.5)	-79.2 (-85.2 to -72)	-86.5 (-90.6 to -80.7)	-79.8 (-86 to -72.1)
		Female	84334 (64641 to 111410)	10502.6 (8200 to 13904.7)	939 (711 to 1270)	128.9 (99.6 to 177.2)	11292 (9422 to 13431)	2105.2 (1754.8 to 2508.8)	117 (96 to 164)	23.3 (19 to 32.7)	-86.6 (-90.5 to -81.6)	-80 (-85.9 to -72.8)	-87.6 (-91.4 to -80.7)	-82 (-87.2 to -73.7)
		Male	96295 (71663 to 133131)	11256.4 (8433.7 to 15656)	1083 (796 to 1530)	141.3 (105.6 to 203.2)	13269 (11159 to 15755)	2419.7 (2021.5 to 2884.2)	156 (132 to 210)	31.7 (26.8 to 43.1)	-86.2 (-90.6 to -80.8)	-78.5 (-85.3 to -70.3)	-85.6 (-90.3 to -79)	-77.6 (-85.1 to -68.9)
	HIV/AIDS and sexually transmitted infections	Both	380 (181 to 759)	28.9 (16.2 to 51.3)	3 (1 to 8)	0.2 (0.1 to 0.5)	543 (374 to 752)	41.4 (27.7 to 57.8)	7 (5 to 10)	0.6 (0.4 to 0.8)	42.8 (-25.1 to 183.8)	43.2 (-13.9 to 133.2)	111.5 (-6.5 to 497.4)	153.1 (25.9 to 455.8)
		Female	193 (98 to 368)	32.5 (18.6 to 54.5)	2 (1 to 3)	0.2 (0.1 to 0.4)	360 (233 to 507)	53.7 (34.4 to 76.4)	5 (3 to 7)	0.8 (0.5 to 1.1)	86.1 (-1.3 to 254.5)	65.2 (0.1 to 177.7)	213.8 (27.2 to 835.1)	239.1 (53.5 to 668)
		Male	187 (80 to 386)	25.2 (13.1 to 47.9)	2 (1 to 4)	0.2 (0.1 to 0.5)	183 (130 to 255)	29.7 (20.1 to 42.7)	2 (1 to 3)	0.4 (0.2 to 0.6)	-2 (-47.8 to 102.7)	17.6 (-29.2 to 96)	23.8 (-41.5 to 223.5)	71.6 (-12.6 to 281)
	HIV/AIDS	Both	16 (9 to 21)	1.6 (0.9 to 2.2)	0 (0 to 0)	0 (0 to 0)	275 (144 to 412)	20.2 (10.3 to 30.5)	5 (2 to 7)	0.3 (0.2 to 0.5)	1615 (737.8 to 3239.6)	1156.3 (496.4 to 2368.4)	2231.6 (923.7 to 5852.9)	1399.8 (522.9 to 4140.7)
		Female	8 (5 to 11)	1.5 (0.9 to 2.1)	0 (0 to 0)	0 (0 to 0)	201 (93 to 316)	29.3 (13.5 to 45.3)	4 (2 to 6)	0.5 (0.2 to 0.9)	2455 (1071.1 to 5056.9)	1790.7 (728.5 to 3574.8)	3642.9 (1407.6 to 9593.4)	2310.3 (829.9 to 6708.7)
		Male	8 (5 to 11)	1.7 (0.9 to 2.3)	0 (0 to 0)	0 (0 to 0)	74 (48 to 106)	11.5 (7.1 to 17.1)	1 (1 to 1)	0.2 (0.1 to 0.2)	802.5 (416.4 to 1614.3)	581.9 (265.9 to 1265.6)	905.5 (456.7 to 2432.8)	563.6 (244.9 to 1822.4)
	Sexually transmitted infections excluding HIV	Both	364 (164 to 738)	27.3 (14.8 to 49.9)	3 (1 to 7)	0.2 (0.1 to 0.4)	268 (173 to 406)	21.3 (13.4 to 33.6)	3 (1 to 4)	0.2 (0.1 to 0.4)	-26.4 (-55.8 to 26.5)	-22.2 (-43.9 to 2.8)	-20.5 (-63.6 to 98.6)	20.2 (-42.6 to 144.6)
		Female	185 (89 to 361)	31 (17.1 to 53.1)	1 (0 to 3)	0.2 (0.1 to 0.4)	158 (100 to 244)	24.4 (15.4 to 38.2)	1 (0 to 2)	0.3 (0.1 to 0.4)	-14.5 (-47.9 to 40.9)	-21.1 (-41.1 to 1.1)	-11.1 (-60.5 to 133.5)	23.5 (-41.5 to 144.1)
		Male	179 (71 to 379)	23.6 (11.3 to 45.8)	2 (1 to 4)	0.2 (0.1 to 0.5)	110 (66 to 169)	18.2 (10.2 to 29.4)	1 (0 to 2)	0.2 (0.1 to 0.4)	-38.7 (-64 to 15)	-22.7 (-49.5 to 16.5)	-28.6 (-72.7 to 97)	16.9 (-55.1 to 180.4)
	Respiratory infections and tuberculosis	Both	26032 (17259 to 39974)	1675.7 (1168.6 to 2455.7)	308 (208 to 462)	27.2 (20.9 to 37.7)	3755 (2980 to 5430)	320.6 (257.2 to 474.7)	93 (76 to 171)	9.3 (7.6 to 17.4)	-85.6 (-91.4 to -76.7)	-80.9 (-88 to -70.9)	-69.9 (-81.2 to -44.6)	-65.7 (-75.2 to -50.2)
		Female	12503 (7723 to 20330)	1628 (1061.2 to 2539.6)	146 (90 to 232)	25.3 (17.7 to 37)	1612 (1226 to 2400)	278 (213.2 to 419.2)	37 (28 to 77)	7.2 (5.4 to 15)	-87.1 (-92.7 to -75.9)	-82.9 (-89.8 to -70.5)	-74.6 (-85.6 to -42.4)	-71.5 (-81.6 to -52.5)
		Male	13529 (8429 to 22244)	1720.4 (1149.1 to 2684.1)	162 (105 to 260)	29.1 (21.4 to 42.6)	2143 (1723 to 3108)	365.1 (295.5 to 551.6)	56 (46 to 104)	11.7 (9.5 to 21.9)	-84.2 (-90.7 to -74)	-78.8 (-87 to -67.6)	-65.7 (-79.2 to -38.3)	-59.8 (-71.9 to -42.4)

Province	Cause	Sex	1990				2019				1990-2019 percent change (%)			
			DALYs		Death		DALYs		Death		DALYs		Death	
			Number	Rate	Number	Rate	Number	Rate	Number	Rate	Number	Rate	Number	Rate
	Tuberculosis	Both	1079 (788 to 1434)	105.4 (78.2 to 128.6)	19 (14 to 23)	2.9 (2.1 to 3.5)	295 (255 to 343)	23.3 (20.3 to 26.9)	9 (8 to 11)	0.8 (0.7 to 1)	-72.7 (-79.4 to -60)	-77.9 (-82.5 to -67.8)	-52.8 (-63.2 to -27.3)	-72.1 (-78.2 to -55.9)
		Female	539 (280 to 768)	102.9 (53.2 to 137.2)	9 (4 to 12)	2.7 (1.2 to 3.7)	124 (102 to 156)	20.1 (16.7 to 25.3)	4 (3 to 5)	0.7 (0.6 to 0.9)	-77 (-84.9 to -47.7)	-80.5 (-86.2 to -53.8)	-56.5 (-71.2 to 19.2)	-74.5 (-83.1 to -24.9)
		Male	539 (405 to 727)	107.6 (82.5 to 135.7)	10 (8 to 13)	3.1 (2.4 to 3.9)	171 (142 to 203)	26.6 (22.4 to 31.5)	5 (4 to 6)	0.9 (0.8 to 1.1)	-68.4 (-76.8 to -56.7)	-75.3 (-81.1 to -66.8)	-49.6 (-62.9 to -31.8)	-69.6 (-77.6 to -58.1)
	Lower respiratory infections	Both	23407 (14728 to 36973)	1451.4 (944.7 to 2225.9)	289 (189 to 441)	24.2 (18 to 34.9)	1989 (1639 to 3492)	182.3 (149.7 to 320.8)	84 (68 to 162)	8.5 (6.8 to 16.5)	-91.5 (-95.1 to -83.7)	-87.4 (-92.5 to -77.8)	-71.1 (-82.5 to -43.8)	-65 (-75.5 to -47.9)
		Female	11241 (6410 to 18754)	1411.2 (847.3 to 2291.9)	137 (81 to 221)	22.6 (15 to 34.5)	806 (620 to 1576)	148.2 (112.8 to 285.7)	33 (24 to 73)	6.5 (4.8 to 14.3)	-92.8 (-96.3 to -82.8)	-89.5 (-94.4 to -77.9)	-75.8 (-86.8 to -41.6)	-71.2 (-82.2 to -50.4)
		Male	12166 (7183 to 20653)	1489.3 (927.7 to 2465.7)	152 (94 to 250)	26 (18.6 to 39.1)	1183 (974 to 2049)	218.5 (179.9 to 383.3)	50 (41 to 98)	10.7 (8.6 to 21)	-90.3 (-94.7 to -81.7)	-85.3 (-91.7 to -74.5)	-66.9 (-80.6 to -38)	-58.8 (-72 to -39.8)
	Upper respiratory infections	Both	1097 (660 to 1732)	85.2 (51.7 to 132.6)	0 (0 to 1)	0 (0 to 0.1)	1102 (670 to 1721)	85.8 (52.5 to 134.9)	0 (0 to 1)	0 (0 to 0.1)	0.4 (-9.4 to 11.8)	0.7 (-5.6 to 6.5)	90.1 (-95.4 to 4395)	137.5 (-93.8 to 3598.9)
		Female	525 (311 to 818)	83.2 (49.7 to 128.3)	0 (0 to 0)	0 (0 to 0)	519 (318 to 817)	83.3 (50.3 to 133.1)	0 (0 to 0)	0 (0 to 0)	-1.1 (-10.8 to 11.4)	0.1 (-6.8 to 8)	27.5 (-97 to 4565.9)	67.2 (-95.6 to 3332.4)
		Male	572 (342 to 903)	87.1 (52.5 to 136.8)	0 (0 to 1)	0 (0 to 0.1)	582 (353 to 917)	88.1 (53.7 to 138.2)	0 (0 to 1)	0.1 (0 to 0.1)	1.8 (-9.9 to 14.2)	1.2 (-7.6 to 10)	126.6 (-94.5 to 5212.3)	186.5 (-92.6 to 4466.1)
	Otitis media	Both	450 (255 to 747)	33.7 (19.3 to 55.7)	0 (0 to 0)	0 (0 to 0)	369 (211 to 617)	29.2 (16.7 to 48.9)	0 (0 to 0)	0 (0 to 0)	-17.9 (-26.5 to -8)	-13.4 (-19.2 to -7.2)	-78.8 (-92.4 to -32.5)	-77.2 (-89.6 to -41.1)
		Female	198 (111 to 333)	30.8 (17.3 to 51.1)	0 (0 to 0)	0 (0 to 0)	163 (92 to 270)	26.4 (15.1 to 43.5)	0 (0 to 0)	0 (0 to 0)	-17.5 (-27.7 to -5.4)	-14.3 (-22 to -5.6)	-26.8 (-80.4 to 158.6)	-61.9 (-85.4 to 32.1)
		Male	252 (142 to 417)	36.5 (20.8 to 60.2)	0 (0 to 0)	0 (0 to 0)	206 (117 to 346)	31.8 (17.9 to 53.5)	0 (0 to 0)	0 (0 to 0)	-18.2 (-28.2 to -5.9)	-12.9 (-20.8 to -5)	-89.3 (-96.8 to -56.5)	-85.3 (-94.9 to -51.9)
	Enteric infections	Both	11318 (7153 to 18154)	734 (485.3 to 1126.8)	123 (73 to 200)	10.7 (6.8 to 16)	3129 (2311 to 4062)	267.8 (198.9 to 346.6)	19 (13 to 29)	1.8 (1.2 to 2.9)	-72.4 (-83.6 to -54.5)	-63.5 (-77.4 to -42.9)	-84.9 (-92.1 to -72.4)	-83.2 (-89.5 to -73.5)
		Female	5204 (2947 to 9428)	699.3 (428.2 to 1207)	57 (29 to 105)	10.7 (5.4 to 18.7)	1500 (1090 to 1984)	261.2 (191 to 344.5)	9 (5 to 16)	1.7 (0.9 to 3.1)	-71.2 (-84.3 to -48.3)	-62.6 (-78.6 to -37)	-84.6 (-92.9 to -68.5)	-84.4 (-91 to -71.7)
		Male	6114 (3577 to 11218)	766.2 (478.5 to 1330)	66 (36 to 121)	10.6 (6.4 to 18.5)	1629 (1204 to 2121)	274.5 (204.5 to 357.9)	10 (7 to 18)	1.9 (1.3 to 3.7)	-73.4 (-86.8 to -51.4)	-64.2 (-80.7 to -39.8)	-85.2 (-93.4 to -68.1)	-81.7 (-90.4 to -69.8)
	Diarrheal diseases	Both	10137 (6120 to 16936)	661.3 (420.9 to 1053.7)	107 (61 to 182)	9.7 (5.8 to 14.7)	2856 (2060 to 3798)	244.2 (176 to 323.2)	14 (9 to 25)	1.4 (0.9 to 2.6)	-71.8 (-83.9 to -51.7)	-63.1 (-77.8 to -40.3)	-86.6 (-93.4 to -72.9)	-85 (-91.2 to -75)
		Female	4612 (2468 to 8643)	624.9 (360.2 to 1124.3)	49 (23 to 95)	9.7 (4.4 to 17.4)	1374 (983 to 1849)	239 (169.8 to 320)	7 (3 to 14)	1.3 (0.6 to 2.8)	-70.2 (-84.5 to -42.2)	-61.8 (-79.3 to -32.7)	-86.1 (-94.8 to -67.6)	-86.1 (-92.9 to -72.7)
		Male	5525 (3094 to 10620)	695.1 (413.9 to 1260.8)	58 (30 to 114)	9.6 (5.5 to 17.3)	1482 (1073 to 1962)	249.6 (180.9 to 330.1)	8 (5 to 16)	1.6 (1 to 3.3)	-73.2 (-87.4 to -48.3)	-64.1 (-81.7 to -36.7)	-87 (-94.7 to -69.6)	-83.7 (-92 to -71.6)
	Typhoid and paratyphoid	Both	855 (330 to 1720)	52.7 (21 to 104.7)	11 (4 to 23)	0.8 (0.3 to 1.5)	194 (80 to 380)	16.8 (7 to 33.1)	3 (1 to 5)	0.2 (0.1 to 0.5)	-77.3 (-83.9 to -67.5)	-68.1 (-77 to -55.1)	-75.3 (-82.3 to -63.7)	-68.4 (-76.4 to -55.1)
		Female	397 (153 to 781)	50.3 (19.5 to 97.9)	5 (2 to 10)	0.7 (0.3 to 1.4)	89 (37 to 178)	16 (6.6 to 31.7)	1 (1 to 3)	0.2 (0.1 to 0.5)	-77.5 (-84 to -66.7)	-68.3 (-77.1 to -50.8)	-75.5 (-82.2 to -63)	-68.8 (-76.8 to -52.4)
		Male	457 (178 to 923)	54.9 (21.7 to 111.4)	6 (2 to 13)	0.8 (0.3 to 1.6)	104 (43 to 206)	17.6 (7.1 to 34.6)	2 (1 to 3)	0.3 (0.1 to 0.5)	-77.2 (-84.5 to -65.8)	-68 (-77.9 to -53.9)	-75.1 (-82.7 to -62.3)	-68 (-77 to -54)
		Both	258 (135 to 447)	16.1 (8.5 to 26.8)	3 (2 to 6)	0.3 (0.1 to 0.4)	74 (38 to 131)	6.4 (3.2 to 11.2)	1 (1 to 2)	0.1 (0.1 to 0.2)	-71.3 (-82.7 to -57.3)	-60.4 (-75.6 to -42.8)	-62.8 (-76.1 to -45.6)	-56.8 (-69.7 to -41.6)

Province	Cause	Sex	1990				2019				1990-2019 percent change (%)			
			DALYs		Death		DALYs		Death		DALYs		Death	
			Number	Rate	Number	Rate	Number	Rate	Number	Rate	Number	Rate	Number	Rate
	Invasive Non-typhoidal Salmonella (iNTS)	Female	154 (80 to 269)	19.4 (10.3 to 32.9)	2 (1 to 4)	0.3 (0.2 to 0.5)	33 (17 to 57)	5.8 (2.9 to 10.2)	1 (0 to 1)	0.1 (0.1 to 0.2)	-78.4 (-87.5 to -66.7)	-69.9 (-81.5 to -54.9)	-71.1 (-82.2 to -56)	-66.3 (-77.2 to -52.4)
		Male	103 (53 to 182)	12.9 (6.7 to 22)	1 (1 to 2)	0.2 (0.1 to 0.4)	41 (21 to 72)	6.9 (3.5 to 12.2)	1 (0 to 1)	0.1 (0.1 to 0.2)	-60.6 (-76.9 to -39.1)	-46.7 (-67.3 to -20.4)	-50.8 (-69 to -26.6)	-43.7 (-61.6 to -20.2)
	Other intestinal infectious diseases	Both	70 (12 to 161)	4 (0.7 to 9)	1 (0 to 2)	0 (0 to 0.1)	5 (3 to 8)	0.5 (0.3 to 0.7)	0 (0 to 0)	0 (0 to 0)	-92.4 (-97.1 to -53.6)	-88.5 (-95.7 to -37.6)	-92.7 (-97.6 to -53.6)	-89.1 (-96.3 to -51.4)
		Female	41 (2 to 118)	4.7 (0.4 to 13.7)	0 (0 to 1)	0.1 (0 to 0.2)	3 (1 to 4)	0.5 (0.2 to 0.8)	0 (0 to 0)	0 (0 to 0)	-93.6 (-98.1 to 12)	-90.1 (-97.2 to 28.8)	-93.8 (-98.4 to 41.4)	-90.7 (-97.7 to -8.3)
		Male	29 (3 to 82)	3.2 (0.4 to 9)	0 (0 to 1)	0 (0 to 0.1)	3 (2 to 4)	0.5 (0.3 to 0.7)	0 (0 to 0)	0 (0 to 0)	-90.7 (-97.2 to 4.4)	-86.1 (-95.7 to 15.4)	-91.2 (-98 to 33.8)	-87 (-96.7 to -7.9)
	Neglected tropical diseases and malaria	Both	4644 (517 to 31208)	358.3 (37.6 to 2641.9)	66 (3 to 506)	6.2 (0.3 to 50.4)	265 (144 to 883)	21.7 (11.8 to 72.6)	2 (1 to 11)	0.2 (0.1 to 0.9)	-94.3 (-99.3 to -61.4)	-93.9 (-99.3 to -57.2)	-96.8 (-99.7 to -63)	-97.2 (-99.8 to -65.2)
		Female	2152 (238 to 14891)	334.9 (38.5 to 2411.3)	29 (1 to 223)	5.3 (0.2 to 42.9)	123 (72 to 303)	20.6 (11.8 to 52.2)	1 (0 to 4)	0.1 (0.1 to 0.6)	-94.3 (-99.3 to -59.4)	-93.9 (-99.2 to -57.8)	-97.1 (-99.7 to -54.8)	-97.3 (-99.8 to -62.1)
		Male	2492 (219 to 16301)	379.2 (30.7 to 2627.1)	37 (1 to 263)	7 (0.2 to 55.7)	141 (66 to 544)	22.8 (10.3 to 89.6)	1 (0 to 7)	0.2 (0.1 to 1.2)	-94.3 (-99.4 to -55.8)	-94 (-99.4 to -47.9)	-96.6 (-99.7 to -57.7)	-97.1 (-99.8 to -60.2)
	Malaria	Both	2827 (18 to 28979)	237.1 (1.4 to 2427.3)	46 (0 to 470)	4.7 (0 to 48.2)	15 (10 to 20)	1.1 (0.8 to 1.5)	0 (0 to 0)	0 (0 to 0)	-99.5 (-99.9 to -20.1)	-99.5 (-100 to -24.4)	-100 (-100 to -100)	-100 (-100 to -100)
		Female	1431 (10 to 14574)	233.4 (1.6 to 2365.8)	22 (0 to 221)	4.1 (0 to 42.5)	7 (5 to 10)	1.1 (0.8 to 1.5)	0 (0 to 0)	0 (0 to 0)	-99.5 (-100 to -22.6)	-99.5 (-100 to -29.2)	-100 (-100 to -100)	-100 (-100 to -100)
		Male	1396 (8 to 14850)	238.9 (1.2 to 2523.8)	24 (0 to 254)	5.2 (0 to 53.7)	7 (5 to 10)	1.1 (0.8 to 1.5)	0 (0 to 0)	0 (0 to 0)	-99.5 (-100 to -12.5)	-99.5 (-100 to -14.5)	-100 (-100 to -100)	-100 (-100 to -100)
	Leishmaniasis	Both	1219 (3 to 12430)	78.9 (0.2 to 766.5)	16 (0 to 163)	1.2 (0 to 11.2)	69 (0 to 658)	5.7 (0 to 55)	1 (0 to 10)	0.1 (0 to 0.8)	-94.3 (-95.6 to -85.3)	-92.8 (-94.8 to -82.1)	-93.2 (-94.3 to -88.2)	-92.7 (-94.4 to -90.5)
		Female	417 (1 to 4178)	54.8 (0.1 to 518.6)	6 (0 to 55)	0.8 (0 to 7.4)	22 (0 to 205)	3.7 (0 to 35.3)	0 (0 to 3)	0.1 (0 to 0.5)	-94.8 (-95.9 to -79.9)	-93.3 (-95.2 to -76.5)	-93.6 (-94.9 to -88.2)	-93.1 (-94.8 to -90.7)
		Male	802 (2 to 8329)	102.1 (0.3 to 1014.4)	11 (0 to 111)	1.6 (0 to 14.8)	47 (0 to 457)	7.5 (0 to 74.5)	1 (0 to 7)	0.1 (0 to 1.1)	-94.1 (-95.5 to -87.8)	-92.6 (-94.8 to -85.1)	-93 (-94.1 to -87.8)	-92.5 (-94.3 to -90)
	Schistosomiasis	Both	0 (0 to 0)	0 (0 to 0)	0 (0 to 0)	0 (0 to 0)	0 (0 to 0)	0 (0 to 0)	0 (0 to 0)	0 (0 to 0)	0 (0 to 0)	0 (0 to 0)	0 (0 to 0)	0 (0 to 0)
		Female	0 (0 to 0)	0 (0 to 0)	0 (0 to 0)	0 (0 to 0)	0 (0 to 0)	0 (0 to 0)	0 (0 to 0)	0 (0 to 0)	0 (0 to 0)	0 (0 to 0)	0 (0 to 0)	0 (0 to 0)
		Male	0 (0 to 0)	0 (0 to 0)	0 (0 to 0)	0 (0 to 0)	0 (0 to 0)	0 (0 to 0)	0 (0 to 0)	0 (0 to 0)	0 (0 to 0)	0 (0 to 0)	0 (0 to 0)	0 (0 to 0)
	Cystic echinococcosis	Both	102 (46 to 182)	8.2 (4.8 to 12.7)	1 (1 to 2)	0.1 (0.1 to 0.2)	38 (26 to 53)	2.8 (1.9 to 3.9)	0 (0 to 0)	0 (0 to 0)	-62.8 (-80.5 to -13.3)	-65.4 (-79.4 to -38.6)	-77 (-89.9 to -43.2)	-80 (-90.5 to -61)
		Female	45 (15 to 92)	7.4 (3.6 to 12.5)	1 (0 to 1)	0.1 (0 to 0.2)	20 (13 to 28)	3 (1.9 to 4.2)	0 (0 to 0)	0 (0 to 0)	-55.9 (-81.2 to 30.3)	-60.2 (-79.8 to -18.3)	-76.3 (-92.9 to 2.4)	-79.5 (-93.4 to -36.1)
		Male	57 (18 to 117)	8.8 (4.2 to 15.4)	1 (0 to 2)	0.2 (0.1 to 0.3)	18 (12 to 26)	2.7 (1.7 to 3.8)	0 (0 to 0)	0 (0 to 0.1)	-68.2 (-86 to 1.8)	-69.4 (-84.1 to -32.6)	-77.4 (-93.3 to -18.6)	-80 (-93.3 to -47.7)
	Trachoma	Both	11 (6 to 18)	2.6 (1.5 to 4.2)	--	--	4 (2 to 7)	0.4 (0.2 to 0.7)	--	--	-63.1 (-72.5 to -51.9)	-85 (-88.8 to -81)	--	--
		Female	8 (5 to 14)	4.3 (2.4 to 6.9)	--	--	3 (2 to 5)	0.5 (0.3 to 1)	--	--	-65.3 (-75.6 to -52)	-87.6 (-91.2 to -83.4)	--	--
		Male	3 (1 to 5)	1.1 (0.6 to 2)	--	--	1 (1 to 2)	0.2 (0.1 to 0.4)	--	--	-56 (-71 to -31.5)	-79.4 (-86.1 to -70.4)	--	--

Province	Cause	Sex	1990				2019				1990-2019 percent change (%)			
			DALYs		Death		DALYs		Death		DALYs		Death	
			Number	Rate	Number	Rate	Number	Rate	Number	Rate	Number	Rate	Number	Rate
	Rabies	Both	6 (1 to 12)	0.7 (0.1 to 1.4)	0 (0 to 0)	0 (0 to 0)	2 (0 to 4)	0.2 (0 to 0.3)	0 (0 to 0)	0 (0 to 0)	-62.9 (-83.5 to -19.5)	-76.3 (-88.9 to -50.1)	-59 (-81.8 to -14.6)	-77.2 (-89 to -52.7)
		Female	5 (0 to 10)	1.4 (0 to 2.7)	0 (0 to 0)	0 (0 to 0.1)	2 (0 to 3)	0.3 (0 to 0.5)	0 (0 to 0)	0 (0 to 0)	-61.4 (-88 to -16.5)	-77.7 (-89.1 to -51.6)	-59.4 (-80.6 to -13.1)	-79.6 (-90.2 to -56.9)
		Male	1 (0 to 7)	0.1 (0 to 1.1)	0 (0 to 0)	0 (0 to 0)	0 (0 to 1)	0 (0 to 0.2)	0 (0 to 0)	0 (0 to 0)	-71.1 (-86.3 to -9.5)	-71.5 (-86.4 to -23.1)	-55.3 (-79.2 to 20.2)	-63.4 (-83 to -13)
	Intestinal nematode infections	Both	130 (55 to 241)	8.3 (4 to 14.7)	1 (0 to 2)	0.1 (0 to 0.1)	14 (7 to 24)	1.1 (0.6 to 1.9)	0 (0 to 0)	0 (0 to 0)	-89.4 (-95.4 to -73.2)	-86.5 (-93.7 to -70.8)	-96.3 (-98.8 to -79.7)	-94 (-98 to -74.7)
		Female	67 (24 to 144)	9.3 (3.9 to 18.1)	0 (0 to 1)	0.1 (0 to 0.2)	8 (4 to 15)	1.4 (0.7 to 2.4)	0 (0 to 0)	0 (0 to 0)	-87.5 (-95.2 to -6.4)	-85.3 (-93.6 to -65.6)	-96.7 (-99.3 to -6.4)	-94.7 (-98.9 to -34.1)
		Male	63 (15 to 152)	7.2 (2.2 to 16.6)	1 (0 to 2)	0.1 (0 to 0.2)	5 (3 to 10)	0.9 (0.5 to 1.6)	0 (0 to 0)	0 (0 to 0)	-91.3 (-97.2 to -64.7)	-87.9 (-95.8 to -59.8)	-96.1 (-99 to -38.6)	-93.4 (-98.4 to -42)
	Food-borne trematodiases	Both	4 (1 to 8)	0.5 (0.1 to 1)	--	--	14 (3 to 28)	1 (0.2 to 2)	--	--	255.3 (165.8 to 369.7)	108.1 (60.5 to 163.8)	--	--
		Female	2 (0 to 3)	0.4 (0.1 to 0.8)	--	--	5 (1 to 11)	0.8 (0.2 to 1.7)	--	--	247.7 (159.7 to 375.7)	100.6 (50.7 to 168.7)	--	--
		Male	2 (1 to 5)	0.6 (0.1 to 1.1)	--	--	8 (2 to 17)	1.2 (0.3 to 2.4)	--	--	260.3 (129.9 to 426.9)	114 (47.1 to 199.3)	--	--
	Leprosy	Both	0 (0 to 1)	0.1 (0 to 0.1)	--	--	0 (0 to 1)	0 (0 to 0.1)	--	--	23 (11.5 to 34.5)	-40.9 (-46 to -35.6)	--	--
		Female	0 (0 to 0)	0 (0 to 0.1)	--	--	0 (0 to 0)	0 (0 to 0)	--	--	19 (2.6 to 35.9)	-44.8 (-51.9 to -37.7)	--	--
		Male	0 (0 to 0)	0.1 (0 to 0.1)	--	--	0 (0 to 0)	0 (0 to 0.1)	--	--	25 (10.9 to 40)	-37.3 (-43.9 to -30.5)	--	--
	Other neglected tropical diseases	Both	345 (212 to 546)	22 (14.3 to 33.7)	1 (1 to 3)	0.1 (0.1 to 0.2)	108 (65 to 166)	9.4 (5.7 to 14.6)	1 (0 to 1)	0.1 (0 to 0.1)	-68.7 (-80.6 to -50.7)	-57.3 (-72.7 to -35.1)	-43.6 (-75.7 to 10.8)	-40.7 (-68.4 to 6.1)
		Female	177 (103 to 281)	23.9 (15.1 to 35.9)	1 (0 to 1)	0.1 (0 to 0.2)	55 (32 to 91)	9.8 (5.6 to 16.2)	0 (0 to 0)	0.1 (0 to 0.1)	-68.8 (-81.7 to -46.6)	-59.1 (-75 to -34)	-42.8 (-74.7 to 27.9)	-43.8 (-68.4 to 12)
		Male	168 (81 to 312)	20.3 (10.6 to 36.2)	0 (0 to 2)	0.1 (0 to 0.3)	53 (27 to 97)	9.1 (4.5 to 17)	0 (0 to 1)	0.1 (0 to 0.1)	-68.6 (-84.7 to -39.1)	-55.4 (-77.6 to -16)	-44.4 (-78.8 to 50.7)	-36.4 (-70.2 to 46.8)
	Other infectious diseases	Both	23287 (11670 to 45137)	1369.2 (711.1 to 2582.7)	278 (140 to 530)	18.6 (10.5 to 33.8)	1485 (1232 to 1911)	125.7 (103.4 to 166)	30 (25 to 35)	2.7 (2.3 to 3.2)	-93.6 (-96.8 to -86.8)	-90.8 (-95.2 to -82)	-89.3 (-94.6 to -78.5)	-85.6 (-92.1 to -74.1)
		Female	12569 (6178 to 23987)	1500.6 (772.8 to 2783.9)	148 (73 to 281)	19.9 (10.9 to 34.7)	661 (530 to 884)	115.3 (91.1 to 159.4)	11 (9 to 14)	2.1 (1.7 to 2.6)	-94.7 (-97.2 to -88.9)	-92.3 (-96 to -84.4)	-92.4 (-96 to -84.5)	-89.7 (-94.2 to -81)
		Male	10717 (5255 to 21469)	1242.2 (641.6 to 2448.4)	130 (65 to 258)	17.2 (9.7 to 32.6)	824 (676 to 1020)	136.9 (112.5 to 173.7)	18 (15 to 22)	3.3 (2.7 to 4.1)	-92.3 (-96.3 to -83.9)	-89 (-94.4 to -78.2)	-85.8 (-93 to -70.6)	-80.7 (-90 to -63.7)
	Meningitis	Both	3514 (2252 to 5437)	215.1 (142.8 to 323.8)	42 (27 to 64)	2.9 (2 to 4.3)	348 (294 to 415)	29 (24.4 to 34.8)	6 (5 to 7)	0.6 (0.5 to 0.7)	-90.1 (-94 to -83.8)	-86.5 (-91.6 to -78.5)	-84.7 (-90.3 to -75.3)	-80.4 (-87.1 to -70.3)
		Female	1996 (1204 to 3287)	249.2 (157 to 398.3)	23 (14 to 38)	3.3 (2.2 to 5.1)	190 (154 to 238)	32.4 (25.6 to 41.5)	3 (3 to 4)	0.6 (0.5 to 0.7)	-90.5 (-94.5 to -83.6)	-87 (-92.4 to -78.5)	-86.8 (-92.4 to -78)	-83.2 (-89.8 to -73.9)
		Male	1518 (884 to 2555)	182.3 (108.7 to 301.5)	18 (11 to 30)	2.5 (1.6 to 3.9)	157 (128 to 194)	25.9 (21.1 to 31.8)	3 (3 to 4)	0.6 (0.5 to 0.7)	-89.6 (-94.2 to -80.5)	-85.8 (-91.9 to -73.9)	-82.1 (-89.5 to -65.8)	-76.6 (-85.7 to -58.7)

Province	Cause	Sex	1990				2019				1990-2019 percent change (%)			
			DALYs		Death		DALYs		Death		DALYs		Death	
			Number	Rate	Number	Rate	Number	Rate	Number	Rate	Number	Rate	Number	Rate
	Encephalitis	Both	338 (215 to 593)	22.7 (15.5 to 37.2)	4 (3 to 7)	0.3 (0.2 to 0.5)	179 (145 to 219)	15.1 (12 to 18.8)	3 (2 to 4)	0.3 (0.2 to 0.3)	-47.1 (-71.9 to -13.2)	-33.5 (-62.3 to 5.7)	-22.6 (-55.8 to 29.1)	-16.1 (-46.4 to 29.8)
		Female	195 (111 to 392)	26.2 (16.3 to 49.1)	2 (1 to 4)	0.4 (0.2 to 0.6)	89 (69 to 117)	15.5 (11.5 to 20.9)	1 (1 to 2)	0.3 (0.2 to 0.3)	-54 (-79.8 to -13.9)	-41 (-72.8 to 6.3)	-36.4 (-71.3 to 23.4)	-29.2 (-61.6 to 24.1)
		Male	143 (78 to 270)	19.3 (11.6 to 33.7)	2 (1 to 3)	0.3 (0.2 to 0.5)	89 (70 to 116)	14.8 (11.4 to 19.3)	2 (1 to 2)	0.3 (0.2 to 0.4)	-37.8 (-68.7 to 22.1)	-23.4 (-57.8 to 36.9)	-4.9 (-49.2 to 89.6)	-0.1 (-42.6 to 80.7)
	Diphtheria	Both	141 (52 to 295)	7.9 (3 to 16.4)	2 (1 to 3)	0.1 (0 to 0.2)	1 (1 to 1)	0.1 (0 to 0.1)	0 (0 to 0)	0 (0 to 0)	-99.4 (-99.8 to -97.9)	-99 (-99.6 to -97)	-99.2 (-99.7 to -97.5)	-98.8 (-99.5 to -96.6)
		Female	68 (16 to 170)	7.7 (1.9 to 19.4)	1 (0 to 2)	0.1 (0 to 0.2)	0 (0 to 1)	0.1 (0 to 0.1)	0 (0 to 0)	0 (0 to 0)	-99.5 (-99.9 to -97.3)	-99.2 (-99.8 to -95.9)	-99.4 (-99.8 to -97)	-99 (-99.7 to -95.8)
		Male	73 (18 to 182)	8.1 (2.2 to 19.5)	1 (0 to 2)	0.1 (0 to 0.2)	1 (0 to 1)	0.1 (0 to 0.2)	0 (0 to 0)	0 (0 to 0)	-99.3 (-99.8 to -96.7)	-98.9 (-99.7 to -95.2)	-99 (-99.7 to -95.7)	-98.6 (-99.6 to -94.9)
	Whooping cough	Both	5365 (269 to 21954)	296.6 (14.8 to 1218.6)	61 (2 to 253)	3.4 (0.1 to 14)	108 (8 to 465)	10.4 (0.8 to 44.9)	1 (0 to 5)	0.1 (0 to 0.5)	-98 (-99.9 to -55.6)	-96.5 (-99.8 to -22.4)	-98 (-99.9 to -45.9)	-96.6 (-99.9 to -5.6)
		Female	3100 (163 to 12581)	350.3 (18.3 to 1424.2)	35 (1 to 145)	4 (0.2 to 16.5)	60 (5 to 252)	12 (0.9 to 50.1)	1 (0 to 3)	0.1 (0 to 0.6)	-98.1 (-99.9 to -57)	-96.6 (-99.8 to -23.4)	-98.1 (-99.9 to -48)	-96.7 (-99.9 to -7.6)
		Male	2265 (112 to 9405)	245.3 (12.1 to 1022.6)	26 (1 to 109)	2.8 (0.1 to 11.8)	48 (4 to 208)	9 (0.7 to 39.1)	1 (0 to 2)	0.1 (0 to 0.5)	-97.9 (-99.9 to -53.9)	-96.3 (-99.8 to -19.6)	-97.9 (-99.9 to -38.6)	-96.4 (-99.9 to 6.5)
	Tetanus	Both	277 (70 to 698)	16.8 (4.6 to 42.3)	3 (1 to 8)	0.2 (0.1 to 0.6)	10 (8 to 18)	0.8 (0.6 to 1.5)	0 (0 to 0)	0 (0 to 0)	-96.4 (-98.6 to -84.2)	-95 (-98.1 to -79.7)	-94.2 (-97.7 to -79.1)	-93 (-97.3 to -79.4)
		Female	131 (23 to 369)	16.1 (3.4 to 46.1)	2 (0 to 4)	0.2 (0.1 to 0.6)	5 (4 to 6)	0.8 (0.6 to 1.1)	0 (0 to 0)	0 (0 to 0)	-96.4 (-98.8 to -78.4)	-94.9 (-98.2 to -74.2)	-94.3 (-98 to -72.6)	-93 (-97.4 to -75.5)
		Male	145 (34 to 411)	17.5 (4.5 to 49.2)	2 (0 to 5)	0.3 (0.1 to 0.7)	5 (4 to 12)	0.9 (0.6 to 2)	0 (0 to 0)	0 (0 to 0)	-96.4 (-98.8 to -83.4)	-95 (-98.3 to -77.4)	-94.2 (-97.9 to -76.5)	-93 (-97.5 to -74.3)
	Measles	Both	10915 (3384 to 25317)	590.9 (182.3 to 1371.5)	127 (39 to 294)	6.9 (2.1 to 16)	87 (26 to 201)	8.3 (2.5 to 19.1)	1 (0 to 2)	0.1 (0 to 0.2)	-99.2 (-99.7 to -98.3)	-98.6 (-99.5 to -97)	-99.2 (-99.7 to -98.3)	-98.6 (-99.5 to -97.1)
		Female	5685 (1729 to 12915)	629.6 (190.4 to 1434.5)	66 (20 to 150)	7.3 (2.2 to 16.6)	43 (13 to 104)	8.5 (2.6 to 20.3)	0 (0 to 1)	0.1 (0 to 0.2)	-99.2 (-99.7 to -98.4)	-98.7 (-99.5 to -97.1)	-99.3 (-99.7 to -98.4)	-98.7 (-99.5 to -97.1)
		Male	5230 (1571 to 12267)	553.8 (166.1 to 1299.6)	61 (18 to 142)	6.4 (1.9 to 15.1)	44 (13 to 102)	8.1 (2.4 to 18.9)	0 (0 to 1)	0.1 (0 to 0.2)	-99.2 (-99.7 to -98.3)	-98.5 (-99.5 to -96.9)	-99.2 (-99.7 to -98.3)	-98.6 (-99.5 to -97)
	Varicella and herpes zoster	Both	193 (108 to 332)	14.4 (9.4 to 21.9)	3 (1 to 4)	0.3 (0.2 to 0.5)	58 (41 to 83)	4.9 (3.5 to 6.9)	1 (1 to 1)	0.1 (0.1 to 0.1)	-69.7 (-84.4 to -39.9)	-65.8 (-80.5 to -42.1)	-70.2 (-84.2 to -42.4)	-76.7 (-86.1 to -60)
		Female	98 (45 to 188)	14.8 (8.7 to 25.9)	1 (1 to 2)	0.3 (0.2 to 0.5)	29 (20 to 42)	5 (3.3 to 7.1)	0 (0 to 1)	0.1 (0 to 0.1)	-70.2 (-86.9 to -31.3)	-66.5 (-83.3 to -36.6)	-69.6 (-86.9 to -28.4)	-76.9 (-88.9 to -53.3)
		Male	95 (44 to 186)	14 (8 to 23.7)	1 (1 to 2)	0.3 (0.2 to 0.5)	29 (19 to 42)	4.9 (3.2 to 7.2)	0 (0 to 1)	0.1 (0 to 0.1)	-69.2 (-86.6 to -27.1)	-65.1 (-82.6 to -33)	-70.8 (-87.2 to -30.1)	-76.6 (-88.7 to -53)
	Acute hepatitis	Both	1545 (802 to 2578)	134.9 (70 to 220.2)	26 (13 to 43)	3.3 (1.6 to 5.7)	374 (302 to 465)	29.6 (24 to 36.6)	10 (8 to 13)	0.9 (0.7 to 1.1)	-75.8 (-85.9 to -47.7)	-78.1 (-87 to -53.3)	-59.6 (-77.2 to -9.8)	-72.6 (-85.4 to -38.3)
		Female	879 (335 to 1589)	145 (53.8 to 236.1)	13 (5 to 22)	3.2 (1.1 to 5)	102 (82 to 128)	16.5 (13.3 to 20.5)	2 (2 to 3)	0.4 (0.3 to 0.6)	-88.4 (-93.8 to -68.4)	-88.6 (-93.2 to -68.2)	-81.7 (-89.4 to -43.8)	-87 (-92.4 to -57.5)
		Male	666 (326 to 1386)	124 (62.1 to 248.4)	12 (6 to 26)	3.2 (1.5 to 6.9)	272 (208 to 355)	43 (32.8 to 55.9)	8 (6 to 11)	1.4 (1 to 1.9)	-59.1 (-81.8 to -7.2)	-65.3 (-84.2 to -24.9)	-36 (-72.8 to 50.4)	-56.8 (-81.7 to 0.6)
	Other unspecified infectious diseases	Both	999 (552 to 1692)	69.9 (43.6 to 110.9)	11 (6 to 19)	1.1 (0.8 to 1.6)	320 (248 to 406)	27.5 (21.1 to 35.2)	7 (5 to 8)	0.6 (0.4 to 0.8)	-68 (-82.4 to -37.4)	-60.7 (-76.8 to -32.8)	-39.5 (-71.1 to 19.3)	-45.3 (-66.4 to -9.4)
		Female	417 (214 to 863)	61.6 (35.6 to 112.8)	4 (2 to 9)	1 (0.6 to 1.6)	141 (104 to 186)	24.7 (18 to 33.1)	3 (2 to 4)	0.5 (0.3 to 0.6)	-66.1 (-85 to -32.2)	-59.9 (-79.9 to -29.7)	-39 (-74.5 to 29.2)	-50.9 (-71 to -10.1)

Province	Cause	Sex	1990				2019				1990-2019 percent change (%)			
			DALYs		Death		DALYs		Death		DALYs		Death	
			Number	Rate	Number	Rate	Number	Rate	Number	Rate	Number	Rate	Number	Rate
		Male	581 (291 to 1059)	77.9 (43.3 to 132.4)	7 (3 to 12)	1.3 (0.7 to 2.1)	178 (131 to 243)	30.4 (22 to 41.8)	4 (3 to 5)	0.8 (0.5 to 1)	-69.4 (-84.4 to -28.5)	-61 (-78.9 to -19.3)	-39.8 (-72.4 to 38.4)	-39.9 (-66.9 to 11.4)
	Maternal and neonatal disorders	Both	108386 (84021 to 137665)	6272.7 (4873.1 to 7953.2)	1215 (941 to 1546)	70.5 (54.5 to 89.5)	13538 (10908 to 16403)	1333.7 (1061.2 to 1624.4)	121 (93 to 151)	12.6 (9.6 to 15.7)	-87.5 (-91 to -82.8)	-78.7 (-84.8 to -70.8)	-90.1 (-93.3 to -86.2)	-82.2 (-87.9 to -75.3)
		Female	48395 (37310 to 62916)	5810.6 (4503.3 to 7532.5)	544 (420 to 707)	65.6 (51.1 to 85.1)	6046 (4911 to 7262)	1209.6 (970.7 to 1468.9)	53 (41 to 66)	11.2 (8.6 to 14)	-87.5 (-91.2 to -82.4)	-79.2 (-85.4 to -70.8)	-90.3 (-93.4 to -85.9)	-82.9 (-88.4 to -75.4)
		Male	59991 (44080 to 80025)	6700.5 (4917.9 to 8947.6)	672 (493 to 896)	74.9 (55 to 100)	7492 (5947 to 9188)	1451 (1144.9 to 1793.1)	68 (52 to 86)	13.8 (10.5 to 17.6)	-87.5 (-91.4 to -81.9)	-78.3 (-85.3 to -68.7)	-89.9 (-93.4 to -85.2)	-81.5 (-87.9 to -72.9)
	Maternal disorders	Female	1130 (650 to 1541)	234.5 (139.2 to 325.9)	16 (8 to 23)	3.5 (1.9 to 5.1)	239 (188 to 301)	32.3 (25.3 to 41.1)	3 (2 to 4)	0.4 (0.3 to 0.5)	-78.8 (-84.9 to -63.5)	-86.2 (-90.5 to -77.4)	-82.2 (-88.3 to -64)	-89.4 (-93.1 to -80.2)
	Neonatal disorders	Both	107256 (82937 to 136278)	6154.4 (4761.6 to 7818.3)	1200 (926 to 1528)	68.7 (53 to 87.6)	13299 (10673 to 16156)	1318.3 (1045.7 to 1607.5)	118 (90 to 148)	12.4 (9.4 to 15.5)	-87.6 (-91.1 to -82.9)	-78.6 (-84.8 to -70.4)	-90.2 (-93.4 to -86.2)	-82 (-87.8 to -74.8)
		Female	47266 (36403 to 61765)	5576.1 (4293.8 to 7287.2)	528 (406 to 691)	62.1 (47.8 to 81.3)	5806 (4690 to 7023)	1177.4 (939.4 to 1437.1)	50 (38 to 63)	10.8 (8.2 to 13.7)	-87.7 (-91.4 to -82.6)	-78.9 (-85.4 to -70)	-90.5 (-93.6 to -86)	-82.6 (-88.3 to -74.4)
		Male	59991 (44080 to 80025)	6700.5 (4917.9 to 8947.6)	672 (493 to 896)	74.9 (55 to 100)	7492 (5947 to 9188)	1451 (1144.9 to 1793.1)	68 (52 to 86)	13.8 (10.5 to 17.6)	-87.5 (-91.4 to -81.9)	-78.3 (-85.3 to -68.7)	-89.9 (-93.4 to -85.2)	-81.5 (-87.9 to -72.9)
	Nutritional deficiencies	Both	6581 (4507 to 9293)	458.4 (320.8 to 634)	28 (15 to 49)	2 (1.2 to 3.2)	1847 (1165 to 2770)	153.2 (96.4 to 231.7)	2 (2 to 3)	0.2 (0.2 to 0.3)	-71.9 (-80.6 to -60.3)	-66.6 (-76.1 to -54.3)	-91.8 (-95.4 to -84.2)	-88.4 (-92.8 to -79.6)
		Female	3316 (2118 to 5079)	496.6 (331.7 to 723)	13 (5 to 28)	1.9 (0.9 to 3.7)	991 (598 to 1498)	166.7 (99.7 to 256.6)	1 (1 to 1)	0.2 (0.2 to 0.2)	-70.1 (-82 to -52.9)	-66.4 (-78.6 to -50.3)	-92.5 (-96.5 to -81)	-89.6 (-94.7 to -77.1)
		Male	3265 (2057 to 4933)	422.6 (277.7 to 620.3)	15 (7 to 29)	2.2 (1.1 to 3.7)	856 (496 to 1371)	139.7 (80.8 to 225.5)	1 (1 to 2)	0.3 (0.2 to 0.3)	-73.8 (-84.3 to -58.6)	-66.9 (-79.6 to -49.4)	-91.2 (-95.5 to -80.2)	-87.1 (-92.6 to -74.8)
	Protein-energy malnutrition	Both	2697 (1562 to 4545)	162.8 (97.4 to 266.9)	27 (14 to 47)	1.9 (1.1 to 3.1)	389 (255 to 563)	32.4 (21.6 to 46.3)	2 (1 to 2)	0.2 (0.1 to 0.2)	-85.6 (-92.7 to -72.6)	-80.1 (-89.7 to -63.8)	-94.5 (-96.9 to -89.2)	-91.9 (-95 to -85.8)
		Female	1299 (624 to 2566)	159.5 (78.9 to 309.4)	13 (5 to 27)	1.8 (0.9 to 3.6)	183 (113 to 272)	31.2 (19.7 to 45.9)	1 (1 to 1)	0.1 (0.1 to 0.2)	-85.9 (-93.7 to -68.7)	-80.4 (-91 to -58.6)	-94.7 (-97.5 to -86.6)	-92.5 (-96.1 to -83.3)
		Male	1397 (737 to 2577)	165.8 (91.6 to 299.1)	14 (7 to 28)	2 (1.1 to 3.5)	206 (129 to 306)	33.6 (21.6 to 48.5)	1 (1 to 1)	0.2 (0.1 to 0.2)	-85.2 (-92.8 to -68.6)	-79.7 (-89.8 to -59.4)	-94.2 (-97.1 to -86.7)	-91.2 (-95 to -82.2)
	Iodine deficiency	Both	159 (98 to 237)	15.4 (10 to 22.3)	--	--	89 (51 to 145)	6.6 (3.8 to 10.6)	--	--	-43.8 (-59 to -26.5)	-57.3 (-68.8 to -46)	--	--
		Female	97 (59 to 145)	19.3 (12.3 to 28.4)	--	--	55 (31 to 90)	8.3 (4.7 to 13.4)	--	--	-43.4 (-61.7 to -23.1)	-56.8 (-70.2 to -43.3)	--	--
		Male	62 (37 to 93)	11.8 (7.4 to 17.1)	--	--	34 (20 to 55)	5 (2.9 to 8)	--	--	-44.6 (-61 to -26.5)	-57.9 (-68.7 to -46.3)	--	--
	Vitamin A deficiency	Both	257 (129 to 461)	13.5 (6.7 to 24.2)	--	--	9 (3 to 19)	0.8 (0.3 to 1.8)	--	--	-96.7 (-98.6 to -92.1)	-93.9 (-97.4 to -85.8)	--	--
		Female	94 (43 to 168)	10.1 (4.7 to 18)	--	--	4 (1 to 8)	0.7 (0.2 to 1.6)	--	--	-96.2 (-98.7 to -90.4)	-93 (-97.6 to -82.4)	--	--
		Male	163 (64 to 325)	16.7 (6.7 to 33.1)	--	--	5 (1 to 13)	0.9 (0.2 to 2.4)	--	--	-96.9 (-99.2 to -89.7)	-94.5 (-98.5 to -81.7)	--	--
	Dietary iron deficiency	Both	3301 (1933 to 5150)	253 (154.9 to 387.1)	--	--	1237 (693 to 2021)	103.8 (58.6 to 169.9)	--	--	-62.5 (-75.8 to -42.7)	-59 (-72.3 to -40)	--	--
		Female	1757 (980 to 2783)	296.2 (174.1 to 448.4)	--	--	693 (359 to 1154)	117.5 (61.3 to 196.9)	--	--	-60.6 (-76.3 to -34.8)	-60.3 (-75.5 to -38.7)	--	--

Province	Cause	Sex	1990				2019				1990-2019 percent change (%)			
			DALYs		Death		DALYs		Death		DALYs		Death	
			Number	Rate	Number	Rate	Number	Rate	Number	Rate	Number	Rate	Number	Rate
		Male	1544 (759 to 2794)	212.6 (118.2 to 363.4)	--	--	544 (265 to 1001)	90.1 (42.4 to 165.4)	--	--	-64.8 (-82.9 to -32.3)	-57.6 (-78.7 to -20.8)	--	--
	Other nutritional deficiencies	Both	168 (109 to 242)	13.7 (9.5 to 19.3)	1 (1 to 2)	0.1 (0.1 to 0.2)	123 (80 to 183)	9.5 (6.3 to 14)	1 (1 to 1)	0.1 (0.1 to 0.1)	-26.6 (-50 to 7.8)	-30.4 (-49.1 to -4.7)	-27.5 (-57.3 to 49.1)	-33.4 (-55.3 to 30.4)
		Female	69 (42 to 108)	11.5 (7.2 to 17.1)	0 (0 to 1)	0.1 (0 to 0.2)	57 (34 to 88)	8.9 (5.5 to 13.7)	0 (0 to 0)	0.1 (0 to 0.1)	-17.1 (-49.6 to 28.3)	-22.3 (-49.4 to 17.1)	-16.3 (-60.4 to 104.5)	-26.6 (-64.5 to 79.1)
		Male	100 (62 to 155)	15.7 (10.4 to 23)	1 (0 to 1)	0.2 (0.1 to 0.2)	67 (42 to 103)	10.1 (6.5 to 15.3)	0 (0 to 1)	0.1 (0.1 to 0.1)	-33.1 (-60.7 to 8.8)	-35.7 (-56.8 to -3.2)	-33.2 (-66.1 to 65.7)	-35.8 (-61.7 to 33.8)
	**Non-communicable diseases**	Both	285180 (229996 to 342276)	31376.2 (26662.6 to 36048.4)	4304 (3532 to 5072)	821.4 (715.9 to 926.4)	264239 (227980 to 307366)	21679.9 (18913.8 to 24894.2)	6291 (5810 to 6777)	615.4 (570.4 to 660.7)	-7.3 (-23.9 to 15.1)	-30.9 (-40 to -19.6)	46.2 (21.6 to 81.5)	-25.1 (-35.1 to -12.1)
		Female	133103 (103497 to 160969)	30077.3 (25078.8 to 34934.8)	1801 (1451 to 2179)	723.4 (613.4 to 837.8)	126205 (106251 to 150303)	20383.2 (17397.3 to 24053)	2607 (2377 to 2830)	498 (456.4 to 537.8)	-5.2 (-23.2 to 18.8)	-32.2 (-41.7 to -20.9)	44.8 (17.9 to 84.9)	-31.2 (-41.9 to -16.7)
		Male	152077 (116902 to 184182)	32387.9 (27188.9 to 37976.9)	2503 (1975 to 3057)	909.9 (760.1 to 1087.4)	138034 (119913 to 158260)	23158.3 (20307.5 to 26251.3)	3684 (3280 to 4071)	741.3 (664.6 to 813.5)	-9.2 (-27.3 to 20.6)	-28.5 (-39.9 to -13)	47.2 (17.1 to 92.3)	-18.5 (-33.6 to 0.7)
	Neoplasms	Both	24816 (19085 to 30283)	3518.6 (2739.3 to 4251.3)	681 (532 to 826)	135.2 (106.6 to 162.4)	37737 (31647 to 42880)	3150.5 (2668.7 to 3569.5)	1465 (1248 to 1659)	134.2 (115 to 151.5)	52.1 (22 to 103)	-10.5 (-26.9 to 19.2)	115.1 (75.4 to 186.7)	-0.8 (-18 to 28)
		Female	10826 (7810 to 13722)	3038.2 (2336.8 to 3779.1)	269 (208 to 336)	108.2 (83.8 to 132.6)	14869 (12494 to 17101)	2384.4 (2009.5 to 2734.9)	539 (457 to 616)	93.9 (80 to 107.1)	37.3 (4.1 to 91.1)	-21.5 (-38.8 to 4.8)	100.1 (57.2 to 167.3)	-13.2 (-31.8 to 12.8)
		Male	13990 (10132 to 18496)	3906 (2886.3 to 5080.8)	412 (307 to 538)	159.3 (118.5 to 202.8)	22868 (18438 to 26866)	3975.5 (3250.7 to 4620.5)	926 (764 to 1065)	177.9 (148.3 to 204.3)	63.5 (22.6 to 129.7)	1.8 (-22.8 to 44)	124.9 (69.5 to 216.8)	11.7 (-13.9 to 56.7)
	Lip and oral cavity cancer	Both	97 (73 to 126)	17.1 (12.9 to 22.1)	3 (2 to 4)	0.7 (0.5 to 0.9)	230 (197 to 267)	19 (16.3 to 21.9)	9 (8 to 11)	0.8 (0.7 to 1)	137.1 (69.6 to 235.6)	11 (-19.9 to 56.3)	180.5 (102 to 295)	17.2 (-14.9 to 66.2)
		Female	32 (24 to 42)	12 (9 to 15.1)	1 (1 to 1)	0.5 (0.4 to 0.6)	95 (79 to 113)	15.4 (12.8 to 18.3)	4 (3 to 5)	0.7 (0.6 to 0.8)	192.6 (114.7 to 324.4)	28.8 (-4.8 to 84.7)	264.8 (168 to 422.3)	37.7 (1.4 to 99.6)
		Male	64 (44 to 92)	21.5 (14.6 to 30.3)	2 (1 to 3)	0.9 (0.6 to 1.3)	135 (110 to 165)	22.8 (18.9 to 27.4)	5 (4 to 6)	1 (0.9 to 1.2)	109.1 (32.6 to 239.2)	6 (-31.6 to 70.4)	140.7 (53.9 to 289)	10.7 (-26.7 to 75.2)
	Nasopharynx cancer	Both	52 (38 to 65)	7.4 (5.5 to 9.1)	1 (1 to 2)	0.3 (0.2 to 0.3)	72 (62 to 84)	5.7 (4.8 to 6.6)	2 (2 to 3)	0.2 (0.2 to 0.2)	39.8 (6.3 to 99)	-23.1 (-40.7 to 7.4)	67.9 (29 to 133.6)	-19.7 (-38 to 11.6)
		Female	20 (13 to 27)	6.1 (4.1 to 7.9)	1 (0 to 1)	0.2 (0.1 to 0.3)	25 (20 to 31)	4 (3.2 to 4.8)	1 (1 to 1)	0.1 (0.1 to 0.2)	22.7 (-11.9 to 101.9)	-34.5 (-52.9 to 4.5)	53.1 (9.4 to 142.9)	-33.5 (-54.4 to 2)
		Male	31 (22 to 42)	8.6 (6.1 to 11.4)	1 (1 to 1)	0.3 (0.2 to 0.4)	47 (38 to 58)	7.4 (6 to 9)	2 (1 to 2)	0.3 (0.2 to 0.3)	50.8 (4.5 to 134.2)	-13.2 (-38.6 to 34)	77 (25.3 to 171.9)	-7 (-33.2 to 42.2)
	Other pharynx cancer	Both	37 (28 to 47)	6.6 (5.1 to 8.2)	1 (1 to 2)	0.3 (0.2 to 0.3)	74 (63 to 88)	6.1 (5.2 to 7.3)	3 (3 to 4)	0.3 (0.2 to 0.3)	99.2 (47.4 to 184.9)	-7.5 (-30.4 to 30.5)	134.6 (74.7 to 229.7)	-0.8 (-24.9 to 39.1)
		Female	17 (12 to 25)	6.6 (4.6 to 8.8)	1 (0 to 1)	0.3 (0.2 to 0.4)	32 (26 to 39)	5.1 (4.2 to 6.2)	1 (1 to 2)	0.2 (0.2 to 0.3)	81.7 (23.5 to 189.9)	-22.3 (-45.8 to 18.8)	123 (56.2 to 241.7)	-16.3 (-42.2 to 28)
		Male	20 (14 to 27)	6.5 (4.7 to 8.9)	1 (0 to 1)	0.3 (0.2 to 0.4)	42 (33 to 52)	7.2 (5.7 to 9)	2 (1 to 2)	0.3 (0.3 to 0.4)	114.8 (41.1 to 229.9)	10.3 (-26.6 to 69.4)	144.1 (62.2 to 279.1)	17.3 (-21 to 79.4)

Province	Cause	Sex	1990				2019				1990-2019 percent change (%)			
			DALYs		Death		DALYs		Death		DALYs		Death	
			Number	Rate	Number	Rate	Number	Rate	Number	Rate	Number	Rate	Number	Rate
	Esophageal cancer	Both	1705 (494 to 2318)	317.4 (92.3 to 431.2)	62 (18 to 84)	13.9 (4 to 18.9)	2983 (1018 to 3648)	255.1 (86.3 to 311.6)	130 (44 to 159)	12.2 (4 to 14.8)	74.9 (30.4 to 152.4)	-19.6 (-39 to 12.7)	111.1 (60.7 to 196.3)	-12.3 (-33.2 to 18.3)
		Female	668 (204 to 971)	271.6 (85.1 to 403.1)	24 (7 to 35)	11.9 (3.8 to 18.9)	1027 (332 to 1311)	171.1 (55.8 to 217)	45 (15 to 56)	8.1 (2.7 to 10.2)	53.8 (8 to 143.3)	-37 (-56 to -3.5)	86.8 (29.8 to 185.3)	-32.4 (-54.8 to -0.2)
		Male	1037 (268 to 1561)	356.2 (93.2 to 524.7)	38 (10 to 56)	15.6 (4.1 to 22.5)	1956 (635 to 2462)	344.2 (110.9 to 433.8)	86 (28 to 107)	16.6 (5.3 to 20.6)	88.6 (28.9 to 204.6)	-3.4 (-32.4 to 53.1)	126.4 (57.4 to 257.7)	6.3 (-24.1 to 65.2)
	Stomach cancer	Both	5891 (4396 to 7248)	1097.7 (837 to 1346.6)	213 (162 to 262)	48.5 (38.2 to 59.1)	8583 (7528 to 9927)	736.2 (646.2 to 850.2)	373 (329 to 429)	34.9 (30.8 to 40.1)	45.7 (13.3 to 103.2)	-32.9 (-47.1 to -8.6)	75.1 (37.5 to 140.1)	-28 (-43 to -4.6)
		Female	2169 (1494 to 2817)	833 (608.6 to 1072.6)	73 (53 to 94)	35.1 (26.8 to 46)	2845 (2400 to 3376)	466.3 (393.4 to 551.6)	120 (102 to 141)	21.4 (18.2 to 25.1)	31.2 (-4.5 to 99.2)	-44 (-58.4 to -20.7)	64.6 (21.9 to 134.8)	-38.9 (-56.1 to -16.5)
		Male	3722 (2670 to 4944)	1313.1 (949.2 to 1728.2)	140 (101 to 187)	60.1 (43.5 to 78.3)	5737 (4728 to 6867)	1025.1 (855.2 to 1221.7)	253 (212 to 301)	49.4 (41.6 to 58.8)	54.2 (10.7 to 131.9)	-21.9 (-43.2 to 15.5)	80.6 (31.1 to 168.7)	-17.8 (-38.4 to 21.6)
	Colon and rectum cancer	Both	874 (666 to 1118)	158.7 (124.1 to 201.5)	30 (23 to 38)	6.8 (5.3 to 8.5)	2744 (2371 to 3173)	227.5 (197.2 to 262.4)	111 (97 to 128)	10.2 (8.9 to 11.8)	213.8 (128.6 to 337)	43.4 (5.5 to 97.8)	272.4 (172.6 to 417.4)	50.5 (12.2 to 105.9)
		Female	412 (296 to 575)	158.9 (116.1 to 214.9)	14 (10 to 19)	6.7 (4.9 to 8.9)	1124 (926 to 1339)	182.1 (151.1 to 216.9)	46 (38 to 54)	8.1 (6.7 to 9.6)	172.6 (79.9 to 321.3)	14.6 (-23.1 to 74.2)	233.4 (122.7 to 404.8)	21.2 (-16 to 80)
		Male	462 (315 to 662)	157.4 (107.9 to 224.5)	16 (11 to 23)	6.8 (4.7 to 9.5)	1620 (1333 to 1951)	276 (228.5 to 331.8)	65 (54 to 79)	12.4 (10.3 to 14.9)	250.6 (127 to 451.9)	75.3 (15.9 to 171.9)	305.6 (167 to 532.3)	83.1 (23.3 to 178.7)
	Liver cancer	Both	379 (283 to 489)	65 (48.2 to 84)	13 (9 to 17)	2.9 (2.1 to 3.7)	1449 (1227 to 1698)	122.7 (103.6 to 143.7)	59 (50 to 70)	5.5 (4.6 to 6.5)	282 (182.8 to 453)	88.6 (37.6 to 173)	364.6 (239.3 to 572.1)	90.5 (37.7 to 179.4)
		Female	144 (99 to 202)	51.4 (35.1 to 75.2)	5 (3 to 7)	2.3 (1.5 to 3.5)	466 (380 to 565)	76.5 (62.2 to 92.4)	19 (15 to 23)	3.4 (2.7 to 4.1)	223.6 (103.5 to 411.1)	48.8 (-10.7 to 134.3)	313.8 (153.9 to 549.6)	47.7 (-14.7 to 142.2)
		Male	235 (162 to 334)	76.1 (52.8 to 106.5)	8 (6 to 12)	3.4 (2.4 to 4.7)	983 (782 to 1213)	171.7 (137.2 to 212.9)	41 (33 to 50)	7.8 (6.3 to 9.5)	317.8 (181.4 to 525.5)	125.6 (51.4 to 242.2)	392.7 (228.9 to 652.9)	129 (52.4 to 244.3)
	Gallbladder and biliary tract cancer	Both	174 (124 to 220)	32.8 (24.4 to 40.4)	6 (5 to 8)	1.5 (1.1 to 1.8)	331 (258 to 390)	28.3 (22.5 to 33.2)	14 (12 to 17)	1.4 (1.1 to 1.6)	90.4 (45.8 to 170.1)	-13.9 (-33.1 to 18.7)	128 (77.6 to 213)	-7.5 (-26.5 to 24.1)
		Female	92 (59 to 124)	37.8 (25.1 to 49.7)	3 (2 to 4)	1.7 (1.2 to 2.2)	162 (112 to 201)	27.1 (18.8 to 33.7)	7 (5 to 9)	1.3 (0.9 to 1.6)	76.5 (24.1 to 171.4)	-28.4 (-48.3 to 6.9)	115.6 (55.2 to 215.4)	-22.6 (-42.8 to 10.6)
		Male	82 (55 to 113)	28.6 (19.4 to 38.7)	3 (2 to 4)	1.3 (0.9 to 1.7)	170 (120 to 207)	29.6 (21.6 to 36)	7 (5 to 9)	1.4 (1.1 to 1.7)	105.8 (41.4 to 226.7)	3.4 (-27.2 to 63.4)	141.6 (69.4 to 280.9)	10.8 (-20.1 to 69.5)
	Pancreatic cancer	Both	258 (185 to 342)	48.5 (35.2 to 64.3)	10 (7 to 13)	2.1 (1.5 to 2.8)	1245 (1072 to 1452)	106.3 (91.9 to 123.8)	53 (46 to 62)	4.9 (4.3 to 5.7)	382.8 (243.7 to 617.9)	119.3 (56.6 to 227.3)	460.4 (296.9 to 731.9)	131.2 (66.3 to 242.2)
		Female	96 (68 to 132)	39.4 (28.7 to 54)	4 (3 to 5)	1.7 (1.3 to 2.4)	483 (394 to 593)	81 (66.4 to 99.1)	21 (17 to 25)	3.8 (3.1 to 4.6)	403.9 (231.6 to 660.7)	105.4 (35.7 to 206.2)	502.7 (294 to 806.1)	119.7 (43.1 to 222.5)
		Male	162 (103 to 249)	56.3 (35.9 to 84.2)	6 (4 to 9)	2.5 (1.6 to 3.7)	762 (620 to 924)	132.8 (108.6 to 161)	32 (26 to 39)	6.1 (5.1 to 7.4)	370.3 (193.2 to 721.9)	135.9 (49.4 to 310)	435.8 (235.3 to 822)	145.9 (54.6 to 315.6)
	Larynx cancer	Both	372 (274 to 471)	66 (50.1 to 83.1)	13 (10 to 16)	2.7 (2 to 3.3)	642 (535 to 749)	53.5 (44.9 to 62.1)	25 (22 to 29)	2.3 (2 to 2.7)	72.5 (28.9 to 149.6)	-19.1 (-38.6 to 13.1)	100.4 (52.9 to 184.8)	-12.9 (-32.8 to 21.1)
		Female	97 (67 to 129)	35.7 (25.8 to 47)	3 (2 to 4)	1.4 (1 to 1.8)	149 (121 to 181)	24.1 (19.6 to 29)	6 (5 to 7)	1 (0.8 to 1.2)	54 (8.6 to 127.3)	-32.6 (-50.9 to -1.3)	89.6 (39.1 to 180.5)	-25.5 (-44.4 to 5.2)

Province	Cause	Sex	1990				2019				1990-2019 percent change (%)			
			DALYs		Death		DALYs		Death		DALYs		Death	
			Number	Rate	Number	Rate	Number	Rate	Number	Rate	Number	Rate	Number	Rate
		Male	275 (195 to 372)	91.8 (65.4 to 121.7)	10 (7 to 13)	3.8 (2.7 to 5)	493 (395 to 591)	84.4 (67.9 to 100.9)	20 (16 to 23)	3.7 (3 to 4.4)	79 (27.5 to 174.5)	-8.1 (-33.4 to 38.2)	103.8 (47 to 210.3)	-2 (-28.4 to 46)
	Tracheal, bronchus, and lung cancer	Both	1675 (1225 to 2233)	298.7 (222.7 to 393.3)	58 (43 to 77)	12.5 (9.3 to 16.4)	4568 (3926 to 5253)	382.7 (329.6 to 439.1)	185 (160 to 212)	16.9 (14.6 to 19.4)	172.7 (93.5 to 296.5)	28.1 (-8.6 to 84)	217 (127.5 to 355.5)	35.4 (-2.5 to 92.4)
		Female	355 (254 to 509)	129 (94.8 to 181.3)	11 (8 to 16)	5.1 (3.8 to 7.1)	1278 (1050 to 1532)	205.7 (170 to 244.5)	51 (42 to 60)	8.9 (7.4 to 10.5)	260.4 (124.3 to 420.4)	59.5 (0.1 to 127.5)	349.2 (178.4 to 538.4)	73.8 (10.8 to 144.3)
		Male	1320 (890 to 1865)	441.7 (307.4 to 612.7)	47 (32 to 66)	18.9 (13.5 to 25.9)	3290 (2705 to 3942)	570.1 (470.7 to 680.4)	135 (113 to 159)	25.5 (21.4 to 30)	149.2 (64.6 to 295.7)	29.1 (-13.7 to 102.4)	185.5 (90.3 to 348)	35.1 (-7.7 to 103.4)
	Malignant skin melanoma	Both	110 (58 to 168)	18.1 (9.4 to 27.2)	3 (2 to 5)	0.8 (0.4 to 1.1)	154 (107 to 192)	12.5 (8.4 to 15.3)	6 (4 to 7)	0.5 (0.3 to 0.7)	39.6 (-11.7 to 138.8)	-31.3 (-54.6 to 16)	73.9 (18.5 to 180.3)	-30.5 (-51.5 to 12.7)
		Female	46 (22 to 79)	15.3 (7.5 to 24.2)	1 (1 to 2)	0.6 (0.3 to 0.9)	61 (39 to 80)	9.6 (6.1 to 12.4)	2 (1 to 3)	0.4 (0.2 to 0.5)	32.6 (-21.7 to 139.6)	-37.5 (-60.5 to 3.7)	67.5 (9.9 to 167.6)	-37.9 (-56.6 to -6.9)
		Male	65 (32 to 103)	20.6 (9.6 to 32.5)	2 (1 to 3)	0.9 (0.4 to 1.5)	93 (56 to 131)	15.6 (9.1 to 21.1)	4 (2 to 5)	0.7 (0.4 to 0.9)	44.5 (-8.7 to 161.4)	-24.4 (-51.6 to 38.1)	78 (16.2 to 212.3)	-23.4 (-50.7 to 43.1)
	Non-melanoma skin cancer	Both	33 (24 to 42)	6.8 (5.1 to 8.6)	1 (1 to 2)	0.4 (0.3 to 0.5)	98 (83 to 115)	8.6 (7.3 to 10)	5 (4 to 6)	0.5 (0.4 to 0.6)	198.4 (118.5 to 331.3)	26.3 (-7.4 to 81.8)	265.5 (165.2 to 432.7)	33.9 (-1.6 to 94.9)
		Female	5 (4 to 8)	2.3 (1.5 to 3.3)	0 (0 to 0)	0.1 (0.1 to 0.2)	23 (18 to 28)	3.8 (2.9 to 4.7)	1 (1 to 1)	0.2 (0.2 to 0.3)	327.5 (157.8 to 611.3)	62.6 (3.1 to 179.3)	415.8 (223.3 to 802.3)	63.5 (-1.3 to 206.3)
		Male	27 (20 to 37)	10.7 (7.9 to 14.1)	1 (1 to 1)	0.6 (0.4 to 0.8)	75 (62 to 90)	13.7 (11.3 to 16.4)	4 (3 to 5)	0.8 (0.6 to 0.9)	173.5 (92.3 to 302.4)	27.3 (-9.6 to 86)	236.9 (137.6 to 398.5)	34.2 (-4.3 to 96.1)
	Breast cancer	Both	949 (684 to 1290)	157.3 (111.7 to 215.2)	27 (19 to 38)	5.1 (3.6 to 7.2)	2178 (1814 to 2627)	161.6 (135.4 to 193.4)	67 (56 to 79)	5.4 (4.6 to 6.5)	129.5 (56.1 to 233.8)	2.7 (-30.2 to 52)	143.3 (63.2 to 262)	5.9 (-31.5 to 59.5)
		Female	937 (674 to 1279)	326.9 (231.3 to 449)	27 (19 to 37)	10.8 (7.4 to 15.2)	2158 (1796 to 2603)	318.5 (266.7 to 381.6)	66 (55 to 79)	10.6 (8.8 to 12.5)	130.4 (56.2 to 236.5)	-2.6 (-34.3 to 44.4)	144.2 (62.6 to 263.4)	-1.8 (-36.9 to 48.3)
		Male	13 (8 to 19)	4 (2.5 to 5.9)	0 (0 to 1)	0.2 (0.1 to 0.2)	20 (15 to 28)	3.4 (2.6 to 4.7)	1 (1 to 1)	0.1 (0.1 to 0.2)	63.4 (-4.7 to 182.8)	-13.5 (-47.8 to 52.5)	87 (11.5 to 230.4)	-9.2 (-45.1 to 62.5)
	Cervical cancer	Female	298 (178 to 405)	103 (64.5 to 137.6)	9 (5 to 12)	3.6 (2.3 to 4.7)	378 (267 to 466)	57.5 (40.3 to 70.6)	13 (9 to 16)	2.2 (1.5 to 2.6)	26.9 (-7.9 to 105.7)	-44.2 (-58.5 to -12.7)	50.1 (11.4 to 133.3)	-39.7 (-55.5 to -11.8)
	Uterine cancer	Female	36 (24 to 59)	13.9 (9.4 to 23)	1 (1 to 2)	0.5 (0.4 to 1)	118 (79 to 149)	18.6 (12.9 to 23.3)	4 (3 to 5)	0.7 (0.5 to 0.9)	224.7 (70.2 to 433.6)	34.1 (-29 to 119)	249.5 (93.2 to 472.4)	30.4 (-28.3 to 108.8)
	Ovarian cancer	Female	137 (80 to 279)	45.8 (27.4 to 90)	4 (2 to 8)	1.5 (0.9 to 3)	528 (374 to 649)	81.2 (57.6 to 99.4)	17 (13 to 21)	2.9 (2.1 to 3.5)	284.3 (60.4 to 591.4)	77.2 (-23.2 to 212.8)	341.2 (91.1 to 679.1)	86.9 (-16.4 to 222.6)
	Prostate cancer	Male	345 (239 to 466)	165.4 (116.6 to 222.8)	17 (11 to 22)	10.3 (7.1 to 14.1)	1230 (939 to 1472)	248.5 (192.2 to 298.3)	70 (55 to 85)	15.2 (12 to 18.4)	256.4 (158.3 to 432.8)	50.3 (10 to 124.1)	325.8 (209.4 to 543.9)	47.8 (8.1 to 120.1)
	Testicular cancer	Male	33 (19 to 57)	5.8 (3.5 to 8.6)	1 (0 to 1)	0.1 (0.1 to 0.2)	71 (53 to 94)	10.4 (7.9 to 13.6)	1 (1 to 2)	0.2 (0.2 to 0.3)	112.7 (13.5 to 317.6)	77.5 (7.6 to 216.4)	170 (63.9 to 376.6)	78.3 (11.5 to 188)
	Kidney cancer	Both	454 (312 to 645)	46.6 (32.8 to 62)	9 (6 to 12)	1.5 (1 to 2)	793 (662 to 922)	66.2 (55.7 to 76.8)	29 (24 to 34)	2.6 (2.2 to 3)	74.8 (14.1 to 164.4)	42.1 (0.2 to 115.8)	211.8 (119.3 to 378.5)	75.4 (27 to 194.7)
		Female	190 (122 to 315)	38.5 (27.8 to 53.5)	4 (3 to 5)	1.2 (0.8 to 1.6)	262 (217 to 316)	43.5 (35.9 to 52.3)	9 (7 to 10)	1.5 (1.3 to 1.8)	38.2 (-18.6 to 128.9)	12.9 (-23.6 to 64)	146.1 (66.9 to 264.6)	28.7 (-9.3 to 120.8)
		Male	264 (153 to 425)	53.7 (32.8 to 80.3)	6 (3 to 8)	1.7 (1 to 2.5)	530 (413 to 645)	90.4 (70.9 to 109.9)	20 (16 to 24)	3.8 (2.9 to 4.5)	101.1 (16.2 to 257.8)	68.4 (4 to 194)	252.7 (121 to 525.7)	115.4 (37 to 290.1)

Province	Cause	Sex	1990				2019				1990-2019 percent change (%)			
			DALYs		Death		DALYs		Death		DALYs		Death	
			Number	Rate	Number	Rate	Number	Rate	Number	Rate	Number	Rate	Number	Rate
	Bladder cancer	Both	174 (122 to 229)	35.9 (25.2 to 46.8)	7 (5 to 9)	1.8 (1.3 to 2.3)	461 (389 to 541)	41.3 (34.9 to 48.5)	21 (18 to 25)	2.1 (1.8 to 2.5)	165 (89 to 315.5)	15 (-16.8 to 79.5)	210 (121.2 to 387.1)	16.7 (-15.8 to 83.3)
		Female	36 (24 to 49)	16.6 (11.1 to 22.7)	1 (1 to 2)	0.9 (0.6 to 1.2)	78 (64 to 93)	13.7 (11.3 to 16.4)	4 (3 to 5)	0.8 (0.6 to 0.9)	117.7 (44.1 to 252.3)	-17.7 (-44.3 to 32.2)	175.3 (86.4 to 342.1)	-12.4 (-42 to 43.2)
		Male	138 (91 to 192)	52.4 (35.1 to 70.9)	5 (4 to 8)	2.6 (1.8 to 3.5)	383 (314 to 457)	70.8 (58 to 84.6)	18 (14 to 21)	3.5 (2.9 to 4.3)	177.2 (81.5 to 358.6)	35.1 (-9 to 119.8)	219.1 (115.8 to 426.8)	34 (-8.5 to 118.7)
	Brain and central nervous system cancer	Both	1669 (1003 to 2784)	150.4 (100 to 227.6)	30 (20 to 46)	3.8 (2.7 to 5.3)	1860 (1092 to 2329)	146 (86.6 to 181.3)	52 (31 to 65)	4.4 (2.5 to 5.5)	11.5 (-53.2 to 79.7)	-2.9 (-55.2 to 45)	74.6 (-19.7 to 161.1)	16 (-40.4 to 70.2)
		Female	669 (302 to 1497)	123.5 (63.9 to 246.7)	12 (6 to 23)	3 (1.8 to 5.5)	792 (492 to 997)	125.8 (78 to 159)	22 (13 to 28)	3.7 (2.2 to 4.7)	18.5 (-59.2 to 170.1)	1.8 (-60.3 to 110.7)	89.9 (-28.4 to 291.2)	21 (-47.2 to 133.7)
		Male	1000 (497 to 1781)	174.6 (98.3 to 284.1)	18 (10 to 30)	4.4 (2.8 to 6.7)	1067 (510 to 1453)	166.5 (79.9 to 224.9)	30 (14 to 41)	5.1 (2.4 to 6.9)	6.8 (-58.2 to 106.5)	-4.6 (-57.5 to 69.5)	64.9 (-28 to 194.6)	15.6 (-42.8 to 93.4)
	Thyroid cancer	Both	60 (44 to 75)	10.1 (7.7 to 12.5)	2 (1 to 2)	0.4 (0.3 to 0.5)	169 (118 to 201)	13.6 (9.6 to 16.1)	6 (4 to 7)	0.5 (0.4 to 0.6)	179.5 (83 to 300.7)	34.9 (-10.3 to 90.4)	211.9 (120.6 to 338.3)	29.4 (-10.5 to 77.8)
		Female	39 (27 to 52)	14.1 (10.2 to 18.5)	1 (1 to 2)	0.6 (0.4 to 0.7)	97 (59 to 123)	15.4 (9.6 to 19.4)	3 (2 to 4)	0.6 (0.4 to 0.7)	150.7 (50.1 to 287.4)	9.4 (-33.4 to 64.3)	174.7 (75.6 to 312.5)	1.1 (-37.6 to 48.4)
		Male	22 (15 to 31)	6.6 (4.5 to 9.4)	1 (0 to 1)	0.3 (0.2 to 0.4)	72 (54 to 88)	11.8 (9.1 to 14.5)	2 (2 to 3)	0.5 (0.4 to 0.6)	230.9 (107.1 to 429.1)	79.5 (13.2 to 186.3)	277.1 (140 to 507.1)	81.9 (15.7 to 190.7)
	Mesothelioma	Both	10 (6 to 16)	1.6 (1.1 to 2.4)	0 (0 to 0)	0.1 (0 to 0.1)	23 (18 to 31)	1.7 (1.4 to 2.4)	1 (1 to 1)	0.1 (0.1 to 0.1)	135 (38.5 to 295.9)	8.6 (-33.3 to 78.8)	165.8 (69.5 to 329.7)	12.3 (-24.7 to 77.7)
		Female	5 (2 to 10)	1.7 (0.8 to 3.1)	0 (0 to 0)	0.1 (0 to 0.1)	7 (4 to 10)	1.1 (0.6 to 1.5)	0 (0 to 0)	0 (0 to 0.1)	39.5 (-34 to 209.3)	-36.9 (-67.6 to 27.2)	60 (-17.4 to 213.8)	-34.4 (-63.1 to 21.9)
		Male	5 (3 to 8)	1.5 (1 to 2.4)	0 (0 to 0)	0.1 (0 to 0.1)	16 (12 to 24)	2.5 (1.8 to 4)	1 (0 to 1)	0.1 (0.1 to 0.2)	238.2 (87.1 to 476.2)	58.5 (-9.8 to 167.3)	267.1 (113.5 to 500.1)	57.5 (-4.3 to 164.6)
	Hodgkin lymphoma	Both	280 (150 to 407)	28.7 (17 to 38.8)	5 (3 to 8)	0.8 (0.5 to 1)	311 (130 to 443)	23.5 (9.8 to 33.5)	8 (3 to 11)	0.6 (0.3 to 0.9)	11.1 (-29.1 to 60)	-18.1 (-53.2 to 18.5)	44.5 (-13.6 to 109.4)	-14.6 (-52.9 to 22.8)
		Female	101 (45 to 160)	22.7 (11.2 to 33.2)	2 (1 to 3)	0.6 (0.3 to 0.9)	110 (42 to 165)	16.9 (6.4 to 25.5)	3 (1 to 4)	0.4 (0.2 to 0.7)	9.1 (-29.4 to 83.1)	-25.5 (-56.3 to 14.7)	34.3 (-17.4 to 107.2)	-30.6 (-60.2 to 7.2)
		Male	179 (90 to 269)	34.4 (19.3 to 48)	3 (2 to 5)	0.9 (0.5 to 1.2)	201 (74 to 294)	30.1 (11.1 to 43.8)	5 (2 to 8)	0.9 (0.3 to 1.2)	12.2 (-34.8 to 73.3)	-12.4 (-53.8 to 35.6)	50.5 (-17.8 to 133.6)	-1.2 (-49.7 to 56.1)
	Non-Hodgkin lymphoma	Both	425 (300 to 559)	51.7 (38.9 to 65.5)	10 (7 to 13)	1.7 (1.3 to 2.1)	798 (682 to 919)	63.3 (54.5 to 72.6)	25 (22 to 29)	2.2 (1.9 to 2.5)	87.8 (34.8 to 177.8)	22.4 (-8.5 to 70.8)	149.8 (90.3 to 247.7)	29.1 (1.2 to 79.5)
		Female	148 (97 to 217)	38.7 (25.8 to 53.7)	3 (2 to 5)	1.3 (0.8 to 1.7)	264 (214 to 320)	41.8 (34.2 to 50.5)	8 (7 to 10)	1.4 (1.1 to 1.7)	78.4 (8.3 to 207.9)	7.9 (-30 to 82.1)	136.1 (53.5 to 300.1)	8.3 (-25.8 to 91.4)
		Male	277 (189 to 377)	63.5 (45.5 to 84.4)	7 (5 to 9)	2 (1.5 to 2.7)	534 (435 to 635)	85.1 (70.1 to 100.7)	17 (14 to 20)	3 (2.5 to 3.5)	92.8 (36 to 199.1)	34 (-2.6 to 97.1)	157.2 (86.7 to 279.3)	46.5 (9.2 to 108.1)
	Multiple myeloma	Both	108 (76 to 157)	19.8 (14.2 to 28.8)	4 (3 to 6)	0.8 (0.6 to 1.2)	233 (184 to 332)	19.5 (15.2 to 28.4)	9 (7 to 13)	0.8 (0.6 to 1.2)	116.2 (45.6 to 205.5)	-1.2 (-32 to 37.3)	137.6 (63 to 228.2)	0.7 (-28.8 to 35.1)
		Female	48 (33 to 68)	19.7 (14 to 26.9)	2 (1 to 2)	0.8 (0.6 to 1.1)	89 (71 to 119)	14.6 (11.5 to 19.6)	3 (3 to 5)	0.6 (0.5 to 0.8)	86.1 (24.8 to 179)	-26.2 (-48 to 8.5)	100.3 (40.8 to 196.1)	-27.7 (-47.9 to 4.6)
		Male	60 (38 to 99)	20 (12.5 to 34.1)	2 (1 to 4)	0.8 (0.5 to 1.4)	144 (103 to 228)	24.8 (17.7 to 40.3)	6 (4 to 9)	1.1 (0.7 to 1.8)	140 (44.2 to 271)	24.1 (-24.4 to 90.5)	168 (64 to 314.6)	30.8 (-20.2 to 97)
	Leukemia	Both	5357 (2864 to 8303)	427.4 (244.5 to 620)	86 (49 to 125)	9.8 (6.3 to 13.3)	2753 (1744 to 3405)	223.8 (143.3 to 276.3)	79 (53 to 96)	6.9 (4.7 to 8.4)	-48.6 (-65.8 to -18.4)	-47.6 (-63.1 to -22.9)	-8.1 (-34.6 to 34.8)	-30.3 (-46.3 to -3.5)

Province	Cause	Sex	1990				2019				1990-2019 percent change (%)			
			DALYs		Death		DALYs		Death		DALYs		Death	
			Number	Rate	Number	Rate	Number	Rate	Number	Rate	Number	Rate	Number	Rate
		Female	2733 (896 to 4631)	427.5 (199.6 to 673.4)	41 (19 to 65)	9 (5.2 to 12.7)	1075 (652 to 1294)	173.8 (106.1 to 208.7)	29 (19 to 34)	4.8 (3.2 to 5.8)	-60.7 (-76.4 to -8.1)	-59.3 (-73.7 to -29.2)	-30.1 (-54 to 31.4)	-46.3 (-61.8 to -21.1)
		Male	2624 (872 to 4300)	425 (182.4 to 656.8)	45 (19 to 69)	10.6 (5.7 to 15.3)	1678 (895 to 2207)	275.1 (148.7 to 360)	50 (28 to 65)	9 (5.2 to 11.7)	-36.1 (-58.3 to 44.4)	-35.3 (-56.3 to 17.2)	12.1 (-24.5 to 115.8)	-15 (-39.2 to 34.8)
	Other malignant neoplasms	Both	2779 (1787 to 3729)	275.1 (175.4 to 358.7)	54 (35 to 71)	8.2 (5.1 to 11)	2602 (1640 to 3159)	216.8 (135.4 to 263)	83 (51 to 103)	7.5 (4.5 to 9.3)	-6.4 (-34.1 to 28.9)	-21.2 (-40.3 to 9)	54.2 (16.6 to 115.9)	-8.5 (-29.1 to 32.9)
		Female	1276 (761 to 1846)	240.9 (158.7 to 320)	22 (15 to 30)	6.5 (4.7 to 8.4)	1118 (927 to 1314)	186.2 (155.1 to 218.2)	34 (27 to 40)	6 (4.7 to 7)	-12.4 (-41.7 to 28.8)	-22.7 (-44.5 to 8.9)	51.2 (9 to 114.3)	-8.3 (-33.7 to 30.4)
		Male	1503 (629 to 2438)	303.8 (135.8 to 451.4)	32 (14 to 47)	9.8 (4.5 to 14.6)	1484 (690 to 1950)	249.5 (114 to 328.7)	49 (21 to 66)	9.2 (3.9 to 12.3)	-1.3 (-38.7 to 57.8)	-17.9 (-44.2 to 26.9)	56.4 (5.3 to 142.1)	-5.7 (-35.8 to 50.1)
	Other neoplasms	Both	44 (29 to 58)	6 (3.8 to 7.6)	1 (1 to 1)	0.2 (0.1 to 0.3)	59 (49 to 74)	5.1 (4.3 to 6.4)	2 (2 to 3)	0.2 (0.2 to 0.3)	32.7 (-2.2 to 104.2)	-14.6 (-34.7 to 31.5)	102.6 (43.4 to 256.1)	-10.5 (-38.6 to 60.9)
		Female	20 (14 to 31)	5.6 (3.4 to 7.9)	0 (0 to 1)	0.2 (0.1 to 0.3)	24 (20 to 30)	4.1 (3.4 to 5.1)	1 (1 to 1)	0.1 (0.1 to 0.2)	20.8 (-22.9 to 85.3)	-25.9 (-50 to 29.5)	88 (16.8 to 282.9)	-26.2 (-53.7 to 82.8)
		Male	24 (14 to 35)	6.3 (3.8 to 8.4)	1 (0 to 1)	0.2 (0.1 to 0.3)	34 (27 to 48)	6.1 (4.7 to 8.7)	1 (1 to 2)	0.2 (0.2 to 0.4)	42.6 (-8.3 to 158.6)	-2.2 (-32.6 to 52.5)	112.8 (33 to 277.8)	5.4 (-32.7 to 70.7)
	Cardiovascular diseases	Both	54943 (46153 to 64501)	9899.8 (8378.2 to 11497.3)	1844 (1557 to 2159)	484.5 (413.9 to 557)	71541 (64642 to 78492)	6393.6 (5792.1 to 6968.7)	3503 (3148 to 3818)	349.9 (312.8 to 381.8)	30.2 (8.9 to 56.7)	-35.4 (-46.1 to -23.9)	90 (56 to 126.1)	-27.8 (-38.9 to -16)
		Female	21791 (17940 to 26337)	8464.7 (7098.9 to 10118)	733 (608 to 882)	427.2 (353.5 to 502.7)	28018 (24894 to 31272)	5003.3 (4442.2 to 5565.2)	1479 (1296 to 1635)	289.6 (251.8 to 319.2)	28.6 (2.7 to 61)	-40.9 (-52.2 to -27.3)	101.9 (61.8 to 149.9)	-32.2 (-44.4 to -17.2)
		Male	33151 (26483 to 41167)	11156.4 (8940.1 to 13805.4)	1111 (873 to 1387)	536.1 (435.2 to 655.3)	43524 (38189 to 48957)	7841.5 (6898 to 8756.6)	2024 (1772 to 2254)	414.1 (362.1 to 458.7)	31.3 (1.6 to 66)	-29.7 (-45.8 to -11.2)	82.1 (38.7 to 132.1)	-22.8 (-39.2 to -3.8)
	Rheumatic heart disease	Both	852 (636 to 1128)	87.9 (65.2 to 118.6)	12 (9 to 18)	2 (1.4 to 3.2)	775 (613 to 971)	58.9 (47.6 to 72.9)	15 (12 to 18)	1.4 (1.1 to 1.6)	-9.1 (-34.2 to 17.9)	-33 (-54 to -12.6)	21.5 (-29.2 to 80.9)	-30.3 (-63.8 to 9.7)
		Female	470 (306 to 648)	97.4 (65.5 to 132.4)	7 (4 to 10)	2.2 (1.4 to 3.6)	366 (283 to 469)	56.1 (43.9 to 71.2)	7 (5 to 8)	1.2 (1 to 1.5)	-22 (-46.2 to 22.7)	-42.4 (-60.4 to -10.7)	1.5 (-38.8 to 91.4)	-43.9 (-70.9 to 1)
		Male	382 (246 to 562)	78.5 (51.3 to 115.9)	6 (3 to 10)	1.8 (1 to 3.1)	408 (318 to 522)	61.9 (48.8 to 78.5)	8 (6 to 11)	1.5 (1.2 to 1.9)	6.8 (-33.1 to 69.3)	-21.2 (-52.3 to 21.2)	44.8 (-26.9 to 182.8)	-13.1 (-59.8 to 64.9)
	Ischemic heart disease	Both	27253 (21729 to 33137)	5620.8 (4619 to 6679.2)	1086 (890 to 1296)	293 (245.7 to 341.8)	40225 (35942 to 44613)	3590.9 (3220.2 to 3958.4)	2099 (1875 to 2302)	209.2 (186.3 to 230.1)	47.6 (16.6 to 85.6)	-36.1 (-48.1 to -21.4)	93.4 (56.8 to 139.9)	-28.6 (-40.5 to -13.6)
		Female	9129 (7155 to 11325)	4320.2 (3479.4 to 5202.9)	391 (314 to 473)	242.4 (194.4 to 286.8)	14198 (12432 to 15884)	2560.7 (2251 to 2844.6)	843 (734 to 937)	165.7 (143.7 to 184.4)	55.5 (20.3 to 105.5)	-40.7 (-52.7 to -24.4)	115.9 (73.1 to 175.2)	-31.6 (-43.6 to -13.9)
		Male	18124 (13674 to 23292)	6757.3 (5248.4 to 8506.1)	695 (535 to 879)	338.1 (270.3 to 414.2)	26027 (22442 to 29745)	4661.7 (4054.1 to 5286.9)	1256 (1099 to 1418)	255.5 (223.1 to 287.3)	43.6 (5.2 to 96.1)	-31 (-48.4 to -8.5)	80.7 (36.7 to 138.5)	-24.4 (-40.6 to -3.6)
	Stroke	Both	11688 (9825 to 14144)	2289.6 (1903.9 to 2751)	402 (324 to 493)	111.8 (87.9 to 136.9)	16181 (14354 to 18068)	1456.5 (1293.1 to 1618.5)	747 (654 to 832)	74.8 (65.2 to 83.6)	38.4 (12.4 to 68.3)	-36.4 (-49 to -21.5)	86 (45.8 to 136.6)	-33 (-47.5 to -13.5)
		Female	5234 (4265 to 6509)	2202.5 (1755.5 to 2765.8)	175 (135 to 223)	105.2 (79.4 to 130.4)	7257 (6265 to 8283)	1285.1 (1111.6 to 1463.7)	336 (286 to 386)	65.4 (55.4 to 75.1)	38.7 (6 to 77.6)	-41.7 (-54.9 to -23.8)	91.9 (43.3 to 162.1)	-37.8 (-52.8 to -13.5)

Province	Cause	Sex	1990				2019				1990-2019 percent change (%)			
			DALYs		Death		DALYs		Death		DALYs		Death	
			Number	Rate	Number	Rate	Number	Rate	Number	Rate	Number	Rate	Number	Rate
		Male	6455 (5068 to 8285)	2368.2 (1861.1 to 3016.6)	226 (172 to 296)	118.4 (88.9 to 152.4)	8924 (7641 to 10243)	1637.6 (1401.5 to 1864.9)	411 (349 to 467)	85 (71.8 to 96.6)	38.3 (3.2 to 82.8)	-30.9 (-48.7 to -9.5)	81.5 (32.8 to 146)	-28.3 (-46.5 to -5)
	Hypertensive heart disease	Both	3234 (2225 to 4275)	740.7 (520 to 990.2)	139 (94 to 187)	42.2 (29.2 to 56.4)	6664 (5594 to 7569)	618.5 (514.7 to 704)	365 (291 to 419)	37.1 (29 to 42.9)	106.1 (46.3 to 203.5)	-16.5 (-39.3 to 21.2)	162.8 (82.3 to 288.3)	-12.1 (-39.6 to 27.4)
		Female	1518 (1029 to 2258)	762.8 (517.2 to 1185.7)	68 (46 to 109)	44.4 (29.3 to 74)	2953 (2332 to 3514)	539.7 (422.6 to 642.4)	174 (129 to 210)	34.2 (25.2 to 41.5)	94.6 (17.5 to 202.9)	-29.2 (-59.8 to 7.5)	154.2 (40.5 to 288.9)	-22.9 (-59.8 to 20)
		Male	1716 (1045 to 2416)	721.2 (450.8 to 990.7)	70 (42 to 99)	40.1 (24.8 to 54.3)	3711 (2946 to 4388)	701.2 (549.4 to 836)	191 (144 to 230)	40.2 (30.1 to 48.1)	116.2 (44.7 to 253.1)	-2.8 (-32.7 to 54.1)	171.1 (78.4 to 342.9)	0.3 (-30.6 to 61.7)
	Non-rheumatic valvular heart disease	Both	252 (175 to 336)	41.5 (30.8 to 52.6)	8 (6 to 10)	1.8 (1.4 to 2.2)	473 (407 to 549)	39 (33.7 to 45.1)	19 (17 to 23)	1.8 (1.6 to 2.1)	87.7 (33.3 to 177.1)	-5.9 (-29.4 to 32.6)	150.3 (87.8 to 251.9)	1.8 (-21.2 to 35.9)
		Female	124 (77 to 180)	44.8 (30.9 to 59.3)	4 (3 to 5)	2 (1.4 to 2.7)	207 (171 to 247)	34.2 (28.3 to 40.8)	9 (7 to 11)	1.7 (1.4 to 2)	67 (10.7 to 179.9)	-23.7 (-45.7 to 16.4)	131.8 (61.8 to 256.7)	-16.3 (-42.7 to 23.7)
		Male	128 (83 to 189)	38.4 (26.4 to 54.9)	4 (3 to 5)	1.6 (1.1 to 2.2)	266 (211 to 325)	44 (35.3 to 53.9)	10 (8 to 13)	1.9 (1.6 to 2.4)	107.6 (26.8 to 243.5)	14.5 (-27.8 to 76.8)	169.3 (69.8 to 319.9)	25.4 (-17.5 to 85.7)
	Cardiomyopathy and myocarditis	Both	648 (369 to 1487)	65.5 (44 to 119.8)	13 (8 to 24)	2.2 (1.6 to 3.4)	419 (288 to 770)	35.9 (24.7 to 65.7)	15 (10 to 28)	1.4 (0.9 to 2.6)	-35.3 (-66 to 55.1)	-45.2 (-65.9 to 3.3)	17.1 (-29.6 to 128.9)	-36.8 (-59.9 to 6.8)
		Female	280 (139 to 645)	57.1 (34.7 to 108.3)	5 (3 to 10)	2 (1.2 to 3.8)	131 (84 to 264)	22.8 (14.9 to 45)	5 (3 to 10)	0.9 (0.6 to 1.8)	-53.3 (-79.6 to 43)	-60.1 (-77.6 to -9.8)	-9 (-49.4 to 113.9)	-55.1 (-75.7 to -17.2)
		Male	368 (188 to 935)	73.1 (44.3 to 148)	7 (4 to 15)	2.4 (1.6 to 3.9)	289 (190 to 545)	49.3 (32.7 to 93.2)	10 (6 to 20)	1.9 (1.2 to 3.8)	-21.6 (-62.4 to 89.1)	-32.5 (-62.6 to 32.4)	35.9 (-25.7 to 173.2)	-19.1 (-53.5 to 39.5)
	Atrial fibrillation and flutter	Both	254 (193 to 328)	72.5 (55.1 to 92.1)	7 (5 to 9)	2.8 (1.8 to 3.6)	834 (664 to 1035)	82.3 (66.2 to 101.8)	33 (28 to 38)	3.6 (3 to 4.1)	228.1 (188.5 to 281.5)	13.6 (-0.7 to 35.4)	386.9 (273.2 to 613.7)	29.6 (-1.9 to 98.6)
		Female	130 (96 to 168)	82.9 (58.5 to 107.4)	4 (3 to 6)	3.5 (2 to 4.8)	449 (355 to 557)	85.8 (68.2 to 106.3)	19 (15 to 22)	3.8 (3.1 to 4.5)	244.4 (190.5 to 347.4)	3.4 (-14.1 to 39.7)	331.8 (209.2 to 693.5)	9 (-22.6 to 107.6)
		Male	124 (88 to 166)	62 (45.5 to 81.7)	3 (2 to 4)	2 (1.3 to 2.8)	385 (295 to 481)	78.6 (60 to 97.7)	15 (10 to 18)	3.4 (2.2 to 4.2)	211 (152.1 to 278)	26.6 (3.5 to 52.8)	480.3 (273 to 774.2)	69.6 (10.2 to 149.8)
	Aortic aneurysm	Both	109 (75 to 151)	21.8 (15.6 to 29.4)	4 (3 to 6)	1.1 (0.8 to 1.4)	236 (200 to 278)	20.8 (17.8 to 24.3)	12 (10 to 14)	1.1 (1 to 1.3)	117.5 (45.2 to 237.9)	-4.4 (-34.1 to 44.4)	174.1 (90.5 to 311.5)	2.4 (-27.1 to 47.7)
		Female	25 (19 to 35)	12.1 (9.2 to 15.9)	1 (1 to 1)	0.7 (0.5 to 0.9)	50 (42 to 59)	8.6 (7.2 to 10.1)	3 (2 to 3)	0.5 (0.4 to 0.6)	95.4 (34.6 to 181.4)	-29.1 (-48.6 to -1.5)	134.9 (75.1 to 222.9)	-28.8 (-47.5 to -3.6)
		Male	83 (50 to 124)	30.3 (18.7 to 44.5)	3 (2 to 5)	1.5 (0.9 to 2.1)	187 (153 to 225)	33.8 (27.8 to 40.7)	9 (8 to 11)	1.8 (1.5 to 2.2)	124.2 (36.1 to 304.1)	11.5 (-30.6 to 96.3)	187.6 (78.9 to 403.2)	22.5 (-20.8 to 110)
	Peripheral artery disease	Both	25 (14 to 43)	7 (3.9 to 11.5)	0 (0 to 0)	0.1 (0.1 to 0.2)	118 (83 to 166)	11.6 (8.1 to 16.4)	4 (3 to 5)	0.4 (0.3 to 0.5)	366.4 (250.1 to 559.5)	65 (23.7 to 129.3)	1416 (676.8 to 2266)	330.6 (116.7 to 578.7)
		Female	13 (7 to 23)	8.2 (4.4 to 14.1)	0 (0 to 0)	0.1 (0 to 0.2)	57 (30 to 89)	11 (5.8 to 17.3)	2 (0 to 2)	0.3 (0.1 to 0.5)	327.2 (184.5 to 509.4)	33.1 (-10 to 90.2)	1331.9 (234.5 to 3640.7)	265.3 (-13.9 to 908.3)
		Male	12 (7 to 19)	5.9 (3.4 to 9.5)	0 (0 to 0)	0.1 (0.1 to 0.2)	61 (46 to 80)	12.2 (9.3 to 16.2)	2 (2 to 3)	0.5 (0.4 to 0.6)	410.5 (258.6 to 657)	105.9 (49.9 to 199.9)	1481.4 (862.1 to 2607.1)	406.8 (211.5 to 777.1)
	Endocarditis	Both	148 (83 to 278)	16.2 (9.2 to 30.5)	3 (2 to 6)	0.5 (0.3 to 1.1)	134 (79 to 274)	10.8 (6.5 to 21.9)	5 (3 to 10)	0.4 (0.3 to 0.9)	-9.2 (-47.6 to 75.8)	-33.2 (-51.9 to -5.6)	57.8 (11.7 to 127.9)	-18 (-35.8 to 2.6)

Province	Cause	Sex	1990				2019				1990-2019 percent change (%)			
			DALYs		Death		DALYs		Death		DALYs		Death	
			Number	Rate	Number	Rate	Number	Rate	Number	Rate	Number	Rate	Number	Rate
		Female	72 (37 to 141)	14.9 (8.2 to 29.9)	1 (1 to 3)	0.5 (0.3 to 1.2)	48 (25 to 123)	8 (4.2 to 20.2)	2 (1 to 5)	0.4 (0.2 to 0.9)	-33 (-73.6 to 91.5)	-46.1 (-71.1 to -11.4)	38.8 (-28.1 to 139.2)	-29 (-54.8 to -1.6)
		Male	76 (38 to 167)	17.3 (9 to 35.5)	2 (1 to 3)	0.6 (0.3 to 1.1)	86 (51 to 169)	13.5 (8 to 26.6)	3 (2 to 6)	0.5 (0.3 to 1.1)	13.5 (-32.9 to 87.1)	-22 (-47.4 to 16.9)	73.3 (17 to 159.4)	-7.6 (-33 to 29.8)
	Other cardiovascular and circulatory diseases	Both	10479 (7244 to 13667)	936.3 (658.1 to 1207.1)	171 (115 to 225)	27 (18 to 38.9)	5481 (4432 to 6265)	468.3 (372 to 531.9)	189 (139 to 215)	18.5 (13.6 to 21)	-47.7 (-61.7 to -28.8)	-50 (-62.9 to -35.2)	10.7 (-22.1 to 48.2)	-31.6 (-56.7 to -10)
		Female	4796 (3387 to 6656)	861.8 (664.1 to 1141.6)	76 (56 to 102)	24.3 (18.6 to 37.1)	2302 (1962 to 2689)	391.5 (336 to 454.5)	80 (67 to 94)	15.5 (12.9 to 18)	-52 (-66.7 to -30.2)	-54.6 (-67.7 to -37.9)	6.4 (-28 to 53.4)	-36.4 (-63.4 to -12)
		Male	5683 (2997 to 7980)	1004 (516.6 to 1355.8)	95 (46 to 133)	29.6 (13.3 to 43)	3180 (2046 to 3728)	547.5 (343.9 to 638.9)	108 (55 to 128)	21.6 (10.9 to 25.6)	-44 (-61.8 to -19.2)	-45.5 (-61.8 to -25.4)	14.1 (-23.9 to 60.5)	-26.7 (-52.8 to 2)
	Chronic respiratory diseases	Both	7020 (5702 to 8678)	1017.2 (850.7 to 1239.2)	139 (112 to 183)	36.3 (28.9 to 48)	8399 (7339 to 9718)	720.1 (630.4 to 829.5)	240 (207 to 290)	23.8 (20.6 to 29)	19.6 (0.7 to 41.4)	-29.2 (-42.2 to -17.1)	72.2 (31.1 to 117.8)	-34.3 (-51.2 to -17.1)
		Female	3154 (2400 to 4073)	938.1 (718 to 1205)	57 (40 to 80)	31.7 (22.1 to 45.9)	3548 (3022 to 4251)	597.8 (511 to 717.3)	91 (75 to 116)	17.5 (14.4 to 22.3)	12.5 (-9.8 to 48.5)	-36.3 (-49.3 to -17.8)	58.2 (14 to 137.3)	-44.8 (-62.7 to -19.2)
		Male	3865 (3053 to 4994)	1083.3 (861.1 to 1436.3)	82 (62 to 117)	40.6 (30.8 to 59.3)	4851 (4162 to 5713)	850.8 (731.3 to 1016.9)	149 (123 to 193)	30.7 (25.5 to 40.6)	25.5 (-2.1 to 55.1)	-21.5 (-41 to -2.9)	81.9 (22.1 to 149)	-24.2 (-50.1 to 2.6)
	Chronic obstructive pulmonary disease	Both	2194 (1786 to 2827)	392.3 (315 to 534.9)	54 (40 to 82)	15.1 (10.7 to 23.9)	4627 (4042 to 5486)	405.4 (355.3 to 488)	153 (129 to 202)	15.4 (12.9 to 20.5)	110.9 (72.9 to 156.7)	3.3 (-16.9 to 27.3)	183.4 (114.3 to 282.9)	2.4 (-23.3 to 40.3)
		Female	954 (694 to 1354)	345.3 (246.8 to 523.4)	21 (11 to 37)	12.2 (6.9 to 23.3)	1896 (1609 to 2375)	324.9 (274.9 to 410.6)	58 (45 to 83)	11.3 (8.8 to 16)	98.8 (53.9 to 184.1)	-5.9 (-28.6 to 32)	179 (89.5 to 439.2)	-7.9 (-40.5 to 73.2)
		Male	1240 (966 to 1676)	432.7 (338.4 to 593.9)	33 (24 to 51)	17.8 (12.5 to 27.5)	2731 (2346 to 3244)	491.6 (422.2 to 594.6)	96 (78 to 128)	20 (16.3 to 26.9)	120.3 (63 to 183.5)	13.6 (-18 to 48.9)	186.2 (91.8 to 325.4)	12.2 (-24 to 67)
	Pneumoconiosis	Both	15 (10 to 22)	2.5 (1.6 to 3.4)	0 (0 to 1)	0.1 (0.1 to 0.1)	50 (40 to 62)	4.1 (3.3 to 5)	1 (1 to 2)	0.1 (0.1 to 0.2)	224.9 (113.7 to 411.2)	66.2 (11.2 to 159.4)	250.1 (119.9 to 532.8)	54.3 (-2.6 to 177.9)
		Female	4 (3 to 6)	1.3 (0.9 to 1.7)	0 (0 to 0)	0 (0 to 0)	24 (17 to 33)	3.7 (2.7 to 5)	0 (0 to 0)	0.1 (0 to 0.1)	460.5 (311.8 to 675.7)	181.6 (106.6 to 274.1)	430.3 (209.7 to 874.7)	106.3 (13.9 to 263.5)
		Male	11 (6 to 17)	3.5 (2 to 5.3)	0 (0 to 1)	0.1 (0.1 to 0.2)	26 (19 to 34)	4.6 (3.5 to 6.1)	1 (1 to 1)	0.2 (0.2 to 0.3)	133.7 (37.6 to 347.6)	33.5 (-19.7 to 152.3)	217.5 (83 to 550)	53.4 (-11.4 to 211.3)
	Asthma	Both	4604 (3573 to 5913)	600.2 (468.1 to 753.4)	83 (63 to 110)	20.7 (14.7 to 29.3)	3195 (2534 to 4042)	269.5 (215.5 to 338.9)	75 (62 to 89)	7.3 (6 to 8.6)	-30.6 (-42 to -17.6)	-55.1 (-65.1 to -45)	-9.2 (-34.6 to 21.9)	-64.6 (-76.4 to -50.9)
		Female	2089 (1523 to 2693)	568.8 (403.1 to 753.3)	36 (23 to 51)	19 (10.7 to 30.9)	1393 (1056 to 1803)	232.3 (175.4 to 303.4)	29 (20 to 37)	5.5 (3.7 to 7)	-33.3 (-48.5 to -15.5)	-59.2 (-70.3 to -46.1)	-17.7 (-46.5 to 25.3)	-70.9 (-83.6 to -54.2)
		Male	2515 (1909 to 3277)	625.2 (479.8 to 835)	47 (34 to 67)	22.1 (15.9 to 32.9)	1802 (1418 to 2275)	308.7 (245.7 to 388.5)	46 (36 to 59)	9.2 (7.4 to 12.1)	-28.3 (-43.4 to -10.8)	-50.6 (-63.8 to -35.8)	-2.7 (-38.8 to 44.1)	-58.2 (-74.7 to -38)
	Interstitial lung disease and pulmonary sarcoidosis	Both	61 (45 to 80)	10 (7.2 to 13.8)	1 (1 to 2)	0.4 (0.2 to 0.6)	163 (126 to 199)	13.5 (10.5 to 16.4)	5 (4 to 7)	0.5 (0.4 to 0.7)	167.9 (97.1 to 262)	34.2 (-0.8 to 91.3)	282.2 (158.1 to 528.8)	43.7 (-4.4 to 151.1)
		Female	27 (18 to 37)	9.3 (6.2 to 14)	1 (0 to 1)	0.3 (0.2 to 0.6)	65 (48 to 85)	10.4 (7.6 to 13.6)	2 (1 to 3)	0.3 (0.2 to 0.5)	143.9 (84.2 to 234.1)	11.4 (-18.3 to 65.9)	219.4 (106.4 to 491.7)	5.4 (-36.1 to 124.6)
		Male	34 (24 to 50)	10.7 (7.3 to 16.2)	1 (0 to 1)	0.4 (0.2 to 0.7)	98 (69 to 126)	16.8 (11.9 to 21.4)	4 (2 to 5)	0.7 (0.5 to 1)	186.7 (79.1 to 335.5)	56.7 (-1.5 to 146.7)	325.4 (148.5 to 686.3)	78.9 (4.9 to 249.5)
	Other chronic respiratory diseases	Both	146 (101 to 243)	12.3 (9.1 to 18.3)	1 (1 to 2)	0.1 (0.1 to 0.2)	363 (243 to 469)	27.7 (18.1 to 36.1)	5 (1 to 8)	0.4 (0.1 to 0.7)	148.9 (13.6 to 286.2)	125.8 (12.5 to 234.2)	409 (-28.9 to 1263.6)	244.6 (-36.8 to 702.7)

Province	Cause	Sex	1990				2019				1990-2019 percent change (%)			
			DALYs		Death		DALYs		Death		DALYs		Death	
			Number	Rate	Number	Rate	Number	Rate	Number	Rate	Number	Rate	Number	Rate
		Female	80 (53 to 138)	13.4 (9.6 to 20.2)	0 (0 to 1)	0.1 (0.1 to 0.2)	170 (120 to 215)	26.5 (18.4 to 33.9)	2 (0 to 3)	0.3 (0.1 to 0.6)	111.6 (3.7 to 230.5)	98.8 (8.9 to 194.8)	279.4 (-49.9 to 1017.3)	154.8 (-53.1 to 552.8)
		Male	66 (44 to 113)	11.2 (8.2 to 17)	0 (0 to 1)	0.1 (0.1 to 0.2)	193 (116 to 266)	29.1 (16.7 to 40.3)	3 (1 to 6)	0.6 (0.1 to 1)	194.6 (24.1 to 406.7)	159.4 (14 to 321.5)	534.7 (-10.2 to 1780.3)	336.4 (-24.8 to 1118.1)
	Digestive diseases	Both	7228 (5264 to 9591)	819.4 (655 to 986.5)	127 (97 to 155)	23.9 (19.1 to 29.9)	5843 (4863 to 7162)	466.5 (393.7 to 563.5)	145 (126 to 166)	13.7 (12 to 15.7)	-19.2 (-41.2 to 14.9)	-43.1 (-53.9 to -27.2)	14.6 (-11.2 to 61)	-42.4 (-54.3 to -23.9)
		Female	3435 (2391 to 4835)	785.9 (617.9 to 957.7)	55 (42 to 71)	22.2 (16.5 to 30.2)	2572 (2059 to 3234)	408.8 (333.2 to 507.9)	56 (48 to 65)	10.5 (8.9 to 12.3)	-25.1 (-49.4 to 9.3)	-48 (-59.5 to -33)	1.5 (-25.3 to 43.7)	-52.7 (-65.6 to -33.8)
		Male	3793 (2665 to 5237)	845.5 (658.9 to 1059.1)	71 (53 to 93)	25.1 (19.4 to 31.8)	3272 (2710 to 3944)	527.9 (442.6 to 624.2)	89 (74 to 105)	17.2 (14.4 to 20.2)	-13.7 (-40.2 to 29.6)	-37.6 (-52.4 to -14.9)	24.7 (-10.8 to 84.7)	-31.5 (-49.1 to -4.5)
	Cirrhosis and other chronic liver diseases	Both	2986 (2049 to 4103)	356.2 (268.6 to 437.3)	70 (52 to 87)	13.1 (10.1 to 16.5)	2197 (1844 to 2585)	180.8 (152.1 to 213.4)	84 (71 to 102)	7.7 (6.4 to 9.4)	-26.4 (-49.5 to 17.7)	-49.2 (-61.6 to -26.7)	20.1 (-9.5 to 72.6)	-41.4 (-55.3 to -17.9)
		Female	1422 (879 to 2163)	328 (233.1 to 430.2)	31 (22 to 41)	12.1 (8.7 to 16.6)	795 (653 to 949)	132.7 (109.9 to 158.6)	32 (26 to 39)	5.8 (4.8 to 7.1)	-44.1 (-66.2 to -3.2)	-59.5 (-70.9 to -40.5)	4.5 (-26.6 to 57)	-51.6 (-66.4 to -28.8)
		Male	1564 (1032 to 2200)	379.3 (273.1 to 500.6)	39 (28 to 52)	13.8 (10.1 to 18.4)	1403 (1108 to 1735)	230.7 (184.3 to 284.6)	52 (42 to 65)	9.6 (7.7 to 12.4)	-10.3 (-42.7 to 51)	-39.2 (-56.9 to -8.9)	32.3 (-6 to 98.9)	-30.6 (-49.8 to 2.5)
	Upper digestive system diseases	Both	1590 (1133 to 2274)	214.6 (158.5 to 295.2)	20 (16 to 26)	4.7 (3.4 to 6.5)	1934 (1241 to 3025)	143.4 (94.2 to 220.8)	19 (12 to 24)	1.9 (1.2 to 2.4)	21.6 (-7.6 to 46.1)	-33.2 (-45.8 to -22)	-4.7 (-36.6 to 34.6)	-59.3 (-71 to -42)
		Female	739 (495 to 1097)	206.1 (146.3 to 296.4)	8 (5 to 11)	4 (2.5 to 6.3)	968 (592 to 1566)	143.7 (89.7 to 228.9)	7 (5 to 9)	1.4 (0.9 to 1.9)	31 (-2.9 to 56.3)	-30.3 (-43.6 to -17.8)	-5.9 (-45.5 to 63.2)	-65.3 (-78.7 to -39.3)
		Male	851 (607 to 1199)	221.3 (161 to 303)	12 (9 to 17)	5.2 (3.6 to 7.3)	966 (632 to 1473)	143.8 (96.7 to 213.6)	12 (8 to 15)	2.4 (1.5 to 3.1)	13.5 (-19.6 to 43.9)	-35 (-50.7 to -21.1)	-3.9 (-40.3 to 47.6)	-53.2 (-68.7 to -29.8)
	Appendicitis	Both	329 (161 to 559)	28.4 (18 to 41)	5 (3 to 8)	0.8 (0.6 to 1.1)	104 (78 to 135)	8.3 (6.3 to 10.8)	2 (2 to 3)	0.2 (0.2 to 0.3)	-68.4 (-84 to -23.1)	-70.7 (-82.6 to -44.9)	-56.7 (-76.8 to -5.1)	-72.8 (-82.2 to -57.6)
		Female	138 (60 to 288)	24.4 (14.3 to 41.9)	2 (1 to 4)	0.7 (0.5 to 1.2)	43 (31 to 57)	7 (5.1 to 9.3)	1 (1 to 1)	0.2 (0.1 to 0.3)	-69.1 (-86.4 to -19.4)	-71.3 (-85.2 to -43.4)	-60.5 (-83.7 to -7.1)	-76.8 (-88.4 to -58.2)
		Male	191 (83 to 336)	32.1 (17.5 to 49)	3 (2 to 5)	0.9 (0.5 to 1.3)	61 (42 to 83)	9.6 (6.6 to 13)	1 (1 to 2)	0.3 (0.2 to 0.4)	-68 (-85.6 to -11.7)	-70 (-84 to -34.4)	-54.1 (-79.9 to 16.1)	-69.2 (-82.8 to -37.1)
	Paralytic ileus and intestinal obstruction	Both	1410 (710 to 2554)	97.4 (57.7 to 162.3)	19 (11 to 31)	2.2 (1.6 to 3)	290 (206 to 380)	27.5 (19.6 to 36.2)	11 (7 to 14)	1.1 (0.7 to 1.4)	-79.4 (-90.6 to -54.2)	-71.8 (-86 to -46.1)	-42.2 (-71.9 to 8.2)	-49.4 (-70 to -23.1)
		Female	684 (296 to 1426)	97.8 (51.5 to 185.9)	9 (5 to 18)	2.2 (1.4 to 3.3)	117 (86 to 162)	22.3 (16.4 to 31.3)	4 (3 to 6)	0.8 (0.6 to 1.1)	-83 (-92.5 to -56.8)	-77.2 (-89 to -50.6)	-54.1 (-78.2 to -2.3)	-63.2 (-78.4 to -33.9)
		Male	726 (305 to 1594)	97 (48.5 to 196.5)	10 (5 to 19)	2.1 (1.3 to 3.3)	174 (109 to 243)	33 (20.7 to 46.3)	7 (4 to 9)	1.4 (0.8 to 1.9)	-76 (-91.3 to -34.4)	-66 (-86.4 to -21.4)	-31 (-74.1 to 61.9)	-32.9 (-68.3 to 12.9)
	Inguinal, femoral, and abdominal hernia	Both	224 (128 to 428)	20.2 (13.2 to 31.6)	1 (1 to 3)	0.2 (0.2 to 0.4)	218 (149 to 304)	17.5 (12.1 to 24)	2 (1 to 2)	0.2 (0.1 to 0.2)	-2.7 (-53.9 to 48.2)	-13.3 (-49.4 to 14.4)	38.9 (-70 to 295.7)	-26 (-66.7 to 38.4)
		Female	89 (44 to 222)	15 (9 to 30.6)	1 (0 to 2)	0.2 (0.1 to 0.3)	77 (49 to 111)	12.7 (8 to 18.3)	1 (0 to 1)	0.1 (0 to 0.2)	-12.7 (-72 to 63.4)	-15.1 (-66 to 34.2)	48.2 (-85.1 to 465.2)	-19.8 (-79.5 to 83.4)
		Male	135 (79 to 240)	24.9 (16.1 to 37.1)	1 (0 to 2)	0.3 (0.2 to 0.6)	141 (95 to 199)	22.3 (15.4 to 31.2)	1 (0 to 1)	0.2 (0.1 to 0.3)	3.9 (-41.5 to 53.3)	-10.4 (-38.2 to 17.1)	32.5 (-60.9 to 240.1)	-29.1 (-66.4 to 40)
	Inflammatory bowel disease	Both	64 (46 to 91)	9.4 (7 to 12.4)	1 (1 to 1)	0.2 (0.1 to 0.2)	132 (90 to 178)	9.7 (6.5 to 13)	2 (1 to 2)	0.2 (0.1 to 0.2)	105.7 (25.4 to 163.6)	3.6 (-24.3 to 30.9)	131 (-12.9 to 285.3)	0.8 (-49.9 to 67.8)
		Female	30 (21 to 45)	9 (6.4 to 12.5)	0 (0 to 0)	0.1 (0.1 to 0.3)	53 (37 to 76)	7.7 (5.4 to 10.9)	0 (0 to 0)	0.1 (0 to 0.1)	76.5 (20.7 to 134.2)	-14.6 (-35.3 to 11.4)	24.8 (-33.3 to 133.2)	-51.1 (-77.7 to -8.4)

Province	Cause	Sex	1990				2019				1990-2019 percent change (%)			
			DALYs		Death		DALYs		Death		DALYs		Death	
			Number	Rate	Number	Rate	Number	Rate	Number	Rate	Number	Rate	Number	Rate
		Male	34 (25 to 47)	9.6 (7.1 to 12.9)	0 (0 to 1)	0.2 (0.1 to 0.3)	78 (47 to 107)	11.8 (6.9 to 16)	1 (0 to 2)	0.3 (0.1 to 0.4)	131.9 (23.4 to 223.2)	22.2 (-23 to 67.8)	196 (-13.5 to 443.1)	42.3 (-48.8 to 157.6)
	Vascular intestinal disorders	Both	45 (33 to 59)	10.5 (7.2 to 14.8)	2 (1 to 3)	0.6 (0.4 to 0.9)	90 (60 to 110)	8.3 (5.5 to 10.1)	5 (3 to 6)	0.5 (0.3 to 0.6)	99.5 (46.9 to 182.1)	-20.8 (-43.7 to 12.8)	150.1 (77 to 262.9)	-18.7 (-45 to 19.8)
		Female	20 (13 to 29)	11.4 (6.2 to 18.1)	1 (1 to 2)	0.7 (0.3 to 1.2)	39 (26 to 49)	7.2 (4.8 to 9)	2 (1 to 3)	0.4 (0.3 to 0.6)	94.4 (29.8 to 207.3)	-36.7 (-60.4 to 3.5)	128.6 (40.1 to 283.8)	-37.3 (-63.4 to 11.7)
		Male	25 (17 to 35)	9.7 (6.8 to 13.7)	1 (1 to 1)	0.5 (0.4 to 0.8)	50 (31 to 64)	9.5 (5.8 to 12.2)	3 (2 to 3)	0.6 (0.3 to 0.7)	103.7 (34.5 to 216.3)	-1.8 (-33.5 to 48.4)	171.7 (81 to 327.3)	7.5 (-28.6 to 64)
	Gallbladder and biliary diseases	Both	329 (224 to 473)	49 (33.9 to 69.2)	3 (2 to 5)	0.8 (0.6 to 1.4)	537 (370 to 766)	42 (29.7 to 58.5)	8 (6 to 12)	0.8 (0.6 to 1.2)	63.4 (31.7 to 94.3)	-14.3 (-29.4 to -0.3)	162.3 (65.8 to 297.2)	-3.5 (-36.3 to 43.3)
		Female	207 (133 to 302)	63.1 (42 to 89)	1 (1 to 3)	0.8 (0.4 to 1.5)	345 (224 to 511)	52.3 (34.9 to 75.7)	3 (2 to 6)	0.6 (0.4 to 1.1)	66.3 (33 to 100.8)	-17.1 (-30.8 to -2.2)	138.5 (46.9 to 304.6)	-21.5 (-48.2 to 25.2)
		Male	121 (84 to 169)	35.7 (25.5 to 50.7)	2 (1 to 3)	0.8 (0.5 to 1.5)	192 (140 to 260)	32.2 (23.9 to 43)	5 (3 to 7)	1 (0.7 to 1.4)	58.3 (18.4 to 107.8)	-9.9 (-32.3 to 15.1)	181.5 (62.3 to 397.8)	16 (-32.7 to 100.5)
	Pancreatitis	Both	96 (69 to 144)	16.1 (11.1 to 26.1)	3 (2 to 5)	0.7 (0.4 to 1.2)	127 (96 to 204)	10.4 (8 to 16.8)	5 (4 to 8)	0.5 (0.3 to 0.7)	32.1 (-6.3 to 79.4)	-35.4 (-54.9 to -10.3)	73.1 (14.9 to 148.9)	-32.3 (-56.5 to 2.6)
		Female	37 (25 to 62)	13.7 (8.9 to 24.3)	1 (1 to 2)	0.7 (0.4 to 1.3)	48 (36 to 75)	8.1 (6.1 to 12.8)	2 (1 to 3)	0.4 (0.3 to 0.6)	28.5 (-16.8 to 84.5)	-40.7 (-60.1 to -12)	81.4 (12.1 to 186.1)	-39.2 (-66.3 to 6.8)
		Male	59 (39 to 93)	18.3 (11.9 to 28.8)	2 (1 to 3)	0.7 (0.4 to 1.1)	79 (56 to 135)	12.8 (9.1 to 21.4)	3 (2 to 5)	0.5 (0.4 to 0.9)	34.3 (-15.3 to 100)	-30.2 (-55.9 to 3.9)	67.4 (-0.5 to 161.4)	-23.4 (-54.8 to 16.9)
	Other digestive diseases	Both	155 (89 to 289)	17.6 (12.5 to 24.6)	3 (2 to 5)	0.7 (0.5 to 1)	215 (124 to 265)	18.5 (10.9 to 22.8)	8 (4 to 10)	0.8 (0.4 to 1)	38.8 (-45.7 to 168.3)	5.2 (-45.2 to 63.7)	169.9 (27.2 to 361.3)	18.1 (-32.7 to 77.7)
		Female	68 (39 to 150)	17.4 (11 to 26.2)	1 (1 to 2)	0.7 (0.4 to 1.4)	87 (51 to 113)	14.9 (8.9 to 19.5)	3 (2 to 4)	0.6 (0.3 to 0.8)	27.3 (-56.9 to 149.7)	-14.4 (-54.7 to 50.4)	130.8 (3.1 to 354.3)	-14.4 (-55.4 to 61.2)
		Male	86 (43 to 168)	17.7 (11.3 to 26.7)	2 (1 to 3)	0.6 (0.4 to 0.9)	128 (69 to 163)	22.3 (12.1 to 28.3)	5 (2 to 6)	1 (0.5 to 1.3)	47.8 (-42.5 to 222.8)	26.2 (-41 to 124)	204 (25.2 to 493)	62.4 (-19 to 170.2)
	Neurological disorders	Both	13371 (7278 to 22081)	1531 (891.7 to 2460.9)	111 (61 to 217)	33 (13.8 to 78)	17446 (9301 to 29545)	1410.4 (789.1 to 2320.6)	308 (136 to 685)	33.2 (14 to 74.9)	30.5 (-0.9 to 66)	-7.9 (-22.2 to 6.9)	177.8 (84.3 to 286.9)	0.7 (-12.3 to 40.2)
		Female	6987 (3538 to 12209)	1651.2 (901.5 to 2802.8)	53 (26 to 107)	32.1 (12.3 to 78.2)	9671 (4736 to 16969)	1545.9 (795.3 to 2663.5)	156 (61 to 358)	31.8 (12 to 73.2)	38.4 (7.2 to 73.1)	-6.4 (-21.5 to 8.3)	193.6 (83.5 to 350.9)	-0.8 (-17.8 to 44.4)
		Male	6385 (3703 to 10100)	1413.3 (863.5 to 2208.3)	58 (32 to 112)	33.8 (14.5 to 80.1)	7774 (4511 to 12582)	1283.9 (778 to 2055.9)	152 (74 to 331)	34.6 (15.8 to 76.8)	21.8 (-9.8 to 59.4)	-9.2 (-25.4 to 9.3)	163.3 (74.1 to 275.6)	2.2 (-15.9 to 40.6)
	Alzheimer's disease and other dementias	Both	1068 (458 to 2515)	387.9 (170.1 to 899)	59 (14 to 165)	25.5 (6.1 to 71.1)	3712 (1618 to 8196)	391 (171.9 to 862.9)	230 (56 to 614)	25.7 (6.3 to 67.8)	247.7 (208.5 to 335.6)	0.8 (-9.2 to 26.4)	288.8 (237.1 to 458.1)	0.9 (-11.9 to 45)
		Female	534 (229 to 1238)	398 (175.2 to 906.3)	31 (8 to 85)	26 (6.2 to 71.7)	2020 (893 to 4439)	404.5 (179.5 to 892.3)	127 (31 to 332)	26.5 (6.5 to 68.5)	278.2 (222.3 to 394.9)	1.6 (-11.5 to 33.3)	305.8 (235.5 to 523.5)	2 (-15 to 56)
		Male	534 (231 to 1264)	378.5 (165.6 to 885.4)	28 (6 to 80)	25 (5.8 to 70.9)	1692 (740 to 3805)	376.3 (164 to 844.1)	103 (25 to 286)	24.8 (6 to 68)	217.2 (166.2 to 298)	-0.6 (-14.5 to 22.5)	269.7 (197.6 to 422.4)	-0.6 (-17.8 to 39.3)
	Parkinson's disease	Both	244 (201 to 292)	70.5 (58.8 to 84.4)	11 (9 to 14)	4.1 (3.3 to 5.1)	880 (742 to 987)	86.8 (73.6 to 97.6)	52 (45 to 58)	5.4 (4.6 to 6.1)	260.8 (192.3 to 355.2)	23.1 (-2.2 to 53.3)	368.4 (268.9 to 507.7)	32.5 (2.5 to 68.3)
		Female	91 (73 to 115)	54.8 (44 to 70.8)	4 (3 to 6)	3 (2.3 to 4.3)	327 (280 to 373)	62 (52.8 to 70.4)	19 (16 to 22)	3.7 (3.1 to 4.5)	259.6 (177 to 367.5)	13.1 (-14.5 to 45.6)	361.1 (242.8 to 519.8)	22.1 (-10.5 to 62.5)

Province	Cause	Sex	1990				2019				1990-2019 percent change (%)			
			DALYs		Death		DALYs		Death		DALYs		Death	
			Number	Rate	Number	Rate	Number	Rate	Number	Rate	Number	Rate	Number	Rate
		Male	153 (117 to 193)	85.8 (66.2 to 107.3)	7 (5 to 9)	5.2 (3.9 to 6.7)	553 (429 to 635)	113.4 (87.7 to 130.5)	33 (26 to 39)	7.3 (5.7 to 8.4)	261.4 (170.7 to 396.6)	32.1 (-1.2 to 81.5)	372.6 (241.5 to 585)	39.8 (1.6 to 99.5)
	Idiopathic epilepsy	Both	4314 (2338 to 6665)	291.5 (165.8 to 449.4)	34 (17 to 50)	2.5 (1.5 to 3.4)	1844 (849 to 3117)	147.8 (67.5 to 250.8)	13 (10 to 15)	1 (0.8 to 1.2)	-57.3 (-81.8 to -11.7)	-49.3 (-78.8 to 3.6)	-63.2 (-75.5 to -25.1)	-60.5 (-71.9 to -32.4)
		Female	1918 (929 to 3045)	266.3 (139.3 to 421.6)	15 (5 to 24)	2.3 (1.2 to 3.3)	784 (351 to 1351)	129.8 (57.2 to 224.2)	5 (4 to 6)	0.8 (0.6 to 0.9)	-59.1 (-83.6 to -4.3)	-51.3 (-80.4 to 4.2)	-68.3 (-81.3 to -17.6)	-66.3 (-78.1 to -30.8)
		Male	2397 (1259 to 3851)	315.4 (174.3 to 497.6)	19 (8 to 30)	2.8 (1.5 to 4.1)	1060 (500 to 1778)	165.1 (77.2 to 281.6)	8 (6 to 9)	1.2 (0.9 to 1.5)	-55.8 (-81 to -8.8)	-47.6 (-78 to 7.3)	-59.2 (-75.1 to -9.4)	-55.6 (-70.5 to -16.9)
	Multiple sclerosis	Both	235 (170 to 345)	32.4 (24 to 45)	3 (2 to 5)	0.5 (0.3 to 0.8)	451 (358 to 590)	31.4 (25.1 to 40.7)	7 (5 to 10)	0.5 (0.4 to 0.8)	91.7 (27.8 to 157.5)	-3.1 (-24.3 to 25.5)	124.3 (43.9 to 258.9)	6.4 (-26.5 to 67.2)
		Female	137 (95 to 222)	37.8 (27.1 to 55.9)	2 (1 to 3)	0.5 (0.3 to 0.9)	253 (200 to 317)	35.4 (27.8 to 44.1)	3 (3 to 5)	0.5 (0.4 to 0.7)	85 (9.7 to 162.3)	-6.5 (-37.5 to 27.8)	105.5 (0.7 to 288.9)	-5.7 (-47.3 to 80.7)
		Male	98 (67 to 168)	27.1 (18.3 to 47.2)	1 (1 to 3)	0.5 (0.3 to 1)	198 (148 to 294)	27.6 (20.9 to 40.3)	4 (3 to 6)	0.6 (0.4 to 0.9)	101 (29.1 to 186)	2 (-33.1 to 39.9)	145.4 (32.4 to 312.8)	22.1 (-32.9 to 101.9)
	Motor neuron disease	Both	18 (14 to 22)	2.1 (1.6 to 2.7)	0 (0 to 0)	0 (0 to 0.1)	28 (23 to 34)	2.2 (1.8 to 2.6)	1 (0 to 1)	0 (0 to 0.1)	59.7 (19.3 to 112.4)	3.7 (-24.3 to 42.6)	97.2 (28 to 216.4)	9.6 (-29.9 to 76.4)
		Female	10 (8 to 13)	2.2 (1.6 to 2.9)	0 (0 to 0)	0 (0 to 0.1)	15 (12 to 18)	2.3 (1.9 to 2.8)	0 (0 to 0)	0.1 (0 to 0.1)	52.3 (10.3 to 104.6)	7.3 (-24.5 to 48.6)	107 (27.7 to 222.7)	19.5 (-29 to 97.1)
		Male	8 (6 to 11)	2 (1.4 to 2.7)	0 (0 to 0)	0 (0 to 0.1)	13 (10 to 17)	2 (1.6 to 2.5)	0 (0 to 0)	0 (0 to 0.1)	68.8 (14.7 to 145.1)	1.6 (-33.2 to 53.1)	87.4 (3.4 to 246.4)	3.7 (-41.2 to 92.6)
	Headache disorders	Both	6906 (1460 to 15213)	703.3 (171 to 1512)	--	--	9947 (2539 to 21031)	703.3 (172.5 to 1498.6)	--	--	44 (30.2 to 79.5)	0 (-5.3 to 6.3)	--	--
		Female	4061 (793 to 9103)	855.2 (191.4 to 1879.2)	--	--	6029 (1365 to 13015)	871.1 (193.3 to 1883.2)	--	--	48.5 (34.1 to 78.1)	1.8 (-5 to 8.5)	--	--
		Male	2845 (639 to 6256)	554.9 (150.8 to 1177.3)	--	--	3918 (1145 to 8117)	544.4 (153.9 to 1138.8)	--	--	37.7 (20.9 to 81.6)	-1.9 (-8.7 to 7.8)	--	--
	Other neurological disorders	Both	587 (284 to 1022)	43.4 (24 to 71.3)	3 (2 to 4)	0.3 (0.2 to 0.4)	584 (342 to 898)	48 (27.5 to 75.4)	6 (5 to 7)	0.5 (0.4 to 0.6)	-0.4 (-52.8 to 111.6)	10.7 (-45 to 115)	84.3 (26.2 to 178.2)	46.7 (4.8 to 120.1)
		Female	236 (106 to 425)	36.9 (19.7 to 61.6)	1 (1 to 1)	0.2 (0.2 to 0.3)	243 (139 to 381)	40.8 (22.5 to 64.4)	2 (2 to 2)	0.3 (0.3 to 0.4)	2.8 (-54.3 to 148.4)	10.7 (-48.7 to 128.5)	93.9 (39.1 to 175.8)	29.5 (-9.8 to 100.1)
		Male	350 (176 to 593)	49.6 (27.5 to 79.7)	2 (1 to 3)	0.4 (0.3 to 0.6)	341 (205 to 519)	55 (32.9 to 85.3)	4 (3 to 5)	0.7 (0.6 to 0.8)	-2.6 (-51.3 to 95.9)	11 (-42.6 to 119.3)	79.8 (13.8 to 205)	59.8 (5.5 to 155)
	Mental disorders	Both	23059 (16972 to 30417)	2296.7 (1701 to 3033)	0 (0 to 0)	0 (0 to 0)	32518 (23898 to 43184)	2338.7 (1728.2 to 3101.7)	0 (0 to 0)	0 (0 to 0)	41 (33.5 to 48.3)	1.8 (-1 to 4.5)	159.9 (22.2 to 693.5)	46.2 (-30.9 to 399.9)
		Female	12538 (9189 to 16600)	2598.1 (1920.4 to 3435.8)	0 (0 to 0)	0 (0 to 0)	18013 (13140 to 23801)	2646.3 (1936.5 to 3498.1)	0 (0 to 0)	0 (0 to 0)	43.7 (35.8 to 52.3)	1.9 (-1.7 to 5.6)	114.8 (-6.5 to 759.8)	23.9 (-45.7 to 524.1)
		Male	10521 (7661 to 13978)	2005.8 (1486.5 to 2647.3)	0 (0 to 0)	0 (0 to 0)	14505 (10658 to 19233)	2046.1 (1505.3 to 2719.8)	0 (0 to 0)	0 (0 to 0)	37.9 (29.4 to 46.6)	2 (-1.3 to 5.7)	420.1 (41.7 to 1798)	223.2 (-8 to 1137.6)
	Schizophrenia	Both	1261 (906 to 1622)	160.4 (116.4 to 204.9)	--	--	2456 (1784 to 3119)	161.4 (118.4 to 203.8)	--	--	94.8 (75.4 to 115.2)	0.7 (-7.4 to 9.1)	--	--
		Female	588 (419 to 764)	151.8 (109 to 194.3)	--	--	1132 (828 to 1454)	152.8 (111.1 to 194.8)	--	--	92.6 (68.7 to 120.8)	0.7 (-10.2 to 14.5)	--	--

Province	Cause	Sex	1990				2019				1990-2019 percent change (%)			
			DALYs		Death		DALYs		Death		DALYs		Death	
			Number	Rate	Number	Rate	Number	Rate	Number	Rate	Number	Rate	Number	Rate
		Male	673 (485 to 866)	168.9 (120.5 to 218)	--	--	1324 (941 to 1708)	169.5 (122.4 to 218.3)	--	--	96.6 (71.5 to 123.1)	0.4 (-10.9 to 12.3)	--	--
	Depressive disorders	Both	8010 (5347 to 11114)	891.9 (609.2 to 1231.8)	--	--	13413 (9093 to 18729)	942.2 (641 to 1307.8)	--	--	67.5 (53.4 to 82.7)	5.6 (0.9 to 10.3)	--	--
		Female	4941 (3308 to 6860)	1113.8 (754.9 to 1538.8)	--	--	8012 (5452 to 11201)	1154.8 (786.6 to 1602.2)	--	--	62.1 (46.4 to 78.4)	3.7 (-1.9 to 9.9)	--	--
		Male	3068 (2054 to 4288)	678.7 (463.8 to 938.2)	--	--	5402 (3652 to 7566)	739.3 (503.7 to 1031.2)	--	--	76 (59.3 to 93.3)	8.9 (2.9 to 15.2)	--	--
	Bipolar disorder	Both	1603 (967 to 2453)	173.4 (105.3 to 262.1)	--	--	2498 (1533 to 3823)	175.4 (108.1 to 268.1)	--	--	55.8 (40.1 to 72.9)	1.1 (-5.8 to 8.4)	--	--
		Female	861 (515 to 1335)	187 (113.9 to 284.2)	--	--	1305 (803 to 1979)	188.9 (116.3 to 289.9)	--	--	51.5 (32.8 to 71.9)	1 (-8.8 to 11.3)	--	--
		Male	742 (443 to 1118)	160.3 (96.9 to 241.9)	--	--	1193 (728 to 1810)	162.8 (99.7 to 246.5)	--	--	60.8 (40 to 83.7)	1.5 (-8.6 to 12.5)	--	--
	Anxiety disorders	Both	7187 (4939 to 9823)	670.3 (463.8 to 916.3)	--	--	9410 (6595 to 12875)	693.9 (485.2 to 952.8)	--	--	30.9 (18.6 to 44.4)	3.5 (-2 to 9.7)	--	--
		Female	4230 (2942 to 5817)	822.4 (570.5 to 1123.7)	--	--	5671 (3949 to 7735)	853.1 (598.5 to 1160)	--	--	34.1 (20.4 to 49.5)	3.7 (-3.6 to 12)	--	--
		Male	2957 (2015 to 4111)	523.6 (361.9 to 723.3)	--	--	3739 (2582 to 5192)	542.9 (377.6 to 763.6)	--	--	26.5 (11.6 to 43.2)	3.7 (-5.4 to 12.9)	--	--
	Eating disorders	Both	460 (283 to 697)	40.6 (24.8 to 60.9)	0 (0 to 0)	0 (0 to 0)	744 (460 to 1111)	51.7 (32 to 77)	0 (0 to 0)	0 (0 to 0)	61.6 (38.3 to 90.9)	27.3 (13.2 to 40.9)	159.9 (22.2 to 693.5)	46.2 (-30.9 to 399.9)
		Female	283 (171 to 430)	50.5 (30.9 to 75.9)	0 (0 to 0)	0 (0 to 0)	446 (272 to 664)	65 (39.3 to 96.8)	0 (0 to 0)	0 (0 to 0)	57.4 (32.7 to 88.7)	28.7 (13 to 47.9)	114.8 (-6.5 to 759.8)	23.9 (-45.7 to 524.1)
		Male	177 (108 to 275)	30.9 (18.8 to 48.1)	0 (0 to 0)	0 (0 to 0)	298 (179 to 458)	39.5 (24.1 to 60.7)	0 (0 to 0)	0 (0 to 0)	68.3 (34.7 to 111.6)	27.8 (6 to 54)	420.1 (41.7 to 1798)	223.2 (-8 to 1137.6)
	Autism spectrum disorders	Both	567 (369 to 825)	44.6 (29 to 65)	--	--	581 (380 to 848)	45.2 (29.3 to 66.1)	--	--	2.5 (-8.2 to 14.8)	1.3 (-8.3 to 13.1)	--	--
		Female	138 (89 to 204)	22.3 (14.6 to 32.6)	--	--	140 (90 to 206)	22.4 (14.5 to 33)	--	--	1.1 (-13.4 to 18.7)	0.7 (-14.2 to 17.4)	--	--
		Male	428 (279 to 629)	66 (43.3 to 96.9)	--	--	441 (290 to 646)	66.8 (43.6 to 97.8)	--	--	3 (-10.4 to 17.8)	1.2 (-11.2 to 16)	--	--
	Attention-deficit/hyperactivity disorder	Both	476 (265 to 830)	32.5 (18.2 to 55.3)	--	--	421 (237 to 710)	33.7 (18.9 to 57.1)	--	--	-11.7 (-24.2 to 5)	3.7 (-9.9 to 17.1)	--	--
		Female	142 (78 to 246)	20.1 (11.1 to 34.9)	--	--	126 (70 to 210)	20.8 (11.7 to 35)	--	--	-11.7 (-27.2 to 9.5)	3.7 (-13.7 to 24.4)	--	--
		Male	334 (183 to 571)	44.5 (24.7 to 75.6)	--	--	295 (166 to 500)	45.8 (25.6 to 77.7)	--	--	-11.7 (-27.5 to 8)	2.9 (-14.8 to 21.9)	--	--
	Conduct disorder	Both	1402 (800 to 2200)	75.9 (43.2 to 119)	--	--	767 (435 to 1222)	74.6 (42.2 to 118.5)	--	--	-45.3 (-50.2 to -39.8)	-1.8 (-10.8 to 8)	--	--
		Female	471 (250 to 759)	52.5 (27.8 to 84.9)	--	--	255 (135 to 424)	51 (27.1 to 84.9)	--	--	-45.9 (-53.9 to -36.6)	-2.8 (-17 to 14.1)	--	--
		Male	931 (538 to 1464)	97.8 (56.3 to 155.1)	--	--	512 (299 to 808)	96.7 (56.4 to 152.4)	--	--	-45 (-51.2 to -37.9)	-1.1 (-12.2 to 11.7)	--	--
		Both	1368 (760 to 2166)	106.5 (60 to 167)	--	--	774 (398 to 1291)	59.9 (30.8 to 100.2)	--	--	-43.4 (-52.2 to -37.3)	-43.7 (-53.1 to -37.5)	--	--

Province	Cause	Sex	1990				2019				1990-2019 percent change (%)			
			DALYs		Death		DALYs		Death		DALYs		Death	
			Number	Rate	Number	Rate	Number	Rate	Number	Rate	Number	Rate	Number	Rate
	Idiopathic developmental intellectual disability	Female	597 (331 to 948)	95.2 (52.9 to 150.2)	--	--	343 (175 to 569)	54.7 (28 to 90.8)	--	--	-42.5 (-51.5 to -35.8)	-42.5 (-51.6 to -35.7)	--	--
		Male	772 (435 to 1214)	117.3 (66.5 to 184.4)	--	--	431 (220 to 725)	64.8 (33.1 to 109.3)	--	--	-44.1 (-53.9 to -37.1)	-44.8 (-54.6 to -37.8)	--	--
	Other mental disorders	Both	724 (457 to 1102)	100.6 (64.1 to 153.9)	--	--	1454 (935 to 2192)	100.7 (65.3 to 151.4)	--	--	100.9 (85.2 to 118.2)	0.2 (-5.1 to 6)	--	--
		Female	287 (178 to 438)	82.5 (52.3 to 125.5)	--	--	585 (373 to 897)	82.7 (53 to 126.5)	--	--	103.6 (81.8 to 126.4)	0.2 (-9.2 to 8.7)	--	--
		Male	437 (279 to 665)	117.9 (76.5 to 180.1)	--	--	870 (558 to 1292)	118.2 (76.3 to 176)	--	--	99.1 (81.4 to 120.2)	0.3 (-6.7 to 7.7)	--	--
	Substance use disorders	Both	2121 (1636 to 2655)	236.1 (186.2 to 291.9)	14 (10 to 18)	2 (1.5 to 2.5)	4641 (3652 to 5692)	309.7 (244.1 to 379.3)	35 (29 to 44)	2.5 (2 to 3)	118.8 (90.1 to 160.3)	31.2 (14.6 to 54.3)	151.5 (83.5 to 282.8)	24.3 (-8.9 to 79.8)
		Female	743 (543 to 979)	165.1 (123.4 to 212.1)	4 (3 to 6)	1.3 (0.8 to 1.7)	1203 (898 to 1571)	167.4 (123.4 to 218.2)	6 (4 to 7)	0.8 (0.7 to 1)	62 (35.8 to 97.9)	1.4 (-14.7 to 20.7)	30.2 (-14.2 to 123)	-33 (-53.1 to 3.7)
		Male	1378 (1071 to 1737)	306.7 (241.3 to 387.4)	10 (7 to 13)	2.7 (1.9 to 3.7)	3437 (2715 to 4194)	443.2 (348.9 to 541.2)	30 (24 to 38)	4 (3.3 to 5)	149.4 (108.8 to 203.1)	44.5 (21.4 to 74.8)	206 (109.1 to 383.7)	50.1 (3.2 to 125.7)
	Alcohol use disorders	Both	411 (301 to 545)	48.8 (36.1 to 63.8)	3 (2 to 4)	0.4 (0.3 to 0.5)	626 (458 to 825)	41.8 (31 to 55.1)	4 (3 to 5)	0.3 (0.2 to 0.4)	52.2 (30.9 to 78.6)	-14.2 (-26.1 to -0.7)	60.5 (11.4 to 155.4)	-21.9 (-45.3 to 19.3)
		Female	117 (83 to 159)	26.6 (19.1 to 35.7)	1 (0 to 1)	0.1 (0.1 to 0.2)	173 (120 to 241)	24.2 (16.8 to 33.2)	1 (0 to 1)	0.1 (0.1 to 0.1)	48.2 (20.5 to 81.3)	-9.1 (-23.6 to 8.5)	6.3 (-25.1 to 69.7)	-43 (-58.8 to -17.6)
		Male	294 (211 to 392)	70.7 (52 to 93.6)	2 (1 to 3)	0.6 (0.4 to 0.9)	452 (337 to 588)	58.7 (44.1 to 76.8)	4 (3 to 5)	0.5 (0.4 to 0.7)	53.8 (25.6 to 88.5)	-17 (-31.5 to 1.5)	73.8 (14.2 to 190.9)	-15.1 (-44 to 40.5)
	Drug use disorders	Both	1710 (1295 to 2190)	187.3 (145.7 to 233.9)	12 (8 to 15)	1.6 (1.2 to 2)	4015 (3122 to 5012)	267.9 (208.8 to 333.6)	31 (25 to 39)	2.2 (1.8 to 2.6)	134.8 (99.7 to 181.2)	43 (23.5 to 69.7)	171.8 (93.6 to 309.4)	35.6 (-0.7 to 95.1)
		Female	626 (451 to 840)	138.5 (102 to 180.8)	4 (2 to 5)	1.1 (0.7 to 1.5)	1030 (751 to 1367)	143.2 (103.9 to 189.9)	5 (4 to 7)	0.8 (0.6 to 0.9)	64.6 (34.6 to 104.1)	3.4 (-15 to 25.1)	33.3 (-14.5 to 130.9)	-31.7 (-53.4 to 7.4)
		Male	1084 (827 to 1393)	236 (183.3 to 299)	8 (5 to 11)	2.1 (1.4 to 2.9)	2985 (2323 to 3677)	384.6 (299.3 to 473.4)	26 (21 to 34)	3.5 (2.8 to 4.4)	175.3 (126 to 242.8)	63 (34.1 to 99.9)	241.8 (129.2 to 446.3)	69.2 (14.7 to 159.2)
	Diabetes and kidney diseases	Both	8441 (6853 to 10091)	1200.2 (1013.5 to 1389.4)	161 (131 to 189)	34.8 (28.9 to 43)	17017 (14487 to 20162)	1458.5 (1247.9 to 1719)	446 (397 to 495)	43.7 (38.8 to 48.5)	101.6 (68.2 to 146.5)	21.5 (5.9 to 40.9)	177.6 (127.6 to 247.7)	25.5 (1.5 to 55.1)
		Female	4171 (3254 to 5138)	1224.1 (1010.1 to 1466.8)	76 (59 to 94)	34.1 (27.1 to 44.8)	8453 (7065 to 10199)	1424.5 (1198.9 to 1702.7)	212 (183 to 241)	40.2 (34.8 to 45.6)	102.6 (59.6 to 156.7)	16.4 (-3.6 to 39.4)	179.4 (115.8 to 263.7)	17.8 (-10.9 to 51.9)
		Male	4270 (3391 to 5364)	1176.8 (975.9 to 1416.4)	85 (65 to 106)	35.6 (27.4 to 44.8)	8564 (7214 to 10112)	1496.3 (1268.5 to 1750.6)	234 (202 to 268)	47.5 (41.2 to 54.4)	100.6 (58.6 to 155.4)	27.1 (5.3 to 56.3)	176.1 (110.9 to 273.9)	33.4 (1.6 to 81.4)
	Diabetes mellitus	Both	2659 (2101 to 3312)	468.3 (370.5 to 584.2)	39 (31 to 48)	8.9 (7.1 to 11.1)	9789 (7760 to 12426)	824.6 (659.5 to 1032.9)	164 (144 to 186)	15.8 (13.8 to 18.1)	268.2 (227 to 311.6)	76.1 (56.7 to 96.5)	324.8 (232.8 to 448.8)	78 (37.9 to 134)
		Female	1300 (1017 to 1634)	488 (376.6 to 616.5)	19 (14 to 26)	9.7 (7.1 to 13.6)	5075 (3965 to 6479)	841.3 (659 to 1065.1)	82 (69 to 97)	15.3 (12.9 to 18.3)	290.4 (230.2 to 355.3)	72.4 (44.8 to 100.4)	320.4 (196.9 to 500.7)	58.5 (9.5 to 126.1)
		Male	1359 (1051 to 1722)	450.5 (349.1 to 569.5)	19 (14 to 25)	8.2 (6.1 to 10.5)	4714 (3745 to 5955)	807.9 (647 to 1008.5)	82 (68 to 98)	16.3 (13.5 to 19.5)	246.9 (195.4 to 298.6)	79.3 (55.8 to 105.7)	329.3 (209.7 to 511.8)	99.7 (45.6 to 180.9)

Province	Cause	Sex	1990				2019				1990-2019 percent change (%)			
			DALYs		Death		DALYs		Death		DALYs		Death	
			Number	Rate	Number	Rate	Number	Rate	Number	Rate	Number	Rate	Number	Rate
	Chronic kidney disease	Both	5642 (4368 to 7033)	720.5 (601.5 to 837.8)	120 (97 to 142)	25.7 (21.2 to 32.5)	7172 (6346 to 8034)	629.3 (559.6 to 703.1)	280 (245 to 314)	27.8 (24.1 to 31.1)	27.1 (0.9 to 65.6)	-12.7 (-26.7 to 8.2)	134 (90.6 to 199.6)	8.2 (-16.4 to 34)
		Female	2798 (2013 to 3666)	724.5 (584.4 to 881.3)	55 (43 to 69)	24.2 (18.9 to 34.4)	3352 (2901 to 3872)	579 (500.9 to 668.5)	129 (109 to 147)	24.7 (20.7 to 28.2)	19.8 (-10.6 to 69.1)	-20.1 (-36.2 to 1.7)	133.8 (77.2 to 209.6)	2.2 (-30.1 to 34.7)
		Male	2844 (2063 to 3807)	715.3 (562.3 to 875.6)	65 (49 to 81)	27.2 (20.7 to 34.5)	3820 (3275 to 4418)	683.4 (587.3 to 786.9)	151 (128 to 177)	31 (26.4 to 35.9)	34.3 (-2.4 to 86.2)	-4.5 (-25.1 to 24)	134.2 (76.6 to 222.7)	14.3 (-14.7 to 54.8)
	Acute glomerulonephritis	Both	140 (87 to 223)	11.4 (7.9 to 15.9)	2 (2 to 3)	0.3 (0.2 to 0.4)	56 (24 to 70)	4.7 (2.2 to 5.9)	2 (1 to 3)	0.2 (0.1 to 0.2)	-60.4 (-84.7 to -26.7)	-59 (-79.7 to -33.4)	-15.5 (-62.4 to 37.1)	-40.4 (-66.4 to -8.7)
		Female	74 (34 to 134)	11.6 (6 to 18.8)	1 (1 to 2)	0.3 (0.2 to 0.5)	25 (11 to 34)	4.3 (1.9 to 5.8)	1 (0 to 1)	0.2 (0.1 to 0.2)	-65.7 (-85 to -15.4)	-63.2 (-81.5 to -17.7)	-20.7 (-60.6 to 83.4)	-43.2 (-70.4 to 21.1)
		Male	66 (32 to 132)	11 (5.8 to 17.4)	1 (1 to 2)	0.3 (0.2 to 0.5)	30 (9 to 43)	5.1 (1.7 to 7.1)	1 (0 to 2)	0.2 (0.1 to 0.3)	-54.4 (-90.6 to 30.4)	-54.2 (-85.9 to 11.9)	-10.3 (-78.3 to 114.3)	-36.8 (-76.4 to 42)
	Skin and subcutaneous diseases	Both	5051 (3362 to 7330)	413.2 (278.2 to 605.2)	1 (1 to 2)	0.2 (0.1 to 0.4)	5265 (3487 to 7793)	421.3 (278.8 to 623.3)	3 (2 to 4)	0.3 (0.2 to 0.4)	4.3 (-0.2 to 8.4)	2 (-1 to 4.8)	232.9 (82.1 to 434.1)	26.4 (-34.1 to 122.3)
		Female	2617 (1729 to 3766)	438 (291.7 to 642.8)	0 (0 to 1)	0.2 (0.1 to 0.5)	2721 (1812 to 4013)	445.3 (296.5 to 657.5)	1 (1 to 2)	0.2 (0.2 to 0.3)	4 (-1.3 to 8.9)	1.7 (-1.8 to 5.2)	244 (34.9 to 533)	16.9 (-59 to 154.4)
		Male	2434 (1639 to 3565)	389.4 (262.5 to 569.4)	0 (0 to 1)	0.2 (0.1 to 0.6)	2545 (1681 to 3779)	398.7 (263.2 to 591.2)	1 (1 to 2)	0.3 (0.2 to 0.5)	4.5 (-0.4 to 9.3)	2.4 (-1.6 to 6.2)	223.4 (103.8 to 467.1)	35.6 (-19.1 to 167.7)
	Dermatitis	Both	1153 (674 to 1837)	93.4 (56.9 to 143.7)	--	--	1209 (738 to 1851)	93.9 (57.2 to 145.2)	--	--	4.9 (-3.7 to 15.8)	0.6 (-4.2 to 5.5)	--	--
		Female	625 (358 to 999)	103.7 (62.3 to 161.1)	--	--	656 (393 to 1012)	104.5 (62.8 to 163.5)	--	--	4.9 (-5.9 to 17.4)	0.8 (-6.2 to 7.6)	--	--
		Male	527 (312 to 832)	83.5 (50.7 to 128.3)	--	--	553 (338 to 843)	83.9 (51.3 to 128.1)	--	--	4.9 (-5.6 to 17.5)	0.4 (-6.5 to 7.8)	--	--
	Psoriasis	Both	464 (323 to 619)	50.5 (35.3 to 67.1)	--	--	489 (346 to 652)	35.6 (25.3 to 47.5)	--	--	5.5 (-6.1 to 18)	-29.4 (-36.2 to -22.6)	--	--
		Female	239 (168 to 323)	52.4 (36.8 to 69.8)	--	--	248 (174 to 333)	37 (25.8 to 49.8)	--	--	3.8 (-10.2 to 19.6)	-29.4 (-38.2 to -19.7)	--	--
		Male	225 (154 to 303)	48.7 (33.9 to 64.9)	--	--	241 (170 to 325)	34.4 (24.2 to 46.6)	--	--	7.2 (-9.2 to 26.7)	-29.4 (-38.2 to -19.1)	--	--
	Bacterial skin diseases	Both	72 (45 to 121)	7.3 (5 to 11.5)	1 (0 to 1)	0.2 (0.1 to 0.3)	87 (61 to 135)	7.1 (5.1 to 10.8)	2 (1 to 3)	0.2 (0.1 to 0.3)	20 (-3.6 to 42.5)	-2.5 (-23.4 to 18.2)	158.1 (68.1 to 320.6)	2.4 (-33.8 to 92.3)
		Female	32 (20 to 53)	6.9 (4.4 to 11.2)	0 (0 to 1)	0.2 (0.1 to 0.3)	38 (26 to 58)	6.4 (4.4 to 9.8)	1 (1 to 1)	0.2 (0.1 to 0.2)	17.3 (-4.4 to 43.7)	-7.3 (-34 to 22.1)	166.9 (28.9 to 398.5)	-5 (-59 to 123.8)
		Male	40 (25 to 68)	7.8 (4.9 to 13)	0 (0 to 1)	0.2 (0.1 to 0.4)	49 (34 to 75)	7.9 (5.5 to 12.1)	1 (1 to 2)	0.2 (0.1 to 0.4)	22.2 (-3.6 to 50.7)	1.6 (-21.5 to 29)	150.9 (64.6 to 334.3)	8.9 (-30.8 to 115.7)
	Scabies	Both	322 (175 to 521)	25.3 (13.9 to 40.5)	--	--	320 (174 to 518)	25.1 (13.7 to 40.6)	--	--	-0.7 (-9.1 to 9.2)	-0.7 (-5.4 to 4.1)	--	--
		Female	158 (85 to 256)	25.4 (13.9 to 41)	--	--	155 (85 to 249)	25.2 (13.7 to 40.4)	--	--	-1.7 (-11.2 to 10.6)	-0.8 (-7 to 5.8)	--	--
		Male	164 (89 to 264)	25.2 (13.9 to 40.7)	--	--	164 (91 to 268)	25 (13.8 to 40.7)	--	--	0.3 (-9.6 to 11.1)	-0.7 (-6.6 to 5.7)	--	--
	Fungal skin diseases	Both	155 (62 to 329)	19 (7.6 to 40.2)	--	--	231 (93 to 494)	18.5 (7.5 to 39.7)	--	--	48.7 (37.3 to 60.2)	-2.4 (-4.4 to -0.7)	--	--
		Female	69 (28 to 147)	17.7 (7.1 to 38)	--	--	107 (44 to 232)	17.3 (7 to 37.3)	--	--	55.3 (41.9 to 68.2)	-2.1 (-4.7 to 0.5)	--	--

Province	Cause	Sex	1990				2019				1990-2019 percent change (%)			
			DALYs		Death		DALYs		Death		DALYs		Death	
			Number	Rate	Number	Rate	Number	Rate	Number	Rate	Number	Rate	Number	Rate
		Male	86 (34 to 183)	20.2 (8.1 to 42.8)	--	--	123 (50 to 266)	19.7 (7.9 to 42.3)	--	--	43.5 (31.7 to 55.8)	-2.3 (-4.9 to 0.2)	--	--
	Viral skin diseases	Both	813 (518 to 1231)	51.2 (32.9 to 76.9)	--	--	605 (386 to 900)	51.4 (32.7 to 76.8)	--	--	-25.5 (-29 to -21.8)	0.3 (-4 to 4.6)	--	--
		Female	392 (248 to 601)	50.8 (32.4 to 77.7)	--	--	291 (188 to 436)	50.9 (33 to 76.5)	--	--	-25.7 (-30.5 to -20.3)	0.3 (-5.6 to 6.6)	--	--
		Male	420 (267 to 637)	51.6 (32.9 to 78)	--	--	314 (199 to 468)	51.8 (32.6 to 77.1)	--	--	-25.2 (-30.2 to -20.4)	0.3 (-5.7 to 6.3)	--	--
	Acne vulgaris	Both	764 (459 to 1210)	49.9 (30.2 to 78.8)	--	--	791 (470 to 1248)	69.7 (41.5 to 110.4)	--	--	3.5 (-5.4 to 13.6)	39.6 (28.2 to 52)	--	--
		Female	356 (214 to 564)	48.4 (29.1 to 76.6)	--	--	374 (222 to 589)	67.7 (40 to 107.4)	--	--	4.9 (-5.9 to 18.5)	40.1 (26 to 57)	--	--
		Male	408 (240 to 653)	51.2 (30.2 to 81.7)	--	--	417 (249 to 665)	71.4 (42.4 to 114.1)	--	--	2.3 (-9.5 to 17.2)	39.3 (23.4 to 58)	--	--
	Alopecia areata	Both	62 (39 to 93)	6.3 (4.1 to 9.6)	--	--	91 (58 to 135)	6.3 (4 to 9.5)	--	--	47 (31.1 to 64.6)	0 (-9.1 to 10.4)	--	--
		Female	40 (25 to 61)	8.3 (5.3 to 12.7)	--	--	58 (36 to 87)	8.3 (5.1 to 12.6)	--	--	46.6 (27.3 to 67.9)	0.4 (-11.8 to 13.9)	--	--
		Male	22 (14 to 34)	4.5 (2.8 to 6.7)	--	--	33 (21 to 50)	4.5 (2.8 to 6.7)	--	--	47.6 (22.8 to 79.7)	0.2 (-15.9 to 19)	--	--
	Pruritus	Both	94 (45 to 170)	10 (4.8 to 17.9)	--	--	139 (66 to 250)	10.5 (5 to 18.9)	--	--	48.5 (35.6 to 63.3)	5.7 (0.5 to 11.4)	--	--
		Female	50 (23 to 92)	10.9 (5.2 to 19.7)	--	--	75 (36 to 132)	11.5 (5.5 to 20.5)	--	--	50.1 (34.5 to 67.9)	5.4 (-2.1 to 13.5)	--	--
		Male	44 (21 to 81)	9.1 (4.4 to 16.5)	--	--	64 (30 to 116)	9.6 (4.5 to 17.5)	--	--	46.7 (32 to 63.6)	5.6 (-1.6 to 13.3)	--	--
	Urticaria	Both	787 (520 to 1161)	57.9 (38.3 to 84.3)	--	--	729 (476 to 1037)	58.2 (38 to 83.5)	--	--	-7.4 (-15.8 to 2.1)	0.4 (-4.8 to 6.1)	--	--
		Female	476 (309 to 702)	70.8 (46.8 to 102.8)	--	--	430 (281 to 616)	71 (46.6 to 102.6)	--	--	-9.6 (-18.4 to 0.5)	0.4 (-6.4 to 7.5)	--	--
		Male	311 (200 to 463)	45.7 (29.6 to 66.4)	--	--	299 (194 to 436)	46 (30.2 to 67.3)	--	--	-3.9 (-15.8 to 9.4)	0.6 (-7.9 to 9.9)	--	--
	Decubitus ulcer	Both	3 (2 to 7)	0.8 (0.5 to 1.8)	0 (0 to 0)	0 (0 to 0.1)	14 (11 to 18)	1.4 (1.1 to 1.8)	1 (0 to 1)	0.1 (0 to 0.1)	311.5 (96 to 477.6)	71.9 (-26.7 to 149.7)	808 (118.5 to 2411.5)	146.4 (-40.7 to 597.2)
		Female	1 (1 to 3)	0.7 (0.4 to 1.8)	0 (0 to 0)	0 (0 to 0.1)	5 (4 to 7)	1 (0.7 to 1.4)	0 (0 to 0)	0 (0 to 0.1)	269.8 (68.8 to 462.1)	39.9 (-46.2 to 127.9)	579.8 (25.1 to 2259.3)	78.4 (-69.2 to 545.7)
		Male	2 (1 to 4)	0.9 (0.5 to 2.1)	0 (0 to 0)	0 (0 to 0.1)	9 (6 to 12)	1.8 (1.3 to 2.4)	0 (0 to 1)	0.1 (0.1 to 0.1)	342 (106.7 to 552)	103 (-16.6 to 210.3)	1029.8 (162 to 3535.3)	215 (-24.2 to 969.9)
	Other skin and subcutaneous diseases	Both	363 (173 to 671)	41.6 (19.9 to 75.5)	0 (0 to 0)	0 (0 to 0)	561 (272 to 1017)	43.6 (21.1 to 78.8)	0 (0 to 0)	0 (0 to 0)	54.6 (49.8 to 59.7)	4.8 (2.5 to 7.3)	789.3 (141.4 to 2115.8)	129.9 (-35.3 to 578.6)
		Female	177 (85 to 327)	42.1 (20.1 to 76.2)	0 (0 to 0)	0 (0 to 0)	282 (136 to 514)	44.3 (21.4 to 80.5)	0 (0 to 0)	0 (0 to 0)	59.4 (53.2 to 66.8)	5.3 (1.8 to 9.1)	1255.3 (147 to 4446)	230.5 (-42.8 to 1118.3)

Province	Cause	Sex	1990				2019				1990-2019 percent change (%)			
			DALYs		Death		DALYs		Death		DALYs		Death	
			Number	Rate	Number	Rate	Number	Rate	Number	Rate	Number	Rate	Number	Rate
		Male	185 (87 to 341)	41.1 (19.6 to 74.9)	0 (0 to 0)	0 (0 to 0)	278 (133 to 510)	42.8 (20.5 to 78.6)	0 (0 to 0)	0 (0 to 0)	50 (44.1 to 56.2)	4.3 (0.9 to 7.6)	169.2 (34.9 to 594.2)	-20.9 (-63.2 to 113.6)
	Sense organ diseases	Both	6298 (4464 to 8450)	1056.3 (759.3 to 1410.4)	--	--	9439 (6651 to 12879)	814.8 (576.7 to 1102.8)	--	--	49.9 (41 to 59)	-22.9 (-25.8 to -19.6)	--	--
		Female	2957 (2092 to 3995)	1057.8 (765.1 to 1408.5)	--	--	4774 (3402 to 6464)	815.3 (583.8 to 1099.2)	--	--	61.5 (51.6 to 71)	-22.9 (-26.1 to -19.4)	--	--
		Male	3341 (2373 to 4489)	1054.7 (756 to 1407.2)	--	--	4664 (3284 to 6422)	814.2 (573.8 to 1103.9)	--	--	39.6 (30.5 to 49)	-22.8 (-26.1 to -19.2)	--	--
	Blindness and vision loss	Both	2924 (2077 to 3956)	515.6 (369.5 to 687.6)	--	--	4221 (2987 to 5709)	375 (265.6 to 505.4)	--	--	44.3 (37.9 to 50.8)	-27.3 (-30.3 to -24.2)	--	--
		Female	1442 (1024 to 1945)	544.6 (390.6 to 721)	--	--	2264 (1603 to 3067)	395.3 (281.2 to 530.6)	--	--	57 (49.3 to 65.2)	-27.4 (-30.8 to -23.8)	--	--
		Male	1482 (1047 to 2007)	490.7 (351 to 655.9)	--	--	1957 (1389 to 2649)	353.6 (250.9 to 476.3)	--	--	32.1 (25.6 to 39.3)	-27.9 (-31.4 to -24)	--	--
	Age-related and other hearing loss	Both	3063 (2109 to 4228)	499.6 (356.5 to 689)	--	--	4677 (3238 to 6544)	396.8 (277.9 to 551.4)	--	--	52.7 (36.2 to 68.9)	-20.6 (-24.4 to -16.4)	--	--
		Female	1354 (946 to 1865)	468.4 (329.9 to 640.5)	--	--	2219 (1533 to 3114)	373.5 (260.8 to 517.5)	--	--	63.9 (43.5 to 83.9)	-20.3 (-25.2 to -15.5)	--	--
		Male	1709 (1182 to 2369)	526.2 (373.3 to 726)	--	--	2459 (1688 to 3464)	421 (292 to 589.9)	--	--	43.9 (27.5 to 59.7)	-20 (-24.7 to -15.4)	--	--
	Other sense organ diseases	Both	311 (188 to 475)	41.1 (24.2 to 63.6)	--	--	540 (321 to 835)	43.1 (25.6 to 66.2)	--	--	73.7 (56.6 to 92.2)	4.7 (-0.4 to 10.3)	--	--
		Female	161 (96 to 247)	44.8 (26.9 to 69.6)	--	--	291 (171 to 449)	46.5 (27.6 to 71.7)	--	--	80.7 (61.3 to 103.1)	3.9 (-2.9 to 11)	--	--
		Male	149 (89 to 232)	37.8 (22.4 to 59)	--	--	248 (148 to 384)	39.5 (23.7 to 61.2)	--	--	66.3 (47.4 to 88.9)	4.6 (-2.7 to 13.3)	--	--
	Musculoskeletal disorders	Both	13252 (9382 to 17672)	1866.9 (1332.8 to 2466.4)	3 (2 to 4)	0.5 (0.4 to 0.7)	26795 (18974 to 35442)	1948.6 (1383.1 to 2574.1)	8 (6 to 10)	0.7 (0.6 to 0.9)	102.2 (92.3 to 112.4)	4.4 (1 to 8)	229.6 (113.8 to 377.3)	50.8 (4 to 114.1)
		Female	6909 (4910 to 9144)	2022.5 (1437.9 to 2661.4)	2 (1 to 3)	0.6 (0.4 to 1)	14604 (10418 to 19261)	2149.4 (1537.5 to 2849)	5 (3 to 6)	0.8 (0.5 to 1)	111.4 (99.2 to 122.7)	6.3 (2 to 10.7)	222.4 (88.9 to 424.7)	42.6 (-16.5 to 140.5)
		Male	6344 (4483 to 8474)	1724.3 (1217.1 to 2299.7)	1 (1 to 1)	0.4 (0.3 to 0.5)	12191 (8569 to 16234)	1749.1 (1237.2 to 2314.3)	3 (3 to 4)	0.7 (0.5 to 0.8)	92.2 (80.8 to 104.4)	1.4 (-3.1 to 6.5)	240.7 (90.2 to 401.8)	60.9 (0.7 to 133.4)
	Rheumatoid arthritis	Both	82 (57 to 111)	11.8 (8.3 to 15.7)	0 (0 to 1)	0.1 (0 to 0.1)	184 (129 to 248)	13.3 (9.5 to 17.7)	1 (1 to 1)	0.1 (0.1 to 0.1)	124.2 (88.8 to 159.5)	12.8 (-4.9 to 30.2)	223.4 (37.6 to 509.9)	23.8 (-46 to 134.2)
		Female	57 (39 to 77)	16.9 (11.6 to 22.9)	0 (0 to 1)	0.1 (0.1 to 0.3)	128 (89 to 176)	18.6 (13 to 25.2)	1 (0 to 1)	0.1 (0 to 0.2)	125.2 (80.1 to 177.4)	9.9 (-14 to 34.1)	184.4 (-12.9 to 592)	-1.9 (-68.8 to 150.2)
		Male	25 (17 to 34)	7 (4.8 to 9.6)	0 (0 to 0)	0 (0 to 0.1)	56 (39 to 75)	8.1 (5.7 to 10.8)	0 (0 to 0)	0.1 (0 to 0.1)	121.9 (85.2 to 163.7)	15.6 (-2.6 to 37.7)	337.5 (166.8 to 707.9)	90.7 (17.2 to 249.9)
	Osteoarthritis	Both	914 (462 to 1782)	173.8 (87.9 to 338.3)	--	--	2276 (1144 to 4474)	190.4 (95.8 to 376.7)	--	--	149.1 (139.1 to 158.3)	9.6 (6 to 13.1)	--	--

Province	Cause	Sex	1990				2019				1990-2019 percent change (%)			
			DALYs		Death		DALYs		Death		DALYs		Death	
			Number	Rate	Number	Rate	Number	Rate	Number	Rate	Number	Rate	Number	Rate
		Female	479 (239 to 929)	198.6 (99.8 to 385.3)	--	--	1313 (663 to 2546)	216 (109.2 to 419.3)	--	--	174.2 (159.6 to 188.2)	8.7 (3.7 to 13.6)	--	--
		Male	435 (216 to 865)	152.5 (76.1 to 300.4)	--	--	963 (481 to 1911)	163.6 (82.3 to 325.1)	--	--	121.4 (108.2 to 134.4)	7.3 (1.6 to 12.3)	--	--
	Low back pain	Both	7773 (5455 to 10391)	1034.2 (728.6 to 1382.3)	--	--	12796 (8907 to 17276)	931.4 (658.3 to 1241.4)	--	--	64.6 (53.6 to 75.8)	-9.9 (-13.9 to -5.9)	--	--
		Female	3599 (2523 to 4850)	983.7 (693.1 to 1320.8)	--	--	6199 (4288 to 8281)	922.4 (648.2 to 1232.3)	--	--	72.2 (57.2 to 86.4)	-6.2 (-11.7 to -0.4)	--	--
		Male	4174 (2918 to 5676)	1082.1 (760.3 to 1445.6)	--	--	6597 (4585 to 8897)	939.2 (656.6 to 1261.6)	--	--	58.1 (45.4 to 72.7)	-13.2 (-18.5 to -7.5)	--	--
	Neck pain	Both	2777 (1808 to 4051)	402.2 (265.2 to 582.1)	--	--	5750 (3761 to 8451)	404.9 (266.4 to 586.7)	--	--	107.1 (93.1 to 120.4)	0.7 (-1.4 to 2.8)	--	--
		Female	1642 (1078 to 2399)	492.1 (325.6 to 719.6)	--	--	3467 (2282 to 5087)	493.1 (323.9 to 721.1)	--	--	111.2 (96.2 to 124.7)	0.2 (-2.5 to 2.9)	--	--
		Male	1135 (732 to 1663)	317.9 (206.9 to 464.4)	--	--	2283 (1480 to 3340)	318.5 (208.6 to 462.6)	--	--	101.1 (85.1 to 117.5)	0.2 (-2.9 to 3.6)	--	--
	Gout	Both	78 (49 to 116)	14.2 (9 to 20.5)	--	--	193 (119 to 279)	15.2 (9.5 to 22.1)	--	--	146.4 (108.9 to 191.6)	7.2 (-6.7 to 23.6)	--	--
		Female	16 (9 to 23)	6 (3.7 to 8.9)	--	--	44 (26 to 65)	7 (4.2 to 10.4)	--	--	183.9 (125.8 to 266.5)	15.7 (-6.8 to 45.4)	--	--
		Male	63 (40 to 93)	21.5 (13.5 to 31.1)	--	--	148 (93 to 218)	23.7 (14.6 to 34.6)	--	--	137.1 (96.4 to 190.5)	10 (-6.8 to 30.5)	--	--
	Other musculoskeletal disorders	Both	1629 (1044 to 2346)	230.7 (148.3 to 333.1)	2 (2 to 3)	0.4 (0.3 to 0.6)	5596 (3770 to 7871)	393.4 (267.8 to 547.5)	7 (6 to 9)	0.7 (0.5 to 0.8)	243.6 (206.5 to 289.9)	70.6 (52 to 94.6)	230.4 (121.5 to 388.9)	55.5 (11.1 to 121.5)
		Female	1116 (743 to 1563)	325.1 (217.8 to 459.5)	1 (1 to 2)	0.5 (0.3 to 0.8)	3452 (2362 to 4848)	492.4 (335.2 to 685.8)	4 (3 to 6)	0.7 (0.5 to 0.9)	209.2 (176.4 to 247.1)	51.4 (35.4 to 70)	228.7 (108.5 to 432.6)	53.7 (-3.3 to 150.4)
		Male	512 (290 to 796)	143.3 (80.1 to 221.8)	1 (1 to 1)	0.4 (0.3 to 0.5)	2144 (1374 to 3125)	296 (190.8 to 426.9)	3 (2 to 4)	0.6 (0.4 to 0.7)	318.6 (244.7 to 445.6)	106.5 (72.1 to 167.9)	232.9 (78.9 to 393)	58.3 (-5.5 to 128.5)
	Other non-communicable diseases	Both	119580 (75943 to 166391)	7520.9 (5006.5 to 10265.2)	1224 (741 to 1747)	71 (43.5 to 101.2)	27598 (21619 to 34943)	2247.1 (1788.1 to 2810.9)	137 (112 to 171)	13.4 (10.9 to 16.9)	-76.9 (-85 to -60.6)	-70.1 (-79.8 to -52.5)	-88.8 (-92.8 to -78.7)	-81.1 (-87.8 to -64.1)
		Female	56975 (32693 to 80312)	7693.5 (4714.9 to 10424.6)	551 (275 to 813)	65.7 (34.1 to 96)	17759 (13582 to 23134)	2794.7 (2163.5 to 3562.5)	63 (50 to 79)	12.5 (9.9 to 15.9)	-68.8 (-80 to -42.7)	-63.7 (-75.5 to -39.6)	-88.6 (-93.1 to -75.1)	-81.1 (-88.5 to -59.3)
		Male	62605 (26399 to 90249)	7325.7 (3318 to 10440.7)	673 (262 to 984)	76 (30.4 to 110.5)	9839 (7794 to 12489)	1731.1 (1378.2 to 2193.5)	75 (59 to 94)	14.4 (11.4 to 18.4)	-84.3 (-90 to -61.7)	-76.4 (-84.8 to -45.2)	-88.9 (-93 to -70)	-81.1 (-88.1 to -50.1)
	Congenital birth defects	Both	103077 (59841 to 149064)	5888.6 (3436.8 to 8518.9)	1155 (661 to 1674)	66 (37.9 to 95.7)	9904 (7795 to 12876)	971.4 (757.3 to 1271.4)	103 (80 to 135)	10.2 (7.9 to 13.5)	-90.4 (-94.1 to -80.3)	-83.5 (-90 to -65.9)	-91.1 (-94.5 to -81.3)	-84.5 (-90.6 to -67.2)

Province	Cause	Sex	1990				2019				1990-2019 percent change (%)			
			DALYs		Death		DALYs		Death		DALYs		Death	
			Number	Rate	Number	Rate	Number	Rate	Number	Rate	Number	Rate	Number	Rate
		Female	46155 (21017 to 69358)	5404.1 (2481.9 to 8113.5)	519 (234 to 784)	60.7 (27.7 to 91.5)	4538 (3502 to 5926)	915.7 (701.3 to 1208.3)	48 (36 to 63)	9.7 (7.3 to 13)	-90.2 (-94.3 to -75.8)	-83.1 (-90.2 to -58.4)	-90.8 (-94.7 to -76.7)	-84 (-90.9 to -59.8)
		Male	56922 (19462 to 84560)	6347.6 (2193.1 to 9396.9)	637 (210 to 953)	71 (23.7 to 105.9)	5366 (4121 to 7044)	1024.1 (781 to 1364.6)	55 (42 to 74)	10.7 (8 to 14.3)	-90.6 (-94.3 to -69.1)	-83.9 (-90.4 to -47.6)	-91.3 (-94.9 to -70.5)	-84.9 (-91.2 to -49.3)
	Urinary diseases and male infertility	Both	237 (171 to 322)	28.8 (20 to 40.3)	1 (1 to 3)	0.2 (0.1 to 0.7)	402 (296 to 540)	32.1 (23.9 to 42.6)	5 (4 to 6)	0.5 (0.4 to 0.6)	69.7 (41.8 to 93.1)	11.5 (-12.8 to 27.7)	294.1 (112 to 472.2)	154.5 (-16.2 to 350)
		Female	83 (61 to 109)	15.9 (11.3 to 25)	1 (0 to 2)	0.2 (0.1 to 0.8)	108 (86 to 137)	17.2 (13.6 to 21.7)	2 (2 to 3)	0.4 (0.3 to 0.6)	30.6 (5.8 to 61.5)	8 (-29.3 to 37)	200.9 (75.7 to 382.1)	73.7 (-23.6 to 253.3)
		Male	154 (104 to 225)	40 (26.3 to 58.9)	1 (0 to 1)	0.2 (0.1 to 0.6)	294 (204 to 415)	47.5 (33.4 to 66.3)	3 (2 to 4)	0.6 (0.5 to 0.8)	90.8 (58.3 to 120.2)	18.9 (-6.1 to 37.4)	397.9 (102.4 to 664.7)	265.9 (-9.9 to 538.2)
	Gynecological diseases	Female	4426 (3011 to 6191)	1103.8 (749.9 to 1526.7)	0 (0 to 0)	0 (0 to 0)	7884 (5380 to 11027)	1046 (713.2 to 1456.7)	0 (0 to 0)	0 (0 to 0)	78.2 (66.9 to 88.8)	-5.2 (-8.5 to -2.4)	197.7 (-59.6 to 1339.1)	53.2 (-75.8 to 523.5)
	Hemoglobinopathies and hemolytic anemias	Both	2411 (1726 to 3340)	183.5 (129.8 to 253.5)	10 (7 to 15)	0.8 (0.6 to 1)	898 (606 to 1336)	71.2 (48.3 to 104.5)	4 (3 to 4)	0.3 (0.3 to 0.3)	-62.7 (-71.9 to -51.3)	-61.2 (-69.7 to -51.2)	-66 (-77 to -47.6)	-60.6 (-70.9 to -45.3)
		Female	1481 (978 to 2096)	243 (162.8 to 348.4)	6 (3 to 9)	0.8 (0.6 to 1.2)	575 (361 to 875)	90.5 (57.7 to 136.3)	2 (1 to 2)	0.3 (0.2 to 0.3)	-61.2 (-72.7 to -44.2)	-62.7 (-72.7 to -49.5)	-70.5 (-82.3 to -44.1)	-66.2 (-77.6 to -44)
		Male	930 (634 to 1313)	125.1 (87.7 to 177.6)	5 (3 to 7)	0.7 (0.5 to 0.9)	324 (215 to 493)	53 (35.1 to 81.3)	2 (2 to 2)	0.3 (0.3 to 0.4)	-65.2 (-76.5 to -50)	-57.6 (-71.1 to -40.7)	-60.8 (-74.2 to -36.5)	-53.5 (-67.1 to -33.1)
	Endocrine, metabolic, blood, and immune disorders	Both	4216 (2989 to 5994)	377.8 (274.4 to 505.7)	23 (15 to 41)	2.1 (1.6 to 3.3)	4236 (3049 to 5663)	330.8 (242.1 to 444.4)	23 (16 to 28)	2.1 (1.5 to 2.6)	0.5 (-30.5 to 24.5)	-12.4 (-32.7 to 0.4)	-1.8 (-54.1 to 58.7)	0.3 (-44.2 to 44.4)
		Female	2385 (1599 to 3374)	453.6 (315.7 to 627.9)	11 (6 to 21)	2.1 (1.3 to 3.7)	2581 (1813 to 3528)	402.6 (287.3 to 548.8)	10 (7 to 12)	1.8 (1.2 to 2.3)	8.2 (-26.1 to 35.4)	-11.2 (-30.9 to 2.6)	-6.6 (-59.6 to 72.5)	-13.4 (-55.4 to 48.8)
		Male	1831 (1149 to 2805)	304.6 (215.4 to 430.6)	13 (6 to 22)	2.2 (1.5 to 3.4)	1655 (1202 to 2187)	263 (191.8 to 344.6)	13 (8 to 17)	2.5 (1.5 to 3.2)	-9.6 (-40.8 to 27.8)	-13.6 (-36.5 to 7.4)	2.2 (-50.8 to 72.3)	14.7 (-34.3 to 72.7)
	Oral disorders	Both	2238 (1323 to 3517)	320 (189.8 to 498.5)	--	--	4083 (2420 to 6508)	313.1 (185.6 to 492.1)	--	--	82.5 (74.3 to 91.4)	-2.1 (-4.6 to 0.2)	--	--
		Female	1058 (622 to 1673)	308.9 (184 to 483.1)	--	--	1982 (1167 to 3150)	303.9 (180.8 to 474.3)	--	--	87.4 (77.2 to 97.9)	-1.6 (-4.6 to 1.3)	--	--
		Male	1180 (702 to 1839)	329.1 (196.6 to 509.3)	--	--	2101 (1243 to 3354)	323.4 (192.5 to 507.5)	--	--	78 (69.3 to 88.1)	-1.7 (-4.8 to 1.4)	--	--
	Sudden infant death syndrome	Both	2976 (353 to 10315)	171.9 (20.4 to 595.9)	34 (4 to 117)	1.9 (0.2 to 6.7)	191 (91 to 389)	19.5 (9.3 to 39.7)	2 (1 to 4)	0.2 (0.1 to 0.4)	-93.6 (-98.1 to -48.2)	-88.7 (-96.7 to -8.4)	-93.6 (-98.1 to -48.2)	-88.7 (-96.7 to -8.4)
		Female	1388 (180 to 5249)	164.2 (21.4 to 621.1)	16 (2 to 59)	1.9 (0.2 to 7)	90 (44 to 185)	18.9 (9.2 to 38.8)	1 (0 to 2)	0.2 (0.1 to 0.4)	-93.5 (-98.1 to -47.2)	-88.5 (-96.6 to -6.3)	-93.5 (-98.1 to -47.2)	-88.5 (-96.6 to -6.3)
		Male	1588 (154 to 5977)	179.3 (17.4 to 674.7)	18 (2 to 68)	2 (0.2 to 7.6)	100 (43 to 220)	20 (8.5 to 43.8)	1 (0 to 2)	0.2 (0.1 to 0.5)	-93.7 (-98.2 to -41.7)	-88.9 (-96.8 to 2.8)	-93.7 (-98.2 to -41.7)	-88.9 (-96.8 to 2.8)
	**Injuries**	Both	94437 (73002 to 116589)	7279.4 (5755 to 8608.3)	1226 (931 to 1493)	108.9 (85.8 to 129.4)	35701 (31175 to 40913)	2625.8 (2305.7 to 3003.1)	578 (496 to 692)	44.6 (38.3 to 53.7)	-62.2 (-70.2 to -50.6)	-63.9 (-70.7 to -53.3)	-52.9 (-64.1 to -35.1)	-59.1 (-67.7 to -45.1)
		Female	34128 (25768 to 44459)	5013.2 (3869.5 to 6306)	411 (304 to 532)	67.9 (50.6 to 84.1)	9185 (7965 to 10548)	1408.2 (1221.1 to 1615.9)	129 (109 to 150)	20.8 (17.6 to 24.2)	-73.1 (-79.9 to -63.2)	-71.9 (-78.3 to -62.9)	-68.6 (-77.6 to -55)	-69.4 (-77.2 to -57.5)
		Male	60309 (46960 to 74122)	9459.7 (7520.2 to 11393.6)	815 (632 to 1010)	148 (115 to 181.1)	26516 (22845 to 30677)	3789.5 (3279.9 to 4404.7)	449 (377 to 543)	68 (57.1 to 83.1)	-56 (-66.1 to -41.6)	-59.9 (-68.5 to -47.4)	-44.9 (-58.8 to -23.5)	-54.1 (-65.2 to -36.9)

Province	Cause	Sex	1990				2019				1990-2019 percent change (%)			
			DALYs		Death		DALYs		Death		DALYs		Death	
			Number	Rate	Number	Rate	Number	Rate	Number	Rate	Number	Rate	Number	Rate
	Transport injuries	Both	44366 (32429 to 57878)	3366.2 (2551.3 to 4218.2)	628 (460 to 801)	56.4 (42.6 to 69.1)	14948 (12470 to 19500)	1111.9 (931.4 to 1458)	290 (236 to 397)	22.2 (18.1 to 30.9)	-66.3 (-75.4 to -52.1)	-67 (-74.9 to -53.9)	-53.9 (-66.1 to -33.2)	-60.6 (-70 to -44.6)
		Female	15554 (10619 to 22087)	2183.9 (1554.3 to 2955.7)	202 (140 to 281)	32.4 (23.5 to 42.2)	3167 (2682 to 3676)	493 (420.7 to 571.8)	58 (47 to 68)	9.1 (7.5 to 10.6)	-79.6 (-86.1 to -69.5)	-77.4 (-83.8 to -67.2)	-71.4 (-80 to -57.4)	-71.8 (-79.3 to -59.8)
		Male	28812 (21079 to 37697)	4504.5 (3356.6 to 5673.2)	426 (314 to 550)	79.2 (58.7 to 101.1)	11781 (9586 to 16036)	1702.4 (1383.6 to 2337.8)	232 (184 to 336)	35.1 (27.8 to 52.2)	-59.1 (-70.7 to -41)	-62.2 (-72 to -46.6)	-45.5 (-60.6 to -19.6)	-55.6 (-67.5 to -36.6)
	Road injuries	Both	43714 (31880 to 57170)	3310.2 (2502.9 to 4149.5)	619 (452 to 791)	55.5 (41.9 to 68.1)	14288 (11911 to 18549)	1063.1 (884.2 to 1392.2)	278 (226 to 381)	21.3 (17.3 to 29.8)	-67.3 (-76.2 to -53.5)	-67.9 (-75.7 to -54.9)	-55.1 (-67.2 to -35)	-61.6 (-70.7 to -45.7)
		Female	15401 (10512 to 21788)	2158.1 (1534.8 to 2918.7)	200 (139 to 278)	32.1 (23.2 to 41.8)	3052 (2584 to 3557)	475.2 (404.5 to 553.6)	56 (46 to 66)	8.9 (7.3 to 10.3)	-80.2 (-86.5 to -70.2)	-78 (-84.3 to -68)	-72 (-80.4 to -58.3)	-72.4 (-79.8 to -60.7)
		Male	28313 (20722 to 37216)	4419.4 (3292.6 to 5566.5)	419 (308 to 541)	77.8 (57.3 to 99.2)	11235 (9076 to 15349)	1623.9 (1318 to 2238.1)	222 (176 to 321)	33.5 (26.4 to 49.9)	-60.3 (-71.7 to -42.8)	-63.3 (-73 to -47.9)	-47.1 (-61.9 to -22.3)	-56.9 (-68.5 to -37.9)
	Other transport injuries	Both	652 (466 to 972)	56 (41.6 to 78.9)	9 (6 to 13)	0.9 (0.6 to 1.3)	660 (536 to 897)	48.7 (39.7 to 66.4)	12 (9 to 18)	0.9 (0.7 to 1.4)	1.3 (-33.5 to 47.1)	-13 (-39.6 to 20.8)	35.9 (-13.6 to 108.8)	3.6 (-32.4 to 56.7)
		Female	153 (106 to 228)	25.8 (18.6 to 35.8)	2 (1 to 3)	0.3 (0.2 to 0.5)	115 (94 to 142)	17.7 (14.6 to 21.6)	2 (1 to 2)	0.3 (0.2 to 0.3)	-25 (-48.4 to 9.2)	-31.2 (-49.3 to -5.2)	-7.6 (-40.3 to 51.5)	-20.4 (-45.8 to 25.4)
		Male	499 (335 to 817)	85.1 (61 to 130)	7 (5 to 12)	1.4 (1 to 2.2)	545 (429 to 777)	78.5 (61.8 to 113.1)	10 (8 to 16)	1.6 (1.2 to 2.5)	9.3 (-35.9 to 66)	-7.7 (-41.4 to 34.6)	46.3 (-11.5 to 134)	10.5 (-31.9 to 74.9)
	Unintentional injuries	Both	36898 (28477 to 46839)	2693.6 (2152 to 3283.2)	450 (343 to 574)	37.7 (30.1 to 45.4)	11938 (10071 to 14161)	899 (763.5 to 1059.3)	163 (138 to 199)	13.3 (11.3 to 16.2)	-67.6 (-75.9 to -56.1)	-66.6 (-73.9 to -57)	-63.8 (-73.6 to -48.5)	-64.6 (-72.9 to -52.2)
		Female	13995 (9947 to 19394)	1976.2 (1468 to 2603.4)	165 (116 to 229)	26.6 (19.4 to 34.4)	3426 (2814 to 4205)	541.5 (446.6 to 660.4)	42 (34 to 55)	7.3 (5.9 to 9.4)	-75.5 (-83.4 to -63.6)	-72.6 (-80.4 to -61.9)	-74.4 (-83.5 to -58.8)	-72.7 (-81.4 to -59.7)
		Male	22902 (17302 to 29388)	3382.5 (2651.7 to 4200.1)	285 (212 to 367)	48.3 (36.8 to 60.8)	8513 (7162 to 10272)	1243.8 (1045.4 to 1481.2)	121 (100 to 150)	19.4 (16.2 to 24.2)	-62.8 (-72.6 to -48.4)	-63.2 (-71.5 to -51)	-57.6 (-69.8 to -37.2)	-59.8 (-69.8 to -43.2)
	Falls	Both	4582 (3375 to 6151)	449.6 (344.4 to 573.6)	46 (31 to 66)	5.7 (4.1 to 7.6)	3661 (2905 to 4596)	276.5 (221 to 344.3)	43 (35 to 56)	3.8 (3.1 to 4.9)	-20.1 (-42.4 to 7.1)	-38.5 (-50.5 to -25.3)	-7.4 (-38.4 to 42.6)	-33.7 (-49.2 to -6.7)
		Female	1183 (761 to 1858)	235.4 (174 to 321.2)	11 (7 to 20)	3.1 (2 to 5)	834 (626 to 1108)	134.1 (101.2 to 177.3)	9 (6 to 15)	1.6 (1.2 to 2.8)	-29.5 (-57.6 to 13.8)	-43 (-57.9 to -26.6)	-21.2 (-55.8 to 58.9)	-46.4 (-61 to -18)
		Male	3399 (2327 to 4770)	653.1 (491.2 to 848.4)	35 (22 to 51)	8.1 (5.7 to 11)	2827 (2242 to 3551)	415.6 (331.9 to 519.1)	34 (27 to 47)	5.9 (4.8 to 8.2)	-16.8 (-42.9 to 27)	-36.4 (-50.3 to -16.3)	-3.2 (-38.3 to 76)	-27 (-48.1 to 10.6)
	Drowning	Both	6677 (4223 to 9382)	391.1 (253.9 to 542.9)	84 (54 to 116)	5.2 (3.5 to 7.2)	740 (595 to 901)	59.2 (47.8 to 72.3)	13 (10 to 15)	1 (0.8 to 1.2)	-88.9 (-92.5 to -82.5)	-84.8 (-89.7 to -76.4)	-84.6 (-89.5 to -75.9)	-80.6 (-86.7 to -69.9)
		Female	2199 (1251 to 3693)	251.3 (146.8 to 414.2)	26 (15 to 44)	3.1 (1.9 to 5)	125 (101 to 156)	21.5 (17.1 to 27.3)	2 (2 to 2)	0.3 (0.3 to 0.4)	-94.3 (-96.8 to -89.5)	-91.4 (-95.1 to -84.4)	-92.6 (-95.8 to -86.8)	-89.4 (-93.8 to -81.4)
		Male	4478 (2433 to 6948)	525.1 (296.1 to 795.4)	57 (32 to 87)	7.3 (4.2 to 10.7)	615 (480 to 773)	94.7 (74 to 119.1)	11 (9 to 14)	1.7 (1.3 to 2.1)	-86.3 (-91.7 to -74.3)	-82 (-88.8 to -67.4)	-81 (-88.2 to -66)	-77 (-85 to -59.9)
	Fire, heat, and hot substances	Both	5683 (2971 to 8948)	405.9 (233.2 to 604.2)	75 (41 to 117)	6.5 (3.9 to 9.3)	997 (683 to 1601)	75.5 (51.5 to 123.6)	21 (13 to 36)	1.7 (1.1 to 3)	-82.5 (-91.9 to -56.5)	-81.4 (-91 to -56.9)	-72.4 (-87.7 to -28.8)	-73.9 (-87.3 to -43.2)
		Female	2487 (552 to 4884)	368.4 (116.1 to 674.9)	33 (8 to 63)	5.8 (2.3 to 10)	484 (309 to 807)	75.9 (47.9 to 127.1)	10 (6 to 18)	1.6 (0.9 to 3)	-80.5 (-93.1 to 9.6)	-79.4 (-92 to -16.2)	-69.7 (-90 to 70.6)	-71.8 (-89.6 to -7)
		Male	3196 (1183 to 4828)	442.1 (193.3 to 628)	43 (16 to 63)	7.2 (3.5 to 9.8)	513 (351 to 930)	75.1 (51.1 to 140.4)	11 (7 to 22)	1.8 (1.1 to 3.6)	-84 (-91.5 to -22.8)	-83 (-90.7 to -33.4)	-74.5 (-86.9 to 17.7)	-75.4 (-86.9 to -14.8)
	Poisonings	Both	5800 (3050 to 9057)	376.8 (204.9 to 555.1)	75 (40 to 113)	5.6 (3.1 to 7.7)	824 (618 to 995)	60.5 (46.7 to 72.9)	17 (12 to 20)	1.2 (0.9 to 1.5)	-85.8 (-91.3 to -75)	-83.9 (-89.6 to -72.4)	-77.9 (-85.7 to -63.3)	-77.7 (-84.7 to -64.4)

Province	Cause	Sex	1990				2019				1990-2019 percent change (%)			
			DALYs		Death		DALYs		Death		DALYs		Death	
			Number	Rate	Number	Rate	Number	Rate	Number	Rate	Number	Rate	Number	Rate
		Female	2515 (1221 to 4409)	318.7 (167.4 to 537.3)	31 (16 to 53)	4.4 (2.5 to 7)	259 (204 to 315)	40.9 (32.4 to 49.4)	5 (4 to 6)	0.8 (0.6 to 0.9)	-89.7 (-94.6 to -79.3)	-87.2 (-93 to -75.5)	-84.2 (-91.2 to -69.5)	-82.2 (-89.4 to -69)
		Male	3284 (1536 to 5626)	433.4 (209.1 to 704.1)	43 (21 to 72)	6.7 (3.1 to 10.2)	565 (373 to 730)	79.2 (54.2 to 101.5)	12 (7 to 15)	1.7 (1.1 to 2.1)	-82.8 (-90.3 to -65.4)	-81.7 (-89.3 to -65.9)	-73.3 (-84.5 to -49.8)	-74.6 (-84.5 to -56.1)
	Exposure to mechanical forces	Both	3234 (2060 to 5181)	287.4 (193.3 to 448.4)	38 (22 to 67)	3.7 (2.2 to 6.6)	1983 (1549 to 2582)	145.6 (114.8 to 187.4)	24 (17 to 34)	1.8 (1.3 to 2.5)	-38.7 (-62.8 to -6.6)	-49.3 (-66 to -27.2)	-38 (-63.8 to 7.5)	-51.6 (-70.4 to -17.9)
		Female	818 (432 to 1783)	133.8 (83 to 241.1)	8 (4 to 20)	1.4 (0.8 to 2.7)	452 (351 to 594)	70 (54 to 92.4)	4 (3 to 5)	0.6 (0.5 to 0.9)	-44.8 (-75.4 to 5.3)	-47.7 (-72.2 to -16.8)	-53.3 (-81.1 to 12.3)	-55.3 (-78.1 to -12.9)
		Male	2416 (1442 to 3942)	435.3 (280.7 to 705.1)	30 (16 to 55)	5.9 (3.3 to 11.4)	1531 (1167 to 2051)	218.3 (169.3 to 287.2)	20 (14 to 30)	2.9 (2.1 to 4.3)	-36.6 (-61.2 to 4.5)	-49.9 (-67.2 to -23.4)	-33.8 (-61 to 23)	-50.4 (-71.2 to -12.3)
	Adverse effects of medical treatment	Both	2368 (1235 to 3370)	194.9 (107.7 to 277.9)	37 (21 to 54)	4.3 (2.4 to 6.5)	721 (478 to 922)	56.9 (38.6 to 71.5)	20 (14 to 26)	1.8 (1.2 to 2.2)	-69.6 (-78.5 to -52)	-70.8 (-79.4 to -56.9)	-45.2 (-61.9 to -18.9)	-59.4 (-74.1 to -39)
		Female	957 (457 to 1442)	156 (89.1 to 222.3)	14 (8 to 21)	3.4 (1.8 to 5.4)	252 (170 to 313)	40.7 (27.9 to 50.4)	7 (5 to 9)	1.2 (0.8 to 1.5)	-73.7 (-82.9 to -50.2)	-73.9 (-81.7 to -60)	-51.2 (-67 to -24.8)	-64 (-78.8 to -42.5)
		Male	1411 (676 to 2191)	231.6 (112.5 to 365.1)	23 (11 to 36)	5.3 (2.5 to 8.6)	469 (274 to 644)	73.1 (44.2 to 97.4)	13 (8 to 18)	2.3 (1.4 to 3.1)	-66.8 (-78.1 to -47.9)	-68.4 (-79.7 to -49.2)	-41.3 (-63.4 to -5.3)	-55.6 (-73.5 to -25.4)
	Animal contact	Both	292 (178 to 463)	20.5 (13.7 to 31.7)	3 (2 to 5)	0.2 (0.1 to 0.4)	111 (80 to 155)	8.5 (6.2 to 12)	1 (0 to 2)	0.1 (0 to 0.1)	-62.1 (-75.5 to -39.4)	-58.7 (-71.1 to -39.9)	-77.9 (-86.5 to -57.6)	-73.3 (-82.7 to -54.5)
		Female	126 (62 to 206)	18.5 (10.7 to 28.1)	1 (0 to 2)	0.2 (0.1 to 0.3)	55 (39 to 77)	8.5 (6.1 to 11.9)	0 (0 to 1)	0 (0 to 0.1)	-56.5 (-74.2 to -8.3)	-53.8 (-69.4 to -22.8)	-80.9 (-90.6 to -41.9)	-76.1 (-87.3 to -47.3)
		Male	166 (90 to 289)	22.4 (13.5 to 36.2)	2 (1 to 3)	0.3 (0.1 to 0.5)	56 (41 to 83)	8.4 (6.3 to 12.8)	0 (0 to 1)	0.1 (0.1 to 0.2)	-66.4 (-79.4 to -37.6)	-62.4 (-75.4 to -38.5)	-75.9 (-87.4 to -45.7)	-70.9 (-83.2 to -44.5)
	Foreign body	Both	4763 (2966 to 7678)	285.9 (182.4 to 453.3)	54 (33 to 87)	3.3 (2.2 to 5.2)	673 (551 to 807)	55.7 (45.5 to 67)	8 (7 to 10)	0.7 (0.6 to 0.9)	-85.9 (-91.7 to -76.4)	-80.5 (-88.4 to -67.9)	-84.8 (-90.9 to -73.8)	-78.4 (-86.9 to -64.8)
		Female	2460 (1290 to 4660)	298.1 (162.2 to 553.7)	28 (14 to 52)	3.4 (1.9 to 6.3)	259 (203 to 326)	45 (34.4 to 58.2)	3 (2 to 4)	0.5 (0.4 to 0.7)	-89.5 (-94.8 to -78.1)	-84.9 (-92.4 to -69.5)	-89.5 (-94.7 to -78.5)	-84.3 (-91.9 to -69.4)
		Male	2303 (1048 to 3890)	274.3 (135.8 to 450.1)	26 (12 to 44)	3.2 (1.7 to 5.3)	414 (326 to 509)	65.9 (51.1 to 82.5)	5 (4 to 6)	0.9 (0.7 to 1.1)	-82 (-90 to -65)	-76 (-86.6 to -55.9)	-79.8 (-88.8 to -60)	-72.2 (-84 to -48.2)
	Environmental heat and cold exposure	Both	227 (170 to 294)	21.5 (16.5 to 28.3)	2 (1 to 3)	0.2 (0.1 to 0.4)	170 (123 to 228)	12.6 (9.1 to 17)	1 (1 to 2)	0.1 (0.1 to 0.1)	-24.8 (-41.8 to -3.5)	-41.4 (-52.4 to -29.8)	-48.4 (-63.4 to -25.8)	-63.2 (-73.7 to -46.9)
		Female	89 (62 to 125)	17.6 (12.9 to 23.8)	0 (0 to 1)	0.1 (0.1 to 0.3)	78 (56 to 109)	11.8 (8.5 to 16.3)	0 (0 to 0)	0 (0 to 0.1)	-11.9 (-36.8 to 12.3)	-33.2 (-47.2 to -22.3)	-49.2 (-68.4 to -19.7)	-61.4 (-73.4 to -38.6)
		Male	137 (95 to 186)	25.2 (18 to 34.5)	1 (1 to 2)	0.3 (0.2 to 0.6)	92 (67 to 122)	13.3 (9.8 to 17.8)	1 (0 to 1)	0.1 (0.1 to 0.2)	-33.1 (-50.7 to -7.9)	-47.1 (-58.9 to -33)	-48.1 (-65.8 to -20.2)	-63.1 (-75.4 to -44.5)
	Exposure to forces of nature	Both	36 (31 to 40)	3.2 (2.8 to 3.7)	0 (0 to 0)	0 (0 to 0)	468 (320 to 681)	31.8 (22.1 to 45.8)	0 (0 to 0)	0 (0 to 0)	1215.2 (886.8 to 1638.9)	878.7 (644.1 to 1170.2)	-81.9 (-81.9 to -81.9)	-86.3 (-86.3 to -86.3)
		Female	13 (12 to 15)	2.4 (2.1 to 2.8)	0 (0 to 0)	0 (0 to 0)	156 (105 to 233)	21.5 (14.5 to 31.6)	0 (0 to 0)	0 (0 to 0)	1061.1 (741.6 to 1475.4)	779.9 (549.8 to 1065.4)	-82.4 (-82.4 to -82.4)	-86.6 (-86.6 to -86.6)
		Male	22 (19 to 25)	4 (3.5 to 4.6)	0 (0 to 0)	0.1 (0 to 0.1)	312 (217 to 451)	42 (29.6 to 59.8)	0 (0 to 0)	0 (0 to 0)	1308.8 (958.2 to 1743.4)	940.5 (699.6 to 1235.6)	-81.6 (-81.6 to -81.6)	-86.2 (-86.2 to -86.2)
	Other unintentional injuries	Both	3237 (2329 to 4577)	256.9 (190.1 to 352.9)	35 (24 to 53)	2.8 (2 to 4.3)	1591 (1230 to 2008)	116.1 (90.2 to 145.9)	16 (12 to 20)	1.2 (0.9 to 1.5)	-50.9 (-67.8 to -27.8)	-54.8 (-69.4 to -37.6)	-54.4 (-74 to -23.5)	-58.1 (-75.9 to -30.5)

Province	Cause	Sex	1990				2019				1990-2019 percent change (%)			
			DALYs		Death		DALYs		Death		DALYs		Death	
			Number	Rate	Number	Rate	Number	Rate	Number	Rate	Number	Rate	Number	Rate
		Female	1148 (725 to 1739)	175.9 (120.5 to 250.6)	11 (6 to 19)	1.6 (1 to 2.5)	471 (336 to 643)	71.5 (51.5 to 97)	2 (2 to 3)	0.4 (0.3 to 0.5)	-58.9 (-76.6 to -31.6)	-59.4 (-74.1 to -39.3)	-78 (-88 to -55.4)	-76.1 (-86.4 to -55.4)
		Male	2089 (1409 to 3221)	336 (237.6 to 501.7)	24 (15 to 40)	4 (2.6 to 6.9)	1119 (860 to 1394)	158.2 (123.2 to 195.9)	14 (10 to 18)	1.9 (1.4 to 2.5)	-46.4 (-68.5 to -16.4)	-52.9 (-72.2 to -30.2)	-43.3 (-71.1 to 5.2)	-51.3 (-76.4 to -13.3)
	Self-harm and interpersonal violence	Both	13174 (10334 to 15758)	1219.6 (973.4 to 1452.6)	148 (108 to 178)	14.8 (11 to 17.8)	8815 (7328 to 10390)	614.9 (508.7 to 721.2)	125 (99 to 149)	9 (7 to 10.6)	-33.1 (-44.6 to -14.6)	-49.6 (-58.7 to -36.8)	-15.6 (-35.1 to 19.3)	-39.2 (-53.4 to -15.4)
		Female	4579 (3225 to 5998)	853 (619 to 1090.4)	44 (26 to 59)	8.8 (5.7 to 11.5)	2592 (2162 to 3160)	373.7 (315.9 to 448.6)	29 (24 to 35)	4.4 (3.6 to 5.3)	-43.4 (-54.5 to -22.1)	-56.2 (-64.5 to -41.4)	-34.3 (-53.2 to 14.4)	-50.5 (-64.3 to -22.5)
		Male	8595 (6845 to 10217)	1572.8 (1240.6 to 1875.8)	104 (77 to 128)	20.5 (15.2 to 25.5)	6223 (4751 to 7459)	843.2 (637.9 to 1008.5)	96 (70 to 118)	13.4 (9.8 to 16.4)	-27.6 (-42.9 to -6.4)	-46.4 (-58.5 to -31)	-7.6 (-32.4 to 30.2)	-34.5 (-53.1 to -6.6)
	Self-harm	Both	4486 (2566 to 5731)	430.6 (264.3 to 549.8)	75 (44 to 96)	8 (5.2 to 10.2)	4166 (2881 to 5154)	292.8 (201.6 to 361.4)	81 (56 to 99)	5.8 (4 to 7.1)	-7.1 (-33.5 to 66.5)	-32 (-52.2 to 11)	8.3 (-22.3 to 86.6)	-27.7 (-48.8 to 13.5)
		Female	2065 (1068 to 2796)	392.1 (223.9 to 525.5)	33 (17 to 45)	6.7 (4 to 9.2)	1014 (799 to 1256)	151.7 (120.7 to 186.3)	18 (14 to 23)	2.7 (2.2 to 3.4)	-50.9 (-67 to -7.7)	-61.3 (-73.1 to -33.7)	-43.7 (-62.1 to 6.3)	-59.1 (-71.9 to -33.3)
		Male	2421 (1303 to 3414)	467.4 (283.5 to 650.2)	42 (24 to 59)	9.2 (5.7 to 13.1)	3151 (1844 to 4082)	425.7 (244.2 to 550.9)	62 (37 to 80)	8.7 (5.1 to 11)	30.2 (-13.6 to 140.1)	-8.9 (-43.3 to 61.6)	48.7 (-1.7 to 165)	-5.1 (-40.8 to 63.5)
	Interpersonal violence	Both	2655 (1822 to 3432)	242 (169.1 to 307.2)	35 (22 to 48)	3.5 (2.2 to 4.7)	2648 (2219 to 3158)	191.4 (160 to 227.2)	37 (30 to 46)	2.7 (2.2 to 3.3)	-0.3 (-27.4 to 47.5)	-20.9 (-41.2 to 12.2)	5.4 (-27.5 to 69.3)	-23.8 (-47 to 20.4)
		Female	941 (631 to 1292)	158.8 (110.5 to 210)	10 (6 to 15)	1.8 (1.1 to 2.5)	865 (693 to 1066)	130.5 (104.8 to 159.7)	10 (7 to 12)	1.5 (1.1 to 1.9)	-8.1 (-36.7 to 38.9)	-17.8 (-40.4 to 19.8)	-6.5 (-41.3 to 62.8)	-20.1 (-46.8 to 34.6)
		Male	1714 (1051 to 2413)	323.1 (200.5 to 439)	25 (14 to 36)	5.2 (2.9 to 7.4)	1783 (1449 to 2209)	249.1 (201.7 to 307.3)	28 (22 to 36)	3.9 (3 to 5)	4 (-28.2 to 72.5)	-22.9 (-46.7 to 22.5)	10.3 (-28.9 to 98.6)	-25.1 (-51.4 to 33)
	Conflict and terrorism	Both	5553 (4238 to 7527)	500.3 (380 to 673.7)	32 (29 to 35)	2.5 (2.3 to 2.8)	1513 (984 to 2390)	96.9 (63.5 to 150.3)	0 (0 to 1)	0 (0 to 0)	-72.7 (-77.5 to -67.5)	-80.6 (-84.2 to -76.5)	-98.4 (-98.4 to -98.4)	-98.5 (-98.5 to -98.5)
		Female	1479 (888 to 2468)	284.2 (178.1 to 453.5)	0 (0 to 0)	0 (0 to 0)	636 (382 to 1095)	80.1 (49 to 135.3)	0 (0 to 0)	0 (0 to 0)	-57 (-63.4 to -50.7)	-71.8 (-77.2 to -66.5)	35.7 (35.7 to 35.7)	11.6 (11.6 to 11.6)
		Male	4074 (3327 to 5075)	707.1 (573.1 to 899.5)	31 (29 to 35)	4.9 (4.5 to 5.4)	877 (595 to 1302)	113.9 (77.2 to 168.8)	0 (0 to 0)	0.1 (0 to 0.1)	-78.5 (-82.7 to -73.7)	-83.9 (-87.1 to -80.5)	-98.8 (-98.8 to -98.8)	-99 (-99 to -99)
	Executions and police conflict	Both	480 (421 to 553)	46.7 (40.6 to 54)	7 (6 to 8)	0.8 (0.7 to 0.9)	488 (407 to 604)	33.7 (28.4 to 41.1)	7 (6 to 9)	0.5 (0.4 to 0.6)	1.7 (-15 to 23.2)	-27.8 (-40 to -12.8)	-2.6 (-20.8 to 24.2)	-36.2 (-47.8 to -19.4)
		Female	93 (82 to 106)	18 (15.8 to 20.6)	1 (1 to 1)	0.3 (0.3 to 0.3)	76 (65 to 91)	11.4 (9.8 to 13.5)	1 (1 to 1)	0.2 (0.1 to 0.2)	-17.9 (-23.8 to -9.9)	-36.6 (-41.1 to -31.4)	-19.7 (-21 to -18.2)	-45.6 (-46.2 to -44.8)
		Male	387 (336 to 455)	75.2 (64.8 to 88.6)	6 (5 to 7)	1.2 (1.1 to 1.5)	411 (338 to 519)	54.6 (45.4 to 67.6)	6 (5 to 8)	0.8 (0.7 to 1)	6.4 (-14 to 32.1)	-27.4 (-41.8 to -10.1)	1 (-21 to 33.7)	-35.6 (-49.4 to -15.3)
**Bushehr**	**All causes**	Both	276766 (239976 to 320553)	42337 (37723.6 to 47310)	3732 (3254 to 4252)	993 (886.6 to 1103.5)	262390 (227772 to 302154)	25350.7 (22333.8 to 28754.3)	4652 (4315 to 5053)	651.5 (608.2 to 704.9)	-5.2 (-18.8 to 9.8)	-40.1 (-46.8 to -33.2)	24.7 (7.3 to 46.3)	-34.4 (-42.2 to -24.5)
		Female	127328 (109366 to 146989)	39044.9 (34389.5 to 44118.2)	1592 (1377 to 1831)	854.4 (750.2 to 964.7)	120484 (102085 to 141460)	23856 (20551.6 to 27686.7)	1888 (1728 to 2045)	546.4 (505.2 to 586.8)	-5.4 (-19.8 to 9.6)	-38.9 (-46 to -31.8)	18.6 (0.2 to 39.6)	-36.1 (-44.3 to -25.8)
		Male	149438 (129370 to 173451)	45475.9 (39665.1 to 51696.1)	2140 (1828 to 2477)	1131.2 (962.3 to 1309.6)	141906 (123922 to 161710)	26898.7 (23819.7 to 30223.8)	2765 (2493 to 3079)	757.7 (689 to 840.8)	-5 (-19.4 to 11.7)	-40.9 (-48.8 to -32)	29.2 (7.8 to 57.4)	-33 (-44.3 to -18)
	**Communicable, maternal, neonatal, and nutritional diseases**	Both	108576 (84553 to 143707)	9959.4 (7798.7 to 13795.6)	1226 (942 to 1706)	134.2 (105.6 to 224.7)	26398 (22425 to 30896)	2444.1 (2075.4 to 2862.9)	297 (256 to 342)	35.7 (31.1 to 40.7)	-75.7 (-82.2 to -67.6)	-75.5 (-83 to -67.4)	-75.8 (-83.5 to -67)	-73.4 (-84.3 to -64.9)

Province	Cause	Sex	1990				2019				1990-2019 percent change (%)			
			DALYs		Death		DALYs		Death		DALYs		Death	
			Number	Rate	Number	Rate	Number	Rate	Number	Rate	Number	Rate	Number	Rate
		Female	50909 (40282 to 66978)	9583.1 (7625.9 to 12924)	571 (447 to 781)	125.1 (100.2 to 191)	11885 (10029 to 13996)	2264.5 (1920 to 2675.7)	125 (105 to 148)	30.1 (25.4 to 35.5)	-76.7 (-83 to -68.7)	-76.4 (-83.5 to -68.5)	-78.2 (-84.8 to -70.5)	-75.9 (-84.3 to -68.2)
		Male	57667 (43822 to 77242)	10310.7 (7882.7 to 14888.5)	656 (494 to 937)	143.4 (109.9 to 267.1)	14514 (12236 to 17036)	2619.2 (2218.3 to 3075.3)	172 (147 to 199)	41.3 (35.8 to 47.3)	-74.8 (-82 to -66)	-74.6 (-82.8 to -65.2)	-73.8 (-82.4 to -63.5)	-71.2 (-84.2 to -60.6)
	HIV/AIDS and sexually transmitted infections	Both	239 (129 to 440)	29.8 (18.9 to 47.8)	2 (1 to 5)	0.3 (0.2 to 0.5)	1011 (741 to 1381)	77.7 (56.4 to 105.9)	16 (11 to 23)	1.2 (0.9 to 1.8)	322.1 (128.7 to 701.6)	161.1 (64.8 to 316.1)	603.3 (224.8 to 1578.5)	358.2 (141.6 to 790.2)
		Female	114 (62 to 208)	29.9 (17.9 to 49.2)	1 (0 to 2)	0.2 (0.1 to 0.4)	391 (254 to 559)	65.8 (42.2 to 94.8)	5 (3 to 8)	0.9 (0.5 to 1.5)	241.5 (85.4 to 535.8)	120.2 (36.6 to 270.1)	491.6 (136.4 to 1557.2)	368.3 (110.2 to 930)
		Male	125 (66 to 236)	29.6 (18.5 to 47.8)	1 (1 to 3)	0.3 (0.2 to 0.6)	620 (485 to 860)	87.8 (67.9 to 119.9)	10 (8 to 15)	1.5 (1.1 to 2.2)	395.9 (164.9 to 872.9)	196.2 (82.4 to 384.5)	678.3 (276.7 to 1622.6)	342.5 (148.2 to 776.1)
	HIV/AIDS	Both	36 (17 to 50)	6.3 (2.9 to 9)	1 (0 to 1)	0.1 (0 to 0.2)	786 (568 to 1118)	59.3 (42.1 to 84.3)	14 (10 to 22)	1.1 (0.8 to 1.7)	2110.8 (1158.3 to 4768)	833.8 (429.9 to 2053.2)	2532.4 (1286.2 to 7705.1)	895.1 (409.7 to 3030.2)
		Female	9 (4 to 13)	2.6 (1.3 to 3.7)	0 (0 to 0)	0 (0 to 0.1)	262 (147 to 395)	44.1 (24.4 to 66.9)	5 (2 to 8)	0.8 (0.4 to 1.3)	2923.1 (1326.7 to 6529.9)	1588 (704.7 to 3618.6)	3706.2 (1514.8 to 10075.3)	1745 (677.8 to 5439)
		Male	27 (13 to 38)	10 (4.3 to 14.2)	0 (0 to 1)	0.2 (0.1 to 0.3)	524 (395 to 750)	72.1 (53.5 to 103.2)	10 (7 to 14)	1.4 (1 to 2.1)	1848.9 (1072.5 to 4312.3)	620 (333.5 to 1634.9)	2190.7 (1171.9 to 7418.9)	666.4 (328.2 to 2458)
	Sexually transmitted infections excluding HIV	Both	204 (96 to 407)	23.4 (13.1 to 41.7)	2 (1 to 4)	0.2 (0.1 to 0.3)	225 (138 to 364)	18.5 (10.9 to 30.8)	1 (1 to 2)	0.1 (0.1 to 0.2)	10.3 (-25.4 to 71)	-21.1 (-38.7 to -3)	-25.4 (-50.6 to 26)	-16.1 (-43.2 to 27.1)
		Female	106 (53 to 200)	27.3 (15.4 to 46.6)	1 (0 to 2)	0.2 (0.1 to 0.3)	129 (77 to 208)	21.7 (12.9 to 36.4)	1 (0 to 1)	0.1 (0.1 to 0.2)	21.7 (-20.4 to 77.8)	-20.3 (-36.4 to -5)	-22.9 (-50.1 to 33)	-16.1 (-44.6 to 25.9)
		Male	98 (41 to 205)	19.6 (9.9 to 37.1)	1 (0 to 2)	0.2 (0.1 to 0.4)	96 (55 to 159)	15.7 (8.5 to 27.2)	1 (0 to 1)	0.1 (0.1 to 0.3)	-1.9 (-36 to 62.8)	-20 (-42.7 to 6.2)	-27.4 (-53.6 to 25.9)	-15.6 (-48.9 to 39.2)
	Respiratory infections and tuberculosis	Both	12061 (8486 to 16646)	1322.3 (1005.5 to 1695.2)	152 (110 to 202)	28.6 (23.7 to 35)	4560 (3832 to 5531)	461.3 (396.3 to 546)	107 (94 to 122)	16.4 (14.2 to 19)	-62.2 (-73.9 to -44.4)	-65.1 (-73.7 to -52.7)	-29.5 (-48.7 to -0.4)	-42.9 (-53 to -27.6)
		Female	6088 (3942 to 8851)	1292.8 (922.7 to 1751.4)	74 (50 to 104)	25.3 (19.6 to 32.4)	1922 (1554 to 2392)	395.2 (325.8 to 483.1)	42 (36 to 52)	13 (10.9 to 16.3)	-68.4 (-79.4 to -49.8)	-69.4 (-78.3 to -55.6)	-42.7 (-61.7 to -12.6)	-48.6 (-60.8 to -32.5)
		Male	5973 (4096 to 8505)	1355.1 (1034.4 to 1751.7)	78 (56 to 107)	32.5 (25.8 to 41.4)	2638 (2220 to 3169)	526.8 (450.2 to 622.3)	65 (56 to 76)	19.8 (16.7 to 23.8)	-55.8 (-70.3 to -34.1)	-61.1 (-71.8 to -46.8)	-16.9 (-42.2 to 19.7)	-39 (-53 to -18.5)
	Tuberculosis	Both	758 (554 to 933)	139.4 (103.3 to 171.4)	15 (11 to 18)	4.5 (3.2 to 5.6)	273 (240 to 311)	26.3 (23.3 to 29.8)	7 (7 to 9)	1 (0.8 to 1.1)	-64 (-72.1 to -49.5)	-81.1 (-85.1 to -73.3)	-49.3 (-61 to -25.3)	-78.6 (-83.6 to -68.3)
		Female	322 (173 to 451)	110.4 (60 to 158)	6 (3 to 8)	3.3 (1.6 to 5)	97 (81 to 122)	19.7 (16.5 to 25.2)	3 (2 to 4)	0.7 (0.6 to 0.9)	-69.9 (-79.4 to -34.3)	-82.2 (-87.9 to -60.4)	-53.6 (-69.9 to 17.7)	-78.9 (-86.7 to -45.3)
		Male	436 (326 to 551)	167.9 (124 to 210.8)	9 (7 to 12)	5.7 (4.2 to 7.4)	176 (150 to 204)	32.6 (28.1 to 37.5)	5 (4 to 6)	1.2 (1 to 1.4)	-59.6 (-69.6 to -43.6)	-80.6 (-85.2 to -73.4)	-46.5 (-60.3 to -24.9)	-78.7 (-84 to -69.2)
	Lower respiratory infections	Both	10354 (6891 to 15062)	1065.4 (765.7 to 1431.7)	137 (96 to 187)	24.2 (19.6 to 29.9)	2854 (2456 to 3317)	321 (278.5 to 372.1)	99 (87 to 115)	15.4 (13.3 to 17.9)	-72.4 (-82 to -56.5)	-69.9 (-78.6 to -56.2)	-27.4 (-48.5 to 5.7)	-36.3 (-49.2 to -18.6)
		Female	5318 (3253 to 8048)	1069.6 (717.2 to 1515)	68 (45 to 98)	22 (17 to 29)	1176 (968 to 1443)	266.5 (221.5 to 326.3)	40 (34 to 49)	12.3 (10.3 to 15.5)	-77.9 (-86.5 to -61.1)	-75.1 (-83.8 to -59.7)	-41.8 (-62.4 to -8.8)	-44.1 (-58.3 to -25.1)

Province	Cause	Sex	1990				2019				1990-2019 percent change (%)			
			DALYs		Death		DALYs		Death		DALYs		Death	
			Number	Rate	Number	Rate	Number	Rate	Number	Rate	Number	Rate	Number	Rate
		Male	5035 (3245 to 7561)	1065.1 (762 to 1477)	69 (48 to 97)	26.8 (21.2 to 34.8)	1678 (1424 to 1974)	376 (321.2 to 442.4)	60 (51 to 70)	18.6 (15.6 to 22.5)	-66.7 (-79 to -45.9)	-64.7 (-75.9 to -48.7)	-13 (-41.1 to 29.2)	-30.7 (-46.9 to -7.8)
	Upper respiratory infections	Both	677 (405 to 1085)	84.7 (51.1 to 132.8)	0 (0 to 0)	0 (0 to 0)	1075 (650 to 1698)	85.2 (51.7 to 134.1)	0 (0 to 0)	0 (0 to 0)	58.7 (46.9 to 72)	0.6 (-4.8 to 6)	104 (-89.1 to 3967.5)	81.9 (-90.3 to 2303.7)
		Female	327 (196 to 520)	82.8 (50.1 to 129.7)	0 (0 to 0)	0 (0 to 0)	496 (296 to 780)	83.2 (50.3 to 131.8)	0 (0 to 0)	0 (0 to 0)	51.5 (37.7 to 67.2)	0.5 (-6.8 to 8.7)	106.1 (-93.1 to 5675.8)	105.1 (-92.7 to 3025.4)
		Male	350 (206 to 559)	86.6 (51.6 to 136.1)	0 (0 to 0)	0 (0 to 0)	579 (348 to 911)	87.1 (52.7 to 137)	0 (0 to 0)	0 (0 to 0)	65.4 (50.5 to 83.3)	0.6 (-6.7 to 8.2)	102.8 (-86.6 to 3573)	71.2 (-89.2 to 2211.4)
	Otitis media	Both	272 (154 to 441)	32.9 (18.8 to 54.3)	0 (0 to 0)	0 (0 to 0)	358 (202 to 589)	28.7 (16.2 to 47.1)	0 (0 to 0)	0 (0 to 0)	31.5 (19.3 to 45.4)	-12.8 (-18.6 to -6.5)	47.4 (-75.2 to 343.7)	-50.1 (-85.5 to 74.2)
		Female	121 (68 to 198)	30 (16.9 to 48.7)	0 (0 to 0)	0 (0 to 0)	153 (87 to 255)	25.9 (14.9 to 42.8)	0 (0 to 0)	0 (0 to 0)	27.3 (13.6 to 43.6)	-13.7 (-21.6 to -5)	179.2 (-62.2 to 883.1)	-38 (-84 to 167.3)
		Male	152 (86 to 251)	35.6 (20.2 to 58.6)	0 (0 to 0)	0 (0 to 0)	205 (114 to 345)	31.2 (17.5 to 52.1)	0 (0 to 0)	0 (0 to 0)	34.8 (19.4 to 52.8)	-12.4 (-20.1 to -4.4)	-71.6 (-87.5 to -14.9)	-78.8 (-90 to -40.4)
	Enteric infections	Both	6845 (4440 to 10362)	668.6 (450.9 to 963.6)	72 (45 to 113)	9.7 (6 to 14)	3013 (2270 to 3920)	270.3 (206 to 348.4)	16 (11 to 21)	2 (1.4 to 2.8)	-56 (-72.7 to -31.2)	-59.6 (-73.7 to -40.2)	-78.5 (-87.2 to -62.7)	-79.7 (-85.9 to -67.6)
		Female	3288 (1882 to 5333)	651.6 (400.8 to 1011.3)	35 (18 to 58)	9.6 (5 to 15.8)	1397 (1037 to 1866)	259.7 (194.5 to 340.8)	7 (4 to 11)	1.7 (0.9 to 3)	-57.5 (-75.1 to -22.1)	-60.1 (-75.3 to -34.4)	-81.2 (-91 to -60.8)	-82.7 (-90 to -67.6)
		Male	3557 (2171 to 5947)	684.1 (439.6 to 1078.8)	38 (21 to 65)	9.7 (5.5 to 15.3)	1615 (1208 to 2109)	280.8 (211.7 to 361.3)	9 (6 to 13)	2.3 (1.4 to 3.6)	-54.6 (-74.5 to -23.9)	-59 (-75.7 to -36.2)	-76 (-87.3 to -55.7)	-76.5 (-85.6 to -62.4)
	Diarrheal diseases	Both	6317 (3969 to 9901)	614.5 (406.2 to 915)	65 (39 to 104)	8.9 (5.4 to 13.1)	2781 (2045 to 3674)	251.4 (187.7 to 326.8)	12 (8 to 17)	1.7 (1.1 to 2.6)	-56 (-73.3 to -29.5)	-59.1 (-73.9 to -38.6)	-81.5 (-89.6 to -65.9)	-81.2 (-87.2 to -68.9)
		Female	3032 (1653 to 5083)	598.5 (352.6 to 954.4)	31 (15 to 54)	8.8 (4.2 to 15)	1295 (941 to 1739)	242.3 (177.3 to 322.7)	5 (3 to 9)	1.4 (0.7 to 2.7)	-57.3 (-75.8 to -18.4)	-59.5 (-75.6 to -30.6)	-84 (-93.1 to -64)	-84.2 (-91.5 to -68.7)
		Male	3285 (1918 to 5682)	629.1 (387.3 to 1012.7)	34 (18 to 62)	8.9 (4.8 to 14.4)	1485 (1087 to 1957)	260.4 (192.5 to 338.7)	7 (4 to 11)	2 (1.2 to 3.3)	-54.8 (-75.4 to -21.7)	-58.6 (-75.9 to -33.7)	-79.1 (-89.4 to -58.2)	-77.8 (-86.8 to -62.7)
	Typhoid and paratyphoid	Both	400 (141 to 815)	41.4 (15 to 84.1)	5 (2 to 11)	0.6 (0.2 to 1.2)	168 (71 to 326)	13.5 (5.6 to 26.2)	2 (1 to 5)	0.2 (0.1 to 0.4)	-58 (-69.4 to -32.2)	-67.3 (-76 to -47.1)	-55.1 (-66.9 to -25.4)	-67.6 (-75.4 to -48.5)
		Female	181 (62 to 373)	38.1 (13.4 to 78.1)	2 (1 to 5)	0.6 (0.2 to 1.1)	74 (30 to 144)	12.5 (5.1 to 24.1)	1 (0 to 2)	0.2 (0.1 to 0.3)	-59.1 (-71.8 to -29.4)	-67.3 (-77.6 to -43.6)	-56.5 (-69.4 to -22.2)	-68 (-77.6 to -45.8)
		Male	220 (79 to 457)	44.5 (16.2 to 90.6)	3 (1 to 6)	0.6 (0.2 to 1.3)	94 (39 to 186)	14.5 (5.9 to 28.6)	1 (1 to 3)	0.2 (0.1 to 0.4)	-57.1 (-69.1 to -29.9)	-67.3 (-76 to -48)	-54 (-66.6 to -23.9)	-67.3 (-75.6 to -49.1)
	Invasive Non-typhoidal Salmonella (iNTS)	Both	107 (52 to 183)	11 (5.3 to 18.3)	1 (1 to 2)	0.2 (0.1 to 0.3)	59 (30 to 102)	5 (2.6 to 8.6)	1 (1 to 2)	0.1 (0 to 0.2)	-44.5 (-65.2 to -10.3)	-54.3 (-69.9 to -29.3)	-31.8 (-54.3 to 3.5)	-51.2 (-64 to -31.8)
		Female	63 (29 to 109)	12.9 (6.2 to 21.8)	1 (0 to 1)	0.2 (0.1 to 0.4)	26 (13 to 45)	4.5 (2.3 to 7.9)	0 (0 to 1)	0.1 (0 to 0.1)	-59.1 (-75.4 to -30.3)	-65.1 (-77.9 to -45.1)	-48.3 (-67.3 to -15.1)	-61.9 (-73.3 to -44.1)
		Male	44 (22 to 77)	9.1 (4.6 to 15.3)	1 (0 to 1)	0.2 (0.1 to 0.3)	34 (17 to 60)	5.5 (2.7 to 9.7)	1 (0 to 1)	0.1 (0.1 to 0.2)	-23.7 (-52.6 to 28.3)	-39.6 (-60 to -1.5)	-9.1 (-38.5 to 42.2)	-36.7 (-54.4 to -7.9)
	Other intestinal infectious diseases	Both	20 (4 to 46)	1.8 (0.5 to 3.9)	0 (0 to 1)	0 (0 to 0)	5 (3 to 7)	0.4 (0.3 to 0.6)	0 (0 to 0)	0 (0 to 0)	-76.8 (-90.9 to 17.9)	-77.9 (-91.1 to -16.6)	-81.8 (-93.8 to 5.6)	-81.1 (-92.8 to -36.3)
		Female	12 (1 to 35)	2.2 (0.3 to 6)	0 (0 to 0)	0 (0 to 0.1)	2 (1 to 4)	0.4 (0.2 to 0.7)	0 (0 to 0)	0 (0 to 0)	-80.9 (-94.4 to 96)	-81 (-94.2 to 40.2)	-84.6 (-96.3 to 164.3)	-83.1 (-95.6 to 5)
		Male	8 (1 to 21)	1.4 (0.3 to 3.6)	0 (0 to 0)	0 (0 to 0)	2 (1 to 4)	0.4 (0.2 to 0.6)	0 (0 to 0)	0 (0 to 0)	-70.5 (-89.9 to 80)	-73.5 (-90.5 to 16.4)	-77.4 (-94.2 to 97.9)	-78.4 (-93.3 to -10.5)

Province	Cause	Sex	1990				2019				1990-2019 percent change (%)			
			DALYs		Death		DALYs		Death		DALYs		Death	
			Number	Rate	Number	Rate	Number	Rate	Number	Rate	Number	Rate	Number	Rate
	Neglected tropical diseases and malaria	Both	3726 (308 to 35102)	512.2 (36.9 to 4969.5)	58 (1 to 593)	10.8 (0.2 to 115.6)	205 (142 to 294)	17.4 (12.3 to 24.7)	1 (1 to 2)	0.1 (0.1 to 0.2)	-94.5 (-99.4 to -35.1)	-96.6 (-99.7 to -54.4)	-98.1 (-99.8 to -26)	-98.9 (-99.9 to -52.1)
		Female	1841 (152 to 17396)	465.2 (38.7 to 4519)	26 (1 to 267)	8.2 (0.2 to 87.6)	97 (65 to 140)	17.2 (11.5 to 24.8)	0 (0 to 1)	0.1 (0.1 to 0.1)	-94.7 (-99.5 to -37.4)	-96.3 (-99.6 to -56.3)	-98.4 (-99.9 to -22.2)	-98.9 (-99.9 to -50.1)
		Male	1885 (139 to 17835)	556.8 (32.1 to 5533)	32 (1 to 333)	13.3 (0.3 to 141)	108 (68 to 172)	17.7 (11.2 to 27.7)	1 (0 to 1)	0.2 (0.1 to 0.2)	-94.3 (-99.4 to -23.1)	-96.8 (-99.7 to -45.8)	-97.9 (-99.8 to -14.3)	-98.9 (-99.9 to -45.7)
	Malaria	Both	3313 (27 to 34853)	465.2 (3.2 to 4927.4)	55 (0 to 590)	10.4 (0 to 115.1)	15 (10 to 20)	1.1 (0.8 to 1.6)	0 (0 to 0)	0 (0 to 0)	-99.6 (-100 to -47.7)	-99.8 (-100 to -66.6)	-100 (-100 to -56.1)	-100 (-100 to -75)
		Female	1649 (16 to 17176)	419 (4 to 4462)	25 (0 to 266)	7.9 (0 to 87.3)	7 (5 to 10)	1.2 (0.8 to 1.6)	0 (0 to 0)	0 (0 to 0)	-99.6 (-100 to -56.4)	-99.7 (-100 to -70.9)	-100 (-100 to -60.1)	-100 (-100 to -74.7)
		Male	1664 (11 to 17661)	509.1 (2.4 to 5484)	31 (0 to 330)	12.8 (0 to 140.5)	8 (5 to 10)	1.1 (0.8 to 1.5)	0 (0 to 0)	0 (0 to 0)	-99.5 (-100 to -33.6)	-99.8 (-100 to -56.5)	-100 (-100 to -52.1)	-100 (-100 to -74.6)
	Leishmaniasis	Both	84 (1 to 891)	8.9 (0.1 to 88.1)	1 (0 to 12)	0.1 (0 to 1.3)	6 (0 to 51)	0.5 (0 to 4.1)	0 (0 to 1)	0 (0 to 0.1)	-92.8 (-94.5 to 38.5)	-94.5 (-95.9 to -5.8)	-93 (-94 to -87.5)	-95.4 (-96.3 to -93.7)
		Female	29 (0 to 281)	6.1 (0.1 to 55.9)	0 (0 to 4)	0.1 (0 to 0.8)	2 (0 to 15)	0.4 (0 to 2.6)	0 (0 to 0)	0 (0 to 0)	-92.4 (-95.1 to 83.8)	-93.9 (-96 to 23.3)	-93.6 (-94.8 to -87.8)	-95.6 (-96.6 to -93.8)
		Male	55 (0 to 593)	11.6 (0.1 to 116.8)	1 (0 to 8)	0.2 (0 to 1.8)	4 (0 to 36)	0.6 (0 to 5.5)	0 (0 to 1)	0 (0 to 0.1)	-92.9 (-94.3 to 3.4)	-94.8 (-96 to -25.6)	-92.7 (-93.7 to -87)	-95.3 (-96.4 to -93.5)
	Schistosomiasis	Both	0 (0 to 0)	0 (0 to 0)	0 (0 to 0)	0 (0 to 0)	0 (0 to 0)	0 (0 to 0)	0 (0 to 0)	0 (0 to 0)	0 (0 to 0)	0 (0 to 0)	0 (0 to 0)	0 (0 to 0)
		Female	0 (0 to 0)	0 (0 to 0)	0 (0 to 0)	0 (0 to 0)	0 (0 to 0)	0 (0 to 0)	0 (0 to 0)	0 (0 to 0)	0 (0 to 0)	0 (0 to 0)	0 (0 to 0)	0 (0 to 0)
		Male	0 (0 to 0)	0 (0 to 0)	0 (0 to 0)	0 (0 to 0)	0 (0 to 0)	0 (0 to 0)	0 (0 to 0)	0 (0 to 0)	0 (0 to 0)	0 (0 to 0)	0 (0 to 0)	0 (0 to 0)
	Cystic echinococcosis	Both	40 (22 to 62)	6.2 (4 to 8.7)	1 (0 to 1)	0.1 (0.1 to 0.2)	33 (22 to 46)	2.7 (1.8 to 3.7)	0 (0 to 0)	0 (0 to 0)	-19.1 (-53.4 to 52.7)	-56.9 (-72.6 to -30.9)	-61.4 (-82.2 to -12.5)	-78.2 (-89.9 to -53.5)
		Female	19 (9 to 34)	5.9 (3.3 to 9.1)	0 (0 to 0)	0.1 (0 to 0.2)	17 (11 to 25)	2.9 (1.9 to 4.2)	0 (0 to 0)	0 (0 to 0)	-11.1 (-58.3 to 94.3)	-49.9 (-71.6 to -8.2)	-58.1 (-87.3 to 49.3)	-74 (-92.4 to -18.6)
		Male	21 (10 to 37)	6.6 (3.6 to 10.2)	0 (0 to 1)	0.1 (0.1 to 0.2)	16 (10 to 22)	2.5 (1.6 to 3.5)	0 (0 to 0)	0 (0 to 0)	-26.3 (-63.6 to 67.6)	-62.6 (-79.2 to -29.6)	-63.9 (-87.6 to 6)	-81.2 (-94 to -49.3)
	Trachoma	Both	4 (2 to 7)	1.9 (1.1 to 3.1)	--	--	2 (1 to 4)	0.3 (0.2 to 0.6)	--	--	-45 (-60 to -27.3)	-82.7 (-87.3 to -77.5)	--	--
		Female	3 (2 to 6)	3 (1.7 to 4.8)	--	--	2 (1 to 3)	0.4 (0.2 to 0.8)	--	--	-50.4 (-66 to -29.8)	-84.9 (-89.4 to -79)	--	--
		Male	1 (0 to 2)	0.8 (0.4 to 1.4)	--	--	1 (0 to 1)	0.2 (0.1 to 0.4)	--	--	-26.7 (-50.5 to 15)	-75.7 (-83.8 to -63.8)	--	--
	Rabies	Both	0 (0 to 3)	0.1 (0 to 0.4)	0 (0 to 0)	0 (0 to 0)	0 (0 to 1)	0 (0 to 0.1)	0 (0 to 0)	0 (0 to 0)	-45.8 (-70.9 to 9.8)	-72.2 (-86.9 to -49.4)	-32.8 (-68.2 to 23)	-69.9 (-86.1 to -47.4)
		Female	0 (0 to 0)	0 (0 to 0.1)	0 (0 to 0)	0 (0 to 0)	0 (0 to 0)	0 (0 to 0)	0 (0 to 0)	0 (0 to 0)	-40.7 (-70.9 to 36.5)	-75 (-87.9 to -48.7)	-38.1 (-68.9 to 28.7)	-76.4 (-88.9 to -50.8)
		Male	0 (0 to 2)	0.1 (0 to 0.7)	0 (0 to 0)	0 (0 to 0)	0 (0 to 1)	0 (0 to 0.2)	0 (0 to 0)	0 (0 to 0)	-47.6 (-81 to 28.1)	-71.4 (-90.1 to -39.4)	-30.3 (-76.2 to 47.7)	-66.1 (-88.3 to -35.7)
	Intestinal nematode infections	Both	59 (28 to 106)	6.3 (3.4 to 10.7)	0 (0 to 1)	0 (0 to 0.1)	13 (7 to 22)	1 (0.6 to 1.8)	0 (0 to 0)	0 (0 to 0)	-78.3 (-90 to -53.2)	-83.6 (-91.6 to -69.4)	-91 (-96.9 to -62.1)	-89.9 (-96.3 to -68.9)
		Female	34 (14 to 66)	7.8 (3.7 to 13.7)	0 (0 to 1)	0 (0 to 0.1)	8 (4 to 14)	1.3 (0.7 to 2.3)	0 (0 to 0)	0 (0 to 0)	-77.1 (-90.3 to -41.3)	-83 (-92 to -62.8)	-91.6 (-98.1 to 28.8)	-89.9 (-97.6 to -32)

Province	Cause	Sex	1990				2019				1990-2019 percent change (%)			
			DALYs		Death		DALYs		Death		DALYs		Death	
			Number	Rate	Number	Rate	Number	Rate	Number	Rate	Number	Rate	Number	Rate
		Male	24 (8 to 55)	4.8 (2 to 9.7)	0 (0 to 1)	0 (0 to 0.1)	5 (3 to 9)	0.8 (0.4 to 1.4)	0 (0 to 0)	0 (0 to 0)	-79.9 (-92.5 to -39.5)	-83.9 (-93.5 to -61.4)	-90.4 (-97.8 to -16.3)	-90 (-97.4 to -54.4)
	Food-borne trematodiases	Both	2 (1 to 5)	0.5 (0.1 to 1)	--	--	12 (3 to 26)	1 (0.2 to 2.1)	--	--	412.2 (279.4 to 566.8)	109.5 (60.1 to 168.1)	--	--
		Female	1 (0 to 2)	0.4 (0.1 to 0.8)	--	--	5 (1 to 9)	0.8 (0.2 to 1.7)	--	--	369.5 (254.7 to 506.8)	100.6 (49.3 to 160.4)	--	--
		Male	1 (0 to 3)	0.6 (0.1 to 1.1)	--	--	8 (2 to 17)	1.2 (0.3 to 2.6)	--	--	441.9 (240.3 to 681)	113.6 (45.5 to 195.1)	--	--
	Leprosy	Both	0 (0 to 0)	0 (0 to 0.1)	--	--	0 (0 to 1)	0 (0 to 0)	--	--	110.5 (92 to 128.1)	-26.9 (-32.5 to -21)	--	--
		Female	0 (0 to 0)	0 (0 to 0)	--	--	0 (0 to 0)	0 (0 to 0)	--	--	87 (61.5 to 111.9)	-32.8 (-40.9 to -24.4)	--	--
		Male	0 (0 to 0)	0.1 (0 to 0.1)	--	--	0 (0 to 0)	0 (0 to 0.1)	--	--	123 (95.6 to 147.6)	-24.2 (-32.2 to -16.2)	--	--
	Other neglected tropical diseases	Both	222 (142 to 335)	23.1 (15.1 to 33.6)	1 (0 to 1)	0.1 (0.1 to 0.2)	124 (74 to 195)	10.7 (6.5 to 16.4)	1 (0 to 1)	0.1 (0 to 0.1)	-44.1 (-63.8 to -14.4)	-53.6 (-69 to -32.5)	-1.6 (-49.8 to 67.8)	-33.5 (-59.8 to 4)
		Female	105 (59 to 167)	22.9 (14.1 to 34.8)	0 (0 to 1)	0.1 (0 to 0.2)	56 (32 to 93)	10.1 (5.9 to 16.1)	0 (0 to 0)	0.1 (0 to 0.1)	-46.2 (-68.8 to -9.9)	-56.1 (-72.9 to -30.4)	-7.7 (-48.7 to 88.9)	-40.1 (-63.8 to 11.1)
		Male	117 (62 to 199)	23.1 (13.2 to 37)	0 (0 to 1)	0.2 (0.1 to 0.2)	67 (34 to 122)	11.4 (5.8 to 19.8)	0 (0 to 1)	0.1 (0 to 0.2)	-42.3 (-69.9 to 6)	-50.9 (-73.1 to -16.9)	2.1 (-53.1 to 100.2)	-29.2 (-58.3 to 30.8)
	Other infectious diseases	Both	7809 (4243 to 13751)	741.2 (438.4 to 1232.1)	94 (53 to 163)	11.3 (7.3 to 17.6)	1243 (1068 to 1490)	113.4 (97.5 to 135.5)	22 (19 to 25)	2.6 (2.1 to 3)	-84.1 (-90.9 to -71.1)	-84.7 (-90.8 to -73.8)	-76.7 (-86.6 to -57.3)	-77.3 (-85.3 to -63.4)
		Female	4297 (2295 to 7745)	820.1 (466 to 1411.1)	51 (27 to 91)	12.2 (7.5 to 19.2)	567 (470 to 705)	106.1 (88.2 to 131.5)	9 (7 to 11)	2.1 (1.7 to 2.5)	-86.8 (-92.8 to -75)	-87.1 (-92.5 to -76.9)	-82.8 (-90.4 to -67.6)	-83 (-89.4 to -71.1)
		Male	3512 (1910 to 6248)	664.9 (397.6 to 1122.7)	43 (24 to 75)	10.5 (6.7 to 16.4)	676 (573 to 815)	120.8 (102.4 to 145.1)	13 (11 to 16)	3.1 (2.4 to 3.6)	-80.8 (-88.8 to -65)	-81.8 (-89.1 to -69)	-69.4 (-82.7 to -43.9)	-70.6 (-81.9 to -52.5)
	Meningitis	Both	1523 (1073 to 2104)	150.7 (110.6 to 198.4)	18 (13 to 25)	2.4 (1.8 to 3.1)	379 (314 to 455)	34.1 (28.3 to 40.6)	6 (6 to 8)	0.7 (0.6 to 0.9)	-75.1 (-83.2 to -62.1)	-77.4 (-84.2 to -66.9)	-64.9 (-75.6 to -47.2)	-68.5 (-76.8 to -55.4)
		Female	808 (525 to 1185)	160.6 (111.9 to 225.6)	10 (6 to 14)	2.4 (1.7 to 3.4)	185 (148 to 226)	34.3 (27.6 to 41.7)	3 (2 to 3)	0.6 (0.5 to 0.8)	-77.1 (-85.6 to -63.1)	-78.7 (-86 to -67)	-71.1 (-81.5 to -54.1)	-73.2 (-82.1 to -60.9)
		Male	715 (480 to 1052)	141.2 (99.5 to 196.4)	9 (6 to 13)	2.3 (1.7 to 3)	194 (157 to 241)	34 (27.5 to 42.3)	4 (3 to 5)	0.8 (0.7 to 1.1)	-72.9 (-83 to -55.4)	-75.9 (-83.8 to -61.6)	-58.2 (-72.5 to -30.3)	-63.7 (-74.6 to -42.3)
	Encephalitis	Both	132 (89 to 199)	15.2 (10.9 to 23)	1 (1 to 2)	0.2 (0.2 to 0.4)	131 (104 to 173)	11.5 (9.2 to 14.7)	2 (2 to 3)	0.2 (0.2 to 0.3)	-0.3 (-34.1 to 51.3)	-24.3 (-45.1 to 8.1)	29 (-14.6 to 105.2)	-12.7 (-41.1 to 29)
		Female	83 (53 to 132)	19 (13.2 to 27)	1 (1 to 1)	0.3 (0.2 to 0.4)	74 (57 to 93)	13.4 (10.2 to 17.1)	1 (1 to 1)	0.2 (0.2 to 0.3)	-11.4 (-50 to 46.3)	-29.1 (-55.9 to 8.9)	10.9 (-36.5 to 90.1)	-22.2 (-50.5 to 22)
		Male	48 (29 to 103)	11.5 (7.6 to 23.5)	1 (0 to 1)	0.2 (0.1 to 0.4)	57 (43 to 99)	9.6 (7.2 to 16.5)	1 (1 to 2)	0.2 (0.1 to 0.3)	18.9 (-23.4 to 86.2)	-16 (-43.1 to 23)	61 (-3 to 184.1)	4.1 (-34.6 to 71.5)
	Diphtheria	Both	9 (3 to 16)	0.8 (0.3 to 1.4)	0 (0 to 0)	0 (0 to 0)	1 (0 to 1)	0.1 (0 to 0.1)	0 (0 to 0)	0 (0 to 0)	-91.6 (-96.7 to -75.4)	-92.2 (-96.9 to -79.3)	-89.6 (-95.6 to -71.6)	-91 (-96 to -78.4)
		Female	4 (1 to 10)	0.8 (0.2 to 1.8)	0 (0 to 0)	0 (0 to 0)	0 (0 to 1)	0.1 (0 to 0.1)	0 (0 to 0)	0 (0 to 0)	-93.1 (-98.1 to -66.6)	-93.3 (-98.1 to -69.9)	-91.7 (-97.5 to -63.5)	-92.3 (-97.7 to -70.7)

Province	Cause	Sex	1990				2019				1990-2019 percent change (%)			
			DALYs		Death		DALYs		Death		DALYs		Death	
			Number	Rate	Number	Rate	Number	Rate	Number	Rate	Number	Rate	Number	Rate
		Male	4 (1 to 9)	0.7 (0.3 to 1.6)	0 (0 to 0)	0 (0 to 0)	0 (0 to 1)	0.1 (0 to 0.1)	0 (0 to 0)	0 (0 to 0)	-90.1 (-96.8 to -61.3)	-91.2 (-97 to -69.7)	-87.5 (-95.7 to -56)	-89.9 (-96.2 to -70.2)
	Whooping cough	Both	1269 (69 to 5141)	104.3 (5.6 to 422.9)	14 (0 to 59)	1.2 (0 to 4.9)	46 (3 to 194)	4.1 (0.3 to 17.6)	1 (0 to 2)	0 (0 to 0.2)	-96.4 (-99.8 to -8.2)	-96 (-99.8 to 0.3)	-96.5 (-99.9 to 40.5)	-96.1 (-99.9 to 54.4)
		Female	744 (39 to 3082)	125 (6.6 to 518)	8 (0 to 35)	1.4 (0 to 6)	25 (2 to 109)	4.7 (0.4 to 20.2)	0 (0 to 1)	0.1 (0 to 0.2)	-96.6 (-99.8 to -1)	-96.2 (-99.8 to 10)	-96.6 (-99.9 to 37.3)	-96.3 (-99.9 to 51.9)
		Male	525 (29 to 2229)	84.5 (4.7 to 358.8)	6 (0 to 26)	1 (0 to 4.1)	20 (2 to 89)	3.5 (0.3 to 15.6)	0 (0 to 1)	0 (0 to 0.2)	-96.1 (-99.8 to -3)	-95.8 (-99.8 to 5.7)	-96.2 (-99.9 to 46.7)	-95.9 (-99.9 to 59.5)
	Tetanus	Both	363 (98 to 1133)	32.8 (11.3 to 96.1)	4 (1 to 13)	0.5 (0.2 to 1.2)	14 (10 to 22)	1.3 (0.8 to 2.1)	0 (0 to 0)	0 (0 to 0)	-96.2 (-98.9 to -82.1)	-96.1 (-98.9 to -85.3)	-94.2 (-98.3 to -78.8)	-94.4 (-98.2 to -85.7)
		Female	256 (52 to 918)	46.5 (12.3 to 159.1)	3 (1 to 11)	0.7 (0.2 to 2)	8 (5 to 13)	1.4 (0.9 to 2.6)	0 (0 to 0)	0 (0 to 0)	-97.1 (-99.3 to -80.3)	-96.9 (-99.2 to -84.8)	-95.5 (-98.9 to -77.8)	-95.5 (-98.7 to -84.9)
		Male	107 (29 to 358)	19.6 (5.9 to 61)	1 (0 to 4)	0.3 (0.1 to 0.8)	6 (4 to 11)	1.1 (0.7 to 1.9)	0 (0 to 0)	0 (0 to 0)	-94 (-98.5 to -72.3)	-94.3 (-98.5 to -76.5)	-91 (-97.8 to -63.8)	-92 (-97.7 to -70.4)
	Measles	Both	3514 (1105 to 7908)	288.6 (90.6 to 649.5)	41 (13 to 92)	3.4 (1.1 to 7.6)	52 (15 to 132)	4.6 (1.3 to 11.6)	1 (0 to 2)	0.1 (0 to 0.1)	-98.5 (-99.4 to -96.8)	-98.4 (-99.4 to -96.5)	-98.6 (-99.4 to -96.8)	-98.5 (-99.4 to -96.6)
		Female	1865 (569 to 4475)	313.2 (95.3 to 753.3)	22 (7 to 52)	3.6 (1.1 to 8.8)	26 (7 to 65)	4.7 (1.3 to 11.7)	0 (0 to 1)	0.1 (0 to 0.1)	-98.6 (-99.4 to -96.8)	-98.5 (-99.4 to -96.6)	-98.6 (-99.5 to -96.9)	-98.5 (-99.4 to -96.6)
		Male	1649 (523 to 3726)	265 (84 to 598.8)	19 (6 to 43)	3.1 (1 to 7)	26 (8 to 67)	4.5 (1.3 to 11.5)	0 (0 to 1)	0.1 (0 to 0.1)	-98.4 (-99.4 to -96.6)	-98.3 (-99.3 to -96.3)	-98.4 (-99.4 to -96.6)	-98.3 (-99.4 to -96.3)
	Varicella and herpes zoster	Both	76 (44 to 123)	9.7 (6.5 to 13.8)	1 (1 to 1)	0.2 (0.2 to 0.3)	47 (32 to 67)	4.4 (3.1 to 6.1)	0 (0 to 1)	0.1 (0 to 0.1)	-38.2 (-66.7 to 12.3)	-54.9 (-71.7 to -29.7)	-54.2 (-75.1 to -12.2)	-72.3 (-83.1 to -52.7)
		Female	39 (18 to 75)	10 (6 to 16)	0 (0 to 1)	0.2 (0.1 to 0.4)	23 (15 to 34)	4.4 (3 to 6.5)	0 (0 to 0)	0.1 (0 to 0.1)	-42.3 (-73.7 to 32.8)	-55.9 (-76.6 to -24.1)	-55.9 (-80.5 to 8.3)	-71.6 (-86 to -40.6)
		Male	37 (18 to 67)	9.4 (6 to 14.7)	0 (0 to 1)	0.2 (0.1 to 0.4)	24 (16 to 36)	4.3 (2.8 to 6.4)	0 (0 to 0)	0.1 (0 to 0.1)	-33.7 (-66.8 to 40.5)	-53.9 (-72.8 to -21.6)	-52.3 (-77.8 to 5.2)	-73.4 (-87.8 to -44)
	Acute hepatitis	Both	642 (374 to 974)	105.2 (62.5 to 156.8)	12 (7 to 18)	2.9 (1.6 to 4.7)	239 (199 to 284)	22.1 (18.5 to 26.1)	6 (5 to 7)	0.6 (0.5 to 0.8)	-62.8 (-76.8 to -33.2)	-79 (-86.5 to -62.1)	-51.8 (-70.1 to -8.6)	-78.1 (-87.6 to -57.3)
		Female	364 (172 to 579)	111.4 (51.5 to 165.3)	6 (3 to 9)	3 (1.2 to 4.7)	82 (66 to 100)	15.3 (12.3 to 18.6)	2 (1 to 2)	0.4 (0.3 to 0.5)	-77.6 (-86.6 to -49.5)	-86.2 (-91.3 to -67.8)	-72.9 (-83.2 to -32.3)	-86.9 (-92.9 to -64.8)
		Male	278 (155 to 506)	98.6 (58.6 to 175)	6 (3 to 10)	2.8 (1.6 to 5.2)	157 (128 to 195)	28.6 (23.4 to 35.4)	4 (3 to 5)	0.9 (0.7 to 1.1)	-43.4 (-70.4 to 6.5)	-71 (-84.6 to -48.3)	-28.5 (-64.4 to 33.6)	-68.7 (-84.5 to -42.1)
	Other unspecified infectious diseases	Both	281 (163 to 618)	34.1 (20.7 to 68.2)	2 (1 to 7)	0.5 (0.2 to 1.3)	335 (227 to 432)	31.4 (20.4 to 40.5)	6 (3 to 8)	0.8 (0.4 to 1)	18.9 (-58.2 to 112.5)	-8.1 (-64.7 to 59.5)	158.1 (-40.4 to 569.1)	49.4 (-56.1 to 282.7)
		Female	131 (77 to 291)	33.7 (20.3 to 65.4)	1 (0 to 3)	0.5 (0.2 to 1.4)	145 (98 to 192)	27.7 (18.5 to 36.6)	2 (1 to 3)	0.6 (0.3 to 0.8)	10.3 (-61 to 92.3)	-17.8 (-65.9 to 39.4)	125.9 (-44.5 to 507)	15.6 (-63.1 to 235.2)
		Male	150 (80 to 353)	34.4 (19.9 to 76.1)	1 (1 to 4)	0.5 (0.3 to 1.3)	190 (116 to 262)	35 (20.2 to 48.1)	4 (2 to 5)	1 (0.4 to 1.3)	26.4 (-59.4 to 160.3)	1.7 (-66.3 to 96.9)	184 (-40.1 to 660.4)	86.6 (-54.6 to 349.3)
	Maternal and neonatal disorders	Both	72188 (56382 to 89092)	6081.5 (4783.4 to 7499.3)	809 (630 to 1000)	68.3 (53.4 to 84)	14408 (11472 to 17735)	1332.5 (1055.4 to 1651.3)	129 (99 to 165)	12.4 (9.4 to 15.9)	-80 (-85.5 to -72.9)	-78.1 (-84.3 to -70.2)	-84 (-89 to -77.4)	-81.9 (-87.5 to -74.2)
		Female	32152 (25147 to 39894)	5638.4 (4414.8 to 6980.3)	362 (282 to 451)	64.1 (50 to 79.8)	6512 (5235 to 7996)	1237.3 (987.5 to 1525.9)	58 (45 to 75)	11.4 (8.7 to 14.7)	-79.7 (-85.6 to -72.3)	-78.1 (-84.5 to -70.3)	-83.9 (-88.9 to -77.3)	-82.1 (-87.7 to -74.9)
		Male	40035 (30576 to 50662)	6495.9 (4962.1 to 8211.7)	447 (341 to 566)	72.2 (55.1 to 91.6)	7896 (6168 to 9885)	1424.4 (1108.9 to 1797.7)	71 (52 to 92)	13.3 (9.9 to 17.4)	-80.3 (-85.9 to -71.9)	-78.1 (-84.4 to -68.7)	-84.2 (-89.2 to -76.7)	-81.5 (-87.4 to -72.8)

Province	Cause	Sex	1990				2019				1990-2019 percent change (%)			
			DALYs		Death		DALYs		Death		DALYs		Death	
			Number	Rate	Number	Rate	Number	Rate	Number	Rate	Number	Rate	Number	Rate
	Maternal disorders	Female	1091 (589 to 1528)	341.4 (195.4 to 476.6)	16 (8 to 23)	5.4 (2.9 to 7.8)	293 (231 to 362)	43.2 (34 to 53.4)	4 (3 to 5)	0.5 (0.4 to 0.7)	-73.1 (-81.9 to -49.6)	-87.4 (-91.5 to -77.2)	-77.6 (-85.7 to -53.9)	-90.2 (-93.7 to -81)
	Neonatal disorders	Both	71097 (55613 to 88019)	5911.4 (4634.4 to 7310.3)	792 (616 to 982)	65.6 (51 to 81.3)	14115 (11189 to 17424)	1312.6 (1035.4 to 1633)	125 (95 to 162)	12.1 (9.2 to 15.7)	-80.1 (-85.6 to -72.9)	-77.8 (-84.2 to -69.6)	-84.2 (-89.1 to -77.4)	-81.5 (-87.2 to -73.6)
		Female	31061 (24013 to 38805)	5297 (4104.6 to 6604)	346 (267 to 434)	58.6 (45.3 to 73.6)	6219 (4931 to 7647)	1194.1 (943.5 to 1478.1)	55 (41 to 71)	10.9 (8.2 to 14.1)	-80 (-86 to -72.5)	-77.5 (-84.3 to -68.8)	-84.2 (-89.4 to -77.3)	-81.4 (-87.5 to -73.4)
		Male	40035 (30576 to 50662)	6495.9 (4962.1 to 8211.7)	447 (341 to 566)	72.2 (55.1 to 91.6)	7896 (6168 to 9885)	1424.4 (1108.9 to 1797.7)	71 (52 to 92)	13.3 (9.9 to 17.4)	-80.3 (-85.9 to -71.9)	-78.1 (-84.4 to -68.7)	-84.2 (-89.2 to -76.7)	-81.5 (-87.4 to -72.8)
	Nutritional deficiencies	Both	5708 (4162 to 7768)	603.8 (448.1 to 799.9)	39 (25 to 56)	5.2 (3.8 to 7.1)	1958 (1302 to 2905)	171.5 (114 to 249.1)	7 (5 to 8)	1 (0.9 to 1.2)	-65.7 (-76.6 to -50.9)	-71.6 (-79.8 to -61.2)	-83.2 (-88.9 to -73)	-79.9 (-86.4 to -71.2)
		Female	3128 (2161 to 4500)	685.1 (486.6 to 952.3)	22 (13 to 36)	5.6 (3.4 to 9.2)	999 (622 to 1549)	183.3 (115.6 to 279)	3 (2 to 4)	0.9 (0.7 to 1.2)	-68.1 (-80.5 to -50.2)	-73.2 (-82.5 to -60.3)	-86.9 (-92.9 to -75.9)	-83.5 (-91.4 to -70.9)
		Male	2580 (1678 to 3771)	524.2 (360.9 to 734.2)	17 (9 to 27)	4.9 (3.1 to 6.8)	959 (569 to 1563)	160.8 (99.1 to 254.7)	4 (3 to 4)	1.2 (0.9 to 1.4)	-62.8 (-77.2 to -39.4)	-69.3 (-80.4 to -51.6)	-78.2 (-87.5 to -57.5)	-76 (-84.8 to -60.2)
	Protein-energy malnutrition	Both	3297 (2169 to 4763)	306.4 (208.4 to 434.6)	37 (24 to 54)	4.8 (3.5 to 6.7)	479 (340 to 635)	43.8 (31.9 to 56.9)	4 (4 to 5)	0.7 (0.6 to 0.8)	-85.5 (-91 to -76)	-85.7 (-90.7 to -77.3)	-88.6 (-92.7 to -81.5)	-85.7 (-90.5 to -78.8)
		Female	1882 (1123 to 3016)	350 (216.9 to 549.8)	21 (12 to 35)	5.2 (3.2 to 8.7)	219 (152 to 296)	41.7 (29.9 to 55.6)	2 (2 to 3)	0.6 (0.5 to 0.9)	-88.4 (-93.6 to -78.7)	-88.1 (-93.3 to -79.1)	-90.7 (-94.9 to -82.7)	-87.7 (-93.6 to -77.7)
		Male	1415 (829 to 2289)	264.9 (162.3 to 407)	16 (9 to 26)	4.5 (2.8 to 6.3)	260 (182 to 354)	45.7 (32.8 to 60.5)	2 (2 to 3)	0.7 (0.6 to 0.9)	-81.6 (-89.6 to -66)	-82.7 (-89.7 to -70.1)	-85.7 (-92.1 to -73.5)	-83.5 (-90.2 to -73.5)
	Iodine deficiency	Both	95 (59 to 142)	15 (9.4 to 22)	--	--	79 (44 to 126)	6.3 (3.5 to 10.1)	--	--	-17.1 (-39.3 to 9.9)	-58.2 (-68.7 to -46.6)	--	--
		Female	58 (36 to 88)	18.5 (11.4 to 27.7)	--	--	47 (26 to 76)	8 (4.5 to 12.6)	--	--	-17.9 (-43.6 to 15.8)	-56.6 (-69.4 to -41.8)	--	--
		Male	37 (22 to 55)	11.6 (7.3 to 17.2)	--	--	31 (18 to 51)	4.7 (2.7 to 7.6)	--	--	-15.8 (-39 to 13.8)	-59.4 (-70.1 to -47.5)	--	--
	Vitamin A deficiency	Both	136 (68 to 244)	11.2 (5.6 to 20)	--	--	8 (3 to 17)	0.7 (0.3 to 1.5)	--	--	-94.4 (-97.7 to -87.4)	-94.1 (-97.6 to -86.7)	--	--
		Female	50 (23 to 91)	8.4 (3.9 to 15.2)	--	--	3 (1 to 8)	0.6 (0.2 to 1.4)	--	--	-93.3 (-97.8 to -83.7)	-93 (-97.7 to -83.2)	--	--
		Male	86 (33 to 170)	13.8 (5.4 to 27)	--	--	4 (1 to 12)	0.7 (0.2 to 2)	--	--	-95 (-98.8 to -83.7)	-94.7 (-98.7 to -83.2)	--	--
	Dietary iron deficiency	Both	2030 (1168 to 3218)	250.5 (156.2 to 382.9)	--	--	1228 (679 to 2056)	105.7 (58.9 to 174.6)	--	--	-39.5 (-61.6 to -8.3)	-57.8 (-72.1 to -38.5)	--	--
		Female	1065 (579 to 1708)	288.8 (171 to 439.8)	--	--	656 (341 to 1157)	119.3 (63.4 to 206.6)	--	--	-38.4 (-64.5 to 3.2)	-58.7 (-74.3 to -34.8)	--	--
		Male	965 (475 to 1801)	211.6 (113.6 to 361.9)	--	--	572 (253 to 1123)	93.1 (42.1 to 183)	--	--	-40.7 (-71.8 to 17.6)	-56 (-78.3 to -18.4)	--	--
	Other nutritional deficiencies	Both	150 (98 to 232)	20.8 (14.7 to 29.6)	1 (1 to 3)	0.4 (0.2 to 0.6)	165 (121 to 216)	15.1 (11.5 to 19.3)	2 (2 to 3)	0.4 (0.3 to 0.4)	10.1 (-31.7 to 69.4)	-27.4 (-49.1 to 2.9)	50.9 (-11.4 to 185.7)	-5.1 (-41.2 to 70.7)
		Female	73 (45 to 126)	19.4 (12.5 to 30.9)	1 (0 to 1)	0.3 (0.2 to 0.6)	73 (51 to 99)	13.7 (9.8 to 18)	1 (1 to 1)	0.3 (0.2 to 0.4)	0.1 (-43.8 to 72.2)	-29.4 (-58.4 to 13.4)	24.1 (-46.2 to 181.4)	-16.9 (-62.4 to 85.9)
		Male	77 (46 to 128)	22.2 (14.5 to 35.2)	1 (0 to 1)	0.4 (0.2 to 0.7)	92 (65 to 126)	16.5 (12.2 to 22.1)	1 (1 to 2)	0.4 (0.3 to 0.6)	19.7 (-32.8 to 102.8)	-25.8 (-53.7 to 15.2)	76.4 (-12.5 to 371.8)	3.5 (-46 to 126.8)

Province	Cause	Sex	1990				2019				1990-2019 percent change (%)			
			DALYs		Death		DALYs		Death		DALYs		Death	
			Number	Rate	Number	Rate	Number	Rate	Number	Rate	Number	Rate	Number	Rate
	**Non-communicable diseases**	Both	123449 (101241 to 145355)	26125 (22248.3 to 29851.6)	1866 (1539 to 2163)	749.7 (653.6 to 841.4)	201494 (171144 to 236466)	20100.1 (17511.3 to 23035)	3775 (3493 to 4127)	562.8 (524.8 to 610.3)	63.2 (38.3 to 94.4)	-23.1 (-32 to -12.5)	102.3 (70.6 to 151.8)	-24.9 (-34.4 to -12.1)
		Female	60912 (49167 to 72847)	25431.1 (21636.2 to 29460)	814 (659 to 966)	663.7 (567.6 to 758.6)	99232 (82624 to 118233)	19957.2 (16948.4 to 23426.9)	1618 (1476 to 1757)	487.8 (450.8 to 523.8)	62.9 (39.1 to 92.6)	-21.5 (-30.1 to -10.7)	98.7 (64.7 to 150.3)	-26.5 (-37 to -12.6)
		Male	62537 (49937 to 74937)	26747.8 (22189.7 to 31171)	1052 (822 to 1279)	836.1 (680.6 to 983.3)	102262 (87514 to 119254)	20418.4 (17825.3 to 23259.7)	2157 (1933 to 2417)	640.3 (579.1 to 714.5)	63.5 (33.8 to 102.5)	-23.7 (-35.5 to -9.5)	105 (62.8 to 166)	-23.4 (-37.6 to -3.4)
	Neoplasms	Both	8998 (6809 to 10764)	2320.1 (1837.7 to 2756.8)	250 (197 to 299)	91.8 (73.7 to 110.1)	21087 (18579 to 23565)	2210.8 (1956.1 to 2470.7)	742 (658 to 832)	94 (83.5 to 105.2)	134.3 (89.4 to 218.4)	-4.7 (-22.1 to 24)	196.1 (142.7 to 291.5)	2.5 (-15.4 to 32)
		Female	4292 (2960 to 5482)	2113.2 (1560.5 to 2673.8)	110 (80 to 139)	77 (58.6 to 99.6)	9766 (8467 to 11016)	1976 (1730.6 to 2220.3)	316 (278 to 356)	75.6 (66.7 to 84.8)	127.5 (75.7 to 225.2)	-6.5 (-26.9 to 27.1)	187.3 (125 to 290.6)	-1.8 (-24 to 29.5)
		Male	4706 (3320 to 5974)	2523.4 (1840.6 to 3175.7)	140 (102 to 177)	107.7 (80.3 to 134.4)	11320 (9654 to 13001)	2479.3 (2134.3 to 2848.3)	425 (366 to 490)	114 (98.6 to 130.8)	140.5 (77.2 to 245.2)	-1.7 (-25.1 to 38.1)	203 (128.5 to 327.4)	5.8 (-17.1 to 45.1)
	Lip and oral cavity cancer	Both	50 (36 to 64)	15.7 (11.7 to 20)	2 (1 to 2)	0.7 (0.5 to 0.8)	145 (126 to 166)	15.6 (13.5 to 17.9)	5 (5 to 6)	0.7 (0.6 to 0.8)	188 (111.3 to 331)	-1 (-26.7 to 48.1)	224.2 (137.5 to 387.3)	5.9 (-21.7 to 54.5)
		Female	17 (12 to 22)	10.9 (7.8 to 14)	1 (0 to 1)	0.5 (0.3 to 0.6)	59 (50 to 68)	12.8 (10.9 to 14.8)	2 (2 to 3)	0.6 (0.5 to 0.7)	245.9 (148.9 to 415.5)	17.5 (-15.3 to 70.3)	298.9 (188.3 to 488.1)	25 (-8 to 81.5)
		Male	33 (22 to 46)	20.4 (13.8 to 27.7)	1 (1 to 2)	0.9 (0.6 to 1.2)	86 (69 to 106)	18.4 (14.9 to 22.5)	3 (3 to 4)	0.8 (0.7 to 1)	158.5 (70.7 to 324)	-9.5 (-39.1 to 44.2)	187.3 (92.1 to 362.8)	-3.6 (-33.9 to 50)
	Nasopharynx cancer	Both	31 (22 to 39)	8.1 (6 to 10.1)	1 (1 to 1)	0.3 (0.2 to 0.4)	50 (42 to 58)	4.8 (4.1 to 5.7)	2 (1 to 2)	0.2 (0.2 to 0.2)	59.1 (20.4 to 137.7)	-40.1 (-55.1 to -15.6)	78 (33.3 to 151.1)	-38.7 (-54.4 to -15.3)
		Female	15 (9 to 20)	7.7 (5.2 to 10.8)	0 (0 to 1)	0.3 (0.2 to 0.4)	20 (16 to 24)	4 (3.3 to 4.9)	1 (0 to 1)	0.1 (0.1 to 0.2)	34.9 (-6.3 to 133.7)	-48.3 (-65.1 to -15.3)	49.1 (-1.2 to 141.9)	-49.2 (-68.2 to -21.3)
		Male	17 (12 to 22)	8.4 (5.9 to 11.1)	0 (0 to 1)	0.3 (0.2 to 0.4)	30 (24 to 38)	5.7 (4.6 to 7.1)	1 (1 to 1)	0.2 (0.2 to 0.3)	79.9 (21.3 to 181.4)	-32.5 (-54.4 to 1.2)	102.4 (35.6 to 205.2)	-28.3 (-50.1 to 5.2)
	Other pharynx cancer	Both	23 (17 to 29)	7.4 (5.5 to 9.3)	1 (1 to 1)	0.3 (0.2 to 0.4)	53 (45 to 62)	5.7 (4.8 to 6.7)	2 (2 to 2)	0.3 (0.2 to 0.3)	127.2 (67.7 to 228.9)	-22.9 (-41.8 to 12.2)	153 (90.5 to 265.1)	-18.3 (-40.1 to 18.6)
		Female	11 (8 to 15)	7.2 (5 to 10.3)	0 (0 to 1)	0.3 (0.2 to 0.5)	21 (17 to 25)	4.5 (3.7 to 5.4)	1 (1 to 1)	0.2 (0.2 to 0.2)	85.2 (27 to 197.5)	-38.3 (-59.1 to -3)	107.2 (37.2 to 230)	-35.3 (-59.7 to 1.1)
		Male	12 (8 to 16)	7.5 (5.1 to 10)	0 (0 to 1)	0.3 (0.2 to 0.4)	32 (25 to 40)	7 (5.4 to 8.7)	1 (1 to 2)	0.3 (0.2 to 0.4)	166.6 (73.5 to 315.7)	-7 (-38.5 to 43.2)	193.9 (93.3 to 356.8)	-0.4 (-33.3 to 52.7)
	Esophageal cancer	Both	170 (117 to 330)	58.7 (42 to 113.1)	6 (4 to 12)	2.7 (1.9 to 5.2)	398 (300 to 880)	45.5 (34.3 to 99.4)	16 (12 to 36)	2.2 (1.7 to 4.7)	134.9 (67.4 to 236)	-22.4 (-44.4 to 12.7)	163.4 (90.2 to 278)	-17.8 (-42.8 to 16.9)
		Female	80 (52 to 149)	58 (38.1 to 104.9)	3 (2 to 5)	2.7 (1.7 to 4.8)	153 (113 to 317)	34.9 (25.8 to 71.7)	6 (5 to 13)	1.7 (1.3 to 3.4)	89.6 (27.4 to 200)	-39.8 (-60.5 to -9)	111.1 (38.1 to 222)	-37.1 (-61.3 to -4)
		Male	89 (54 to 189)	59.3 (37.1 to 123.5)	3 (2 to 7)	2.7 (1.8 to 5.4)	246 (171 to 618)	56.3 (39.7 to 138.9)	10 (7 to 25)	2.8 (2 to 6.7)	175.7 (78.5 to 336.7)	-5 (-37.6 to 49.4)	211.2 (103.2 to 381.5)	1.8 (-32.4 to 56.4)
	Stomach cancer	Both	918 (700 to 1140)	304.7 (236.4 to 378.3)	32 (25 to 40)	13.7 (10.9 to 17)	1634 (1438 to 1844)	183.9 (162 to 208.1)	67 (59 to 75)	8.9 (7.9 to 10.1)	78 (37.1 to 140.6)	-39.6 (-52.8 to -19.7)	104.9 (58.9 to 172.9)	-34.8 (-49 to -15.9)
		Female	400 (276 to 538)	265.6 (190.6 to 361.5)	14 (10 to 19)	11.6 (8.6 to 17.1)	653 (553 to 774)	144.2 (122.8 to 171)	26 (22 to 31)	6.8 (5.7 to 8)	63 (13.8 to 144)	-45.7 (-61.3 to -22.6)	89.3 (35 to 172.1)	-41.7 (-60.4 to -18.8)

Province	Cause	Sex	1990				2019				1990-2019 percent change (%)			
			DALYs		Death		DALYs		Death		DALYs		Death	
			Number	Rate	Number	Rate	Number	Rate	Number	Rate	Number	Rate	Number	Rate
		Male	518 (362 to 687)	341.4 (242.8 to 439.3)	19 (13 to 25)	15.7 (11.3 to 20)	981 (822 to 1168)	225.2 (189.1 to 265.7)	41 (34 to 48)	11.1 (9.4 to 13.1)	89.6 (35.5 to 184.7)	-34.1 (-51.9 to -1.7)	116.2 (56.2 to 220.9)	-29.2 (-47.4 to 0.8)
	Colon and rectum cancer	Both	424 (299 to 559)	138.6 (99.1 to 181.2)	14 (10 to 19)	6.1 (4.4 to 7.7)	1638 (1412 to 1874)	178.9 (155.2 to 205.8)	63 (55 to 73)	8.3 (7.2 to 9.5)	286.1 (168.5 to 477)	29.1 (-9.1 to 88.7)	335.4 (207.3 to 541.6)	36.6 (-1.2 to 97)
		Female	199 (134 to 279)	134.7 (94.8 to 187.2)	7 (5 to 9)	5.9 (4.2 to 8)	728 (604 to 859)	160.6 (133.4 to 190.7)	28 (24 to 34)	7.4 (6.2 to 8.9)	266.9 (140.2 to 473.4)	19.2 (-20.7 to 79.8)	316.8 (177.8 to 533.3)	25.9 (-14 to 89.2)
		Male	226 (143 to 318)	142 (90.5 to 196.4)	8 (5 to 11)	6.2 (4.1 to 8.4)	910 (745 to 1087)	198 (163.9 to 233.8)	35 (29 to 41)	9.2 (7.6 to 10.9)	303 (159.2 to 572.7)	39.4 (-7.6 to 123.6)	351.8 (201.9 to 635)	47.8 (-0.9 to 136.2)
	Liver cancer	Both	381 (280 to 486)	122.4 (91.6 to 156.6)	13 (10 to 17)	5.5 (4.1 to 7.1)	726 (613 to 863)	80.7 (68 to 95.5)	29 (24 to 34)	3.7 (3.1 to 4.4)	90.4 (39.6 to 175.7)	-34.1 (-52.2 to -5.1)	115.3 (54.9 to 211.6)	-32.6 (-52.3 to -2.4)
		Female	162 (110 to 241)	107.7 (72 to 168.6)	6 (4 to 9)	4.9 (3.1 to 7.9)	263 (215 to 321)	57.6 (46.7 to 69.6)	10 (8 to 12)	2.6 (2.1 to 3.1)	62.9 (0.8 to 157.2)	-46.5 (-68.4 to -14.7)	79.1 (5.2 to 188.5)	-48.2 (-70.3 to -13.1)
		Male	220 (154 to 298)	135.7 (96.3 to 181.2)	8 (5 to 10)	6.1 (4.5 to 7.9)	463 (367 to 579)	104.4 (83.3 to 131.1)	19 (15 to 23)	4.9 (4 to 6.1)	110.6 (44 to 221.9)	-23.1 (-47.1 to 14)	141.7 (65.2 to 264.6)	-18.9 (-42.8 to 18.3)
	Gallbladder and biliary tract cancer	Both	69 (51 to 100)	24.5 (18.8 to 35.8)	3 (2 to 4)	1.1 (0.9 to 1.7)	187 (157 to 240)	21.4 (17.9 to 28.2)	8 (6 to 10)	1 (0.9 to 1.4)	171.3 (104.5 to 299.9)	-12.8 (-32.3 to 23.7)	195.5 (129.7 to 320.9)	-9.5 (-29.1 to 23.7)
		Female	36 (24 to 57)	26.9 (18.7 to 43.9)	1 (1 to 2)	1.3 (0.9 to 2.1)	89 (71 to 131)	20.5 (16.4 to 30.6)	4 (3 to 6)	1 (0.8 to 1.5)	149.4 (79.8 to 285)	-23.6 (-45.6 to 12.4)	167.6 (89.4 to 294.3)	-22.1 (-45.9 to 9.7)
		Male	33 (23 to 48)	22.3 (15.8 to 32.1)	1 (1 to 2)	1 (0.7 to 1.5)	98 (75 to 130)	22.3 (17.3 to 29.6)	4 (3 to 5)	1.1 (0.9 to 1.5)	194.8 (100.3 to 371.3)	0.1 (-30.7 to 57)	227.1 (125.8 to 424.9)	6.6 (-24.5 to 63.2)
	Pancreatic cancer	Both	126 (88 to 169)	42.9 (30.2 to 57.1)	5 (3 to 6)	1.9 (1.3 to 2.5)	841 (739 to 965)	95 (83.7 to 109.6)	34 (30 to 39)	4.5 (3.9 to 5.1)	566.9 (379.2 to 923.2)	121.5 (60.4 to 227.9)	636.7 (429.3 to 988.9)	135.1 (68 to 243.4)
		Female	49 (33 to 72)	34.7 (24.1 to 51.2)	2 (1 to 3)	1.5 (1.1 to 2.3)	344 (289 to 404)	79.2 (66.7 to 92.4)	14 (12 to 17)	3.8 (3.2 to 4.4)	608.5 (349.6 to 997.7)	128.3 (45.8 to 246.3)	695.3 (411 to 1083.7)	143.7 (57 to 263.4)
		Male	78 (48 to 111)	50.7 (31.7 to 72.2)	3 (2 to 4)	2.3 (1.4 to 3.2)	497 (413 to 595)	110.7 (91.8 to 131.7)	20 (16 to 24)	5.2 (4.3 to 6.2)	540.9 (318.5 to 1000.6)	118.3 (44.8 to 266.6)	599.2 (360.3 to 1081.6)	129.6 (52.8 to 285.5)
	Larynx cancer	Both	261 (194 to 329)	84.7 (65 to 105.1)	9 (7 to 11)	3.5 (2.7 to 4.3)	510 (429 to 606)	55.8 (47 to 66.7)	19 (16 to 23)	2.4 (2.1 to 2.9)	95.2 (47.5 to 181.5)	-34.1 (-49.5 to -6.5)	114.6 (63.3 to 207.9)	-29.9 (-45.8 to -1.8)
		Female	70 (46 to 92)	44.6 (30.9 to 58.7)	2 (2 to 3)	1.7 (1.2 to 2.3)	120 (98 to 146)	26 (21.4 to 31.3)	4 (4 to 5)	1.1 (0.9 to 1.3)	71.8 (22.7 to 158.3)	-41.7 (-57.2 to -14.7)	96.9 (43 to 195.2)	-36.2 (-52.8 to -6.4)
		Male	191 (132 to 253)	122.6 (86.7 to 160.8)	7 (5 to 9)	5.2 (3.8 to 6.8)	390 (315 to 481)	85.6 (69.9 to 105.2)	15 (12 to 18)	3.8 (3.1 to 4.7)	103.8 (43.7 to 216.7)	-30.2 (-49.9 to 10)	120.5 (57.5 to 251.9)	-27 (-46.8 to 12.7)
	Tracheal, bronchus, and lung cancer	Both	1004 (730 to 1300)	330.7 (248.7 to 425.2)	36 (26 to 46)	14.5 (10.9 to 18.5)	3395 (2925 to 3937)	375.7 (324 to 432.1)	134 (116 to 155)	17.3 (15 to 19.8)	238.1 (138.7 to 398.3)	13.6 (-18.6 to 62.8)	277.6 (171 to 443.7)	19.8 (-12.6 to 67.1)
		Female	254 (176 to 378)	161.6 (114.8 to 238.8)	8 (6 to 12)	6.7 (4.8 to 9.8)	1195 (1024 to 1397)	258.8 (222.3 to 301.9)	45 (39 to 53)	11.4 (9.8 to 13.4)	370.3 (187.5 to 643.2)	60.2 (-0.5 to 143.2)	451.5 (242.1 to 730.9)	71.3 (7.1 to 152.5)

Province	Cause	Sex	1990				2019				1990-2019 percent change (%)			
			DALYs		Death		DALYs		Death		DALYs		Death	
			Number	Rate	Number	Rate	Number	Rate	Number	Rate	Number	Rate	Number	Rate
		Male	750 (527 to 1011)	491.8 (351.5 to 652)	27 (19 to 36)	22.3 (16.1 to 29.1)	2200 (1808 to 2635)	495.6 (411.2 to 592.7)	89 (74 to 106)	23.5 (19.7 to 27.8)	193.4 (100.2 to 359.8)	0.8 (-31.1 to 52.6)	225.2 (123.1 to 397)	5.4 (-26.3 to 54.7)
	Malignant skin melanoma	Both	88 (33 to 138)	26.4 (9.4 to 40.7)	3 (1 to 4)	1.2 (0.4 to 1.9)	137 (81 to 173)	14.2 (8 to 18.2)	5 (3 to 6)	0.6 (0.3 to 0.8)	54.8 (-3.7 to 239.4)	-46.1 (-64.6 to 13.2)	74.8 (15.9 to 259.1)	-46.7 (-62.4 to 8.8)
		Female	44 (12 to 77)	26 (7.7 to 43.4)	1 (0 to 2)	1.1 (0.3 to 2)	63 (30 to 89)	13 (5.9 to 18.2)	2 (1 to 3)	0.5 (0.2 to 0.8)	43.8 (-12.4 to 220)	-50 (-66.5 to -2)	58.6 (6.8 to 208.1)	-52 (-66.1 to -15.9)
		Male	44 (17 to 68)	26.7 (9.1 to 42)	1 (0 to 2)	1.3 (0.4 to 2)	73 (43 to 104)	15.5 (8.3 to 21.3)	3 (1 to 4)	0.7 (0.3 to 1)	65.7 (-5.3 to 289.3)	-41.9 (-66.3 to 40.1)	90 (11.1 to 335.1)	-41.9 (-65.6 to 46.4)
	Non-melanoma skin cancer	Both	17 (12 to 21)	6.1 (4.3 to 7.7)	1 (0 to 1)	0.3 (0.2 to 0.4)	59 (51 to 68)	7.1 (6.1 to 8.2)	3 (2 to 3)	0.4 (0.3 to 0.5)	254.4 (168.4 to 412.9)	16.9 (-11.1 to 66.6)	311.1 (209.2 to 496.8)	27.3 (-4.1 to 83.4)
		Female	3 (2 to 4)	2 (1.2 to 2.9)	0 (0 to 0)	0.1 (0.1 to 0.2)	11 (9 to 13)	2.6 (2.2 to 3.1)	0 (0 to 1)	0.1 (0.1 to 0.2)	324.7 (181.4 to 614.4)	33.3 (-14.4 to 133.6)	371.5 (203.4 to 733.8)	37.8 (-15.2 to 165.3)
		Male	14 (10 to 18)	10.2 (7.2 to 13)	1 (0 to 1)	0.5 (0.4 to 0.7)	48 (40 to 56)	11.7 (9.8 to 13.7)	2 (2 to 3)	0.7 (0.6 to 0.8)	241.3 (149.5 to 397.9)	14.7 (-15.5 to 62.8)	299.8 (193.7 to 485)	23 (-8.1 to 72.4)
	Breast cancer	Both	556 (363 to 810)	158.4 (104.8 to 232.4)	16 (10 to 23)	5.2 (3.4 to 8.1)	2104 (1755 to 2450)	186.1 (155.9 to 216.8)	58 (49 to 68)	6 (5.1 to 7)	278.5 (154.4 to 481.7)	17.5 (-22.9 to 79)	276.6 (140.4 to 486.6)	15.1 (-29.5 to 78.2)
		Female	550 (356 to 803)	321.2 (212.4 to 473.6)	15 (10 to 23)	10.4 (6.7 to 16.2)	2090 (1743 to 2436)	384.1 (321.8 to 447.8)	58 (49 to 68)	12.1 (10.2 to 14.1)	279.7 (154.3 to 489.7)	19.6 (-21.8 to 84.4)	277.7 (140.1 to 497.5)	15.8 (-29 to 80.6)
		Male	6 (3 to 9)	3.3 (2 to 5.1)	0 (0 to 0)	0.1 (0.1 to 0.2)	15 (11 to 19)	3 (2.2 to 4)	0 (0 to 1)	0.1 (0.1 to 0.2)	162.3 (51.8 to 362.8)	-7.6 (-46.4 to 61.7)	181.6 (62.7 to 401.1)	-4.9 (-45.2 to 64.3)
	Cervical cancer	Female	175 (100 to 245)	101.3 (60 to 141.7)	5 (3 to 7)	3.5 (2.2 to 5.2)	300 (203 to 366)	59.3 (40.5 to 71.9)	10 (7 to 12)	2.2 (1.5 to 2.7)	72 (19.9 to 191.6)	-41.5 (-58 to -7.4)	93.6 (37.1 to 210.7)	-37.2 (-57.9 to -6.1)
	Uterine cancer	Female	37 (20 to 53)	23.9 (14.1 to 35.3)	1 (1 to 2)	0.9 (0.6 to 1.4)	158 (60 to 206)	32.8 (13.4 to 42.6)	5 (2 to 7)	1.2 (0.6 to 1.6)	324.4 (126.3 to 578.2)	37 (-28.8 to 117.1)	335.6 (131.9 to 588.7)	34.3 (-28.3 to 114)
	Ovarian cancer	Female	71 (40 to 130)	40.9 (23.5 to 73.9)	2 (1 to 4)	1.4 (0.8 to 2.5)	412 (284 to 511)	81.6 (57.7 to 100.9)	13 (9 to 16)	2.9 (2.1 to 3.6)	479.1 (153.9 to 988.3)	99.6 (-10.8 to 267.5)	539 (188.1 to 1079)	110.2 (-4.4 to 281.9)
	Prostate cancer	Male	210 (134 to 289)	192.9 (128.5 to 261.5)	10 (6 to 13)	11.9 (7.9 to 16.2)	965 (678 to 1179)	278 (197.2 to 338.1)	51 (36 to 62)	17.1 (12.3 to 20.8)	359.3 (242.7 to 570.6)	44.1 (9.2 to 100)	421.6 (289.7 to 638)	43.1 (8.2 to 97.8)
	Testicular cancer	Male	3 (2 to 4)	0.7 (0.5 to 0.9)	0 (0 to 0)	0 (0 to 0)	67 (48 to 89)	10.5 (7.5 to 13.8)	1 (1 to 2)	0.2 (0.2 to 0.3)	2156.2 (1361.8 to 3961.4)	1331 (881.4 to 2294.4)	2816.3 (1911.4 to 4804.3)	1595.8 (1104.2 to 2655.2)
	Kidney cancer	Both	93 (62 to 123)	20.8 (13 to 26.8)	2 (1 to 3)	0.8 (0.5 to 1)	284 (244 to 326)	29.8 (25.5 to 34.2)	10 (8 to 11)	1.2 (1 to 1.4)	205 (113.7 to 382.9)	43.3 (4 to 147.4)	320.3 (199.2 to 638.7)	56.2 (12.8 to 188.8)
		Female	40 (27 to 56)	17.6 (11 to 23.6)	1 (1 to 1)	0.7 (0.3 to 0.9)	95 (79 to 111)	19.4 (16.3 to 22.7)	3 (2 to 3)	0.7 (0.6 to 0.8)	134.4 (54.6 to 262.8)	10.1 (-23.7 to 85.8)	205 (110.4 to 423.3)	7.8 (-27.6 to 121)
		Male	53 (32 to 75)	23.8 (13.8 to 33.2)	1 (1 to 2)	0.9 (0.5 to 1.2)	189 (151 to 229)	40.2 (32.6 to 48.2)	7 (6 to 8)	1.7 (1.4 to 2.1)	259 (123.3 to 545.5)	69.3 (9 to 211.1)	400.4 (214.5 to 813.3)	93.1 (25 to 262.1)

Province	Cause	Sex	1990				2019				1990-2019 percent change (%)			
			DALYs		Death		DALYs		Death		DALYs		Death	
			Number	Rate	Number	Rate	Number	Rate	Number	Rate	Number	Rate	Number	Rate
	Bladder cancer	Both	222 (148 to 292)	84.2 (57.9 to 110.4)	9 (6 to 12)	4.3 (3 to 5.7)	765 (647 to 903)	94.2 (79.3 to 110.7)	33 (28 to 39)	4.9 (4.1 to 5.7)	245.3 (149.7 to 439.6)	11.9 (-18.6 to 69.8)	278.9 (175.2 to 484.8)	13.5 (-17.2 to 69.5)
		Female	47 (31 to 68)	37.7 (24.9 to 57)	2 (1 to 3)	2 (1.3 to 3.2)	159 (130 to 192)	39.4 (32.4 to 47.2)	7 (6 to 9)	2.2 (1.8 to 2.6)	237.2 (123 to 459.2)	4.6 (-33 to 70.4)	285.6 (143.3 to 531.4)	9.2 (-36 to 81.3)
		Male	174 (110 to 241)	129.6 (85.9 to 175.9)	7 (4 to 10)	6.7 (4.6 to 9)	606 (489 to 738)	150.2 (122.1 to 182.6)	26 (21 to 32)	7.7 (6.3 to 9.4)	247.4 (129.7 to 474)	15.8 (-20.5 to 87.5)	277.1 (152.8 to 518.6)	14.1 (-19.9 to 79.1)
	Brain and central nervous system cancer	Both	942 (486 to 1295)	163.9 (87.2 to 221.1)	19 (10 to 25)	4.6 (2.6 to 6.2)	1623 (859 to 2053)	147.1 (77.4 to 185.6)	43 (22 to 55)	4.6 (2.3 to 5.9)	72.3 (-10 to 164.7)	-10.3 (-48.7 to 30.2)	130.7 (28.5 to 234.8)	-0.6 (-39.1 to 39.1)
		Female	425 (162 to 750)	145.8 (62 to 244.7)	8 (3 to 14)	3.9 (1.8 to 6.3)	770 (405 to 1041)	143.5 (75.5 to 193.5)	20 (10 to 27)	4.3 (2.2 to 5.8)	81.4 (-18.5 to 288.7)	-1.6 (-52.8 to 94.2)	149 (19.2 to 388.1)	9.8 (-42.4 to 96.1)
		Male	517 (236 to 773)	180.9 (86.7 to 267.5)	11 (5 to 16)	5.3 (2.6 to 7.7)	852 (384 to 1185)	151 (67.7 to 208.1)	23 (10 to 32)	4.9 (2.1 to 6.7)	64.9 (-16.5 to 180.1)	-16.5 (-53.3 to 35.6)	116.7 (17.5 to 248.5)	-7.8 (-44.8 to 44)
	Thyroid cancer	Both	29 (21 to 39)	9.2 (6.8 to 12.4)	1 (1 to 1)	0.4 (0.3 to 0.6)	147 (90 to 178)	15 (9.6 to 18.1)	5 (3 to 5)	0.6 (0.4 to 0.7)	404.8 (148.3 to 652.3)	64.2 (-15.3 to 139.9)	393.6 (163.9 to 628)	48.5 (-18.5 to 118.1)
		Female	19 (13 to 26)	12.6 (8.9 to 18.4)	1 (0 to 1)	0.5 (0.4 to 0.8)	99 (45 to 126)	20.5 (9.7 to 26.1)	3 (1 to 4)	0.7 (0.4 to 0.9)	425.3 (103.3 to 715)	63.1 (-32.6 to 149.1)	389.1 (113.1 to 661.8)	40.1 (-39 to 122.2)
		Male	10 (7 to 15)	5.8 (3.9 to 8.8)	0 (0 to 0)	0.2 (0.2 to 0.3)	48 (38 to 60)	9.8 (7.7 to 12.2)	2 (1 to 2)	0.4 (0.3 to 0.5)	367.2 (179.3 to 647.8)	67.3 (1.5 to 162.9)	402.3 (206.8 to 706.1)	69.3 (3.4 to 166)
	Mesothelioma	Both	6 (2 to 12)	1.7 (0.6 to 3.5)	0 (0 to 0)	0.1 (0 to 0.1)	22 (13 to 27)	2.2 (1.4 to 2.8)	1 (0 to 1)	0.1 (0.1 to 0.1)	275.3 (98.3 to 784.3)	33.1 (-34.5 to 188.4)	345.9 (116.5 to 846.3)	52.2 (-29.6 to 212.4)
		Female	3 (1 to 4)	1.6 (0.8 to 2.3)	0 (0 to 0)	0.1 (0 to 0.1)	5 (3 to 7)	1 (0.6 to 1.3)	0 (0 to 0)	0 (0 to 0)	86.7 (8.8 to 307.4)	-37 (-59.4 to 18.2)	102.7 (31.2 to 280.4)	-36.6 (-55.2 to 3.1)
		Male	3 (0 to 9)	1.7 (0.2 to 5.3)	0 (0 to 0)	0.1 (0 to 0.2)	17 (9 to 21)	3.5 (1.9 to 4.4)	1 (0 to 1)	0.2 (0.1 to 0.2)	454.8 (103.2 to 2835.4)	98.3 (-32.1 to 999.1)	560.1 (120.9 to 3458.9)	130.1 (-27.7 to 1237.8)
	Hodgkin lymphoma	Both	58 (27 to 126)	11.8 (6.2 to 24.2)	1 (1 to 3)	0.4 (0.2 to 0.7)	137 (97 to 164)	11.8 (8.3 to 14)	3 (2 to 4)	0.3 (0.2 to 0.4)	136.3 (4.4 to 431.1)	0.3 (-53.9 to 108.5)	156.8 (19.2 to 435.7)	-4.6 (-54.2 to 91.1)
		Female	20 (9 to 44)	8.9 (4.3 to 18.8)	0 (0 to 1)	0.3 (0.1 to 0.6)	53 (32 to 69)	9.5 (5.7 to 12.2)	1 (1 to 2)	0.3 (0.2 to 0.3)	161.6 (6.1 to 566)	6.6 (-55.7 to 148.4)	164.2 (11.2 to 519.1)	-12.3 (-61.2 to 97.4)
		Male	38 (18 to 87)	14.5 (7.4 to 32.4)	1 (0 to 2)	0.4 (0.2 to 0.9)	84 (55 to 106)	14 (9.3 to 17.7)	2 (1 to 3)	0.4 (0.3 to 0.5)	122.5 (-4.6 to 407.9)	-3.1 (-56.3 to 109.1)	152.5 (14.1 to 445.8)	1.8 (-51.9 to 109.1)
	Non-Hodgkin lymphoma	Both	208 (142 to 265)	46.5 (34.5 to 57.7)	5 (4 to 6)	1.6 (1.2 to 1.9)	527 (455 to 602)	50.4 (43.6 to 57.2)	16 (14 to 18)	1.8 (1.5 to 2)	153.5 (81.2 to 289.1)	8.3 (-18.5 to 53.4)	205 (127 to 329.5)	12.6 (-12.2 to 57.8)
		Female	81 (50 to 115)	36.8 (22.2 to 48.7)	2 (1 to 3)	1.3 (0.7 to 1.7)	217 (182 to 258)	42.3 (35.5 to 50.2)	6 (5 to 8)	1.4 (1.2 to 1.7)	169.2 (72.9 to 377.5)	15 (-20.3 to 108.9)	220 (120.9 to 485.7)	13 (-19.1 to 114.4)
		Male	127 (86 to 164)	55.7 (39.3 to 70.7)	3 (2 to 4)	1.9 (1.4 to 2.4)	310 (257 to 367)	58.3 (48.6 to 68.5)	9 (8 to 11)	2.1 (1.8 to 2.5)	143.5 (73.7 to 284.7)	4.7 (-23.4 to 53.9)	195.5 (116.3 to 340)	13.2 (-15 to 58.5)
	Multiple myeloma	Both	59 (41 to 89)	19.7 (13.8 to 30.2)	2 (1 to 3)	0.8 (0.6 to 1.3)	172 (126 to 263)	18.9 (13.6 to 29.6)	7 (5 to 10)	0.8 (0.6 to 1.3)	191.7 (89.2 to 309.6)	-4.2 (-35.8 to 30.8)	205.3 (105.4 to 321.8)	-2.8 (-32.7 to 31.2)
		Female	27 (18 to 40)	19.7 (13.2 to 29)	1 (1 to 2)	0.9 (0.6 to 1.3)	67 (51 to 93)	14.8 (11.3 to 21.1)	3 (2 to 4)	0.6 (0.5 to 0.9)	146.2 (77 to 265.2)	-24.9 (-44.8 to 12.7)	147.1 (80.2 to 274.3)	-28 (-48.4 to 10.7)

Province	Cause	Sex	1990				2019				1990-2019 percent change (%)			
			DALYs		Death		DALYs		Death		DALYs		Death	
			Number	Rate	Number	Rate	Number	Rate	Number	Rate	Number	Rate	Number	Rate
		Male	32 (19 to 56)	19.8 (12.1 to 35.1)	1 (1 to 2)	0.8 (0.5 to 1.5)	105 (68 to 182)	23.1 (14.8 to 41)	4 (3 to 7)	1 (0.6 to 1.8)	230.3 (84.6 to 431.4)	16.5 (-34.9 to 85.1)	259.1 (100.9 to 470.2)	24.8 (-30 to 91.6)
	Leukemia	Both	2104 (1201 to 2947)	323.9 (196.3 to 423.8)	38 (23 to 50)	8.9 (5.6 to 11.3)	2229 (1421 to 2684)	207.3 (135 to 248.5)	60 (40 to 71)	6.7 (4.7 to 8)	5.9 (-25.5 to 68.2)	-36 (-51.7 to -7.3)	57.6 (18.7 to 131.3)	-24.2 (-40.2 to 6.8)
		Female	1166 (426 to 1672)	343 (155.9 to 453.8)	20 (9 to 27)	8.7 (4.6 to 11.7)	1029 (528 to 1256)	192 (102.3 to 232.5)	26 (14 to 31)	5.6 (3.3 to 6.8)	-11.7 (-41.5 to 66.6)	-44 (-59.7 to -13)	30.7 (-6.1 to 106.9)	-35.1 (-51.4 to -7.7)
		Male	938 (430 to 1375)	305.8 (164.4 to 420.4)	18 (10 to 25)	9.2 (5.4 to 12.4)	1200 (738 to 1534)	224.2 (138.8 to 281.4)	34 (21 to 42)	7.9 (5.2 to 9.9)	27.9 (-17 to 138.5)	-26.7 (-49.7 to 20.5)	86.9 (27 to 218.3)	-13.8 (-39.3 to 35.4)
	Other malignant neoplasms	Both	638 (441 to 795)	126.2 (92 to 154.2)	14 (10 to 17)	4.3 (3.1 to 5.2)	1352 (1091 to 1549)	132.1 (105.5 to 152.2)	39 (31 to 45)	4.7 (3.6 to 5.5)	112.1 (43.8 to 206.6)	4.6 (-24.9 to 49.7)	175.7 (96.2 to 297.9)	8.4 (-20.5 to 59.9)
		Female	280 (190 to 355)	108.6 (78.6 to 136.9)	6 (4 to 8)	3.6 (2.5 to 4.6)	572 (491 to 657)	113 (97.9 to 129.3)	16 (14 to 18)	3.8 (3.2 to 4.3)	104.2 (53.8 to 213.9)	4 (-21.7 to 50.9)	166.6 (99.1 to 294.7)	5.4 (-22.8 to 57.8)
		Male	358 (244 to 488)	143.2 (98.9 to 186.4)	8 (6 to 11)	5 (3.5 to 6.5)	781 (550 to 959)	151.2 (104 to 186.9)	23 (16 to 29)	5.6 (3.7 to 7)	118.2 (21.5 to 251.9)	5.5 (-36.1 to 69.4)	182.4 (66.1 to 358)	10.6 (-32.2 to 74.4)
	Other neoplasms	Both	24 (16 to 31)	6.2 (3.8 to 8)	1 (0 to 1)	0.2 (0.1 to 0.3)	47 (39 to 61)	5.1 (4.3 to 6.8)	1 (1 to 2)	0.2 (0.2 to 0.3)	92.4 (43.2 to 208.2)	-17.5 (-40 to 39.9)	151.3 (71 to 384.3)	-17.6 (-44.7 to 71.4)
		Female	12 (8 to 16)	5.9 (3.2 to 8.1)	0 (0 to 0)	0.2 (0.1 to 0.3)	20 (16 to 24)	4.3 (3.5 to 5.2)	1 (0 to 1)	0.2 (0.1 to 0.2)	66.6 (18.5 to 182.1)	-28.1 (-50 to 40.7)	115.7 (40.2 to 392.8)	-32.8 (-57.8 to 89.1)
		Male	12 (8 to 16)	6.4 (3.9 to 8.5)	0 (0 to 0)	0.3 (0.1 to 0.4)	27 (21 to 40)	6 (4.6 to 9.1)	1 (1 to 1)	0.2 (0.2 to 0.4)	117.3 (45.2 to 272.5)	-6.7 (-36.9 to 56.3)	180.8 (76 to 431.3)	-3.2 (-40.2 to 78.7)
	Cardiovascular diseases	Both	26981 (22068 to 31730)	9825.7 (8289.3 to 11354.6)	997 (830 to 1162)	507.1 (431.3 to 578.5)	47609 (43399 to 52624)	5982.4 (5432 to 6585.8)	2150 (1942 to 2375)	339.5 (304.6 to 372.5)	76.5 (45.9 to 122.3)	-39.1 (-49 to -25.9)	115.7 (77.9 to 167.3)	-33 (-43.1 to -20.7)
		Female	10874 (8679 to 13186)	8312.7 (6786 to 9886.4)	414 (335 to 498)	446.4 (370.1 to 523.6)	18979 (16786 to 21062)	4966.3 (4419.6 to 5470)	906 (797 to 999)	295.3 (257.3 to 323.8)	74.5 (41.6 to 121.4)	-40.3 (-50.3 to -26.6)	119 (80.3 to 173.1)	-33.8 (-44.1 to -20)
		Male	16107 (12261 to 19922)	11247.9 (8787.5 to 13648.7)	583 (447 to 714)	566.3 (452.6 to 670.6)	28631 (25308 to 32310)	6985.9 (6200.4 to 7869.3)	1244 (1101 to 1409)	384.4 (339.1 to 433.2)	77.8 (37.7 to 139.3)	-37.9 (-51.1 to -18.1)	113.4 (65.8 to 186.8)	-32.1 (-45.6 to -13.3)
	Rheumatic heart disease	Both	377 (247 to 517)	68.7 (48 to 101)	8 (5 to 11)	2.2 (1.6 to 3.7)	203 (173 to 238)	20.6 (17.5 to 24)	6 (5 to 8)	0.8 (0.7 to 1)	-46.2 (-64.8 to -17.1)	-70.1 (-81.9 to -55.2)	-16.4 (-50.8 to 30)	-62.9 (-80.7 to -42.4)
		Female	237 (142 to 335)	85 (54.5 to 128)	5 (3 to 7)	2.7 (1.7 to 4.7)	112 (93 to 142)	23.3 (19.3 to 29.1)	4 (3 to 4)	0.9 (0.7 to 1.2)	-52.6 (-69.5 to -10.2)	-72.6 (-84 to -50.7)	-23.6 (-57.3 to 43.3)	-65.4 (-83.3 to -37.3)
		Male	140 (80 to 213)	52.7 (32 to 83.3)	3 (2 to 5)	1.8 (1.1 to 3.1)	90 (71 to 116)	18 (14.4 to 22.7)	3 (2 to 4)	0.7 (0.6 to 0.9)	-35.4 (-62.9 to 28.2)	-65.8 (-81.4 to -37.8)	-5.1 (-50.3 to 79.6)	-58.8 (-79.8 to -27.2)
	Ischemic heart disease	Both	15977 (12769 to 19169)	5944.6 (4886.6 to 6994.7)	626 (514 to 739)	304.3 (254.3 to 352.2)	26877 (24136 to 30442)	3366.2 (3031.1 to 3778.4)	1268 (1137 to 1428)	198.1 (177 to 221.4)	68.2 (34.9 to 118.4)	-43.4 (-53.4 to -28.8)	102.7 (65.8 to 157.7)	-34.9 (-45.3 to -21.1)
		Female	5667 (4403 to 7011)	4575.5 (3557.5 to 5526.6)	238 (184 to 289)	250.7 (201.9 to 299.2)	9505 (8331 to 10720)	2543.5 (2244.9 to 2838.7)	501 (440 to 559)	164.4 (142.7 to 182.4)	67.7 (31.9 to 117.4)	-44.4 (-54.9 to -28.8)	110.6 (69.5 to 169.1)	-34.4 (-45.8 to -18.7)
		Male	10310 (7660 to 12985)	7240.6 (5521.3 to 8953.9)	388 (292 to 483)	357.2 (279.7 to 430.7)	17373 (15099 to 20194)	4171.1 (3642.9 to 4835.6)	767 (670 to 887)	232.1 (202.6 to 266)	68.5 (25.9 to 133.2)	-42.4 (-56 to -21.8)	97.8 (50.1 to 168.4)	-35 (-49.4 to -15.1)
	Stroke	Both	7686 (6177 to 9155)	2917.7 (2326.6 to 3426.7)	279 (217 to 333)	157.7 (122.6 to 184.2)	13818 (12284 to 15308)	1763.9 (1542.3 to 1951.9)	603 (515 to 669)	97 (81 to 107.6)	79.8 (44.9 to 123.4)	-39.5 (-50.9 to -26)	116.1 (71.7 to 171.4)	-38.5 (-49.8 to -24.3)

Province	Cause	Sex	1990				2019				1990-2019 percent change (%)			
			DALYs		Death		DALYs		Death		DALYs		Death	
			Number	Rate	Number	Rate	Number	Rate	Number	Rate	Number	Rate	Number	Rate
		Female	3548 (2816 to 4361)	2748.4 (2158.8 to 3341.5)	129 (98 to 159)	148.2 (114.1 to 181.5)	6401 (5603 to 7177)	1645.1 (1414.3 to 1836.4)	276 (233 to 309)	89.2 (73.8 to 100.2)	80.4 (43 to 134.8)	-40.1 (-52 to -23.2)	114.8 (68.4 to 183.1)	-39.8 (-52 to -22.4)
		Male	4138 (3084 to 5185)	3074.8 (2196.3 to 3801)	151 (105 to 191)	166.9 (112.8 to 208.6)	7417 (6225 to 8466)	1888.4 (1550.4 to 2155.6)	327 (260 to 378)	105.2 (81.1 to 121.4)	79.2 (36.4 to 143.7)	-38.6 (-53 to -19.4)	117.2 (61.4 to 198.3)	-36.9 (-52.2 to -16.9)
	Hypertensive heart disease	Both	1217 (886 to 1962)	521.3 (384 to 780.3)	51 (37 to 79)	29.7 (21.4 to 44.6)	3540 (3100 to 4244)	467.7 (408.9 to 553.6)	172 (149 to 203)	28.1 (24.1 to 33)	190.9 (100 to 311.2)	-10.3 (-37.3 to 24.9)	236.2 (126.7 to 389.1)	-5.3 (-37.4 to 33.3)
		Female	595 (405 to 990)	536.8 (369.1 to 912.9)	26 (18 to 46)	31.4 (21 to 56.2)	1595 (1311 to 1932)	431.3 (353.8 to 512.4)	82 (66 to 98)	27.1 (21.4 to 32.4)	168.3 (55.2 to 320.2)	-19.6 (-56.8 to 21.6)	209.2 (60.6 to 385.3)	-13.7 (-58.6 to 34.5)
		Male	622 (409 to 1093)	502.8 (349.7 to 796.8)	25 (16 to 41)	27.5 (18.8 to 42.5)	1944 (1609 to 2425)	504.2 (419.1 to 623.2)	90 (74 to 112)	29.1 (24.1 to 35.7)	212.4 (99 to 389.5)	0.3 (-33.1 to 50.1)	265.3 (130.6 to 469.5)	5.6 (-30.2 to 58.4)
	Non-rheumatic valvular heart disease	Both	109 (76 to 150)	33.3 (25.6 to 42.4)	3 (3 to 4)	1.5 (1.2 to 2)	259 (222 to 342)	27.9 (23.9 to 37)	10 (8 to 13)	1.3 (1.1 to 1.8)	137.2 (73.3 to 242.5)	-16.2 (-35.1 to 13.5)	181.8 (117.3 to 286.8)	-12.9 (-33.8 to 18.1)
		Female	61 (40 to 85)	39.5 (28.8 to 52.1)	2 (1 to 3)	1.9 (1.4 to 2.6)	133 (112 to 156)	29.7 (25.2 to 34.7)	5 (4 to 6)	1.4 (1.2 to 1.7)	117.9 (50.3 to 238.5)	-25 (-45.9 to 4.5)	156.7 (83.6 to 262.3)	-22.7 (-47 to 8.9)
		Male	48 (31 to 80)	27 (18.1 to 42.2)	1 (1 to 2)	1.2 (0.8 to 1.8)	126 (98 to 206)	26.2 (20.5 to 43.8)	5 (4 to 8)	1.2 (0.9 to 2.1)	161.8 (59.1 to 328.8)	-3 (-37.9 to 50)	216.8 (99.9 to 398)	5 (-30.2 to 55.2)
	Cardiomyopathy and myocarditis	Both	349 (195 to 802)	56.4 (36.8 to 106.8)	6 (4 to 13)	1.9 (1.3 to 3.4)	352 (243 to 618)	36.5 (25.6 to 63.3)	11 (7 to 19)	1.4 (1 to 2.5)	0.7 (-46.9 to 77.4)	-35.2 (-58.8 to 0)	65.1 (0.1 to 167.3)	-25.8 (-51.6 to 14.3)
		Female	164 (83 to 424)	52 (31 to 105)	3 (2 to 6)	1.9 (1 to 4)	122 (88 to 214)	26.3 (19.3 to 45.2)	4 (3 to 7)	1 (0.7 to 1.7)	-25.5 (-63.2 to 51)	-49.5 (-67.6 to -16.3)	24 (-23.9 to 117.4)	-45.4 (-67.9 to -1.1)
		Male	185 (105 to 419)	60.4 (38 to 118.6)	3 (2 to 7)	1.9 (1.2 to 3.7)	229 (146 to 433)	46.5 (29.7 to 88.6)	7 (4 to 14)	1.8 (1.1 to 3.7)	23.9 (-38.8 to 121.4)	-22.9 (-56 to 25.9)	100.4 (8.9 to 240.3)	-5.5 (-41.9 to 46.7)
	Atrial fibrillation and flutter	Both	139 (104 to 181)	74.8 (55 to 95.9)	4 (2 to 5)	3.1 (1.9 to 4)	529 (417 to 671)	79.5 (63.7 to 99.4)	18 (15 to 20)	3.5 (3 to 4)	280.2 (229.6 to 351.6)	6.3 (-9.6 to 32.1)	378.4 (254.8 to 655.8)	15.6 (-15.5 to 88.7)
		Female	77 (54 to 103)	86.3 (58 to 113.4)	2 (1 to 3)	3.8 (2 to 5.2)	281 (222 to 356)	84 (67.2 to 104.2)	10 (8 to 12)	3.8 (3.1 to 4.6)	264.5 (204.9 to 395.8)	-2.6 (-20.8 to 39.4)	295.6 (181.3 to 692.1)	-1.4 (-31 to 105.6)
		Male	62 (45 to 83)	61.9 (46.4 to 80.7)	1 (1 to 2)	2.1 (1.5 to 2.9)	248 (186 to 321)	74.7 (57.4 to 94.5)	8 (5 to 10)	3.3 (2.2 to 4.1)	299.6 (222 to 376.5)	20.7 (-2.9 to 44.4)	545.8 (297.3 to 850.4)	53.8 (-2.5 to 121.7)
	Aortic aneurysm	Both	57 (39 to 78)	20.7 (14.6 to 27.2)	2 (2 to 3)	1.1 (0.8 to 1.4)	153 (127 to 182)	18.3 (15.2 to 21.6)	7 (6 to 8)	1 (0.8 to 1.2)	167 (80.5 to 318.9)	-11.6 (-38.5 to 32.6)	210.6 (113.5 to 371.5)	-6.2 (-33.3 to 36.1)
		Female	15 (11 to 20)	12.5 (8.9 to 16.1)	1 (0 to 1)	0.7 (0.5 to 0.9)	36 (30 to 42)	8.7 (7.2 to 10.2)	2 (1 to 2)	0.5 (0.4 to 0.6)	139.7 (75 to 236.2)	-30.8 (-48.6 to -2)	152.2 (84.8 to 259.5)	-32.3 (-49.6 to -1.5)
		Male	42 (26 to 63)	28.5 (17.7 to 40.8)	2 (1 to 2)	1.4 (0.9 to 2)	117 (93 to 145)	28 (22.2 to 34.8)	5 (4 to 7)	1.5 (1.2 to 1.9)	176.7 (67.8 to 392.8)	-1.8 (-39 to 72.1)	235 (105.4 to 489.9)	7.7 (-32 to 85.2)
	Peripheral artery disease	Both	14 (8 to 23)	7 (3.9 to 11.4)	0 (0 to 0)	0.1 (0.1 to 0.2)	135 (88 to 174)	19.2 (12.4 to 25)	6 (3 to 7)	1 (0.6 to 1.3)	887.2 (509 to 1562.5)	176.7 (70.8 to 367.6)	4076.7 (1618.7 to 6730.4)	893.6 (270.7 to 1563.8)
		Female	8 (4 to 13)	8.1 (4.3 to 13.8)	0 (0 to 0)	0.1 (0 to 0.2)	52 (19 to 76)	15.4 (5.6 to 22.3)	2 (0 to 3)	0.7 (0.1 to 1.1)	570.8 (201.6 to 1071.9)	90.6 (-17 to 231.7)	2995 (197.3 to 8440.5)	650.6 (-27.9 to 2042.6)

Province	Cause	Sex	1990				2019				1990-2019 percent change (%)			
			DALYs		Death		DALYs		Death		DALYs		Death	
			Number	Rate	Number	Rate	Number	Rate	Number	Rate	Number	Rate	Number	Rate
		Male	6 (3 to 10)	5.8 (3.4 to 9.3)	0 (0 to 0)	0.1 (0.1 to 0.1)	83 (65 to 104)	23.2 (18.2 to 29.1)	4 (3 to 5)	1.3 (1 to 1.6)	1297.8 (809.1 to 2211.8)	299 (160 to 551.5)	5039.1 (3301.6 to 8610)	1153.6 (727.8 to 1957.5)
	Endocarditis	Both	78 (41 to 164)	14.2 (7.4 to 30.3)	2 (1 to 3)	0.5 (0.2 to 1.2)	100 (59 to 217)	10.1 (5.9 to 21.9)	3 (2 to 7)	0.4 (0.2 to 0.9)	29.2 (-18.5 to 125.7)	-29.2 (-48.3 to 0.7)	103.5 (48.7 to 192.7)	-16.5 (-34.7 to 18.5)
		Female	39 (20 to 82)	13.3 (6.5 to 30.3)	1 (0 to 2)	0.5 (0.2 to 1.4)	39 (21 to 99)	8.4 (4.6 to 21.2)	1 (1 to 4)	0.4 (0.2 to 1)	0.3 (-52.9 to 121.8)	-36.7 (-58.8 to -9.5)	79.3 (6.1 to 170.1)	-21.8 (-46 to 19.6)
		Male	39 (18 to 91)	15.1 (7.2 to 33.7)	1 (0 to 2)	0.5 (0.3 to 1.1)	61 (35 to 128)	11.6 (6.7 to 24.8)	2 (1 to 4)	0.5 (0.3 to 1)	58.4 (-7.9 to 170.3)	-23.1 (-50.7 to 22.2)	125 (44.3 to 264.8)	-11.7 (-38.8 to 31.8)
	Other cardiovascular and circulatory diseases	Both	979 (727 to 1380)	167 (131 to 241)	16 (12 to 24)	5.1 (3.9 to 7.9)	1644 (1409 to 1912)	172.5 (149.5 to 198.1)	47 (40 to 54)	6.8 (5.7 to 7.9)	68 (24.8 to 121.4)	3.3 (-25.7 to 32.6)	197.8 (102.4 to 314)	33.2 (-19.3 to 81.9)
		Female	464 (322 to 643)	155.4 (116.7 to 215.7)	7 (5 to 11)	4.6 (3.3 to 8.2)	702 (588 to 831)	150.7 (128.1 to 176)	20 (16 to 23)	5.9 (4.8 to 6.9)	51.4 (3.6 to 124.5)	-3 (-36.7 to 30.9)	174.4 (63.8 to 303.2)	28.8 (-39.3 to 84.5)
		Male	515 (368 to 926)	178.3 (131.8 to 342.2)	8 (6 to 18)	5.7 (4 to 11.6)	942 (801 to 1173)	194 (166.5 to 234.9)	27 (22 to 33)	7.7 (6.4 to 9.3)	82.9 (20.8 to 157.5)	8.8 (-31.2 to 48.2)	217.8 (94.9 to 370.7)	35.9 (-18.9 to 100.8)
	Chronic respiratory diseases	Both	3173 (2525 to 3970)	844.8 (690.9 to 1061.2)	58 (44 to 81)	29.8 (22.9 to 41.1)	6044 (5174 to 7095)	655 (571 to 750.7)	137 (120 to 165)	21.4 (18.6 to 25.9)	90.5 (63.2 to 123.7)	-22.5 (-35.1 to -7.7)	134.6 (79.4 to 214.5)	-28.2 (-45.9 to -6.2)
		Female	1508 (1167 to 1940)	797.1 (615.3 to 1057.6)	26 (17 to 39)	26.7 (17.9 to 41.3)	2681 (2250 to 3152)	580.2 (492.9 to 672.6)	54 (44 to 65)	16.8 (13.7 to 20.1)	77.7 (47.3 to 125.6)	-27.2 (-44.1 to -5.5)	108.6 (39.7 to 223.2)	-37.1 (-61.3 to -2.1)
		Male	1664 (1308 to 2184)	892.4 (718.4 to 1214.1)	32 (24 to 49)	33.4 (25 to 50.3)	3363 (2861 to 4020)	733.4 (632.1 to 886.4)	83 (70 to 110)	26.3 (22.1 to 35.9)	102.1 (67.5 to 145.2)	-17.8 (-34.4 to 1.7)	155.5 (84.2 to 259.8)	-21.2 (-41.4 to 6.9)
	Chronic obstructive pulmonary disease	Both	1153 (944 to 1489)	389.3 (320 to 523.8)	27 (20 to 42)	15.1 (11.5 to 23.2)	3398 (3019 to 3870)	404.3 (360 to 463.2)	97 (84 to 119)	15.6 (13.3 to 19.2)	194.7 (142.4 to 254.2)	3.8 (-16.4 to 25.5)	255.2 (163.9 to 385.8)	3.2 (-25 to 33.9)
		Female	524 (410 to 682)	347.4 (268.1 to 483.5)	11 (7 to 18)	12.6 (8.4 to 21.6)	1416 (1209 to 1655)	335.4 (288 to 391)	36 (30 to 46)	11.6 (9.3 to 14.5)	170.3 (111.4 to 257.8)	-3.5 (-27.4 to 29.2)	222.7 (110 to 440.2)	-7.6 (-41 to 47.4)
		Male	629 (495 to 864)	431.5 (341.3 to 601.3)	16 (11 to 25)	18 (12.7 to 28)	1982 (1704 to 2333)	475.4 (410.4 to 577.2)	61 (51 to 82)	19.8 (16.5 to 26.9)	215.1 (146.2 to 304.4)	10.2 (-14.2 to 44.4)	278 (160.7 to 474.2)	10 (-20.6 to 55.6)
	Pneumoconiosis	Both	1 (1 to 2)	0.4 (0.3 to 0.6)	0 (0 to 0)	0 (0 to 0)	13 (9 to 17)	1.3 (1 to 1.7)	0 (0 to 0)	0 (0 to 0)	817.6 (549.6 to 1149.9)	184.4 (98.8 to 286.7)	448.8 (234.4 to 842.2)	78.6 (12 to 195)
		Female	1 (0 to 1)	0.3 (0.2 to 0.4)	0 (0 to 0)	0 (0 to 0)	10 (7 to 14)	1.9 (1.4 to 2.7)	0 (0 to 0)	0 (0 to 0)	1615 (1289.5 to 2096.2)	525 (413.9 to 664.2)	1720.3 (1018.9 to 3012)	419.4 (200.8 to 778.1)
		Male	1 (1 to 1)	0.6 (0.4 to 0.9)	0 (0 to 0)	0 (0 to 0)	3 (2 to 4)	0.7 (0.6 to 0.9)	0 (0 to 0)	0 (0 to 0)	254.6 (123.7 to 500)	21.4 (-20.1 to 99.2)	256.7 (109 to 593.9)	17.8 (-27.6 to 121.7)
	Asthma	Both	1908 (1410 to 2545)	433.1 (323.4 to 575.9)	30 (21 to 42)	14.2 (9.3 to 21.4)	2230 (1663 to 3024)	213.9 (164.3 to 281)	34 (28 to 40)	5 (4.1 to 6)	16.9 (-1 to 37.5)	-50.6 (-62.4 to -37.6)	13.6 (-23.6 to 64.2)	-64.5 (-78 to -48.2)
		Female	927 (663 to 1259)	427 (295.3 to 649.5)	14 (8 to 24)	13.6 (6.9 to 25.9)	1060 (774 to 1434)	208 (155.1 to 274.2)	16 (10 to 19)	4.5 (2.9 to 5.7)	14.3 (-10.6 to 40.9)	-51.3 (-68.1 to -34.2)	9.5 (-40.7 to 83.1)	-66.7 (-84.1 to -42.1)
		Male	981 (730 to 1348)	439 (336.5 to 596.2)	16 (11 to 24)	14.8 (10.5 to 22.9)	1170 (867 to 1607)	220.8 (168.6 to 292)	18 (15 to 25)	5.6 (4.5 to 7.7)	19.3 (-1.4 to 43.5)	-49.7 (-61.5 to -35.2)	17.4 (-19 to 80.8)	-62.5 (-74.6 to -44.1)

Province	Cause	Sex	1990				2019				1990-2019 percent change (%)			
			DALYs		Death		DALYs		Death		DALYs		Death	
			Number	Rate	Number	Rate	Number	Rate	Number	Rate	Number	Rate	Number	Rate
	Interstitial lung disease and pulmonary sarcoidosis	Both	39 (27 to 58)	11.5 (7.7 to 18.9)	1 (1 to 2)	0.4 (0.2 to 0.9)	130 (103 to 165)	13.7 (10.8 to 18.4)	4 (3 to 5)	0.5 (0.4 to 0.8)	236.4 (134.9 to 346.1)	19.1 (-19.6 to 68.3)	309.5 (155.9 to 551.4)	20.1 (-26.3 to 104.7)
		Female	18 (13 to 28)	11.5 (7 to 20)	0 (0 to 1)	0.4 (0.2 to 1)	56 (42 to 77)	11.7 (8.7 to 16.4)	1 (1 to 2)	0.4 (0.3 to 0.6)	207.9 (134.3 to 325.7)	2.1 (-28.9 to 58.1)	227.3 (106.6 to 511.8)	-11.6 (-47.5 to 84.7)
		Male	20 (13 to 36)	11.4 (7.3 to 21.7)	0 (0 to 1)	0.4 (0.2 to 1)	73 (53 to 97)	15.7 (11.5 to 22.3)	2 (2 to 4)	0.7 (0.4 to 1.1)	262.3 (111.7 to 430.1)	37.2 (-21.5 to 109.8)	387.8 (150.6 to 765.7)	54.7 (-19.7 to 187.4)
	Other chronic respiratory diseases	Both	72 (52 to 109)	10.4 (7.8 to 14.6)	0 (0 to 1)	0.1 (0 to 0.2)	274 (213 to 334)	21.9 (16.5 to 26.8)	2 (1 to 3)	0.2 (0.1 to 0.4)	279.3 (117.9 to 419.1)	110.5 (26.3 to 182.4)	519.4 (11.2 to 1635.4)	176.1 (-37.7 to 588.5)
		Female	39 (28 to 57)	11 (8.3 to 15.4)	0 (0 to 0)	0.1 (0 to 0.2)	139 (104 to 172)	23.2 (17.1 to 29)	1 (0 to 1)	0.2 (0.1 to 0.3)	259.5 (112.9 to 392.9)	111.7 (31.8 to 185.6)	557.7 (-0.4 to 1952.7)	168.9 (-43.3 to 611.8)
		Male	34 (22 to 53)	9.9 (7 to 14.7)	0 (0 to 0)	0.1 (0 to 0.2)	135 (101 to 170)	20.8 (15.2 to 26.3)	1 (0 to 2)	0.3 (0.1 to 0.4)	302 (123.2 to 496.4)	111 (20.7 to 204.4)	492 (23.4 to 1701.7)	182.6 (-36.2 to 779)
	Digestive diseases	Both	3349 (2677 to 3967)	785.6 (624.3 to 961.8)	67 (50 to 84)	26.7 (20.1 to 35.8)	5638 (4612 to 6892)	559.7 (467.6 to 668.5)	134 (115 to 158)	18.7 (16.1 to 21.8)	68.3 (36.3 to 115.3)	-28.8 (-41.9 to -11)	101.9 (58.5 to 175.6)	-29.9 (-47.2 to -6.4)
		Female	1563 (1214 to 1908)	724.8 (543.9 to 941.8)	28 (20 to 39)	23.8 (15.4 to 35.9)	2459 (1983 to 3067)	498.4 (412.2 to 608.2)	52 (45 to 62)	15 (12.9 to 17.6)	57.4 (28.6 to 100.2)	-31.2 (-45.7 to -14.3)	84.7 (35 to 166.4)	-36.7 (-58.3 to -5.1)
		Male	1786 (1381 to 2204)	842.1 (646.4 to 1041.7)	38 (28 to 48)	29.6 (21.8 to 37.4)	3179 (2618 to 3926)	621.5 (517.6 to 752.9)	82 (67 to 100)	22.5 (18.5 to 27.2)	77.9 (34 to 143.7)	-26.2 (-42.8 to -1.1)	114.7 (57.9 to 215.6)	-24.2 (-40.8 to 7)
	Cirrhosis and other chronic liver diseases	Both	1388 (998 to 1720)	347 (254.2 to 447.8)	38 (28 to 48)	14.2 (10.3 to 19.4)	2362 (1940 to 2845)	247.9 (206.6 to 295.6)	85 (70 to 101)	11.1 (9.2 to 13.1)	70.1 (28.8 to 152.3)	-28.5 (-45.8 to -0.7)	124.7 (69.9 to 219.2)	-22 (-42.6 to 8.1)
		Female	601 (411 to 787)	291.9 (196.5 to 426.9)	16 (10 to 23)	12.3 (7.4 to 19.8)	822 (685 to 988)	178.1 (148.5 to 211.6)	30 (25 to 36)	8.1 (6.7 to 9.6)	36.8 (-1.6 to 122.6)	-39 (-58.3 to -9.4)	91.7 (31.3 to 191.2)	-34.2 (-60.8 to 6.5)
		Male	788 (548 to 1042)	398.2 (281.9 to 522.9)	22 (15 to 29)	16.1 (11.3 to 21.4)	1540 (1218 to 1941)	316.2 (250.2 to 395.3)	55 (43 to 68)	14.1 (11.2 to 17.5)	95.5 (31.9 to 213.8)	-20.6 (-44.4 to 21.5)	148.1 (73.6 to 280.6)	-12.4 (-35.1 to 30)
	Upper digestive system diseases	Both	1015 (738 to 1396)	255 (188.2 to 340.2)	15 (11 to 20)	7.2 (5 to 9.9)	1735 (1140 to 2664)	155.3 (106 to 231.4)	17 (14 to 21)	2.7 (2.2 to 3.3)	71 (39.6 to 101.6)	-39.1 (-51.9 to -25.9)	14.8 (-15.4 to 61.5)	-61.7 (-72.7 to -45.5)
		Female	482 (336 to 693)	238.6 (163.2 to 340.3)	6 (4 to 9)	6 (3.5 to 10)	856 (544 to 1348)	159.1 (104.8 to 243.9)	7 (6 to 9)	2.4 (1.9 to 3)	77.4 (43.3 to 109.1)	-33.3 (-50.1 to -17.5)	22.7 (-20.4 to 102.5)	-60.1 (-76.7 to -28.8)
		Male	533 (381 to 722)	271.1 (198.8 to 354.5)	9 (6 to 12)	8.4 (5.9 to 11.5)	880 (575 to 1342)	152.6 (106 to 224.4)	10 (7 to 12)	3.1 (2.3 to 3.8)	65.2 (25.1 to 105.9)	-43.7 (-57 to -29.2)	9.5 (-22.8 to 58.4)	-63.1 (-73.4 to -46.7)
	Appendicitis	Both	137 (86 to 191)	23.7 (15.5 to 32.1)	3 (2 to 3)	0.8 (0.5 to 1.2)	111 (82 to 146)	10 (7.5 to 13.2)	2 (2 to 3)	0.3 (0.2 to 0.4)	-19.4 (-48.3 to 50.4)	-57.8 (-71.2 to -32.6)	-9.8 (-40.8 to 62)	-61.6 (-76.1 to -42.2)
		Female	58 (31 to 96)	19.8 (11.5 to 32.3)	1 (1 to 2)	0.7 (0.4 to 1.3)	48 (33 to 65)	8.9 (6.5 to 12)	1 (1 to 1)	0.3 (0.2 to 0.3)	-17.6 (-55 to 71.6)	-54.8 (-75.9 to -21.8)	-11.4 (-55.4 to 67.9)	-62.2 (-82.8 to -31.4)
		Male	80 (43 to 115)	27.6 (15.4 to 37.6)	1 (1 to 2)	0.9 (0.5 to 1.3)	63 (45 to 87)	11.1 (7.9 to 15.4)	1 (1 to 2)	0.4 (0.2 to 0.5)	-20.8 (-52.3 to 75)	-59.7 (-74.2 to -21.7)	-8.8 (-45.8 to 98.5)	-61.1 (-75.6 to -28)
	Paralytic ileus and intestinal obstruction	Both	349 (212 to 567)	45.6 (33.4 to 65.2)	5 (4 to 8)	1.5 (1.1 to 2.1)	277 (203 to 374)	30.4 (22.8 to 39.7)	8 (6 to 10)	1.1 (0.9 to 1.5)	-20.5 (-58.2 to 53.3)	-33.4 (-59.3 to 5.4)	45.9 (-12.8 to 130.9)	-25.3 (-48.6 to 6.7)
		Female	179 (95 to 313)	48.8 (31.3 to 73.3)	3 (2 to 4)	1.7 (1.1 to 2.7)	126 (86 to 175)	27.8 (19.6 to 37.7)	3 (2 to 4)	1 (0.7 to 1.3)	-29.5 (-66.6 to 52.6)	-43 (-66.7 to -2.4)	20.6 (-29.5 to 113.8)	-42.6 (-65.9 to -3.5)

Province	Cause	Sex	1990				2019				1990-2019 percent change (%)			
			DALYs		Death		DALYs		Death		DALYs		Death	
			Number	Rate	Number	Rate	Number	Rate	Number	Rate	Number	Rate	Number	Rate
		Male	170 (91 to 315)	42.3 (27.3 to 71.7)	2 (2 to 4)	1.3 (0.9 to 2.2)	151 (104 to 221)	33 (23.3 to 46.2)	4 (3 to 6)	1.3 (1 to 1.8)	-11 (-59.8 to 104.7)	-21.9 (-59.3 to 43.5)	74.1 (-15.7 to 231.9)	-1.6 (-43.3 to 52.2)
	Inguinal, femoral, and abdominal hernia	Both	116 (73 to 168)	17.8 (12.3 to 24.5)	0 (0 to 1)	0.2 (0.1 to 0.4)	192 (131 to 273)	16.9 (11.8 to 23.5)	1 (1 to 1)	0.2 (0.1 to 0.2)	65.9 (19.6 to 118.6)	-4.8 (-21.8 to 14.5)	106.6 (5.3 to 281.3)	-24 (-55.5 to 18.1)
		Female	44 (26 to 72)	12.8 (8.4 to 18.3)	0 (0 to 0)	0.2 (0.1 to 0.3)	65 (42 to 93)	11.8 (7.8 to 16.5)	0 (0 to 1)	0.1 (0.1 to 0.2)	48.8 (-12.9 to 123.7)	-7.9 (-34.5 to 19.7)	113.5 (-33.8 to 347.2)	-25.1 (-60.6 to 36.8)
		Male	72 (46 to 106)	22.5 (15.5 to 31.7)	0 (0 to 0)	0.3 (0.2 to 0.5)	127 (85 to 183)	21.7 (14.8 to 30.6)	1 (0 to 1)	0.2 (0.1 to 0.3)	76.3 (32.1 to 137.2)	-3.5 (-22.2 to 19.7)	102.2 (8.7 to 292.3)	-25.6 (-61.7 to 29.8)
	Inflammatory bowel disease	Both	39 (28 to 51)	9.7 (7 to 12.9)	0 (0 to 1)	0.1 (0.1 to 0.2)	116 (82 to 159)	10.3 (7.5 to 13.9)	1 (1 to 2)	0.2 (0.1 to 0.2)	199.4 (132 to 265)	6.4 (-13.4 to 28.6)	235.4 (79.4 to 430.5)	13.5 (-31.1 to 81)
		Female	19 (13 to 26)	9.6 (6.5 to 13.5)	0 (0 to 0)	0.1 (0.1 to 0.3)	54 (38 to 74)	10 (7.1 to 13.4)	1 (0 to 1)	0.1 (0.1 to 0.2)	183.9 (110.4 to 268.4)	4.1 (-20.3 to 35.5)	241.7 (68.2 to 566.5)	10 (-52.2 to 127.5)
		Male	20 (14 to 26)	9.9 (7.2 to 13)	0 (0 to 0)	0.2 (0.1 to 0.2)	62 (43 to 88)	10.8 (7.6 to 14.8)	1 (0 to 1)	0.2 (0.1 to 0.2)	214.2 (128.7 to 313.6)	9.1 (-17 to 39.3)	230.8 (69.9 to 455.4)	16.3 (-34.9 to 87.7)
	Vascular intestinal disorders	Both	25 (17 to 38)	11 (6.4 to 17.6)	1 (1 to 2)	0.6 (0.3 to 1.1)	64 (49 to 78)	8.2 (6.3 to 10)	3 (2 to 4)	0.5 (0.4 to 0.6)	151.9 (67.8 to 257)	-24.7 (-53.5 to 15.2)	184.6 (75 to 341.7)	-23.3 (-55.3 to 30.3)
		Female	13 (6 to 23)	12.7 (5.1 to 24.2)	1 (0 to 1)	0.8 (0.3 to 1.5)	30 (23 to 37)	7.8 (6 to 9.6)	2 (1 to 2)	0.5 (0.4 to 0.6)	125.1 (31.9 to 310.5)	-38.8 (-67 to 33.5)	139.9 (27.3 to 445.4)	-39 (-69 to 55.5)
		Male	12 (9 to 17)	9.1 (6.4 to 12.3)	0 (0 to 1)	0.5 (0.3 to 0.7)	34 (24 to 44)	8.7 (6.1 to 11.1)	2 (1 to 2)	0.5 (0.4 to 0.7)	181 (94.3 to 327.2)	-4.3 (-33.4 to 37.3)	245.7 (138.3 to 414.2)	3.6 (-27.5 to 47.4)
	Gallbladder and biliary diseases	Both	184 (123 to 263)	49 (33.6 to 67.5)	2 (1 to 3)	0.9 (0.6 to 1.4)	489 (345 to 675)	49.2 (36.3 to 65.3)	7 (5 to 9)	1.2 (0.9 to 1.5)	165.3 (107.1 to 218.5)	0.3 (-20.5 to 19.8)	365.2 (144.8 to 634.4)	37.1 (-18.7 to 111.9)
		Female	125 (82 to 178)	65.4 (43.8 to 92)	1 (0 to 1)	1 (0.6 to 1.6)	330 (225 to 462)	66.4 (47.3 to 90)	4 (3 to 5)	1.3 (0.8 to 1.7)	164 (107.6 to 220.3)	1.5 (-19.2 to 21.6)	380.3 (129 to 740)	39 (-25.8 to 147.1)
		Male	59 (40 to 86)	33.3 (23.4 to 47.9)	1 (0 to 1)	0.8 (0.5 to 1.5)	159 (116 to 218)	33.3 (25 to 43.8)	3 (2 to 4)	1.1 (0.8 to 1.5)	167.9 (93.2 to 250.4)	0.1 (-27.9 to 32.2)	347.7 (129.5 to 715.7)	35.6 (-26.9 to 135.2)
	Pancreatitis	Both	48 (30 to 79)	14.7 (9.2 to 24.9)	1 (1 to 3)	0.6 (0.4 to 1.2)	100 (76 to 157)	10.6 (8.3 to 16.5)	3 (3 to 6)	0.5 (0.4 to 0.8)	109.7 (53.9 to 200.5)	-27.7 (-48.9 to 5.7)	144.6 (66 to 276.3)	-22.5 (-50.6 to 26.3)
		Female	18 (11 to 33)	12.6 (7 to 23.7)	1 (0 to 1)	0.6 (0.3 to 1.3)	39 (29 to 61)	9 (6.8 to 14.2)	1 (1 to 2)	0.4 (0.3 to 0.8)	110 (39.6 to 229.7)	-28.8 (-57.1 to 19.3)	148.7 (40.8 to 348.8)	-25.7 (-62 to 48.3)
		Male	29 (18 to 50)	16.6 (10.6 to 27.8)	1 (1 to 1)	0.6 (0.4 to 1)	61 (44 to 101)	12.2 (9.1 to 20.2)	2 (1 to 3)	0.5 (0.4 to 0.8)	109.5 (39.9 to 214.6)	-26.7 (-50.1 to 7.1)	141.6 (59.2 to 267.3)	-18.1 (-45.9 to 22.1)
	Other digestive diseases	Both	48 (37 to 67)	12.1 (8.6 to 17.9)	1 (1 to 2)	0.5 (0.3 to 0.9)	193 (121 to 245)	20.7 (13.5 to 26)	6 (4 to 8)	0.9 (0.6 to 1.2)	300 (69.3 to 461.6)	71.3 (-13.1 to 129.8)	530.2 (165.7 to 845.5)	88.8 (-7.3 to 223.5)
		Female	24 (17 to 34)	12.6 (7.6 to 21.8)	1 (0 to 1)	0.5 (0.2 to 1.1)	91 (50 to 120)	19.7 (11.3 to 25.4)	3 (1 to 4)	0.8 (0.5 to 1.1)	281 (64.6 to 471.6)	55.4 (-19.9 to 144.9)	457.6 (132.3 to 959.8)	55.9 (-31.9 to 271.8)
		Male	24 (18 to 35)	11.5 (8.4 to 15.9)	0 (0 to 1)	0.4 (0.3 to 0.7)	102 (65 to 133)	21.9 (14.4 to 28.1)	3 (2 to 4)	1 (0.7 to 1.4)	318.5 (76.4 to 557.6)	90.6 (-10.8 to 181.9)	606.3 (201.2 to 1067.8)	134.8 (8.4 to 263.7)
	Neurological disorders	Both	6832 (3059 to 12251)	1378.9 (727.9 to 2334.5)	44 (23 to 94)	30.1 (11.7 to 74.7)	14165 (6914 to 24913)	1353.7 (731 to 2292.1)	163 (71 to 364)	31.8 (12.5 to 74.3)	107.3 (67.5 to 164.4)	-1.8 (-14.4 to 14.3)	272.1 (189 to 404.8)	5.4 (-7.7 to 38.6)
		Female	3764 (1593 to 7059)	1508.6 (761.6 to 2629.1)	22 (11 to 47)	28.8 (10.6 to 68.9)	7796 (3411 to 14336)	1515.7 (753.8 to 2621.5)	80 (33 to 186)	30.9 (11.2 to 74)	107.1 (71.3 to 156.7)	0.5 (-11.9 to 15.2)	266.1 (169.9 to 428.4)	7.6 (-9.1 to 51.5)

Province	Cause	Sex	1990				2019				1990-2019 percent change (%)			
			DALYs		Death		DALYs		Death		DALYs		Death	
			Number	Rate	Number	Rate	Number	Rate	Number	Rate	Number	Rate	Number	Rate
		Male	3068 (1471 to 5289)	1256.7 (696.8 to 2041)	22 (12 to 46)	32.1 (12.6 to 79.8)	6368 (3380 to 10787)	1213 (708.4 to 1965.7)	82 (39 to 177)	32.6 (13.9 to 74.8)	107.6 (64.1 to 174.7)	-3.5 (-17.8 to 15.3)	278.1 (187.7 to 408.5)	1.8 (-14.9 to 33.3)
	Alzheimer's disease and other dementias	Both	523 (226 to 1210)	372.3 (164.9 to 851.2)	27 (6 to 76)	24.4 (5.8 to 69.2)	2158 (953 to 4784)	384.3 (169.9 to 851.9)	118 (28 to 320)	25.2 (6 to 68.1)	312.7 (266.1 to 405.5)	3.2 (-6.2 to 26.8)	337.6 (278.3 to 506.9)	3.6 (-8.9 to 44.2)
		Female	275 (121 to 638)	376.5 (169.5 to 835.4)	15 (4 to 40)	24.1 (5.9 to 64.6)	1149 (512 to 2559)	398.4 (176.8 to 894.1)	63 (15 to 170)	25.9 (6.2 to 69.9)	317.5 (260.2 to 448)	5.8 (-6.4 to 38)	328.2 (253.8 to 535.4)	7.7 (-9 to 59.9)
		Male	248 (108 to 588)	370.4 (161.6 to 879.1)	12 (3 to 36)	25 (5.9 to 73.5)	1009 (439 to 2267)	369.5 (163.9 to 826.6)	56 (13 to 152)	24.5 (5.7 to 67.4)	307.3 (243.5 to 394.8)	-0.2 (-13.4 to 17.9)	348.7 (262.9 to 487.4)	-2.1 (-17.9 to 28)
	Parkinson's disease	Both	127 (102 to 152)	67.6 (55.4 to 83.4)	6 (4 to 7)	3.9 (3.2 to 5.2)	557 (478 to 638)	82.4 (71 to 94.3)	30 (25 to 35)	5.1 (4.3 to 5.9)	339.9 (249.1 to 455.7)	22 (-3.4 to 54.4)	431.8 (309.6 to 603.2)	30.4 (0.4 to 70.4)
		Female	50 (40 to 65)	52.6 (42.2 to 73.2)	2 (2 to 3)	3 (2.3 to 4.6)	219 (188 to 250)	62.5 (53.8 to 72)	11 (9 to 13)	3.7 (3.1 to 4.7)	336.8 (230.3 to 475.7)	18.8 (-11.7 to 54.2)	408.3 (269.8 to 598.9)	25.2 (-10.4 to 69.4)
		Male	77 (58 to 96)	84.2 (65 to 104.8)	3 (3 to 4)	5.1 (3.9 to 6.6)	338 (277 to 400)	103.5 (84.8 to 122.8)	19 (15 to 23)	6.6 (5.3 to 8)	341.9 (230.3 to 511.3)	23 (-9.5 to 66.5)	446.9 (294.6 to 686.9)	30.3 (-7.7 to 83.7)
	Idiopathic epilepsy	Both	1478 (747 to 2430)	176.5 (85.5 to 289.8)	8 (6 to 11)	1.2 (0.8 to 1.5)	1491 (584 to 2801)	123.4 (47.9 to 234.4)	7 (6 to 9)	0.6 (0.5 to 0.8)	0.9 (-59.2 to 151.9)	-30.1 (-71.8 to 75.6)	-19.8 (-43.3 to 33.6)	-49.1 (-61.2 to -21)
		Female	696 (342 to 1144)	166.9 (82.1 to 274.9)	4 (2 to 6)	1.1 (0.7 to 1.5)	663 (256 to 1256)	115.3 (44 to 218.2)	3 (2 to 4)	0.5 (0.4 to 0.7)	-4.8 (-62.5 to 133)	-30.9 (-72.7 to 69)	-30.5 (-56.6 to 53.5)	-54.1 (-68.2 to -16.5)
		Male	782 (380 to 1319)	185.9 (88.6 to 314.6)	4 (3 to 6)	1.3 (0.9 to 1.7)	829 (325 to 1557)	130.8 (51.2 to 246.1)	4 (3 to 5)	0.7 (0.6 to 1)	6 (-56.8 to 165)	-29.6 (-71.3 to 83.6)	-9.9 (-37.5 to 63.1)	-44.8 (-59 to -6.6)
	Multiple sclerosis	Both	83 (53 to 145)	19.5 (12.4 to 35.1)	1 (0 to 3)	0.3 (0.1 to 0.7)	238 (159 to 387)	19.5 (13.1 to 32)	3 (2 to 7)	0.3 (0.2 to 0.7)	187.9 (117.8 to 276.7)	-0.3 (-25.3 to 28.9)	246.1 (120.4 to 500.6)	13.3 (-29.7 to 102.6)
		Female	47 (31 to 76)	22 (14.3 to 37.4)	0 (0 to 1)	0.3 (0.1 to 0.7)	137 (102 to 191)	23.4 (17.4 to 32.9)	2 (1 to 3)	0.3 (0.2 to 0.6)	193.7 (94.8 to 300.1)	6.5 (-29.4 to 46.9)	255.6 (78.4 to 654.5)	15.8 (-37.4 to 156.1)
		Male	36 (20 to 76)	17.1 (9.3 to 36.9)	0 (0 to 2)	0.3 (0.1 to 0.8)	101 (55 to 221)	16 (8.7 to 34.9)	2 (1 to 5)	0.3 (0.1 to 0.9)	180.5 (82.5 to 284.1)	-6.4 (-40.3 to 27.4)	236.5 (72.8 to 462.5)	13 (-41.7 to 88.7)
	Motor neuron disease	Both	10 (8 to 13)	2.1 (1.6 to 2.7)	0 (0 to 0)	0 (0 to 0.1)	23 (20 to 27)	2.1 (1.8 to 2.4)	0 (0 to 1)	0 (0 to 0.1)	134.8 (74.5 to 215.4)	1.8 (-27.4 to 41.1)	178.1 (79.9 to 328.2)	4.7 (-33.9 to 64.8)
		Female	5 (4 to 7)	2 (1.4 to 2.8)	0 (0 to 0)	0 (0 to 0.1)	12 (10 to 14)	2.3 (1.9 to 2.7)	0 (0 to 0)	0.1 (0 to 0.1)	139.5 (69 to 239)	16.4 (-25.2 to 64.5)	219.6 (86 to 411.7)	29.4 (-30.1 to 112.9)
		Male	5 (4 to 6)	2.1 (1.5 to 3)	0 (0 to 0)	0.1 (0 to 0.1)	11 (9 to 13)	1.9 (1.6 to 2.3)	0 (0 to 0)	0 (0 to 0.1)	129.7 (55.2 to 219.5)	-10.3 (-42.5 to 34.5)	141.3 (34 to 323.5)	-13.1 (-50.3 to 53.2)
	Headache disorders	Both	4320 (892 to 9592)	704.5 (169.9 to 1508.7)	--	--	9188 (2225 to 19675)	698.5 (171.3 to 1496.8)	--	--	112.7 (95.7 to 154.5)	-0.9 (-5.9 to 5.5)	--	--
		Female	2570 (496 to 5754)	856.9 (191.3 to 1890.9)	--	--	5384 (1179 to 11707)	872.4 (193.4 to 1901.6)	--	--	109.5 (90.6 to 140.7)	1.8 (-5.4 to 8.4)	--	--
		Male	1751 (395 to 3817)	555.9 (150.2 to 1173.6)	--	--	3804 (1048 to 7915)	546 (152.1 to 1140.1)	--	--	117.3 (95.1 to 172.4)	-1.8 (-8.5 to 7.3)	--	--

Province	Cause	Sex	1990				2019				1990-2019 percent change (%)			
			DALYs		Death		DALYs		Death		DALYs		Death	
			Number	Rate	Number	Rate	Number	Rate	Number	Rate	Number	Rate	Number	Rate
	Other neurological disorders	Both	292 (137 to 494)	36.5 (20.1 to 58.5)	2 (1 to 2)	0.3 (0.2 to 0.4)	509 (272 to 862)	43.4 (24.4 to 72.1)	4 (3 to 4)	0.4 (0.4 to 0.5)	74.5 (-13.9 to 299.6)	19 (-36.2 to 143.6)	154.5 (78.1 to 292.8)	37.1 (-3.2 to 117.9)
		Female	122 (53 to 210)	31.7 (16.8 to 52.2)	1 (0 to 1)	0.2 (0.1 to 0.3)	233 (126 to 389)	41.4 (23.2 to 67.9)	2 (1 to 2)	0.4 (0.3 to 0.4)	90.7 (-7.2 to 381.6)	30.6 (-31.8 to 179.7)	233 (130.1 to 421.4)	56.4 (3.3 to 171.7)
		Male	170 (86 to 283)	41.2 (22.9 to 64.3)	1 (1 to 1)	0.4 (0.2 to 0.5)	277 (146 to 477)	45.3 (24.9 to 76.5)	2 (2 to 3)	0.4 (0.4 to 0.5)	62.9 (-17.5 to 254.3)	10 (-40.9 to 124.6)	114.4 (40 to 267.6)	25.4 (-14.9 to 106.3)
	Mental disorders	Both	14310 (10404 to 19004)	2296.7 (1696.6 to 3025.4)	0 (0 to 0)	0 (0 to 0)	30150 (22313 to 39764)	2330.8 (1723.2 to 3080)	0 (0 to 0)	0 (0 to 0)	110.7 (101.2 to 121.3)	1.5 (-1.2 to 4.3)	294.7 (78.9 to 910.2)	44.8 (-36.1 to 266.7)
		Female	7893 (5736 to 10584)	2598.5 (1915.8 to 3442.3)	0 (0 to 0)	0 (0 to 0)	16091 (11818 to 21215)	2652.8 (1956.6 to 3489.4)	0 (0 to 0)	0 (0 to 0)	103.9 (92.7 to 115.6)	2.1 (-1.7 to 6.1)	286.3 (51 to 1059.6)	55.9 (-39.3 to 447.7)
		Male	6417 (4661 to 8441)	2001.3 (1471.9 to 2630.6)	0 (0 to 0)	0 (0 to 0)	14059 (10306 to 18492)	2047.7 (1505.4 to 2701.3)	0 (0 to 0)	0 (0 to 0)	119.1 (107.2 to 131.1)	2.3 (-1.2 to 5.9)	317.6 (16 to 1422.2)	36.2 (-62.3 to 437.5)
	Schizophrenia	Both	817 (588 to 1050)	160.5 (115.8 to 204.9)	--	--	2206 (1610 to 2856)	162.4 (118.1 to 209)	--	--	170.1 (144.7 to 197.4)	1.2 (-7.2 to 9.7)	--	--
		Female	381 (269 to 491)	151.8 (108.9 to 193.6)	--	--	965 (695 to 1257)	153.3 (111.8 to 197.8)	--	--	153.4 (119.4 to 193.4)	1 (-10.7 to 14.6)	--	--
		Male	436 (314 to 563)	168.9 (122.4 to 215.1)	--	--	1240 (889 to 1607)	170.3 (123.1 to 218.9)	--	--	184.7 (150.8 to 221.9)	0.8 (-10.1 to 12.8)	--	--
	Depressive disorders	Both	5067 (3429 to 7041)	896 (611.7 to 1243.2)	--	--	12129 (8163 to 16977)	939.1 (634.3 to 1305.7)	--	--	139.4 (120.9 to 158.9)	4.8 (0.2 to 9.3)	--	--
		Female	3156 (2129 to 4397)	1118.1 (764.7 to 1555.6)	--	--	7003 (4714 to 9830)	1159.2 (785.1 to 1617.8)	--	--	121.9 (102.9 to 142.2)	3.7 (-2.5 to 9.8)	--	--
		Male	1911 (1276 to 2666)	679.6 (461.4 to 943.2)	--	--	5126 (3430 to 7212)	744 (504.2 to 1035.1)	--	--	168.3 (143.6 to 193)	9.5 (3.1 to 16.3)	--	--
	Bipolar disorder	Both	1009 (613 to 1572)	173.2 (107.5 to 267.7)	--	--	2280 (1396 to 3500)	174.2 (107.3 to 267.2)	--	--	125.9 (105.6 to 148)	0.5 (-6.4 to 7.8)	--	--
		Female	546 (326 to 855)	186.2 (112.6 to 289.3)	--	--	1148 (704 to 1774)	187.7 (114.9 to 287.9)	--	--	110.2 (86.7 to 139)	0.8 (-8 to 10.9)	--	--
		Male	463 (282 to 708)	160.4 (99.5 to 247)	--	--	1132 (694 to 1743)	162.3 (99.7 to 245.7)	--	--	144.5 (115.6 to 175.6)	1.2 (-8.7 to 10.6)	--	--
	Anxiety disorders	Both	4456 (3097 to 6171)	674.7 (471.9 to 925.3)	--	--	8865 (6111 to 12231)	689.1 (483.4 to 951.2)	--	--	98.9 (82.2 to 117)	2.1 (-3.8 to 8.4)	--	--
		Female	2658 (1829 to 3697)	826.1 (577.3 to 1136.8)	--	--	5183 (3552 to 7144)	855.8 (589.1 to 1176)	--	--	95 (74.8 to 115.9)	3.6 (-4.9 to 11.8)	--	--

Province	Cause	Sex	1990				2019				1990-2019 percent change (%)			
			DALYs		Death		DALYs		Death		DALYs		Death	
			Number	Rate	Number	Rate	Number	Rate	Number	Rate	Number	Rate	Number	Rate
		Male	1799 (1246 to 2491)	526.7 (367.2 to 724.9)	--	--	3682 (2549 to 5168)	541.6 (379.4 to 756.7)	--	--	104.7 (81.6 to 128.2)	2.8 (-6.3 to 12.3)	--	--
	Eating disorders	Both	314 (190 to 477)	42.7 (26.1 to 64.2)	0 (0 to 0)	0 (0 to 0)	753 (460 to 1132)	53.4 (32.8 to 80.7)	0 (0 to 0)	0 (0 to 0)	139.8 (107 to 175.4)	25.1 (11.6 to 38.8)	294.7 (78.9 to 910.2)	44.8 (-36.1 to 266.7)
		Female	194 (116 to 292)	53 (32.4 to 79.1)	0 (0 to 0)	0 (0 to 0)	440 (273 to 657)	67.9 (42.1 to 101.8)	0 (0 to 0)	0 (0 to 0)	126.9 (92.1 to 166.3)	28.1 (11.3 to 47.3)	286.3 (51 to 1059.6)	55.9 (-39.3 to 447.7)
		Male	120 (72 to 186)	32.4 (19.6 to 49.3)	0 (0 to 0)	0 (0 to 0)	313 (187 to 489)	40.9 (24.4 to 63.4)	0 (0 to 0)	0 (0 to 0)	160.6 (113.3 to 215.5)	26 (6.7 to 49.3)	317.6 (16 to 1422.2)	36.2 (-62.3 to 437.5)
	Autism spectrum disorders	Both	352 (228 to 520)	44.8 (29.2 to 65.6)	--	--	581 (378 to 845)	46 (29.9 to 67.1)	--	--	65.1 (48 to 84.8)	2.8 (-7.5 to 14.1)	--	--
		Female	87 (55 to 129)	22.4 (14.3 to 33.2)	--	--	134 (87 to 195)	22.5 (14.6 to 32.7)	--	--	54 (29.5 to 83.3)	0.4 (-15 to 17.8)	--	--
		Male	265 (170 to 390)	66.5 (43.2 to 97.6)	--	--	447 (289 to 652)	67.3 (43.7 to 97.5)	--	--	68.7 (48.2 to 94.5)	1.2 (-10.4 to 15.4)	--	--
	Attention-deficit/hyperactivity disorder	Both	286 (161 to 490)	32.4 (18.4 to 55.2)	--	--	442 (249 to 748)	34 (19.2 to 57.6)	--	--	54.4 (32.5 to 79.5)	4.9 (-9 to 19.2)	--	--
		Female	87 (48 to 150)	20.1 (11.2 to 34.6)	--	--	128 (70 to 213)	20.9 (11.5 to 34.8)	--	--	47.5 (21.2 to 78.5)	4 (-13.9 to 23.7)	--	--
		Male	200 (112 to 339)	44.5 (25 to 75)	--	--	314 (178 to 538)	45.8 (26.1 to 78.5)	--	--	57.4 (29.8 to 92.7)	2.9 (-14.6 to 22.4)	--	--
	Conduct disorder	Both	794 (450 to 1243)	75.7 (42.9 to 118.6)	--	--	906 (517 to 1424)	74.8 (42.6 to 117.6)	--	--	14.1 (3.6 to 24.8)	-1.2 (-10.3 to 8)	--	--
		Female	271 (141 to 446)	52.7 (27.2 to 85.9)	--	--	298 (159 to 495)	51 (27.3 to 84.8)	--	--	10 (-5.4 to 27.8)	-3.1 (-16.7 to 12.4)	--	--
		Male	523 (305 to 817)	97.9 (57.1 to 153.4)	--	--	608 (349 to 948)	96.7 (55.7 to 150.7)	--	--	16.2 (2.9 to 30)	-1.2 (-12.4 to 10.6)	--	--
	Idiopathic developmental intellectual disability	Both	759 (415 to 1220)	95.9 (52.9 to 153.4)	--	--	716 (364 to 1196)	56.3 (28.6 to 94.2)	--	--	-5.6 (-20.1 to 4.5)	-41.3 (-50.4 to -34.8)	--	--
		Female	332 (183 to 540)	85.4 (46.8 to 137.9)	--	--	309 (156 to 514)	51.5 (26 to 86)	--	--	-7 (-21.3 to 4.4)	-39.6 (-49.2 to -32.4)	--	--
		Male	427 (234 to 688)	106.1 (58.5 to 170.9)	--	--	408 (204 to 688)	60.6 (30.2 to 102.1)	--	--	-4.6 (-21.1 to 7.8)	-42.9 (-52.9 to -35.6)	--	--
	Other mental disorders	Both	456 (289 to 690)	100.7 (65 to 152.6)	--	--	1273 (818 to 1950)	101.5 (65.6 to 154.6)	--	--	179.1 (158.2 to 200.5)	0.8 (-4.5 to 6.4)	--	--
		Female	182 (115 to 279)	82.7 (52.4 to 124.3)	--	--	484 (305 to 745)	83 (53.1 to 126)	--	--	165.5 (138.9 to 197.2)	0.3 (-8.1 to 8.9)	--	--
		Male	274 (171 to 416)	118.3 (76 to 178.2)	--	--	789 (500 to 1196)	118.2 (75.9 to 178.6)	--	--	188.2 (161.8 to 215.1)	-0.1 (-6.8 to 6.9)	--	--
	Substance use disorders	Both	1527 (1158 to 1918)	267.8 (207.2 to 329.7)	11 (7 to 15)	2.6 (1.9 to 3.4)	3899 (3035 to 4866)	284.5 (223.9 to 352.5)	27 (23 to 32)	2.3 (1.9 to 2.6)	155.4 (122 to 201.5)	6.2 (-8.1 to 24.4)	142.9 (77.8 to 263.2)	-13.8 (-35.7 to 20.4)
		Female	520 (373 to 678)	178.5 (131.3 to 226.5)	3 (2 to 5)	1.6 (1.1 to 2.2)	987 (733 to 1278)	156.1 (116.8 to 200.7)	5 (4 to 6)	0.9 (0.7 to 1.1)	89.9 (57 to 132)	-12.6 (-26.5 to 5.3)	40.5 (-7 to 143.3)	-45 (-61.5 to -17.2)

Province	Cause	Sex	1990				2019				1990-2019 percent change (%)			
			DALYs		Death		DALYs		Death		DALYs		Death	
			Number	Rate	Number	Rate	Number	Rate	Number	Rate	Number	Rate	Number	Rate
		Male	1007 (759 to 1258)	355.4 (270.5 to 439.8)	8 (5 to 10)	3.6 (2.4 to 4.8)	2912 (2293 to 3662)	396.4 (311.3 to 493.1)	22 (18 to 27)	3.5 (2.9 to 4.2)	189.2 (143.1 to 249.2)	11.6 (-7.2 to 35)	187.6 (98.7 to 346.2)	-2.8 (-31.6 to 43.2)
	Alcohol use disorders	Both	259 (180 to 347)	48 (34.4 to 63.4)	1 (1 to 2)	0.3 (0.2 to 0.4)	519 (362 to 707)	38.3 (26.9 to 51.8)	2 (2 to 3)	0.2 (0.2 to 0.3)	100.3 (73.1 to 131.8)	-20.1 (-30.7 to -6.9)	109.1 (43.7 to 262.1)	-27.4 (-49.7 to 20.6)
		Female	67 (45 to 95)	23.2 (16 to 32)	0 (0 to 0)	0.1 (0 to 0.1)	141 (92 to 204)	22.4 (14.8 to 31.9)	0 (0 to 0)	0 (0 to 0)	111.3 (75.8 to 158.6)	-3.4 (-18.5 to 14.2)	36.2 (-3.4 to 129)	-44 (-58.1 to -16.2)
		Male	192 (135 to 258)	72.1 (51.8 to 95.4)	1 (1 to 2)	0.5 (0.3 to 0.8)	377 (266 to 511)	52.3 (37.5 to 69.5)	2 (2 to 3)	0.4 (0.3 to 0.6)	96.5 (65.6 to 135.9)	-27.4 (-39 to -12.6)	118.8 (44.6 to 293.3)	-26.2 (-50.3 to 26.5)
	Drug use disorders	Both	1268 (950 to 1614)	219.8 (169.6 to 274.8)	10 (7 to 13)	2.4 (1.7 to 3)	3380 (2621 to 4234)	246.2 (191.8 to 306.7)	25 (21 to 29)	2.1 (1.7 to 2.4)	166.7 (127.1 to 220.4)	12 (-4.6 to 34.3)	146.6 (79 to 271.8)	-12.1 (-35.3 to 24.9)
		Female	453 (318 to 599)	155.3 (113.3 to 200.8)	3 (2 to 5)	1.6 (1.1 to 2.1)	846 (622 to 1109)	133.7 (98.8 to 173.7)	5 (4 to 6)	0.9 (0.7 to 1)	86.8 (51.8 to 135.5)	-13.9 (-29.1 to 7.5)	40.7 (-7.2 to 146.7)	-45 (-61.9 to -16.8)
		Male	815 (605 to 1059)	283.2 (213.2 to 355.4)	7 (4 to 9)	3.1 (2.1 to 4.2)	2534 (1971 to 3194)	344.1 (270.2 to 430.9)	20 (16 to 25)	3.2 (2.6 to 3.8)	211.1 (155.8 to 284.6)	21.5 (-0.9 to 51.4)	197.5 (100.4 to 368.6)	1 (-30.1 to 51.4)
	Diabetes and kidney diseases	Both	3559 (2939 to 4186)	996.1 (840.2 to 1176.5)	65 (53 to 79)	28 (23.5 to 35.2)	13503 (11346 to 15927)	1501 (1273.1 to 1764.7)	294 (259 to 330)	41.8 (37.1 to 46.8)	279.4 (222 to 341.5)	50.7 (28.4 to 72.3)	352.2 (250.6 to 471.4)	49.3 (11.8 to 83.4)
		Female	1738 (1407 to 2096)	977.6 (795.4 to 1210.6)	30 (23 to 40)	26.1 (20.5 to 38.4)	6780 (5572 to 8145)	1525.8 (1264.8 to 1824.7)	141 (117 to 162)	40 (33.3 to 45.8)	290.2 (223.3 to 366.6)	56.1 (23.4 to 81.6)	371 (206.1 to 524.6)	53.3 (-7.5 to 102.6)
		Male	1822 (1442 to 2194)	1013.5 (818.1 to 1215)	35 (26 to 45)	30.1 (22.2 to 37)	6723 (5620 to 7958)	1480 (1252.4 to 1734.9)	153 (132 to 178)	43.8 (37.6 to 50.9)	269 (205.4 to 345)	46 (22.9 to 75.9)	336.1 (237.2 to 490.3)	45.5 (15.2 to 96.6)
	Diabetes mellitus	Both	1399 (1074 to 1770)	441.6 (342.9 to 561)	16 (13 to 21)	6.9 (5.5 to 9.9)	9455 (7648 to 11637)	1046.6 (850.4 to 1278.7)	174 (147 to 199)	24 (20.5 to 27.3)	575.8 (469.5 to 673)	137 (92.2 to 169.7)	981.7 (546.9 to 1349)	247.6 (99 to 356.9)
		Female	696 (533 to 889)	449.9 (346.5 to 580.1)	8 (6 to 13)	7.2 (5.2 to 12.3)	4903 (3864 to 6093)	1102 (878 to 1354.5)	88 (65 to 105)	24.3 (18.6 to 28.8)	604 (408.5 to 722.9)	145 (68.7 to 185.6)	971.7 (305.3 to 1501)	235.6 (24.4 to 399.9)
		Male	703 (532 to 893)	433.7 (333 to 546)	8 (6 to 10)	6.6 (4.9 to 8.4)	4552 (3636 to 5571)	992.5 (802.5 to 1203.6)	85 (71 to 102)	23.7 (19.6 to 28.2)	547.8 (461 to 661.6)	128.8 (98.2 to 169.6)	992.2 (697.8 to 1487.8)	260.2 (166.2 to 394.9)
	Chronic kidney disease	Both	2157 (1757 to 2546)	554.1 (467.5 to 647.7)	49 (40 to 59)	21.1 (17.5 to 26.6)	3991 (3494 to 4537)	448.9 (396.5 to 507)	118 (104 to 134)	17.7 (15.3 to 20)	85.1 (54.9 to 127.6)	-19 (-32 to -4)	142.4 (95.6 to 202)	-16.3 (-37 to 3.1)
		Female	1040 (828 to 1276)	527.4 (426.2 to 682.8)	22 (17 to 30)	18.9 (14.4 to 30.6)	1852 (1562 to 2142)	418.8 (358.2 to 478.6)	52 (44 to 60)	15.6 (13.1 to 18.1)	78.1 (42.7 to 126.9)	-20.6 (-40.1 to -1.4)	139.6 (64.4 to 222.4)	-17.4 (-52.8 to 10.1)
		Male	1117 (835 to 1391)	579.1 (439.1 to 707.1)	27 (20 to 35)	23.5 (16.9 to 29.4)	2139 (1846 to 2468)	481.4 (417.3 to 558)	66 (56 to 79)	19.9 (16.8 to 23.6)	91.6 (50.2 to 154.8)	-16.9 (-33.9 to 7.7)	144.7 (85.2 to 241.1)	-15.4 (-35.2 to 15.5)
	Acute glomerulonephritis	Both	4 (1 to 45)	0.5 (0.1 to 5.6)	0 (0 to 1)	0 (0 to 0.1)	57 (17 to 76)	5.5 (1.7 to 7.2)	2 (0 to 2)	0.2 (0.1 to 0.3)	1491 (-62 to 8745.5)	1072.9 (-67.7 to 4516.1)	2913.1 (-29.2 to 14647.2)	1574.4 (-53.6 to 6399.7)
		Female	1 (0 to 14)	0.3 (0.1 to 3.6)	0 (0 to 0)	0 (0 to 0.1)	24 (8 to 35)	5 (1.7 to 7.1)	1 (0 to 1)	0.2 (0.1 to 0.3)	2130.2 (-44.8 to 10069.8)	1505.7 (-55.4 to 5840.5)	4101.2 (1.5 to 17765.8)	2187.7 (-37.6 to 8601.3)

Province	Cause	Sex	1990				2019				1990-2019 percent change (%)			
			DALYs		Death		DALYs		Death		DALYs		Death	
			Number	Rate	Number	Rate	Number	Rate	Number	Rate	Number	Rate	Number	Rate
		Male	2 (0 to 33)	0.6 (0.1 to 7.3)	0 (0 to 0)	0 (0 to 0.2)	32 (6 to 49)	6.1 (1.3 to 9)	1 (0 to 1)	0.2 (0 to 0.4)	1207.4 (-78.4 to 12795.8)	862.3 (-80.8 to 6379.8)	2375.2 (-61.1 to 21395.3)	1250.4 (-72.6 to 8367.7)
	Skin and subcutaneous diseases	Both	3100 (2047 to 4466)	417.2 (281 to 609.5)	1 (1 to 1)	0.6 (0.4 to 0.8)	5144 (3429 to 7663)	424.7 (282.5 to 631.6)	4 (2 to 5)	0.6 (0.3 to 0.7)	65.9 (58.8 to 72)	1.8 (-1.3 to 4.6)	237.4 (124.9 to 391.3)	0.4 (-35.8 to 63)
		Female	1624 (1078 to 2339)	442.8 (300.2 to 644.6)	1 (0 to 1)	0.7 (0.4 to 1)	2582 (1721 to 3836)	447.7 (299.8 to 668.1)	2 (1 to 2)	0.6 (0.2 to 0.7)	59 (51.3 to 65.7)	1.1 (-2.9 to 4.6)	181.1 (53.3 to 386.9)	-20.1 (-60.7 to 64.5)
		Male	1477 (973 to 2170)	392.3 (261.5 to 575.3)	1 (0 to 1)	0.5 (0.3 to 0.7)	2562 (1690 to 3802)	403.8 (268.7 to 598.2)	2 (1 to 3)	0.7 (0.3 to 0.8)	73.5 (65.6 to 81.6)	2.9 (-1.1 to 6.8)	304.9 (180.6 to 544.6)	32.4 (-11.8 to 118.4)
	Dermatitis	Both	714 (417 to 1128)	93.6 (56.3 to 142.8)	--	--	1167 (710 to 1785)	93.7 (56.7 to 143.7)	--	--	63.5 (51 to 78.3)	0.1 (-4.9 to 5.1)	--	--
		Female	390 (227 to 617)	103.8 (62.5 to 159.6)	--	--	616 (373 to 950)	104.4 (63.4 to 160.9)	--	--	58 (43.7 to 74.1)	0.6 (-5.5 to 7.9)	--	--
		Male	324 (189 to 511)	83.7 (50.4 to 127.5)	--	--	550 (334 to 837)	84 (51.1 to 128.2)	--	--	70.1 (54 to 90.1)	0.4 (-6.5 to 7.9)	--	--
	Psoriasis	Both	257 (181 to 349)	45.6 (32.3 to 60.7)	--	--	396 (276 to 529)	32 (22.5 to 42.6)	--	--	53.9 (37 to 72.9)	-29.8 (-36.7 to -22.6)	--	--
		Female	134 (93 to 182)	47.2 (33.7 to 63.2)	--	--	193 (137 to 261)	33.1 (23.4 to 44.4)	--	--	44.8 (23.6 to 70.5)	-29.8 (-39 to -19)	--	--
		Male	124 (85 to 169)	44.1 (30.4 to 59.3)	--	--	203 (139 to 274)	31 (21.5 to 41.5)	--	--	63.8 (37.4 to 95.9)	-29.6 (-38.9 to -18)	--	--
	Bacterial skin diseases	Both	64 (42 to 93)	13.2 (8.1 to 18)	1 (0 to 1)	0.5 (0.2 to 0.7)	109 (76 to 155)	10.5 (7.5 to 14.4)	2 (1 to 3)	0.3 (0.2 to 0.5)	70.1 (43.2 to 106.7)	-20.2 (-38.1 to 13.7)	129.7 (62 to 308.6)	-34.2 (-58.3 to 51.3)
		Female	30 (20 to 44)	13.6 (7.9 to 19.2)	1 (0 to 1)	0.6 (0.2 to 0.9)	45 (30 to 67)	9.1 (6 to 13.2)	1 (0 to 2)	0.3 (0.1 to 0.5)	50.7 (15.6 to 109.1)	-32.8 (-56.3 to 24.8)	83.1 (0 to 379.9)	-49.5 (-75.6 to 84.9)
		Male	34 (20 to 50)	12.7 (7.3 to 17.5)	0 (0 to 1)	0.4 (0.2 to 0.6)	64 (38 to 90)	11.9 (7.4 to 16.2)	1 (1 to 2)	0.4 (0.2 to 0.5)	87.2 (50 to 142.1)	-6.6 (-26.8 to 24.4)	182.3 (96.2 to 343.2)	-11.8 (-40.8 to 44.6)
	Scabies	Both	199 (109 to 319)	25.2 (13.8 to 40.7)	--	--	314 (172 to 508)	25.1 (13.9 to 40.4)	--	--	58.3 (47.2 to 71.2)	-0.5 (-4.8 to 4.3)	--	--
		Female	98 (53 to 161)	25.3 (13.8 to 40.8)	--	--	149 (82 to 238)	25.1 (14 to 40.1)	--	--	51 (38.3 to 65.6)	-0.5 (-6.7 to 6.5)	--	--
		Male	100 (54 to 161)	25.1 (13.8 to 40.4)	--	--	166 (90 to 267)	25 (13.7 to 40.5)	--	--	65.4 (51.4 to 82.2)	-0.4 (-6.6 to 6.7)	--	--
	Fungal skin diseases	Both	94 (37 to 199)	18.8 (7.6 to 40.1)	--	--	197 (78 to 428)	18.5 (7.4 to 39.3)	--	--	110.7 (97.2 to 123.8)	-1.8 (-3.8 to 0)	--	--
		Female	42 (17 to 91)	17.6 (7.1 to 38.3)	--	--	88 (35 to 190)	17.3 (6.9 to 37.4)	--	--	106.6 (92.2 to 121.1)	-1.9 (-4.6 to 0.8)	--	--
		Male	51 (20 to 110)	20.1 (8.1 to 42.6)	--	--	109 (44 to 235)	19.7 (7.9 to 42.2)	--	--	114.1 (99.5 to 129.4)	-2 (-4.5 to 0.3)	--	--
	Viral skin diseases	Both	497 (316 to 750)	51.2 (32.8 to 76.9)	--	--	631 (399 to 936)	51.4 (32.5 to 76.3)	--	--	27 (21.2 to 33.3)	0.3 (-4 to 4.7)	--	--
		Female	241 (152 to 363)	50.8 (32.1 to 76.1)	--	--	299 (191 to 449)	51 (32.5 to 76.4)	--	--	24.1 (16.2 to 32.8)	0.3 (-5.5 to 6.8)	--	--
		Male	255 (161 to 384)	51.6 (32.7 to 77.5)	--	--	331 (211 to 491)	51.8 (33 to 77)	--	--	29.8 (21.4 to 38.2)	0.3 (-5.8 to 6)	--	--

Province	Cause	Sex	1990				2019				1990-2019 percent change (%)			
			DALYs		Death		DALYs		Death		DALYs		Death	
			Number	Rate	Number	Rate	Number	Rate	Number	Rate	Number	Rate	Number	Rate
	Acne vulgaris	Both	462 (276 to 728)	51.4 (30.7 to 81)	--	--	879 (522 to 1388)	70.9 (41.9 to 112.4)	--	--	90.5 (75.7 to 106.4)	38 (27.9 to 49.5)	--	--
		Female	220 (133 to 348)	49.9 (30 to 78.9)	--	--	403 (238 to 634)	68.5 (40.5 to 108.2)	--	--	82.8 (63.4 to 103.6)	37.3 (23.1 to 53.1)	--	--
		Male	241 (142 to 378)	52.8 (31.2 to 83.1)	--	--	477 (285 to 753)	73 (43.7 to 115.7)	--	--	97.5 (76.5 to 123.2)	38.2 (24.1 to 55)	--	--
	Alopecia areata	Both	40 (25 to 59)	6.4 (4 to 9.4)	--	--	83 (52 to 126)	6.3 (4 to 9.4)	--	--	110.7 (87.2 to 137.4)	-1.2 (-11.1 to 9.5)	--	--
		Female	25 (16 to 38)	8.3 (5.3 to 12.2)	--	--	52 (33 to 79)	8.3 (5.3 to 12.6)	--	--	102.6 (73.4 to 132.9)	0.1 (-12.3 to 13.8)	--	--
		Male	14 (9 to 22)	4.5 (2.8 to 6.8)	--	--	32 (20 to 48)	4.5 (2.8 to 6.8)	--	--	125.4 (84.5 to 178.2)	0.5 (-15.3 to 20.4)	--	--
	Pruritus	Both	58 (27 to 106)	10.1 (4.8 to 18.3)	--	--	126 (60 to 225)	10.6 (5.1 to 19)	--	--	116.2 (99.6 to 134.7)	4.9 (-0.3 to 10.6)	--	--
		Female	31 (15 to 57)	11 (5.2 to 19.8)	--	--	65 (31 to 118)	11.6 (5.5 to 20.7)	--	--	108.2 (89.6 to 129.7)	4.9 (-2.3 to 12.5)	--	--
		Male	27 (13 to 49)	9.1 (4.3 to 16.7)	--	--	60 (29 to 110)	9.6 (4.6 to 17.5)	--	--	125.5 (102.9 to 151.6)	5.4 (-1.6 to 13.5)	--	--
	Urticaria	Both	492 (320 to 717)	58.1 (38 to 83.1)	--	--	719 (471 to 1034)	58 (38.1 to 83.8)	--	--	46.1 (34.4 to 58.7)	-0.2 (-5.7 to 5.4)	--	--
		Female	299 (193 to 435)	70.9 (46.6 to 102.2)	--	--	419 (275 to 606)	71.1 (46.7 to 103.4)	--	--	40.3 (27.6 to 54.2)	0.3 (-6.6 to 7.6)	--	--
		Male	194 (125 to 283)	45.8 (29.6 to 65.3)	--	--	300 (196 to 434)	46 (30.1 to 66.5)	--	--	55.1 (39.1 to 73.7)	0.4 (-8 to 9.4)	--	--
	Decubitus ulcer	Both	3 (2 to 5)	1.5 (1 to 2.3)	0 (0 to 0)	0.1 (0 to 0.2)	28 (8 to 38)	4.2 (1.3 to 5.7)	1 (0 to 2)	0.3 (0.1 to 0.4)	863 (127.5 to 1440.5)	186.2 (-34.3 to 372.4)	1367.4 (93 to 2963.5)	222.5 (-51.4 to 558.9)
		Female	2 (1 to 3)	1.6 (0.9 to 2.8)	0 (0 to 0)	0.1 (0 to 0.2)	14 (3 to 20)	4 (1 to 5.8)	1 (0 to 1)	0.3 (0 to 0.4)	798.9 (86.3 to 1723.9)	150.1 (-49.1 to 437.7)	1040.7 (11 to 3110.9)	161.2 (-72.3 to 613.4)
		Male	1 (1 to 2)	1.3 (0.9 to 2.3)	0 (0 to 0)	0.1 (0 to 0.2)	15 (4 to 20)	4.4 (1.3 to 5.9)	1 (0 to 1)	0.3 (0.1 to 0.4)	930.2 (152.6 to 1437.3)	236.5 (-17.7 to 410.6)	1943.2 (167.9 to 3894.1)	337.5 (-32.2 to 774.7)
	Other skin and subcutaneous diseases	Both	222 (106 to 407)	42.2 (20.2 to 76.9)	0 (0 to 0)	0 (0 to 0.1)	494 (236 to 901)	43.6 (20.9 to 79.4)	0 (0 to 0)	0 (0 to 0)	122.3 (115.9 to 129.1)	3.4 (0.8 to 5.7)	52.4 (-18.8 to 277.2)	-68.4 (-83.9 to -7.9)
		Female	111 (53 to 202)	42.8 (20.7 to 77.4)	0 (0 to 0)	0 (0 to 0.1)	239 (116 to 434)	44.2 (21.4 to 80.3)	0 (0 to 0)	0 (0 to 0)	116.3 (107.8 to 124.9)	3.2 (-0.4 to 6.6)	36.7 (-47.3 to 436.3)	-72 (-89.7 to 27.4)
		Male	112 (53 to 205)	41.5 (19.7 to 76.4)	0 (0 to 0)	0 (0 to 0)	255 (121 to 469)	43 (20.5 to 79.2)	0 (0 to 0)	0 (0 to 0)	128.3 (119.4 to 137.5)	3.6 (0.3 to 6.9)	84.5 (-0.7 to 247.2)	-58 (-79.3 to -12.7)
	Sense organ diseases	Both	3601 (2552 to 4874)	1036.3 (745.3 to 1380.5)	--	--	7271 (5066 to 10015)	800.4 (566 to 1088.6)	--	--	101.9 (89.8 to 113.9)	-22.8 (-25.8 to -19.5)	--	--

Province	Cause	Sex	1990				2019				1990-2019 percent change (%)			
			DALYs		Death		DALYs		Death		DALYs		Death	
			Number	Rate	Number	Rate	Number	Rate	Number	Rate	Number	Rate	Number	Rate
		Female	1760 (1242 to 2375)	1039.2 (749.9 to 1387.6)	--	--	3546 (2478 to 4832)	800.7 (566.5 to 1079.7)	--	--	101.5 (88.3 to 113.9)	-23 (-26.3 to -19.5)	--	--
		Male	1841 (1305 to 2494)	1032.4 (737.6 to 1376.9)	--	--	3726 (2597 to 5164)	799.8 (565.5 to 1081.8)	--	--	102.4 (89.1 to 115.5)	-22.5 (-25.9 to -18.9)	--	--
	Blindness and vision loss	Both	1636 (1161 to 2226)	500.7 (360.4 to 670.7)	--	--	3196 (2257 to 4351)	365.7 (260.5 to 493.1)	--	--	95.3 (87.1 to 103.4)	-27 (-29.9 to -23.8)	--	--
		Female	843 (598 to 1146)	528 (378 to 706.8)	--	--	1663 (1178 to 2247)	386.1 (275.6 to 519.9)	--	--	97.3 (88 to 106.7)	-26.9 (-30.2 to -23.4)	--	--
		Male	794 (562 to 1086)	474.2 (338.8 to 635.2)	--	--	1533 (1079 to 2106)	345.3 (245.6 to 467.6)	--	--	93.2 (83.2 to 102.7)	-27.2 (-30.6 to -23.8)	--	--
	Age-related and other hearing loss	Both	1776 (1234 to 2450)	494.2 (353.1 to 684.9)	--	--	3616 (2499 to 5082)	391.4 (272.6 to 545)	--	--	103.6 (81.2 to 126.2)	-20.8 (-25 to -16.5)	--	--
		Female	817 (563 to 1128)	466.3 (330.3 to 642.6)	--	--	1644 (1134 to 2320)	367.7 (256 to 513)	--	--	101.1 (76.4 to 126.4)	-21.1 (-25.9 to -16.1)	--	--
		Male	959 (659 to 1320)	520.2 (371.8 to 718.6)	--	--	1973 (1356 to 2771)	414.7 (287.7 to 586.2)	--	--	105.7 (83.6 to 128.8)	-20.3 (-25.4 to -15.3)	--	--
	Other sense organ diseases	Both	188 (114 to 286)	41.4 (24.7 to 63.8)	--	--	459 (272 to 708)	43.2 (25.8 to 67.1)	--	--	144 (122 to 164.6)	4.4 (-0.6 to 9.7)	--	--
		Female	100 (60 to 153)	44.9 (26.7 to 70.2)	--	--	239 (141 to 367)	46.8 (28.1 to 73.2)	--	--	140.4 (115 to 165.2)	4.3 (-2.5 to 11.9)	--	--
		Male	89 (53 to 136)	38 (22.6 to 58.4)	--	--	220 (130 to 344)	39.8 (23.6 to 61.3)	--	--	148 (120.5 to 178.6)	4.7 (-2.5 to 12.4)	--	--
	Musculoskeletal disorders	Both	7966 (5635 to 10665)	1855.2 (1323.2 to 2462.9)	1 (1 to 2)	0.4 (0.3 to 0.7)	22732 (16235 to 30328)	1932.3 (1390.9 to 2558.7)	6 (5 to 7)	0.8 (0.5 to 0.9)	185.3 (172.5 to 199.3)	4.2 (0.9 to 7.6)	461.6 (193.1 to 813.6)	98 (0 to 222.9)
		Female	4238 (3022 to 5638)	2016.7 (1439.2 to 2679.6)	1 (0 to 2)	0.5 (0.3 to 1.1)	11945 (8559 to 15821)	2149.9 (1541.2 to 2826.6)	4 (3 to 5)	1 (0.6 to 1.2)	181.9 (167.8 to 197.7)	6.6 (2.7 to 10.8)	486.9 (141.9 to 998.3)	103.2 (-23.5 to 315.4)
		Male	3729 (2623 to 5021)	1699.4 (1197.3 to 2276)	0 (0 to 1)	0.3 (0.2 to 0.4)	10787 (7665 to 14430)	1731.7 (1228 to 2309)	2 (2 to 2)	0.5 (0.4 to 0.6)	189.3 (172.3 to 208.3)	1.9 (-2.6 to 6.4)	411.3 (137.8 to 708.1)	96.7 (-9.8 to 190.7)
	Rheumatoid arthritis	Both	49 (34 to 67)	11.5 (8 to 15.8)	0 (0 to 0)	0.1 (0 to 0.2)	151 (105 to 207)	12.8 (9 to 17.2)	1 (0 to 1)	0.1 (0 to 0.1)	208.1 (157 to 261.4)	11 (-10.1 to 30.4)	316.5 (20 to 863.5)	20.9 (-66.3 to 190.6)
		Female	34 (23 to 48)	16.4 (11.3 to 22.9)	0 (0 to 0)	0.1 (0 to 0.3)	103 (70 to 145)	18.3 (12.7 to 25.1)	0 (0 to 1)	0.1 (0 to 0.1)	200.6 (133 to 271.3)	11.6 (-17.1 to 37.9)	261.7 (-24.4 to 1033.8)	2.5 (-79.3 to 236)
		Male	14 (10 to 20)	6.8 (4.7 to 9.5)	0 (0 to 0)	0 (0 to 0)	47 (33 to 65)	7.8 (5.6 to 10.5)	0 (0 to 0)	0 (0 to 0.1)	226 (173.4 to 292.3)	14.1 (-2.4 to 37)	529.8 (298.4 to 1030.8)	98.9 (28.5 to 254.4)
	Osteoarthritis	Both	509 (256 to 996)	177.7 (89.3 to 348.3)	--	--	1750 (879 to 3432)	191.1 (96.8 to 376.7)	--	--	243.8 (231.4 to 257)	7.6 (4.1 to 10.9)	--	--
		Female	277 (141 to 542)	201.3 (101.8 to 394.9)	--	--	976 (493 to 1927)	217.4 (110.2 to 428.8)	--	--	252.3 (235.3 to 270)	8 (3.2 to 12.8)	--	--
		Male	232 (115 to 461)	155.2 (77.6 to 306)	--	--	774 (386 to 1522)	165.2 (83.2 to 326.3)	--	--	233.6 (214.4 to 253.2)	6.5 (1.3 to 11.9)	--	--

Province	Cause	Sex	1990				2019				1990-2019 percent change (%)			
			DALYs		Death		DALYs		Death		DALYs		Death	
			Number	Rate	Number	Rate	Number	Rate	Number	Rate	Number	Rate	Number	Rate
	Low back pain	Both	4618 (3207 to 6202)	1001.7 (705.7 to 1349)	--	--	10742 (7491 to 14425)	901.1 (634.9 to 1202.4)	--	--	132.6 (118.3 to 147.8)	-10 (-13.6 to -5.9)	--	--
		Female	2158 (1490 to 2890)	950.6 (660.8 to 1279.9)	--	--	4983 (3479 to 6692)	892.3 (627.1 to 1193.3)	--	--	130.9 (112.4 to 150.8)	-6.1 (-11.9 to 0.2)	--	--
		Male	2460 (1709 to 3348)	1050 (743.3 to 1426.1)	--	--	5759 (3931 to 7762)	907.3 (634.6 to 1225.2)	--	--	134.1 (115.7 to 152.4)	-13.6 (-18.7 to -8.2)	--	--
	Neck pain	Both	1699 (1092 to 2484)	403.8 (265.9 to 585.3)	--	--	4876 (3186 to 7148)	401.8 (265.4 to 580)	--	--	186.9 (168.1 to 205.1)	-0.5 (-2.5 to 1.6)	--	--
		Female	1014 (661 to 1489)	492.7 (327.1 to 714.1)	--	--	2833 (1868 to 4168)	493.3 (328.4 to 720)	--	--	179.3 (159.5 to 197.8)	0.1 (-2.5 to 2.7)	--	--
		Male	685 (442 to 1001)	318.4 (204.5 to 462.7)	--	--	2043 (1308 to 2982)	319 (208.7 to 463.6)	--	--	198.3 (177.1 to 220.1)	0.2 (-2.9 to 3.6)	--	--
	Gout	Both	46 (28 to 68)	14.4 (9 to 21)	--	--	157 (96 to 230)	15.6 (9.6 to 22.8)	--	--	242.7 (193.6 to 302.7)	8.6 (-5 to 23.2)	--	--
		Female	10 (6 to 14)	6.2 (3.9 to 9.2)	--	--	33 (21 to 50)	7 (4.4 to 10.4)	--	--	251.1 (176 to 359.5)	12.3 (-8.8 to 42.4)	--	--
		Male	36 (22 to 54)	22.3 (13.8 to 32.7)	--	--	124 (75 to 183)	23.9 (14.7 to 35)	--	--	240.5 (183.7 to 304.2)	7.4 (-8.3 to 24.7)	--	--
	Other musculoskeletal disorders	Both	1046 (682 to 1498)	246.1 (161.1 to 349.2)	1 (1 to 2)	0.3 (0.2 to 0.5)	5057 (3435 to 7042)	410 (280.7 to 564.8)	6 (4 to 7)	0.7 (0.5 to 0.8)	383.6 (338.1 to 440.7)	66.6 (52.9 to 85.4)	481.2 (210.9 to 858.8)	113 (12.5 to 245.2)
		Female	745 (496 to 1044)	349.5 (234.9 to 489.3)	1 (0 to 1)	0.4 (0.2 to 0.8)	3016 (2093 to 4185)	521.5 (364 to 713.3)	4 (2 to 5)	0.9 (0.5 to 1.1)	305 (265.4 to 351.9)	49.2 (36.1 to 65.2)	524.2 (181.8 to 1119.4)	128.3 (-2.4 to 362.2)
		Male	301 (169 to 469)	146.6 (85.2 to 226.3)	0 (0 to 1)	0.2 (0.2 to 0.4)	2040 (1350 to 2924)	308.4 (201.4 to 439.8)	2 (1 to 2)	0.5 (0.4 to 0.6)	578.1 (455.8 to 774.6)	110.3 (75.6 to 168.4)	402 (117.9 to 685.9)	96.5 (-15.9 to 188.1)
	Other non-communicable diseases	Both	40052 (26478 to 55261)	4100.6 (2874.9 to 5394.5)	372 (216 to 541)	32.4 (19.3 to 46.6)	24252 (18724 to 30999)	2064.7 (1604.2 to 2626)	118 (93 to 152)	11.8 (9.4 to 15)	-39.4 (-59.9 to -0.9)	-49.6 (-64.5 to -25.2)	-68.3 (-80.7 to -36.7)	-63.7 (-77.5 to -29.7)
		Female	21138 (13790 to 28781)	4721.1 (3304.1 to 6170.1)	180 (103 to 265)	32.2 (19 to 47.2)	15619 (11872 to 20237)	2687.5 (2058.3 to 3447.2)	57 (44 to 75)	11.6 (9.1 to 15.1)	-26.1 (-50.3 to 15.8)	-43.1 (-58.4 to -20.4)	-68.3 (-80.9 to -35.4)	-64.1 (-78.1 to -29.8)
		Male	18914 (10988 to 27719)	3491.2 (2178 to 4950.4)	192 (102 to 292)	32.6 (17.9 to 49.5)	8633 (6657 to 11255)	1526.1 (1182.1 to 1974)	61 (47 to 82)	12 (9.3 to 15.8)	-54.4 (-72 to -12.4)	-56.3 (-71.8 to -22.8)	-68.3 (-81.4 to -29.4)	-63.2 (-77.9 to -21)
	Congenital birth defects	Both	30747 (17662 to 45234)	2581.8 (1494.7 to 3778.8)	340 (191 to 504)	28.4 (16.1 to 42.1)	8688 (6596 to 11549)	799.1 (600.6 to 1069.5)	88 (64 to 120)	8.2 (5.9 to 11.2)	-71.7 (-83.5 to -40.3)	-69.1 (-81.8 to -34.5)	-74.2 (-85.4 to -42.6)	-71.3 (-83.8 to -36.2)
		Female	14666 (7787 to 21820)	2520.2 (1350.2 to 3733.5)	163 (85 to 244)	27.9 (14.9 to 41.8)	4106 (3071 to 5549)	779.2 (578.1 to 1059.6)	42 (31 to 59)	8.1 (5.8 to 11.4)	-72 (-83.9 to -39.9)	-69.1 (-82.3 to -33.7)	-74 (-85.6 to -41.5)	-71 (-83.9 to -34.8)

Province	Cause	Sex	1990				2019				1990-2019 percent change (%)			
			DALYs		Death		DALYs		Death		DALYs		Death	
			Number	Rate	Number	Rate	Number	Rate	Number	Rate	Number	Rate	Number	Rate
		Male	16081 (8128 to 24630)	2640.1 (1348.8 to 4019.3)	177 (87 to 274)	28.9 (14.4 to 44.7)	4581 (3377 to 6350)	817.7 (599.3 to 1142.7)	45 (32 to 65)	8.2 (5.8 to 11.9)	-71.5 (-83.9 to -31.3)	-69 (-82.5 to -25.4)	-74.4 (-86.1 to -34)	-71.6 (-84.6 to -26.7)
	Urinary diseases and male infertility	Both	133 (95 to 184)	27.6 (19.2 to 39)	1 (0 to 1)	0.2 (0.1 to 0.7)	373 (266 to 505)	36.8 (27.2 to 48.3)	5 (3 to 6)	0.8 (0.5 to 0.9)	180.2 (121.3 to 224.6)	33.3 (-6.4 to 58.2)	635.8 (171.1 to 1058.1)	240.5 (-11.7 to 494.3)
		Female	46 (34 to 60)	15.5 (11.2 to 23.9)	0 (0 to 1)	0.3 (0.1 to 0.8)	117 (80 to 146)	23.7 (16.1 to 28.8)	3 (2 to 3)	0.8 (0.5 to 1)	154 (70.7 to 226.8)	52.5 (-24.4 to 108.6)	571.9 (121.6 to 1092.2)	187.3 (-19.5 to 474.9)
		Male	87 (58 to 133)	39.3 (25.7 to 57.8)	0 (0 to 1)	0.2 (0.1 to 0.6)	255 (175 to 369)	49.7 (35 to 69.1)	3 (1 to 3)	0.8 (0.5 to 1)	194.2 (142.4 to 237.5)	26.4 (-4.1 to 47.3)	713.5 (159.1 to 1167.7)	321.1 (-13.3 to 621.8)
	Gynecological diseases	Female	2872 (1978 to 4034)	1099.6 (755.9 to 1531.7)	0 (0 to 0)	0 (0 to 0)	6772 (4632 to 9446)	1048 (717.2 to 1457.7)	0 (0 to 0)	0 (0 to 0)	135.8 (122.7 to 148.5)	-4.7 (-7.6 to -1.6)	92 (-40.6 to 1469.6)	-21.1 (-75.8 to 422.9)
	Hemoglobinopathies and hemolytic anemias	Both	1970 (1459 to 2570)	227.8 (169 to 298.2)	13 (9 to 18)	1.6 (1.1 to 2)	1139 (847 to 1547)	93.7 (70 to 126.1)	8 (7 to 10)	0.8 (0.7 to 0.9)	-42.2 (-56.8 to -23.3)	-58.9 (-67.8 to -47.9)	-38.3 (-56.7 to -3.5)	-52.3 (-63.9 to -30.2)
		Female	1215 (855 to 1609)	295.8 (211.1 to 398.9)	8 (5 to 11)	1.9 (1.3 to 2.4)	697 (491 to 997)	119.4 (85.5 to 167.7)	5 (4 to 6)	0.9 (0.7 to 1)	-42.7 (-60.8 to -19.5)	-59.6 (-70.5 to -46.4)	-41.6 (-62.6 to 3.7)	-54.1 (-67.5 to -27.5)
		Male	755 (537 to 1013)	160.6 (117.8 to 210.5)	6 (4 to 8)	1.3 (1 to 1.7)	442 (322 to 624)	70.9 (51.4 to 100.3)	4 (3 to 4)	0.7 (0.6 to 0.8)	-41.5 (-58.9 to -14.4)	-55.8 (-67.8 to -38.3)	-33.9 (-53.2 to 5.1)	-49.2 (-62 to -25.9)
	Endocrine, metabolic, blood, and immune disorders	Both	1876 (1271 to 2788)	308.9 (210.9 to 436.3)	5 (3 to 14)	1.1 (0.6 to 2.5)	3659 (2596 to 4963)	316.6 (227 to 424.3)	15 (11 to 17)	1.8 (1.4 to 2.2)	95.1 (39.4 to 127.7)	2.5 (-15.9 to 14.7)	166.9 (-0.2 to 422)	68.7 (-25.8 to 227.6)
		Female	1150 (780 to 1697)	390.5 (265.6 to 550.2)	2 (1 to 7)	1.1 (0.6 to 3)	2229 (1541 to 3088)	395.7 (276.8 to 545.7)	6 (5 to 8)	1.6 (1.1 to 2)	93.8 (45.2 to 121.8)	1.3 (-16.3 to 13.5)	159.6 (-17.2 to 421.8)	46.3 (-42.9 to 207.6)
		Male	725 (478 to 1162)	230.1 (156.7 to 332.1)	3 (1 to 7)	1.1 (0.5 to 2.5)	1430 (1028 to 1912)	246.1 (179.1 to 326.6)	8 (6 to 11)	2.1 (1.4 to 2.7)	97.2 (29.2 to 149.2)	7 (-16.6 to 26)	172.9 (10.2 to 484.4)	92.4 (-5.2 to 300.1)
	Oral disorders	Both	1332 (786 to 2109)	317.8 (190 to 499.3)	--	--	3421 (2002 to 5504)	312.1 (185.6 to 492.7)	--	--	156.9 (145.8 to 167.8)	-1.8 (-3.9 to 0.3)	--	--
		Female	647 (378 to 1024)	307.9 (182.2 to 485)	--	--	1600 (938 to 2573)	302.4 (179.5 to 479.1)	--	--	147.5 (135.5 to 159.6)	-1.8 (-5 to 1.2)	--	--
		Male	685 (409 to 1086)	327.4 (197.3 to 513.3)	--	--	1821 (1066 to 2956)	322.4 (192.9 to 505.3)	--	--	165.7 (153.4 to 178.4)	-1.5 (-4.6 to 1.3)	--	--
	Sudden infant death syndrome	Both	1122 (200 to 3860)	92.7 (16.6 to 318.7)	13 (2 to 44)	1 (0.2 to 3.6)	201 (102 to 401)	19.2 (9.7 to 38.2)	2 (1 to 5)	0.2 (0.1 to 0.4)	-82.1 (-94.2 to 6.6)	-79.3 (-93.4 to 23.1)	-82.1 (-94.2 to 6.6)	-79.3 (-93.4 to 23.1)
		Female	542 (95 to 2035)	91.6 (16 to 343.9)	6 (1 to 23)	1 (0.2 to 3.9)	98 (48 to 196)	19.1 (9.3 to 38.4)	1 (1 to 2)	0.2 (0.1 to 0.4)	-82 (-94.5 to 8.2)	-79.1 (-93.6 to 25.4)	-82 (-94.5 to 8.2)	-79.1 (-93.6 to 25.4)
		Male	580 (87 to 2079)	93.7 (14 to 335.7)	7 (1 to 24)	1.1 (0.2 to 3.8)	103 (47 to 224)	19.2 (8.7 to 41.6)	1 (1 to 3)	0.2 (0.1 to 0.5)	-82.2 (-94.6 to 5)	-79.5 (-93.8 to 20.8)	-82.2 (-94.6 to 5)	-79.5 (-93.8 to 20.7)
	**Injuries**	Both	44742 (30951 to 53178)	6252.6 (4545.6 to 7384.2)	639 (432 to 772)	109.2 (79.7 to 131.3)	34498 (30829 to 38526)	2806.5 (2506.1 to 3135.4)	581 (512 to 661)	53.1 (46.8 to 60.6)	-22.9 (-37.3 to 11)	-55.1 (-62.9 to -37.9)	-9.1 (-28.2 to 35.7)	-51.4 (-61.1 to -32.4)

Province	Cause	Sex	1990				2019				1990-2019 percent change (%)			
			DALYs		Death		DALYs		Death		DALYs		Death	
			Number	Rate	Number	Rate	Number	Rate	Number	Rate	Number	Rate	Number	Rate
		Female	15508 (10296 to 19445)	4030.8 (2870.3 to 4948)	206 (132 to 262)	65.6 (46.5 to 81.3)	9368 (8291 to 10491)	1634.2 (1445.8 to 1827.7)	145 (128 to 163)	28.5 (25.2 to 32)	-39.6 (-53.8 to -9.1)	-59.5 (-67.9 to -41.4)	-29.8 (-47 to 11)	-56.6 (-66.5 to -37.6)
		Male	29234 (20406 to 35001)	8417.4 (5951.2 to 10133.6)	433 (293 to 532)	151.6 (106.1 to 188.2)	25131 (22005 to 28599)	3861.1 (3367 to 4396.4)	436 (373 to 508)	76.1 (64.6 to 89.2)	-14 (-31.5 to 23.8)	-54.1 (-63.3 to -35.9)	0.8 (-22.5 to 50)	-49.8 (-62 to -28.3)
	Transport injuries	Both	23689 (15839 to 29047)	3356.5 (2327.9 to 4094.8)	372 (252 to 459)	65.2 (44.4 to 79.9)	17955 (15256 to 20497)	1462.4 (1238 to 1669.8)	353 (288 to 408)	32 (26.2 to 37.2)	-24.2 (-40.4 to 13.5)	-56.4 (-65.1 to -38.1)	-5.2 (-25.7 to 39.6)	-51 (-61.3 to -30.5)
		Female	7200 (4657 to 9505)	1848.1 (1271.2 to 2331.6)	102 (67 to 132)	32.3 (22.7 to 40)	3991 (3417 to 4603)	694.6 (594 to 798.5)	73 (63 to 87)	14 (12.1 to 16.5)	-44.6 (-60.2 to -13.1)	-62.4 (-71.8 to -44.6)	-27.8 (-47.1 to 12.4)	-56.8 (-66.6 to -35.5)
		Male	16489 (10695 to 20599)	4824.9 (3103.1 to 6070.7)	270 (169 to 344)	97.3 (61 to 124.2)	13964 (11230 to 16271)	2155.2 (1727.4 to 2506.7)	279 (215 to 332)	48.9 (38.2 to 58.4)	-15.3 (-34.1 to 30.7)	-55.3 (-65.9 to -33.3)	3.4 (-21.2 to 58.6)	-49.8 (-62.2 to -25.9)
	Road injuries	Both	23111 (15421 to 28320)	3267.5 (2282.6 to 3979.3)	363 (246 to 448)	63.5 (43.2 to 78)	17244 (14608 to 19699)	1404.6 (1183.8 to 1604)	340 (277 to 393)	30.8 (25.3 to 35.8)	-25.4 (-41.3 to 11.8)	-57 (-65.6 to -38.8)	-6.4 (-26.5 to 37.7)	-51.5 (-61.7 to -31.1)
		Female	7079 (4582 to 9341)	1815.2 (1248.5 to 2291.9)	100 (66 to 130)	31.8 (22.3 to 39.5)	3893 (3332 to 4495)	677.4 (579.6 to 779.9)	72 (62 to 85)	13.7 (11.9 to 16.2)	-45 (-60.4 to -13.8)	-62.7 (-72.1 to -45)	-28.1 (-47.2 to 11.7)	-56.9 (-66.7 to -36)
		Male	16032 (10415 to 20016)	4681.7 (3009 to 5898.7)	263 (164 to 335)	94.4 (59.6 to 120.6)	13351 (10683 to 15532)	2060.7 (1642.7 to 2395)	268 (204 to 318)	46.8 (36.2 to 55.9)	-16.7 (-35.2 to 29.3)	-56 (-66.3 to -34)	1.8 (-22.3 to 56.2)	-50.4 (-62.9 to -27)
	Other transport injuries	Both	578 (370 to 753)	89 (58.7 to 114)	9 (5 to 12)	1.7 (0.9 to 2.2)	711 (551 to 903)	57.8 (45.2 to 72.9)	13 (9 to 17)	1.2 (0.9 to 1.5)	23.2 (-14.4 to 143.9)	-35.1 (-55.1 to 21.6)	46.6 (-5.7 to 225.8)	-29.4 (-54.6 to 40.8)
		Female	121 (78 to 171)	32.9 (22.9 to 43.4)	1 (1 to 2)	0.5 (0.3 to 0.7)	98 (77 to 121)	17.2 (13.5 to 20.9)	1 (1 to 2)	0.3 (0.2 to 0.3)	-18.7 (-46.6 to 30.3)	-47.9 (-63.7 to -21)	-12.8 (-46.4 to 57.9)	-47.2 (-67 to -12.6)
		Male	457 (269 to 619)	143.2 (83.5 to 192.3)	7 (4 to 10)	2.8 (1.4 to 3.9)	613 (451 to 801)	94.5 (69.8 to 121.9)	12 (8 to 15)	2.1 (1.4 to 2.7)	34.2 (-12.7 to 181.8)	-34 (-57 to 27.6)	58.7 (-2.8 to 251)	-27.5 (-55.1 to 40.3)
	Unintentional injuries	Both	14541 (10687 to 17850)	1912.8 (1483.2 to 2278.7)	184 (130 to 226)	30.2 (22.8 to 36.5)	9685 (8220 to 11497)	817.3 (692.5 to 969.6)	126 (112 to 148)	13.1 (11.5 to 15.5)	-33.4 (-47.9 to -7.1)	-57.3 (-65.1 to -42.6)	-31.4 (-46.5 to 0.4)	-56.7 (-65.6 to -39.5)
		Female	5705 (3984 to 7672)	1408.2 (1035.3 to 1811.9)	69 (46 to 93)	21.6 (15.4 to 27.7)	3069 (2550 to 3740)	564.1 (469.9 to 682.9)	38 (33 to 44)	8.8 (7.6 to 10.3)	-46.2 (-61 to -19.2)	-59.9 (-69.7 to -44.4)	-44.7 (-61.1 to -12.6)	-59.3 (-69.6 to -40.9)
		Male	8836 (6474 to 11039)	2403.1 (1839.9 to 2972.2)	116 (80 to 149)	38.5 (28.3 to 49)	6617 (5580 to 7946)	1044.5 (883.7 to 1254.4)	89 (76 to 109)	17 (14.6 to 21.1)	-25.1 (-42.6 to 3.5)	-56.5 (-65.5 to -41.3)	-23.6 (-43.1 to 15.9)	-55.8 (-67 to -35.7)
	Falls	Both	2274 (1725 to 2853)	399.5 (316.1 to 488.8)	25 (18 to 31)	6.4 (4.7 to 7.7)	3137 (2478 to 3970)	281.1 (223.6 to 353.1)	35 (28 to 44)	4.4 (3.4 to 5.5)	37.9 (8.3 to 73.4)	-29.6 (-40.3 to -16.6)	40.5 (5.8 to 102.9)	-30.4 (-46.7 to -0.6)
		Female	784 (452 to 1085)	268.5 (193 to 337.9)	8 (4 to 11)	4.7 (3.1 to 6.3)	911 (692 to 1174)	183.3 (139.7 to 232.8)	10 (7 to 12)	3.1 (2 to 3.7)	16.3 (-17.8 to 76.3)	-31.7 (-42.4 to -17.2)	25.3 (-8 to 91)	-34.8 (-52.8 to -0.9)
		Male	1491 (1065 to 1899)	525.1 (397.9 to 649.1)	17 (11 to 22)	7.9 (5.4 to 9.9)	2226 (1769 to 2825)	369.5 (294.5 to 466.4)	25 (20 to 33)	5.7 (4.4 to 7.6)	49.3 (15.4 to 107.9)	-29.6 (-43 to -9.8)	48 (3.7 to 152.7)	-27.7 (-47.3 to 13)
	Drowning	Both	3763 (2159 to 5243)	372.2 (214.6 to 518.4)	49 (28 to 68)	5.4 (3.1 to 7.4)	888 (664 to 1050)	71.1 (53.4 to 84.9)	15 (11 to 18)	1.3 (0.9 to 1.5)	-76.4 (-84 to -59.9)	-80.9 (-86.7 to -68)	-69.4 (-78.8 to -48)	-76.4 (-83.2 to -61.7)
		Female	1495 (819 to 2401)	273.3 (155 to 421.3)	18 (10 to 29)	3.6 (2.1 to 5.3)	181 (145 to 231)	31.3 (24.9 to 40.3)	3 (2 to 3)	0.5 (0.4 to 0.6)	-87.9 (-93 to -76.7)	-88.6 (-93.2 to -78.4)	-84.7 (-91 to -71.5)	-85.6 (-90.9 to -74)
		Male	2268 (920 to 3398)	469 (198.9 to 689.3)	31 (13 to 46)	7.2 (3.1 to 10.8)	706 (481 to 859)	106.8 (73.1 to 130.4)	12 (8 to 15)	2 (1.4 to 2.4)	-68.8 (-79.8 to -39.6)	-77.2 (-85.1 to -57.8)	-60.3 (-74 to -25)	-72.7 (-81.6 to -50.4)
	Fire, heat, and hot substances	Both	2262 (1209 to 3504)	292.3 (166.7 to 428.5)	32 (17 to 49)	5.5 (3.4 to 7.5)	949 (756 to 1263)	79.1 (62.6 to 107.2)	19 (15 to 26)	1.9 (1.5 to 2.8)	-58 (-77.1 to -8.3)	-72.9 (-84.3 to -44.9)	-41.9 (-68.2 to 29.7)	-64.9 (-79.2 to -30.7)

Province	Cause	Sex	1990				2019				1990-2019 percent change (%)			
			DALYs		Death		DALYs		Death		DALYs		Death	
			Number	Rate	Number	Rate	Number	Rate	Number	Rate	Number	Rate	Number	Rate
		Female	1098 (321 to 2080)	285.4 (112 to 495.9)	15 (5 to 29)	5.2 (2.6 to 8.2)	483 (367 to 683)	85.4 (64.9 to 122.7)	9 (7 to 14)	2 (1.5 to 3.1)	-56 (-80.2 to 68.6)	-70.1 (-85.5 to -14.3)	-38.8 (-72.8 to 132.6)	-61.7 (-80.5 to -13.4)
		Male	1164 (535 to 1619)	298.8 (146.1 to 399.6)	17 (7 to 23)	5.7 (3 to 7.6)	466 (352 to 743)	73.2 (55.4 to 118.7)	9 (7 to 16)	1.8 (1.3 to 3.2)	-60 (-75.9 to 33.8)	-75.5 (-84.7 to -26)	-44.7 (-66.8 to 88.8)	-67.7 (-80 to -8.3)
	Poisonings	Both	539 (336 to 989)	59.9 (38.7 to 114.4)	7 (4 to 14)	1 (0.6 to 1.9)	272 (212 to 500)	21.5 (16.9 to 38.9)	5 (4 to 9)	0.4 (0.3 to 0.8)	-49.5 (-66.2 to -17.8)	-64.2 (-75.3 to -44.7)	-31.7 (-54 to 9)	-55.5 (-69.4 to -33.7)
		Female	203 (108 to 340)	42 (24.7 to 69.3)	3 (1 to 4)	0.6 (0.4 to 1)	88 (70 to 114)	15 (12 to 19)	1 (1 to 2)	0.3 (0.2 to 0.3)	-56.4 (-75 to -15.5)	-64.3 (-78.1 to -38.2)	-43 (-66.4 to 7.6)	-55.6 (-71 to -26.2)
		Male	336 (177 to 756)	77.3 (43.4 to 178.9)	5 (2 to 11)	1.3 (0.8 to 3.1)	183 (133 to 376)	27.2 (19.8 to 55.7)	3 (3 to 7)	0.6 (0.4 to 1.1)	-45.4 (-67.4 to 0.5)	-64.8 (-78 to -38.5)	-25.6 (-54.3 to 35.2)	-56.2 (-72.2 to -28.8)
	Exposure to mechanical forces	Both	1249 (789 to 2111)	204 (134.3 to 331)	15 (8 to 29)	2.7 (1.5 to 5.3)	1441 (1076 to 2006)	116.7 (87.1 to 160.7)	15 (11 to 25)	1.3 (1 to 2.1)	15.4 (-20.7 to 68.7)	-42.8 (-59.6 to -21.4)	5.3 (-29.2 to 86.8)	-49.8 (-66.5 to -17.8)
		Female	297 (204 to 488)	91.7 (66.6 to 135.1)	3 (2 to 5)	1 (0.6 to 2.1)	350 (261 to 477)	61.5 (45.8 to 84.1)	3 (2 to 4)	0.5 (0.4 to 0.8)	17.5 (-22.5 to 66.4)	-33 (-50.1 to -13.6)	-7.7 (-39.8 to 65.6)	-49.2 (-66.2 to -17.5)
		Male	951 (569 to 1716)	313.2 (193.3 to 541.8)	12 (6 to 25)	4.3 (2.2 to 9.2)	1091 (798 to 1595)	166.3 (122.8 to 236)	13 (9 to 22)	2.1 (1.5 to 3.6)	14.7 (-24.2 to 76.8)	-46.9 (-64.4 to -22.3)	8.4 (-30.6 to 105.2)	-51 (-69.6 to -12.2)
	Adverse effects of medical treatment	Both	1103 (517 to 1714)	165.4 (77.5 to 270)	19 (9 to 31)	4.2 (2 to 7)	472 (368 to 546)	43.1 (34.4 to 49.8)	12 (10 to 14)	1.4 (1.1 to 1.6)	-57.2 (-72.6 to -17.3)	-73.9 (-84.1 to -49.6)	-36 (-62 to 26.8)	-66.7 (-81.7 to -35.3)
		Female	443 (192 to 682)	127.1 (60.8 to 201.2)	7 (3 to 11)	3.1 (1.5 to 5.7)	198 (137 to 242)	37.5 (26.1 to 45.5)	5 (3 to 6)	1.2 (0.8 to 1.4)	-55.3 (-70.6 to -14.4)	-70.5 (-81.3 to -49.2)	-31.3 (-57.3 to 19.9)	-63.3 (-80.8 to -32.7)
		Male	661 (272 to 1128)	202.5 (81.3 to 348.2)	12 (5 to 20)	5.2 (2.2 to 9)	274 (205 to 336)	48.4 (37.3 to 59.1)	7 (5 to 9)	1.6 (1.3 to 2.1)	-58.5 (-75.9 to -6.6)	-76.1 (-86.8 to -42.7)	-38.9 (-66.8 to 51)	-68.7 (-83.6 to -26.3)
	Animal contact	Both	453 (208 to 656)	49.8 (26 to 67.5)	6 (2 to 8)	0.8 (0.4 to 1.1)	185 (141 to 229)	15.9 (12 to 19.7)	2 (2 to 3)	0.3 (0.2 to 0.3)	-59.1 (-73.8 to -27.3)	-68.1 (-78.3 to -47.6)	-56 (-72.3 to -16)	-66.6 (-77.6 to -43.3)
		Female	193 (65 to 323)	41.5 (18.3 to 64.2)	2 (1 to 4)	0.6 (0.2 to 0.9)	78 (56 to 102)	13.8 (9.9 to 18)	1 (0 to 1)	0.2 (0.1 to 0.2)	-59.6 (-77.4 to -0.3)	-66.8 (-79.9 to -36)	-63.8 (-80.1 to -10.5)	-70.1 (-82 to -40.8)
		Male	260 (119 to 381)	57.8 (28.8 to 80.2)	3 (1 to 5)	1 (0.5 to 1.4)	107 (76 to 134)	17.9 (12.6 to 22.2)	2 (1 to 2)	0.4 (0.2 to 0.4)	-58.8 (-74.9 to -15.6)	-69.1 (-80.4 to -44.7)	-50.8 (-71.6 to -0.3)	-64.6 (-80.2 to -36.7)
	Foreign body	Both	1011 (672 to 1457)	100.1 (70.9 to 137.6)	11 (7 to 16)	1.1 (0.8 to 1.6)	490 (386 to 610)	42 (33.3 to 51.8)	5 (4 to 6)	0.5 (0.4 to 0.6)	-51.5 (-68.9 to -22.3)	-58.1 (-72 to -38)	-55.8 (-72.4 to -26.6)	-57.3 (-71.7 to -35.2)
		Female	576 (345 to 919)	111.1 (71.5 to 170)	6 (4 to 10)	1.3 (0.8 to 1.9)	208 (157 to 270)	37.9 (28.8 to 48.8)	2 (2 to 3)	0.4 (0.3 to 0.5)	-63.8 (-79.7 to -32.7)	-65.8 (-80 to -41.5)	-67.2 (-82.1 to -37)	-65.5 (-79.7 to -40.9)
		Male	435 (285 to 672)	89.7 (63.4 to 130.1)	5 (3 to 7)	1 (0.7 to 1.5)	282 (218 to 360)	45.5 (35.3 to 57.3)	3 (2 to 3)	0.5 (0.4 to 0.7)	-35.2 (-61 to 1.6)	-49.2 (-67.2 to -26.8)	-40.2 (-65.6 to -0.7)	-48 (-67 to -21.4)
	Environmental heat and cold exposure	Both	102 (75 to 141)	17.8 (13.2 to 24.3)	1 (0 to 1)	0.2 (0.1 to 0.3)	176 (125 to 236)	14.5 (10.4 to 19.2)	1 (0 to 1)	0.1 (0.1 to 0.1)	72.9 (16.5 to 107.4)	-18.9 (-42.3 to -4.1)	78.6 (-32.5 to 223.1)	-28.9 (-66.9 to 24.8)
		Female	44 (31 to 62)	15.6 (11.1 to 21.7)	0 (0 to 0)	0.1 (0 to 0.2)	75 (52 to 104)	13 (9.1 to 17.9)	0 (0 to 0)	0.1 (0 to 0.1)	70.2 (27.8 to 97.1)	-17 (-35 to -5.9)	65.1 (-31.2 to 262.4)	-30.1 (-73.2 to 57)
		Male	58 (42 to 85)	20 (14.5 to 29.2)	0 (0 to 1)	0.3 (0.1 to 0.5)	102 (68 to 134)	15.8 (10.6 to 20.7)	1 (0 to 1)	0.2 (0.1 to 0.2)	74.9 (7.1 to 119.5)	-21 (-50.8 to -1.3)	82.6 (-34.8 to 225.5)	-29.9 (-70.8 to 21.9)
	Exposure to forces of nature	Both	28 (23 to 34)	4.1 (3.4 to 5)	0 (0 to 0)	0 (0 to 0)	92 (66 to 127)	7.3 (5.3 to 10)	0 (0 to 0)	0 (0 to 0)	231.4 (171.6 to 289.7)	79.3 (49.7 to 107.2)	-71.5 (-71.5 to -71.5)	-85.3 (-85.3 to -85.3)
		Female	10 (9 to 12)	2.9 (2.4 to 3.6)	0 (0 to 0)	0 (0 to 0)	31 (22 to 44)	5.3 (3.8 to 7.5)	0 (0 to 0)	0 (0 to 0)	209.2 (149.7 to 273.2)	82 (50.6 to 116.8)	-72.2 (-72.2 to -72.2)	-84.9 (-84.9 to -84.9)

Province	Cause	Sex	1990				2019				1990-2019 percent change (%)			
			DALYs		Death		DALYs		Death		DALYs		Death	
			Number	Rate	Number	Rate	Number	Rate	Number	Rate	Number	Rate	Number	Rate
		Male	18 (15 to 21)	5.2 (4.3 to 6.4)	0 (0 to 0)	0.1 (0 to 0.1)	61 (44 to 83)	9.1 (6.7 to 12.2)	0 (0 to 0)	0 (0 to 0)	244 (182.9 to 302.7)	74.6 (47.2 to 101.4)	-71 (-71 to -71)	-85.7 (-85.7 to -85.7)
	Other unintentional injuries	Both	1759 (1225 to 2448)	247.7 (177.6 to 343.5)	20 (12 to 31)	3 (1.9 to 4.6)	1584 (1213 to 1996)	125.1 (95.7 to 158.1)	17 (12 to 20)	1.4 (1 to 1.7)	-9.9 (-38.9 to 31.8)	-49.5 (-65 to -29.5)	-16.2 (-49.6 to 41.3)	-53.1 (-71.4 to -23.3)
		Female	562 (376 to 795)	148.9 (106 to 198.6)	5 (3 to 8)	1.4 (0.9 to 2.1)	465 (348 to 620)	80.1 (59.8 to 106.7)	3 (3 to 4)	0.6 (0.5 to 0.8)	-17.2 (-45.4 to 26.9)	-46.2 (-61.4 to -24.8)	-38.9 (-64.8 to 16)	-56.2 (-73.1 to -20.3)
		Male	1196 (787 to 1840)	344.5 (236.8 to 518.5)	15 (8 to 25)	4.5 (2.7 to 7.5)	1119 (787 to 1395)	164.8 (115.3 to 205.7)	14 (8 to 17)	2.1 (1.2 to 2.6)	-6.5 (-46.2 to 44.9)	-52.2 (-71.3 to -27.8)	-7.9 (-52.5 to 66)	-53.7 (-75.9 to -17.2)
	Self-harm and interpersonal violence	Both	6512 (4471 to 8001)	983.3 (700 to 1202.4)	82 (51 to 105)	13.8 (9.2 to 17.6)	6858 (5989 to 8011)	526.8 (461.5 to 610.2)	102 (88 to 124)	8.1 (7 to 9.8)	5.3 (-14.3 to 50.6)	-46.4 (-55.8 to -26.6)	23.4 (-4.2 to 99.9)	-41.6 (-54.3 to -13.3)
		Female	2603 (1444 to 3493)	774.5 (463.9 to 1031)	36 (18 to 50)	11.7 (6.7 to 16.2)	2308 (1977 to 2634)	375.6 (323.8 to 426.8)	34 (28 to 39)	5.7 (4.8 to 6.6)	-11.3 (-35.3 to 63.4)	-51.5 (-63.8 to -19)	-6.7 (-35.4 to 87.3)	-51.3 (-65 to -14.7)
		Male	3909 (2873 to 4929)	1189.4 (892.3 to 1514.4)	46 (32 to 61)	15.9 (11.2 to 21.5)	4549 (3853 to 5585)	661.4 (563.2 to 806.4)	68 (56 to 88)	10.2 (8.4 to 13.2)	16.4 (-5.7 to 50.3)	-44.4 (-54.7 to -27.9)	46.7 (10.1 to 118.3)	-35.8 (-51.4 to -4.7)
	Self-harm	Both	2771 (1357 to 3779)	423.1 (235.2 to 584.4)	46 (24 to 64)	8 (4.7 to 10.9)	2500 (2091 to 3379)	188.1 (158.1 to 251.3)	48 (40 to 66)	3.9 (3.3 to 5.2)	-9.8 (-34.1 to 74.4)	-55.5 (-66.6 to -22.2)	3 (-23.6 to 93.9)	-51.4 (-63.2 to -20.6)
		Female	1698 (750 to 2407)	505.9 (252.4 to 713.3)	27 (13 to 39)	9 (4.8 to 12.7)	1042 (839 to 1259)	168.2 (135.7 to 202.6)	19 (15 to 23)	3.2 (2.6 to 3.8)	-38.6 (-58.8 to 31.4)	-66.8 (-77.3 to -36.5)	-31.2 (-53.9 to 45.1)	-64.3 (-75.7 to -36.6)
		Male	1073 (569 to 1709)	340.6 (193 to 561.3)	19 (11 to 31)	6.9 (4.2 to 11.7)	1457 (1129 to 2247)	205.8 (161.8 to 312.4)	29 (22 to 45)	4.5 (3.5 to 6.8)	35.8 (-4.9 to 155.1)	-39.6 (-57.7 to 3.7)	52.7 (6.5 to 175.5)	-35.1 (-53.9 to 8.3)
	Interpersonal violence	Both	1618 (1092 to 2099)	244.1 (168.2 to 306.4)	22 (14 to 29)	3.8 (2.4 to 4.9)	2768 (2374 to 3206)	211.7 (181.3 to 243.2)	39 (33 to 46)	3.1 (2.6 to 3.6)	71.1 (28.3 to 160.2)	-13.3 (-33.6 to 27.6)	77.7 (28.2 to 197.5)	-17.9 (-40.2 to 32)
		Female	661 (432 to 900)	183.8 (127.7 to 241.4)	8 (4 to 11)	2.4 (1.5 to 3.4)	1078 (876 to 1328)	176.6 (144.2 to 217.2)	14 (11 to 17)	2.3 (1.8 to 2.9)	63 (15.9 to 163.9)	-3.9 (-29.1 to 46.8)	74 (15 to 224.1)	-5.8 (-37.2 to 68.9)
		Male	956 (574 to 1323)	302.9 (188.8 to 409.4)	14 (8 to 20)	5.1 (2.8 to 7.2)	1690 (1358 to 2046)	242.4 (197.1 to 290.4)	26 (21 to 32)	3.8 (3.1 to 4.7)	76.7 (23.2 to 194)	-20 (-43.8 to 28.9)	79.8 (17.6 to 235.1)	-24.7 (-50.2 to 37.5)
	Conflict and terrorism	Both	1708 (1252 to 2371)	250.3 (182 to 346.1)	7 (7 to 8)	0.9 (0.9 to 1)	780 (505 to 1193)	67.7 (44.2 to 102.4)	0 (0 to 1)	0 (0 to 0)	-54.3 (-61.1 to -48.2)	-73 (-76.9 to -69)	-93.5 (-93.5 to -93.5)	-95.9 (-95.9 to -95.9)
		Female	197 (120 to 328)	70.8 (44.8 to 114)	0 (0 to 0)	0 (0 to 0)	122 (74 to 211)	19.8 (12.2 to 33.6)	0 (0 to 0)	0 (0 to 0)	-38 (-48.8 to -26.9)	-72 (-77.7 to -66.2)	270.4 (270.4 to 270.4)	112.4 (112.4 to 112.4)
		Male	1511 (1124 to 2059)	429 (317.3 to 584.4)	7 (7 to 8)	1.9 (1.7 to 2)	658 (425 to 990)	111.9 (73.1 to 166.2)	0 (0 to 0)	0.1 (0 to 0.1)	-56.4 (-63.2 to -50.3)	-73.9 (-77.9 to -70.2)	-95.3 (-95.3 to -95.3)	-97.2 (-97.2 to -97.2)
	Executions and police conflict	Both	416 (271 to 593)	65.8 (41.2 to 93.1)	7 (4 to 9)	1.2 (0.7 to 1.7)	809 (437 to 1212)	59.3 (33.5 to 88.9)	14 (7 to 22)	1.1 (0.5 to 1.7)	94.7 (21.2 to 224.5)	-9.9 (-43.8 to 48.8)	116.8 (27.8 to 278.2)	-7.5 (-45.6 to 58.6)
		Female	47 (41 to 53)	14 (12.4 to 16)	1 (1 to 1)	0.2 (0.2 to 0.2)	66 (57 to 77)	11 (9.6 to 12.8)	1 (1 to 1)	0.2 (0.2 to 0.2)	39.5 (32.6 to 47.4)	-21.7 (-25.3 to -17.3)	52.9 (50.4 to 56.5)	-25.7 (-26.7 to -24.2)
		Male	369 (224 to 545)	116.9 (68.3 to 170.3)	6 (3 to 9)	2.1 (1.1 to 3.1)	744 (375 to 1141)	101.3 (53.3 to 156)	13 (6 to 21)	1.9 (0.9 to 3)	101.8 (19.4 to 253)	-13.3 (-48.5 to 49.1)	123.4 (25.8 to 311.1)	-10.1 (-49.3 to 60.1)
**Chahar Mahaal and Bakhtiari**	**All causes**	Both	267824 (236328 to 299697)	40121.5 (35892.4 to 44828.3)	3689 (3258 to 4139)	927.7 (823.6 to 1042.2)	205538 (176151 to 240705)	22317.7 (19278.5 to 25860.8)	4055 (3623 to 4519)	538.6 (483.5 to 601.4)	-23.3 (-33.4 to -11.8)	-44.4 (-50.7 to -37.3)	9.9 (-6.1 to 29.6)	-41.9 (-50.4 to -31.1)
		Female	118833 (104281 to 134176)	36013.9 (31391.4 to 40981.4)	1493 (1310 to 1696)	768 (662.2 to 891)	92882 (76709 to 111268)	19970.6 (16669.7 to 23815)	1517 (1313 to 1810)	398.7 (346.1 to 475)	-21.8 (-32.9 to -9.8)	-44.5 (-51.5 to -36.6)	1.6 (-17.7 to 28.2)	-48.1 (-58.7 to -34.2)

Province	Cause	Sex	1990				2019				1990-2019 percent change (%)			
			DALYs		Death		DALYs		Death		DALYs		Death	
			Number	Rate	Number	Rate	Number	Rate	Number	Rate	Number	Rate	Number	Rate
		Male	148991 (131055 to 168001)	43891.6 (38605.2 to 50017.6)	2196 (1878 to 2570)	1082.8 (912.5 to 1301.6)	112656 (97190 to 130404)	24778.9 (21511.5 to 28457.2)	2539 (2205 to 2887)	686.6 (599.8 to 774.6)	-24.4 (-35.6 to -12.5)	-43.5 (-52 to -34.2)	15.6 (-5.6 to 39.8)	-36.6 (-49 to -22.2)
	**Communicable, maternal, neonatal, and nutritional diseases**	Both	93763 (75484 to 114253)	8347.7 (6794.1 to 10325.6)	1026 (824 to 1278)	101.2 (82.7 to 141.7)	15581 (12769 to 18739)	1710.1 (1396.5 to 2052.5)	141 (110 to 197)	17.8 (13.9 to 25.6)	-83.4 (-87.7 to -78.3)	-79.5 (-84.7 to -73.6)	-86.3 (-90.2 to -78.4)	-82.5 (-87.8 to -74.6)
		Female	43710 (35026 to 53446)	8008.9 (6469.8 to 9801.5)	474 (377 to 591)	95 (77 to 124.5)	7250 (5848 to 8797)	1613.5 (1297.8 to 1959)	59 (44 to 82)	14.7 (11.1 to 21)	-83.4 (-87.9 to -78.2)	-79.9 (-85.1 to -73.8)	-87.7 (-91.4 to -80.8)	-84.6 (-89.3 to -77.4)
		Male	50053 (39917 to 62161)	8663 (6948.3 to 10987.1)	552 (434 to 698)	107.1 (85.4 to 157.3)	8331 (6712 to 10024)	1805.2 (1451.7 to 2190.8)	82 (63 to 123)	21.1 (16.4 to 32.5)	-83.4 (-88 to -77.9)	-79.2 (-84.8 to -72.6)	-85.1 (-89.6 to -76.2)	-80.3 (-87.1 to -71)
	HIV/AIDS and sexually transmitted infections	Both	217 (106 to 423)	25.7 (15 to 44.9)	2 (1 to 4)	0.2 (0.1 to 0.4)	262 (175 to 390)	25.4 (16.6 to 39.3)	2 (1 to 3)	0.2 (0.1 to 0.4)	20.7 (-26.6 to 94.9)	-0.8 (-28.4 to 27.7)	8.8 (-37.2 to 124.2)	8 (-32.4 to 69.4)
		Female	110 (57 to 203)	29.1 (16.5 to 48.9)	1 (0 to 2)	0.2 (0.1 to 0.4)	139 (90 to 214)	27.3 (17.1 to 43)	1 (0 to 1)	0.2 (0.1 to 0.3)	26.1 (-21.3 to 89.8)	-6.4 (-28.6 to 18.2)	-4 (-45.3 to 99.4)	-2.2 (-40.3 to 57)
		Male	107 (48 to 222)	22.3 (12.2 to 41.6)	1 (0 to 2)	0.2 (0.1 to 0.4)	123 (82 to 186)	23.7 (15.2 to 38.3)	1 (1 to 2)	0.2 (0.2 to 0.4)	15 (-29.9 to 102)	6.4 (-27.6 to 47)	19.4 (-30.5 to 154.9)	17.3 (-25.8 to 95.3)
	HIV/AIDS	Both	7 (4 to 10)	1.2 (0.6 to 1.7)	0 (0 to 0)	0 (0 to 0)	61 (45 to 84)	5.3 (4 to 7.3)	1 (1 to 1)	0.1 (0.1 to 0.1)	741.5 (434 to 1505.7)	339.6 (173.6 to 790.7)	894 (480 to 2605.7)	360.6 (163.5 to 1392.7)
		Female	2 (1 to 3)	0.6 (0.3 to 0.9)	0 (0 to 0)	0 (0 to 0)	21 (15 to 29)	4 (2.8 to 5.4)	0 (0 to 0)	0.1 (0 to 0.1)	876.2 (485.3 to 1715.2)	519.1 (269.7 to 1138.4)	1038.7 (509.4 to 2662.3)	518.6 (227.9 to 1680.6)
		Male	5 (3 to 7)	1.8 (0.9 to 2.4)	0 (0 to 0)	0 (0 to 0)	39 (29 to 55)	6.6 (5 to 9.2)	1 (0 to 1)	0.1 (0.1 to 0.2)	683.4 (398.7 to 1447.9)	273.3 (135.9 to 681.1)	835 (439.8 to 2699.5)	302.7 (132.8 to 1227.1)
	Sexually transmitted infections excluding HIV	Both	210 (99 to 415)	24.4 (13.7 to 43.6)	2 (1 to 4)	0.2 (0.1 to 0.4)	201 (120 to 332)	20.1 (11.4 to 33.6)	1 (0 to 2)	0.1 (0.1 to 0.3)	-4 (-39.7 to 48.1)	-17.7 (-40.5 to 5)	-39.9 (-63 to -4.4)	-28.6 (-56.5 to 6.3)
		Female	108 (54 to 201)	28.5 (15.8 to 48.4)	1 (0 to 2)	0.2 (0.1 to 0.4)	118 (69 to 192)	23.3 (13.3 to 38.5)	0 (0 to 1)	0.1 (0 to 0.3)	9.1 (-31 to 62.6)	-18.3 (-37 to 1.6)	-40.1 (-63.2 to 0.9)	-31.5 (-59.7 to 6.7)
		Male	102 (44 to 216)	20.5 (10.6 to 39.3)	1 (0 to 2)	0.2 (0.1 to 0.4)	83 (46 to 144)	17.1 (9 to 31)	1 (0 to 1)	0.1 (0.1 to 0.3)	-18 (-50.6 to 31.7)	-16.5 (-45.2 to 15.5)	-39.6 (-64.6 to -0.2)	-25.3 (-56.1 to 16.1)
	Respiratory infections and tuberculosis	Both	8480 (6148 to 12387)	873.9 (657.6 to 1200.1)	99 (72 to 141)	16.3 (12.8 to 26)	2410 (1825 to 3421)	264.7 (204.1 to 384.7)	50 (40 to 99)	7 (5.5 to 13.8)	-71.6 (-82.2 to -57.2)	-69.7 (-79.5 to -58.4)	-49.1 (-68.4 to -13.8)	-56.9 (-68.1 to -43.1)
		Female	4059 (2680 to 6473)	829.6 (583.5 to 1229.4)	46 (31 to 72)	14.2 (10.4 to 23.1)	1027 (753 to 1468)	225 (165.7 to 324.3)	19 (14 to 39)	5.1 (3.6 to 10.6)	-74.7 (-85.4 to -58.8)	-72.9 (-83.3 to -60.3)	-59.1 (-78.4 to -21)	-64.2 (-76.5 to -49.5)
		Male	4421 (3013 to 6601)	917.8 (675 to 1298.8)	53 (36 to 78)	18.7 (14.4 to 31.9)	1384 (1056 to 2019)	306 (237.8 to 471.7)	32 (25 to 65)	9.2 (7.2 to 19)	-68.7 (-80.6 to -51.9)	-66.7 (-77.6 to -52.8)	-40.4 (-63.4 to -0.4)	-51 (-64.1 to -33)
	Tuberculosis	Both	219 (170 to 272)	37 (28.9 to 44.4)	4 (3 to 5)	1 (0.8 to 1.3)	55 (44 to 69)	5.6 (4.6 to 7)	1 (1 to 1)	0.2 (0.1 to 0.2)	-75.1 (-81 to -65)	-84.7 (-87.9 to -78.8)	-68.6 (-76.3 to -51.8)	-85.4 (-89.1 to -76.8)
		Female	107 (64 to 147)	34.3 (20.3 to 44.4)	2 (1 to 2)	0.8 (0.4 to 1.1)	24 (17 to 33)	4.8 (3.5 to 6.5)	0 (0 to 1)	0.1 (0.1 to 0.2)	-77.9 (-85.6 to -56.7)	-86 (-90.3 to -71.7)	-73.8 (-83.3 to -26)	-87.6 (-92.2 to -63.2)
		Male	113 (90 to 143)	39.5 (31.5 to 49.4)	2 (2 to 3)	1.2 (0.9 to 1.6)	31 (25 to 38)	6.5 (5.4 to 7.9)	1 (1 to 1)	0.2 (0.2 to 0.2)	-72.4 (-79.3 to -63.1)	-83.5 (-87.5 to -78.1)	-64.9 (-74.3 to -51.8)	-83.7 (-88.2 to -77.6)

Province	Cause	Sex	1990				2019				1990-2019 percent change (%)			
			DALYs		Death		DALYs		Death		DALYs		Death	
			Number	Rate	Number	Rate	Number	Rate	Number	Rate	Number	Rate	Number	Rate
	Lower respiratory infections	Both	7270 (4966 to 11087)	717.1 (517.4 to 1021.1)	95 (68 to 137)	15.2 (11.9 to 24.9)	1209 (947 to 2109)	145 (114.2 to 256.4)	49 (39 to 98)	6.8 (5.4 to 13.7)	-83.4 (-90.6 to -70.5)	-79.8 (-87.7 to -68.5)	-48.3 (-68.5 to -11.6)	-55 (-66.8 to -40.8)
		Female	3487 (2175 to 5833)	680.1 (450 to 1062.8)	45 (29 to 70)	13.3 (9.6 to 22.3)	466 (349 to 854)	110.9 (82.8 to 203.5)	18 (13 to 39)	5 (3.5 to 10.5)	-86.6 (-93.5 to -73.1)	-83.7 (-91.4 to -71.3)	-58.5 (-78.4 to -19.1)	-62.7 (-76.5 to -47.2)
		Male	3783 (2401 to 5865)	754.2 (515.3 to 1124.7)	51 (34 to 76)	17.4 (13.3 to 30.5)	743 (571 to 1335)	180.9 (139.7 to 335.6)	31 (24 to 64)	8.9 (7 to 18.8)	-80.4 (-89.1 to -64)	-76 (-85.5 to -61.2)	-39.4 (-63.3 to 2.5)	-48.7 (-62.9 to -29.7)
	Upper respiratory infections	Both	697 (412 to 1107)	85 (51.3 to 133.6)	0 (0 to 0)	0 (0 to 0)	858 (516 to 1336)	85 (51.6 to 133.9)	0 (0 to 0)	0 (0 to 0)	23.1 (12.8 to 34.8)	0.1 (-5.7 to 5.4)	-63.1 (-90.4 to 619.8)	-64.3 (-90.4 to 377.9)
		Female	335 (199 to 530)	83.2 (50.5 to 130.2)	0 (0 to 0)	0 (0 to 0)	409 (247 to 642)	83 (49.8 to 130.7)	0 (0 to 0)	0 (0 to 0)	22.2 (10.8 to 35.6)	-0.3 (-7.2 to 6.6)	-73.2 (-94 to 696)	-71.7 (-93.3 to 390)
		Male	362 (214 to 576)	86.6 (51.6 to 136.7)	0 (0 to 0)	0 (0 to 0.1)	449 (269 to 707)	87 (52.2 to 137.1)	0 (0 to 0)	0 (0 to 0)	24 (11.1 to 36.8)	0.4 (-7.4 to 8.1)	-57.1 (-88.4 to 674.1)	-59.3 (-88.8 to 444.2)
	Otitis media	Both	294 (169 to 479)	34.8 (20 to 57.4)	0 (0 to 0)	0 (0 to 0)	288 (165 to 482)	29 (16.3 to 48.3)	0 (0 to 0)	0 (0 to 0)	-1.9 (-11.2 to 9.2)	-16.7 (-22.5 to -10.7)	-78.5 (-92.1 to -36.5)	-80.1 (-91.4 to -53.8)
		Female	130 (75 to 210)	32 (18.3 to 52.9)	0 (0 to 0)	0 (0 to 0)	128 (73 to 216)	26.2 (14.9 to 43.7)	0 (0 to 0)	0 (0 to 0)	-1.6 (-12.5 to 13.7)	-18 (-25.4 to -9.9)	-50.3 (-81.3 to 84.5)	-67.2 (-85.7 to -2.9)
		Male	164 (93 to 270)	37.5 (21.4 to 61.7)	0 (0 to 0)	0 (0 to 0)	161 (90 to 271)	31.6 (17.8 to 52.8)	0 (0 to 0)	0 (0 to 0)	-2 (-12.2 to 10.5)	-15.6 (-22.7 to -7.3)	-83.3 (-94.8 to -34.2)	-83.3 (-93.5 to -50.8)
	Enteric infections	Both	3315 (2309 to 4794)	360.7 (264.4 to 489.3)	31 (20 to 47)	5.4 (3.4 to 8.8)	2498 (1801 to 3320)	267 (193.2 to 352.7)	10 (6 to 17)	1.2 (0.7 to 2.3)	-24.6 (-50.5 to 6.9)	-26 (-48.5 to -2.5)	-68.6 (-83.7 to -46.7)	-76.8 (-85.2 to -65.3)
		Female	1653 (1088 to 2658)	371.9 (256.1 to 543.2)	16 (9 to 27)	6 (2.9 to 11.2)	1212 (876 to 1628)	262.7 (190.6 to 352.4)	5 (3 to 9)	1.2 (0.6 to 2.4)	-26.7 (-55.3 to 10.8)	-29.4 (-53 to -1.1)	-70.1 (-85.5 to -46.3)	-80.1 (-87.3 to -67.1)
		Male	1662 (1120 to 2557)	347.8 (240.5 to 513.3)	15 (9 to 26)	4.4 (2.4 to 9.7)	1286 (914 to 1726)	271.2 (192.6 to 360.4)	5 (3 to 12)	1.3 (0.6 to 3.2)	-22.6 (-53 to 14.4)	-22 (-48.9 to 8)	-66.9 (-86 to -32.1)	-70.9 (-83.9 to -56.4)
	Diarrheal diseases	Both	2724 (1777 to 4082)	302.8 (214 to 424.7)	23 (13 to 39)	4.5 (2.6 to 7.9)	2302 (1613 to 3118)	246.3 (173.6 to 331.8)	7 (3 to 14)	0.9 (0.4 to 2)	-15.5 (-47 to 22.6)	-18.6 (-44.4 to 9.6)	-70.6 (-87.4 to -42.6)	-79.2 (-87.8 to -65.8)
		Female	1360 (833 to 2366)	313.6 (205.4 to 479.5)	12 (6 to 23)	5.2 (2.1 to 10.3)	1123 (795 to 1521)	243.8 (173.2 to 330.3)	3 (1 to 8)	0.9 (0.4 to 2.1)	-17.4 (-53.2 to 29.9)	-22.3 (-50.5 to 11.9)	-71.5 (-89.1 to -41.3)	-82.2 (-89.6 to -67.4)
		Male	1364 (862 to 2265)	290.2 (193.4 to 453.9)	11 (5 to 22)	3.5 (1.6 to 8.5)	1178 (819 to 1629)	248.9 (173.5 to 338)	3 (1 to 10)	0.9 (0.4 to 2.9)	-13.6 (-49.6 to 34)	-14.2 (-46.6 to 21.6)	-69.5 (-90 to -24.1)	-73.4 (-87.7 to -57.7)
	Typhoid and paratyphoid	Both	444 (178 to 907)	43.9 (18.3 to 87)	6 (2 to 12)	0.6 (0.3 to 1.2)	140 (59 to 272)	14.7 (6.2 to 28.9)	2 (1 to 4)	0.2 (0.1 to 0.4)	-68.5 (-77 to -57.4)	-66.4 (-75.1 to -55.3)	-66 (-75 to -53.9)	-67 (-75.1 to -56.6)
		Female	206 (83 to 409)	41.5 (17.1 to 82.1)	3 (1 to 6)	0.6 (0.2 to 1.2)	64 (26 to 127)	13.6 (5.5 to 27.2)	1 (0 to 2)	0.2 (0.1 to 0.4)	-69.1 (-77.9 to -55.3)	-67.3 (-76.1 to -54.1)	-66.5 (-76.3 to -51.2)	-68.1 (-76.9 to -55.4)
		Male	239 (95 to 489)	46.1 (18.7 to 91.9)	3 (1 to 6)	0.7 (0.3 to 1.3)	77 (31 to 153)	15.8 (6.5 to 32)	1 (0 to 2)	0.2 (0.1 to 0.5)	-67.9 (-77.7 to -54.5)	-65.6 (-75.4 to -52.8)	-65.6 (-75.7 to -51.8)	-66.1 (-75.4 to -53.3)
	Invasive Non-typhoidal Salmonella (iNTS)	Both	119 (63 to 205)	11.7 (6.4 to 19.2)	2 (1 to 3)	0.2 (0.1 to 0.3)	52 (26 to 93)	5.5 (2.8 to 9.8)	1 (0 to 2)	0.1 (0 to 0.2)	-56.1 (-73.6 to -35.6)	-53.1 (-70.3 to -33.8)	-46 (-65.3 to -23.7)	-51.8 (-66 to -36.5)
		Female	71 (37 to 126)	14 (7.5 to 24)	1 (1 to 2)	0.2 (0.1 to 0.4)	23 (11 to 42)	4.9 (2.4 to 9.1)	0 (0 to 1)	0.1 (0 to 0.1)	-67.5 (-80.8 to -50.3)	-64.9 (-78.1 to -48.9)	-58.8 (-74.8 to -38.3)	-63.2 (-74.6 to -48.2)
		Male	48 (26 to 82)	9.5 (5.3 to 15.6)	1 (0 to 1)	0.2 (0.1 to 0.3)	29 (14 to 52)	6 (3 to 10.7)	0 (0 to 1)	0.1 (0.1 to 0.2)	-39.3 (-64 to -9.9)	-36.3 (-59.4 to -8.8)	-27.7 (-53.8 to 2)	-36.1 (-55.7 to -12.3)
	Other intestinal infectious diseases	Both	28 (5 to 63)	2.4 (0.6 to 5.4)	0 (0 to 1)	0 (0 to 0.1)	4 (3 to 6)	0.4 (0.3 to 0.6)	0 (0 to 0)	0 (0 to 0)	-85.1 (-94 to -19.2)	-82 (-92.8 to -24.6)	-87.8 (-95.8 to -28.6)	-85.5 (-94.5 to -48.3)
		Female	16 (1 to 44)	2.8 (0.4 to 7.5)	0 (0 to 0)	0 (0 to 0.1)	2 (1 to 3)	0.4 (0.2 to 0.7)	0 (0 to 0)	0 (0 to 0)	-87.6 (-96.2 to 52.7)	-84.9 (-95.3 to 30.9)	-90 (-97.5 to 67.7)	-87.9 (-96.8 to -20.3)

Province	Cause	Sex	1990				2019				1990-2019 percent change (%)			
			DALYs		Death		DALYs		Death		DALYs		Death	
			Number	Rate	Number	Rate	Number	Rate	Number	Rate	Number	Rate	Number	Rate
		Male	12 (2 to 30)	2 (0.4 to 5)	0 (0 to 0)	0 (0 to 0.1)	2 (1 to 3)	0.4 (0.3 to 0.7)	0 (0 to 0)	0 (0 to 0)	-81.5 (-93.7 to 41.6)	-78.2 (-92.5 to 17.6)	-84.7 (-96.1 to 54.3)	-82.3 (-94.8 to -24.5)
	Neglected tropical diseases and malaria	Both	1260 (262 to 14588)	178.5 (31.8 to 2255.7)	18 (1 to 266)	3.4 (0.2 to 52.5)	148 (100 to 220)	15.3 (10.4 to 22.6)	1 (0 to 1)	0.1 (0 to 0.1)	-88.3 (-99 to -43)	-91.4 (-99.3 to -51.6)	-96.9 (-99.8 to -50.4)	-98 (-99.9 to -64.9)
		Female	626 (124 to 6755)	168 (32.6 to 1920.3)	8 (0 to 111)	2.6 (0.1 to 37.3)	71 (46 to 105)	15 (9.8 to 22.4)	0 (0 to 0)	0 (0 to 0.1)	-88.6 (-98.9 to -41.6)	-91.1 (-99.2 to -53.9)	-97.6 (-99.8 to -47.4)	-98.3 (-99.9 to -66.6)
		Male	635 (119 to 7323)	187.7 (27.4 to 2461.3)	10 (1 to 149)	4.1 (0.2 to 63)	77 (49 to 119)	15.5 (9.8 to 24.3)	0 (0 to 1)	0.1 (0.1 to 0.2)	-87.9 (-99 to -33.3)	-91.7 (-99.4 to -39.8)	-96.3 (-99.8 to -42)	-97.8 (-99.9 to -56.1)
	Malaria	Both	929 (14 to 14360)	140.4 (1.7 to 2216.8)	16 (0 to 265)	3.1 (0 to 52.3)	11 (8 to 15)	1.1 (0.8 to 1.5)	0 (0 to 0)	0 (0 to 0)	-98.8 (-99.9 to -19.9)	-99.2 (-99.9 to -36.6)	-100 (-100 to -100)	-100 (-100 to -100)
		Female	463 (8 to 6618)	127.2 (2 to 1886.2)	7 (0 to 110)	2.4 (0 to 37.2)	6 (4 to 8)	1.1 (0.8 to 1.5)	0 (0 to 0)	0 (0 to 0)	-98.8 (-99.9 to -25.9)	-99.1 (-99.9 to -43.3)	-100 (-100 to -100)	-100 (-100 to -100)
		Male	466 (6 to 7229)	152.1 (1.4 to 2439)	9 (0 to 148)	3.8 (0 to 62.8)	6 (4 to 8)	1.1 (0.8 to 1.5)	0 (0 to 0)	0 (0 to 0)	-98.8 (-99.9 to -10.2)	-99.3 (-100 to -25.9)	-100 (-100 to -100)	-100 (-100 to -100)
	Leishmaniasis	Both	0 (0 to 1)	0 (0 to 0.1)	0 (0 to 0)	0 (0 to 0)	0 (0 to 0)	0 (0 to 0)	0 (0 to 0)	0 (0 to 0)	-28.9 (-80.8 to 234.6)	-41.3 (-84 to 180.4)	0 (0 to 0)	0 (0 to 0)
		Female	0 (0 to 0)	0 (0 to 0.1)	0 (0 to 0)	0 (0 to 0)	0 (0 to 0)	0 (0 to 0)	0 (0 to 0)	0 (0 to 0)	-24 (-79.4 to 270.1)	-42.8 (-84.5 to 174.7)	0 (0 to 0)	0 (0 to 0)
		Male	0 (0 to 0)	0 (0 to 0)	0 (0 to 0)	0 (0 to 0)	0 (0 to 0)	0 (0 to 0)	0 (0 to 0)	0 (0 to 0)	-36.4 (-82.6 to 185.3)	-39.8 (-83.7 to 175.6)	0 (0 to 0)	0 (0 to 0)
	Schistosomiasis	Both	0 (0 to 0)	0 (0 to 0)	0 (0 to 0)	0 (0 to 0)	0 (0 to 0)	0 (0 to 0)	0 (0 to 0)	0 (0 to 0)	0 (0 to 0)	0 (0 to 0)	0 (0 to 0)	0 (0 to 0)
		Female	0 (0 to 0)	0 (0 to 0)	0 (0 to 0)	0 (0 to 0)	0 (0 to 0)	0 (0 to 0)	0 (0 to 0)	0 (0 to 0)	0 (0 to 0)	0 (0 to 0)	0 (0 to 0)	0 (0 to 0)
		Male	0 (0 to 0)	0 (0 to 0)	0 (0 to 0)	0 (0 to 0)	0 (0 to 0)	0 (0 to 0)	0 (0 to 0)	0 (0 to 0)	0 (0 to 0)	0 (0 to 0)	0 (0 to 0)	0 (0 to 0)
	Cystic echinococcosis	Both	50 (25 to 83)	6.9 (4.4 to 9.9)	1 (0 to 1)	0.1 (0.1 to 0.2)	27 (18 to 38)	2.6 (1.8 to 3.7)	0 (0 to 0)	0 (0 to 0)	-46.6 (-71.5 to 6.7)	-61.9 (-77 to -37.5)	-73.1 (-88.1 to -37.7)	-81.9 (-91.4 to -63.8)
		Female	22 (9 to 43)	6.3 (3.3 to 10)	0 (0 to 1)	0.1 (0 to 0.2)	14 (9 to 21)	2.8 (1.8 to 4)	0 (0 to 0)	0 (0 to 0)	-37.1 (-71 to 58.7)	-55.3 (-75.6 to -14.9)	-72 (-91.8 to 5.1)	-80.5 (-94.2 to -41.9)
		Male	27 (11 to 50)	7.5 (3.9 to 11.6)	0 (0 to 1)	0.1 (0.1 to 0.2)	12 (8 to 18)	2.5 (1.6 to 3.5)	0 (0 to 0)	0 (0 to 0)	-54.3 (-79.1 to 15.5)	-67.1 (-82.7 to -34.8)	-73.8 (-91.7 to -18)	-82.7 (-94.4 to -54.1)
	Trachoma	Both	9 (5 to 13)	3.1 (1.9 to 5)	--	--	3 (2 to 5)	0.4 (0.2 to 0.7)	--	--	-62.3 (-71.2 to -51)	-86.5 (-89.5 to -82.7)	--	--
		Female	7 (4 to 11)	5.6 (3.3 to 9)	--	--	2 (1 to 4)	0.6 (0.4 to 1.1)	--	--	-65.3 (-74 to -54.2)	-88.6 (-91.4 to -85)	--	--
		Male	1 (1 to 2)	0.9 (0.5 to 1.6)	--	--	1 (0 to 1)	0.2 (0.1 to 0.3)	--	--	-46.9 (-62.2 to -23.3)	-78.5 (-84.3 to -71)	--	--
	Rabies	Both	5 (1 to 9)	1 (0.1 to 1.6)	0 (0 to 0)	0 (0 to 0)	3 (0 to 4)	0.3 (0 to 0.4)	0 (0 to 0)	0 (0 to 0)	-49.9 (-76.1 to -11.3)	-72 (-84.8 to -50.3)	-40.4 (-67.7 to 4.2)	-71.2 (-83.9 to -49.7)
		Female	1 (0 to 3)	0.8 (0 to 1.6)	0 (0 to 0)	0 (0 to 0.1)	1 (0 to 1)	0.2 (0 to 0.3)	0 (0 to 0)	0 (0 to 0)	-37.9 (-81.7 to 35.8)	-77.8 (-89.7 to -51.5)	-40.1 (-73.2 to 35.4)	-78.7 (-90.3 to -52.1)
		Male	4 (1 to 7)	1.2 (0.2 to 2.1)	0 (0 to 0)	0 (0 to 0)	2 (0 to 3)	0.4 (0 to 0.6)	0 (0 to 0)	0 (0 to 0)	-54 (-77.9 to -7)	-68.8 (-84.7 to -38.9)	-40.6 (-70.9 to 15)	-65.2 (-82.6 to -35.5)
	Intestinal nematode infections	Both	75 (33 to 135)	7.7 (3.9 to 13)	1 (0 to 1)	0 (0 to 0.1)	15 (8 to 27)	1.6 (0.8 to 2.8)	0 (0 to 0)	0 (0 to 0)	-79.7 (-91.2 to -54.7)	-79.8 (-90.5 to -60.7)	-93.7 (-97.9 to -60.5)	-91.5 (-97 to -67.7)

Province	Cause	Sex	1990				2019				1990-2019 percent change (%)			
			DALYs		Death		DALYs		Death		DALYs		Death	
			Number	Rate	Number	Rate	Number	Rate	Number	Rate	Number	Rate	Number	Rate
		Female	41 (15 to 86)	9.1 (4 to 16.7)	0 (0 to 1)	0 (0 to 0.1)	9 (4 to 15)	1.8 (0.9 to 3.3)	0 (0 to 0)	0 (0 to 0)	-78.9 (-91.7 to -43.5)	-80 (-91.1 to -55.9)	-94.2 (-98.8 to 29.4)	-92.2 (-98.2 to -31.8)
		Male	34 (11 to 74)	6.4 (2.5 to 12.8)	0 (0 to 1)	0 (0 to 0.1)	7 (3 to 12)	1.3 (0.6 to 2.4)	0 (0 to 0)	0 (0 to 0)	-80.6 (-93.4 to -38.3)	-79.4 (-92.2 to -47.1)	-93.3 (-98.4 to -7.2)	-91 (-97.7 to -43)
	Food-borne trematodiases	Both	2 (1 to 5)	0.5 (0.1 to 1)	--	--	10 (2 to 21)	1 (0.2 to 2.1)	--	--	326.5 (224.7 to 446.2)	109.4 (62.9 to 162.8)	--	--
		Female	1 (0 to 2)	0.4 (0.1 to 0.8)	--	--	4 (1 to 8)	0.8 (0.2 to 1.7)	--	--	318.2 (211.8 to 446.1)	102.4 (49.1 to 163.8)	--	--
		Male	1 (0 to 3)	0.6 (0.1 to 1.2)	--	--	6 (1 to 13)	1.2 (0.3 to 2.5)	--	--	332 (174.7 to 507.5)	115.3 (45.7 to 191.9)	--	--
	Leprosy	Both	0 (0 to 0)	0 (0 to 0.1)	--	--	0 (0 to 0)	0 (0 to 0.1)	--	--	81.2 (67 to 98)	-26.8 (-32 to -20.6)	--	--
		Female	0 (0 to 0)	0 (0 to 0.1)	--	--	0 (0 to 0)	0 (0 to 0)	--	--	74.9 (49.6 to 101.6)	-31.9 (-40.9 to -22.4)	--	--
		Male	0 (0 to 0)	0.1 (0 to 0.1)	--	--	0 (0 to 0)	0 (0 to 0.1)	--	--	84.3 (66.2 to 108.3)	-22.6 (-29.7 to -13.6)	--	--
	Other neglected tropical diseases	Both	190 (113 to 301)	18.8 (11.7 to 28.7)	0 (0 to 1)	0.1 (0 to 0.2)	78 (45 to 132)	8.3 (4.7 to 14)	0 (0 to 1)	0 (0 to 0.1)	-59 (-74.5 to -32.4)	-56.1 (-71.5 to -31.8)	-37.1 (-63.9 to 13.8)	-50.6 (-66.8 to -16.4)
		Female	89 (48 to 151)	18.6 (10.8 to 29.8)	0 (0 to 1)	0 (0 to 0.2)	35 (18 to 63)	7.6 (3.9 to 13.7)	0 (0 to 0)	0 (0 to 0.1)	-60.7 (-78.5 to -27)	-59.1 (-76.9 to -30.4)	-44.5 (-73.2 to 24.2)	-60.3 (-76.5 to -21.6)
		Male	100 (52 to 169)	19 (10.6 to 30.9)	0 (0 to 1)	0.1 (0 to 0.2)	43 (22 to 82)	8.9 (4.4 to 16.9)	0 (0 to 1)	0.1 (0 to 0.1)	-57.4 (-78.6 to -12.8)	-53.3 (-75.5 to -10.2)	-34.1 (-67 to 37.2)	-44.8 (-67 to 4.8)
	Other infectious diseases	Both	10964 (5371 to 19953)	960.3 (506.3 to 1680.3)	130 (66 to 234)	13.2 (7.7 to 21.8)	859 (687 to 1118)	91.3 (72.9 to 119.7)	14 (12 to 18)	1.7 (1.4 to 2.1)	-92.2 (-95.7 to -84.3)	-90.5 (-94.6 to -82.5)	-89 (-93.9 to -78.5)	-87.1 (-92.3 to -77.4)
		Female	5810 (2813 to 10533)	1025.1 (525.8 to 1814.3)	68 (33 to 123)	13.6 (7.7 to 23.2)	391 (301 to 515)	84.2 (64.6 to 111.6)	5 (4 to 7)	1.3 (1 to 1.7)	-93.3 (-96.3 to -86.6)	-91.8 (-95.4 to -84.4)	-92.2 (-95.7 to -84)	-90.7 (-94.7 to -83.1)
		Male	5154 (2456 to 9593)	896.3 (466.3 to 1610.9)	62 (31 to 114)	12.7 (7.4 to 21.2)	468 (369 to 612)	98.7 (77.3 to 129.3)	9 (7 to 12)	2.2 (1.7 to 2.8)	-90.9 (-95.1 to -81.5)	-89 (-93.8 to -78.9)	-85.6 (-92.4 to -70.4)	-82.9 (-90 to -69.2)
	Meningitis	Both	883 (594 to 1337)	85.3 (60.8 to 124.6)	10 (7 to 16)	1.2 (0.9 to 1.7)	170 (131 to 214)	17.8 (13.8 to 22.4)	2 (2 to 3)	0.3 (0.2 to 0.3)	-80.7 (-87.6 to -68.7)	-79.2 (-86 to -68.1)	-78 (-86.2 to -62.8)	-77.9 (-84.9 to -64.3)
		Female	458 (276 to 840)	89.4 (58.1 to 153.5)	5 (3 to 9)	1.2 (0.8 to 1.9)	89 (66 to 119)	18.6 (13.7 to 25.3)	1 (1 to 1)	0.2 (0.1 to 0.3)	-80.6 (-89 to -67.1)	-79.2 (-87.6 to -67.3)	-83.8 (-90.7 to -70.9)	-83.3 (-90.1 to -72.9)
		Male	425 (281 to 644)	81.3 (56.7 to 116.9)	5 (3 to 8)	1.2 (0.9 to 1.7)	82 (62 to 109)	17 (12.9 to 22.6)	1 (1 to 2)	0.3 (0.3 to 0.5)	-80.7 (-88.3 to -65.6)	-79.1 (-86.9 to -63.9)	-72.3 (-83.3 to -46.9)	-72.5 (-82.4 to -49.4)
	Encephalitis	Both	142 (94 to 225)	15.6 (11.1 to 25.1)	2 (1 to 3)	0.2 (0.2 to 0.4)	94 (68 to 149)	9.8 (7.1 to 15.3)	1 (1 to 2)	0.2 (0.1 to 0.3)	-33.8 (-60.1 to -1)	-37.4 (-58.7 to -12.4)	-19 (-51.6 to 26.4)	-36.4 (-56.7 to -10.6)
		Female	78 (47 to 127)	17.2 (11.4 to 26.3)	1 (0 to 1)	0.3 (0.2 to 0.4)	46 (32 to 73)	9.6 (6.8 to 15)	1 (0 to 1)	0.1 (0.1 to 0.2)	-41.3 (-69.8 to -2.9)	-44 (-68.2 to -15.4)	-35.7 (-68.8 to 8.5)	-49.1 (-69.7 to -22.7)
		Male	63 (37 to 117)	14 (9.1 to 26)	1 (0 to 2)	0.2 (0.1 to 0.5)	48 (33 to 86)	9.9 (6.9 to 17.7)	1 (1 to 2)	0.2 (0.1 to 0.3)	-24.4 (-58.4 to 30.3)	-29.3 (-57.6 to 12.4)	0.3 (-44.9 to 79.5)	-21.6 (-51.4 to 26.2)
	Diphtheria	Both	14 (6 to 28)	1.2 (0.6 to 2.3)	0 (0 to 0)	0 (0 to 0)	0 (0 to 1)	0.1 (0 to 0.1)	0 (0 to 0)	0 (0 to 0)	-96.5 (-98.8 to -90.6)	-95.8 (-98.5 to -89)	-95.6 (-98.3 to -88.8)	-95.1 (-98.1 to -88.4)
		Female	7 (2 to 16)	1.2 (0.3 to 2.8)	0 (0 to 0)	0 (0 to 0)	0 (0 to 0)	0 (0 to 0.1)	0 (0 to 0)	0 (0 to 0)	-97.3 (-99.2 to -88.3)	-96.6 (-99 to -86.4)	-96.7 (-98.9 to -86.5)	-96.1 (-98.7 to -86.1)

Province	Cause	Sex	1990				2019				1990-2019 percent change (%)			
			DALYs		Death		DALYs		Death		DALYs		Death	
			Number	Rate	Number	Rate	Number	Rate	Number	Rate	Number	Rate	Number	Rate
		Male	7 (2 to 17)	1.3 (0.4 to 2.8)	0 (0 to 0)	0 (0 to 0)	0 (0 to 1)	0.1 (0 to 0.1)	0 (0 to 0)	0 (0 to 0)	-95.8 (-98.7 to -82.2)	-95.1 (-98.5 to -82.7)	-94.7 (-98.2 to -79.2)	-94.2 (-98 to -81.8)
	Whooping cough	Both	1664 (92 to 7505)	134.9 (7.4 to 610.8)	19 (1 to 86)	1.5 (0.1 to 7)	43 (3 to 169)	4.7 (0.3 to 18.5)	0 (0 to 2)	0.1 (0 to 0.2)	-97.4 (-99.9 to -33.9)	-96.5 (-99.8 to -10.7)	-97.5 (-99.9 to -13.1)	-96.6 (-99.9 to 17.1)
		Female	968 (53 to 4293)	160.1 (8.8 to 708.3)	11 (0 to 49)	1.8 (0.1 to 8.2)	24 (2 to 93)	5.3 (0.4 to 21)	0 (0 to 1)	0.1 (0 to 0.2)	-97.6 (-99.9 to -41.3)	-96.7 (-99.8 to -19.8)	-97.6 (-99.9 to -16.9)	-96.8 (-99.9 to 13.1)
		Male	697 (38 to 3152)	110.7 (6 to 502.4)	8 (0 to 36)	1.3 (0 to 5.8)	19 (1 to 80)	4.1 (0.3 to 17.1)	0 (0 to 1)	0 (0 to 0.2)	-97.2 (-99.8 to -28.9)	-96.3 (-99.8 to -3.3)	-97.3 (-99.9 to -4.4)	-96.4 (-99.9 to 27.9)
	Tetanus	Both	139 (43 to 281)	12.8 (4.6 to 26.3)	2 (1 to 4)	0.2 (0.1 to 0.4)	7 (6 to 12)	0.8 (0.6 to 1.2)	0 (0 to 0)	0 (0 to 0)	-94.7 (-97.6 to -80.6)	-94 (-97.2 to -80.5)	-91.7 (-96 to -74.7)	-92.2 (-96 to -79)
		Female	70 (17 to 165)	12.8 (3.7 to 29.6)	1 (0 to 2)	0.2 (0.1 to 0.4)	3 (3 to 5)	0.7 (0.5 to 1)	0 (0 to 0)	0 (0 to 0)	-95.2 (-98.1 to -77.4)	-94.5 (-97.8 to -78.5)	-92.6 (-97 to -72.4)	-92.9 (-96.8 to -79.9)
		Male	69 (21 to 153)	12.8 (4.1 to 28.9)	1 (0 to 2)	0.2 (0.1 to 0.5)	4 (3 to 7)	0.8 (0.6 to 1.4)	0 (0 to 0)	0 (0 to 0)	-94.1 (-97.7 to -75.9)	-93.4 (-97.3 to -75.1)	-90.9 (-96.1 to -64.5)	-91.3 (-96 to -69.3)
	Measles	Both	7146 (2394 to 15371)	573.1 (191.3 to 1234.7)	83 (28 to 179)	6.7 (2.2 to 14.4)	99 (30 to 246)	10.8 (3.2 to 26.8)	1 (0 to 3)	0.1 (0 to 0.3)	-98.6 (-99.4 to -97.3)	-98.1 (-99.3 to -96.3)	-98.6 (-99.5 to -97.3)	-98.1 (-99.3 to -96.4)
		Female	3745 (1252 to 8114)	613.6 (203.7 to 1332.3)	44 (15 to 94)	7.2 (2.4 to 15.5)	49 (15 to 120)	10.9 (3.3 to 26.8)	1 (0 to 1)	0.1 (0 to 0.3)	-98.7 (-99.5 to -97.4)	-98.2 (-99.3 to -96.5)	-98.7 (-99.5 to -97.5)	-98.2 (-99.3 to -96.6)
		Male	3401 (1128 to 7327)	534.2 (177.6 to 1153)	40 (13 to 85)	6.2 (2.1 to 13.4)	51 (15 to 126)	10.7 (3.1 to 26.4)	1 (0 to 1)	0.1 (0 to 0.3)	-98.5 (-99.4 to -97)	-98 (-99.2 to -95.9)	-98.5 (-99.4 to -97)	-98 (-99.3 to -96)
	Varicella and herpes zoster	Both	76 (43 to 126)	9.2 (6.2 to 13.1)	1 (1 to 1)	0.2 (0.1 to 0.3)	38 (26 to 54)	4.1 (2.8 to 5.7)	0 (0 to 1)	0.1 (0 to 0.1)	-49.6 (-73.3 to -9)	-55.4 (-73 to -31.9)	-58.1 (-77.5 to -21.3)	-73.8 (-84.6 to -55.7)
		Female	39 (18 to 74)	9.5 (5.7 to 15.4)	0 (0 to 1)	0.2 (0.1 to 0.3)	19 (12 to 27)	4.1 (2.7 to 5.9)	0 (0 to 0)	0.1 (0 to 0.1)	-51.2 (-77.8 to 10.5)	-56.6 (-76.7 to -23.5)	-58.9 (-81.8 to -1.8)	-73.9 (-87.5 to -46.4)
		Male	37 (19 to 69)	8.9 (5.8 to 14.1)	0 (0 to 1)	0.2 (0.1 to 0.3)	19 (12 to 28)	4.1 (2.7 to 5.8)	0 (0 to 0)	0.1 (0 to 0.1)	-48 (-76.4 to 5.3)	-54.3 (-75 to -22.6)	-57.3 (-81.1 to -10.6)	-74 (-87.3 to -46)
	Acute hepatitis	Both	599 (359 to 899)	93.3 (56.4 to 136.8)	11 (6 to 16)	2.5 (1.5 to 3.9)	188 (148 to 237)	19.7 (15.5 to 24.8)	5 (3 to 6)	0.5 (0.4 to 0.7)	-68.7 (-80.8 to -41.7)	-78.9 (-87.1 to -61.1)	-58.5 (-76.2 to -15.7)	-79 (-88.4 to -55.9)
		Female	302 (143 to 494)	86.1 (40.6 to 128.9)	5 (2 to 8)	2.2 (0.9 to 3.6)	56 (43 to 73)	11.8 (9 to 15.3)	1 (1 to 1)	0.2 (0.2 to 0.3)	-81.3 (-89.9 to -56.7)	-86.3 (-91.6 to -67.9)	-78.3 (-87.7 to -40.2)	-88.6 (-94.1 to -67.4)
		Male	297 (174 to 520)	98.4 (57.9 to 166.2)	6 (3 to 11)	2.8 (1.6 to 4.9)	131 (97 to 175)	27.7 (20.6 to 37.2)	3 (2 to 5)	0.8 (0.6 to 1.2)	-55.8 (-77.2 to -13.6)	-71.8 (-85.5 to -44.7)	-42.5 (-73 to 19.8)	-70.2 (-86.3 to -37.7)
	Other unspecified infectious diseases	Both	302 (172 to 661)	35 (21 to 70.9)	3 (1 to 7)	0.6 (0.3 to 1.5)	219 (160 to 311)	23.7 (17.2 to 33.6)	4 (3 to 6)	0.5 (0.3 to 0.7)	-27.4 (-70.5 to 36.9)	-32.5 (-69.4 to 23.4)	43.2 (-56.9 to 285.2)	-10 (-65.3 to 131.7)
		Female	144 (77 to 295)	35.3 (20.3 to 67.1)	1 (1 to 3)	0.6 (0.2 to 1.7)	105 (69 to 149)	23.1 (15.1 to 32.7)	2 (1 to 3)	0.4 (0.2 to 0.6)	-26.9 (-73.5 to 42.5)	-34.5 (-72.4 to 18.6)	36.5 (-61.3 to 284)	-23.9 (-70.9 to 112.9)
		Male	158 (78 to 388)	34.7 (18.8 to 82.3)	2 (1 to 5)	0.5 (0.2 to 1.5)	114 (80 to 180)	24.3 (17 to 38.4)	2 (1 to 3)	0.6 (0.4 to 0.9)	-27.9 (-70.9 to 54.1)	-30 (-69.3 to 38.8)	49 (-54.7 to 359)	7.7 (-60.8 to 183.7)
	Maternal and neonatal disorders	Both	66631 (53328 to 80456)	5620.3 (4511.5 to 6770.4)	742 (591 to 897)	62.4 (49.7 to 75.3)	8004 (6204 to 9968)	900.5 (689.3 to 1132.4)	63 (45 to 84)	7.5 (5.3 to 9.9)	-88 (-91.2 to -83.9)	-84 (-88.5 to -78.5)	-91.5 (-94.4 to -88.1)	-88 (-92.1 to -83.2)
		Female	29960 (23929 to 36734)	5220.7 (4181.1 to 6400.4)	333 (266 to 410)	58 (46.5 to 71.3)	3678 (2827 to 4583)	842.5 (642.3 to 1057.1)	28 (20 to 37)	6.9 (4.8 to 9.1)	-87.7 (-91.2 to -83.3)	-83.9 (-88.6 to -77.9)	-91.5 (-94.4 to -87.7)	-88.2 (-92.3 to -82.9)
		Male	36670 (28866 to 45097)	5999 (4727.3 to 7369.8)	408 (319 to 504)	66.6 (52.1 to 82.1)	4326 (3299 to 5368)	955.3 (720.9 to 1200.8)	35 (24 to 47)	8.1 (5.5 to 10.9)	-88.2 (-91.7 to -83.7)	-84.1 (-88.9 to -78)	-91.5 (-94.7 to -87.8)	-87.9 (-92.5 to -82.6)
	Maternal disorders	Female	476 (328 to 642)	154.1 (107.1 to 208)	6 (4 to 9)	2 (1.3 to 2.9)	145 (106 to 188)	25.5 (18.8 to 33.2)	1 (1 to 2)	0.2 (0.2 to 0.3)	-69.5 (-78.9 to -55.1)	-83.4 (-88.5 to -75.5)	-78.8 (-87.4 to -63.2)	-89.6 (-93.8 to -82.8)

Province	Cause	Sex	1990				2019				1990-2019 percent change (%)			
			DALYs		Death		DALYs		Death		DALYs		Death	
			Number	Rate	Number	Rate	Number	Rate	Number	Rate	Number	Rate	Number	Rate
	Neonatal disorders	Both	66154 (52858 to 79952)	5544.3 (4432.6 to 6698.6)	736 (586 to 891)	61.4 (48.9 to 74.3)	7859 (6064 to 9803)	888.1 (678.7 to 1120.2)	62 (44 to 82)	7.4 (5.2 to 9.8)	-88.1 (-91.4 to -84)	-84 (-88.5 to -78.3)	-91.6 (-94.5 to -88.2)	-88 (-92.2 to -83.1)
		Female	29484 (23448 to 36257)	5066.7 (4029.6 to 6228.3)	327 (260 to 404)	56 (44.5 to 69)	3533 (2706 to 4439)	817 (619.7 to 1030.9)	27 (19 to 36)	6.6 (4.6 to 8.9)	-88 (-91.5 to -83.6)	-83.9 (-88.7 to -77.8)	-91.7 (-94.7 to -87.9)	-88.1 (-92.3 to -82.7)
		Male	36670 (28866 to 45097)	5999 (4727.3 to 7369.8)	408 (319 to 504)	66.6 (52.1 to 82.1)	4326 (3299 to 5368)	955.3 (720.9 to 1200.8)	35 (24 to 47)	8.1 (5.5 to 10.9)	-88.2 (-91.7 to -83.7)	-84.1 (-88.9 to -78)	-91.5 (-94.7 to -87.8)	-87.9 (-92.5 to -82.6)
	Nutritional deficiencies	Both	2897 (1879 to 4280)	328.3 (220.4 to 473.1)	4 (2 to 7)	0.5 (0.3 to 0.7)	1399 (847 to 2138)	146 (89.8 to 221.5)	0 (0 to 1)	0.1 (0 to 0.1)	-51.7 (-65.2 to -32.6)	-55.5 (-66.8 to -40.4)	-88.6 (-93.6 to -78.9)	-87 (-91.8 to -79.1)
		Female	1492 (946 to 2280)	364.4 (236.7 to 538.1)	2 (1 to 4)	0.5 (0.3 to 0.9)	731 (437 to 1184)	156.8 (92.8 to 247.1)	0 (0 to 0)	0 (0 to 0.1)	-51 (-67.4 to -26.8)	-57 (-70.1 to -39.3)	-91.5 (-96.1 to -80.1)	-90.2 (-95 to -81)
		Male	1405 (838 to 2228)	292.1 (182.7 to 445.7)	2 (1 to 3)	0.4 (0.3 to 0.7)	668 (382 to 1130)	134.7 (77.8 to 227.7)	0 (0 to 0)	0.1 (0.1 to 0.1)	-52.5 (-71.6 to -19)	-53.9 (-70.7 to -27.2)	-85.1 (-92.9 to -66.6)	-83.4 (-90.7 to -69.1)
	Protein-energy malnutrition	Both	584 (404 to 855)	54.9 (37.9 to 78.6)	4 (2 to 6)	0.4 (0.3 to 0.6)	297 (181 to 440)	30.5 (18.9 to 44.8)	0 (0 to 0)	0 (0 to 0.1)	-49.1 (-68.7 to -26.1)	-44.4 (-64.3 to -22.2)	-91.9 (-95.6 to -84.7)	-90.1 (-93.9 to -83.2)
		Female	307 (193 to 514)	57.5 (36.9 to 94.7)	2 (1 to 4)	0.4 (0.2 to 0.8)	142 (86 to 214)	29.9 (18.3 to 44.5)	0 (0 to 0)	0 (0 to 0)	-53.7 (-75.7 to -24.5)	-48.1 (-71.5 to -19.4)	-93.9 (-97.2 to -85.1)	-92.5 (-96.3 to -84.7)
		Male	277 (182 to 425)	52.4 (35 to 77.9)	2 (1 to 3)	0.4 (0.2 to 0.6)	155 (93 to 238)	31.2 (19 to 47.2)	0 (0 to 0)	0 (0 to 0.1)	-44.1 (-68.1 to -16)	-40.4 (-64.2 to -14.4)	-89.4 (-95.2 to -76.8)	-87.2 (-93.1 to -75.8)
	Iodine deficiency	Both	115 (70 to 174)	17.8 (11.5 to 26.2)	--	--	66 (37 to 105)	6.4 (3.6 to 10.2)	--	--	-42.7 (-60.1 to -24.5)	-63.9 (-73.4 to -54.5)	--	--
		Female	70 (41 to 106)	22 (13.8 to 32.7)	--	--	41 (23 to 67)	8.1 (4.6 to 13.1)	--	--	-41.3 (-60.4 to -17.8)	-63.2 (-73.9 to -51.1)	--	--
		Male	45 (27 to 69)	13.8 (8.6 to 20.7)	--	--	25 (14 to 40)	4.8 (2.8 to 7.7)	--	--	-44.9 (-63.1 to -26.7)	-65.2 (-75.1 to -55.6)	--	--
	Vitamin A deficiency	Both	212 (107 to 361)	16.7 (8.5 to 28.2)	--	--	13 (5 to 26)	1.4 (0.5 to 2.8)	--	--	-94.1 (-97.5 to -86.6)	-91.9 (-96.6 to -81.9)	--	--
		Female	78 (36 to 145)	12.7 (5.8 to 23.2)	--	--	5 (2 to 12)	1.1 (0.4 to 2.6)	--	--	-93.4 (-97.8 to -83.9)	-90.9 (-96.9 to -78.4)	--	--
		Male	133 (54 to 258)	20.6 (8.5 to 39.6)	--	--	7 (2 to 20)	1.6 (0.4 to 4.2)	--	--	-94.4 (-98.5 to -82.6)	-92.4 (-97.9 to -76.3)	--	--
	Dietary iron deficiency	Both	1907 (1077 to 3073)	228.1 (137.6 to 350.4)	--	--	936 (518 to 1567)	99 (56.2 to 166.2)	--	--	-50.9 (-68.5 to -22)	-56.6 (-70.8 to -36.1)	--	--
		Female	1001 (541 to 1684)	262.4 (154.1 to 415.4)	--	--	502 (264 to 879)	109.4 (59.1 to 185.8)	--	--	-49.8 (-70.8 to -13.5)	-58.3 (-73.9 to -34.6)	--	--
		Male	907 (425 to 1578)	193.7 (100.9 to 323)	--	--	434 (205 to 849)	88.2 (41.4 to 172.1)	--	--	-52.1 (-77 to 1.6)	-54.5 (-76.6 to -13.7)	--	--
	Other nutritional deficiencies	Both	79 (50 to 115)	10.8 (6.9 to 15.7)	0 (0 to 0)	0.1 (0 to 0.1)	87 (50 to 136)	8.6 (5 to 13.3)	0 (0 to 0)	0 (0 to 0)	10.8 (-17.2 to 44.5)	-20.2 (-40 to 3)	-42.5 (-63.7 to 12.9)	-63.6 (-76 to -34.9)
		Female	36 (22 to 55)	9.8 (6.1 to 14.9)	0 (0 to 0)	0 (0 to 0.1)	41 (24 to 66)	8.3 (4.8 to 13.2)	0 (0 to 0)	0 (0 to 0)	13.5 (-22.6 to 64.1)	-15.9 (-42.1 to 20.5)	-49.2 (-74.6 to 5.6)	-67.8 (-83.4 to -33)
		Male	43 (27 to 65)	11.7 (7.3 to 17.9)	0 (0 to 0)	0.1 (0 to 0.1)	46 (26 to 74)	8.9 (5.1 to 14.2)	0 (0 to 0)	0 (0 to 0)	8.5 (-26.8 to 55.5)	-23.5 (-48.2 to 9.5)	-37.2 (-67.6 to 57.9)	-60.8 (-77.4 to -15.2)
	**Non-communicable diseases**	Both	125371 (104092 to 146507)	25161.2 (21804.3 to 28572.1)	1991 (1686 to 2316)	717 (632.8 to 809.1)	162870 (137278 to 193174)	17959.4 (15436.4 to 21125.3)	3462 (3099 to 3887)	473.8 (425.6 to 532.5)	29.9 (10.6 to 51.4)	-28.6 (-37.7 to -19.9)	73.9 (44.1 to 110.4)	-33.9 (-44.1 to -21.2)

Province	Cause	Sex	1990				2019				1990-2019 percent change (%)			
			DALYs		Death		DALYs		Death		DALYs		Death	
			Number	Rate	Number	Rate	Number	Rate	Number	Rate	Number	Rate	Number	Rate
		Female	59248 (47922 to 69863)	23976.7 (20226.3 to 28009.2)	821 (679 to 976)	613.2 (518 to 723.2)	78421 (64018 to 95730)	16915.6 (13962.8 to 20449.8)	1354 (1168 to 1625)	362 (313.8 to 433.5)	32.4 (10.7 to 58.4)	-29.5 (-39.6 to -18.4)	65 (27.1 to 116.6)	-41 (-53.7 to -23.1)
		Male	66122 (54367 to 78336)	26164.5 (22146.3 to 30601)	1170 (946 to 1435)	819.1 (680.3 to 984.5)	84449 (72015 to 99798)	19150.7 (16536 to 22220.1)	2107 (1828 to 2387)	593.4 (518.1 to 667.6)	27.7 (5.4 to 55.9)	-26.8 (-39.2 to -13.8)	80.2 (38.9 to 128.5)	-27.6 (-42.4 to -9.9)
	Neoplasms	Both	10253 (8376 to 12223)	2501.1 (2046.3 to 2962.7)	298 (243 to 354)	99.3 (81.8 to 117.8)	16945 (14485 to 20026)	1934.8 (1656.9 to 2285.5)	642 (553 to 756)	81.5 (70.5 to 96)	65.3 (28.4 to 115.8)	-22.6 (-38.7 to 1.7)	115.4 (69.2 to 184)	-17.9 (-34.3 to 6.5)
		Female	4185 (3159 to 5321)	2028.5 (1573.2 to 2570.5)	112 (87 to 141)	75.5 (58.9 to 96.1)	6716 (5480 to 8427)	1468.6 (1199.9 to 1844.4)	232 (189 to 293)	55.9 (45.2 to 71)	60.5 (12.9 to 124.2)	-27.6 (-47.2 to -0.4)	107.3 (49.8 to 189.8)	-25.9 (-45.1 to 3)
		Male	6068 (4592 to 7796)	2929.3 (2258.8 to 3775)	186 (143 to 240)	123.4 (95.7 to 155.4)	10229 (8495 to 12363)	2433.3 (2030.6 to 2907.2)	410 (343 to 484)	109.3 (91.4 to 128.6)	68.6 (20.6 to 135.8)	-16.9 (-39.7 to 16.4)	120.2 (59.5 to 208.1)	-11.4 (-33.7 to 22.9)
	Lip and oral cavity cancer	Both	63 (49 to 81)	18.1 (13.9 to 23)	2 (2 to 3)	0.8 (0.6 to 0.9)	128 (105 to 157)	14.6 (12 to 17.9)	5 (4 to 6)	0.7 (0.5 to 0.8)	102.3 (44.6 to 195)	-18.9 (-42 to 18.5)	142.1 (75.2 to 256.5)	-14.1 (-37.2 to 26.8)
		Female	21 (15 to 27)	12.2 (9.2 to 15.8)	1 (0 to 1)	0.5 (0.4 to 0.6)	49 (38 to 62)	11 (8.4 to 14.1)	2 (1 to 3)	0.5 (0.4 to 0.6)	137 (56.7 to 251.5)	-10.1 (-38.9 to 34.7)	192.3 (99.6 to 350)	-6.1 (-35.1 to 47.5)
		Male	43 (30 to 58)	23.3 (16.5 to 32.1)	1 (1 to 2)	1 (0.7 to 1.3)	79 (59 to 103)	18.5 (13.8 to 23.8)	3 (2 to 4)	0.8 (0.6 to 1.1)	85.6 (13 to 198.5)	-20.6 (-51.1 to 25.8)	119 (34.3 to 249.2)	-16.1 (-46.4 to 30.1)
	Nasopharynx cancer	Both	40 (32 to 50)	9.8 (7.7 to 12.2)	1 (1 to 1)	0.3 (0.3 to 0.4)	49 (39 to 61)	5.3 (4.2 to 6.6)	2 (1 to 2)	0.2 (0.2 to 0.2)	20.6 (-13.1 to 74)	-46.6 (-61.2 to -24.6)	39.5 (1.5 to 96.6)	-45.4 (-59.3 to -23.6)
		Female	11 (8 to 15)	5.8 (4.1 to 8.1)	0 (0 to 0)	0.2 (0.1 to 0.3)	12 (9 to 15)	2.5 (2 to 3.3)	0 (0 to 0)	0.1 (0.1 to 0.1)	3 (-30.1 to 73)	-56.6 (-70.7 to -30.5)	15.3 (-23.2 to 83.9)	-59.2 (-74.5 to -34.3)
		Male	29 (22 to 39)	13.6 (10.2 to 17.9)	1 (1 to 1)	0.5 (0.4 to 0.6)	37 (28 to 49)	8.1 (6.1 to 10.5)	1 (1 to 2)	0.3 (0.2 to 0.4)	27.3 (-17.2 to 102.3)	-40.6 (-60.3 to -9.6)	48.5 (-0.4 to 124.3)	-37.3 (-57.5 to -7.9)
	Other pharynx cancer	Both	29 (22 to 36)	8.6 (6.7 to 10.7)	1 (1 to 1)	0.4 (0.3 to 0.5)	49 (41 to 60)	5.6 (4.7 to 6.9)	2 (2 to 2)	0.3 (0.2 to 0.3)	72.1 (25.4 to 140.8)	-34.5 (-51.6 to -9.4)	93.9 (44.7 to 167.8)	-32.5 (-50.7 to -6.7)
		Female	16 (11 to 21)	9.8 (6.8 to 13.4)	1 (0 to 1)	0.4 (0.3 to 0.6)	22 (17 to 29)	5 (3.8 to 6.5)	1 (1 to 1)	0.2 (0.2 to 0.3)	44.7 (-7.4 to 129.1)	-49.4 (-67.2 to -19.4)	64.2 (6 to 162.1)	-48 (-66.7 to -14.2)
		Male	13 (9 to 18)	7.3 (5.3 to 10)	0 (0 to 1)	0.3 (0.2 to 0.4)	27 (20 to 35)	6.3 (4.8 to 8.2)	1 (1 to 1)	0.3 (0.2 to 0.4)	104.5 (32.3 to 221.7)	-13.9 (-43.8 to 32.3)	128 (47.9 to 250.9)	-9.8 (-39.2 to 36.3)
	Esophageal cancer	Both	184 (136 to 315)	58.6 (44.3 to 101.1)	7 (5 to 12)	2.7 (2 to 4.7)	360 (269 to 644)	42.9 (32.2 to 76.2)	16 (12 to 28)	2.1 (1.6 to 3.5)	95.9 (39.6 to 179.9)	-26.8 (-47.1 to 1.7)	125.1 (62.3 to 213.8)	-23 (-43.7 to 5.7)
		Female	71 (49 to 134)	49.3 (34 to 94.3)	3 (2 to 5)	2.3 (1.6 to 4.2)	104 (71 to 222)	24.2 (16.5 to 50.8)	5 (3 to 9)	1.2 (0.8 to 2.3)	46.4 (-3.1 to 128.2)	-50.9 (-67.4 to -23.9)	66.2 (9.8 to 157.9)	-49.1 (-67.5 to -22.3)
		Male	113 (75 to 204)	66.9 (45.8 to 119.1)	4 (3 to 8)	3.1 (2.1 to 5.3)	257 (186 to 461)	62.5 (45.3 to 112.9)	11 (8 to 20)	3 (2.2 to 5.3)	126.9 (45.5 to 253.7)	-6.6 (-39.8 to 42.3)	163.1 (74.7 to 311.3)	-1.3 (-34.9 to 49.5)
	Stomach cancer	Both	2190 (1687 to 2702)	675.3 (530.3 to 829.4)	81 (64 to 100)	30.1 (24.3 to 36.8)	3232 (2683 to 3869)	382.2 (318.9 to 456.4)	141 (118 to 167)	18.4 (15.4 to 21.8)	47.6 (9.5 to 102)	-43.4 (-57.6 to -24.5)	74.3 (31.2 to 131.8)	-39 (-53.2 to -21.3)
		Female	675 (495 to 902)	433.2 (327.7 to 593.9)	24 (18 to 33)	19 (14.3 to 28.5)	946 (744 to 1230)	214.5 (168.9 to 280.8)	40 (32 to 52)	10 (7.9 to 13)	40.2 (-5.4 to 105.3)	-50.5 (-65.7 to -28.4)	67.3 (13.8 to 145)	-47 (-65 to -22.6)
		Male	1515 (1091 to 2032)	892.5 (652.2 to 1175.8)	57 (41 to 75)	40.9 (30.1 to 52.6)	2286 (1827 to 2801)	558 (449.9 to 678.7)	101 (82 to 123)	27.2 (22.3 to 32.8)	50.8 (4.5 to 121.1)	-37.5 (-55.7 to -9.6)	77.3 (24.7 to 159.6)	-33.5 (-51.5 to -5.5)

Province	Cause	Sex	1990				2019				1990-2019 percent change (%)			
			DALYs		Death		DALYs		Death		DALYs		Death	
			Number	Rate	Number	Rate	Number	Rate	Number	Rate	Number	Rate	Number	Rate
	Colon and rectum cancer	Both	427 (334 to 534)	130.9 (103.5 to 162.2)	15 (12 to 19)	5.8 (4.6 to 7.1)	1205 (1003 to 1477)	140.8 (116.1 to 173.3)	50 (41 to 62)	6.5 (5.3 to 8)	182.5 (103.1 to 313.3)	7.5 (-22.7 to 56.7)	230 (140.4 to 379.2)	12.8 (-16.8 to 61.7)
		Female	183 (137 to 240)	121.1 (91.3 to 158.2)	7 (5 to 9)	5.4 (4.1 to 6.9)	511 (378 to 683)	117.6 (86.4 to 159.8)	22 (16 to 29)	5.5 (4 to 7.5)	178.5 (72.4 to 343.8)	-2.9 (-38.8 to 54.1)	229.2 (106 to 422.8)	2.2 (-34.5 to 62.5)
		Male	243 (176 to 334)	139.1 (102.2 to 187.3)	9 (6 to 12)	6.1 (4.5 to 8.1)	695 (555 to 873)	165.2 (132 to 207.7)	29 (23 to 36)	7.6 (6.1 to 9.5)	185.6 (85.5 to 341.9)	18.7 (-22.4 to 81.3)	230.6 (115.7 to 412.1)	24.2 (-16.8 to 87.5)
	Liver cancer	Both	516 (396 to 657)	149.4 (113.2 to 189.1)	18 (14 to 23)	6.6 (5 to 8.4)	620 (507 to 772)	73 (59.8 to 90.2)	26 (21 to 31)	3.3 (2.8 to 4.1)	20.1 (-13.4 to 74.7)	-51.1 (-64.5 to -29.1)	41.6 (3.4 to 108.6)	-50 (-63.3 to -27)
		Female	194 (137 to 276)	119.5 (79.3 to 178.7)	7 (4 to 10)	5.4 (3.4 to 8.4)	178 (133 to 241)	40.4 (30.2 to 54.6)	7 (5 to 9)	1.7 (1.3 to 2.3)	-8.2 (-45.4 to 48.4)	-66.2 (-80.7 to -44.9)	3.9 (-41 to 73.3)	-67.9 (-82 to -44.6)
		Male	322 (226 to 446)	175.4 (124 to 238)	11 (8 to 16)	7.8 (5.6 to 10.5)	441 (348 to 567)	107.4 (85 to 136.8)	19 (15 to 23)	5 (4 to 6.3)	37.2 (-10.8 to 108.4)	-38.7 (-59.8 to -7.1)	63.9 (7.5 to 148.8)	-35.5 (-57 to -2.4)
	Gallbladder and biliary tract cancer	Both	74 (57 to 98)	24.2 (19 to 32.1)	3 (2 to 4)	1.1 (0.9 to 1.5)	155 (122 to 200)	18.5 (14.7 to 24)	7 (5 to 9)	0.9 (0.7 to 1.2)	109.2 (48.9 to 211.1)	-23.4 (-44.4 to 9.4)	134.9 (71.7 to 233.7)	-21.3 (-40.1 to 7.9)
		Female	36 (25 to 52)	25.8 (18.1 to 37.8)	1 (1 to 2)	1.2 (0.8 to 1.9)	68 (49 to 96)	15.9 (11.6 to 22.7)	3 (2 to 4)	0.8 (0.6 to 1.1)	88 (24.6 to 201.2)	-38.3 (-58 to -2.9)	108.6 (43 to 228.8)	-36.9 (-57 to -2.5)
		Male	38 (26 to 56)	22.7 (15.5 to 32.9)	1 (1 to 2)	1 (0.7 to 1.5)	87 (65 to 119)	21.2 (15.8 to 29.2)	4 (3 to 5)	1 (0.8 to 1.4)	129.2 (50.7 to 289.5)	-6.5 (-38.1 to 55.6)	161.3 (74.8 to 334.4)	-2.3 (-34 to 59.5)
	Pancreatic cancer	Both	150 (114 to 193)	46.9 (36.2 to 59.9)	6 (4 to 7)	2.1 (1.6 to 2.6)	620 (517 to 744)	73.8 (61.5 to 89.3)	26 (22 to 32)	3.4 (2.9 to 4.1)	313.3 (199.9 to 475.6)	57.4 (15.1 to 115.7)	365 (240.4 to 538.7)	65.1 (23.1 to 125.4)
		Female	55 (40 to 73)	37.5 (27.5 to 49.5)	2 (2 to 3)	1.7 (1.2 to 2.2)	256 (201 to 330)	60.2 (47.2 to 77.8)	11 (9 to 14)	2.8 (2.2 to 3.6)	366.5 (217.1 to 595)	60.5 (9.8 to 133.4)	427.1 (261.2 to 668.5)	68 (17.8 to 142.4)
		Male	95 (65 to 134)	55.3 (38.4 to 76.8)	4 (2 to 5)	2.5 (1.7 to 3.4)	364 (284 to 460)	88 (68.5 to 110.7)	15 (12 to 19)	4.1 (3.2 to 5.1)	282.7 (134.9 to 527.6)	59 (-1.2 to 157.1)	328.9 (170.5 to 593.4)	65.5 (6.7 to 163.1)
	Larynx cancer	Both	192 (148 to 244)	57.8 (44.5 to 72.4)	7 (5 to 9)	2.4 (1.9 to 3)	299 (242 to 375)	34.7 (28.2 to 43.8)	12 (10 to 15)	1.5 (1.2 to 1.9)	55.8 (10.7 to 141.3)	-39.9 (-56.7 to -10.3)	74.3 (24.1 to 162.3)	-36.9 (-53.8 to -7.1)
		Female	51 (38 to 69)	32.1 (24.1 to 42.4)	2 (1 to 2)	1.3 (1 to 1.6)	63 (49 to 82)	14 (11 to 18.3)	2 (2 to 3)	0.6 (0.5 to 0.8)	22.8 (-17.6 to 85.9)	-56.3 (-70.6 to -35.7)	40.2 (-4.8 to 108.1)	-54 (-68.6 to -32.1)
		Male	141 (99 to 193)	80.6 (57.2 to 107.3)	5 (4 to 7)	3.4 (2.5 to 4.6)	236 (184 to 308)	56.2 (44.4 to 72.9)	9 (7 to 12)	2.5 (2 to 3.2)	67.8 (12 to 180.4)	-30.2 (-52.9 to 16.3)	85.7 (24.9 to 210.9)	-27.8 (-49.5 to 17.9)
	Tracheal, bronchus, and lung cancer	Both	842 (634 to 1099)	253.5 (194 to 326.7)	31 (23 to 40)	10.9 (8.5 to 14)	2175 (1736 to 2695)	254.3 (202.6 to 315.1)	90 (72 to 111)	11.6 (9.2 to 14.2)	158.2 (71.5 to 287.9)	0.3 (-32.5 to 48.4)	195 (96.4 to 339)	6.1 (-28.1 to 53.5)
		Female	190 (141 to 253)	117.6 (86.9 to 155)	6 (5 to 8)	4.9 (3.6 to 6.3)	662 (508 to 862)	148.7 (112.8 to 194.9)	26 (20 to 34)	6.5 (4.9 to 8.5)	248.4 (123.1 to 435)	26.4 (-18.5 to 97.8)	310.1 (160.2 to 538.3)	32.8 (-13.7 to 104.5)
		Male	652 (470 to 910)	374 (273.3 to 509.3)	24 (17 to 33)	16.6 (12.2 to 22.3)	1513 (1099 to 1945)	365.1 (267.6 to 464.1)	64 (47 to 81)	17 (12.6 to 21.5)	132 (36.5 to 276)	-2.4 (-41.1 to 54.1)	164.6 (58.9 to 313.7)	2.2 (-36.8 to 56.5)
	Malignant skin melanoma	Both	90 (36 to 144)	25.4 (9.9 to 39.9)	3 (1 to 5)	1.1 (0.4 to 1.8)	149 (63 to 216)	16.8 (6.7 to 24.8)	6 (2 to 9)	0.8 (0.3 to 1.2)	64.4 (3.3 to 168.6)	-34 (-56.5 to 4.4)	100.7 (33.9 to 196.9)	-33.5 (-52.7 to -2.5)
		Female	40 (13 to 70)	23 (7.5 to 37.9)	1 (0 to 2)	1 (0.3 to 1.7)	61 (21 to 98)	13 (4.5 to 21)	2 (1 to 4)	0.5 (0.2 to 0.9)	51.1 (-10.7 to 169)	-43.2 (-63 to -6.4)	72.6 (15.6 to 172.6)	-46.9 (-61.5 to -20.4)

Province	Cause	Sex	1990				2019				1990-2019 percent change (%)			
			DALYs		Death		DALYs		Death		DALYs		Death	
			Number	Rate	Number	Rate	Number	Rate	Number	Rate	Number	Rate	Number	Rate
		Male	50 (19 to 80)	27.7 (9.9 to 45.3)	2 (1 to 3)	1.3 (0.4 to 2.2)	88 (37 to 131)	20.8 (7.9 to 31.6)	4 (1 to 6)	1 (0.3 to 1.6)	75 (4.5 to 194.2)	-24.8 (-54.8 to 27)	121.9 (33.3 to 254.3)	-22.1 (-52 to 28.4)
	Non-melanoma skin cancer	Both	18 (14 to 23)	5.9 (4.6 to 7.6)	1 (1 to 1)	0.3 (0.2 to 0.4)	47 (38 to 56)	5.7 (4.7 to 6.8)	2 (2 to 3)	0.3 (0.3 to 0.4)	163.5 (93.4 to 265.9)	-3.4 (-28.3 to 32.5)	227.3 (140 to 355.2)	5.2 (-22 to 45.2)
		Female	3 (2 to 4)	1.8 (1.2 to 2.8)	0 (0 to 0)	0.1 (0.1 to 0.2)	6 (5 to 8)	1.4 (1.1 to 1.9)	0 (0 to 0)	0.1 (0.1 to 0.1)	135.5 (50 to 285.7)	-22.5 (-51.6 to 34.4)	180.2 (70.7 to 408.3)	-20.5 (-53.6 to 53.4)
		Male	15 (11 to 20)	9.8 (7.4 to 12.9)	1 (0 to 1)	0.5 (0.4 to 0.7)	41 (33 to 50)	10.3 (8.2 to 12.5)	2 (2 to 2)	0.6 (0.5 to 0.7)	168.3 (88 to 292.7)	4.5 (-26.2 to 49.7)	235.5 (135.5 to 373.2)	10.9 (-19.6 to 54.9)
	Breast cancer	Both	470 (316 to 666)	133.3 (88 to 191.3)	14 (9 to 21)	4.5 (2.8 to 6.8)	1156 (874 to 1483)	118.7 (89.7 to 151.2)	34 (26 to 44)	3.9 (3 to 5)	146.1 (67.7 to 264.3)	-11 (-39.5 to 31.4)	145.1 (62.5 to 273.8)	-14.5 (-44.2 to 34.2)
		Female	464 (309 to 661)	276.9 (181.1 to 399.7)	14 (9 to 20)	9.3 (5.8 to 14.1)	1146 (864 to 1471)	235.7 (177.7 to 300.9)	34 (25 to 43)	7.5 (5.7 to 9.7)	146.9 (67.5 to 266.9)	-14.9 (-42.7 to 27)	145.8 (62.3 to 276.8)	-18.6 (-47.4 to 28.6)
		Male	6 (4 to 9)	3.1 (2.1 to 4.8)	0 (0 to 0)	0.1 (0.1 to 0.2)	10 (7 to 14)	2.4 (1.6 to 3.2)	0 (0 to 1)	0.1 (0.1 to 0.1)	78.9 (-0.8 to 205.3)	-23.2 (-57.5 to 29.3)	96.1 (6.2 to 246.7)	-23.1 (-56.3 to 33.4)
	Cervical cancer	Female	136 (87 to 189)	79.3 (52.3 to 108.3)	4 (3 to 6)	2.8 (1.9 to 4)	189 (134 to 258)	39.5 (28 to 54.3)	6 (4 to 9)	1.4 (1 to 2)	38.5 (-6.4 to 115)	-50.1 (-65.4 to -24.8)	51.2 (4.5 to 129.2)	-49.5 (-65.4 to -23.9)
	Uterine cancer	Female	18 (13 to 32)	11.7 (8.2 to 21.3)	1 (0 to 1)	0.5 (0.3 to 0.9)	35 (25 to 57)	7.8 (5.6 to 13)	1 (1 to 2)	0.3 (0.2 to 0.5)	94.4 (28 to 196.6)	-33.3 (-55.7 to -0.1)	102.2 (32.5 to 212)	-35.7 (-58.7 to -2.2)
	Ovarian cancer	Female	62 (36 to 116)	35.5 (20.9 to 65.5)	2 (1 to 3)	1.2 (0.7 to 2.2)	258 (158 to 348)	55.4 (33.1 to 75.2)	8 (5 to 11)	1.9 (1.2 to 2.7)	312.8 (46 to 673.3)	55.9 (-44 to 189.9)	356.5 (64.4 to 755.4)	61.4 (-41.2 to 198)
	Prostate cancer	Male	213 (143 to 306)	166.8 (115.8 to 239)	10 (7 to 15)	10.3 (7.2 to 14.8)	700 (521 to 948)	197.4 (146.4 to 272)	40 (30 to 56)	12.1 (8.9 to 16.9)	228.2 (136.4 to 359.3)	18.3 (-11.4 to 58.7)	301.2 (189.2 to 451.9)	17.2 (-11.8 to 57.7)
	Testicular cancer	Male	3 (2 to 4)	0.7 (0.5 to 0.8)	0 (0 to 0)	0 (0 to 0)	7 (5 to 11)	1.5 (1 to 2.2)	0 (0 to 0)	0 (0 to 0)	158.6 (67.6 to 289.9)	119.7 (47.6 to 224.9)	249.1 (137.7 to 425.2)	163.3 (80.5 to 307.9)
	Kidney cancer	Both	138 (106 to 181)	26.9 (20.2 to 34.3)	3 (2 to 4)	1 (0.7 to 1.3)	272 (220 to 325)	30.6 (24.9 to 36.6)	9 (8 to 11)	1.2 (1 to 1.4)	96.8 (36.9 to 177.4)	14.1 (-19.7 to 67.6)	189 (102.1 to 333.7)	21.4 (-15.3 to 100.2)
		Female	60 (44 to 85)	23.1 (16.6 to 29.9)	1 (1 to 2)	0.8 (0.5 to 1.1)	98 (78 to 122)	21.5 (17 to 26.7)	3 (2 to 4)	0.7 (0.6 to 0.9)	62.2 (1.4 to 145.7)	-6.9 (-37.7 to 37.4)	126.5 (48.9 to 253.9)	-12.4 (-42.8 to 66.1)
		Male	78 (52 to 111)	30.2 (20.9 to 42.8)	2 (1 to 3)	1.1 (0.8 to 1.5)	174 (133 to 221)	40.2 (31.2 to 50.9)	6 (5 to 8)	1.7 (1.3 to 2.1)	123.7 (37 to 269.9)	33.5 (-15.6 to 121)	231.7 (109.8 to 457.3)	51 (-3 to 152.1)
	Bladder cancer	Both	101 (77 to 131)	34.9 (26.5 to 44.7)	4 (3 to 5)	1.8 (1.3 to 2.2)	252 (203 to 311)	31.7 (25.4 to 38.9)	12 (10 to 15)	1.6 (1.3 to 2)	149.9 (75.6 to 258.8)	-9.2 (-34.7 to 29)	189.3 (107.8 to 317.7)	-7.5 (-32 to 30.1)
		Female	21 (15 to 30)	16.4 (11.4 to 24.4)	1 (1 to 1)	0.9 (0.6 to 1.4)	47 (36 to 62)	11.5 (8.7 to 15.1)	2 (2 to 3)	0.6 (0.5 to 0.8)	120.3 (39.9 to 254.2)	-29.9 (-57.4 to 15.1)	157.5 (52.9 to 327.8)	-28.4 (-58.5 to 20)
		Male	80 (58 to 108)	52 (38.3 to 68.3)	3 (2 to 4)	2.7 (2 to 3.5)	206 (158 to 266)	53.1 (40.8 to 68.7)	10 (7 to 12)	2.7 (2.1 to 3.5)	157.8 (62.1 to 288)	2.2 (-34.2 to 52.4)	198.2 (89.4 to 346.3)	1.5 (-31.2 to 49.7)
	Brain and central nervous system cancer	Both	1046 (599 to 1496)	168 (94.6 to 234)	21 (11 to 28)	4.6 (2.6 to 6.3)	1249 (666 to 1627)	130.8 (69.5 to 169.7)	34 (18 to 45)	3.9 (2 to 5.2)	19.5 (-35.3 to 82.8)	-22.2 (-54.6 to 12.2)	67.2 (-2.9 to 137.4)	-15.1 (-47 to 18.2)
		Female	414 (157 to 776)	131.8 (54.9 to 229.7)	8 (3 to 13)	3.5 (1.6 to 5.7)	534 (283 to 769)	112.1 (59.4 to 162.8)	14 (8 to 21)	3.2 (1.7 to 4.7)	29 (-52.2 to 183.8)	-14.9 (-66.1 to 75.5)	86.8 (-26 to 285)	-6.5 (-60.7 to 75.1)
		Male	632 (293 to 1004)	200.6 (95.9 to 316)	13 (6 to 20)	5.7 (2.7 to 8.8)	715 (310 to 1047)	149.8 (64.4 to 218.4)	20 (8 to 29)	4.6 (1.9 to 6.7)	13.2 (-42.3 to 96.2)	-25.3 (-58.7 to 23.6)	55.5 (-13.5 to 149.4)	-18.3 (-51.4 to 25.5)
	Thyroid cancer	Both	30 (24 to 40)	8.8 (6.9 to 11.8)	1 (1 to 1)	0.4 (0.3 to 0.5)	89 (63 to 110)	9.9 (7.1 to 12.2)	3 (2 to 4)	0.4 (0.3 to 0.4)	194.5 (92.9 to 318.4)	12.1 (-24.8 to 58)	195.4 (103.3 to 321)	-0.2 (-31.9 to 42.1)

Province	Cause	Sex	1990				2019				1990-2019 percent change (%)			
			DALYs		Death		DALYs		Death		DALYs		Death	
			Number	Rate	Number	Rate	Number	Rate	Number	Rate	Number	Rate	Number	Rate
		Female	17 (13 to 25)	10.9 (7.9 to 16.4)	1 (0 to 1)	0.5 (0.3 to 0.7)	44 (29 to 61)	9.7 (6.6 to 13.1)	1 (1 to 2)	0.3 (0.2 to 0.5)	158.8 (53.7 to 304.4)	-11.1 (-44.3 to 34.9)	144.9 (53.1 to 273.4)	-25.1 (-53.7 to 15.6)
		Male	13 (9 to 18)	6.9 (4.7 to 9.7)	0 (0 to 1)	0.3 (0.2 to 0.4)	45 (30 to 58)	10.1 (6.9 to 13.1)	2 (1 to 2)	0.4 (0.3 to 0.5)	240.7 (94.4 to 440.2)	46.5 (-14.9 to 137.4)	263.9 (112.4 to 503.6)	42.3 (-16.8 to 128.4)
	Mesothelioma	Both	9 (2 to 18)	2.3 (0.5 to 4.9)	0 (0 to 1)	0.1 (0 to 0.2)	38 (12 to 54)	4.1 (1.3 to 5.8)	1 (0 to 2)	0.2 (0.1 to 0.2)	326.4 (77.7 to 846.7)	74.4 (-30.9 to 255)	417.9 (97.7 to 934.3)	93.9 (-29 to 269.8)
		Female	4 (1 to 8)	2.3 (0.7 to 4)	0 (0 to 0)	0.1 (0 to 0.1)	6 (2 to 10)	1.3 (0.5 to 2)	0 (0 to 0)	0 (0 to 0.1)	45.4 (-35.7 to 227.8)	-44.9 (-72.7 to 10.5)	62.6 (-16.2 to 217.7)	-44.5 (-69.8 to -1.8)
		Male	5 (0 to 13)	2.3 (0.2 to 7)	0 (0 to 0)	0.1 (0 to 0.3)	31 (9 to 46)	6.9 (2.1 to 10.1)	1 (0 to 2)	0.3 (0.1 to 0.4)	594 (84.4 to 2983.2)	196 (-25.2 to 1207.8)	729.5 (105.3 to 3474.3)	234.2 (-19.9 to 1341.5)
	Hodgkin lymphoma	Both	55 (26 to 131)	10.7 (5.2 to 24.6)	1 (1 to 3)	0.3 (0.2 to 0.7)	64 (39 to 125)	6.5 (4 to 12.5)	2 (1 to 3)	0.2 (0.1 to 0.3)	16.7 (-15 to 90.5)	-39.5 (-54.8 to -4.7)	27.4 (-5.1 to 100.9)	-45 (-58.5 to -15.6)
		Female	19 (7 to 47)	7.9 (3.3 to 19.6)	0 (0 to 1)	0.3 (0.1 to 0.6)	25 (14 to 51)	5 (2.8 to 10)	1 (0 to 1)	0.1 (0.1 to 0.3)	32.4 (-13.8 to 140.3)	-37.3 (-58.8 to 12)	31.4 (-13.6 to 128.6)	-51.4 (-67 to -16)
		Male	36 (17 to 94)	13.3 (6.3 to 32.8)	1 (0 to 2)	0.4 (0.2 to 0.9)	40 (23 to 84)	8.1 (4.7 to 17)	1 (1 to 2)	0.2 (0.1 to 0.5)	8.6 (-25.2 to 81.8)	-39.5 (-57 to -0.3)	25.3 (-11.6 to 104.6)	-38.9 (-56 to -3.1)
	Non-Hodgkin lymphoma	Both	195 (147 to 247)	42.8 (33.5 to 53)	5 (4 to 6)	1.5 (1.2 to 1.8)	344 (286 to 415)	37.1 (31.2 to 44.4)	11 (9 to 13)	1.3 (1.1 to 1.5)	76.7 (26 to 156.1)	-13.4 (-36.6 to 22.2)	112.8 (54.9 to 196.6)	-12.2 (-33.7 to 19.9)
		Female	63 (45 to 88)	30.7 (22.3 to 40.4)	2 (1 to 2)	1.1 (0.8 to 1.5)	119 (91 to 156)	25.7 (19.8 to 33.2)	4 (3 to 5)	0.9 (0.7 to 1.2)	88.6 (17.4 to 201)	-16.3 (-44.5 to 33.6)	119.3 (47.1 to 257.9)	-20.3 (-46.2 to 33.5)
		Male	131 (94 to 176)	54 (39.1 to 71.8)	3 (2 to 4)	1.8 (1.3 to 2.3)	224 (180 to 277)	48.6 (39 to 59.7)	7 (6 to 9)	1.7 (1.4 to 2.1)	70.9 (14.4 to 161.1)	-9.9 (-38.2 to 33.8)	109.4 (44.6 to 214.4)	-5.2 (-34.1 to 37.4)
	Multiple myeloma	Both	62 (45 to 99)	19.2 (13.8 to 30.7)	2 (2 to 4)	0.8 (0.6 to 1.3)	133 (91 to 208)	15.5 (10.5 to 24.6)	5 (3 to 8)	0.6 (0.4 to 1.1)	112.8 (36.5 to 196.6)	-19.3 (-46.6 to 10)	122.5 (48.1 to 201.4)	-20 (-45.2 to 6.7)
		Female	26 (17 to 40)	18.3 (11.9 to 28.4)	1 (1 to 2)	0.8 (0.5 to 1.3)	47 (31 to 75)	10.9 (7.2 to 17.2)	2 (1 to 3)	0.4 (0.3 to 0.7)	79.5 (18 to 171.4)	-40.4 (-59.5 to -11.2)	78.3 (21.3 to 168.3)	-44.1 (-61 to -17)
		Male	36 (23 to 63)	20.1 (12.8 to 35.7)	1 (1 to 2)	0.8 (0.5 to 1.5)	86 (54 to 142)	20.3 (12.8 to 34.6)	3 (2 to 6)	0.9 (0.5 to 1.5)	137 (35.5 to 272.1)	1 (-41.5 to 57)	157.3 (46.2 to 302.3)	4.6 (-39.6 to 60.9)
	Leukemia	Both	2102 (1254 to 2972)	299.5 (185 to 396.2)	37 (23 to 50)	8 (5.2 to 10.1)	1730 (1001 to 2280)	185.2 (109.2 to 243.3)	48 (30 to 62)	5.7 (3.6 to 7.3)	-17.7 (-45.5 to 27.9)	-38.2 (-56.5 to -10)	29.4 (-8.1 to 85.7)	-28 (-46.5 to 0.1)
		Female	982 (461 to 1471)	267.6 (148 to 366.2)	16 (9 to 22)	6.5 (3.9 to 8.5)	665 (365 to 895)	139.6 (78.1 to 186.4)	17 (10 to 22)	3.8 (2.4 to 5)	-32.2 (-60.9 to 26.4)	-47.9 (-67.4 to -17.4)	4.3 (-33.9 to 62.8)	-41.2 (-59.3 to -16.3)
		Male	1120 (499 to 1669)	328.3 (174.4 to 465.7)	21 (11 to 30)	9.4 (5.7 to 13.1)	1065 (532 to 1456)	232.3 (116.8 to 313.6)	32 (16 to 42)	7.8 (4.1 to 10.5)	-4.9 (-39.2 to 67.5)	-29.3 (-52.8 to 13.8)	48.3 (-0.1 to 143.6)	-17.4 (-42.1 to 27)
	Other malignant neoplasms	Both	770 (614 to 932)	139.7 (111.5 to 170.7)	17 (13 to 21)	4.6 (3.5 to 5.7)	1295 (930 to 1614)	143.9 (101 to 179.6)	40 (28 to 51)	5 (3.3 to 6.4)	68.3 (10.5 to 142.1)	3 (-28.6 to 50.6)	137.4 (59 to 250.6)	7.1 (-25.1 to 64.5)
		Female	339 (260 to 440)	121.6 (93.4 to 157.5)	7 (5 to 9)	3.9 (2.9 to 5.2)	546 (432 to 672)	120.2 (94.2 to 148.3)	16 (13 to 20)	3.9 (3 to 5)	61.4 (13.8 to 133)	-1.1 (-29.5 to 42.1)	130.5 (60.9 to 232.5)	0.8 (-30.4 to 50.6)
		Male	431 (309 to 566)	156.5 (113.1 to 207.1)	10 (7 to 13)	5.4 (3.8 to 7.2)	749 (464 to 1013)	168.6 (102.7 to 227.8)	24 (14 to 33)	6.1 (3.5 to 8.3)	73.7 (-5.4 to 186.2)	7.7 (-36 to 78.6)	142.2 (37.9 to 302.6)	12.8 (-33.5 to 91.7)
	Other neoplasms	Both	28 (18 to 35)	6.2 (3.9 to 7.9)	1 (0 to 1)	0.2 (0.1 to 0.3)	46 (37 to 57)	5.5 (4.3 to 6.7)	2 (1 to 2)	0.2 (0.2 to 0.3)	68.8 (24.9 to 150.8)	-12.6 (-34.3 to 31.3)	133.4 (63.4 to 289.6)	-13.7 (-40.2 to 49.6)
		Female	13 (9 to 18)	6 (3.7 to 7.9)	0 (0 to 0)	0.2 (0.1 to 0.3)	20 (16 to 25)	4.5 (3.5 to 5.7)	1 (0 to 1)	0.1 (0.1 to 0.2)	49.9 (-6 to 142.1)	-25.4 (-50.3 to 36.2)	97.6 (18.9 to 335.6)	-33.3 (-61 to 69.3)

Province	Cause	Sex	1990				2019				1990-2019 percent change (%)			
			DALYs		Death		DALYs		Death		DALYs		Death	
			Number	Rate	Number	Rate	Number	Rate	Number	Rate	Number	Rate	Number	Rate
		Male	14 (8 to 20)	6.4 (3.9 to 8.7)	0 (0 to 1)	0.2 (0.1 to 0.3)	27 (18 to 35)	6.5 (4.5 to 8.5)	1 (1 to 1)	0.3 (0.2 to 0.4)	86.2 (21.5 to 191.9)	1.7 (-29.1 to 45.8)	161.7 (67.1 to 315.7)	6.1 (-30.8 to 58.2)
	Cardiovascular diseases	Both	28497 (24300 to 33343)	9308.3 (8031.2 to 10803.7)	1079 (922 to 1257)	483.7 (416.8 to 551.1)	43039 (37863 to 48440)	5417.4 (4778.4 to 6117.7)	2204 (1913 to 2500)	308.6 (265.7 to 350.5)	51 (22.3 to 87)	-41.8 (-52 to -29)	104.2 (67.3 to 149.3)	-36.2 (-46.3 to -23.8)
		Female	11081 (8945 to 13593)	7765.3 (6377.3 to 9379.3)	433 (353 to 526)	417.5 (344.5 to 499)	15930 (13416 to 19330)	3987.1 (3354.9 to 4840.6)	868 (711 to 1063)	238.1 (194.2 to 291.7)	43.8 (7.6 to 90.1)	-48.7 (-60.7 to -32.6)	100.2 (50.9 to 167.7)	-43 (-55.8 to -25.2)
		Male	17416 (13941 to 21873)	10682.3 (8643.9 to 13203.2)	646 (516 to 807)	547.3 (451.8 to 662.6)	27109 (23017 to 31477)	6910.6 (5916.5 to 7954.4)	1336 (1142 to 1528)	383.9 (328 to 435.4)	55.7 (18.8 to 103.8)	-35.3 (-50 to -16.6)	106.9 (58.9 to 168.9)	-29.9 (-44.6 to -12.4)
	Rheumatic heart disease	Both	1929 (1491 to 2529)	359.3 (271.1 to 505.4)	40 (30 to 58)	11.4 (7.9 to 18.1)	969 (784 to 1178)	102.1 (83.5 to 122.9)	25 (20 to 31)	3.1 (2.5 to 3.8)	-49.7 (-63.2 to -30.8)	-71.6 (-80.7 to -59.4)	-37.7 (-60.6 to -6)	-72.5 (-84.5 to -55.7)
		Female	936 (656 to 1294)	352.6 (242.2 to 545.2)	19 (13 to 30)	11 (6.9 to 20.2)	438 (342 to 576)	91.3 (72.3 to 119.1)	11 (8 to 14)	2.6 (2 to 3.5)	-53.2 (-69.2 to -23.1)	-74.1 (-84.2 to -56.5)	-43.7 (-68.4 to 2.9)	-76.5 (-88.4 to -55.6)
		Male	993 (688 to 1389)	363.9 (245.8 to 534.1)	21 (14 to 32)	11.8 (7.7 to 18.8)	532 (409 to 699)	113.5 (88 to 147.7)	14 (11 to 19)	3.7 (2.8 to 4.9)	-46.5 (-64.8 to -10.4)	-68.8 (-80.7 to -45.8)	-32.5 (-60.4 to 23.4)	-68.3 (-82.7 to -41.3)
	Ischemic heart disease	Both	16512 (13884 to 19655)	5812.1 (4919.1 to 6754.5)	696 (589 to 810)	315.8 (268.8 to 360.7)	26098 (22663 to 29835)	3306.4 (2884 to 3785.9)	1426 (1233 to 1628)	199.7 (172.3 to 227.8)	58.1 (26.1 to 99.5)	-43.1 (-53.8 to -29.4)	104.9 (65.8 to 153.5)	-36.8 (-47.1 to -23.2)
		Female	5680 (4460 to 7167)	4463.7 (3526.1 to 5501.3)	260 (204 to 321)	258.4 (201 to 314.7)	8804 (7248 to 10910)	2242.3 (1842.2 to 2781.3)	542 (442 to 669)	149.3 (121.6 to 184.3)	55 (13.2 to 115.2)	-49.8 (-62.8 to -31.1)	108.4 (54.4 to 183.5)	-42.2 (-56.4 to -22.5)
		Male	10832 (8460 to 13790)	7025.2 (5657.6 to 8704.7)	436 (347 to 544)	371.8 (305.9 to 450.5)	17295 (14485 to 20196)	4414.6 (3735.9 to 5120.2)	884 (755 to 1014)	253.5 (217.7 to 288.7)	59.7 (18.8 to 113.2)	-37.2 (-52.2 to -18.3)	102.7 (54.2 to 163.1)	-31.8 (-46.3 to -13.9)
	Stroke	Both	6064 (5078 to 7349)	2015.3 (1668.7 to 2412.1)	218 (175 to 268)	101.6 (80.4 to 123.6)	9022 (7839 to 10292)	1138.9 (978.2 to 1303.9)	427 (358 to 500)	60.2 (50.1 to 70.2)	48.8 (17.1 to 87.2)	-43.5 (-54.7 to -28.9)	95.8 (52.7 to 155.4)	-40.7 (-53.6 to -23.6)
		Female	2669 (2130 to 3399)	1887.6 (1468.7 to 2375.5)	97 (73 to 124)	94.8 (69 to 121.5)	3890 (3236 to 4646)	960.8 (797.1 to 1153.7)	180 (142 to 226)	49.2 (38.7 to 62)	45.7 (7.8 to 92.3)	-49.1 (-61.8 to -31.5)	85.8 (31.9 to 167.5)	-48.1 (-62.8 to -24.3)
		Male	3395 (2605 to 4342)	2124.1 (1645 to 2748.5)	121 (91 to 161)	107.9 (81.6 to 141.3)	5132 (4326 to 6047)	1326.8 (1113.4 to 1550.1)	247 (199 to 292)	71.9 (57.9 to 84.6)	51.2 (12.4 to 109.1)	-37.5 (-53.8 to -14.1)	103.9 (48.4 to 186.2)	-33.4 (-51.2 to -9.1)
	Hypertensive heart disease	Both	1781 (1323 to 2303)	682.8 (508.9 to 894.2)	79 (58 to 104)	39.3 (28.5 to 53.4)	3769 (2641 to 4537)	491.9 (338.4 to 591.5)	211 (131 to 255)	30 (18.5 to 36.3)	111.7 (48.4 to 197.5)	-28 (-48.4 to 2)	164.9 (81.6 to 281.7)	-23.6 (-48.9 to 8.9)
		Female	765 (522 to 1188)	644.2 (444.2 to 1071.7)	37 (25 to 63)	38.4 (25.9 to 68.3)	1519 (1065 to 1954)	390.7 (273.8 to 505)	91 (59 to 118)	25.1 (16.1 to 32.7)	98.6 (14.2 to 221)	-39.4 (-67.5 to -3.6)	145.8 (23 to 295.6)	-34.6 (-69.6 to 5.6)
		Male	1016 (665 to 1522)	713.7 (479.5 to 1037.7)	43 (27 to 64)	39.9 (25.9 to 57.4)	2251 (1319 to 2830)	598.5 (343.5 to 748.8)	120 (64 to 152)	35.3 (18.1 to 44.6)	121.5 (37.9 to 269.2)	-16.1 (-46 to 35.6)	181.3 (71.1 to 375.9)	-11.5 (-43.3 to 43.5)
	Non-rheumatic valvular heart disease	Both	175 (129 to 225)	50.7 (39 to 62.7)	6 (5 to 7)	2.3 (1.8 to 2.9)	352 (272 to 434)	39.9 (30.6 to 48.9)	14 (11 to 17)	1.8 (1.4 to 2.2)	101.2 (38.5 to 195.2)	-21.4 (-42.7 to 9.6)	142.4 (76.2 to 232.3)	-21.6 (-43.1 to 4.2)
		Female	87 (60 to 119)	55.3 (40.2 to 73)	3 (2 to 4)	2.6 (1.9 to 3.6)	157 (120 to 202)	35.1 (27.2 to 45.5)	6 (5 to 8)	1.6 (1.3 to 2.1)	79.5 (11.3 to 190.4)	-36.6 (-57.7 to -1.1)	109 (39.4 to 228.4)	-38.5 (-59.5 to -5.3)
		Male	88 (56 to 125)	45.9 (30.6 to 64.1)	3 (2 to 4)	2 (1.4 to 2.7)	196 (131 to 258)	44.8 (30.1 to 59)	8 (5 to 10)	2 (1.3 to 2.6)	122.8 (24.1 to 280.3)	-2.2 (-42.8 to 60.9)	179.6 (64.2 to 360)	3.9 (-36.3 to 61.9)
	Cardiomyopathy and myocarditis	Both	430 (263 to 738)	68.4 (48.7 to 106.9)	8 (6 to 13)	2.4 (1.7 to 3.8)	422 (276 to 540)	48.7 (30.3 to 61.4)	16 (9 to 20)	2.1 (1.1 to 2.6)	-2 (-58.1 to 78.2)	-28.8 (-63.3 to 10.5)	88.1 (-13.7 to 205.7)	-13.5 (-54.2 to 33)

Province	Cause	Sex	1990				2019				1990-2019 percent change (%)			
			DALYs		Death		DALYs		Death		DALYs		Death	
			Number	Rate	Number	Rate	Number	Rate	Number	Rate	Number	Rate	Number	Rate
		Female	190 (96 to 380)	60.6 (37.4 to 113.4)	4 (2 to 7)	2.2 (1.2 to 4.7)	138 (79 to 185)	31.8 (17.9 to 42.4)	5 (3 to 7)	1.3 (0.6 to 1.8)	-27.7 (-72.8 to 56.2)	-47.5 (-73 to -4.6)	41.2 (-35.5 to 172.2)	-40 (-70.2 to 17.8)
		Male	240 (137 to 458)	75 (49.6 to 118.5)	5 (3 to 8)	2.5 (1.7 to 3.7)	284 (161 to 392)	66.1 (36.6 to 89.6)	11 (5 to 14)	2.8 (1.4 to 3.8)	18.4 (-52.8 to 126.7)	-12 (-58.2 to 45.7)	124.7 (-2.6 to 284.9)	14 (-43.5 to 84.8)
	Atrial fibrillation and flutter	Both	152 (113 to 200)	70.5 (52.8 to 90.9)	4 (3 to 5)	2.7 (1.7 to 3.5)	486 (371 to 631)	66.7 (51.1 to 85.6)	16 (13 to 19)	2.4 (1.9 to 2.9)	220.8 (182.8 to 273.9)	-5.4 (-18.7 to 13.6)	306.7 (193.2 to 526.8)	-10.8 (-36.6 to 42)
		Female	80 (57 to 107)	79 (56.1 to 104.7)	3 (1 to 4)	3.2 (1.8 to 4.5)	251 (187 to 330)	67.1 (50.1 to 87.7)	8 (6 to 11)	2.3 (1.8 to 3.2)	214.9 (160.5 to 310.7)	-15.1 (-31.6 to 14.4)	218 (109.6 to 552.3)	-28.7 (-53.5 to 49.2)
		Male	72 (51 to 98)	60.7 (44.9 to 81)	1 (1 to 2)	2 (1.1 to 2.7)	235 (176 to 308)	66.3 (49.6 to 86.1)	8 (5 to 10)	2.5 (1.5 to 3.3)	227.4 (176 to 288.3)	9.3 (-8.7 to 27.1)	470.6 (273.4 to 691)	26.8 (-15.6 to 71.3)
	Aortic aneurysm	Both	85 (65 to 112)	27.5 (21.2 to 34.7)	3 (3 to 4)	1.4 (1.1 to 1.7)	251 (197 to 315)	29.4 (23.2 to 36.7)	11 (9 to 14)	1.5 (1.2 to 1.8)	193.8 (94 to 337.1)	6.9 (-27.6 to 55.8)	235.7 (126.6 to 385.5)	6.7 (-24.7 to 52.2)
		Female	22 (16 to 28)	16.4 (12.2 to 21.4)	1 (1 to 1)	0.9 (0.6 to 1.2)	68 (53 to 86)	15.2 (11.9 to 19.3)	3 (2 to 4)	0.7 (0.6 to 0.9)	210.4 (114.7 to 352.4)	-7.3 (-33.9 to 34.3)	202.8 (117.9 to 333.9)	-19.9 (-42.1 to 17.2)
		Male	64 (44 to 89)	37.6 (26.4 to 51.4)	2 (2 to 3)	1.8 (1.3 to 2.4)	183 (135 to 244)	44.2 (32.6 to 58.1)	8 (6 to 11)	2.2 (1.7 to 2.9)	188.1 (69.4 to 387.4)	17.5 (-29.4 to 94.6)	248.9 (109.6 to 485.6)	23 (-24.2 to 99.3)
	Peripheral artery disease	Both	15 (8 to 24)	6.5 (3.6 to 10.7)	0 (0 to 0)	0.1 (0.1 to 0.1)	46 (27 to 77)	6.4 (3.8 to 10.7)	1 (1 to 1)	0.1 (0.1 to 0.1)	212.9 (178 to 261.7)	-1 (-10.3 to 10.4)	385.2 (230.5 to 636.6)	18.7 (-20 to 86.7)
		Female	8 (4 to 14)	7.6 (4.1 to 12.9)	0 (0 to 0)	0.1 (0 to 0.2)	25 (14 to 44)	6.9 (3.7 to 11.9)	0 (0 to 0)	0.1 (0 to 0.1)	215.9 (176.7 to 267.3)	-9.3 (-20.1 to 3.5)	280.3 (127.6 to 758.9)	-13.2 (-48 to 103)
		Male	7 (4 to 11)	5.4 (3.1 to 8.5)	0 (0 to 0)	0.1 (0.1 to 0.1)	20 (13 to 33)	5.9 (3.8 to 9.5)	0 (0 to 1)	0.1 (0.1 to 0.2)	209.2 (157.7 to 276.9)	10.1 (-4.2 to 30)	478.4 (272.3 to 795.1)	56.1 (1.4 to 136.3)
	Endocarditis	Both	208 (82 to 412)	29.4 (16.8 to 46.4)	4 (2 to 6)	0.9 (0.6 to 1.3)	172 (88 to 236)	19.1 (9.5 to 26.3)	6 (3 to 9)	0.8 (0.4 to 1.1)	-17.5 (-54.5 to 76.1)	-35 (-56.9 to -5.1)	73.7 (16.9 to 150.6)	-10.9 (-42.4 to 26.3)
		Female	125 (31 to 284)	33.4 (12.3 to 62.9)	2 (1 to 4)	1 (0.4 to 1.6)	79 (31 to 117)	17.6 (6.9 to 26.1)	3 (1 to 5)	0.8 (0.3 to 1.2)	-37 (-70.1 to 87.4)	-47.2 (-68.6 to -14.7)	49.3 (-13.2 to 143.8)	-22.2 (-52.8 to 21.3)
		Male	84 (42 to 154)	25.3 (16.8 to 38)	2 (1 to 2)	0.8 (0.6 to 1.1)	93 (52 to 131)	20.6 (11.1 to 29.1)	3 (2 to 5)	0.8 (0.4 to 1.2)	11.6 (-40.7 to 113.9)	-18.7 (-52.7 to 38.5)	105.5 (22 to 242)	6.5 (-41 to 72.6)
	Other cardiovascular and circulatory diseases	Both	1145 (856 to 1543)	186 (147.4 to 250.1)	20 (15 to 28)	5.8 (4.5 to 8.5)	1450 (1229 to 1699)	168 (143.3 to 194.4)	51 (43 to 59)	6.9 (5.8 to 8.1)	26.7 (-10.3 to 73.7)	-9.7 (-33.9 to 16.3)	157.9 (74.3 to 256.5)	19.3 (-25.5 to 62.2)
		Female	518 (343 to 816)	165 (123.9 to 232.1)	8 (6 to 13)	4.9 (3.5 to 8.3)	562 (452 to 695)	128.3 (104 to 157.9)	19 (15 to 24)	5 (3.9 to 6.3)	8.5 (-34.7 to 74.3)	-22.3 (-48.2 to 8.2)	124.4 (32.3 to 243.9)	3.4 (-47.3 to 53.5)
		Male	627 (445 to 988)	205.7 (155 to 316.7)	11 (8 to 19)	6.7 (4.9 to 10.7)	888 (737 to 1068)	209.3 (174 to 248.5)	32 (26 to 38)	8.9 (7.3 to 10.7)	41.7 (-8.7 to 104.3)	1.8 (-37.8 to 42.3)	183.1 (56.1 to 317.3)	31.9 (-27.9 to 95.1)
	Chronic respiratory diseases	Both	3558 (2830 to 4852)	888.1 (726.7 to 1266.8)	71 (55 to 120)	32 (24.8 to 54.8)	5147 (4297 to 6507)	592.3 (496.6 to 757)	120 (97 to 191)	16.6 (13.3 to 26.9)	44.7 (24.2 to 66.6)	-33.3 (-44.5 to -21.8)	68.9 (27.9 to 118.5)	-48.1 (-61.5 to -32.8)
		Female	1539 (1179 to 2335)	775.6 (593.4 to 1364.2)	28 (19 to 61)	26.1 (17.3 to 59.8)	2204 (1775 to 3058)	495.4 (398.8 to 703)	41 (29 to 83)	11 (7.7 to 22.1)	43.2 (19.4 to 76.4)	-36.1 (-50.7 to -19.1)	48.4 (-3.8 to 126.9)	-57.7 (-73.5 to -34.9)
		Male	2019 (1605 to 2686)	993.6 (798.8 to 1331.5)	43 (32 to 67)	38.3 (29 to 57)	2944 (2430 to 3755)	695.2 (579.8 to 900.2)	78 (63 to 126)	22.7 (18.2 to 37.1)	45.8 (19.9 to 75.2)	-30 (-44.7 to -14.3)	82.1 (29.4 to 151.7)	-40.8 (-57.6 to -19.2)

Province	Cause	Sex	1990				2019				1990-2019 percent change (%)			
			DALYs		Death		DALYs		Death		DALYs		Death	
			Number	Rate	Number	Rate	Number	Rate	Number	Rate	Number	Rate	Number	Rate
	Chronic obstructive pulmonary disease	Both	1327 (1065 to 2133)	420.5 (333.6 to 713.4)	35 (25 to 70)	17 (12.3 to 34.9)	2861 (2429 to 3830)	342.3 (290.4 to 463.4)	80 (62 to 133)	11.2 (8.7 to 18.9)	115.5 (72.6 to 159.5)	-18.6 (-37.8 to -1)	130.2 (70.9 to 213)	-34.4 (-53.3 to -11.1)
		Female	597 (437 to 1178)	383 (275.5 to 826.1)	14 (9 to 41)	14.5 (8.7 to 42.6)	1216 (968 to 1905)	283.8 (226.4 to 454.6)	28 (18 to 67)	7.7 (5 to 18.1)	103.7 (50.9 to 176.4)	-25.9 (-47.6 to 1.8)	96 (33.9 to 240.5)	-47.1 (-64 to -10.6)
		Male	730 (577 to 1039)	457 (355.8 to 637.9)	20 (14 to 32)	19.8 (14.2 to 31)	1644 (1381 to 2106)	404.2 (340.9 to 533.3)	51 (40 to 80)	14.9 (11.7 to 23.8)	125.2 (76.6 to 188.2)	-11.5 (-30.4 to 13.6)	154.9 (72.7 to 275.4)	-24.7 (-46 to 7.8)
	Pneumoconiosis	Both	2 (2 to 3)	0.7 (0.5 to 1)	0 (0 to 0)	0 (0 to 0)	16 (12 to 22)	1.8 (1.4 to 2.4)	0 (0 to 1)	0.1 (0 to 0.1)	627.7 (406.9 to 1007.3)	165.1 (85.3 to 303.2)	461.2 (242 to 972)	94.5 (21.7 to 250.1)
		Female	1 (0 to 1)	0.3 (0.2 to 0.4)	0 (0 to 0)	0 (0 to 0)	9 (6 to 13)	1.9 (1.3 to 2.7)	0 (0 to 0)	0 (0 to 0)	1494.4 (1105 to 1937.2)	509.6 (348.6 to 680.9)	679 (351.1 to 1237.3)	122.4 (24.4 to 292.2)
		Male	2 (1 to 3)	1 (0.6 to 1.6)	0 (0 to 0)	0 (0 to 0.1)	7 (5 to 11)	1.9 (1.4 to 2.8)	0 (0 to 1)	0.1 (0.1 to 0.2)	331.4 (162.6 to 697.9)	78.1 (11.5 to 217.5)	438.7 (207.6 to 1007.2)	99.2 (19.3 to 282.4)
	Asthma	Both	2106 (1578 to 2781)	444.3 (341.5 to 574.5)	35 (25 to 49)	14.4 (9.7 to 21.3)	1963 (1466 to 2607)	217.1 (163.8 to 284.8)	36 (29 to 50)	4.9 (3.9 to 6.9)	-6.8 (-19.9 to 7.1)	-51.1 (-61.2 to -39.4)	3.1 (-26.8 to 45)	-66.1 (-77.7 to -50.3)
		Female	879 (624 to 1237)	369.5 (253 to 547.6)	13 (7 to 22)	11 (4.9 to 21)	832 (596 to 1137)	180.1 (127.5 to 243.9)	11 (6 to 16)	3 (1.5 to 4.2)	-5.4 (-22.5 to 12.8)	-51.3 (-66.2 to -37.1)	-9.3 (-50.2 to 43.4)	-72.9 (-86.8 to -54.1)
		Male	1227 (926 to 1654)	513.2 (396.1 to 698.9)	22 (16 to 33)	17.9 (12.6 to 26.8)	1131 (856 to 1519)	256.3 (195.5 to 345.5)	24 (19 to 38)	7 (5.3 to 11.1)	-7.8 (-24.4 to 10.5)	-50.1 (-62.4 to -36.9)	10.2 (-25.6 to 62.4)	-61 (-74.7 to -41.2)
	Interstitial lung disease and pulmonary sarcoidosis	Both	40 (27 to 61)	11 (7.2 to 19.1)	1 (1 to 2)	0.4 (0.2 to 0.9)	93 (67 to 139)	10.4 (7.2 to 16.2)	3 (2 to 5)	0.3 (0.2 to 0.7)	134.3 (78.7 to 196.4)	-5.7 (-29.3 to 24.3)	167.3 (73.5 to 291.8)	-16.5 (-44.7 to 26.4)
		Female	18 (12 to 33)	10.7 (6.7 to 21.1)	0 (0 to 1)	0.4 (0.2 to 1)	40 (26 to 65)	8.5 (5.5 to 14.4)	1 (0 to 2)	0.2 (0.1 to 0.5)	119.6 (65.5 to 190)	-20.3 (-42 to 14.9)	99.1 (22.7 to 220.5)	-44.4 (-65.5 to -4.6)
		Male	21 (14 to 36)	11.3 (7 to 20.8)	1 (0 to 1)	0.4 (0.2 to 0.9)	53 (36 to 85)	12.4 (8.2 to 21)	2 (1 to 3)	0.5 (0.3 to 1)	146.7 (63.6 to 243.9)	9.2 (-24.8 to 53.8)	224.2 (84.3 to 420.4)	10.6 (-32.7 to 81.1)
	Other chronic respiratory diseases	Both	83 (57 to 160)	11.6 (8.3 to 20.7)	0 (0 to 2)	0.1 (0.1 to 0.3)	215 (172 to 258)	20.7 (16.6 to 25)	1 (1 to 2)	0.1 (0.1 to 0.2)	159.1 (39.5 to 252.8)	78.7 (6.5 to 133.9)	156.8 (-11.2 to 590.8)	25 (-45.3 to 193.6)
		Female	44 (29 to 86)	12.1 (8.6 to 21.3)	0 (0 to 1)	0.1 (0 to 0.3)	107 (85 to 133)	21.1 (16.6 to 26.3)	0 (0 to 1)	0.1 (0.1 to 0.2)	145.2 (30 to 245)	73.8 (4.5 to 132.4)	109.8 (-43 to 561)	-3.7 (-59.7 to 148)
		Male	39 (25 to 78)	11.1 (7.4 to 19.8)	0 (0 to 1)	0.1 (0 to 0.3)	107 (83 to 133)	20.4 (15.8 to 25.5)	1 (1 to 1)	0.2 (0.1 to 0.3)	174.8 (51.8 to 310.1)	84.7 (9.8 to 167)	194 (9.7 to 759)	50.6 (-35.7 to 313.1)
	Digestive diseases	Both	2556 (2099 to 3094)	584.9 (480.4 to 707.6)	49 (39 to 60)	18.1 (13.9 to 23.5)	3523 (2750 to 4600)	375.9 (296.3 to 479.9)	74 (59 to 94)	9.7 (7.7 to 12.3)	37.8 (10.2 to 73.6)	-35.7 (-46.8 to -20.9)	50.9 (13.2 to 107.6)	-46.6 (-59.3 to -28.4)
		Female	1140 (914 to 1409)	533.4 (408.2 to 690.6)	19 (14 to 27)	15.8 (10.2 to 24)	1557 (1163 to 2055)	327.8 (247.5 to 428.4)	25 (19 to 33)	6.6 (5 to 8.6)	36.6 (9.3 to 67.6)	-38.6 (-51.2 to -24.9)	30.3 (-9.6 to 91)	-58.5 (-74 to -35.5)
		Male	1416 (1112 to 1748)	628.8 (493.6 to 769.5)	29 (22 to 38)	20.1 (15.2 to 25.7)	1966 (1522 to 2545)	426.6 (335.1 to 544.7)	48 (38 to 63)	13 (10.2 to 16.8)	38.9 (3.1 to 89.6)	-32.2 (-48.8 to -8)	64.5 (12.6 to 151.6)	-35.4 (-54.3 to -3.6)
	Cirrhosis and other chronic liver diseases	Both	896 (684 to 1162)	217.6 (166.1 to 282.6)	26 (19 to 33)	8.6 (6.2 to 11.4)	1148 (860 to 1509)	129.2 (97 to 168.1)	43 (32 to 56)	5.4 (4 to 7.1)	28 (-10.9 to 102.5)	-40.6 (-57 to -10.8)	68.4 (23.6 to 151.8)	-36.9 (-52.6 to -10.1)
		Female	348 (255 to 454)	170.6 (118.8 to 238.9)	10 (7 to 14)	7.2 (4.3 to 11.1)	355 (248 to 475)	80.1 (56.3 to 106.3)	14 (9 to 18)	3.4 (2.4 to 4.6)	1.8 (-31.5 to 61.3)	-53.1 (-67.3 to -29.4)	39.8 (-6.7 to 107.8)	-52.6 (-71.4 to -24.4)
		Male	548 (380 to 792)	258.5 (181.2 to 375.8)	16 (11 to 23)	9.9 (6.8 to 14.4)	793 (572 to 1085)	179.5 (130.2 to 242.1)	30 (21 to 40)	7.6 (5.4 to 10.2)	44.7 (-7 to 147.8)	-30.6 (-54.5 to 15.7)	85.9 (23 to 215.3)	-23 (-47.7 to 27)

Province	Cause	Sex	1990				2019				1990-2019 percent change (%)			
			DALYs		Death		DALYs		Death		DALYs		Death	
			Number	Rate	Number	Rate	Number	Rate	Number	Rate	Number	Rate	Number	Rate
	Upper digestive system diseases	Both	834 (574 to 1207)	195.2 (131.7 to 276.5)	10 (5 to 14)	4.2 (2 to 5.9)	1328 (819 to 2121)	131.7 (82.6 to 208.7)	9 (4 to 12)	1.3 (0.6 to 1.7)	59.2 (30.1 to 82.1)	-32.5 (-45.7 to -19.6)	-8.2 (-34.8 to 28.3)	-69.7 (-78.1 to -57.2)
		Female	382 (246 to 579)	179.6 (114 to 267.7)	3 (1 to 5)	3 (1.1 to 5.3)	678 (403 to 1111)	134.6 (80.8 to 218)	3 (1 to 5)	0.8 (0.4 to 1.3)	77.5 (50.6 to 99.3)	-25.1 (-40.2 to -12)	-5 (-43.6 to 61.3)	-72.5 (-85.1 to -48.8)
		Male	452 (318 to 647)	209.8 (143.9 to 293.1)	7 (4 to 9)	5.4 (2.7 to 7.5)	650 (413 to 1030)	129.5 (84.2 to 198.4)	6 (3 to 8)	1.7 (0.8 to 2.4)	43.8 (9.9 to 71.2)	-38.3 (-52.9 to -24)	-9.8 (-41.5 to 35)	-67.6 (-78.5 to -51.9)
	Appendicitis	Both	103 (65 to 140)	17 (11.4 to 23.2)	2 (1 to 3)	0.6 (0.3 to 0.9)	71 (47 to 101)	7.4 (4.9 to 10.4)	1 (1 to 2)	0.2 (0.1 to 0.3)	-31.6 (-57 to 30.8)	-56.4 (-70.9 to -28.9)	-25.4 (-54.8 to 37.8)	-67.7 (-80.2 to -48.5)
		Female	44 (25 to 68)	14.7 (8.7 to 23.6)	1 (0 to 1)	0.5 (0.2 to 0.9)	32 (20 to 46)	6.8 (4.2 to 9.7)	1 (0 to 1)	0.1 (0.1 to 0.2)	-26.3 (-58.7 to 47.3)	-53.8 (-73.2 to -19.3)	-26 (-65.3 to 44.5)	-70.8 (-86.3 to -47.4)
		Male	60 (33 to 85)	19.1 (11.4 to 26.4)	1 (1 to 2)	0.6 (0.3 to 0.9)	39 (24 to 58)	8.1 (4.7 to 12.1)	1 (0 to 1)	0.2 (0.1 to 0.3)	-35.5 (-62.8 to 47.5)	-57.8 (-74 to -11.7)	-24.9 (-62 to 78.1)	-64.4 (-79.3 to -26)
	Paralytic ileus and intestinal obstruction	Both	240 (154 to 374)	38.3 (28.7 to 54.2)	5 (3 to 7)	1.6 (1.1 to 2.3)	147 (104 to 200)	17.9 (12.6 to 24.4)	5 (4 to 8)	0.8 (0.5 to 1.2)	-38.7 (-66 to 9.3)	-53.3 (-68.7 to -31.9)	20.8 (-22.7 to 79.5)	-51.8 (-66.6 to -32.8)
		Female	119 (72 to 201)	40.9 (26.4 to 60.3)	2 (2 to 4)	1.8 (1 to 3)	62 (40 to 87)	14.8 (9.7 to 21)	2 (1 to 3)	0.6 (0.3 to 0.9)	-48.2 (-74 to 1.4)	-63.8 (-76.7 to -41.3)	-12.8 (-47.4 to 44.4)	-68.7 (-83.2 to -48)
		Male	121 (67 to 226)	35.4 (23.4 to 59.1)	2 (1 to 4)	1.3 (0.9 to 2.3)	85 (56 to 126)	21.2 (13.8 to 31.7)	3 (2 to 6)	1 (0.6 to 1.7)	-29.5 (-68.5 to 50.3)	-40.2 (-66.7 to 2.9)	58.4 (-17.9 to 181.9)	-26.1 (-54.2 to 19.3)
	Inguinal, femoral, and abdominal hernia	Both	121 (79 to 178)	18.7 (13.1 to 25.9)	1 (0 to 1)	0.2 (0.1 to 0.4)	154 (101 to 222)	15.9 (10.4 to 22.9)	1 (0 to 1)	0.1 (0 to 0.2)	27.6 (-0.1 to 63.2)	-14.8 (-29.6 to 0.7)	67 (-5 to 225.5)	-46 (-69.4 to -5.2)
		Female	45 (28 to 68)	13.4 (9 to 18.9)	0 (0 to 0)	0.2 (0 to 0.4)	53 (33 to 80)	11 (6.9 to 16.4)	0 (0 to 0)	0.1 (0 to 0.1)	17.9 (-16 to 65.3)	-18.4 (-40.2 to 5.6)	48 (-39.7 to 188.5)	-56.5 (-79.8 to -15.1)
		Male	76 (49 to 112)	23.6 (16.5 to 32.8)	0 (0 to 1)	0.3 (0.1 to 0.6)	101 (66 to 147)	20.9 (13.6 to 30.1)	1 (0 to 1)	0.2 (0.1 to 0.3)	33.4 (0.2 to 75.5)	-11.4 (-29.1 to 7.1)	79.3 (-1.3 to 280.5)	-39.1 (-67.7 to 28.4)
	Inflammatory bowel disease	Both	38 (27 to 51)	9.5 (6.6 to 12.9)	0 (0 to 1)	0.2 (0.1 to 0.3)	84 (58 to 117)	8.4 (5.9 to 11.6)	1 (0 to 1)	0.1 (0 to 0.1)	120.1 (80.1 to 168.2)	-11.1 (-28 to 7.5)	83.2 (13.3 to 185.1)	-35.6 (-60.4 to -0.4)
		Female	18 (12 to 26)	9.4 (6.1 to 13.6)	0 (0 to 0)	0.1 (0 to 0.3)	39 (26 to 56)	7.8 (5.2 to 11.1)	0 (0 to 0)	0.1 (0 to 0.1)	113.3 (63.1 to 174.4)	-16.9 (-40 to 7.5)	53.9 (-21.4 to 186.3)	-51.5 (-78.1 to -3.2)
		Male	20 (14 to 27)	9.6 (6.5 to 12.9)	0 (0 to 0)	0.2 (0.1 to 0.3)	45 (31 to 62)	9.1 (6.2 to 12.5)	1 (0 to 1)	0.1 (0 to 0.2)	126.3 (69.3 to 194.8)	-4.8 (-27.4 to 22.8)	104.8 (4.3 to 261.5)	-20.6 (-55.5 to 36.5)
	Vascular intestinal disorders	Both	31 (15 to 46)	12 (5.2 to 19.1)	1 (1 to 2)	0.7 (0.3 to 1.2)	57 (28 to 78)	7.3 (3.6 to 10)	3 (1 to 4)	0.4 (0.2 to 0.6)	83.8 (26.3 to 161.8)	-39.1 (-59.8 to -10.2)	113.4 (38.2 to 223)	-40.1 (-62.9 to -4.5)
		Female	16 (5 to 29)	14.3 (4.1 to 27.2)	1 (0 to 2)	0.9 (0.2 to 1.7)	25 (11 to 37)	6.3 (2.8 to 9.4)	1 (1 to 2)	0.4 (0.2 to 0.5)	54.9 (-3.4 to 168)	-55.5 (-73.4 to -12.2)	63.2 (-6.7 to 241.7)	-58.2 (-77.2 to -5.3)
		Male	15 (9 to 21)	9.7 (5.6 to 13.8)	1 (0 to 1)	0.5 (0.3 to 0.8)	32 (14 to 45)	8.4 (3.7 to 11.8)	2 (1 to 2)	0.5 (0.2 to 0.7)	115.4 (38.2 to 244.6)	-13.8 (-43.3 to 36.5)	183.4 (79.6 to 352.1)	-6.3 (-39.3 to 46.3)
	Gallbladder and biliary diseases	Both	187 (126 to 270)	48.3 (33.2 to 69)	2 (1 to 3)	0.9 (0.6 to 1.5)	371 (252 to 548)	39.4 (27.4 to 56.6)	4 (3 to 7)	0.6 (0.4 to 1)	98 (66.5 to 133)	-18.4 (-30.3 to -5.5)	156.4 (80.6 to 292.7)	-26.9 (-47.9 to 8.3)
		Female	124 (81 to 183)	64.6 (43 to 92.5)	1 (1 to 2)	0.9 (0.5 to 1.7)	250 (162 to 369)	52 (34.7 to 76.5)	2 (1 to 3)	0.6 (0.4 to 0.9)	101.9 (68.1 to 137.7)	-19.5 (-33.1 to -6.6)	130.4 (41.8 to 291.8)	-38.5 (-65.1 to 7.7)
		Male	64 (42 to 93)	32.9 (22.8 to 48.7)	1 (0 to 2)	0.8 (0.5 to 1.5)	121 (84 to 175)	27.2 (19.5 to 38.4)	2 (1 to 4)	0.7 (0.4 to 1.2)	90.5 (48.4 to 148.4)	-17.5 (-35.4 to 7.4)	184.8 (73.3 to 439.8)	-10.9 (-43 to 70.5)
	Pancreatitis	Both	53 (35 to 98)	15.9 (10 to 30.3)	2 (1 to 3)	0.7 (0.4 to 1.5)	79 (55 to 137)	8.8 (6.1 to 15.3)	3 (2 to 5)	0.4 (0.2 to 0.7)	49.4 (8 to 104.3)	-44.3 (-59.8 to -22.3)	66.6 (15.3 to 146.4)	-46.8 (-65.5 to -18.4)
		Female	20 (12 to 42)	13.4 (6.7 to 30.7)	1 (0 to 2)	0.6 (0.3 to 1.7)	28 (18 to 50)	6.4 (4.1 to 11.5)	1 (1 to 2)	0.3 (0.2 to 0.6)	41.4 (-8.1 to 111)	-52.5 (-71.1 to -22.1)	51.4 (-18.4 to 179.2)	-57.8 (-78.5 to -14.9)

Province	Cause	Sex	1990				2019				1990-2019 percent change (%)			
			DALYs		Death		DALYs		Death		DALYs		Death	
			Number	Rate	Number	Rate	Number	Rate	Number	Rate	Number	Rate	Number	Rate
		Male	33 (21 to 57)	17.8 (11 to 30.7)	1 (1 to 2)	0.7 (0.4 to 1.2)	51 (32 to 90)	11.3 (7.1 to 20.1)	2 (1 to 3)	0.5 (0.3 to 0.8)	54.2 (0.5 to 132.2)	-36.7 (-58.3 to -6.3)	77.3 (12.5 to 173.8)	-33.9 (-57.1 to -1.3)
	Other digestive diseases	Both	52 (39 to 67)	12.4 (8.2 to 17.1)	1 (1 to 2)	0.5 (0.2 to 0.8)	84 (50 to 113)	9.7 (5.7 to 13)	3 (1 to 4)	0.4 (0.2 to 0.5)	62.9 (7.1 to 133.7)	-22 (-45.1 to 9.8)	138.1 (49.6 to 271.1)	-27.4 (-56.3 to 10.3)
		Female	24 (16 to 33)	12.5 (6.3 to 20.7)	1 (0 to 1)	0.5 (0.1 to 1.1)	35 (20 to 49)	8 (4.6 to 11.1)	1 (0 to 1)	0.3 (0.1 to 0.4)	47.6 (-1.5 to 121.5)	-35.8 (-59 to -7)	82 (-0.9 to 224.7)	-50.8 (-76.1 to 7.9)
		Male	28 (20 to 38)	12.3 (8.6 to 17.1)	1 (0 to 1)	0.5 (0.3 to 0.7)	49 (27 to 68)	11.5 (6.1 to 15.8)	2 (1 to 3)	0.5 (0.2 to 0.7)	76.1 (0.1 to 189.5)	-6.4 (-41.9 to 51.7)	190.4 (58.7 to 434.4)	4.1 (-39.1 to 85.4)
	Neurological disorders	Both	7451 (3603 to 13173)	1428.5 (783.7 to 2346.2)	57 (30 to 120)	31 (12.1 to 73.6)	12444 (6353 to 21608)	1337.8 (732.7 to 2267.4)	195 (74 to 454)	29.1 (10.7 to 68.6)	67 (32 to 117.6)	-6.3 (-20 to 9)	243.4 (127.9 to 367)	-6.3 (-20.5 to 25.7)
		Female	3991 (1783 to 7269)	1552.7 (790.9 to 2696.5)	28 (13 to 61)	29.6 (10.8 to 74.9)	7001 (3272 to 12665)	1485.1 (738.1 to 2633.9)	99 (33 to 242)	28 (9.1 to 69.4)	75.4 (39.6 to 128.1)	-4.4 (-18.3 to 11)	248.5 (119.9 to 421.4)	-5.4 (-27 to 37.6)
		Male	3459 (1833 to 5811)	1314.6 (758 to 2103.9)	29 (15 to 56)	32.9 (13.5 to 78.3)	5444 (3066 to 9067)	1197.8 (698.9 to 1982.3)	97 (41 to 223)	30.2 (11.8 to 70.8)	57.4 (21.4 to 112.1)	-8.9 (-23.8 to 8.8)	238.4 (117.4 to 368.1)	-8.2 (-26.6 to 21.2)
	Alzheimer's disease and other dementias	Both	651 (281 to 1523)	385.5 (168.3 to 874.7)	34 (8 to 95)	25.1 (5.9 to 67.7)	2604 (1173 to 5618)	379.3 (171.1 to 815.8)	159 (38 to 424)	24.4 (5.9 to 64.7)	299.7 (241.7 to 399.5)	-1.6 (-13.7 to 21.2)	362.8 (285.8 to 547)	-2.7 (-16.8 to 35.6)
		Female	337 (150 to 766)	388.5 (173.2 to 887.6)	19 (4 to 52)	24.8 (5.9 to 70.5)	1394 (634 to 3017)	388.9 (177.3 to 837.4)	86 (21 to 231)	24.8 (6 to 66.8)	313.8 (241.5 to 451.9)	0.1 (-16.6 to 32.1)	354.2 (256.6 to 597.1)	-0.2 (-21.3 to 55.9)
		Male	315 (134 to 729)	384.7 (163.2 to 871.5)	16 (4 to 43)	25.5 (6 to 69.7)	1210 (529 to 2712)	368.7 (160.8 to 835.4)	73 (18 to 201)	23.9 (5.9 to 65.6)	284.7 (208.5 to 373.5)	-4.2 (-18.1 to 15)	373.2 (271.2 to 527.9)	-6.4 (-23.4 to 20.4)
	Parkinson's disease	Both	133 (113 to 161)	61.1 (52.6 to 76.2)	6 (5 to 8)	3.5 (2.9 to 4.8)	401 (331 to 495)	55.4 (45.7 to 69.1)	23 (18 to 30)	3.3 (2.6 to 4.3)	201.8 (140.5 to 285.5)	-9.4 (-27.3 to 14.1)	277.8 (189.7 to 401.1)	-6.5 (-26.7 to 21.8)
		Female	48 (38 to 62)	45.5 (36 to 62.2)	2 (2 to 3)	2.5 (2 to 3.8)	138 (111 to 178)	36.8 (29.5 to 47.8)	7 (5 to 11)	2 (1.5 to 3)	189.2 (121 to 279.3)	-19.2 (-38.5 to 6.4)	231.5 (141.2 to 367.7)	-20.5 (-42 to 11.4)
		Male	85 (67 to 108)	78.3 (62.7 to 100.8)	4 (3 to 5)	4.7 (3.7 to 6.3)	263 (213 to 334)	75.3 (61.1 to 96.2)	15 (12 to 21)	4.6 (3.7 to 6.2)	208.9 (128 to 334.5)	-3.7 (-27.1 to 34.2)	304.2 (188.3 to 501.4)	-1.1 (-27.4 to 43.5)
	Idiopathic epilepsy	Both	1894 (979 to 2994)	212.5 (110.8 to 343.5)	13 (8 to 17)	1.7 (1.2 to 2.2)	1281 (571 to 2187)	131.5 (58 to 224)	7 (6 to 9)	0.8 (0.6 to 1)	-32.4 (-71.7 to 60.9)	-38.1 (-74.2 to 43.4)	-44.5 (-62.9 to 3.8)	-56 (-68.3 to -25.6)
		Female	865 (403 to 1378)	196.5 (95.7 to 314.1)	6 (3 to 9)	1.6 (0.9 to 2.1)	573 (246 to 994)	120 (51.2 to 208.6)	3 (2 to 4)	0.6 (0.4 to 0.9)	-33.7 (-72.7 to 70.8)	-39 (-74.8 to 52.7)	-50.9 (-71.5 to 12.1)	-61.2 (-74.9 to -24.4)
		Male	1028 (514 to 1684)	227.8 (117.6 to 377.5)	7 (4 to 10)	1.9 (1.3 to 2.5)	707 (322 to 1203)	142.8 (64.3 to 245)	4 (3 to 6)	0.9 (0.7 to 1.2)	-31.2 (-71 to 64)	-37.3 (-73.8 to 42.4)	-39.3 (-61.6 to 28.5)	-51.5 (-66.8 to -9.1)
	Multiple sclerosis	Both	100 (70 to 155)	23 (16.1 to 37.3)	1 (1 to 3)	0.3 (0.2 to 0.8)	230 (165 to 347)	21.9 (15.8 to 32.8)	3 (2 to 6)	0.3 (0.2 to 0.6)	130.6 (71.8 to 195.7)	-4.9 (-27.6 to 20.6)	160.5 (63 to 312.3)	-1 (-35.4 to 60.8)
		Female	59 (39 to 88)	27.4 (18.9 to 41.3)	1 (0 to 1)	0.4 (0.2 to 0.8)	140 (102 to 186)	26.8 (19.6 to 35.4)	2 (1 to 3)	0.4 (0.2 to 0.6)	138.8 (55.8 to 231.3)	-2.1 (-34.7 to 31.4)	162.7 (26.1 to 378.9)	-4.1 (-49.5 to 86.9)
		Male	41 (25 to 81)	18.8 (11.5 to 38.7)	1 (0 to 2)	0.3 (0.1 to 0.9)	90 (54 to 174)	17.1 (10.5 to 32.9)	1 (1 to 4)	0.3 (0.2 to 0.8)	118.7 (40.8 to 203.3)	-9 (-39.6 to 23.8)	157.9 (32 to 327)	4.2 (-45 to 72.9)
	Motor neuron disease	Both	11 (9 to 14)	2.2 (1.7 to 2.8)	0 (0 to 0)	0.1 (0 to 0.1)	22 (17 to 28)	2.3 (1.8 to 2.9)	0 (0 to 1)	0.1 (0 to 0.1)	99.5 (46 to 169.7)	1.4 (-28.4 to 40.5)	131 (44.4 to 264.6)	-1.4 (-38.8 to 58.5)
		Female	6 (4 to 7)	2.1 (1.6 to 2.8)	0 (0 to 0)	0 (0 to 0.1)	10 (8 to 12)	2 (1.6 to 2.5)	0 (0 to 0)	0 (0 to 0.1)	79.1 (27.6 to 138.8)	-3.5 (-34.7 to 35.6)	109 (23.9 to 227.2)	-10.8 (-50.2 to 47.8)

Province	Cause	Sex	1990				2019				1990-2019 percent change (%)			
			DALYs		Death		DALYs		Death		DALYs		Death	
			Number	Rate	Number	Rate	Number	Rate	Number	Rate	Number	Rate	Number	Rate
		Male	6 (4 to 7)	2.3 (1.7 to 3.2)	0 (0 to 0)	0.1 (0 to 0.1)	12 (9 to 16)	2.5 (1.9 to 3.4)	0 (0 to 0)	0.1 (0 to 0.1)	119.5 (45 to 228)	7.9 (-31.1 to 64.5)	148.2 (38.1 to 341.4)	9.8 (-38.9 to 92.8)
	Headache disorders	Both	4324 (899 to 9573)	704.1 (171.1 to 1503.4)	--	--	7526 (1842 to 16046)	707.4 (172.4 to 1510.4)	--	--	74 (59.5 to 112.6)	0.5 (-4.5 to 6.7)	--	--
		Female	2540 (489 to 5714)	858.8 (191.9 to 1882.7)	--	--	4577 (1011 to 9929)	874.8 (193.4 to 1903)	--	--	80.2 (63.6 to 113.5)	1.9 (-4.7 to 8.7)	--	--
		Male	1784 (399 to 3876)	556.7 (148.5 to 1166.4)	--	--	2949 (826 to 6167)	547 (151.3 to 1144.3)	--	--	65.3 (47.6 to 113.7)	-1.8 (-7.9 to 7.8)	--	--
	Other neurological disorders	Both	338 (175 to 582)	40.1 (22.8 to 64.5)	2 (1 to 3)	0.3 (0.3 to 0.4)	381 (205 to 612)	40.1 (21.6 to 64.5)	3 (2 to 3)	0.3 (0.2 to 0.3)	12.7 (-44.1 to 149.5)	0.1 (-49 to 101.5)	29.8 (-9.3 to 87.1)	-15.9 (-39.9 to 24.4)
		Female	137 (61 to 248)	33.8 (17.7 to 56.9)	1 (0 to 1)	0.2 (0.2 to 0.3)	168 (87 to 280)	35.8 (18.4 to 59.9)	1 (1 to 1)	0.2 (0.2 to 0.2)	22.3 (-43.4 to 200.5)	5.8 (-48.9 to 129.4)	43.3 (2.7 to 106.5)	-20.6 (-45.5 to 17.2)
		Male	201 (108 to 338)	46 (26.9 to 73.7)	1 (1 to 2)	0.4 (0.3 to 0.6)	213 (116 to 342)	44.3 (24.3 to 70.5)	2 (1 to 2)	0.4 (0.3 to 0.5)	6.1 (-47 to 115.8)	-3.7 (-50.4 to 86.6)	23.6 (-22 to 102.6)	-11.9 (-41.2 to 39.7)
	Mental disorders	Both	13179 (9644 to 17570)	2084.7 (1537.8 to 2740.7)	0 (0 to 0)	0 (0 to 0)	22105 (16260 to 29285)	2107.7 (1557 to 2801.6)	0 (0 to 0)	0 (0 to 0)	67.7 (58.3 to 78.1)	1.1 (-1.8 to 4.2)	285 (66.4 to 999.5)	76.3 (-21.5 to 485.7)
		Female	7122 (5164 to 9447)	2356.5 (1733.6 to 3089.8)	0 (0 to 0)	0 (0 to 0)	12243 (9017 to 16344)	2376.6 (1765.5 to 3174.4)	0 (0 to 0)	0 (0 to 0)	71.9 (61.6 to 84.2)	0.9 (-3.3 to 5.3)	268.9 (49.3 to 1145.1)	71.7 (-31 to 569.6)
		Male	6058 (4400 to 8116)	1826.6 (1346.9 to 2407.8)	0 (0 to 0)	0 (0 to 0)	9863 (7203 to 13073)	1847.7 (1353.9 to 2451.1)	0 (0 to 0)	0 (0 to 0)	62.8 (52.3 to 74)	1.2 (-2.5 to 5.1)	452.5 (57.4 to 1599.9)	170.8 (-24.1 to 889.6)
	Schizophrenia	Both	802 (579 to 1039)	161.9 (116.5 to 207.1)	--	--	1844 (1349 to 2385)	163.2 (120 to 209.6)	--	--	130 (108.4 to 153.3)	0.9 (-7.5 to 9.6)	--	--
		Female	370 (263 to 481)	153.7 (110.1 to 198.2)	--	--	851 (626 to 1113)	154.5 (113.7 to 199.6)	--	--	130 (103.2 to 164.5)	0.5 (-11 to 14.6)	--	--
		Male	432 (309 to 560)	169.6 (123.3 to 218)	--	--	993 (718 to 1290)	171.6 (125 to 220.3)	--	--	130 (102.2 to 160.3)	1.2 (-9.1 to 13.1)	--	--
	Depressive disorders	Both	3719 (2368 to 5432)	673.7 (444.5 to 972.9)	--	--	7535 (4999 to 11071)	707.7 (473.6 to 1030.5)	--	--	102.6 (81.1 to 124.8)	5 (0.1 to 10.3)	--	--
		Female	2349 (1521 to 3423)	864.2 (570.3 to 1246.3)	--	--	4584 (3051 to 6692)	880.1 (589.6 to 1272.6)	--	--	95.2 (74 to 119.4)	1.8 (-4.6 to 9.2)	--	--
		Male	1371 (872 to 2053)	493.3 (321.2 to 725.3)	--	--	2951 (1907 to 4443)	541.1 (356.1 to 805.7)	--	--	115.3 (90 to 143.7)	9.7 (2.2 to 17.3)	--	--
	Bipolar disorder	Both	1007 (613 to 1510)	174.5 (108 to 262.9)	--	--	1879 (1148 to 2907)	177.1 (108.4 to 270.7)	--	--	86.6 (68.3 to 105.8)	1.5 (-5.3 to 8.9)	--	--
		Female	540 (321 to 823)	188.6 (114.4 to 284.3)	--	--	991 (609 to 1526)	191.2 (117 to 292.4)	--	--	83.6 (61 to 109)	1.4 (-8 to 12.7)	--	--
		Male	467 (285 to 707)	161 (99.5 to 244.7)	--	--	888 (541 to 1375)	163.6 (101 to 252)	--	--	90.2 (69.3 to 114.8)	1.6 (-8 to 12.2)	--	--
	Anxiety disorders	Both	4512 (3117 to 6236)	673.6 (467 to 920.9)	--	--	7167 (4939 to 9896)	695.9 (487 to 957.6)	--	--	58.8 (44.1 to 77.1)	3.3 (-2.3 to 9.6)	--	--
		Female	2660 (1836 to 3697)	828.1 (575 to 1133.8)	--	--	4340 (2954 to 5971)	854.9 (591.6 to 1172.3)	--	--	63.1 (46.4 to 85)	3.2 (-4.6 to 11.4)	--	--
		Male	1852 (1280 to 2561)	526.5 (364.8 to 726.1)	--	--	2827 (1969 to 3958)	542.4 (381.4 to 756.3)	--	--	52.7 (35 to 72.6)	3 (-5.7 to 11.8)	--	--

Province	Cause	Sex	1990				2019				1990-2019 percent change (%)			
			DALYs		Death		DALYs		Death		DALYs		Death	
			Number	Rate	Number	Rate	Number	Rate	Number	Rate	Number	Rate	Number	Rate
	Eating disorders	Both	283 (176 to 424)	39.1 (24 to 57.9)	0 (0 to 0)	0 (0 to 0)	596 (372 to 888)	53.9 (33.8 to 79.9)	0 (0 to 0)	0 (0 to 0)	111.1 (81.5 to 149.5)	37.9 (23 to 53.4)	285 (66.4 to 999.5)	76.3 (-21.5 to 485.7)
		Female	173 (107 to 261)	48.5 (30.4 to 72.7)	0 (0 to 0)	0 (0 to 0)	359 (225 to 528)	67.5 (42.2 to 99.1)	0 (0 to 0)	0 (0 to 0)	108.2 (75.9 to 149)	39.2 (21.3 to 59.7)	268.9 (49.3 to 1145.1)	71.7 (-31 to 569.6)
		Male	110 (67 to 166)	29.9 (18 to 45.8)	0 (0 to 0)	0 (0 to 0)	237 (141 to 368)	40.9 (25.1 to 61.7)	0 (0 to 0)	0 (0 to 0)	115.6 (73.8 to 167.7)	36.8 (14.6 to 61.9)	452.5 (57.4 to 1599.9)	170.8 (-24.1 to 889.6)
	Autism spectrum disorders	Both	360 (232 to 525)	44.6 (29 to 64.8)	--	--	455 (294 to 662)	45.1 (29.2 to 65.9)	--	--	26.3 (14.3 to 40.1)	1.2 (-7.7 to 11.8)	--	--
		Female	88 (55 to 135)	22.3 (14.3 to 33.8)	--	--	111 (71 to 162)	22.5 (14.4 to 33)	--	--	25.7 (5.9 to 50.4)	0.9 (-14.1 to 19)	--	--
		Male	272 (175 to 392)	65.8 (42.8 to 95.1)	--	--	344 (222 to 498)	66.9 (43 to 97)	--	--	26.5 (12.2 to 43.8)	1.6 (-8.9 to 14.9)	--	--
	Attention-deficit/hyperactivity disorder	Both	298 (166 to 516)	32.5 (18.3 to 55.9)	--	--	334 (187 to 560)	33.7 (18.9 to 56.8)	--	--	12.2 (-4.7 to 32.4)	3.4 (-10 to 16.7)	--	--
		Female	89 (50 to 153)	20.1 (11.3 to 34.8)	--	--	101 (57 to 168)	20.9 (11.7 to 34.7)	--	--	13.4 (-6 to 39.4)	4.1 (-12.8 to 25)	--	--
		Male	209 (115 to 359)	44.5 (24.8 to 76)	--	--	233 (130 to 396)	45.9 (25.7 to 78.2)	--	--	11.7 (-8.1 to 37.2)	3 (-13.5 to 22.2)	--	--
	Conduct disorder	Both	863 (490 to 1360)	75.8 (43.1 to 120.6)	--	--	658 (376 to 1048)	74.3 (42.4 to 117.8)	--	--	-23.8 (-30.8 to -16)	-1.9 (-11.1 to 8.1)	--	--
		Female	294 (161 to 478)	52.6 (28.7 to 85.6)	--	--	222 (116 to 364)	51 (26.6 to 83.8)	--	--	-24.6 (-35.4 to -11.8)	-3.1 (-17.1 to 13)	--	--
		Male	569 (330 to 881)	97.9 (56.3 to 151.4)	--	--	436 (250 to 690)	96.7 (55.2 to 152.8)	--	--	-23.3 (-32.5 to -13.8)	-1.2 (-13.2 to 10.9)	--	--
	Idiopathic developmental intellectual disability	Both	883 (494 to 1404)	108.1 (60.6 to 170.8)	--	--	562 (282 to 944)	55.6 (27.9 to 93.8)	--	--	-36.4 (-47.2 to -29.7)	-48.6 (-57.6 to -43.1)	--	--
		Female	382 (208 to 614)	95.7 (52.6 to 154)	--	--	252 (128 to 428)	50.9 (25.7 to 87.1)	--	--	-34 (-44.8 to -26.2)	-46.8 (-55.7 to -40.8)	--	--
		Male	502 (279 to 797)	119.9 (67.4 to 189.4)	--	--	310 (153 to 525)	60 (29.4 to 101.6)	--	--	-38.1 (-49.7 to -30.1)	-50 (-59.3 to -43.5)	--	--
	Other mental disorders	Both	453 (289 to 693)	101 (65.1 to 153.1)	--	--	1076 (691 to 1653)	101.2 (65.4 to 154.7)	--	--	137.3 (119.7 to 156.7)	0.2 (-5 to 5.6)	--	--
		Female	178 (112 to 272)	82.8 (52.5 to 127.4)	--	--	432 (271 to 666)	83.1 (52.7 to 127.2)	--	--	143.4 (119.6 to 172.2)	0.4 (-7.5 to 9.6)	--	--
		Male	276 (175 to 420)	118.2 (76.4 to 178.7)	--	--	644 (416 to 990)	118.8 (76.7 to 181.5)	--	--	133.4 (111.5 to 156.6)	0.5 (-6.6 to 7.8)	--	--
	Substance use disorders	Both	1178 (897 to 1499)	206.5 (160.1 to 258.3)	6 (5 to 8)	1.5 (1.1 to 1.9)	3270 (2597 to 4015)	290.2 (229.8 to 355.8)	24 (20 to 30)	2.2 (1.8 to 2.7)	177.6 (141.6 to 223.6)	40.5 (23.5 to 61.8)	278.6 (171.2 to 446)	49.7 (7.1 to 113.5)
		Female	391 (281 to 527)	137 (100.7 to 181.4)	1 (1 to 2)	0.7 (0.5 to 0.9)	806 (602 to 1053)	147.5 (109.3 to 191.5)	4 (3 to 5)	0.7 (0.5 to 0.9)	106.3 (76 to 143.2)	7.7 (-7.2 to 24.8)	147.8 (59.1 to 297.8)	-1.6 (-33.6 to 44.1)

Province	Cause	Sex	1990				2019				1990-2019 percent change (%)			
			DALYs		Death		DALYs		Death		DALYs		Death	
			Number	Rate	Number	Rate	Number	Rate	Number	Rate	Number	Rate	Number	Rate
		Male	788 (594 to 997)	273.2 (211.3 to 339.9)	5 (3 to 7)	2.2 (1.6 to 3)	2465 (1949 to 3009)	426.3 (339.7 to 521.8)	21 (16 to 26)	3.7 (2.9 to 4.6)	212.9 (165.8 to 275)	56 (32.7 to 85.7)	317.7 (179.7 to 527.5)	66.4 (13.1 to 147.7)
	Alcohol use disorders	Both	280 (206 to 363)	53.5 (39.2 to 68.5)	2 (1 to 3)	0.5 (0.4 to 0.7)	430 (306 to 587)	38.6 (27.4 to 51.9)	2 (2 to 3)	0.2 (0.2 to 0.3)	53.4 (29.8 to 82.6)	-27.9 (-39.5 to -13.6)	13.8 (-25.1 to 100.7)	-53.7 (-69 to -21.2)
		Female	82 (59 to 110)	30.5 (22.4 to 40.6)	1 (0 to 1)	0.3 (0.2 to 0.3)	130 (88 to 183)	23.9 (16.3 to 33.2)	0 (0 to 0)	0.1 (0.1 to 0.1)	57.1 (25.9 to 94.3)	-21.6 (-35.9 to -5.4)	-26.5 (-52.3 to 14.8)	-69 (-79 to -54)
		Male	198 (143 to 255)	75.5 (55.6 to 96.7)	2 (1 to 2)	0.7 (0.5 to 1)	301 (217 to 409)	52.7 (38.2 to 70.8)	2 (1 to 3)	0.4 (0.3 to 0.6)	51.9 (22.5 to 89.8)	-30.2 (-44.5 to -12.4)	27.3 (-25.2 to 148.1)	-47.6 (-68.5 to -0.2)
	Drug use disorders	Both	898 (659 to 1182)	153 (115.6 to 197.9)	4 (3 to 6)	1 (0.7 to 1.3)	2840 (2228 to 3529)	251.6 (197.8 to 312.1)	22 (17 to 27)	2 (1.6 to 2.4)	216.3 (167.4 to 276.6)	64.4 (41.7 to 92.7)	400.5 (247.4 to 643.2)	102.7 (42.6 to 194.8)
		Female	308 (214 to 434)	106.5 (75.1 to 146.5)	1 (1 to 1)	0.5 (0.3 to 0.6)	676 (497 to 897)	123.6 (91.2 to 162.7)	3 (2 to 4)	0.6 (0.5 to 0.8)	119.4 (82.4 to 167.7)	16 (-1.7 to 37.6)	239.2 (108.3 to 468.5)	35.5 (-12.1 to 109.1)
		Male	590 (438 to 769)	197.7 (151.5 to 252.9)	3 (2 to 5)	1.5 (1.1 to 2)	2164 (1717 to 2659)	373.6 (296.6 to 459.6)	19 (14 to 24)	3.3 (2.6 to 4.2)	266.9 (204 to 353.4)	88.9 (58.3 to 129.4)	446.1 (253.2 to 756)	122.9 (45.8 to 238)
	Diabetes and kidney diseases	Both	2805 (2316 to 3310)	720 (602.1 to 854.2)	49 (40 to 60)	18.4 (15.5 to 23)	7608 (6108 to 9204)	880.6 (715.6 to 1060.2)	132 (111 to 156)	17.8 (14.9 to 21.3)	171.3 (131.6 to 216.4)	22.3 (5.6 to 39.7)	169.2 (114.4 to 243.9)	-3.4 (-23.8 to 21)
		Female	1299 (1043 to 1588)	681.4 (555.6 to 841.6)	21 (16 to 28)	16.4 (12.6 to 23.2)	3545 (2760 to 4390)	810.1 (632.9 to 1000.4)	53 (42 to 68)	13.8 (11 to 18.1)	173 (122.4 to 230.5)	18.9 (-1 to 40.6)	149 (80.8 to 247.6)	-15.7 (-40.8 to 18.5)
		Male	1506 (1202 to 1810)	753.6 (606.5 to 905)	28 (21 to 36)	20.4 (15.6 to 25.8)	4063 (3299 to 4880)	955.1 (779.5 to 1142.6)	79 (64 to 94)	22 (17.7 to 26.2)	169.8 (123.1 to 223.9)	26.7 (6.4 to 49.5)	184.6 (112.1 to 285.5)	7.5 (-19.3 to 42.8)
	Diabetes mellitus	Both	1149 (897 to 1447)	340.5 (264.6 to 430.4)	14 (11 to 21)	5.4 (4.3 to 8.9)	5150 (3937 to 6570)	594.8 (455.7 to 754.3)	63 (53 to 79)	8.5 (7.1 to 10.8)	348.1 (294.3 to 397.6)	74.7 (54 to 92.4)	351.9 (242.8 to 501.1)	56.4 (17.8 to 110.9)
		Female	497 (381 to 667)	312.2 (239.5 to 427)	6 (4 to 12)	4.8 (3.4 to 11.1)	2415 (1792 to 3135)	552.1 (413.4 to 717.5)	25 (19 to 34)	6.5 (5 to 9.1)	386 (303.3 to 457.8)	76.8 (42.4 to 101.7)	324.7 (179.1 to 547.1)	35.4 (-14.3 to 107.3)
		Male	652 (504 to 814)	366.2 (284.7 to 457.5)	8 (6 to 11)	6.1 (4.7 to 8)	2735 (2101 to 3453)	639.5 (496.2 to 796.7)	39 (31 to 47)	10.7 (8.4 to 13)	319.3 (264.8 to 372.1)	74.6 (53.4 to 98.1)	371.1 (233.9 to 560.6)	74.4 (26.2 to 141.3)
	Chronic kidney disease	Both	1650 (1316 to 1964)	378.7 (318.6 to 439.2)	35 (28 to 42)	12.9 (10.6 to 15.9)	2422 (2035 to 2842)	281.9 (237.6 to 328.6)	67 (55 to 79)	9.1 (7.4 to 10.7)	46.8 (19.6 to 80.4)	-25.6 (-38.1 to -11)	92.7 (50.5 to 148.3)	-29.7 (-46.4 to -10.4)
		Female	799 (624 to 1001)	368.5 (295.3 to 454.3)	15 (11 to 20)	11.6 (8.6 to 16.8)	1115 (910 to 1339)	254.6 (206.9 to 305.1)	27 (21 to 35)	7.2 (5.6 to 9.2)	39.5 (8.2 to 79.9)	-30.9 (-45.1 to -13)	79.6 (27.7 to 154.9)	-37.8 (-60.4 to -10.6)
		Male	850 (653 to 1065)	386.5 (307.1 to 478.3)	19 (14 to 25)	14.3 (10.8 to 18.4)	1307 (1083 to 1561)	311 (259.2 to 369.8)	40 (31 to 49)	11.1 (8.7 to 13.7)	53.7 (19.2 to 99.2)	-19.5 (-37.4 to 1.2)	102.9 (47.5 to 175)	-22.3 (-42.8 to 6.5)
	Acute glomerulonephritis	Both	6 (2 to 50)	0.8 (0.4 to 5.2)	0 (0 to 1)	0 (0 to 0.1)	36 (12 to 51)	4 (1.5 to 5.6)	1 (0 to 2)	0.2 (0.1 to 0.2)	524.4 (-76.4 to 1865.1)	395.8 (-74.6 to 1033.1)	1141.7 (-49.6 to 2932.9)	561.5 (-62.4 to 1287.1)
		Female	2 (1 to 13)	0.7 (0.3 to 3)	0 (0 to 0)	0 (0 to 0.1)	15 (6 to 22)	3.3 (1.4 to 4.8)	1 (0 to 1)	0.1 (0.1 to 0.2)	522 (-68.3 to 1745.4)	382.8 (-66.8 to 1107.2)	1191 (-29.7 to 3314.4)	570.4 (-53.9 to 1555.9)

Province	Cause	Sex	1990				2019				1990-2019 percent change (%)			
			DALYs		Death		DALYs		Death		DALYs		Death	
			Number	Rate	Number	Rate	Number	Rate	Number	Rate	Number	Rate	Number	Rate
		Male	3 (1 to 33)	0.9 (0.3 to 6.9)	0 (0 to 1)	0 (0 to 0.2)	21 (5 to 32)	4.6 (1.1 to 7)	1 (0 to 1)	0.2 (0 to 0.3)	526.1 (-84.2 to 3100.4)	411 (-82.3 to 1736.6)	1107.3 (-66.9 to 4958.7)	554.4 (-74.4 to 2118)
	Skin and subcutaneous diseases	Both	3188 (2139 to 4651)	412.8 (276.2 to 604.1)	0 (0 to 1)	0.2 (0.1 to 0.4)	4094 (2728 to 6101)	419.6 (279.7 to 624.6)	1 (1 to 2)	0.1 (0.1 to 0.2)	28.4 (22.7 to 33.3)	1.7 (-1.4 to 4.2)	117.7 (55.4 to 219.6)	-31.2 (-52.6 to 10.7)
		Female	1658 (1111 to 2378)	437.7 (295.8 to 638.7)	0 (0 to 0)	0.2 (0.1 to 0.4)	2132 (1421 to 3142)	443.7 (294 to 656.9)	0 (0 to 1)	0.1 (0 to 0.1)	28.6 (22 to 34.5)	1.4 (-2.5 to 4.6)	58.6 (-0.1 to 188.1)	-53.9 (-73.3 to 1.9)
		Male	1530 (1012 to 2216)	389.1 (258.1 to 569.8)	0 (0 to 1)	0.2 (0.1 to 0.5)	1962 (1292 to 2904)	396.6 (261.7 to 585.6)	1 (0 to 1)	0.2 (0.1 to 0.4)	28.3 (21.9 to 33.8)	1.9 (-2.3 to 5.3)	169.9 (85.1 to 319.9)	-7.6 (-35.1 to 67.3)
	Dermatitis	Both	734 (423 to 1174)	93.5 (55.8 to 144.2)	--	--	947 (570 to 1444)	94.2 (56.9 to 144.6)	--	--	29.1 (17.8 to 41.6)	0.7 (-4.1 to 5.6)	--	--
		Female	399 (231 to 639)	103.9 (61.8 to 160.6)	--	--	517 (311 to 806)	104.7 (62.8 to 162.6)	--	--	29.5 (16.2 to 44.9)	0.7 (-6.1 to 7.5)	--	--
		Male	335 (194 to 531)	83.6 (50.3 to 129.6)	--	--	431 (259 to 651)	84 (50.6 to 126.8)	--	--	28.5 (16 to 43.9)	0.5 (-6.3 to 7.5)	--	--
	Psoriasis	Both	294 (207 to 397)	51.4 (36 to 68.1)	--	--	388 (272 to 515)	37.8 (26.7 to 50)	--	--	32.1 (18.8 to 47.5)	-26.5 (-33 to -18.9)	--	--
		Female	151 (107 to 203)	53.3 (37.5 to 71.3)	--	--	197 (137 to 263)	39.2 (27.6 to 52)	--	--	30.9 (12 to 53.3)	-26.5 (-35.9 to -16.2)	--	--
		Male	143 (98 to 194)	49.5 (34.1 to 66.3)	--	--	191 (133 to 258)	36.5 (25.6 to 49)	--	--	33.4 (13 to 55.3)	-26.4 (-36.4 to -15.6)	--	--
	Bacterial skin diseases	Both	42 (25 to 73)	6.5 (4.3 to 10.6)	0 (0 to 1)	0.1 (0.1 to 0.3)	55 (34 to 91)	5.7 (3.6 to 9.4)	1 (0 to 1)	0.1 (0.1 to 0.2)	29.1 (17.3 to 45.1)	-12.1 (-26.2 to 1.6)	98.9 (43.7 to 200.3)	-33.5 (-55.4 to 12.4)
		Female	19 (11 to 33)	6 (3.7 to 9.9)	0 (0 to 0)	0.1 (0.1 to 0.2)	23 (14 to 41)	4.8 (2.9 to 8.4)	0 (0 to 0)	0 (0 to 0.1)	21 (7.8 to 35.1)	-19.4 (-37.6 to -2)	33.6 (-18.7 to 140.5)	-59.5 (-77.1 to -9.4)
		Male	23 (14 to 41)	7 (4.5 to 12.3)	0 (0 to 0)	0.1 (0.1 to 0.4)	32 (20 to 52)	6.7 (4.3 to 11.3)	0 (0 to 1)	0.1 (0.1 to 0.3)	35.6 (18 to 60.4)	-5.3 (-20.8 to 12.7)	153.2 (73.2 to 302.1)	-7.9 (-36.9 to 72)
	Scabies	Both	203 (110 to 329)	25.2 (13.8 to 40.7)	--	--	249 (136 to 402)	25.1 (13.8 to 40.4)	--	--	22.5 (13.3 to 33.2)	-0.4 (-5 to 4)	--	--
		Female	100 (54 to 160)	25.2 (13.8 to 40.3)	--	--	122 (66 to 197)	25.2 (13.7 to 40.8)	--	--	22.4 (11.4 to 34.6)	-0.2 (-6.3 to 6.4)	--	--
		Male	103 (57 to 168)	25.1 (13.7 to 40.5)	--	--	127 (69 to 205)	25 (13.7 to 40.4)	--	--	22.6 (11.3 to 35.5)	-0.6 (-6.8 to 6.3)	--	--
	Fungal skin diseases	Both	94 (38 to 204)	18.6 (7.5 to 39.6)	--	--	171 (68 to 366)	18.3 (7.3 to 39.3)	--	--	80.5 (68.5 to 92.6)	-1.5 (-3.4 to 0.2)	--	--
		Female	42 (17 to 91)	17.4 (6.9 to 37.8)	--	--	79 (32 to 172)	17.1 (6.8 to 37.1)	--	--	87.6 (74.1 to 101.8)	-1.5 (-4.1 to 1.2)	--	--
		Male	52 (21 to 113)	19.8 (8 to 42.1)	--	--	91 (37 to 197)	19.5 (7.8 to 41.8)	--	--	74.7 (62.7 to 87.4)	-1.5 (-3.8 to 0.8)	--	--
	Viral skin diseases	Both	520 (329 to 781)	51.3 (32.5 to 76.3)	--	--	497 (316 to 740)	51.5 (32.6 to 76.9)	--	--	-4.4 (-8.7 to 0.2)	0.4 (-3.6 to 4.6)	--	--
		Female	252 (161 to 384)	50.9 (32.5 to 76.5)	--	--	242 (153 to 363)	51.1 (32.5 to 76.7)	--	--	-4.2 (-10.1 to 2.1)	0.4 (-5.5 to 6.5)	--	--
		Male	268 (168 to 400)	51.6 (33 to 76.5)	--	--	255 (163 to 379)	51.8 (33 to 76.8)	--	--	-4.6 (-10.7 to 1.8)	0.3 (-5.4 to 6.5)	--	--

Province	Cause	Sex	1990				2019				1990-2019 percent change (%)			
			DALYs		Death		DALYs		Death		DALYs		Death	
			Number	Rate	Number	Rate	Number	Rate	Number	Rate	Number	Rate	Number	Rate
	Acne vulgaris	Both	468 (282 to 748)	49.8 (30.1 to 79.6)	--	--	605 (361 to 969)	67.3 (40.2 to 107.8)	--	--	29.5 (19 to 41.7)	35 (25 to 47.2)	--	--
		Female	221 (130 to 353)	48.3 (28.9 to 76.9)	--	--	291 (172 to 468)	65.5 (39.1 to 106.1)	--	--	31.8 (16.3 to 49)	35.6 (19.8 to 53)	--	--
		Male	247 (145 to 397)	51.3 (30.4 to 82.8)	--	--	314 (188 to 501)	69.1 (41.3 to 110.1)	--	--	27.4 (13.5 to 43.9)	34.6 (20.8 to 51.6)	--	--
	Alopecia areata	Both	39 (25 to 59)	6.3 (4 to 9.5)	--	--	69 (44 to 104)	6.4 (4.1 to 9.6)	--	--	75.8 (55.9 to 97.4)	0.4 (-9.8 to 11.3)	--	--
		Female	25 (16 to 38)	8.3 (5.4 to 12.5)	--	--	44 (28 to 66)	8.3 (5.3 to 12.5)	--	--	76.6 (53.2 to 105.4)	0.3 (-11.6 to 13.8)	--	--
		Male	14 (9 to 22)	4.5 (2.7 to 6.8)	--	--	25 (15 to 38)	4.4 (2.8 to 6.8)	--	--	74.4 (41.1 to 113.7)	-0.3 (-17 to 19.3)	--	--
	Pruritus	Both	59 (28 to 106)	9.9 (4.8 to 17.8)	--	--	106 (50 to 192)	10.6 (5.1 to 19.2)	--	--	80 (65.2 to 97.6)	6.9 (1.3 to 12.6)	--	--
		Female	31 (14 to 57)	10.9 (5.1 to 19.6)	--	--	57 (27 to 104)	11.6 (5.5 to 21)	--	--	82.1 (63.2 to 102.6)	6.5 (-0.7 to 14.4)	--	--
		Male	27 (13 to 50)	9 (4.3 to 16.5)	--	--	49 (23 to 88)	9.6 (4.6 to 17.4)	--	--	77.6 (60.4 to 98.2)	6.9 (-0.7 to 14.8)	--	--
	Urticaria	Both	506 (326 to 749)	58.1 (37.9 to 83.7)	--	--	582 (377 to 846)	58.4 (38.2 to 85.4)	--	--	14.9 (5.2 to 26.7)	0.6 (-5 to 6.5)	--	--
		Female	306 (199 to 447)	70.9 (46.4 to 102.2)	--	--	346 (227 to 502)	71.3 (47 to 104.2)	--	--	13 (2.4 to 26.8)	0.6 (-6.6 to 7.8)	--	--
		Male	200 (127 to 299)	45.9 (29.4 to 66)	--	--	236 (151 to 344)	46.1 (29.9 to 67.2)	--	--	17.8 (4.4 to 32.9)	0.5 (-7.8 to 9.4)	--	--
	Decubitus ulcer	Both	2 (1 to 4)	0.8 (0.5 to 1.6)	0 (0 to 0)	0 (0 to 0.1)	6 (4 to 10)	0.8 (0.5 to 1.4)	0 (0 to 0)	0 (0 to 0.1)	189.5 (132.3 to 244.7)	3.5 (-26 to 27.5)	254.1 (85.9 to 566.5)	-19.1 (-59 to 63.1)
		Female	1 (1 to 2)	0.7 (0.5 to 1.7)	0 (0 to 0)	0 (0 to 0.1)	3 (2 to 4)	0.7 (0.5 to 1.1)	0 (0 to 0)	0 (0 to 0)	196.6 (100.1 to 287.8)	-2.4 (-41.8 to 36.2)	181.6 (11.9 to 672.6)	-34.6 (-74.9 to 91.3)
		Male	1 (1 to 2)	0.8 (0.5 to 2)	0 (0 to 0)	0 (0 to 0.1)	3 (2 to 7)	0.9 (0.6 to 1.9)	0 (0 to 0)	0 (0 to 0.1)	184 (132.5 to 245)	10 (-14.8 to 37.7)	342.2 (147.6 to 774.2)	-1.1 (-44.7 to 108.5)
	Other skin and subcutaneous diseases	Both	226 (108 to 413)	41.4 (19.9 to 75)	0 (0 to 0)	0 (0 to 0)	420 (201 to 764)	43.6 (21 to 79.1)	0 (0 to 0)	0 (0 to 0)	85.9 (80.2 to 92.2)	5.4 (3 to 7.8)	212.6 (49.9 to 531.5)	-30.9 (-69.6 to 53.8)
		Female	110 (53 to 203)	41.9 (20 to 76.3)	0 (0 to 0)	0 (0 to 0)	211 (100 to 386)	44.2 (21.1 to 80.4)	0 (0 to 0)	0 (0 to 0)	90.7 (83.4 to 98.6)	5.5 (2 to 9)	219.6 (19.7 to 805.6)	-29.9 (-74.9 to 123.9)
		Male	115 (55 to 213)	40.9 (19.7 to 74.3)	0 (0 to 0)	0 (0 to 0)	209 (100 to 377)	43 (20.7 to 77.6)	0 (0 to 0)	0 (0 to 0)	81.2 (74.7 to 89.3)	5.3 (2.1 to 8.8)	201.3 (52.4 to 577.2)	-28 (-64 to 71.6)
	Sense organ diseases	Both	3960 (2792 to 5365)	1063.5 (765.3 to 1420.7)	--	--	7115 (5017 to 9743)	841.4 (596.6 to 1141.7)	--	--	79.7 (68.7 to 90.8)	-20.9 (-23.8 to -17.5)	--	--
		Female	1886 (1338 to 2537)	1066 (770 to 1414.8)	--	--	3584 (2532 to 4872)	842 (600.2 to 1132.4)	--	--	90 (78.3 to 102.1)	-21 (-24.3 to -17.3)	--	--
		Male	2074 (1468 to 2819)	1059.6 (759.2 to 1419.2)	--	--	3531 (2488 to 4855)	840.4 (595 to 1144.6)	--	--	70.2 (58.8 to 81.5)	-20.7 (-23.9 to -17)	--	--
	Blindness and vision loss	Both	1792 (1271 to 2428)	508.7 (364.7 to 681)	--	--	3124 (2214 to 4243)	381.4 (270.9 to 514.4)	--	--	74.3 (66.9 to 81.9)	-25 (-28.1 to -21.9)	--	--

Province	Cause	Sex	1990				2019				1990-2019 percent change (%)			
			DALYs		Death		DALYs		Death		DALYs		Death	
			Number	Rate	Number	Rate	Number	Rate	Number	Rate	Number	Rate	Number	Rate
		Female	896 (636 to 1217)	536.6 (385.6 to 716.7)	--	--	1672 (1193 to 2268)	402.8 (288.1 to 545.1)	--	--	86.6 (77.3 to 96)	-24.9 (-28.3 to -21.2)	--	--
		Male	896 (629 to 1218)	483.1 (345.5 to 648.9)	--	--	1452 (1026 to 1971)	358.8 (255.2 to 483.2)	--	--	61.9 (53.9 to 70.2)	-25.7 (-29.2 to -22.2)	--	--
	Age-related and other hearing loss	Both	1975 (1368 to 2724)	513.9 (367.3 to 712)	--	--	3596 (2498 to 5087)	417.4 (292.9 to 580.2)	--	--	82.1 (63.1 to 102.3)	-18.8 (-22.9 to -14.5)	--	--
		Female	890 (621 to 1221)	484.8 (348.3 to 666.1)	--	--	1699 (1185 to 2367)	393.1 (275.7 to 543.2)	--	--	91 (68.5 to 115.1)	-18.9 (-23.9 to -13.8)	--	--
		Male	1085 (747 to 1509)	539 (383.8 to 746.9)	--	--	1896 (1312 to 2683)	442.4 (310.3 to 618.6)	--	--	74.8 (55.6 to 93.9)	-17.9 (-22.7 to -13)	--	--
	Other sense organ diseases	Both	193 (117 to 290)	40.9 (24.4 to 63.6)	--	--	395 (235 to 609)	42.6 (25.6 to 66.5)	--	--	104.6 (86 to 125)	4.2 (-0.9 to 9.7)	--	--
		Female	100 (60 to 151)	44.5 (26.3 to 69.1)	--	--	213 (125 to 326)	46.1 (27.6 to 71.5)	--	--	112 (86.7 to 136.7)	3.5 (-3.3 to 11)	--	--
		Male	93 (56 to 142)	37.5 (22.6 to 58)	--	--	182 (109 to 284)	39.1 (23.4 to 61.5)	--	--	96.6 (74 to 120.1)	4.3 (-3.3 to 12.2)	--	--
	Musculoskeletal disorders	Both	7957 (5634 to 10672)	1822.2 (1289 to 2422.4)	1 (1 to 2)	0.3 (0.2 to 0.6)	19128 (13607 to 25452)	1898.5 (1362.5 to 2526.3)	2 (2 to 4)	0.3 (0.2 to 0.5)	140.4 (127.9 to 151.9)	4.2 (0.6 to 7.7)	156.5 (84.5 to 268.5)	-5.6 (-33.1 to 37.6)
		Female	4140 (2927 to 5544)	1981.7 (1406.2 to 2619.9)	1 (0 to 2)	0.4 (0.2 to 1)	10398 (7373 to 13764)	2094.3 (1498.8 to 2761.2)	2 (1 to 3)	0.3 (0.2 to 0.8)	151.2 (137.5 to 165.2)	5.7 (1.5 to 9.8)	174.3 (75.2 to 344)	-6 (-42 to 60.3)
		Male	3818 (2666 to 5170)	1675.3 (1172.5 to 2264.8)	0 (0 to 1)	0.2 (0.2 to 0.4)	8729 (6192 to 11673)	1704.3 (1209.9 to 2258.4)	1 (1 to 1)	0.2 (0.2 to 0.4)	128.6 (114.8 to 143.9)	1.7 (-3.2 to 6.7)	129.5 (51 to 247.3)	-5.6 (-34.9 to 39.7)
	Rheumatoid arthritis	Both	47 (32 to 65)	11 (7.7 to 15.1)	0 (0 to 0)	0.1 (0 to 0.2)	119 (83 to 163)	11.7 (8.3 to 15.9)	0 (0 to 1)	0 (0 to 0.1)	153.3 (115.5 to 193.1)	6.2 (-10.6 to 23)	137.3 (22.7 to 366.6)	-24.1 (-61.6 to 58.1)
		Female	32 (22 to 45)	15.7 (10.8 to 22)	0 (0 to 0)	0.1 (0 to 0.3)	83 (58 to 115)	16.4 (11.5 to 22.5)	0 (0 to 0)	0.1 (0 to 0.1)	156.7 (105.6 to 214.2)	4.6 (-17.2 to 28)	118.1 (2.8 to 479)	-34.5 (-69.3 to 80.4)
		Male	15 (10 to 20)	6.7 (4.5 to 9.1)	0 (0 to 0)	0 (0 to 0)	36 (24 to 50)	7.1 (4.8 to 9.8)	0 (0 to 0)	0 (0 to 0)	145.7 (106.5 to 189.8)	6.3 (-9.2 to 26)	199.9 (75.4 to 450.9)	10.6 (-33.8 to 91.1)
	Osteoarthritis	Both	543 (272 to 1067)	174.7 (88.5 to 341.5)	--	--	1614 (808 to 3171)	189.6 (94.8 to 369.4)	--	--	197.1 (186.3 to 209.1)	8.5 (5.1 to 12.4)	--	--
		Female	287 (144 to 562)	199.3 (101.3 to 390.4)	--	--	924 (465 to 1809)	215.3 (108.5 to 419.3)	--	--	222.6 (207.3 to 239.5)	8 (3.3 to 13.1)	--	--
		Male	256 (129 to 515)	152.6 (76.9 to 304.2)	--	--	689 (339 to 1379)	163 (80.4 to 326.5)	--	--	168.7 (153.7 to 183.8)	6.8 (1.6 to 12.3)	--	--
	Low back pain	Both	4730 (3315 to 6405)	1017.8 (713.2 to 1365.1)	--	--	9253 (6444 to 12397)	915.1 (645.3 to 1226.4)	--	--	95.6 (83.5 to 109.3)	-10.1 (-14 to -6.5)	--	--

Province	Cause	Sex	1990				2019				1990-2019 percent change (%)			
			DALYs		Death		DALYs		Death		DALYs		Death	
			Number	Rate	Number	Rate	Number	Rate	Number	Rate	Number	Rate	Number	Rate
		Female	2180 (1515 to 2959)	967.7 (681.7 to 1301.1)	--	--	4483 (3105 to 5995)	906.2 (633.8 to 1221.7)	--	--	105.6 (89 to 125.8)	-6.4 (-11.8 to -0.2)	--	--
		Male	2550 (1784 to 3470)	1062.6 (751.7 to 1423.6)	--	--	4771 (3327 to 6451)	922.9 (650.4 to 1241.3)	--	--	87.1 (72.6 to 102.4)	-13.1 (-18.5 to -7.9)	--	--
	Neck pain	Both	1705 (1108 to 2493)	402.8 (265.7 to 585.8)	--	--	4205 (2767 to 6191)	406.4 (267.2 to 588.5)	--	--	146.6 (131.3 to 160.6)	0.9 (-1.1 to 3)	--	--
		Female	1002 (658 to 1465)	493.8 (325.3 to 723.2)	--	--	2530 (1661 to 3708)	495.2 (326 to 719.8)	--	--	152.4 (135.7 to 167.3)	0.3 (-2.3 to 2.7)	--	--
		Male	703 (457 to 1028)	318.1 (208.8 to 460.7)	--	--	1675 (1085 to 2476)	319.6 (209.7 to 467)	--	--	138.4 (121 to 155.1)	0.5 (-2.5 to 3.8)	--	--
	Gout	Both	48 (30 to 70)	14.3 (8.9 to 20.4)	--	--	140 (86 to 204)	15.4 (9.5 to 22.5)	--	--	191.5 (149.9 to 242.2)	7.8 (-5.9 to 25.4)	--	--
		Female	10 (6 to 14)	6.2 (3.8 to 9.1)	--	--	32 (19 to 48)	7.1 (4.3 to 10.4)	--	--	230.6 (159.1 to 321.6)	14.6 (-9.5 to 43.7)	--	--
		Male	38 (23 to 56)	21.8 (13.6 to 31.3)	--	--	108 (67 to 159)	23.9 (14.9 to 34.8)	--	--	181.7 (137.1 to 242.3)	9.5 (-6.5 to 30)	--	--
	Other musculoskeletal disorders	Both	884 (543 to 1294)	201.7 (126 to 295.8)	1 (1 to 2)	0.2 (0.2 to 0.5)	3796 (2538 to 5340)	360.3 (240.1 to 501.1)	2 (1 to 4)	0.2 (0.2 to 0.4)	329.6 (277.4 to 405.1)	78.7 (57.1 to 109.8)	160 (89.8 to 269)	-1.1 (-29.2 to 39)
		Female	628 (408 to 883)	299 (194.1 to 418.4)	0 (0 to 1)	0.3 (0.1 to 0.7)	2346 (1575 to 3272)	454.1 (308.4 to 630.2)	1 (1 to 3)	0.3 (0.2 to 0.6)	273.3 (229.1 to 327.4)	51.9 (33.6 to 74.6)	187.9 (87.9 to 361)	3.4 (-34.7 to 68.2)
		Male	255 (125 to 427)	113.6 (54 to 189.6)	0 (0 to 1)	0.2 (0.1 to 0.4)	1450 (933 to 2132)	268 (171.6 to 385.9)	1 (1 to 1)	0.2 (0.1 to 0.3)	468.1 (342.5 to 755.7)	135.9 (83.5 to 251.2)	122.6 (46.5 to 235.4)	-7.6 (-35.1 to 36.8)
	Other non-communicable diseases	Both	40789 (27298 to 53991)	4140.6 (2932.1 to 5345.3)	380 (232 to 533)	32.7 (20.3 to 45.4)	18451 (13957 to 23941)	1863.3 (1420.7 to 2409.2)	69 (52 to 93)	7.9 (5.9 to 10.8)	-54.8 (-70.1 to -27.2)	-55 (-68.3 to -33.3)	-82 (-89.2 to -63.1)	-75.8 (-85.3 to -51.3)
		Female	20818 (14014 to 27662)	4660.7 (3346.1 to 5944.2)	177 (104 to 256)	31.2 (18.9 to 44.9)	12306 (9128 to 16272)	2437.4 (1827 to 3205.7)	32 (23 to 44)	7.4 (5.4 to 10.4)	-40.9 (-61.3 to -9)	-47.7 (-62.4 to -26.9)	-82.1 (-89.8 to -61.8)	-76.2 (-86.2 to -50.4)
		Male	19971 (11974 to 28210)	3638.4 (2295.9 to 5000.4)	203 (113 to 296)	34.1 (19.6 to 49.2)	6145 (4703 to 8276)	1316.7 (1006.5 to 1753.7)	37 (27 to 53)	8.4 (6.3 to 12)	-69.2 (-80.7 to -41)	-63.8 (-76.4 to -34.8)	-81.9 (-89.4 to -57.9)	-75.4 (-85.4 to -44.3)
	Congenital birth defects	Both	31852 (18677 to 45585)	2670 (1573.6 to 3805.8)	352 (203 to 508)	29.4 (17 to 42.3)	5564 (4031 to 7821)	628 (452.3 to 891.5)	54 (38 to 80)	6.2 (4.3 to 9.1)	-82.5 (-90.1 to -61.6)	-76.5 (-86.7 to -48.3)	-84.5 (-91.5 to -64)	-78.8 (-88.4 to -51)
		Female	14715 (8161 to 21702)	2522.7 (1413.3 to 3706.3)	163 (89 to 243)	27.9 (15.2 to 41.4)	2565 (1827 to 3598)	594.2 (419.1 to 841.3)	26 (17 to 38)	6 (4.1 to 8.9)	-82.6 (-90.4 to -58.9)	-76.4 (-87.1 to -44.5)	-84.3 (-91.7 to -61.3)	-78.5 (-88.7 to -47.1)
		Male	17138 (9109 to 25307)	2810.5 (1510.2 to 4137.5)	189 (97 to 281)	30.9 (16 to 45.8)	2999 (2153 to 4425)	660.2 (471.1 to 981.4)	29 (20 to 45)	6.4 (4.4 to 10.1)	-82.5 (-90.2 to -55.5)	-76.5 (-86.8 to -40.4)	-84.7 (-91.9 to -58.1)	-79.1 (-88.9 to -43)
	Urinary diseases and male infertility	Both	136 (98 to 189)	26.9 (18.6 to 37.8)	1 (0 to 1)	0.2 (0.1 to 0.6)	241 (161 to 355)	25.3 (17.1 to 36.3)	1 (1 to 3)	0.1 (0.1 to 0.4)	77.5 (56 to 99)	-5.9 (-15.5 to 1.8)	63.1 (13.6 to 155.4)	-15.5 (-46.6 to 28.9)

Province	Cause	Sex	1990				2019				1990-2019 percent change (%)			
			DALYs		Death		DALYs		Death		DALYs		Death	
			Number	Rate	Number	Rate	Number	Rate	Number	Rate	Number	Rate	Number	Rate
		Female	46 (34 to 60)	14.2 (10.2 to 21.4)	0 (0 to 1)	0.2 (0.1 to 0.7)	63 (45 to 86)	12.7 (9.2 to 17.5)	1 (0 to 1)	0.1 (0.1 to 0.4)	37.1 (11.5 to 66.9)	-10.6 (-31.8 to 7.7)	64 (3.6 to 157.6)	-22.4 (-60.9 to 39.1)
		Male	90 (60 to 135)	38.3 (24.7 to 56.7)	0 (0 to 1)	0.1 (0.1 to 0.6)	178 (112 to 274)	38.4 (24.6 to 57.1)	0 (0 to 2)	0.1 (0.1 to 0.5)	98 (74.9 to 121.1)	0.1 (-8.7 to 8.5)	62.1 (4.6 to 171.1)	-4.7 (-29.2 to 41.1)
	Gynecological diseases	Female	2759 (1884 to 3832)	1103.1 (752.3 to 1536.7)	0 (0 to 0)	0 (0 to 0)	5901 (4029 to 8186)	1050.9 (718.3 to 1456.4)	0 (0 to 0)	0 (0 to 0.1)	113.9 (101.9 to 126.7)	-4.7 (-7.9 to -1.8)	432.8 (-52.7 to 2033.7)	114 (-78.7 to 712.8)
	Hemoglobinopathies and hemolytic anemias	Both	1586 (1180 to 2111)	183.3 (134.9 to 244.6)	8 (6 to 11)	1 (0.8 to 1.2)	719 (489 to 1068)	73.4 (50.5 to 108)	3 (3 to 4)	0.4 (0.3 to 0.4)	-54.6 (-65.9 to -39.1)	-59.9 (-68.9 to -48.6)	-60.5 (-73.2 to -40.8)	-61.5 (-71.9 to -46.8)
		Female	925 (643 to 1282)	232.1 (160.1 to 324.4)	4 (3 to 6)	1 (0.7 to 1.3)	441 (287 to 675)	90 (59.2 to 135.6)	2 (1 to 2)	0.4 (0.3 to 0.5)	-52.3 (-66.9 to -30.7)	-61.2 (-72.3 to -47.6)	-61.6 (-78.4 to -29.5)	-63.5 (-77.4 to -39.6)
		Male	661 (477 to 895)	135.6 (100.9 to 180.9)	4 (3 to 6)	0.9 (0.7 to 1.2)	278 (187 to 417)	57.3 (38.9 to 85.3)	2 (1 to 2)	0.4 (0.3 to 0.5)	-57.9 (-71.5 to -38.8)	-57.7 (-70.2 to -41.3)	-59.5 (-72.2 to -38.5)	-59.2 (-70.4 to -42.2)
	Endocrine, metabolic, blood, and immune disorders	Both	1916 (1341 to 2704)	303.3 (213.1 to 416)	6 (3 to 12)	1.1 (0.7 to 2.1)	2918 (2001 to 3983)	296.8 (204.7 to 405.4)	8 (6 to 13)	1 (0.8 to 1.6)	52.3 (15.5 to 77.9)	-2.1 (-16.4 to 7.3)	37.4 (-35 to 125.4)	-4.1 (-43.4 to 55.7)
		Female	1153 (794 to 1631)	382.2 (264.7 to 529.2)	3 (1 to 6)	1 (0.6 to 2.4)	1827 (1226 to 2510)	372.2 (253.9 to 515.5)	3 (2 to 6)	0.8 (0.6 to 1.4)	58.4 (25 to 83.3)	-2.6 (-15.7 to 8.5)	21 (-44.8 to 134.4)	-25.7 (-56.5 to 37.1)
		Male	762 (512 to 1133)	228.3 (157.8 to 321.7)	3 (2 to 7)	1.1 (0.6 to 2.5)	1091 (763 to 1498)	224.8 (157.3 to 305.9)	5 (4 to 9)	1.3 (0.9 to 2.4)	43.1 (-0.9 to 77.6)	-1.6 (-21.2 to 12)	50.2 (-25.3 to 177.3)	18.1 (-31 to 99.4)
	Oral disorders	Both	1384 (821 to 2163)	320.6 (190.4 to 499.9)	--	--	2998 (1757 to 4797)	313.6 (185.9 to 493.8)	--	--	116.6 (106.5 to 127.3)	-2.2 (-5 to 0.3)	--	--
		Female	656 (386 to 1045)	310.7 (184.1 to 494.8)	--	--	1456 (853 to 2324)	304.8 (179.2 to 482.3)	--	--	122 (110.4 to 135.3)	-1.9 (-5.4 to 1.1)	--	--
		Male	728 (432 to 1123)	329.6 (197.6 to 514.3)	--	--	1542 (909 to 2492)	323.2 (192 to 508.1)	--	--	111.7 (101.3 to 122.6)	-1.9 (-5.5 to 1.5)	--	--
	Sudden infant death syndrome	Both	1156 (168 to 4032)	95.9 (14 to 334.5)	13 (2 to 46)	1.1 (0.2 to 3.8)	109 (36 to 240)	12.7 (4.1 to 27.9)	1 (0 to 3)	0.1 (0 to 0.3)	-90.5 (-97.4 to -33.6)	-86.7 (-96.4 to -7.2)	-90.5 (-97.4 to -33.6)	-86.8 (-96.4 to -7.2)
		Female	564 (81 to 2138)	95.8 (13.7 to 363)	6 (1 to 24)	1.1 (0.2 to 4.1)	53 (17 to 124)	12.7 (4 to 29.5)	1 (0 to 1)	0.1 (0 to 0.3)	-90.6 (-97.6 to -35.8)	-86.8 (-96.6 to -10)	-90.6 (-97.6 to -35.8)	-86.8 (-96.6 to -10)
		Male	592 (71 to 2106)	96.1 (11.6 to 341.9)	7 (1 to 24)	1.1 (0.1 to 3.9)	56 (16 to 127)	12.8 (3.7 to 28.8)	1 (0 to 1)	0.1 (0 to 0.3)	-90.5 (-97.4 to -28.8)	-86.7 (-96.4 to -0.7)	-90.5 (-97.4 to -28.8)	-86.7 (-96.4 to -0.7)
	**Injuries**	Both	48690 (39846 to 57757)	6612.6 (5452.4 to 7765.4)	672 (543 to 799)	109.4 (87 to 131)	27086 (23484 to 31350)	2648.2 (2289.7 to 3060.1)	453 (383 to 529)	47.1 (40 to 54.7)	-44.4 (-54.7 to -31.1)	-60 (-67.2 to -50.6)	-32.6 (-47 to -13.8)	-57 (-66.1 to -44.9)
		Female	15875 (12483 to 19900)	4028.3 (3245.3 to 4957.5)	198 (151 to 250)	59.7 (47 to 74.6)	7211 (6063 to 8406)	1441.5 (1218.8 to 1672.4)	104 (85 to 126)	22 (17.9 to 26.7)	-54.6 (-65.6 to -40.3)	-64.2 (-72 to -54.2)	-47.5 (-62.1 to -25.8)	-63.2 (-73 to -49.5)
		Male	32816 (26703 to 38921)	9064.1 (7387.7 to 10882.7)	474 (374 to 575)	156.6 (121 to 196.8)	19876 (16774 to 23413)	3823 (3227.2 to 4492.8)	349 (283 to 420)	72.1 (58.9 to 85.7)	-39.4 (-51.6 to -23.7)	-57.8 (-66.4 to -46.8)	-26.4 (-43.1 to -3.3)	-53.9 (-65.2 to -38.5)
	Transport injuries	Both	24242 (18677 to 29716)	3294.4 (2540 to 4006.2)	377 (288 to 460)	63.4 (47.1 to 77.9)	13740 (11111 to 16287)	1348.3 (1093.7 to 1590.2)	276 (214 to 330)	28.5 (22.3 to 33.9)	-43.3 (-55.5 to -26)	-59.1 (-67.8 to -46.9)	-26.9 (-43.1 to -3.1)	-55.1 (-65.5 to -41.5)
		Female	7391 (5391 to 9630)	1801 (1388.4 to 2278.8)	102 (75 to 133)	30.8 (23.4 to 38.8)	3149 (2539 to 3716)	633 (516.1 to 742.4)	58 (46 to 71)	12.2 (9.6 to 14.9)	-57.4 (-69.6 to -40.1)	-64.8 (-73.6 to -52.2)	-43.1 (-58.9 to -18.5)	-60.4 (-70.6 to -45.3)

Province	Cause	Sex	1990				2019				1990-2019 percent change (%)			
			DALYs		Death		DALYs		Death		DALYs		Death	
			Number	Rate	Number	Rate	Number	Rate	Number	Rate	Number	Rate	Number	Rate
		Male	16851 (12720 to 20869)	4707.9 (3473.2 to 5906.7)	275 (200 to 345)	94.2 (65.2 to 120.7)	10590 (8140 to 12970)	2044.2 (1582 to 2489.7)	217 (159 to 271)	44.7 (32.8 to 55.2)	-37.2 (-52.1 to -15)	-56.6 (-66.9 to -41.4)	-20.8 (-40.8 to 9.6)	-52.6 (-65.7 to -34)
	Road injuries	Both	23790 (18295 to 29202)	3227.3 (2486.7 to 3927.4)	370 (282 to 452)	62.2 (46.2 to 76.5)	13469 (10889 to 15983)	1321.9 (1072.5 to 1561.3)	272 (211 to 326)	28 (22 to 33.4)	-43.4 (-55.6 to -26)	-59 (-67.8 to -46.8)	-26.7 (-42.9 to -2.7)	-54.9 (-65.4 to -41.3)
		Female	7282 (5294 to 9497)	1772.1 (1364.8 to 2245.3)	101 (74 to 132)	30.4 (23.1 to 38.4)	3090 (2485 to 3652)	621.1 (505.5 to 729)	58 (45 to 70)	12.1 (9.5 to 14.7)	-57.6 (-69.8 to -40.4)	-65 (-73.7 to -52.2)	-42.9 (-58.8 to -18.2)	-60.3 (-70.5 to -45.2)
		Male	16508 (12424 to 20442)	4604.6 (3394.9 to 5769.4)	269 (197 to 340)	92.2 (63.9 to 119)	10379 (7975 to 12737)	2003.8 (1546.5 to 2445)	214 (156 to 266)	44 (32.1 to 54.4)	-37.1 (-52.2 to -15)	-56.5 (-66.8 to -41.2)	-20.5 (-40.5 to 10.1)	-52.3 (-65.5 to -33.6)
	Other transport injuries	Both	452 (336 to 595)	67.2 (50.9 to 86.8)	7 (5 to 9)	1.2 (0.8 to 1.6)	270 (220 to 348)	26.4 (21.5 to 34.3)	4 (3 to 6)	0.4 (0.3 to 0.6)	-40.1 (-54.7 to -17)	-60.7 (-69.4 to -45.9)	-39.7 (-55.7 to -8.6)	-65.8 (-75.6 to -47.9)
		Female	109 (77 to 160)	28.9 (21.9 to 40.4)	1 (1 to 2)	0.4 (0.3 to 0.6)	59 (46 to 75)	12 (9.3 to 15.1)	1 (0 to 1)	0.1 (0.1 to 0.2)	-45.5 (-63 to -21.6)	-58.5 (-69.6 to -43.7)	-58.2 (-73.1 to -35.4)	-71.6 (-80.8 to -55.7)
		Male	343 (242 to 472)	103.3 (73.3 to 138.6)	5 (4 to 7)	2 (1.3 to 2.8)	211 (165 to 278)	40.4 (31.9 to 54.5)	3 (2 to 5)	0.7 (0.5 to 1)	-38.4 (-56.6 to -7.4)	-60.9 (-71.4 to -43.1)	-35.2 (-55.4 to 2.7)	-63.9 (-75.2 to -41.6)
	Unintentional injuries	Both	16588 (13197 to 20520)	2097.6 (1739 to 2483.3)	205 (164 to 254)	31.3 (25.7 to 37.1)	8190 (6689 to 9990)	820.5 (671.1 to 995.4)	109 (89 to 134)	12 (9.9 to 14.8)	-50.6 (-62.4 to -35.5)	-60.9 (-68.9 to -51.3)	-47.1 (-61 to -26.3)	-61.6 (-70.6 to -49)
		Female	5733 (4180 to 7803)	1368.9 (1037.6 to 1777.1)	67 (48 to 93)	19.7 (14.6 to 25.8)	2265 (1771 to 2870)	470.6 (369.6 to 595.6)	25 (19 to 35)	5.8 (4.4 to 8)	-60.5 (-73.6 to -41)	-65.6 (-75.7 to -51.5)	-62.5 (-77.9 to -35.8)	-70.4 (-81.4 to -52.6)
		Male	10856 (8610 to 13301)	2788.5 (2279.8 to 3330.3)	138 (108 to 170)	42.4 (33.8 to 52.6)	5925 (4839 to 7243)	1162.1 (951.1 to 1414.1)	83 (67 to 106)	18.3 (14.9 to 23.2)	-45.4 (-57.8 to -28.9)	-58.3 (-66.6 to -47.9)	-39.5 (-55.7 to -14.9)	-56.8 (-67.6 to -40.8)
	Falls	Both	3075 (2273 to 3989)	517.7 (399.3 to 646.6)	33 (23 to 43)	7.3 (5.2 to 9.2)	3108 (2412 to 3963)	316.8 (244.6 to 402.8)	37 (26 to 48)	4.5 (3.2 to 5.7)	1.1 (-23.5 to 28.4)	-38.8 (-49.6 to -26.7)	14 (-21.7 to 70)	-38.6 (-55.7 to -10.9)
		Female	741 (498 to 1065)	249.7 (190.9 to 313.1)	7 (5 to 10)	3.7 (2.6 to 5.5)	698 (531 to 925)	150.3 (115.3 to 197.5)	7 (5 to 10)	1.8 (1.4 to 2.6)	-5.9 (-36.8 to 37.9)	-39.8 (-51.7 to -27.3)	1.1 (-36.4 to 67.7)	-49.4 (-63.9 to -24.6)
		Male	2334 (1567 to 3153)	769.2 (565.3 to 988.4)	26 (15 to 36)	10.8 (7 to 14.5)	2411 (1820 to 3070)	480.6 (359.4 to 609.6)	30 (19 to 40)	7.2 (4.4 to 9.7)	3.3 (-25.2 to 48.8)	-37.5 (-50.7 to -20.1)	17.6 (-24.5 to 104.7)	-33.3 (-55.1 to 2.6)
	Drowning	Both	3185 (1885 to 4414)	304.2 (178.2 to 409)	41 (24 to 56)	4.4 (2.5 to 5.7)	642 (420 to 802)	65 (42.7 to 81.5)	11 (7 to 14)	1.1 (0.7 to 1.4)	-79.8 (-86 to -69.6)	-78.6 (-84.5 to -68.3)	-73.2 (-81 to -60.1)	-74.1 (-81.2 to -62.2)
		Female	936 (582 to 1441)	164.6 (106 to 244.9)	11 (7 to 17)	2.1 (1.4 to 3.1)	88 (71 to 110)	18.4 (14.8 to 23.2)	1 (1 to 2)	0.3 (0.2 to 0.4)	-90.6 (-94.5 to -83.7)	-88.8 (-93.1 to -80.8)	-88.4 (-92.9 to -80.2)	-86.7 (-91.5 to -78.2)
		Male	2250 (998 to 3259)	438.3 (205.4 to 608.9)	30 (14 to 42)	6.6 (3.1 to 9.2)	555 (330 to 714)	109.8 (65.7 to 141.6)	10 (6 to 12)	2 (1.2 to 2.5)	-75.3 (-83.4 to -59.2)	-74.9 (-83 to -59.6)	-67.5 (-78.1 to -47.5)	-70 (-79.1 to -53)
	Fire, heat, and hot substances	Both	2699 (1551 to 4371)	328.2 (200.1 to 498.2)	38 (22 to 60)	5.9 (3.7 to 8.5)	557 (319 to 999)	55.3 (31.4 to 101.3)	11 (6 to 23)	1.2 (0.6 to 2.5)	-79.4 (-92 to -39.5)	-83.2 (-93.2 to -56.4)	-70.5 (-89.5 to -7.6)	-79 (-92 to -46.9)
		Female	1252 (327 to 2488)	313.6 (114 to 578.5)	17 (5 to 34)	5.5 (2.6 to 9.6)	260 (123 to 583)	52.7 (24.8 to 118.1)	5 (2 to 13)	1.1 (0.4 to 2.8)	-79.2 (-94.6 to 39.6)	-83.2 (-95.4 to -17.1)	-71.3 (-94 to 96.6)	-80 (-95.4 to -18.2)
		Male	1447 (678 to 2144)	342 (172.6 to 478.2)	21 (10 to 30)	6.2 (3.4 to 8.5)	297 (186 to 602)	57.8 (35.9 to 119.5)	6 (4 to 14)	1.4 (0.8 to 3.1)	-79.5 (-90.2 to -7.3)	-83.1 (-91.5 to -37.7)	-69.8 (-85.8 to 29.3)	-77.9 (-89.2 to -29.3)
	Poisonings	Both	977 (647 to 1454)	103.3 (73.5 to 149.6)	13 (9 to 19)	1.6 (1.2 to 2.3)	355 (284 to 447)	34 (27.2 to 42.8)	7 (6 to 9)	0.7 (0.6 to 0.9)	-63.6 (-76.1 to -42.2)	-67.1 (-77.7 to -49.8)	-46.5 (-64.2 to -17)	-57.8 (-70.3 to -38.4)
		Female	338 (211 to 544)	67.1 (45.1 to 102)	4 (3 to 7)	1 (0.7 to 1.4)	92 (73 to 118)	18.4 (14.7 to 23.2)	2 (1 to 2)	0.3 (0.3 to 0.4)	-72.6 (-84.2 to -54.1)	-72.5 (-83.5 to -56.4)	-61.5 (-77.4 to -36.3)	-65.2 (-77.6 to -44.9)
		Male	639 (392 to 1033)	138 (90.4 to 213.8)	9 (6 to 14)	2.3 (1.6 to 3.6)	263 (198 to 349)	48.9 (37.1 to 64.6)	5 (4 to 7)	1 (0.8 to 1.3)	-58.9 (-75.1 to -25.4)	-64.6 (-77.9 to -41.1)	-39.3 (-62.6 to 6)	-54.4 (-70.5 to -25.3)

Province	Cause	Sex	1990				2019				1990-2019 percent change (%)			
			DALYs		Death		DALYs		Death		DALYs		Death	
			Number	Rate	Number	Rate	Number	Rate	Number	Rate	Number	Rate	Number	Rate
	Exposure to mechanical forces	Both	1574 (1026 to 2702)	238.6 (159.9 to 404.9)	19 (11 to 38)	3.2 (1.9 to 6.7)	1255 (919 to 1778)	122.6 (90.4 to 171)	14 (9 to 25)	1.4 (0.9 to 2.5)	-20.3 (-46.4 to 15.2)	-48.6 (-62.5 to -30)	-26 (-53.8 to 20.1)	-55.7 (-69.4 to -29.8)
		Female	416 (250 to 782)	114.8 (78.9 to 177.7)	4 (2 to 8)	1.3 (0.8 to 2.6)	324 (235 to 451)	65.7 (48.2 to 91.8)	2 (2 to 4)	0.5 (0.4 to 1)	-22.2 (-60.2 to 32.4)	-42.8 (-64.5 to -17.9)	-40 (-73.1 to 28.7)	-58.4 (-76.4 to -28.9)
		Male	1158 (722 to 2170)	356.2 (228.8 to 672)	15 (8 to 33)	5 (2.8 to 12)	931 (648 to 1441)	178 (125.5 to 271.6)	11 (7 to 22)	2.3 (1.4 to 4.4)	-19.6 (-44.9 to 21.4)	-50 (-64 to -29.1)	-22 (-52 to 34.5)	-54.4 (-70 to -21.7)
	Adverse effects of medical treatment	Both	935 (564 to 1229)	135.3 (81.5 to 183.6)	16 (10 to 22)	3.4 (2 to 5.7)	369 (281 to 551)	38.2 (29.6 to 58.9)	10 (7 to 16)	1.1 (0.8 to 1.9)	-60.6 (-73 to -39)	-71.7 (-80.9 to -57.4)	-39.5 (-61.5 to -5.5)	-66.7 (-79.5 to -45.4)
		Female	368 (204 to 540)	102.1 (59.3 to 183.1)	6 (3 to 11)	2.5 (1.3 to 7)	136 (96 to 222)	28.2 (20.4 to 47)	3 (2 to 6)	0.8 (0.5 to 1.4)	-63.2 (-75.9 to -38.5)	-72.4 (-81.3 to -57.8)	-43.8 (-64.4 to -10.1)	-69.4 (-82.2 to -47.4)
		Male	567 (310 to 791)	166.2 (84.9 to 251.3)	10 (5 to 16)	4.3 (2.2 to 8.4)	233 (165 to 412)	48.4 (33.9 to 89.5)	6 (4 to 13)	1.5 (1 to 3.2)	-58.9 (-75.4 to -31.8)	-70.9 (-82.8 to -49.3)	-37 (-64.8 to 13.5)	-64.4 (-79.8 to -34.5)
	Animal contact	Both	309 (184 to 432)	32.9 (21.3 to 42.9)	4 (2 to 5)	0.5 (0.3 to 0.6)	118 (92 to 151)	12.1 (9.4 to 15.4)	1 (1 to 2)	0.2 (0.1 to 0.2)	-61.7 (-74.4 to -35.9)	-63.3 (-74.2 to -44.3)	-62.9 (-76.2 to -33.4)	-66.3 (-76.9 to -46)
		Female	144 (55 to 231)	30.6 (14.9 to 45.3)	2 (0 to 3)	0.4 (0.2 to 0.6)	55 (40 to 73)	11.5 (8.2 to 15.1)	0 (0 to 1)	0.1 (0.1 to 0.2)	-61.4 (-79 to -9.9)	-62.6 (-77.3 to -30.5)	-69.7 (-83.6 to -23.8)	-71.3 (-83.3 to -39.4)
		Male	165 (88 to 249)	35.1 (21.2 to 49.4)	2 (1 to 3)	0.5 (0.3 to 0.7)	63 (49 to 80)	12.8 (9.8 to 16.3)	1 (1 to 1)	0.2 (0.1 to 0.3)	-62 (-76.7 to -27.7)	-63.6 (-75.9 to -39.8)	-57.4 (-74.6 to -16.4)	-62.2 (-75.8 to -33.1)
	Foreign body	Both	2046 (1261 to 3109)	187.7 (121.4 to 274.6)	23 (14 to 35)	2.2 (1.5 to 3.2)	541 (423 to 669)	56.3 (43.7 to 70)	7 (5 to 8)	0.7 (0.6 to 0.9)	-73.5 (-84.6 to -55.7)	-70 (-81.8 to -53.5)	-71.2 (-82.9 to -53.3)	-66.7 (-79.4 to -49)
		Female	1032 (570 to 1747)	188.5 (109.7 to 311.2)	12 (6 to 20)	2.2 (1.3 to 3.6)	211 (158 to 277)	45.3 (33.7 to 60.1)	2 (2 to 3)	0.5 (0.4 to 0.7)	-79.6 (-89.7 to -58.5)	-76 (-87.6 to -54)	-79.5 (-89.8 to -57.8)	-75.1 (-87.1 to -52.6)
		Male	1014 (420 to 1665)	186.8 (92 to 295.5)	11 (5 to 19)	2.3 (1.2 to 3.5)	331 (239 to 420)	66.9 (48.1 to 85.6)	4 (3 to 5)	0.9 (0.7 to 1.2)	-67.4 (-81.7 to -37.6)	-64.2 (-79.1 to -39.8)	-62.8 (-79.7 to -27.2)	-58.6 (-75.9 to -31.3)
	Environmental heat and cold exposure	Both	94 (69 to 134)	16.1 (11.8 to 23.1)	1 (0 to 1)	0.1 (0.1 to 0.3)	120 (85 to 164)	11.8 (8.4 to 16.1)	1 (0 to 1)	0.1 (0 to 0.1)	27.6 (-5.6 to 49.6)	-26.7 (-42.6 to -15.8)	-7.6 (-39.9 to 46.8)	-56.8 (-70.3 to -32.3)
		Female	39 (27 to 57)	14 (9.8 to 19.6)	0 (0 to 0)	0.1 (0 to 0.2)	56 (39 to 79)	11.2 (7.7 to 15.7)	0 (0 to 0)	0 (0 to 0.1)	41.9 (6.2 to 63.9)	-20.2 (-36 to -10.5)	-16 (-47.9 to 57.9)	-60.6 (-78.4 to -17.6)
		Male	54 (40 to 84)	18 (13.3 to 28.3)	0 (0 to 1)	0.2 (0.1 to 0.5)	64 (47 to 88)	12.4 (9 to 16.9)	0 (0 to 1)	0.1 (0.1 to 0.2)	17.3 (-15.9 to 42.3)	-31.6 (-49.9 to -17.3)	-5.2 (-40.6 to 53.4)	-55.4 (-70.6 to -28.5)
	Exposure to forces of nature	Both	22 (19 to 25)	3.3 (2.8 to 3.7)	0 (0 to 0)	0 (0 to 0)	17 (13 to 22)	1.6 (1.2 to 2.1)	0 (0 to 0)	0 (0 to 0)	-23.9 (-37.3 to -8.6)	-50.1 (-58.2 to -41.1)	-77.7 (-77.7 to -77.7)	-86 (-86 to -86)
		Female	8 (7 to 9)	2.4 (2.1 to 2.8)	0 (0 to 0)	0 (0 to 0)	6 (4 to 8)	1.1 (0.9 to 1.5)	0 (0 to 0)	0 (0 to 0)	-29.2 (-42.5 to -13.6)	-52.2 (-60.1 to -42.3)	-78.2 (-78.2 to -78.2)	-86.2 (-86.2 to -86.2)
		Male	14 (12 to 16)	4.1 (3.5 to 4.7)	0 (0 to 0)	0.1 (0 to 0.1)	11 (8 to 14)	2.1 (1.6 to 2.8)	0 (0 to 0)	0 (0 to 0)	-20.7 (-34.4 to -5.7)	-48.6 (-57 to -39.9)	-77.3 (-77.3 to -77.3)	-85.8 (-85.8 to -85.8)
	Other unintentional injuries	Both	1671 (1245 to 2271)	230.3 (175.3 to 307.8)	19 (13 to 27)	2.7 (1.9 to 3.8)	1107 (842 to 1443)	106.9 (81.4 to 140)	10 (7 to 13)	1 (0.7 to 1.3)	-33.7 (-55.6 to -9.6)	-53.6 (-68.5 to -39.7)	-44.8 (-69.2 to -14.5)	-62.4 (-78.4 to -43)
		Female	458 (322 to 635)	121.5 (89.5 to 162.2)	4 (2 to 6)	1 (0.7 to 1.4)	339 (234 to 483)	67.9 (47.1 to 96.5)	1 (1 to 2)	0.3 (0.2 to 0.4)	-25.8 (-52.5 to 9)	-44.1 (-59.9 to -25.9)	-66.4 (-81.7 to -32.5)	-71.9 (-83.7 to -48.3)
		Male	1214 (866 to 1745)	334.6 (244.1 to 476.6)	15 (10 to 22)	4.2 (2.9 to 6.5)	768 (581 to 988)	144.5 (108.5 to 184.2)	9 (6 to 12)	1.7 (1.2 to 2.2)	-36.7 (-61.7 to -8.8)	-56.8 (-73.2 to -40.3)	-38.8 (-68.5 to -0.9)	-60 (-79.3 to -34.9)
	Self-harm and interpersonal violence	Both	7861 (6487 to 9380)	1220.6 (1012.9 to 1464.1)	90 (70 to 107)	14.8 (11.7 to 17.7)	5157 (4386 to 6112)	479.4 (408.2 to 565.1)	69 (58 to 86)	6.6 (5.6 to 8.2)	-34.4 (-44.7 to -19.6)	-60.7 (-66.8 to -52.3)	-23.4 (-39.7 to 3.2)	-55.2 (-64.8 to -40.3)
		Female	2752 (1997 to 3643)	858.5 (650.1 to 1120.8)	28 (18 to 38)	9.3 (6.4 to 12.4)	1796 (1497 to 2186)	337.9 (283.8 to 409.1)	20 (16 to 25)	4 (3.2 to 4.9)	-34.7 (-49.2 to -12.8)	-60.6 (-69.1 to -49.2)	-27.2 (-50.6 to 14.3)	-56.9 (-70.1 to -36.7)

Province	Cause	Sex	1990				2019				1990-2019 percent change (%)			
			DALYs		Death		DALYs		Death		DALYs		Death	
			Number	Rate	Number	Rate	Number	Rate	Number	Rate	Number	Rate	Number	Rate
		Male	5109 (4271 to 6070)	1567.7 (1298.2 to 1890.5)	62 (50 to 76)	20 (15.7 to 24.9)	3360 (2740 to 4168)	616.7 (506.2 to 762.9)	49 (38 to 64)	9.2 (7.3 to 12)	-34.2 (-46.5 to -15.5)	-60.7 (-68.1 to -49.5)	-21.6 (-42.2 to 10.6)	-54.1 (-66.3 to -34)
	Self-harm	Both	2835 (1862 to 3593)	439.8 (308.5 to 562.2)	48 (32 to 61)	8.3 (6 to 10.6)	2285 (1873 to 2976)	215.2 (176.3 to 277.7)	44 (36 to 58)	4.2 (3.5 to 5.5)	-19.4 (-41.6 to 25.3)	-51.1 (-63.9 to -28.2)	-8.3 (-33.8 to 37.3)	-48.9 (-62.4 to -25.6)
		Female	1496 (874 to 2074)	454.5 (288.8 to 616.3)	24 (14 to 33)	8 (5.3 to 10.9)	944 (740 to 1195)	181.5 (142.9 to 227.7)	17 (13 to 21)	3.3 (2.6 to 4.2)	-36.9 (-58 to 6.7)	-60.1 (-72.8 to -37.6)	-29 (-53.1 to 17.2)	-58.4 (-71.9 to -36.1)
		Male	1339 (864 to 1848)	424.7 (290.8 to 593.8)	24 (16 to 33)	8.5 (5.8 to 12)	1341 (997 to 1985)	248 (182.7 to 359.9)	27 (20 to 40)	5.1 (3.8 to 7.6)	0.1 (-33.9 to 78.9)	-41.6 (-60.5 to 2.3)	12.6 (-25.1 to 104)	-39.8 (-58.9 to 6.9)
	Interpersonal violence	Both	1344 (973 to 1707)	208.3 (153.5 to 262.7)	18 (12 to 24)	3.1 (2.1 to 4.2)	1372 (1115 to 1685)	129.1 (105.7 to 158.2)	17 (13 to 23)	1.7 (1.3 to 2.2)	2 (-22.5 to 40.6)	-38 (-52.1 to -16.4)	-4.4 (-31.7 to 46.3)	-47.3 (-62.5 to -21)
		Female	373 (274 to 494)	106.7 (81.9 to 137.7)	4 (2 to 5)	1.1 (0.7 to 1.5)	384 (299 to 481)	73.6 (57.8 to 91.9)	3 (2 to 4)	0.5 (0.4 to 0.7)	3 (-27.7 to 39.4)	-31.1 (-48.5 to -11.2)	-25.3 (-57.3 to 26)	-51.9 (-70.7 to -22.1)
		Male	972 (646 to 1292)	304.8 (202.9 to 402.9)	15 (9 to 20)	5.1 (3.1 to 7.1)	988 (775 to 1275)	182.8 (143.8 to 234.6)	15 (11 to 20)	2.8 (2 to 3.7)	1.7 (-27 to 52.5)	-40 (-56.4 to -12.8)	0.6 (-31.3 to 63)	-45.7 (-63.3 to -11.4)
	Conflict and terrorism	Both	3370 (2596 to 4554)	522.4 (394.3 to 705.8)	19 (18 to 21)	2.5 (2.3 to 2.8)	1033 (670 to 1644)	92.6 (61.1 to 146)	0 (0 to 0)	0 (0 to 0)	-69.4 (-75 to -62.9)	-82.3 (-85.7 to -78.5)	-98.1 (-98.1 to -98.1)	-98.5 (-98.5 to -98.5)
		Female	836 (505 to 1414)	282.5 (177.4 to 451.7)	0 (0 to 0)	0 (0 to 0)	415 (247 to 749)	72.4 (43.5 to 128)	0 (0 to 0)	0 (0 to 0)	-50.3 (-57.6 to -43.2)	-74.4 (-79.4 to -69.1)	68.1 (68.1 to 68.1)	11 (11 to 11)
		Male	2533 (2067 to 3211)	753.9 (607.6 to 961.4)	19 (18 to 21)	4.9 (4.5 to 5.4)	617 (417 to 927)	112.9 (76.7 to 169.1)	0 (0 to 0)	0.1 (0 to 0.1)	-75.6 (-80.6 to -69.9)	-85 (-87.9 to -81.5)	-98.6 (-98.6 to -98.6)	-98.9 (-98.9 to -98.9)
	Executions and police conflict	Both	312 (245 to 409)	50.2 (38.5 to 66.4)	5 (4 to 7)	0.9 (0.7 to 1.2)	468 (330 to 636)	42.5 (31.1 to 56.1)	8 (5 to 11)	0.7 (0.5 to 1)	50.1 (3.1 to 114.8)	-15.3 (-41.7 to 18.7)	57.7 (5.2 to 137.6)	-17.7 (-44.7 to 22.1)
		Female	47 (41 to 53)	14.8 (13 to 17.1)	1 (1 to 1)	0.2 (0.2 to 0.2)	53 (46 to 62)	10.4 (9.1 to 12.1)	1 (1 to 1)	0.2 (0.1 to 0.2)	13.2 (6.9 to 20.8)	-29.7 (-34.1 to -25)	21.9 (19.8 to 24.3)	-31.5 (-32.6 to -30.4)
		Male	265 (200 to 362)	84.3 (61.9 to 115.4)	4 (3 to 6)	1.5 (1.1 to 2.1)	415 (277 to 582)	73 (50.9 to 99.2)	7 (4 to 10)	1.2 (0.8 to 1.7)	56.6 (1.4 to 135.1)	-13.4 (-43.4 to 27.1)	62.9 (3.5 to 155.3)	-15.8 (-46.1 to 30.8)
**East Azarbayejan**	**All causes**	Both	1460157 (1311104 to 1639940)	47001.6 (42218.6 to 52329.9)	21347 (18924 to 23830)	1158.2 (1037.5 to 1293.4)	1039898 (903064 to 1182919)	26623 (23334.6 to 30112.2)	23650 (21110 to 26438)	756.7 (683.6 to 837.6)	-28.8 (-37.9 to -18.7)	-43.4 (-49.4 to -36.3)	10.8 (-5.5 to 29.6)	-34.7 (-44 to -24.4)
		Female	648505 (573323 to 733812)	42917.3 (37604.1 to 48636)	8805 (7651 to 10166)	1025.8 (887.7 to 1195.2)	479018 (404516 to 559535)	24817 (21161.9 to 28743.5)	10184 (8833 to 11730)	678.7 (596.1 to 771.6)	-26.1 (-36.7 to -14.8)	-42.2 (-49.6 to -34.3)	15.7 (-6.6 to 40.4)	-33.8 (-46.1 to -19.7)
		Male	811653 (718879 to 919291)	50731.4 (44476.2 to 57774.2)	12542 (10749 to 14570)	1278.3 (1073.4 to 1488.4)	560880 (481818 to 641198)	28412.3 (24603.4 to 32333.2)	13465 (11428 to 15771)	834.8 (719 to 965.2)	-30.9 (-41.9 to -18.5)	-44 (-52.4 to -34.3)	7.4 (-14.7 to 34.3)	-34.7 (-48.2 to -19.1)
	**Communicable, maternal, neonatal, and nutritional diseases**	Both	489358 (396294 to 606613)	10821.9 (8881.8 to 13262.3)	5565 (4461 to 6897)	144.4 (121.1 to 172.2)	72818 (62658 to 83439)	2115.1 (1815 to 2438)	1010 (880 to 1150)	34.6 (30.1 to 39.5)	-85.1 (-88.8 to -80.8)	-80.5 (-84.9 to -75)	-81.8 (-86.1 to -76.2)	-76.1 (-80.9 to -69.7)
		Female	227704 (184025 to 283074)	10335.3 (8458.1 to 12679.8)	2569 (2072 to 3191)	136.3 (112.2 to 164.7)	34075 (28690 to 40045)	2011.2 (1694.4 to 2362.5)	456 (381 to 543)	32.1 (26.9 to 38)	-85 (-88.8 to -80.4)	-80.5 (-85.5 to -74.7)	-82.2 (-86.8 to -75.9)	-76.4 (-82 to -69.5)
		Male	261654 (207898 to 329093)	11272.1 (9036.2 to 14073.8)	2996 (2359 to 3786)	151.9 (123.3 to 186.4)	38743 (32933 to 44822)	2215.2 (1884.6 to 2560.2)	554 (473 to 648)	37 (31.7 to 43.3)	-85.2 (-89.2 to -80.7)	-80.3 (-85.5 to -74.4)	-81.5 (-86.2 to -75.3)	-75.6 (-81 to -68.6)
	HIV/AIDS and sexually transmitted infections	Both	993 (517 to 1847)	27 (15.7 to 47)	8 (3 to 18)	0.2 (0.1 to 0.4)	1405 (955 to 2010)	33.7 (21.9 to 49)	15 (9 to 23)	0.4 (0.2 to 0.6)	41.5 (-20.6 to 149.7)	24.6 (-19.8 to 89.7)	80.1 (-19.8 to 364.9)	79.1 (-10.1 to 280.7)

Province	Cause	Sex	1990				2019				1990-2019 percent change (%)			
			DALYs		Death		DALYs		Death		DALYs		Death	
			Number	Rate	Number	Rate	Number	Rate	Number	Rate	Number	Rate	Number	Rate
		Female	506 (270 to 919)	29.7 (17.2 to 50.8)	4 (1 to 8)	0.2 (0.1 to 0.4)	791 (477 to 1200)	37.8 (22.9 to 57.2)	8 (4 to 14)	0.4 (0.2 to 0.7)	56.5 (-13.8 to 176.1)	27.5 (-18.9 to 106.7)	118.1 (-20.3 to 575.9)	118.7 (-7.3 to 439.5)
		Male	488 (238 to 944)	24.4 (13.7 to 43.6)	5 (2 to 10)	0.2 (0.1 to 0.5)	614 (450 to 849)	29.7 (20.3 to 43.1)	7 (5 to 10)	0.3 (0.2 to 0.5)	25.9 (-27 to 129.3)	21.4 (-22.8 to 79.3)	50.4 (-21.4 to 237.1)	47.8 (-13.2 to 175.1)
	HIV/AIDS	Both	73 (37 to 102)	2.6 (1.3 to 3.6)	1 (0 to 2)	0 (0 to 0.1)	666 (395 to 1013)	15 (8.7 to 23.1)	12 (6 to 19)	0.3 (0.1 to 0.4)	810.5 (353.8 to 1901.6)	484.7 (180.3 to 1224.3)	977.1 (370.2 to 3099.5)	506.9 (159.5 to 1760.9)
		Female	24 (12 to 34)	1.5 (0.8 to 2.2)	0 (0 to 1)	0 (0 to 0)	342 (147 to 601)	16 (6.8 to 27.9)	6 (2 to 12)	0.3 (0.1 to 0.5)	1338.1 (448.8 to 3348.5)	939 (299.8 to 2291.8)	1755.3 (465.1 to 5385)	1043.9 (247.1 to 3345)
		Male	49 (25 to 69)	3.6 (1.7 to 5.1)	1 (0 to 1)	0.1 (0 to 0.1)	324 (242 to 450)	14.1 (10.1 to 20)	5 (4 to 8)	0.2 (0.2 to 0.3)	556.2 (289.3 to 1278.5)	292.6 (130.6 to 761.8)	623.3 (308.2 to 2211.4)	283.8 (114.3 to 1136.3)
	Sexually transmitted infections excluding HIV	Both	920 (448 to 1764)	24.5 (13.3 to 44.6)	7 (2 to 17)	0.2 (0.1 to 0.4)	739 (446 to 1175)	18.7 (10.9 to 31.3)	3 (2 to 7)	0.1 (0 to 0.2)	-19.6 (-49.9 to 24.7)	-23.7 (-44.7 to -4.5)	-52.3 (-70.6 to -19.9)	-29.8 (-55.7 to 5.6)
		Female	482 (244 to 903)	28.1 (15.7 to 49.4)	3 (1 to 8)	0.2 (0.1 to 0.4)	449 (265 to 735)	21.9 (12.6 to 35.6)	2 (1 to 3)	0.1 (0.1 to 0.2)	-6.8 (-40.4 to 37.2)	-22.3 (-40.8 to -5.8)	-47 (-66.5 to -5.9)	-24.6 (-50.8 to 17.9)
		Male	438 (191 to 896)	20.8 (10.1 to 39.7)	4 (1 to 9)	0.2 (0.1 to 0.4)	290 (170 to 474)	15.6 (8.3 to 26.3)	2 (1 to 4)	0.1 (0 to 0.2)	-33.8 (-60.4 to 11.9)	-25.4 (-51.4 to 1.4)	-56.8 (-75.3 to -30.4)	-34.5 (-62.3 to 3.1)
	Respiratory infections and tuberculosis	Both	90445 (65978 to 128381)	2158.5 (1648.5 to 2914.7)	1152 (876 to 1559)	42.5 (35.4 to 51)	18343 (15651 to 21574)	513.6 (440.8 to 597.8)	567 (488 to 663)	20.1 (17.2 to 23.5)	-79.7 (-86.4 to -71.3)	-76.2 (-83.2 to -67.8)	-50.8 (-65.4 to -32.2)	-52.7 (-62.6 to -40.9)
		Female	44966 (30885 to 64838)	2153 (1561.2 to 2992.5)	561 (399 to 784)	40.5 (32.2 to 51.1)	8184 (6802 to 9832)	472.1 (395.8 to 563.7)	257 (209 to 318)	18.8 (15.2 to 23)	-81.8 (-88.1 to -72.2)	-78.1 (-85 to -68.1)	-54.2 (-69.2 to -32.7)	-53.7 (-65.6 to -38.9)
		Male	45479 (32194 to 67172)	2162.1 (1615.5 to 3034.1)	591 (435 to 835)	44.4 (35.8 to 55.7)	10159 (8439 to 12087)	554.8 (464.3 to 659.7)	310 (253 to 384)	21.4 (17.6 to 26.4)	-77.7 (-85.4 to -67.2)	-74.3 (-82.5 to -64.2)	-47.5 (-64.6 to -24.1)	-51.8 (-63.4 to -37.1)
	Tuberculosis	Both	3248 (2518 to 3975)	110.5 (84.9 to 131.6)	62 (47 to 75)	3.3 (2.4 to 4)	840 (718 to 990)	20.9 (18.1 to 24.6)	27 (22 to 32)	0.8 (0.7 to 0.9)	-74.1 (-79.8 to -62.5)	-81.1 (-85 to -72.6)	-57.2 (-67.1 to -34.3)	-76.5 (-82.2 to -63.2)
		Female	1534 (861 to 2029)	100.5 (58.4 to 128.4)	27 (14 to 35)	2.8 (1.4 to 3.9)	346 (276 to 461)	17.8 (14.3 to 23.8)	11 (8 to 16)	0.6 (0.5 to 0.9)	-77.4 (-83.9 to -52)	-82.3 (-87.1 to -60.9)	-59.4 (-72.4 to 4.5)	-77.2 (-84.6 to -37.7)
		Male	1714 (1350 to 2143)	119.7 (95.1 to 147.9)	36 (28 to 44)	3.8 (3 to 4.8)	494 (408 to 596)	24 (19.9 to 28.8)	16 (13 to 19)	0.9 (0.8 to 1.1)	-71.2 (-78.4 to -61.4)	-79.9 (-84.6 to -73.3)	-55.5 (-67.1 to -38.6)	-75.8 (-82.4 to -67.2)
	Lower respiratory infections	Both	82756 (59038 to 119652)	1931.5 (1441.2 to 2650.1)	1090 (820 to 1492)	39.2 (32.4 to 47.5)	12914 (11104 to 14900)	378.5 (326.6 to 435.1)	540 (464 to 633)	19.3 (16.5 to 22.6)	-84.4 (-89.9 to -76.8)	-80.4 (-86.8 to -72)	-50.4 (-65.6 to -30.7)	-50.7 (-61.1 to -37.9)
		Female	41341 (27578 to 61002)	1940.6 (1341.6 to 2769)	535 (373 to 756)	37.7 (29.5 to 47.7)	5691 (4682 to 6965)	345 (284.5 to 421)	246 (198 to 306)	18.1 (14.5 to 22.3)	-86.2 (-91.3 to -78)	-82.2 (-88.5 to -72.6)	-54 (-69.4 to -31.1)	-52 (-64.5 to -36.7)
		Male	41416 (28435 to 63158)	1921.5 (1386.8 to 2799.5)	555 (404 to 802)	40.6 (32.6 to 51.4)	7223 (5988 to 8639)	411.9 (342.4 to 491)	294 (238 to 366)	20.5 (16.7 to 25.3)	-82.6 (-89.3 to -73)	-78.6 (-86.3 to -68.4)	-47 (-65.2 to -22.3)	-49.6 (-62 to -33.6)
	Upper respiratory infections	Both	3195 (1925 to 5104)	84.8 (50.7 to 133.3)	0 (0 to 2)	0 (0 to 0)	3440 (2061 to 5428)	85.2 (51.3 to 134.5)	0 (0 to 1)	0 (0 to 0)	7.6 (-1.1 to 17.6)	0.5 (-5.3 to 5.9)	-5.4 (-92.4 to 2001.2)	6.5 (-91.1 to 1593.2)
		Female	1540 (919 to 2464)	82.9 (49.2 to 129.7)	0 (0 to 1)	0 (0 to 0)	1636 (978 to 2567)	83.1 (50.3 to 129.9)	0 (0 to 0)	0 (0 to 0)	6.3 (-3.9 to 17.6)	0.2 (-6.8 to 7.9)	15.3 (-94.5 to 3696.4)	42.7 (-92.7 to 2829.5)
		Male	1656 (998 to 2578)	86.6 (52.2 to 135.3)	0 (0 to 1)	0 (0 to 0.1)	1803 (1094 to 2859)	87.3 (53.2 to 138.8)	0 (0 to 0)	0 (0 to 0)	8.9 (-1.7 to 20.7)	0.8 (-7.2 to 8.5)	-17.9 (-91.3 to 1444.5)	-13.1 (-90.5 to 1103.9)
	Otitis media	Both	1245 (704 to 2043)	31.7 (18.1 to 53.1)	0 (0 to 0)	0 (0 to 0)	1149 (651 to 1925)	28.9 (16.4 to 48)	0 (0 to 0)	0 (0 to 0)	-7.7 (-16.6 to 3)	-8.7 (-14.6 to -2.7)	-74.8 (-88 to -38.5)	-74.4 (-86.3 to -42.4)

Province	Cause	Sex	1990				2019				1990-2019 percent change (%)			
			DALYs		Death		DALYs		Death		DALYs		Death	
			Number	Rate	Number	Rate	Number	Rate	Number	Rate	Number	Rate	Number	Rate
		Female	552 (318 to 908)	29 (16.8 to 48.3)	0 (0 to 0)	0 (0 to 0)	511 (289 to 845)	26.2 (14.9 to 42.6)	0 (0 to 0)	0 (0 to 0)	-7.4 (-18.2 to 5.1)	-9.6 (-17.8 to -0.9)	-31.6 (-75.1 to 124.7)	-52.6 (-80.8 to 50.5)
		Male	693 (388 to 1142)	34.3 (19.4 to 57)	0 (0 to 0)	0 (0 to 0)	638 (358 to 1071)	31.5 (17.7 to 52.7)	0 (0 to 0)	0 (0 to 0)	-8 (-18.6 to 4.9)	-8.1 (-16 to 0.8)	-82.7 (-93.4 to -47.9)	-80.6 (-91.4 to -44.9)
	Enteric infections	Both	28919 (19182 to 43848)	669.6 (463.3 to 979.9)	315 (204 to 488)	9.9 (6.5 to 14.5)	10039 (7511 to 13004)	270.9 (203.6 to 347.6)	61 (43 to 97)	2 (1.4 to 3.2)	-65.3 (-78.7 to -44.7)	-59.5 (-74.4 to -39.3)	-80.6 (-89.1 to -66.5)	-79.9 (-86.6 to -68.9)
		Female	13905 (8366 to 23013)	661.5 (409.1 to 1047.7)	152 (85 to 259)	10.4 (5.3 to 17.4)	4820 (3579 to 6288)	265.7 (197.8 to 345.6)	29 (16 to 54)	2 (1.1 to 3.7)	-65.3 (-81.1 to -39.9)	-59.8 (-76.9 to -33.6)	-80.9 (-90.9 to -62)	-81 (-89.3 to -64.3)
		Male	15014 (9303 to 25142)	675.8 (434.4 to 1085)	162 (95 to 284)	9.4 (5.8 to 14.9)	5219 (3859 to 6726)	276.1 (204.4 to 357.1)	32 (22 to 52)	2 (1.4 to 3.4)	-65.2 (-81 to -41.4)	-59.1 (-76.9 to -35.2)	-80.3 (-90.2 to -62.5)	-78.4 (-87.7 to -65.1)
	Diarrheal diseases	Both	25437 (16397 to 39934)	594.2 (399.1 to 893.6)	268 (162 to 428)	8.8 (5.6 to 13.2)	9021 (6628 to 11937)	242.7 (179.7 to 319.6)	46 (29 to 77)	1.6 (1 to 2.8)	-64.5 (-79 to -42)	-59.2 (-74.6 to -37)	-82.9 (-91.1 to -67.8)	-82.1 (-88.7 to -71.6)
		Female	12194 (6889 to 21275)	586.1 (343.7 to 954.9)	129 (64 to 233)	9.3 (4.4 to 16.1)	4355 (3162 to 5830)	239.2 (174.1 to 318.3)	22 (10 to 46)	1.6 (0.7 to 3.3)	-64.3 (-81.5 to -34.1)	-59.2 (-77.8 to -27.9)	-83 (-93.1 to -61.3)	-83 (-91.3 to -65.5)
		Male	13243 (7750 to 22882)	600.5 (365.6 to 986.4)	139 (75 to 246)	8.3 (4.9 to 13.7)	4666 (3385 to 6128)	246.2 (179.5 to 323.5)	24 (14 to 42)	1.6 (1 to 2.8)	-64.8 (-81.5 to -37.2)	-59 (-77.3 to -31.8)	-82.9 (-92.2 to -63.7)	-80.8 (-89.8 to -67.3)
	Typhoid and paratyphoid	Both	2719 (975 to 5436)	58.7 (21.7 to 116.6)	37 (13 to 73)	0.8 (0.3 to 1.6)	746 (308 to 1483)	20.8 (8.5 to 42.3)	11 (4 to 21)	0.3 (0.1 to 0.6)	-72.6 (-80.7 to -63.3)	-64.6 (-75 to -53.1)	-70.7 (-78.5 to -60.5)	-64.9 (-74.3 to -54)
		Female	1259 (457 to 2542)	55.5 (20.4 to 110.1)	17 (6 to 34)	0.8 (0.3 to 1.6)	343 (138 to 674)	19.7 (7.9 to 39.3)	5 (2 to 10)	0.3 (0.1 to 0.5)	-72.8 (-81.2 to -61.7)	-64.5 (-75.5 to -50.2)	-70.8 (-79.6 to -58.6)	-64.9 (-75.3 to -51.2)
		Male	1460 (530 to 2962)	61.8 (22.9 to 124.1)	20 (7 to 40)	0.9 (0.3 to 1.7)	403 (162 to 820)	21.8 (8.7 to 44.5)	6 (2 to 12)	0.3 (0.1 to 0.6)	-72.4 (-81.2 to -60.8)	-64.7 (-75.5 to -50.6)	-70.5 (-79.4 to -57.9)	-64.9 (-75.2 to -51.1)
	Invasive Non-typhoidal Salmonella (iNTS)	Both	625 (312 to 1077)	13.8 (6.9 to 23)	9 (4 to 14)	0.2 (0.1 to 0.4)	255 (125 to 450)	7 (3.4 to 12.3)	4 (2 to 7)	0.1 (0.1 to 0.2)	-59.1 (-74.4 to -41.5)	-49.1 (-67.2 to -28.8)	-49.4 (-66.2 to -29.4)	-47.1 (-61.8 to -30.4)
		Female	372 (180 to 645)	16.5 (8.1 to 28.3)	5 (2 to 9)	0.3 (0.1 to 0.4)	114 (57 to 205)	6.3 (3.1 to 11.5)	2 (1 to 3)	0.1 (0.1 to 0.2)	-69.4 (-81.9 to -53.2)	-61.6 (-76.2 to -43.9)	-60.1 (-74.3 to -40.9)	-58.1 (-71.5 to -41.6)
		Male	253 (126 to 428)	11.1 (5.7 to 18.4)	4 (2 to 6)	0.2 (0.1 to 0.3)	142 (69 to 254)	7.6 (3.7 to 13.7)	2 (1 to 4)	0.1 (0.1 to 0.2)	-44 (-65.6 to -15.4)	-31.4 (-56.5 to 3.3)	-34.2 (-56.5 to -2)	-32.2 (-52.3 to -2.7)
	Other intestinal infectious diseases	Both	138 (35 to 285)	2.8 (0.8 to 5.7)	2 (0 to 3)	0 (0 to 0.1)	17 (11 to 24)	0.5 (0.3 to 0.7)	0 (0 to 0)	0 (0 to 0)	-87.9 (-95 to -51.2)	-83.9 (-93.2 to -38.9)	-88.3 (-95.4 to -50)	-84.2 (-93.5 to -50.7)
		Female	80 (8 to 194)	3.4 (0.4 to 8)	1 (0 to 2)	0 (0 to 0.1)	8 (5 to 13)	0.5 (0.2 to 0.8)	0 (0 to 0)	0 (0 to 0)	-89.7 (-96.7 to 7.4)	-86 (-95.5 to 16.8)	-89.6 (-97.1 to 24.6)	-85.5 (-96.2 to -8.9)
		Male	58 (8 to 144)	2.4 (0.4 to 5.7)	1 (0 to 2)	0 (0 to 0.1)	8 (5 to 13)	0.4 (0.3 to 0.7)	0 (0 to 0)	0 (0 to 0)	-85.5 (-94.9 to 16.8)	-81.1 (-93.2 to 11.7)	-86.4 (-96.3 to 22.5)	-82.5 (-94.6 to -17.2)
	Neglected tropical diseases and malaria	Both	10007 (1361 to 52299)	261.4 (34.7 to 1346.5)	144 (8 to 811)	4.7 (0.3 to 24.2)	715 (472 to 1276)	18.5 (12.2 to 33.6)	5 (3 to 14)	0.1 (0.1 to 0.4)	-92.9 (-98.7 to -53.1)	-92.9 (-98.7 to -51.9)	-96.5 (-99.5 to -48.3)	-96.9 (-99.5 to -55.2)
		Female	4478 (674 to 24565)	233.3 (36.6 to 1261.1)	60 (3 to 363)	3.7 (0.2 to 20.4)	352 (235 to 521)	18.5 (12.3 to 27.6)	2 (1 to 5)	0.1 (0.1 to 0.3)	-92.1 (-98.7 to -51)	-92.1 (-98.6 to -53.4)	-96.4 (-99.4 to -43.6)	-96.5 (-99.4 to -49.6)
		Male	5529 (621 to 27763)	287.5 (31.1 to 1423.8)	84 (4 to 446)	5.6 (0.3 to 27.1)	362 (219 to 759)	18.5 (11.1 to 39.4)	3 (1 to 9)	0.2 (0.1 to 0.5)	-93.4 (-98.8 to -46.4)	-93.6 (-98.9 to -43.1)	-96.7 (-99.5 to -42.8)	-97.2 (-99.5 to -52.7)
	Malaria	Both	7138 (48 to 50529)	192.8 (1.3 to 1299)	117 (0 to 794)	3.9 (0 to 23.7)	47 (34 to 63)	1.1 (0.8 to 1.5)	0 (0 to 0)	0 (0 to 0)	-99.3 (-99.9 to -3.6)	-99.4 (-99.9 to -15)	-100 (-100 to -100)	-100 (-100 to -100)
		Female	3225 (25 to 23155)	170.1 (1.4 to 1189.5)	50 (0 to 351)	3.1 (0 to 20.1)	24 (17 to 32)	1.1 (0.8 to 1.5)	0 (0 to 0)	0 (0 to 0)	-99.3 (-99.9 to -6.9)	-99.3 (-99.9 to -19.5)	-100 (-100 to -100)	-100 (-100 to -100)
		Male	3913 (22 to 26757)	213.7 (1.1 to 1374.1)	67 (0 to 435)	4.7 (0 to 26.5)	23 (17 to 32)	1.1 (0.8 to 1.5)	0 (0 to 0)	0 (0 to 0)	-99.4 (-99.9 to 2.1)	-99.5 (-99.9 to -7.6)	-100 (-100 to -100)	-100 (-100 to -100)

Province	Cause	Sex	1990				2019				1990-2019 percent change (%)			
			DALYs		Death		DALYs		Death		DALYs		Death	
			Number	Rate	Number	Rate	Number	Rate	Number	Rate	Number	Rate	Number	Rate
	Leishmaniasis	Both	1238 (4 to 13350)	28 (0.1 to 286.6)	17 (0 to 178)	0.4 (0 to 4.1)	66 (1 to 633)	1.7 (0 to 17.1)	1 (0 to 10)	0 (0 to 0.2)	-94.7 (-95.6 to -75.1)	-93.9 (-95.3 to -73)	-93.7 (-94.5 to -88.9)	-93.8 (-95 to -92)
		Female	420 (1 to 4343)	19.3 (0.1 to 185.6)	6 (0 to 57)	0.3 (0 to 2.7)	21 (0 to 188)	1.1 (0 to 10.2)	0 (0 to 3)	0 (0 to 0.2)	-95 (-96.1 to -61.9)	-94.2 (-95.7 to -61.2)	-94 (-95 to -88.7)	-94.1 (-95.6 to -91.8)
		Male	818 (2 to 8983)	36.5 (0.1 to 378.4)	11 (0 to 119)	0.6 (0 to 5.5)	45 (0 to 441)	2.3 (0 to 22.9)	1 (0 to 7)	0 (0 to 0.3)	-94.5 (-95.5 to -82.2)	-93.7 (-95.2 to -80.1)	-93.5 (-94.5 to -88.2)	-93.6 (-95 to -91.3)
	Schistosomiasis	Both	0 (0 to 0)	0 (0 to 0)	0 (0 to 0)	0 (0 to 0)	0 (0 to 0)	0 (0 to 0)	0 (0 to 0)	0 (0 to 0)	0 (0 to 0)	0 (0 to 0)	0 (0 to 0)	0 (0 to 0)
		Female	0 (0 to 0)	0 (0 to 0)	0 (0 to 0)	0 (0 to 0)	0 (0 to 0)	0 (0 to 0)	0 (0 to 0)	0 (0 to 0)	0 (0 to 0)	0 (0 to 0)	0 (0 to 0)	0 (0 to 0)
		Male	0 (0 to 0)	0 (0 to 0)	0 (0 to 0)	0 (0 to 0)	0 (0 to 0)	0 (0 to 0)	0 (0 to 0)	0 (0 to 0)	0 (0 to 0)	0 (0 to 0)	0 (0 to 0)	0 (0 to 0)
	Cystic echinococcosis	Both	261 (142 to 401)	7.5 (4.7 to 10.5)	3 (2 to 5)	0.1 (0.1 to 0.2)	121 (84 to 174)	2.8 (2 to 4)	1 (1 to 2)	0 (0 to 0)	-53.5 (-72.8 to -13)	-62.1 (-76.3 to -38.4)	-70.9 (-86 to -35.1)	-76.9 (-88.5 to -55.9)
		Female	116 (49 to 202)	6.8 (3.7 to 10.3)	1 (1 to 3)	0.1 (0 to 0.2)	64 (42 to 93)	3 (2 to 4.3)	0 (0 to 1)	0 (0 to 0.1)	-44.5 (-72.8 to 35.3)	-55.8 (-75.3 to -17.4)	-68.1 (-90.2 to 9.7)	-74.2 (-91.8 to -27.8)
		Male	145 (58 to 252)	8.1 (4.2 to 12.6)	2 (1 to 3)	0.1 (0.1 to 0.2)	57 (37 to 81)	2.6 (1.7 to 3.8)	1 (0 to 1)	0 (0 to 0.1)	-60.8 (-80.5 to -1.9)	-67.2 (-81.8 to -35.3)	-72.8 (-91.3 to -22.9)	-78.7 (-92.3 to -47.3)
	Trachoma	Both	31 (18 to 53)	2.2 (1.3 to 3.6)	--	--	14 (7 to 23)	0.4 (0.2 to 0.6)	--	--	-56.8 (-67.5 to -43.4)	-82.7 (-86.8 to -78.1)	--	--
		Female	23 (13 to 38)	3.5 (2 to 5.9)	--	--	9 (5 to 16)	0.5 (0.3 to 0.9)	--	--	-59.2 (-71.3 to -44.1)	-85 (-89.3 to -80)	--	--
		Male	8 (4 to 15)	1 (0.5 to 1.8)	--	--	4 (2 to 7)	0.2 (0.1 to 0.4)	--	--	-50 (-66 to -22.3)	-77.5 (-84.3 to -66.8)	--	--
	Rabies	Both	15 (3 to 37)	0.4 (0.1 to 1)	0 (0 to 1)	0 (0 to 0)	7 (3 to 12)	0.2 (0.1 to 0.3)	0 (0 to 0)	0 (0 to 0)	-54.1 (-83.4 to 173.2)	-61.1 (-85.2 to 115.5)	-40 (-76.2 to 215)	-57 (-82.5 to 114.5)
		Female	2 (0 to 4)	0.1 (0 to 0.4)	0 (0 to 0)	0 (0 to 0)	1 (0 to 2)	0.1 (0 to 0.1)	0 (0 to 0)	0 (0 to 0)	-20.8 (-78.5 to 444.5)	-54 (-83.8 to 212.1)	-23.4 (-73 to 416.7)	-59 (-86 to 170.5)
		Male	14 (2 to 35)	0.7 (0.1 to 1.8)	0 (0 to 1)	0 (0 to 0)	6 (2 to 10)	0.3 (0.1 to 0.5)	0 (0 to 0)	0 (0 to 0)	-58.1 (-86.1 to 192.6)	-62.5 (-87.7 to 144.7)	-43.1 (-80.1 to 264)	-56.1 (-85.3 to 151.3)
	Intestinal nematode infections	Both	290 (138 to 517)	6.7 (3.5 to 11.1)	2 (1 to 5)	0 (0 to 0.1)	42 (23 to 74)	1.1 (0.6 to 1.9)	0 (0 to 0)	0 (0 to 0)	-85.4 (-93.3 to -69.7)	-83.7 (-91.8 to -69.2)	-94.2 (-98 to -78.4)	-91.1 (-97 to -71.8)
		Female	161 (66 to 312)	8 (3.6 to 14.3)	1 (0 to 3)	0 (0 to 0.1)	26 (14 to 45)	1.3 (0.7 to 2.3)	0 (0 to 0)	0 (0 to 0)	-84 (-93.3 to -62.8)	-83.1 (-92.2 to -64.6)	-94.3 (-98.7 to -29.2)	-91 (-98.1 to -46.3)
		Male	129 (40 to 280)	5.4 (2 to 11)	1 (0 to 3)	0 (0 to 0.1)	17 (8 to 29)	0.8 (0.4 to 1.5)	0 (0 to 0)	0 (0 to 0)	-87.1 (-95.5 to -62.5)	-84.4 (-94 to -60.7)	-94.1 (-98.5 to -50)	-91.2 (-97.6 to -58.7)
	Food-borne trematodiases	Both	12 (3 to 25)	0.5 (0.1 to 1)	--	--	44 (10 to 93)	1 (0.2 to 2.1)	--	--	262.6 (170.5 to 369.4)	109.8 (61.7 to 164.1)	--	--
		Female	5 (1 to 10)	0.4 (0.1 to 0.8)	--	--	18 (4 to 37)	0.8 (0.2 to 1.7)	--	--	253.5 (158.3 to 367.2)	101.5 (48.1 to 160)	--	--
		Male	7 (2 to 15)	0.6 (0.1 to 1.1)	--	--	27 (6 to 57)	1.2 (0.3 to 2.5)	--	--	268.8 (134.3 to 419.9)	116.1 (46.1 to 197.8)	--	--
	Leprosy	Both	1 (1 to 2)	0.1 (0 to 0.1)	--	--	2 (1 to 2)	0 (0 to 0.1)	--	--	41.8 (27.9 to 55.9)	-31.6 (-37.9 to -25.8)	--	--

Province	Cause	Sex	1990				2019				1990-2019 percent change (%)			
			DALYs		Death		DALYs		Death		DALYs		Death	
			Number	Rate	Number	Rate	Number	Rate	Number	Rate	Number	Rate	Number	Rate
		Female	0 (0 to 1)	0 (0 to 0.1)	--	--	0 (0 to 1)	0 (0 to 0)	--	--	34.7 (17.8 to 52.7)	-36.7 (-43.6 to -29)	--	--
		Male	1 (0 to 1)	0.1 (0 to 0.1)	--	--	1 (1 to 2)	0 (0 to 0.1)	--	--	45.3 (28.1 to 63.3)	-27.7 (-35.6 to -19.1)	--	--
	Other neglected tropical diseases	Both	1020 (650 to 1501)	23.3 (15.5 to 33.3)	4 (2 to 7)	0.1 (0.1 to 0.2)	372 (240 to 547)	10.2 (6.5 to 15.1)	3 (1 to 4)	0.1 (0 to 0.1)	-63.5 (-76 to -45.2)	-56.2 (-70.5 to -35.3)	-33.4 (-65.9 to 12.4)	-39.6 (-62.7 to -8)
		Female	525 (310 to 800)	25 (15.6 to 36.4)	2 (1 to 4)	0.1 (0.1 to 0.2)	189 (116 to 287)	10.4 (6.5 to 16.2)	1 (0 to 2)	0.1 (0 to 0.1)	-64.1 (-77.3 to -41.4)	-58.3 (-73 to -33.9)	-33.8 (-64.6 to 37.9)	-40.9 (-61.9 to 4.5)
		Male	495 (277 to 810)	21.5 (12.6 to 34)	2 (1 to 5)	0.1 (0.1 to 0.3)	183 (103 to 315)	9.9 (5.5 to 17.5)	1 (1 to 2)	0.1 (0 to 0.1)	-63 (-80 to -30.2)	-53.9 (-74.4 to -14.9)	-33.1 (-71.1 to 39.1)	-37.5 (-65.2 to 18.6)
	Other infectious diseases	Both	48205 (25276 to 86121)	1028.9 (569.7 to 1781.3)	588 (316 to 1021)	14.6 (9 to 23.6)	4471 (3750 to 5437)	118.2 (97.7 to 147.8)	89 (76 to 107)	2.6 (2.2 to 3.1)	-90.7 (-94.9 to -82.2)	-88.5 (-93.6 to -79.1)	-84.8 (-91.4 to -71.4)	-82.5 (-89.3 to -70.9)
		Female	25315 (12901 to 47084)	1089.8 (586.6 to 1962.7)	303 (156 to 555)	15 (8.8 to 25.4)	1969 (1574 to 2545)	108.3 (85.2 to 144.5)	35 (28 to 45)	2.1 (1.7 to 2.6)	-92.2 (-96 to -84.2)	-90.1 (-94.7 to -80.7)	-88.5 (-93.9 to -76.3)	-86.1 (-92.2 to -74.8)
		Male	22890 (12236 to 41748)	969.1 (546.7 to 1722.6)	285 (156 to 507)	14.2 (8.8 to 23.1)	2502 (2075 to 3114)	128.2 (106.3 to 161.8)	54 (44 to 68)	3 (2.5 to 3.7)	-89.1 (-94.1 to -78.9)	-86.8 (-92.6 to -75.8)	-80.9 (-89.3 to -63.6)	-78.7 (-87 to -64.2)
	Meningitis	Both	9250 (6790 to 12741)	206.9 (155.4 to 278.6)	115 (85 to 156)	3.1 (2.4 to 4.1)	1359 (1133 to 1612)	35.8 (29.8 to 42.4)	28 (23 to 34)	0.8 (0.7 to 1)	-85.3 (-90 to -78.3)	-82.7 (-88 to -74.8)	-75.4 (-83.1 to -63.9)	-73.7 (-81.2 to -62.7)
		Female	4494 (3143 to 6507)	204.1 (145.6 to 289.3)	55 (39 to 78)	3 (2.3 to 4.2)	627 (497 to 756)	33.9 (26.6 to 41.3)	12 (9 to 14)	0.7 (0.6 to 0.9)	-86.1 (-90.9 to -79.1)	-83.4 (-89 to -75.5)	-78.7 (-85.9 to -68)	-76.7 (-84.2 to -66.9)
		Male	4756 (3273 to 7028)	209.5 (146.9 to 301.4)	60 (42 to 87)	3.3 (2.4 to 4.4)	733 (593 to 919)	37.8 (30.5 to 47.2)	17 (13 to 22)	1 (0.8 to 1.2)	-84.6 (-90.5 to -74.3)	-82 (-88.6 to -70.4)	-72.5 (-82.7 to -54.3)	-70.8 (-80.6 to -53.3)
	Encephalitis	Both	906 (625 to 1279)	22.1 (16.2 to 29.7)	12 (8 to 16)	0.4 (0.3 to 0.5)	638 (505 to 764)	17 (13 to 20.8)	12 (8 to 14)	0.3 (0.2 to 0.4)	-29.6 (-53.8 to 5.6)	-23 (-47.5 to 13.8)	2.2 (-32.5 to 52.5)	-10.5 (-43 to 27.5)
		Female	482 (292 to 790)	23.7 (15.5 to 36.2)	6 (4 to 9)	0.4 (0.3 to 0.5)	303 (231 to 384)	16.7 (12.3 to 21.6)	5 (3 to 7)	0.3 (0.2 to 0.4)	-37.1 (-66.9 to 3.3)	-29.6 (-61 to 13.1)	-11.9 (-51.3 to 45)	-20.1 (-50.3 to 21)
		Male	425 (279 to 627)	20.6 (14.3 to 29)	6 (4 to 8)	0.4 (0.3 to 0.5)	336 (251 to 413)	17.4 (12.7 to 21.8)	7 (4 to 8)	0.4 (0.2 to 0.5)	-21 (-50.6 to 24.6)	-15.5 (-45.9 to 29)	16.9 (-27.8 to 85.8)	-0.2 (-40.6 to 50.4)
	Diphtheria	Both	139 (60 to 269)	2.8 (1.3 to 5.4)	2 (1 to 3)	0 (0 to 0.1)	3 (2 to 5)	0.1 (0 to 0.1)	0 (0 to 0)	0 (0 to 0)	-97.9 (-99.1 to -94.3)	-97.2 (-98.9 to -92.4)	-97.3 (-98.9 to -93.1)	-96.7 (-98.6 to -92.1)
		Female	67 (17 to 166)	2.7 (0.7 to 6.7)	1 (0 to 2)	0 (0 to 0.1)	1 (0 to 2)	0.1 (0 to 0.1)	0 (0 to 0)	0 (0 to 0)	-98.3 (-99.5 to -92.5)	-97.6 (-99.4 to -89.7)	-97.9 (-99.4 to -91.4)	-97.3 (-99.2 to -89.4)
		Male	72 (22 to 159)	2.8 (1 to 6.2)	1 (0 to 2)	0 (0 to 0.1)	2 (1 to 3)	0.1 (0 to 0.2)	0 (0 to 0)	0 (0 to 0)	-97.6 (-99.2 to -91.2)	-96.8 (-99 to -89)	-96.8 (-98.9 to -89.3)	-96.2 (-98.6 to -88.4)
	Whooping cough	Both	10869 (556 to 45004)	216.6 (11.1 to 901.4)	124 (5 to 519)	2.5 (0.1 to 10.4)	247 (19 to 1034)	7.8 (0.6 to 32.5)	3 (0 to 12)	0.1 (0 to 0.4)	-97.7 (-99.9 to -43.2)	-96.4 (-99.8 to -9.8)	-97.8 (-99.9 to -25.1)	-96.5 (-99.9 to 16.4)
		Female	6340 (340 to 25913)	257.9 (13.8 to 1054.9)	72 (3 to 299)	2.9 (0.1 to 12.2)	138 (11 to 542)	8.9 (0.7 to 35.3)	2 (0 to 6)	0.1 (0 to 0.4)	-97.8 (-99.9 to -47)	-96.5 (-99.8 to -15.8)	-97.9 (-99.9 to -33.6)	-96.6 (-99.9 to 4.8)
		Male	4529 (236 to 18785)	177.1 (9.3 to 729.7)	52 (2 to 217)	2 (0.1 to 8.4)	109 (9 to 457)	6.6 (0.5 to 28.1)	1 (0 to 5)	0.1 (0 to 0.3)	-97.6 (-99.8 to -39.5)	-96.2 (-99.8 to -5.8)	-97.7 (-99.9 to -16.3)	-96.4 (-99.9 to 31.2)
	Tetanus	Both	533 (150 to 1615)	12.1 (3.8 to 35.4)	7 (2 to 19)	0.2 (0.1 to 0.5)	35 (27 to 59)	0.9 (0.7 to 1.6)	1 (1 to 1)	0 (0 to 0)	-93.5 (-97.9 to -75)	-92.3 (-97.5 to -73.6)	-90.1 (-96.6 to -67.5)	-90.2 (-96.2 to -72.5)
		Female	173 (40 to 575)	7.9 (2.1 to 25.2)	2 (1 to 7)	0.1 (0 to 0.3)	15 (11 to 18)	0.8 (0.6 to 1)	0 (0 to 0)	0 (0 to 0)	-91.4 (-97.4 to -62.6)	-89.9 (-96.8 to -61.9)	-87.2 (-95.9 to -55.2)	-87.7 (-95.5 to -65.2)
		Male	361 (87 to 1346)	16.1 (4.4 to 57.8)	5 (1 to 16)	0.3 (0.1 to 0.7)	20 (14 to 40)	1.1 (0.7 to 2)	0 (0 to 1)	0 (0 to 0)	-94.5 (-98.6 to -75.9)	-93.4 (-98.3 to -70.5)	-91.4 (-97.6 to -64.1)	-91.3 (-97.4 to -66.5)

Province	Cause	Sex	1990				2019				1990-2019 percent change (%)			
			DALYs		Death		DALYs		Death		DALYs		Death	
			Number	Rate	Number	Rate	Number	Rate	Number	Rate	Number	Rate	Number	Rate
	Measles	Both	21240 (6696 to 47147)	413 (129.4 to 914.9)	247 (78 to 550)	4.8 (1.5 to 10.7)	220 (66 to 520)	6.8 (2 to 16)	3 (1 to 6)	0.1 (0 to 0.2)	-99 (-99.6 to -97.9)	-98.4 (-99.3 to -96.6)	-99 (-99.6 to -97.9)	-98.4 (-99.4 to -96.7)
		Female	11159 (3546 to 24653)	443.3 (141.2 to 978.5)	130 (41 to 286)	5.2 (1.7 to 11.4)	110 (32 to 266)	6.9 (2 to 16.8)	1 (0 to 3)	0.1 (0 to 0.2)	-99 (-99.6 to -97.9)	-98.4 (-99.4 to -96.7)	-99 (-99.6 to -97.9)	-98.5 (-99.4 to -96.7)
		Male	10081 (3169 to 23060)	383.9 (120.5 to 878.7)	117 (37 to 268)	4.5 (1.4 to 10.2)	110 (34 to 263)	6.6 (2 to 15.7)	1 (0 to 3)	0.1 (0 to 0.2)	-98.9 (-99.6 to -97.8)	-98.3 (-99.3 to -96.5)	-98.9 (-99.6 to -97.8)	-98.3 (-99.3 to -96.5)
	Varicella and herpes zoster	Both	474 (269 to 798)	12.8 (8.4 to 18.8)	6 (4 to 10)	0.3 (0.2 to 0.4)	179 (129 to 246)	4.8 (3.4 to 6.5)	2 (1 to 3)	0.1 (0.1 to 0.1)	-62.2 (-79.7 to -31.6)	-62.8 (-77.5 to -41.9)	-67.4 (-82.5 to -39.5)	-75.2 (-85.2 to -58)
		Female	243 (109 to 464)	13.3 (7.6 to 22.4)	3 (2 to 6)	0.3 (0.2 to 0.5)	90 (61 to 130)	4.8 (3.4 to 6.9)	1 (1 to 2)	0.1 (0 to 0.1)	-63 (-83.6 to -15.5)	-63.5 (-80.8 to -33.7)	-67.9 (-85.7 to -25.4)	-75.3 (-88 to -47.7)
		Male	232 (116 to 435)	12.4 (7.6 to 20.4)	3 (2 to 5)	0.3 (0.2 to 0.5)	90 (60 to 129)	4.7 (3.2 to 6.7)	1 (1 to 2)	0.1 (0 to 0.1)	-61.3 (-81.8 to -21.4)	-62.1 (-79.2 to -33.8)	-66.9 (-85 to -27.1)	-75.2 (-88 to -49.5)
	Acute hepatitis	Both	3443 (2182 to 4973)	107.5 (68.5 to 150.1)	63 (39 to 90)	2.8 (1.7 to 4.1)	984 (809 to 1198)	23.5 (19.4 to 28.6)	26 (20 to 33)	0.7 (0.5 to 0.9)	-71.4 (-81.3 to -52.4)	-78.1 (-85.8 to -63.5)	-58.6 (-74.6 to -28.7)	-75.6 (-85.5 to -58.1)
		Female	1719 (904 to 2723)	102.2 (52.9 to 149.1)	28 (14 to 42)	2.5 (1.2 to 3.7)	298 (235 to 371)	15 (11.9 to 18.7)	7 (5 to 9)	0.4 (0.3 to 0.5)	-82.6 (-89.7 to -64)	-85.3 (-90.9 to -69.1)	-75.1 (-85.1 to -42.1)	-84.6 (-91.4 to -62.3)
		Male	1725 (1028 to 2938)	111.4 (68.3 to 185.6)	35 (21 to 59)	3 (1.8 to 5.1)	686 (534 to 889)	32.1 (25.1 to 41.3)	19 (14 to 25)	1 (0.7 to 1.3)	-60.2 (-78.7 to -27.3)	-71.2 (-84.6 to -49.8)	-45 (-72.3 to 0.4)	-67.4 (-83.4 to -42.4)
	Other unspecified infectious diseases	Both	1351 (741 to 3564)	35 (19.8 to 86.5)	12 (5 to 41)	0.5 (0.2 to 1.4)	806 (592 to 1281)	21.5 (15.7 to 34.5)	15 (11 to 25)	0.5 (0.3 to 0.7)	-40.3 (-72.9 to -3.2)	-38.7 (-69.7 to -4.6)	21.6 (-55.1 to 180.1)	-12.8 (-58.9 to 95.9)
		Female	640 (348 to 1710)	34.9 (19.7 to 82.5)	5 (2 to 19)	0.5 (0.2 to 1.4)	388 (273 to 606)	21.2 (14.8 to 33.2)	7 (5 to 12)	0.4 (0.3 to 0.7)	-39.4 (-74.2 to -2.6)	-39.3 (-70.5 to -6.1)	25.7 (-55.1 to 200)	-18 (-60.2 to 111.8)
		Male	711 (355 to 2097)	35.2 (18.6 to 97)	7 (3 to 25)	0.5 (0.2 to 1.5)	418 (291 to 741)	21.8 (15 to 39.5)	8 (6 to 15)	0.5 (0.4 to 0.9)	-41.2 (-74.3 to 6.9)	-37.9 (-71.4 to 7)	18.3 (-58 to 195)	-6.5 (-60.6 to 104.5)
	Maternal and neonatal disorders	Both	289833 (236403 to 350547)	6166.8 (5034.8 to 7453.8)	3243 (2642 to 3929)	69.1 (56.4 to 83.6)	31781 (25706 to 37966)	1000.9 (803.7 to 1212.5)	254 (193 to 318)	8.7 (6.6 to 10.9)	-89 (-92 to -85.8)	-83.8 (-88.2 to -78.8)	-92.2 (-94.6 to -89.2)	-87.4 (-91.3 to -82.6)
		Female	127418 (103185 to 156033)	5599.3 (4548.9 to 6846.7)	1428 (1157 to 1752)	63 (51 to 77.1)	14682 (11915 to 17488)	935.4 (739.4 to 1135.6)	116 (86 to 146)	8 (6 to 10.1)	-88.5 (-91.6 to -84.7)	-83.3 (-87.9 to -77.6)	-91.9 (-94.5 to -88.9)	-87.3 (-91.3 to -82.5)
		Male	162415 (130454 to 200104)	6700.3 (5382.4 to 8251.3)	1815 (1452 to 2240)	74.8 (59.8 to 92.3)	17099 (13705 to 20492)	1062.8 (839.8 to 1294.8)	139 (102 to 176)	9.4 (6.9 to 11.9)	-89.5 (-92.4 to -85.7)	-84.1 (-88.7 to -78.4)	-92.4 (-94.9 to -89.2)	-87.5 (-91.7 to -82.3)
	Maternal disorders	Female	3433 (2368 to 4738)	222.2 (152 to 309.3)	48 (31 to 71)	3.3 (2.2 to 4.7)	727 (555 to 936)	33.2 (25.6 to 43)	8 (6 to 12)	0.4 (0.3 to 0.5)	-78.8 (-86.3 to -67.7)	-85 (-90.4 to -77.4)	-82.6 (-89.7 to -70.5)	-88.8 (-93.3 to -80.8)
	Neonatal disorders	Both	286399 (233197 to 347006)	6055.9 (4930.9 to 7335.3)	3195 (2598 to 3879)	67.4 (54.9 to 81.9)	31054 (24957 to 37333)	984.8 (786.4 to 1194.4)	246 (185 to 310)	8.5 (6.4 to 10.7)	-89.2 (-92.1 to -85.8)	-83.7 (-88.3 to -78.5)	-92.3 (-94.8 to -89.3)	-87.4 (-91.4 to -82.5)
		Female	123984 (100400 to 152601)	5377.1 (4361.5 to 6609.7)	1380 (1110 to 1702)	59.7 (48 to 73.7)	13956 (11150 to 16792)	902.1 (707.4 to 1098.5)	107 (79 to 136)	7.6 (5.7 to 9.7)	-88.7 (-91.9 to -84.9)	-83.2 (-88 to -77.4)	-92.2 (-94.8 to -89.2)	-87.2 (-91.4 to -82.1)
		Male	162415 (130454 to 200104)	6700.3 (5382.4 to 8251.3)	1815 (1452 to 2240)	74.8 (59.8 to 92.3)	17099 (13705 to 20492)	1062.8 (839.8 to 1294.8)	139 (102 to 176)	9.4 (6.9 to 11.9)	-89.5 (-92.4 to -85.7)	-84.1 (-88.7 to -78.4)	-92.4 (-94.9 to -89.2)	-87.5 (-91.7 to -82.3)
	Nutritional deficiencies	Both	20956 (15013 to 28378)	509.7 (371.5 to 682)	115 (72 to 170)	3.4 (2.3 to 4.6)	6064 (3901 to 8734)	159.2 (103.1 to 228.7)	19 (16 to 22)	0.7 (0.6 to 0.9)	-71.1 (-79.9 to -59.9)	-68.8 (-77.3 to -58.4)	-83.7 (-89.3 to -73.2)	-78.6 (-84.7 to -67.6)
		Female	11117 (7645 to 15681)	568.8 (399.8 to 780.4)	60 (34 to 100)	3.5 (2.3 to 5.6)	3276 (2081 to 4906)	173.4 (110.8 to 257.2)	10 (8 to 12)	0.8 (0.6 to 0.9)	-70.5 (-81 to -55.7)	-69.5 (-79.7 to -56.6)	-84.2 (-90.8 to -72.5)	-78.6 (-87 to -66.2)
		Male	9839 (6683 to 13936)	452.8 (313.6 to 626.4)	54 (29 to 85)	3.3 (1.9 to 4.5)	2788 (1712 to 4474)	145.1 (88.6 to 234.8)	9 (7 to 11)	0.7 (0.6 to 0.9)	-71.7 (-81.8 to -56.1)	-67.9 (-78.9 to -51.1)	-83.2 (-89.6 to -68.1)	-78.7 (-85.3 to -62.9)
	Protein-energy malnutrition	Both	10165 (6717 to 14955)	225.4 (151 to 319.9)	112 (70 to 168)	3.3 (2.3 to 4.5)	1502 (1079 to 2012)	41 (30 to 53.8)	16 (14 to 19)	0.6 (0.5 to 0.7)	-85.2 (-91.1 to -75.2)	-81.8 (-88.8 to -70.4)	-85.7 (-90.6 to -76.5)	-80.8 (-86.4 to -71.2)

Province	Cause	Sex	1990				2019				1990-2019 percent change (%)			
			DALYs		Death		DALYs		Death		DALYs		Death	
			Number	Rate	Number	Rate	Number	Rate	Number	Rate	Number	Rate	Number	Rate
		Female	5366 (3168 to 8595)	240.4 (146.9 to 382.2)	60 (34 to 99)	3.5 (2.2 to 5.5)	730 (515 to 995)	40.8 (29.3 to 54.6)	8 (7 to 10)	0.7 (0.5 to 0.8)	-86.4 (-92.5 to -75.9)	-83 (-90.4 to -70.9)	-85.9 (-91.8 to -75.5)	-80.6 (-88.2 to -69.3)
		Male	4799 (2820 to 7500)	210.9 (128.2 to 321.3)	53 (29 to 83)	3.2 (1.8 to 4.4)	772 (542 to 1057)	41.2 (29.5 to 55.3)	8 (6 to 9)	0.6 (0.5 to 0.7)	-83.9 (-90.8 to -69.9)	-80.5 (-88.4 to -65.3)	-85.4 (-91 to -72.7)	-81.2 (-87 to -67)
	Iodine deficiency	Both	485 (291 to 728)	15.5 (9.8 to 22.9)	--	--	277 (158 to 442)	6.5 (3.7 to 10.3)	--	--	-42.9 (-59.1 to -26.2)	-58.3 (-69.5 to -48)	--	--
		Female	296 (175 to 451)	19.2 (11.9 to 28.7)	--	--	172 (96 to 276)	8.2 (4.7 to 13)	--	--	-41.8 (-60.4 to -18.3)	-57.3 (-69.7 to -43.9)	--	--
		Male	189 (112 to 286)	11.9 (7.5 to 17.6)	--	--	104 (60 to 170)	4.8 (2.8 to 7.8)	--	--	-44.7 (-59.5 to -27.4)	-59.6 (-70.5 to -48.2)	--	--
	Vitamin A deficiency	Both	556 (283 to 1036)	10.5 (5.4 to 19.3)	--	--	25 (10 to 57)	0.8 (0.3 to 1.7)	--	--	-95.4 (-98.1 to -88.9)	-92.7 (-97 to -82.6)	--	--
		Female	205 (95 to 368)	7.9 (3.7 to 14.2)	--	--	11 (3 to 26)	0.7 (0.2 to 1.6)	--	--	-94.7 (-98.3 to -87.6)	-91.6 (-97.2 to -80.2)	--	--
		Male	351 (142 to 723)	13 (5.3 to 26.6)	--	--	15 (4 to 41)	0.9 (0.2 to 2.4)	--	--	-95.9 (-98.9 to -86.5)	-93.4 (-98.2 to -79.2)	--	--
	Dietary iron deficiency	Both	9347 (5388 to 14362)	246.4 (151.5 to 367.6)	--	--	3865 (2178 to 6214)	101.2 (57.4 to 162.3)	--	--	-58.7 (-73 to -38.1)	-58.9 (-72 to -41.8)	--	--
		Female	5077 (2816 to 7814)	291 (174.8 to 440)	--	--	2174 (1212 to 3620)	114.2 (63.4 to 189.5)	--	--	-57.2 (-73.8 to -30.2)	-60.8 (-75.2 to -38.6)	--	--
		Male	4270 (2230 to 7357)	203.5 (109.5 to 338.5)	--	--	1691 (805 to 3125)	88.3 (42 to 166.2)	--	--	-60.4 (-79.9 to -23.7)	-56.6 (-76.8 to -17.1)	--	--
	Other nutritional deficiencies	Both	403 (268 to 584)	11.9 (8 to 17)	2 (1 to 3)	0.1 (0.1 to 0.1)	395 (258 to 557)	9.7 (6.5 to 13.7)	3 (2 to 3)	0.1 (0.1 to 0.1)	-1.9 (-29.5 to 29.1)	-17.9 (-37.8 to 5.5)	21 (-29.9 to 125.2)	1.6 (-35.8 to 84)
		Female	172 (110 to 255)	10.2 (6.6 to 15)	1 (0 to 1)	0.1 (0 to 0.1)	189 (118 to 282)	9.5 (6.1 to 14)	1 (1 to 1)	0.1 (0.1 to 0.1)	10 (-26.8 to 58.6)	-7.4 (-35.2 to 30.2)	43.8 (-31.7 to 215.4)	22.2 (-39.8 to 162.5)
		Male	231 (147 to 353)	13.4 (8.8 to 20.5)	1 (1 to 2)	0.1 (0.1 to 0.2)	206 (129 to 310)	10 (6.3 to 14.8)	1 (1 to 2)	0.1 (0.1 to 0.1)	-10.7 (-42.5 to 31.2)	-25.5 (-48 to 3.5)	7.3 (-43.6 to 145)	-11.4 (-46.6 to 82.9)
	**Non-communicable diseases**	Both	709369 (582655 to 832737)	29115.1 (24893.7 to 33283.6)	12114 (10220 to 14004)	897.7 (791.1 to 1009.7)	838748 (718956 to 967079)	21456.9 (18614.3 to 24474.9)	20425 (18197 to 22810)	667.1 (601.8 to 737.9)	18.2 (0.9 to 40.6)	-26.3 (-35.4 to -14.9)	68.6 (37.7 to 107.6)	-25.7 (-37.2 to -12.2)
		Female	335258 (271278 to 398763)	28151.5 (23578.8 to 32860.3)	5124 (4131 to 6165)	822.3 (695.9 to 968.3)	412716 (347134 to 487644)	21195.3 (18019.9 to 24729.6)	9211 (7970 to 10645)	618.9 (542.8 to 705.5)	23.1 (2.4 to 49.3)	-24.7 (-36.1 to -11)	79.8 (39.4 to 135.8)	-24.7 (-39.3 to -6.5)
		Male	374111 (303158 to 449648)	29852.2 (24813.5 to 35181.7)	6990 (5484 to 8561)	963.3 (788.1 to 1145.8)	426032 (362876 to 490941)	21766.6 (18687 to 24912.5)	11214 (9496 to 13181)	716.3 (615.3 to 830.3)	13.9 (-7.1 to 43)	-27.1 (-39.7 to -11.3)	60.4 (22.1 to 113.6)	-25.6 (-42.2 to -5.3)
	Neoplasms	Both	71108 (57692 to 85333)	3210 (2624 to 3812.7)	2015 (1636 to 2398)	128.1 (102.6 to 152.2)	120349 (102894 to 139283)	3012.4 (2569 to 3469.8)	4664 (4002 to 5383)	132 (113.7 to 151.9)	69.2 (34.1 to 121.9)	-6.2 (-24.5 to 22.5)	131.4 (84.8 to 202.5)	3 (-17.2 to 37)
		Female	30859 (22549 to 39621)	2746.8 (2091 to 3465.4)	799 (606 to 1010)	102.4 (79.1 to 128.8)	51745 (42606 to 62056)	2542.6 (2097.7 to 3042.2)	1887 (1553 to 2251)	104.3 (86.7 to 123.4)	67.7 (22.6 to 141.8)	-7.4 (-32.1 to 28.4)	136.3 (73.7 to 231.8)	1.9 (-23.7 to 38.3)
		Male	40249 (29376 to 51671)	3613.9 (2708.1 to 4611.4)	1216 (901 to 1560)	152.3 (116.1 to 192.1)	68604 (54051 to 83234)	3497.3 (2773.4 to 4236.1)	2776 (2229 to 3358)	160.3 (129.9 to 192.5)	70.4 (22.5 to 147.6)	-3.2 (-29 to 37.4)	128.2 (67.1 to 226.4)	5.3 (-20.8 to 45.5)

Province	Cause	Sex	1990				2019				1990-2019 percent change (%)			
			DALYs		Death		DALYs		Death		DALYs		Death	
			Number	Rate	Number	Rate	Number	Rate	Number	Rate	Number	Rate	Number	Rate
	Lip and oral cavity cancer	Both	319 (241 to 406)	16.7 (12.6 to 21.2)	11 (8 to 14)	0.7 (0.5 to 0.9)	688 (575 to 827)	17.1 (14.4 to 20.4)	27 (23 to 33)	0.8 (0.7 to 0.9)	115.9 (55.1 to 212.8)	2.8 (-25.8 to 47.6)	155.7 (80.9 to 273.5)	10 (-20.4 to 59.4)
		Female	97 (70 to 127)	10.3 (7.4 to 13.4)	3 (2 to 4)	0.4 (0.3 to 0.6)	254 (204 to 312)	12.8 (10.4 to 15.7)	10 (8 to 13)	0.6 (0.5 to 0.7)	163.2 (85.8 to 297.6)	24.3 (-10.7 to 83.8)	234.9 (134.8 to 401.9)	35.8 (-3.1 to 101.4)
		Male	222 (153 to 306)	22.3 (15.4 to 30.1)	8 (5 to 11)	1 (0.7 to 1.3)	434 (339 to 546)	21.6 (17 to 27.2)	17 (13 to 22)	1 (0.8 to 1.2)	95.3 (26.8 to 219.4)	-3.5 (-36.1 to 53.5)	124.5 (47 to 263.6)	1.3 (-32.1 to 59.4)
	Nasopharynx cancer	Both	231 (183 to 287)	10 (8.1 to 12.3)	6 (5 to 8)	0.4 (0.3 to 0.4)	256 (214 to 307)	6.1 (5.2 to 7.3)	8 (7 to 10)	0.2 (0.2 to 0.3)	10.7 (-16.3 to 49.5)	-39 (-53.9 to -18)	32.3 (-0.5 to 82)	-37.5 (-52.7 to -16.5)
		Female	91 (62 to 121)	8.2 (5.8 to 10.9)	2 (2 to 3)	0.3 (0.2 to 0.4)	88 (71 to 112)	4.4 (3.5 to 5.5)	3 (2 to 4)	0.2 (0.1 to 0.2)	-2.5 (-33.3 to 54)	-46.3 (-63.7 to -16.9)	20.8 (-19.6 to 93.5)	-45.4 (-65 to -15.3)
		Male	140 (99 to 188)	11.7 (8.4 to 15.3)	4 (3 to 5)	0.4 (0.3 to 0.5)	167 (127 to 212)	7.8 (6 to 9.9)	5 (4 to 7)	0.3 (0.2 to 0.4)	19.3 (-19.7 to 91.1)	-33.2 (-54.4 to 3)	39.4 (-5.8 to 115.4)	-30.8 (-52.7 to 2.9)
	Other pharynx cancer	Both	152 (113 to 195)	8.2 (6.2 to 10.4)	5 (4 to 7)	0.4 (0.3 to 0.5)	251 (206 to 302)	6.2 (5.2 to 7.5)	10 (8 to 12)	0.3 (0.2 to 0.3)	65.3 (19.7 to 139.1)	-24.3 (-44.7 to 6.5)	93.1 (39.6 to 175.2)	-20.2 (-41.3 to 12.5)
		Female	70 (49 to 96)	8 (5.6 to 10.8)	2 (2 to 3)	0.4 (0.3 to 0.5)	103 (80 to 131)	5.2 (4.1 to 6.5)	4 (3 to 5)	0.2 (0.2 to 0.3)	46.6 (-1.7 to 145.1)	-35.5 (-56.7 to 2.3)	79.1 (17.4 to 187.7)	-30.8 (-56.7 to 8.2)
		Male	82 (55 to 116)	8.3 (5.7 to 11.7)	3 (2 to 4)	0.4 (0.3 to 0.5)	148 (113 to 190)	7.3 (5.6 to 9.4)	6 (5 to 8)	0.3 (0.3 to 0.4)	81.4 (13.7 to 190.7)	-11.9 (-44 to 38.6)	104.4 (29.1 to 228)	-7.9 (-40.4 to 41.1)
	Esophageal cancer	Both	3941 (1718 to 5150)	231.4 (99.2 to 300.7)	148 (64 to 193)	11.1 (4.6 to 14.6)	7021 (3307 to 8769)	180.7 (83.6 to 225.2)	318 (143 to 399)	9.3 (4.1 to 11.6)	78.1 (33 to 147.7)	-21.9 (-41.3 to 7.2)	114.9 (61.7 to 195.1)	-16.2 (-37 to 13)
		Female	1611 (724 to 2263)	204.7 (88.8 to 290.2)	61 (27 to 87)	10 (4.2 to 14.6)	2631 (1207 to 3396)	136.2 (62.1 to 174.9)	123 (55 to 158)	7.2 (3.2 to 9.2)	63.3 (13.3 to 154)	-33.5 (-54.1 to 0.3)	101.7 (38.8 to 202.1)	-28.4 (-51.3 to 4.5)
		Male	2330 (944 to 3265)	255.2 (100.2 to 350.4)	87 (35 to 121)	12.1 (4.6 to 16.3)	4390 (1952 to 5822)	225.6 (98 to 297)	196 (84 to 259)	11.5 (4.8 to 15)	88.4 (27.2 to 191)	-11.6 (-39.6 to 33.7)	124.1 (53.8 to 238.8)	-5.3 (-32.9 to 37.7)
	Stomach cancer	Both	13537 (10064 to 16944)	768.7 (578.3 to 954)	496 (371 to 618)	35.6 (27.5 to 44)	21486 (18145 to 25077)	551.5 (467.3 to 644.5)	960 (814 to 1124)	27.8 (23.6 to 32.3)	58.7 (18.5 to 118.8)	-28.2 (-45.8 to -4.1)	93.7 (45.4 to 161.3)	-22 (-40.1 to 2.5)
		Female	4743 (3270 to 6459)	568.1 (406.8 to 758.3)	168 (119 to 226)	26.4 (19.6 to 35.8)	7328 (5924 to 9041)	376.8 (306.4 to 462.2)	331 (268 to 403)	19.4 (15.8 to 23.5)	54.5 (5.3 to 136.5)	-33.7 (-54.3 to -2.7)	97.6 (34.2 to 190.6)	-26.5 (-49.4 to 4.1)
		Male	8794 (5991 to 11597)	944.3 (659.7 to 1218.1)	328 (226 to 427)	44 (31.6 to 55.7)	14158 (11246 to 17410)	729.7 (583.5 to 892.6)	629 (503 to 765)	36.4 (29.5 to 44.1)	61 (9.3 to 139.6)	-22.7 (-46.9 to 12)	91.7 (31.1 to 182.6)	-17.4 (-42.1 to 20.2)
	Colon and rectum cancer	Both	4167 (3042 to 5599)	226.2 (166.8 to 302.4)	141 (103 to 190)	9.9 (7.3 to 13.1)	12743 (10942 to 14859)	319 (274.5 to 369.9)	527 (451 to 609)	15.1 (12.9 to 17.3)	205.8 (112.9 to 339.6)	41 (-0.1 to 102.5)	274.6 (165.4 to 440.7)	51.9 (10 to 115.4)
		Female	1766 (1219 to 2649)	201.2 (140.5 to 298.2)	58 (40 to 87)	8.9 (6.1 to 13)	5644 (4596 to 6826)	283.4 (232 to 340.7)	239 (195 to 285)	13.7 (11.2 to 16.4)	219.5 (101.4 to 402.4)	40.9 (-9.9 to 117.4)	309.6 (163.7 to 534.6)	54.6 (0.6 to 141.3)
		Male	2401 (1582 to 3384)	247.6 (167.2 to 346.9)	82 (55 to 117)	10.8 (7.5 to 15)	7099 (5750 to 8670)	355.4 (289.5 to 431.6)	288 (232 to 352)	16.5 (13.4 to 20)	195.7 (90 to 367.3)	43.5 (-7.5 to 123.3)	249.7 (125.7 to 454.5)	52.2 (1.6 to 136.9)
	Liver cancer	Both	2240 (1694 to 2840)	121.2 (90.9 to 153.5)	78 (58 to 99)	5.5 (4 to 7)	3151 (2584 to 3821)	79.7 (65.7 to 96.2)	131 (108 to 158)	3.7 (3.1 to 4.5)	40.7 (2.4 to 99.7)	-34.3 (-52.9 to -6.9)	67 (17 to 138.9)	-32.3 (-52.8 to -1.9)
		Female	884 (596 to 1261)	101.3 (67.8 to 151.2)	30 (20 to 45)	4.7 (3 to 7.3)	1167 (919 to 1464)	59.2 (47 to 73.8)	48 (38 to 60)	2.8 (2.2 to 3.5)	32 (-15.5 to 116.5)	-41.6 (-64.2 to -2.4)	62.7 (-2.1 to 175.2)	-40.2 (-65.7 to 1)
		Male	1356 (953 to 1852)	137.9 (98.7 to 185.8)	49 (34 to 66)	6.2 (4.5 to 8.2)	1985 (1493 to 2593)	100.7 (76.5 to 130.3)	82 (63 to 106)	4.7 (3.6 to 6)	46.4 (-1 to 122.1)	-27 (-49.1 to 9.1)	69.6 (15.8 to 156.6)	-24.9 (-47.4 to 12)
	Gallbladder and biliary tract cancer	Both	495 (373 to 659)	29.2 (22.7 to 39.1)	19 (14 to 25)	1.4 (1.1 to 1.9)	1242 (862 to 1505)	31.8 (22.5 to 38.6)	55 (39 to 67)	1.6 (1.2 to 1.9)	150.9 (55.3 to 279.6)	9 (-32.2 to 58.1)	193.5 (87.1 to 328.2)	14.9 (-27 to 61.3)

Province	Cause	Sex	1990				2019				1990-2019 percent change (%)			
			DALYs		Death		DALYs		Death		DALYs		Death	
			Number	Rate	Number	Rate	Number	Rate	Number	Rate	Number	Rate	Number	Rate
		Female	243 (163 to 383)	31.3 (22.3 to 49.7)	9 (7 to 15)	1.5 (1.1 to 2.4)	630 (421 to 808)	32.6 (22.1 to 41.4)	29 (20 to 37)	1.7 (1.2 to 2.2)	159 (52.2 to 327.4)	4.1 (-38.8 to 61.5)	209.5 (84.4 to 385.3)	11.2 (-35.9 to 66.5)
		Male	252 (171 to 351)	27.3 (19 to 37.1)	9 (7 to 13)	1.3 (0.9 to 1.7)	612 (388 to 788)	31 (20.1 to 39.9)	26 (18 to 34)	1.5 (1 to 2)	143.2 (44.6 to 295.3)	13.7 (-30.5 to 80.9)	177.6 (71.6 to 345.2)	18.9 (-25.6 to 87.3)
	Pancreatic cancer	Both	909 (661 to 1209)	50.8 (37.5 to 66.8)	33 (24 to 44)	2.3 (1.7 to 3)	4883 (4115 to 5764)	122.4 (103.1 to 143.9)	207 (175 to 244)	5.8 (4.9 to 6.9)	437.2 (273.6 to 691.1)	140.9 (67.5 to 251.6)	520.9 (333.9 to 802.6)	154.7 (78.4 to 266.8)
		Female	303 (213 to 438)	36.7 (26.3 to 53.2)	11 (8 to 16)	1.7 (1.2 to 2.4)	1865 (1475 to 2307)	94.5 (74.7 to 116.4)	82 (65 to 102)	4.7 (3.7 to 5.8)	515.9 (278.5 to 858.8)	157.7 (59 to 292.6)	641.1 (350.8 to 1035.2)	179.1 (68.4 to 328.6)
		Male	606 (392 to 893)	63.5 (41.9 to 91.3)	22 (15 to 32)	2.8 (1.9 to 4.1)	3018 (2368 to 3756)	150.6 (119.8 to 187.7)	125 (100 to 156)	7 (5.6 to 8.6)	397.9 (208.4 to 734.3)	137.3 (49.9 to 291.3)	461 (247 to 831.4)	145.1 (56.4 to 295.6)
	Larynx cancer	Both	1167 (885 to 1489)	63.7 (49.2 to 79.8)	41 (31 to 52)	2.7 (2.1 to 3.4)	1828 (1509 to 2240)	45.3 (37.5 to 55.2)	74 (61 to 90)	2.1 (1.7 to 2.5)	56.7 (13.6 to 131.4)	-28.9 (-47.1 to 2.5)	81.5 (33.4 to 160.7)	-24.4 (-42.9 to 6.8)
		Female	296 (206 to 400)	33.1 (23.7 to 43.5)	10 (7 to 13)	1.4 (1 to 1.8)	415 (325 to 525)	20.7 (16.4 to 25.9)	17 (13 to 21)	1 (0.8 to 1.2)	40.4 (-6.9 to 117.5)	-37.4 (-57 to -5.4)	76.3 (19.4 to 171.6)	-29.6 (-51.1 to 1.9)
		Male	871 (622 to 1186)	91.1 (66.8 to 121.3)	31 (22 to 42)	4 (2.9 to 5.2)	1413 (1104 to 1818)	70.3 (55.5 to 90)	57 (45 to 73)	3.2 (2.5 to 4)	62.2 (11.8 to 152.9)	-22.8 (-46 to 18.6)	83.1 (26.6 to 183.1)	-20.2 (-43.1 to 21.1)
	Tracheal, bronchus, and lung cancer	Both	5874 (4305 to 7641)	318.3 (232.9 to 412.4)	207 (152 to 272)	13.9 (10.2 to 17.8)	15310 (12988 to 18109)	383.2 (326 to 452.5)	643 (546 to 765)	18 (15.4 to 21.3)	160.6 (85.9 to 274.4)	20.4 (-13.5 to 74)	210 (122.4 to 348.2)	29.3 (-6 to 84.3)
		Female	1377 (981 to 2039)	152.2 (109 to 222.2)	45 (32 to 66)	6.5 (4.6 to 9.6)	4912 (3915 to 6086)	244.5 (196 to 301.6)	204 (162 to 253)	11.4 (9.1 to 14.1)	256.6 (127.8 to 454.2)	60.7 (0.8 to 144.1)	354.7 (180.4 to 600.4)	75.7 (7.1 to 170.4)
		Male	4497 (3050 to 6091)	465.5 (324 to 619.8)	163 (111 to 220)	20.8 (14.7 to 27.3)	10399 (8242 to 12898)	524.5 (421.2 to 647.3)	439 (354 to 541)	24.8 (20.1 to 30.3)	131.2 (51.1 to 273)	12.7 (-25.7 to 76.6)	170 (77.4 to 323.5)	19.3 (-18.2 to 80.8)
	Malignant skin melanoma	Both	434 (195 to 656)	22.4 (9.7 to 33.4)	14 (6 to 20)	1 (0.4 to 1.6)	516 (367 to 648)	12.8 (8.9 to 16.1)	20 (13 to 25)	0.6 (0.4 to 0.8)	18.8 (-26.2 to 133.1)	-42.8 (-63 to 10.4)	43.9 (-4.3 to 160)	-43.7 (-61.5 to 3.3)
		Female	179 (73 to 289)	18.8 (7.7 to 29.1)	5 (2 to 8)	0.8 (0.3 to 1.3)	211 (136 to 289)	10.5 (6.8 to 14.4)	8 (5 to 11)	0.5 (0.3 to 0.6)	17.4 (-31.1 to 144.1)	-44.1 (-65.5 to 6.4)	44.9 (-8.2 to 165.2)	-45 (-64.7 to -6.4)
		Male	255 (103 to 403)	25.8 (9.5 to 41.5)	8 (3 to 13)	1.2 (0.4 to 2)	305 (186 to 415)	15.2 (8.9 to 20.5)	12 (6 to 16)	0.7 (0.4 to 1)	19.7 (-31 to 143.3)	-41.1 (-65.6 to 24.1)	43.2 (-14.4 to 179.4)	-42.5 (-65.3 to 21.8)
	Non-melanoma skin cancer	Both	105 (78 to 135)	6.5 (4.9 to 8.2)	4 (3 to 5)	0.4 (0.3 to 0.4)	364 (307 to 433)	9.8 (8.3 to 11.7)	18 (15 to 21)	0.6 (0.5 to 0.7)	248.3 (149.8 to 390.7)	50.6 (10 to 113.1)	322.2 (205.3 to 504.7)	64.3 (23.5 to 137.8)
		Female	14 (9 to 20)	1.8 (1.2 to 2.6)	1 (0 to 1)	0.1 (0.1 to 0.2)	78 (59 to 99)	4.3 (3.2 to 5.4)	4 (3 to 5)	0.3 (0.2 to 0.3)	464.3 (210.2 to 838)	136.2 (27.9 to 305.7)	600.5 (289.3 to 1119.9)	148.7 (38.4 to 356.1)
		Male	91 (66 to 121)	10.9 (8.2 to 14)	4 (3 to 5)	0.6 (0.4 to 0.8)	286 (231 to 349)	15.5 (12.6 to 18.9)	14 (11 to 17)	0.9 (0.7 to 1.1)	215.3 (114.9 to 360)	42.5 (-0.8 to 106.2)	281.9 (163 to 466.7)	51.8 (9.1 to 118.8)
	Breast cancer	Both	3127 (2162 to 4298)	150.4 (103.6 to 208.6)	85 (58 to 120)	4.7 (3.2 to 6.8)	9146 (7388 to 11335)	197.6 (160.9 to 243.4)	260 (210 to 324)	6.2 (5 to 7.7)	192.5 (96.1 to 340.6)	31.3 (-12.6 to 98)	204.7 (99.6 to 364.9)	31.7 (-17.1 to 101.9)
		Female	3083 (2099 to 4258)	306.8 (209.7 to 426.9)	84 (57 to 118)	9.6 (6.5 to 14)	9045 (7287 to 11205)	392.5 (318 to 483.1)	257 (206 to 319)	12.3 (9.9 to 15.2)	193.4 (95.5 to 343.7)	27.9 (-15.4 to 93.8)	205.6 (98.9 to 370.4)	27.5 (-19.8 to 97.8)

Province	Cause	Sex	1990				2019				1990-2019 percent change (%)			
			DALYs		Death		DALYs		Death		DALYs		Death	
			Number	Rate	Number	Rate	Number	Rate	Number	Rate	Number	Rate	Number	Rate
		Male	44 (27 to 67)	4.2 (2.7 to 6.6)	1 (1 to 2)	0.2 (0.1 to 0.3)	101 (72 to 139)	4.8 (3.5 to 6.7)	4 (2 to 5)	0.2 (0.1 to 0.3)	131.6 (35.1 to 306.9)	14.2 (-32.9 to 93.9)	152.6 (44.6 to 337)	14.5 (-33.8 to 96.7)
	Cervical cancer	Female	1046 (609 to 1432)	106.4 (65.2 to 141.8)	30 (18 to 41)	3.8 (2.4 to 5.1)	1277 (929 to 1604)	59 (43.4 to 73.9)	43 (32 to 55)	2.2 (1.7 to 2.8)	22 (-16.1 to 99.8)	-44.5 (-61.2 to -13.6)	43.3 (-0.9 to 124.6)	-40.4 (-58.3 to -11.4)
	Uterine cancer	Female	178 (114 to 257)	19.8 (13.4 to 28)	6 (4 to 8)	0.8 (0.5 to 1.2)	570 (278 to 758)	27.3 (13.7 to 35.9)	21 (11 to 27)	1.1 (0.6 to 1.4)	219.4 (61.4 to 445.8)	37.8 (-31.2 to 130.4)	258.2 (85.1 to 504.7)	40.1 (-28.6 to 133)
	Ovarian cancer	Female	465 (285 to 821)	44 (27.1 to 76.4)	13 (8 to 22)	1.4 (0.9 to 2.6)	1893 (1245 to 2447)	87.2 (58.3 to 112.2)	61 (42 to 78)	3 (2.2 to 3.9)	306.9 (95.3 to 644.5)	98.2 (-5.7 to 259.7)	377.8 (129.8 to 762.4)	109.7 (-0.6 to 274.9)
	Prostate cancer	Male	1273 (883 to 1741)	195.1 (139.9 to 260.1)	61 (43 to 82)	12.7 (9.2 to 16.9)	4414 (2691 to 5771)	263.2 (162.8 to 342.2)	250 (154 to 331)	17 (10.3 to 22.3)	246.8 (129.3 to 443.2)	34.9 (-8.2 to 105.7)	312.5 (168.9 to 546.2)	34.1 (-8.2 to 102.3)
	Testicular cancer	Male	19 (13 to 26)	1 (0.7 to 1.3)	0 (0 to 0)	0 (0 to 0)	291 (210 to 392)	13.7 (10.1 to 18.4)	6 (4 to 8)	0.3 (0.2 to 0.4)	1419.8 (847.3 to 2529.9)	1304.3 (779.9 to 2321.5)	1959.9 (1279.7 to 3439.4)	1458.1 (961.6 to 2455.3)
	Kidney cancer	Both	939 (698 to 1319)	34.4 (25.2 to 45)	21 (15 to 28)	1.2 (0.8 to 1.5)	1815 (1532 to 2149)	45.4 (38.4 to 53.3)	66 (54 to 78)	1.8 (1.5 to 2.2)	93.3 (28.3 to 182.1)	32.1 (-7.7 to 95.6)	206.7 (113.5 to 369.9)	53.5 (9.2 to 152.9)
		Female	394 (285 to 634)	29.1 (21.5 to 40)	8 (6 to 11)	1 (0.6 to 1.3)	655 (533 to 801)	33.4 (27.4 to 40.4)	22 (17 to 28)	1.2 (1 to 1.5)	66.1 (-2.8 to 150.4)	14.9 (-24.1 to 64.4)	162.7 (71.5 to 298.3)	23.4 (-18.6 to 113.3)
		Male	545 (349 to 805)	39.2 (25.2 to 55.6)	13 (8 to 19)	1.4 (0.9 to 1.9)	1160 (905 to 1440)	57.7 (45.5 to 70.8)	44 (34 to 54)	2.4 (1.9 to 3)	112.9 (26.6 to 264.5)	47.1 (-10.6 to 151.5)	235.2 (106.3 to 489.1)	78.1 (12.1 to 214.4)
	Bladder cancer	Both	875 (631 to 1131)	56.8 (41.4 to 72.7)	36 (26 to 46)	3 (2.2 to 3.9)	2365 (1984 to 2834)	63.9 (53.8 to 76.2)	113 (94 to 136)	3.5 (2.9 to 4.2)	170.3 (92.7 to 300.3)	12.5 (-18.1 to 66.1)	218.2 (130 to 377.7)	15 (-15.4 to 70)
		Female	194 (131 to 269)	27.8 (19.1 to 38.7)	8 (5 to 11)	1.6 (1.1 to 2.2)	528 (422 to 652)	29.3 (23.4 to 36.2)	27 (22 to 34)	1.8 (1.4 to 2.2)	172.5 (72.6 to 343.2)	5.4 (-32.1 to 66.5)	243.2 (120.2 to 453.6)	12.3 (-29.6 to 80)
		Male	681 (458 to 920)	83.2 (57.7 to 111.3)	28 (19 to 38)	4.5 (3.2 to 6)	1837 (1477 to 2281)	99.3 (80 to 122.4)	86 (68 to 107)	5.3 (4.3 to 6.6)	169.7 (80 to 329.7)	19.3 (-17.9 to 88.3)	210.9 (107.6 to 396.4)	17.7 (-17.7 to 84.6)
	Brain and central nervous system cancer	Both	7505 (3183 to 10641)	233.7 (107.6 to 329.9)	144 (68 to 203)	6.1 (3 to 8.5)	7736 (3628 to 10208)	186.3 (87.7 to 245.3)	216 (99 to 285)	5.5 (2.5 to 7.3)	3.1 (-44.9 to 56.7)	-20.3 (-52.6 to 9.7)	49.8 (-13.3 to 104.2)	-8.7 (-42.3 to 23.2)
		Female	3224 (1004 to 5746)	200.9 (69.9 to 341.6)	59 (21 to 99)	5 (2 to 8.1)	3672 (1593 to 5207)	180.4 (79.1 to 255.3)	101 (44 to 140)	5.2 (2.3 to 7.2)	13.9 (-49.8 to 151.1)	-10.2 (-56.7 to 78)	70.8 (-17.9 to 233.5)	4.9 (-44.4 to 85)
		Male	4281 (1576 to 6835)	263.2 (103.8 to 409.3)	85 (34 to 133)	7 (2.9 to 11)	4064 (1654 to 5890)	192.6 (77.6 to 278.1)	115 (44 to 168)	5.9 (2.2 to 8.6)	-5.1 (-51.2 to 64.1)	-26.8 (-58.9 to 18.9)	35.3 (-26.3 to 119)	-16.7 (-50.2 to 27.9)
	Thyroid cancer	Both	177 (135 to 251)	9.3 (7 to 13.3)	6 (4 to 8)	0.4 (0.3 to 0.6)	696 (409 to 848)	16.8 (10.3 to 20.3)	23 (15 to 28)	0.6 (0.4 to 0.8)	293.2 (76.9 to 473.6)	80.4 (-15.2 to 162.6)	316.9 (112.3 to 512.5)	64.5 (-15.2 to 142.5)
		Female	101 (71 to 161)	11.6 (8.2 to 18.7)	3 (2 to 5)	0.5 (0.3 to 0.8)	384 (211 to 498)	18.7 (10.7 to 23.9)	13 (8 to 17)	0.7 (0.4 to 0.9)	279.7 (54.8 to 523.7)	61.4 (-34.5 to 158.2)	298.5 (72.6 to 559.2)	44.1 (-38.3 to 134)
		Male	76 (52 to 109)	7.2 (5 to 10.2)	2 (2 to 3)	0.3 (0.2 to 0.4)	311 (180 to 402)	14.9 (9 to 19.4)	11 (7 to 14)	0.6 (0.4 to 0.7)	311.3 (95 to 561.7)	106.4 (0.3 to 232.3)	341.8 (126.8 to 624.6)	97.7 (2.6 to 218.8)
	Mesothelioma	Both	17 (10 to 30)	0.8 (0.5 to 1.5)	0 (0 to 1)	0 (0 to 0)	60 (48 to 83)	1.3 (1.1 to 1.9)	2 (2 to 3)	0 (0 to 0.1)	255.5 (99.8 to 588.2)	66.1 (-7.6 to 209)	297 (115.4 to 650.8)	75.3 (-5.2 to 216.8)
		Female	11 (7 to 17)	1.1 (0.7 to 1.7)	0 (0 to 0)	0 (0 to 0.1)	19 (14 to 26)	0.8 (0.6 to 1.2)	1 (0 to 1)	0 (0 to 0)	75.8 (-1.3 to 221.2)	-22.7 (-54.3 to 32.9)	90.3 (14.2 to 227.5)	-21.8 (-49.4 to 25.2)
		Male	6 (2 to 19)	0.6 (0.2 to 1.7)	0 (0 to 1)	0 (0 to 0.1)	41 (31 to 60)	1.8 (1.4 to 2.9)	1 (1 to 2)	0.1 (0 to 0.1)	565.4 (109.1 to 2567.5)	231.5 (2.3 to 1221.3)	669.6 (128.6 to 3048.7)	272.7 (8.6 to 1327.4)

Province	Cause	Sex	1990				2019				1990-2019 percent change (%)			
			DALYs		Death		DALYs		Death		DALYs		Death	
			Number	Rate	Number	Rate	Number	Rate	Number	Rate	Number	Rate	Number	Rate
	Hodgkin lymphoma	Both	406 (225 to 832)	14.5 (8.2 to 29)	9 (5 to 18)	0.4 (0.2 to 0.8)	728 (437 to 941)	17.4 (10.3 to 22.5)	18 (11 to 23)	0.5 (0.3 to 0.6)	79.3 (-27.7 to 281.6)	20.2 (-51.4 to 149.2)	105.6 (-15.6 to 321.9)	11.2 (-54 to 120.1)
		Female	145 (71 to 311)	11 (5.5 to 23.7)	3 (2 to 7)	0.3 (0.2 to 0.7)	294 (155 to 424)	14.6 (7.5 to 21)	7 (4 to 10)	0.4 (0.2 to 0.5)	103.1 (-27.7 to 421.9)	32.3 (-53.3 to 239.9)	127.5 (-16.9 to 467.3)	11.8 (-59.7 to 170.9)
		Male	261 (137 to 587)	17.8 (9.5 to 38.9)	6 (3 to 12)	0.5 (0.3 to 1)	434 (246 to 570)	20.3 (11.4 to 26.7)	11 (6 to 14)	0.5 (0.3 to 0.7)	66 (-35.5 to 272.2)	14.1 (-54.9 to 144.9)	93.4 (-22.7 to 328.9)	12.7 (-54.5 to 143.9)
	Non-Hodgkin lymphoma	Both	1700 (1219 to 2264)	61.5 (46.5 to 79.5)	38 (29 to 50)	1.9 (1.4 to 2.4)	2647 (2281 to 3065)	64.3 (55.3 to 74.2)	81 (69 to 94)	2.1 (1.8 to 2.5)	55.7 (8.8 to 133.3)	4.5 (-24.2 to 50.3)	109.6 (52.7 to 199.3)	11.5 (-15.1 to 57.1)
		Female	561 (346 to 861)	42 (25.4 to 62)	12 (7 to 18)	1.3 (0.8 to 1.8)	929 (744 to 1150)	46.1 (37.2 to 57)	28 (22 to 34)	1.5 (1.2 to 1.8)	65.6 (-3.4 to 195.6)	9.8 (-33 to 99.1)	126.4 (40.4 to 317.5)	13.9 (-24.6 to 111.6)
		Male	1138 (796 to 1532)	79.5 (57.2 to 104.2)	26 (19 to 34)	2.5 (1.8 to 3.1)	1718 (1397 to 2077)	82.4 (67.5 to 98.8)	53 (43 to 64)	2.8 (2.3 to 3.4)	50.9 (1.3 to 136)	3.6 (-29.3 to 57)	101.7 (39.2 to 207.7)	12.4 (-20.1 to 66.5)
	Multiple myeloma	Both	371 (265 to 566)	20.2 (14.6 to 31.3)	13 (9 to 20)	0.9 (0.6 to 1.4)	913 (695 to 1117)	22.5 (17.3 to 27.5)	36 (28 to 45)	1 (0.8 to 1.2)	146.2 (44.6 to 270.7)	11.1 (-33.9 to 65.7)	172.3 (66.1 to 305.3)	13.5 (-29.7 to 70.9)
		Female	152 (100 to 234)	18.2 (12.1 to 28)	5 (4 to 8)	0.8 (0.5 to 1.2)	337 (259 to 439)	16.5 (12.8 to 21.7)	13 (10 to 18)	0.7 (0.6 to 1)	121.5 (43.6 to 253.1)	-9.2 (-39.8 to 45.3)	142.1 (59.4 to 287.3)	-9.7 (-38.6 to 47.7)
		Male	219 (141 to 365)	22.1 (14.4 to 37.8)	8 (5 to 13)	0.9 (0.6 to 1.6)	576 (394 to 752)	28.5 (19.8 to 37.1)	23 (16 to 29)	1.2 (0.9 to 1.6)	163.4 (32.1 to 336.6)	28.8 (-36.1 to 114.2)	193.8 (50.3 to 390.2)	33.9 (-31.8 to 120.6)
	Leukemia	Both	15099 (8464 to 21549)	418.4 (254 to 564.7)	256 (157 to 345)	10 (6.9 to 12.7)	9498 (6057 to 11747)	240 (153.8 to 296.9)	264 (180 to 323)	7.2 (5 to 8.7)	-37.1 (-55.3 to -6.5)	-42.6 (-57.3 to -17.5)	3.3 (-23.5 to 45.8)	-28.7 (-44.2 to -2.4)
		Female	7686 (2930 to 11034)	418.6 (197.4 to 578.8)	122 (58 to 169)	9.2 (5.6 to 11.9)	4004 (2483 to 4994)	205.7 (128.9 to 255.3)	107 (72 to 133)	5.8 (4 to 7.1)	-47.9 (-65.8 to 10.7)	-50.9 (-66.3 to -16.2)	-12.5 (-40.3 to 50.5)	-37.2 (-54.3 to -8.9)
		Male	7413 (2877 to 11540)	417 (195.7 to 607.1)	133 (63 to 195)	10.9 (6.2 to 14.9)	5494 (3035 to 7333)	274 (153.7 to 365)	157 (93 to 206)	8.6 (5.2 to 11.1)	-25.9 (-51.4 to 43.5)	-34.3 (-55.2 to 8.7)	17.7 (-19.2 to 99.3)	-21.2 (-43.2 to 18)
	Other malignant neoplasms	Both	4227 (3359 to 5300)	148.4 (117.1 to 181.3)	91 (71 to 112)	4.8 (3.6 to 5.8)	6376 (4792 to 7653)	162.3 (121.9 to 194.3)	200 (149 to 245)	5.6 (4.2 to 6.9)	50.9 (-5 to 109.4)	9.4 (-27.1 to 54.9)	119.8 (42.2 to 221.8)	18 (-20.2 to 77.2)
		Female	1898 (1467 to 2466)	129.4 (99.3 to 167.2)	38 (29 to 49)	3.9 (2.8 to 5.1)	2738 (2223 to 3269)	142.2 (116.2 to 167.8)	82 (66 to 101)	4.7 (3.8 to 5.7)	44.2 (1.6 to 99.3)	10 (-23.9 to 55.5)	118.9 (50.6 to 218.3)	20.2 (-20.3 to 81.2)
		Male	2328 (1673 to 3208)	165.5 (117.8 to 215.1)	54 (38 to 70)	5.6 (4 to 7.3)	3638 (2268 to 4737)	182.8 (113.4 to 237.4)	118 (68 to 157)	6.6 (3.8 to 8.8)	56.2 (-19.9 to 161.3)	10.5 (-38.7 to 80.4)	120.5 (15.9 to 265.7)	18 (-35.2 to 92.7)
	Other neoplasms	Both	114 (81 to 143)	5.8 (3.8 to 7.3)	3 (2 to 4)	0.2 (0.1 to 0.3)	188 (156 to 236)	4.8 (4 to 6.1)	6 (5 to 8)	0.2 (0.1 to 0.2)	64.4 (25.3 to 135.9)	-16.2 (-38.2 to 26.4)	98.2 (36.6 to 231.4)	-22.9 (-46.9 to 34.2)
		Female	45 (30 to 59)	4.6 (2.8 to 6.2)	1 (1 to 2)	0.2 (0.1 to 0.2)	75 (61 to 96)	3.8 (3.1 to 4.8)	2 (2 to 3)	0.1 (0.1 to 0.2)	67.9 (18.1 to 167.9)	-16.1 (-42.9 to 50.5)	113.9 (30.3 to 378.4)	-25.8 (-56.3 to 88.5)
		Male	69 (45 to 92)	6.8 (4.2 to 9.2)	2 (1 to 3)	0.3 (0.2 to 0.4)	113 (84 to 154)	5.8 (4.4 to 8)	4 (3 to 6)	0.2 (0.2 to 0.4)	62.2 (11.9 to 142.5)	-14 (-41.6 to 30.2)	90.4 (19 to 217.1)	-19.3 (-49.4 to 31.7)
	Cardiovascular diseases	Both	161269 (135806 to 191867)	10012.9 (8510.1 to 11707.5)	5962 (4979 to 7075)	543.1 (458.3 to 626.6)	215690 (189191 to 246433)	6044 (5329.7 to 6839.6)	10566 (9287 to 11981)	360.6 (314.3 to 405.9)	33.7 (7.4 to 66)	-39.6 (-50.8 to -27.1)	77.2 (42.1 to 116.8)	-33.6 (-45 to -20.8)
		Female	64602 (51927 to 80443)	8851.1 (7194.3 to 10804.8)	2467 (1976 to 3052)	507.1 (415.2 to 610)	92245 (78775 to 108075)	5397.3 (4632.9 to 6260.5)	4931 (4181 to 5733)	350.4 (297.6 to 405.2)	42.8 (7.6 to 87.2)	-39 (-53.1 to -21.1)	99.9 (52.4 to 163.8)	-30.9 (-45.6 to -12.8)
		Male	96667 (73792 to 121261)	11014.6 (8594.1 to 13551.6)	3495 (2654 to 4372)	572 (457.1 to 691.5)	123445 (102490 to 147365)	6687.7 (5609.4 to 7911.8)	5635 (4689 to 6698)	370.9 (310.8 to 436)	27.7 (-4.9 to 72.4)	-39.3 (-54 to -20)	61.2 (20 to 117.5)	-35.2 (-49.4 to -17.9)

Province	Cause	Sex	1990				2019				1990-2019 percent change (%)			
			DALYs		Death		DALYs		Death		DALYs		Death	
			Number	Rate	Number	Rate	Number	Rate	Number	Rate	Number	Rate	Number	Rate
	Rheumatic heart disease	Both	6379 (4857 to 8029)	208.8 (161.2 to 276.7)	113 (85 to 156)	5.6 (4 to 8.5)	3288 (2661 to 4063)	79.3 (64.8 to 97.1)	77 (61 to 94)	2.2 (1.8 to 2.7)	-48.5 (-63.1 to -28)	-62 (-74 to -47.6)	-31.9 (-58.3 to 1.2)	-60.1 (-78.6 to -40)
		Female	3535 (2370 to 4576)	234.1 (158.3 to 315.2)	61 (40 to 84)	6.2 (4 to 9.9)	1659 (1283 to 2100)	81.5 (63.2 to 102.6)	40 (30 to 51)	2.3 (1.7 to 3)	-53.1 (-67.5 to -25.2)	-65.2 (-76.9 to -45.3)	-34.9 (-61.8 to 14.9)	-61.9 (-80.9 to -33.2)
		Male	2845 (1815 to 4037)	183.9 (119.7 to 267.8)	52 (32 to 81)	5 (3.1 to 8.3)	1628 (1250 to 2073)	77.2 (59.6 to 97.4)	37 (28 to 50)	2.1 (1.6 to 2.8)	-42.8 (-63.9 to -1.9)	-58 (-74.4 to -28.3)	-28.3 (-61.2 to 38)	-57.8 (-79 to -21.8)
	Ischemic heart disease	Both	89480 (72806 to 109010)	5673.4 (4726.6 to 6731.1)	3543 (2917 to 4233)	309.7 (258.7 to 359.8)	106057 (91327 to 129526)	2926.1 (2528 to 3538.6)	5297 (4574 to 6436)	178.1 (153.6 to 214.8)	18.5 (-8.6 to 52)	-48.4 (-58.9 to -35.9)	49.5 (17.6 to 86.9)	-42.5 (-52.9 to -30.3)
		Female	30417 (23197 to 38815)	4432.7 (3481.8 to 5514.4)	1301 (1017 to 1622)	263.9 (209.1 to 322.5)	39563 (33016 to 48078)	2328.9 (1950.4 to 2817.5)	2266 (1886 to 2779)	161.4 (135 to 196.9)	30.1 (-5.7 to 79.5)	-47.5 (-60.5 to -28.8)	74.2 (30.5 to 134.9)	-38.8 (-53 to -19.6)
		Male	59063 (43585 to 75893)	6788.9 (5153 to 8518.3)	2243 (1683 to 2839)	351.6 (276.5 to 428.4)	66494 (54001 to 84329)	3523.3 (2869.7 to 4435.2)	3031 (2468 to 3840)	194.9 (160.4 to 243.4)	12.6 (-19.3 to 59.5)	-48.1 (-61.8 to -29.4)	35.2 (-2.1 to 86.1)	-44.6 (-58 to -27.4)
	Stroke	Both	40443 (33845 to 48949)	2710.6 (2215.7 to 3230)	1509 (1219 to 1856)	152.9 (124.4 to 182.7)	50988 (44182 to 59705)	1447.4 (1252.4 to 1692)	2404 (2047 to 2900)	83.2 (70.3 to 100.1)	26.1 (-3.3 to 58.5)	-46.6 (-58.2 to -30.5)	59.3 (19.8 to 113.2)	-45.6 (-57.3 to -27.9)
		Female	18737 (14836 to 23881)	2714.7 (2130.1 to 3417.5)	709 (533 to 924)	155.6 (115.7 to 197.3)	25039 (20853 to 30062)	1447.1 (1205.7 to 1737.6)	1216 (981 to 1499)	85.8 (69.2 to 105.8)	33.6 (-1.2 to 79.3)	-46.7 (-60.1 to -27.9)	71.5 (23.1 to 142.1)	-44.9 (-58.5 to -21.3)
		Male	21706 (16552 to 27887)	2682 (2058.1 to 3455.7)	800 (592 to 1070)	148.2 (112.4 to 190.1)	25949 (21535 to 31260)	1445.9 (1210.6 to 1739.7)	1189 (970 to 1450)	80.6 (66.1 to 97.7)	19.5 (-13.6 to 65.5)	-46.1 (-61.2 to -25.1)	48.5 (3.5 to 118.9)	-45.6 (-61.4 to -21.7)
	Hypertensive heart disease	Both	12213 (9532 to 15634)	904.7 (721.6 to 1150.6)	535 (420 to 683)	56.1 (44.5 to 75)	35821 (21145 to 42068)	1058.2 (603.6 to 1240.7)	2051 (1081 to 2424)	72.5 (37.5 to 85.3)	193.3 (68.3 to 301.6)	17 (-30.6 to 54.1)	283.6 (112.5 to 415.2)	29.2 (-26.3 to 70.8)
		Female	5876 (4192 to 8512)	962.4 (711.5 to 1487.5)	275 (201 to 428)	62.3 (45.4 to 101.8)	17126 (9877 to 21036)	1041.5 (572.7 to 1269.4)	1057 (546 to 1307)	76.4 (38.2 to 93.3)	191.5 (61.3 to 339.8)	8.2 (-42 to 59.6)	285.1 (95.9 to 473.5)	22.6 (-39.1 to 83)
		Male	6337 (4426 to 8823)	841.1 (600.7 to 1137.4)	260 (182 to 362)	49.1 (34.7 to 65.6)	18695 (9750 to 23297)	1074.5 (534.5 to 1333.7)	994 (468 to 1243)	68.6 (31.8 to 84.9)	195 (53.2 to 351.4)	27.8 (-31.7 to 85.8)	282 (91 to 475.4)	39.7 (-26.3 to 99.6)
	Non-rheumatic valvular heart disease	Both	1097 (815 to 1432)	55.8 (43.7 to 68.9)	34 (26 to 42)	2.5 (2.1 to 3.1)	1791 (1515 to 2122)	45.9 (39 to 54.5)	73 (62 to 87)	2.2 (1.9 to 2.6)	63.3 (17 to 132.4)	-17.8 (-37.7 to 11.5)	116.6 (64.5 to 194.7)	-12.4 (-32.2 to 14)
		Female	570 (367 to 812)	63 (44.6 to 84.5)	18 (13 to 24)	3 (2.2 to 4)	882 (692 to 1115)	46.5 (37 to 58.4)	39 (31 to 50)	2.4 (1.9 to 3.1)	54.8 (0.5 to 157.8)	-26.2 (-49 to 11.9)	113.6 (47.6 to 224.2)	-19.1 (-45.4 to 19)
		Male	527 (339 to 754)	48.6 (33.5 to 67.4)	16 (11 to 22)	2 (1.5 to 2.7)	909 (685 to 1154)	45.2 (34.5 to 56.4)	35 (26 to 43)	2 (1.6 to 2.5)	72.5 (7.7 to 169.6)	-7.1 (-40.8 to 39.9)	120.1 (39.4 to 234.3)	-0.9 (-34.7 to 44.9)
	Cardiomyopathy and myocarditis	Both	2744 (1535 to 4434)	94.2 (59 to 129.6)	56 (36 to 77)	3.3 (2.2 to 4.4)	2557 (1548 to 3141)	67.7 (41 to 83.4)	88 (51 to 110)	2.7 (1.6 to 3.4)	-6.8 (-54 to 51.7)	-28.2 (-58.2 to 5.2)	57 (-8.9 to 131.3)	-18.8 (-47.3 to 11.4)
		Female	1164 (610 to 2140)	81.4 (48.5 to 128.1)	23 (14 to 37)	3 (1.9 to 4.7)	964 (551 to 1264)	53.1 (30.1 to 68.8)	35 (19 to 46)	2.2 (1.2 to 3)	-17.2 (-65.7 to 53.9)	-34.8 (-66.1 to 8)	51.1 (-21.9 to 157.4)	-25.6 (-64.7 to 32.9)
		Male	1579 (807 to 2506)	105.8 (58.1 to 149.9)	33 (18 to 47)	3.6 (2 to 4.9)	1593 (907 to 2072)	82 (47.4 to 105.3)	53 (30 to 69)	3.2 (1.8 to 4.2)	0.9 (-50.9 to 71.7)	-22.4 (-55.7 to 20.5)	61.2 (-8.1 to 144)	-12.2 (-41 to 25.4)
	Atrial fibrillation and flutter	Both	873 (672 to 1143)	81.4 (62.4 to 102.8)	25 (17 to 31)	3.8 (2.6 to 4.8)	2759 (2194 to 3471)	87.9 (70.8 to 108.8)	106 (90 to 126)	4.5 (3.7 to 5.4)	215.9 (175.7 to 280.9)	8 (-7.1 to 35.5)	329.3 (224.7 to 563.3)	16.8 (-11.9 to 87.2)

Province	Cause	Sex	1990				2019				1990-2019 percent change (%)			
			DALYs		Death		DALYs		Death		DALYs		Death	
			Number	Rate	Number	Rate	Number	Rate	Number	Rate	Number	Rate	Number	Rate
		Female	465 (342 to 609)	95.7 (69.3 to 121.7)	16 (10 to 22)	5 (3.1 to 6.5)	1537 (1196 to 1935)	98.2 (77.7 to 123)	64 (52 to 84)	5.3 (4.2 to 7.2)	230.6 (169.1 to 348.5)	2.5 (-18.1 to 46.9)	291.8 (170.8 to 634.1)	6.1 (-26.4 to 105.8)
		Male	408 (294 to 551)	66.1 (49.8 to 87.1)	8 (6 to 11)	2.5 (1.7 to 3.3)	1221 (924 to 1553)	77.5 (58.6 to 96.9)	42 (27 to 52)	3.7 (2.3 to 4.6)	199.2 (141 to 267.2)	17.3 (-6.3 to 43)	402 (214.9 to 644.6)	46.4 (-5.1 to 112.8)
	Aortic aneurysm	Both	471 (332 to 634)	27.3 (19.9 to 35.8)	17 (13 to 23)	1.4 (1 to 1.8)	1307 (1075 to 1623)	33.3 (27.6 to 40.9)	55 (46 to 68)	1.6 (1.4 to 2)	177.7 (90 to 325.3)	21.8 (-14.5 to 81.9)	215.9 (120.5 to 372.5)	18.5 (-14.7 to 73.3)
		Female	116 (86 to 155)	15.4 (11.9 to 19.7)	5 (4 to 6)	0.9 (0.7 to 1.1)	364 (288 to 460)	18.7 (15 to 23.5)	15 (12 to 20)	0.9 (0.7 to 1.2)	212.3 (112.1 to 359.9)	21.1 (-15.2 to 73)	239.6 (138.4 to 383.5)	8.7 (-21.1 to 56.4)
		Male	354 (228 to 514)	38.1 (25.1 to 54.3)	13 (8 to 18)	1.9 (1.2 to 2.6)	943 (715 to 1226)	48 (36.6 to 61.6)	40 (30 to 51)	2.3 (1.8 to 3)	166.4 (60.6 to 370.1)	26 (-21.7 to 115.2)	207.5 (91.2 to 436.7)	25.7 (-19.6 to 113.1)
	Peripheral artery disease	Both	83 (45 to 142)	7 (3.9 to 11.7)	1 (1 to 1)	0.1 (0.1 to 0.2)	265 (165 to 417)	8 (5 to 12.5)	5 (4 to 7)	0.2 (0.2 to 0.3)	218.7 (174.3 to 286.3)	14.1 (0.7 to 35.5)	615.6 (387.1 to 940.5)	108.3 (34.6 to 217)
		Female	45 (23 to 80)	8.2 (4.4 to 14.4)	0 (0 to 1)	0.1 (0 to 0.2)	136 (76 to 227)	8.2 (4.6 to 13.6)	2 (1 to 3)	0.2 (0.1 to 0.3)	202.1 (159.8 to 258)	-0.6 (-14.2 to 18.3)	495.2 (207.6 to 1175.6)	65.7 (-18.6 to 276.8)
		Male	38 (22 to 62)	5.9 (3.5 to 9.6)	0 (0 to 1)	0.1 (0.1 to 0.1)	129 (89 to 189)	7.8 (5.3 to 11.5)	3 (3 to 4)	0.2 (0.2 to 0.3)	238.1 (170.5 to 349.2)	33.3 (10.3 to 68.8)	704.2 (414.5 to 1160.2)	158.5 (70.6 to 301.9)
	Endocarditis	Both	653 (442 to 964)	22.2 (15.5 to 34.1)	13 (9 to 20)	0.7 (0.5 to 1.2)	609 (462 to 952)	15.5 (12 to 23.5)	22 (17 to 34)	0.7 (0.5 to 1)	-6.8 (-40.4 to 59.5)	-30.1 (-49 to -3.9)	68.6 (23 to 126.8)	-10.3 (-34.2 to 28.1)
		Female	359 (177 to 571)	23.5 (15.8 to 36.6)	7 (5 to 10)	0.8 (0.5 to 1.5)	280 (205 to 460)	14.9 (11.1 to 23.7)	11 (8 to 18)	0.7 (0.5 to 1.1)	-22.1 (-57.1 to 76)	-36.5 (-59 to -6.6)	68.3 (11.4 to 147.4)	-8.1 (-39.3 to 45.3)
		Male	294 (173 to 537)	20.7 (12.4 to 37.8)	6 (4 to 12)	0.7 (0.4 to 1.2)	329 (229 to 541)	16 (11.3 to 26.1)	11 (8 to 18)	0.6 (0.4 to 1)	11.9 (-30.5 to 81.8)	-22.9 (-49.9 to 21.4)	68.8 (11 to 159.7)	-11.5 (-39.8 to 35.4)
	Other cardiovascular and circulatory diseases	Both	6833 (5304 to 9307)	227.4 (182.2 to 315.4)	116 (92 to 170)	6.9 (5.4 to 10.2)	10249 (8700 to 12032)	274.8 (233.8 to 321.7)	386 (315 to 464)	12.6 (10.2 to 15.3)	50 (12.9 to 100.1)	20.8 (-11.3 to 57)	232.9 (126.1 to 359.8)	84 (14.3 to 151.4)
		Female	3318 (2440 to 4364)	219.9 (171.2 to 283.7)	53 (40 to 70)	6.4 (4.9 to 10.1)	4696 (3797 to 5854)	258.9 (210.7 to 322.3)	186 (144 to 237)	12.7 (9.7 to 16.1)	41.5 (0.2 to 102)	17.7 (-19.2 to 62.1)	251.2 (116.7 to 411.4)	98.7 (1.3 to 183.4)
		Male	3514 (2580 to 5727)	233.6 (176 to 407.1)	63 (46 to 118)	7.3 (5.2 to 13.9)	5554 (4547 to 6778)	290.3 (238.9 to 353.3)	200 (158 to 250)	12.6 (10 to 15.8)	58 (-5.4 to 128.1)	24.3 (-30.8 to 76.4)	217.4 (61.8 to 384.4)	72.5 (-14 to 159.4)
	Chronic respiratory diseases	Both	29234 (24117 to 34446)	1579.8 (1265.9 to 1884.3)	746 (555 to 918)	72.2 (51.2 to 90.2)	40256 (33183 to 45686)	1114.5 (894.4 to 1264.6)	1413 (1009 to 1653)	49.3 (34.2 to 57.8)	37.7 (14.5 to 64)	-29.5 (-41 to -15.1)	89.5 (46.2 to 144.5)	-31.7 (-47.5 to -12.5)
		Female	13224 (9952 to 16175)	1477.6 (1031 to 1920.6)	321 (199 to 437)	66.5 (37.5 to 95.1)	17831 (13823 to 20950)	997.3 (744.2 to 1176.6)	609 (346 to 777)	43.2 (23.2 to 54.9)	34.8 (7.1 to 77.4)	-32.5 (-49 to -10.6)	89.4 (26.7 to 185)	-35 (-58.3 to -5.2)
		Male	16010 (12824 to 19556)	1668 (1312.5 to 2070.9)	424 (313 to 552)	77.5 (57.9 to 99.9)	22425 (18187 to 26272)	1233.7 (994.7 to 1440.5)	804 (596 to 975)	55.6 (40.1 to 66.7)	40.1 (9.9 to 77.6)	-26 (-43.2 to -5)	89.6 (31.6 to 170.5)	-28.3 (-48.7 to -3.8)
	Chronic obstructive pulmonary disease	Both	11425 (9416 to 13302)	727.9 (587.4 to 853.7)	345 (260 to 421)	36.2 (26.5 to 44.7)	26390 (21142 to 29976)	751.4 (592.6 to 851.7)	1059 (744 to 1249)	37.8 (25.9 to 44.4)	131 (88.3 to 181.9)	3.2 (-14.9 to 26.3)	207.1 (132.5 to 308.7)	4.5 (-19.7 to 35.2)
		Female	4923 (3495 to 6181)	662.3 (456.6 to 834.9)	144 (83 to 195)	32.8 (18.1 to 44.1)	11169 (8170 to 13560)	645.9 (455.1 to 786.7)	441 (237 to 585)	32.3 (16.4 to 42.5)	126.9 (75.5 to 238.7)	-2.5 (-25.8 to 41.8)	206.8 (102.1 to 428.9)	-1.4 (-33.7 to 60.8)
		Male	6503 (5011 to 8284)	785.7 (604.9 to 991.9)	201 (144 to 266)	39.5 (29.3 to 51.7)	15220 (12130 to 17741)	858.7 (681.4 to 996.5)	618 (455 to 751)	43.5 (31.9 to 52.3)	134.1 (76.8 to 210.9)	9.3 (-16.9 to 43.3)	207.3 (114.2 to 339)	10 (-21.6 to 50.9)

Province	Cause	Sex	1990				2019				1990-2019 percent change (%)			
			DALYs		Death		DALYs		Death		DALYs		Death	
			Number	Rate	Number	Rate	Number	Rate	Number	Rate	Number	Rate	Number	Rate
	Pneumoconiosis	Both	9 (6 to 12)	0.5 (0.4 to 0.7)	0 (0 to 0)	0 (0 to 0)	64 (49 to 84)	1.6 (1.2 to 2.1)	1 (1 to 2)	0 (0 to 0.1)	616.5 (396 to 938.6)	214.6 (120.6 to 358.3)	405.1 (211.7 to 826.6)	116.6 (38 to 274.9)
		Female	3 (2 to 4)	0.3 (0.2 to 0.4)	0 (0 to 0)	0 (0 to 0)	40 (27 to 58)	1.9 (1.3 to 2.7)	0 (0 to 0)	0 (0 to 0)	1462.7 (1131 to 1895.3)	636.3 (485.5 to 822)	1213.8 (720.9 to 2242.8)	365.1 (174.1 to 719.8)
		Male	6 (4 to 9)	0.7 (0.5 to 1)	0 (0 to 0)	0 (0 to 0)	24 (18 to 31)	1.3 (1 to 1.7)	1 (1 to 1)	0.1 (0 to 0.1)	276.6 (134.5 to 565.2)	79.8 (15.7 to 205.1)	325 (149.2 to 746.9)	89.7 (15.4 to 264.6)
	Asthma	Both	17177 (13483 to 21246)	828.6 (629 to 1065.5)	393 (276 to 518)	35.4 (23.1 to 50)	12125 (9602 to 15150)	320.5 (253.7 to 397.7)	323 (237 to 400)	10.6 (7.7 to 13.3)	-29.4 (-41.9 to -12.7)	-61.3 (-70.7 to -50)	-17.9 (-43.5 to 19.2)	-70.2 (-80.8 to -56.3)
		Female	7996 (5772 to 10245)	793 (509 to 1148.6)	175 (94 to 270)	33.3 (16.1 to 57.8)	5845 (4443 to 7385)	311 (236.9 to 391)	156 (97 to 204)	10.2 (6.1 to 13.8)	-26.9 (-44.7 to -6.3)	-60.8 (-74.7 to -44.6)	-10.8 (-49.6 to 42.8)	-69.3 (-84.6 to -47.7)
		Male	9181 (7094 to 11632)	858 (643.1 to 1101.8)	219 (153 to 292)	37.3 (26.2 to 50.6)	6280 (4850 to 7950)	330 (257.3 to 417.8)	167 (122 to 213)	10.9 (7.8 to 13.8)	-31.6 (-46.9 to -11.5)	-61.5 (-71.6 to -47.7)	-23.6 (-49.8 to 19.1)	-70.8 (-81.4 to -55.5)
	Interstitial lung disease and pulmonary sarcoidosis	Both	203 (152 to 274)	10.5 (7.6 to 15.2)	5 (3 to 8)	0.4 (0.2 to 0.7)	572 (388 to 724)	14.4 (9.7 to 17.9)	19 (11 to 24)	0.6 (0.3 to 0.7)	181.5 (79.9 to 304.5)	36.3 (-13 to 109.3)	285.1 (111.3 to 588.5)	42 (-23 to 178.8)
		Female	89 (62 to 128)	9.5 (6.2 to 15.4)	2 (1 to 4)	0.3 (0.2 to 0.7)	257 (169 to 335)	12.8 (8.2 to 16.5)	8 (4 to 11)	0.5 (0.2 to 0.6)	190.1 (90.6 to 343.7)	33.9 (-20.1 to 126.5)	293.2 (94.4 to 755.1)	33.8 (-39.6 to 247.5)
		Male	115 (82 to 168)	11.5 (7.9 to 17.9)	3 (2 to 5)	0.4 (0.3 to 0.8)	315 (179 to 414)	16 (9.2 to 21)	11 (5 to 15)	0.7 (0.3 to 0.9)	174.8 (49.5 to 334.7)	39.4 (-25.1 to 136.8)	279.6 (80.5 to 645.4)	49.5 (-29.9 to 209.6)
	Other chronic respiratory diseases	Both	420 (294 to 774)	12.3 (9 to 20.4)	2 (1 to 7)	0.1 (0.1 to 0.3)	1105 (861 to 1349)	26.6 (20.4 to 32.9)	11 (4 to 17)	0.3 (0.1 to 0.5)	163.2 (27.2 to 283)	115.8 (14.7 to 205.7)	363.6 (-26.6 to 1430)	193.7 (-41.5 to 756.7)
		Female	214 (147 to 395)	12.5 (9.1 to 20.6)	1 (0 to 3)	0.1 (0 to 0.2)	520 (418 to 627)	25.7 (20.3 to 31.3)	4 (2 to 6)	0.2 (0.1 to 0.3)	143 (21.3 to 243.5)	104.8 (14.2 to 183)	302.1 (-34.5 to 1410.8)	142.7 (-43.8 to 647.2)
		Male	206 (133 to 376)	12.1 (8.3 to 20.7)	1 (1 to 4)	0.1 (0.1 to 0.3)	585 (417 to 751)	27.6 (19.4 to 35.6)	7 (2 to 12)	0.4 (0.1 to 0.7)	184.2 (29.3 to 369.5)	128 (11.5 to 261.1)	403.8 (-26.3 to 1735.5)	230.5 (-43.9 to 1001.8)
	Digestive diseases	Both	20332 (16339 to 24123)	860.7 (710.7 to 1022.3)	426 (342 to 515)	30.3 (23.7 to 39.1)	23988 (20040 to 28687)	601.6 (509.3 to 711.5)	692 (585 to 815)	21.5 (18.3 to 25.1)	18 (-4.8 to 55.1)	-30.1 (-41.7 to -11.7)	62.6 (27.3 to 118.9)	-29.2 (-46.5 to -6.3)
		Female	9284 (7240 to 11297)	804.6 (628 to 1011.1)	179 (131 to 235)	28.2 (20.1 to 41.3)	10765 (8804 to 13184)	551.2 (454 to 663.4)	297 (244 to 362)	19.2 (15.9 to 23.2)	15.9 (-11.6 to 55.3)	-31.5 (-45.8 to -11.4)	66.2 (19.6 to 136.5)	-32 (-55.5 to 0.2)
		Male	11048 (8372 to 13756)	907.7 (700.1 to 1110.6)	247 (183 to 309)	31.9 (23.8 to 40.9)	13223 (10683 to 16013)	652.6 (530 to 786.7)	395 (315 to 486)	23.8 (19.2 to 28.8)	19.7 (-10.3 to 69.4)	-28.1 (-44.9 to -1.8)	60 (15.8 to 137.9)	-25.6 (-44.8 to 4.9)
	Cirrhosis and other chronic liver diseases	Both	8822 (6762 to 10796)	364.3 (287.9 to 445.6)	226 (176 to 280)	14.5 (10.8 to 19.1)	10496 (8777 to 12537)	265.2 (221.8 to 315.2)	408 (342 to 487)	12 (10.1 to 14.3)	19 (-12.8 to 75.1)	-27.2 (-44.3 to 1)	80.9 (36.7 to 150.5)	-17.4 (-40.1 to 14.4)
		Female	3947 (2696 to 5186)	327.2 (233.1 to 439.4)	96 (68 to 129)	13.5 (8.8 to 20.4)	4060 (3262 to 5045)	214.4 (175.1 to 264)	168 (136 to 209)	10.3 (8.3 to 12.7)	2.9 (-29 to 64.7)	-34.5 (-53.8 to -2.7)	75.2 (22.3 to 171)	-24.1 (-52.6 to 21.3)
		Male	4875 (3415 to 6410)	396.1 (285.2 to 516.3)	130 (94 to 171)	15.2 (11.1 to 20.1)	6436 (5009 to 8113)	316.2 (247.1 to 396.5)	241 (188 to 301)	13.7 (10.8 to 17)	32 (-10.4 to 111.5)	-20.2 (-44.5 to 22.2)	85 (29.3 to 183.7)	-9.9 (-35.6 to 28.8)
	Upper digestive system diseases	Both	6564 (5040 to 8686)	308.2 (234.7 to 399.2)	124 (92 to 162)	10.4 (7.4 to 14.1)	7002 (4843 to 10510)	167.1 (119.1 to 242.9)	109 (88 to 129)	3.7 (3 to 4.4)	6.7 (-16.5 to 28.7)	-45.8 (-58.1 to -33.8)	-11.8 (-35 to 22.4)	-64.4 (-75.1 to -50.5)
		Female	2912 (2075 to 3942)	283.7 (202.3 to 393.4)	48 (33 to 70)	9.2 (5.9 to 14.5)	3488 (2322 to 5270)	168.3 (116.4 to 248.5)	50 (38 to 64)	3.5 (2.7 to 4.4)	19.8 (-8.5 to 47.9)	-40.7 (-56.3 to -24.8)	2.4 (-35.4 to 62.4)	-62.3 (-78.4 to -34.8)

Province	Cause	Sex	1990				2019				1990-2019 percent change (%)			
			DALYs		Death		DALYs		Death		DALYs		Death	
			Number	Rate	Number	Rate	Number	Rate	Number	Rate	Number	Rate	Number	Rate
		Male	3653 (2707 to 4829)	329.6 (244.6 to 429.8)	75 (52 to 102)	11.4 (7.8 to 15.6)	3514 (2463 to 5126)	166.1 (119.2 to 236.8)	60 (46 to 75)	3.9 (3 to 4.8)	-3.8 (-27.7 to 25.8)	-49.6 (-62.3 to -34.6)	-20.9 (-46.2 to 19.9)	-65.7 (-76.5 to -49.3)
	Appendicitis	Both	726 (380 to 1097)	21.8 (13.3 to 30.4)	12 (7 to 18)	0.6 (0.4 to 1)	362 (285 to 458)	9.3 (7.3 to 11.9)	8 (7 to 12)	0.3 (0.2 to 0.4)	-50.2 (-70.2 to 10.6)	-57.2 (-72 to -14.9)	-32.1 (-56.7 to 44.3)	-59.4 (-74.3 to -28.8)
		Female	300 (151 to 556)	18.6 (11 to 31)	5 (3 to 9)	0.6 (0.3 to 1.1)	160 (120 to 220)	8.7 (6.5 to 12)	4 (3 to 6)	0.2 (0.2 to 0.4)	-46.8 (-69.8 to 27.2)	-53.5 (-73.5 to -7.4)	-27.3 (-62.2 to 74)	-57.9 (-79.9 to -19.2)
		Male	426 (200 to 674)	24.8 (12.9 to 35.8)	7 (4 to 11)	0.7 (0.4 to 1)	202 (152 to 276)	10 (7.6 to 13.6)	5 (3 to 7)	0.3 (0.2 to 0.4)	-52.6 (-73.8 to 19.6)	-59.6 (-75.1 to -7.4)	-35.4 (-62.8 to 73.5)	-60.2 (-75.8 to -12)
	Paralytic ileus and intestinal obstruction	Both	1712 (1019 to 2787)	50.2 (34.4 to 76.1)	27 (19 to 42)	1.6 (1.2 to 2.3)	1139 (764 to 1472)	33.6 (22.8 to 43.4)	44 (30 to 56)	1.5 (1 to 1.9)	-33.5 (-67.7 to 30.1)	-33 (-65.3 to 12.4)	60.7 (-15.6 to 164)	-7.9 (-46.4 to 41.9)
		Female	835 (452 to 1500)	50.7 (33.1 to 81.4)	13 (9 to 21)	1.7 (1.1 to 2.5)	476 (340 to 629)	28.9 (20.5 to 38.4)	18 (13 to 24)	1.2 (0.9 to 1.6)	-43 (-72.3 to 23.5)	-43.1 (-69.2 to 4.3)	33.8 (-24 to 142.9)	-27.2 (-56.1 to 26)
		Male	877 (450 to 1499)	49.5 (31.1 to 80.5)	14 (9 to 23)	1.5 (1 to 2.4)	664 (376 to 905)	38.4 (22.3 to 52.3)	26 (14 to 35)	1.7 (1 to 2.3)	-24.3 (-69.7 to 76)	-22.4 (-66.5 to 51)	86.3 (-21.9 to 246.9)	15 (-44.9 to 89.4)
	Inguinal, femoral, and abdominal hernia	Both	532 (336 to 800)	17 (11.7 to 23.5)	2 (2 to 4)	0.2 (0.1 to 0.3)	702 (485 to 1000)	17.4 (12.2 to 24.3)	6 (3 to 8)	0.2 (0.1 to 0.3)	32 (-8.9 to 79.3)	1.9 (-20.9 to 25.4)	131.8 (0.8 to 347.3)	-6.7 (-44.3 to 50)
		Female	202 (116 to 355)	12.3 (8 to 18.6)	1 (0 to 2)	0.2 (0.1 to 0.3)	238 (160 to 349)	12.1 (8.1 to 17.6)	2 (1 to 3)	0.2 (0.1 to 0.2)	18.1 (-33.6 to 79.3)	-1.6 (-36 to 34.1)	145.9 (-31.6 to 458.5)	-1.9 (-41.6 to 76.8)
		Male	330 (209 to 489)	21.5 (14.6 to 29.6)	2 (1 to 2)	0.3 (0.2 to 0.5)	464 (319 to 659)	22.6 (15.7 to 31.6)	3 (2 to 5)	0.2 (0.1 to 0.3)	40.5 (5.2 to 90.9)	5.2 (-14.6 to 32.4)	123.5 (3.8 to 342.8)	-10.2 (-54.3 to 59.6)
	Inflammatory bowel disease	Both	204 (150 to 282)	9.1 (6.8 to 12.2)	2 (1 to 3)	0.1 (0.1 to 0.2)	464 (324 to 614)	10.5 (7.4 to 13.7)	7 (3 to 9)	0.2 (0.1 to 0.2)	126.9 (54.1 to 190.5)	15.5 (-13 to 46.2)	223.4 (43.4 to 437.6)	43.4 (-25.4 to 141.4)
		Female	98 (68 to 143)	8.9 (6.2 to 12.6)	1 (0 to 1)	0.1 (0.1 to 0.2)	208 (146 to 284)	9.5 (6.7 to 12.8)	2 (1 to 3)	0.1 (0.1 to 0.2)	111.5 (48.9 to 175.6)	6.6 (-19.8 to 37.7)	206.1 (48.4 to 480.6)	30.4 (-40.1 to 160.8)
		Male	106 (77 to 146)	9.3 (6.7 to 12.7)	1 (1 to 2)	0.2 (0.1 to 0.2)	256 (161 to 339)	11.6 (7.2 to 15.2)	4 (2 to 6)	0.2 (0.1 to 0.3)	141.2 (47.7 to 231.9)	24.8 (-16.7 to 69.1)	234.1 (28.6 to 508.3)	54.7 (-34.7 to 176.5)
	Vascular intestinal disorders	Both	142 (97 to 195)	9.9 (6.3 to 14.5)	6 (4 to 9)	0.6 (0.4 to 0.9)	366 (292 to 442)	10.2 (8.2 to 12.3)	19 (15 to 23)	0.6 (0.5 to 0.7)	158.3 (79.5 to 267.5)	2.8 (-31.8 to 55.9)	214.4 (107.8 to 375.6)	4.8 (-33.6 to 71.1)
		Female	62 (36 to 100)	10.2 (5.1 to 17.5)	3 (1 to 5)	0.6 (0.3 to 1.1)	163 (126 to 208)	9.3 (7.3 to 11.7)	9 (7 to 11)	0.6 (0.5 to 0.7)	162 (59.8 to 331.7)	-9.6 (-48.8 to 72.2)	203.6 (65 to 488.2)	-10 (-52.5 to 99.7)
		Male	80 (54 to 111)	9.6 (6.6 to 13.2)	3 (2 to 4)	0.5 (0.4 to 0.7)	204 (147 to 260)	11.2 (8.2 to 14.2)	10 (7 to 13)	0.6 (0.5 to 0.8)	155.5 (68 to 287.8)	17.1 (-21.7 to 72.4)	224.4 (113.3 to 397.5)	25.1 (-15.1 to 83.9)
	Gallbladder and biliary diseases	Both	1045 (699 to 1489)	50.5 (35.2 to 70)	10 (7 to 17)	1 (0.7 to 1.6)	2178 (1607 to 2987)	54.4 (41.1 to 73)	42 (32 to 52)	1.5 (1.2 to 1.9)	108.4 (59.9 to 156)	7.7 (-16.1 to 31.7)	314.4 (125.5 to 595.7)	48 (-12.2 to 137.7)
		Female	683 (446 to 979)	66.1 (45.1 to 92.5)	5 (3 to 9)	1.1 (0.6 to 1.8)	1421 (1008 to 2003)	69.8 (50.7 to 95.6)	22 (16 to 29)	1.6 (1.2 to 2.1)	108.2 (61.2 to 154.6)	5.7 (-15.6 to 28.6)	351.7 (125.3 to 686.5)	53.3 (-15.2 to 167.1)
		Male	363 (242 to 523)	35.7 (25 to 50)	5 (3 to 9)	1 (0.6 to 1.6)	757 (551 to 976)	39.3 (29.3 to 49.8)	20 (14 to 26)	1.4 (1 to 1.8)	108.8 (46.4 to 185.8)	10.2 (-23.2 to 50.5)	279.2 (99 to 606.8)	44.6 (-20.3 to 162.5)
	Pancreatitis	Both	333 (235 to 490)	18.2 (12.6 to 27.2)	10 (7 to 16)	0.8 (0.5 to 1.3)	685 (517 to 818)	17.8 (13.1 to 21.2)	28 (20 to 35)	0.9 (0.6 to 1.1)	105.9 (33 to 217.2)	-2.4 (-37.9 to 52.4)	171.8 (64.4 to 347.3)	7.5 (-40.7 to 79.7)
		Female	132 (86 to 210)	16.1 (9.9 to 26)	4 (3 to 7)	0.8 (0.5 to 1.5)	297 (192 to 379)	16.3 (10.6 to 20.6)	14 (9 to 18)	0.9 (0.6 to 1.2)	125.6 (32 to 273.8)	1 (-45.3 to 75)	217.8 (62.6 to 483.7)	11.5 (-48.5 to 115.4)
		Male	201 (130 to 312)	19.9 (13.2 to 30)	6 (4 to 9)	0.8 (0.5 to 1.2)	388 (281 to 496)	19.2 (13.5 to 24.3)	14 (9 to 19)	0.9 (0.5 to 1.1)	93 (19 to 229.7)	-3.7 (-39.9 to 59.7)	138.5 (39.1 to 324.6)	6.6 (-35.3 to 78.6)
	Other digestive diseases	Both	252 (189 to 410)	11.3 (8.6 to 16.3)	5 (4 to 8)	0.5 (0.3 to 0.7)	593 (467 to 719)	16 (12.7 to 19.2)	21 (16 to 25)	0.7 (0.5 to 0.8)	135.5 (18 to 240.1)	41.4 (-17 to 94.4)	292.4 (108.7 to 485.6)	52.6 (-10.1 to 146.1)

Province	Cause	Sex	1990				2019				1990-2019 percent change (%)			
			DALYs		Death		DALYs		Death		DALYs		Death	
			Number	Rate	Number	Rate	Number	Rate	Number	Rate	Number	Rate	Number	Rate
		Female	114 (84 to 191)	10.7 (7.4 to 17)	2 (1 to 4)	0.4 (0.2 to 0.8)	254 (196 to 322)	14 (10.9 to 17.6)	9 (6 to 11)	0.6 (0.4 to 0.8)	123.4 (30.5 to 233)	30.7 (-19.9 to 83.9)	280.1 (101.7 to 559.8)	32.6 (-33.9 to 176.3)
		Male	138 (98 to 218)	11.7 (8.4 to 16.4)	3 (2 to 5)	0.4 (0.3 to 0.6)	339 (240 to 433)	18 (12.9 to 22.8)	12 (9 to 16)	0.8 (0.6 to 1)	145.5 (24.8 to 288)	53 (-12.6 to 128.9)	301.4 (121.6 to 568.2)	76.6 (0.3 to 177.8)
	Neurological disorders	Both	43328 (24393 to 70590)	1621.3 (981.7 to 2596.3)	349 (202 to 655)	35.1 (15.4 to 79.3)	56668 (30983 to 94590)	1472.4 (843.1 to 2389.5)	907 (429 to 1986)	35.6 (15.3 to 79.4)	30.8 (-0.5 to 68.6)	-9.2 (-25.5 to 7.8)	159.9 (73.9 to 279.8)	1.6 (-14.3 to 45.3)
		Female	22823 (11998 to 38926)	1737.4 (983.6 to 2882.8)	165 (88 to 327)	33.8 (13.8 to 81.1)	31470 (15656 to 53961)	1627.4 (856.2 to 2778.2)	445 (193 to 1009)	35.1 (14 to 80.2)	37.9 (4 to 77.5)	-6.3 (-23.3 to 11)	169.6 (69 to 327.9)	3.9 (-19 to 61.2)
		Male	20505 (12098 to 31863)	1509.3 (965.1 to 2363.6)	184 (105 to 331)	36.4 (16.8 to 80.2)	25197 (14870 to 39743)	1324.1 (807.3 to 2094.8)	462 (236 to 973)	36.2 (16.8 to 79.5)	22.9 (-7.8 to 65.7)	-12.3 (-28.7 to 7.5)	151.3 (67.6 to 261.6)	-0.8 (-19.4 to 35.8)
	Alzheimer's disease and other dementias	Both	3181 (1359 to 7399)	383.6 (165.8 to 874.6)	160 (37 to 447)	25.4 (5.9 to 69)	11100 (4839 to 25610)	395.7 (172.4 to 885.9)	610 (144 to 1680)	26.4 (6.3 to 71.4)	249 (196.9 to 350.7)	3.1 (-8.5 to 35.1)	282.2 (215.6 to 455.6)	3.9 (-11.6 to 55.3)
		Female	1584 (682 to 3602)	390.7 (170.9 to 918.1)	82 (19 to 233)	25.7 (5.8 to 72.4)	5890 (2546 to 13504)	413.9 (178.8 to 925.8)	322 (73 to 882)	27.5 (6.5 to 73.3)	272 (202.1 to 407.8)	5.9 (-11 to 48.3)	290.1 (202.2 to 530)	7.3 (-14.8 to 78.9)
		Male	1597 (692 to 3777)	377.1 (162.2 to 843.6)	77 (18 to 220)	25.2 (5.8 to 68.1)	5209 (2296 to 12071)	377.1 (165 to 853.2)	289 (68 to 795)	25.3 (6.1 to 68.8)	226.1 (167.5 to 312.4)	0 (-14.1 to 24.1)	273.8 (193.4 to 420.9)	0.3 (-17.2 to 40.4)
	Parkinson's disease	Both	892 (735 to 1057)	83.2 (69.6 to 99.6)	42 (34 to 51)	5.3 (4.4 to 6.8)	3163 (2629 to 3637)	96.8 (81.7 to 110.9)	189 (159 to 219)	6.5 (5.5 to 7.5)	254.8 (178.4 to 353.4)	16.4 (-10.7 to 46.6)	348.6 (239.7 to 484.6)	23.2 (-8.6 to 59.2)
		Female	337 (263 to 446)	65.4 (50.9 to 92.5)	16 (12 to 24)	4.1 (3.1 to 6.6)	1290 (1077 to 1531)	77.9 (65.2 to 92.4)	76 (63 to 92)	5.2 (4.2 to 6.3)	282.8 (179.2 to 418)	19 (-13.8 to 60.9)	372.1 (231 to 569.1)	25.4 (-11.7 to 76.6)
		Male	555 (415 to 696)	101.2 (77.5 to 124.3)	26 (20 to 33)	6.6 (5 to 8.2)	1874 (1381 to 2262)	116 (86.2 to 138.8)	114 (82 to 139)	7.9 (5.8 to 9.6)	237.8 (143.3 to 369.2)	14.6 (-17.4 to 58.6)	334.2 (198.2 to 533)	20.2 (-17.2 to 71)
	Idiopathic epilepsy	Both	15121 (7204 to 23165)	365.1 (183.6 to 566.7)	126 (53 to 167)	3.4 (1.7 to 4.4)	7178 (3694 to 12034)	182.9 (93.4 to 307.6)	58 (37 to 72)	1.5 (0.9 to 1.8)	-52.5 (-76.6 to 0.1)	-49.9 (-75.8 to 5)	-53.9 (-66.5 to -18.5)	-56.4 (-67.1 to -29.8)
		Female	6953 (3267 to 10642)	341.5 (169.8 to 528.3)	58 (18 to 83)	3.2 (1.3 to 4.3)	3245 (1652 to 5487)	170.9 (86.5 to 290.5)	25 (15 to 32)	1.3 (0.8 to 1.7)	-53.3 (-77.8 to 4.6)	-49.9 (-76.1 to 11.5)	-56.7 (-71.8 to -1.5)	-58.2 (-71.2 to -19.5)
		Male	8168 (3731 to 12694)	387.6 (186.9 to 603.8)	68 (28 to 96)	3.6 (1.7 to 4.9)	3933 (1994 to 6654)	194.6 (96.5 to 327)	33 (20 to 43)	1.7 (1 to 2.1)	-51.9 (-77.1 to 3.3)	-49.8 (-76 to 6.7)	-51.6 (-67.7 to -8.2)	-54.6 (-68.2 to -22.1)
	Multiple sclerosis	Both	808 (613 to 1068)	34.5 (26.1 to 46.7)	11 (8 to 18)	0.6 (0.4 to 0.9)	1758 (1464 to 2091)	37.6 (31.3 to 44.6)	30 (23 to 37)	0.7 (0.5 to 0.8)	117.4 (61.9 to 189.3)	9.1 (-21.8 to 44.9)	161.2 (58.5 to 310.3)	21.5 (-28.5 to 94.8)
		Female	443 (312 to 620)	38 (26.9 to 52.1)	6 (3 to 9)	0.6 (0.3 to 0.9)	1005 (753 to 1242)	43.6 (32.6 to 53.7)	16 (11 to 20)	0.7 (0.5 to 0.9)	127 (44.9 to 221.1)	14.6 (-26.6 to 63.8)	174.7 (34.2 to 425.2)	25.4 (-37 to 148.6)
		Male	366 (263 to 558)	31 (22.2 to 48.7)	6 (4 to 11)	0.6 (0.4 to 1)	753 (592 to 983)	32 (25.4 to 40.6)	14 (10 to 20)	0.7 (0.5 to 0.9)	105.9 (34.2 to 184.1)	3 (-33.7 to 44.2)	147.6 (29.6 to 311.5)	18.6 (-38.9 to 96)
	Motor neuron disease	Both	61 (48 to 79)	2.3 (1.8 to 3)	1 (1 to 2)	0.1 (0 to 0.1)	100 (84 to 121)	2.3 (1.9 to 2.8)	2 (2 to 3)	0.1 (0 to 0.1)	65.6 (18.9 to 119.7)	2.7 (-27.8 to 39.5)	95.7 (20.9 to 200.1)	3.7 (-36.3 to 59.6)
		Female	31 (24 to 40)	2.1 (1.6 to 2.8)	0 (0 to 1)	0 (0 to 0.1)	43 (35 to 52)	2.1 (1.7 to 2.5)	1 (1 to 1)	0 (0 to 0)	39.3 (-0.7 to 84.4)	-3.3 (-33.6 to 32)	68.2 (2.1 to 162)	-4.1 (-44.8 to 52.7)
		Male	30 (22 to 43)	2.3 (1.7 to 3.4)	1 (0 to 1)	0.1 (0 to 0.1)	57 (45 to 71)	2.6 (2.1 to 3.2)	1 (1 to 2)	0.1 (0 to 0.1)	93.2 (22.8 to 180.4)	10.8 (-31.1 to 65.4)	117 (18.5 to 279.8)	13.8 (-36.7 to 96.6)
	Headache disorders	Both	21428 (4609 to 47097)	705.7 (171.1 to 1507.6)	--	--	31419 (7890 to 66807)	706.8 (172.2 to 1510.8)	--	--	46.6 (33.1 to 77)	0.2 (-4.7 to 6.1)	--	--

Province	Cause	Sex	1990				2019				1990-2019 percent change (%)			
			DALYs		Death		DALYs		Death		DALYs		Death	
			Number	Rate	Number	Rate	Number	Rate	Number	Rate	Number	Rate	Number	Rate
		Female	12705 (2543 to 28483)	859.1 (193.3 to 1879)	--	--	19146 (4360 to 41438)	874.4 (194.7 to 1908.6)	--	--	50.7 (37.3 to 78.1)	1.8 (-4.8 to 8.3)	--	--
		Male	8723 (2040 to 18929)	556.8 (150.5 to 1173.3)	--	--	12273 (3530 to 25369)	546.2 (151.1 to 1137.9)	--	--	40.7 (25.2 to 78.6)	-1.9 (-8.5 to 7.5)	--	--
	Other neurological disorders	Both	1837 (866 to 3312)	47 (24.7 to 79.8)	9 (7 to 11)	0.3 (0.2 to 0.4)	1949 (1153 to 3142)	50.2 (29.1 to 82.9)	18 (15 to 21)	0.5 (0.4 to 0.5)	6.1 (-46.6 to 141.4)	6.9 (-45.2 to 123.6)	101.4 (43.4 to 193.5)	46.6 (5.2 to 114.6)
		Female	770 (332 to 1443)	40.5 (19.9 to 71.6)	3 (2 to 4)	0.2 (0.2 to 0.3)	851 (486 to 1374)	44.7 (24.6 to 73.6)	7 (5 to 8)	0.3 (0.3 to 0.4)	10.4 (-46.8 to 177.4)	10.4 (-44.6 to 145.4)	131.8 (63.1 to 235.7)	53.6 (2.9 to 139.5)
		Male	1067 (521 to 1892)	53.2 (28.3 to 88.8)	6 (4 to 8)	0.4 (0.3 to 0.5)	1099 (658 to 1771)	55.5 (32.9 to 90.5)	11 (9 to 14)	0.6 (0.5 to 0.7)	3 (-47.7 to 119.4)	4.4 (-46.6 to 112.2)	87.3 (22.4 to 198)	44.7 (-3.1 to 124.1)
	Mental disorders	Both	63402 (46196 to 83895)	2052.9 (1508.9 to 2699.1)	0 (0 to 0)	0 (0 to 0)	91713 (67020 to 120689)	2095.2 (1539.8 to 2762.6)	0 (0 to 0)	0 (0 to 0)	44.7 (36.4 to 53.3)	2.1 (-0.8 to 4.9)	1300.2 (290.3 to 3574.1)	841.7 (164 to 2365.6)
		Female	34477 (25061 to 45846)	2304.5 (1706.3 to 3041.4)	0 (0 to 0)	0 (0 to 0)	50667 (36763 to 66975)	2351.9 (1714.1 to 3107.1)	0 (0 to 0)	0 (0 to 0)	47 (37.5 to 56.3)	2.1 (-2 to 5.9)	1383.6 (249.6 to 4250.4)	920.5 (135.3 to 2983.7)
		Male	28925 (21024 to 38107)	1809.7 (1334 to 2380.3)	0 (0 to 0)	0 (0 to 0)	41046 (29972 to 54108)	1848.7 (1354 to 2444.1)	0 (0 to 0)	0 (0 to 0)	41.9 (33 to 51.6)	2.2 (-1.6 to 6)	546.7 (75.1 to 1929.1)	328.8 (15.4 to 1278.6)
	Schizophrenia	Both	4129 (2984 to 5302)	160.5 (116.3 to 205.1)	--	--	7789 (5607 to 9875)	162.1 (116.9 to 205.5)	--	--	88.7 (71.7 to 107.9)	1 (-7.2 to 9.8)	--	--
		Female	1927 (1376 to 2522)	152.4 (109.8 to 196.6)	--	--	3617 (2614 to 4634)	153.2 (111.3 to 196.7)	--	--	87.7 (65 to 115.3)	0.5 (-10.5 to 14.1)	--	--
		Male	2202 (1599 to 2822)	168.4 (121.8 to 215.3)	--	--	4172 (2965 to 5308)	170.6 (122.2 to 216.2)	--	--	89.5 (66.3 to 115.7)	1.3 (-9.9 to 13.6)	--	--
	Depressive disorders	Both	18286 (11775 to 26941)	658 (437.8 to 956.9)	--	--	31492 (20470 to 46594)	696.8 (460.7 to 1016.3)	--	--	72.2 (53.6 to 90.5)	5.9 (1.1 to 10.9)	--	--
		Female	11334 (7334 to 16872)	824.1 (549.1 to 1207.8)	--	--	18975 (12421 to 28082)	855.9 (564.3 to 1262.5)	--	--	67.4 (48.5 to 88.2)	3.9 (-2.7 to 10.8)	--	--
		Male	6952 (4443 to 10334)	497.9 (328.5 to 732.7)	--	--	12517 (8182 to 18694)	543.7 (354.4 to 796.4)	--	--	80.1 (60.1 to 101.6)	9.2 (1.7 to 17.2)	--	--
	Bipolar disorder	Both	5031 (3065 to 7756)	173 (107 to 268)	--	--	7802 (4886 to 11918)	175.4 (108.4 to 266.2)	--	--	55.1 (39.1 to 70.5)	1.4 (-5.7 to 8.6)	--	--
		Female	2699 (1615 to 4151)	186.2 (114.6 to 284.4)	--	--	4094 (2543 to 6322)	188.9 (115.7 to 288.7)	--	--	51.7 (32.8 to 71.5)	1.4 (-8 to 12.1)	--	--
		Male	2332 (1421 to 3617)	160.1 (99 to 251.2)	--	--	3707 (2296 to 5680)	162.7 (100.9 to 248)	--	--	59 (40.4 to 80.7)	1.6 (-7.8 to 12.5)	--	--
	Anxiety disorders	Both	21915 (15313 to 30687)	675.1 (470.7 to 933.2)	--	--	29671 (20640 to 40806)	696.7 (490.4 to 953.9)	--	--	35.4 (22.5 to 48.4)	3.2 (-2.4 to 9.6)	--	--
		Female	13048 (9119 to 18107)	829.4 (575.3 to 1142.4)	--	--	17977 (12350 to 24790)	857.8 (598.4 to 1175.6)	--	--	37.8 (23.4 to 52.2)	3.4 (-3.9 to 11.8)	--	--
		Male	8867 (6137 to 12342)	525.9 (369.9 to 732.1)	--	--	11694 (8126 to 16235)	542.4 (377.2 to 750.2)	--	--	31.9 (17.1 to 48.9)	3.1 (-5.4 to 12.3)	--	--
	Eating disorders	Both	1566 (969 to 2361)	45.4 (28.3 to 68.2)	0 (0 to 0)	0 (0 to 0)	2307 (1411 to 3461)	53.3 (33 to 79.9)	0 (0 to 0)	0 (0 to 0)	47.3 (26.7 to 74.9)	17.4 (5.8 to 29.8)	1300.2 (290.3 to 3574.1)	841.7 (164 to 2365.6)

Province	Cause	Sex	1990				2019				1990-2019 percent change (%)			
			DALYs		Death		DALYs		Death		DALYs		Death	
			Number	Rate	Number	Rate	Number	Rate	Number	Rate	Number	Rate	Number	Rate
		Female	969 (599 to 1474)	56.5 (35.3 to 85)	0 (0 to 0)	0 (0 to 0)	1395 (848 to 2099)	67.1 (40.9 to 100.1)	0 (0 to 0)	0 (0 to 0)	44 (21.9 to 73)	18.7 (3.4 to 36.1)	1383.6 (249.6 to 4250.4)	920.5 (135.3 to 2983.7)
		Male	597 (364 to 912)	34.4 (20.8 to 53.9)	0 (0 to 0)	0 (0 to 0)	912 (540 to 1413)	40.4 (24.3 to 62.2)	0 (0 to 0)	0 (0 to 0)	52.8 (24.3 to 89.9)	17.4 (-0.9 to 39)	546.7 (75.1 to 1929.1)	328.8 (15.4 to 1278.6)
	Autism spectrum disorders	Both	1657 (1093 to 2407)	44.5 (29.5 to 64.8)	--	--	1820 (1183 to 2651)	45.1 (29.2 to 65.8)	--	--	9.9 (-1.6 to 21.5)	1.3 (-9.2 to 11.5)	--	--
		Female	409 (263 to 603)	22.3 (14.7 to 32.9)	--	--	445 (292 to 655)	22.5 (14.8 to 32.9)	--	--	8.8 (-8 to 28.9)	0.9 (-14 to 18.2)	--	--
		Male	1248 (820 to 1815)	65.9 (43.3 to 96)	--	--	1376 (890 to 2011)	66.6 (43.1 to 97.2)	--	--	10.2 (-4 to 24.1)	1.1 (-11.8 to 13.1)	--	--
	Attention-deficit/hyperactivity disorder	Both	1364 (757 to 2334)	32.5 (18.2 to 55.3)	--	--	1311 (738 to 2224)	33.8 (18.9 to 57.4)	--	--	-3.8 (-17.3 to 13.6)	3.9 (-9.3 to 18.2)	--	--
		Female	412 (225 to 710)	20.1 (11.1 to 34.2)	--	--	396 (218 to 658)	20.9 (11.4 to 34.7)	--	--	-4.1 (-20.4 to 18.4)	3.9 (-11.5 to 24.1)	--	--
		Male	951 (527 to 1629)	44.6 (25.1 to 75.3)	--	--	916 (508 to 1559)	45.9 (25.8 to 78.4)	--	--	-3.7 (-20.5 to 17.6)	2.9 (-13.7 to 21.8)	--	--
	Conduct disorder	Both	3807 (2139 to 5978)	75.7 (42.6 to 119.5)	--	--	2546 (1438 to 4061)	74.6 (42.7 to 118.3)	--	--	-33.1 (-39.1 to -26.7)	-1.4 (-10 to 8.2)	--	--
		Female	1305 (701 to 2132)	52.7 (28.2 to 84.9)	--	--	848 (441 to 1406)	51.1 (26.7 to 84)	--	--	-35 (-44.1 to -24.6)	-3.1 (-16.7 to 13)	--	--
		Male	2502 (1442 to 3893)	97.9 (56.4 to 151.5)	--	--	1698 (969 to 2669)	96.7 (55.3 to 150.9)	--	--	-32.1 (-39.9 to -23.8)	-1.2 (-12.3 to 10.9)	--	--
	Idiopathic developmental intellectual disability	Both	3261 (1784 to 5226)	87.2 (48.1 to 138.6)	--	--	2302 (1155 to 3877)	56.6 (28 to 95.8)	--	--	-29.4 (-41 to -22.1)	-35.1 (-46.5 to -28.2)	--	--
		Female	1426 (779 to 2300)	77.9 (42.8 to 124.8)	--	--	1030 (506 to 1738)	51.8 (25.3 to 87.5)	--	--	-27.8 (-39.5 to -19.9)	-33.6 (-44.2 to -26)	--	--
		Male	1835 (995 to 2940)	96.2 (52.8 to 152.9)	--	--	1272 (639 to 2159)	61.1 (30.7 to 104.1)	--	--	-30.6 (-42.9 to -21.8)	-36.4 (-48.5 to -28.2)	--	--
	Other mental disorders	Both	2388 (1510 to 3614)	101 (64.4 to 151.2)	--	--	4672 (3004 to 7154)	100.9 (65 to 154.8)	--	--	95.6 (80.4 to 111.2)	-0.1 (-5.4 to 5.6)	--	--
		Female	949 (590 to 1448)	82.9 (52 to 126.8)	--	--	1890 (1201 to 2887)	82.8 (52.8 to 125.8)	--	--	99.2 (78.7 to 121.8)	-0.1 (-8.2 to 9.2)	--	--
		Male	1439 (907 to 2166)	118.4 (75.9 to 176.6)	--	--	2782 (1777 to 4192)	118.5 (76.1 to 178)	--	--	93.3 (76.4 to 111.9)	0.1 (-6.6 to 7.6)	--	--
	Substance use disorders	Both	9345 (7206 to 11613)	324.9 (254.5 to 399.5)	75 (58 to 98)	3.2 (2.5 to 4.1)	17432 (14036 to 20924)	383.4 (308.7 to 461.2)	157 (129 to 189)	3.5 (2.9 to 4.2)	86.5 (60.6 to 120.3)	18 (1.5 to 37.4)	107.8 (49.3 to 190.5)	9.4 (-19.8 to 51.4)
		Female	2823 (2106 to 3669)	195.9 (148.4 to 249.8)	20 (14 to 29)	1.8 (1.3 to 2.5)	4053 (3085 to 5112)	185.2 (139.7 to 234.9)	26 (20 to 33)	1.3 (1 to 1.6)	43.6 (17 to 76.2)	-5.5 (-22.6 to 13)	26.8 (-17.9 to 99.8)	-30.6 (-53.7 to 4.8)
		Male	6521 (4993 to 8240)	451.5 (349.4 to 562.5)	55 (40 to 74)	4.6 (3.3 to 6)	13379 (10756 to 16070)	572.2 (459.9 to 686.5)	131 (103 to 163)	5.7 (4.5 to 7)	105.2 (71 to 151.3)	26.7 (6 to 53.7)	137.7 (64.4 to 258.2)	24.8 (-14.7 to 86.4)
	Alcohol use disorders	Both	1443 (1065 to 1928)	53.3 (39.1 to 70.1)	8 (6 to 11)	0.4 (0.3 to 0.5)	1904 (1396 to 2537)	41 (30.1 to 53.8)	13 (10 to 16)	0.3 (0.2 to 0.4)	31.9 (14.9 to 52.3)	-23 (-32.9 to -11.6)	52.5 (4.5 to 136)	-24.1 (-47 to 15.5)
		Female	400 (285 to 557)	28.5 (20.5 to 39.2)	2 (1 to 3)	0.2 (0.1 to 0.2)	556 (389 to 771)	25 (17.7 to 34.2)	2 (2 to 3)	0.1 (0.1 to 0.1)	39 (15.5 to 66.7)	-12.2 (-25.8 to 3.7)	10.6 (-25.2 to 68.1)	-36.7 (-56 to -6.6)

Province	Cause	Sex	1990				2019				1990-2019 percent change (%)			
			DALYs		Death		DALYs		Death		DALYs		Death	
			Number	Rate	Number	Rate	Number	Rate	Number	Rate	Number	Rate	Number	Rate
		Male	1043 (743 to 1390)	77.4 (55.7 to 102)	6 (4 to 9)	0.6 (0.4 to 0.9)	1348 (995 to 1779)	56.5 (41.4 to 73.9)	10 (8 to 14)	0.5 (0.4 to 0.6)	29.2 (9.4 to 55.9)	-27 (-39.1 to -12)	65.5 (5.7 to 182.1)	-18.5 (-47.3 to 36.3)
	Drug use disorders	Both	7901 (6087 to 9972)	271.6 (209.2 to 339.6)	67 (51 to 88)	2.8 (2.2 to 3.6)	15528 (12377 to 18833)	342.3 (274.4 to 416.2)	144 (118 to 175)	3.2 (2.6 to 3.9)	96.5 (66.6 to 137.2)	26 (6.7 to 48.8)	114.5 (51.2 to 203.1)	13.9 (-17.7 to 58.3)
		Female	2423 (1770 to 3197)	167.4 (124.7 to 216.7)	18 (12 to 26)	1.6 (1.2 to 2.3)	3497 (2617 to 4490)	160.2 (119.7 to 205.5)	24 (18 to 31)	1.2 (0.9 to 1.5)	44.3 (15.6 to 80.6)	-4.3 (-23.2 to 16.9)	28.5 (-18 to 105.4)	-29.9 (-53.7 to 8.5)
		Male	5478 (4146 to 7002)	374.2 (285.3 to 474.2)	49 (35 to 67)	4 (2.9 to 5.3)	12032 (9577 to 14551)	515.8 (412.2 to 625.6)	121 (95 to 150)	5.2 (4.1 to 6.5)	119.6 (79.9 to 172.9)	37.8 (12.8 to 72)	146.9 (68.5 to 280.4)	30.9 (-10.9 to 96.7)
	Diabetes and kidney diseases	Both	24894 (20847 to 29170)	1242.9 (1062.3 to 1446.7)	537 (443 to 640)	42.5 (35.5 to 51.8)	61933 (52253 to 72055)	1609.7 (1368.7 to 1869.2)	1619 (1401 to 1847)	51.7 (44.5 to 58.4)	148.8 (110.8 to 196.4)	29.5 (11.6 to 49.7)	201.3 (142.6 to 275.8)	21.6 (-4.2 to 48.2)
		Female	11913 (9383 to 14470)	1230.5 (999.2 to 1502.7)	248 (194 to 323)	41.5 (32.4 to 57.5)	31051 (25849 to 36843)	1622.1 (1359.1 to 1907.5)	814 (672 to 978)	52.4 (43.5 to 62.5)	160.7 (109.8 to 225.3)	31.8 (6.4 to 61.1)	227.6 (137.6 to 342.7)	26.2 (-11.8 to 64.9)
		Male	12981 (10486 to 15772)	1250.5 (1026 to 1488.7)	289 (224 to 361)	43.3 (33.6 to 53.3)	30882 (25549 to 36316)	1596.7 (1333.5 to 1874.4)	805 (661 to 974)	51 (42 to 61.1)	137.9 (90.9 to 194.3)	27.7 (5.4 to 55.1)	178.7 (110.1 to 280.6)	17.7 (-12 to 57)
	Diabetes mellitus	Both	7976 (6190 to 9974)	440 (343.7 to 552.2)	113 (89 to 153)	8.8 (6.9 to 13.1)	36421 (28603 to 44942)	915.3 (727 to 1126.5)	620 (536 to 718)	18.9 (16.4 to 21.7)	356.6 (292 to 409.7)	108 (76.4 to 133.2)	447.6 (282.5 to 622.9)	113.9 (42.9 to 188)
		Female	3694 (2819 to 4772)	432.2 (336.5 to 563)	54 (38 to 85)	9.1 (6.3 to 15.3)	18683 (14688 to 23374)	942.2 (748.8 to 1169.3)	326 (263 to 395)	20.1 (16.3 to 24.2)	405.7 (293.3 to 499)	118 (65.8 to 158.9)	507.3 (240.7 to 817.8)	119.5 (20.8 to 242.3)
		Male	4282 (3302 to 5417)	446 (341.7 to 560.2)	59 (44 to 79)	8.5 (6.4 to 10.9)	17738 (13757 to 22051)	887.5 (697 to 1095.1)	293 (235 to 365)	17.7 (14.4 to 21.8)	314.3 (254.8 to 379.9)	99 (71.5 to 129.2)	393.7 (249.7 to 613)	108.3 (53.2 to 188.5)
	Chronic kidney disease	Both	16882 (13829 to 20015)	801.9 (680.4 to 935.7)	423 (347 to 509)	33.6 (27.9 to 42.3)	24886 (21839 to 28292)	678.6 (597.8 to 768.3)	980 (842 to 1127)	32.2 (27.7 to 36.6)	47.4 (22.4 to 83.2)	-15.4 (-29.4 to 1.8)	131.4 (83 to 189.8)	-4.2 (-27.1 to 19)
		Female	8205 (6325 to 10235)	797.6 (642.2 to 997)	194 (151 to 252)	32.3 (24.4 to 47.4)	12114 (10190 to 14282)	666 (564.1 to 783.7)	479 (395 to 581)	31.8 (26.4 to 38.5)	47.6 (13.3 to 96)	-16.5 (-35.5 to 6.4)	146.2 (79.6 to 234.3)	-1.8 (-34.6 to 31.7)
		Male	8677 (6640 to 10747)	803.3 (640.9 to 961.2)	229 (177 to 285)	34.8 (26.9 to 43.8)	12771 (10635 to 15320)	691.5 (578.4 to 824.3)	501 (405 to 613)	32.7 (26.7 to 39.6)	47.2 (13.8 to 95.8)	-13.9 (-32.5 to 12.2)	118.8 (62.2 to 198.7)	-6.1 (-30.5 to 28)
	Acute glomerulonephritis	Both	36 (10 to 385)	1 (0.3 to 8.9)	1 (0 to 6)	0 (0 to 0.2)	627 (89 to 852)	15.8 (2.6 to 21.2)	19 (3 to 26)	0.6 (0.1 to 0.7)	1664.7 (-78.9 to 7225.7)	1557.3 (-73 to 5356.9)	3191.3 (-55.2 to 11426.6)	2046.2 (-58.2 to 6078.3)
		Female	13 (5 to 105)	0.8 (0.3 to 5.2)	0 (0 to 2)	0 (0 to 0.1)	254 (40 to 360)	13.9 (2.4 to 19.4)	9 (1 to 13)	0.6 (0.1 to 0.8)	1828.3 (-67.6 to 6700.1)	1743 (-60.9 to 5112.7)	3829.7 (-33.4 to 12350.7)	2487.5 (-43.4 to 7021.8)
		Male	22 (3 to 263)	1.1 (0.2 to 12.4)	0 (0 to 4)	0 (0 to 0.3)	373 (31 to 580)	17.7 (1.8 to 26.9)	11 (1 to 16)	0.6 (0.1 to 0.9)	1568.3 (-87.4 to 14739.1)	1443.5 (-83.7 to 9588.1)	2801.9 (-74.2 to 20643.1)	1743.3 (-77 to 9744.9)
	Skin and subcutaneous diseases	Both	14925 (9961 to 21757)	417 (281.5 to 609.1)	4 (3 to 5)	0.4 (0.3 to 0.6)	16806 (11228 to 25134)	425.3 (284.7 to 636.1)	14 (9 to 17)	0.5 (0.3 to 0.6)	12.6 (8.1 to 16.6)	2 (-1.1 to 4.9)	238.9 (86.2 to 419.1)	22.5 (-36.3 to 103.8)
		Female	7779 (5208 to 11193)	442 (298.6 to 647.9)	2 (1 to 3)	0.4 (0.3 to 0.6)	8705 (5836 to 13001)	448.9 (300.6 to 666.7)	7 (3 to 9)	0.5 (0.2 to 0.7)	11.9 (6.6 to 16.9)	1.6 (-2.2 to 5)	236.9 (34.8 to 494.5)	13.1 (-59.5 to 119.9)
		Male	7146 (4717 to 10457)	392.9 (264 to 571.4)	2 (1 to 3)	0.4 (0.2 to 0.6)	8101 (5362 to 12096)	402.7 (268.3 to 602.8)	7 (4 to 9)	0.5 (0.3 to 0.7)	13.4 (8.2 to 18.2)	2.5 (-1.4 to 6.5)	240.8 (90.8 to 458.3)	37.1 (-29.7 to 136.6)

Province	Cause	Sex	1990				2019				1990-2019 percent change (%)			
			DALYs		Death		DALYs		Death		DALYs		Death	
			Number	Rate	Number	Rate	Number	Rate	Number	Rate	Number	Rate	Number	Rate
	Dermatitis	Both	3397 (2019 to 5372)	93.5 (56.7 to 144.2)	--	--	3833 (2324 to 5853)	94.1 (57.1 to 145.5)	--	--	12.8 (3.7 to 23.5)	0.6 (-4.1 to 5.7)	--	--
		Female	1851 (1096 to 2909)	103.8 (62.6 to 159.2)	--	--	2087 (1267 to 3200)	104.6 (62.9 to 162.3)	--	--	12.7 (1.9 to 24.9)	0.8 (-5.8 to 7.8)	--	--
		Male	1546 (910 to 2416)	83.6 (50.7 to 127.8)	--	--	1746 (1067 to 2666)	84.1 (50.7 to 127.8)	--	--	12.9 (1.3 to 25.7)	0.5 (-6.3 to 8.1)	--	--
	Psoriasis	Both	1392 (967 to 1850)	48.7 (34.4 to 64)	--	--	1561 (1107 to 2070)	35.4 (24.9 to 46.9)	--	--	12.1 (-0.1 to 25.2)	-27.4 (-34.2 to -19.9)	--	--
		Female	716 (487 to 961)	50.5 (35.3 to 66.8)	--	--	793 (546 to 1079)	36.7 (25.4 to 49.7)	--	--	10.7 (-4.9 to 28.6)	-27.3 (-36.7 to -17)	--	--
		Male	675 (468 to 902)	47.1 (32.8 to 62)	--	--	768 (538 to 1026)	34.2 (24.1 to 45.4)	--	--	13.7 (-3.9 to 33.8)	-27.4 (-37.1 to -15.9)	--	--
	Bacterial skin diseases	Both	264 (184 to 394)	9.9 (7.1 to 13.5)	4 (2 to 5)	0.3 (0.2 to 0.4)	316 (225 to 458)	8.1 (5.8 to 11.7)	6 (5 to 9)	0.2 (0.2 to 0.3)	19.8 (3.5 to 45.5)	-17.9 (-34.7 to 6.5)	81.9 (23.9 to 207.9)	-35.3 (-59.8 to 38.1)
		Female	118 (80 to 181)	9.4 (6.3 to 13.6)	2 (1 to 2)	0.3 (0.2 to 0.5)	134 (89 to 201)	7.1 (4.7 to 10.8)	3 (2 to 5)	0.2 (0.1 to 0.4)	12.9 (-9.6 to 54.3)	-25.1 (-48.7 to 20.5)	64.4 (-15.4 to 322.1)	-45.3 (-75.3 to 93.2)
		Male	146 (98 to 216)	10.3 (7.3 to 14.5)	2 (1 to 3)	0.3 (0.2 to 0.4)	183 (126 to 267)	9.2 (6.4 to 13.3)	4 (3 to 5)	0.2 (0.2 to 0.3)	25.4 (2.6 to 56.4)	-10.9 (-30 to 13.9)	97.3 (34.3 to 204.2)	-22.9 (-51.1 to 31.1)
	Scabies	Both	940 (507 to 1532)	25.2 (13.7 to 40.8)	--	--	993 (550 to 1614)	25.1 (13.8 to 40.8)	--	--	5.6 (-2.1 to 14.8)	-0.3 (-4.8 to 4.3)	--	--
		Female	465 (249 to 756)	25.3 (14 to 41)	--	--	485 (271 to 786)	25.2 (14 to 40.4)	--	--	4.3 (-5.1 to 14.9)	-0.6 (-6.6 to 6)	--	--
		Male	475 (255 to 772)	25.1 (13.6 to 40.4)	--	--	508 (279 to 829)	25 (13.9 to 40.9)	--	--	6.9 (-2.4 to 17.3)	-0.1 (-6.3 to 6.9)	--	--
	Fungal skin diseases	Both	477 (193 to 1025)	19 (7.7 to 40.4)	--	--	737 (299 to 1592)	18.5 (7.5 to 39.8)	--	--	54.4 (44 to 64.5)	-2.6 (-4.4 to -0.7)	--	--
		Female	214 (86 to 456)	17.7 (7.2 to 38.3)	--	--	342 (138 to 744)	17.3 (7 to 37.6)	--	--	59.5 (48.8 to 71.5)	-2.4 (-4.9 to 0.2)	--	--
		Male	263 (104 to 572)	20.2 (8.1 to 43.3)	--	--	395 (159 to 855)	19.7 (8 to 42.4)	--	--	50.3 (39.5 to 60.7)	-2.6 (-5.2 to -0.2)	--	--
	Viral skin diseases	Both	2306 (1471 to 3437)	51.3 (32.7 to 76)	--	--	1919 (1223 to 2875)	51.4 (32.6 to 77.5)	--	--	-16.8 (-20.4 to -12.6)	0.2 (-3.6 to 4.9)	--	--
		Female	1122 (713 to 1691)	50.9 (32.5 to 76.6)	--	--	929 (597 to 1399)	51 (32.7 to 77)	--	--	-17.2 (-22.1 to -11.6)	0.2 (-5.3 to 6.6)	--	--
		Male	1184 (753 to 1767)	51.7 (33 to 76.7)	--	--	990 (625 to 1467)	51.8 (32.7 to 77.2)	--	--	-16.4 (-21.2 to -10.6)	0.3 (-5.2 to 6.7)	--	--
	Acne vulgaris	Both	2213 (1322 to 3520)	51 (30.6 to 81.1)	--	--	2458 (1470 to 3886)	69.7 (41.8 to 111.1)	--	--	11.1 (2.8 to 21)	36.7 (26.7 to 47.8)	--	--
		Female	1053 (633 to 1675)	49.6 (29.8 to 78.6)	--	--	1165 (708 to 1823)	67.6 (41 to 106)	--	--	10.6 (-0.2 to 23.5)	36.3 (23.5 to 51.3)	--	--
		Male	1160 (685 to 1870)	52.3 (30.9 to 83.9)	--	--	1293 (751 to 2083)	71.6 (41.7 to 115.9)	--	--	11.5 (0.4 to 24.9)	36.8 (23.5 to 52.9)	--	--
	Alopecia areata	Both	192 (122 to 290)	6.3 (4 to 9.5)	--	--	285 (180 to 421)	6.4 (4 to 9.4)	--	--	48.2 (32.9 to 65.4)	0.2 (-9.6 to 11.1)	--	--
		Female	124 (77 to 185)	8.3 (5.2 to 12.2)	--	--	183 (116 to 271)	8.3 (5.3 to 12.3)	--	--	48.2 (29.1 to 69.8)	0.4 (-11.6 to 13.9)	--	--

Province	Cause	Sex	1990				2019				1990-2019 percent change (%)			
			DALYs		Death		DALYs		Death		DALYs		Death	
			Number	Rate	Number	Rate	Number	Rate	Number	Rate	Number	Rate	Number	Rate
		Male	69 (42 to 107)	4.5 (2.7 to 6.9)	--	--	102 (64 to 153)	4.5 (2.8 to 6.7)	--	--	48.1 (22.9 to 80.8)	0.1 (-14.6 to 20.2)	--	--
	Pruritus	Both	293 (138 to 526)	10.1 (4.8 to 18.2)	--	--	448 (214 to 795)	10.6 (5 to 18.9)	--	--	53 (41.2 to 66.9)	4.2 (-1 to 9.5)	--	--
		Female	157 (73 to 290)	11.1 (5.3 to 20.1)	--	--	241 (115 to 433)	11.6 (5.6 to 20.6)	--	--	54.1 (38.8 to 70.2)	3.9 (-2.8 to 11)	--	--
		Male	136 (64 to 246)	9.2 (4.4 to 16.5)	--	--	207 (99 to 376)	9.6 (4.6 to 17.5)	--	--	51.7 (36.7 to 68.7)	4.3 (-3 to 11.9)	--	--
	Urticaria	Both	2292 (1504 to 3385)	58.2 (38.3 to 84.1)	--	--	2305 (1516 to 3306)	58.3 (38.4 to 84.2)	--	--	0.5 (-7.9 to 10.5)	0.2 (-4.9 to 6.1)	--	--
		Female	1388 (921 to 2055)	71 (47.2 to 102.9)	--	--	1368 (895 to 1970)	71.2 (46.9 to 102.8)	--	--	-1.5 (-11.1 to 9.4)	0.4 (-6.5 to 7.4)	--	--
		Male	904 (598 to 1337)	45.8 (30.3 to 67)	--	--	937 (611 to 1347)	46 (29.9 to 66.5)	--	--	3.7 (-7.3 to 17.5)	0.3 (-7.8 to 9.2)	--	--
	Decubitus ulcer	Both	14 (10 to 26)	1.3 (0.9 to 2.4)	0 (0 to 1)	0.1 (0 to 0.2)	116 (44 to 153)	3.8 (1.5 to 5)	7 (2 to 9)	0.3 (0.1 to 0.4)	706.6 (86.3 to 1175.8)	202.5 (-33.3 to 395.4)	1409.8 (76.8 to 3140.2)	272.2 (-48 to 678.6)
		Female	7 (4 to 14)	1.3 (0.8 to 2.8)	0 (0 to 1)	0.1 (0 to 0.2)	58 (20 to 84)	3.8 (1.3 to 5.6)	4 (1 to 5)	0.3 (0.1 to 0.4)	743.5 (62.6 to 1571.8)	188 (-50.3 to 489.2)	1239.9 (26.1 to 3685.1)	234.3 (-67.4 to 812.6)
		Male	8 (5 to 14)	1.2 (0.8 to 2.4)	0 (0 to 1)	0.1 (0 to 0.2)	58 (20 to 79)	3.8 (1.4 to 5)	3 (1 to 4)	0.3 (0.1 to 0.3)	673 (89.2 to 1127.4)	224 (-25.4 to 432.7)	1664.6 (95.8 to 3924.2)	349.5 (-41.2 to 906.4)
	Other skin and subcutaneous diseases	Both	1144 (550 to 2097)	42.5 (20.5 to 77.6)	0 (0 to 0)	0 (0 to 0)	1836 (880 to 3345)	43.9 (21.1 to 79.8)	1 (1 to 2)	0 (0 to 0.1)	60.5 (56.2 to 65.7)	3.5 (1.1 to 6)	515.5 (202.2 to 1293.7)	47.6 (-27.3 to 308.1)
		Female	562 (270 to 1050)	43 (20.7 to 79.4)	0 (0 to 0)	0 (0 to 0.1)	921 (444 to 1676)	44.5 (21.5 to 80.9)	1 (0 to 1)	0 (0 to 0.1)	63.8 (57.4 to 70.7)	3.5 (0 to 7.4)	485.3 (83.9 to 2146.8)	36.7 (-55.8 to 482.9)
		Male	581 (277 to 1068)	41.9 (20.4 to 76.8)	0 (0 to 0)	0 (0 to 0)	915 (435 to 1665)	43.4 (20.8 to 78.6)	0 (0 to 1)	0 (0 to 0)	57.4 (51.7 to 64.2)	3.5 (0.3 to 7)	557.6 (252.7 to 1117.6)	76.2 (-12.6 to 257.5)
	Sense organ diseases	Both	19830 (14140 to 26849)	1045.9 (752.2 to 1400.6)	--	--	30938 (21819 to 42327)	813.7 (576.2 to 1108.3)	--	--	56 (48 to 64.3)	-22.2 (-25.1 to -19)	--	--
		Female	9447 (6702 to 12823)	1049.6 (759.1 to 1398.3)	--	--	15463 (10969 to 21083)	814.5 (579.2 to 1100.6)	--	--	63.7 (54.3 to 73.4)	-22.4 (-25.6 to -18.9)	--	--
		Male	10383 (7327 to 14196)	1042 (748.9 to 1396.6)	--	--	15475 (10842 to 21223)	812.9 (574.2 to 1105.8)	--	--	49 (40.7 to 57.5)	-22 (-25.4 to -18.6)	--	--
	Blindness and vision loss	Both	9189 (6551 to 12423)	509 (365.9 to 683.7)	--	--	13832 (9785 to 18664)	373.1 (265.7 to 500.4)	--	--	50.5 (44.3 to 57)	-26.7 (-29.6 to -23.7)	--	--
		Female	4599 (3286 to 6191)	537.9 (387.4 to 719.3)	--	--	7331 (5208 to 9947)	393.8 (281.1 to 532)	--	--	59.4 (51.5 to 67.5)	-26.8 (-30.1 to -23.1)	--	--
		Male	4591 (3249 to 6235)	483.2 (347.3 to 651.2)	--	--	6501 (4577 to 8786)	352.1 (249.3 to 472.4)	--	--	41.6 (34.5 to 49)	-27.1 (-30.5 to -23.5)	--	--
	Age-related and other hearing loss	Both	9659 (6653 to 13472)	495.4 (352.9 to 687.3)	--	--	15327 (10560 to 21548)	397.4 (278 to 554.2)	--	--	58.7 (44.1 to 74.7)	-19.8 (-23.9 to -15.7)	--	--
		Female	4335 (2999 to 6012)	466.7 (332.9 to 643.7)	--	--	7174 (5009 to 10083)	373.9 (262.3 to 519.6)	--	--	65.5 (47.4 to 84.3)	-19.9 (-24.5 to -15)	--	--

Province	Cause	Sex	1990				2019				1990-2019 percent change (%)			
			DALYs		Death		DALYs		Death		DALYs		Death	
			Number	Rate	Number	Rate	Number	Rate	Number	Rate	Number	Rate	Number	Rate
		Male	5323 (3661 to 7489)	520.7 (369 to 724.1)	--	--	8153 (5609 to 11537)	421.1 (293.4 to 590.3)	--	--	53.1 (38.4 to 67.6)	-19.1 (-24 to -14.7)	--	--
	Other sense organ diseases	Both	982 (595 to 1508)	41.4 (24.6 to 64.6)	--	--	1779 (1036 to 2748)	43.2 (25.6 to 66.8)	--	--	81.2 (64.7 to 96.9)	4.4 (-0.5 to 9.4)	--	--
		Female	513 (309 to 785)	45.1 (26.7 to 69.8)	--	--	958 (562 to 1479)	46.8 (27.6 to 72.3)	--	--	86.8 (67 to 106.6)	3.9 (-2.6 to 11.2)	--	--
		Male	469 (285 to 719)	38.1 (22.6 to 59.2)	--	--	822 (481 to 1265)	39.7 (23.7 to 61.2)	--	--	75.1 (56.8 to 94.3)	4.3 (-2.8 to 11.8)	--	--
	Musculoskeletal disorders	Both	40385 (28626 to 53885)	1765.8 (1259 to 2340.8)	7 (5 to 13)	0.4 (0.3 to 0.7)	80924 (58020 to 107523)	1808.1 (1295.1 to 2388.9)	30 (22 to 36)	0.9 (0.6 to 1.1)	100.4 (89.8 to 111.1)	2.4 (-1.8 to 6.3)	317.9 (114.3 to 554.6)	104.4 (4.2 to 218.6)
		Female	20966 (14904 to 27903)	1893.3 (1349.9 to 2505)	5 (3 to 10)	0.6 (0.3 to 1.2)	44588 (31900 to 58798)	2012.5 (1448 to 2650.6)	21 (13 to 26)	1.2 (0.7 to 1.5)	112.7 (101.8 to 124.7)	6.3 (1.9 to 10.8)	335.6 (81.7 to 690)	112.4 (-19 to 305.1)
		Male	19419 (13550 to 26385)	1646.1 (1169.4 to 2210.2)	2 (2 to 5)	0.3 (0.2 to 0.5)	36336 (25683 to 48676)	1607 (1140.6 to 2141.7)	10 (7 to 12)	0.6 (0.4 to 0.7)	87.1 (73.9 to 100.2)	-2.4 (-8 to 2.9)	284.5 (73.9 to 488)	91.6 (-12.2 to 183.1)
	Rheumatoid arthritis	Both	263 (181 to 360)	11.4 (7.9 to 15.7)	1 (0 to 2)	0.1 (0 to 0.2)	584 (410 to 798)	12.9 (9.1 to 17.5)	3 (2 to 3)	0.1 (0 to 0.1)	122 (83.1 to 158.9)	12.9 (-8.9 to 31.7)	246.7 (9.7 to 646.1)	27.1 (-61.2 to 182.5)
		Female	183 (123 to 251)	16.3 (11 to 22.4)	1 (0 to 2)	0.1 (0 to 0.3)	406 (279 to 564)	18 (12.5 to 24.6)	2 (1 to 2)	0.1 (0 to 0.1)	122.3 (71.6 to 173.7)	10.7 (-18.3 to 36.8)	199 (-32.2 to 802.4)	4.1 (-77.2 to 225.9)
		Male	80 (55 to 112)	6.9 (4.7 to 9.5)	0 (0 to 0)	0 (0 to 0)	178 (123 to 241)	7.9 (5.5 to 10.6)	1 (1 to 1)	0.1 (0 to 0.1)	121.4 (85.9 to 164.8)	15.1 (-3 to 36.5)	395.5 (211.1 to 847.4)	103.6 (27.5 to 290.1)
	Osteoarthritis	Both	3108 (1580 to 6269)	175.8 (89.5 to 349.6)	--	--	7740 (3894 to 15325)	190 (95.7 to 375.5)	--	--	149 (139.7 to 158.3)	8 (4.6 to 11.7)	--	--
		Female	1658 (844 to 3261)	200.3 (101.9 to 397.2)	--	--	4420 (2243 to 8629)	216.1 (109.8 to 425.4)	--	--	166.6 (153.5 to 180.1)	7.9 (3.3 to 12.9)	--	--
		Male	1450 (732 to 2903)	153.9 (77.4 to 308)	--	--	3319 (1639 to 6569)	163.5 (81.5 to 325.4)	--	--	128.9 (117.8 to 141.1)	6.2 (1.1 to 11.5)	--	--
	Low back pain	Both	21733 (15316 to 29991)	905.7 (637.2 to 1249.8)	--	--	34120 (23727 to 46660)	773.1 (543.3 to 1045.1)	--	--	57 (44.8 to 70)	-14.6 (-19.8 to -9.3)	--	--
		Female	9567 (6739 to 13020)	817 (576.5 to 1106.2)	--	--	16357 (11430 to 22020)	755.5 (532.3 to 1009.8)	--	--	71 (56.8 to 87.6)	-7.5 (-14.2 to -0.5)	--	--
		Male	12167 (8496 to 16902)	988.9 (695.2 to 1382.7)	--	--	17762 (12335 to 24812)	790.4 (551.1 to 1089.9)	--	--	46 (30.9 to 61)	-20.1 (-26.6 to -13.8)	--	--
	Neck pain	Both	9200 (5974 to 13304)	403.8 (265.1 to 587.2)	--	--	18792 (12393 to 27500)	405.7 (266.5 to 583.2)	--	--	104.3 (91.8 to 115)	0.5 (-1.6 to 2.5)	--	--
		Female	5461 (3569 to 8077)	494.2 (325.5 to 721.4)	--	--	11361 (7514 to 16491)	494.3 (327.2 to 718.1)	--	--	108 (94.4 to 120.4)	0 (-2.5 to 2.6)	--	--
		Male	3739 (2420 to 5502)	318.6 (207.8 to 464.5)	--	--	7431 (4800 to 11008)	318.8 (207.7 to 462.3)	--	--	98.8 (85 to 112.2)	0.1 (-3 to 3.5)	--	--
	Gout	Both	265 (164 to 386)	14.3 (9 to 20.6)	--	--	646 (404 to 953)	15.3 (9.6 to 22.5)	--	--	143.6 (109.7 to 188.9)	6.8 (-6.3 to 22.9)	--	--

Province	Cause	Sex	1990				2019				1990-2019 percent change (%)			
			DALYs		Death		DALYs		Death		DALYs		Death	
			Number	Rate	Number	Rate	Number	Rate	Number	Rate	Number	Rate	Number	Rate
		Female	53 (32 to 80)	6.1 (3.8 to 9.1)	--	--	145 (89 to 219)	6.9 (4.3 to 10.4)	--	--	172.3 (113.2 to 250)	13.3 (-9.7 to 42.5)	--	--
		Male	212 (129 to 313)	21.9 (13.6 to 31.7)	--	--	500 (311 to 724)	23.7 (14.7 to 34.4)	--	--	136.3 (96 to 188.9)	8.3 (-7.4 to 27.9)	--	--
	Other musculoskeletal disorders	Both	5815 (3899 to 8137)	254.7 (170 to 357.4)	6 (5 to 11)	0.4 (0.3 to 0.6)	19043 (12979 to 26563)	411.1 (279.5 to 567)	28 (20 to 33)	0.8 (0.6 to 1)	227.5 (199.2 to 262.6)	61.4 (47.6 to 78.8)	326.4 (120.3 to 565.8)	116.8 (13.1 to 236.4)
		Female	4044 (2769 to 5632)	359.4 (247.7 to 497.4)	4 (3 to 8)	0.5 (0.3 to 0.9)	11898 (8185 to 16313)	521.6 (361.4 to 709.6)	19 (12 to 24)	1.1 (0.6 to 1.4)	194.2 (164.3 to 227.1)	45.1 (31.3 to 61.4)	354.6 (104.9 to 716.9)	134.6 (1.2 to 344.1)
		Male	1770 (1050 to 2653)	155.9 (94 to 235.2)	2 (2 to 4)	0.3 (0.2 to 0.5)	7145 (4642 to 10321)	302.6 (196.9 to 430.2)	9 (7 to 11)	0.5 (0.4 to 0.7)	303.6 (237.7 to 393.3)	94.2 (62.7 to 137.3)	275.6 (64.7 to 476.5)	90.5 (-17.1 to 183.1)
	Other non-communicable diseases	Both	211317 (136458 to 285422)	4981.1 (3346.7 to 6554)	1992 (1167 to 2853)	42.3 (25.1 to 59.9)	82051 (63273 to 105110)	2076.7 (1629.9 to 2624.3)	364 (297 to 455)	11.6 (9.5 to 14.6)	-61.2 (-74.3 to -35.9)	-58.3 (-70.9 to -35.3)	-81.7 (-88.5 to -64.4)	-72.6 (-82.7 to -47.1)
		Female	107062 (68500 to 147603)	5418 (3649.3 to 7189.4)	917 (499 to 1370)	40 (22.1 to 59.6)	54133 (40769 to 70524)	2644.3 (2032.4 to 3418.6)	174 (139 to 220)	11.3 (9 to 14.3)	-49.4 (-66.8 to -18.6)	-51.2 (-65.6 to -27.3)	-81 (-88.6 to -60.2)	-71.7 (-83 to -41.9)
		Male	104254 (55980 to 145821)	4546.1 (2589.3 to 6234.1)	1075 (535 to 1550)	44.6 (22.6 to 64)	27917 (21708 to 35663)	1531 (1203.8 to 1944.2)	189 (151 to 242)	11.8 (9.4 to 15.2)	-73.2 (-83 to -43.4)	-66.3 (-77.9 to -32.8)	-82.4 (-89.1 to -58.1)	-73.4 (-83.6 to -38)
	Congenital birth defects	Both	167499 (97028 to 242752)	3485.1 (2024.7 to 5034.7)	1870 (1059 to 2731)	38.9 (22.1 to 56.6)	25481 (20084 to 32869)	806.2 (627.2 to 1052.9)	260 (200 to 341)	8.3 (6.3 to 11.1)	-84.8 (-90.8 to -68.3)	-76.9 (-86.1 to -51.6)	-86.1 (-91.8 to -70.2)	-78.6 (-87.4 to -53.8)
		Female	76742 (39583 to 114804)	3260.7 (1684.7 to 4869.3)	860 (434 to 1299)	36.5 (18.6 to 55.1)	11727 (9083 to 15157)	760.6 (580.9 to 993.5)	121 (91 to 161)	8 (5.9 to 10.7)	-84.7 (-91.1 to -64.3)	-76.7 (-86.5 to -45.1)	-85.9 (-92.1 to -66.1)	-78.2 (-87.8 to -47.2)
		Male	90757 (40324 to 132919)	3698 (1657.5 to 5413.5)	1010 (432 to 1486)	41.1 (17.8 to 60.6)	13754 (10656 to 18221)	849.5 (649.6 to 1140.8)	139 (106 to 190)	8.7 (6.6 to 12)	-84.8 (-91 to -60.5)	-77 (-86.3 to -39.7)	-86.3 (-92 to -62.3)	-78.9 (-87.8 to -42)
	Urinary diseases and male infertility	Both	690 (494 to 968)	27.7 (18.8 to 40.6)	3 (2 to 9)	0.2 (0.1 to 0.9)	1294 (951 to 1749)	32.3 (24.2 to 42.7)	16 (13 to 20)	0.6 (0.4 to 0.7)	87.7 (56.3 to 113.7)	16.6 (-12.2 to 34.9)	387.4 (109.8 to 664.3)	169.5 (-25.1 to 431.2)
		Female	228 (172 to 313)	15.2 (10.8 to 28.2)	2 (1 to 5)	0.3 (0.1 to 1.1)	379 (300 to 475)	19.5 (15.6 to 24.4)	8 (6 to 12)	0.6 (0.4 to 0.9)	66 (25.8 to 112.2)	28 (-24.4 to 70.2)	348.6 (91.8 to 696.7)	129 (-23 to 417.6)
		Male	461 (303 to 687)	38.9 (25 to 57.8)	1 (1 to 4)	0.2 (0.1 to 0.8)	915 (632 to 1312)	45.2 (31.6 to 64)	8 (6 to 10)	0.5 (0.4 to 0.7)	98.5 (67.6 to 127)	16.2 (-8.3 to 31.8)	436.8 (96.7 to 770)	236.2 (-20.7 to 534.1)
	Gynecological diseases	Female	14263 (9744 to 19963)	1100 (749.1 to 1526.9)	0 (0 to 0)	0 (0 to 0)	25100 (16960 to 34800)	1048.5 (713.6 to 1460.6)	0 (0 to 0)	0 (0 to 0)	76 (65.7 to 85.6)	-4.7 (-7.6 to -1.8)	92.2 (-53.4 to 1235.1)	6.3 (-69.9 to 469.2)
	Hemoglobinopathies and hemolytic anemias	Both	5947 (4210 to 8088)	158.6 (111.5 to 215.6)	20 (15 to 27)	0.6 (0.5 to 0.7)	2666 (1722 to 4039)	67.6 (44.1 to 101.3)	9 (8 to 10)	0.3 (0.2 to 0.3)	-55.2 (-66.2 to -41.8)	-57.4 (-67 to -45.9)	-55.2 (-67.6 to -37.2)	-53.6 (-63.5 to -39.8)
		Female	3665 (2445 to 5155)	209 (138.4 to 298.2)	10 (6 to 14)	0.6 (0.4 to 0.7)	1701 (1041 to 2650)	85.8 (53.3 to 130.7)	4 (3 to 5)	0.2 (0.2 to 0.3)	-53.6 (-67.7 to -35.5)	-58.9 (-70.2 to -44.3)	-57.4 (-73.3 to -26)	-55.7 (-69.2 to -33.3)
		Male	2282 (1559 to 3214)	109.2 (75.9 to 150.3)	10 (7 to 14)	0.6 (0.4 to 0.7)	965 (629 to 1479)	50.2 (32.5 to 78)	5 (4 to 6)	0.3 (0.2 to 0.3)	-57.7 (-70.6 to -38.9)	-54 (-67.3 to -33)	-53.2 (-67.3 to -27.8)	-51.2 (-63.5 to -30.3)
		Both	10109 (6826 to 15418)	328.8 (222.6 to 467.7)	35 (18 to 92)	1.3 (0.7 to 3.2)	13673 (9845 to 18278)	328 (239.4 to 435.1)	73 (52 to 88)	2.2 (1.6 to 2.7)	35.3 (-11.3 to 61.8)	-0.2 (-25.9 to 14.7)	111.1 (-31.6 to 333.6)	70.5 (-34.8 to 235.6)

Province	Cause	Sex	1990				2019				1990-2019 percent change (%)			
			DALYs		Death		DALYs		Death		DALYs		Death	
			Number	Rate	Number	Rate	Number	Rate	Number	Rate	Number	Rate	Number	Rate
	Endocrine, metabolic, blood, and immune disorders	Female	6140 (4159 to 9045)	412.5 (281.1 to 584.4)	16 (8 to 45)	1.3 (0.7 to 3.5)	8542 (6031 to 11632)	411.5 (292.7 to 556.3)	38 (21 to 49)	2.3 (1.3 to 3)	39.1 (-4.7 to 64.9)	-0.2 (-24.5 to 15.4)	139.6 (-39.8 to 428.8)	77.2 (-46.2 to 295.5)
		Male	3969 (2589 to 6702)	248 (165.7 to 377)	19 (9 to 51)	1.3 (0.7 to 3.1)	5131 (3747 to 6808)	247.7 (182.6 to 326.4)	35 (24 to 46)	2.2 (1.5 to 2.8)	29.3 (-21.1 to 66.9)	-0.1 (-29.1 to 21)	87.2 (-30.6 to 326.2)	64.4 (-26.4 to 242.8)
	Oral disorders	Both	7168 (4222 to 11323)	317.9 (187.6 to 499)	--	--	13395 (7925 to 21438)	313.2 (185.8 to 494.4)	--	--	86.9 (78.9 to 94.8)	-1.5 (-3.7 to 0.6)	--	--
		Female	3410 (1995 to 5421)	307.7 (180.4 to 483)	--	--	6473 (3833 to 10462)	303.7 (180.5 to 487.3)	--	--	89.8 (80 to 99.8)	-1.3 (-4.1 to 1.5)	--	--
		Male	3758 (2216 to 5909)	326.9 (195.1 to 513.7)	--	--	6922 (4097 to 10930)	323.3 (193 to 502)	--	--	84.2 (75.6 to 92.9)	-1.1 (-4.1 to 1.9)	--	--
	Sudden infant death syndrome	Both	5643 (909 to 18845)	119.1 (19.2 to 397.9)	64 (10 to 213)	1.3 (0.2 to 4.5)	442 (216 to 855)	14.9 (7.3 to 28.8)	5 (2 to 10)	0.2 (0.1 to 0.3)	-92.2 (-97.7 to -39.8)	-87.5 (-96.3 to -3.9)	-92.2 (-97.7 to -39.8)	-87.5 (-96.3 to -3.9)
		Female	2615 (457 to 9015)	112.9 (19.7 to 389.4)	30 (5 to 102)	1.3 (0.2 to 4.4)	212 (99 to 440)	14.7 (6.8 to 30.5)	2 (1 to 5)	0.2 (0.1 to 0.3)	-91.9 (-97.7 to -40.6)	-87 (-96.2 to -4.7)	-91.9 (-97.7 to -40.6)	-87 (-96.2 to -4.7)
		Male	3028 (390 to 10655)	125.1 (16.1 to 440.1)	34 (4 to 120)	1.4 (0.2 to 5)	230 (101 to 469)	15.1 (6.6 to 30.7)	3 (1 to 5)	0.2 (0.1 to 0.3)	-92.4 (-97.8 to -35.9)	-87.9 (-96.6 to 1.8)	-92.4 (-97.8 to -35.9)	-87.9 (-96.6 to 1.8)
	**Injuries**	Both	261431 (214977 to 311097)	7064.7 (5902.6 to 8318.1)	3669 (2956 to 4364)	116.2 (95.2 to 137.7)	128332 (113630 to 145683)	3051 (2710.2 to 3448.1)	2215 (1916 to 2589)	55 (47.8 to 64.4)	-50.9 (-60.5 to -38.4)	-56.8 (-64.8 to -47.2)	-39.6 (-52.1 to -23)	-52.6 (-62.3 to -40.2)
		Female	85542 (66446 to 107869)	4430.4 (3513.9 to 5453.5)	1112 (855 to 1401)	67.2 (52.5 to 84.1)	32228 (27528 to 37663)	1610.6 (1381.2 to 1872.2)	517 (427 to 627)	27.7 (23.1 to 33.2)	-62.3 (-71.6 to -49)	-63.6 (-72.4 to -52.7)	-53.5 (-66.5 to -34.8)	-58.8 (-69.8 to -43.3)
		Male	175888 (143277 to 209605)	9607.2 (7911.8 to 11371.2)	2556 (2024 to 3099)	163.1 (128.1 to 197.9)	96105 (82322 to 112174)	4430.6 (3812.7 to 5156.2)	1697 (1414 to 2052)	81.5 (68.1 to 98.4)	-45.4 (-56.5 to -30.8)	-53.9 (-63.2 to -41.7)	-33.6 (-49.2 to -12.7)	-50.1 (-61.8 to -33.7)
	Transport injuries	Both	140115 (108366 to 170505)	3769.9 (2963.1 to 4513.6)	2118 (1660 to 2574)	67.4 (52.2 to 82.2)	66079 (56348 to 78188)	1577.6 (1349.4 to 1871.9)	1299 (1081 to 1598)	31.6 (26.5 to 39.4)	-52.8 (-63.3 to -37.8)	-58.2 (-67.1 to -45.8)	-38.7 (-53.1 to -18.8)	-53.1 (-64 to -37.9)
		Female	43736 (30696 to 56561)	2202.2 (1578.2 to 2796.6)	597 (416 to 768)	34.8 (24.7 to 44.4)	14859 (12270 to 17645)	737.1 (611.1 to 877.8)	279 (224 to 339)	14.1 (11.4 to 17.1)	-66 (-76.1 to -50.4)	-66.5 (-76 to -51.7)	-53.2 (-67.2 to -30.4)	-59.4 (-71 to -40.2)
		Male	96378 (73077 to 118925)	5282.5 (4055.6 to 6488.3)	1521 (1143 to 1890)	98.7 (72.8 to 126.1)	51221 (42453 to 62761)	2380.8 (1986.5 to 2915)	1020 (830 to 1299)	48.6 (39.7 to 62.8)	-46.9 (-60.3 to -26.9)	-54.9 (-66.3 to -38.4)	-33 (-51.2 to -5.5)	-50.8 (-64.2 to -29.9)
	Road injuries	Both	137413 (106436 to 167089)	3691.4 (2907.3 to 4417.4)	2078 (1623 to 2522)	66 (51.2 to 80.1)	64220 (54781 to 76196)	1533.3 (1313.3 to 1823.2)	1266 (1052 to 1559)	30.8 (25.8 to 38.4)	-53.3 (-63.6 to -38.5)	-58.5 (-67.4 to -46.1)	-39.1 (-53.4 to -19.3)	-53.3 (-64.3 to -38.3)
		Female	43119 (30324 to 55961)	2168.8 (1556.7 to 2760.7)	589 (411 to 761)	34.3 (24.4 to 43.8)	14499 (11950 to 17234)	719.4 (595.9 to 857.1)	274 (220 to 333)	13.9 (11.2 to 16.8)	-66.4 (-76.4 to -50.8)	-66.8 (-76.2 to -52.1)	-53.5 (-67.4 to -30.8)	-59.6 (-71.3 to -40.6)
		Male	94294 (71562 to 116516)	5160.7 (3966.6 to 6343.6)	1489 (1126 to 1856)	96.5 (70.8 to 123.7)	49721 (41157 to 60639)	2310.9 (1921.8 to 2813)	992 (803 to 1263)	47.3 (38.5 to 60.9)	-47.3 (-60.7 to -27.4)	-55.2 (-66.6 to -39)	-33.4 (-51.5 to -6.2)	-51 (-64.4 to -30.2)
	Other transport injuries	Both	2702 (1736 to 4266)	78.5 (51.1 to 120)	40 (23 to 65)	1.4 (0.8 to 2.2)	1859 (1555 to 2365)	44.4 (36.9 to 56.6)	33 (26 to 44)	0.8 (0.7 to 1.1)	-31.2 (-58.2 to 22.8)	-43.5 (-64.5 to -3.4)	-18 (-52.7 to 58.4)	-40.7 (-66 to 13.6)
		Female	618 (374 to 938)	33.5 (21.3 to 48.1)	8 (4 to 12)	0.5 (0.2 to 0.7)	360 (288 to 438)	17.8 (14.3 to 21.5)	5 (4 to 6)	0.3 (0.2 to 0.3)	-41.8 (-64.1 to -2.3)	-46.9 (-66.3 to -15.6)	-35 (-63.5 to 23.9)	-43.7 (-67.6 to 4.6)
		Male	2084 (1083 to 3709)	121.8 (69.5 to 205.8)	32 (16 to 57)	2.2 (1.2 to 3.8)	1500 (1206 to 2000)	69.9 (56.3 to 93.2)	28 (21 to 39)	1.3 (1 to 1.9)	-28.1 (-60.7 to 47.3)	-42.6 (-67.3 to 7.3)	-14 (-54.4 to 86.3)	-39.8 (-68 to 19.5)

Province	Cause	Sex	1990				2019				1990-2019 percent change (%)			
			DALYs		Death		DALYs		Death		DALYs		Death	
			Number	Rate	Number	Rate	Number	Rate	Number	Rate	Number	Rate	Number	Rate
	Unintentional injuries	Both	91255 (69976 to 113819)	2391.6 (1910.2 to 2918.7)	1171 (908 to 1450)	36.5 (29.1 to 43.9)	39251 (33605 to 45549)	944.8 (814.6 to 1091.3)	577 (500 to 680)	15.5 (13.5 to 18.2)	-57 (-66.9 to -41.9)	-60.5 (-68.5 to -49)	-50.7 (-63 to -29.4)	-57.5 (-66.7 to -42.4)
		Female	33510 (22336 to 47357)	1728.8 (1191.7 to 2350.3)	413 (263 to 589)	26 (17.2 to 35)	12033 (10021 to 14573)	612.4 (514 to 732.8)	172 (142 to 208)	10.2 (8.5 to 12.2)	-64.1 (-75.5 to -43.7)	-64.6 (-75.1 to -48.5)	-58.4 (-73.2 to -33.2)	-60.7 (-73.3 to -40)
		Male	57745 (44240 to 71904)	3028.6 (2394.2 to 3690.5)	758 (576 to 949)	46.6 (36.1 to 57.7)	27217 (22814 to 31964)	1264.6 (1064.8 to 1479.5)	405 (333 to 499)	20.7 (17.1 to 25.3)	-52.9 (-64.2 to -35.1)	-58.2 (-67.3 to -44.6)	-46.6 (-61 to -20.1)	-55.7 (-66.7 to -36.7)
	Falls	Both	13430 (10472 to 16851)	437.6 (349.5 to 531.7)	153 (117 to 197)	6.9 (5.3 to 8.9)	12187 (9614 to 14929)	294.3 (234.8 to 359.2)	158 (131 to 202)	4.8 (4.1 to 6)	-9.3 (-30.2 to 16.4)	-32.7 (-44.2 to -18.7)	3.4 (-23.4 to 47.4)	-30.1 (-46.4 to -2.4)
		Female	4087 (2618 to 5848)	267 (203.6 to 344.9)	44 (29 to 64)	4.5 (3.2 to 6.4)	3339 (2634 to 4261)	174.8 (140.2 to 218.7)	46 (37 to 58)	3.3 (2.6 to 4.1)	-18.3 (-46 to 29.8)	-34.6 (-50.2 to -16.6)	6.8 (-31.1 to 66.4)	-27.8 (-48.6 to 17)
		Male	9342 (6661 to 11971)	599.6 (463.7 to 750)	109 (75 to 150)	9.1 (6.6 to 12.2)	8848 (6862 to 11105)	410.5 (321.8 to 514.3)	112 (87 to 155)	6.3 (5.1 to 8.4)	-5.3 (-29.4 to 38.8)	-31.5 (-45.3 to -8.2)	2 (-28.8 to 70)	-30.4 (-48.4 to 2.5)
	Drowning	Both	11484 (7927 to 16198)	242.2 (169 to 335.4)	148 (103 to 206)	3.3 (2.3 to 4.6)	1538 (1241 to 2359)	40 (31.9 to 61.4)	26 (21 to 41)	0.7 (0.5 to 1.1)	-86.6 (-91.3 to -76.5)	-83.5 (-89.1 to -71.9)	-82.3 (-88.4 to -69.7)	-79.9 (-86.6 to -66)
		Female	3414 (1858 to 5532)	139.7 (78.4 to 223)	41 (23 to 66)	1.8 (1 to 2.7)	225 (180 to 283)	12.1 (9.6 to 15.5)	3 (3 to 4)	0.2 (0.1 to 0.2)	-93.4 (-96.3 to -86.5)	-91.3 (-95.1 to -82.4)	-91.9 (-95.5 to -83.7)	-89.7 (-94 to -80.3)
		Male	8070 (5033 to 11975)	341.2 (213.4 to 497.6)	107 (66 to 156)	4.9 (3.1 to 7)	1313 (1032 to 2146)	66.3 (50.8 to 107.4)	23 (18 to 38)	1.1 (0.9 to 1.9)	-83.7 (-89.9 to -68.4)	-80.6 (-87.9 to -63.3)	-78.6 (-86.5 to -58.8)	-76.6 (-84.9 to -56)
	Fire, heat, and hot substances	Both	24255 (9783 to 40584)	601.2 (267.8 to 965.5)	336 (137 to 552)	10 (4.7 to 15.4)	5345 (3888 to 6720)	130.6 (96.4 to 166.1)	111 (83 to 141)	3 (2.3 to 3.7)	-78 (-87.1 to -50)	-78.3 (-86.8 to -54.4)	-66.8 (-80.3 to -25.5)	-70.5 (-81.7 to -40.9)
		Female	10531 (1883 to 21892)	539.7 (126 to 1061.1)	145 (26 to 295)	9 (2.6 to 16.6)	2435 (1722 to 3347)	124.8 (88.2 to 173.5)	50 (36 to 68)	2.8 (2 to 3.8)	-76.9 (-89.7 to 16.9)	-76.9 (-89.3 to -10.4)	-65.4 (-84.9 to 79.3)	-68.9 (-85.1 to -3.7)
		Male	13724 (3806 to 21778)	660.3 (212.7 to 1007.8)	191 (55 to 296)	11 (3.9 to 16.4)	2910 (1831 to 3753)	136.2 (87.2 to 175.4)	61 (38 to 80)	3.1 (2 to 4)	-78.8 (-86.7 to -20.5)	-79.4 (-86.7 to -32.9)	-67.9 (-79.8 to 17.8)	-71.6 (-81.8 to -18.8)
	Poisonings	Both	11550 (6729 to 16590)	267.8 (160 to 369.6)	155 (92 to 215)	4.1 (2.5 to 5.4)	3112 (2198 to 3735)	73.7 (53.9 to 88.7)	62 (42 to 76)	1.5 (1 to 1.8)	-73.1 (-82.4 to -56)	-72.5 (-81.2 to -56.9)	-59.8 (-73.1 to -37)	-63.7 (-74.3 to -46.9)
		Female	4850 (2595 to 7851)	218 (122.3 to 342.1)	62 (35 to 98)	3.1 (1.9 to 4.6)	969 (743 to 1235)	49.6 (38.2 to 62.6)	19 (14 to 24)	1 (0.8 to 1.2)	-80 (-88.5 to -64.8)	-77.3 (-86.4 to -61.8)	-70.1 (-82 to -49.5)	-69.2 (-80.4 to -51.2)
		Male	6700 (3388 to 10477)	315.9 (169.6 to 476.1)	93 (49 to 142)	5.1 (2.7 to 7.4)	2143 (1248 to 2749)	96.9 (59.1 to 123.5)	44 (24 to 57)	2 (1.1 to 2.6)	-68 (-81 to -40.1)	-69.3 (-80.8 to -46)	-52.9 (-70.8 to -16.6)	-60.4 (-74.1 to -32.5)
	Exposure to mechanical forces	Both	8967 (6004 to 13538)	273.4 (189.8 to 403.7)	111 (68 to 186)	3.7 (2.4 to 6.3)	6900 (5598 to 8682)	161.1 (131.8 to 201.6)	87 (69 to 117)	2.1 (1.7 to 2.8)	-23.1 (-46 to 14.2)	-41.1 (-57.7 to -17)	-21.9 (-48.1 to 32.4)	-43.9 (-62.5 to -8.4)
		Female	2002 (1369 to 3232)	117.6 (85.3 to 172)	21 (13 to 37)	1.3 (0.9 to 2.4)	1510 (1199 to 2004)	73.8 (58.3 to 97.4)	14 (11 to 20)	0.7 (0.6 to 1.1)	-24.6 (-53 to 13.7)	-37.3 (-56.3 to -13.9)	-31.5 (-58.5 to 19.1)	-44.5 (-63.5 to -15.1)
		Male	6965 (4392 to 10846)	423.5 (276.6 to 661.8)	91 (52 to 156)	6 (3.6 to 10.6)	5390 (4326 to 6893)	245.1 (197.2 to 310.4)	73 (56 to 100)	3.4 (2.6 to 4.6)	-22.6 (-46.5 to 21.1)	-42.1 (-59.9 to -12.8)	-19.8 (-48.7 to 43.8)	-43.7 (-64.8 to -1.5)
	Adverse effects of medical treatment	Both	5041 (3261 to 6662)	148.3 (97.7 to 198.5)	86 (57 to 115)	3.5 (2.4 to 4.9)	1667 (1351 to 2263)	41.6 (33.7 to 56.9)	46 (36 to 66)	1.3 (1 to 1.9)	-66.9 (-78.1 to -44)	-72 (-82 to -54.3)	-45.9 (-67.1 to -8.8)	-63.4 (-78.6 to -38.6)
		Female	2093 (1228 to 2933)	121.4 (78.9 to 164.6)	34 (22 to 47)	2.8 (1.8 to 4.9)	690 (537 to 910)	35.6 (27.9 to 47.5)	19 (14 to 27)	1.1 (0.8 to 1.6)	-67 (-78.6 to -43.3)	-70.6 (-80.6 to -53.4)	-43.1 (-64.3 to -7.5)	-61.2 (-78 to -34.7)
		Male	2948 (1774 to 4259)	173.8 (100.5 to 253.3)	52 (30 to 75)	4.2 (2.5 to 6.3)	977 (730 to 1477)	47.4 (35.3 to 71.3)	27 (19 to 43)	1.5 (1 to 2.4)	-66.9 (-80.5 to -36.2)	-72.7 (-84.4 to -46.5)	-47.8 (-71.6 to 7)	-64.7 (-81 to -29.8)
	Animal contact	Both	895 (563 to 1253)	21.8 (15 to 29.6)	10 (5 to 14)	0.3 (0.2 to 0.4)	376 (282 to 511)	9.1 (6.9 to 12.4)	3 (2 to 5)	0.1 (0.1 to 0.1)	-58 (-71.2 to -32.1)	-58.2 (-69.6 to -37.9)	-71.2 (-81.9 to -44.5)	-70.1 (-80 to -49.9)

Province	Cause	Sex	1990				2019				1990-2019 percent change (%)			
			DALYs		Death		DALYs		Death		DALYs		Death	
			Number	Rate	Number	Rate	Number	Rate	Number	Rate	Number	Rate	Number	Rate
		Female	321 (180 to 547)	16.5 (10.3 to 25.9)	3 (1 to 6)	0.2 (0.1 to 0.3)	173 (123 to 250)	8.5 (6.1 to 12.3)	1 (1 to 2)	0 (0 to 0.1)	-46 (-68.8 to -2)	-48.6 (-66.8 to -20.6)	-74.9 (-88.5 to -32.1)	-71.9 (-85.3 to -43.9)
		Male	575 (309 to 845)	26.9 (16 to 37.5)	7 (3 to 10)	0.4 (0.2 to 0.5)	203 (157 to 270)	9.7 (7.6 to 13)	2 (2 to 3)	0.1 (0.1 to 0.2)	-64.7 (-77.2 to -34.1)	-63.8 (-75.5 to -39.4)	-69.6 (-82 to -37.2)	-69.1 (-81.1 to -45.3)
	Foreign body	Both	7185 (4924 to 10435)	160.2 (114.2 to 226.9)	80 (55 to 116)	1.9 (1.4 to 2.6)	1984 (1643 to 2351)	51.4 (42.1 to 60.5)	24 (21 to 28)	0.7 (0.6 to 0.8)	-72.4 (-82.4 to -58.4)	-67.9 (-79.2 to -53.6)	-69.7 (-80.9 to -54.6)	-63.2 (-76 to -47.7)
		Female	3421 (2182 to 5470)	153.6 (100.9 to 240.2)	38 (24 to 61)	1.8 (1.2 to 2.7)	725 (577 to 892)	39.1 (31 to 48.8)	8 (7 to 10)	0.5 (0.4 to 0.6)	-78.8 (-88 to -63.2)	-74.5 (-85.3 to -57.3)	-78.6 (-87.9 to -62.2)	-71.7 (-83.1 to -52.3)
		Male	3764 (2289 to 5640)	166.6 (107.7 to 244.4)	42 (26 to 64)	2 (1.4 to 2.9)	1260 (1019 to 1526)	63.1 (50.7 to 76.4)	16 (13 to 20)	0.9 (0.7 to 1.1)	-66.5 (-79.6 to -47.2)	-62.1 (-76.7 to -42.2)	-61.8 (-77.2 to -36.9)	-55.9 (-72.8 to -32.6)
	Environmental heat and cold exposure	Both	498 (354 to 797)	16.9 (12.2 to 26.5)	3 (2 to 9)	0.2 (0.1 to 0.4)	553 (407 to 731)	12.9 (9.5 to 17)	3 (2 to 5)	0.1 (0.1 to 0.1)	10.9 (-26.8 to 33.8)	-23.9 (-46.7 to -9.8)	1.2 (-48.4 to 78)	-42.2 (-67.4 to 1)
		Female	219 (152 to 342)	15.2 (10.8 to 22.4)	1 (1 to 3)	0.1 (0 to 0.3)	266 (190 to 366)	12.6 (9 to 17.3)	1 (1 to 2)	0.1 (0 to 0.1)	21.4 (-21.3 to 47.4)	-17.2 (-41.2 to -2.5)	28 (-52.2 to 180.6)	-23.5 (-72 to 74.1)
		Male	279 (196 to 493)	18.5 (13.1 to 32.4)	2 (1 to 6)	0.2 (0.1 to 0.6)	286 (210 to 386)	13.1 (9.6 to 17.6)	2 (1 to 4)	0.1 (0.1 to 0.2)	2.7 (-33.7 to 28.6)	-29.4 (-52.5 to -11.7)	-9.4 (-49.8 to 60.8)	-49.8 (-70.3 to -15.1)
	Exposure to forces of nature	Both	135 (113 to 164)	4 (3.3 to 4.8)	1 (1 to 1)	0 (0 to 0)	404 (291 to 550)	9.1 (6.6 to 12.3)	0 (0 to 0)	0 (0 to 0)	199.9 (148.5 to 250.9)	130 (94.9 to 167.2)	-80.4 (-80.4 to -80.4)	-85 (-85 to -85)
		Female	49 (41 to 59)	2.9 (2.4 to 3.5)	0 (0 to 1)	0 (0 to 0)	134 (95 to 188)	6.3 (4.5 to 8.8)	0 (0 to 0)	0 (0 to 0)	174.4 (121.9 to 231)	120.6 (82.6 to 162.1)	-80.9 (-80.9 to -80.9)	-85.3 (-85.3 to -85.3)
		Male	86 (72 to 105)	5.1 (4.2 to 6.2)	1 (1 to 1)	0 (0 to 0.1)	270 (195 to 364)	11.8 (8.6 to 16)	0 (0 to 0)	0 (0 to 0)	214.4 (161.6 to 268.2)	134.2 (98.4 to 170)	-80.1 (-80.1 to -80.1)	-84.9 (-84.9 to -84.9)
	Other unintentional injuries	Both	7816 (6099 to 10443)	218.3 (170.2 to 289.1)	87 (64 to 128)	2.5 (1.9 to 3.6)	5185 (4144 to 6398)	120.9 (96.8 to 148.8)	55 (44 to 67)	1.3 (1 to 1.6)	-33.7 (-54.1 to -10.1)	-44.6 (-60.9 to -27.8)	-37.2 (-61.4 to -4.9)	-47.7 (-68 to -22.2)
		Female	2525 (1728 to 3438)	137.2 (99.6 to 179.9)	24 (15 to 35)	1.3 (0.8 to 1.8)	1567 (1134 to 2105)	75.2 (54.9 to 100.6)	10 (7 to 13)	0.5 (0.4 to 0.7)	-37.9 (-59.2 to -3.8)	-45.2 (-61.1 to -22.7)	-59.6 (-76.5 to -24)	-59.6 (-75.2 to -28.4)
		Male	5292 (3947 to 7754)	297.1 (221.8 to 429.4)	63 (43 to 101)	3.7 (2.5 to 5.9)	3617 (2833 to 4395)	164.5 (130 to 199)	45 (34 to 57)	2.1 (1.6 to 2.6)	-31.6 (-57.3 to -1)	-44.6 (-64.8 to -21.5)	-28.7 (-61.5 to 18.1)	-44 (-70.5 to -7.3)
	Self-harm and interpersonal violence	Both	30061 (24026 to 36003)	903.1 (731.1 to 1077.7)	380 (286 to 467)	12.3 (9.3 to 14.9)	23002 (18887 to 26819)	528.6 (434.4 to 617.6)	339 (258 to 412)	7.9 (6 to 9.5)	-23.5 (-37.8 to -4.7)	-41.5 (-52.3 to -27.8)	-10.8 (-32.2 to 19.9)	-35.9 (-51.6 to -14.4)
		Female	8295 (6162 to 10656)	499.4 (381.5 to 629.8)	103 (70 to 139)	6.5 (4.7 to 8.7)	5335 (4466 to 6402)	261 (217 to 311.7)	66 (52 to 84)	3.3 (2.6 to 4.2)	-35.7 (-51.7 to -10.9)	-47.7 (-60.6 to -29.8)	-35.4 (-55.5 to -0.8)	-48.5 (-64.1 to -22)
		Male	21766 (15947 to 26869)	1296.1 (968.3 to 1588.6)	277 (187 to 354)	17.8 (12.1 to 22.7)	17667 (13320 to 21183)	785.2 (586.6 to 953.4)	273 (188 to 342)	12.2 (8.4 to 15.3)	-18.8 (-36.2 to 6.9)	-39.4 (-52.3 to -20.1)	-1.6 (-27.9 to 44.3)	-31.5 (-50.4 to 0.9)
	Self-harm	Both	15565 (9584 to 19936)	464.3 (298.6 to 593.1)	258 (158 to 332)	8.5 (5.5 to 10.9)	11971 (8278 to 14971)	278.2 (191.1 to 348.5)	230 (156 to 288)	5.4 (3.6 to 6.7)	-23.1 (-44 to 12.2)	-40.1 (-56 to -15.2)	-10.7 (-35 to 31.3)	-37 (-53.9 to -9.4)
		Female	5460 (3549 to 7483)	320.8 (216.9 to 431.7)	84 (55 to 117)	5.3 (3.7 to 7.2)	2604 (1952 to 3353)	131.6 (99.4 to 171.3)	45 (33 to 58)	2.3 (1.7 to 2.9)	-52.3 (-68.5 to -22.5)	-59 (-72.8 to -34)	-47 (-65.9 to -13.6)	-57.5 (-72.1 to -32.3)
		Male	10105 (4640 to 13830)	603 (310.6 to 810.6)	173 (84 to 236)	11.5 (6.1 to 15.5)	9367 (5406 to 12295)	418.1 (237.3 to 548.7)	185 (107 to 243)	8.3 (4.7 to 10.8)	-7.3 (-36.6 to 52.4)	-30.7 (-51.7 to 11.6)	6.9 (-26.2 to 75.3)	-27.6 (-49.7 to 16.6)
	Interpersonal violence	Both	4953 (3456 to 6680)	150.7 (108.3 to 199.1)	60 (37 to 87)	2 (1.2 to 2.8)	6052 (5103 to 7154)	141.9 (119.1 to 169.5)	76 (60 to 96)	1.8 (1.4 to 2.2)	22.2 (-11 to 73.3)	-5.8 (-29.4 to 31.6)	25.7 (-16.3 to 103)	-9.6 (-38.3 to 44.3)

Province	Cause	Sex	1990				2019				1990-2019 percent change (%)			
			DALYs		Death		DALYs		Death		DALYs		Death	
			Number	Rate	Number	Rate	Number	Rate	Number	Rate	Number	Rate	Number	Rate
		Female	1626 (1144 to 2164)	96.8 (70.4 to 125.6)	14 (8 to 21)	0.9 (0.5 to 1.3)	2001 (1603 to 2446)	97.6 (77.6 to 118.8)	18 (12 to 24)	0.9 (0.6 to 1.2)	23 (-8.2 to 69)	0.8 (-22.1 to 34.1)	25 (-22.3 to 115.9)	2.7 (-32.9 to 78.1)
		Male	3326 (2040 to 4834)	202.6 (127.2 to 287.4)	46 (25 to 71)	3 (1.6 to 4.6)	4051 (3249 to 5043)	184.3 (147.2 to 232.1)	58 (44 to 77)	2.6 (2 to 3.5)	21.8 (-18 to 96.8)	-9 (-37.1 to 44)	25.9 (-21.2 to 133.3)	-12.9 (-44.7 to 63.3)
	Conflict and terrorism	Both	7725 (5654 to 10571)	231.9 (169 to 320.9)	35 (31 to 38)	0.9 (0.8 to 1)	2922 (1882 to 4436)	62.4 (40.3 to 93.8)	2 (1 to 2)	0 (0 to 0)	-62.2 (-67.8 to -56.9)	-73.1 (-77.1 to -69.3)	-95.5 (-95.5 to -95.5)	-95.6 (-95.6 to -95.6)
		Female	915 (554 to 1519)	64.1 (40.1 to 102.9)	0 (0 to 0)	0 (0 to 0)	469 (290 to 794)	19.4 (12.2 to 32.4)	0 (0 to 0)	0 (0 to 0)	-48.8 (-56.6 to -39.7)	-69.7 (-75.2 to -63.9)	156.7 (156.7 to 156.7)	124.1 (124.1 to 124.1)
		Male	6811 (5062 to 9228)	396.3 (292.7 to 538.6)	34 (31 to 38)	1.8 (1.6 to 2)	2453 (1584 to 3658)	104.8 (67.9 to 154.8)	1 (1 to 1)	0.1 (0.1 to 0.1)	-64 (-69.6 to -58.4)	-73.6 (-77.6 to -69.7)	-96.7 (-96.7 to -96.7)	-96.9 (-96.9 to -96.9)
	Executions and police conflict	Both	1818 (1493 to 2276)	56.3 (45.1 to 69.7)	27 (22 to 34)	0.9 (0.7 to 1.2)	2057 (1522 to 2835)	46.1 (34.8 to 62.7)	31 (21 to 46)	0.7 (0.5 to 1)	13.2 (-15.5 to 61.5)	-18.1 (-38.8 to 17.9)	15.8 (-19.1 to 75.6)	-23.6 (-46.1 to 14.6)
		Female	294 (253 to 355)	17.7 (15.2 to 21.7)	4 (3 to 5)	0.3 (0.2 to 0.4)	262 (218 to 323)	12.4 (10.5 to 15.1)	3 (3 to 4)	0.2 (0.1 to 0.2)	-11 (-25.9 to 5.6)	-29.8 (-42.7 to -18.1)	-13.9 (-31.1 to 8.1)	-41.2 (-53.1 to -28.7)
		Male	1524 (1210 to 1951)	94.2 (73.6 to 120.4)	23 (18 to 30)	1.5 (1.2 to 2)	1795 (1271 to 2574)	78.1 (56.7 to 109.8)	28 (18 to 42)	1.2 (0.8 to 1.8)	17.8 (-15.8 to 76.1)	-17.1 (-41.4 to 25.1)	21 (-19.4 to 90.4)	-21.3 (-47.4 to 23.7)
**Fars**	**All causes**	Both	1333408 (1187385 to 1487345)	42603.9 (38207.7 to 47593.2)	19069 (16945 to 21599)	1018.3 (896.6 to 1152.6)	1305200 (1122927 to 1494990)	27203.4 (23642.1 to 30884.8)	25829 (22689 to 28920)	663.7 (586.8 to 738.5)	-2.1 (-13.7 to 11.5)	-36.1 (-43.5 to -28.4)	35.5 (13.6 to 62.2)	-34.8 (-45.2 to -22.9)
		Female	571664 (500382 to 655056)	37327.7 (32468.9 to 43400.8)	7521 (6487 to 8737)	854.5 (731.9 to 993.3)	577333 (483658 to 683985)	24549.7 (20874.7 to 28770.5)	10380 (8910 to 12039)	576 (500 to 660.9)	1 (-12.2 to 15.7)	-34.2 (-42.5 to -25.7)	38 (12.3 to 71.2)	-32.6 (-45.1 to -16.6)
		Male	761744 (670713 to 855573)	47562.3 (41185.1 to 54281)	11548 (9745 to 13616)	1175.8 (973.8 to 1391.1)	727866 (624543 to 837512)	29803.3 (25643.4 to 34047.3)	15449 (13035 to 18197)	751.6 (638.2 to 881)	-4.4 (-19.4 to 11.8)	-37.3 (-47.3 to -26.1)	33.8 (4.9 to 69.2)	-36.1 (-50 to -18.7)
	**Communicable, maternal, neonatal, and nutritional diseases**	Both	395364 (323236 to 496275)	8176.2 (6708.1 to 10864.6)	4479 (3600 to 6164)	111.8 (91 to 172.2)	107420 (92820 to 123000)	2550.2 (2195.9 to 2949.7)	1456 (1275 to 1634)	38.3 (33.4 to 43.2)	-72.8 (-78.9 to -65.6)	-68.8 (-76.9 to -60.5)	-67.5 (-76.5 to -58.1)	-65.7 (-77.7 to -56.1)
		Female	179257 (146561 to 226277)	7600.3 (6232.7 to 10016)	2002 (1608 to 2706)	100.6 (81.1 to 148.7)	47382 (40510 to 54750)	2304.5 (1965.6 to 2673.3)	594 (505 to 687)	33.1 (28.2 to 38.2)	-73.6 (-80 to -66.4)	-69.7 (-77.9 to -61.4)	-70.3 (-78.9 to -60.9)	-67.1 (-78.5 to -57.5)
		Male	216108 (174453 to 274634)	8727 (7045.6 to 11945.8)	2477 (1958 to 3510)	123.2 (97.3 to 197.7)	60038 (52174 to 69182)	2785.6 (2391.7 to 3229.5)	862 (738 to 994)	43.3 (37 to 49.8)	-72.2 (-79.2 to -64.5)	-68.1 (-76.7 to -59.1)	-65.2 (-75.5 to -54.1)	-64.8 (-78.2 to -53.4)
	HIV/AIDS and sexually transmitted infections	Both	1269 (756 to 2163)	34.8 (22.1 to 53.1)	12 (6 to 22)	0.3 (0.2 to 0.5)	7625 (6004 to 10099)	135.1 (105.1 to 176.4)	128 (98 to 181)	2.3 (1.7 to 3.1)	501 (248.3 to 930.5)	288.4 (152.1 to 512.5)	986.4 (456.2 to 2154.9)	585 (293.3 to 1188.2)
		Female	568 (323 to 998)	31.8 (19.4 to 51.7)	4 (2 to 9)	0.2 (0.1 to 0.4)	2524 (1740 to 3479)	97.7 (66.9 to 133.7)	37 (24 to 57)	1.5 (0.9 to 2.2)	344.3 (151.8 to 711.2)	207 (86.1 to 425.4)	782.3 (283.7 to 2126.1)	578.2 (232.2 to 1322.2)
		Male	701 (412 to 1167)	37.6 (23.5 to 56.7)	8 (4 to 13)	0.4 (0.2 to 0.7)	5101 (4182 to 6819)	171 (140.7 to 224.7)	91 (72 to 128)	3 (2.4 to 4.2)	627.9 (316 to 1156)	354.8 (193.7 to 620.8)	1101.1 (556.4 to 2308.6)	590.5 (318.1 to 1262.4)

Province	Cause	Sex	1990				2019				1990-2019 percent change (%)			
			DALYs		Death		DALYs		Death		DALYs		Death	
			Number	Rate	Number	Rate	Number	Rate	Number	Rate	Number	Rate	Number	Rate
	HIV/AIDS	Both	279 (125 to 399)	9.5 (4 to 13.5)	4 (1 to 7)	0.2 (0 to 0.2)	6579 (5115 to 8927)	114.2 (88.5 to 152.2)	123 (94 to 176)	2.1 (1.6 to 3)	2261.8 (1356.6 to 5405.4)	1107.7 (638.8 to 2855.4)	2708.8 (1503.4 to 8505.5)	1167.8 (624.7 to 3914.1)
		Female	55 (26 to 81)	3.2 (1.5 to 4.6)	1 (0 to 1)	0.1 (0 to 0.1)	1922 (1222 to 2782)	74.4 (47.5 to 106)	35 (22 to 55)	1.3 (0.8 to 2)	3370.2 (1752 to 7453.2)	2204.7 (1120.8 to 4794.3)	4259.5 (2012.1 to 11490.4)	2302.4 (1066.5 to 6754.2)
		Male	223 (98 to 322)	15.5 (6.3 to 22.2)	4 (1 to 5)	0.3 (0.1 to 0.4)	4657 (3773 to 6359)	152.6 (124.6 to 204.8)	88 (69 to 126)	2.9 (2.3 to 4.1)	1986.8 (1219 to 4840)	884.2 (523.3 to 2413.2)	2361.3 (1344.7 to 7872.4)	944.4 (517.8 to 3393.5)
	Sexually transmitted infections excluding HIV	Both	990 (505 to 1865)	25.3 (14.3 to 43.4)	7 (2 to 17)	0.2 (0.1 to 0.3)	1046 (631 to 1710)	20.8 (12.1 to 35.2)	5 (2 to 9)	0.1 (0.1 to 0.3)	5.6 (-28.1 to 57)	-17.8 (-34 to -1.9)	-32.8 (-53.2 to 17.8)	-16.4 (-39.5 to 24.6)
		Female	513 (269 to 936)	28.6 (16.2 to 49)	3 (1 to 8)	0.2 (0.1 to 0.3)	602 (351 to 1000)	23.3 (13.7 to 38.4)	2 (1 to 4)	0.1 (0.1 to 0.2)	17.5 (-22.9 to 68.6)	-18.4 (-33.5 to -4.8)	-28.5 (-51.7 to 31.1)	-13.8 (-39.4 to 28.2)
		Male	478 (228 to 957)	22.1 (11.9 to 40)	4 (1 to 9)	0.2 (0.1 to 0.4)	443 (256 to 750)	18.4 (10 to 31.6)	3 (1 to 5)	0.1 (0.1 to 0.3)	-7.2 (-37.7 to 46.5)	-16.8 (-36.4 to 5.2)	-36.5 (-58.8 to 14.4)	-17.3 (-46.7 to 34.2)
	Respiratory infections and tuberculosis	Both	50135 (39098 to 64806)	1213.3 (996 to 1476.6)	667 (544 to 827)	28.7 (24 to 33.6)	23884 (20406 to 27939)	541.4 (461.7 to 625.8)	692 (581 to 804)	19 (16 to 22.1)	-52.4 (-64.4 to -37.4)	-55.4 (-65 to -43.9)	3.8 (-20.7 to 33.6)	-33.8 (-46.2 to -15.4)
		Female	23539 (17336 to 32157)	1116.7 (849.5 to 1448.8)	301 (228 to 394)	24.4 (19.5 to 30.7)	9843 (8119 to 11811)	465.8 (389.7 to 559.4)	282 (224 to 343)	16.6 (13.2 to 20)	-58.2 (-70.8 to -41.5)	-58.3 (-69.5 to -45)	-6.2 (-33.9 to 31.4)	-32.3 (-49.1 to -8.9)
		Male	26596 (19991 to 35287)	1312.3 (1051.1 to 1650.4)	366 (282 to 468)	33.5 (27.2 to 41)	14041 (11658 to 16629)	615 (512 to 723.8)	410 (332 to 495)	21.3 (17.2 to 25.8)	-47.2 (-61.7 to -26.6)	-53.1 (-64.8 to -38)	12.1 (-19.5 to 54.6)	-36.3 (-51.8 to -15.5)
	Tuberculosis	Both	2232 (1752 to 2721)	77 (58.3 to 95.5)	42 (31 to 54)	2.3 (1.6 to 3)	898 (757 to 1051)	17.8 (15.1 to 20.9)	23 (20 to 28)	0.6 (0.5 to 0.7)	-59.8 (-68.9 to -43.3)	-76.9 (-82.4 to -66.9)	-44.6 (-59.3 to -12)	-75.9 (-82.5 to -61.8)
		Female	939 (581 to 1346)	62.9 (37.8 to 92.8)	16 (8 to 25)	1.8 (0.9 to 2.8)	336 (265 to 427)	14 (11.3 to 17.7)	8 (6 to 12)	0.4 (0.3 to 0.6)	-64.3 (-76.1 to -31.7)	-77.8 (-85.4 to -55.3)	-48.3 (-69.2 to 33.1)	-76.4 (-86.3 to -37.2)
		Male	1292 (1024 to 1620)	90.9 (70.1 to 115.3)	26 (20 to 34)	2.9 (2.1 to 3.8)	562 (458 to 685)	21.6 (17.7 to 26.2)	15 (12 to 19)	0.7 (0.5 to 0.9)	-56.5 (-67.7 to -40.3)	-76.2 (-82.6 to -66.3)	-42.4 (-59.4 to -15.1)	-76 (-83.1 to -63.7)
	Lower respiratory infections	Both	43062 (32685 to 57233)	1018.6 (826.1 to 1280)	624 (502 to 783)	26.3 (22.1 to 31.1)	17350 (14858 to 19936)	410 (349 to 469.9)	668 (562 to 777)	18.4 (15.5 to 21.4)	-59.7 (-71.7 to -44.2)	-59.7 (-70.2 to -46.5)	7.1 (-18.7 to 39.3)	-30.1 (-43.5 to -11.4)
		Female	20333 (14331 to 28580)	940.9 (694.3 to 1255.5)	285 (211 to 380)	22.7 (18 to 28.8)	6867 (5621 to 8243)	343.4 (282.1 to 411.2)	274 (217 to 332)	16.1 (12.8 to 19.6)	-66.2 (-77.9 to -49.4)	-63.5 (-74.9 to -48.3)	-3.9 (-33.2 to 36.9)	-28.9 (-46.7 to -3.5)
		Male	22729 (16381 to 31252)	1099.1 (849.3 to 1419.3)	339 (260 to 438)	30.6 (24.8 to 37.4)	10482 (8655 to 12561)	475.2 (389.4 to 564.7)	394 (319 to 477)	20.6 (16.7 to 24.9)	-53.9 (-68.9 to -31.8)	-56.8 (-69 to -40.2)	16.3 (-17.2 to 61.5)	-32.6 (-49.2 to -10.7)
	Upper respiratory infections	Both	3441 (2054 to 5451)	84.5 (51.1 to 132.7)	0 (0 to 1)	0 (0 to 0)	4253 (2555 to 6685)	85.1 (51.4 to 135.6)	1 (0 to 1)	0 (0 to 0)	23.6 (13.3 to 35.2)	0.8 (-4.5 to 6)	125.1 (-88 to 4422.3)	98.2 (-89.1 to 2758.6)
		Female	1650 (999 to 2630)	82.6 (49.8 to 130.4)	0 (0 to 0)	0 (0 to 0)	2024 (1225 to 3202)	82.9 (49.5 to 130.9)	0 (0 to 0)	0 (0 to 0)	22.7 (11.4 to 36.8)	0.3 (-5.9 to 8.4)	94.4 (-92.2 to 5298.2)	96.6 (-91.2 to 3392.4)
		Male	1791 (1072 to 2847)	86.3 (52 to 135.9)	0 (0 to 1)	0 (0 to 0)	2229 (1341 to 3491)	87.3 (52.3 to 138.6)	0 (0 to 1)	0 (0 to 0)	24.5 (12.5 to 38.1)	1.2 (-5.8 to 8.7)	141 (-86.1 to 4521.1)	98.2 (-88.3 to 2713)
	Otitis media	Both	1401 (796 to 2291)	33.3 (18.9 to 54.9)	0 (0 to 0)	0 (0 to 0)	1383 (785 to 2295)	28.4 (16 to 47.1)	0 (0 to 0)	0 (0 to 0)	-1.2 (-11 to 10.5)	-14.7 (-20.7 to -8.7)	-66.4 (-84.2 to -25.4)	-71 (-84.5 to -42.3)
		Female	616 (359 to 1018)	30.4 (17.2 to 50.3)	0 (0 to 0)	0 (0 to 0)	616 (341 to 1034)	25.7 (14.4 to 42.7)	0 (0 to 0)	0 (0 to 0)	-0.1 (-12.2 to 14.2)	-15.5 (-23.6 to -7.1)	-10.8 (-64 to 147.7)	-51 (-79 to 37.7)
		Male	785 (439 to 1290)	36 (20.5 to 59.9)	0 (0 to 0)	0 (0 to 0)	768 (433 to 1266)	30.9 (17.5 to 52.1)	0 (0 to 0)	0 (0 to 0)	-2.1 (-13.5 to 11.7)	-14.2 (-22.2 to -6.5)	-74.6 (-88.7 to -33.8)	-76.5 (-88.7 to -42.4)

Province	Cause	Sex	1990				2019				1990-2019 percent change (%)			
			DALYs		Death		DALYs		Death		DALYs		Death	
			Number	Rate	Number	Rate	Number	Rate	Number	Rate	Number	Rate	Number	Rate
	Enteric infections	Both	23737 (17065 to 32329)	506 (377.3 to 669.2)	244 (169 to 345)	7.1 (5 to 10.1)	11867 (8749 to 15358)	265 (196.9 to 344.5)	67 (47 to 103)	1.8 (1.2 to 2.8)	-50 (-66.3 to -29.4)	-47.6 (-63 to -28.9)	-72.4 (-83 to -55.6)	-75.1 (-83 to -63.8)
		Female	11086 (7424 to 16542)	485 (330.8 to 698)	114 (70 to 176)	7.1 (3.9 to 11.8)	5671 (4174 to 7508)	258.5 (191.7 to 340.8)	32 (19 to 58)	1.7 (1 to 3.3)	-48.8 (-67.6 to -21.1)	-46.7 (-64.8 to -21.7)	-72.4 (-85.7 to -49.1)	-75.7 (-85.8 to -59.5)
		Male	12652 (8689 to 18587)	524.7 (375.4 to 743.1)	130 (84 to 196)	6.9 (4.7 to 10.4)	6196 (4532 to 8038)	271.4 (198.7 to 350.9)	36 (25 to 59)	1.8 (1.3 to 3.1)	-51 (-69.6 to -26.8)	-48.3 (-66.3 to -27.1)	-72.4 (-84.4 to -51.1)	-73.9 (-83.6 to -60.7)
	Diarrheal diseases	Both	20618 (14681 to 28976)	444 (324.6 to 603.6)	203 (134 to 295)	6.2 (4.1 to 9.1)	10685 (7759 to 14094)	238.2 (173.5 to 312.9)	50 (33 to 82)	1.4 (0.9 to 2.3)	-48.2 (-66.5 to -25)	-46.4 (-62.9 to -25.6)	-75.4 (-85.9 to -56.4)	-77.8 (-85.3 to -65.5)
		Female	9589 (6130 to 14865)	424.5 (282.8 to 633.5)	95 (54 to 155)	6.2 (3.2 to 10.9)	5133 (3703 to 6901)	233.5 (169 to 312.5)	23 (11 to 50)	1.4 (0.6 to 2.9)	-46.5 (-67.5 to -14.1)	-45 (-65 to -16.6)	-75.2 (-89.4 to -47.4)	-78.1 (-88.2 to -61)
		Male	11030 (7349 to 16869)	461.4 (321.7 to 667.5)	108 (66 to 174)	6 (4 to 9.5)	5552 (4001 to 7369)	242.8 (174.7 to 321.5)	26 (17 to 49)	1.4 (0.9 to 2.7)	-49.7 (-70.4 to -22)	-47.4 (-67 to -23.3)	-75.7 (-87.6 to -51.4)	-76.8 (-86.3 to -62.9)
	Typhoid and paratyphoid	Both	2458 (946 to 4968)	48.7 (19.1 to 99.4)	33 (13 to 67)	0.7 (0.3 to 1.4)	881 (361 to 1734)	20.1 (8.2 to 39.4)	13 (5 to 25)	0.3 (0.1 to 0.6)	-64.2 (-72.6 to -50.6)	-58.9 (-68.3 to -44.2)	-61.3 (-70.2 to -45.6)	-59.5 (-68.2 to -45.3)
		Female	1110 (420 to 2346)	44.9 (17.4 to 93.8)	15 (6 to 31)	0.6 (0.2 to 1.3)	403 (165 to 800)	18.8 (7.7 to 37.6)	6 (2 to 11)	0.3 (0.1 to 0.5)	-63.7 (-74.4 to -44.9)	-58.1 (-70.6 to -36.9)	-60.7 (-72.2 to -38.8)	-58.9 (-70.8 to -37.6)
		Male	1348 (528 to 2738)	52.4 (20.7 to 104.3)	18 (7 to 36)	0.7 (0.3 to 1.5)	478 (194 to 933)	21.2 (8.5 to 41.3)	7 (3 to 14)	0.3 (0.1 to 0.6)	-64.5 (-73.5 to -50.9)	-59.5 (-69.4 to -45.4)	-61.8 (-71.8 to -46.6)	-60 (-69.9 to -46)
	Invasive Non-typhoidal Salmonella (iNTS)	Both	567 (302 to 974)	11.5 (6.2 to 19.4)	8 (4 to 13)	0.2 (0.1 to 0.3)	280 (139 to 501)	6.3 (3.1 to 11.3)	5 (2 to 8)	0.1 (0.1 to 0.2)	-50.6 (-67 to -31.3)	-45 (-62.4 to -26.1)	-39 (-56.3 to -15.6)	-44.3 (-58.8 to -27.2)
		Female	335 (177 to 575)	13.6 (7.2 to 23.1)	5 (2 to 8)	0.2 (0.1 to 0.4)	125 (61 to 230)	5.7 (2.7 to 10.6)	2 (1 to 4)	0.1 (0 to 0.2)	-62.7 (-76.3 to -44.8)	-58.1 (-72.3 to -38.7)	-51.6 (-67.1 to -27.3)	-55 (-67.9 to -36.5)
		Male	233 (123 to 392)	9.5 (4.9 to 15.6)	3 (2 to 5)	0.2 (0.1 to 0.3)	155 (75 to 273)	6.9 (3.3 to 12.1)	3 (1 to 4)	0.1 (0.1 to 0.2)	-33.1 (-56.4 to -5.4)	-27 (-51.2 to 3)	-21.6 (-45.2 to 9.4)	-30.3 (-50.2 to -5)
	Other intestinal infectious diseases	Both	94 (26 to 181)	1.8 (0.6 to 3.3)	1 (0 to 2)	0 (0 to 0)	22 (14 to 31)	0.5 (0.3 to 0.7)	0 (0 to 0)	0 (0 to 0)	-77.1 (-89.7 to -15.9)	-72.8 (-87.7 to -15)	-78 (-91.4 to -17.5)	-76.1 (-89.7 to -34.5)
		Female	52 (6 to 124)	2 (0.3 to 4.7)	1 (0 to 1)	0 (0 to 0.1)	10 (5 to 17)	0.5 (0.2 to 0.8)	0 (0 to 0)	0 (0 to 0)	-80.4 (-93.5 to 67.4)	-76 (-92.2 to 57.9)	-81.1 (-94.8 to 104)	-77.9 (-93.9 to 11.9)
		Male	42 (8 to 95)	1.6 (0.4 to 3.4)	0 (0 to 1)	0 (0 to 0)	11 (6 to 17)	0.5 (0.3 to 0.8)	0 (0 to 0)	0 (0 to 0)	-72.9 (-90.2 to 52.8)	-68.9 (-88.4 to 35.5)	-74.1 (-92.5 to 67.9)	-73.6 (-91.5 to -5.6)
	Neglected tropical diseases and malaria	Both	15526 (1413 to 106362)	438.6 (33.8 to 3173.4)	258 (7 to 1953)	9.3 (0.2 to 71.1)	933 (591 to 2076)	19.7 (12.3 to 44.8)	7 (3 to 24)	0.1 (0.1 to 0.5)	-94 (-99.2 to -47.3)	-95.5 (-99.5 to -54)	-97.5 (-99.7 to -38.3)	-98.4 (-99.8 to -56.8)
		Female	6634 (692 to 45389)	382 (35.3 to 2762.2)	105 (3 to 808)	7.5 (0.2 to 58.4)	453 (290 to 792)	19.3 (12.3 to 34.4)	3 (1 to 8)	0.1 (0.1 to 0.3)	-93.2 (-99.1 to -43.7)	-94.9 (-99.4 to -53.7)	-97.5 (-99.7 to -29)	-98.3 (-99.8 to -49.6)
		Male	8892 (658 to 64414)	494.6 (30.3 to 3714.3)	153 (4 to 1193)	11.3 (0.3 to 85.7)	480 (274 to 1266)	20 (11.3 to 53.5)	4 (2 to 16)	0.2 (0.1 to 0.7)	-94.6 (-99.4 to -43.1)	-96 (-99.5 to -46.8)	-97.5 (-99.8 to -38.6)	-98.5 (-99.9 to -58.9)
	Malaria	Both	11795 (137 to 103285)	357.1 (3.2 to 3121.1)	220 (0 to 1940)	8.3 (0 to 70.4)	58 (41 to 79)	1.1 (0.8 to 1.5)	0 (0 to 0)	0 (0 to 0)	-99.5 (-99.9 to -58.7)	-99.7 (-100 to -67.2)	-100 (-100 to 22.4)	-100 (-100 to -22)
		Female	5133 (80 to 44157)	313.2 (4.1 to 2732.9)	92 (0 to 805)	6.8 (0 to 58.1)	29 (21 to 40)	1.2 (0.8 to 1.5)	0 (0 to 0)	0 (0 to 0)	-99.4 (-99.9 to -64.7)	-99.6 (-100 to -73)	-100 (-100 to 28.2)	-100 (-100 to -16)
		Male	6663 (56 to 60366)	401.2 (2.4 to 3557.6)	128 (0 to 1141)	10 (0 to 85)	29 (20 to 39)	1.1 (0.8 to 1.5)	0 (0 to 0)	0 (0 to 0)	-99.6 (-100 to -47.5)	-99.7 (-100 to -55.7)	-100 (-100 to 16.4)	-100 (-100 to -27.2)
	Leishmaniasis	Both	2203 (17 to 22478)	46 (0.4 to 445.4)	30 (0 to 301)	0.7 (0 to 6.6)	150 (9 to 1328)	3.2 (0.2 to 29)	2 (0 to 20)	0 (0 to 0.4)	-93.2 (-94.7 to 0.6)	-93.1 (-94.7 to -8.4)	-92.7 (-93.8 to -87.9)	-93.7 (-95.2 to -92.2)
		Female	737 (8 to 7549)	31.3 (0.4 to 299.9)	10 (0 to 99)	0.5 (0 to 4.3)	52 (5 to 408)	2.3 (0.2 to 18.2)	1 (0 to 6)	0 (0 to 0.3)	-92.9 (-95 to 49.1)	-92.7 (-94.9 to 19.1)	-92.9 (-94.1 to -86.9)	-93.9 (-95.5 to -91.6)

Province	Cause	Sex	1990				2019				1990-2019 percent change (%)			
			DALYs		Death		DALYs		Death		DALYs		Death	
			Number	Rate	Number	Rate	Number	Rate	Number	Rate	Number	Rate	Number	Rate
		Male	1466 (9 to 15175)	60.2 (0.4 to 597.9)	20 (0 to 205)	1 (0 to 8.9)	97 (3 to 924)	4 (0.1 to 39.4)	1 (0 to 14)	0.1 (0 to 0.6)	-93.3 (-94.4 to -28.8)	-93.3 (-94.9 to -29.7)	-92.6 (-93.7 to -87.5)	-93.7 (-95.4 to -91.7)
	Schistosomiasis	Both	0 (0 to 0)	0 (0 to 0)	0 (0 to 0)	0 (0 to 0)	0 (0 to 0)	0 (0 to 0)	0 (0 to 0)	0 (0 to 0)	0 (0 to 0)	0 (0 to 0)	0 (0 to 0)	0 (0 to 0)
		Female	0 (0 to 0)	0 (0 to 0)	0 (0 to 0)	0 (0 to 0)	0 (0 to 0)	0 (0 to 0)	0 (0 to 0)	0 (0 to 0)	0 (0 to 0)	0 (0 to 0)	0 (0 to 0)	0 (0 to 0)
		Male	0 (0 to 0)	0 (0 to 0)	0 (0 to 0)	0 (0 to 0)	0 (0 to 0)	0 (0 to 0)	0 (0 to 0)	0 (0 to 0)	0 (0 to 0)	0 (0 to 0)	0 (0 to 0)	0 (0 to 0)
	Cystic echinococcosis	Both	229 (135 to 336)	6.6 (4.4 to 9)	3 (2 to 5)	0.1 (0.1 to 0.2)	146 (101 to 206)	2.8 (1.9 to 3.8)	1 (1 to 2)	0 (0 to 0)	-36.1 (-61.7 to 12.8)	-58.1 (-72.5 to -34.5)	-65.4 (-83 to -27.4)	-79 (-89.4 to -58.6)
		Female	95 (47 to 159)	5.7 (3.3 to 8.6)	1 (0 to 2)	0.1 (0 to 0.2)	77 (50 to 113)	2.9 (1.9 to 4.2)	0 (0 to 1)	0 (0 to 0)	-19.4 (-58.3 to 66.1)	-48.9 (-69.1 to -13.8)	-61.4 (-87.5 to 28.1)	-75.5 (-91.9 to -30.5)
		Male	134 (63 to 215)	7.5 (4.2 to 11.1)	2 (1 to 3)	0.1 (0.1 to 0.2)	70 (45 to 101)	2.6 (1.7 to 3.7)	1 (0 to 1)	0 (0 to 0)	-47.9 (-73.4 to 19.8)	-64.9 (-80.3 to -33.5)	-67.8 (-88.5 to -11.8)	-81.1 (-93 to -51.3)
	Trachoma	Both	23 (13 to 38)	1.6 (0.9 to 2.7)	--	--	13 (7 to 22)	0.3 (0.2 to 0.5)	--	--	-42.9 (-58.2 to -23.8)	-80.4 (-85.5 to -74.6)	--	--
		Female	17 (10 to 29)	2.5 (1.4 to 4.2)	--	--	9 (5 to 16)	0.4 (0.2 to 0.8)	--	--	-48.6 (-64.8 to -28.2)	-82.5 (-87.9 to -75.9)	--	--
		Male	5 (2 to 10)	0.7 (0.4 to 1.3)	--	--	4 (2 to 7)	0.2 (0.1 to 0.4)	--	--	-24.5 (-50.5 to 17.1)	-72.6 (-81.9 to -59)	--	--
	Rabies	Both	25 (4 to 57)	0.8 (0.1 to 1.6)	0 (0 to 1)	0 (0 to 0)	12 (2 to 19)	0.2 (0 to 0.4)	0 (0 to 0)	0 (0 to 0)	-51.6 (-80.4 to 4.9)	-67.3 (-85.9 to -31.5)	-43.3 (-75.1 to 20.1)	-68 (-85.1 to -36.5)
		Female	5 (0 to 10)	0.4 (0 to 0.8)	0 (0 to 0)	0 (0 to 0)	3 (0 to 5)	0.1 (0 to 0.2)	0 (0 to 0)	0 (0 to 0)	-34.3 (-69.1 to 47.1)	-68.1 (-84.4 to -25.7)	-36.2 (-67.5 to 39.5)	-72.5 (-86.1 to -40.1)
		Male	20 (3 to 51)	1.1 (0.1 to 2.6)	0 (0 to 1)	0 (0 to 0.1)	9 (2 to 15)	0.4 (0.1 to 0.6)	0 (0 to 0)	0 (0 to 0)	-55.7 (-84.5 to 19.3)	-67.1 (-88.2 to -16.6)	-45.7 (-80.5 to 38)	-65.7 (-87 to -18.1)
	Intestinal nematode infections	Both	255 (135 to 428)	5.6 (3.2 to 8.8)	2 (0 to 3)	0 (0 to 0.1)	50 (28 to 83)	1 (0.6 to 1.7)	0 (0 to 0)	0 (0 to 0)	-80.3 (-90 to -62.6)	-81.6 (-90 to -68.2)	-89.6 (-96 to -58.9)	-87 (-94.8 to -62.9)
		Female	142 (66 to 256)	6.8 (3.6 to 11.4)	1 (0 to 2)	0 (0 to 0.1)	31 (17 to 56)	1.3 (0.7 to 2.2)	0 (0 to 0)	0 (0 to 0)	-78 (-90.1 to -54.1)	-81.2 (-90.7 to -65.3)	-89.7 (-97.5 to 19.1)	-86.3 (-96.6 to -28.4)
		Male	113 (39 to 219)	4.4 (1.8 to 7.8)	1 (0 to 2)	0 (0 to 0.1)	19 (10 to 32)	0.8 (0.4 to 1.4)	0 (0 to 0)	0 (0 to 0)	-83.3 (-93.1 to -51.5)	-82 (-92.1 to -56.5)	-89.5 (-97.3 to -18.5)	-87.5 (-96.3 to -47.7)
	Food-borne trematodiases	Both	13 (3 to 26)	0.5 (0.1 to 1)	--	--	54 (12 to 112)	1 (0.2 to 2)	--	--	327.5 (217.7 to 459)	108 (60.6 to 165.1)	--	--
		Female	5 (1 to 11)	0.4 (0.1 to 0.8)	--	--	21 (5 to 45)	0.8 (0.2 to 1.7)	--	--	317.8 (210 to 446.2)	101 (50.4 to 165.7)	--	--
		Male	8 (2 to 16)	0.6 (0.1 to 1.1)	--	--	33 (7 to 68)	1.2 (0.3 to 2.4)	--	--	334 (180.5 to 512.6)	113.5 (46.9 to 197.4)	--	--
	Leprosy	Both	1 (0 to 1)	0 (0 to 0.1)	--	--	2 (1 to 2)	0 (0 to 0)	--	--	101.2 (84.4 to 121.1)	-19.1 (-25.1 to -11.9)	--	--
		Female	0 (0 to 0)	0 (0 to 0)	--	--	1 (0 to 1)	0 (0 to 0)	--	--	89 (64.9 to 116.5)	-25.8 (-34.5 to -15.9)	--	--
		Male	1 (0 to 1)	0 (0 to 0.1)	--	--	1 (1 to 2)	0 (0 to 0.1)	--	--	107.5 (86.2 to 133.2)	-15.3 (-23.4 to -5.5)	--	--
	Other neglected tropical diseases	Both	982 (596 to 1498)	20.4 (13 to 30.2)	3 (2 to 6)	0.1 (0.1 to 0.2)	447 (294 to 683)	10 (6.4 to 15.6)	3 (2 to 4)	0.1 (0 to 0.1)	-54.5 (-69.1 to -31)	-50.9 (-65.9 to -27.8)	-6.1 (-46.5 to 68.6)	-30.7 (-54.1 to 12)

Province	Cause	Sex	1990				2019				1990-2019 percent change (%)			
			DALYs		Death		DALYs		Death		DALYs		Death	
			Number	Rate	Number	Rate	Number	Rate	Number	Rate	Number	Rate	Number	Rate
		Female	499 (280 to 812)	21.8 (13.1 to 33.5)	1 (1 to 3)	0.1 (0 to 0.2)	229 (139 to 353)	10.3 (6.3 to 16.4)	1 (1 to 2)	0.1 (0 to 0.1)	-54.1 (-72 to -22.5)	-52.7 (-69.9 to -25.8)	-6.9 (-46.6 to 79.1)	-30.8 (-57.5 to 27)
		Male	484 (252 to 830)	18.9 (10.8 to 31)	2 (1 to 4)	0.1 (0 to 0.2)	218 (124 to 378)	9.7 (5.4 to 16.8)	1 (1 to 3)	0.1 (0 to 0.1)	-54.9 (-76.1 to -11.4)	-48.9 (-71.6 to -5.1)	-5.4 (-50.7 to 100.3)	-30.3 (-56.1 to 33.6)
	Other infectious diseases	Both	32237 (18469 to 56411)	645.9 (396.4 to 1074.2)	398 (235 to 684)	9.8 (6.7 to 14.9)	5851 (5053 to 6758)	126.2 (109 to 146.2)	114 (96 to 133)	2.7 (2.2 to 3.1)	-81.8 (-89.5 to -68.6)	-80.5 (-88 to -68.4)	-71.4 (-83.4 to -50.9)	-72.9 (-82.3 to -58.5)
		Female	16643 (9435 to 29205)	664.9 (400.5 to 1111.3)	200 (116 to 343)	9.6 (6.3 to 14.9)	2472 (2074 to 2937)	111.1 (93.5 to 133.3)	43 (35 to 51)	2.1 (1.7 to 2.5)	-85.1 (-91.8 to -74.3)	-83.3 (-90.3 to -72.7)	-78.7 (-87.8 to -62.5)	-78 (-86.2 to -65.1)
		Male	15594 (8975 to 27336)	626.5 (387.4 to 1031)	198 (117 to 336)	10 (6.7 to 14.8)	3379 (2840 to 3969)	141.1 (118.1 to 165.9)	71 (55 to 87)	3.2 (2.5 to 3.9)	-78.3 (-87.5 to -62.3)	-77.5 (-86.5 to -63.5)	-64 (-79.7 to -38.7)	-67.9 (-80 to -50.1)
	Meningitis	Both	7321 (5788 to 9534)	156.2 (126.2 to 198.5)	93 (74 to 120)	2.6 (2.1 to 3.1)	2003 (1688 to 2349)	43.3 (36.3 to 51)	39 (32 to 46)	0.9 (0.8 to 1.1)	-72.6 (-79.9 to -62.8)	-72.2 (-79.3 to -62.3)	-58.5 (-69.4 to -42.7)	-64.6 (-73.1 to -51.7)
		Female	3720 (2717 to 5231)	159.4 (120.9 to 216.9)	46 (34 to 63)	2.5 (1.9 to 3.4)	914 (745 to 1102)	41 (33.1 to 49.6)	16 (13 to 19)	0.8 (0.6 to 1)	-75.4 (-83.3 to -64.4)	-74.3 (-82.1 to -63.4)	-65.3 (-76.9 to -49.6)	-68.4 (-78.4 to -55.9)
		Male	3601 (2638 to 4833)	152.9 (115.5 to 200.1)	47 (35 to 62)	2.6 (2 to 3.3)	1089 (868 to 1356)	45.7 (36.6 to 56.7)	23 (17 to 29)	1 (0.8 to 1.3)	-69.8 (-79.4 to -54.9)	-70.1 (-79 to -55.8)	-51.9 (-67.4 to -26)	-60.8 (-72.6 to -39.4)
	Encephalitis	Both	699 (528 to 975)	16.2 (12.4 to 22.3)	9 (6 to 13)	0.3 (0.2 to 0.4)	678 (569 to 807)	14.7 (12.3 to 17.6)	11 (9 to 13)	0.3 (0.2 to 0.3)	-3 (-31.4 to 35.9)	-8.9 (-35.4 to 24.2)	30.3 (-11.7 to 87.3)	-3 (-36.1 to 40)
		Female	341 (238 to 501)	15.9 (11.5 to 22.8)	4 (3 to 6)	0.2 (0.2 to 0.4)	303 (238 to 379)	13.5 (10.4 to 17.1)	5 (3 to 6)	0.2 (0.2 to 0.3)	-10.9 (-46.4 to 33)	-15.1 (-44.4 to 24.8)	13.7 (-29.8 to 77.9)	-11.2 (-44.2 to 35)
		Male	358 (251 to 529)	16.4 (11.8 to 23.9)	5 (3 to 7)	0.3 (0.2 to 0.4)	374 (300 to 460)	15.9 (12.5 to 19.6)	7 (5 to 8)	0.3 (0.2 to 0.4)	4.6 (-31.1 to 61.7)	-3.1 (-36.1 to 45.3)	44.5 (-7.1 to 128.7)	3.9 (-36.4 to 64.4)
	Diphtheria	Both	29 (13 to 54)	0.5 (0.3 to 1)	0 (0 to 1)	0 (0 to 0)	4 (2 to 6)	0.1 (0.1 to 0.1)	0 (0 to 0)	0 (0 to 0)	-86.5 (-93.9 to -67.1)	-83.6 (-92.8 to -61)	-83.4 (-92.2 to -61.1)	-81.7 (-91.2 to -61.4)
		Female	13 (4 to 31)	0.5 (0.1 to 1.1)	0 (0 to 0)	0 (0 to 0)	1 (1 to 3)	0.1 (0 to 0.1)	0 (0 to 0)	0 (0 to 0)	-89.1 (-96.7 to -55.8)	-86.1 (-95.9 to -45.1)	-87 (-95.7 to -51.3)	-84.6 (-94.9 to -45.9)
		Male	16 (5 to 32)	0.6 (0.2 to 1.1)	0 (0 to 0)	0 (0 to 0)	2 (1 to 4)	0.1 (0 to 0.2)	0 (0 to 0)	0 (0 to 0)	-84.3 (-94.5 to -49.9)	-81.5 (-93.5 to -46)	-80.6 (-92.5 to -43.1)	-79.5 (-92 to -46.4)
	Whooping cough	Both	4370 (255 to 17922)	77.7 (4.5 to 320.6)	49 (2 to 206)	0.9 (0 to 3.7)	95 (7 to 402)	2.4 (0.2 to 10.4)	1 (0 to 5)	0 (0 to 0.1)	-97.8 (-99.9 to -57)	-96.9 (-99.8 to -37.9)	-97.9 (-99.9 to -42.2)	-96.9 (-99.9 to -16.7)
		Female	2544 (145 to 10556)	92.4 (5.2 to 383.3)	29 (1 to 121)	1 (0 to 4.4)	53 (4 to 228)	2.8 (0.2 to 12.1)	1 (0 to 3)	0 (0 to 0.1)	-97.9 (-99.9 to -58.7)	-96.9 (-99.8 to -40.2)	-97.9 (-99.9 to -40.1)	-97 (-99.9 to -12.4)
		Male	1826 (111 to 7490)	63.7 (3.9 to 262)	21 (1 to 86)	0.7 (0 to 3)	42 (3 to 176)	2.1 (0.1 to 8.9)	0 (0 to 2)	0 (0 to 0.1)	-97.7 (-99.9 to -56.1)	-96.7 (-99.8 to -36.8)	-97.8 (-99.9 to -38.5)	-96.8 (-99.9 to -11)
	Tetanus	Both	311 (117 to 906)	6.6 (2.7 to 18.5)	4 (2 to 11)	0.1 (0 to 0.3)	40 (32 to 62)	0.9 (0.7 to 1.3)	1 (1 to 1)	0 (0 to 0)	-87.1 (-95.3 to -65.1)	-86.8 (-95 to -66.9)	-81.8 (-92.9 to -56.8)	-85.2 (-93.6 to -65)
		Female	138 (39 to 499)	5.8 (1.8 to 20.6)	2 (1 to 6)	0.1 (0 to 0.3)	18 (14 to 22)	0.8 (0.6 to 1)	0 (0 to 0)	0 (0 to 0)	-86.9 (-96.1 to -54)	-86.2 (-95.9 to -56.9)	-81.1 (-94.2 to -42.3)	-84 (-94.3 to -59.5)
		Male	173 (59 to 531)	7.3 (2.6 to 20.8)	2 (1 to 6)	0.1 (0 to 0.3)	22 (17 to 36)	0.9 (0.7 to 1.6)	0 (0 to 1)	0 (0 to 0)	-87.3 (-95.8 to -61.8)	-87.2 (-95.5 to -61.3)	-82.3 (-93.6 to -46.2)	-86 (-94.6 to -56.9)
	Measles	Both	15186 (5066 to 33618)	262.7 (87.6 to 582.5)	176 (59 to 392)	3.1 (1 to 6.8)	197 (58 to 472)	5 (1.5 to 11.9)	2 (1 to 5)	0.1 (0 to 0.1)	-98.7 (-99.4 to -97.6)	-98.1 (-99.1 to -96.5)	-98.7 (-99.4 to -97.6)	-98.2 (-99.2 to -96.6)
		Female	7902 (2632 to 17825)	279.7 (93.1 to 630.1)	92 (30 to 208)	3.3 (1.1 to 7.3)	98 (29 to 232)	5.1 (1.5 to 12.1)	1 (0 to 3)	0.1 (0 to 0.1)	-98.8 (-99.4 to -97.7)	-98.2 (-99.2 to -96.6)	-98.8 (-99.5 to -97.7)	-98.2 (-99.2 to -96.7)
		Male	7283 (2451 to 16246)	246.5 (83.2 to 548.1)	85 (29 to 188)	2.9 (1 to 6.4)	99 (29 to 239)	4.9 (1.4 to 11.8)	1 (0 to 3)	0.1 (0 to 0.1)	-98.6 (-99.4 to -97.5)	-98 (-99.1 to -96.3)	-98.7 (-99.4 to -97.5)	-98.1 (-99.1 to -96.4)

Province	Cause	Sex	1990				2019				1990-2019 percent change (%)			
			DALYs		Death		DALYs		Death		DALYs		Death	
			Number	Rate	Number	Rate	Number	Rate	Number	Rate	Number	Rate	Number	Rate
	Varicella and herpes zoster	Both	368 (227 to 578)	9.4 (6.6 to 13.1)	4 (3 to 7)	0.2 (0.1 to 0.3)	204 (139 to 283)	4.4 (3 to 6.2)	2 (2 to 3)	0.1 (0 to 0.1)	-44.7 (-67.6 to -5.5)	-53.3 (-69.8 to -30.4)	-50.8 (-72.7 to -11.8)	-70.2 (-82.7 to -48.9)
		Female	187 (91 to 332)	9.7 (5.9 to 15.3)	2 (1 to 4)	0.2 (0.1 to 0.3)	101 (67 to 150)	4.4 (2.9 to 6.5)	1 (1 to 2)	0.1 (0 to 0.1)	-46 (-72.5 to 9.8)	-54.2 (-73.7 to -25.4)	-53.9 (-78.5 to 5.4)	-70.5 (-85.7 to -40.4)
		Male	182 (98 to 326)	9.2 (5.8 to 14.1)	2 (1 to 4)	0.2 (0.1 to 0.3)	103 (68 to 148)	4.4 (2.9 to 6.3)	1 (1 to 2)	0.1 (0 to 0.1)	-43.4 (-71.8 to 9.4)	-52.5 (-73.6 to -22.6)	-47.5 (-75 to 12.8)	-69.7 (-85.2 to -40.5)
	Acute hepatitis	Both	2555 (1605 to 3739)	82.1 (52 to 120.6)	48 (29 to 73)	2.2 (1.4 to 3.4)	1074 (885 to 1311)	21.2 (17.5 to 25.6)	26 (20 to 32)	0.6 (0.4 to 0.7)	-58 (-73 to -28.2)	-74.2 (-83.7 to -56.4)	-47.1 (-68.3 to -6.6)	-74.9 (-85.2 to -55.2)
		Female	1155 (580 to 1876)	68.6 (33.7 to 104.1)	19 (9 to 30)	1.7 (0.8 to 2.7)	311 (244 to 386)	13 (10.1 to 16.1)	6 (4 to 8)	0.3 (0.2 to 0.4)	-73 (-84.3 to -44.4)	-81.1 (-88.3 to -59.7)	-68 (-81.4 to -24.3)	-83.5 (-90.6 to -60.2)
		Male	1400 (843 to 2440)	94 (57.4 to 158.6)	29 (17 to 50)	2.6 (1.5 to 4.5)	762 (596 to 979)	29.3 (22.9 to 37.5)	19 (14 to 26)	0.8 (0.6 to 1.1)	-45.6 (-71.4 to -5)	-68.8 (-83.2 to -44.8)	-33.1 (-65.7 to 26.1)	-68.7 (-84 to -41.5)
	Other unspecified infectious diseases	Both	1397 (864 to 2884)	34.5 (22.1 to 66.8)	13 (7 to 33)	0.6 (0.3 to 1.2)	1558 (1065 to 1974)	34.2 (23.5 to 43.3)	32 (19 to 42)	0.8 (0.4 to 1)	11.5 (-55.9 to 94.1)	-1 (-57.9 to 68.6)	145.7 (-31.7 to 470)	42.6 (-51.2 to 224.1)
		Female	642 (379 to 1270)	33 (20.5 to 59.7)	6 (3 to 13)	0.5 (0.2 to 1.2)	672 (442 to 896)	30.4 (19.9 to 40.8)	13 (7 to 17)	0.6 (0.4 to 0.9)	4.6 (-58.2 to 84.6)	-7.8 (-58.7 to 60.1)	130.3 (-34 to 450.2)	28.9 (-56.1 to 232.4)
		Male	755 (439 to 1720)	35.9 (21.9 to 75.6)	8 (4 to 21)	0.6 (0.3 to 1.3)	886 (558 to 1165)	37.9 (23.7 to 50.5)	20 (10 to 27)	0.9 (0.4 to 1.3)	17.3 (-57.3 to 134.4)	5.4 (-59.1 to 95.1)	156.8 (-34.6 to 517.6)	54.7 (-52.6 to 241.8)
	Maternal and neonatal disorders	Both	258507 (209638 to 308427)	5011.4 (4077.9 to 5967.2)	2879 (2333 to 3446)	55.8 (45.4 to 66.8)	50237 (41524 to 60697)	1314.6 (1073.1 to 1596.6)	443 (351 to 554)	12.3 (9.7 to 15.4)	-80.6 (-85 to -74.8)	-73.8 (-80 to -65.8)	-84.6 (-88.7 to -79.3)	-77.9 (-83.7 to -70.3)
		Female	113429 (91968 to 138102)	4550 (3708.6 to 5511.7)	1265 (1028 to 1540)	50.9 (41.2 to 61.9)	22589 (18621 to 27123)	1189.2 (968.8 to 1436.5)	195 (154 to 241)	10.9 (8.6 to 13.6)	-80.1 (-84.7 to -74.1)	-73.9 (-80 to -65.8)	-84.6 (-88.6 to -79.4)	-78.5 (-84 to -71.3)
		Male	145078 (115318 to 176616)	5448.5 (4335.7 to 6627)	1613 (1276 to 1970)	60.5 (47.9 to 73.8)	27648 (22466 to 33898)	1433 (1159.5 to 1767.4)	248 (193 to 314)	13.6 (10.6 to 17.3)	-80.9 (-85.8 to -74.6)	-73.7 (-80.6 to -64.7)	-84.6 (-89 to -78.7)	-77.4 (-83.9 to -68.8)
	Maternal disorders	Female	3611 (2048 to 5148)	219.6 (127.8 to 310.6)	51 (25 to 76)	3.2 (1.7 to 4.9)	1103 (847 to 1439)	36.7 (28.4 to 47.4)	14 (10 to 19)	0.4 (0.3 to 0.6)	-69.4 (-80.1 to -44.1)	-83.3 (-89 to -70.3)	-73.1 (-84.3 to -43.7)	-86.4 (-92.1 to -72.9)
	Neonatal disorders	Both	254896 (206453 to 305063)	4903.3 (3972.6 to 5865.3)	2828 (2286 to 3397)	54.2 (43.9 to 65.2)	49133 (40519 to 59502)	1296.7 (1054.3 to 1580.1)	429 (336 to 538)	12.1 (9.5 to 15.2)	-80.7 (-85.2 to -74.9)	-73.6 (-79.8 to -65.3)	-84.8 (-88.9 to -79.4)	-77.7 (-83.6 to -69.7)
		Female	109819 (88494 to 133970)	4330.4 (3488 to 5274.7)	1215 (979 to 1491)	47.7 (38.4 to 58.5)	21485 (17607 to 25978)	1152.5 (935.7 to 1400.8)	181 (141 to 228)	10.5 (8.2 to 13.2)	-80.4 (-85.2 to -74.4)	-73.4 (-80 to -65)	-85.1 (-89.1 to -79.6)	-78 (-83.9 to -70)
		Male	145078 (115318 to 176616)	5448.5 (4335.7 to 6627)	1613 (1276 to 1970)	60.5 (47.9 to 73.8)	27648 (22466 to 33898)	1433 (1159.5 to 1767.4)	248 (193 to 314)	13.6 (10.6 to 17.3)	-80.9 (-85.8 to -74.6)	-73.7 (-80.6 to -64.7)	-84.6 (-89 to -78.7)	-77.4 (-83.9 to -68.8)
	Nutritional deficiencies	Both	13953 (9161 to 20720)	326.2 (223.2 to 468.7)	22 (15 to 31)	0.7 (0.5 to 0.9)	7024 (4445 to 10665)	148.3 (94.6 to 221)	5 (4 to 6)	0.2 (0.1 to 0.2)	-49.7 (-63 to -31.2)	-54.6 (-65.2 to -40.4)	-76.7 (-84.3 to -65.5)	-77 (-84.2 to -67.1)
		Female	7359 (4685 to 11100)	369.9 (237.5 to 536.5)	12 (8 to 20)	0.7 (0.5 to 1.2)	3830 (2362 to 5990)	162.8 (100.2 to 248.1)	3 (2 to 3)	0.2 (0.1 to 0.2)	-47.9 (-64.2 to -22.5)	-56 (-68.1 to -38.8)	-79.7 (-88.6 to -66.6)	-78 (-87.8 to -64.4)
		Male	6594 (3855 to 10768)	282.8 (177.6 to 441.9)	10 (6 to 15)	0.6 (0.4 to 0.8)	3193 (1846 to 5171)	134.1 (75.6 to 216.9)	3 (2 to 3)	0.1 (0.1 to 0.2)	-51.6 (-70.4 to -21.2)	-52.6 (-69.7 to -26)	-72.7 (-83.3 to -51.8)	-75.7 (-84.3 to -59.4)
	Protein-energy malnutrition	Both	2907 (2182 to 3837)	60.3 (45.8 to 79.5)	21 (14 to 30)	0.6 (0.4 to 0.8)	1436 (913 to 2133)	30.8 (19.7 to 45.2)	3 (2 to 4)	0.1 (0.1 to 0.1)	-50.6 (-66.8 to -31.6)	-49 (-64.5 to -31.2)	-85.2 (-90.3 to -77.4)	-84.6 (-89.6 to -77.7)
		Female	1551 (1091 to 2251)	64.6 (46.4 to 91.9)	12 (7 to 19)	0.6 (0.4 to 1.1)	676 (419 to 1011)	29.8 (19 to 44)	2 (1 to 2)	0.1 (0.1 to 0.1)	-56.4 (-73.3 to -33.3)	-53.9 (-71.2 to -32.5)	-86.4 (-92.3 to -77.1)	-84.4 (-91.3 to -74.4)
		Male	1356 (964 to 1832)	56.2 (40.3 to 75.3)	9 (5 to 14)	0.5 (0.3 to 0.7)	760 (461 to 1157)	31.8 (19.7 to 47.6)	1 (1 to 2)	0.1 (0.1 to 0.1)	-44 (-62.5 to -17.9)	-43.5 (-61.1 to -19.8)	-83.6 (-90.4 to -70.7)	-84.5 (-90.3 to -74.2)

Province	Cause	Sex	1990				2019				1990-2019 percent change (%)			
			DALYs		Death		DALYs		Death		DALYs		Death	
			Number	Rate	Number	Rate	Number	Rate	Number	Rate	Number	Rate	Number	Rate
	Iodine deficiency	Both	521 (322 to 782)	15.8 (10.1 to 23)	--	--	332 (189 to 548)	6.3 (3.6 to 10.4)	--	--	-36.3 (-54.5 to -15.9)	-60.3 (-70.7 to -49.8)	--	--
		Female	317 (187 to 483)	19.6 (12 to 29.4)	--	--	206 (115 to 348)	7.9 (4.4 to 13.3)	--	--	-35 (-56.7 to -9.2)	-59.6 (-71.8 to -46.4)	--	--
		Male	205 (125 to 314)	12.2 (7.7 to 18.2)	--	--	126 (72 to 208)	4.7 (2.7 to 7.7)	--	--	-38.2 (-55.6 to -17.1)	-61.4 (-71.1 to -50.1)	--	--
	Vitamin A deficiency	Both	566 (282 to 1021)	9.5 (4.8 to 17)	--	--	24 (10 to 49)	0.6 (0.2 to 1.2)	--	--	-95.8 (-98.2 to -90.5)	-93.7 (-97.3 to -85.9)	--	--
		Female	208 (96 to 394)	7.1 (3.3 to 13.4)	--	--	10 (3 to 22)	0.5 (0.2 to 1.2)	--	--	-95 (-98.3 to -87.3)	-92.4 (-97.4 to -81.3)	--	--
		Male	358 (142 to 744)	11.7 (4.7 to 24.2)	--	--	13 (3 to 35)	0.6 (0.2 to 1.7)	--	--	-96.3 (-99 to -87.9)	-94.5 (-98.5 to -82.3)	--	--
	Dietary iron deficiency	Both	9563 (5373 to 15632)	229.6 (140.5 to 359.3)	--	--	4757 (2674 to 7840)	101.2 (57.7 to 168.1)	--	--	-50.3 (-67.5 to -22.4)	-55.9 (-70 to -35.2)	--	--
		Female	5104 (2805 to 8377)	268.6 (155.1 to 419.7)	--	--	2719 (1476 to 4654)	115.7 (63 to 193)	--	--	-46.7 (-68.2 to -6.3)	-56.9 (-72.5 to -31.4)	--	--
		Male	4459 (2102 to 8140)	190.8 (100.8 to 336.8)	--	--	2038 (959 to 3801)	87 (40.8 to 163.7)	--	--	-54.3 (-76.8 to -5.8)	-54.4 (-76 to -10.8)	--	--
	Other nutritional deficiencies	Both	396 (265 to 585)	11 (7.4 to 16.2)	2 (1 to 2)	0.1 (0 to 0.1)	475 (300 to 707)	9.4 (6 to 13.8)	2 (2 to 3)	0.1 (0 to 0.1)	20 (-8.1 to 53.4)	-14.5 (-33 to 7.8)	35.2 (-15.8 to 145.1)	-14.9 (-44.9 to 44.6)
		Female	179 (112 to 268)	10 (6.3 to 14.7)	1 (0 to 1)	0.1 (0 to 0.1)	219 (131 to 337)	8.8 (5.4 to 13.4)	1 (1 to 1)	0.1 (0 to 0.1)	22.1 (-16.3 to 74.9)	-11.5 (-37.8 to 25.1)	32.9 (-33.3 to 156.3)	-11 (-56.3 to 76.3)
		Male	216 (139 to 337)	11.9 (7.7 to 18.5)	1 (0 to 1)	0.1 (0 to 0.1)	256 (154 to 399)	9.9 (6 to 15.3)	1 (1 to 2)	0.1 (0 to 0.1)	18.3 (-17.3 to 68.6)	-16.9 (-40.9 to 18.2)	36.9 (-28.1 to 216.2)	-19.5 (-54.4 to 67.1)
	**Non-communicable diseases**	Both	637884 (540214 to 744660)	26250 (22594.6 to 30047)	10313 (8827 to 11907)	770.1 (668.5 to 873.1)	978366 (823577 to 1141165)	20499.3 (17457.1 to 23741)	20671 (18165 to 23264)	552.6 (489.5 to 616.1)	53.4 (33.5 to 76.8)	-21.9 (-31.2 to -10.3)	100.4 (64.3 to 148.6)	-28.2 (-40.1 to -13.3)
		Female	300056 (245786 to 355382)	24892 (20978.4 to 29527)	4328 (3556 to 5192)	680.8 (571.4 to 795.8)	476061 (393956 to 570437)	20102 (16806.4 to 23876.8)	8946 (7667 to 10378)	507.3 (439.6 to 581.9)	58.7 (38 to 84.3)	-19.2 (-29.4 to -7.2)	106.7 (63.8 to 169.5)	-25.5 (-39.5 to -6.2)
		Male	337829 (279080 to 400942)	27464.1 (22852.3 to 32222.1)	5985 (4751 to 7342)	855.2 (689.3 to 1019.4)	502305 (420734 to 586862)	20935.9 (17675.8 to 24313.9)	11726 (9778 to 13910)	599.6 (505.1 to 705.1)	48.7 (22.9 to 79.2)	-23.8 (-37.1 to -6.9)	95.9 (49 to 165.2)	-29.9 (-45.9 to -7.2)
	Neoplasms	Both	49948 (39170 to 61553)	2238.8 (1803.3 to 2721.8)	1393 (1117 to 1710)	86.2 (69.5 to 103.8)	105752 (90009 to 123075)	2207.6 (1886.2 to 2561.8)	3716 (3191 to 4302)	87.9 (75.9 to 101.4)	111.7 (63.1 to 176.8)	-1.4 (-23.5 to 28.8)	166.8 (105.4 to 251.8)	2 (-20.2 to 33.6)
		Female	20755 (15116 to 27232)	1817.6 (1359 to 2374.1)	535 (398 to 697)	64.7 (48.7 to 83.4)	45630 (37300 to 55319)	1858.3 (1528.4 to 2234.8)	1479 (1218 to 1788)	68.1 (56.3 to 81.5)	119.9 (57.9 to 212.4)	2.2 (-26.5 to 43.6)	176.2 (99.4 to 291.4)	5.2 (-23.3 to 47.9)
		Male	29193 (20938 to 37771)	2657.7 (1952.4 to 3381.5)	858 (630 to 1097)	109.8 (83.6 to 136.7)	60122 (48178 to 73626)	2557.5 (2070.5 to 3114.4)	2237 (1781 to 2722)	107.2 (86.3 to 130.2)	105.9 (47 to 200.8)	-3.8 (-31 to 41.8)	160.8 (84.6 to 283.9)	-2.3 (-28.4 to 43.1)
	Lip and oral cavity cancer	Both	287 (209 to 382)	15.5 (11.2 to 20.5)	10 (7 to 13)	0.7 (0.5 to 0.8)	715 (591 to 852)	15 (12.4 to 17.8)	27 (23 to 33)	0.7 (0.5 to 0.8)	148.8 (71.2 to 272.5)	-3.3 (-32.7 to 44.8)	184.1 (94.3 to 328.6)	1.6 (-29.4 to 52.7)
		Female	87 (60 to 118)	9.5 (6.8 to 12.8)	3 (2 to 4)	0.4 (0.3 to 0.5)	273 (216 to 334)	11.5 (9.2 to 13.9)	10 (8 to 12)	0.5 (0.4 to 0.6)	214 (118 to 382.7)	21.3 (-14.4 to 84.7)	269.1 (156.9 to 475.8)	29 (-9.5 to 92.5)

Province	Cause	Sex	1990				2019				1990-2019 percent change (%)			
			DALYs		Death		DALYs		Death		DALYs		Death	
			Number	Rate	Number	Rate	Number	Rate	Number	Rate	Number	Rate	Number	Rate
		Male	200 (133 to 288)	21.3 (14.3 to 30.1)	7 (5 to 10)	0.9 (0.6 to 1.3)	442 (336 to 564)	18.5 (14.2 to 23.5)	17 (13 to 22)	0.8 (0.6 to 1)	120.4 (34.8 to 281.7)	-13.2 (-47 to 49.5)	150 (55 to 327.6)	-11.4 (-45 to 48.6)
	Nasopharynx cancer	Both	127 (95 to 162)	5.8 (4.4 to 7.4)	4 (3 to 5)	0.2 (0.2 to 0.3)	203 (167 to 246)	4 (3.3 to 4.8)	6 (5 to 7)	0.1 (0.1 to 0.2)	59.4 (16 to 134.4)	-31.1 (-49.5 to -1.8)	74 (26 to 151.5)	-32.9 (-50.2 to -4.6)
		Female	46 (30 to 65)	4.3 (2.9 to 6.1)	1 (1 to 2)	0.2 (0.1 to 0.2)	60 (47 to 76)	2.5 (1.9 to 3.1)	2 (1 to 2)	0.1 (0.1 to 0.1)	30.5 (-12.6 to 115)	-41.7 (-60.4 to -6.6)	44.8 (-3.9 to 133.1)	-45 (-64.8 to -11.6)
		Male	81 (56 to 109)	7.2 (5 to 9.6)	2 (2 to 3)	0.2 (0.2 to 0.3)	142 (111 to 181)	5.5 (4.3 to 6.9)	4 (3 to 6)	0.2 (0.1 to 0.2)	75.7 (17.6 to 177.9)	-23 (-49 to 21.3)	89.6 (25.2 to 199.9)	-23.1 (-48.6 to 18.4)
	Other pharynx cancer	Both	98 (72 to 126)	5.4 (4 to 6.9)	3 (2 to 4)	0.2 (0.2 to 0.3)	202 (163 to 246)	4.3 (3.4 to 5.2)	8 (6 to 10)	0.2 (0.2 to 0.2)	107.1 (48.4 to 207.6)	-21.3 (-43.2 to 16.6)	128.8 (66.1 to 240.7)	-18.2 (-40.7 to 18.9)
		Female	35 (24 to 49)	4 (2.7 to 5.5)	1 (1 to 2)	0.2 (0.1 to 0.2)	64 (50 to 82)	2.7 (2.1 to 3.4)	2 (2 to 3)	0.1 (0.1 to 0.1)	83.3 (18.5 to 203.2)	-32.7 (-56 to 8.8)	103.2 (34.2 to 225.4)	-30.7 (-55.3 to 9.7)
		Male	63 (41 to 88)	6.7 (4.6 to 9.4)	2 (2 to 3)	0.3 (0.2 to 0.4)	138 (103 to 180)	5.9 (4.4 to 7.5)	5 (4 to 7)	0.3 (0.2 to 0.3)	120.4 (38.4 to 263.6)	-12.8 (-44.4 to 42.7)	142.1 (52.8 to 296.8)	-10 (-42.3 to 41.1)
	Esophageal cancer	Both	930 (635 to 1936)	54.5 (37.7 to 114.3)	34 (24 to 73)	2.5 (1.7 to 5.1)	1982 (1415 to 4600)	42.9 (30.9 to 98.9)	83 (60 to 190)	2 (1.5 to 4.7)	113 (47.3 to 206.3)	-21.2 (-44.9 to 13.4)	141.9 (70.5 to 246.2)	-17.4 (-41.5 to 17.4)
		Female	367 (225 to 792)	45.7 (28.3 to 98.3)	14 (9 to 30)	2.1 (1.3 to 4.7)	664 (454 to 1537)	29.3 (20.2 to 67.5)	29 (20 to 66)	1.4 (1 to 3.3)	80.6 (17.6 to 188.9)	-35.9 (-57.2 to 0.7)	105.2 (34.5 to 223.5)	-32.2 (-55.4 to 6.3)
		Male	563 (345 to 1217)	62.7 (39.1 to 134)	20 (13 to 44)	2.8 (1.8 to 5.9)	1318 (880 to 3198)	56.5 (37.7 to 137.3)	54 (37 to 130)	2.6 (1.8 to 6.2)	134.1 (46.3 to 282.7)	-10 (-43.3 to 45.4)	167 (69.5 to 328)	-6.3 (-39.6 to 44.3)
	Stomach cancer	Both	7487 (5800 to 9378)	429.4 (335.6 to 526.5)	271 (211 to 333)	19.2 (15.2 to 23.1)	12576 (10433 to 15076)	269.5 (225.8 to 321.7)	515 (432 to 610)	12.6 (10.5 to 14.9)	68 (22 to 130.5)	-37.2 (-53.1 to -15.3)	90.3 (42.1 to 161)	-34.6 (-49.8 to -12.9)
		Female	2579 (1834 to 3596)	304 (220.6 to 416.3)	92 (66 to 125)	13.6 (10 to 19.1)	4341 (3474 to 5390)	185.5 (149.3 to 228.5)	176 (142 to 217)	8.7 (7.1 to 10.6)	68.3 (10.9 to 167.5)	-39 (-58.5 to -5.3)	92.1 (30.4 to 193)	-36.2 (-56 to -5.7)
		Male	4908 (3500 to 6466)	549.5 (397.7 to 714.7)	179 (127 to 235)	24.9 (18.1 to 31.8)	8235 (6463 to 10280)	353.6 (279.1 to 440.3)	340 (268 to 420)	16.4 (13 to 20.2)	67.8 (13.7 to 157.6)	-35.6 (-55.6 to -2.8)	89.4 (29.3 to 189.9)	-34.2 (-53.8 to -1.9)
	Colon and rectum cancer	Both	2288 (1723 to 2924)	127.7 (97.5 to 162.7)	78 (60 to 101)	5.5 (4.2 to 6.9)	8485 (7097 to 10062)	179.1 (150.7 to 211.6)	332 (279 to 393)	8 (6.7 to 9.5)	270.8 (169.6 to 434.9)	40.3 (2.2 to 98.5)	324.4 (206.8 to 507.7)	46 (7.7 to 104)
		Female	963 (668 to 1336)	111.2 (78.4 to 150.2)	33 (23 to 44)	4.8 (3.4 to 6.3)	3494 (2786 to 4354)	147.5 (118.6 to 182.3)	135 (108 to 167)	6.6 (5.2 to 8)	262.7 (131.8 to 469.6)	32.6 (-13.4 to 104.2)	312.9 (170.5 to 537.7)	38.5 (-7.2 to 106.7)
		Male	1325 (889 to 1842)	143.3 (97.1 to 196.6)	46 (31 to 63)	6.3 (4.4 to 8.5)	4991 (3843 to 6302)	210.7 (162.8 to 265.3)	197 (153 to 246)	9.4 (7.4 to 11.8)	276.7 (141.4 to 512.1)	47 (-5.2 to 138.3)	332.6 (178.1 to 613.5)	50.9 (-0.6 to 138.9)
	Liver cancer	Both	1790 (1338 to 2345)	97 (73 to 126.2)	62 (47 to 82)	4.2 (3.1 to 5.6)	3550 (2844 to 4449)	76.7 (61.9 to 95.8)	141 (113 to 177)	3.4 (2.8 to 4.3)	98.3 (37.3 to 189.1)	-21 (-45.6 to 16.6)	127.1 (55.1 to 236.4)	-19.4 (-45 to 20.2)
		Female	668 (451 to 984)	73.2 (48 to 111.4)	22 (15 to 35)	3.2 (2.1 to 5)	1274 (985 to 1625)	55.1 (42.7 to 68.9)	50 (39 to 63)	2.5 (1.9 to 3.1)	90.9 (12.3 to 222.7)	-24.8 (-55.6 to 24.1)	124.3 (26.5 to 272.3)	-22.6 (-56.3 to 28)
		Male	1123 (787 to 1565)	119.5 (84.3 to 164.4)	40 (28 to 56)	5.3 (3.8 to 7.2)	2276 (1643 to 3175)	98.4 (71.8 to 135.2)	91 (67 to 124)	4.4 (3.2 to 6)	102.7 (30.5 to 223.3)	-17.7 (-47.1 to 29)	128.6 (45.6 to 259.6)	-17.9 (-47.2 to 29.1)
	Gallbladder and biliary tract cancer	Both	401 (303 to 595)	24.1 (18.5 to 35.9)	15 (12 to 23)	1.1 (0.9 to 1.7)	956 (787 to 1207)	20.7 (17.1 to 26.9)	40 (33 to 53)	1 (0.8 to 1.3)	138.5 (77.9 to 240.1)	-14 (-34.8 to 20.5)	163.1 (100.6 to 271.5)	-11.6 (-31.8 to 22.2)
		Female	191 (124 to 304)	24.2 (16.3 to 39.9)	7 (5 to 12)	1.1 (0.8 to 1.9)	461 (350 to 650)	20.4 (15.6 to 29.2)	20 (15 to 29)	1 (0.8 to 1.5)	141.6 (61.1 to 297.2)	-15.8 (-42.3 to 33.6)	168.3 (85.1 to 328.8)	-11.1 (-39.1 to 38.5)

Province	Cause	Sex	1990				2019				1990-2019 percent change (%)			
			DALYs		Death		DALYs		Death		DALYs		Death	
			Number	Rate	Number	Rate	Number	Rate	Number	Rate	Number	Rate	Number	Rate
		Male	210 (146 to 306)	23.9 (16.6 to 35.1)	8 (5 to 11)	1.1 (0.8 to 1.6)	495 (376 to 644)	21 (15.9 to 27.6)	20 (15 to 27)	1 (0.7 to 1.3)	135.8 (52.9 to 278.4)	-12.1 (-41.4 to 39.8)	158.2 (74.7 to 306.1)	-11.5 (-38.9 to 37.6)
	Pancreatic cancer	Both	719 (508 to 962)	41.2 (29.4 to 54.7)	26 (19 to 35)	1.8 (1.3 to 2.4)	4485 (3698 to 5355)	96.2 (79.8 to 114.2)	183 (152 to 216)	4.4 (3.6 to 5.2)	523.9 (327.1 to 841.3)	133.3 (61.3 to 249.4)	590.3 (374.9 to 925.3)	143.7 (70.6 to 258.4)
		Female	240 (163 to 344)	29.1 (20 to 41.5)	9 (6 to 13)	1.3 (0.9 to 1.8)	1678 (1322 to 2086)	72.8 (57.4 to 90.6)	69 (54 to 87)	3.4 (2.7 to 4.2)	599.1 (326.2 to 1045.6)	150.4 (57.1 to 296.2)	680.2 (385.6 to 1149)	165.6 (62.7 to 315.3)
		Male	479 (299 to 700)	52.9 (33.7 to 77.3)	18 (11 to 26)	2.3 (1.5 to 3.4)	2807 (2181 to 3536)	119.6 (93.2 to 149.9)	113 (89 to 142)	5.4 (4.2 to 6.7)	486.2 (257.9 to 896.5)	125.9 (39.6 to 278.6)	544.8 (297.4 to 986.4)	129.3 (41.7 to 275.5)
	Larynx cancer	Both	1106 (841 to 1401)	61.5 (47.2 to 77)	38 (29 to 48)	2.5 (2 to 3.1)	2294 (1852 to 2816)	48.1 (39.2 to 58.6)	86 (71 to 105)	2 (1.7 to 2.5)	107.3 (46 to 208)	-21.9 (-43.9 to 15.3)	129.5 (64.3 to 236.2)	-18.6 (-40.5 to 18.3)
		Female	279 (191 to 389)	31.4 (22.2 to 42.5)	9 (6 to 12)	1.2 (0.9 to 1.6)	465 (365 to 591)	19.6 (15.5 to 24.7)	18 (14 to 22)	0.8 (0.7 to 1.1)	66.5 (12.1 to 163.8)	-37.4 (-57 to -3.2)	92.6 (32.2 to 206)	-31.6 (-51.7 to 4.4)
		Male	827 (569 to 1103)	90.8 (63.9 to 119.7)	28 (20 to 38)	3.8 (2.8 to 5)	1829 (1406 to 2320)	76.5 (59.1 to 96.8)	69 (53 to 87)	3.2 (2.5 to 4.1)	121.1 (45.3 to 267.6)	-15.8 (-43.7 to 38.8)	141.3 (61.1 to 299.8)	-16.1 (-42.5 to 33.4)
	Tracheal, bronchus, and lung cancer	Both	4525 (3322 to 6076)	251.8 (185.5 to 338.1)	161 (117 to 216)	10.8 (8.1 to 14.3)	13158 (10982 to 15782)	280.1 (233.5 to 334)	522 (434 to 623)	12.5 (10.4 to 14.9)	190.8 (93.5 to 345.9)	11.2 (-26 to 67.4)	223.3 (117.4 to 388.2)	15.1 (-21.4 to 67.9)
		Female	899 (604 to 1270)	100.4 (70.2 to 140.5)	30 (21 to 42)	4.2 (3 to 5.9)	3851 (3000 to 4759)	162.6 (127.8 to 199.7)	149 (118 to 183)	7.2 (5.7 to 8.8)	328.3 (166.8 to 581.6)	62 (1.5 to 153.9)	399.5 (212.8 to 685.9)	70.4 (8.7 to 164.6)
		Male	3626 (2481 to 5056)	397.1 (275.3 to 547.9)	131 (90 to 184)	17.7 (12.4 to 24.1)	9308 (7261 to 11762)	397.7 (311.2 to 502.3)	372 (292 to 469)	17.7 (13.9 to 22.2)	156.7 (57.7 to 322.1)	0.2 (-38.4 to 61.5)	183.2 (74.3 to 367.6)	0.2 (-36.3 to 58.6)
	Malignant skin melanoma	Both	395 (191 to 586)	20.1 (9.8 to 29.3)	12 (6 to 18)	0.9 (0.4 to 1.3)	574 (430 to 749)	11.7 (8.7 to 15)	20 (15 to 26)	0.5 (0.3 to 0.6)	45.3 (-13.6 to 192.4)	-41.8 (-64.4 to 11.2)	66.4 (4.8 to 209)	-44.6 (-63.1 to 2.2)
		Female	162 (69 to 251)	16.8 (7.3 to 25.6)	5 (2 to 7)	0.7 (0.3 to 1.1)	241 (153 to 346)	9.9 (6.2 to 13.9)	8 (5 to 11)	0.4 (0.2 to 0.5)	49 (-16.6 to 219.4)	-41.1 (-64.1 to 16.4)	67.7 (2.8 to 222.9)	-44.7 (-64 to -1.7)
		Male	233 (101 to 350)	23.3 (9.7 to 35.3)	7 (3 to 11)	1 (0.4 to 1.6)	333 (228 to 508)	13.5 (8.9 to 19.9)	12 (7 to 17)	0.6 (0.3 to 0.8)	42.7 (-19.6 to 203.1)	-42.1 (-67.1 to 23.7)	65.5 (-3 to 238.7)	-45 (-67.7 to 17.7)
	Non-melanoma skin cancer	Both	102 (70 to 132)	6.4 (4.4 to 8.2)	4 (3 to 5)	0.3 (0.2 to 0.4)	376 (313 to 452)	8.4 (7.1 to 10.1)	18 (15 to 21)	0.5 (0.4 to 0.6)	268 (160.1 to 456.2)	31.6 (-6.2 to 101.4)	336.1 (207.7 to 579.2)	41.4 (2.5 to 116.6)
		Female	13 (8 to 19)	1.6 (1 to 2.4)	0 (0 to 1)	0.1 (0.1 to 0.1)	76 (58 to 97)	3.4 (2.6 to 4.3)	3 (3 to 4)	0.2 (0.1 to 0.2)	482.1 (255.5 to 923)	107.1 (27.9 to 264)	595.7 (317.3 to 1134)	119.1 (35.1 to 312.5)
		Male	89 (59 to 118)	11.3 (7.7 to 14.8)	4 (2 to 5)	0.6 (0.4 to 0.8)	300 (239 to 373)	13.4 (10.8 to 16.7)	14 (12 to 18)	0.7 (0.6 to 0.9)	236.6 (131.2 to 422.4)	18.7 (-18.3 to 85.9)	300.3 (173.7 to 527.4)	20.3 (-16.1 to 86.6)
	Breast cancer	Both	2777 (1870 to 4048)	140.2 (94.6 to 204.8)	77 (51 to 115)	4.5 (3 to 6.8)	10649 (8560 to 13207)	193.2 (156 to 236.5)	295 (238 to 364)	6 (4.8 to 7.3)	283.4 (147.3 to 516.2)	37.7 (-12.4 to 122.2)	281.9 (134.2 to 530.6)	33 (-20.3 to 118.4)
		Female	2739 (1834 to 3999)	284.3 (190.9 to 417.8)	76 (50 to 114)	8.9 (5.8 to 13.5)	10545 (8458 to 13079)	385.4 (311.2 to 472.5)	292 (233 to 361)	11.9 (9.5 to 14.6)	285 (146.8 to 523.1)	35.6 (-14.4 to 119.3)	283.3 (133.7 to 535.8)	32.9 (-20.6 to 118.6)
		Male	38 (23 to 60)	3.9 (2.3 to 6)	1 (1 to 2)	0.1 (0.1 to 0.2)	104 (73 to 148)	4.2 (2.9 to 5.8)	3 (2 to 5)	0.2 (0.1 to 0.2)	171.9 (46.5 to 417.1)	8.1 (-39.3 to 103)	193.1 (56.6 to 454.2)	6 (-39.1 to 93.4)

Province	Cause	Sex	1990				2019				1990-2019 percent change (%)			
			DALYs		Death		DALYs		Death		DALYs		Death	
			Number	Rate	Number	Rate	Number	Rate	Number	Rate	Number	Rate	Number	Rate
	Cervical cancer	Female	753 (467 to 1072)	78.7 (49.5 to 111)	22 (14 to 31)	2.8 (1.9 to 4)	1333 (994 to 1711)	52.1 (39.1 to 67.5)	43 (33 to 56)	1.9 (1.5 to 2.5)	77 (17.8 to 196.9)	-33.7 (-54.9 to 6.4)	96.1 (33.7 to 215.8)	-30.6 (-52.6 to 7.3)
	Uterine cancer	Female	120 (78 to 190)	13.7 (9.1 to 22.3)	4 (3 to 7)	0.5 (0.4 to 0.9)	464 (301 to 597)	18.9 (12.9 to 24.1)	15 (11 to 20)	0.7 (0.5 to 0.9)	286.3 (97.7 to 576.9)	37.6 (-29.2 to 135.6)	292.8 (111.5 to 571.1)	33.1 (-29.1 to 119.2)
	Ovarian cancer	Female	377 (208 to 685)	37.4 (20.9 to 69.2)	11 (6 to 19)	1.2 (0.7 to 2.3)	2079 (1402 to 2684)	81.3 (56.1 to 104.3)	65 (46 to 83)	2.8 (2 to 3.5)	451.1 (133.2 to 990.5)	117.5 (-9.3 to 326)	512 (154.2 to 1072.2)	125.7 (-5.4 to 329.6)
	Prostate cancer	Male	1241 (831 to 1686)	191 (130.9 to 256)	56 (37 to 77)	11.8 (8.2 to 15.8)	4460 (3124 to 5674)	227.2 (161.8 to 288)	250 (179 to 316)	14 (10.1 to 17.6)	259.5 (138 to 472.6)	18.9 (-19.7 to 88.2)	343.2 (194.7 to 619.7)	18.5 (-18 to 87.5)
	Testicular cancer	Male	17 (12 to 22)	0.8 (0.6 to 1)	0 (0 to 0)	0 (0 to 0)	234 (168 to 319)	8.7 (6.3 to 11.7)	4 (3 to 5)	0.2 (0.1 to 0.2)	1289.8 (813.5 to 2178.7)	985.1 (631.7 to 1635.6)	1577.1 (1029.3 to 2548.7)	960.8 (606.2 to 1642.9)
	Kidney cancer	Both	603 (415 to 774)	23.4 (15.1 to 30.4)	15 (9 to 19)	0.8 (0.5 to 1.1)	1637 (1365 to 1933)	34.4 (28.8 to 40.6)	55 (46 to 65)	1.3 (1.1 to 1.5)	171.3 (91.4 to 318.9)	47 (4.6 to 141.5)	274.2 (165.1 to 528.2)	54.7 (9.7 to 177.3)
		Female	231 (161 to 308)	17.5 (11 to 23.4)	5 (3 to 7)	0.6 (0.3 to 0.9)	517 (414 to 632)	22.2 (17.9 to 27)	16 (13 to 20)	0.8 (0.6 to 0.9)	124.4 (53.6 to 250.8)	26.9 (-11.3 to 114.6)	203.8 (104.8 to 448.2)	23.8 (-18.6 to 155.9)
		Male	373 (234 to 524)	29 (17.5 to 40.3)	9 (6 to 13)	1 (0.6 to 1.5)	1119 (871 to 1395)	46.6 (36.7 to 57.9)	39 (30 to 49)	1.8 (1.4 to 2.3)	200.2 (94.7 to 427.7)	61 (4.8 to 189.2)	313.5 (163.4 to 659.5)	72.8 (8.2 to 224.3)
	Bladder cancer	Both	855 (595 to 1132)	54.4 (37.9 to 71.4)	34 (23 to 44)	2.7 (1.9 to 3.6)	2703 (2219 to 3287)	61.9 (50.9 to 75.1)	121 (99 to 147)	3.1 (2.6 to 3.8)	216.2 (114.4 to 389.3)	13.8 (-21.9 to 73.2)	258.5 (143.2 to 461.9)	15 (-20.3 to 75.7)
		Female	151 (100 to 217)	20.6 (13.8 to 29.7)	6 (4 to 9)	1.1 (0.7 to 1.7)	473 (380 to 584)	22.6 (18.1 to 28)	23 (18 to 29)	1.3 (1 to 1.6)	213.3 (99.4 to 433.8)	9.5 (-30.2 to 85.7)	276.1 (137.9 to 541.5)	15.8 (-28.9 to 96.5)
		Male	704 (462 to 975)	88.5 (59.3 to 119.1)	27 (18 to 38)	4.5 (3.1 to 6.1)	2231 (1755 to 2826)	101.3 (79.8 to 126.9)	97 (77 to 123)	5 (3.9 to 6.2)	216.8 (104 to 422.1)	14.5 (-24.5 to 84.3)	254.6 (133.1 to 486.3)	9.1 (-27.1 to 75.7)
	Brain and central nervous system cancer	Both	5847 (3025 to 7928)	178 (97.1 to 239.8)	112 (62 to 150)	4.6 (2.8 to 6.3)	9221 (4594 to 12169)	183.6 (91.9 to 241.8)	243 (116 to 324)	5.2 (2.5 to 6.9)	57.7 (-14.2 to 128.9)	3.1 (-40.2 to 48.4)	117.5 (19.5 to 214.9)	12.3 (-36.5 to 65)
		Female	2595 (957 to 4236)	155.5 (65.2 to 253.1)	47 (20 to 76)	3.9 (1.9 to 6.1)	4161 (1894 to 5782)	170.4 (77.7 to 234.7)	110 (50 to 151)	4.8 (2.2 to 6.6)	60.3 (-18.4 to 225.6)	9.6 (-44.1 to 111.8)	133.8 (19.5 to 344.2)	25.2 (-35.5 to 129.3)
		Male	3252 (1477 to 4855)	199 (95.8 to 293.7)	65 (31 to 95)	5.4 (2.7 to 8)	5061 (2221 to 7307)	196.6 (86.6 to 282.3)	133 (56 to 194)	5.6 (2.3 to 8)	55.6 (-25.5 to 148.6)	-1.2 (-48.5 to 61.6)	105.5 (-0.7 to 237.7)	3.6 (-45.8 to 74.7)
	Thyroid cancer	Both	146 (108 to 230)	7.8 (5.8 to 12.4)	5 (3 to 7)	0.3 (0.2 to 0.5)	560 (458 to 677)	11.3 (9.4 to 13.7)	17 (14 to 21)	0.4 (0.3 to 0.5)	283.1 (124.2 to 453.8)	44.7 (-12.5 to 108.7)	275.6 (139.9 to 454.8)	26.2 (-19.4 to 87)
		Female	85 (58 to 148)	9.6 (6.6 to 16.8)	3 (2 to 5)	0.4 (0.3 to 0.7)	315 (221 to 411)	12.9 (9.4 to 16.8)	10 (7 to 12)	0.5 (0.4 to 0.6)	272.7 (83.7 to 493.4)	35.3 (-29.8 to 112)	256.5 (93.2 to 472.5)	17.9 (-36.6 to 88.7)
		Male	62 (43 to 99)	6.1 (4.1 to 9.7)	2 (1 to 3)	0.2 (0.2 to 0.4)	245 (185 to 312)	9.7 (7.5 to 12.4)	8 (6 to 10)	0.3 (0.3 to 0.4)	297.4 (132.2 to 546.1)	60.1 (-4.4 to 159.7)	302.8 (138.6 to 561.3)	44.9 (-12.3 to 141.6)
	Mesothelioma	Both	16 (9 to 27)	0.8 (0.5 to 1.4)	0 (0 to 1)	0 (0 to 0)	68 (53 to 82)	1.3 (1 to 1.5)	2 (2 to 3)	0 (0 to 0.1)	314.5 (148.4 to 591.9)	54.6 (-8.2 to 153.6)	337.6 (157 to 641.8)	57.4 (-7.7 to 163.5)
		Female	10 (6 to 15)	1 (0.6 to 1.6)	0 (0 to 0)	0 (0 to 0)	23 (16 to 30)	0.8 (0.6 to 1.1)	1 (0 to 1)	0 (0 to 0)	133.4 (31 to 329)	-17.4 (-51.9 to 43.9)	136.1 (42 to 303.8)	-19.1 (-48.1 to 31.8)

Province	Cause	Sex	1990				2019				1990-2019 percent change (%)			
			DALYs		Death		DALYs		Death		DALYs		Death	
			Number	Rate	Number	Rate	Number	Rate	Number	Rate	Number	Rate	Number	Rate
		Male	7 (2 to 18)	0.6 (0.2 to 1.7)	0 (0 to 1)	0 (0 to 0.1)	45 (34 to 57)	1.7 (1.3 to 2.1)	1 (1 to 2)	0.1 (0 to 0.1)	574.4 (157.8 to 1943)	166.5 (-0.7 to 711.2)	645.2 (171.9 to 2241.7)	184.8 (1.4 to 765.7)
	Hodgkin lymphoma	Both	374 (199 to 803)	13.3 (7.5 to 26.9)	8 (5 to 16)	0.4 (0.2 to 0.7)	945 (537 to 1248)	18.1 (10.3 to 23.9)	21 (13 to 28)	0.4 (0.3 to 0.6)	153 (-1.6 to 455.7)	35.7 (-45.6 to 186.6)	162.2 (7.4 to 454.5)	15.1 (-51.3 to 131)
		Female	117 (51 to 255)	8.9 (4.1 to 19.1)	3 (1 to 6)	0.3 (0.1 to 0.6)	332 (177 to 471)	13.1 (7 to 18.8)	7 (4 to 10)	0.3 (0.2 to 0.4)	184.9 (0.4 to 657.1)	47.9 (-48.2 to 271.8)	182.2 (5.2 to 588.6)	15.3 (-55.8 to 174.2)
		Male	257 (135 to 629)	17.5 (9.5 to 38.9)	6 (3 to 12)	0.5 (0.3 to 1)	613 (311 to 836)	22.9 (11.8 to 31.2)	14 (8 to 19)	0.6 (0.3 to 0.8)	138.5 (-11.7 to 454.7)	30.9 (-49.7 to 190.5)	152.6 (-1.7 to 460.1)	16.3 (-53.4 to 148.9)
	Non-Hodgkin lymphoma	Both	1183 (907 to 1475)	46.1 (36.3 to 56.7)	29 (23 to 36)	1.5 (1.2 to 1.9)	2887 (2451 to 3413)	57.7 (48.9 to 68.3)	82 (70 to 97)	1.8 (1.5 to 2.1)	144 (81.4 to 245)	25.1 (-5.1 to 75.1)	184.4 (114.6 to 300.5)	20.3 (-9 to 67.4)
		Female	368 (238 to 503)	29.6 (18.3 to 39.6)	9 (6 to 12)	1 (0.6 to 1.3)	977 (778 to 1218)	39.8 (31.8 to 49.6)	28 (22 to 35)	1.2 (1 to 1.5)	165.7 (66.8 to 362.5)	34.3 (-10.9 to 144)	209.4 (107.4 to 472.8)	26.5 (-14 to 133)
		Male	816 (608 to 1047)	61.7 (46.1 to 79.2)	20 (15 to 26)	2 (1.5 to 2.6)	1910 (1517 to 2348)	75.3 (60.2 to 92.6)	55 (43 to 67)	2.4 (1.9 to 2.9)	134.2 (67.3 to 254.1)	22 (-12.3 to 82.5)	173.1 (92.9 to 307.6)	17 (-16.8 to 75.9)
	Multiple myeloma	Both	347 (238 to 548)	19.5 (13.4 to 31.3)	12 (9 to 20)	0.8 (0.5 to 1.3)	943 (707 to 1319)	19.8 (14.7 to 28.1)	35 (26 to 51)	0.8 (0.6 to 1.2)	171.3 (65.3 to 303.4)	1.2 (-36.6 to 49.1)	181.9 (81.4 to 306.4)	0.8 (-34.4 to 46.8)
		Female	142 (93 to 212)	17.2 (11.3 to 26.1)	5 (3 to 8)	0.7 (0.5 to 1.2)	359 (267 to 492)	15.1 (11.2 to 21)	13 (10 to 19)	0.6 (0.5 to 0.9)	152.2 (61.1 to 320.1)	-11.9 (-41.9 to 43.2)	154.4 (70.9 to 309.9)	-14.3 (-40.9 to 38)
		Male	205 (127 to 365)	21.7 (13.3 to 39)	7 (4 to 13)	0.9 (0.5 to 1.6)	584 (400 to 902)	24.4 (16.7 to 38)	22 (15 to 35)	1 (0.7 to 1.6)	184.6 (55.6 to 377.4)	12.3 (-38.1 to 87.2)	202.1 (68.9 to 398.4)	13 (-35.9 to 85.9)
	Leukemia	Both	11696 (7264 to 16124)	340.6 (210.1 to 456.6)	215 (134 to 290)	9.1 (5.9 to 12)	12239 (7886 to 15481)	253.6 (164.4 to 318.7)	319 (211 to 400)	7.2 (4.8 to 9)	4.7 (-23.9 to 54.2)	-25.5 (-45.2 to 5.8)	48.4 (9.4 to 110.5)	-21 (-41.7 to 9.4)
		Female	5185 (2351 to 7142)	294.1 (156 to 399.3)	90 (48 to 121)	7.2 (4.3 to 9.8)	4785 (2746 to 6098)	201 (117.9 to 255.3)	120 (73 to 150)	5.4 (3.4 to 6.7)	-7.7 (-37.4 to 52.5)	-31.7 (-52.8 to 5.1)	33.5 (-4.7 to 106.3)	-25.8 (-46.3 to 7.8)
		Male	6511 (2765 to 9567)	385.6 (189.1 to 548.1)	125 (61 to 179)	11 (6.1 to 15.3)	7455 (3899 to 10104)	305 (162 to 410.1)	199 (111 to 265)	8.9 (5.1 to 11.8)	14.5 (-24.3 to 89.4)	-20.9 (-46.5 to 26.5)	59 (5 to 155.8)	-18.8 (-44.6 to 25.7)
	Other malignant neoplasms	Both	3232 (2471 to 4549)	113.5 (85.9 to 147.9)	71 (53 to 92)	3.7 (2.7 to 4.7)	5560 (4742 to 6493)	116.9 (99.8 to 136)	158 (132 to 188)	3.7 (3.1 to 4.4)	72 (26.1 to 140.5)	2.9 (-23 to 46.1)	124.1 (66.3 to 222.7)	0.2 (-25.3 to 53.8)
		Female	1310 (973 to 1702)	90 (66.2 to 120)	27 (20 to 36)	2.8 (2 to 3.7)	2242 (1868 to 2690)	96 (80.5 to 115)	61 (50 to 76)	2.9 (2.3 to 3.5)	71.1 (24.2 to 148.3)	6.7 (-22.9 to 53.3)	126.2 (62.2 to 235.9)	3.7 (-25.9 to 58.7)
		Male	1922 (1369 to 2959)	136.5 (95.2 to 196.5)	44 (30 to 63)	4.6 (3.2 to 6.3)	3318 (2630 to 4109)	137.4 (109.2 to 170.2)	97 (76 to 123)	4.5 (3.5 to 5.7)	72.7 (11.1 to 175.6)	0.7 (-33.2 to 63.8)	122.9 (43.7 to 268.6)	-3.2 (-35.6 to 64)
	Other neoplasms	Both	106 (75 to 138)	5.3 (3.4 to 6.9)	3 (2 to 4)	0.2 (0.1 to 0.3)	214 (174 to 270)	4.7 (3.9 to 6)	7 (5 to 9)	0.2 (0.1 to 0.2)	101.7 (53.5 to 190.3)	-10.9 (-34.8 to 36.1)	139.6 (60.8 to 318.9)	-17.2 (-45.1 to 52.3)
		Female	44 (29 to 59)	4.3 (2.5 to 5.9)	1 (0 to 1)	0.2 (0.1 to 0.2)	85 (68 to 111)	3.7 (3 to 4.7)	2 (2 to 3)	0.1 (0.1 to 0.2)	95.6 (37 to 219.1)	-12.9 (-40.5 to 64.7)	129.2 (37.8 to 472.5)	-23.5 (-54.6 to 115.1)
		Male	62 (42 to 85)	6.3 (3.9 to 8.7)	2 (1 to 3)	0.3 (0.2 to 0.4)	129 (97 to 178)	5.7 (4.3 to 7.9)	5 (3 to 7)	0.2 (0.2 to 0.3)	106 (45.2 to 205.3)	-9.1 (-38.7 to 39)	145.5 (52.5 to 308.2)	-15.1 (-46.9 to 39)
	Cardiovascular diseases	Both	155587 (129170 to 184006)	9972.5 (8379.5 to 11547.8)	5910 (4913 to 6932)	528.8 (449.6 to 603.8)	246929 (215000 to 282364)	5821 (5084.4 to 6627.9)	11981 (10369 to 13652)	332.2 (287.3 to 375.4)	58.7 (26.5 to 103.2)	-41.6 (-52.4 to -27)	102.7 (63.8 to 155.9)	-37.2 (-47.8 to -23)
		Female	60831 (47714 to 75356)	8436 (6781 to 10223.2)	2473 (1962 to 3009)	478.3 (390 to 568.6)	101068 (84984 to 118202)	5073.1 (4265.7 to 5886)	5337 (4380 to 6275)	319 (262 to 371.1)	66.1 (28.1 to 119.9)	-39.9 (-52.4 to -22)	115.8 (67.3 to 183.7)	-33.3 (-47.1 to -15.4)

Province	Cause	Sex	1990				2019				1990-2019 percent change (%)			
			DALYs		Death		DALYs		Death		DALYs		Death	
			Number	Rate	Number	Rate	Number	Rate	Number	Rate	Number	Rate	Number	Rate
		Male	94757 (72316 to 119935)	11339.2 (8833.3 to 13941.6)	3437 (2644 to 4303)	572.2 (459.4 to 691.6)	145861 (119925 to 177087)	6580.4 (5449.6 to 7944.4)	6645 (5513 to 8004)	347.9 (288.7 to 414.2)	53.9 (14.4 to 116.8)	-42 (-56.2 to -19.9)	93.3 (44.9 to 171)	-39.2 (-52.8 to -19.1)
	Rheumatic heart disease	Both	3524 (2729 to 4571)	122.3 (93.9 to 163.5)	61 (46 to 84)	3.2 (2.3 to 4.8)	2992 (2375 to 3782)	58.3 (46.5 to 72.9)	55 (43 to 66)	1.3 (1 to 1.6)	-15.1 (-37.4 to 9.7)	-52.4 (-66.5 to -37.1)	-9.9 (-45.3 to 30.6)	-59.8 (-77.3 to -38.7)
		Female	1925 (1358 to 2538)	135.8 (96.6 to 185.8)	33 (23 to 48)	3.6 (2.4 to 5.8)	1556 (1177 to 2008)	62 (47.7 to 79.4)	30 (22 to 39)	1.5 (1.1 to 1.9)	-19.2 (-42 to 21.7)	-54.3 (-68.4 to -32)	-11.2 (-47.7 to 52.6)	-59.5 (-78.8 to -31)
		Male	1599 (1081 to 2182)	108.6 (73.2 to 155.9)	28 (17 to 44)	2.8 (1.7 to 4.8)	1436 (1085 to 1871)	54.6 (41.2 to 70.9)	25 (18 to 34)	1.2 (0.8 to 1.5)	-10.2 (-42.4 to 35.9)	-49.7 (-68.7 to -21.1)	-8.5 (-51.5 to 78.5)	-59 (-79.4 to -22.2)
	Ischemic heart disease	Both	100920 (82007 to 121304)	6570.3 (5468.8 to 7726.1)	4056 (3363 to 4781)	352.9 (297.9 to 404.7)	135016 (115505 to 158144)	3160 (2712.1 to 3677.1)	6840 (5882 to 7918)	188.1 (162.1 to 217.7)	33.8 (5.2 to 79.7)	-51.9 (-61.3 to -37.9)	68.6 (36.2 to 120.5)	-46.7 (-55.9 to -33.3)
		Female	34945 (26792 to 43790)	5077.3 (3991.3 to 6153.7)	1568 (1233 to 1902)	301.9 (242.6 to 358.5)	49579 (40870 to 59011)	2520.5 (2083.2 to 2985.3)	2884 (2383 to 3417)	173.1 (142.3 to 203.9)	41.9 (5.6 to 95.2)	-50.4 (-62.1 to -33.3)	83.9 (40.4 to 146)	-42.7 (-55.2 to -26)
		Male	65974 (49552 to 84387)	7936.9 (6122.4 to 9917.9)	2488 (1894 to 3131)	400.1 (316.6 to 487.9)	85438 (68744 to 105373)	3804.7 (3093.7 to 4680.8)	3955 (3253 to 4801)	204.3 (168.8 to 246.7)	29.5 (-5.8 to 90.5)	-52.1 (-64.6 to -30.7)	59 (17.2 to 128.3)	-48.9 (-61.4 to -29.6)
	Stroke	Both	33221 (27380 to 40251)	2251.2 (1871.7 to 2708.1)	1241 (1008 to 1530)	122.7 (98.3 to 145.2)	61513 (53225 to 70258)	1478.3 (1280.7 to 1680)	2928 (2473 to 3369)	82.7 (69.1 to 94.9)	85.2 (45 to 134.1)	-34.3 (-48.3 to -18.4)	135.9 (81.2 to 202.5)	-32.6 (-46.3 to -16)
		Female	15560 (12373 to 19539)	2197.3 (1728.2 to 2775)	597 (450 to 760)	121.1 (93.9 to 152.7)	28723 (23995 to 33549)	1434.3 (1204.8 to 1663.8)	1403 (1127 to 1667)	83.8 (67.3 to 99.3)	84.6 (40 to 144.9)	-34.7 (-49.7 to -14.2)	135 (73.3 to 221.9)	-30.8 (-47.3 to -8.7)
		Male	17660 (13438 to 22268)	2278 (1758.5 to 2838.2)	644 (483 to 831)	122.2 (92.2 to 153.8)	32789 (26819 to 39768)	1526.3 (1251.6 to 1845)	1525 (1207 to 1864)	82.2 (64.8 to 99.8)	85.7 (34.2 to 159.9)	-33 (-51.2 to -7.3)	136.7 (65.5 to 236.9)	-32.7 (-50.6 to -8)
	Hypertensive heart disease	Both	8164 (6230 to 10962)	591.2 (452.5 to 766.7)	344 (259 to 456)	34.3 (26.8 to 45.4)	26445 (20217 to 30729)	647.7 (479.8 to 752.1)	1455 (996 to 1699)	41.3 (27.9 to 48.1)	223.9 (101.8 to 352.9)	9.5 (-30.5 to 48.9)	322.6 (150.5 to 492.9)	20.1 (-28.6 to 65.2)
		Female	3968 (2799 to 5803)	607.7 (437.7 to 901.6)	181 (127 to 275)	36.8 (27 to 56.3)	11902 (8376 to 14523)	616.1 (421.6 to 747.6)	710 (473 to 870)	42.9 (27.9 to 52.6)	200 (68.6 to 372.4)	1.4 (-43.5 to 53.8)	291.6 (106.5 to 509.1)	16.7 (-38.4 to 75.7)
		Male	4196 (2947 to 6195)	565.4 (405.3 to 788)	163 (113 to 234)	30.9 (22.5 to 42.2)	14542 (10591 to 18050)	681.7 (491.4 to 842.3)	745 (501 to 926)	40 (26.7 to 49.7)	246.5 (93.6 to 443.5)	20.6 (-29.7 to 81.2)	357.2 (140.6 to 620.3)	29.3 (-28.6 to 89.6)
	Non-rheumatic valvular heart disease	Both	795 (583 to 1052)	41 (31.9 to 51)	25 (19 to 31)	1.8 (1.5 to 2.2)	1634 (1370 to 1980)	34.1 (28.5 to 41.9)	61 (51 to 76)	1.5 (1.3 to 1.9)	105.6 (45.7 to 199.3)	-16.8 (-37.3 to 13.8)	149.3 (88.7 to 241.6)	-17 (-35.9 to 10.7)
		Female	402 (262 to 569)	44.6 (32.4 to 58.5)	13 (10 to 17)	2.1 (1.6 to 2.7)	799 (636 to 985)	34.7 (27.9 to 42.6)	32 (26 to 39)	1.7 (1.3 to 2.1)	98.6 (28.1 to 245.6)	-22.2 (-46.3 to 20.3)	143.5 (68.6 to 268.8)	-19.4 (-43.7 to 15.8)
		Male	393 (264 to 577)	36.6 (25.1 to 52.1)	11 (8 to 16)	1.5 (1 to 2)	835 (629 to 1128)	33.5 (25.3 to 46.2)	29 (22 to 42)	1.3 (1 to 1.9)	112.8 (33.1 to 236.2)	-8.4 (-41.1 to 41.9)	156 (64.4 to 296.8)	-7.8 (-38.1 to 38.7)
	Cardiomyopathy and myocarditis	Both	1394 (907 to 3200)	51.7 (36.2 to 96.4)	32 (22 to 60)	1.9 (1.3 to 3.3)	1888 (1403 to 2987)	41.8 (31.2 to 64.8)	62 (47 to 99)	1.6 (1.2 to 2.5)	35.4 (-23 to 100.7)	-19.2 (-46.3 to 13.4)	97.6 (24.1 to 180.3)	-19.2 (-45.5 to 11)
		Female	558 (319 to 1297)	41 (24.8 to 84.9)	12 (7 to 26)	1.6 (0.9 to 3.6)	619 (445 to 1057)	28.9 (21 to 47.9)	21 (15 to 36)	1.1 (0.8 to 1.9)	11 (-37.8 to 94.7)	-29.4 (-56.9 to 14)	71.9 (1.9 to 186.3)	-29.7 (-61.3 to 28.3)
		Male	837 (517 to 1887)	61.7 (39.9 to 117.3)	19 (12 to 37)	2.2 (1.5 to 3.8)	1269 (890 to 2138)	54.4 (38.1 to 90.4)	41 (28 to 69)	2 (1.4 to 3.3)	51.6 (-14.6 to 139.6)	-11.8 (-44.1 to 32.6)	114.2 (32 to 236.5)	-11.2 (-39 to 30.3)

Province	Cause	Sex	1990				2019				1990-2019 percent change (%)			
			DALYs		Death		DALYs		Death		DALYs		Death	
			Number	Rate	Number	Rate	Number	Rate	Number	Rate	Number	Rate	Number	Rate
		Both	951 (702 to 1265)	85.9 (64.6 to 111.4)	21 (15 to 26)	3.1 (2.1 to 3.9)	3344 (2554 to 4313)	88.6 (68.5 to 113.4)	112 (93 to 130)	3.6 (3 to 4.1)	251.9 (212.5 to 317.5)	3.2 (-9.9 to 24.3)	429.6 (299.3 to 733)	13.6 (-14.4 to 81.4)
	Atrial fibrillation and flutter	Female	602 (430 to 808)	109.1 (77.6 to 144.5)	14 (8 to 19)	3.9 (2.3 to 5)	2051 (1535 to 2680)	110.3 (83.6 to 142.5)	63 (50 to 78)	4.1 (3.2 to 5.2)	240.6 (195.4 to 332.6)	1.1 (-13.6 to 32.6)	341.2 (215.2 to 760.3)	6.2 (-24.6 to 108.9)
		Male	348 (250 to 469)	58.9 (44.3 to 77.5)	7 (5 to 9)	2.1 (1.4 to 2.8)	1293 (956 to 1663)	67.4 (50 to 85.9)	49 (34 to 61)	3 (2.1 to 3.7)	271.3 (196.5 to 363.7)	14.4 (-7.7 to 41.5)	611.5 (336.2 to 1000.5)	42.9 (-7.3 to 114)
		Both	339 (233 to 469)	20.5 (14.5 to 27.7)	13 (9 to 18)	1 (0.7 to 1.3)	871 (705 to 1077)	19.5 (15.8 to 24.1)	40 (32 to 49)	1 (0.8 to 1.3)	156.9 (65.3 to 312.2)	-5 (-37.7 to 49.1)	203 (99.2 to 376.6)	-0.4 (-32.5 to 51.5)
	Aortic aneurysm	Female	74 (53 to 97)	9.8 (7.2 to 12.7)	3 (2 to 4)	0.5 (0.4 to 0.7)	189 (150 to 233)	8.3 (6.7 to 10.2)	8 (7 to 10)	0.4 (0.3 to 0.5)	157.1 (79.2 to 289.4)	-14.6 (-37.7 to 24.1)	169.3 (96.1 to 289.2)	-19.6 (-41.3 to 16.4)
		Male	266 (163 to 390)	31.2 (19.4 to 44.7)	10 (6 to 15)	1.6 (1 to 2.2)	682 (520 to 886)	30.6 (23.4 to 39.4)	32 (24 to 41)	1.6 (1.2 to 2.1)	156.9 (49.8 to 370)	-2 (-41.3 to 73.4)	213.2 (88.9 to 459.9)	1.8 (-37.4 to 77.2)
		Both	75 (41 to 124)	6.3 (3.5 to 10.4)	1 (0 to 1)	0.1 (0.1 to 0.2)	265 (171 to 413)	6.9 (4.5 to 10.7)	6 (5 to 8)	0.2 (0.1 to 0.2)	252.3 (205.1 to 330.7)	9.7 (-3.6 to 30.8)	711.5 (426.9 to 1162)	99 (21.8 to 213.5)
	Peripheral artery disease	Female	42 (21 to 72)	7.2 (3.8 to 12.2)	0 (0 to 1)	0.1 (0 to 0.2)	138 (81 to 228)	7.3 (4.4 to 11.8)	2 (2 to 4)	0.1 (0.1 to 0.2)	224.2 (176.3 to 298.7)	1.1 (-14.1 to 22)	674.6 (247.7 to 1759.1)	94.4 (-16.7 to 394.1)
		Male	33 (19 to 52)	5.3 (3.2 to 8.3)	0 (0 to 1)	0.1 (0.1 to 0.1)	128 (87 to 189)	6.6 (4.4 to 9.7)	3 (3 to 4)	0.2 (0.2 to 0.3)	288.6 (214.8 to 410.5)	23.2 (3.7 to 57.5)	738.1 (438.6 to 1275.6)	100.8 (32.1 to 227.5)
		Both	414 (262 to 769)	15.7 (9.6 to 30.4)	10 (6 to 18)	0.6 (0.3 to 1.2)	642 (436 to 1113)	13.3 (9.3 to 22.6)	21 (15 to 38)	0.5 (0.4 to 0.9)	55.1 (12 to 121.5)	-15.2 (-39.6 to 19.4)	123.8 (60.5 to 208.8)	-11.3 (-34.3 to 30.1)
	Endocarditis	Female	202 (129 to 318)	14.6 (8.9 to 28.6)	4 (3 to 9)	0.6 (0.3 to 1.3)	273 (183 to 518)	12.1 (8.4 to 22.3)	10 (7 to 20)	0.5 (0.4 to 1)	34.9 (-18.7 to 140)	-17.5 (-44.3 to 20.8)	125.1 (53.5 to 225.4)	-6.3 (-37.2 to 53.7)
		Male	212 (107 to 487)	16.5 (8.5 to 35.9)	5 (3 to 11)	0.6 (0.3 to 1.2)	369 (227 to 675)	14.5 (9.1 to 26.4)	11 (7 to 21)	0.5 (0.3 to 0.9)	74.3 (12.8 to 189.7)	-12.3 (-42.4 to 45.4)	122.7 (46.9 to 268.3)	-13.1 (-39.8 to 40.4)
		Both	5791 (4694 to 7950)	216.4 (176.4 to 287.3)	107 (86 to 150)	7.1 (5.6 to 10.2)	12319 (10553 to 14341)	272.5 (233.4 to 315)	401 (332 to 473)	10.6 (8.8 to 12.4)	112.7 (53.8 to 173)	25.9 (-9.1 to 63.1)	274.6 (144.9 to 421.3)	48.2 (-9.6 to 107.2)
	Other cardiovascular and circulatory diseases	Female	2552 (1985 to 3267)	191.6 (148.9 to 260.5)	45 (34 to 67)	6.2 (4.6 to 10.4)	5239 (4332 to 6295)	238.6 (199.2 to 282.9)	173 (138 to 211)	9.7 (7.7 to 11.7)	105.3 (50.2 to 179.3)	24.5 (-14.9 to 63.9)	280.7 (123.9 to 446.9)	56.5 (-19 to 127.4)
		Male	3239 (2532 to 5443)	239.9 (186.4 to 380)	62 (46 to 107)	8.1 (6 to 12.2)	7079 (5816 to 8536)	306.2 (253.5 to 368.4)	228 (180 to 286)	11.5 (9 to 14.3)	118.6 (28.6 to 200.6)	27.6 (-22.6 to 77.9)	270.1 (102.1 to 473.4)	42 (-19.6 to 115.6)
		Both	18095 (14383 to 23924)	860.4 (699.3 to 1177.9)	351 (268 to 547)	30.7 (23.5 to 48.6)	33445 (28799 to 39110)	745.1 (642.7 to 868.5)	895 (743 to 1106)	24.5 (20.3 to 30.5)	84.8 (48.4 to 122)	-13.4 (-32 to 5.1)	155 (80.2 to 248.7)	-20.3 (-43.5 to 8.6)
	Chronic respiratory diseases	Female	8108 (6139 to 11327)	763 (569.1 to 1130.6)	144 (92 to 255)	25.3 (16.4 to 45.9)	14200 (11916 to 17198)	643.2 (539.9 to 780.6)	347 (274 to 468)	19.9 (15.6 to 26)	75.1 (34.5 to 127.5)	-15.7 (-37.8 to 13.3)	141.8 (53.5 to 310.1)	-21.1 (-50.9 to 30.3)
		Male	9987 (7870 to 13903)	961.1 (754.2 to 1373.7)	207 (149 to 352)	37 (27.7 to 59.2)	19245 (16046 to 22913)	847.1 (707.9 to 1010.7)	548 (439 to 695)	28.9 (23.2 to 36.6)	92.7 (46.5 to 147.4)	-11.9 (-34.8 to 14)	164.1 (74.4 to 301)	-21.9 (-47.2 to 13.4)
	Chronic obstructive pulmonary disease	Both	7343 (5920 to 11024)	432.1 (346.7 to 663.5)	185 (135 to 328)	17.4 (12.8 to 31.7)	21818 (18997 to 25502)	499.2 (436 to 584.6)	703 (581 to 891)	19.5 (16.1 to 24.7)	197.1 (121.1 to 271.6)	15.5 (-17.7 to 45.7)	279.5 (143 to 433.4)	11.6 (-29 to 53.7)

Province	Cause	Sex	1990				2019				1990-2019 percent change (%)			
			DALYs		Death		DALYs		Death		DALYs		Death	
			Number	Rate	Number	Rate	Number	Rate	Number	Rate	Number	Rate	Number	Rate
		Female	3164 (2281 to 5293)	370 (262 to 665.5)	73 (41 to 165)	14 (8.3 to 32.6)	8714 (7287 to 10961)	408.9 (342.9 to 514.8)	263 (206 to 374)	15.4 (12.1 to 21.2)	175.5 (82.6 to 299.7)	10.5 (-29.3 to 59.7)	259.4 (89.4 to 583.6)	10.1 (-41.7 to 101.7)
		Male	4179 (3262 to 6356)	496.7 (388.9 to 760.7)	112 (77 to 193)	21.4 (15.6 to 36)	13104 (10998 to 15697)	589.7 (495.6 to 709.9)	440 (350 to 560)	23.4 (18.7 to 29.7)	213.6 (120.3 to 322.1)	18.7 (-15.2 to 58.8)	292.6 (151.5 to 505)	9.2 (-27.9 to 59.8)
	Pneumoconiosis	Both	9 (6 to 13)	0.5 (0.4 to 0.7)	0 (0 to 0)	0 (0 to 0)	60 (46 to 78)	1.3 (1 to 1.6)	1 (1 to 1)	0 (0 to 0)	573.8 (348.2 to 873.7)	145.6 (67.2 to 261.2)	378.2 (192.5 to 802)	66.7 (7.2 to 197.2)
		Female	3 (2 to 4)	0.3 (0.2 to 0.4)	0 (0 to 0)	0 (0 to 0)	36 (25 to 52)	1.4 (1 to 2)	0 (0 to 0)	0 (0 to 0)	1242.7 (981.6 to 1597.8)	440.9 (335.9 to 557.1)	1285 (707.5 to 2283.9)	336.4 (145.8 to 650.1)
		Male	6 (4 to 10)	0.7 (0.5 to 1.2)	0 (0 to 0)	0 (0 to 0.1)	24 (19 to 31)	1.1 (0.8 to 1.4)	1 (1 to 1)	0 (0 to 0.1)	285.7 (134.6 to 590.6)	42.4 (-9.3 to 148.7)	287 (122.6 to 711.4)	28.7 (-22.8 to 163.7)
	Asthma	Both	10125 (7604 to 13366)	405.9 (306.6 to 523.1)	158 (107 to 222)	12.8 (8.3 to 18)	9556 (7277 to 12695)	204.7 (156.2 to 271.7)	159 (129 to 193)	4.2 (3.4 to 5.1)	-5.6 (-21.7 to 11.6)	-49.6 (-60.3 to -36.8)	0.2 (-31.3 to 45.9)	-67.3 (-78.6 to -51)
		Female	4635 (3302 to 6337)	371.4 (254.5 to 528.2)	67 (36 to 110)	10.8 (5.3 to 18.7)	4494 (3353 to 5978)	194.2 (145.2 to 256.9)	71 (48 to 93)	3.8 (2.6 to 5)	-3 (-24.1 to 20.9)	-47.7 (-63.5 to -29.6)	4.7 (-43.9 to 81.9)	-64.9 (-82.7 to -37.5)
		Male	5491 (4126 to 7431)	441.2 (338.4 to 594.6)	91 (63 to 143)	15 (10.6 to 22.9)	5063 (3793 to 6676)	215 (161 to 285.9)	88 (68 to 116)	4.5 (3.4 to 6)	-7.8 (-26.6 to 14.9)	-51.3 (-63.9 to -35.5)	-3.1 (-35.9 to 56.8)	-69.8 (-80.1 to -52.6)
	Interstitial lung disease and pulmonary sarcoidosis	Both	205 (150 to 316)	10.5 (7.5 to 16.6)	5 (3 to 9)	0.4 (0.2 to 0.7)	651 (488 to 810)	13.7 (10.4 to 16.7)	20 (14 to 25)	0.5 (0.4 to 0.7)	218 (95 to 350.1)	29.4 (-20.4 to 92.6)	306.5 (126.8 to 611.3)	29.7 (-26.8 to 136.1)
		Female	92 (63 to 144)	9.7 (6.1 to 16.7)	2 (1 to 4)	0.3 (0.1 to 0.7)	298 (207 to 391)	12.4 (8.6 to 16.1)	8 (5 to 12)	0.4 (0.2 to 0.6)	224 (113.7 to 396.5)	27.7 (-20.4 to 116.2)	293.8 (105.8 to 793.8)	23.4 (-38.3 to 212.1)
		Male	113 (79 to 193)	11.5 (7.6 to 20.1)	3 (2 to 6)	0.4 (0.2 to 0.9)	354 (251 to 465)	14.9 (10.6 to 19.8)	11 (7 to 16)	0.6 (0.4 to 0.9)	213.2 (64.7 to 383.4)	29.7 (-28.2 to 106.4)	316 (104.3 to 696.4)	30.4 (-30.8 to 155.9)
	Other chronic respiratory diseases	Both	413 (275 to 828)	11.3 (7.9 to 20.8)	2 (1 to 8)	0.1 (0.1 to 0.3)	1359 (1067 to 1650)	26.4 (20.3 to 32.3)	13 (6 to 19)	0.3 (0.1 to 0.4)	229.1 (49.4 to 399.8)	133 (16.3 to 237.2)	450.1 (-0.8 to 1707.1)	183 (-36.8 to 713)
		Female	215 (145 to 423)	11.7 (8.3 to 21)	1 (0 to 4)	0.1 (0 to 0.3)	658 (512 to 813)	26.3 (20.1 to 32.8)	5 (2 to 8)	0.3 (0.1 to 0.4)	205.7 (48.4 to 362.9)	124.2 (20.6 to 225.4)	442 (-13.7 to 1991.6)	181.2 (-42.6 to 810.2)
		Male	198 (119 to 447)	10.9 (7.1 to 21.8)	1 (1 to 5)	0.1 (0 to 0.4)	701 (518 to 882)	26.5 (19.5 to 33.5)	8 (3 to 12)	0.3 (0.1 to 0.5)	254.6 (49.4 to 499)	142.6 (10.9 to 288.5)	456 (-1.1 to 1949.6)	181.2 (-39.1 to 859.5)
	Digestive diseases	Both	20801 (17083 to 24730)	895 (712.7 to 1090.5)	443 (350 to 565)	31.4 (23.6 to 41.4)	30676 (25298 to 36955)	630.5 (524.1 to 753.5)	817 (684 to 971)	20.5 (17.2 to 24.2)	47.5 (17.6 to 85.7)	-29.6 (-44 to -10.8)	84.3 (36.9 to 152.8)	-34.7 (-52.3 to -10.2)
		Female	8819 (6954 to 10999)	763.5 (590.1 to 998.4)	174 (125 to 240)	26.3 (17.8 to 39.1)	12543 (10049 to 15561)	531.2 (430.4 to 648.7)	308 (251 to 379)	16.7 (13.7 to 20.3)	42.2 (12.5 to 81.2)	-30.4 (-45.9 to -10.9)	77.5 (22.9 to 155.1)	-36.5 (-58.2 to -2.9)
		Male	11982 (9208 to 14960)	1017.6 (776.1 to 1274.6)	270 (196 to 349)	36.4 (26.7 to 47.9)	18133 (14478 to 22362)	728.7 (584.5 to 888)	509 (402 to 636)	24.3 (19.3 to 30.3)	51.3 (12.4 to 111.8)	-28.4 (-46 to -0.1)	88.7 (33.1 to 184.6)	-33.4 (-52.1 to -2.5)
	Cirrhosis and other chronic liver diseases	Both	10245 (7833 to 12687)	449 (333.4 to 584.4)	277 (206 to 362)	18.4 (13.2 to 25)	15449 (12633 to 18976)	321.3 (263.8 to 392.3)	552 (455 to 668)	13.3 (11 to 15.9)	50.8 (9.9 to 109.1)	-28.5 (-47.2 to -1)	99.5 (44.9 to 180.7)	-27.9 (-49.2 to 3.9)
		Female	3978 (2759 to 5341)	346.6 (233.6 to 497.3)	106 (71 to 153)	15 (9.3 to 23.5)	5046 (3982 to 6300)	223.9 (179 to 278.1)	196 (157 to 244)	10.1 (8.2 to 12.5)	26.9 (-13.8 to 90.4)	-35.4 (-55.6 to -4.3)	84.3 (21.5 to 179.9)	-32.6 (-58.6 to 8.8)
		Male	6267 (4501 to 8106)	544.1 (386.2 to 711.3)	171 (122 to 224)	21.6 (15.3 to 29)	10402 (7950 to 13196)	417.3 (321.6 to 530.1)	357 (275 to 451)	16.3 (12.7 to 20.6)	66 (12.2 to 152)	-23.3 (-47.3 to 15.6)	109 (41.4 to 218.3)	-24.5 (-47.8 to 15)

Province	Cause	Sex	1990				2019				1990-2019 percent change (%)			
			DALYs		Death		DALYs		Death		DALYs		Death	
			Number	Rate	Number	Rate	Number	Rate	Number	Rate	Number	Rate	Number	Rate
	Upper digestive system diseases	Both	5763 (4281 to 7959)	264.9 (197.8 to 352.5)	93 (70 to 124)	7.7 (5.6 to 10.6)	8150 (5425 to 12353)	156.4 (107.3 to 229.6)	99 (80 to 119)	2.7 (2.2 to 3.3)	41.4 (10.4 to 69.7)	-41 (-54.3 to -27.3)	5.4 (-23.3 to 49.7)	-64.9 (-75 to -50)
		Female	2563 (1786 to 3704)	238.5 (168.7 to 333)	35 (24 to 53)	6.4 (4 to 10.2)	4074 (2615 to 6392)	158.2 (105.4 to 241.1)	41 (31 to 52)	2.5 (1.9 to 3.1)	58.9 (27.5 to 87.6)	-33.7 (-49.7 to -18.7)	17 (-25.5 to 94.2)	-61.5 (-77.3 to -34.6)
		Male	3200 (2357 to 4347)	290.3 (216 to 387.9)	58 (41 to 81)	9.2 (6.5 to 12.7)	4076 (2739 to 5980)	154.7 (107 to 222)	57 (44 to 72)	3 (2.3 to 3.8)	27.4 (-7.3 to 62.5)	-46.7 (-60.9 to -30.6)	-1.5 (-33.5 to 53.5)	-67.5 (-77.9 to -51.2)
	Appendicitis	Both	749 (471 to 1063)	22.9 (16.2 to 31.3)	13 (9 to 18)	0.7 (0.5 to 1)	514 (402 to 676)	10.7 (8.5 to 13.9)	11 (9 to 15)	0.3 (0.2 to 0.4)	-31.4 (-55.4 to 33.6)	-53.4 (-68.8 to -21.7)	-15.8 (-46.3 to 59.4)	-61.1 (-76.1 to -37.7)
		Female	294 (174 to 487)	18.4 (12 to 29.8)	5 (3 to 9)	0.6 (0.4 to 1.1)	218 (160 to 306)	9.6 (7.1 to 13.4)	5 (3 to 7)	0.3 (0.2 to 0.4)	-25.7 (-59.1 to 60.6)	-48 (-70.7 to -6.7)	-8.2 (-54.7 to 84.1)	-57.9 (-80.1 to -21.6)
		Male	456 (244 to 718)	27.2 (16 to 39.8)	8 (4 to 12)	0.8 (0.5 to 1.2)	296 (221 to 417)	11.8 (8.9 to 16.6)	6 (5 to 9)	0.3 (0.2 to 0.4)	-35.1 (-60.5 to 56.8)	-56.7 (-73.1 to -7.9)	-20.6 (-52.4 to 89.3)	-62.8 (-77.2 to -24.1)
	Paralytic ileus and intestinal obstruction	Both	1586 (1037 to 2390)	45.6 (33 to 63.5)	26 (18 to 37)	1.6 (1.1 to 2.3)	1304 (980 to 1715)	32.7 (24.7 to 43.3)	43 (34 to 55)	1.2 (0.9 to 1.5)	-17.8 (-51.7 to 43.8)	-28.3 (-55.2 to 12.1)	64.6 (4.6 to 148.8)	-23.1 (-48.9 to 9.5)
		Female	744 (436 to 1277)	43.5 (28.8 to 66.2)	12 (8 to 19)	1.5 (1 to 2.3)	522 (384 to 700)	27.1 (19.8 to 36.6)	17 (12 to 23)	0.9 (0.7 to 1.3)	-29.8 (-61.7 to 30.1)	-37.7 (-62 to 5)	32.9 (-17.9 to 116.5)	-36.9 (-60.3 to -0.3)
		Male	843 (501 to 1415)	47.5 (31.6 to 73)	14 (9 to 21)	1.6 (1.1 to 2.5)	782 (523 to 1098)	38.1 (25.5 to 53.5)	27 (18 to 35)	1.4 (1 to 1.9)	-7.2 (-54.1 to 88)	-19.9 (-58.4 to 43.3)	93.3 (2.2 to 239.1)	-11.5 (-48.1 to 42)
	Inguinal, femoral, and abdominal hernia	Both	575 (379 to 849)	17.6 (12.3 to 24.3)	2 (2 to 4)	0.2 (0.1 to 0.3)	786 (537 to 1127)	16.1 (11.2 to 22.9)	5 (4 to 7)	0.1 (0.1 to 0.2)	36.7 (3.7 to 75.5)	-8.4 (-23 to 7.8)	107.3 (30.8 to 248.4)	-31.9 (-60.1 to 6.1)
		Female	213 (126 to 331)	12.3 (8.2 to 17.9)	1 (0 to 2)	0.1 (0.1 to 0.3)	269 (177 to 398)	11.3 (7.5 to 16.5)	2 (1 to 3)	0.1 (0.1 to 0.2)	25.9 (-17.2 to 81.2)	-8.6 (-31.2 to 21.1)	117.6 (8.5 to 319.3)	-24.2 (-59 to 31.9)
		Male	361 (236 to 538)	22.8 (15.7 to 31.6)	2 (1 to 2)	0.3 (0.2 to 0.5)	517 (343 to 739)	20.9 (14.1 to 29.7)	3 (2 to 4)	0.2 (0.1 to 0.2)	43.1 (5.4 to 84.3)	-8.4 (-26.6 to 10.5)	101.8 (13.8 to 255.7)	-41 (-69.7 to 2.8)
	Inflammatory bowel disease	Both	205 (148 to 280)	9.4 (6.8 to 12.5)	2 (1 to 3)	0.1 (0.1 to 0.2)	550 (399 to 732)	10.3 (7.7 to 13.6)	6 (4 to 8)	0.1 (0.1 to 0.2)	168.7 (97.6 to 231.2)	10.1 (-11 to 34)	211.2 (70.7 to 404.3)	11.4 (-30.7 to 80.7)
		Female	97 (65 to 139)	8.9 (6.2 to 12.8)	1 (0 to 1)	0.1 (0.1 to 0.2)	252 (178 to 345)	9.4 (6.7 to 12.7)	2 (1 to 3)	0.1 (0.1 to 0.1)	160.1 (93.2 to 237)	5.5 (-18.2 to 35.7)	203.7 (66.3 to 484.6)	9 (-41.5 to 119.2)
		Male	108 (78 to 151)	9.8 (7.1 to 13.2)	1 (1 to 2)	0.2 (0.1 to 0.3)	299 (213 to 402)	11.2 (8 to 14.9)	4 (2 to 6)	0.2 (0.1 to 0.3)	176.5 (87.3 to 270)	14.4 (-16.4 to 49.2)	215.5 (54.7 to 451.8)	11 (-38.9 to 90.6)
	Vascular intestinal disorders	Both	141 (96 to 200)	10.1 (6.5 to 15.1)	6 (4 to 9)	0.6 (0.4 to 0.9)	367 (297 to 451)	8.8 (7 to 10.7)	19 (15 to 23)	0.5 (0.4 to 0.6)	159.3 (78 to 285.6)	-13.5 (-44.2 to 33.7)	205.3 (96.6 to 380.6)	-12.8 (-45.3 to 41.4)
		Female	63 (35 to 108)	9.9 (4.8 to 17.8)	3 (1 to 6)	0.6 (0.3 to 1.1)	155 (116 to 202)	7.7 (5.8 to 9.9)	8 (6 to 11)	0.5 (0.4 to 0.6)	144.7 (45.9 to 317.2)	-22.4 (-56.4 to 47.6)	169.1 (44.6 to 444.1)	-23.3 (-59.9 to 69.8)
		Male	78 (54 to 111)	10.2 (7 to 14.5)	3 (2 to 5)	0.6 (0.4 to 0.8)	212 (158 to 277)	9.8 (7.4 to 12.8)	11 (8 to 14)	0.6 (0.4 to 0.7)	171.2 (73.1 to 325.4)	-3.7 (-37.3 to 46.1)	240.7 (116.4 to 440.7)	-1.3 (-36.5 to 50.5)
	Gallbladder and biliary diseases	Both	993 (648 to 1464)	48 (32.5 to 70.4)	9 (6 to 17)	0.9 (0.6 to 1.6)	2223 (1587 to 3039)	45.3 (33.3 to 61.2)	36 (28 to 46)	1 (0.8 to 1.3)	123.9 (81.8 to 169.5)	-5.6 (-23.6 to 12)	299.6 (139.4 to 552.1)	15.6 (-25.1 to 79.4)
		Female	651 (418 to 969)	62.4 (41.9 to 90.9)	4 (2 to 9)	0.9 (0.5 to 1.6)	1473 (1014 to 2087)	59.5 (42.7 to 82.1)	18 (14 to 24)	1.1 (0.8 to 1.5)	126.3 (80.3 to 171)	-4.6 (-22.8 to 14.3)	325.1 (134.1 to 657.4)	27.9 (-25.2 to 128.3)
		Male	342 (222 to 522)	34.3 (23 to 52.2)	5 (3 to 9)	0.9 (0.5 to 1.7)	750 (545 to 1021)	31.6 (23.6 to 42)	17 (13 to 24)	0.9 (0.7 to 1.3)	119.3 (61.9 to 189.2)	-7.9 (-32 to 21.9)	275.4 (116.5 to 603.8)	6 (-35.1 to 84.8)
	Pancreatitis	Both	286 (176 to 480)	15.8 (9.8 to 26.2)	9 (5 to 15)	0.7 (0.4 to 1.2)	567 (422 to 931)	11.9 (9 to 18.9)	21 (16 to 33)	0.5 (0.4 to 0.8)	98.6 (41.2 to 201.4)	-24.7 (-47.8 to 16.5)	136.2 (56.4 to 285.3)	-24.1 (-51 to 25.9)
		Female	101 (58 to 182)	12.3 (6.9 to 23)	3 (2 to 7)	0.6 (0.3 to 1.3)	212 (155 to 348)	9.8 (7.2 to 15.8)	9 (6 to 15)	0.5 (0.4 to 0.8)	110.6 (37.2 to 233.3)	-20.7 (-52 to 32.8)	166.6 (48.5 to 384.9)	-17 (-56.1 to 61.6)

Province	Cause	Sex	1990				2019				1990-2019 percent change (%)			
			DALYs		Death		DALYs		Death		DALYs		Death	
			Number	Rate	Number	Rate	Number	Rate	Number	Rate	Number	Rate	Number	Rate
		Male	185 (106 to 329)	18.8 (11.2 to 31.4)	5 (3 to 9)	0.7 (0.5 to 1.2)	355 (236 to 632)	14 (9.7 to 23.5)	12 (8 to 19)	0.5 (0.4 to 0.9)	92.1 (20.8 to 225.4)	-25.5 (-53.6 to 23.1)	116.9 (31.2 to 282.6)	-26.5 (-55.2 to 21.3)
	Other digestive diseases	Both	257 (195 to 420)	11.6 (8.5 to 17.3)	5 (4 to 9)	0.5 (0.3 to 0.8)	766 (604 to 952)	17 (13.7 to 21)	26 (21 to 32)	0.7 (0.6 to 0.9)	197.5 (53.9 to 316.8)	46.8 (-12 to 98.5)	378.4 (163.2 to 627.8)	49.7 (-14.2 to 140.4)
		Female	115 (83 to 191)	10.5 (7.1 to 16.9)	2 (1 to 4)	0.4 (0.2 to 0.8)	321 (236 to 421)	14.7 (11 to 19.1)	10 (8 to 14)	0.6 (0.4 to 0.8)	179.4 (44.7 to 305.1)	39.3 (-14.5 to 100.6)	346.2 (132.8 to 720)	37.3 (-29.9 to 192.4)
		Male	143 (101 to 238)	12.5 (9 to 19.5)	3 (2 to 5)	0.5 (0.3 to 0.8)	445 (327 to 579)	19.3 (14.3 to 24.8)	16 (11 to 20)	0.8 (0.6 to 1)	212 (67.7 to 387.8)	54.2 (-12.4 to 136.1)	402.6 (169 to 754.2)	60.7 (-10.1 to 161.9)
	Neurological disorders	Both	38284 (18095 to 67243)	1465.7 (786.9 to 2426.5)	270 (143 to 544)	32 (12.7 to 75.8)	69447 (35606 to 120226)	1451.5 (777.9 to 2442)	1077 (461 to 2467)	32.9 (13.2 to 76.7)	81.4 (49.4 to 129.5)	-1 (-14.3 to 16.4)	299.6 (184 to 444.1)	2.7 (-13.7 to 39.6)
		Female	21113 (9159 to 39319)	1626.2 (813.6 to 2879.9)	136 (64 to 301)	31.1 (11.6 to 80.1)	39340 (17784 to 71836)	1639.2 (804.8 to 2903.4)	510 (196 to 1160)	32.6 (11.7 to 76.9)	86.3 (56.1 to 135.2)	0.8 (-12.2 to 18.5)	275.3 (160 to 432.7)	4.7 (-16.3 to 49.5)
		Male	17171 (8978 to 28276)	1314.6 (782.7 to 2070.4)	134 (73 to 257)	33.1 (13.2 to 74.3)	30106 (17325 to 48766)	1270.8 (748.6 to 2056.1)	568 (255 to 1291)	33.2 (13.9 to 77.9)	75.3 (38.4 to 136.8)	-3.3 (-19.9 to 16.9)	324.2 (184.1 to 505.1)	0.3 (-21.9 to 37.4)
	Alzheimer's disease and other dementias	Both	3193 (1383 to 7300)	384.5 (166.7 to 871.4)	160 (37 to 437)	25.4 (6 to 69.9)	13425 (5844 to 30324)	391.1 (170.4 to 879.6)	804 (197 to 2193)	25.8 (6.2 to 69.7)	320.4 (257.6 to 433.9)	1.7 (-11.2 to 28.2)	403.7 (310.4 to 616)	1.4 (-14.3 to 43.2)
		Female	1713 (729 to 3916)	393.2 (171 to 914)	89 (20 to 255)	25.8 (6 to 74.7)	6865 (2967 to 15235)	409.4 (176.2 to 894.3)	401 (95 to 1067)	26.9 (6.3 to 72.2)	300.7 (230.9 to 430.2)	4.1 (-11.8 to 37)	348.9 (256.5 to 583)	4.3 (-16.3 to 56.4)
		Male	1480 (632 to 3468)	374.8 (162.9 to 840.2)	70 (16 to 192)	25 (5.7 to 65.7)	6560 (2844 to 15232)	373.5 (164.1 to 864.5)	402 (95 to 1114)	24.8 (6 to 69.6)	343.3 (256.6 to 476.2)	-0.4 (-16.7 to 24.6)	473.6 (333.9 to 724)	-0.8 (-20.9 to 37.4)
	Parkinson's disease	Both	792 (646 to 958)	70.4 (58.1 to 87)	36 (28 to 44)	4.2 (3.4 to 5.5)	2962 (2517 to 3443)	79.2 (67.5 to 91.9)	173 (145 to 203)	5 (4.2 to 5.9)	274 (193.9 to 388.1)	12.5 (-12.6 to 44.6)	386 (270.2 to 554.3)	20.2 (-10.1 to 58.6)
		Female	306 (238 to 398)	54.5 (42.3 to 77.9)	14 (10 to 21)	3.2 (2.4 to 5.3)	1159 (963 to 1397)	62.4 (51.5 to 75.1)	65 (52 to 83)	3.9 (3.1 to 5)	279.1 (186.8 to 413.2)	14.4 (-14.1 to 54)	362.4 (240 to 554.7)	20.9 (-11.9 to 69.6)
		Male	486 (364 to 614)	89.3 (68.4 to 111)	21 (16 to 27)	5.4 (4.1 to 6.9)	1803 (1426 to 2191)	95.6 (75.6 to 115.7)	108 (83 to 131)	6.1 (4.8 to 7.5)	270.8 (167.5 to 445.7)	7.1 (-23.2 to 52.9)	401.5 (253.2 to 655.6)	12.3 (-21.8 to 62.7)
	Idiopathic epilepsy	Both	8709 (4743 to 13813)	205.7 (115.4 to 327.6)	58 (40 to 72)	1.6 (1.2 to 2)	7437 (3605 to 13195)	153.7 (73 to 273.3)	49 (41 to 61)	1 (0.8 to 1.2)	-14.6 (-60 to 88.8)	-25.3 (-66 to 65.1)	-14.5 (-37.8 to 31.9)	-38.2 (-53.7 to -7)
		Female	3912 (2037 to 6287)	186.4 (98.6 to 302.2)	25 (14 to 33)	1.4 (0.9 to 1.9)	3171 (1471 to 5753)	135.3 (62.7 to 244.2)	19 (15 to 25)	0.8 (0.6 to 1)	-19 (-62.5 to 89.8)	-27.4 (-67.4 to 68.4)	-25 (-49.9 to 49.1)	-43.4 (-60.3 to -1.9)
		Male	4796 (2571 to 7551)	224.3 (119.7 to 353.8)	32 (21 to 42)	1.8 (1.3 to 2.3)	4266 (2128 to 7417)	171.4 (84.2 to 300.4)	30 (24 to 38)	1.2 (0.9 to 1.5)	-11 (-58.3 to 95)	-23.6 (-65.1 to 70.5)	-6.4 (-34.5 to 57.6)	-34.4 (-53.5 to 3.7)
	Multiple sclerosis	Both	778 (581 to 1048)	33.3 (24.7 to 45.6)	9 (6 to 14)	0.4 (0.3 to 0.7)	2263 (1837 to 2794)	39.7 (32.4 to 49)	30 (23 to 41)	0.6 (0.4 to 0.8)	191.1 (121.1 to 273.3)	19.1 (-12.5 to 54.5)	236.7 (94.2 to 462.5)	25.9 (-29.3 to 119)
		Female	478 (348 to 642)	41.5 (30.5 to 55.2)	4 (2 to 7)	0.5 (0.2 to 0.7)	1473 (1109 to 1844)	52.4 (39.7 to 65.4)	16 (10 to 22)	0.6 (0.4 to 0.8)	208.4 (115.3 to 315.1)	26.3 (-11.4 to 70.1)	267.5 (83.1 to 664.7)	36.4 (-32.4 to 192.9)
		Male	300 (202 to 495)	25.4 (16.8 to 43.2)	4 (2 to 9)	0.4 (0.2 to 0.9)	791 (570 to 1208)	27.3 (19.9 to 41)	13 (9 to 24)	0.5 (0.3 to 0.9)	163.6 (66.6 to 273.2)	7.6 (-34.4 to 53.8)	205.5 (51.6 to 451.9)	16.7 (-42.3 to 113.4)

Province	Cause	Sex	1990				2019				1990-2019 percent change (%)			
			DALYs		Death		DALYs		Death		DALYs		Death	
			Number	Rate	Number	Rate	Number	Rate	Number	Rate	Number	Rate	Number	Rate
	Motor neuron disease	Both	55 (43 to 69)	2.1 (1.6 to 2.7)	1 (1 to 1)	0 (0 to 0.1)	122 (100 to 147)	2.4 (2 to 2.8)	3 (2 to 3)	0.1 (0 to 0.1)	121.2 (58.6 to 201.5)	14.1 (-19.7 to 60.2)	168.6 (70.8 to 328.3)	16 (-28.1 to 87)
		Female	28 (22 to 37)	1.9 (1.4 to 2.7)	0 (0 to 1)	0 (0 to 0.1)	54 (44 to 65)	2.1 (1.7 to 2.6)	1 (1 to 1)	0 (0 to 0.1)	90.9 (33.3 to 158.7)	11.4 (-27.2 to 55.1)	145.8 (41.5 to 285)	16 (-38.8 to 89.2)
		Male	27 (19 to 36)	2.2 (1.5 to 2.9)	1 (0 to 1)	0.1 (0 to 0.1)	68 (53 to 86)	2.6 (2 to 3.3)	2 (1 to 2)	0.1 (0 to 0.1)	153.3 (64.5 to 290.7)	18.6 (-25.6 to 90.3)	186.1 (56.1 to 426.1)	19.1 (-34.1 to 119.5)
	Headache disorders	Both	23295 (4697 to 51914)	733 (167.7 to 1600.2)	--	--	40958 (9838 to 88371)	737.2 (173.3 to 1605.1)	--	--	75.8 (59.8 to 114)	0.6 (-4.3 to 6.7)	--	--
		Female	14049 (2570 to 32082)	916.1 (187.1 to 2054.1)	--	--	25609 (5439 to 57782)	934.2 (194.1 to 2131.2)	--	--	82.3 (66.6 to 114.5)	2 (-4.7 to 8.2)	--	--
		Male	9246 (2070 to 20123)	557.9 (149.9 to 1166.5)	--	--	15349 (4336 to 31830)	547.4 (150.4 to 1151.2)	--	--	66 (47 to 117.1)	-1.9 (-8.5 to 7.9)	--	--
	Other neurological disorders	Both	1462 (710 to 2475)	36.7 (20.4 to 58.8)	7 (5 to 9)	0.3 (0.2 to 0.4)	2279 (1364 to 3614)	48.3 (27.9 to 78.7)	19 (16 to 22)	0.4 (0.3 to 0.5)	55.9 (-19.2 to 246.3)	31.6 (-29.1 to 169.5)	171.8 (93.5 to 312.6)	53.6 (9 to 143.3)
		Female	626 (277 to 1101)	32.5 (17.2 to 52.6)	2 (2 to 3)	0.2 (0.1 to 0.3)	1010 (601 to 1646)	43.4 (24.9 to 71.5)	7 (6 to 9)	0.3 (0.3 to 0.4)	61.4 (-16.8 to 273.9)	33.6 (-27.5 to 167.4)	207.4 (103.8 to 402.4)	60.4 (1.7 to 182.9)
		Male	836 (426 to 1404)	40.7 (23.1 to 64.8)	5 (3 to 6)	0.3 (0.2 to 0.5)	1269 (752 to 2032)	53 (30.4 to 86.2)	12 (10 to 15)	0.5 (0.4 to 0.6)	51.7 (-21.7 to 235.6)	30.1 (-31.8 to 164.7)	154.7 (70.2 to 317)	50 (0.6 to 143)
	Mental disorders	Both	81164 (59344 to 107752)	2526.9 (1861.2 to 3345.2)	0 (0 to 0)	0 (0 to 0)	139348 (101741 to 184494)	2546.6 (1868.8 to 3392.9)	0 (0 to 0)	0 (0 to 0)	71.7 (62.1 to 81.2)	0.8 (-1.9 to 3.4)	1666.9 (473.8 to 4360)	758.9 (184.7 to 2151.2)
		Female	42138 (30638 to 56029)	2707 (1984.8 to 3570.1)	0 (0 to 0)	0 (0 to 0)	73225 (53732 to 96881)	2717 (1991.5 to 3596.2)	0 (0 to 0)	0 (0 to 0)	73.8 (63.4 to 84.5)	0.4 (-3.2 to 4.1)	1770.2 (440.5 to 5041.7)	815.1 (164.6 to 2385.8)
		Male	39026 (28508 to 51807)	2354.5 (1725.1 to 3095.4)	0 (0 to 0)	0 (0 to 0)	66123 (48223 to 87303)	2382.9 (1742.8 to 3156.2)	0 (0 to 0)	0 (0 to 0)	69.4 (59.1 to 79.9)	1.2 (-2.5 to 5)	836.1 (188.8 to 2491.3)	387.1 (45.8 to 1354.1)
	Schizophrenia	Both	4246 (3057 to 5471)	159.2 (115.1 to 202.7)	--	--	9681 (7076 to 12462)	160.7 (119 to 205.4)	--	--	128 (106.8 to 150.9)	0.9 (-7 to 9.5)	--	--
		Female	1967 (1400 to 2548)	151.4 (108.4 to 194.1)	--	--	4512 (3263 to 5836)	152.5 (111.3 to 195.9)	--	--	129.3 (98.8 to 163.2)	0.7 (-11.4 to 14.1)	--	--
		Male	2279 (1606 to 2920)	166.6 (119 to 213.2)	--	--	5169 (3707 to 6716)	168.6 (122.7 to 217.1)	--	--	126.8 (101 to 157.8)	1.2 (-9.3 to 14)	--	--
	Depressive disorders	Both	33261 (22349 to 46482)	1130.7 (771.4 to 1563.6)	--	--	64678 (43744 to 90922)	1154.5 (782.6 to 1596.5)	--	--	94.5 (77.8 to 112.3)	2.1 (-2.2 to 6.9)	--	--
		Female	17801 (11872 to 24958)	1227.3 (834.4 to 1702.1)	--	--	33728 (22742 to 47887)	1227.3 (830.9 to 1721.6)	--	--	89.5 (71.3 to 109.3)	0 (-5.2 to 5.9)	--	--
		Male	15459 (10378 to 21766)	1038.8 (708.3 to 1451.1)	--	--	30950 (21127 to 43411)	1084.4 (745 to 1511.6)	--	--	100.2 (82 to 121)	4.4 (-1.8 to 11.7)	--	--
	Bipolar disorder	Both	5177 (3153 to 7895)	171.3 (105 to 260.6)	--	--	9707 (5889 to 14797)	173.7 (106 to 266)	--	--	87.5 (70.4 to 106.7)	1.4 (-5.1 to 8.4)	--	--
		Female	2778 (1675 to 4203)	185.4 (114.4 to 280.5)	--	--	5133 (3132 to 7798)	187.8 (113.6 to 285.1)	--	--	84.8 (63.2 to 108.5)	1.3 (-8 to 12)	--	--
		Male	2399 (1448 to 3656)	157.7 (97.8 to 240.6)	--	--	4574 (2787 to 7164)	160.3 (98.7 to 250.3)	--	--	90.6 (69.1 to 116.4)	1.6 (-7.5 to 11.8)	--	--

Province	Cause	Sex	1990				2019				1990-2019 percent change (%)			
			DALYs		Death		DALYs		Death		DALYs		Death	
			Number	Rate	Number	Rate	Number	Rate	Number	Rate	Number	Rate	Number	Rate
	Anxiety disorders	Both	23045 (16047 to 31658)	672 (468.7 to 921.4)	--	--	36893 (25757 to 50918)	694.5 (486.2 to 957.6)	--	--	60.1 (44.9 to 76.2)	3.3 (-2.5 to 9.5)	--	--
		Female	13651 (9454 to 18956)	825.9 (575.5 to 1132.2)	--	--	22413 (15767 to 30696)	853.7 (602 to 1164.2)	--	--	64.2 (46.8 to 84.1)	3.4 (-4.6 to 11.3)	--	--
		Male	9395 (6467 to 12946)	524.1 (367.5 to 720.1)	--	--	14480 (9977 to 20261)	541.5 (373 to 757.2)	--	--	54.1 (35.7 to 73.9)	3.3 (-5.8 to 12.6)	--	--
	Eating disorders	Both	1541 (943 to 2293)	41.2 (25.3 to 61.4)	0 (0 to 0)	0 (0 to 0)	3086 (1897 to 4641)	54.3 (33.7 to 81)	0 (0 to 0)	0 (0 to 0)	100.3 (71.2 to 134.9)	31.7 (17.3 to 47.2)	1666.9 (473.8 to 4360)	758.9 (184.7 to 2151.2)
		Female	942 (580 to 1411)	51.4 (31.6 to 76.2)	0 (0 to 0)	0 (0 to 0)	1869 (1152 to 2761)	68.2 (42.1 to 103.5)	0 (0 to 0)	0 (0 to 0)	98.5 (65.6 to 138.7)	32.7 (15.3 to 52.8)	1770.2 (440.5 to 5041.7)	815.1 (164.6 to 2385.8)
		Male	599 (360 to 915)	31.4 (19 to 48.5)	0 (0 to 0)	0 (0 to 0)	1217 (707 to 1881)	41.1 (24.3 to 62.5)	0 (0 to 0)	0 (0 to 0)	103.1 (63.7 to 152.5)	30.9 (10.9 to 55.3)	836.1 (188.8 to 2491.3)	387.1 (45.8 to 1354.1)
	Autism spectrum disorders	Both	1789 (1167 to 2638)	44.4 (29 to 65.2)	--	--	2237 (1449 to 3262)	44.8 (29.4 to 65.3)	--	--	25.1 (12.4 to 39)	1 (-8.7 to 11.8)	--	--
		Female	439 (280 to 656)	22.3 (14.2 to 33.1)	--	--	549 (351 to 806)	22.5 (14.5 to 32.9)	--	--	25.1 (5.5 to 48.9)	0.9 (-13.7 to 18.7)	--	--
		Male	1350 (878 to 1980)	65.6 (42.6 to 96.1)	--	--	1688 (1093 to 2454)	66.2 (43.1 to 96)	--	--	25 (10.1 to 40.7)	1.1 (-10.7 to 13.5)	--	--
	Attention-deficit/hyperactivity disorder	Both	1478 (822 to 2571)	32.5 (18.3 to 55.3)	--	--	1624 (916 to 2737)	33.6 (19.2 to 56.8)	--	--	9.9 (-6.5 to 29.4)	3.5 (-10 to 17.4)	--	--
		Female	443 (246 to 768)	20.1 (11.2 to 34.6)	--	--	491 (273 to 820)	20.8 (11.5 to 34.9)	--	--	10.8 (-8 to 36.5)	3.7 (-13.6 to 25.1)	--	--
		Male	1035 (573 to 1765)	44.4 (24.9 to 74.9)	--	--	1132 (640 to 1909)	45.8 (26.1 to 77.6)	--	--	9.4 (-10 to 35.4)	3.1 (-13.6 to 22.3)	--	--
	Conduct disorder	Both	4145 (2361 to 6506)	75.6 (42.8 to 118.7)	--	--	2925 (1680 to 4651)	74.4 (42.8 to 118)	--	--	-29.4 (-36.6 to -22.8)	-1.5 (-11.4 to 7.6)	--	--
		Female	1414 (755 to 2313)	52.6 (28 to 86.4)	--	--	973 (517 to 1601)	50.9 (27 to 83.7)	--	--	-31.2 (-42.1 to -20.8)	-3.3 (-18.2 to 11.7)	--	--
		Male	2731 (1570 to 4270)	97.6 (55.9 to 151.8)	--	--	1952 (1118 to 3069)	96.7 (55.4 to 152.4)	--	--	-28.5 (-36.3 to -20.2)	-0.8 (-11.6 to 10.4)	--	--
	Idiopathic developmental intellectual disability	Both	4058 (2244 to 6485)	99.6 (55.6 to 158.1)	--	--	2789 (1410 to 4707)	55.5 (28 to 93.8)	--	--	-31.3 (-41.7 to -23.5)	-44.2 (-53.4 to -37.8)	--	--
		Female	1745 (961 to 2818)	88.1 (48.3 to 142.3)	--	--	1246 (641 to 2130)	50.7 (26 to 86.9)	--	--	-28.6 (-39.9 to -19.8)	-42.4 (-51.7 to -35.6)	--	--
		Male	2312 (1274 to 3676)	110.6 (61.8 to 174.1)	--	--	1543 (765 to 2600)	60.2 (29.9 to 100.9)	--	--	-33.3 (-45 to -25.1)	-45.6 (-55.7 to -38.8)	--	--
	Other mental disorders	Both	2424 (1537 to 3687)	100.4 (65.2 to 151.9)	--	--	5728 (3655 to 8727)	100.6 (64.7 to 152.8)	--	--	136.3 (118.3 to 155.7)	0.1 (-5.2 to 5.7)	--	--
		Female	958 (601 to 1468)	82.5 (52.8 to 126.7)	--	--	2311 (1465 to 3541)	82.6 (52.7 to 126.4)	--	--	141.3 (116.6 to 170.2)	0.1 (-7.7 to 9.1)	--	--
		Male	1466 (936 to 2219)	117.7 (75.2 to 178.9)	--	--	3417 (2180 to 5194)	118 (75.6 to 178.6)	--	--	133 (112.4 to 156.4)	0.3 (-6.6 to 7.8)	--	--

Province	Cause	Sex	1990				2019				1990-2019 percent change (%)			
			DALYs		Death		DALYs		Death		DALYs		Death	
			Number	Rate	Number	Rate	Number	Rate	Number	Rate	Number	Rate	Number	Rate
	Substance use disorders	Both	10943 (8450 to 13569)	365.6 (286.3 to 450.2)	97 (68 to 127)	4 (2.9 to 5.1)	27373 (22006 to 33212)	464 (371.8 to 559.1)	261 (212 to 318)	4.6 (3.8 to 5.5)	150.1 (108.6 to 206.2)	26.9 (5.4 to 54)	170 (86.9 to 302.9)	14.7 (-18.4 to 67.9)
		Female	3149 (2276 to 4122)	211.8 (156.8 to 272.4)	25 (14 to 36)	2.2 (1.4 to 3.1)	6582 (4978 to 8292)	231.9 (175.6 to 292.3)	46 (35 to 60)	1.8 (1.4 to 2.3)	109 (66.8 to 171)	9.5 (-11.2 to 39.1)	86.4 (18.5 to 235.4)	-19.2 (-46 to 33)
		Male	7795 (5988 to 9757)	513.8 (398.4 to 643.4)	72 (48 to 98)	5.7 (3.9 to 7.8)	20791 (16651 to 25152)	686.3 (549.5 to 826.3)	214 (166 to 266)	7.3 (5.7 to 9)	166.7 (114.2 to 241.5)	33.6 (6.7 to 70.4)	199.1 (97.6 to 363.8)	27.4 (-15.9 to 96.1)
	Alcohol use disorders	Both	1816 (1302 to 2412)	63.5 (46.4 to 83.2)	11 (7 to 15)	0.5 (0.3 to 0.6)	2810 (2116 to 3657)	47.5 (36.2 to 61.1)	22 (17 to 27)	0.4 (0.3 to 0.5)	54.7 (33 to 83.5)	-25.2 (-36.6 to -11.8)	95.1 (37.1 to 208.6)	-18.4 (-42.5 to 26.7)
		Female	440 (316 to 594)	30.3 (22.4 to 40.3)	2 (1 to 3)	0.2 (0.1 to 0.3)	764 (541 to 1029)	26.9 (19.3 to 35.9)	4 (3 to 5)	0.2 (0.1 to 0.2)	73.5 (41.9 to 117)	-11.2 (-25.8 to 7.7)	48.7 (1 to 168.5)	-32 (-52 to 7.2)
		Male	1376 (978 to 1845)	95.4 (68.8 to 124.8)	9 (5 to 12)	0.7 (0.5 to 1)	2046 (1535 to 2641)	67.3 (50.6 to 85.9)	18 (13 to 23)	0.6 (0.5 to 0.8)	48.7 (23 to 82.9)	-29.4 (-41.7 to -12.5)	108.5 (34.8 to 255.1)	-13.7 (-43.8 to 47.6)
	Drug use disorders	Both	9127 (6873 to 11422)	302.1 (233 to 373.5)	85 (61 to 112)	3.5 (2.6 to 4.5)	24563 (19512 to 29900)	416.5 (331.2 to 504.7)	239 (193 to 291)	4.2 (3.4 to 5.1)	169.1 (121 to 236.1)	37.9 (12.5 to 70.2)	179.8 (93.2 to 335.7)	19.3 (-16.7 to 74)
		Female	2708 (1933 to 3599)	181.5 (132 to 235.8)	22 (13 to 33)	2 (1.3 to 2.8)	5818 (4358 to 7483)	205 (153.4 to 264.6)	43 (31 to 56)	1.6 (1.2 to 2.1)	114.8 (68 to 186.2)	12.9 (-10.6 to 46.8)	90.5 (19.6 to 248.6)	-17.7 (-46.2 to 35.3)
		Male	6419 (4834 to 8220)	418.4 (319.5 to 528)	63 (42 to 88)	5 (3.4 to 6.9)	18745 (14870 to 22829)	618.9 (493.4 to 751.9)	196 (152 to 248)	6.6 (5.2 to 8.2)	192 (127.2 to 282.3)	47.9 (15.3 to 92.3)	211.6 (103.1 to 400.4)	33.5 (-13 to 113.3)
	Diabetes and kidney diseases	Both	18434 (15426 to 21449)	914.5 (771.6 to 1074.1)	362 (298 to 442)	27.4 (22.8 to 33.9)	60376 (49829 to 71527)	1314.4 (1090.3 to 1545.8)	1372 (1170 to 1579)	36 (30.8 to 41.1)	227.5 (179 to 288.2)	43.7 (22.4 to 67.4)	278.7 (195.1 to 381.8)	31 (3.4 to 66)
		Female	8602 (6943 to 10433)	870.9 (708.7 to 1070.8)	163 (127 to 215)	25.2 (19.6 to 36.2)	29907 (24275 to 35899)	1319.2 (1083 to 1574.6)	661 (542 to 789)	35.7 (29.5 to 42.5)	247.7 (186.3 to 324.8)	51.5 (22.5 to 82.1)	306.8 (193 to 461.2)	41.8 (-2.2 to 95.1)
		Male	9831 (7844 to 11907)	955.9 (771.7 to 1155.4)	200 (152 to 251)	29.8 (22.8 to 37.1)	30469 (25020 to 36462)	1309.8 (1087 to 1568.2)	711 (575 to 862)	36.2 (29.5 to 43.7)	209.9 (149.6 to 291.4)	37 (10.9 to 71.9)	255.8 (156.2 to 414)	21.6 (-10.7 to 71.5)
	Diabetes mellitus	Both	6447 (5093 to 8141)	358.9 (282.9 to 456.6)	92 (73 to 126)	6.9 (5.4 to 10.7)	38926 (30972 to 48626)	833.7 (665.2 to 1036)	658 (556 to 767)	16.6 (14.1 to 19.3)	503.7 (410.9 to 589.5)	132.3 (93.7 to 166.7)	614.4 (391.3 to 862.3)	142.4 (54.7 to 222.2)
		Female	3050 (2342 to 4004)	353.3 (273.2 to 465.7)	44 (31 to 78)	6.8 (4.7 to 13.4)	19728 (15362 to 24967)	855 (674.4 to 1073.1)	329 (263 to 401)	17.1 (13.9 to 20.7)	546.8 (389.9 to 672.6)	142 (74.4 to 188.4)	649.4 (279.4 to 1063.1)	151.6 (20.7 to 290)
		Male	3397 (2658 to 4270)	363.3 (285.9 to 452.8)	48 (35 to 63)	6.9 (5.2 to 8.7)	19198 (14938 to 23951)	812.6 (644 to 1016.3)	329 (258 to 408)	16.2 (12.8 to 20)	465.1 (381.3 to 577.3)	123.7 (90.9 to 167.7)	582.4 (374 to 912.5)	136.2 (67.6 to 243.3)
	Chronic kidney disease	Both	11965 (10127 to 14069)	555.1 (469.9 to 646)	270 (223 to 323)	20.6 (17 to 25.3)	21138 (18257 to 24209)	474.4 (411.1 to 541)	705 (597 to 810)	19.1 (16.1 to 22)	76.7 (47 to 114.6)	-14.5 (-28.9 to 2.1)	161.3 (104.3 to 233.1)	-7.1 (-28.5 to 18)
		Female	5546 (4503 to 6808)	517.3 (424.4 to 636.1)	118 (93 to 154)	18.4 (14.1 to 25.5)	10085 (8488 to 11871)	460 (388.6 to 540.1)	329 (266 to 400)	18.5 (15.1 to 22.2)	81.8 (44.7 to 134.4)	-11.1 (-29.8 to 12.5)	177.5 (100.4 to 280.2)	0.4 (-32.2 to 38.2)
		Male	6419 (5064 to 7791)	591.9 (467.5 to 730.3)	152 (114 to 192)	22.9 (17.4 to 28.9)	11053 (9271 to 13086)	488.9 (412.2 to 578.2)	377 (301 to 459)	19.7 (15.8 to 24)	72.2 (33.2 to 130.5)	-17.4 (-35.5 to 11.6)	148.6 (78.5 to 267.3)	-13.8 (-36.9 to 23.3)

Province	Cause	Sex	1990				2019				1990-2019 percent change (%)			
			DALYs		Death		DALYs		Death		DALYs		Death	
			Number	Rate	Number	Rate	Number	Rate	Number	Rate	Number	Rate	Number	Rate
	Acute glomerulonephritis	Both	21 (5 to 274)	0.5 (0.2 to 5.8)	0 (0 to 4)	0 (0 to 0.1)	312 (96 to 446)	6.3 (2.3 to 8.6)	9 (3 to 12)	0.2 (0.1 to 0.3)	1384 (-63.3 to 8327.6)	1107.6 (-62.3 to 4996.7)	2572.6 (-30.2 to 13073.7)	1414.1 (-52.1 to 5627.3)
		Female	6 (2 to 74)	0.3 (0.1 to 3.5)	0 (0 to 1)	0 (0 to 0.1)	94 (44 to 142)	4.2 (2.2 to 6.2)	3 (1 to 4)	0.2 (0.1 to 0.2)	1553.6 (-46.9 to 6899)	1268.9 (-48 to 4874.1)	3096.5 (5.2 to 13782.7)	1695.2 (-36.7 to 6492.2)
		Male	15 (2 to 186)	0.7 (0.2 to 8)	0 (0 to 3)	0 (0 to 0.2)	218 (37 to 353)	8.3 (1.7 to 12.8)	6 (1 to 9)	0.3 (0.1 to 0.4)	1321 (-78.3 to 11778)	1038.3 (-76 to 6928.8)	2370.7 (-60.6 to 17709.1)	1247.7 (-72.1 to 8036.3)
	Skin and subcutaneous diseases	Both	15848 (10499 to 23039)	410.2 (277.3 to 604.1)	3 (2 to 4)	0.3 (0.2 to 0.4)	20374 (13470 to 30441)	420.1 (279.8 to 627.7)	10 (8 to 13)	0.3 (0.2 to 0.4)	28.6 (22.8 to 33.6)	2.4 (-0.7 to 5.2)	267.5 (150.6 to 452.7)	11.9 (-25.9 to 78.9)
		Female	8249 (5494 to 11955)	434.8 (294.7 to 637.7)	1 (1 to 2)	0.2 (0.1 to 0.4)	10561 (7019 to 15797)	443.2 (295.7 to 663.2)	4 (3 to 5)	0.2 (0.2 to 0.3)	28 (21.3 to 34.2)	1.9 (-1.9 to 5.4)	226 (73.2 to 467.6)	0.7 (-52.2 to 97.9)
		Male	7599 (5007 to 11041)	386.6 (257.4 to 565.1)	2 (1 to 3)	0.3 (0.2 to 0.5)	9813 (6502 to 14598)	397.9 (264.5 to 593.4)	6 (5 to 8)	0.3 (0.3 to 0.5)	29.1 (22.9 to 35)	2.9 (-0.8 to 6.6)	302.4 (153.6 to 567.1)	20.6 (-27.4 to 114.6)
	Dermatitis	Both	3665 (2141 to 5821)	93.3 (56.4 to 144.1)	--	--	4706 (2853 to 7187)	93.9 (56.6 to 144.6)	--	--	28.4 (17.5 to 41.1)	0.6 (-4.3 to 5.6)	--	--
		Female	1991 (1154 to 3181)	103.6 (62.1 to 161.2)	--	--	2568 (1544 to 3954)	104.5 (62.2 to 160.9)	--	--	29 (16.9 to 43.4)	0.8 (-5.7 to 8)	--	--
		Male	1673 (988 to 2680)	83.4 (50.9 to 129.5)	--	--	2138 (1289 to 3288)	83.8 (50.7 to 129.8)	--	--	27.8 (15.5 to 42.8)	0.4 (-5.9 to 8)	--	--
	Psoriasis	Both	1329 (929 to 1795)	44.7 (31.9 to 59.8)	--	--	1718 (1213 to 2315)	31.8 (22.4 to 42.7)	--	--	29.3 (15.4 to 46)	-28.7 (-35.6 to -20.9)	--	--
		Female	686 (478 to 919)	46.5 (32.7 to 61.9)	--	--	875 (610 to 1179)	33.1 (23.3 to 44.4)	--	--	27.6 (8.4 to 50.8)	-28.8 (-38 to -18.2)	--	--
		Male	643 (437 to 868)	42.9 (29.4 to 57.4)	--	--	843 (581 to 1150)	30.6 (21.3 to 41.8)	--	--	31.1 (10.3 to 55.4)	-28.6 (-38.6 to -16.8)	--	--
	Bacterial skin diseases	Both	239 (158 to 377)	7.9 (5.6 to 11.5)	2 (2 to 3)	0.2 (0.1 to 0.3)	353 (249 to 530)	7.4 (5.3 to 11)	6 (5 to 9)	0.2 (0.1 to 0.2)	47.3 (30.7 to 76.7)	-6.9 (-21.7 to 14.3)	155.3 (81.7 to 302.6)	-19.1 (-44.8 to 40.7)
		Female	105 (68 to 173)	7.1 (4.6 to 10.9)	1 (1 to 2)	0.2 (0.1 to 0.3)	141 (93 to 226)	6.1 (4.1 to 9.7)	2 (2 to 3)	0.1 (0.1 to 0.2)	34.3 (15.9 to 66.4)	-14.6 (-33.9 to 18.7)	100.2 (20 to 346.1)	-37.3 (-65.9 to 78)
		Male	134 (88 to 209)	8.8 (5.9 to 12.9)	1 (1 to 2)	0.2 (0.1 to 0.4)	211 (147 to 311)	8.7 (6.2 to 12.7)	4 (3 to 6)	0.2 (0.2 to 0.3)	57.5 (32.2 to 99.6)	-0.8 (-21.1 to 28.3)	198.2 (97.6 to 381.2)	-6.4 (-40.3 to 65.2)
	Scabies	Both	1009 (547 to 1623)	25.1 (13.9 to 40.3)	--	--	1236 (677 to 1972)	25.1 (13.7 to 40.2)	--	--	22.5 (12.8 to 34)	-0.2 (-4.9 to 4.8)	--	--
		Female	496 (261 to 801)	25.2 (13.7 to 40.7)	--	--	605 (333 to 973)	25.1 (13.8 to 40.4)	--	--	22.1 (10.9 to 34.7)	-0.1 (-6.7 to 6.6)	--	--
		Male	513 (282 to 821)	25 (13.8 to 40.2)	--	--	631 (345 to 1010)	25 (13.7 to 40.4)	--	--	22.9 (11.7 to 36.8)	-0.2 (-6.5 to 6.9)	--	--
	Fungal skin diseases	Both	492 (195 to 1052)	18.7 (7.6 to 39.7)	--	--	892 (360 to 1916)	18.5 (7.5 to 39.3)	--	--	81.4 (69 to 93.9)	-1.3 (-3.1 to 0.6)	--	--
		Female	222 (88 to 475)	17.5 (7 to 38.1)	--	--	411 (166 to 888)	17.3 (7 to 37.1)	--	--	85.1 (72.2 to 98.3)	-1.4 (-3.8 to 1.1)	--	--
		Male	270 (106 to 583)	19.9 (8 to 42.3)	--	--	481 (193 to 1040)	19.6 (7.9 to 42.2)	--	--	78.3 (64.6 to 91.6)	-1.4 (-3.8 to 1.1)	--	--

Province	Cause	Sex	1990				2019				1990-2019 percent change (%)			
			DALYs		Death		DALYs		Death		DALYs		Death	
			Number	Rate	Number	Rate	Number	Rate	Number	Rate	Number	Rate	Number	Rate
	Viral skin diseases	Both	2537 (1605 to 3804)	51.3 (32.6 to 76.6)	--	--	2327 (1490 to 3493)	51.4 (33 to 77)	--	--	-8.3 (-12.4 to -3.9)	0.2 (-3.9 to 4.2)	--	--
		Female	1230 (782 to 1865)	50.9 (32.7 to 76.9)	--	--	1129 (727 to 1710)	51 (32.7 to 77.1)	--	--	-8.2 (-14 to -2)	0.2 (-5.6 to 6.3)	--	--
		Male	1307 (817 to 1944)	51.6 (32.5 to 76.8)	--	--	1197 (759 to 1792)	51.7 (33 to 77.4)	--	--	-8.4 (-14.1 to -2.3)	0.2 (-5.8 to 5.7)	--	--
	Acne vulgaris	Both	2394 (1420 to 3805)	52.1 (31.1 to 83.3)	--	--	3128 (1897 to 4891)	71.1 (42.9 to 111.4)	--	--	30.6 (20.8 to 42)	36.5 (26.3 to 48)	--	--
		Female	1137 (672 to 1805)	50.7 (30 to 80.7)	--	--	1489 (885 to 2363)	69 (41.2 to 110.5)	--	--	31 (16.7 to 46)	36.1 (21.4 to 51)	--	--
		Male	1258 (742 to 2023)	53.4 (31.3 to 86.3)	--	--	1639 (995 to 2586)	73.1 (44.3 to 115.5)	--	--	30.3 (17.4 to 46.1)	36.7 (24.4 to 52.5)	--	--
	Alopecia areata	Both	204 (128 to 307)	6.3 (4 to 9.4)	--	--	358 (229 to 534)	6.3 (4 to 9.5)	--	--	75.9 (57.8 to 98.3)	0.4 (-9.2 to 11.8)	--	--
		Female	130 (81 to 197)	8.3 (5.2 to 12.5)	--	--	230 (146 to 347)	8.3 (5.3 to 12.5)	--	--	76.8 (54.6 to 100.8)	0.2 (-11.2 to 12.9)	--	--
		Male	74 (45 to 112)	4.4 (2.8 to 6.7)	--	--	128 (80 to 190)	4.5 (2.8 to 6.7)	--	--	74.3 (44.4 to 110.5)	0.4 (-15 to 18.8)	--	--
	Pruritus	Both	303 (142 to 546)	10 (4.8 to 17.8)	--	--	548 (261 to 1009)	10.6 (5.1 to 19.1)	--	--	80.8 (65.1 to 97.5)	5.9 (0.7 to 11.5)	--	--
		Female	162 (76 to 296)	11 (5.3 to 19.6)	--	--	295 (140 to 539)	11.6 (5.5 to 21.1)	--	--	81.9 (62.6 to 103.1)	5.6 (-1.5 to 13.3)	--	--
		Male	141 (66 to 252)	9.1 (4.3 to 16.2)	--	--	253 (121 to 458)	9.6 (4.6 to 17.3)	--	--	79.5 (62.5 to 100)	6.2 (-1.4 to 14.3)	--	--
	Urticaria	Both	2494 (1621 to 3634)	58.1 (38 to 82.7)	--	--	2831 (1867 to 4055)	58.3 (38.6 to 84.5)	--	--	13.5 (2.9 to 25.7)	0.3 (-5 to 5.8)	--	--
		Female	1507 (974 to 2211)	70.9 (46 to 101.6)	--	--	1679 (1110 to 2402)	71.1 (47.4 to 102.3)	--	--	11.5 (-0.2 to 25.1)	0.3 (-6.3 to 7.7)	--	--
		Male	987 (634 to 1442)	45.8 (29.8 to 65.4)	--	--	1151 (752 to 1652)	46 (29.9 to 67.1)	--	--	16.6 (3.1 to 33.1)	0.4 (-8.5 to 9.4)	--	--
	Decubitus ulcer	Both	12 (9 to 23)	1 (0.7 to 2.1)	0 (0 to 1)	0 (0 to 0.1)	72 (45 to 90)	1.9 (1.2 to 2.4)	4 (2 to 5)	0.1 (0.1 to 0.1)	477.7 (129 to 754.9)	93.8 (-29.1 to 192.7)	1102.9 (162.3 to 2475.2)	145.4 (-43.9 to 398.2)
		Female	6 (4 to 11)	1 (0.6 to 2.1)	0 (0 to 1)	0 (0 to 0.1)	33 (18 to 44)	1.8 (1 to 2.5)	2 (1 to 3)	0.1 (0 to 0.2)	478.7 (103.8 to 894.5)	92.2 (-38.5 to 243.6)	928.7 (70.2 to 2595)	136.8 (-60.3 to 491.1)
		Male	7 (5 to 13)	1 (0.7 to 2.2)	0 (0 to 0)	0 (0 to 0.2)	39 (23 to 51)	2 (1.2 to 2.6)	2 (1 to 2)	0.1 (0.1 to 0.2)	477 (135.4 to 739.5)	96.8 (-25.6 to 200.5)	1335.3 (208.3 to 3372.4)	160 (-37.7 to 527.5)
	Other skin and subcutaneous diseases	Both	1168 (555 to 2146)	41.7 (20.1 to 75.9)	0 (0 to 0)	0 (0 to 0)	2205 (1057 to 4061)	43.8 (21.1 to 80.1)	1 (0 to 1)	0 (0 to 0)	88.8 (83.1 to 95)	5.1 (2.8 to 7.5)	545.4 (214.8 to 1344.4)	28.8 (-33.9 to 226.5)
		Female	576 (276 to 1065)	42.2 (20.3 to 77)	0 (0 to 0)	0 (0 to 0)	1103 (527 to 2032)	44.4 (21.2 to 81.5)	0 (0 to 1)	0 (0 to 0)	91.7 (84.3 to 99.6)	5.1 (1.8 to 8.4)	509 (115.5 to 1930.6)	30.4 (-52.8 to 389.7)
		Male	593 (280 to 1090)	41.2 (19.7 to 75.5)	0 (0 to 0)	0 (0 to 0)	1102 (520 to 2033)	43.3 (20.7 to 79.3)	0 (0 to 1)	0 (0 to 0)	86 (78.6 to 93.9)	5.1 (1.8 to 8.6)	592.6 (277.4 to 1250.3)	36.5 (-26 to 180.9)

Province	Cause	Sex	1990				2019				1990-2019 percent change (%)			
			DALYs		Death		DALYs		Death		DALYs		Death	
			Number	Rate	Number	Rate	Number	Rate	Number	Rate	Number	Rate	Number	Rate
	Sense organ diseases	Both	19622 (14029 to 26496)	1018.3 (735.3 to 1367.8)	--	--	35648 (25213 to 48840)	798.9 (565.3 to 1084.5)	--	--	81.7 (71.8 to 91.3)	-21.5 (-24.5 to -18.5)	--	--
		Female	9529 (6822 to 12856)	1020.7 (737.7 to 1366.9)	--	--	17658 (12464 to 24062)	799.7 (569.2 to 1080.9)	--	--	85.3 (74.3 to 95.2)	-21.7 (-24.9 to -18.4)	--	--
		Male	10093 (7156 to 13690)	1014.2 (728.4 to 1361.6)	--	--	17990 (12689 to 24753)	798.3 (564.4 to 1089.7)	--	--	78.2 (67 to 89.2)	-21.3 (-24.7 to -17.7)	--	--
	Blindness and vision loss	Both	8988 (6372 to 12212)	490.5 (349.2 to 655)	--	--	15815 (11246 to 21252)	365.1 (262.2 to 488)	--	--	76 (69.3 to 83)	-25.6 (-28.6 to -22.5)	--	--
		Female	4607 (3289 to 6234)	517.2 (371.3 to 690.5)	--	--	8304 (5931 to 11227)	385.5 (276.3 to 518.6)	--	--	80.2 (71.8 to 88.7)	-25.5 (-29 to -22.1)	--	--
		Male	4381 (3077 to 5979)	464 (330.8 to 620.2)	--	--	7511 (5325 to 10132)	344.7 (245.9 to 463.2)	--	--	71.4 (63.4 to 80.6)	-25.7 (-29.2 to -22.3)	--	--
	Age-related and other hearing loss	Both	9622 (6701 to 13308)	486.3 (345.9 to 674.7)	--	--	17708 (12321 to 24894)	390.4 (272.2 to 546.4)	--	--	84 (65.4 to 101.7)	-19.7 (-23.7 to -15.7)	--	--
		Female	4390 (3073 to 6034)	458.5 (326 to 635.5)	--	--	8215 (5664 to 11556)	367.2 (255.3 to 516.2)	--	--	87.1 (66.5 to 108.3)	-19.9 (-24.8 to -15.1)	--	--
		Male	5232 (3615 to 7304)	512.2 (363.4 to 710)	--	--	9493 (6585 to 13391)	413.7 (286.9 to 581.3)	--	--	81.5 (61.8 to 100.3)	-19.2 (-24 to -14.5)	--	--
	Other sense organ diseases	Both	1013 (620 to 1551)	41.4 (24.7 to 63.9)	--	--	2126 (1253 to 3299)	43.4 (25.9 to 67.6)	--	--	109.9 (89 to 129.7)	4.7 (-0.1 to 10.2)	--	--
		Female	532 (322 to 812)	45 (26.8 to 69.7)	--	--	1139 (668 to 1765)	47 (28 to 73.1)	--	--	114.1 (92.1 to 139.2)	4.4 (-2.3 to 11.2)	--	--
		Male	481 (288 to 748)	38 (22.6 to 58.6)	--	--	987 (571 to 1522)	39.9 (23.7 to 62)	--	--	105.2 (81.1 to 130.4)	5 (-2.4 to 13.2)	--	--
	Musculoskeletal disorders	Both	42104 (29664 to 56328)	1807.6 (1282.2 to 2403.3)	7 (4 to 12)	0.4 (0.3 to 0.7)	104014 (73906 to 138259)	1921.7 (1372.9 to 2545.9)	34 (25 to 42)	0.8 (0.6 to 0.9)	147 (134.3 to 160.6)	6.3 (2.5 to 10.3)	424.5 (157.5 to 766.5)	101.3 (-2.6 to 219)
		Female	22264 (15853 to 29563)	1974.4 (1404 to 2621.1)	4 (2 to 9)	0.5 (0.3 to 1)	57066 (40689 to 75764)	2139 (1535.5 to 2838.6)	25 (15 to 32)	1.1 (0.6 to 1.5)	156.3 (142.3 to 170.9)	8.3 (4 to 12.8)	472.4 (125.7 to 1019.1)	129.2 (-14.8 to 333.9)
		Male	19840 (13791 to 26720)	1648.9 (1149.5 to 2207.8)	2 (2 to 5)	0.3 (0.2 to 0.5)	46948 (32936 to 62729)	1709.2 (1202.8 to 2271.8)	9 (7 to 12)	0.4 (0.3 to 0.6)	136.6 (123 to 152.7)	3.7 (-1.1 to 9.1)	328.4 (125.8 to 566.7)	62.2 (-7.5 to 143.2)
	Rheumatoid arthritis	Both	265 (183 to 357)	11.6 (7.9 to 15.8)	1 (0 to 2)	0.1 (0 to 0.2)	724 (506 to 987)	13.2 (9.4 to 18)	3 (2 to 4)	0.1 (0 to 0.1)	172.8 (126.9 to 220.9)	14.5 (-7.1 to 34.8)	311.7 (20.8 to 796.7)	29.5 (-63.1 to 192.6)
		Female	184 (125 to 258)	16.4 (11.1 to 23.2)	1 (0 to 2)	0.1 (0 to 0.3)	504 (351 to 696)	18.5 (13 to 25.4)	2 (1 to 3)	0.1 (0 to 0.1)	173.9 (114.9 to 240.2)	13.2 (-14.4 to 41.4)	252.7 (-24.2 to 990.3)	13.4 (-75.8 to 263.3)
		Male	81 (54 to 113)	7 (4.6 to 9.6)	0 (0 to 0)	0 (0 to 0)	220 (153 to 302)	8.1 (5.6 to 11)	1 (1 to 1)	0.1 (0 to 0.1)	170.4 (126.8 to 221.3)	15.6 (-2.8 to 37.4)	497.8 (267 to 1044.1)	94.5 (20.3 to 256.3)
	Osteoarthritis	Both	2955 (1500 to 5782)	177.2 (89.6 to 347.4)	--	--	8916 (4484 to 17590)	191.5 (96.7 to 374.4)	--	--	201.8 (191.4 to 212.9)	8.1 (4.6 to 11.4)	--	--

Province	Cause	Sex	1990				2019				1990-2019 percent change (%)			
			DALYs		Death		DALYs		Death		DALYs		Death	
			Number	Rate	Number	Rate	Number	Rate	Number	Rate	Number	Rate	Number	Rate
		Female	1601 (811 to 3176)	201 (102.1 to 398.9)	--	--	5062 (2541 to 9930)	217.8 (109.7 to 427.7)	--	--	216.2 (202.4 to 231.5)	8.4 (3.8 to 13)	--	--
		Male	1354 (680 to 2649)	154.4 (77.3 to 305.5)	--	--	3855 (1924 to 7529)	165.2 (83.2 to 323.7)	--	--	184.7 (169.1 to 200.2)	7 (1.7 to 11.9)	--	--
	Low back pain	Both	24827 (17486 to 33219)	1007.5 (710.5 to 1346.1)	--	--	48860 (34387 to 65940)	905.8 (644.4 to 1211.6)	--	--	96.8 (83.9 to 109.9)	-10.1 (-13.8 to -6.3)	--	--
		Female	11526 (8148 to 15515)	959.7 (683.2 to 1290.7)	--	--	23690 (16599 to 32038)	898.6 (634.6 to 1203.9)	--	--	105.5 (89 to 122.4)	-6.4 (-11.8 to -0.9)	--	--
		Male	13301 (9325 to 17827)	1050.7 (736.7 to 1416.5)	--	--	25170 (17641 to 34182)	913 (643.6 to 1227.7)	--	--	89.2 (74.5 to 105.4)	-13.1 (-18 to -7.6)	--	--
	Neck pain	Both	9138 (5944 to 13337)	402.2 (265.5 to 582.9)	--	--	22616 (14808 to 32895)	404.4 (265.1 to 582.7)	--	--	147.5 (131.6 to 161.9)	0.5 (-1.5 to 2.7)	--	--
		Female	5415 (3536 to 7919)	491.8 (326.4 to 718.4)	--	--	13652 (8996 to 19830)	492.7 (325 to 718.2)	--	--	152.1 (135.3 to 167.2)	0.2 (-2.3 to 2.9)	--	--
		Male	3723 (2405 to 5414)	316.9 (207 to 459.3)	--	--	8964 (5768 to 13198)	317.9 (209.9 to 466.2)	--	--	140.8 (123.3 to 158.7)	0.3 (-3.2 to 3.7)	--	--
	Gout	Both	261 (157 to 385)	14.4 (8.7 to 21.2)	--	--	766 (486 to 1105)	15.6 (9.8 to 22.4)	--	--	193.2 (149.6 to 240)	8.3 (-5.8 to 23.8)	--	--
		Female	54 (33 to 79)	6.3 (3.9 to 9.1)	--	--	172 (103 to 250)	7.1 (4.3 to 10.2)	--	--	218.5 (152.6 to 298.8)	12.8 (-8.8 to 38.6)	--	--
		Male	207 (126 to 307)	22.3 (13.7 to 33)	--	--	594 (377 to 869)	24 (15.1 to 34.8)	--	--	186.7 (139.6 to 242.1)	7.3 (-8.5 to 25.3)	--	--
	Other musculoskeletal disorders	Both	4657 (2714 to 6956)	194.8 (112 to 296.6)	6 (4 to 11)	0.3 (0.2 to 0.6)	22131 (14747 to 31360)	391.3 (259.6 to 547.4)	31 (23 to 39)	0.7 (0.5 to 0.9)	375.2 (302.6 to 497.4)	100.9 (69.3 to 153.9)	439 (179.9 to 798.8)	113.9 (14.9 to 243)
		Female	3484 (2211 to 5060)	299.2 (184.7 to 434.6)	4 (2 to 8)	0.4 (0.2 to 0.8)	13985 (9537 to 19213)	504.2 (343.9 to 691.5)	23 (14 to 30)	1 (0.6 to 1.3)	301.4 (250.8 to 367.9)	68.5 (46.6 to 100.9)	505.1 (166.4 to 1106.8)	153.9 (6.3 to 389.9)
		Male	1173 (421 to 2134)	97.5 (35.9 to 173.8)	2 (1 to 5)	0.2 (0.2 to 0.5)	8146 (5042 to 11917)	281.1 (172.9 to 406.1)	8 (7 to 10)	0.4 (0.3 to 0.5)	594.2 (389 to 1331.8)	188.1 (103.6 to 461.9)	313.2 (107.8 to 555)	58.6 (-13.8 to 139.1)
	Other non-communicable diseases	Both	167054 (121361 to 212211)	3774.6 (2837.6 to 4709.1)	1477 (974 to 1985)	28.9 (19.3 to 39)	104983 (82344 to 133830)	2177.7 (1731.3 to 2721.1)	506 (421 to 621)	13 (10.7 to 16.1)	-37.2 (-54.7 to -10.1)	-42.3 (-56.1 to -22.3)	-65.7 (-76.5 to -43.1)	-54.9 (-68.9 to -25.6)
		Female	86500 (62966 to 110770)	4266 (3224.2 to 5356.4)	673 (430 to 930)	27 (17.8 to 37.5)	68280 (51730 to 88821)	2707.2 (2084.8 to 3491.1)	228 (184 to 283)	12.1 (9.7 to 15)	-21.1 (-43 to 10.2)	-36.5 (-50.7 to -17.8)	-66.2 (-78 to -42.3)	-55.3 (-70.9 to -25)
		Male	80554 (52494 to 107505)	3300.1 (2244.7 to 4310.7)	804 (485 to 1106)	30.7 (18.9 to 42.2)	36704 (29052 to 46093)	1667 (1330.4 to 2080.9)	279 (222 to 350)	14 (11.1 to 17.8)	-54.4 (-69.1 to -24.4)	-49.5 (-64.4 to -19.3)	-65.3 (-76.9 to -35.8)	-54.5 (-69.7 to -16.8)

Province	Cause	Sex	1990				2019				1990-2019 percent change (%)			
			DALYs		Death		DALYs		Death		DALYs		Death	
			Number	Rate	Number	Rate	Number	Rate	Number	Rate	Number	Rate	Number	Rate
	Congenital birth defects	Both	121320 (80227 to 164788)	2290.3 (1522.4 to 3111.7)	1337 (871 to 1836)	25.2 (16.4 to 34.7)	32671 (25688 to 42250)	848.6 (660.1 to 1108.9)	335 (258 to 438)	8.8 (6.7 to 11.6)	-73.1 (-82.4 to -52.8)	-62.9 (-75.9 to -35)	-75 (-84 to -55)	-65.1 (-77.7 to -36.7)
		Female	54946 (34375 to 77630)	2117.9 (1334.8 to 2980.4)	608 (373 to 866)	23.4 (14.4 to 33.3)	14547 (10998 to 18927)	774.7 (582.5 to 1022.2)	150 (112 to 200)	8.1 (6 to 10.9)	-73.5 (-83.5 to -50.4)	-63.4 (-77.2 to -31.7)	-75.3 (-84.9 to -51.8)	-65.3 (-78.9 to -32.3)
		Male	66375 (38580 to 92117)	2454.6 (1433.1 to 3396.8)	729 (411 to 1028)	26.9 (15.3 to 37.9)	18125 (14057 to 23983)	918.7 (706.7 to 1236.2)	184 (140 to 247)	9.5 (7.1 to 12.9)	-72.7 (-82.3 to -44.5)	-62.6 (-76 to -24.5)	-74.7 (-84 to -46)	-64.9 (-77.9 to -25.4)
	Urinary diseases and male infertility	Both	678 (473 to 947)	26.8 (18.4 to 38)	3 (2 to 7)	0.2 (0.1 to 0.7)	1831 (1322 to 2417)	38.6 (28.1 to 49.9)	33 (18 to 40)	0.9 (0.5 to 1.1)	170.1 (93.1 to 222.7)	43.9 (-5.4 to 77.9)	881.2 (174.7 to 1458.8)	298.8 (-11.3 to 616.1)
		Female	219 (163 to 297)	14.6 (10.6 to 23.6)	2 (1 to 4)	0.3 (0.1 to 0.9)	560 (386 to 697)	24.4 (17 to 30.3)	15 (9 to 19)	0.9 (0.5 to 1.1)	156.3 (55.6 to 238.4)	67.6 (-17.5 to 128.7)	732.6 (114.6 to 1416.1)	235.5 (-27.8 to 594.2)
		Male	459 (301 to 688)	38.8 (25.1 to 57.2)	2 (1 to 3)	0.2 (0.1 to 0.7)	1271 (888 to 1767)	52.7 (36.9 to 71.8)	18 (8 to 23)	0.9 (0.5 to 1.2)	176.7 (103.8 to 230.9)	36 (-5.4 to 64.9)	1055.7 (159.9 to 1771.7)	398 (-22 to 790.9)
	Gynecological diseases	Female	14735 (10131 to 20853)	1097.8 (749.7 to 1522)	0 (0 to 0)	0 (0 to 0)	31601 (21658 to 43904)	1045.7 (715.9 to 1445.6)	0 (0 to 1)	0 (0 to 0)	114.5 (101.6 to 126.9)	-4.7 (-7.8 to -1.9)	324.2 (-40.9 to 1593.1)	83.4 (-71.7 to 573.1)
	Hemoglobinopathies and hemolytic anemias	Both	9415 (7263 to 12128)	219 (168 to 284.1)	62 (47 to 78)	1.5 (1.1 to 1.8)	5302 (4020 to 7119)	107 (82.7 to 142.7)	39 (34 to 45)	0.8 (0.7 to 1)	-43.7 (-55.1 to -27.8)	-51.1 (-59.9 to -39.3)	-36.6 (-52 to -11.2)	-41.6 (-54.2 to -21.1)
		Female	5385 (3837 to 7135)	269.8 (190.7 to 363.1)	30 (20 to 40)	1.4 (1 to 1.8)	3194 (2191 to 4563)	127.1 (90 to 178.8)	18 (14 to 23)	0.8 (0.6 to 1)	-40.7 (-56.7 to -18.6)	-52.9 (-63.9 to -37.6)	-39.5 (-60 to -1.5)	-43.5 (-60.6 to -11.7)
		Male	4030 (3050 to 5300)	169.4 (129.2 to 220.9)	32 (24 to 42)	1.5 (1.1 to 1.8)	2108 (1599 to 2774)	87.8 (66.6 to 116.8)	21 (17 to 26)	0.9 (0.7 to 1.1)	-47.7 (-61.1 to -28.2)	-48.2 (-60.7 to -29.7)	-33.9 (-52.9 to -1.7)	-39.6 (-55.4 to -13.5)
	Endocrine, metabolic, blood, and immune disorders	Both	9788 (6630 to 14016)	309.1 (211.7 to 439.1)	30 (18 to 73)	1.2 (0.7 to 2.6)	17075 (12305 to 22955)	341.1 (248.1 to 454.7)	91 (65 to 110)	2.2 (1.6 to 2.7)	74.4 (27 to 108.5)	10.3 (-11.1 to 27.1)	200.7 (14.2 to 480.6)	94.8 (-19.1 to 284.3)
		Female	5931 (4003 to 8545)	387.9 (263.6 to 546.2)	13 (7 to 34)	1.1 (0.6 to 2.9)	10413 (7235 to 14243)	416.3 (292.7 to 567.9)	40 (25 to 51)	2 (1.2 to 2.6)	75.6 (31.6 to 106.2)	7.3 (-12.4 to 22.6)	207.7 (-5.6 to 528)	89.6 (-34.7 to 313.3)
		Male	3857 (2556 to 6029)	234.2 (157.4 to 340.1)	17 (9 to 43)	1.2 (0.6 to 2.8)	6662 (4849 to 8713)	268.7 (197.8 to 346)	51 (30 to 66)	2.4 (1.5 to 3.2)	72.7 (15 to 122.2)	14.7 (-12.5 to 41.7)	195.5 (23.7 to 565.1)	98.6 (-15.8 to 342.7)
	Oral disorders	Both	7213 (4273 to 11379)	316.4 (190 to 493.5)	--	--	15801 (9305 to 25361)	309.7 (182.7 to 485.8)	--	--	119.1 (109.6 to 128.9)	-2.1 (-4.8 to 0.4)	--	--
		Female	3463 (2038 to 5455)	307.3 (183.5 to 483.2)	--	--	7635 (4461 to 12348)	300.4 (178.6 to 474.9)	--	--	120.5 (108.9 to 132)	-2.3 (-5.6 to 0.7)	--	--
		Male	3750 (2226 to 5896)	325.7 (196.4 to 509.5)	--	--	8166 (4823 to 12965)	319.1 (189.6 to 497.4)	--	--	117.8 (107.5 to 128.4)	-2 (-5.4 to 1.1)	--	--
	Sudden infant death syndrome	Both	3906 (873 to 12583)	74.2 (16.6 to 239)	44 (10 to 142)	0.8 (0.2 to 2.7)	703 (341 to 1393)	19.3 (9.4 to 38.3)	8 (4 to 16)	0.2 (0.1 to 0.4)	-82 (-93.6 to -25.6)	-74 (-90.8 to 7.7)	-82 (-93.6 to -25.6)	-74 (-90.8 to 7.7)
		Female	1822 (404 to 6106)	70.8 (15.7 to 237.2)	21 (5 to 69)	0.8 (0.2 to 2.7)	330 (159 to 641)	18.7 (9 to 36.2)	4 (2 to 7)	0.2 (0.1 to 0.4)	-81.9 (-93.6 to -25.6)	-73.6 (-90.7 to 8)	-81.9 (-93.6 to -25.6)	-73.7 (-90.7 to 8)
		Male	2084 (378 to 6935)	77.4 (14.1 to 257.5)	24 (4 to 78)	0.9 (0.2 to 2.9)	372 (167 to 797)	20 (9 to 42.7)	4 (2 to 9)	0.2 (0.1 to 0.5)	-82.1 (-94 to -21.5)	-74.2 (-91.3 to 13.2)	-82.1 (-94 to -21.5)	-74.2 (-91.3 to 13.2)

Province	Cause	Sex	1990				2019				1990-2019 percent change (%)			
			DALYs		Death		DALYs		Death		DALYs		Death	
			Number	Rate	Number	Rate	Number	Rate	Number	Rate	Number	Rate	Number	Rate
	**Injuries**	Both	300160 (242589 to 351590)	8177.8 (6598.7 to 9582.6)	4277 (3336 to 5123)	136.4 (104.3 to 165.9)	219413 (193289 to 248825)	4154 (3670.5 to 4708.4)	3702 (3184 to 4274)	72.8 (62.8 to 83.8)	-26.9 (-40.1 to -6.4)	-49.2 (-58.7 to -35.3)	-13.4 (-32 to 14.7)	-46.6 (-58.4 to -30)
		Female	92351 (71778 to 112139)	4835.3 (3806.5 to 5904.2)	1191 (869 to 1503)	73.1 (53.8 to 92.5)	53891 (46092 to 62583)	2143.2 (1852.4 to 2467.5)	841 (694 to 1010)	35.7 (29.8 to 42.3)	-41.6 (-53.8 to -20.6)	-55.7 (-64.8 to -41.6)	-29.4 (-47.6 to 1.6)	-51.2 (-63.6 to -32.2)
		Male	207808 (163288 to 247532)	11371.2 (8944 to 13702.9)	3086 (2297 to 3809)	197.4 (141 to 247.2)	165523 (142265 to 191244)	6081.8 (5224.8 to 7029.9)	2861 (2377 to 3390)	108.6 (90.3 to 128.9)	-20.3 (-37 to 4.4)	-46.5 (-58.2 to -29.9)	-7.3 (-30.4 to 27.7)	-45 (-59.4 to -23.7)
	Transport injuries	Both	164625 (123219 to 198777)	4433.3 (3228.3 to 5393.9)	2628 (1870 to 3222)	85.5 (58.3 to 106)	117106 (98117 to 135806)	2239.9 (1880.2 to 2592.9)	2253 (1823 to 2660)	44.3 (35.9 to 52.1)	-28.9 (-43.5 to -4.4)	-49.5 (-60.7 to -31.6)	-14.3 (-33.5 to 16.6)	-48.2 (-60.6 to -28.7)
		Female	48324 (35676 to 59880)	2435.4 (1820.9 to 3016.9)	703 (514 to 881)	43.2 (32.2 to 54.5)	25938 (21194 to 30915)	1037.8 (856.9 to 1229.5)	491 (385 to 597)	20.2 (16.1 to 24.4)	-46.3 (-59.7 to -24.2)	-57.4 (-67.9 to -41.2)	-30.2 (-48.7 to -1.8)	-53.2 (-65.5 to -33.9)
		Male	116302 (79875 to 143543)	6340.3 (4158.7 to 8010)	1926 (1253 to 2443)	126.2 (77.1 to 164.1)	91167 (72037 to 108803)	3391.1 (2687.3 to 4034.3)	1763 (1323 to 2150)	67.5 (50.4 to 81.9)	-21.6 (-40.3 to 11.2)	-46.5 (-60.1 to -23.5)	-8.5 (-32.5 to 30.9)	-46.5 (-61.2 to -22.8)
	Road injuries	Both	158593 (119523 to 191150)	4263.5 (3109.1 to 5205)	2532 (1814 to 3115)	82.2 (56.1 to 102.2)	101832 (83436 to 118361)	1951.1 (1608.9 to 2262.8)	1950 (1542 to 2311)	38.3 (30.5 to 45.4)	-35.8 (-49.3 to -12.5)	-54.2 (-64.5 to -37.6)	-23 (-40.3 to 6.2)	-53.4 (-64.3 to -35.4)
		Female	46830 (34460 to 58260)	2359.2 (1767.2 to 2922.5)	682 (500 to 855)	41.9 (31.3 to 52.8)	23006 (18629 to 27581)	920.3 (753.8 to 1095)	433 (339 to 529)	17.8 (14 to 21.5)	-50.9 (-62.9 to -29.9)	-61 (-70.6 to -45.9)	-36.4 (-53.4 to -9.6)	-57.5 (-68.6 to -39.6)
		Male	111763 (76586 to 138258)	6081.5 (3995.3 to 7676)	1850 (1204 to 2350)	121 (74.9 to 157.2)	78826 (62472 to 94418)	2937.8 (2333 to 3511.4)	1517 (1154 to 1849)	58.1 (44.1 to 70.9)	-29.5 (-46.6 to 0)	-51.7 (-63.9 to -29.9)	-18 (-39.8 to 19)	-52 (-65.2 to -29.3)
	Other transport injuries	Both	6033 (3471 to 8319)	169.7 (98.4 to 229.1)	96 (50 to 133)	3.3 (1.8 to 4.6)	15274 (10948 to 19612)	288.8 (209.4 to 369.1)	303 (207 to 392)	5.9 (4 to 7.6)	153.2 (59.6 to 364.3)	70.1 (7.2 to 215.7)	216 (90 to 547.5)	80.1 (12 to 237.1)
		Female	1494 (980 to 2141)	76.2 (51.7 to 103.8)	21 (13 to 30)	1.3 (0.8 to 1.8)	2933 (2291 to 3683)	117.5 (93 to 145.9)	57 (44 to 73)	2.4 (1.8 to 3)	96.4 (26.7 to 212.4)	54.3 (6.6 to 132.5)	174.6 (78.7 to 344.3)	85.8 (25.2 to 196.5)
		Male	4539 (2330 to 6592)	258.9 (134.3 to 370.8)	75 (35 to 111)	5.2 (2.4 to 7.7)	12341 (8083 to 16258)	453.4 (297.8 to 602.3)	246 (150 to 331)	9.3 (5.7 to 12.6)	171.9 (41.8 to 450.1)	75.1 (-3.8 to 261.6)	227.5 (81.3 to 651.4)	79.3 (-2.9 to 299.2)
	Unintentional injuries	Both	83010 (65505 to 100271)	2152.9 (1731 to 2577.7)	1076 (829 to 1329)	33.4 (26.3 to 40.7)	55140 (46884 to 64548)	1067.6 (913.5 to 1242.4)	791 (680 to 912)	16.7 (14.4 to 19.1)	-33.6 (-46.9 to -14.4)	-50.4 (-59.6 to -37.3)	-26.5 (-43.6 to -0.2)	-50.2 (-61.3 to -33.8)
		Female	28497 (20553 to 37728)	1422.3 (1048.3 to 1874.8)	349 (240 to 478)	21.4 (15.3 to 28.6)	15644 (12978 to 18732)	648.9 (545 to 773.7)	215 (178 to 259)	10.2 (8.6 to 12.2)	-45.1 (-60.2 to -23.2)	-54.4 (-66.3 to -38.6)	-38.5 (-57.7 to -7.1)	-52.4 (-66.1 to -31.3)
		Male	54513 (41902 to 67194)	2851.4 (2245.6 to 3475.1)	726 (548 to 921)	45.1 (34.1 to 57.4)	39496 (33196 to 46736)	1470.6 (1243.9 to 1734.2)	576 (479 to 697)	22.9 (19.1 to 27.5)	-27.5 (-44.2 to -4.3)	-48.4 (-59.9 to -33.5)	-20.7 (-42.8 to 11.9)	-49.2 (-63.3 to -28.5)
	Falls	Both	14940 (11560 to 18287)	488.5 (389.5 to 594.6)	157 (114 to 197)	6.7 (5.1 to 8.3)	17952 (14261 to 22301)	347.8 (278.9 to 428.6)	218 (179 to 262)	5.1 (4.1 to 6)	20.2 (-2.9 to 46.7)	-28.8 (-39 to -16.9)	39.1 (4.3 to 95.4)	-24.4 (-42.6 to 5.3)
		Female	3878 (2682 to 5025)	253.2 (197.2 to 315.7)	37 (26 to 49)	3.6 (2.7 to 5.1)	4164 (3260 to 5343)	178.6 (140.9 to 225.1)	51 (40 to 62)	2.9 (2.2 to 3.5)	7.4 (-20.3 to 49.2)	-29.5 (-41.1 to -16.4)	37.6 (-2.7 to 105.6)	-20.7 (-45.9 to 21.4)
		Male	11062 (7847 to 13825)	712.7 (541.6 to 881.6)	120 (74 to 156)	9.7 (7 to 12.4)	13788 (10897 to 17183)	512.1 (406.8 to 634.1)	167 (130 to 208)	7.2 (5.5 to 8.9)	24.6 (-1.2 to 66.8)	-28.1 (-40.7 to -11.6)	39.6 (-2.1 to 122.9)	-26.1 (-46.2 to 7.3)
	Drowning	Both	15525 (10419 to 21171)	306.7 (207.6 to 417.2)	203 (137 to 276)	4.4 (3 to 5.8)	3683 (3044 to 4479)	76.7 (63.5 to 94.3)	61 (50 to 74)	1.3 (1 to 1.5)	-76.3 (-83.8 to -61.5)	-75 (-82.8 to -60.2)	-69.8 (-79.2 to -51.5)	-71.3 (-80 to -54)
		Female	4962 (3007 to 7328)	186.2 (115.5 to 269.3)	61 (37 to 89)	2.4 (1.6 to 3.5)	647 (517 to 816)	28.9 (22.8 to 36.9)	10 (8 to 12)	0.4 (0.4 to 0.6)	-87 (-92.1 to -77)	-84.5 (-90.4 to -72.8)	-83.5 (-89.9 to -71.6)	-81.6 (-88.3 to -69.3)

Province	Cause	Sex	1990				2019				1990-2019 percent change (%)			
			DALYs		Death		DALYs		Death		DALYs		Death	
			Number	Rate	Number	Rate	Number	Rate	Number	Rate	Number	Rate	Number	Rate
		Male	10562 (5785 to 15286)	422.2 (238.3 to 600.4)	142 (80 to 204)	6.3 (3.6 to 8.8)	3036 (2426 to 3821)	122.1 (97.3 to 153.7)	51 (41 to 65)	2 (1.6 to 2.6)	-71.3 (-81.7 to -44.6)	-71.1 (-81.2 to -45.3)	-64 (-77.3 to -30.8)	-67.5 (-79 to -40.2)
	Fire, heat, and hot substances	Both	16506 (7493 to 26507)	409.2 (198.3 to 637.1)	244 (112 to 389)	7.6 (4 to 11.4)	7058 (5239 to 8986)	136.3 (101.7 to 174.1)	140 (103 to 178)	2.9 (2.2 to 3.7)	-57.2 (-74.4 to -14.3)	-66.7 (-79.4 to -33.9)	-42.4 (-65.3 to 22.8)	-61.8 (-75.9 to -30.1)
		Female	7491 (1755 to 14873)	376.1 (110.5 to 714)	108 (27 to 213)	6.8 (2.5 to 12.3)	3174 (2078 to 4528)	129.1 (84.7 to 184.6)	63 (40 to 87)	2.8 (1.9 to 3.8)	-57.6 (-80.1 to 64.7)	-65.7 (-83.1 to 6.8)	-42.1 (-72.9 to 123.2)	-59.3 (-79 to 3.6)
		Male	9015 (3534 to 13369)	440.2 (182.2 to 632.5)	135 (53 to 198)	8.3 (3.9 to 11.9)	3884 (2800 to 5057)	143.2 (106.4 to 188.9)	78 (54 to 102)	3 (2.2 to 4.1)	-56.9 (-72 to 6.4)	-67.5 (-78.8 to -23.3)	-42.6 (-63.2 to 38.3)	-63.4 (-76.7 to -20.8)
	Poisonings	Both	5642 (3926 to 8192)	126.1 (90.9 to 179.4)	78 (55 to 111)	2.1 (1.5 to 2.9)	2912 (2345 to 3597)	54.4 (44.8 to 68.1)	56 (45 to 68)	1.1 (0.9 to 1.3)	-48.4 (-65.9 to -21.5)	-56.8 (-70.7 to -36.4)	-28.3 (-52.5 to 6.8)	-48.5 (-64.7 to -25.5)
		Female	2146 (1348 to 3408)	90.8 (59.3 to 135.8)	28 (17 to 43)	1.4 (0.9 to 2)	776 (606 to 1028)	31.4 (24.8 to 41.2)	14 (11 to 19)	0.6 (0.5 to 0.8)	-63.8 (-79.3 to -36.9)	-65.4 (-79.2 to -42.7)	-49 (-70 to -13.2)	-56.8 (-72.5 to -31)
		Male	3496 (2164 to 5443)	159.9 (105.7 to 244.5)	50 (32 to 77)	2.7 (1.8 to 4)	2136 (1640 to 2793)	76.5 (59 to 101.2)	41 (31 to 53)	1.5 (1.2 to 1.9)	-38.9 (-63 to 4.5)	-52.2 (-70.1 to -22)	-16.8 (-49.7 to 38.8)	-44.4 (-65.3 to -10.7)
	Exposure to mechanical forces	Both	6237 (3730 to 12725)	190.9 (117.1 to 366.2)	74 (36 to 182)	2.5 (1.2 to 5.9)	7444 (5474 to 11886)	138.4 (102.5 to 217.7)	87 (59 to 165)	1.6 (1.1 to 3.1)	19.3 (-24.1 to 80.6)	-27.5 (-52.1 to 4.2)	16.5 (-30.7 to 118.7)	-34.5 (-62.4 to 19.3)
		Female	1303 (898 to 2383)	78.5 (55.5 to 128.2)	11 (6 to 27)	0.7 (0.4 to 1.8)	1563 (1140 to 2268)	61.7 (45.2 to 90.8)	11 (8 to 23)	0.5 (0.3 to 1)	19.9 (-19.8 to 58.5)	-21.4 (-39.5 to -3)	1.7 (-33.6 to 64.5)	-33 (-56.7 to 6)
		Male	4934 (2738 to 10789)	298.6 (172.9 to 617.4)	63 (28 to 159)	4.2 (1.9 to 10.5)	5881 (4218 to 9719)	212 (153.6 to 345)	75 (49 to 144)	2.7 (1.8 to 5.1)	19.2 (-28.7 to 93.3)	-29 (-56.2 to 10.9)	19.1 (-33.4 to 139.7)	-34.9 (-64.5 to 27.9)
	Adverse effects of medical treatment	Both	7059 (2941 to 12167)	207.1 (86.4 to 364.3)	124 (51 to 220)	4.9 (2.1 to 8.7)	2640 (1958 to 3175)	53.2 (40.5 to 63.2)	68 (52 to 81)	1.5 (1.2 to 1.8)	-62.6 (-78.2 to -18.4)	-74.3 (-85.5 to -43.2)	-45.3 (-69.9 to 23.2)	-68.9 (-83.5 to -35)
		Female	2080 (1015 to 3226)	118.9 (60.8 to 195.1)	35 (18 to 57)	2.9 (1.5 to 5.2)	973 (676 to 1233)	40.9 (29.1 to 51)	25 (18 to 32)	1.2 (0.9 to 1.5)	-53.2 (-70.5 to -17.5)	-65.6 (-79.3 to -39.8)	-28.3 (-60.1 to 28.8)	-59.3 (-79.8 to -27.3)
		Male	4980 (1645 to 9534)	291.3 (93.8 to 564.6)	90 (29 to 173)	6.9 (2.4 to 13.4)	1667 (1067 to 2118)	65.2 (43.2 to 82)	43 (29 to 55)	1.9 (1.3 to 2.4)	-66.5 (-82.4 to -7.5)	-77.6 (-88.5 to -36.7)	-51.9 (-76.5 to 39.6)	-72.9 (-86.7 to -28.7)
	Animal contact	Both	1738 (928 to 2361)	38.3 (22.6 to 49.6)	21 (11 to 29)	0.6 (0.3 to 0.7)	740 (584 to 898)	15.3 (12.1 to 18.7)	9 (7 to 12)	0.2 (0.2 to 0.3)	-57.4 (-70.5 to -29.8)	-60 (-70.6 to -39.2)	-55.1 (-68.8 to -22.7)	-61.5 (-72.2 to -38.1)
		Female	710 (279 to 1138)	31.1 (15.2 to 46.3)	8 (3 to 13)	0.4 (0.2 to 0.6)	304 (226 to 393)	12.9 (9.5 to 16.9)	3 (2 to 4)	0.1 (0.1 to 0.2)	-57.2 (-75.4 to -4.8)	-58.7 (-74.5 to -25)	-63.7 (-79.1 to -11.5)	-65 (-78.2 to -32.3)
		Male	1028 (536 to 1453)	45 (25.1 to 60.4)	13 (6 to 19)	0.7 (0.4 to 1)	436 (335 to 535)	17.7 (13.5 to 22.1)	7 (5 to 8)	0.3 (0.2 to 0.4)	-57.6 (-71.9 to -26.7)	-60.8 (-73 to -35.8)	-49.8 (-67.1 to -9.9)	-59.9 (-73.1 to -30.3)
	Foreign body	Both	5078 (3822 to 6756)	107.5 (82.3 to 138.8)	56 (41 to 74)	1.3 (1 to 1.6)	2630 (2171 to 3142)	56.1 (46.3 to 67.1)	31 (26 to 37)	0.7 (0.6 to 0.8)	-48.2 (-62.5 to -27.7)	-47.8 (-62.1 to -30)	-43.6 (-60.5 to -19.7)	-43.5 (-59.2 to -22.8)
		Female	2600 (1781 to 3699)	109 (78 to 151.4)	28 (19 to 40)	1.3 (0.9 to 1.7)	1019 (806 to 1247)	45.9 (36.2 to 56.5)	11 (9 to 14)	0.6 (0.5 to 0.7)	-60.8 (-75 to -39)	-57.8 (-72.9 to -36.1)	-59.6 (-74.4 to -34.6)	-54.4 (-69.6 to -30.7)
		Male	2478 (1761 to 3454)	106.1 (78.8 to 144.2)	27 (19 to 38)	1.3 (1 to 1.7)	1611 (1311 to 1977)	65.9 (53.2 to 80)	20 (16 to 25)	0.9 (0.7 to 1.1)	-35 (-56.3 to -6.3)	-37.9 (-56.7 to -13.2)	-26.9 (-52.7 to 12.9)	-33.4 (-54.4 to -4)
	Environmental heat and cold exposure	Both	476 (338 to 701)	15.8 (11.2 to 23.3)	3 (2 to 6)	0.1 (0.1 to 0.3)	677 (486 to 911)	12.7 (9.2 to 17.1)	4 (2 to 5)	0.1 (0.1 to 0.1)	42.4 (3.8 to 67.7)	-19.2 (-38.1 to -6.4)	29.7 (-30.1 to 129.5)	-41.8 (-66.3 to 1)
		Female	210 (146 to 302)	14.4 (10.1 to 20.1)	1 (0 to 2)	0.1 (0 to 0.2)	322 (228 to 446)	12.4 (8.8 to 17.1)	1 (1 to 2)	0 (0 to 0.1)	53.3 (14.5 to 78.1)	-13.9 (-32.6 to -2.7)	48.4 (-31.7 to 209.4)	-27.9 (-68 to 56.4)
		Male	265 (187 to 429)	17.2 (12.3 to 27.8)	2 (1 to 5)	0.2 (0.1 to 0.5)	355 (258 to 484)	13.1 (9.6 to 17.7)	2 (1 to 4)	0.1 (0.1 to 0.2)	33.8 (-6.8 to 66.1)	-23.8 (-45.3 to -6.2)	23.2 (-34 to 120.4)	-48 (-70.9 to -8.6)
	Exposure to forces of nature	Both	1683 (1501 to 1882)	44.6 (39.7 to 49.8)	22 (20 to 25)	0.7 (0.6 to 0.8)	1637 (1460 to 1830)	32.5 (29.2 to 36.3)	26 (23 to 28)	0.5 (0.5 to 0.6)	-2.8 (-5.1 to -0.4)	-27.1 (-29.3 to -25.1)	15 (15 to 15)	-26.3 (-26.3 to -26.3)

Province	Cause	Sex	1990				2019				1990-2019 percent change (%)			
			DALYs		Death		DALYs		Death		DALYs		Death	
			Number	Rate	Number	Rate	Number	Rate	Number	Rate	Number	Rate	Number	Rate
		Female	631 (564 to 705)	33.1 (29.5 to 37.1)	8 (8 to 9)	0.5 (0.5 to 0.6)	590 (527 to 662)	24.5 (21.9 to 27.4)	9 (9 to 10)	0.4 (0.4 to 0.4)	-6.4 (-8.5 to -4)	-25.8 (-28.1 to -23.9)	12.1 (12.1 to 12.1)	-25.7 (-25.7 to -25.7)
		Male	1053 (937 to 1177)	55.7 (49.5 to 62.3)	14 (13 to 15)	0.9 (0.8 to 1)	1046 (933 to 1167)	40.2 (36 to 44.8)	16 (15 to 18)	0.6 (0.6 to 0.7)	-0.6 (-3.2 to 1.7)	-27.8 (-30.2 to -25.7)	16.8 (16.8 to 16.8)	-26.8 (-26.8 to -26.8)
	Other unintentional injuries	Both	8125 (6185 to 10834)	218.3 (166.5 to 287.1)	94 (66 to 134)	2.6 (1.9 to 3.8)	7767 (6090 to 9538)	144 (114 to 176.3)	90 (68 to 110)	1.7 (1.3 to 2)	-4.4 (-34.6 to 29.6)	-34.1 (-54.2 to -13)	-3.4 (-42.3 to 48.4)	-36 (-61.4 to -1.8)
		Female	2486 (1715 to 3340)	130.9 (93.9 to 171.8)	24 (14 to 35)	1.3 (0.8 to 1.8)	2112 (1578 to 2770)	82.6 (62.3 to 108.5)	16 (12 to 21)	0.7 (0.5 to 0.8)	-15 (-42.2 to 25.2)	-36.9 (-54.4 to -13.7)	-33.4 (-60.4 to 25.9)	-46.7 (-67.7 to -4.8)
		Male	5639 (4093 to 8285)	302.3 (221.7 to 435.8)	70 (47 to 111)	3.9 (2.7 to 6.3)	5655 (4232 to 6985)	202.6 (154.8 to 248.4)	75 (52 to 94)	2.6 (1.9 to 3.3)	0.3 (-42.6 to 50)	-33 (-61 to -3.4)	6.9 (-48.1 to 80.1)	-32.7 (-67.2 to 11.9)
	Self-harm and interpersonal violence	Both	52524 (41786 to 63956)	1591.6 (1283.8 to 1958.2)	573 (429 to 727)	17.5 (13.3 to 22.5)	47168 (39957 to 56612)	846.5 (723.9 to 1005.6)	657 (537 to 826)	11.9 (9.8 to 14.8)	-10.2 (-26.3 to 14.4)	-46.8 (-56.6 to -33)	14.8 (-11.6 to 58.9)	-31.8 (-47.5 to -6.2)
		Female	15530 (11030 to 20680)	977.7 (730.3 to 1296.8)	139 (77 to 196)	8.5 (5 to 11.9)	12308 (10209 to 14864)	456.4 (379.8 to 544.3)	135 (106 to 169)	5.3 (4.2 to 6.5)	-20.7 (-38.1 to 12)	-53.3 (-63.5 to -35.9)	-2.4 (-34.9 to 85.7)	-38.3 (-58.2 to 6.7)
		Male	36994 (30053 to 45401)	2179.5 (1771.1 to 2689.7)	434 (330 to 567)	26.1 (19.7 to 35.3)	34860 (28497 to 43343)	1220.1 (999.3 to 1497.6)	522 (408 to 683)	18.3 (14.4 to 23.6)	-5.8 (-26.2 to 21.8)	-44 (-56.2 to -27.1)	20.3 (-11.1 to 66)	-29.8 (-48.9 to -2.2)
	Self-harm	Both	15322 (9053 to 21821)	443.5 (279.4 to 660.1)	254 (154 to 372)	8.1 (5.3 to 12.6)	18485 (14643 to 25541)	331.4 (264.2 to 444)	340 (268 to 483)	6.2 (4.9 to 8.6)	20.6 (-11.7 to 101.8)	-25.3 (-44.8 to 15.4)	33.8 (-1.9 to 117.8)	-24.4 (-44.9 to 15.5)
		Female	6850 (3491 to 9763)	392.4 (212 to 550.6)	108 (55 to 156)	6.7 (3.7 to 9.5)	4900 (3729 to 6258)	188.3 (145.4 to 240)	84 (64 to 108)	3.3 (2.5 to 4.2)	-28.5 (-53 to 50.4)	-52 (-67.8 to -5.3)	-22 (-49.7 to 63.7)	-51.3 (-67.8 to -8.9)
		Male	8472 (5081 to 14210)	492.2 (306.2 to 873.1)	146 (90 to 254)	9.5 (6.1 to 17.4)	13585 (10169 to 20530)	468.2 (351.8 to 694.3)	256 (190 to 405)	8.9 (6.7 to 13.8)	60.4 (9.6 to 169)	-4.9 (-36.5 to 58.5)	75.2 (18 to 192.8)	-6.2 (-36.9 to 54.9)
	Interpersonal violence	Both	10639 (7264 to 14028)	311.3 (211.7 to 406.8)	155 (99 to 213)	5 (3.2 to 6.7)	17401 (13796 to 21003)	322.6 (255.5 to 391.2)	261 (199 to 324)	4.8 (3.7 to 6)	63.6 (17.6 to 145.4)	3.6 (-24.3 to 52.6)	68.7 (16.2 to 174.2)	-3.7 (-33.5 to 55.4)
		Female	2445 (1735 to 3312)	134.9 (98.5 to 180.3)	26 (16 to 39)	1.5 (1 to 2.2)	4011 (3187 to 5111)	155.6 (123.9 to 197.9)	46 (33 to 63)	1.8 (1.3 to 2.4)	64.1 (15.3 to 142.9)	15.3 (-17.1 to 65)	76.6 (7.4 to 216.4)	18.3 (-26 to 104.8)
		Male	8195 (4950 to 11455)	480.1 (287.4 to 662.1)	128 (71 to 184)	8.2 (4.6 to 11.7)	13390 (10006 to 16805)	481.9 (362.8 to 611.5)	215 (154 to 275)	7.6 (5.5 to 9.8)	63.4 (9.4 to 165.3)	0.4 (-32.1 to 61.2)	67.1 (7.3 to 197.5)	-7.3 (-40.8 to 63.9)
	Conflict and terrorism	Both	24576 (18787 to 33109)	776.6 (582.7 to 1072.1)	133 (121 to 146)	3.4 (3 to 3.7)	7996 (5199 to 12540)	136.1 (90.5 to 210.9)	2 (2 to 2)	0 (0 to 0)	-67.5 (-73.2 to -61.3)	-82.5 (-85.7 to -78.9)	-98.6 (-98.6 to -98.6)	-98.9 (-98.9 to -98.9)
		Female	5940 (3666 to 10039)	431.8 (273.6 to 700.3)	0 (0 to 0)	0 (0 to 0)	3092 (1858 to 5430)	101 (61.2 to 173.6)	1 (0 to 1)	0 (0 to 0)	-48 (-56.4 to -40.1)	-76.6 (-81.3 to -71.7)	36.3 (36.3 to 36.3)	-6 (-6 to -6)
		Male	18635 (15070 to 23629)	1107 (881.3 to 1417.6)	132 (120 to 146)	6.6 (6 to 7.2)	4904 (3286 to 7365)	170.7 (115.8 to 250.4)	1 (1 to 2)	0.1 (0 to 0.1)	-73.7 (-78.7 to -68.5)	-84.6 (-87.4 to -81.6)	-98.9 (-98.9 to -98.9)	-99.2 (-99.2 to -99.2)
	Executions and police conflict	Both	1987 (1485 to 2726)	60.2 (44.6 to 79.9)	31 (23 to 43)	1 (0.7 to 1.4)	3287 (1901 to 5515)	56.5 (34.3 to 94.5)	55 (29 to 94)	0.9 (0.5 to 1.6)	65.4 (-2.5 to 194.7)	-6.2 (-42.4 to 61.9)	75.4 (0.3 to 222.8)	-7.6 (-45.5 to 65.6)
		Female	295 (255 to 351)	18.6 (15.8 to 22.5)	4 (3 to 5)	0.3 (0.2 to 0.3)	305 (251 to 390)	11.5 (9.7 to 14.2)	4 (4 to 6)	0.2 (0.1 to 0.2)	3.6 (-12.2 to 27.9)	-37.9 (-47.2 to -25.8)	9 (-10.1 to 44.3)	-38.8 (-49.6 to -23.8)
		Male	1692 (1212 to 2422)	100.2 (70.8 to 139.8)	27 (19 to 39)	1.7 (1.2 to 2.4)	2981 (1611 to 5217)	99.4 (56.7 to 173.5)	50 (25 to 90)	1.7 (0.9 to 2.9)	76.2 (-3.3 to 225.4)	-0.8 (-42.9 to 81.4)	85.1 (-1.8 to 255.7)	-3.1 (-46.8 to 83.9)
**Gilan**	**All causes**	Both	2583871 (2379604 to 2802013)	110748.9 (102092.4 to 119969.6)	40722 (37648 to 44114)	2312.2 (2144.6 to 2491.4)	710947 (616340 to 813998)	24590.8 (21362.5 to 28075.4)	17318 (15532 to 19289)	630.3 (568.8 to 698.3)	-72.5 (-76.4 to -68.6)	-77.8 (-80.7 to -74.8)	-57.5 (-62.8 to -51.4)	-72.7 (-76 to -69)
		Female	1040363 (953652 to 1131501)	89525.5 (82141.6 to 97408.5)	15883 (14602 to 17282)	1843.9 (1682.6 to 2013.5)	334659 (280042 to 393328)	23116.9 (19391.3 to 27076.3)	7748 (6731 to 8882)	579 (507.1 to 658.8)	-67.8 (-72.8 to -62.4)	-74.2 (-77.9 to -70.3)	-51.2 (-58.8 to -42.8)	-68.6 (-73 to -63.3)

Province	Cause	Sex	1990				2019				1990-2019 percent change (%)			
			DALYs		Death		DALYs		Death		DALYs		Death	
			Number	Rate	Number	Rate	Number	Rate	Number	Rate	Number	Rate	Number	Rate
		Male	1543508 (1420008 to 1678976)	132058.8 (121197.1 to 143802.4)	24839 (22765 to 27145)	2803.7 (2565.7 to 3065.4)	376288 (324621 to 433277)	26086.9 (22589.8 to 29889.6)	9570 (8150 to 11207)	685 (587 to 799.3)	-75.6 (-79.2 to -71.7)	-80.2 (-82.9 to -77.3)	-61.5 (-68.2 to -54)	-75.6 (-79.8 to -70.9)
	**Communicable, maternal, neonatal, and nutritional diseases**	Both	193880 (147260 to 267323)	7022.8 (5405.1 to 9901.4)	2172 (1616 to 3319)	90 (69.4 to 150.5)	34518 (29063 to 40639)	1606 (1359.1 to 1892.7)	481 (414 to 626)	21 (18 to 26.4)	-82.2 (-87.4 to -75.9)	-77.1 (-84.3 to -69.3)	-77.8 (-85.8 to -68.2)	-76.7 (-86.3 to -68.3)
		Female	88687 (67067 to 121964)	6542.3 (4999 to 9029.9)	979 (735 to 1437)	81.4 (62.6 to 124.6)	16441 (13655 to 19562)	1533.3 (1273.3 to 1818.4)	213 (175 to 305)	19.1 (15.9 to 26.1)	-81.5 (-87 to -74.6)	-76.6 (-83.6 to -68.4)	-78.2 (-85.5 to -66.2)	-76.5 (-84.6 to -66.4)
		Male	105193 (77979 to 148111)	7484 (5588.3 to 10880.3)	1193 (859 to 1898)	99.3 (74.4 to 183.9)	18078 (15255 to 21328)	1676.4 (1413.3 to 1983.5)	268 (225 to 354)	22.9 (19.3 to 29.4)	-82.8 (-88.2 to -76.4)	-77.6 (-85.3 to -69.1)	-77.5 (-86.4 to -66.4)	-77 (-87.7 to -67.7)
	HIV/AIDS and sexually transmitted infections	Both	654 (333 to 1208)	27.3 (15.2 to 48.7)	5 (2 to 12)	0.2 (0.1 to 0.4)	794 (553 to 1116)	30 (20.1 to 43.7)	9 (6 to 14)	0.3 (0.2 to 0.5)	21.4 (-32.3 to 116.3)	9.9 (-30.1 to 63)	68.1 (-29 to 371.1)	60 (-21.7 to 240.7)
		Female	342 (182 to 627)	30.3 (17.1 to 53.2)	2 (1 to 5)	0.2 (0.1 to 0.4)	469 (289 to 716)	34.2 (21.2 to 50.8)	5 (3 to 9)	0.4 (0.2 to 0.6)	37 (-26.6 to 144.2)	12.9 (-30.5 to 78.1)	111.3 (-27.6 to 582.3)	90.8 (-21.8 to 373.5)
		Male	312 (148 to 619)	24.3 (12.6 to 45)	3 (1 to 6)	0.2 (0.1 to 0.5)	325 (242 to 441)	25.8 (17.7 to 38.4)	4 (3 to 5)	0.3 (0.2 to 0.4)	4.3 (-41.6 to 90.6)	6.6 (-32.8 to 60.3)	32.2 (-32.9 to 221.8)	35 (-23.5 to 164.7)
	HIV/AIDS	Both	39 (20 to 54)	1.9 (1 to 2.7)	1 (0 to 1)	0 (0 to 0)	328 (204 to 517)	10.7 (6.7 to 16.6)	6 (3 to 11)	0.2 (0.1 to 0.3)	742.5 (341.6 to 1726.7)	449.3 (186.1 to 1078.2)	996.3 (377 to 3293.5)	531.5 (170.4 to 1902)
		Female	13 (7 to 18)	1.3 (0.7 to 1.8)	0 (0 to 0)	0 (0 to 0)	180 (79 to 344)	11.8 (5.5 to 21.7)	4 (1 to 8)	0.2 (0.1 to 0.5)	1279.8 (425.1 to 3235.2)	837.4 (272.1 to 2097.7)	1808.7 (445.1 to 5978.2)	1019.7 (236 to 3422.2)
		Male	26 (13 to 36)	2.6 (1.3 to 3.7)	0 (0 to 1)	0 (0 to 0.1)	148 (115 to 205)	9.6 (7.5 to 13)	3 (2 to 4)	0.2 (0.1 to 0.2)	471.5 (266.5 to 1044.7)	261.3 (126.8 to 661.1)	582.8 (294.3 to 2070.2)	285.1 (122.6 to 1172.4)
	Sexually transmitted infections excluding HIV	Both	615 (300 to 1178)	25.4 (13.3 to 46.6)	5 (2 to 11)	0.2 (0.1 to 0.4)	466 (290 to 725)	19.3 (11.4 to 32.1)	3 (1 to 5)	0.1 (0.1 to 0.3)	-24.3 (-55.5 to 22)	-23.8 (-47.9 to -0.3)	-41.5 (-67.7 to 35.5)	-19.4 (-52.4 to 39.8)
		Female	329 (170 to 611)	29 (16 to 52)	2 (1 to 5)	0.2 (0.1 to 0.4)	289 (173 to 474)	22.4 (13.2 to 36.3)	1 (1 to 2)	0.2 (0.1 to 0.3)	-12.3 (-48 to 32.4)	-23 (-45.2 to -3.8)	-33.2 (-64 to 58)	-14.6 (-50 to 47.3)
		Male	286 (120 to 598)	21.6 (9.9 to 42.6)	3 (1 to 6)	0.2 (0.1 to 0.4)	177 (108 to 280)	16.3 (8.9 to 28.4)	1 (0 to 2)	0.1 (0.1 to 0.3)	-38.1 (-65.2 to 8.6)	-24.6 (-53.4 to 8.6)	-48.9 (-74.1 to 32.5)	-22.1 (-58.5 to 55)
	Respiratory infections and tuberculosis	Both	22693 (16734 to 32216)	932.4 (714.8 to 1250.2)	305 (232 to 410)	20 (16.4 to 28.5)	9110 (7507 to 12014)	349.3 (288.3 to 453.5)	296 (246 to 429)	11.2 (9.4 to 16.6)	-59.9 (-72 to -45.3)	-62.5 (-72.7 to -51.2)	-3.2 (-29.6 to 32.1)	-43.8 (-54.8 to -30.1)
		Female	10667 (7513 to 15654)	865.1 (626.6 to 1189)	140 (101 to 196)	17.4 (13.2 to 26.8)	3939 (3115 to 5449)	309.9 (246.3 to 424)	129 (100 to 214)	10.1 (7.9 to 16.8)	-63.1 (-75.3 to -44.9)	-64.2 (-75 to -50)	-7.7 (-37.9 to 36.3)	-41.7 (-55.4 to -22.9)
		Male	12026 (8566 to 17104)	1007.9 (762.7 to 1366.5)	165 (123 to 229)	23.6 (18.7 to 34.3)	5171 (4216 to 6736)	388.9 (315.6 to 507.2)	167 (133 to 254)	12.3 (9.9 to 19)	-57 (-70.9 to -39.1)	-61.4 (-72.7 to -48)	0.7 (-30 to 43.4)	-47.7 (-60.1 to -29)
	Tuberculosis	Both	1666 (1301 to 2060)	91 (69.1 to 112)	37 (28 to 46)	2.9 (2.1 to 3.6)	725 (622 to 842)	24 (20.7 to 27.9)	24 (21 to 29)	0.8 (0.7 to 1)	-56.5 (-66 to -40.4)	-73.6 (-79.4 to -63.5)	-34.3 (-51.5 to -4.5)	-70.8 (-78.5 to -56.8)
		Female	590 (341 to 840)	63 (35.6 to 91.2)	12 (6 to 19)	1.9 (0.9 to 2.9)	233 (192 to 292)	15.9 (13.2 to 19.9)	8 (6 to 10)	0.5 (0.4 to 0.7)	-60.5 (-74.2 to -20.7)	-74.7 (-83.4 to -48.3)	-36.1 (-62.5 to 65.3)	-71 (-83.1 to -24)
		Male	1075 (834 to 1397)	120.9 (94.6 to 154.4)	24 (19 to 32)	4 (3 to 5.1)	491 (408 to 585)	32.2 (26.8 to 38.2)	16 (13 to 20)	1.1 (0.9 to 1.3)	-54.3 (-66.5 to -37.6)	-73.4 (-80.5 to -64)	-33.5 (-53 to -6.4)	-72.3 (-79.8 to -61.3)
	Lower respiratory infections	Both	18029 (12317 to 27012)	723.4 (523.2 to 1028.5)	269 (199 to 374)	17.1 (13.8 to 25.4)	5560 (4701 to 7908)	211.3 (179.6 to 297.3)	271 (224 to 406)	10.4 (8.6 to 15.7)	-69.2 (-80.4 to -52.8)	-70.8 (-80.3 to -58.9)	1 (-29.7 to 39.8)	-39.4 (-51.9 to -24.6)

Province	Cause	Sex	1990				2019				1990-2019 percent change (%)			
			DALYs		Death		DALYs		Death		DALYs		Death	
			Number	Rate	Number	Rate	Number	Rate	Number	Rate	Number	Rate	Number	Rate
		Female	8651 (5545 to 13570)	688.6 (469.3 to 1013.3)	128 (90 to 184)	15.5 (11.6 to 25.1)	2364 (1878 to 3793)	184.7 (147.8 to 290.8)	121 (93 to 207)	9.6 (7.4 to 16.3)	-72.7 (-83.4 to -52.4)	-73.2 (-83 to -58.2)	-5.1 (-38.4 to 42.8)	-38.1 (-53.3 to -17.5)
		Male	9378 (6207 to 14350)	764.6 (541.4 to 1111.2)	141 (101 to 203)	19.6 (15.2 to 30.4)	3196 (2583 to 4435)	238.2 (194.4 to 336.6)	150 (118 to 236)	11.2 (8.9 to 17.7)	-65.9 (-79.3 to -45.6)	-68.9 (-79.9 to -53.9)	6.5 (-28.5 to 54)	-42.7 (-57.3 to -22)
	Upper respiratory infections	Both	2138 (1278 to 3391)	84.7 (51.2 to 131.4)	0 (0 to 1)	0 (0 to 0)	2126 (1287 to 3341)	85.2 (51 to 135)	0 (0 to 1)	0 (0 to 0)	-0.5 (-9.1 to 8.9)	0.6 (-4.9 to 5.9)	172.5 (-87.1 to 3894.3)	108.1 (-89.4 to 2393.3)
		Female	1040 (631 to 1628)	83 (49.8 to 128.9)	0 (0 to 0)	0 (0 to 0)	1025 (614 to 1622)	83.2 (50 to 131.4)	0 (0 to 0)	0 (0 to 0)	-1.4 (-10.6 to 9.4)	0.2 (-6.9 to 7.9)	231.7 (-90.5 to 6663)	173.1 (-91.2 to 4053)
		Male	1098 (656 to 1751)	86.4 (51.5 to 135.2)	0 (0 to 0)	0 (0 to 0)	1101 (664 to 1736)	87.2 (52.5 to 135.7)	0 (0 to 0)	0 (0 to 0)	0.3 (-9.5 to 12.2)	0.9 (-6.5 to 8.5)	142.4 (-85.3 to 3198.6)	75.1 (-88.9 to 1837.4)
	Otitis media	Both	860 (484 to 1428)	33.2 (18.8 to 55.2)	0 (0 to 0)	0 (0 to 0)	699 (396 to 1178)	28.8 (16.4 to 47.8)	0 (0 to 0)	0 (0 to 0)	-18.7 (-27.6 to -8.2)	-13.4 (-19.2 to -7.7)	-54.6 (-80.1 to 8.2)	-65.4 (-83.2 to -27.4)
		Female	385 (213 to 644)	30.4 (17.1 to 51.4)	0 (0 to 0)	0 (0 to 0)	317 (177 to 527)	26.1 (14.8 to 43)	0 (0 to 0)	0 (0 to 0)	-17.8 (-27.9 to -4.8)	-14.2 (-22.3 to -6.1)	15.2 (-59.3 to 244)	-41.3 (-77.9 to 80.5)
		Male	474 (268 to 791)	36 (20.6 to 59.2)	0 (0 to 0)	0 (0 to 0)	382 (218 to 648)	31.4 (18.1 to 52.7)	0 (0 to 0)	0 (0 to 0)	-19.5 (-29.4 to -7.9)	-12.8 (-20.6 to -5.1)	-73.4 (-88.8 to -28.8)	-76 (-89.4 to -39.3)
	Enteric infections	Both	11631 (7964 to 17367)	441.3 (310.7 to 635.4)	120 (76 to 186)	6.3 (4.1 to 9.4)	6062 (4499 to 7923)	257.1 (191 to 335.2)	42 (27 to 64)	1.7 (1.2 to 2.6)	-47.9 (-66.7 to -23.3)	-41.7 (-61.3 to -17.6)	-65.2 (-79.9 to -41.9)	-72.3 (-81.2 to -60)
		Female	5455 (3613 to 8411)	420.3 (288.1 to 630.8)	57 (34 to 94)	6.2 (3.4 to 10.6)	2911 (2144 to 3807)	249.8 (185.5 to 329.9)	19 (10 to 39)	1.7 (0.9 to 3.2)	-46.6 (-67.3 to -17.6)	-40.6 (-62.5 to -12.5)	-65.9 (-83.6 to -34.9)	-73 (-83.2 to -56.4)
		Male	6176 (3852 to 10322)	459.9 (301 to 730.1)	63 (37 to 108)	6.1 (3.8 to 9.8)	3151 (2320 to 4119)	264.4 (196.8 to 345.4)	22 (13 to 36)	1.8 (1.1 to 2.9)	-49 (-69.2 to -19.5)	-42.5 (-64.5 to -13.4)	-64.6 (-81.9 to -34.6)	-70.3 (-82.7 to -53.6)
	Diarrheal diseases	Both	9973 (6500 to 15513)	385.4 (261.5 to 568.6)	97 (58 to 161)	5.4 (3.3 to 8.4)	5520 (4066 to 7351)	230.7 (168.4 to 309.1)	33 (19 to 56)	1.3 (0.8 to 2.2)	-44.7 (-66.3 to -15.7)	-40.1 (-61.9 to -13.3)	-66 (-82 to -38.3)	-75.3 (-84.1 to -62.5)
		Female	4621 (2887 to 7328)	363.4 (235.9 to 562.1)	45 (24 to 80)	5.3 (2.6 to 9.7)	2661 (1927 to 3544)	225.2 (163.7 to 302.9)	15 (6 to 35)	1.3 (0.6 to 2.8)	-42.4 (-66.7 to -6.7)	-38 (-62.8 to -4.5)	-66.2 (-86.5 to -27.6)	-75.6 (-86.6 to -57.2)
		Male	5352 (3197 to 9209)	405.1 (256.1 to 659.1)	51 (27 to 94)	5.3 (3.1 to 9)	2858 (2076 to 3830)	236.3 (169.6 to 318.1)	18 (9 to 30)	1.4 (0.8 to 2.4)	-46.6 (-69.1 to -11.8)	-41.7 (-65.1 to -9.1)	-65.8 (-84.4 to -29.8)	-73.6 (-85.5 to -56.2)
	Typhoid and paratyphoid	Both	1315 (524 to 2656)	44 (17.9 to 85.6)	18 (7 to 35)	0.6 (0.3 to 1.2)	407 (164 to 791)	20 (7.7 to 39.9)	6 (2 to 12)	0.3 (0.1 to 0.6)	-69.1 (-77.2 to -54.5)	-54.5 (-66.5 to -32.1)	-66.1 (-74.7 to -49.1)	-55.4 (-66.4 to -33.6)
		Female	630 (237 to 1333)	42.7 (16.4 to 88.9)	9 (3 to 18)	0.6 (0.2 to 1.3)	187 (76 to 372)	18.7 (7.4 to 38.2)	3 (1 to 6)	0.3 (0.1 to 0.5)	-70.3 (-79.1 to -47.2)	-56.2 (-69.5 to -24)	-67.4 (-76.6 to -40.1)	-57.1 (-70.4 to -27.2)
		Male	684 (271 to 1351)	45.2 (18.1 to 88)	9 (4 to 18)	0.7 (0.3 to 1.3)	220 (87 to 431)	21.2 (8.3 to 43)	3 (1 to 6)	0.3 (0.1 to 0.6)	-67.9 (-77.2 to -55.4)	-53 (-66.4 to -35.6)	-64.9 (-74.3 to -49.7)	-53.9 (-66.4 to -36.3)
	Invasive Non-typhoidal Salmonella (iNTS)	Both	299 (155 to 513)	10.4 (5.4 to 17.5)	4 (2 to 7)	0.2 (0.1 to 0.3)	128 (66 to 230)	6 (3 to 10.8)	2 (1 to 4)	0.1 (0.1 to 0.2)	-57.3 (-72.2 to -38.4)	-42.3 (-62.1 to -17.6)	-42.8 (-59.8 to -19)	-41.3 (-56.6 to -20.9)
		Female	179 (88 to 314)	12.5 (6.3 to 21.5)	3 (1 to 4)	0.2 (0.1 to 0.3)	59 (30 to 104)	5.5 (2.7 to 10)	1 (1 to 2)	0.1 (0 to 0.2)	-67.2 (-79.8 to -47.4)	-56.1 (-72 to -32.5)	-53.7 (-69.6 to -27.1)	-52.5 (-66.9 to -30.6)
		Male	120 (63 to 208)	8.4 (4.5 to 14.3)	2 (1 to 3)	0.1 (0.1 to 0.2)	69 (34 to 126)	6.5 (3.2 to 11.9)	1 (1 to 2)	0.1 (0.1 to 0.2)	-42.4 (-64.2 to -17.1)	-22.5 (-50.7 to 11.3)	-27.1 (-51.1 to 3.9)	-25.5 (-48.2 to 3.7)
	Other intestinal infectious diseases	Both	44 (13 to 90)	1.5 (0.5 to 3)	1 (0 to 1)	0 (0 to 0)	8 (6 to 11)	0.4 (0.3 to 0.5)	0 (0 to 0)	0 (0 to 0)	-81.4 (-91.7 to -36.3)	-74.5 (-88.7 to -20.4)	-79.6 (-91.8 to -24.7)	-78.6 (-90.7 to -36.7)
		Female	25 (3 to 63)	1.7 (0.3 to 4.3)	0 (0 to 1)	0 (0 to 0.1)	4 (2 to 6)	0.4 (0.2 to 0.6)	0 (0 to 0)	0 (0 to 0)	-84.2 (-94.4 to 15.6)	-78 (-92.5 to 32.1)	-81.7 (-94.8 to 60.9)	-80.1 (-94.4 to 2.6)

Province	Cause	Sex	1990				2019				1990-2019 percent change (%)			
			DALYs		Death		DALYs		Death		DALYs		Death	
			Number	Rate	Number	Rate	Number	Rate	Number	Rate	Number	Rate	Number	Rate
		Male	19 (4 to 46)	1.3 (0.3 to 3)	0 (0 to 1)	0 (0 to 0)	4 (3 to 6)	0.4 (0.2 to 0.6)	0 (0 to 0)	0 (0 to 0)	-77.8 (-91.4 to 11.8)	-70.2 (-88.1 to 27)	-77 (-93.2 to 40.2)	-76.3 (-92.2 to -12.5)
	Neglected tropical diseases and malaria	Both	5653 (606 to 71465)	241.3 (24.3 to 3116.4)	93 (3 to 1315)	4.8 (0.2 to 67.2)	351 (249 to 481)	14.4 (10 to 20.6)	2 (2 to 3)	0.1 (0.1 to 0.1)	-93.8 (-99.5 to -46)	-94 (-99.5 to -43.4)	-97.8 (-99.8 to -23.5)	-98.4 (-99.9 to -49.4)
		Female	2634 (292 to 32954)	214.9 (24.4 to 2711.5)	39 (1 to 554)	3.6 (0.1 to 49.7)	179 (122 to 259)	14.5 (9.8 to 21.4)	1 (1 to 1)	0.1 (0 to 0.1)	-93.2 (-99.4 to -42.2)	-93.3 (-99.5 to -44.7)	-97.8 (-99.8 to -10.2)	-98.2 (-99.9 to -38.1)
		Male	3019 (275 to 39220)	270.6 (21.4 to 3540.9)	54 (2 to 761)	6.2 (0.2 to 87.5)	171 (119 to 252)	14.3 (9.5 to 22.4)	1 (1 to 2)	0.1 (0.1 to 0.2)	-94.3 (-99.6 to -37.1)	-94.7 (-99.6 to -32.2)	-97.8 (-99.9 to -14.1)	-98.6 (-99.9 to -43.5)
	Malaria	Both	4884 (31 to 70862)	211.1 (1.2 to 3092.3)	90 (0 to 1313)	4.6 (0 to 67.1)	29 (21 to 40)	1.1 (0.8 to 1.5)	0 (0 to 0)	0 (0 to 0)	-99.4 (-100 to -8.4)	-99.5 (-100 to -14)	-100 (-100 to -100)	-100 (-100 to -100)
		Female	2246 (17 to 32582)	183.2 (1.3 to 2681.1)	38 (0 to 553)	3.5 (0 to 49.6)	15 (11 to 20)	1.1 (0.8 to 1.5)	0 (0 to 0)	0 (0 to 0)	-99.3 (-100 to -12.2)	-99.4 (-100 to -18.7)	-100 (-100 to -100)	-100 (-100 to -100)
		Male	2638 (14 to 38688)	242 (1.1 to 3512)	52 (0 to 759)	5.9 (0 to 87.2)	14 (10 to 19)	1.1 (0.8 to 1.5)	0 (0 to 0)	0 (0 to 0)	-99.5 (-100 to -4.4)	-99.6 (-100 to -6.9)	-100 (-100 to -100)	-100 (-100 to -100)
	Leishmaniasis	Both	0 (0 to 0)	0 (0 to 0)	0 (0 to 0)	0 (0 to 0)	0 (0 to 0)	0 (0 to 0)	0 (0 to 0)	0 (0 to 0)	-42.5 (-84.5 to 170)	-42 (-84.2 to 176.9)	0 (0 to 0)	0 (0 to 0)
		Female	0 (0 to 0)	0 (0 to 0)	0 (0 to 0)	0 (0 to 0)	0 (0 to 0)	0 (0 to 0)	0 (0 to 0)	0 (0 to 0)	-37.7 (-83.1 to 200.9)	-42.8 (-84.5 to 174.7)	0 (0 to 0)	0 (0 to 0)
		Male	0 (0 to 0)	0 (0 to 0)	0 (0 to 0)	0 (0 to 0)	0 (0 to 0)	0 (0 to 0)	0 (0 to 0)	0 (0 to 0)	-50.5 (-86.5 to 123.6)	-39.8 (-83.7 to 175.6)	0 (0 to 0)	0 (0 to 0)
	Schistosomiasis	Both	0 (0 to 0)	0 (0 to 0)	0 (0 to 0)	0 (0 to 0)	0 (0 to 0)	0 (0 to 0)	0 (0 to 0)	0 (0 to 0)	0 (0 to 0)	0 (0 to 0)	0 (0 to 0)	0 (0 to 0)
		Female	0 (0 to 0)	0 (0 to 0)	0 (0 to 0)	0 (0 to 0)	0 (0 to 0)	0 (0 to 0)	0 (0 to 0)	0 (0 to 0)	0 (0 to 0)	0 (0 to 0)	0 (0 to 0)	0 (0 to 0)
		Male	0 (0 to 0)	0 (0 to 0)	0 (0 to 0)	0 (0 to 0)	0 (0 to 0)	0 (0 to 0)	0 (0 to 0)	0 (0 to 0)	0 (0 to 0)	0 (0 to 0)	0 (0 to 0)	0 (0 to 0)
	Cystic echinococcosis	Both	131 (78 to 196)	6 (3.8 to 8.4)	2 (1 to 3)	0.1 (0.1 to 0.2)	79 (54 to 112)	2.6 (1.8 to 3.8)	1 (0 to 1)	0 (0 to 0)	-39.8 (-63.6 to 0.3)	-55.8 (-71.7 to -31.9)	-64.5 (-83.7 to -25.8)	-79.1 (-89.9 to -57.9)
		Female	56 (30 to 91)	5.2 (3 to 8)	1 (0 to 1)	0.1 (0 to 0.1)	43 (28 to 62)	2.8 (1.8 to 4.1)	0 (0 to 1)	0 (0 to 0)	-24.4 (-59.1 to 42)	-46.3 (-67.5 to -10.5)	-58.2 (-88 to 38.5)	-75.1 (-92.6 to -28.8)
		Male	75 (37 to 122)	6.7 (3.7 to 10.2)	1 (0 to 2)	0.1 (0.1 to 0.2)	36 (24 to 53)	2.5 (1.6 to 3.6)	0 (0 to 1)	0 (0 to 0)	-51.4 (-74.2 to 4.9)	-63.2 (-79 to -30.8)	-68.4 (-89.4 to -7)	-81.9 (-93.4 to -50.2)
	Trachoma	Both	17 (9 to 31)	1.6 (0.8 to 2.7)	--	--	8 (4 to 14)	0.3 (0.1 to 0.5)	--	--	-54.2 (-65.7 to -39.1)	-83.1 (-87.2 to -78)	--	--
		Female	13 (7 to 23)	2.4 (1.2 to 4.1)	--	--	5 (3 to 10)	0.4 (0.2 to 0.7)	--	--	-59.2 (-70.5 to -42.8)	-84.4 (-88.8 to -78.6)	--	--
		Male	4 (2 to 8)	0.7 (0.3 to 1.3)	--	--	2 (1 to 4)	0.2 (0.1 to 0.3)	--	--	-36.6 (-58.4 to 0.9)	-75.8 (-83 to -65.2)	--	--
	Rabies	Both	11 (2 to 32)	0.5 (0.1 to 1.4)	0 (0 to 1)	0 (0 to 0)	7 (2 to 10)	0.2 (0.1 to 0.4)	0 (0 to 0)	0 (0 to 0)	-41.5 (-80.6 to 247.9)	-53.2 (-83.8 to 166.1)	-27.2 (-75 to 318.9)	-52.4 (-83.3 to 164.6)
		Female	1 (0 to 3)	0.1 (0 to 0.3)	0 (0 to 0)	0 (0 to 0)	1 (0 to 2)	0.1 (0 to 0.1)	0 (0 to 0)	0 (0 to 0)	-14.5 (-72.2 to 501.9)	-52.7 (-84.6 to 252.9)	-11.7 (-72.3 to 525.1)	-56.4 (-85.6 to 230.7)
		Male	10 (1 to 31)	0.9 (0.1 to 2.7)	0 (0 to 1)	0 (0 to 0.1)	6 (1 to 9)	0.4 (0.1 to 0.7)	0 (0 to 0)	0 (0 to 0)	-44.2 (-84.4 to 277.6)	-53.4 (-87 to 206.3)	-29.4 (-79.5 to 367.4)	-52.1 (-86.3 to 199.3)
	Intestinal nematode infections	Both	111 (59 to 181)	4.2 (2.3 to 6.8)	1 (0 to 1)	0 (0 to 0)	21 (11 to 37)	0.8 (0.4 to 1.5)	0 (0 to 0)	0 (0 to 0)	-81.3 (-90.4 to -66.2)	-79.9 (-88.7 to -65.6)	-89.5 (-96.1 to -54.6)	-88.4 (-95.4 to -64.3)

Province	Cause	Sex	1990				2019				1990-2019 percent change (%)			
			DALYs		Death		DALYs		Death		DALYs		Death	
			Number	Rate	Number	Rate	Number	Rate	Number	Rate	Number	Rate	Number	Rate
		Female	65 (31 to 114)	5.2 (2.6 to 8.8)	0 (0 to 1)	0 (0 to 0)	13 (6 to 24)	1 (0.5 to 1.8)	0 (0 to 0)	0 (0 to 0)	-80.1 (-90.1 to -61)	-80 (-89.5 to -63.6)	-88.2 (-97.6 to 8.9)	-86.9 (-97.2 to -29.4)
		Male	46 (18 to 90)	3.2 (1.4 to 5.9)	0 (0 to 1)	0 (0 to 0)	8 (4 to 14)	0.6 (0.3 to 1.1)	0 (0 to 0)	0 (0 to 0)	-83 (-93.1 to -58.9)	-79.5 (-91.4 to -55.9)	-90.3 (-97.4 to -21.7)	-89.5 (-97 to -53.9)
	Food-borne trematodiases	Both	9 (2 to 18)	0.5 (0.1 to 1)	--	--	31 (7 to 65)	1 (0.2 to 2.1)	--	--	252.8 (158.6 to 363.4)	107.8 (59 to 164.7)	--	--
		Female	4 (1 to 8)	0.4 (0.1 to 0.8)	--	--	12 (3 to 25)	0.8 (0.2 to 1.7)	--	--	238.6 (138.1 to 351.1)	98.8 (45.2 to 160.6)	--	--
		Male	5 (1 to 11)	0.6 (0.1 to 1.2)	--	--	19 (4 to 40)	1.2 (0.3 to 2.6)	--	--	262.8 (135.8 to 430.7)	114 (43.9 to 206.8)	--	--
	Leprosy	Both	1 (0 to 1)	0 (0 to 0.1)	--	--	1 (1 to 1)	0 (0 to 0)	--	--	80.3 (63.8 to 97.7)	-14 (-21 to -7.1)	--	--
		Female	0 (0 to 0)	0 (0 to 0)	--	--	0 (0 to 1)	0 (0 to 0)	--	--	65.4 (45.5 to 87.3)	-21.9 (-30.9 to -12.7)	--	--
		Male	0 (0 to 1)	0 (0 to 0.1)	--	--	1 (0 to 1)	0 (0 to 0.1)	--	--	88.6 (68.1 to 112.7)	-10.4 (-19.9 to 1.1)	--	--
	Other neglected tropical diseases	Both	489 (293 to 756)	17.4 (10.8 to 26.5)	1 (1 to 3)	0.1 (0 to 0.2)	176 (112 to 266)	8.3 (5.1 to 13.1)	1 (1 to 2)	0 (0 to 0.1)	-64.1 (-76.6 to -44.5)	-52.5 (-68.8 to -27.8)	-3.5 (-48.1 to 72)	-31.3 (-55.9 to 14.3)
		Female	248 (140 to 404)	18.3 (11 to 28.6)	1 (0 to 1)	0.1 (0 to 0.2)	90 (54 to 147)	8.3 (4.7 to 13.9)	1 (0 to 1)	0 (0 to 0.1)	-63.8 (-78 to -39.8)	-55 (-72.6 to -28.4)	-8.8 (-47.6 to 79.3)	-34.3 (-58.1 to 19.8)
		Male	240 (117 to 426)	16.4 (8.8 to 27.4)	1 (0 to 2)	0.1 (0 to 0.2)	86 (48 to 150)	8.3 (4.2 to 15.3)	1 (0 to 2)	0.1 (0 to 0.1)	-64.4 (-81.8 to -30.6)	-49.6 (-73.7 to -3.5)	0.8 (-52 to 113.5)	-28.9 (-58.9 to 35.5)
	Other infectious diseases	Both	22235 (11101 to 44043)	745.1 (390.5 to 1444.9)	268 (136 to 516)	10.1 (5.9 to 17.9)	2258 (1933 to 2699)	92 (76.6 to 117.4)	50 (42 to 58)	1.9 (1.6 to 2.2)	-89.8 (-94.8 to -79.1)	-87.7 (-93.6 to -76.1)	-81.5 (-90.1 to -63.1)	-81.5 (-89.4 to -67.6)
		Female	11675 (5641 to 23221)	788.9 (398.3 to 1548.4)	139 (68 to 270)	10.3 (5.7 to 19)	1003 (810 to 1231)	85 (66.4 to 111.7)	20 (16 to 24)	1.5 (1.3 to 1.9)	-91.4 (-95.8 to -81.9)	-89.2 (-94.8 to -78.1)	-85.8 (-92.7 to -70)	-85 (-91.7 to -72.3)
		Male	10560 (5257 to 20541)	702.8 (375.3 to 1341.4)	130 (68 to 249)	9.9 (5.8 to 17.2)	1255 (1035 to 1514)	99.2 (81.6 to 123.7)	30 (23 to 37)	2.2 (1.7 to 2.7)	-88.1 (-93.8 to -76.2)	-85.9 (-92.6 to -73)	-76.8 (-87.4 to -54.5)	-77.7 (-87.1 to -61.5)
	Meningitis	Both	2613 (1860 to 3533)	98.3 (72.7 to 129.5)	35 (26 to 46)	1.7 (1.3 to 2.2)	672 (574 to 789)	26.1 (22.3 to 30.9)	15 (13 to 19)	0.6 (0.5 to 0.7)	-74.3 (-81.8 to -62)	-73.5 (-81.1 to -61.5)	-57.1 (-69.4 to -36.6)	-67 (-75.7 to -53.4)
		Female	1312 (878 to 1921)	99.8 (69.7 to 141.2)	18 (12 to 25)	1.7 (1.3 to 2.3)	335 (275 to 399)	26.3 (21.7 to 31.4)	7 (5 to 8)	0.5 (0.4 to 0.6)	-74.5 (-83.3 to -61.4)	-73.7 (-82.3 to -61.5)	-61.3 (-73.7 to -43.2)	-69.6 (-79.2 to -57)
		Male	1301 (896 to 1865)	96.7 (68.7 to 134.9)	18 (13 to 25)	1.7 (1.3 to 2.2)	337 (270 to 429)	25.9 (20.7 to 33)	8 (6 to 11)	0.6 (0.5 to 0.8)	-74.1 (-83.2 to -57.4)	-73.2 (-82.5 to -57.1)	-53 (-69.8 to -22)	-64.1 (-75.8 to -42.5)
	Encephalitis	Both	243 (156 to 489)	10 (6.8 to 19.4)	4 (2 to 7)	0.2 (0.1 to 0.4)	267 (213 to 383)	10.6 (8.3 to 15.8)	5 (4 to 7)	0.2 (0.2 to 0.3)	9.6 (-31.2 to 64.9)	6.2 (-28 to 52.5)	44.5 (-14.5 to 127)	-1 (-35.3 to 48.9)
		Female	95 (49 to 249)	7.8 (4.4 to 19.5)	1 (1 to 4)	0.1 (0.1 to 0.4)	100 (69 to 191)	8 (5.4 to 16.1)	1 (1 to 3)	0.1 (0.1 to 0.3)	5.8 (-34.9 to 72.2)	1.6 (-33.2 to 52.7)	15.8 (-24.6 to 96.9)	-17.8 (-42.6 to 28.5)
		Male	149 (98 to 258)	12.2 (8.4 to 19.7)	2 (2 to 4)	0.3 (0.2 to 0.4)	166 (135 to 214)	13.2 (10.6 to 17.1)	4 (3 to 5)	0.3 (0.2 to 0.3)	12 (-32.2 to 76.1)	8.4 (-32.3 to 62.6)	60.5 (-11.3 to 154.8)	4.6 (-37.9 to 65.3)
	Diphtheria	Both	41 (19 to 79)	1.4 (0.6 to 2.6)	1 (0 to 1)	0 (0 to 0)	1 (1 to 2)	0.1 (0 to 0.1)	0 (0 to 0)	0 (0 to 0)	-96.9 (-98.6 to -92.3)	-95.7 (-98.2 to -89.5)	-95.7 (-98.1 to -90)	-94.9 (-97.7 to -88.7)

Province	Cause	Sex	1990				2019				1990-2019 percent change (%)			
			DALYs		Death		DALYs		Death		DALYs		Death	
			Number	Rate	Number	Rate	Number	Rate	Number	Rate	Number	Rate	Number	Rate
		Female	18 (5 to 41)	1.2 (0.4 to 2.7)	0 (0 to 0)	0 (0 to 0)	0 (0 to 1)	0 (0 to 0.1)	0 (0 to 0)	0 (0 to 0)	-97.5 (-99.1 to -90.1)	-96.5 (-98.8 to -86)	-96.6 (-98.8 to -87.1)	-95.8 (-98.5 to -84.9)
		Male	23 (8 to 50)	1.5 (0.5 to 3.2)	0 (0 to 1)	0 (0 to 0)	1 (0 to 1)	0.1 (0 to 0.1)	0 (0 to 0)	0 (0 to 0)	-96.3 (-98.8 to -87.8)	-95.2 (-98.4 to -84.8)	-94.9 (-98.2 to -83.8)	-94.2 (-97.9 to -83.3)
	Whooping cough	Both	5273 (232 to 20388)	170.1 (7.5 to 659.2)	60 (2 to 235)	1.9 (0 to 7.6)	95 (8 to 398)	6.2 (0.5 to 26.1)	1 (0 to 5)	0.1 (0 to 0.3)	-98.2 (-99.9 to -62.7)	-96.4 (-99.8 to -24.1)	-98.2 (-99.9 to -45.4)	-96.5 (-99.9 to 7.5)
		Female	3054 (135 to 11460)	200.9 (8.8 to 756.8)	35 (1 to 132)	2.3 (0.1 to 8.7)	55 (5 to 231)	7.3 (0.6 to 31)	1 (0 to 3)	0.1 (0 to 0.4)	-98.2 (-99.9 to -61.9)	-96.4 (-99.8 to -22.2)	-98.2 (-99.9 to -45.4)	-96.5 (-99.9 to 8.6)
		Male	2219 (100 to 8930)	140.6 (6.3 to 565.9)	25 (1 to 102)	1.6 (0 to 6.5)	40 (4 to 170)	5.1 (0.5 to 21.8)	0 (0 to 2)	0.1 (0 to 0.3)	-98.2 (-99.9 to -64)	-96.4 (-99.8 to -27.2)	-98.2 (-99.9 to -46.5)	-96.5 (-99.9 to 6)
	Tetanus	Both	355 (119 to 1160)	13.4 (5.4 to 41)	5 (2 to 14)	0.2 (0.1 to 0.5)	23 (17 to 31)	1 (0.8 to 1.4)	1 (0 to 1)	0 (0 to 0)	-93.4 (-98.2 to -78.9)	-92.3 (-97.7 to -77.9)	-88 (-96.7 to -67.5)	-89.9 (-96.5 to -75.6)
		Female	174 (44 to 638)	13.1 (3.8 to 46)	2 (1 to 7)	0.2 (0.1 to 0.6)	10 (8 to 13)	0.9 (0.7 to 1.2)	0 (0 to 0)	0 (0 to 0)	-94 (-98.5 to -75.3)	-92.8 (-98.2 to -73.9)	-88.7 (-97.3 to -64.2)	-90.3 (-97.1 to -73)
		Male	182 (55 to 653)	13.7 (4.6 to 45.1)	3 (1 to 8)	0.2 (0.1 to 0.6)	13 (8 to 19)	1.1 (0.7 to 1.7)	0 (0 to 1)	0 (0 to 0)	-92.9 (-98.3 to -73.7)	-91.8 (-97.8 to -71.6)	-87.3 (-97.1 to -54.8)	-89.6 (-97 to -66)
	Measles	Both	12009 (3883 to 26509)	375 (120.7 to 832)	140 (45 to 308)	4.4 (1.4 to 9.7)	96 (29 to 233)	6.1 (1.8 to 14.7)	1 (0 to 3)	0.1 (0 to 0.2)	-99.2 (-99.7 to -98.4)	-98.4 (-99.3 to -96.8)	-99.2 (-99.7 to -98.4)	-98.4 (-99.3 to -96.9)
		Female	6270 (2040 to 13695)	399.4 (129.1 to 871.2)	73 (24 to 160)	4.6 (1.5 to 10.2)	50 (15 to 121)	6.5 (1.9 to 15.7)	1 (0 to 1)	0.1 (0 to 0.2)	-99.2 (-99.7 to -98.4)	-98.4 (-99.3 to -96.7)	-99.2 (-99.7 to -98.4)	-98.4 (-99.3 to -96.8)
		Male	5739 (1864 to 13221)	351.6 (113.2 to 810.2)	67 (22 to 154)	4.1 (1.3 to 9.4)	46 (14 to 113)	5.7 (1.7 to 14.1)	1 (0 to 1)	0.1 (0 to 0.2)	-99.2 (-99.7 to -98.4)	-98.4 (-99.3 to -96.8)	-99.2 (-99.7 to -98.4)	-98.4 (-99.4 to -96.8)
	Varicella and herpes zoster	Both	210 (130 to 344)	9 (6.2 to 13.3)	3 (2 to 4)	0.2 (0.1 to 0.3)	112 (78 to 154)	4.3 (3.1 to 5.9)	1 (1 to 2)	0.1 (0 to 0.1)	-46.6 (-69 to -9.5)	-52 (-69.4 to -29)	-46.1 (-69.7 to -3.4)	-68.8 (-81.2 to -48.8)
		Female	108 (59 to 195)	9.3 (5.8 to 15.1)	1 (1 to 2)	0.2 (0.1 to 0.3)	57 (39 to 79)	4.4 (3 to 6.2)	1 (0 to 1)	0.1 (0 to 0.1)	-47.8 (-73.7 to -2.4)	-52.6 (-73.5 to -21.2)	-50.8 (-77.3 to 2.6)	-68.8 (-84.4 to -38.6)
		Male	101 (54 to 184)	8.7 (5.5 to 14.1)	1 (1 to 2)	0.2 (0.1 to 0.3)	55 (37 to 78)	4.3 (2.9 to 5.9)	1 (0 to 1)	0.1 (0 to 0.1)	-45.3 (-72 to 2.8)	-51.3 (-72.4 to -19.6)	-40.7 (-74.1 to 33.1)	-68.6 (-84.6 to -36.3)
	Acute hepatitis	Both	668 (473 to 929)	33.5 (24.1 to 48)	13 (9 to 19)	0.9 (0.6 to 1.3)	342 (279 to 415)	11.8 (9.6 to 14.4)	8 (6 to 10)	0.3 (0.2 to 0.3)	-48.8 (-64.6 to -25.9)	-64.8 (-76.1 to -48.6)	-38.2 (-62.3 to -1.5)	-70 (-81.9 to -51.4)
		Female	260 (162 to 381)	24.5 (14.7 to 35.8)	4 (2 to 7)	0.6 (0.3 to 0.9)	100 (76 to 127)	7.5 (5.6 to 9.6)	2 (1 to 2)	0.1 (0.1 to 0.2)	-61.7 (-76.2 to -35.4)	-69.5 (-80.8 to -46.9)	-58.7 (-76.7 to -13.5)	-79.3 (-88.5 to -54.6)
		Male	408 (272 to 639)	42.2 (28.5 to 67.8)	9 (6 to 15)	1.2 (0.7 to 2)	243 (191 to 304)	16.2 (12.9 to 20.1)	6 (5 to 8)	0.4 (0.3 to 0.5)	-40.5 (-64.5 to -6.9)	-61.7 (-77.1 to -39.7)	-28 (-61.4 to 23.2)	-65 (-81.6 to -40.5)
	Other unspecified infectious diseases	Both	822 (533 to 1460)	34.4 (22.8 to 58.8)	9 (5 to 18)	0.6 (0.4 to 1.1)	650 (479 to 799)	25.8 (19.3 to 32.8)	17 (11 to 21)	0.6 (0.4 to 0.8)	-20.9 (-62.4 to 26.5)	-24.8 (-61.7 to 13.4)	91.7 (-28.8 to 272.8)	7 (-52.4 to 103.5)
		Female	384 (245 to 666)	32.8 (21.3 to 54)	4 (2 to 8)	0.5 (0.3 to 1.2)	296 (213 to 379)	24 (17 to 31.7)	7 (5 to 10)	0.6 (0.4 to 0.7)	-22.9 (-64.1 to 24.5)	-26.9 (-63.4 to 12.7)	86.8 (-27.8 to 288.9)	2.6 (-56 to 123.2)
		Male	438 (267 to 845)	35.6 (23.2 to 65.4)	5 (3 to 11)	0.6 (0.4 to 1.2)	354 (248 to 469)	27.7 (19.9 to 38.3)	10 (5 to 13)	0.7 (0.4 to 0.9)	-19.2 (-64.4 to 44.7)	-22.3 (-62.9 to 31.1)	95.6 (-34.5 to 300.5)	12.3 (-55.5 to 109.1)
	Maternal and neonatal disorders	Both	123403 (92191 to 161596)	4334.4 (3244.8 to 5671.3)	1369 (1016 to 1803)	48.1 (35.9 to 63.3)	12602 (10362 to 15128)	722.6 (581 to 884.8)	79 (59 to 103)	5.6 (4.2 to 7.3)	-89.8 (-92.8 to -85.8)	-83.3 (-88.4 to -76.6)	-94.2 (-96.1 to -91.5)	-88.4 (-92.3 to -82.8)
		Female	53826 (40036 to 71550)	3884.8 (2896.8 to 5145.2)	597 (442 to 799)	43.2 (32.1 to 57.6)	6044 (4906 to 7242)	685.5 (538.9 to 840.3)	37 (27 to 47)	5.2 (3.7 to 6.7)	-88.8 (-92.2 to -84.5)	-82.4 (-87.9 to -75.4)	-93.8 (-96 to -91)	-88 (-92.4 to -82.3)
		Male	69577 (50657 to 91686)	4758.8 (3466.4 to 6268.6)	773 (560 to 1019)	52.8 (38.3 to 69.6)	6558 (5293 to 7980)	757.3 (590.9 to 940.3)	43 (30 to 56)	6 (4.2 to 7.9)	-90.6 (-93.5 to -86.3)	-84.1 (-89.3 to -76.9)	-94.5 (-96.7 to -91.5)	-88.7 (-93.1 to -82.5)

Province	Cause	Sex	1990				2019				1990-2019 percent change (%)			
			DALYs		Death		DALYs		Death		DALYs		Death	
			Number	Rate	Number	Rate	Number	Rate	Number	Rate	Number	Rate	Number	Rate
	Maternal disorders	Female	1538 (919 to 2132)	140.4 (85.2 to 194.4)	20 (10 to 30)	2 (1 to 2.9)	302 (234 to 382)	21.1 (16.5 to 26.9)	3 (2 to 4)	0.2 (0.2 to 0.3)	-80.3 (-86.7 to -65.6)	-85 (-89.9 to -74.7)	-83.5 (-89.9 to -65.4)	-88.7 (-93.2 to -78)
	Neonatal disorders	Both	121865 (90608 to 160106)	4263.9 (3171.8 to 5604.2)	1349 (994 to 1781)	47.2 (34.8 to 62.3)	12300 (10054 to 14815)	712.2 (572.4 to 874.3)	76 (56 to 99)	5.5 (4.1 to 7.2)	-89.9 (-92.9 to -85.9)	-83.3 (-88.4 to -76.5)	-94.4 (-96.3 to -91.6)	-88.4 (-92.4 to -82.7)
		Female	52288 (38542 to 70016)	3744.4 (2763 to 5005.7)	576 (422 to 778)	41.2 (30.2 to 55.6)	5742 (4636 to 6924)	664.4 (517 to 820.8)	33 (23 to 44)	5 (3.5 to 6.5)	-89 (-92.4 to -84.6)	-82.3 (-88.1 to -74.9)	-94.2 (-96.4 to -91.2)	-88 (-92.6 to -81.7)
		Male	69577 (50657 to 91686)	4758.8 (3466.4 to 6268.6)	773 (560 to 1019)	52.8 (38.3 to 69.6)	6558 (5293 to 7980)	757.3 (590.9 to 940.3)	43 (30 to 56)	6 (4.2 to 7.9)	-90.6 (-93.5 to -86.3)	-84.1 (-89.3 to -76.9)	-94.5 (-96.7 to -91.5)	-88.7 (-93.1 to -82.5)
	Nutritional deficiencies	Both	7611 (5038 to 10989)	301 (204.3 to 426.1)	11 (7 to 16)	0.6 (0.4 to 0.8)	3341 (2103 to 4939)	140.6 (87.7 to 213.6)	4 (3 to 5)	0.2 (0.1 to 0.2)	-56.1 (-67.7 to -40.7)	-53.3 (-64.6 to -37.9)	-62.7 (-75.7 to -40.1)	-71.8 (-80 to -58.7)
		Female	4087 (2600 to 5969)	338 (220.5 to 484.9)	6 (4 to 9)	0.6 (0.4 to 0.9)	1895 (1155 to 2924)	154.5 (94.2 to 234.8)	2 (2 to 3)	0.2 (0.1 to 0.2)	-53.6 (-68.1 to -32.9)	-54.3 (-67.4 to -36.2)	-62.6 (-78.5 to -36)	-69.4 (-81.5 to -50.5)
		Male	3524 (2126 to 5636)	259.7 (164.3 to 398.3)	5 (3 to 7)	0.5 (0.3 to 0.7)	1446 (872 to 2328)	126.4 (73.1 to 207.5)	2 (1 to 2)	0.1 (0.1 to 0.2)	-59 (-74.9 to -36.5)	-51.3 (-69.9 to -23.4)	-62.9 (-77.7 to -31.8)	-74.1 (-82.1 to -58.4)
	Protein-energy malnutrition	Both	1478 (1061 to 2012)	54.1 (39.2 to 73)	10 (6 to 14)	0.5 (0.4 to 0.7)	719 (447 to 1066)	31.2 (19.7 to 45.5)	2 (2 to 3)	0.1 (0.1 to 0.1)	-51.4 (-67.8 to -34.1)	-42.4 (-60.1 to -23.7)	-76.2 (-84.9 to -61)	-81 (-87 to -72.1)
		Female	771 (533 to 1098)	56.7 (39.8 to 79.1)	5 (3 to 9)	0.5 (0.4 to 0.8)	346 (216 to 515)	30.4 (18.9 to 44.6)	1 (1 to 2)	0.1 (0.1 to 0.1)	-55.1 (-71.8 to -34.4)	-46.5 (-65.7 to -23.8)	-75.8 (-86.7 to -57.6)	-79.3 (-87.8 to -65.9)
		Male	707 (484 to 972)	51.6 (36.4 to 70.8)	4 (2 to 6)	0.4 (0.3 to 0.6)	373 (228 to 576)	31.9 (19.7 to 49.3)	1 (1 to 1)	0.1 (0.1 to 0.1)	-47.3 (-66 to -24.5)	-38.1 (-59 to -13)	-76.7 (-86.6 to -56.2)	-82.6 (-88.5 to -71.2)
	Iodine deficiency	Both	352 (221 to 523)	16.1 (10.5 to 23.8)	--	--	186 (109 to 296)	6.5 (3.8 to 10.2)	--	--	-47.2 (-61.5 to -31.6)	-59.8 (-70.5 to -49)	--	--
		Female	217 (136 to 324)	19.9 (12.5 to 29.1)	--	--	116 (67 to 185)	8.2 (4.7 to 12.9)	--	--	-46.6 (-63.5 to -26.9)	-59 (-71.5 to -45.3)	--	--
		Male	135 (82 to 204)	12.4 (7.8 to 18.4)	--	--	70 (41 to 113)	4.9 (2.8 to 7.9)	--	--	-48.2 (-63.3 to -31.3)	-60.8 (-71.3 to -50.1)	--	--
	Vitamin A deficiency	Both	279 (134 to 500)	8.4 (4.1 to 15.1)	--	--	9 (4 to 20)	0.6 (0.2 to 1.3)	--	--	-96.8 (-98.8 to -91.8)	-93.4 (-97.5 to -83.3)	--	--
		Female	104 (45 to 198)	6.4 (2.8 to 12)	--	--	4 (1 to 10)	0.5 (0.2 to 1.2)	--	--	-96.2 (-98.8 to -89.4)	-92.1 (-97.4 to -78.8)	--	--
		Male	174 (65 to 356)	10.3 (3.9 to 20.8)	--	--	5 (1 to 13)	0.6 (0.1 to 1.6)	--	--	-97.2 (-99.3 to -89)	-94.1 (-98.5 to -78.2)	--	--
	Dietary iron deficiency	Both	5249 (3052 to 8184)	211.3 (127.4 to 323.2)	--	--	2173 (1221 to 3469)	92.8 (52.6 to 155)	--	--	-58.6 (-73.4 to -35.8)	-56.1 (-70.2 to -34.4)	--	--
		Female	2879 (1599 to 4637)	244.9 (139.4 to 378.8)	--	--	1307 (692 to 2213)	106.2 (57 to 183.9)	--	--	-54.6 (-72.8 to -24.2)	-56.6 (-72.5 to -32.4)	--	--
		Male	2371 (1147 to 4326)	173.3 (88.6 to 299.7)	--	--	866 (423 to 1626)	79.1 (36 to 155.6)	--	--	-63.5 (-81.5 to -26.9)	-54.3 (-77.5 to -11.3)	--	--
	Other nutritional deficiencies	Both	254 (164 to 371)	11.1 (7.3 to 16)	1 (1 to 1)	0.1 (0 to 0.1)	255 (161 to 378)	9.5 (6.1 to 14.1)	2 (1 to 2)	0.1 (0.1 to 0.1)	0.5 (-22.9 to 29)	-14 (-33.3 to 9.3)	69.1 (11.2 to 173.3)	-10.2 (-41.5 to 36.9)
		Female	116 (73 to 175)	10.1 (6.3 to 14.9)	0 (0 to 1)	0.1 (0 to 0.1)	123 (75 to 188)	9.2 (5.7 to 14.3)	1 (1 to 1)	0.1 (0.1 to 0.1)	5.3 (-25.1 to 46.8)	-8.6 (-33.7 to 26.1)	95 (-1.3 to 292.1)	12.1 (-42.2 to 119.5)
		Male	137 (86 to 215)	12.1 (7.7 to 18.4)	1 (0 to 1)	0.1 (0 to 0.1)	132 (80 to 204)	9.8 (6 to 15.2)	1 (0 to 1)	0.1 (0 to 0.1)	-3.6 (-33.2 to 37.4)	-18.8 (-42.8 to 14.6)	46.9 (-19.8 to 167.1)	-29.6 (-55.4 to 19.7)

Province	Cause	Sex	1990				2019				1990-2019 percent change (%)			
			DALYs		Death		DALYs		Death		DALYs		Death	
			Number	Rate	Number	Rate	Number	Rate	Number	Rate	Number	Rate	Number	Rate
	**Non-communicable diseases**	Both	424758 (355008 to 494068)	26154.4 (22540.7 to 29882)	7830 (6581 to 8988)	791.9 (695 to 890)	587831 (505052 to 679199)	19867 (17071.8 to 22893.2)	15476 (13872 to 17216)	561 (505.8 to 621.4)	38.4 (20.4 to 60.8)	-24 (-33.1 to -13.5)	97.6 (65 to 142.3)	-29.2 (-39.6 to -16.3)
		Female	206841 (169688 to 245797)	25211.7 (21261.8 to 29396.7)	3549 (2913 to 4243)	714.7 (602.4 to 839.3)	294010 (245114 to 346707)	19908.8 (16613.6 to 23469.1)	7224 (6263 to 8252)	537.5 (469.3 to 611.1)	42.1 (22 to 67)	-21 (-31.7 to -8.8)	103.6 (59.9 to 161.4)	-24.8 (-39.4 to -5.7)
		Male	217917 (176478 to 256944)	27086 (22493.3 to 31929.4)	4281 (3457 to 5211)	873 (734.1 to 1032.8)	293821 (250710 to 341292)	19879.2 (17016.5 to 23088)	8252 (7002 to 9662)	588.3 (502.3 to 685.8)	34.8 (10.9 to 66.4)	-26.6 (-39.4 to -10.9)	92.7 (46.7 to 160.8)	-32.6 (-47.4 to -12.2)
	Neoplasms	Both	39250 (31114 to 46739)	2563.1 (2064.6 to 3031.9)	1215 (980 to 1441)	100.6 (82.8 to 117.3)	75714 (65543 to 86533)	2437.1 (2113.2 to 2779.3)	2962 (2569 to 3378)	99.8 (86.6 to 113.6)	92.9 (54.8 to 156)	-4.9 (-22.5 to 24.6)	143.8 (97.9 to 221.7)	-0.8 (-18.9 to 27.7)
		Female	16527 (12096 to 20835)	2103.1 (1589 to 2648.9)	485 (370 to 612)	77.6 (61.3 to 96.8)	32545 (27260 to 38649)	2054.8 (1730.4 to 2431.6)	1203 (1011 to 1426)	79.5 (67 to 93.9)	96.9 (45.1 to 186.1)	-2.3 (-26.8 to 38.3)	148.3 (87 to 253.8)	2.4 (-21.2 to 40.9)
		Male	22723 (16965 to 28669)	3076.7 (2338.7 to 3851.3)	730 (549 to 919)	129.7 (100.8 to 158.6)	43169 (35025 to 51798)	2832.3 (2317.3 to 3384.3)	1759 (1445 to 2096)	120.6 (99.7 to 143.4)	90 (38.7 to 173.8)	-7.9 (-31.8 to 32)	140.8 (76.5 to 247.4)	-7.1 (-29.9 to 30.4)
	Lip and oral cavity cancer	Both	219 (166 to 285)	15.5 (11.7 to 19.9)	8 (6 to 10)	0.6 (0.5 to 0.8)	478 (404 to 560)	15.2 (12.9 to 17.7)	20 (17 to 23)	0.7 (0.6 to 0.8)	117.9 (55 to 214.7)	-1.9 (-29.3 to 39.6)	163.5 (85 to 282.1)	4 (-25.5 to 49.7)
		Female	72 (51 to 96)	10 (7.2 to 13.4)	2 (2 to 3)	0.4 (0.3 to 0.5)	201 (162 to 249)	12.7 (10.3 to 15.6)	8 (7 to 10)	0.6 (0.5 to 0.7)	180.9 (96.5 to 337.7)	27.2 (-10.4 to 90.4)	250.2 (147.7 to 427.9)	39.3 (-1.2 to 104.8)
		Male	148 (101 to 208)	21.4 (14.9 to 29.7)	5 (3 to 7)	0.9 (0.7 to 1.3)	276 (215 to 345)	17.7 (13.9 to 21.8)	11 (9 to 14)	0.8 (0.6 to 1)	87.3 (17.5 to 204.2)	-17.3 (-47.1 to 31)	123.6 (40.9 to 265.4)	-16.9 (-45.7 to 30.7)
	Nasopharynx cancer	Both	125 (92 to 157)	7.8 (5.9 to 9.9)	4 (3 to 5)	0.3 (0.2 to 0.3)	166 (137 to 198)	5.3 (4.4 to 6.2)	6 (5 to 7)	0.2 (0.2 to 0.2)	32.9 (-2.7 to 93.6)	-32.3 (-49.7 to -5)	53.6 (12.3 to 114.5)	-33.3 (-50.3 to -10.2)
		Female	45 (30 to 61)	5.7 (4 to 7.8)	1 (1 to 2)	0.2 (0.1 to 0.3)	53 (42 to 67)	3.4 (2.7 to 4.3)	2 (1 to 2)	0.1 (0.1 to 0.2)	19.1 (-19.2 to 96.3)	-39.7 (-58.5 to -3)	40.2 (-5.4 to 123.2)	-40.2 (-60.7 to -6.1)
		Male	80 (57 to 108)	9.8 (7.2 to 13.2)	2 (2 to 3)	0.3 (0.3 to 0.4)	112 (86 to 140)	7.1 (5.5 to 8.8)	4 (3 to 5)	0.2 (0.2 to 0.3)	40.7 (-8 to 119.1)	-27.6 (-51.9 to 8.2)	61.3 (6.7 to 146.3)	-29.3 (-51.7 to 5)
	Other pharynx cancer	Both	84 (63 to 107)	6 (4.6 to 7.7)	3 (2 to 4)	0.3 (0.2 to 0.3)	150 (125 to 180)	4.7 (3.9 to 5.6)	6 (5 to 7)	0.2 (0.2 to 0.2)	77.7 (27.8 to 154.8)	-22.1 (-43.3 to 11.4)	108.2 (51.8 to 200.3)	-18.6 (-40 to 15.9)
		Female	35 (24 to 48)	5.1 (3.5 to 6.9)	1 (1 to 2)	0.2 (0.1 to 0.3)	57 (44 to 70)	3.6 (2.8 to 4.4)	2 (2 to 3)	0.2 (0.1 to 0.2)	61.4 (9.3 to 164.8)	-29.6 (-51.4 to 14.2)	93.2 (31.1 to 211.8)	-24.7 (-50 to 21.2)
		Male	49 (34 to 71)	7 (4.9 to 9.9)	2 (1 to 3)	0.3 (0.2 to 0.4)	93 (71 to 119)	5.9 (4.5 to 7.5)	4 (3 to 5)	0.3 (0.2 to 0.3)	89.4 (16.1 to 208.5)	-15.9 (-46.9 to 33.5)	118.7 (34.8 to 257)	-13.7 (-44.5 to 35.6)
	Esophageal cancer	Both	1330 (1015 to 1681)	99.8 (77.5 to 126.3)	49 (38 to 62)	4.5 (3.5 to 5.7)	2574 (2127 to 3155)	81.7 (67.7 to 99.5)	115 (96 to 140)	3.9 (3.3 to 4.7)	93.5 (42 to 170.4)	-18.1 (-39 to 13.2)	133 (73.7 to 225.4)	-12.9 (-35.4 to 19.5)
		Female	508 (351 to 725)	78 (55.2 to 110.5)	19 (14 to 28)	3.6 (2.5 to 5.2)	850 (657 to 1203)	53.8 (41.6 to 75.8)	39 (30 to 54)	2.6 (2.1 to 3.7)	67.1 (13.3 to 159.5)	-31.1 (-52.7 to 4.1)	100 (36 to 202.6)	-26.2 (-49.8 to 9.7)
		Male	821 (573 to 1113)	122.5 (87.2 to 162.5)	30 (21 to 40)	5.5 (3.9 to 7.2)	1724 (1326 to 2176)	110.4 (85.6 to 139.2)	76 (59 to 95)	5.2 (4.1 to 6.4)	109.9 (37.1 to 228.9)	-9.9 (-39.4 to 38.3)	154.5 (72.3 to 296.1)	-6.1 (-34.3 to 40.7)
	Stomach cancer	Both	8894 (6799 to 10998)	651.8 (511.8 to 797.5)	322 (253 to 393)	28.4 (22.8 to 34.4)	13014 (11053 to 15032)	413.2 (353.3 to 476.1)	563 (482 to 646)	19.1 (16.3 to 21.8)	46.3 (10.9 to 102.8)	-36.6 (-51.3 to -14)	75.1 (35.4 to 137.8)	-33 (-47.4 to -11)
		Female	3313 (2326 to 4446)	488.2 (349.7 to 645.7)	119 (87 to 159)	21.3 (15.7 to 28.6)	4790 (3899 to 5799)	302.2 (247.5 to 365.4)	209 (171 to 251)	14.2 (11.6 to 16.9)	44.6 (0.8 to 120.7)	-38.1 (-56.3 to -9.1)	75.7 (25.8 to 156.7)	-33.5 (-53.3 to -5.2)

Province	Cause	Sex	1990				2019				1990-2019 percent change (%)			
			DALYs		Death		DALYs		Death		DALYs		Death	
			Number	Rate	Number	Rate	Number	Rate	Number	Rate	Number	Rate	Number	Rate
		Male	5582 (3893 to 7403)	823.3 (585.7 to 1068.1)	202 (141 to 264)	36.6 (26.1 to 46)	8224 (6542 to 10090)	528 (423.5 to 641.3)	354 (285 to 427)	24.1 (19.5 to 28.9)	47.3 (1.6 to 125.6)	-35.9 (-55.1 to -3)	74.8 (22.8 to 166.3)	-34.1 (-52.2 to -1.3)
	Colon and rectum cancer	Both	2444 (1834 to 3167)	177.1 (133.6 to 227.5)	84 (64 to 109)	7.5 (5.8 to 9.5)	7869 (6741 to 9095)	247.1 (212.6 to 284.7)	320 (274 to 370)	10.7 (9.2 to 12.4)	221.9 (129.8 to 352.2)	39.5 (0.6 to 91.4)	281.6 (170.9 to 430.3)	43.1 (4.2 to 95.3)
		Female	1063 (740 to 1494)	155 (109.7 to 214.9)	37 (26 to 52)	6.6 (4.7 to 9)	3346 (2709 to 4105)	207.9 (169.4 to 252.9)	139 (114 to 169)	9.3 (7.6 to 11.3)	214.7 (106 to 397.8)	34.2 (-11 to 107.4)	274.7 (150.4 to 476.2)	40.6 (-3.8 to 110.8)
		Male	1381 (939 to 1965)	200.2 (138.9 to 284.1)	47 (32 to 67)	8.6 (6.1 to 11.9)	4524 (3653 to 5475)	287.5 (233.4 to 346.5)	182 (147 to 219)	12.3 (9.9 to 14.8)	227.5 (109.2 to 420.1)	43.6 (-6.7 to 123.6)	287 (148.2 to 510.9)	43.5 (-4.3 to 119.8)
	Liver cancer	Both	1232 (928 to 1593)	89.9 (67 to 115)	45 (33 to 58)	4 (3 to 5.1)	2461 (2040 to 2928)	78.3 (65.3 to 92.2)	104 (86 to 123)	3.5 (2.9 to 4.1)	99.7 (41.3 to 190)	-12.9 (-37.7 to 23.5)	129.6 (62.3 to 233.7)	-13.1 (-38.2 to 25.4)
		Female	434 (288 to 638)	63.9 (41.7 to 97.9)	16 (10 to 25)	2.9 (1.9 to 4.5)	809 (629 to 1014)	51.3 (40.2 to 64)	34 (27 to 43)	2.3 (1.8 to 2.9)	86.4 (12.4 to 205.9)	-19.9 (-51.8 to 31.6)	114.9 (27.1 to 258.5)	-20.2 (-53.6 to 32.1)
		Male	798 (567 to 1092)	117.2 (84.7 to 158.2)	29 (21 to 39)	5.3 (3.9 to 7.1)	1653 (1280 to 2106)	106.1 (83.1 to 134.3)	69 (54 to 88)	4.7 (3.7 to 5.9)	107 (36.4 to 211.9)	-9.5 (-40 to 32.9)	137.6 (56.3 to 254.9)	-11.9 (-40.8 to 28.5)
	Gallbladder and biliary tract cancer	Both	323 (243 to 460)	24.8 (19.1 to 36.2)	12 (9 to 18)	1.2 (0.9 to 1.7)	647 (541 to 843)	20.6 (17.2 to 27.2)	29 (24 to 40)	1 (0.8 to 1.4)	100.2 (50.7 to 183.8)	-16.9 (-36.5 to 14.7)	135.4 (80.5 to 224)	-14 (-32.6 to 15.3)
		Female	163 (114 to 261)	25.3 (18 to 41.5)	6 (5 to 11)	1.2 (0.8 to 2)	330 (256 to 515)	20.9 (16.3 to 32.9)	15 (12 to 25)	1 (0.8 to 1.7)	101.9 (41.2 to 211.6)	-17.3 (-40.9 to 23.3)	136.9 (68.6 to 257.2)	-12.1 (-36.2 to 27.6)
		Male	160 (111 to 219)	24.2 (17.3 to 33.7)	6 (4 to 8)	1.1 (0.8 to 1.5)	317 (240 to 418)	20.3 (15.5 to 27)	14 (11 to 19)	0.9 (0.7 to 1.3)	98.5 (36.3 to 211)	-16.1 (-42.6 to 30)	133.8 (59 to 258.9)	-15.2 (-41 to 29.4)
	Pancreatic cancer	Both	606 (432 to 798)	44.5 (32 to 58.4)	22 (16 to 30)	1.9 (1.4 to 2.5)	3010 (2598 to 3487)	94.7 (81.8 to 109.6)	130 (112 to 150)	4.3 (3.8 to 5)	396.3 (250.4 to 634.4)	112.9 (51.2 to 211.2)	478 (308.8 to 752.4)	124.5 (60.2 to 229.4)
		Female	223 (154 to 327)	33.2 (23.1 to 48.6)	8 (6 to 12)	1.4 (1 to 2.1)	1254 (1006 to 1534)	78 (62.8 to 95.3)	55 (44 to 67)	3.6 (2.9 to 4.4)	461.2 (246.5 to 788.7)	135.2 (47.7 to 271.6)	552.3 (301.3 to 932.8)	151.9 (55.3 to 298.1)
		Male	383 (239 to 556)	56.6 (35.9 to 80.9)	14 (9 to 20)	2.5 (1.6 to 3.6)	1756 (1409 to 2172)	111.7 (90 to 137.8)	75 (60 to 92)	5.1 (4.1 to 6.2)	358.5 (194.4 to 688.7)	97.4 (28.8 to 235.7)	433.7 (245.9 to 806.3)	100.4 (33.1 to 236.3)
	Larynx cancer	Both	1656 (1145 to 2118)	117.5 (83.5 to 147.7)	58 (41 to 73)	4.8 (3.5 to 5.8)	2298 (1750 to 2765)	72.2 (54.9 to 86.1)	94 (72 to 113)	3.2 (2.4 to 3.8)	38.7 (2.2 to 99.9)	-38.6 (-54 to -13.1)	62.2 (20.7 to 133.2)	-33.6 (-49.9 to -7.2)
		Female	385 (268 to 517)	54.9 (39.2 to 72.8)	13 (9 to 18)	2.2 (1.6 to 2.9)	535 (425 to 663)	33.6 (26.8 to 41.6)	22 (18 to 27)	1.5 (1.2 to 1.8)	38.9 (-5.2 to 113.2)	-38.7 (-57.7 to -7.6)	68.4 (16.2 to 151.6)	-32 (-52.1 to -0.3)
		Male	1271 (779 to 1703)	183.2 (115.9 to 238.7)	45 (28 to 59)	7.7 (5 to 9.9)	1763 (1235 to 2188)	112 (78.8 to 138.4)	72 (50 to 89)	4.9 (3.4 to 6)	38.7 (-3.3 to 113.2)	-38.8 (-57.1 to -7)	60.3 (12.6 to 146.1)	-37 (-55.2 to -6)
	Tracheal, bronchus, and lung cancer	Both	3915 (2912 to 5046)	276.8 (209.6 to 353.8)	139 (105 to 179)	11.5 (8.7 to 14.7)	9778 (8154 to 11710)	307.1 (257.3 to 364.7)	402 (336 to 475)	13.4 (11.2 to 15.8)	149.7 (77.2 to 263.5)	10.9 (-20.7 to 59.1)	188.6 (104.7 to 320.7)	16.2 (-15.4 to 66.4)
		Female	695 (484 to 1025)	99.1 (70.5 to 143.8)	24 (17 to 35)	4.1 (3 to 5.9)	2596 (2116 to 3175)	161.6 (132.2 to 197.7)	106 (87 to 129)	7 (5.8 to 8.6)	273.7 (131.4 to 488.6)	63 (1 to 150.2)	345.2 (174.8 to 584.2)	71.3 (5.2 to 157.9)
		Male	3221 (2257 to 4279)	463.8 (327.1 to 608)	116 (81 to 153)	20.1 (14.5 to 26.2)	7182 (5704 to 9041)	457.5 (364.3 to 570.4)	296 (237 to 367)	19.9 (16 to 24.4)	123 (48.6 to 255.6)	-1.3 (-33.3 to 53.3)	156.3 (72.9 to 301)	-1.1 (-31.2 to 53.3)
	Malignant skin melanoma	Both	258 (140 to 367)	18.4 (9.7 to 25.9)	8 (5 to 12)	0.8 (0.4 to 1.2)	347 (253 to 468)	11.2 (8.1 to 15)	14 (9 to 17)	0.5 (0.3 to 0.6)	34.7 (-15.6 to 147.5)	-39 (-60.8 to 13.5)	63.2 (9.4 to 181)	-42.3 (-60.5 to 0.8)

Province	Cause	Sex	1990				2019				1990-2019 percent change (%)			
			DALYs		Death		DALYs		Death		DALYs		Death	
			Number	Rate	Number	Rate	Number	Rate	Number	Rate	Number	Rate	Number	Rate
		Female	123 (52 to 196)	17.4 (7.5 to 26.7)	4 (2 to 6)	0.8 (0.3 to 1.2)	162 (98 to 221)	10.5 (6.4 to 14.2)	6 (4 to 8)	0.4 (0.3 to 0.6)	31.2 (-22.2 to 155.4)	-39.9 (-62.4 to 10.3)	56.8 (2.6 to 168)	-42.1 (-61.8 to -3.8)
		Male	135 (72 to 198)	19.5 (9.4 to 28.2)	4 (2 to 6)	0.9 (0.4 to 1.3)	186 (128 to 304)	12 (8.3 to 19.3)	7 (5 to 11)	0.5 (0.3 to 0.7)	37.9 (-17.2 to 169.5)	-38.3 (-63.2 to 25)	69.2 (5.5 to 215.8)	-43 (-65 to 16.6)
	Non-melanoma skin cancer	Both	76 (55 to 99)	6.1 (4.4 to 7.8)	3 (2 to 4)	0.3 (0.2 to 0.4)	237 (200 to 277)	7.8 (6.6 to 9.1)	12 (10 to 14)	0.4 (0.4 to 0.5)	211 (124.3 to 350.2)	28.5 (-5.4 to 85)	298.5 (187.1 to 485.7)	39.9 (1.9 to 104.3)
		Female	11 (7 to 16)	1.7 (1.1 to 2.4)	0 (0 to 1)	0.1 (0.1 to 0.1)	54 (43 to 67)	3.6 (2.8 to 4.4)	3 (2 to 3)	0.2 (0.2 to 0.2)	389.3 (193.8 to 710.2)	105.7 (24.2 to 242.3)	519.2 (278.8 to 949.2)	119 (33.4 to 280)
		Male	65 (46 to 87)	11.2 (8 to 14.7)	3 (2 to 3)	0.6 (0.4 to 0.8)	183 (149 to 221)	12.1 (9.9 to 14.6)	9 (8 to 11)	0.7 (0.6 to 0.8)	180.8 (92.3 to 311)	8.3 (-24.3 to 59.2)	262 (152 to 447.3)	8.3 (-22.7 to 62.2)
	Breast cancer	Both	2353 (1652 to 3258)	157 (109.9 to 217.1)	66 (46 to 95)	5 (3.4 to 7.2)	6877 (5605 to 8304)	202.3 (165.1 to 244.1)	205 (166 to 247)	6.3 (5.1 to 7.6)	192.2 (95.3 to 354.4)	28.9 (-13.6 to 98.5)	208.9 (105.8 to 380.5)	26.2 (-17.6 to 98.1)
		Female	2314 (1620 to 3225)	307.6 (214.4 to 429.8)	65 (44 to 94)	9.5 (6.4 to 13.6)	6799 (5527 to 8219)	396.9 (322.7 to 480.1)	202 (163 to 244)	12.3 (10 to 14.9)	193.8 (95.9 to 363.1)	29 (-14.3 to 100.5)	210.4 (105.2 to 391.9)	29.8 (-15.6 to 104.9)
		Male	40 (26 to 59)	5.4 (3.4 to 8)	1 (1 to 2)	0.2 (0.1 to 0.3)	78 (56 to 109)	4.9 (3.5 to 6.8)	3 (2 to 4)	0.2 (0.1 to 0.3)	97.3 (15.1 to 244.1)	-9.7 (-46 to 54.4)	125.7 (31.5 to 299.5)	-11 (-47.2 to 56)
	Cervical cancer	Female	719 (434 to 964)	96.8 (60.6 to 130.3)	22 (14 to 29)	3.4 (2.2 to 4.6)	1022 (662 to 1295)	61.6 (40.3 to 77.6)	36 (24 to 45)	2.3 (1.6 to 2.9)	42 (-2.1 to 121.4)	-36.3 (-55.4 to -2.4)	66.1 (15.6 to 153.8)	-31.9 (-52.3 to 1.5)
	Uterine cancer	Female	104 (71 to 158)	14.8 (10.1 to 23.4)	3 (2 to 6)	0.6 (0.4 to 1)	335 (225 to 430)	20.4 (13.9 to 25.9)	12 (9 to 15)	0.8 (0.6 to 1)	223.1 (79.3 to 438.5)	37.3 (-24.6 to 126.7)	248.8 (97.8 to 481)	36.7 (-23.6 to 124.1)
	Ovarian cancer	Female	322 (195 to 552)	42 (25.3 to 71.6)	10 (6 to 16)	1.4 (0.8 to 2.4)	1391 (1019 to 1781)	83.5 (61.6 to 106.2)	47 (36 to 60)	2.9 (2.2 to 3.7)	331.6 (108.1 to 650.7)	98.8 (-3.9 to 241.6)	392.9 (141.4 to 751.6)	110.6 (2.7 to 261.7)
	Prostate cancer	Male	969 (666 to 1316)	201.2 (144.1 to 265.1)	43 (30 to 57)	12.3 (8.8 to 16.1)	3331 (2175 to 4256)	232.9 (153.8 to 296)	188 (126 to 239)	13.9 (9.4 to 17.6)	243.7 (139 to 417.8)	15.8 (-18.3 to 71.7)	339.9 (204.6 to 571)	12.6 (-20.3 to 68.3)
	Testicular cancer	Male	17 (11 to 25)	1.4 (0.9 to 2.1)	0 (0 to 0)	0 (0 to 0)	186 (129 to 251)	13.6 (9.2 to 18.2)	4 (3 to 5)	0.3 (0.2 to 0.4)	982.3 (565.1 to 1695.9)	862.1 (508.3 to 1456.8)	1351.3 (766.7 to 2424.6)	824.2 (473.2 to 1460)
	Kidney cancer	Both	357 (246 to 464)	21.6 (14.4 to 27.8)	10 (6 to 13)	0.8 (0.5 to 1)	940 (790 to 1105)	30.5 (25.9 to 35.7)	36 (30 to 43)	1.2 (1 to 1.4)	163.6 (88.3 to 307.4)	41.5 (1.8 to 126.6)	255.5 (152.3 to 499.2)	49.4 (7.4 to 162.7)
		Female	148 (104 to 200)	17.7 (11.6 to 23.5)	4 (3 to 6)	0.7 (0.4 to 0.9)	338 (272 to 417)	22.4 (18.3 to 27.2)	12 (10 to 15)	0.8 (0.7 to 1)	128.5 (57.5 to 244.9)	26.5 (-11.5 to 101.3)	204.8 (106 to 438.2)	28.1 (-14.3 to 146.4)
		Male	209 (131 to 299)	25.5 (15.6 to 36.3)	6 (4 to 9)	1 (0.6 to 1.4)	602 (471 to 755)	38.9 (30.6 to 48.1)	23 (18 to 29)	1.6 (1.2 to 2)	188.4 (75.5 to 392.6)	52.4 (-4.2 to 165.8)	290.1 (142.8 to 597.6)	61.9 (1.9 to 191.1)
	Bladder cancer	Both	488 (343 to 644)	39.5 (28.1 to 51.5)	19 (13 to 25)	2 (1.4 to 2.5)	1299 (1086 to 1532)	42.6 (35.7 to 49.8)	61 (51 to 72)	2.2 (1.8 to 2.5)	166.4 (89.1 to 296.7)	7.8 (-22.2 to 59.6)	220.9 (129.2 to 378.4)	10.7 (-19.3 to 64.7)
		Female	104 (71 to 142)	17.7 (12.1 to 24.4)	4 (3 to 6)	0.9 (0.6 to 1.3)	274 (220 to 334)	18.2 (14.7 to 22.2)	14 (11 to 17)	1 (0.8 to 1.3)	164.3 (70.7 to 312)	2.7 (-33 to 58.9)	225 (108.9 to 414.2)	8.1 (-30.4 to 69.1)
		Male	384 (253 to 527)	64.1 (43.3 to 85.1)	15 (10 to 20)	3.3 (2.2 to 4.3)	1025 (821 to 1250)	67.7 (54.3 to 82.2)	47 (38 to 58)	3.3 (2.7 to 4)	167 (77.8 to 335.8)	5.6 (-29.1 to 69.6)	219.6 (113.8 to 421.7)	1.3 (-32 to 61.4)
	Brain and central nervous system cancer	Both	2385 (1711 to 3382)	115.9 (85.2 to 160.3)	51 (37 to 70)	3.1 (2.4 to 4.3)	3593 (2366 to 4351)	121.4 (81.4 to 145.7)	111 (70 to 135)	3.7 (2.4 to 4.5)	50.7 (-18.3 to 121.4)	4.8 (-41.9 to 49.5)	117.8 (17.5 to 216.6)	16.6 (-35.6 to 69.8)
		Female	982 (563 to 1610)	95.7 (57 to 151)	21 (13 to 32)	2.6 (1.6 to 3.9)	1605 (1060 to 2096)	109.1 (73 to 141.4)	50 (32 to 65)	3.3 (2.2 to 4.3)	63.5 (-24.2 to 225.3)	14 (-46.5 to 117)	144 (17.8 to 357.3)	30.5 (-36 to 135.8)

Province	Cause	Sex	1990				2019				1990-2019 percent change (%)			
			DALYs		Death		DALYs		Death		DALYs		Death	
			Number	Rate	Number	Rate	Number	Rate	Number	Rate	Number	Rate	Number	Rate
		Male	1403 (926 to 2222)	136 (91.4 to 207.4)	30 (20 to 46)	3.8 (2.6 to 5.5)	1988 (1066 to 2630)	133.8 (73 to 177)	60 (31 to 80)	4 (2.1 to 5.3)	41.7 (-31.7 to 133.6)	-1.7 (-49.7 to 61.1)	99.8 (-3.1 to 228.3)	6.2 (-45.7 to 76.4)
	Thyroid cancer	Both	122 (92 to 173)	8.7 (6.6 to 12.7)	4 (3 to 6)	0.4 (0.3 to 0.5)	387 (306 to 459)	12.2 (9.6 to 14.4)	13 (11 to 16)	0.4 (0.4 to 0.5)	216.6 (85.5 to 343.1)	39.9 (-15.7 to 95.5)	243.1 (119.9 to 387.7)	26.8 (-20.6 to 78)
		Female	75 (53 to 118)	10.8 (7.8 to 17.1)	2 (2 to 4)	0.4 (0.3 to 0.7)	229 (158 to 293)	14.3 (10.1 to 18.3)	8 (6 to 10)	0.5 (0.4 to 0.7)	204.6 (59.5 to 378.3)	32.5 (-29.2 to 106.4)	225.7 (84.9 to 409)	22.4 (-31.5 to 88.8)
		Male	47 (33 to 68)	6.4 (4.5 to 9.5)	1 (1 to 2)	0.3 (0.2 to 0.4)	158 (119 to 198)	10 (7.7 to 12.4)	5 (4 to 7)	0.4 (0.3 to 0.5)	235.7 (99.7 to 423.3)	56.4 (-7.1 to 140.9)	272.2 (130.5 to 490.4)	45.2 (-11.7 to 124.5)
	Mesothelioma	Both	13 (8 to 21)	0.8 (0.5 to 1.4)	0 (0 to 1)	0 (0 to 0)	43 (34 to 54)	1.3 (1 to 1.6)	1 (1 to 2)	0 (0 to 0.1)	238.4 (97.9 to 510.5)	53.2 (-10.2 to 165.4)	280 (121.1 to 589.5)	58 (-6.8 to 163.8)
		Female	8 (5 to 13)	1.1 (0.7 to 1.7)	0 (0 to 0)	0 (0 to 0.1)	15 (11 to 20)	0.9 (0.7 to 1.2)	0 (0 to 1)	0 (0 to 0)	81.4 (3.3 to 235.7)	-19.3 (-52.4 to 43.4)	95.6 (19.1 to 240.5)	-18.7 (-47.7 to 34.8)
		Male	4 (2 to 13)	0.6 (0.2 to 1.7)	0 (0 to 0)	0 (0 to 0.1)	28 (21 to 37)	1.7 (1.3 to 2.3)	1 (1 to 1)	0.1 (0 to 0.1)	538.9 (113 to 1799.4)	196.7 (-2.5 to 741.5)	649 (133.7 to 2135.3)	211.1 (-3.1 to 756.9)
	Hodgkin lymphoma	Both	257 (155 to 463)	13.6 (8.6 to 23.8)	6 (4 to 10)	0.4 (0.3 to 0.7)	476 (283 to 622)	16.5 (9.7 to 21.7)	13 (7 to 16)	0.4 (0.2 to 0.6)	85.4 (-17.5 to 256.7)	21.6 (-44.4 to 130.3)	109.7 (-3.9 to 293.1)	7.6 (-49.7 to 95.7)
		Female	89 (46 to 171)	9.7 (5.4 to 18.6)	2 (1 to 4)	0.3 (0.2 to 0.6)	168 (96 to 229)	11.9 (6.7 to 16.1)	5 (3 to 6)	0.3 (0.2 to 0.4)	87.6 (-14.3 to 322.6)	21.8 (-44.1 to 162.6)	109.6 (-4.5 to 356.4)	3 (-52.4 to 123.9)
		Male	167 (102 to 319)	17.3 (10.8 to 32)	4 (2 to 7)	0.5 (0.3 to 0.9)	309 (163 to 424)	21.1 (11.1 to 29)	8 (4 to 11)	0.5 (0.3 to 0.7)	84.3 (-24.9 to 276.4)	22.1 (-48.8 to 143.5)	109.7 (-11.2 to 312.6)	10.8 (-51.9 to 115.6)
	Non-Hodgkin lymphoma	Both	789 (587 to 995)	45.5 (34.4 to 56.2)	21 (16 to 26)	1.5 (1.2 to 1.9)	1631 (1391 to 1885)	53.8 (46 to 62.1)	53 (46 to 61)	1.8 (1.5 to 2)	106.7 (51.4 to 197.7)	18.2 (-11.2 to 65.5)	151 (88.8 to 250.8)	16.7 (-9.4 to 60.3)
		Female	270 (169 to 383)	31.7 (19.7 to 43.7)	7 (5 to 10)	1.1 (0.7 to 1.4)	589 (479 to 732)	38.7 (31.5 to 47.7)	20 (16 to 24)	1.3 (1.1 to 1.6)	117.9 (37.9 to 280.2)	22.3 (-20.4 to 113.9)	168.4 (80.3 to 381.4)	22.8 (-14.3 to 127)
		Male	518 (370 to 674)	59.6 (44.1 to 76.7)	14 (10 to 18)	2 (1.5 to 2.6)	1042 (848 to 1258)	68.9 (56.4 to 82.9)	34 (27 to 40)	2.2 (1.8 to 2.7)	100.9 (41.3 to 206.2)	15.6 (-18.2 to 70.3)	141.8 (69.4 to 254.5)	10.6 (-19.8 to 58)
	Multiple myeloma	Both	267 (186 to 407)	19.3 (13.3 to 30)	10 (7 to 15)	0.8 (0.5 to 1.3)	587 (435 to 866)	18.3 (13.6 to 27.1)	23 (17 to 36)	0.8 (0.6 to 1.2)	120.1 (46.9 to 205.5)	-4.9 (-36 to 31.2)	140.8 (64.3 to 224.5)	-4.2 (-32.3 to 30.1)
		Female	119 (80 to 180)	17.6 (11.9 to 27)	4 (3 to 7)	0.7 (0.5 to 1.2)	250 (183 to 360)	15.4 (11.3 to 22.3)	10 (7 to 15)	0.6 (0.5 to 1)	109.3 (43.5 to 216.6)	-12.8 (-38.9 to 31.4)	122.1 (57.6 to 234.2)	-13.7 (-37.5 to 30.6)
		Male	148 (92 to 253)	20.9 (13.1 to 36.6)	5 (3 to 9)	0.9 (0.5 to 1.6)	338 (222 to 564)	21.4 (14.1 to 36)	13 (9 to 23)	0.9 (0.6 to 1.6)	128.8 (31.9 to 251.8)	2 (-39.9 to 58.8)	156.7 (49.9 to 294.4)	3.3 (-38.5 to 59.6)
	Leukemia	Both	6134 (3810 to 8454)	286.2 (187.3 to 374.8)	124 (82 to 160)	7.8 (5.5 to 9.8)	5851 (3909 to 7144)	209.6 (140.5 to 257)	180 (128 to 217)	6.2 (4.5 to 7.5)	-4.6 (-31.2 to 43.2)	-26.7 (-44.9 to 6)	45.4 (11.8 to 103)	-19.7 (-36.5 to 8.6)
		Female	2987 (1439 to 4249)	272.3 (147.9 to 372.9)	57 (32 to 78)	7 (4.5 to 9.2)	2479 (1485 to 3071)	177.1 (105.5 to 219.2)	76 (50 to 93)	5.3 (3.5 to 6.4)	-17 (-45.4 to 44.1)	-35 (-55.8 to 3.7)	32.6 (-6.9 to 109.1)	-24.4 (-45.2 to 8.9)
		Male	3148 (1638 to 4541)	301.8 (174.9 to 417.6)	66 (39 to 92)	8.8 (5.8 to 11.7)	3372 (2016 to 4432)	242.1 (145.1 to 315.6)	104 (65 to 134)	7.2 (4.6 to 9.3)	7.1 (-28.6 to 76.9)	-19.8 (-45.2 to 25.5)	56.6 (8.7 to 148.4)	-17.7 (-41.1 to 21.5)
	Other malignant neoplasms	Both	2709 (2034 to 3338)	146.8 (111 to 182.2)	66 (49 to 82)	4.8 (3.6 to 6.1)	4596 (3353 to 5520)	159.1 (117 to 189.5)	159 (113 to 192)	5.5 (4 to 6.6)	69.6 (19.6 to 137.4)	8.4 (-20.6 to 52.7)	140.7 (72.2 to 245.7)	13.6 (-18.1 to 60.3)
		Female	1180 (812 to 1520)	125.5 (91.9 to 160.7)	28 (21 to 36)	4 (2.9 to 5.1)	1957 (1588 to 2381)	137.4 (112.3 to 164.5)	67 (54 to 83)	4.7 (3.8 to 5.7)	65.8 (15.3 to 155.9)	9.5 (-21.8 to 63.4)	141.9 (68.6 to 264.6)	18.1 (-17.8 to 77.5)
		Male	1529 (1094 to 2010)	170 (117.9 to 227.5)	38 (26 to 51)	5.9 (4.1 to 8)	2638 (1553 to 3462)	181.3 (109.9 to 234.8)	91 (51 to 121)	6.3 (3.6 to 8.3)	72.5 (1.4 to 169.4)	6.6 (-31.6 to 68.3)	139.9 (43.9 to 285.6)	7 (-32.1 to 72)
	Other neoplasms	Both	81 (56 to 104)	5.4 (3.5 to 6.9)	2 (1 to 3)	0.2 (0.1 to 0.3)	142 (117 to 180)	4.8 (3.9 to 6)	5 (4 to 7)	0.2 (0.1 to 0.2)	74 (31.7 to 153.1)	-12.1 (-33.9 to 33.8)	122.9 (52.5 to 295.9)	-15.5 (-42.6 to 56.9)

Province	Cause	Sex	1990				2019				1990-2019 percent change (%)			
			DALYs		Death		DALYs		Death		DALYs		Death	
			Number	Rate	Number	Rate	Number	Rate	Number	Rate	Number	Rate	Number	Rate
		Female	35 (23 to 48)	4.5 (2.7 to 6.1)	1 (0 to 1)	0.2 (0.1 to 0.2)	60 (47 to 76)	4 (3.2 to 5)	2 (1 to 2)	0.1 (0.1 to 0.2)	69.5 (16.9 to 174.7)	-11.6 (-38.7 to 53.5)	120.9 (36.8 to 412.8)	-15.9 (-48 to 115.5)
		Male	46 (30 to 62)	6.5 (4 to 8.8)	1 (1 to 2)	0.3 (0.1 to 0.4)	82 (61 to 118)	5.6 (4.2 to 7.9)	3 (2 to 5)	0.2 (0.2 to 0.3)	77.3 (22.6 to 173.4)	-13.8 (-40 to 32.2)	124.2 (41.3 to 288)	-19.8 (-49.2 to 37.1)
	Cardiovascular diseases	Both	127405 (105470 to 148861)	10517.4 (8931.8 to 12111.2)	4833 (4069 to 5601)	549.3 (474.9 to 622.8)	181327 (160853 to 204630)	6127.9 (5430.4 to 6883.3)	9322 (8181 to 10476)	342.2 (299.6 to 384.9)	42.3 (18.3 to 77.9)	-41.7 (-51.1 to -28.6)	92.9 (59.8 to 139.1)	-37.7 (-47.4 to -25.7)
		Female	54861 (44048 to 67317)	9280.4 (7560.6 to 11082.7)	2251 (1829 to 2722)	509.7 (421.8 to 603.5)	80897 (69102 to 93586)	5590.2 (4764.4 to 6443.1)	4531 (3836 to 5242)	344.6 (290.3 to 396.8)	47.5 (14.1 to 93.6)	-39.8 (-52.3 to -22.5)	101.3 (58.9 to 163.2)	-32.4 (-45.7 to -14.2)
		Male	72544 (57013 to 91214)	11680.5 (9396.9 to 14376.9)	2582 (2044 to 3234)	584 (479.9 to 701.6)	100430 (84601 to 118996)	6692.8 (5657.2 to 7897.8)	4791 (4012 to 5644)	342.9 (289.4 to 402.8)	38.4 (2.9 to 89.4)	-42.7 (-56.4 to -23.4)	85.6 (37.8 to 154.3)	-41.3 (-54.6 to -23.4)
	Rheumatic heart disease	Both	1954 (1451 to 2549)	97.8 (73.2 to 130.4)	32 (23 to 46)	2.4 (1.7 to 3.7)	1521 (1188 to 1932)	51.9 (40.6 to 66)	30 (25 to 36)	1.1 (0.9 to 1.3)	-22.2 (-41.7 to 4.7)	-46.9 (-61.4 to -29.8)	-6.2 (-40.7 to 40.8)	-55.7 (-74.6 to -34.7)
		Female	1093 (731 to 1449)	109.6 (75.7 to 145.5)	18 (12 to 26)	2.7 (1.8 to 4.2)	796 (605 to 1020)	54.5 (41.3 to 69.9)	17 (13 to 21)	1.2 (0.9 to 1.5)	-27.2 (-47.4 to 10.5)	-50.3 (-64.5 to -26.5)	-10.3 (-44.8 to 59)	-56.3 (-75.3 to -24.9)
		Male	861 (587 to 1236)	85.1 (58.6 to 125.5)	14 (9 to 22)	2 (1.3 to 3.4)	725 (552 to 932)	49.4 (37.9 to 64.1)	14 (10 to 18)	1 (0.7 to 1.2)	-15.8 (-43.6 to 26.6)	-42 (-61.8 to -13)	-0.7 (-47.2 to 85.2)	-52.5 (-75.5 to -14.1)
	Ischemic heart disease	Both	79385 (64318 to 94486)	6605.6 (5535.7 to 7743.3)	3171 (2644 to 3710)	351 (302.3 to 401.2)	103640 (90529 to 118208)	3468.9 (3036.7 to 3939.1)	5527 (4840 to 6248)	202.1 (176.4 to 228.2)	30.6 (5.3 to 68.3)	-47.5 (-56.7 to -34.4)	74.3 (43.2 to 117.8)	-42.4 (-51.9 to -30.3)
		Female	30898 (24131 to 38287)	5364.3 (4259.2 to 6504.4)	1369 (1094 to 1657)	307.5 (249.4 to 363.9)	42810 (35866 to 50270)	2954.6 (2488.4 to 3455.3)	2575 (2170 to 3005)	196.3 (165 to 228.3)	38.6 (4.5 to 88.7)	-44.9 (-56.9 to -26)	88.1 (47.7 to 150)	-36.2 (-48.8 to -17.6)
		Male	48487 (37599 to 61631)	7849.8 (6268.5 to 9704.5)	1801 (1422 to 2251)	396.9 (327.5 to 475.9)	60830 (50572 to 72484)	4003.2 (3337.5 to 4754.2)	2952 (2459 to 3504)	209.9 (175.5 to 247.3)	25.5 (-9.2 to 76.3)	-49 (-61.9 to -30.6)	63.9 (20.3 to 124.1)	-47.1 (-60 to -30.6)
	Stroke	Both	32777 (26237 to 39088)	2832.2 (2294.7 to 3347.3)	1233 (959 to 1482)	150 (117.8 to 177.3)	45668 (40191 to 51571)	1554.7 (1366.9 to 1749.8)	2240 (1920 to 2557)	82.4 (70.7 to 94)	39.3 (12.5 to 74.8)	-45.1 (-55.1 to -31.8)	81.6 (45.2 to 133.7)	-45.1 (-55.7 to -30)
		Female	16551 (13222 to 20529)	2852.5 (2289 to 3494.7)	656 (504 to 826)	152.7 (118.8 to 188.4)	23094 (19401 to 26909)	1586.2 (1330.5 to 1838.6)	1175 (966 to 1380)	88.7 (72.5 to 104)	39.5 (3.6 to 84.7)	-44.4 (-58.2 to -26.9)	79.1 (31.3 to 146.2)	-41.9 (-56.8 to -21.8)
		Male	16225 (12035 to 20936)	2756.5 (2064.8 to 3509.2)	577 (417 to 754)	142.1 (101.9 to 182.3)	22574 (18779 to 26746)	1526.7 (1275.9 to 1814.2)	1065 (863 to 1276)	76.9 (62.4 to 92)	39.1 (1.9 to 91.7)	-44.6 (-58.5 to -25.3)	84.4 (32.5 to 158.1)	-45.9 (-59.7 to -25.8)
	Hypertensive heart disease	Both	5602 (4313 to 8330)	517.4 (406.9 to 748)	230 (180 to 345)	29.7 (23.1 to 42.7)	18671 (13863 to 21456)	638.8 (467.8 to 733.3)	1077 (722 to 1243)	40.1 (26.5 to 46.3)	233.3 (78 to 351.9)	23.4 (-33.9 to 64.7)	367.5 (122.3 to 541.6)	35.1 (-32 to 83.3)
		Female	2839 (2076 to 4542)	527 (389.1 to 837)	129 (94 to 213)	31.6 (23.1 to 51.7)	8884 (6662 to 10706)	619.9 (457.8 to 745.9)	554 (380 to 673)	42.5 (28.4 to 51.6)	213 (68.3 to 366.2)	17.6 (-37.6 to 72)	328.1 (109.4 to 541.3)	34.5 (-32.7 to 96.3)
		Male	2763 (1994 to 4278)	491.4 (362.6 to 742.6)	101 (72 to 163)	26 (19 to 38.5)	9787 (6884 to 12022)	660.4 (460.8 to 807.3)	523 (342 to 642)	38 (24.7 to 46.6)	254.2 (83.3 to 435.2)	34.4 (-30.9 to 96.3)	418 (138.8 to 692.4)	46.4 (-29.2 to 117.2)
	Non-rheumatic valvular heart disease	Both	355 (253 to 515)	25 (19.2 to 35.5)	11 (9 to 16)	1.1 (0.9 to 1.5)	662 (544 to 994)	21.8 (17.9 to 32.6)	28 (23 to 43)	1 (0.8 to 1.5)	86.4 (34.1 to 164.7)	-13.1 (-34.1 to 17.5)	144.5 (88.7 to 233.2)	-12.1 (-31.2 to 14.2)

Province	Cause	Sex	1990				2019				1990-2019 percent change (%)			
			DALYs		Death		DALYs		Death		DALYs		Death	
			Number	Rate	Number	Rate	Number	Rate	Number	Rate	Number	Rate	Number	Rate
		Female	181 (124 to 249)	26.3 (19.2 to 34.4)	6 (5 to 8)	1.2 (0.9 to 1.6)	324 (260 to 396)	21.3 (17.2 to 25.9)	15 (12 to 18)	1.1 (0.8 to 1.3)	78.5 (20 to 182.6)	-18.9 (-42.9 to 19.3)	137.1 (66 to 247.2)	-12.8 (-38.8 to 21.7)
		Male	174 (115 to 314)	23.1 (15.6 to 42.6)	5 (3 to 10)	0.9 (0.7 to 1.9)	338 (252 to 650)	22.2 (16.6 to 43)	13 (10 to 27)	0.9 (0.7 to 1.9)	94.8 (21.8 to 214.4)	-4 (-37.1 to 45.4)	153.4 (63.5 to 284.6)	-4.5 (-35.3 to 38.7)
	Cardiomyopathy and myocarditis	Both	938 (531 to 1978)	49.8 (31.1 to 95.8)	21 (13 to 41)	1.8 (1.1 to 3.4)	855 (535 to 1708)	30.6 (19 to 62.2)	32 (20 to 65)	1.2 (0.7 to 2.4)	-8.9 (-42.6 to 44.4)	-38.5 (-59.2 to -6.8)	51.9 (0.9 to 124.1)	-33.7 (-57.3 to -2.9)
		Female	403 (208 to 895)	42.5 (23.7 to 94)	9 (5 to 21)	1.6 (0.8 to 3.7)	290 (178 to 638)	21.4 (13.2 to 47.2)	12 (7 to 26)	0.9 (0.5 to 1.9)	-28.1 (-57.3 to 29.1)	-49.6 (-67.8 to -17)	27.5 (-18.7 to 106.6)	-43.6 (-65.9 to -9.2)
		Male	535 (304 to 1130)	56.8 (35.1 to 111.5)	12 (7 to 24)	2 (1.3 to 3.6)	565 (332 to 1177)	39.8 (23.2 to 83.3)	21 (12 to 44)	1.5 (0.8 to 3.1)	5.6 (-40.4 to 73.6)	-30 (-58.4 to 12)	70.7 (2.7 to 173.2)	-25.6 (-54.7 to 12.4)
	Atrial fibrillation and flutter	Both	662 (498 to 858)	76.8 (58.1 to 98.2)	17 (11 to 21)	3.2 (2.1 to 4)	2212 (1760 to 2803)	78.7 (63.2 to 98.8)	85 (71 to 98)	3.4 (2.8 to 3.9)	234.2 (189.7 to 298.7)	2.4 (-12.1 to 25.6)	397.5 (272.7 to 663.5)	7.4 (-19.4 to 67)
		Female	386 (278 to 508)	85.9 (60.8 to 111.6)	12 (7 to 16)	3.8 (2.3 to 5.1)	1238 (985 to 1560)	88.8 (71.1 to 111)	51 (41 to 63)	4.2 (3.3 to 5.2)	220.9 (164.5 to 322.9)	3.3 (-15.3 to 40.4)	314.1 (193.1 to 665.8)	9.9 (-21.7 to 106.1)
		Male	276 (193 to 375)	62.3 (45.9 to 81.6)	5 (3 to 7)	2.1 (1.4 to 2.8)	974 (748 to 1255)	68.8 (53.3 to 87.9)	34 (23 to 42)	2.7 (1.8 to 3.3)	252.8 (187.5 to 339.5)	10.4 (-9.2 to 32.9)	611.8 (340.5 to 983.1)	29.8 (-15.5 to 89.1)
	Aortic aneurysm	Both	251 (176 to 348)	19.6 (14.1 to 26.4)	10 (7 to 13)	1 (0.7 to 1.3)	536 (441 to 642)	17.7 (14.6 to 21.1)	27 (22 to 32)	0.9 (0.8 to 1.1)	113.7 (40.1 to 234.1)	-9.6 (-38.9 to 39.3)	171.9 (84.5 to 317.5)	-3.3 (-32.3 to 45.3)
		Female	59 (43 to 77)	9.6 (7.2 to 12.4)	2 (2 to 3)	0.5 (0.4 to 0.6)	122 (99 to 150)	8 (6.5 to 9.7)	6 (5 to 7)	0.4 (0.3 to 0.5)	108.1 (44.9 to 200.1)	-17.5 (-40.7 to 16.1)	141.3 (73.4 to 239.2)	-17.8 (-40.5 to 14)
		Male	192 (120 to 283)	30.8 (19.6 to 44.4)	7 (5 to 11)	1.6 (1 to 2.2)	414 (323 to 513)	27.7 (21.7 to 34.2)	21 (16 to 26)	1.5 (1.2 to 1.8)	115.5 (25.7 to 280.8)	-10.1 (-45.9 to 56.9)	182.2 (72.6 to 397.7)	-6.9 (-41.7 to 62.1)
	Peripheral artery disease	Both	62 (33 to 104)	6.6 (3.7 to 10.9)	1 (0 to 1)	0.1 (0.1 to 0.2)	232 (154 to 352)	8.2 (5.4 to 12.4)	6 (5 to 7)	0.2 (0.2 to 0.3)	276.3 (214.9 to 381.1)	23.5 (5.9 to 55.7)	951 (519.3 to 1546.8)	160.2 (39.2 to 323.1)
		Female	37 (19 to 64)	7.5 (3.9 to 12.7)	0 (0 to 1)	0.1 (0 to 0.2)	117 (70 to 192)	8.2 (5 to 13.4)	2 (1 to 3)	0.2 (0.1 to 0.2)	220.2 (164.6 to 306.5)	10.6 (-9.6 to 36.9)	756.2 (228.6 to 1913.9)	132.5 (-13.3 to 479.7)
		Male	25 (14 to 40)	5.5 (3.3 to 8.9)	0 (0 to 0)	0.1 (0.1 to 0.1)	115 (81 to 164)	8.1 (5.7 to 11.5)	4 (3 to 4)	0.3 (0.2 to 0.3)	358.4 (260.8 to 529.8)	46.8 (18.2 to 92.3)	1119.8 (669.1 to 1904.4)	175.1 (79.1 to 347)
	Endocarditis	Both	252 (142 to 485)	14.1 (7.8 to 28.7)	6 (3 to 13)	0.5 (0.3 to 1.2)	312 (182 to 668)	10.5 (6.3 to 22.3)	12 (7 to 26)	0.4 (0.2 to 0.9)	24 (-13.5 to 81.9)	-25.3 (-43.4 to -0.4)	93 (48.1 to 156.1)	-18.2 (-34.9 to 11)
		Female	112 (58 to 243)	12.3 (6.2 to 28.9)	3 (1 to 7)	0.5 (0.2 to 1.3)	126 (65 to 320)	8.7 (4.6 to 21.7)	5 (3 to 14)	0.4 (0.2 to 1)	13 (-37.1 to 87.5)	-29.8 (-53.4 to 0)	95.1 (32.3 to 180.4)	-15.4 (-39 to 26.1)
		Male	140 (74 to 286)	15.8 (8.3 to 31.6)	3 (2 to 7)	0.6 (0.3 to 1.1)	186 (107 to 372)	12.4 (7.3 to 24.7)	7 (4 to 13)	0.4 (0.3 to 0.9)	32.8 (-12.2 to 108.9)	-21.7 (-45.8 to 18.3)	91.4 (32.5 to 194)	-19.4 (-41.3 to 17.1)
	Other cardiovascular and circulatory diseases	Both	5169 (4141 to 6485)	272.5 (221.9 to 344.4)	99 (79 to 131)	8.6 (6.9 to 12.4)	7017 (6059 to 8126)	246.3 (214.9 to 283.8)	257 (216 to 300)	9.4 (7.9 to 10.9)	35.8 (5.1 to 73.5)	-9.6 (-31.2 to 13.8)	158.5 (77.8 to 251)	8.4 (-31.9 to 46.8)
		Female	2302 (1712 to 3019)	242.9 (188.2 to 329.6)	44 (33 to 65)	7.6 (5.7 to 12.5)	3095 (2592 to 3682)	218.7 (184 to 256.7)	118 (95 to 142)	8.9 (7.2 to 10.7)	34.5 (-1.6 to 84.9)	-10 (-37.8 to 20.3)	166.5 (56.6 to 281.2)	17.3 (-39 to 66.7)
		Male	2867 (2238 to 3676)	303.4 (238.8 to 387.4)	55 (42 to 73)	9.9 (7.6 to 13.4)	3922 (3205 to 4652)	274.2 (226.7 to 322.6)	139 (110 to 169)	9.9 (7.9 to 12)	36.8 (-0.2 to 86.9)	-9.6 (-34.1 to 21.1)	152.1 (64.9 to 269.4)	0 (-33.9 to 48)

Province	Cause	Sex	1990				2019				1990-2019 percent change (%)			
			DALYs		Death		DALYs		Death		DALYs		Death	
			Number	Rate	Number	Rate	Number	Rate	Number	Rate	Number	Rate	Number	Rate
	Chronic respiratory diseases	Both	13419 (11009 to 16262)	925.6 (776.1 to 1120.1)	303 (242 to 394)	34.1 (27.5 to 44.6)	21250 (18610 to 24064)	734.3 (640.9 to 839.6)	643 (547 to 749)	23.4 (19.9 to 27.2)	58.4 (32.9 to 88)	-20.7 (-34.8 to -6.5)	112.3 (58.4 to 173.5)	-31.3 (-48.9 to -12.3)
		Female	5773 (4510 to 7333)	780.6 (607.3 to 997.4)	123 (85 to 175)	27.2 (19.2 to 38.7)	8585 (7281 to 10105)	600.2 (508.1 to 705.6)	238 (192 to 303)	17.7 (14.3 to 22.4)	48.7 (20.9 to 90.5)	-23.1 (-38.8 to -1.2)	93.4 (34.7 to 196)	-34.8 (-55.8 to -0.9)
		Male	7646 (6080 to 9622)	1092.2 (884.6 to 1384.4)	180 (137 to 247)	43.3 (33.8 to 59.4)	12665 (10689 to 14693)	871.7 (739.8 to 1013.2)	405 (331 to 489)	29.1 (23.8 to 35.3)	65.6 (28.5 to 109.8)	-20.2 (-39.4 to 0.1)	125.3 (51 to 219.3)	-32.8 (-53.1 to -9.1)
	Chronic obstructive pulmonary disease	Both	4980 (4144 to 6256)	393.4 (327.1 to 508.8)	132 (100 to 188)	15.6 (11.8 to 23)	13346 (11741 to 14965)	446.7 (395.7 to 500.8)	444 (376 to 520)	16.3 (13.8 to 19)	168 (112.5 to 228)	13.5 (-10.7 to 39.4)	237.2 (143.7 to 362.1)	4.5 (-25 to 40.1)
		Female	2071 (1574 to 2945)	321 (241.7 to 475.9)	51 (31 to 93)	11.9 (7.4 to 21.8)	5122 (4393 to 6117)	346.7 (297.9 to 415.3)	159 (125 to 212)	12 (9.5 to 15.8)	147.4 (86.6 to 232.5)	8 (-22.3 to 47.7)	211.6 (96.1 to 447.4)	0.9 (-38 to 74.3)
		Male	2909 (2299 to 3916)	478.1 (381.8 to 634.5)	80 (58 to 116)	20.6 (15.3 to 29.3)	8224 (6917 to 9488)	549.5 (464.6 to 634.1)	284 (226 to 345)	20.5 (16.5 to 24.8)	182.7 (96.3 to 277.5)	14.9 (-18.7 to 50.9)	253.4 (122.9 to 422.2)	-0.3 (-35.2 to 42.4)
	Pneumoconiosis	Both	7 (5 to 10)	0.5 (0.4 to 0.7)	0 (0 to 0)	0 (0 to 0)	44 (33 to 57)	1.5 (1.1 to 1.9)	1 (1 to 1)	0 (0 to 0)	503.3 (309.7 to 761.8)	175.1 (89.4 to 289)	365.3 (180.3 to 729.1)	87.5 (17.9 to 225.8)
		Female	2 (1 to 3)	0.3 (0.2 to 0.4)	0 (0 to 0)	0 (0 to 0)	24 (15 to 35)	1.5 (1 to 2.3)	0 (0 to 0)	0 (0 to 0)	1089.6 (858.5 to 1388.4)	492 (378.1 to 607.3)	606.3 (320.9 to 1132.9)	153.6 (51.1 to 344.2)
		Male	5 (3 to 8)	0.8 (0.5 to 1.2)	0 (0 to 0)	0 (0 to 0.1)	20 (15 to 25)	1.4 (1.1 to 1.7)	1 (1 to 1)	0.1 (0 to 0.1)	280.2 (136.7 to 548.8)	65.3 (6.7 to 181)	347.8 (164.2 to 740.7)	63.1 (-1.9 to 206.6)
	Asthma	Both	8058 (6242 to 10294)	511.7 (396.9 to 644.4)	166 (122 to 218)	18.1 (12.8 to 25.2)	6788 (5352 to 8477)	248.6 (194.9 to 320)	177 (143 to 209)	6.4 (5.1 to 7.5)	-15.8 (-29.6 to 1.2)	-51.4 (-61.7 to -40.2)	6.2 (-24.6 to 47.2)	-64.8 (-76 to -51.6)
		Female	3511 (2540 to 4681)	439.6 (306.5 to 615)	70 (41 to 109)	14.9 (8 to 24.5)	2957 (2211 to 3768)	217.4 (161.1 to 283)	70 (45 to 90)	5.1 (3.3 to 6.6)	-15.8 (-34.5 to 6.4)	-50.6 (-65.5 to -35.2)	0.7 (-41.7 to 57.9)	-65.6 (-80.9 to -45.4)
		Male	4547 (3486 to 5862)	592.7 (462.3 to 757.8)	97 (71 to 132)	22.2 (16.4 to 30.3)	3831 (3028 to 4785)	280.2 (219.3 to 352.7)	106 (83 to 135)	7.6 (6 to 9.6)	-15.7 (-33.5 to 6.4)	-52.7 (-64.4 to -39.1)	10.2 (-26.1 to 65.5)	-65.7 (-76.9 to -49.4)
	Interstitial lung disease and pulmonary sarcoidosis	Both	142 (106 to 196)	10 (7.1 to 14.4)	3 (2 to 6)	0.4 (0.2 to 0.6)	423 (300 to 526)	13.7 (9.8 to 17)	14 (10 to 18)	0.5 (0.3 to 0.6)	197.1 (98 to 329.6)	38 (-10.3 to 107.9)	318.3 (141.6 to 610.4)	44 (-20 to 164.4)
		Female	67 (46 to 101)	9.2 (6 to 15.4)	2 (1 to 3)	0.3 (0.1 to 0.7)	178 (126 to 231)	11.4 (8.1 to 14.8)	5 (3 to 8)	0.4 (0.2 to 0.6)	167.7 (89.3 to 307.1)	23.7 (-17.1 to 101.8)	242.2 (98 to 637.2)	21.1 (-34.3 to 184.7)
		Male	76 (54 to 108)	10.9 (7.6 to 16.3)	2 (1 to 3)	0.4 (0.2 to 0.7)	244 (146 to 322)	16.1 (9.8 to 21)	9 (5 to 12)	0.6 (0.4 to 0.9)	223 (75.6 to 413.9)	48.4 (-19.5 to 146.2)	383.2 (143.2 to 794.7)	55.9 (-25.7 to 209.6)
	Other chronic respiratory diseases	Both	232 (170 to 330)	10.1 (7.6 to 14.3)	1 (1 to 3)	0.1 (0 to 0.2)	650 (488 to 794)	23.8 (18.1 to 29.2)	7 (2 to 11)	0.3 (0.1 to 0.4)	180.4 (65.5 to 287.2)	136.3 (46.9 to 218.6)	485.5 (7.5 to 1445.2)	192.7 (-33.9 to 633.4)
		Female	123 (88 to 176)	10.5 (7.7 to 14.6)	1 (0 to 1)	0.1 (0 to 0.2)	305 (228 to 379)	23.2 (17.5 to 29)	3 (1 to 5)	0.2 (0.1 to 0.3)	148.5 (50.8 to 245.4)	121.6 (41.2 to 199.2)	433.5 (-12.1 to 1461.7)	172.6 (-42.4 to 647.3)
		Male	109 (76 to 163)	9.7 (7 to 14)	1 (0 to 2)	0.1 (0 to 0.2)	345 (246 to 434)	24.5 (17.9 to 30.9)	4 (1 to 7)	0.3 (0.1 to 0.5)	216.1 (73.6 to 376.3)	152.4 (49.7 to 267.8)	527.2 (14.4 to 1719.3)	204.8 (-32.6 to 752.2)
	Digestive diseases	Both	10459 (8500 to 12473)	654 (533.1 to 792.3)	223 (173 to 282)	20.9 (16.3 to 27.2)	13905 (11386 to 16846)	457.8 (376.4 to 551.5)	382 (321 to 451)	13.5 (11.4 to 15.9)	33 (7.3 to 64)	-30 (-42.7 to -15)	71.5 (24.9 to 130.5)	-35.5 (-52.5 to -14.6)
		Female	4696 (3727 to 5800)	579.3 (452.8 to 727.1)	92 (67 to 125)	17.5 (12.5 to 25)	6340 (5023 to 7873)	421.5 (338.1 to 518.7)	162 (132 to 199)	11.8 (9.7 to 14.4)	35 (10.3 to 67)	-27.2 (-41.5 to -9.4)	77.1 (23.2 to 153.2)	-32.4 (-55.4 to -1.3)

Province	Cause	Sex	1990				2019				1990-2019 percent change (%)			
			DALYs		Death		DALYs		Death		DALYs		Death	
			Number	Rate	Number	Rate	Number	Rate	Number	Rate	Number	Rate	Number	Rate
		Male	5763 (4457 to 7200)	730.2 (578.1 to 917.9)	131 (97 to 173)	24.8 (18.8 to 32.1)	7565 (6096 to 9214)	495 (399.4 to 600.1)	219 (174 to 273)	15.2 (12.1 to 18.9)	31.3 (-1.8 to 77.8)	-32.2 (-48.6 to -10.7)	67.6 (14 to 144.9)	-38.6 (-56.2 to -13.4)
	Cirrhosis and other chronic liver diseases	Both	4249 (3191 to 5308)	271.3 (204.8 to 346.7)	127 (95 to 163)	10.9 (8 to 14.3)	5429 (4381 to 6535)	176.3 (143 to 211.9)	219 (177 to 267)	7.5 (6.1 to 9.2)	27.8 (-7.6 to 77.7)	-35 (-52.5 to -11.6)	72.5 (23.8 to 139.4)	-31.2 (-50.5 to -5.7)
		Female	1660 (1198 to 2160)	208.4 (149.4 to 282.9)	49 (35 to 69)	8.7 (5.8 to 12.6)	1959 (1526 to 2479)	131 (103.2 to 164.1)	86 (67 to 110)	6 (4.7 to 7.8)	18 (-17.3 to 79.2)	-37.1 (-55.5 to -7.4)	73 (15.4 to 162.9)	-30.3 (-56.3 to 9.2)
		Male	2589 (1859 to 3489)	334.6 (243 to 446.5)	77 (55 to 104)	13.3 (9.8 to 17.5)	3471 (2624 to 4422)	222.4 (169.2 to 283.2)	133 (101 to 169)	9 (6.8 to 11.3)	34 (-12.8 to 103.7)	-33.5 (-56.3 to -0.5)	72.2 (13.7 to 156.8)	-32.6 (-53.1 to -2.4)
	Upper digestive system diseases	Both	3430 (2453 to 4885)	217.3 (156 to 297.9)	50 (33 to 68)	5.3 (3.4 to 7.3)	4469 (2971 to 6906)	143.9 (96.3 to 221.6)	53 (34 to 66)	1.9 (1.3 to 2.4)	30.3 (7.4 to 49.4)	-33.8 (-47.1 to -21.3)	6.3 (-23.1 to 49.7)	-63.5 (-73.5 to -48.6)
		Female	1635 (1122 to 2357)	204.6 (139.1 to 294.8)	20 (11 to 31)	4.5 (2.3 to 7.1)	2303 (1449 to 3655)	148.5 (95.2 to 230.4)	24 (14 to 31)	1.8 (1.1 to 2.4)	40.8 (17.2 to 61.7)	-27.4 (-42.3 to -14.3)	16.2 (-23.9 to 84.4)	-59.8 (-75 to -34.6)
		Male	1794 (1280 to 2512)	230.6 (164.5 to 315.6)	29 (19 to 42)	6.3 (4 to 8.7)	2167 (1478 to 3268)	139.4 (95.9 to 210.3)	29 (18 to 38)	2.1 (1.3 to 2.8)	20.8 (-6.4 to 45.2)	-39.6 (-53.6 to -24.3)	-0.5 (-35.9 to 50.2)	-66.6 (-76.9 to -52.1)
	Appendicitis	Both	338 (214 to 457)	16.9 (11.5 to 22.8)	7 (4 to 9)	0.5 (0.4 to 0.8)	216 (159 to 277)	8.1 (6 to 10.5)	5 (4 to 7)	0.2 (0.1 to 0.3)	-36.2 (-59.1 to 15.8)	-51.8 (-67.9 to -20.6)	-16.4 (-46.5 to 44.2)	-62.8 (-76.9 to -42.2)
		Female	159 (90 to 245)	15.8 (9.3 to 25.3)	3 (2 to 5)	0.5 (0.3 to 0.9)	103 (71 to 137)	8 (5.7 to 10.7)	3 (2 to 4)	0.2 (0.1 to 0.3)	-34.9 (-63.6 to 27.1)	-49.3 (-71 to -11.2)	-11.2 (-53.4 to 68.7)	-58.8 (-79.7 to -26.8)
		Male	179 (105 to 255)	17.8 (10.8 to 25.1)	3 (2 to 5)	0.5 (0.3 to 0.8)	112 (78 to 153)	8.2 (5.7 to 11.4)	3 (2 to 4)	0.2 (0.1 to 0.3)	-37.2 (-61.3 to 29.9)	-53.7 (-70.6 to -9.6)	-21.2 (-54.9 to 76.6)	-65.1 (-78.7 to -27.6)
	Paralytic ileus and intestinal obstruction	Both	700 (452 to 1056)	36.5 (27.2 to 50.7)	14 (10 to 20)	1.3 (1 to 1.9)	583 (380 to 730)	23 (15.4 to 29.1)	27 (18 to 35)	1 (0.7 to 1.3)	-16.8 (-57.4 to 48.4)	-37 (-63.8 to -2.9)	92.6 (12.3 to 186.9)	-23.9 (-51.9 to 8.5)
		Female	333 (203 to 549)	34.5 (23.5 to 51.8)	7 (5 to 10)	1.2 (0.8 to 2)	263 (168 to 354)	21 (13.8 to 28)	12 (8 to 17)	0.9 (0.6 to 1.3)	-21 (-60.9 to 50.1)	-39 (-65.3 to 4.5)	75.9 (-1.5 to 203.2)	-26.3 (-55.9 to 21)
		Male	368 (215 to 606)	38.9 (26.5 to 58.4)	7 (5 to 11)	1.5 (1 to 2.1)	320 (190 to 429)	24.9 (15.3 to 33.4)	15 (9 to 21)	1.1 (0.6 to 1.5)	-13 (-61.1 to 71.1)	-35.8 (-66.1 to 9.3)	108.5 (4.9 to 260)	-24.8 (-55.3 to 17.2)
	Inguinal, femoral, and abdominal hernia	Both	364 (241 to 523)	17.2 (11.9 to 23.7)	2 (1 to 2)	0.2 (0.1 to 0.3)	439 (292 to 629)	15.7 (10.5 to 22.3)	3 (2 to 4)	0.1 (0.1 to 0.2)	20.8 (-5 to 52.3)	-8.8 (-23.7 to 7.6)	103 (31.1 to 232.4)	-32.5 (-58.2 to 0.7)
		Female	133 (84 to 203)	11.9 (8 to 17.1)	1 (0 to 1)	0.1 (0.1 to 0.2)	150 (95 to 219)	11.1 (7.1 to 16.3)	1 (1 to 2)	0.1 (0 to 0.2)	13.3 (-20.4 to 62)	-6.7 (-29.8 to 23)	134 (28.7 to 308.3)	-17.9 (-52.1 to 37.6)
		Male	231 (153 to 331)	23 (16 to 31.6)	1 (1 to 2)	0.3 (0.2 to 0.5)	289 (190 to 413)	20.4 (13.7 to 28.8)	2 (1 to 3)	0.2 (0.1 to 0.2)	25.1 (-5.2 to 61.7)	-11.4 (-28.5 to 7.6)	86.5 (5.9 to 226.5)	-48.6 (-72.2 to -13.1)
	Inflammatory bowel disease	Both	150 (109 to 204)	9.3 (6.7 to 12.7)	1 (1 to 2)	0.1 (0.1 to 0.2)	332 (238 to 443)	10.3 (7.4 to 13.6)	4 (2 to 6)	0.2 (0.1 to 0.2)	121.8 (75.1 to 172.2)	10.1 (-10.7 to 32.5)	197.4 (61 to 369)	19.9 (-31.9 to 85.1)
		Female	73 (51 to 103)	9.1 (6.3 to 12.7)	1 (0 to 1)	0.1 (0.1 to 0.2)	164 (114 to 221)	10.2 (7.1 to 13.6)	2 (1 to 3)	0.2 (0.1 to 0.2)	124.1 (68.1 to 189.1)	12.8 (-13.5 to 45.4)	282.1 (73.5 to 585.9)	59.4 (-26.4 to 199.9)
		Male	76 (55 to 106)	9.7 (7 to 13.4)	1 (1 to 1)	0.2 (0.1 to 0.3)	168 (118 to 229)	10.4 (7.4 to 14)	2 (1 to 3)	0.1 (0.1 to 0.2)	119.6 (66 to 187.3)	6.8 (-18.1 to 39.1)	142.4 (31.3 to 309)	-9.4 (-47.8 to 50.6)
	Vascular intestinal disorders	Both	113 (67 to 163)	10.1 (5.6 to 14.9)	5 (3 to 7)	0.6 (0.3 to 0.9)	286 (187 to 358)	9.7 (6.4 to 12.1)	15 (10 to 19)	0.5 (0.4 to 0.7)	153.6 (71.8 to 257.6)	-4.1 (-37 to 37.4)	215.8 (100.2 to 368.7)	-2.7 (-38.2 to 48.6)
		Female	52 (24 to 85)	9.5 (3.9 to 15.8)	2 (1 to 4)	0.5 (0.2 to 0.9)	137 (81 to 181)	9.4 (5.6 to 12.2)	7 (5 to 10)	0.5 (0.3 to 0.7)	163.2 (59.5 to 336.8)	-1.7 (-43.1 to 75.5)	206.2 (72.6 to 480.6)	-1.3 (-46 to 98.3)
		Male	60 (38 to 87)	10.7 (6.8 to 14.9)	2 (1 to 3)	0.6 (0.4 to 0.8)	148 (92 to 197)	10 (6.3 to 13.2)	8 (5 to 10)	0.6 (0.4 to 0.7)	145.4 (54.1 to 277.1)	-6.3 (-39.3 to 40.7)	225.5 (106.6 to 400.9)	-5.4 (-36.9 to 41.5)
	Gallbladder and biliary diseases	Both	727 (487 to 1044)	48.2 (32.5 to 67.6)	7 (4 to 12)	0.8 (0.6 to 1.4)	1368 (976 to 1897)	44 (31.8 to 60.4)	23 (18 to 30)	0.9 (0.7 to 1.1)	88.1 (53.8 to 124.1)	-8.7 (-24.4 to 6.9)	249.6 (119.4 to 447.5)	4.7 (-30.5 to 59.2)

Province	Cause	Sex	1990				2019				1990-2019 percent change (%)			
			DALYs		Death		DALYs		Death		DALYs		Death	
			Number	Rate	Number	Rate	Number	Rate	Number	Rate	Number	Rate	Number	Rate
		Female	489 (326 to 707)	62.5 (41.7 to 88.6)	4 (2 to 6)	0.8 (0.5 to 1.4)	931 (647 to 1351)	59.2 (42.1 to 84.4)	13 (9 to 18)	1 (0.7 to 1.4)	90.3 (55.9 to 126.6)	-5.3 (-21 to 11.2)	278.3 (117.7 to 524.4)	24.2 (-23.6 to 109.2)
		Male	238 (157 to 346)	33.8 (23.3 to 48.6)	3 (2 to 6)	0.9 (0.6 to 1.6)	437 (323 to 593)	28.7 (21.5 to 38.6)	10 (7 to 15)	0.7 (0.5 to 1.1)	83.6 (42.5 to 136.8)	-15 (-35.1 to 9.3)	217.8 (93.1 to 457.2)	-13.2 (-44.2 to 46.6)
	Pancreatitis	Both	209 (141 to 335)	15.6 (10.3 to 24.8)	7 (4 to 11)	0.7 (0.4 to 1.2)	336 (261 to 491)	11 (8.6 to 15.9)	14 (11 to 20)	0.5 (0.4 to 0.7)	60.5 (13.1 to 131.5)	-29.2 (-50.1 to 2.5)	106.5 (36.2 to 220.1)	-27.9 (-52.4 to 13.3)
		Female	80 (50 to 140)	12.5 (7.5 to 22.8)	3 (2 to 6)	0.6 (0.3 to 1.3)	143 (105 to 209)	9.6 (7.1 to 14)	7 (5 to 10)	0.5 (0.4 to 0.7)	77.8 (18.6 to 174.8)	-22.9 (-50.2 to 20.4)	138.5 (40.6 to 311.1)	-17.6 (-53.4 to 49)
		Male	129 (80 to 210)	18.4 (11.6 to 29.3)	4 (2 to 6)	0.7 (0.5 to 1.2)	193 (141 to 308)	12.4 (9.2 to 19.7)	7 (5 to 11)	0.5 (0.3 to 0.7)	49.6 (-8.3 to 131.6)	-32.5 (-56.9 to 3.1)	83.3 (9.9 to 201)	-33.9 (-58.9 to 3.7)
	Other digestive diseases	Both	179 (139 to 235)	11.6 (8.3 to 15.9)	4 (3 to 6)	0.5 (0.3 to 0.7)	447 (284 to 560)	15.8 (10.3 to 19.6)	18 (11 to 22)	0.7 (0.4 to 0.8)	149.4 (38.8 to 257.6)	36 (-18.3 to 83.7)	324.3 (140 to 517.7)	44.9 (-17.8 to 121.7)
		Female	82 (62 to 113)	10.5 (6.8 to 15.5)	2 (1 to 3)	0.4 (0.2 to 0.7)	187 (118 to 243)	13.4 (8.7 to 17.2)	7 (4 to 10)	0.5 (0.3 to 0.7)	128.2 (37.9 to 242.1)	27.4 (-18.5 to 87.6)	284.3 (103.8 to 561.1)	36 (-29.4 to 155.1)
		Male	98 (72 to 132)	12.8 (9.3 to 16.9)	2 (2 to 3)	0.5 (0.3 to 0.7)	260 (160 to 342)	18.2 (11.3 to 23.7)	11 (6 to 14)	0.8 (0.4 to 1)	167.1 (40 to 313.9)	42.4 (-18.9 to 110.9)	357 (144.8 to 636.8)	48 (-17.7 to 129.2)
	Neurological disorders	Both	23956 (11652 to 42004)	1391.8 (764.4 to 2334.3)	189 (95 to 414)	30.7 (12.2 to 74.3)	38728 (20866 to 63923)	1383.6 (750 to 2306.9)	807 (339 to 1888)	32.1 (13.1 to 75.6)	61.7 (32.4 to 120.4)	-0.6 (-12.9 to 14.5)	327.4 (223.7 to 469.1)	4.6 (-10.2 to 38.1)
		Female	13403 (5970 to 24846)	1521 (772.4 to 2686.4)	105 (47 to 249)	29.9 (11 to 75.4)	21569 (10686 to 37174)	1538.2 (773 to 2652.1)	394 (157 to 929)	32.3 (12.4 to 76.7)	60.9 (34.7 to 111.2)	1.1 (-11.4 to 16.6)	274.6 (179.7 to 429.2)	7.8 (-12.7 to 52.6)
		Male	10553 (5625 to 18083)	1266.7 (745.9 to 2073.3)	84 (45 to 167)	31.8 (13.3 to 73.6)	17160 (9917 to 27375)	1232.2 (709.8 to 1958.7)	413 (183 to 958)	32 (13.9 to 74.5)	62.6 (28.2 to 130.4)	-2.7 (-17.3 to 14.6)	393.7 (244.3 to 586.2)	0.8 (-19.2 to 33.6)
	Alzheimer's disease and other dementias	Both	2447 (1056 to 5803)	374.6 (167.2 to 864.3)	122 (29 to 345)	24.4 (5.9 to 68.3)	10110 (4444 to 22857)	382.9 (169.5 to 859.6)	613 (144 to 1693)	25 (6 to 68.5)	313.2 (254.3 to 411.4)	2.2 (-9.2 to 25.1)	403.5 (317.4 to 599.7)	2.6 (-12.1 to 41.9)
		Female	1412 (626 to 3336)	381 (172.9 to 894)	75 (18 to 218)	24.7 (6 to 70.5)	5261 (2285 to 11886)	402.3 (174.8 to 904.5)	313 (74 to 857)	26.4 (6.3 to 71.5)	272.6 (206 to 383.6)	5.6 (-9.9 to 36.5)	315.8 (230.4 to 522.2)	6.8 (-12.5 to 57.4)
		Male	1035 (443 to 2380)	366.2 (161.8 to 827)	46 (11 to 131)	24 (5.7 to 65.5)	4849 (2147 to 11144)	363.8 (161.7 to 840.7)	300 (70 to 836)	23.8 (5.7 to 66.4)	368.6 (275.9 to 510.2)	-0.7 (-15.5 to 21.4)	545.5 (398.3 to 797.4)	-0.7 (-18.7 to 32.6)
	Parkinson's disease	Both	655 (545 to 774)	73.5 (61.1 to 87.9)	29 (24 to 35)	4.3 (3.5 to 5.6)	2383 (2031 to 2736)	84.5 (72.1 to 96.7)	142 (120 to 164)	5.3 (4.5 to 6.1)	263.9 (190.5 to 362)	14.9 (-10.9 to 43.9)	386.7 (274.4 to 530.6)	22.1 (-8.7 to 57.9)
		Female	284 (224 to 359)	60.2 (48 to 80.4)	13 (10 to 18)	3.5 (2.7 to 5.3)	1015 (849 to 1198)	71.3 (59.7 to 84.1)	59 (49 to 72)	4.4 (3.6 to 5.4)	257.9 (169.7 to 386.9)	18.3 (-13.4 to 58.1)	349.6 (223.2 to 528.8)	25.8 (-12.1 to 74.4)
		Male	371 (280 to 466)	92.6 (71.6 to 112.9)	16 (12 to 20)	5.6 (4.3 to 7)	1368 (1033 to 1635)	97.8 (73.7 to 116.9)	82 (60 to 100)	6.1 (4.4 to 7.4)	268.5 (166 to 406.9)	5.6 (-24.2 to 43.6)	417.2 (260.5 to 644.8)	8.9 (-25.5 to 52.8)
	Idiopathic epilepsy	Both	4437 (2236 to 7332)	173.3 (88.4 to 285)	27 (18 to 34)	1.2 (0.9 to 1.6)	3213 (1497 to 5783)	130.3 (59.5 to 234.3)	20 (17 to 27)	0.7 (0.6 to 1)	-27.6 (-68.4 to 67.3)	-24.8 (-67.7 to 75.7)	-24.3 (-45.5 to 22.3)	-41.1 (-56 to -10.3)
		Female	2044 (1025 to 3475)	160.8 (81.1 to 266.4)	12 (7 to 17)	1.1 (0.7 to 1.5)	1426 (621 to 2649)	117.7 (51.4 to 221.3)	8 (6 to 11)	0.6 (0.5 to 0.8)	-30.2 (-71.9 to 70.7)	-26.8 (-70.4 to 78.9)	-33.4 (-56.3 to 33.2)	-47 (-63.5 to -4.4)
		Male	2393 (1199 to 3901)	185.9 (93.7 to 301.4)	15 (10 to 20)	1.4 (1 to 1.8)	1787 (856 to 3174)	142.6 (66.3 to 255.2)	12 (10 to 16)	0.9 (0.7 to 1.1)	-25.3 (-67 to 69.9)	-23.3 (-66.4 to 74.4)	-16.7 (-42.5 to 37.2)	-36.5 (-54.8 to 0.3)
	Multiple sclerosis	Both	447 (310 to 700)	27 (18.6 to 42.8)	6 (3 to 12)	0.4 (0.2 to 0.8)	912 (683 to 1314)	27.4 (20.7 to 39.2)	14 (10 to 25)	0.4 (0.3 to 0.7)	103.8 (57.8 to 159.7)	1.7 (-22.6 to 29.2)	151 (63.7 to 314.3)	11.5 (-27.4 to 88.7)

Province	Cause	Sex	1990				2019				1990-2019 percent change (%)			
			DALYs		Death		DALYs		Death		DALYs		Death	
			Number	Rate	Number	Rate	Number	Rate	Number	Rate	Number	Rate	Number	Rate
		Female	247 (168 to 379)	29.7 (20 to 46.1)	3 (1 to 6)	0.4 (0.2 to 0.8)	503 (376 to 707)	30.4 (22.8 to 42.5)	7 (5 to 12)	0.4 (0.3 to 0.7)	103.1 (40.6 to 176.7)	2.5 (-28.5 to 36.9)	151.6 (34.8 to 415.4)	12.2 (-37.3 to 136.8)
		Male	200 (127 to 357)	24.2 (15.4 to 43.5)	3 (1 to 7)	0.4 (0.2 to 0.9)	409 (282 to 692)	24.5 (17.1 to 40.7)	7 (4 to 15)	0.5 (0.3 to 0.9)	104.6 (35 to 185.5)	1.4 (-34.8 to 40)	150.4 (32.6 to 333.3)	11.9 (-40.6 to 90.3)
	Motor neuron disease	Both	39 (30 to 51)	2.1 (1.6 to 2.8)	1 (1 to 1)	0 (0 to 0.1)	137 (112 to 166)	4.3 (3.5 to 5.1)	4 (3 to 5)	0.1 (0.1 to 0.1)	248.9 (147.1 to 388.5)	104.3 (43.2 to 188.9)	428.2 (226.7 to 753)	161.1 (59.6 to 325.5)
		Female	20 (15 to 27)	2 (1.5 to 2.8)	0 (0 to 0)	0 (0 to 0.1)	51 (41 to 62)	3.3 (2.7 to 3.9)	1 (1 to 2)	0.1 (0.1 to 0.1)	158.9 (77.4 to 263.9)	65 (10.7 to 132.6)	321.9 (141.9 to 593.8)	120.6 (22.3 to 265.9)
		Male	19 (14 to 27)	2.2 (1.5 to 3.1)	0 (0 to 1)	0.1 (0 to 0.1)	85 (67 to 110)	5.3 (4.1 to 6.7)	3 (2 to 3)	0.2 (0.1 to 0.2)	340.4 (176.6 to 591.6)	142.3 (50.3 to 285)	511.8 (231.3 to 1068.7)	193.6 (60.4 to 453)
	Headache disorders	Both	15045 (3296 to 32934)	705.7 (169.4 to 1513.7)	--	--	20787 (5374 to 44075)	706.5 (171 to 1502.8)	--	--	38.2 (25.3 to 69.9)	0.1 (-5 to 6.4)	--	--
		Female	9021 (1843 to 20132)	856.3 (191.7 to 1883.6)	--	--	12794 (3006 to 27492)	871.4 (193.1 to 1892.5)	--	--	41.8 (27.9 to 67.8)	1.8 (-5.1 to 8.4)	--	--
		Male	6024 (1442 to 12965)	555.4 (150.4 to 1166)	--	--	7994 (2380 to 16604)	544.5 (150.1 to 1139)	--	--	32.7 (17.5 to 71.1)	-2 (-8.6 to 7.5)	--	--
	Other neurological disorders	Both	886 (451 to 1469)	35.6 (20.2 to 55.9)	5 (4 to 7)	0.3 (0.2 to 0.4)	1187 (780 to 1749)	47.9 (29.4 to 74.4)	14 (12 to 17)	0.5 (0.4 to 0.6)	34 (-25.4 to 168.8)	34.4 (-25.2 to 163.1)	183.6 (102.9 to 326.8)	80.8 (31.7 to 172.8)
		Female	376 (181 to 656)	31 (16.9 to 50.3)	2 (1 to 2)	0.2 (0.1 to 0.3)	519 (331 to 796)	42 (24.6 to 68.1)	6 (4 to 7)	0.4 (0.3 to 0.5)	38.2 (-26 to 204.2)	35.6 (-28.6 to 178.1)	224.6 (119.9 to 403.6)	89.1 (25.7 to 213.9)
		Male	510 (265 to 830)	40.2 (23 to 62.3)	3 (2 to 5)	0.4 (0.2 to 0.5)	668 (444 to 988)	53.7 (33.3 to 83.4)	9 (7 to 11)	0.6 (0.5 to 0.8)	31 (-25.6 to 159.4)	33.4 (-25 to 152.3)	162.8 (71.6 to 328)	74.3 (17.4 to 177)
	Mental disorders	Both	45737 (33752 to 60179)	2115.8 (1568.2 to 2768)	0 (0 to 0)	0 (0 to 0)	62280 (46145 to 81887)	2149.1 (1586.1 to 2824.5)	0 (0 to 0)	0 (0 to 0)	36.2 (28.7 to 44)	1.6 (-1.2 to 4.3)	170.5 (21.3 to 547.9)	64.8 (-26.8 to 308.9)
		Female	25676 (18896 to 34054)	2406.3 (1780.8 to 3159.2)	0 (0 to 0)	0 (0 to 0)	35257 (25995 to 46786)	2436.5 (1797.3 to 3241.7)	0 (0 to 0)	0 (0 to 0)	37.3 (28.9 to 45.9)	1.3 (-2.6 to 5.4)	138.9 (-4.5 to 567.6)	42.2 (-42.9 to 322.8)
		Male	20061 (14821 to 26491)	1822.7 (1348.7 to 2399.5)	0 (0 to 0)	0 (0 to 0)	27023 (19737 to 35892)	1866.5 (1366.3 to 2466.2)	0 (0 to 0)	0 (0 to 0)	34.7 (26 to 44.3)	2.4 (-1.4 to 6.2)	337.7 (21.3 to 1400.4)	204.1 (-14.3 to 1021.9)
	Schizophrenia	Both	2975 (2144 to 3830)	160.3 (115.5 to 204.7)	--	--	5269 (3847 to 6783)	161.6 (117.8 to 208.1)	--	--	77.1 (58.7 to 95.6)	0.8 (-8 to 10.1)	--	--
		Female	1413 (1004 to 1829)	152.8 (110.5 to 197.2)	--	--	2483 (1806 to 3199)	152.9 (112 to 197.3)	--	--	75.8 (52.3 to 101.9)	0.1 (-11.5 to 13.2)	--	--
		Male	1562 (1128 to 2011)	167.7 (120.7 to 216)	--	--	2786 (1997 to 3617)	170.1 (122.3 to 220.2)	--	--	78.3 (55.8 to 102.8)	1.5 (-10.1 to 14)	--	--
	Depressive disorders	Both	14170 (9177 to 20374)	709.4 (469.2 to 1010.9)	--	--	22964 (15448 to 32934)	750.8 (505.9 to 1075.8)	--	--	62.1 (45.8 to 80.1)	5.8 (0.2 to 11.5)	--	--
		Female	9279 (6080 to 13036)	919.1 (618.4 to 1285)	--	--	14221 (9620 to 20231)	940.7 (635.5 to 1326.6)	--	--	53.3 (37.9 to 71.1)	2.4 (-4.5 to 10)	--	--
		Male	4891 (3105 to 7282)	499.4 (324.5 to 743.6)	--	--	8743 (5712 to 13122)	563.7 (373 to 839)	--	--	78.7 (57.2 to 104.8)	12.9 (4.7 to 22)	--	--

Province	Cause	Sex	1990				2019				1990-2019 percent change (%)			
			DALYs		Death		DALYs		Death		DALYs		Death	
			Number	Rate	Number	Rate	Number	Rate	Number	Rate	Number	Rate	Number	Rate
	Bipolar disorder	Both	3591 (2167 to 5483)	173.9 (106.3 to 267.3)	--	--	5245 (3230 to 8156)	175.5 (108.6 to 273.2)	--	--	46.1 (32.3 to 60.4)	0.9 (-6.2 to 8.2)	--	--
		Female	1959 (1166 to 3000)	187.9 (113.2 to 290.8)	--	--	2796 (1709 to 4278)	189.9 (114.8 to 290.6)	--	--	42.7 (26.2 to 60.3)	1 (-8.8 to 11.9)	--	--
		Male	1632 (996 to 2470)	160 (97.7 to 242.8)	--	--	2449 (1520 to 3788)	161.6 (99.6 to 251.4)	--	--	50.1 (32.3 to 68.5)	1 (-9.2 to 11.7)	--	--
	Anxiety disorders	Both	15210 (10493 to 21031)	676 (469.9 to 931.3)	--	--	19388 (13415 to 26554)	696.4 (484.9 to 958.9)	--	--	27.5 (15.6 to 40.1)	3 (-2.2 to 9.1)	--	--
		Female	9162 (6257 to 12716)	825.8 (566.4 to 1142.1)	--	--	11899 (8210 to 16235)	855.8 (595.5 to 1176)	--	--	29.9 (16.1 to 45.3)	3.6 (-3.7 to 11.6)	--	--
		Male	6048 (4164 to 8422)	524.8 (362.5 to 727)	--	--	7489 (5187 to 10289)	540.3 (374.9 to 738.7)	--	--	23.8 (9.8 to 39.2)	3 (-5.8 to 11.8)	--	--
	Eating disorders	Both	1006 (633 to 1498)	41.5 (26 to 61.5)	0 (0 to 0)	0 (0 to 0)	1428 (877 to 2146)	53.2 (32.7 to 79.7)	0 (0 to 0)	0 (0 to 0)	41.9 (21.5 to 67.7)	28.2 (14.3 to 42)	170.5 (21.3 to 547.9)	64.8 (-26.8 to 308.9)
		Female	625 (392 to 927)	51.4 (32.2 to 76)	0 (0 to 0)	0 (0 to 0)	873 (537 to 1303)	66.8 (40.8 to 99.1)	0 (0 to 0)	0 (0 to 0)	39.7 (16.7 to 64.1)	29.9 (13.8 to 47.6)	138.9 (-4.5 to 567.6)	42.2 (-42.9 to 322.8)
		Male	382 (230 to 585)	31.5 (19 to 48.3)	0 (0 to 0)	0 (0 to 0)	555 (330 to 866)	40.1 (24.2 to 61.9)	0 (0 to 0)	0 (0 to 0)	45.4 (16.2 to 81.4)	27.1 (6.9 to 51.9)	337.7 (21.3 to 1400.4)	204.1 (-14.3 to 1021.9)
	Autism spectrum disorders	Both	1111 (721 to 1634)	44.3 (28.7 to 64.7)	--	--	1131 (733 to 1675)	45 (29.1 to 66.6)	--	--	1.8 (-8.9 to 12)	1.5 (-8.2 to 12.2)	--	--
		Female	277 (175 to 410)	22.4 (14.2 to 33.1)	--	--	282 (182 to 411)	22.6 (14.5 to 32.8)	--	--	1.7 (-14.2 to 22)	1.1 (-13.5 to 18.9)	--	--
		Male	834 (539 to 1235)	66.1 (43 to 96.8)	--	--	849 (552 to 1266)	66.8 (43 to 99.4)	--	--	1.8 (-10.2 to 15)	1 (-11.2 to 14.5)	--	--
	Attention-deficit/hyperactivity disorder	Both	906 (504 to 1558)	32.4 (18.2 to 55.3)	--	--	771 (438 to 1308)	33.5 (18.8 to 57)	--	--	-14.9 (-27.9 to 0.2)	3.7 (-9.5 to 17.2)	--	--
		Female	277 (155 to 481)	20.1 (11.3 to 34.8)	--	--	237 (134 to 392)	20.8 (11.7 to 34.8)	--	--	-14.3 (-29.1 to 4.8)	3.9 (-12.5 to 24.1)	--	--
		Male	629 (349 to 1072)	44.5 (24.9 to 75.1)	--	--	534 (303 to 903)	45.8 (25.6 to 78.4)	--	--	-15.1 (-30.4 to 4)	3 (-14.1 to 21.6)	--	--
	Conduct disorder	Both	2464 (1389 to 3917)	75.4 (42.7 to 120)	--	--	1350 (767 to 2121)	74.3 (42.5 to 116.4)	--	--	-45.2 (-50.4 to -39.4)	-1.6 (-10.8 to 8.9)	--	--
		Female	845 (449 to 1377)	52.6 (28 to 85.4)	--	--	454 (240 to 751)	50.8 (26.8 to 84.3)	--	--	-46.3 (-54.1 to -36.2)	-3.3 (-17.1 to 14.7)	--	--
		Male	1618 (946 to 2541)	97.6 (56.9 to 153.4)	--	--	896 (518 to 1406)	96.8 (56 to 151.8)	--	--	-44.6 (-51 to -37.5)	-0.8 (-12.3 to 11.7)	--	--
	Idiopathic developmental intellectual disability	Both	2581 (1449 to 4114)	102.4 (57.6 to 162)	--	--	1468 (731 to 2447)	58.4 (29.1 to 98)	--	--	-43.1 (-52.2 to -37.4)	-43 (-52.6 to -36.9)	--	--
		Female	1138 (641 to 1825)	91.6 (52.2 to 145.8)	--	--	666 (337 to 1130)	53.4 (27.2 to 89.6)	--	--	-41.4 (-50.9 to -35)	-41.7 (-51.3 to -35.2)	--	--
		Male	1444 (807 to 2277)	113.1 (63.3 to 177.7)	--	--	801 (406 to 1330)	63.3 (32.4 to 105.4)	--	--	-44.5 (-53.4 to -38)	-44.1 (-53.3 to -37.4)	--	--
	Other mental disorders	Both	1723 (1107 to 2635)	100.2 (64.2 to 151.5)	--	--	3266 (2091 to 4946)	100.5 (64.7 to 151.8)	--	--	89.5 (75.4 to 104.9)	0.2 (-5.1 to 6.2)	--	--

Province	Cause	Sex	1990				2019				1990-2019 percent change (%)			
			DALYs		Death		DALYs		Death		DALYs		Death	
			Number	Rate	Number	Rate	Number	Rate	Number	Rate	Number	Rate	Number	Rate
		Female	702 (443 to 1066)	82.7 (52.6 to 126.1)	--	--	1345 (847 to 2070)	82.7 (52.1 to 126.9)	--	--	91.6 (72 to 113)	0 (-8.5 to 8.6)	--	--
		Male	1021 (645 to 1549)	118.1 (75.5 to 178.9)	--	--	1921 (1222 to 2896)	118.2 (75.7 to 178.7)	--	--	88.1 (71.9 to 106.3)	0.1 (-6.8 to 8.3)	--	--
	Substance use disorders	Both	4606 (3454 to 5819)	224.2 (171.1 to 280.3)	29 (19 to 37)	1.7 (1.3 to 2.2)	9751 (7793 to 11893)	327.3 (260 to 401.9)	79 (64 to 97)	2.6 (2.1 to 3.1)	111.7 (83.1 to 151.6)	46 (27.3 to 70.6)	177.3 (96.7 to 320.3)	46.4 (7.1 to 110.8)
		Female	1616 (1175 to 2140)	156.2 (116.4 to 202.9)	9 (6 to 13)	1.2 (0.8 to 1.6)	2382 (1786 to 3043)	161.3 (120.2 to 206.4)	14 (11 to 18)	0.9 (0.7 to 1.2)	47.4 (24.7 to 79.1)	3.3 (-12.6 to 22.8)	52.6 (-1.8 to 160.6)	-19.1 (-46.3 to 28.2)
		Male	2990 (2262 to 3787)	292.5 (223.6 to 366.3)	19 (13 to 26)	2.3 (1.6 to 3.1)	7369 (5851 to 8981)	488.8 (386.7 to 597.9)	65 (51 to 82)	4.2 (3.2 to 5.2)	146.5 (105.5 to 198.6)	67.1 (40.1 to 101.5)	237.2 (124.7 to 439.6)	77.8 (20.5 to 173)
	Alcohol use disorders	Both	910 (654 to 1208)	46.9 (33.8 to 62.1)	5 (4 to 7)	0.3 (0.2 to 0.5)	1229 (900 to 1637)	39.6 (28.7 to 52.5)	8 (6 to 11)	0.3 (0.2 to 0.3)	35.1 (17.3 to 57.1)	-15.5 (-26.2 to -2.1)	58.7 (7.5 to 142.5)	-23.1 (-47.8 to 16.5)
		Female	263 (184 to 361)	26.1 (18.4 to 35.6)	1 (1 to 1)	0.1 (0.1 to 0.2)	362 (248 to 504)	24.1 (16.6 to 33.5)	1 (1 to 2)	0.1 (0.1 to 0.1)	37.4 (13.8 to 67.6)	-7.4 (-22.4 to 10.2)	21.5 (-16.6 to 102.5)	-35.2 (-54.6 to -2)
		Male	647 (462 to 870)	67.7 (49.1 to 89.9)	4 (3 to 6)	0.5 (0.3 to 0.8)	867 (640 to 1160)	54.8 (40.2 to 73.2)	7 (5 to 10)	0.4 (0.3 to 0.6)	34.1 (11.9 to 61.7)	-18.9 (-32.3 to -2.9)	68.3 (4.7 to 178.9)	-19.9 (-49.3 to 32.9)
	Drug use disorders	Both	3696 (2758 to 4742)	177.4 (134.2 to 224)	24 (16 to 31)	1.4 (1.1 to 1.8)	8522 (6726 to 10550)	287.7 (225.4 to 357.2)	71 (57 to 87)	2.3 (1.8 to 2.8)	130.6 (96.4 to 177.4)	62.2 (39.2 to 93.5)	202.8 (113 to 367.2)	62.3 (18 to 133.7)
		Female	1353 (960 to 1844)	130.2 (94.4 to 174.2)	8 (5 to 11)	1 (0.7 to 1.4)	2020 (1516 to 2595)	137.2 (101.6 to 176)	13 (10 to 16)	0.8 (0.7 to 1.1)	49.3 (23.1 to 86.7)	5.4 (-12.3 to 27.2)	56.5 (0.2 to 174.2)	-17 (-45.4 to 31.6)
		Male	2343 (1742 to 3020)	224.8 (170.4 to 286.4)	15 (10 to 21)	1.8 (1.3 to 2.5)	6502 (5138 to 7984)	433.9 (339.4 to 533.8)	58 (45 to 74)	3.7 (2.9 to 4.8)	177.5 (127.1 to 242.2)	93 (59.4 to 136.4)	281.5 (148.8 to 513.9)	105.8 (36.3 to 218.6)
	Diabetes and kidney diseases	Both	12386 (10286 to 14752)	878.6 (738.7 to 1046.6)	257 (211 to 311)	26.4 (22.1 to 33.1)	44163 (36928 to 52527)	1430 (1202.8 to 1692.4)	1103 (970 to 1251)	39 (34.2 to 44.1)	256.6 (206.5 to 314.2)	62.8 (39.7 to 86.3)	329.6 (236.8 to 432.8)	47.8 (11.3 to 81.2)
		Female	6137 (4928 to 7522)	864.8 (703.5 to 1088.6)	127 (98 to 170)	25.4 (19.8 to 36.2)	23394 (19045 to 28199)	1506.4 (1237.3 to 1797.9)	593 (492 to 705)	42.3 (35.1 to 50.1)	281.2 (204.6 to 361.7)	74.2 (37.3 to 108.3)	367 (223.3 to 549.9)	66.9 (9.3 to 126.2)
		Male	6249 (4981 to 7568)	890 (723.5 to 1072.1)	130 (99 to 167)	27.4 (21.2 to 34.1)	20769 (17255 to 24854)	1352.8 (1127.9 to 1614)	509 (411 to 610)	35.8 (29.2 to 42.5)	232.4 (176.2 to 309.7)	52 (26.9 to 84.5)	292.9 (191 to 443.5)	30.8 (-1 to 74.9)
	Diabetes mellitus	Both	4834 (3737 to 6238)	352.1 (273 to 457)	58 (46 to 98)	5.7 (4.5 to 10.8)	30029 (23764 to 37182)	950.4 (752.7 to 1175.1)	561 (480 to 649)	19.4 (16.7 to 22.4)	521.3 (402.3 to 600.7)	169.9 (105.9 to 204.2)	863.7 (406.3 to 1188.7)	241.5 (61.3 to 352.9)
		Female	2418 (1829 to 3312)	351.2 (267.3 to 492)	30 (22 to 66)	5.7 (4.2 to 13.5)	16485 (12877 to 20545)	1034.1 (813.1 to 1283.8)	323 (256 to 393)	22.4 (17.9 to 27.3)	581.8 (360.4 to 711.7)	194.4 (88 to 252)	966.3 (278.3 to 1526.1)	292.1 (31.2 to 506.5)
		Male	2416 (1838 to 3064)	353.4 (271.4 to 447)	28 (22 to 36)	5.6 (4.4 to 7.1)	13544 (10663 to 16768)	864.8 (683.9 to 1067.7)	239 (190 to 291)	16.5 (13.1 to 20)	460.6 (387.8 to 553.6)	144.7 (113.2 to 184.5)	752.9 (502.5 to 1117.3)	193.2 (111.9 to 299)
	Chronic kidney disease	Both	7528 (6259 to 8924)	525.3 (445.8 to 619.4)	198 (163 to 237)	20.6 (17.1 to 25.6)	13786 (12026 to 15654)	467.3 (406.9 to 529.4)	527 (457 to 603)	19 (16.5 to 21.8)	83.1 (53.6 to 123.4)	-11.1 (-25 to 5.9)	166.1 (113.2 to 235.5)	-7.8 (-27.9 to 14.6)
		Female	3708 (2984 to 4524)	512.5 (421.2 to 626.3)	97 (75 to 125)	19.6 (15.2 to 27.2)	6735 (5705 to 7873)	459.5 (389.2 to 535)	262 (213 to 312)	19.3 (15.7 to 23)	81.7 (47.2 to 132.3)	-10.3 (-28.4 to 13.6)	171.9 (99.3 to 276.5)	-1.5 (-34.2 to 34.3)

Province	Cause	Sex	1990				2019				1990-2019 percent change (%)			
			DALYs		Death		DALYs		Death		DALYs		Death	
			Number	Rate	Number	Rate	Number	Rate	Number	Rate	Number	Rate	Number	Rate
		Male	3821 (2969 to 4808)	535.5 (426.6 to 667.3)	101 (75 to 132)	21.7 (16.6 to 27.2)	7051 (5897 to 8375)	476.1 (399.7 to 562.4)	264 (216 to 319)	18.9 (15.4 to 22.7)	84.5 (42 to 146.5)	-11.1 (-31.2 to 15.8)	160.6 (87.9 to 270.1)	-13.1 (-36.2 to 21.1)
	Acute glomerulonephritis	Both	24 (12 to 117)	1.1 (0.7 to 4.8)	0 (0 to 2)	0 (0 to 0.1)	349 (45 to 457)	12.4 (1.9 to 16.2)	15 (2 to 19)	0.5 (0.1 to 0.7)	1341.2 (-64.2 to 3144.3)	978.4 (-61.5 to 2048.1)	2859 (-17.9 to 5975.8)	1359.4 (-52 to 2767.1)
		Female	12 (6 to 32)	1.1 (0.6 to 2.8)	0 (0 to 1)	0 (0 to 0.1)	174 (23 to 243)	12.8 (1.9 to 18)	8 (1 to 12)	0.6 (0.1 to 0.9)	1392 (-49.8 to 3741.8)	1040.5 (-48.9 to 2622.3)	3264.3 (19.9 to 8007.5)	1629.3 (-33.6 to 3793.7)
		Male	13 (5 to 82)	1.2 (0.5 to 6.5)	0 (0 to 1)	0 (0 to 0.2)	175 (16 to 258)	11.9 (1.3 to 17.4)	6 (1 to 9)	0.4 (0 to 0.7)	1293.9 (-78.8 to 4396.7)	922.7 (-75.5 to 2795.8)	2464.7 (-53.6 to 7556.8)	1118.4 (-72.2 to 3307.2)
	Skin and subcutaneous diseases	Both	10024 (6670 to 14708)	412.7 (275 to 603.6)	2 (1 to 3)	0.2 (0.1 to 0.4)	10551 (7014 to 15768)	421.7 (280.3 to 634.3)	7 (6 to 9)	0.3 (0.2 to 0.3)	5.3 (0.5 to 9.6)	2.2 (-0.8 to 4.9)	290.3 (141.4 to 485.8)	21.4 (-32.4 to 91.4)
		Female	5279 (3499 to 7664)	437.2 (293.7 to 634.9)	1 (1 to 2)	0.2 (0.1 to 0.4)	5527 (3664 to 8211)	445.5 (293.5 to 664.2)	3 (3 to 4)	0.3 (0.2 to 0.3)	4.7 (-0.6 to 9.9)	1.9 (-1.8 to 5.2)	265.3 (67 to 547.6)	23.6 (-49.6 to 128.5)
		Male	4745 (3159 to 7045)	388.4 (258.8 to 572.6)	1 (1 to 2)	0.2 (0.1 to 0.5)	5024 (3374 to 7557)	398.6 (265 to 603.2)	3 (3 to 5)	0.3 (0.2 to 0.4)	5.9 (0.4 to 11)	2.6 (-1.2 to 6.4)	318.5 (179.1 to 558.6)	18.5 (-27.3 to 103.8)
	Dermatitis	Both	2288 (1356 to 3595)	93.6 (56.3 to 144)	--	--	2408 (1481 to 3649)	94 (57.2 to 145.6)	--	--	5.2 (-4.1 to 16.1)	0.5 (-4.4 to 6)	--	--
		Female	1258 (742 to 1985)	103.7 (62.5 to 161.8)	--	--	1322 (808 to 2005)	104.3 (62.4 to 161.6)	--	--	5.1 (-5.5 to 17)	0.6 (-5.7 to 7.6)	--	--
		Male	1030 (607 to 1598)	83.5 (50.1 to 126.6)	--	--	1085 (667 to 1646)	84.1 (51.1 to 129.3)	--	--	5.4 (-5.5 to 18.6)	0.7 (-6.5 to 8)	--	--
	Psoriasis	Both	948 (670 to 1273)	47.3 (33.4 to 63)	--	--	1015 (715 to 1362)	33.7 (23.7 to 45.3)	--	--	7 (-4.2 to 18.7)	-28.7 (-35.6 to -21.8)	--	--
		Female	495 (347 to 663)	49.1 (34.3 to 65.8)	--	--	520 (365 to 692)	35 (24.4 to 46.5)	--	--	5.1 (-10.2 to 22.1)	-28.7 (-38.1 to -17.7)	--	--
		Male	453 (319 to 612)	45.6 (31.6 to 61.1)	--	--	495 (344 to 679)	32.5 (22.6 to 44.6)	--	--	9.2 (-7.3 to 26.2)	-28.6 (-38.5 to -18.7)	--	--
	Bacterial skin diseases	Both	144 (94 to 237)	7.2 (5.1 to 10.9)	1 (1 to 2)	0.2 (0.1 to 0.3)	189 (137 to 283)	7.1 (5 to 10.8)	4 (3 to 6)	0.2 (0.1 to 0.2)	31.8 (14.8 to 61.2)	-2.2 (-17 to 17.6)	181.2 (99.7 to 338.5)	-6.4 (-36 to 63.7)
		Female	67 (44 to 111)	6.8 (4.6 to 10.4)	1 (0 to 1)	0.2 (0.1 to 0.3)	85 (59 to 126)	6.4 (4.4 to 9.8)	2 (1 to 3)	0.1 (0.1 to 0.2)	26.6 (5.7 to 68.7)	-5.7 (-27.4 to 27.6)	143.9 (41.5 to 431.9)	-14.1 (-51.8 to 121.1)
		Male	77 (48 to 124)	7.7 (5.1 to 12.1)	1 (0 to 1)	0.2 (0.1 to 0.4)	105 (75 to 154)	7.7 (5.4 to 11.6)	2 (2 to 4)	0.2 (0.1 to 0.3)	36.4 (15.7 to 68.1)	0.6 (-19.5 to 22.4)	220.2 (118.2 to 388)	-1.9 (-34 to 63.9)
	Scabies	Both	633 (347 to 1018)	25.1 (13.8 to 40.4)	--	--	618 (345 to 999)	25.1 (13.8 to 40.1)	--	--	-2.3 (-10 to 6.4)	-0.2 (-4.7 to 4.6)	--	--
		Female	315 (172 to 510)	25.2 (13.8 to 40.4)	--	--	306 (170 to 494)	25.2 (13.8 to 40.5)	--	--	-2.9 (-11.2 to 7)	0 (-6.3 to 6.7)	--	--
		Male	317 (172 to 513)	25 (13.8 to 40.3)	--	--	312 (174 to 504)	25 (13.7 to 40.3)	--	--	-1.7 (-10.5 to 9.1)	-0.3 (-6.9 to 6.4)	--	--
	Fungal skin diseases	Both	329 (131 to 709)	18.7 (7.5 to 39.7)	--	--	525 (211 to 1125)	18.4 (7.4 to 39.3)	--	--	59.8 (49.4 to 70.9)	-1.2 (-3 to 0.7)	--	--
		Female	153 (62 to 325)	17.5 (7.1 to 38.2)	--	--	246 (99 to 535)	17.2 (6.9 to 37.7)	--	--	60.9 (49.8 to 72.7)	-1.5 (-4 to 1)	--	--

Province	Cause	Sex	1990				2019				1990-2019 percent change (%)			
			DALYs		Death		DALYs		Death		DALYs		Death	
			Number	Rate	Number	Rate	Number	Rate	Number	Rate	Number	Rate	Number	Rate
		Male	176 (70 to 381)	19.9 (8 to 42.6)	--	--	279 (113 to 603)	19.6 (7.9 to 41.8)	--	--	58.8 (47.4 to 70.5)	-1.5 (-3.9 to 0.9)	--	--
	Viral skin diseases	Both	1498 (947 to 2264)	51.3 (32.6 to 77.2)	--	--	1094 (700 to 1624)	51.4 (32.6 to 75.9)	--	--	-27 (-30 to -23.4)	0.3 (-3.7 to 4.3)	--	--
		Female	733 (463 to 1108)	50.9 (32.3 to 77)	--	--	536 (341 to 803)	51 (32.2 to 75.8)	--	--	-26.9 (-31.4 to -21.8)	0.2 (-5.2 to 5.9)	--	--
		Male	765 (487 to 1142)	51.6 (33 to 76.7)	--	--	559 (355 to 832)	51.8 (32.9 to 77.6)	--	--	-27 (-31.5 to -22.3)	0.3 (-5.4 to 6.4)	--	--
	Acne vulgaris	Both	1526 (905 to 2418)	52.2 (31 to 83.1)	--	--	1486 (893 to 2363)	71.4 (43 to 114.2)	--	--	-2.6 (-9.9 to 5.7)	36.8 (26.9 to 48.1)	--	--
		Female	731 (431 to 1158)	50.7 (30 to 80.4)	--	--	717 (432 to 1124)	69.5 (41.7 to 108.8)	--	--	-1.8 (-11.6 to 9.5)	37 (23.7 to 52.3)	--	--
		Male	795 (471 to 1258)	53.7 (31.7 to 84.9)	--	--	768 (454 to 1212)	73.3 (43.7 to 115.8)	--	--	-3.3 (-13.8 to 8.2)	36.5 (21.6 to 52.4)	--	--
	Alopecia areata	Both	136 (85 to 207)	6.4 (4 to 9.7)	--	--	188 (119 to 280)	6.4 (4 to 9.5)	--	--	38 (23.4 to 53)	-0.3 (-10.2 to 9.9)	--	--
		Female	88 (55 to 135)	8.3 (5.2 to 12.7)	--	--	121 (77 to 179)	8.3 (5.3 to 12.4)	--	--	37.9 (19.2 to 58.1)	0 (-12.1 to 13.2)	--	--
		Male	48 (30 to 73)	4.5 (2.8 to 6.8)	--	--	66 (42 to 101)	4.5 (2.8 to 6.9)	--	--	38.2 (13.2 to 66)	-0.1 (-17 to 18.3)	--	--
	Pruritus	Both	202 (97 to 364)	10 (4.8 to 18)	--	--	305 (145 to 553)	10.6 (5 to 18.9)	--	--	51.3 (38.7 to 65.8)	5.1 (-0.1 to 10.7)	--	--
		Female	110 (52 to 198)	11 (5.3 to 20)	--	--	166 (79 to 304)	11.5 (5.5 to 21)	--	--	51.6 (36.4 to 68.6)	5.2 (-2.1 to 12.9)	--	--
		Male	92 (44 to 165)	9.1 (4.4 to 16.2)	--	--	139 (66 to 250)	9.6 (4.5 to 17.1)	--	--	50.9 (36.5 to 67.4)	5.3 (-1.7 to 13.8)	--	--
	Urticaria	Both	1521 (997 to 2233)	58.3 (38.2 to 84.5)	--	--	1399 (924 to 1993)	58.4 (38.5 to 84.1)	--	--	-8 (-16.4 to 1.7)	0.2 (-5.7 to 5.6)	--	--
		Female	926 (601 to 1344)	71 (46.4 to 102)	--	--	833 (553 to 1181)	71.1 (46.8 to 102.4)	--	--	-10 (-18.9 to 1.1)	0.2 (-7.1 to 7.5)	--	--
		Male	595 (384 to 874)	45.8 (30.1 to 66.5)	--	--	565 (366 to 802)	46 (29.6 to 66.2)	--	--	-5 (-16.9 to 8.3)	0.5 (-8.5 to 10)	--	--
	Decubitus ulcer	Both	9 (6 to 17)	0.9 (0.6 to 2)	0 (0 to 1)	0 (0 to 0.1)	46 (32 to 57)	1.7 (1.2 to 2.1)	2 (1 to 3)	0.1 (0.1 to 0.1)	421.1 (121.9 to 667.9)	83.7 (-33.5 to 177.1)	1016.3 (145 to 2395.7)	136.4 (-46 to 417.4)
		Female	4 (3 to 9)	0.9 (0.6 to 2.2)	0 (0 to 0)	0 (0 to 0.2)	25 (14 to 34)	1.9 (1 to 2.5)	1 (1 to 2)	0.1 (0 to 0.2)	480.7 (87.3 to 894.9)	108.9 (-40.1 to 271.9)	952.3 (58.3 to 2714.1)	174.9 (-58.5 to 629.9)
		Male	4 (3 to 9)	0.9 (0.6 to 2.2)	0 (0 to 0)	0 (0 to 0.1)	21 (15 to 26)	1.5 (1.1 to 1.9)	1 (1 to 1)	0.1 (0 to 0.1)	362.7 (145.8 to 543.5)	61.9 (-26.5 to 134)	1135 (243 to 2840)	105.3 (-38.9 to 392.7)
	Other skin and subcutaneous diseases	Both	791 (378 to 1450)	41.7 (20 to 75.7)	0 (0 to 0)	0 (0 to 0)	1278 (617 to 2299)	43.6 (21 to 79)	0 (0 to 1)	0 (0 to 0)	61.5 (57.4 to 67)	4.5 (2.2 to 7.1)	579 (244.8 to 1354.4)	39.5 (-34 to 231.6)
		Female	398 (187 to 737)	42.3 (20 to 77)	0 (0 to 0)	0 (0 to 0)	647 (311 to 1161)	44.1 (21.3 to 79.5)	0 (0 to 0)	0 (0 to 0)	62.5 (56.5 to 69)	4.3 (0.9 to 7.6)	358.7 (70.2 to 1386.4)	11.7 (-58.5 to 274.8)

Province	Cause	Sex	1990				2019				1990-2019 percent change (%)			
			DALYs		Death		DALYs		Death		DALYs		Death	
			Number	Rate	Number	Rate	Number	Rate	Number	Rate	Number	Rate	Number	Rate
		Male	393 (188 to 718)	41.2 (19.9 to 74.9)	0 (0 to 0)	0 (0 to 0)	630 (303 to 1148)	43.1 (20.7 to 78.5)	0 (0 to 0)	0 (0 to 0)	60.6 (55 to 67.7)	4.6 (1.6 to 8.2)	972.8 (474.3 to 1913.9)	88.1 (0.1 to 276.4)
	Sense organ diseases	Both	13686 (9682 to 18555)	991.7 (706.5 to 1322.8)	--	--	22288 (15720 to 30428)	759.3 (536.8 to 1027.9)	--	--	62.9 (53.8 to 72)	-23.4 (-26.8 to -19.8)	--	--
		Female	6888 (4914 to 9358)	992.6 (711.4 to 1324.7)	--	--	11175 (7907 to 15115)	758.1 (537 to 1018.9)	--	--	62.2 (53.1 to 71.4)	-23.6 (-27.3 to -19.6)	--	--
		Male	6798 (4737 to 9270)	987.9 (700.2 to 1323.5)	--	--	11114 (7790 to 15300)	760.6 (534.8 to 1042.3)	--	--	63.5 (52.8 to 74.1)	-23 (-26.9 to -19.2)	--	--
	Blindness and vision loss	Both	6086 (4289 to 8325)	464.8 (328.6 to 625.3)	--	--	9428 (6675 to 12943)	326.5 (231.6 to 446.4)	--	--	54.9 (47.5 to 62.3)	-29.8 (-33.4 to -25.7)	--	--
		Female	3253 (2308 to 4431)	489.2 (349.5 to 656.5)	--	--	5020 (3525 to 6858)	344.8 (245.3 to 469.4)	--	--	54.3 (45.9 to 62.8)	-29.5 (-33.7 to -25)	--	--
		Male	2833 (1982 to 3892)	437.6 (309.4 to 589.9)	--	--	4409 (3117 to 6062)	307.7 (217.6 to 420.9)	--	--	55.6 (46.9 to 64.3)	-29.7 (-33.9 to -25.6)	--	--
	Age-related and other hearing loss	Both	6895 (4759 to 9679)	485.2 (344.4 to 672.8)	--	--	11567 (8046 to 16228)	389.4 (272.6 to 539.3)	--	--	67.8 (52 to 83.4)	-19.7 (-23.6 to -15.7)	--	--
		Female	3255 (2256 to 4507)	458.2 (323.6 to 632.4)	--	--	5453 (3787 to 7635)	366.4 (256.4 to 509.6)	--	--	67.5 (50.6 to 83.2)	-20 (-24.7 to -15.3)	--	--
		Male	3640 (2471 to 5154)	512.3 (358.4 to 716)	--	--	6114 (4223 to 8572)	413 (288.5 to 573.5)	--	--	68 (51.1 to 85.4)	-19.4 (-24.1 to -14.7)	--	--
	Other sense organ diseases	Both	705 (421 to 1091)	41.7 (25.1 to 64.5)	--	--	1293 (757 to 2020)	43.4 (25.8 to 67.2)	--	--	83.4 (66.9 to 101.4)	4.1 (-0.9 to 9.5)	--	--
		Female	381 (229 to 589)	45.1 (27.2 to 69.7)	--	--	702 (409 to 1105)	46.9 (27.7 to 73)	--	--	84.5 (65.9 to 104.8)	4 (-3 to 11.7)	--	--
		Male	325 (191 to 499)	38.1 (22.8 to 59)	--	--	591 (347 to 915)	39.9 (23.8 to 61.4)	--	--	82 (62.9 to 102.8)	4.8 (-2.9 to 12.8)	--	--
	Musculoskeletal disorders	Both	30677 (21556 to 41222)	1829.6 (1295.5 to 2440)	5 (3 to 9)	0.4 (0.3 to 0.7)	61184 (43038 to 81852)	1905.1 (1355.2 to 2537.7)	21 (16 to 25)	0.7 (0.6 to 0.9)	99.4 (89 to 109.9)	4.1 (0.7 to 7.9)	333.9 (138.6 to 587.8)	80.4 (-1.3 to 178.8)
		Female	16528 (11707 to 21970)	1984.1 (1409.3 to 2637.6)	3 (2 to 7)	0.5 (0.3 to 1.1)	34043 (23993 to 45276)	2114.7 (1504.7 to 2812.3)	14 (10 to 18)	1 (0.7 to 1.3)	106 (94.4 to 117.8)	6.6 (2.7 to 11.1)	343.4 (99.4 to 727.9)	99.8 (-13.2 to 266.5)
		Male	14149 (9766 to 18987)	1672.8 (1163.6 to 2242.9)	2 (1 to 3)	0.3 (0.2 to 0.5)	27141 (19098 to 36507)	1694.3 (1196.8 to 2271.4)	7 (5 to 8)	0.5 (0.4 to 0.6)	91.8 (80.8 to 103.2)	1.3 (-3.3 to 6.2)	315.8 (94.7 to 532.9)	59.6 (-11.8 to 131.5)
	Rheumatoid arthritis	Both	201 (137 to 275)	12.1 (8.3 to 16.7)	1 (0 to 2)	0.1 (0 to 0.2)	463 (334 to 608)	14.2 (10.3 to 18.7)	3 (2 to 4)	0.1 (0.1 to 0.1)	130.9 (88.3 to 174.7)	17.6 (-5.6 to 38.9)	388.3 (41.3 to 965.4)	62.5 (-54.8 to 275)
		Female	141 (96 to 197)	16.9 (11.4 to 24.1)	1 (0 to 2)	0.1 (0 to 0.3)	320 (225 to 423)	19.6 (13.9 to 26)	2 (1 to 3)	0.1 (0.1 to 0.2)	126.7 (73 to 180.9)	15.6 (-14.1 to 42.8)	300.9 (-19 to 1117.6)	42.8 (-72.2 to 354.8)
		Male	60 (41 to 82)	7.2 (5 to 9.8)	0 (0 to 0)	0 (0 to 0)	143 (104 to 193)	8.9 (6.5 to 11.8)	1 (1 to 2)	0.1 (0.1 to 0.1)	141 (103.6 to 187.6)	23.1 (4.2 to 47.5)	679.1 (385.2 to 1311.3)	157 (61.9 to 353.7)
	Osteoarthritis	Both	2367 (1185 to 4654)	179.5 (90.8 to 351.5)	--	--	6196 (3122 to 12204)	191.8 (96.5 to 376.9)	--	--	161.7 (152.4 to 171.7)	6.8 (3.4 to 10.5)	--	--

Province	Cause	Sex	1990				2019				1990-2019 percent change (%)			
			DALYs		Death		DALYs		Death		DALYs		Death	
			Number	Rate	Number	Rate	Number	Rate	Number	Rate	Number	Rate	Number	Rate
		Female	1327 (674 to 2628)	201.8 (102.3 to 398.4)	--	--	3563 (1804 to 7016)	217.7 (109.9 to 428.3)	--	--	168.4 (156.3 to 181.6)	7.9 (3.4 to 12.9)	--	--
		Male	1040 (517 to 2064)	155.8 (77.7 to 307.9)	--	--	2633 (1318 to 5245)	165.2 (82.7 to 331)	--	--	153.2 (140.1 to 167.3)	6 (1.2 to 11.5)	--	--
	Low back pain	Both	17902 (12567 to 23987)	1024.6 (719.4 to 1368.3)	--	--	28819 (20223 to 39035)	918.7 (647 to 1235.2)	--	--	61 (50.3 to 72.4)	-10.3 (-14.3 to -6.2)	--	--
		Female	8495 (5952 to 11423)	975.3 (687 to 1310.6)	--	--	14216 (9975 to 19208)	911.3 (642.6 to 1219.2)	--	--	67.3 (53.3 to 82.2)	-6.6 (-11.9 to -0.3)	--	--
		Male	9407 (6629 to 12637)	1070.9 (748.4 to 1432.4)	--	--	14603 (10131 to 19801)	926.2 (646.2 to 1248.2)	--	--	55.2 (43.3 to 67.4)	-13.5 (-18.8 to -8.4)	--	--
	Neck pain	Both	6703 (4385 to 9775)	405.6 (267.7 to 587.3)	--	--	13413 (8851 to 19398)	405.5 (266.9 to 584.9)	--	--	100.1 (86.9 to 111.2)	0 (-2 to 2)	--	--
		Female	4053 (2695 to 5926)	493.1 (327.4 to 717.5)	--	--	8187 (5396 to 12024)	492.5 (327.8 to 716.9)	--	--	102 (88.5 to 114.2)	-0.1 (-2.6 to 2.5)	--	--
		Male	2650 (1698 to 3883)	317.8 (207.4 to 461.2)	--	--	5225 (3397 to 7679)	318.1 (208.2 to 464)	--	--	97.2 (84 to 109.4)	0.1 (-3.2 to 3.4)	--	--
	Gout	Both	194 (116 to 285)	13.8 (8.5 to 20.1)	--	--	492 (303 to 713)	15.3 (9.5 to 22.2)	--	--	153.4 (119.3 to 196.2)	10.3 (-2.9 to 26.5)	--	--
		Female	42 (25 to 63)	6.2 (3.7 to 9)	--	--	113 (68 to 170)	7 (4.2 to 10.5)	--	--	166.8 (109 to 243)	12.6 (-10.3 to 42.8)	--	--
		Male	152 (90 to 225)	22.1 (13.6 to 32.5)	--	--	379 (231 to 552)	23.7 (14.6 to 34.7)	--	--	149.7 (108.9 to 199.9)	7.3 (-7.9 to 26.1)	--	--
	Other musculoskeletal disorders	Both	3310 (1880 to 4997)	194 (111.1 to 294.7)	4 (3 to 7)	0.3 (0.2 to 0.6)	11801 (7537 to 17036)	359.6 (232.7 to 516.7)	18 (14 to 21)	0.6 (0.5 to 0.8)	256.5 (208.4 to 325.3)	85.4 (60 to 124.9)	325.2 (149.7 to 581.9)	84 (13.9 to 183.7)
		Female	2470 (1543 to 3574)	290.7 (184.2 to 423.1)	3 (1 to 6)	0.4 (0.2 to 0.8)	7644 (5101 to 10825)	466.6 (312.6 to 658.8)	12 (9 to 16)	0.8 (0.6 to 1.1)	209.4 (171.8 to 258.1)	60.5 (41 to 87.4)	351.6 (136.9 to 751.4)	113.6 (11 to 299.2)
		Male	840 (329 to 1520)	99 (39.2 to 177.2)	2 (1 to 3)	0.3 (0.2 to 0.4)	4157 (2426 to 6298)	252.2 (148.8 to 378.2)	6 (5 to 7)	0.4 (0.3 to 0.5)	395 (269.3 to 760.2)	154.9 (89.4 to 336.1)	279.1 (83.8 to 476.4)	48.5 (-17.4 to 116)
	Other non-communicable diseases	Both	93153 (59988 to 128087)	3649.8 (2444.7 to 4912.6)	777 (420 to 1156)	27.4 (15.2 to 40.3)	46690 (35170 to 61452)	1733.8 (1322.5 to 2230.4)	151 (128 to 180)	7.5 (6.1 to 9.3)	-49.9 (-66.4 to -21.9)	-52.5 (-66.5 to -30)	-80.5 (-87.9 to -61.5)	-72.7 (-83.3 to -46.1)
		Female	49457 (33678 to 67883)	4106.3 (2870 to 5508.9)	353 (193 to 546)	25.5 (14.3 to 39.3)	32297 (23691 to 42878)	2281.2 (1707.7 to 2988.2)	72 (59 to 88)	7.1 (5.8 to 9.2)	-34.7 (-55.1 to -5.7)	-44.4 (-59.8 to -24.4)	-79.7 (-88.1 to -59.3)	-72.1 (-83.8 to -42.4)
		Male	43696 (25390 to 63440)	3185.5 (1945.9 to 4562.2)	423 (216 to 643)	29.2 (15.3 to 44.1)	14393 (10821 to 19032)	1193.6 (914.6 to 1539)	80 (64 to 101)	7.8 (6.2 to 10.6)	-67.1 (-79.8 to -38.9)	-62.5 (-75.9 to -32.5)	-81.1 (-88.6 to -57.9)	-73.2 (-83.7 to -38.7)
	Congenital birth defects	Both	64623 (33822 to 97781)	2216.1 (1165.9 to 3353.5)	708 (359 to 1082)	24.3 (12.4 to 37)	8032 (6261 to 10330)	468.9 (354.5 to 623.4)	74 (57 to 99)	4.5 (3.3 to 6.2)	-87.6 (-92.8 to -73.1)	-78.8 (-88 to -53.4)	-89.5 (-94.1 to -75.7)	-81.4 (-89.9 to -55.8)
		Female	29049 (14917 to 45100)	2031.4 (1051.8 to 3142.9)	320 (160 to 505)	22.4 (11.2 to 35.3)	3567 (2703 to 4795)	424.9 (314.4 to 601.4)	33 (25 to 48)	4.2 (3 to 6.2)	-87.7 (-93.2 to -71.8)	-79.1 (-88.7 to -50.5)	-89.5 (-94.5 to -74.1)	-81.4 (-90.4 to -53)

Province	Cause	Sex	1990				2019				1990-2019 percent change (%)			
			DALYs		Death		DALYs		Death		DALYs		Death	
			Number	Rate	Number	Rate	Number	Rate	Number	Rate	Number	Rate	Number	Rate
		Male	35574 (17032 to 54737)	2392.3 (1154.1 to 3663.8)	388 (177 to 604)	26.1 (12 to 40.5)	4465 (3406 to 6344)	511.1 (379.9 to 763.5)	41 (31 to 61)	4.9 (3.5 to 7.5)	-87.4 (-92.7 to -69.1)	-78.6 (-87.8 to -45.6)	-89.4 (-94 to -71.3)	-81.4 (-89.7 to -47.9)
	Urinary diseases and male infertility	Both	450 (311 to 638)	25.3 (16.9 to 36.7)	2 (1 to 5)	0.2 (0.1 to 0.7)	870 (623 to 1192)	29.2 (21.1 to 39.8)	9 (7 to 15)	0.3 (0.3 to 0.6)	93.3 (70.3 to 118)	15.6 (-7.1 to 30)	406.3 (150.4 to 712.8)	111.6 (-27.3 to 321.3)
		Female	143 (103 to 199)	13.8 (9.6 to 23.5)	1 (1 to 4)	0.2 (0.1 to 0.9)	243 (187 to 308)	17.2 (13.2 to 22)	5 (4 to 9)	0.4 (0.3 to 0.7)	70.6 (38.7 to 113.5)	24.2 (-17.1 to 60.4)	389.9 (119.9 to 807.6)	110.8 (-23.1 to 364.4)
		Male	308 (198 to 470)	37.7 (24.3 to 55.9)	1 (0 to 2)	0.1 (0.1 to 0.6)	627 (424 to 911)	41.6 (28.3 to 60.1)	4 (3 to 6)	0.3 (0.2 to 0.5)	103.8 (78.1 to 131.8)	10.4 (-5 to 21.8)	430.7 (181.1 to 745)	141.4 (-26.4 to 363.9)
	Gynecological diseases	Female	10220 (6988 to 14310)	1085.8 (744.1 to 1510.3)	0 (0 to 0)	0 (0 to 0)	16839 (11466 to 23347)	1039.5 (714.6 to 1443.6)	0 (0 to 1)	0 (0 to 0)	64.8 (55.2 to 74.4)	-4.3 (-7.4 to -1.4)	352.3 (-47.2 to 1524.1)	108.2 (-70.1 to 599.8)
	Hemoglobinopathies and hemolytic anemias	Both	4230 (3094 to 5697)	165.4 (120.2 to 226.5)	24 (17 to 31)	1 (0.7 to 1.2)	1886 (1380 to 2639)	77.7 (58 to 105.7)	14 (12 to 16)	0.6 (0.5 to 0.7)	-55.4 (-65.2 to -42.3)	-53 (-62.9 to -40.7)	-39.5 (-57.2 to -10.4)	-40 (-55 to -14.9)
		Female	2555 (1748 to 3623)	207.6 (144.3 to 294.2)	12 (7 to 18)	1 (0.7 to 1.4)	1134 (763 to 1656)	90.8 (62.9 to 129.7)	7 (5 to 8)	0.6 (0.4 to 0.7)	-55.6 (-68.3 to -37.7)	-56.3 (-68.3 to -40.1)	-44.6 (-65.4 to -4.6)	-44.6 (-63.5 to -12.1)
		Male	1675 (1204 to 2252)	121.8 (89.3 to 162.7)	12 (8 to 15)	0.9 (0.7 to 1.2)	751 (566 to 1034)	64.9 (47.8 to 91.1)	8 (6 to 9)	0.6 (0.5 to 0.7)	-55.1 (-67.6 to -38.5)	-46.7 (-61.4 to -27.7)	-34.3 (-54.1 to 0.3)	-35.1 (-53 to -7.5)
	Endocrine, metabolic, blood, and immune disorders	Both	6393 (4466 to 8888)	311 (216.8 to 433)	20 (12 to 40)	1.2 (0.8 to 2.6)	9167 (6536 to 12332)	318.1 (227.9 to 428.9)	51 (39 to 61)	1.9 (1.5 to 2.3)	43.4 (13.1 to 65.1)	2.3 (-15 to 14.4)	161.7 (21.3 to 352.3)	56.5 (-27.3 to 175.1)
		Female	3984 (2699 to 5600)	390.7 (265.6 to 542.6)	9 (5 to 22)	1.2 (0.7 to 3.1)	5700 (3980 to 7823)	394.6 (277.7 to 543.5)	25 (17 to 32)	1.9 (1.2 to 2.4)	43.1 (14.8 to 63.8)	1 (-15.2 to 14.2)	167.3 (7.4 to 407.7)	55.4 (-34.7 to 203.4)
		Male	2410 (1661 to 3523)	232.1 (161.1 to 324)	10 (6 to 22)	1.2 (0.8 to 2.6)	3467 (2516 to 4602)	242.8 (178.6 to 320.6)	26 (17 to 35)	2 (1.3 to 2.6)	43.9 (5.6 to 76.6)	4.6 (-17.3 to 22.7)	156.7 (24.4 to 381.1)	58.4 (-19.2 to 190.8)
	Oral disorders	Both	5184 (3074 to 8126)	317.8 (189.2 to 494.1)	--	--	9734 (5765 to 15427)	312.2 (185.5 to 489.5)	--	--	87.7 (79.3 to 96.6)	-1.8 (-4.3 to 0.5)	--	--
		Female	2545 (1499 to 3984)	309.2 (184.1 to 487)	--	--	4734 (2786 to 7561)	302.9 (178 to 479)	--	--	86 (76.5 to 96)	-2 (-5.5 to 0.7)	--	--
		Male	2640 (1562 to 4148)	328.1 (195.4 to 510.8)	--	--	4999 (2980 to 7836)	322 (190.7 to 502.7)	--	--	89.4 (80.4 to 99.9)	-1.9 (-5.1 to 1.5)	--	--
	Sudden infant death syndrome	Both	2052 (466 to 6550)	70.7 (16.1 to 225.5)	23 (5 to 74)	0.8 (0.2 to 2.6)	162 (68 to 346)	11.3 (4.8 to 24.3)	2 (1 to 4)	0.1 (0.1 to 0.3)	-92.1 (-98 to -53.1)	-83.9 (-96 to -4.6)	-92.1 (-98 to -53.1)	-83.9 (-96 to -4.5)
		Female	962 (208 to 3250)	67.7 (14.6 to 228.8)	11 (2 to 37)	0.8 (0.2 to 2.6)	79 (28 to 182)	11.4 (4 to 26.2)	1 (0 to 2)	0.1 (0 to 0.3)	-91.8 (-98 to -54.5)	-83.2 (-95.8 to -6.7)	-91.8 (-98 to -54.5)	-83.2 (-95.8 to -6.7)
		Male	1090 (193 to 3589)	73.5 (13 to 241.9)	12 (2 to 41)	0.8 (0.1 to 2.7)	83 (33 to 180)	11.3 (4.6 to 24.7)	1 (0 to 2)	0.1 (0.1 to 0.3)	-92.4 (-98.1 to -49.8)	-84.6 (-96 to 1.9)	-92.4 (-98.1 to -49.8)	-84.6 (-96 to 1.8)
	**Injuries**	Both	1965233 (1794884 to 2154552)	77571.7 (70904.2 to 84954)	30720 (28053 to 33685)	1430.3 (1306 to 1568)	88597 (78529 to 100921)	3117.8 (2777.6 to 3521)	1361 (1168 to 1618)	48.3 (41.7 to 56.8)	-95.5 (-96.1 to -94.8)	-96 (-96.5 to -95.4)	-95.6 (-96.3 to -94.6)	-96.6 (-97.2 to -96)
		Female	744835 (680450 to 816786)	57771.4 (52828.1 to 63327.1)	11355 (10384 to 12484)	1047.8 (957.8 to 1153.1)	24208 (20476 to 28150)	1674.7 (1431 to 1924.9)	311 (254 to 375)	22.4 (18.4 to 26.8)	-96.7 (-97.3 to -96.2)	-97.1 (-97.5 to -96.6)	-97.3 (-97.8 to -96.7)	-97.9 (-98.3 to -97.4)

Province	Cause	Sex	1990				2019				1990-2019 percent change (%)			
			DALYs		Death		DALYs		Death		DALYs		Death	
			Number	Rate	Number	Rate	Number	Rate	Number	Rate	Number	Rate	Number	Rate
		Male	1220398 (1115020 to 1337344)	97488.8 (89114.8 to 106903.8)	19365 (17680 to 21268)	1831.4 (1669.9 to 2012.9)	64389 (55990 to 74604)	4531.4 (3962.7 to 5221.3)	1050 (876 to 1293)	73.8 (62 to 90.2)	-94.7 (-95.5 to -93.7)	-95.4 (-96 to -94.5)	-94.6 (-95.6 to -93.2)	-96 (-96.7 to -95)
	Transport injuries	Both	76513 (53026 to 94145)	3300.3 (2416.6 to 4063.6)	1277 (905 to 1589)	64.2 (46.8 to 79.8)	38688 (33169 to 46743)	1429.7 (1230.6 to 1703.6)	803 (666 to 1002)	28.5 (23.9 to 35.2)	-49.4 (-60.7 to -25.3)	-56.7 (-66.3 to -36.2)	-37.1 (-52.9 to -4.2)	-55.6 (-66.4 to -33.9)
		Female	21364 (14315 to 27742)	1804 (1274 to 2299.9)	331 (228 to 425)	32.5 (23.5 to 42.3)	8202 (6856 to 9611)	595.9 (500.2 to 694.5)	174 (138 to 208)	12.1 (9.8 to 14.4)	-61.6 (-71.8 to -41.1)	-67 (-75.3 to -51.4)	-47.5 (-62.4 to -18.1)	-62.7 (-73.3 to -45)
		Male	55149 (38662 to 69744)	4801.6 (3428.3 to 6166.1)	946 (656 to 1227)	97.2 (67.3 to 127.6)	30486 (25325 to 38250)	2244.6 (1882.4 to 2775.3)	630 (505 to 827)	44.6 (36.2 to 57.6)	-44.7 (-59.4 to -13.6)	-53.3 (-65.9 to -28.1)	-33.5 (-53.4 to 9.5)	-54.1 (-68 to -25.6)
	Road injuries	Both	74716 (51851 to 91906)	3217.7 (2363.3 to 3943.1)	1248 (888 to 1546)	62.7 (45.7 to 77.9)	37438 (31973 to 45288)	1385.4 (1191.4 to 1648.9)	780 (647 to 972)	27.7 (23.2 to 33.9)	-49.9 (-61.2 to -25.8)	-56.9 (-66.6 to -36.9)	-37.5 (-53.2 to -4.8)	-55.8 (-66.7 to -34.5)
		Female	20981 (14064 to 27259)	1770.4 (1251.7 to 2247.6)	325 (224 to 419)	32 (23.2 to 41.7)	7923 (6601 to 9295)	575.8 (483.5 to 671)	169 (135 to 203)	11.8 (9.5 to 14)	-62.2 (-72.2 to -41.8)	-67.5 (-75.7 to -52.2)	-48.1 (-62.8 to -18.7)	-63.2 (-73.7 to -45.6)
		Male	53735 (37686 to 68077)	4669.8 (3361.4 to 6012.6)	922 (644 to 1199)	94.7 (65.9 to 123.5)	29515 (24527 to 37073)	2176.5 (1821.4 to 2694.2)	611 (489 to 801)	43.3 (35.1 to 55.9)	-45.1 (-59.7 to -13.9)	-53.4 (-66.1 to -28.3)	-33.8 (-53.5 to 8.5)	-54.2 (-68 to -26.2)
	Other transport injuries	Both	1797 (1124 to 2623)	82.7 (53 to 122.2)	29 (17 to 45)	1.5 (0.9 to 2.4)	1250 (1041 to 1595)	44.3 (36.7 to 56.2)	24 (19 to 31)	0.8 (0.7 to 1.1)	-30.4 (-55.4 to 17.9)	-46.4 (-65.7 to -10)	-19.6 (-53.1 to 56.6)	-47.3 (-69.1 to -0.3)
		Female	382 (242 to 558)	33.6 (22.1 to 48.5)	5 (3 to 8)	0.5 (0.3 to 0.8)	279 (221 to 348)	20.1 (16 to 24.9)	5 (4 to 6)	0.3 (0.3 to 0.4)	-27 (-53.4 to 17)	-40.1 (-61 to -5.3)	-10.2 (-46.7 to 65.5)	-36.3 (-62.2 to 17.5)
		Male	1415 (803 to 2264)	131.8 (77 to 209.8)	24 (13 to 40)	2.6 (1.4 to 4.3)	971 (774 to 1313)	68 (54.1 to 91.3)	19 (15 to 27)	1.3 (1 to 1.8)	-31.4 (-60.8 to 31.6)	-48.4 (-70.3 to -2.1)	-21.6 (-59 to 62.8)	-50.4 (-73.4 to 7.1)
	Unintentional injuries	Both	1864726 (1695773 to 2050212)	73156.8 (66525.3 to 80412.4)	29172 (26554 to 32067)	1353.5 (1231.3 to 1488.1)	35168 (29910 to 41358)	1187.3 (1014.8 to 1386.1)	357 (303 to 427)	12.9 (11 to 15.4)	-98.1 (-98.4 to -97.7)	-98.4 (-98.6 to -98.1)	-98.8 (-99 to -98.5)	-99 (-99.2 to -98.8)
		Female	716303 (651057 to 787315)	55272.9 (50267.7 to 60744.2)	10959 (9980 to 12042)	1009 (918.3 to 1110)	11814 (9584 to 14674)	785.5 (642.6 to 958.3)	93 (71 to 123)	7 (5.4 to 9.3)	-98.4 (-98.7 to -98)	-98.6 (-98.8 to -98.3)	-99.2 (-99.4 to -98.9)	-99.3 (-99.5 to -99.1)
		Male	1148423 (1045167 to 1263047)	91149.9 (82967.6 to 100195.7)	18213 (16567 to 20027)	1715.1 (1558.1 to 1886.2)	23354 (19896 to 27214)	1582.4 (1350.4 to 1837.7)	264 (217 to 326)	18.7 (15.5 to 23)	-98 (-98.3 to -97.6)	-98.3 (-98.5 to -97.9)	-98.6 (-98.8 to -98.2)	-98.9 (-99.1 to -98.6)
	Falls	Both	5873 (4505 to 7717)	306.5 (237.4 to 394.4)	62 (44 to 95)	4.3 (3.1 to 6.9)	7362 (5741 to 9314)	246.1 (192.1 to 309.8)	90 (70 to 125)	3.2 (2.5 to 4.5)	25.4 (1.8 to 53)	-19.7 (-31.8 to -5.5)	44 (6.8 to 105.8)	-25.2 (-42.8 to 4.5)
		Female	1409 (1039 to 1957)	153.1 (116.4 to 208.1)	13 (9 to 24)	2.2 (1.4 to 4.9)	1794 (1333 to 2403)	124.1 (93.2 to 164.7)	21 (15 to 39)	1.6 (1.2 to 3.1)	27.3 (-2.5 to 54.6)	-18.9 (-32.3 to -7)	61.2 (16.4 to 129)	-26.2 (-45.3 to 12.5)
		Male	4463 (3355 to 5927)	460.5 (352.1 to 599.4)	49 (34 to 75)	6.6 (4.7 to 9.7)	5567 (4360 to 7061)	367.4 (289 to 468.7)	69 (52 to 102)	4.8 (3.7 to 7.2)	24.7 (-0.9 to 58.2)	-20.2 (-33.9 to -1.8)	39.6 (-2.3 to 113.9)	-26.7 (-45.9 to 5.7)
	Drowning	Both	12830 (6211 to 17973)	438.6 (215.5 to 604.7)	174 (85 to 241)	6.4 (3.2 to 8.8)	3306 (1567 to 4052)	142 (69 to 175.6)	59 (28 to 73)	2.4 (1.1 to 2.9)	-74.2 (-82.2 to -62.1)	-67.6 (-77.4 to -51.9)	-65.8 (-76.4 to -49.4)	-63 (-73.7 to -45.9)
		Female	3910 (2364 to 5818)	260.2 (161.1 to 381.5)	49 (30 to 72)	3.5 (2.2 to 4.9)	477 (394 to 578)	43.9 (35.3 to 54)	9 (7 to 10)	0.7 (0.6 to 0.9)	-87.8 (-92.3 to -79.1)	-83.1 (-89.4 to -71.3)	-82.7 (-88.9 to -69.8)	-79.4 (-86.4 to -65.5)
		Male	8920 (2887 to 13131)	615 (202.4 to 895.5)	125 (41 to 182)	9.4 (3.2 to 13.6)	2829 (1083 to 3546)	236.8 (90.3 to 300.8)	51 (20 to 64)	4 (1.5 to 5)	-68.3 (-79 to -49.7)	-61.5 (-74.3 to -38.6)	-59.1 (-73.1 to -34.8)	-57.4 (-71.4 to -33.2)

Province	Cause	Sex	1990				2019				1990-2019 percent change (%)			
			DALYs		Death		DALYs		Death		DALYs		Death	
			Number	Rate	Number	Rate	Number	Rate	Number	Rate	Number	Rate	Number	Rate
	Fire, heat, and hot substances	Both	5999 (3759 to 8675)	250.7 (165.4 to 349.4)	92 (58 to 131)	4.8 (3.3 to 6.4)	2514 (1975 to 3380)	91.7 (72.1 to 124)	57 (43 to 82)	2 (1.5 to 3)	-58.1 (-75.1 to -25.5)	-63.4 (-77.8 to -37.3)	-37.8 (-64.7 to 14.6)	-57.7 (-74.5 to -31.4)
		Female	2610 (1036 to 4439)	222.6 (103.5 to 361.3)	40 (17 to 66)	4.3 (2.5 to 6.5)	1073 (728 to 1860)	80.8 (54.3 to 142.1)	25 (15 to 48)	1.8 (1.1 to 3.5)	-58.9 (-82.1 to 40)	-63.7 (-83.4 to 6.8)	-37 (-75.3 to 109)	-56.8 (-81 to 2.5)
		Male	3389 (1856 to 4758)	277.8 (162 to 376.1)	52 (29 to 72)	5.3 (3.1 to 7.2)	1440 (1068 to 1963)	102.4 (76.5 to 141.4)	32 (24 to 46)	2.2 (1.7 to 3.3)	-57.5 (-72.3 to -6.9)	-63.1 (-75.2 to -23)	-38.4 (-60.4 to 32.6)	-58.2 (-72.6 to -16.5)
	Poisonings	Both	2079 (1375 to 3145)	79.7 (54.1 to 120.4)	30 (20 to 45)	1.3 (0.9 to 1.9)	791 (627 to 1214)	29.1 (23.2 to 45.4)	16 (12 to 25)	0.6 (0.4 to 0.9)	-61.9 (-74.5 to -39.5)	-63.5 (-74.9 to -44.8)	-46.8 (-64 to -16.4)	-57.8 (-71.2 to -37.5)
		Female	748 (441 to 1149)	55.3 (34.2 to 82.5)	10 (6 to 15)	0.8 (0.5 to 1.2)	227 (179 to 297)	17.7 (14 to 23.3)	4 (3 to 6)	0.3 (0.2 to 0.4)	-69.7 (-81.9 to -46)	-67.9 (-80.2 to -44.8)	-56.8 (-73.2 to -21.2)	-62 (-75.1 to -36.4)
		Male	1331 (814 to 2231)	103.8 (64.9 to 171.9)	20 (12 to 34)	1.8 (1.1 to 3)	564 (417 to 943)	40.2 (29.6 to 69.3)	11 (8 to 19)	0.8 (0.6 to 1.3)	-57.6 (-74.2 to -28.1)	-61.3 (-76 to -36.6)	-41.8 (-64.6 to -2)	-56.1 (-72.9 to -28.8)
	Exposure to mechanical forces	Both	4963 (3481 to 6888)	233.3 (166.9 to 320.3)	63 (41 to 99)	3.3 (2.2 to 5.1)	4523 (3705 to 5648)	156.8 (129.5 to 193.5)	58 (47 to 75)	2 (1.6 to 2.6)	-8.9 (-35.5 to 28.1)	-32.8 (-50.4 to -8.2)	-8.5 (-39.4 to 48.9)	-38.7 (-59.5 to -3)
		Female	1163 (826 to 1515)	108.2 (81.1 to 139.1)	12 (7 to 17)	1.3 (0.9 to 1.9)	1052 (804 to 1377)	74.2 (57.4 to 96.3)	11 (8 to 13)	0.8 (0.6 to 1)	-9.6 (-34.5 to 23)	-31.4 (-46.9 to -10.4)	-12.4 (-42.9 to 45)	-40.6 (-61.6 to -8.3)
		Male	3800 (2524 to 5708)	358.9 (243.1 to 528.7)	51 (31 to 86)	5.3 (3.2 to 8.8)	3472 (2808 to 4386)	237.9 (193.5 to 298.4)	47 (37 to 64)	3.2 (2.5 to 4.3)	-8.6 (-37.8 to 36.9)	-33.7 (-53.5 to -3)	-7.5 (-43.5 to 63.7)	-38.9 (-63 to 4.6)
	Adverse effects of medical treatment	Both	2782 (1536 to 4093)	130 (75.2 to 197.7)	53 (30 to 81)	3.3 (1.9 to 5.1)	1262 (963 to 1506)	44.5 (34.4 to 52.7)	38 (31 to 46)	1.3 (1.1 to 1.6)	-54.6 (-70.5 to -24.3)	-65.8 (-77.7 to -44.4)	-27.6 (-57.2 to 22.9)	-59.7 (-76.4 to -34.3)
		Female	1011 (556 to 1467)	94.4 (53.9 to 139.7)	19 (11 to 29)	2.4 (1.4 to 4.2)	482 (353 to 600)	34.5 (25.7 to 42.7)	15 (11 to 19)	1.1 (0.8 to 1.4)	-52.3 (-69.2 to -22.4)	-63.4 (-76.4 to -40.5)	-19.4 (-53 to 38.3)	-55.4 (-76.6 to -23.7)
		Male	1771 (845 to 2915)	165.9 (80 to 277.5)	34 (16 to 57)	4.3 (2.2 to 7.1)	781 (548 to 997)	54.4 (38.6 to 68.2)	23 (17 to 29)	1.6 (1.2 to 2)	-55.9 (-74.4 to -14)	-67.2 (-81.2 to -36.9)	-32.2 (-63.9 to 42.3)	-62.9 (-80.2 to -27.8)
	Animal contact	Both	361 (254 to 521)	15 (10.8 to 21.2)	3 (2 to 5)	0.2 (0.1 to 0.3)	236 (172 to 329)	8.5 (6.2 to 12.1)	2 (1 to 4)	0.1 (0 to 0.1)	-34.6 (-52.2 to -13.1)	-43 (-55.1 to -28.9)	-48.4 (-65.8 to -18.1)	-59.3 (-72 to -39.6)
		Female	147 (93 to 229)	12.6 (8.4 to 19.2)	1 (0 to 2)	0.1 (0.1 to 0.2)	113 (79 to 163)	8.1 (5.7 to 11.9)	0 (0 to 2)	0 (0 to 0.1)	-23.1 (-46.2 to 7.5)	-35.3 (-50.7 to -17)	-53.6 (-75.7 to -2.9)	-60.6 (-76.6 to -31.1)
		Male	215 (145 to 307)	17.3 (12.3 to 24)	2 (1 to 3)	0.2 (0.1 to 0.3)	124 (93 to 174)	8.9 (6.8 to 12.7)	1 (1 to 2)	0.1 (0.1 to 0.2)	-42.4 (-60 to -16.7)	-48.6 (-61.8 to -29.9)	-46.1 (-66.6 to -11)	-59.7 (-74 to -36.1)
	Foreign body	Both	1759 (1271 to 2461)	68.1 (50.7 to 92.8)	18 (12 to 25)	0.8 (0.6 to 1)	871 (687 to 1122)	32.9 (26.1 to 41.9)	10 (8 to 12)	0.4 (0.3 to 0.5)	-50.5 (-65.3 to -29.8)	-51.8 (-64.9 to -34.6)	-46.2 (-64.3 to -18.9)	-51.3 (-65.7 to -31.7)
		Female	856 (568 to 1333)	66 (45.9 to 99.2)	9 (5 to 14)	0.7 (0.5 to 1.1)	341 (267 to 430)	26.1 (20.5 to 32.8)	4 (3 to 4)	0.3 (0.2 to 0.3)	-60.2 (-75.2 to -38.1)	-60.4 (-75 to -41.4)	-59.8 (-76.1 to -32.3)	-60.9 (-75.4 to -38.5)
		Male	903 (645 to 1301)	70.6 (52.1 to 98.8)	9 (6 to 13)	0.8 (0.6 to 1.2)	530 (413 to 703)	39.4 (31 to 51.6)	6 (5 to 9)	0.5 (0.4 to 0.7)	-41.3 (-59.6 to -17.9)	-44.1 (-60.3 to -24.5)	-33.3 (-57.8 to 2.7)	-44.1 (-61.4 to -19.6)
	Environmental heat and cold exposure	Both	312 (228 to 436)	15.5 (11.4 to 21.7)	2 (1 to 4)	0.1 (0.1 to 0.3)	365 (261 to 492)	12.4 (8.9 to 16.8)	2 (1 to 3)	0.1 (0.1 to 0.1)	17.1 (-4.4 to 32.8)	-20 (-33.9 to -9.4)	8.7 (-26.3 to 70.1)	-45.3 (-63 to -12.5)
		Female	141 (101 to 197)	14.2 (10.2 to 19.8)	0 (0 to 1)	0.1 (0 to 0.2)	177 (125 to 245)	12 (8.5 to 16.6)	1 (0 to 1)	0 (0 to 0.1)	25.5 (2.5 to 41.3)	-15.4 (-29.8 to -5.6)	27.1 (-26.2 to 134.1)	-35.1 (-66.5 to 31)
		Male	170 (125 to 249)	16.9 (12.5 to 25.2)	1 (1 to 3)	0.2 (0.1 to 0.4)	188 (138 to 253)	12.8 (9.4 to 17.1)	1 (1 to 2)	0.1 (0.1 to 0.2)	10.2 (-12.6 to 29.7)	-24.3 (-40.6 to -10.7)	2.2 (-33.5 to 64.2)	-51.8 (-69.2 to -22)
	Exposure to forces of nature	Both	1824499 (1656539 to 2010909)	71474.1 (64882.5 to 78734.4)	28642 (26007 to 31515)	1327.6 (1205.4 to 1460.7)	11039 (8662 to 14266)	322.1 (252.8 to 414.6)	0 (0 to 0)	0 (0 to 0)	-99.4 (-99.5 to -99.2)	-99.5 (-99.6 to -99.4)	-100 (-100 to -100)	-100 (-100 to -100)

Province	Cause	Sex	1990				2019				1990-2019 percent change (%)			
			DALYs		Death		DALYs		Death		DALYs		Death	
			Number	Rate	Number	Rate	Number	Rate	Number	Rate	Number	Rate	Number	Rate
		Female	703318 (638654 to 775302)	54196.8 (49209.2 to 59756.4)	10798 (9805 to 11881)	993.1 (901.7 to 1092.6)	5074 (3751 to 6871)	294.6 (219 to 397.5)	0 (0 to 0)	0 (0 to 0)	-99.3 (-99.5 to -99)	-99.5 (-99.6 to -99.3)	-100 (-100 to -100)	-100 (-100 to -100)
		Male	1121181 (1018757 to 1236004)	88862 (80733.4 to 97954.1)	17844 (16202 to 19633)	1678.9 (1524.4 to 1847.3)	5965 (4803 to 7472)	349.8 (282.7 to 437.1)	0 (0 to 0)	0 (0 to 0)	-99.5 (-99.6 to -99.3)	-99.6 (-99.7 to -99.5)	-100 (-100 to -100)	-100 (-100 to -100)
	Other unintentional injuries	Both	3269 (2413 to 4516)	145.4 (108.3 to 197.9)	33 (22 to 51)	1.5 (1 to 2.3)	2898 (2225 to 3786)	101.3 (78.4 to 131.5)	25 (20 to 32)	0.9 (0.7 to 1.1)	-11.3 (-38.1 to 15.9)	-30.3 (-50.6 to -11)	-23.3 (-56.3 to 25.6)	-40.5 (-66.3 to -4.6)
		Female	990 (710 to 1316)	89.6 (64.6 to 120.6)	7 (4 to 10)	0.6 (0.4 to 0.9)	1004 (688 to 1437)	69.4 (47.9 to 99.8)	4 (3 to 5)	0.3 (0.2 to 0.3)	1.4 (-27.7 to 34.4)	-22.6 (-41.2 to -2.5)	-47.2 (-68.6 to 4.7)	-56.2 (-73.5 to -16.9)
		Male	2279 (1596 to 3404)	201.2 (141.7 to 296.8)	26 (16 to 44)	2.4 (1.5 to 3.9)	1895 (1496 to 2420)	132.4 (104.8 to 168.8)	22 (16 to 29)	1.5 (1.1 to 2)	-16.9 (-48.7 to 21.1)	-34.2 (-59 to -5.7)	-17 (-57.9 to 50.5)	-37.3 (-68.4 to 12.9)
	Self-harm and interpersonal violence	Both	23995 (18916 to 28824)	1114.6 (900 to 1345.3)	271 (198 to 326)	12.6 (9.5 to 15.2)	14741 (12602 to 17373)	500.8 (430.3 to 585.2)	201 (167 to 236)	6.9 (5.7 to 8)	-38.6 (-47.8 to -23.2)	-55.1 (-61.9 to -44.2)	-26 (-41.8 to 1.9)	-45.4 (-57.1 to -25.2)
		Female	7169 (4959 to 9534)	694.6 (503.1 to 918.7)	66 (34 to 90)	6.2 (3.6 to 8.4)	4192 (3412 to 5099)	293.2 (240.4 to 349.2)	45 (36 to 55)	3.2 (2.6 to 4)	-41.5 (-53.7 to -17.8)	-57.8 (-66.6 to -43.1)	-31.8 (-53.9 to 34.7)	-47.7 (-64 to -6.3)
		Male	16825 (13648 to 19901)	1537.3 (1247.5 to 1833.5)	206 (151 to 250)	19 (14.1 to 23.4)	10549 (8776 to 12570)	704.4 (583.5 to 838.8)	156 (121 to 190)	10.4 (8.1 to 12.7)	-37.3 (-48.3 to -21.8)	-54.2 (-62.8 to -42)	-24.2 (-42.5 to 4.4)	-45.2 (-58.6 to -23.7)
	Self-harm	Both	8756 (4893 to 11478)	392.7 (233.3 to 510.3)	152 (88 to 199)	7.5 (4.6 to 9.7)	6652 (5196 to 8006)	232.8 (179.8 to 278.8)	134 (101 to 162)	4.5 (3.4 to 5.5)	-24 (-45.4 to 34.2)	-40.7 (-56.6 to -3.4)	-12 (-37.1 to 49.2)	-39.3 (-55.5 to -3.9)
		Female	3314 (1533 to 4694)	289.7 (146.8 to 406.9)	54 (26 to 77)	5.1 (2.8 to 7.2)	1612 (1275 to 2015)	120.2 (94.7 to 150.4)	31 (24 to 39)	2.2 (1.7 to 2.8)	-51.4 (-68.3 to 11.4)	-58.5 (-72.8 to -14.8)	-43.5 (-63.4 to 22.1)	-56.9 (-72 to -15.2)
		Male	5442 (2739 to 7581)	495.4 (268.4 to 674.6)	98 (52 to 134)	9.9 (5.7 to 13.6)	5041 (3483 to 6346)	342.8 (234.5 to 432)	103 (70 to 131)	6.8 (4.6 to 8.6)	-7.4 (-37 to 63.4)	-30.8 (-53.2 to 14.6)	5.3 (-28.1 to 80.4)	-30.8 (-52.6 to 10)
	Interpersonal violence	Both	3111 (2275 to 3976)	143.2 (105.8 to 182.2)	39 (25 to 53)	1.9 (1.3 to 2.6)	3917 (3304 to 4648)	139.1 (117.7 to 164.6)	50 (40 to 63)	1.7 (1.4 to 2.1)	25.9 (-0.9 to 77.2)	-2.9 (-22.8 to 30.4)	29.1 (-7.9 to 107.6)	-9.4 (-35.6 to 38.9)
		Female	1012 (749 to 1299)	90.2 (68.8 to 114.6)	9 (5 to 12)	0.8 (0.5 to 1.2)	1284 (1027 to 1583)	94.2 (75.3 to 116.2)	12 (9 to 16)	0.9 (0.6 to 1.2)	26.9 (-3.4 to 67.3)	4.4 (-18.1 to 34.3)	37.4 (-13.4 to 141.7)	6.8 (-30.6 to 79.9)
		Male	2099 (1427 to 2848)	196.3 (136.1 to 263.3)	30 (19 to 43)	3 (1.9 to 4.3)	2633 (2133 to 3243)	183.2 (147.6 to 226.9)	39 (29 to 49)	2.6 (2 to 3.3)	25.4 (-9.7 to 88)	-6.7 (-33.2 to 37.1)	26.8 (-18.9 to 112.1)	-14.1 (-44.9 to 42.5)
	Conflict and terrorism	Both	11088 (8587 to 14821)	532.5 (400.4 to 724.3)	65 (59 to 71)	2.4 (2.2 to 2.7)	3094 (2039 to 4778)	90.9 (60 to 140)	1 (1 to 1)	0 (0 to 0)	-72.1 (-77.4 to -66.9)	-82.9 (-86.1 to -79.6)	-98.5 (-98.5 to -98.5)	-98.4 (-98.4 to -98.4)
		Female	2648 (1653 to 4400)	296.8 (188.3 to 470.7)	0 (0 to 0)	0 (0 to 0)	1143 (694 to 1970)	67.3 (41.2 to 115.1)	0 (0 to 0)	0 (0 to 0)	-56.8 (-63.2 to -50.5)	-77.3 (-81.6 to -72.5)	34.9 (34.9 to 34.9)	32 (32 to 32)
		Male	8440 (6919 to 10584)	770.9 (620.2 to 980.8)	65 (59 to 71)	4.9 (4.5 to 5.4)	1951 (1306 to 2920)	114.9 (77.8 to 171.5)	1 (1 to 1)	0.1 (0.1 to 0.1)	-76.9 (-81.6 to -71.9)	-85.1 (-87.9 to -82)	-98.9 (-98.9 to -98.9)	-98.9 (-98.9 to -98.9)
	Executions and police conflict	Both	1039 (910 to 1189)	46.2 (40.1 to 52.9)	16 (14 to 18)	0.7 (0.6 to 0.8)	1079 (858 to 1451)	37.9 (30.7 to 49.5)	16 (12 to 22)	0.5 (0.4 to 0.8)	3.8 (-15.4 to 39.4)	-17.9 (-32.2 to 8.9)	1 (-22.8 to 44.2)	-25.1 (-41.7 to 4)
		Female	195 (174 to 220)	17.9 (15.7 to 20.5)	3 (2 to 3)	0.3 (0.2 to 0.3)	154 (131 to 185)	11.6 (10 to 13.6)	2 (2 to 2)	0.1 (0.1 to 0.2)	-21.1 (-27 to -13.4)	-35.3 (-39.9 to -30)	-24.1 (-25.4 to -22.2)	-41.5 (-42.5 to -40.2)
		Male	844 (734 to 983)	74.7 (64.3 to 86.8)	13 (11 to 15)	1.2 (1 to 1.4)	925 (716 to 1299)	63.5 (50.3 to 86.4)	14 (10 to 20)	0.9 (0.7 to 1.4)	9.6 (-13.9 to 53.4)	-15 (-32.1 to 18.3)	6.1 (-23 to 58.3)	-22.8 (-42.6 to 11.8)
**Golestan**	**All causes**	Both	590956 (526352 to 658919)	47371.4 (42478.5 to 52460.8)	8255 (7336 to 9208)	1104.9 (998.3 to 1224.3)	499191 (438903 to 562113)	29101.2 (25863.9 to 32522.3)	9971 (9206 to 10816)	753.4 (702.4 to 810.5)	-15.5 (-25.7 to -3.9)	-38.6 (-44.5 to -31.8)	20.8 (6 to 39.6)	-31.8 (-39.9 to -22.5)

Province	Cause	Sex	1990				2019				1990-2019 percent change (%)			
			DALYs		Death		DALYs		Death		DALYs		Death	
			Number	Rate	Number	Rate	Number	Rate	Number	Rate	Number	Rate	Number	Rate
		Female	264081 (233370 to 296127)	42701 (37955.9 to 48032.2)	3449 (3042 to 3911)	937.2 (822.2 to 1053.5)	226948 (192876 to 263909)	26066.6 (22359.7 to 29984.5)	4049 (3667 to 4502)	623.3 (571.5 to 685.4)	-14.1 (-25.4 to -0.6)	-39 (-46 to -31.5)	17.4 (-2.2 to 38.8)	-33.5 (-43.7 to -21.7)
		Male	326875 (287130 to 370692)	51830 (45342.1 to 59074)	4806 (4153 to 5558)	1276.6 (1089.4 to 1483.8)	272244 (238724 to 306648)	32229.2 (28436 to 36221.2)	5922 (5209 to 6689)	889.9 (787.2 to 1001.4)	-16.7 (-28.5 to -3.3)	-37.8 (-46.4 to -27.6)	23.2 (1.3 to 50.9)	-30.3 (-42.8 to -15)
	**Communicable, maternal, neonatal, and nutritional diseases**	Both	212744 (174854 to 261897)	10658.6 (8856.5 to 12933.6)	2411 (1972 to 2967)	142.7 (119.8 to 167.2)	45383 (39146 to 52443)	2609 (2253.3 to 3007.9)	555 (492 to 630)	38.2 (34.1 to 43.8)	-78.7 (-83.4 to -73)	-75.5 (-80.6 to -69.4)	-77 (-81.8 to -70.7)	-73.3 (-78.1 to -67.2)
		Female	96024 (77774 to 120544)	9851.7 (8082.2 to 12220.4)	1076 (871 to 1360)	128.3 (105.6 to 154.6)	20524 (17603 to 23710)	2375.7 (2042.6 to 2749.8)	231 (200 to 267)	31.9 (27.7 to 37.3)	-78.6 (-83.7 to -72.7)	-75.9 (-81.4 to -69.6)	-78.6 (-83.7 to -71.9)	-75.2 (-80.6 to -68.7)
		Male	116721 (94126 to 145051)	11418.9 (9349.6 to 13966.2)	1334 (1066 to 1657)	157.1 (129 to 187.1)	24859 (21380 to 28877)	2838.9 (2442.8 to 3297.6)	324 (282 to 375)	44.6 (38.9 to 52.1)	-78.7 (-84.1 to -72.6)	-75.1 (-81.2 to -68.4)	-75.7 (-81.5 to -68.3)	-71.6 (-77.5 to -64)
	HIV/AIDS and sexually transmitted infections	Both	430 (218 to 817)	28.4 (17.6 to 47.4)	4 (2 to 8)	0.2 (0.1 to 0.4)	777 (543 to 1097)	38.7 (26.6 to 54.9)	9 (6 to 14)	0.5 (0.3 to 0.8)	80.7 (9.5 to 220)	36.1 (-5 to 100.2)	146.9 (24.7 to 502.7)	113.6 (25.6 to 300.3)
		Female	215 (116 to 391)	30.7 (18.3 to 50.5)	2 (1 to 4)	0.2 (0.1 to 0.4)	419 (268 to 622)	42 (26.7 to 62)	5 (3 to 8)	0.6 (0.3 to 0.9)	95 (15.6 to 243.7)	37 (-6.4 to 114.3)	190.9 (26.8 to 676.6)	151.3 (26 to 419.6)
		Male	215 (105 to 426)	26.1 (15.5 to 45.2)	2 (1 to 4)	0.2 (0.1 to 0.5)	358 (264 to 492)	35.2 (25.2 to 49.4)	4 (3 to 6)	0.5 (0.3 to 0.6)	66.4 (2.1 to 196.9)	34.8 (-6.7 to 95)	111.1 (15.2 to 366.8)	82.4 (14.1 to 227.6)
	HIV/AIDS	Both	38 (21 to 53)	3.7 (2 to 5.1)	0 (0 to 1)	0 (0 to 0.1)	374 (232 to 571)	17.7 (10.8 to 27.4)	6 (4 to 10)	0.3 (0.2 to 0.5)	874 (419 to 1849.7)	379.9 (152.5 to 847.1)	1233.4 (499.2 to 4004.3)	503.7 (164.9 to 1841)
		Female	11 (6 to 15)	1.9 (1.1 to 2.6)	0 (0 to 0)	0 (0 to 0)	174 (79 to 304)	17 (7.7 to 29.8)	3 (1 to 6)	0.3 (0.1 to 0.6)	1506 (547.8 to 3554.4)	789.3 (266.7 to 1832.3)	2251.7 (629.2 to 7033.2)	1077.9 (252.9 to 3663.1)
		Male	28 (15 to 38)	5.5 (3 to 7.6)	0 (0 to 0)	0.1 (0 to 0.1)	200 (149 to 290)	18.2 (13.6 to 26.6)	3 (2 to 5)	0.3 (0.2 to 0.5)	625.1 (350.1 to 1287.8)	233.4 (108.1 to 539.4)	834.2 (416.2 to 2866.8)	293.5 (117.4 to 1303.4)
	Sexually transmitted infections excluding HIV	Both	392 (183 to 781)	24.8 (14 to 43.8)	3 (1 to 8)	0.2 (0.1 to 0.4)	404 (253 to 647)	21 (12.9 to 33.9)	3 (2 to 5)	0.2 (0.1 to 0.3)	3 (-30.2 to 59.9)	-15 (-32.9 to 3.6)	-5.7 (-42.4 to 87.6)	12.1 (-30.8 to 87.3)
		Female	204 (104 to 382)	28.7 (16.3 to 48.5)	2 (1 to 3)	0.2 (0.1 to 0.4)	245 (154 to 383)	25 (15.7 to 39.4)	2 (1 to 3)	0.3 (0.1 to 0.4)	20.1 (-20.6 to 77.9)	-13 (-30 to 5)	18.4 (-36 to 142.1)	30.8 (-28.1 to 124.4)
		Male	188 (79 to 403)	20.7 (10.1 to 39.7)	2 (1 to 4)	0.2 (0.1 to 0.4)	159 (91 to 267)	17 (9.4 to 29)	1 (0 to 2)	0.2 (0.1 to 0.3)	-15.5 (-44.5 to 37.6)	-17.7 (-39.9 to 10.9)	-27.2 (-54.8 to 43.2)	-5.9 (-44.2 to 81.6)
	Respiratory infections and tuberculosis	Both	35022 (26082 to 48342)	2018.8 (1594.7 to 2593.2)	449 (345 to 592)	39.8 (33.1 to 49.1)	9241 (7979 to 10966)	549.5 (477.4 to 651.9)	225 (198 to 274)	17.6 (15.3 to 21.9)	-73.6 (-81.4 to -63.7)	-72.8 (-79.6 to -64.9)	-50 (-63.5 to -32.2)	-55.8 (-63.6 to -45.9)
		Female	16277 (11482 to 24066)	1850.5 (1389.3 to 2579.2)	203 (146 to 289)	34 (26.5 to 45.8)	3871 (3242 to 4753)	457.7 (383.8 to 565.2)	88 (74 to 119)	13.9 (11.5 to 19)	-76.2 (-84.6 to -64.6)	-75.3 (-82.9 to -65.7)	-56.4 (-70.4 to -36.1)	-59.2 (-68.9 to -45.1)
		Male	18745 (13195 to 26237)	2186.2 (1661.1 to 2861.9)	246 (182 to 332)	46.4 (37.5 to 58.4)	5370 (4585 to 6393)	644.2 (553.3 to 773.4)	136 (116 to 170)	21.4 (17.9 to 27.7)	-71.4 (-80.5 to -59)	-70.5 (-78.9 to -60.8)	-44.6 (-61.6 to -22.6)	-53.9 (-64 to -40.1)
	Tuberculosis	Both	4777 (3695 to 5894)	423.8 (328.3 to 507.1)	91 (69 to 110)	12.9 (9.6 to 15.5)	1577 (1375 to 1822)	91.8 (80.5 to 106.1)	48 (41 to 56)	3.4 (2.9 to 4)	-67 (-74.3 to -52.9)	-78.3 (-82.8 to -68.9)	-47.5 (-58.8 to -23.2)	-73.8 (-79.4 to -60.9)
		Female	2045 (1120 to 2753)	345.5 (183.8 to 446.1)	36 (18 to 47)	9.9 (4.7 to 12.9)	572 (478 to 747)	66.5 (55.4 to 87.1)	17 (14 to 23)	2.4 (1.9 to 3.2)	-72 (-80.8 to -37.1)	-80.8 (-86.2 to -53.9)	-52.7 (-66.9 to 21.8)	-75.8 (-83.3 to -33.7)

Province	Cause	Sex	1990				2019				1990-2019 percent change (%)			
			DALYs		Death		DALYs		Death		DALYs		Death	
			Number	Rate	Number	Rate	Number	Rate	Number	Rate	Number	Rate	Number	Rate
		Male	2732 (2161 to 3377)	500.1 (397.2 to 618.4)	55 (43 to 68)	16.2 (12.4 to 20.3)	1005 (841 to 1190)	118 (99 to 139.1)	31 (25 to 37)	4.4 (3.6 to 5.3)	-63.2 (-72.6 to -51)	-76.4 (-82.1 to -68.9)	-44.1 (-58.3 to -24.5)	-72.7 (-79.7 to -61.8)
	Lower respiratory infections	Both	28479 (20268 to 41131)	1478 (1100.8 to 2027)	358 (265 to 498)	26.9 (21.6 to 35.1)	5397 (4681 to 6517)	342.3 (299.2 to 420.3)	177 (155 to 225)	14.1 (12.2 to 18.6)	-81 (-87.9 to -71.3)	-76.8 (-84.3 to -67.7)	-50.7 (-66.2 to -29.9)	-47.3 (-58.7 to -34)
		Female	13392 (8864 to 21045)	1392.6 (967.8 to 2086.6)	167 (115 to 251)	24.1 (18.3 to 34.7)	2227 (1840 to 2986)	281 (232.5 to 378.7)	71 (59 to 102)	11.5 (9.3 to 16.7)	-83.4 (-90.3 to -72.4)	-79.8 (-87.6 to -68.7)	-57.3 (-73.1 to -32.8)	-52.5 (-66.3 to -35.6)
		Male	15086 (9930 to 22412)	1564.5 (1091.1 to 2200.4)	191 (132 to 272)	30.2 (23.8 to 40.8)	3170 (2677 to 3859)	405.7 (343.5 to 503)	105 (88 to 136)	16.9 (14 to 23)	-79 (-87 to -66.5)	-74.1 (-83 to -61.7)	-45 (-63.9 to -17.3)	-43.9 (-57.2 to -25.5)
	Upper respiratory infections	Both	1267 (746 to 2039)	84.8 (50.4 to 133.8)	0 (0 to 1)	0 (0 to 0)	1687 (1031 to 2675)	85.8 (52.6 to 135.9)	1 (0 to 1)	0 (0 to 0.1)	33.1 (21.6 to 45.3)	1.1 (-5.3 to 7.2)	319.5 (-89.5 to 9887.6)	338.7 (-89.2 to 6960.7)
		Female	617 (365 to 988)	83 (49.5 to 130.9)	0 (0 to 0)	0 (0 to 0)	812 (494 to 1292)	83.3 (51 to 133.9)	0 (0 to 0)	0 (0 to 0.1)	31.6 (19.7 to 45.9)	0.4 (-6.8 to 8.4)	246.2 (-92.8 to 12325.2)	298.1 (-91.9 to 8232.8)
		Male	650 (383 to 1043)	86.7 (51.2 to 136.7)	0 (0 to 0)	0 (0 to 0)	875 (526 to 1388)	88.2 (53.1 to 141.2)	0 (0 to 1)	0.1 (0 to 0.1)	34.6 (20.4 to 49.7)	1.8 (-6.9 to 10.9)	359.9 (-87.6 to 10275.2)	360.8 (-88 to 7196.3)
	Otitis media	Both	499 (284 to 814)	32.2 (18.3 to 53)	0 (0 to 0)	0 (0 to 0)	581 (330 to 960)	29.6 (17.1 to 49.2)	0 (0 to 0)	0 (0 to 0)	16.4 (5.8 to 29.7)	-7.8 (-13 to -1.6)	-61.2 (-86.6 to 15.3)	-70 (-87.4 to -25.2)
		Female	223 (126 to 360)	29.4 (16.5 to 48.8)	0 (0 to 0)	0 (0 to 0)	261 (148 to 428)	26.9 (15.5 to 44.2)	0 (0 to 0)	0 (0 to 0)	17.2 (4.1 to 32.5)	-8.5 (-16.7 to 0.3)	18.8 (-69.3 to 299.7)	-51.9 (-84.3 to 61.3)
		Male	276 (156 to 456)	34.9 (20.1 to 57.7)	0 (0 to 0)	0 (0 to 0)	320 (180 to 539)	32.3 (18.5 to 54)	0 (0 to 0)	0 (0 to 0)	15.7 (3.4 to 31)	-7.3 (-15.2 to 1.5)	-76.7 (-93.6 to -6)	-77.6 (-92.5 to -29)
	Enteric infections	Both	14915 (9703 to 22776)	785.7 (535.3 to 1155)	163 (102 to 250)	11.9 (7.6 to 17.3)	4965 (3752 to 6373)	278.9 (211.9 to 358.1)	30 (21 to 42)	2.1 (1.5 to 3.1)	-66.7 (-79.8 to -45)	-64.5 (-76.9 to -45.2)	-81.7 (-89.3 to -68.1)	-82.2 (-88 to -72.4)
		Female	7184 (4051 to 13236)	770.4 (458.4 to 1307.9)	79 (42 to 146)	12 (6 to 20.8)	2355 (1762 to 3091)	266.5 (198.8 to 348)	13 (8 to 22)	1.9 (1.1 to 3.3)	-67.2 (-82.6 to -37.2)	-65.4 (-80.4 to -40.2)	-83.5 (-92.3 to -65)	-84.1 (-91.3 to -68.4)
		Male	7731 (4560 to 12808)	798.1 (512.8 to 1266.7)	84 (48 to 143)	11.5 (7.2 to 18.3)	2611 (1939 to 3391)	291.3 (218.5 to 378.8)	17 (11 to 24)	2.3 (1.5 to 3.6)	-66.2 (-80.8 to -39.9)	-63.5 (-78 to -40)	-80 (-89.6 to -61.7)	-79.6 (-87.6 to -67.6)
	Diarrheal diseases	Both	13760 (8596 to 21503)	723.2 (486.2 to 1081.2)	148 (89 to 235)	11 (6.8 to 16.2)	4496 (3314 to 5889)	253.8 (188.9 to 332.2)	23 (15 to 34)	1.7 (1.2 to 2.8)	-67.3 (-80.5 to -43.8)	-64.9 (-77.8 to -44.8)	-84.4 (-91.6 to -71)	-84.1 (-89.8 to -74.2)
		Female	6567 (3460 to 12384)	703.8 (394 to 1229)	71 (33 to 136)	11 (5 to 19.8)	2162 (1570 to 2887)	245.8 (177.5 to 328.5)	10 (5 to 19)	1.6 (0.8 to 3.1)	-67.1 (-83.2 to -32.7)	-65.1 (-80.8 to -36.2)	-85.7 (-94 to -65.3)	-85.5 (-92.6 to -69.1)
		Male	7193 (4010 to 12236)	739.5 (453.5 to 1203.9)	77 (41 to 135)	10.6 (6.4 to 17.3)	2334 (1689 to 3101)	262.1 (191.5 to 347.3)	13 (7 to 19)	1.9 (1.1 to 3.1)	-67.6 (-82.3 to -40.6)	-64.6 (-79.4 to -40.1)	-83.3 (-91.9 to -66.4)	-82 (-89.7 to -70.3)
	Typhoid and paratyphoid	Both	859 (350 to 1693)	46.9 (19.4 to 90.9)	12 (5 to 23)	0.7 (0.3 to 1.3)	336 (142 to 665)	18 (7.4 to 36.1)	5 (2 to 9)	0.3 (0.1 to 0.5)	-60.9 (-72.1 to -47.6)	-61.6 (-72 to -49.2)	-58.6 (-69.8 to -45)	-62.4 (-71.8 to -51.1)
		Female	435 (172 to 871)	47.5 (19.2 to 92.9)	6 (2 to 12)	0.7 (0.3 to 1.3)	135 (56 to 269)	14.6 (6 to 29.4)	2 (1 to 4)	0.2 (0.1 to 0.4)	-68.9 (-79.2 to -55.1)	-69.2 (-79.3 to -56.2)	-66.7 (-77.5 to -52.4)	-69.6 (-79.3 to -57.3)
		Male	424 (168 to 851)	46.4 (19.1 to 90.5)	6 (2 to 11)	0.7 (0.3 to 1.3)	200 (81 to 394)	21.3 (8.6 to 41.8)	3 (1 to 6)	0.3 (0.1 to 0.6)	-52.7 (-66.1 to -36.1)	-54.2 (-66.8 to -39.3)	-50.2 (-64 to -33.1)	-55.4 (-67 to -40.7)
	Invasive Non-typhoidal Salmonella (iNTS)	Both	244 (129 to 415)	13.2 (7.1 to 22.1)	3 (2 to 6)	0.2 (0.1 to 0.4)	124 (63 to 226)	6.6 (3.3 to 11.8)	2 (1 to 3)	0.1 (0.1 to 0.2)	-49 (-68.2 to -25.8)	-49.9 (-67 to -28.8)	-39.1 (-59.6 to -14.4)	-48.2 (-61.5 to -31.3)
		Female	152 (78 to 257)	16.3 (8.4 to 27.3)	2 (1 to 3)	0.3 (0.1 to 0.4)	53 (27 to 94)	5.7 (2.9 to 10)	1 (0 to 2)	0.1 (0.1 to 0.2)	-65.2 (-79.4 to -46)	-65.2 (-78.4 to -47.9)	-56.2 (-72.3 to -34.6)	-61.7 (-73.7 to -46.1)
		Male	92 (48 to 160)	10.2 (5.4 to 17)	1 (1 to 2)	0.2 (0.1 to 0.3)	71 (35 to 128)	7.5 (3.7 to 13.3)	1 (1 to 2)	0.1 (0.1 to 0.2)	-22 (-51 to 15.6)	-26.3 (-51.4 to 6.4)	-11.8 (-40.8 to 23.8)	-28.8 (-48.3 to -2.5)

Province	Cause	Sex	1990				2019				1990-2019 percent change (%)			
			DALYs		Death		DALYs		Death		DALYs		Death	
			Number	Rate	Number	Rate	Number	Rate	Number	Rate	Number	Rate	Number	Rate
	Other intestinal infectious diseases	Both	52 (11 to 109)	2.5 (0.6 to 5.1)	1 (0 to 1)	0 (0 to 0.1)	9 (6 to 13)	0.5 (0.3 to 0.7)	0 (0 to 0)	0 (0 to 0)	-82.3 (-92.9 to -8.1)	-80.1 (-91.8 to -18.1)	-83.5 (-93.9 to -10.6)	-81 (-92.5 to -37.4)
		Female	30 (2 to 78)	2.9 (0.3 to 7.3)	0 (0 to 1)	0 (0 to 0.1)	4 (2 to 7)	0.5 (0.2 to 0.8)	0 (0 to 0)	0 (0 to 0)	-85.2 (-95.5 to 75.7)	-83.3 (-94.8 to 47.1)	-86.2 (-96.4 to 140.7)	-83.7 (-95.5 to 16.9)
		Male	22 (3 to 58)	2.1 (0.4 to 5.2)	0 (0 to 1)	0 (0 to 0.1)	5 (3 to 8)	0.5 (0.3 to 0.8)	0 (0 to 0)	0 (0 to 0)	-78.4 (-93.2 to 76.9)	-76 (-92.2 to 47.6)	-79.8 (-95 to 121.5)	-77.5 (-93.4 to 15.9)
	Neglected tropical diseases and malaria	Both	2279 (550 to 6453)	177.2 (34.5 to 481.1)	33 (3 to 101)	3.6 (0.2 to 9)	355 (253 to 493)	18.9 (13.5 to 26.2)	2 (1 to 3)	0.1 (0.1 to 0.2)	-84.4 (-95 to -36.3)	-89.4 (-96.4 to -47.7)	-93.8 (-98.1 to -29.2)	-96.4 (-98.7 to -47)
		Female	1081 (256 to 3435)	164.6 (35.9 to 512.1)	14 (1 to 52)	2.9 (0.2 to 9.1)	178 (118 to 258)	19.1 (12.6 to 27.5)	1 (0 to 1)	0.1 (0.1 to 0.1)	-83.6 (-95.1 to -31.3)	-88.4 (-96.4 to -47.4)	-93.9 (-98.6 to -15.1)	-96.2 (-99 to -39.1)
		Male	1198 (246 to 3129)	189.9 (30.8 to 465.4)	19 (1 to 49)	4.3 (0.3 to 9.3)	177 (118 to 262)	18.6 (12.5 to 27.4)	1 (1 to 2)	0.2 (0.1 to 0.2)	-85.2 (-95.1 to -30.1)	-90.2 (-96.5 to -39.9)	-93.7 (-97.9 to -17.1)	-96.5 (-98.6 to -39.4)
	Malaria	Both	1523 (22 to 5688)	131 (1.5 to 431.4)	28 (0 to 95)	3.2 (0 to 8.6)	23 (16 to 31)	1.1 (0.8 to 1.5)	0 (0 to 0)	0 (0 to 0)	-98.5 (-99.7 to 0.1)	-99.1 (-99.8 to -25.8)	-100 (-100 to -100)	-100 (-100 to -100)
		Female	712 (12 to 3038)	116.8 (1.6 to 457.8)	12 (0 to 50)	2.6 (0 to 8.8)	12 (8 to 16)	1.2 (0.8 to 1.6)	0 (0 to 0)	0 (0 to 0)	-98.4 (-99.7 to -2.4)	-99 (-99.8 to -31.4)	-100 (-100 to -100)	-100 (-100 to -100)
		Male	811 (10 to 2633)	145.4 (1.2 to 408.8)	16 (0 to 46)	3.9 (0 to 8.8)	11 (8 to 15)	1.1 (0.8 to 1.5)	0 (0 to 0)	0 (0 to 0)	-98.6 (-99.6 to 7)	-99.3 (-99.7 to -17.6)	-100 (-100 to -100)	-100 (-100 to -100)
	Leishmaniasis	Both	68 (1 to 700)	3.8 (0.1 to 36.7)	1 (0 to 9)	0.1 (0 to 0.5)	5 (1 to 39)	0.3 (0 to 2)	0 (0 to 1)	0 (0 to 0)	-92.1 (-94.7 to 71.2)	-92.7 (-95.1 to 34.2)	-93.4 (-94.4 to -88.9)	-94.7 (-95.7 to -93.3)
		Female	24 (1 to 233)	2.7 (0.1 to 24.2)	0 (0 to 3)	0 (0 to 0.3)	2 (0 to 13)	0.2 (0 to 1.3)	0 (0 to 0)	0 (0 to 0)	-90.6 (-94.9 to 106.3)	-91.3 (-95.2 to 56.7)	-93.7 (-94.7 to -88.8)	-94.9 (-96.1 to -93)
		Male	44 (0 to 481)	4.8 (0.1 to 49.3)	1 (0 to 6)	0.1 (0 to 0.7)	3 (0 to 26)	0.3 (0 to 2.6)	0 (0 to 0)	0 (0 to 0)	-93 (-94.7 to 27.3)	-93.5 (-95.1 to 11.4)	-93.3 (-94.2 to -88.6)	-94.6 (-95.7 to -92.8)
	Schistosomiasis	Both	0 (0 to 0)	0 (0 to 0)	0 (0 to 0)	0 (0 to 0)	0 (0 to 0)	0 (0 to 0)	0 (0 to 0)	0 (0 to 0)	0 (0 to 0)	0 (0 to 0)	0 (0 to 0)	0 (0 to 0)
		Female	0 (0 to 0)	0 (0 to 0)	0 (0 to 0)	0 (0 to 0)	0 (0 to 0)	0 (0 to 0)	0 (0 to 0)	0 (0 to 0)	0 (0 to 0)	0 (0 to 0)	0 (0 to 0)	0 (0 to 0)
		Male	0 (0 to 0)	0 (0 to 0)	0 (0 to 0)	0 (0 to 0)	0 (0 to 0)	0 (0 to 0)	0 (0 to 0)	0 (0 to 0)	0 (0 to 0)	0 (0 to 0)	0 (0 to 0)	0 (0 to 0)
	Cystic echinococcosis	Both	97 (50 to 157)	7.2 (4.4 to 10.3)	1 (1 to 2)	0.1 (0.1 to 0.2)	58 (40 to 80)	3 (2.1 to 4.1)	0 (0 to 1)	0 (0 to 0)	-40.4 (-66 to 18.8)	-58.6 (-73.4 to -32.7)	-62.2 (-82.7 to -14.5)	-74.3 (-86.8 to -48.5)
		Female	43 (17 to 81)	6.5 (3.5 to 10.3)	1 (0 to 1)	0.1 (0 to 0.2)	31 (20 to 44)	3.2 (2.1 to 4.4)	0 (0 to 0)	0 (0 to 0.1)	-27.6 (-64.1 to 77.5)	-51.5 (-72.5 to -9.7)	-57.5 (-87 to 62.6)	-70.5 (-90.3 to -14.6)
		Male	54 (21 to 98)	7.9 (4.1 to 12.4)	1 (0 to 1)	0.1 (0.1 to 0.2)	27 (17 to 39)	2.8 (1.8 to 4)	0 (0 to 0)	0 (0 to 0.1)	-50.5 (-76.2 to 33.4)	-64.3 (-80.6 to -27.2)	-65.5 (-88.4 to 13.2)	-76.6 (-91.7 to -36.8)
	Trachoma	Both	12 (7 to 21)	2.6 (1.4 to 4.4)	--	--	6 (3 to 10)	0.4 (0.2 to 0.8)	--	--	-53.4 (-63.9 to -39)	-83.6 (-87.2 to -78.8)	--	--
		Female	9 (5 to 15)	4 (2.2 to 6.6)	--	--	4 (2 to 7)	0.6 (0.3 to 1)	--	--	-55.5 (-67.7 to -39.8)	-85.2 (-89.2 to -80.3)	--	--
		Male	3 (2 to 6)	1.3 (0.7 to 2.3)	--	--	2 (1 to 3)	0.3 (0.1 to 0.5)	--	--	-47.9 (-64.9 to -19.1)	-79.6 (-86.8 to -69)	--	--
	Rabies	Both	7 (2 to 11)	0.6 (0.1 to 0.9)	0 (0 to 0)	0 (0 to 0)	4 (1 to 6)	0.2 (0 to 0.3)	0 (0 to 0)	0 (0 to 0)	-42 (-70.5 to 6.6)	-64 (-79.3 to -36.6)	-30.9 (-61.1 to 19.9)	-64.2 (-78.8 to -40.3)
		Female	2 (0 to 3)	0.4 (0 to 0.8)	0 (0 to 0)	0 (0 to 0)	1 (0 to 2)	0.1 (0 to 0.2)	0 (0 to 0)	0 (0 to 0)	-35.1 (-73.4 to 40.5)	-71.1 (-85.4 to -40)	-33.6 (-66.7 to 40.3)	-73.7 (-87.1 to -44.7)

Province	Cause	Sex	1990				2019				1990-2019 percent change (%)			
			DALYs		Death		DALYs		Death		DALYs		Death	
			Number	Rate	Number	Rate	Number	Rate	Number	Rate	Number	Rate	Number	Rate
		Male	5 (1 to 10)	0.7 (0.2 to 1.3)	0 (0 to 0)	0 (0 to 0)	3 (1 to 4)	0.3 (0.1 to 0.4)	0 (0 to 0)	0 (0 to 0)	-44.6 (-75 to 17.9)	-60.2 (-80.2 to -20.7)	-29.5 (-64.5 to 39.1)	-56.8 (-78 to -21.1)
	Intestinal nematode infections	Both	110 (48 to 198)	6.3 (3.2 to 10.8)	1 (0 to 2)	0 (0 to 0.1)	21 (12 to 36)	1.1 (0.6 to 1.9)	0 (0 to 0)	0 (0 to 0)	-80.5 (-91 to -55.1)	-81.9 (-90.9 to -64.3)	-92.2 (-97.4 to -55.4)	-90 (-96.3 to -62.3)
		Female	60 (23 to 121)	7.4 (3.5 to 13.5)	0 (0 to 1)	0 (0 to 0.1)	13 (7 to 23)	1.4 (0.7 to 2.5)	0 (0 to 0)	0 (0 to 0)	-77.5 (-90.1 to -43.3)	-80.6 (-90.7 to -57.8)	-92.2 (-98.4 to 46.2)	-89.7 (-97.7 to -12.7)
		Male	50 (13 to 115)	5 (1.7 to 10.8)	0 (0 to 1)	0 (0 to 0.1)	8 (4 to 13)	0.8 (0.4 to 1.4)	0 (0 to 0)	0 (0 to 0)	-84 (-94.7 to -42.3)	-83.6 (-94.1 to -53.1)	-92.3 (-98.2 to 5)	-90.2 (-97.6 to -39.1)
	Food-borne trematodiases	Both	4 (1 to 9)	0.5 (0.1 to 1)	--	--	19 (4 to 39)	1 (0.2 to 2)	--	--	333.9 (218.7 to 465.6)	107.6 (58.1 to 163.9)	--	--
		Female	2 (0 to 4)	0.4 (0.1 to 0.8)	--	--	8 (2 to 16)	0.8 (0.2 to 1.6)	--	--	324.2 (216.6 to 458.4)	101.1 (49.2 to 164.1)	--	--
		Male	3 (1 to 5)	0.6 (0.1 to 1.2)	--	--	12 (2 to 24)	1.2 (0.2 to 2.4)	--	--	340.7 (166.9 to 541.6)	113.2 (41.2 to 201.1)	--	--
	Leprosy	Both	0 (0 to 1)	0.1 (0 to 0.1)	--	--	1 (0 to 1)	0 (0 to 0.1)	--	--	58.7 (45.8 to 72.2)	-36 (-40.7 to -30.9)	--	--
		Female	0 (0 to 0)	0 (0 to 0.1)	--	--	0 (0 to 0)	0 (0 to 0)	--	--	51.3 (31.4 to 75)	-40.7 (-47.8 to -32.4)	--	--
		Male	0 (0 to 0)	0.1 (0 to 0.1)	--	--	0 (0 to 1)	0.1 (0 to 0.1)	--	--	62.6 (45.1 to 81.3)	-32.5 (-39 to -25)	--	--
	Other neglected tropical diseases	Both	458 (287 to 698)	25.2 (16.4 to 36.9)	2 (1 to 3)	0.1 (0.1 to 0.2)	218 (139 to 333)	11.7 (7.5 to 17.6)	1 (1 to 2)	0.1 (0 to 0.1)	-52.4 (-67.7 to -27)	-53.7 (-67.2 to -32.4)	-15.2 (-55 to 39.1)	-33.2 (-58 to 2.7)
		Female	230 (135 to 359)	26.3 (16.3 to 39.6)	1 (0 to 1)	0.1 (0.1 to 0.2)	106 (61 to 169)	11.5 (6.7 to 18.2)	1 (0 to 1)	0.1 (0 to 0.1)	-53.8 (-71.5 to -22.8)	-56.1 (-71.7 to -29.9)	-19.1 (-56.2 to 64.5)	-38.6 (-62.3 to 16.5)
		Male	228 (121 to 383)	24 (14.1 to 38.5)	1 (0 to 2)	0.1 (0.1 to 0.2)	112 (62 to 187)	11.8 (6.6 to 19.5)	1 (0 to 1)	0.1 (0 to 0.2)	-51 (-73.6 to -9.4)	-51 (-72.1 to -15)	-12.1 (-58.7 to 84.2)	-28.5 (-57.9 to 32.3)
	Other infectious diseases	Both	20589 (11968 to 36384)	1067.1 (670.4 to 1781.6)	255 (154 to 436)	16.5 (11.2 to 25.8)	3443 (2610 to 4200)	192.1 (143.8 to 234.9)	70 (51 to 85)	4.6 (3.3 to 5.6)	-83.3 (-90.7 to -70.5)	-82 (-89.5 to -70)	-72.5 (-84.8 to -51.4)	-72.2 (-83.1 to -55.5)
		Female	10787 (6168 to 19181)	1108.6 (667.2 to 1896.6)	131 (75 to 228)	16.3 (10.4 to 26.1)	1461 (1107 to 1850)	163 (121.6 to 207.8)	27 (19 to 34)	3.5 (2.4 to 4.5)	-86.5 (-92.7 to -75.3)	-85.3 (-91.7 to -74.6)	-79.5 (-89.4 to -62)	-78.5 (-87.7 to -62.4)
		Male	9802 (5667 to 16901)	1024.4 (628.3 to 1687.4)	124 (73 to 209)	16.6 (11 to 25.7)	1982 (1475 to 2429)	222.3 (163.3 to 273.7)	43 (31 to 54)	5.7 (3.9 to 7.2)	-79.8 (-88.7 to -63.8)	-78.3 (-87.3 to -63.5)	-65.1 (-80.7 to -37.6)	-65.5 (-79.2 to -43.3)
	Meningitis	Both	4587 (3228 to 6697)	239.1 (176.3 to 335)	56 (40 to 80)	3.6 (2.8 to 4.8)	920 (787 to 1076)	50.9 (43.6 to 59.6)	18 (15 to 21)	1.1 (1 to 1.3)	-79.9 (-87 to -69.6)	-78.7 (-85.7 to -68.9)	-68.6 (-78.9 to -54.2)	-68.5 (-77 to -56.4)
		Female	2413 (1570 to 3863)	254.1 (175.6 to 393.8)	29 (19 to 46)	3.7 (2.7 to 5.4)	440 (365 to 529)	48.6 (40.5 to 58.6)	8 (6 to 9)	1 (0.8 to 1.2)	-81.8 (-89 to -70.4)	-80.9 (-88 to -70.2)	-73 (-83.8 to -57.3)	-72.8 (-82.6 to -60)
		Male	2174 (1395 to 3276)	224.4 (153.8 to 323.9)	27 (18 to 39)	3.5 (2.5 to 4.8)	480 (385 to 600)	53.2 (42.6 to 66)	10 (8 to 12)	1.3 (1 to 1.6)	-77.9 (-86.4 to -62.4)	-76.3 (-84.9 to -61.9)	-63.9 (-76.8 to -40.4)	-63.7 (-75.6 to -42.3)
	Encephalitis	Both	415 (272 to 632)	24 (17.1 to 34.1)	5 (3 to 8)	0.4 (0.3 to 0.5)	359 (249 to 445)	19.5 (13.1 to 24.3)	6 (4 to 7)	0.4 (0.2 to 0.5)	-13.6 (-47.3 to 36.5)	-18.8 (-46.8 to 23.9)	17.6 (-27.4 to 89.9)	-4.8 (-41.1 to 40.8)
		Female	206 (120 to 364)	23.7 (15.1 to 38)	2 (1 to 4)	0.4 (0.2 to 0.5)	152 (111 to 197)	16.5 (11.7 to 21.7)	2 (1 to 3)	0.3 (0.2 to 0.4)	-26.4 (-60.8 to 36.5)	-30.3 (-61 to 19.5)	-5.4 (-49.8 to 76.7)	-20.6 (-53.2 to 29.5)

Province	Cause	Sex	1990				2019				1990-2019 percent change (%)			
			DALYs		Death		DALYs		Death		DALYs		Death	
			Number	Rate	Number	Rate	Number	Rate	Number	Rate	Number	Rate	Number	Rate
		Male	209 (118 to 339)	24.4 (15.2 to 35.9)	3 (2 to 4)	0.4 (0.3 to 0.6)	207 (127 to 273)	22.5 (13.5 to 29.7)	4 (2 to 5)	0.5 (0.2 to 0.6)	-1 (-45.4 to 82)	-7.7 (-45.7 to 55.4)	38.5 (-21 to 152)	8.9 (-38.6 to 85.3)
		Both	41 (16 to 82)	2 (0.8 to 3.8)	0 (0 to 1)	0 (0 to 0)	2 (1 to 3)	0.1 (0.1 to 0.1)	0 (0 to 0)	0 (0 to 0)	-95.7 (-98.3 to -88.1)	-95.3 (-98.1 to -87.3)	-94.6 (-97.7 to -85.6)	-94.4 (-97.5 to -86.2)
	Diphtheria	Female	19 (5 to 48)	1.8 (0.5 to 4.4)	0 (0 to 1)	0 (0 to 0.1)	1 (0 to 1)	0.1 (0 to 0.1)	0 (0 to 0)	0 (0 to 0)	-96.5 (-99 to -84.2)	-96.2 (-98.9 to -84)	-95.6 (-98.6 to -81.9)	-95.4 (-98.5 to -83.2)
		Male	22 (6 to 54)	2.1 (0.6 to 4.9)	0 (0 to 1)	0 (0 to 0.1)	1 (1 to 2)	0.1 (0.1 to 0.2)	0 (0 to 0)	0 (0 to 0)	-95 (-98.5 to -80.6)	-94.5 (-98.3 to -81.1)	-93.7 (-97.8 to -76.6)	-93.6 (-97.7 to -80.2)
		Both	4400 (220 to 17740)	198.5 (10 to 799.3)	50 (2 to 204)	2.3 (0.1 to 9.2)	158 (11 to 629)	8.7 (0.6 to 34.6)	2 (0 to 7)	0.1 (0 to 0.4)	-96.4 (-99.8 to -12.7)	-95.6 (-99.7 to 6)	-96.5 (-99.9 to 8.2)	-95.7 (-99.9 to 31.1)
	Whooping cough	Female	2566 (128 to 10564)	235.4 (11.7 to 968.2)	29 (1 to 122)	2.7 (0.1 to 11.2)	89 (6 to 363)	10.1 (0.6 to 40.9)	1 (0 to 4)	0.1 (0 to 0.5)	-96.5 (-99.8 to -15.8)	-95.7 (-99.8 to 3)	-96.6 (-99.9 to 5.9)	-95.8 (-99.9 to 29.2)
		Male	1835 (95 to 7351)	162.8 (8.4 to 652.3)	21 (1 to 85)	1.9 (0.1 to 7.5)	69 (5 to 292)	7.4 (0.6 to 31.3)	1 (0 to 3)	0.1 (0 to 0.4)	-96.2 (-99.8 to -12.3)	-95.4 (-99.7 to 6.9)	-96.3 (-99.9 to 22.5)	-95.5 (-99.8 to 47.4)
		Both	511 (136 to 1120)	26 (8.4 to 52.5)	6 (2 to 13)	0.4 (0.1 to 0.7)	27 (17 to 42)	1.5 (0.9 to 2.4)	0 (0 to 1)	0 (0 to 0)	-94.8 (-98 to -75.3)	-94.2 (-97.7 to -75.1)	-92 (-97 to -65.9)	-92 (-96.6 to -70.8)
	Tetanus	Female	85 (21 to 209)	8.7 (2.7 to 20.9)	1 (0 to 2)	0.1 (0 to 0.3)	7 (6 to 9)	0.8 (0.6 to 1)	0 (0 to 0)	0 (0 to 0)	-91.6 (-96.6 to -62.5)	-91.2 (-96.4 to -66.9)	-87.8 (-94.9 to -56.7)	-89.1 (-95 to -69.1)
		Male	427 (108 to 982)	42.6 (12.7 to 93)	5 (2 to 11)	0.6 (0.2 to 1.2)	20 (11 to 33)	2.2 (1.2 to 3.7)	0 (0 to 1)	0 (0 to 0.1)	-95.4 (-98.5 to -74.5)	-94.8 (-98.2 to -72.2)	-92.9 (-97.7 to -63.9)	-92.5 (-97.3 to -68.9)
		Both	7576 (2480 to 17063)	339.3 (111 to 766.3)	88 (29 to 198)	3.9 (1.3 to 8.9)	130 (38 to 295)	7 (2.1 to 15.9)	1 (0 to 3)	0.1 (0 to 0.2)	-98.3 (-99.2 to -96.6)	-97.9 (-99.1 to -96)	-98.3 (-99.3 to -96.7)	-98 (-99.1 to -96)
	Measles	Female	3996 (1275 to 9040)	363.9 (116 to 823.4)	46 (15 to 105)	4.2 (1.3 to 9.6)	65 (19 to 157)	7.2 (2.1 to 17.3)	1 (0 to 2)	0.1 (0 to 0.2)	-98.4 (-99.3 to -96.8)	-98 (-99.2 to -96.1)	-98.4 (-99.3 to -96.8)	-98.1 (-99.2 to -96.2)
		Male	3579 (1160 to 8184)	315.5 (102.3 to 720.9)	42 (13 to 95)	3.7 (1.2 to 8.4)	64 (19 to 145)	6.8 (2 to 15.3)	1 (0 to 2)	0.1 (0 to 0.2)	-98.2 (-99.2 to -96.5)	-97.9 (-99.1 to -95.9)	-98.2 (-99.3 to -96.6)	-97.9 (-99.1 to -95.9)
		Both	246 (138 to 420)	15.5 (10.3 to 23.4)	3 (2 to 5)	0.4 (0.2 to 0.5)	96 (69 to 134)	5.5 (4 to 7.5)	1 (1 to 2)	0.1 (0.1 to 0.1)	-60.8 (-79.7 to -21.6)	-64.7 (-79.1 to -42)	-62.4 (-80.1 to -28.2)	-74.9 (-84.7 to -59.1)
	Varicella and herpes zoster	Female	127 (57 to 252)	16 (9.2 to 26.9)	2 (1 to 3)	0.4 (0.2 to 0.6)	49 (33 to 73)	5.6 (3.8 to 8.1)	1 (0 to 1)	0.1 (0.1 to 0.2)	-61.5 (-82.6 to -9)	-65.1 (-81.9 to -33.8)	-64.2 (-84.2 to -16.9)	-74.7 (-87.5 to -48.8)
		Male	119 (54 to 238)	15 (9 to 25)	2 (1 to 3)	0.4 (0.2 to 0.6)	48 (31 to 70)	5.4 (3.6 to 7.8)	1 (0 to 1)	0.1 (0.1 to 0.1)	-60 (-82.2 to -7.1)	-64.1 (-81.4 to -31.4)	-60.4 (-81.8 to -2.7)	-74.9 (-87.1 to -48.5)
		Both	2118 (1231 to 3330)	178.7 (102.5 to 280.9)	39 (22 to 63)	4.8 (2.7 to 7.9)	724 (593 to 890)	39.9 (32.6 to 49.2)	19 (15 to 24)	1.2 (0.9 to 1.5)	-65.8 (-79.2 to -34.5)	-77.6 (-87 to -57.6)	-51.1 (-72.5 to -2.7)	-74.9 (-87.1 to -51.3)
	Acute hepatitis	Female	1057 (483 to 1709)	163.1 (74.6 to 242.9)	18 (8 to 27)	4.1 (1.7 to 6.3)	208 (161 to 265)	22.7 (17.6 to 28.6)	5 (3 to 7)	0.6 (0.4 to 0.8)	-80.3 (-88.2 to -52.9)	-86.1 (-91.5 to -66.3)	-72.3 (-84.3 to -27.4)	-85.1 (-92.2 to -58.5)
		Male	1061 (596 to 1997)	191.5 (109.4 to 370.3)	22 (12 to 43)	5.4 (3 to 10.7)	516 (400 to 672)	58 (44.9 to 75.4)	14 (10 to 19)	1.8 (1.3 to 2.5)	-51.3 (-76.9 to -5.1)	-69.7 (-85.8 to -41.7)	-33.8 (-70 to 33)	-66 (-85.1 to -31.4)
		Both	694 (419 to 1476)	44.1 (28.3 to 84)	7 (3 to 16)	0.7 (0.4 to 1.4)	1028 (443 to 1426)	59 (24.8 to 81.5)	22 (7 to 32)	1.6 (0.5 to 2.2)	48.1 (-64 to 201)	33.9 (-64.4 to 151.6)	238.8 (-46.2 to 734.6)	126.9 (-54.5 to 423)
	Other unspecified infectious diseases	Female	318 (190 to 673)	41.8 (26.6 to 77)	3 (1 to 7)	0.6 (0.3 to 1.3)	450 (185 to 680)	51.5 (20.9 to 78.9)	9 (3 to 14)	1.3 (0.4 to 2)	41.4 (-65.6 to 196)	23.2 (-67.2 to 143.3)	229.6 (-48.2 to 776.4)	104 (-59 to 426.2)
		Male	376 (194 to 869)	46.2 (27.1 to 96.8)	4 (2 to 10)	0.8 (0.4 to 1.6)	578 (233 to 831)	66.7 (25.7 to 96.4)	13 (4 to 19)	1.9 (0.5 to 2.8)	53.7 (-65.9 to 250.8)	44.5 (-64.8 to 202.8)	245.5 (-47.9 to 822.6)	148.4 (-54.2 to 457.2)

Province	Cause	Sex	1990				2019				1990-2019 percent change (%)			
			DALYs		Death		DALYs		Death		DALYs		Death	
			Number	Rate	Number	Rate	Number	Rate	Number	Rate	Number	Rate	Number	Rate
	Maternal and neonatal disorders	Both	130905 (105858 to 158565)	6078.8 (4930.9 to 7341.4)	1464 (1181 to 1776)	68 (54.9 to 82.3)	23426 (19326 to 28090)	1361 (1115.8 to 1640.6)	213 (168 to 262)	12.8 (10.1 to 15.7)	-82.1 (-86.5 to -76.7)	-77.6 (-83.1 to -70.7)	-85.5 (-89.4 to -80.6)	-81.2 (-86.4 to -74.9)
		Female	56031 (44560 to 68921)	5379.9 (4290.6 to 6597.3)	627 (496 to 773)	60.3 (47.9 to 74.1)	10532 (8630 to 12671)	1241.2 (1011.8 to 1500.4)	94 (74 to 116)	11.5 (9 to 14.2)	-81.2 (-85.8 to -75)	-76.9 (-82.6 to -69.3)	-85 (-89 to -79.3)	-80.9 (-86.1 to -73.9)
		Male	74874 (59023 to 92492)	6737.3 (5314 to 8317.2)	837 (658 to 1037)	75.2 (59.1 to 93.1)	12894 (10512 to 15629)	1474 (1196.2 to 1789)	119 (92 to 148)	14 (10.8 to 17.4)	-82.8 (-87.5 to -77.1)	-78.1 (-84.2 to -70.8)	-85.8 (-90 to -80.6)	-81.4 (-86.9 to -74.6)
	Maternal disorders	Female	1189 (843 to 1630)	199.8 (139.3 to 271.5)	16 (10 to 23)	2.9 (1.8 to 4)	436 (343 to 552)	39.2 (31.1 to 49.3)	5 (4 to 7)	0.5 (0.3 to 0.6)	-63.4 (-74.6 to -43.6)	-80.4 (-86.5 to -70.7)	-67.7 (-79.7 to -43.1)	-84.2 (-90.1 to -72.2)
	Neonatal disorders	Both	129716 (104836 to 157500)	5977.7 (4836.5 to 7252.1)	1448 (1166 to 1763)	66.5 (53.6 to 81)	22990 (18871 to 27679)	1341.6 (1097.2 to 1620.6)	208 (163 to 257)	12.5 (9.9 to 15.5)	-82.3 (-86.6 to -76.8)	-77.6 (-83.2 to -70.5)	-85.7 (-89.7 to -80.8)	-81.2 (-86.4 to -74.7)
		Female	54842 (43381 to 67662)	5180.1 (4106.5 to 6384.4)	611 (481 to 756)	57.5 (45.3 to 71.1)	10097 (8165 to 12231)	1202 (972.2 to 1458.9)	89 (69 to 111)	11 (8.5 to 13.7)	-81.6 (-86.1 to -75.2)	-76.8 (-82.5 to -68.6)	-85.4 (-89.5 to -79.6)	-80.8 (-86.2 to -73.1)
		Male	74874 (59023 to 92492)	6737.3 (5314 to 8317.2)	837 (658 to 1037)	75.2 (59.1 to 93.1)	12894 (10512 to 15629)	1474 (1196.2 to 1789)	119 (92 to 148)	14 (10.8 to 17.4)	-82.8 (-87.5 to -77.1)	-78.1 (-84.2 to -70.8)	-85.8 (-90 to -80.6)	-81.4 (-86.9 to -74.6)
	Nutritional deficiencies	Both	8603 (6115 to 11856)	502.5 (363.2 to 689.4)	42 (26 to 67)	2.7 (1.8 to 3.9)	3174 (2030 to 4725)	169.9 (109.7 to 250.9)	6 (5 to 7)	0.5 (0.4 to 0.6)	-63.1 (-75.1 to -47.4)	-66.2 (-76 to -53.4)	-85.9 (-91.4 to -76.5)	-82.2 (-88.2 to -72.9)
		Female	4448 (2909 to 6533)	547.1 (368.3 to 790.8)	21 (11 to 39)	2.6 (1.5 to 4.6)	1708 (1041 to 2625)	186.2 (115.5 to 283.8)	2 (2 to 3)	0.4 (0.3 to 0.5)	-61.6 (-76.7 to -39.9)	-66 (-78 to -50.4)	-88.1 (-93.9 to -75.8)	-84.1 (-91.4 to -71.5)
		Male	4156 (2655 to 6341)	457 (305.7 to 670.1)	21 (11 to 37)	2.9 (1.7 to 4.4)	1467 (885 to 2397)	153.2 (92.7 to 248.9)	3 (3 to 4)	0.5 (0.4 to 0.7)	-64.7 (-79.2 to -41.8)	-66.5 (-79.2 to -46.4)	-83.8 (-91.1 to -67.3)	-80.7 (-88 to -66.6)
	Protein-energy malnutrition	Both	3812 (2454 to 5973)	189.6 (126.8 to 291.4)	40 (25 to 65)	2.5 (1.7 to 3.7)	666 (457 to 923)	36.3 (25.2 to 49.7)	4 (3 to 5)	0.3 (0.3 to 0.4)	-82.5 (-90.3 to -70.2)	-80.9 (-88.9 to -68.9)	-90.4 (-94.2 to -83.5)	-87.5 (-91.8 to -80.6)
		Female	1914 (1031 to 3546)	192.1 (108.5 to 345.2)	20 (10 to 39)	2.5 (1.4 to 4.5)	316 (210 to 446)	34.9 (23.8 to 48.7)	2 (1 to 2)	0.3 (0.2 to 0.4)	-83.5 (-92 to -67.5)	-81.8 (-90.9 to -65.7)	-90.9 (-95.5 to -81.5)	-87.6 (-93.3 to -77.3)
		Male	1897 (1057 to 3246)	187.5 (109.2 to 311.5)	20 (10 to 35)	2.6 (1.6 to 4)	350 (236 to 499)	37.6 (25.7 to 52.6)	2 (2 to 3)	0.3 (0.3 to 0.4)	-81.6 (-90.6 to -63)	-79.9 (-89.2 to -62.5)	-89.8 (-94.7 to -78.7)	-87.5 (-92.6 to -78.1)
	Iodine deficiency	Both	196 (120 to 291)	16.5 (10.5 to 24.1)	--	--	134 (74 to 217)	6.8 (3.9 to 11.1)	--	--	-31.5 (-51.4 to -10.4)	-58.5 (-69.6 to -47.3)	--	--
		Female	121 (73 to 180)	20.4 (12.9 to 29.7)	--	--	84 (46 to 138)	8.6 (4.7 to 14.1)	--	--	-30.2 (-52.3 to -3.9)	-57.8 (-70.1 to -44.6)	--	--
		Male	75 (46 to 114)	12.7 (7.9 to 18.6)	--	--	50 (29 to 80)	5.1 (3 to 8.2)	--	--	-33.7 (-52.6 to -12)	-59.5 (-69.9 to -48)	--	--
	Vitamin A deficiency	Both	226 (112 to 397)	10 (5 to 17.6)	--	--	14 (6 to 31)	0.8 (0.3 to 1.6)	--	--	-93.6 (-97.4 to -85.3)	-92.3 (-96.8 to -82.5)	--	--
		Female	86 (39 to 157)	7.7 (3.5 to 14.1)	--	--	6 (2 to 14)	0.7 (0.2 to 1.5)	--	--	-92.5 (-97.5 to -81.4)	-91 (-96.9 to -77.7)	--	--
		Male	140 (57 to 295)	12.2 (4.9 to 25.6)	--	--	8 (2 to 22)	0.8 (0.2 to 2.3)	--	--	-94.3 (-98.4 to -80.9)	-93.1 (-98 to -77.3)	--	--
	Dietary iron deficiency	Both	4162 (2375 to 6589)	271.7 (163.8 to 413.3)	--	--	2153 (1196 to 3511)	114.8 (65 to 187.6)	--	--	-48.3 (-66.3 to -19)	-57.7 (-71.4 to -39.1)	--	--
		Female	2251 (1211 to 3647)	316 (183.5 to 480.6)	--	--	1211 (642 to 2035)	132.5 (71.8 to 219.8)	--	--	-46.2 (-68.2 to -11.4)	-58.1 (-74.1 to -36.5)	--	--
		Male	1911 (909 to 3465)	226.1 (120.2 to 387.2)	--	--	942 (436 to 1790)	96.7 (44.8 to 181.8)	--	--	-50.7 (-75.5 to -2.6)	-57.3 (-77.6 to -21.5)	--	--

Province	Cause	Sex	1990				2019				1990-2019 percent change (%)			
			DALYs		Death		DALYs		Death		DALYs		Death	
			Number	Rate	Number	Rate	Number	Rate	Number	Rate	Number	Rate	Number	Rate
	Other nutritional deficiencies	Both	208 (139 to 305)	14.7 (10.2 to 20.3)	2 (1 to 3)	0.2 (0.1 to 0.3)	207 (142 to 289)	11.2 (8 to 15.4)	2 (2 to 2)	0.2 (0.1 to 0.2)	-0.2 (-33.1 to 42.5)	-23.3 (-43.4 to 3.3)	21.7 (-28.4 to 126.9)	-7.6 (-36.6 to 65.2)
		Female	76 (48 to 114)	10.9 (6.8 to 16.1)	0 (0 to 1)	0.1 (0 to 0.2)	90 (58 to 134)	9.6 (6.3 to 14.2)	1 (1 to 1)	0.1 (0.1 to 0.1)	18.3 (-23.1 to 77)	-12.3 (-39.6 to 28.1)	36.9 (-32.5 to 172.6)	5.2 (-48.4 to 120.9)
		Male	132 (84 to 210)	18.4 (12.3 to 27.2)	1 (1 to 2)	0.3 (0.1 to 0.4)	118 (83 to 166)	12.9 (9.4 to 17.7)	1 (1 to 2)	0.2 (0.2 to 0.3)	-10.8 (-48.1 to 44)	-29.8 (-53 to 2.2)	15.9 (-38.3 to 139.7)	-17.5 (-48.6 to 60.5)
	**Non-communicable diseases**	Both	273579 (225165 to 317765)	29407.3 (25446.2 to 33264)	4389 (3732 to 5012)	841.3 (747.4 to 934)	383352 (332572 to 438839)	22840.4 (20033.2 to 25791.8)	8241 (7592 to 8932)	650.6 (605.7 to 698.3)	40.1 (21.1 to 65.8)	-22.3 (-30.4 to -12.2)	87.8 (60.7 to 127.1)	-22.7 (-31.8 to -11)
		Female	133097 (108693 to 156376)	28245.2 (23955.6 to 32457.3)	1911 (1568 to 2250)	737.5 (629.7 to 830.7)	189075 (158913 to 223531)	21895.6 (18606.8 to 25504)	3542 (3181 to 3951)	560.8 (512.8 to 619.3)	42.1 (20.6 to 70.9)	-22.5 (-31.7 to -10.6)	85.3 (50.6 to 134.8)	-24 (-35.6 to -8.7)
		Male	140482 (112241 to 166653)	30433.7 (25677.5 to 35355.9)	2478 (1999 to 2993)	949.2 (795.2 to 1117.8)	194277 (167693 to 223069)	23905.6 (20854.8 to 27190.5)	4699 (4111 to 5344)	746.8 (658.5 to 842.7)	38.3 (13.7 to 70.8)	-21.5 (-33.7 to -7)	89.6 (51.3 to 142.9)	-21.3 (-35.8 to -1.9)
	Neoplasms	Both	21237 (17512 to 25203)	2811 (2324.3 to 3297.3)	601 (494 to 707)	109.3 (90.3 to 127.6)	45513 (40450 to 51208)	2715.4 (2428.6 to 3046.6)	1554 (1394 to 1747)	108.3 (97.3 to 121)	114.3 (74.6 to 181.1)	-3.4 (-20.4 to 24.9)	158.7 (112.3 to 236.2)	-0.9 (-17.8 to 26.9)
		Female	9302 (6927 to 11789)	2368.1 (1835.1 to 2949.2)	242 (186 to 300)	85.4 (66.2 to 104.8)	19841 (17108 to 23038)	2212.2 (1919.4 to 2547.8)	628 (540 to 728)	81.1 (70.2 to 93.1)	113.3 (60.3 to 191.7)	-6.6 (-29.1 to 24.3)	160 (95.1 to 246.4)	-5.1 (-28 to 24.7)
		Male	11935 (8922 to 15100)	3234.2 (2443 to 4041.8)	359 (269 to 450)	135.2 (102.8 to 164.6)	25672 (21623 to 30140)	3258.7 (2772.6 to 3805.8)	926 (787 to 1084)	137.3 (117.4 to 159.6)	115.1 (61.4 to 204.2)	0.8 (-22.8 to 41.4)	157.8 (95.6 to 263.4)	1.5 (-21 to 40.5)
	Lip and oral cavity cancer	Both	106 (80 to 132)	16.4 (12.4 to 20.4)	3 (3 to 4)	0.7 (0.5 to 0.8)	306 (264 to 358)	18.4 (16 to 21.3)	11 (10 to 13)	0.8 (0.7 to 0.9)	187.9 (114.3 to 317.8)	12 (-15.7 to 62.8)	215.5 (135.7 to 361.8)	15.7 (-12.6 to 65)
		Female	35 (26 to 46)	10.6 (7.7 to 13.6)	1 (1 to 1)	0.4 (0.3 to 0.5)	133 (109 to 164)	15.4 (12.7 to 18.8)	5 (4 to 6)	0.6 (0.5 to 0.8)	278.1 (168 to 472.1)	45 (2.8 to 121.9)	333.3 (202.9 to 559.6)	53.6 (8.4 to 132.3)
		Male	71 (48 to 96)	21.9 (15.1 to 29.2)	2 (2 to 3)	0.9 (0.7 to 1.2)	173 (141 to 209)	21.6 (17.8 to 26.1)	6 (5 to 8)	0.9 (0.8 to 1.1)	143.2 (62.4 to 286.5)	-1.4 (-33.4 to 55.3)	163.5 (77.4 to 316.1)	-1.5 (-32.3 to 49.8)
	Nasopharynx cancer	Both	54 (42 to 67)	6.9 (5.4 to 8.7)	1 (1 to 2)	0.2 (0.2 to 0.3)	84 (72 to 100)	4.7 (4 to 5.6)	3 (2 to 3)	0.2 (0.1 to 0.2)	57.2 (18 to 112.1)	-31.9 (-48.9 to -8)	73.5 (30.1 to 140.7)	-32.3 (-49.5 to -6.7)
		Female	22 (15 to 31)	5.6 (3.9 to 8)	1 (0 to 1)	0.2 (0.1 to 0.3)	29 (22 to 36)	3.2 (2.5 to 4)	1 (1 to 1)	0.1 (0.1 to 0.1)	32.6 (-12.4 to 115.7)	-42.7 (-63.4 to -11.3)	49.4 (-5.8 to 129.4)	-43.8 (-66.5 to -15.6)
		Male	32 (23 to 42)	8 (5.9 to 10.4)	1 (1 to 1)	0.3 (0.2 to 0.4)	55 (45 to 67)	6.2 (5 to 7.6)	2 (1 to 2)	0.2 (0.2 to 0.3)	74.3 (23.8 to 151.7)	-22.4 (-44.7 to 12.4)	89.2 (34.2 to 175.8)	-22 (-44.9 to 13.2)
	Other pharynx cancer	Both	51 (39 to 64)	8.1 (6.1 to 10)	2 (1 to 2)	0.3 (0.3 to 0.4)	112 (95 to 133)	6.7 (5.7 to 8)	4 (3 to 5)	0.3 (0.2 to 0.3)	117.5 (63.7 to 209.6)	-16.7 (-36.4 to 19.6)	136.5 (78.6 to 238.8)	-14.8 (-36 to 22.4)
		Female	24 (17 to 33)	7.7 (5.4 to 10.4)	1 (1 to 1)	0.3 (0.2 to 0.4)	48 (38 to 59)	5.5 (4.4 to 6.8)	2 (1 to 2)	0.2 (0.2 to 0.3)	96.2 (32.1 to 211.7)	-28.6 (-52.6 to 10.2)	115 (41.1 to 234.9)	-27.2 (-55 to 11.7)
		Male	27 (19 to 36)	8.3 (5.9 to 11.1)	1 (1 to 1)	0.3 (0.2 to 0.5)	64 (50 to 79)	8 (6.4 to 10)	2 (2 to 3)	0.3 (0.3 to 0.4)	136.8 (60.2 to 265.8)	-3.2 (-33.4 to 48.4)	154.6 (73.7 to 293.1)	-0.7 (-30.8 to 50.7)
	Esophageal cancer	Both	2087 (626 to 2764)	355.6 (107 to 477.8)	76 (23 to 102)	15.8 (4.6 to 21.9)	3883 (1582 to 4771)	245.8 (99.9 to 302.1)	153 (62 to 188)	11.2 (4.5 to 13.6)	86 (38.7 to 182.3)	-30.9 (-48.6 to 3.6)	100.3 (47.8 to 199.9)	-29 (-48.8 to 3.2)

Province	Cause	Sex	1990				2019				1990-2019 percent change (%)			
			DALYs		Death		DALYs		Death		DALYs		Death	
			Number	Rate	Number	Rate	Number	Rate	Number	Rate	Number	Rate	Number	Rate
		Female	865 (256 to 1243)	309.8 (91.7 to 465.4)	32 (9 to 48)	13.8 (4 to 22.4)	1380 (584 to 1766)	169.5 (70.9 to 213.3)	54 (22 to 68)	7.7 (3.1 to 9.7)	59.6 (7 to 165.1)	-45.3 (-64.3 to -10.8)	70.2 (11.4 to 174.7)	-44.4 (-66.5 to -9.6)
		Male	1222 (327 to 1725)	397.2 (107 to 556.4)	45 (12 to 63)	17.7 (4.7 to 24.3)	2502 (905 to 3271)	327.4 (117.5 to 426.3)	99 (36 to 128)	14.9 (5.4 to 19.3)	104.7 (43.6 to 225.9)	-17.6 (-42.2 to 28.6)	121.7 (57.4 to 251.3)	-15.5 (-39.6 to 30.6)
	Stomach cancer	Both	3565 (2772 to 4415)	593.3 (471.8 to 724.8)	126 (100 to 155)	26.1 (21.1 to 31.9)	6741 (5814 to 7796)	422.5 (366.3 to 484.8)	260 (227 to 298)	18.9 (16.5 to 21.5)	89.1 (45 to 162.2)	-28.8 (-44.9 to -4.9)	106.1 (59.7 to 176.2)	-27.6 (-43.1 to -5.7)
		Female	1293 (943 to 1691)	430.3 (316.3 to 562.2)	43 (32 to 57)	18.4 (13.8 to 25)	2306 (1886 to 2771)	273.1 (226.6 to 325.3)	85 (71 to 101)	11.8 (9.8 to 13.9)	78.3 (23.1 to 162)	-36.5 (-55.6 to -9.1)	95.8 (36.3 to 180.5)	-35.8 (-56.7 to -9.2)
		Male	2272 (1600 to 3035)	746.2 (531.6 to 971.2)	83 (59 to 108)	34 (24.8 to 42.9)	4435 (3628 to 5375)	583.2 (479 to 703.9)	175 (144 to 210)	26.4 (21.9 to 31.8)	95.2 (36.4 to 192.6)	-21.9 (-44.2 to 16.8)	111.4 (50.4 to 220)	-22.2 (-42.6 to 16.5)
	Colon and rectum cancer	Both	1035 (784 to 1343)	163.6 (125.1 to 209.5)	34 (26 to 44)	6.8 (5.2 to 8.6)	3969 (3429 to 4607)	240.5 (208.9 to 279.6)	144 (125 to 167)	10.2 (8.9 to 11.8)	283.3 (180.3 to 456.7)	47 (8.9 to 110.5)	322.7 (211.1 to 506.8)	50.8 (11.5 to 112.2)
		Female	439 (308 to 631)	140.7 (99.2 to 193.7)	14 (10 to 19)	5.7 (4.1 to 7.6)	1710 (1405 to 2071)	198.5 (163.6 to 239.1)	61 (50 to 73)	8.2 (6.8 to 9.8)	289.6 (164.1 to 521.8)	41.1 (-2.5 to 121.3)	331.8 (197.4 to 578.7)	44.3 (1.2 to 124.4)
		Male	596 (415 to 819)	185.1 (132.3 to 250.7)	20 (14 to 27)	7.9 (5.7 to 10.5)	2258 (1842 to 2759)	285.9 (235 to 346.2)	83 (68 to 101)	12.3 (10.2 to 15)	278.6 (148.6 to 511.3)	54.5 (4 to 141.8)	316.4 (180 to 547.1)	56.8 (7 to 141.8)
	Liver cancer	Both	893 (674 to 1115)	139 (104.7 to 175.7)	30 (22 to 38)	6 (4.4 to 7.6)	1951 (1601 to 2352)	119.5 (98.9 to 143.5)	71 (59 to 86)	5 (4.2 to 6)	118.5 (56.9 to 211.3)	-14.1 (-38.6 to 23.9)	139.4 (69.7 to 249.8)	-16.1 (-39.9 to 21.9)
		Female	325 (215 to 455)	101.2 (66.5 to 150.5)	10 (7 to 15)	4.3 (2.9 to 6.6)	644 (508 to 808)	75.4 (59.9 to 94.1)	23 (18 to 28)	3.1 (2.4 to 3.8)	98.5 (22.5 to 219.5)	-25.5 (-55.7 to 21.1)	119.7 (29.6 to 255.9)	-29.2 (-59.8 to 18.4)
		Male	568 (414 to 762)	174.4 (128.6 to 230.6)	19 (14 to 26)	7.7 (5.6 to 10.1)	1307 (998 to 1668)	166.2 (128.1 to 212.1)	49 (37 to 62)	7.1 (5.5 to 9)	129.9 (51.3 to 251.4)	-4.7 (-36.7 to 43.7)	149.8 (66.3 to 282.5)	-7.7 (-38.4 to 38.5)
	Gallbladder and biliary tract cancer	Both	155 (117 to 221)	26.9 (20.6 to 38.8)	6 (4 to 8)	1.2 (1 to 1.8)	460 (373 to 539)	29.1 (24.4 to 34.2)	18 (15 to 21)	1.3 (1.1 to 1.6)	196.5 (110.5 to 324.9)	8.2 (-21.5 to 51)	214.1 (130.4 to 333.9)	8.5 (-20.7 to 45.2)
		Female	75 (52 to 125)	27.2 (19.6 to 46.5)	3 (2 to 5)	1.2 (0.9 to 2.2)	222 (175 to 288)	27.3 (21.9 to 35.8)	9 (7 to 12)	1.2 (1 to 1.7)	197.3 (102.8 to 356.9)	0.3 (-31 to 52.1)	212.1 (114.6 to 371.3)	0.6 (-31.6 to 46.9)
		Male	81 (55 to 110)	26.7 (18.4 to 36)	3 (2 to 4)	1.2 (0.9 to 1.6)	239 (167 to 299)	31 (22.3 to 38.6)	9 (7 to 12)	1.4 (1.1 to 1.7)	195.7 (91.8 to 365.7)	16.3 (-21.3 to 78.3)	216.1 (115.4 to 388.8)	16.2 (-17.6 to 74.2)
	Pancreatic cancer	Both	283 (210 to 365)	47.1 (35.4 to 60)	10 (8 to 13)	2 (1.5 to 2.6)	1722 (1466 to 2002)	107.3 (91.6 to 124.4)	66 (56 to 76)	4.7 (4 to 5.5)	507.3 (337.8 to 775.4)	127.6 (64.2 to 225.6)	549.6 (367.2 to 836)	134.3 (69 to 233)
		Female	99 (72 to 140)	34.2 (25.1 to 48.2)	3 (3 to 5)	1.4 (1.1 to 2)	646 (498 to 809)	77.9 (60.8 to 96.4)	25 (19 to 30)	3.4 (2.7 to 4.1)	552.7 (288.6 to 902.6)	127.8 (37.8 to 248.6)	603.5 (325.9 to 982.8)	136.2 (46.1 to 254.2)
		Male	184 (122 to 261)	59.4 (40.6 to 82.7)	7 (4 to 9)	2.6 (1.8 to 3.6)	1075 (876 to 1307)	138.6 (112.8 to 168.5)	41 (34 to 51)	6.1 (4.9 to 7.4)	483 (281.4 to 828.3)	133.3 (53.5 to 267.4)	521.4 (307.6 to 878.3)	134.4 (56.6 to 260.4)
	Larynx cancer	Both	513 (390 to 646)	82.4 (63.9 to 102.6)	17 (13 to 22)	3.3 (2.6 to 4.1)	1003 (845 to 1188)	61.1 (51.7 to 71.7)	36 (31 to 43)	2.5 (2.2 to 3)	95.4 (45.1 to 177.6)	-25.9 (-44.1 to 3.8)	109.1 (57.5 to 193.5)	-23 (-40.8 to 6.4)
		Female	128 (89 to 172)	40.8 (28.8 to 53.1)	4 (3 to 5)	1.6 (1.1 to 2)	233 (188 to 285)	27.3 (22 to 33.2)	8 (7 to 10)	1.1 (0.9 to 1.3)	82.2 (26.9 to 184.5)	-33.2 (-53.4 to 3.4)	103.3 (42.5 to 216.9)	-29.1 (-49.6 to 9.3)

Province	Cause	Sex	1990				2019				1990-2019 percent change (%)			
			DALYs		Death		DALYs		Death		DALYs		Death	
			Number	Rate	Number	Rate	Number	Rate	Number	Rate	Number	Rate	Number	Rate
		Male	385 (272 to 508)	121.2 (87.4 to 159.3)	13 (9 to 18)	5 (3.7 to 6.5)	770 (614 to 949)	97 (78.1 to 118.8)	28 (23 to 34)	4.1 (3.3 to 4.9)	99.7 (39.5 to 205.7)	-20 (-42.9 to 19.5)	110.8 (49.5 to 220.1)	-19.6 (-41.8 to 19.3)
	Tracheal, bronchus, and lung cancer	Both	1733 (1313 to 2259)	278.9 (211 to 360.8)	60 (45 to 78)	11.7 (8.7 to 14.9)	5296 (4579 to 6145)	324.8 (281.5 to 374.9)	196 (170 to 226)	13.8 (12 to 15.8)	205.7 (121 to 340.8)	16.5 (-14.7 to 66.5)	226.5 (139.4 to 371.9)	18.4 (-11 to 65)
		Female	374 (269 to 546)	117.6 (86.2 to 169.5)	12 (9 to 17)	4.7 (3.5 to 6.7)	1591 (1270 to 1933)	185.5 (150.2 to 224.4)	57 (46 to 69)	7.7 (6.3 to 9.3)	325.7 (148.7 to 543.9)	57.8 (-6.8 to 136.7)	378.2 (184.7 to 613.7)	62.3 (-2.6 to 137.5)
		Male	1359 (983 to 1842)	429.1 (308.1 to 569.1)	48 (35 to 65)	18.6 (13.4 to 24.2)	3706 (3061 to 4441)	473.7 (392 to 565.6)	140 (115 to 166)	20.4 (16.9 to 24.4)	172.7 (89 to 313.2)	10.4 (-21.8 to 65.8)	189.2 (103.9 to 336.8)	9.5 (-20.5 to 61.6)
	Malignant skin melanoma	Both	138 (64 to 199)	20 (9.4 to 28.4)	4 (2 to 6)	0.9 (0.4 to 1.2)	202 (159 to 283)	11.8 (9.2 to 16)	7 (5 to 9)	0.5 (0.4 to 0.6)	45.9 (-5.4 to 172.3)	-40.8 (-61 to 5.4)	64.4 (10 to 190.6)	-42.9 (-61 to -2.3)
		Female	51 (24 to 80)	14.4 (7.5 to 20.5)	1 (1 to 2)	0.6 (0.3 to 0.8)	75 (55 to 123)	8.5 (6.3 to 13.6)	2 (2 to 4)	0.3 (0.2 to 0.5)	47.2 (-11.3 to 177.6)	-41.3 (-63.3 to -0.5)	65.9 (4.5 to 180.9)	-43.7 (-63.8 to -10.4)
		Male	87 (34 to 136)	25.6 (9.7 to 40.1)	3 (1 to 4)	1.1 (0.4 to 1.8)	126 (82 to 182)	15.4 (9.6 to 21.2)	4 (3 to 6)	0.6 (0.4 to 0.9)	45.2 (-11.5 to 195.5)	-39.9 (-62.9 to 22.2)	63.6 (3.5 to 225.2)	-43.5 (-64.7 to 16.3)
	Non-melanoma skin cancer	Both	35 (26 to 46)	6.4 (4.7 to 8.2)	1 (1 to 2)	0.3 (0.2 to 0.4)	151 (126 to 178)	9.9 (8.4 to 11.8)	7 (5 to 8)	0.5 (0.4 to 0.6)	326.4 (209.2 to 505.5)	56.1 (12.1 to 123.2)	380.4 (242.2 to 591.7)	62.8 (18.9 to 139.1)
		Female	5 (3 to 7)	1.7 (1.1 to 2.5)	0 (0 to 0)	0.1 (0.1 to 0.1)	30 (23 to 39)	3.6 (2.7 to 4.6)	1 (1 to 1)	0.2 (0.1 to 0.2)	498.9 (237.8 to 936)	110 (23.1 to 276.5)	552 (281.9 to 1080.7)	106.9 (17.1 to 288)
		Male	30 (22 to 40)	11.1 (8.1 to 14.6)	1 (1 to 2)	0.6 (0.4 to 0.8)	121 (98 to 147)	16.6 (13.5 to 20.2)	5 (4 to 7)	0.9 (0.7 to 1.1)	297.9 (176.2 to 492.1)	49.4 (5.5 to 119.1)	354.4 (217 to 576.7)	46.6 (3.9 to 115.2)
	Breast cancer	Both	1010 (700 to 1425)	142.9 (98.3 to 200.9)	27 (19 to 39)	4.5 (3 to 6.5)	3947 (3246 to 4808)	200.4 (164.1 to 243.4)	107 (87 to 131)	6 (4.9 to 7.4)	290.9 (147.2 to 495.2)	40.3 (-12.5 to 114)	289.6 (143.5 to 502.2)	35 (-17.3 to 111.5)
		Female	997 (689 to 1407)	288.9 (197.4 to 408.6)	27 (18 to 39)	8.9 (5.9 to 13)	3915 (3216 to 4777)	391.3 (320.1 to 475.6)	106 (86 to 129)	11.7 (9.5 to 14.3)	292.6 (147.1 to 499.9)	35.4 (-16.1 to 108.2)	291.4 (143.7 to 510.2)	30.9 (-20.4 to 107.7)
		Male	13 (8 to 19)	3.7 (2.3 to 5.6)	0 (0 to 1)	0.1 (0.1 to 0.2)	32 (23 to 44)	3.8 (2.7 to 5.3)	1 (1 to 1)	0.1 (0.1 to 0.2)	151 (46.5 to 338.3)	2.8 (-40.1 to 74.3)	167.3 (52.7 to 372.8)	0.7 (-40.5 to 72.5)
	Cervical cancer	Female	395 (219 to 554)	112.4 (65.9 to 153.9)	11 (6 to 15)	3.7 (2.4 to 5)	723 (494 to 904)	76 (53.1 to 94.2)	22 (15 to 27)	2.6 (1.9 to 3.2)	82.9 (26.7 to 184.4)	-32.4 (-53.1 to 2.3)	99.2 (38 to 197.2)	-30.4 (-53.2 to 0.3)
	Uterine cancer	Female	56 (38 to 81)	18 (12.3 to 27)	2 (1 to 3)	0.7 (0.5 to 1.1)	257 (137 to 334)	28.7 (16.5 to 36.8)	8 (5 to 10)	1 (0.6 to 1.3)	359 (131.4 to 654.5)	59.3 (-21 to 159.3)	359.7 (129.8 to 653.4)	51.6 (-23.6 to 146.3)
	Ovarian cancer	Female	143 (81 to 254)	40.5 (22.5 to 71.6)	4 (2 to 7)	1.3 (0.7 to 2.4)	770 (559 to 955)	82.4 (62.3 to 100.5)	23 (18 to 28)	2.8 (2.2 to 3.4)	436.8 (171.3 to 914.3)	103.5 (3 to 280.4)	489.5 (198.2 to 1005.5)	112.8 (7.6 to 298.5)
	Prostate cancer	Male	366 (262 to 495)	170.6 (123.1 to 232.3)	17 (12 to 23)	10.8 (7.7 to 14.9)	1288 (968 to 1634)	211.4 (164.3 to 267.9)	69 (54 to 87)	12.6 (10.1 to 16.2)	252.5 (147.7 to 428.9)	23.9 (-11.3 to 84.2)	304.6 (191 to 494.3)	17.3 (-14.6 to 74)
	Testicular cancer	Male	7 (5 to 9)	0.9 (0.6 to 1.2)	0 (0 to 0)	0 (0 to 0)	123 (86 to 164)	12.6 (9 to 16.5)	2 (2 to 3)	0.3 (0.2 to 0.3)	1768.9 (1033 to 2982.3)	1327.1 (799.4 to 2181.5)	2175.6 (1392.1 to 3551.1)	1359.1 (923.7 to 2167.4)

Province	Cause	Sex	1990				2019				1990-2019 percent change (%)			
			DALYs		Death		DALYs		Death		DALYs		Death	
			Number	Rate	Number	Rate	Number	Rate	Number	Rate	Number	Rate	Number	Rate
	Kidney cancer	Both	255 (185 to 351)	25.4 (18.1 to 32.7)	6 (4 to 7)	0.9 (0.6 to 1.1)	604 (513 to 708)	35.8 (30.5 to 41.9)	20 (17 to 23)	1.3 (1.1 to 1.6)	137.3 (60.7 to 245.7)	40.8 (1.3 to 109.2)	249.4 (147.3 to 426.1)	56.7 (14.1 to 154.7)
		Female	106 (75 to 161)	20.6 (15 to 27.5)	2 (2 to 3)	0.7 (0.4 to 0.9)	219 (180 to 267)	24.9 (20.5 to 30.2)	6 (5 to 8)	0.8 (0.7 to 1.1)	106.1 (28.1 to 210.6)	21.1 (-18.6 to 82.5)	195.2 (99.9 to 355.2)	25.2 (-15.6 to 120.9)
		Male	148 (96 to 219)	29.9 (19.4 to 42.4)	3 (2 to 5)	1 (0.7 to 1.4)	385 (304 to 477)	47.3 (37.3 to 58.6)	13 (10 to 16)	1.9 (1.5 to 2.3)	159.7 (55.8 to 334.9)	58.4 (0.6 to 158.6)	284 (144.4 to 537.2)	80.3 (15 to 205.2)
	Bladder cancer	Both	194 (137 to 247)	36.3 (26.3 to 47)	8 (5 to 10)	1.8 (1.3 to 2.3)	601 (516 to 706)	41.1 (35.5 to 48)	25 (22 to 30)	2 (1.7 to 2.4)	210.4 (124.6 to 367)	13.1 (-18.2 to 66.5)	235.9 (142 to 399.9)	12 (-19.8 to 64.3)
		Female	58 (40 to 78)	22.7 (16 to 30.9)	2 (2 to 3)	1.2 (0.8 to 1.7)	190 (156 to 231)	25.2 (20.6 to 30.4)	8 (7 to 10)	1.3 (1 to 1.5)	229.6 (118 to 422.8)	11.3 (-29.1 to 73)	257.6 (124.5 to 458.8)	10.7 (-33.8 to 73.4)
		Male	136 (92 to 186)	49.6 (34.4 to 65.8)	5 (4 to 7)	2.5 (1.8 to 3.3)	411 (332 to 507)	58.1 (47.4 to 71.4)	17 (14 to 21)	2.8 (2.3 to 3.5)	202.3 (101.8 to 387)	17.1 (-19.2 to 83.2)	226.6 (121.9 to 425.4)	11.1 (-22.3 to 72.6)
	Brain and central nervous system cancer	Both	1770 (1030 to 2757)	147.6 (93.6 to 209.8)	33 (21 to 48)	3.8 (2.6 to 5.1)	2664 (1809 to 3225)	142.8 (97.2 to 171.7)	68 (46 to 83)	4.1 (2.8 to 5)	50.6 (-18 to 118.5)	-3.2 (-42.1 to 36.2)	105.9 (19.9 to 191.4)	6.6 (-32.4 to 50.7)
		Female	742 (369 to 1344)	123.6 (67.8 to 206.4)	13 (7 to 23)	3.1 (2 to 4.9)	1196 (795 to 1505)	126.5 (85.3 to 158.8)	31 (20 to 38)	3.6 (2.4 to 4.5)	61.1 (-24.5 to 214.6)	2.3 (-47.4 to 86.1)	127.7 (17 to 310.2)	15.6 (-35.8 to 93.1)
		Male	1028 (510 to 1783)	170.1 (91.3 to 276)	20 (11 to 32)	4.5 (2.6 to 6.8)	1469 (817 to 1932)	159.6 (88.9 to 207.3)	37 (20 to 49)	4.6 (2.5 to 6)	42.9 (-25.7 to 135.7)	-6.2 (-46.2 to 45.3)	91 (6.6 to 195.2)	1.6 (-37.2 to 55.2)
	Thyroid cancer	Both	54 (40 to 90)	8.2 (6.2 to 13.9)	2 (1 to 3)	0.3 (0.2 to 0.6)	252 (191 to 300)	14.7 (11.6 to 17.4)	8 (6 to 9)	0.5 (0.4 to 0.6)	368.1 (136.1 to 586.9)	78.8 (-5.9 to 156.7)	363.6 (154 to 569.7)	60.2 (-10.5 to 129.3)
		Female	30 (21 to 58)	9.7 (6.7 to 18.2)	1 (1 to 2)	0.4 (0.3 to 0.8)	131 (94 to 165)	14.9 (11.1 to 18.7)	4 (3 to 5)	0.5 (0.4 to 0.6)	330.3 (99.3 to 583)	53.4 (-26.8 to 141.4)	315.7 (102.9 to 556.1)	34.2 (-35.7 to 116.6)
		Male	23 (16 to 36)	6.8 (4.7 to 10.3)	1 (0 to 1)	0.3 (0.2 to 0.4)	121 (85 to 155)	14.5 (10.5 to 18.4)	4 (3 to 5)	0.5 (0.4 to 0.7)	417.3 (161.6 to 736.2)	114.1 (13.4 to 240.1)	425.6 (187.6 to 747)	100.5 (10.9 to 212.6)
	Mesothelioma	Both	8 (4 to 14)	1.2 (0.6 to 2)	0 (0 to 0)	0 (0 to 0.1)	33 (25 to 44)	1.8 (1.4 to 2.5)	1 (1 to 1)	0.1 (0.1 to 0.1)	307 (121 to 798.9)	52.9 (-16.7 to 234.3)	345.8 (135.3 to 865.7)	52.4 (-19.8 to 248.1)
		Female	5 (2 to 9)	1.4 (0.8 to 2.3)	0 (0 to 0)	0 (0 to 0.1)	11 (7 to 15)	1.1 (0.8 to 1.5)	0 (0 to 0)	0 (0 to 0)	115.8 (10.4 to 316.1)	-23.8 (-58 to 40.6)	121.3 (23.4 to 298.7)	-28.6 (-57.4 to 20.3)
		Male	3 (1 to 8)	0.9 (0.3 to 2.4)	0 (0 to 0)	0 (0 to 0.1)	22 (16 to 33)	2.5 (1.8 to 3.9)	1 (1 to 1)	0.1 (0.1 to 0.2)	618.7 (137.4 to 2452.2)	175.2 (-11 to 913.2)	696.6 (147.2 to 2698)	165.4 (-17.5 to 926)
	Hodgkin lymphoma	Both	125 (54 to 297)	12.8 (5.8 to 29.1)	3 (1 to 6)	0.4 (0.2 to 0.8)	215 (166 to 323)	11.4 (8.8 to 17)	5 (4 to 8)	0.3 (0.2 to 0.5)	71.6 (-1.5 to 264.1)	-11.5 (-49.4 to 82.2)	86.6 (8.6 to 283)	-18.3 (-53.1 to 60.5)
		Female	47 (19 to 116)	9.8 (4 to 24.4)	1 (0 to 3)	0.3 (0.1 to 0.7)	78 (60 to 118)	8.2 (6.2 to 12.3)	2 (1 to 3)	0.2 (0.2 to 0.3)	66.4 (-16.7 to 299.5)	-17.1 (-57.7 to 101.1)	82.5 (-5.4 to 328.9)	-26.2 (-60.5 to 77.9)
		Male	78 (32 to 207)	15.7 (6.7 to 39.1)	2 (1 to 4)	0.4 (0.2 to 1.1)	137 (97 to 236)	14.6 (10.3 to 25.1)	3 (2 to 6)	0.4 (0.3 to 0.7)	74.7 (-0.6 to 287.7)	-7 (-48.5 to 96.7)	89 (5.9 to 296.4)	-11.7 (-51.2 to 81.8)
	Non-Hodgkin lymphoma	Both	496 (366 to 649)	52.7 (39.8 to 67.1)	11 (9 to 15)	1.7 (1.3 to 2)	1193 (1030 to 1379)	66.7 (57.7 to 76.9)	34 (29 to 39)	2.1 (1.9 to 2.5)	140.4 (73.3 to 246.6)	26.4 (-4.1 to 80.4)	194.9 (124.2 to 322.1)	29.1 (0 to 86.3)
		Female	177 (110 to 272)	37.7 (23.9 to 54.9)	4 (2 to 6)	1.2 (0.7 to 1.6)	429 (357 to 526)	46.6 (38.7 to 56.8)	12 (10 to 15)	1.5 (1.2 to 1.8)	142.1 (48.9 to 335.6)	23.5 (-20.8 to 123.2)	204.1 (95.8 to 457.8)	26.3 (-12.6 to 134.1)

Province	Cause	Sex	1990				2019				1990-2019 percent change (%)			
			DALYs		Death		DALYs		Death		DALYs		Death	
			Number	Rate	Number	Rate	Number	Rate	Number	Rate	Number	Rate	Number	Rate
		Male	319 (227 to 419)	67 (48.2 to 86.4)	7 (5 to 10)	2.2 (1.6 to 2.7)	764 (627 to 904)	87.3 (72.2 to 102.6)	22 (18 to 26)	2.9 (2.4 to 3.4)	139.4 (70 to 262)	30.3 (-4.5 to 88.1)	190 (112.7 to 319.8)	32 (-2.9 to 88.1)
	Multiple myeloma	Both	118 (81 to 203)	19.4 (13.1 to 33)	4 (3 to 7)	0.8 (0.5 to 1.4)	337 (266 to 518)	20.7 (16.2 to 32.5)	12 (9 to 19)	0.8 (0.6 to 1.4)	186.1 (102.2 to 306.1)	6.7 (-23.1 to 51.2)	193 (112.4 to 313.8)	6 (-21.9 to 51.6)
		Female	49 (31 to 82)	17.4 (11 to 28.6)	2 (1 to 3)	0.7 (0.5 to 1.2)	134 (102 to 207)	15.9 (12 to 25)	5 (4 to 8)	0.6 (0.5 to 1)	173.4 (95.6 to 325.1)	-8.8 (-33.5 to 38)	169.6 (96.3 to 307.8)	-13.6 (-37.6 to 30.7)
		Male	69 (44 to 132)	21.3 (13.3 to 40.8)	2 (1 to 5)	0.9 (0.5 to 1.7)	203 (148 to 325)	25.8 (18.8 to 41.9)	7 (5 to 12)	1.1 (0.8 to 1.8)	195.2 (82.5 to 353)	21.2 (-25.3 to 87.3)	210.4 (93 to 380.8)	23.1 (-22.9 to 88.9)
	Leukemia	Both	4126 (2845 to 5700)	308.3 (227.9 to 394.5)	70 (51 to 90)	7.7 (5.9 to 9.3)	3747 (3034 to 4379)	208.1 (170.6 to 242.7)	94 (79 to 110)	6 (5.1 to 7)	-9.2 (-33.6 to 33)	-32.5 (-47.2 to -5)	35.1 (4.8 to 87.9)	-21.6 (-38.1 to 8.7)
		Female	2137 (1005 to 3071)	304.9 (187.6 to 409.2)	34 (20 to 46)	6.9 (4.9 to 8.7)	1599 (1160 to 1897)	172.3 (128.7 to 202.1)	40 (31 to 47)	4.8 (3.8 to 5.6)	-25.2 (-49.4 to 34.8)	-43.5 (-59 to -12.3)	15.9 (-16.4 to 81.9)	-31.1 (-48.9 to -2.4)
		Male	1989 (912 to 2919)	311.9 (176.5 to 425.6)	36 (20 to 49)	8.5 (5.8 to 11.2)	2148 (1526 to 2644)	244.8 (176.7 to 298.5)	55 (41 to 68)	7.3 (5.6 to 9.2)	8 (-26 to 95.3)	-21.5 (-43.1 to 23.3)	53.3 (8.4 to 150.2)	-14.5 (-38.4 to 26.2)
	Other malignant neoplasms	Both	1424 (1087 to 1869)	139.7 (105.7 to 175.9)	30 (23 to 39)	4.5 (3.2 to 5.5)	2805 (2146 to 3349)	161.1 (123.7 to 191.8)	78 (60 to 95)	5.3 (4 to 6.3)	97 (26.3 to 185.3)	15.3 (-21.1 to 73.3)	157.4 (72.5 to 288.8)	18.4 (-14.7 to 84.5)
		Female	606 (453 to 807)	113.8 (83.9 to 148.8)	12 (9 to 16)	3.4 (2.4 to 4.5)	1109 (949 to 1290)	124 (107.2 to 143)	30 (25 to 35)	3.9 (3.3 to 4.6)	83.2 (31.2 to 157.6)	8.9 (-21.7 to 60.3)	147.7 (77.3 to 268.1)	15.2 (-18.3 to 76.4)
		Male	819 (571 to 1184)	164.6 (114.4 to 218)	18 (13 to 24)	5.6 (3.8 to 7.3)	1696 (1071 to 2145)	199.6 (125.6 to 250.8)	48 (30 to 62)	6.7 (4.1 to 8.6)	107.2 (4.7 to 234.3)	21.3 (-30.1 to 97.9)	163.7 (46.3 to 339.3)	20.2 (-29.5 to 103.8)
	Other neoplasms	Both	42 (30 to 52)	5.8 (3.8 to 7.3)	1 (1 to 1)	0.2 (0.1 to 0.3)	76 (64 to 96)	4.8 (4 to 6.1)	2 (2 to 3)	0.2 (0.1 to 0.2)	81.6 (41.1 to 158.1)	-16.9 (-37.9 to 25.1)	118.6 (52.8 to 268.7)	-21.7 (-46.3 to 39.2)
		Female	18 (13 to 24)	4.7 (3 to 6.3)	0 (0 to 1)	0.2 (0.1 to 0.2)	32 (26 to 40)	3.8 (3 to 4.8)	1 (1 to 1)	0.1 (0.1 to 0.2)	75.7 (25 to 173.8)	-20.4 (-44.4 to 41.5)	120.8 (40.5 to 374.3)	-26.7 (-55.3 to 83.9)
		Male	24 (15 to 33)	6.8 (4.2 to 9.2)	1 (0 to 1)	0.3 (0.2 to 0.4)	45 (35 to 60)	5.9 (4.6 to 8.1)	1 (1 to 2)	0.2 (0.2 to 0.3)	86.1 (30.6 to 176.6)	-12.6 (-39.6 to 29.3)	117.4 (40.4 to 252.5)	-18.4 (-46.6 to 28)
	Cardiovascular diseases	Both	64875 (55546 to 75947)	11150.2 (9642.3 to 12828.1)	2258 (1919 to 2624)	546.2 (471.5 to 619.6)	111293 (101054 to 122045)	7576.9 (6895.9 to 8277.9)	4741 (4296 to 5184)	389.3 (353.4 to 423.4)	71.5 (43.6 to 107.8)	-32 (-42.4 to -18.8)	110 (77.1 to 154.2)	-28.7 (-38.4 to -16.5)
		Female	26842 (21750 to 32780)	9564.3 (7811.9 to 11259.2)	953 (776 to 1130)	482 (397.7 to 552.4)	46675 (40790 to 52844)	6399.9 (5655.3 to 7174.8)	2059 (1806 to 2326)	344.7 (301.7 to 387.8)	73.9 (37.1 to 123.1)	-33.1 (-45.9 to -16.3)	116 (74.1 to 172.5)	-28.5 (-40.8 to -12.5)
		Male	38034 (30276 to 47176)	12625 (10202 to 15389.8)	1305 (1040 to 1617)	610.7 (499.8 to 732.9)	64617 (55953 to 74315)	8819.5 (7690.3 to 10105.8)	2682 (2328 to 3075)	437.6 (380.8 to 499.8)	69.9 (31.4 to 124.3)	-30.1 (-45.4 to -9.4)	105.5 (59 to 169.9)	-28.3 (-42.5 to -8.8)
	Rheumatic heart disease	Both	1730 (1291 to 2372)	153.9 (114.6 to 215.9)	29 (21 to 42)	3.9 (2.7 to 6.4)	1325 (1076 to 1636)	71.9 (59.2 to 87)	26 (20 to 31)	1.8 (1.4 to 2.1)	-23.4 (-48.2 to 6.6)	-53.3 (-70 to -34)	-10 (-49.1 to 38.7)	-53.9 (-76.2 to -28.5)
		Female	966 (627 to 1379)	166.2 (109.7 to 241)	15 (10 to 23)	4 (2.5 to 6.9)	647 (502 to 818)	69.3 (54.3 to 86.3)	12 (9 to 16)	1.7 (1.3 to 2.2)	-33 (-57.6 to 12.1)	-58.3 (-74.4 to -31.4)	-20.1 (-57.7 to 51.9)	-57.9 (-80.2 to -21.9)
		Male	764 (491 to 1131)	141 (92 to 215.6)	13 (8 to 22)	3.8 (2.3 to 6.4)	679 (533 to 874)	74.6 (58.8 to 95.1)	14 (10 to 18)	1.9 (1.4 to 2.5)	-11.2 (-46.3 to 50.5)	-47.1 (-68.9 to -8.4)	1.7 (-47.6 to 102.2)	-49 (-75.6 to -2.1)
	Ischemic heart disease	Both	37775 (31349 to 45130)	6861.2 (5771.4 to 7998.9)	1434 (1203 to 1677)	345.6 (295.2 to 395.2)	65520 (58787 to 73053)	4432.2 (3998 to 4913.9)	2910 (2631 to 3208)	235.7 (212.9 to 258.8)	73.4 (42.3 to 118.7)	-35.4 (-46 to -20.3)	102.9 (69.2 to 150.2)	-31.8 (-41.5 to -18)

Province	Cause	Sex	1990				2019				1990-2019 percent change (%)			
			DALYs		Death		DALYs		Death		DALYs		Death	
			Number	Rate	Number	Rate	Number	Rate	Number	Rate	Number	Rate	Number	Rate
		Female	13737 (10621 to 17209)	5343.3 (4279.8 to 6400.2)	555 (444 to 666)	285 (228.3 to 333.6)	25173 (21817 to 28828)	3472.8 (3044.7 to 3946.6)	1198 (1048 to 1364)	199.5 (173.9 to 226.3)	83.2 (39.1 to 146.2)	-35 (-48.3 to -16)	116 (71.5 to 179.9)	-30 (-43.2 to -10.6)
		Male	24038 (18612 to 30091)	8312.4 (6577.7 to 10260.5)	879 (692 to 1097)	409.4 (333.4 to 494.2)	40347 (34497 to 46911)	5443 (4684.2 to 6292.3)	1711 (1472 to 1972)	274.5 (236.3 to 314.9)	67.8 (25.9 to 127.2)	-34.5 (-49.7 to -13)	94.7 (47.8 to 160.6)	-32.9 (-46.9 to -13.5)
	Stroke	Both	16568 (13670 to 19608)	2962.6 (2395.2 to 3503.7)	571 (452 to 681)	145.1 (114.7 to 171)	26041 (23188 to 28872)	1808.6 (1605.2 to 1993.8)	1063 (927 to 1177)	89.5 (77.8 to 99)	57.2 (29 to 93.3)	-39 (-49.3 to -23.2)	86.2 (51.7 to 139.9)	-38.3 (-49.4 to -21)
		Female	7902 (6326 to 9824)	2904 (2295.6 to 3509.3)	272 (209 to 334)	141.4 (108.3 to 170.8)	12546 (10807 to 14464)	1713.2 (1472 to 1965.1)	502 (426 to 588)	84.5 (71.1 to 98.2)	58.8 (20.9 to 109)	-41 (-54.8 to -21.7)	84.4 (38.2 to 152.8)	-40.2 (-54.4 to -18)
		Male	8665 (6650 to 11085)	2988.3 (2275.8 to 3814)	299 (220 to 394)	146.8 (109.8 to 187.3)	13496 (11548 to 15798)	1911.7 (1632.6 to 2222.6)	561 (473 to 656)	95.1 (79.5 to 111)	55.7 (16.1 to 110.1)	-36 (-52.2 to -13.4)	87.8 (37.6 to 162.5)	-35.2 (-52.2 to -11.4)
	Hypertensive heart disease	Both	2973 (2128 to 4769)	603.6 (437.7 to 952.3)	121 (86 to 196)	33.1 (23.5 to 51.3)	9123 (7735 to 10441)	667.2 (562.6 to 759.8)	445 (369 to 507)	38.5 (31.1 to 44)	206.9 (81.2 to 339.7)	10.5 (-33.6 to 57.3)	269.3 (111.7 to 434.7)	16.3 (-32.2 to 68.1)
		Female	1415 (941 to 2400)	604.4 (410.9 to 1024.1)	61 (41 to 107)	34 (22.8 to 60.4)	4277 (3384 to 5139)	627.2 (492.3 to 752.4)	218 (167 to 262)	38.1 (28.8 to 45.7)	202.2 (64.1 to 376.7)	3.8 (-47.1 to 63.7)	257.2 (75.7 to 481.2)	12.1 (-46.9 to 78.4)
		Male	1557 (992 to 2608)	596.1 (390.5 to 983.5)	60 (38 to 101)	31.6 (20.2 to 51.8)	4845 (3826 to 5850)	710.8 (562.3 to 865.5)	228 (176 to 277)	39.1 (30 to 47.6)	211.1 (75.9 to 396.9)	19.2 (-32.1 to 86.4)	281.7 (111 to 521.2)	23.9 (-31.5 to 96)
	Non-rheumatic valvular heart disease	Both	278 (201 to 369)	40.4 (30.6 to 51.3)	8 (6 to 11)	1.7 (1.4 to 2.2)	593 (506 to 726)	36 (30.8 to 44.1)	21 (18 to 26)	1.5 (1.3 to 2)	113.4 (64 to 197.8)	-10.9 (-31.5 to 20.1)	154.9 (96.1 to 242.5)	-10.3 (-33.2 to 16.9)
		Female	165 (105 to 232)	49 (33.4 to 65.5)	5 (3 to 7)	2.1 (1.5 to 2.9)	324 (262 to 401)	39 (31.8 to 47.6)	12 (10 to 15)	1.8 (1.4 to 2.2)	96.7 (32.6 to 215.2)	-20.3 (-45.5 to 17.9)	140.7 (63.2 to 252.4)	-16.6 (-45 to 20.4)
		Male	113 (77 to 173)	31.4 (22.2 to 45.9)	3 (2 to 5)	1.3 (0.9 to 1.9)	269 (215 to 362)	32.8 (26.1 to 45.1)	9 (7 to 13)	1.3 (1.1 to 2)	137.6 (56.5 to 264.2)	4.4 (-28.7 to 56.2)	176.3 (88.2 to 311.8)	5.1 (-26.3 to 51.4)
	Cardiomyopathy and myocarditis	Both	651 (367 to 1565)	57.9 (37.8 to 109.8)	13 (8 to 26)	2 (1.3 to 3.5)	683 (519 to 1061)	41.3 (31.6 to 63.5)	21 (16 to 33)	1.5 (1.2 to 2.4)	4.9 (-43.2 to 78.6)	-28.7 (-52.1 to 4.9)	65.6 (5 to 147.8)	-21.6 (-44 to 7.1)
		Female	295 (144 to 803)	50.1 (28.4 to 110.4)	6 (3 to 13)	1.7 (0.9 to 3.9)	231 (167 to 392)	28 (20.5 to 47)	7 (5 to 12)	1.1 (0.8 to 1.8)	-21.9 (-59.2 to 56.8)	-44 (-64.8 to -6)	31.2 (-19.7 to 125.5)	-37.4 (-63.3 to 9.6)
		Male	356 (203 to 887)	65.2 (42.5 to 132.4)	7 (5 to 15)	2.2 (1.5 to 3.9)	452 (329 to 724)	54.8 (39.7 to 87.8)	14 (10 to 23)	2 (1.4 to 3.3)	27 (-32.7 to 123.4)	-15.9 (-45.7 to 28)	92.2 (19.5 to 198.8)	-8.2 (-34.5 to 28.5)
	Atrial fibrillation and flutter	Both	286 (216 to 363)	76 (58.2 to 95.6)	8 (5 to 9)	3.1 (2.1 to 3.9)	968 (765 to 1212)	79.7 (63.7 to 99.5)	33 (28 to 39)	3.3 (2.8 to 3.9)	239 (197.9 to 301.3)	4.9 (-8.8 to 28.2)	341.8 (238.6 to 566.6)	8.1 (-18.3 to 67.7)
		Female	156 (115 to 202)	85.2 (61.3 to 109.4)	5 (3 to 7)	3.7 (2.3 to 4.9)	521 (412 to 657)	84.7 (67 to 107.2)	18 (15 to 23)	3.6 (2.9 to 4.7)	234.3 (177.3 to 348.3)	-0.6 (-18.9 to 37.5)	259.3 (149.2 to 575.1)	-1.2 (-32.3 to 90.6)
		Male	130 (95 to 175)	64.5 (48.2 to 84.2)	3 (2 to 3)	2.3 (1.5 to 3)	447 (343 to 564)	74.5 (57.5 to 93.3)	15 (11 to 19)	3.1 (2.1 to 3.7)	244.6 (184 to 315.5)	15.5 (-4.1 to 38.7)	507.4 (299.4 to 769.1)	35.2 (-6.7 to 93.4)
	Aortic aneurysm	Both	125 (92 to 162)	21.5 (16.1 to 27.3)	5 (3 to 6)	1 (0.8 to 1.3)	322 (274 to 376)	21 (17.9 to 24.5)	13 (11 to 16)	1 (0.9 to 1.2)	157.3 (80.9 to 274.8)	-2.2 (-30.4 to 39.9)	190 (108.5 to 318.3)	0.3 (-25.6 to 40.9)
		Female	31 (23 to 41)	11.2 (8.4 to 14.3)	1 (1 to 1)	0.6 (0.4 to 0.7)	78 (64 to 94)	9.4 (7.8 to 11.4)	3 (2 to 4)	0.4 (0.4 to 0.5)	149.1 (74.3 to 262.9)	-16.1 (-38.7 to 18)	153.5 (83.7 to 256)	-23.6 (-43.6 to 8.6)
		Male	94 (62 to 130)	31.6 (21.1 to 42.6)	3 (2 to 5)	1.6 (1.1 to 2.1)	244 (199 to 295)	33.3 (27.2 to 40.1)	10 (8 to 12)	1.7 (1.4 to 2)	160.1 (66 to 328.4)	5.3 (-31.8 to 71.7)	202.4 (95.4 to 396.5)	7.2 (-28.6 to 73.7)

Province	Cause	Sex	1990				2019				1990-2019 percent change (%)			
			DALYs		Death		DALYs		Death		DALYs		Death	
			Number	Rate	Number	Rate	Number	Rate	Number	Rate	Number	Rate	Number	Rate
	Peripheral artery disease	Both	28 (15 to 47)	7.1 (3.9 to 11.9)	0 (0 to 0)	0.1 (0.1 to 0.2)	109 (73 to 162)	8.8 (5.9 to 13.3)	3 (2 to 3)	0.2 (0.2 to 0.3)	289.6 (223.3 to 407.4)	24.6 (5.7 to 56.2)	924.4 (454 to 1490.4)	165.9 (34.5 to 324.9)
		Female	16 (8 to 27)	8.1 (4.2 to 13.9)	0 (0 to 0)	0.1 (0 to 0.2)	56 (33 to 88)	8.9 (5.3 to 14.3)	1 (0 to 1)	0.2 (0.1 to 0.3)	257.2 (175.3 to 360.5)	10.3 (-15 to 40.1)	833.3 (177.5 to 2354.4)	149.4 (-23.9 to 598.7)
		Male	12 (7 to 20)	6 (3.5 to 9.7)	0 (0 to 0)	0.1 (0.1 to 0.1)	53 (39 to 76)	8.7 (6.2 to 12.6)	2 (1 to 2)	0.3 (0.2 to 0.3)	330.5 (235.8 to 496.4)	45.5 (18.8 to 90.8)	991.9 (599.1 to 1690.9)	177.3 (84.8 to 350.9)
	Endocarditis	Both	196 (120 to 338)	17.8 (10.8 to 32.7)	4 (2 to 7)	0.6 (0.3 to 1.2)	239 (165 to 414)	13.8 (9.7 to 23.7)	7 (5 to 13)	0.5 (0.4 to 0.9)	22 (-22 to 89.9)	-22.6 (-39.6 to 4.7)	91.9 (46.2 to 152.9)	-10.8 (-31.6 to 27.9)
		Female	100 (55 to 184)	16.7 (9.5 to 33)	2 (1 to 4)	0.5 (0.3 to 1.3)	98 (64 to 197)	11.6 (7.6 to 23.1)	3 (2 to 7)	0.5 (0.3 to 1)	-1.7 (-49.8 to 92.1)	-30.6 (-53.9 to 2.1)	79.1 (13 to 159.8)	-11.1 (-38.3 to 46.6)
		Male	96 (52 to 190)	18.9 (10.5 to 38.2)	2 (1 to 4)	0.6 (0.4 to 1.2)	140 (93 to 241)	16.1 (10.8 to 27.4)	4 (3 to 7)	0.6 (0.4 to 1)	46.6 (-5.4 to 126.1)	-15 (-39.8 to 27.1)	103.4 (43.9 to 201.7)	-9.3 (-33.1 to 34.9)
	Other cardiovascular and circulatory diseases	Both	4266 (3304 to 5434)	348.2 (280.1 to 439.4)	67 (52 to 87)	10 (8 to 14.5)	6370 (5376 to 7241)	396.5 (331.7 to 448.9)	198 (157 to 227)	15.6 (12.3 to 17.9)	49.3 (10 to 99.3)	13.8 (-17.4 to 46.3)	196.8 (97.7 to 301.9)	56.7 (-6.1 to 108)
		Female	2059 (1521 to 2847)	326.1 (253.6 to 438.3)	31 (23 to 44)	8.9 (6.7 to 14.4)	2725 (2291 to 3171)	335.7 (280.9 to 386.4)	84 (66 to 99)	13.4 (10.4 to 15.8)	32.4 (-9.4 to 89.5)	2.9 (-31.7 to 37.2)	169.2 (63.3 to 285)	49.9 (-24.2 to 108.2)
		Male	2207 (1639 to 2925)	369.6 (288.8 to 482)	35 (27 to 48)	11.1 (8.4 to 15.9)	3644 (2705 to 4327)	459.3 (324 to 546.6)	114 (73 to 138)	18 (11 to 21.9)	65.1 (5.6 to 135.5)	24.3 (-24 to 70.6)	221.1 (74.2 to 373.8)	61.6 (-14.4 to 139.6)
	Chronic respiratory diseases	Both	8459 (6861 to 10235)	1132.2 (944 to 1338.1)	176 (140 to 213)	41.4 (33 to 51.4)	15170 (12822 to 17296)	973.9 (827.4 to 1104.8)	419 (336 to 478)	34.4 (27.7 to 39.2)	79.3 (48.7 to 116.9)	-14 (-28.5 to 4.7)	138.1 (81.8 to 214.9)	-16.9 (-36.7 to 8.1)
		Female	3790 (2852 to 4765)	998.7 (754.2 to 1235.9)	72 (50 to 95)	34 (23.7 to 47.7)	6464 (5086 to 7556)	805.9 (620.4 to 936.5)	160 (99 to 200)	26.1 (16.2 to 32.5)	70.5 (37.3 to 122)	-19.3 (-38.1 to 5.8)	122.4 (47.3 to 237.7)	-23.4 (-53.6 to 14.4)
		Male	4669 (3712 to 5880)	1266.1 (1006.9 to 1584.7)	104 (77 to 138)	49.9 (37.5 to 65.4)	8706 (7228 to 10099)	1152.7 (961.6 to 1326.8)	258 (211 to 306)	43.1 (35.3 to 51.2)	86.5 (42.7 to 135.7)	-9 (-30.6 to 15.5)	149 (73.2 to 245.8)	-13.5 (-37.8 to 18.6)
	Chronic obstructive pulmonary disease	Both	2186 (1812 to 2637)	357.6 (293.7 to 450.5)	51 (38 to 70)	13.3 (9.8 to 19.1)	7729 (6773 to 8705)	527.3 (464.4 to 592.5)	245 (203 to 283)	20.8 (17.2 to 24)	253.6 (184.4 to 333.8)	47.5 (13.6 to 85.4)	380.5 (237.9 to 578.8)	55.6 (4.7 to 119.6)
		Female	941 (696 to 1240)	299.3 (221.3 to 413.2)	19 (11 to 31)	9.9 (5.8 to 17.1)	3014 (2423 to 3604)	400.7 (321.7 to 478.5)	85 (58 to 112)	14.4 (9.8 to 18.9)	220.3 (132 to 354.6)	33.9 (-10.9 to 98.1)	345.9 (131.5 to 824.6)	44.8 (-28.8 to 187.5)
		Male	1245 (990 to 1559)	419.1 (333.3 to 522.3)	32 (23 to 43)	17.5 (12.5 to 23.6)	4715 (3899 to 5483)	662.1 (556.2 to 768.2)	160 (129 to 190)	27.4 (22.1 to 32.7)	278.8 (183.9 to 390.7)	58 (17.8 to 106.9)	401.1 (238.1 to 632.3)	56.5 (10.8 to 128.7)
	Pneumoconiosis	Both	5 (3 to 6)	0.7 (0.5 to 1)	0 (0 to 0)	0 (0 to 0)	69 (53 to 87)	4.2 (3.3 to 5.2)	2 (1 to 2)	0.1 (0.1 to 0.2)	1393.9 (939.2 to 2132.4)	469.6 (290.5 to 748.3)	1361.3 (748.7 to 2579.7)	435.8 (219 to 892.8)
		Female	2 (1 to 2)	0.4 (0.3 to 0.6)	0 (0 to 0)	0 (0 to 0)	36 (26 to 50)	4 (2.9 to 5.5)	0 (0 to 1)	0.1 (0 to 0.1)	2128.1 (1669.3 to 2837.5)	832.3 (625.9 to 1087.8)	3245.1 (1752.7 to 6385.2)	1056.3 (510.5 to 2034.7)
		Male	3 (2 to 4)	1 (0.6 to 1.5)	0 (0 to 0)	0 (0 to 0.1)	32 (24 to 43)	4.4 (3.3 to 5.9)	1 (1 to 2)	0.2 (0.1 to 0.3)	991.9 (521.3 to 1976.1)	332.4 (155.1 to 696.4)	1114.6 (570.3 to 2369.1)	345.4 (152 to 828.8)
	Asthma	Both	6068 (4660 to 7609)	754.1 (582.1 to 942.4)	123 (90 to 158)	27.6 (19.8 to 37.7)	6482 (5142 to 7920)	393.6 (312.8 to 474.7)	156 (118 to 183)	12.3 (9.3 to 14.4)	6.8 (-13.1 to 30.8)	-47.8 (-59.4 to -33.6)	27.2 (-8.8 to 80.7)	-55.3 (-69.6 to -36.8)

Province	Cause	Sex	1990				2019				1990-2019 percent change (%)			
			DALYs		Death		DALYs		Death		DALYs		Death	
			Number	Rate	Number	Rate	Number	Rate	Number	Rate	Number	Rate	Number	Rate
		Female	2747 (1904 to 3614)	680 (440.2 to 922)	52 (28 to 76)	23.7 (12.4 to 37.6)	3013 (2192 to 3798)	358.3 (253.3 to 448.9)	69 (38 to 90)	10.7 (5.9 to 14)	9.7 (-15.6 to 42.9)	-47.3 (-64.1 to -28.8)	32.1 (-24.6 to 105.6)	-54.7 (-76.2 to -28.2)
		Male	3321 (2549 to 4279)	825.6 (619.7 to 1064.1)	71 (50 to 96)	31.8 (22.3 to 43.7)	3470 (2735 to 4282)	431.2 (346.1 to 526.7)	87 (69 to 106)	14 (11.1 to 17.3)	4.5 (-18.9 to 36.1)	-47.8 (-61.1 to -29.2)	23.6 (-15.6 to 89.9)	-55.9 (-70.4 to -34.6)
	Interstitial lung disease and pulmonary sarcoidosis	Both	64 (47 to 87)	9.4 (6.4 to 13.4)	1 (1 to 2)	0.3 (0.2 to 0.6)	246 (162 to 315)	15 (10.2 to 18.7)	7 (4 to 9)	0.6 (0.4 to 0.7)	283.4 (131.1 to 470)	60.1 (-3.7 to 155.7)	420.3 (164.7 to 905.2)	70.3 (-13.7 to 267.5)
		Female	28 (20 to 41)	8.4 (5.3 to 13.9)	1 (0 to 1)	0.3 (0.1 to 0.6)	113 (63 to 155)	13.1 (7.2 to 17.7)	3 (1 to 4)	0.4 (0.2 to 0.6)	298.1 (144 to 558.2)	56.8 (-12.9 to 193.1)	429.1 (130.6 to 1272.4)	61.7 (-36 to 372.7)
		Male	36 (25 to 56)	10.4 (6.9 to 16.7)	1 (0 to 1)	0.4 (0.2 to 0.7)	133 (89 to 172)	17 (11.6 to 22)	4 (3 to 6)	0.7 (0.4 to 0.9)	271.7 (106.4 to 475.3)	63 (-6.2 to 162.6)	414.2 (154.7 to 924.6)	73.1 (-10.7 to 258.5)
	Other chronic respiratory diseases	Both	136 (98 to 208)	10.3 (7.8 to 14.6)	1 (0 to 2)	0.1 (0.1 to 0.2)	644 (375 to 868)	33.8 (18.6 to 46)	9 (2 to 16)	0.6 (0.1 to 1.1)	372.2 (105.1 to 650.5)	227.3 (47.4 to 413)	1205.4 (24.4 to 3655)	576.4 (-20.9 to 1716.8)
		Female	72 (51 to 111)	10.6 (8 to 15)	0 (0 to 1)	0.1 (0 to 0.2)	287 (180 to 376)	29.7 (17.9 to 39.3)	3 (1 to 5)	0.4 (0.1 to 0.7)	298 (94.2 to 513)	180 (45.8 to 315.4)	1025.7 (5.2 to 3375.9)	460.8 (-32.4 to 1556.9)
		Male	64 (43 to 104)	10 (7.2 to 15)	0 (0 to 1)	0.1 (0.1 to 0.2)	356 (184 to 512)	38 (18.2 to 56.1)	6 (1 to 11)	0.8 (0.1 to 1.6)	455.8 (104.2 to 889.7)	279.4 (42.7 to 565.1)	1325.7 (32.9 to 4532.8)	644.4 (-17.1 to 2178.4)
	Digestive diseases	Both	9534 (7708 to 11461)	1079.5 (873.6 to 1299.3)	196 (157 to 241)	36.6 (28.2 to 47.6)	12639 (10735 to 14917)	738 (634.1 to 857.9)	330 (284 to 385)	24.3 (21.1 to 28.3)	32.6 (6.4 to 70.4)	-31.6 (-44.8 to -13.7)	67.9 (29.6 to 123.8)	-33.6 (-51.2 to -12.1)
		Female	4327 (3404 to 5448)	964.2 (755.6 to 1215.8)	83 (62 to 109)	31.9 (22 to 46)	5380 (4486 to 6519)	619.3 (524.5 to 739.4)	126 (108 to 149)	19 (15.9 to 22.4)	24.3 (-4 to 64.6)	-35.8 (-51.1 to -17.4)	53.2 (8.1 to 112.4)	-40.5 (-61.5 to -11.7)
		Male	5207 (3908 to 6598)	1185.8 (923.2 to 1476.6)	114 (86 to 144)	41.4 (31.3 to 53.6)	7259 (5981 to 8679)	863.2 (712.3 to 1030.4)	203 (164 to 248)	30 (24.3 to 36.4)	39.4 (2.7 to 96.5)	-27.2 (-44.6 to -0.7)	78.6 (30.6 to 156)	-27.6 (-46.9 to 5.2)
	Cirrhosis and other chronic liver diseases	Both	4830 (3652 to 6005)	562.4 (428.1 to 714.2)	122 (93 to 155)	21.7 (15.7 to 28.9)	6258 (5318 to 7351)	371.6 (316.7 to 435.3)	214 (182 to 250)	15 (12.8 to 17.5)	29.6 (-3.7 to 78.6)	-33.9 (-51.5 to -9.3)	75 (28.7 to 141.8)	-31 (-50.9 to -3.2)
		Female	2082 (1347 to 2897)	467.4 (318 to 638.2)	50 (34 to 68)	18.5 (11.9 to 27.4)	2166 (1810 to 2585)	258.2 (214.1 to 305.3)	76 (62 to 91)	10.8 (8.8 to 12.9)	4 (-29 to 63.2)	-44.7 (-62.8 to -16.2)	50.9 (-0.1 to 134.8)	-41.6 (-64.7 to -2.8)
		Male	2748 (1958 to 3635)	650.1 (472.7 to 841.2)	72 (52 to 94)	24.9 (17.7 to 33)	4093 (3252 to 5071)	490.6 (390.4 to 603.7)	138 (110 to 170)	19.4 (15.6 to 23.8)	48.9 (0.9 to 122.4)	-24.5 (-46.5 to 11.5)	91.8 (34.9 to 183.8)	-22 (-44.3 to 17.7)
	Upper digestive system diseases	Both	2360 (1727 to 3253)	300.5 (223.6 to 395.6)	40 (29 to 51)	8.9 (6.4 to 12)	3145 (2154 to 4743)	172.3 (122.4 to 252)	40 (34 to 48)	3.3 (2.7 to 4)	33.3 (2 to 63.4)	-42.6 (-54.9 to -29.3)	0.6 (-27.4 to 43.3)	-63.4 (-74.8 to -47.2)
		Female	1079 (751 to 1537)	275.3 (191.3 to 376.7)	16 (10 to 23)	7.6 (4.8 to 11.8)	1592 (1050 to 2461)	171 (117.3 to 255.1)	17 (13 to 21)	2.8 (2.2 to 3.6)	47.6 (11.2 to 83.1)	-37.9 (-54.3 to -21.4)	4.7 (-35.5 to 76.6)	-63.5 (-79.2 to -36.3)
		Male	1281 (912 to 1806)	324.1 (238.3 to 430.8)	24 (17 to 33)	10.3 (7.4 to 14.3)	1553 (1071 to 2294)	174 (124.4 to 247.5)	23 (18 to 30)	3.8 (2.9 to 4.9)	21.3 (-14.2 to 58.5)	-46.3 (-59.8 to -29.6)	-2.1 (-33 to 49.4)	-63.2 (-74.8 to -45.1)
	Appendicitis	Both	346 (192 to 606)	28.3 (19 to 42.6)	6 (4 to 9)	0.9 (0.6 to 1.3)	198 (158 to 258)	11.1 (8.9 to 14.6)	4 (3 to 6)	0.3 (0.2 to 0.4)	-42.7 (-66.5 to 27)	-60.6 (-74.5 to -29.7)	-29.3 (-56.8 to 41.7)	-64.7 (-78.6 to -45.2)
		Female	172 (88 to 331)	28.4 (17.6 to 46.8)	3 (2 to 5)	0.9 (0.5 to 1.6)	95 (67 to 126)	10.7 (7.7 to 14)	2 (1 to 3)	0.3 (0.2 to 0.4)	-44.9 (-71.2 to 28.3)	-62.2 (-79.1 to -30.5)	-32.6 (-65.4 to 41.9)	-66.3 (-84 to -38.6)
		Male	174 (87 to 365)	27.9 (16.4 to 49.8)	3 (2 to 6)	0.8 (0.5 to 1.3)	103 (78 to 142)	11.6 (8.7 to 16)	2 (2 to 3)	0.3 (0.2 to 0.5)	-40.6 (-70.8 to 46.4)	-58.6 (-75.5 to -15.1)	-26 (-59 to 71.4)	-61.4 (-75.4 to -26)

Province	Cause	Sex	1990				2019				1990-2019 percent change (%)			
			DALYs		Death		DALYs		Death		DALYs		Death	
			Number	Rate	Number	Rate	Number	Rate	Number	Rate	Number	Rate	Number	Rate
	Paralytic ileus and intestinal obstruction	Both	1017 (567 to 1698)	63.2 (41.3 to 98.8)	14 (9 to 23)	1.7 (1.2 to 2.7)	574 (400 to 814)	36.7 (26.4 to 50.8)	16 (13 to 21)	1.3 (1 to 1.7)	-43.5 (-72.5 to 23)	-41.9 (-69 to 10.9)	12.6 (-38.1 to 104.3)	-25.6 (-54.6 to 17.8)
		Female	483 (230 to 911)	61 (34.4 to 103.9)	7 (4 to 12)	1.7 (1 to 2.8)	235 (165 to 358)	29.8 (21.3 to 44.6)	6 (4 to 8)	0.9 (0.7 to 1.3)	-51.3 (-77.9 to 22.6)	-51.1 (-74.8 to 1.2)	-13.6 (-53.5 to 76.7)	-44.7 (-66.5 to 0.3)
		Male	533 (245 to 1020)	65.5 (37.4 to 113.9)	7 (4 to 13)	1.8 (1.2 to 3)	339 (203 to 502)	43.9 (27 to 63)	10 (6 to 14)	1.7 (1.1 to 2.3)	-36.4 (-75 to 72.8)	-33 (-72 to 44.7)	36.6 (-39.5 to 199.3)	-8.6 (-54.1 to 56.2)
	Inguinal, femoral, and abdominal hernia	Both	220 (136 to 344)	17.6 (11.9 to 24.7)	1 (1 to 2)	0.2 (0.1 to 0.4)	323 (221 to 451)	17.4 (12.3 to 24)	2 (1 to 3)	0.2 (0.1 to 0.2)	46.7 (-5.5 to 102.1)	-1.1 (-24.3 to 23.2)	89.1 (-18 to 301.8)	-21.6 (-56.5 to 25.5)
		Female	86 (48 to 169)	12.7 (8.1 to 20.6)	0 (0 to 1)	0.1 (0.1 to 0.2)	114 (77 to 164)	12.2 (8.3 to 17.4)	1 (0 to 1)	0.1 (0.1 to 0.2)	32.4 (-36.6 to 115.6)	-3.7 (-42.6 to 35.6)	77.9 (-48 to 348.7)	-19.9 (-53.8 to 51.1)
		Male	134 (85 to 198)	22.5 (15.3 to 31.5)	1 (0 to 1)	0.3 (0.2 to 0.6)	209 (141 to 291)	22.8 (15.6 to 31.2)	1 (1 to 2)	0.2 (0.2 to 0.3)	55.9 (12.6 to 115)	0.9 (-20.9 to 27.4)	96 (-4.1 to 297.9)	-29.7 (-67.5 to 21.5)
	Inflammatory bowel disease	Both	77 (54 to 114)	9.7 (7 to 13.2)	1 (1 to 1)	0.2 (0.1 to 0.2)	204 (147 to 271)	10.8 (7.8 to 14.2)	3 (2 to 4)	0.2 (0.1 to 0.3)	166.6 (61.7 to 250.3)	11.5 (-21.6 to 44.7)	238.4 (39.9 to 482.1)	29.6 (-34.3 to 124.7)
		Female	37 (25 to 55)	9.2 (6.5 to 12.8)	0 (0 to 1)	0.1 (0.1 to 0.2)	99 (68 to 132)	10.3 (7.2 to 13.5)	1 (1 to 2)	0.2 (0.1 to 0.2)	169.2 (67.1 to 269.9)	11.2 (-21.9 to 50.8)	278.3 (45.1 to 672.1)	40.4 (-39.8 to 202)
		Male	40 (28 to 59)	10.2 (7.2 to 14)	1 (0 to 1)	0.2 (0.1 to 0.3)	105 (70 to 146)	11.4 (7.7 to 15.6)	2 (1 to 2)	0.2 (0.1 to 0.3)	164.3 (55.9 to 278.7)	12.4 (-26.7 to 59.2)	214 (30.3 to 500.3)	21.3 (-44.3 to 131.5)
	Vascular intestinal disorders	Both	50 (37 to 68)	10.2 (7 to 14.6)	2 (1 to 3)	0.6 (0.4 to 0.9)	118 (98 to 142)	8.4 (7 to 10.1)	5 (5 to 7)	0.5 (0.4 to 0.6)	134.9 (55.4 to 240.7)	-18.4 (-48.7 to 24)	162.2 (63.8 to 302.7)	-20.2 (-51.3 to 29.4)
		Female	22 (14 to 35)	10 (5.4 to 17.2)	1 (1 to 2)	0.6 (0.3 to 1)	50 (40 to 63)	6.9 (5.5 to 8.7)	2 (2 to 3)	0.4 (0.3 to 0.5)	126.1 (27.6 to 303.4)	-30.8 (-64.4 to 39.5)	125.7 (13.3 to 368.7)	-36.2 (-69.6 to 43.4)
		Male	28 (19 to 39)	10.5 (7.5 to 15)	1 (1 to 2)	0.6 (0.4 to 0.8)	67 (52 to 85)	9.9 (7.7 to 12.6)	3 (2 to 4)	0.6 (0.4 to 0.7)	141.9 (58.7 to 275.1)	-5.7 (-36.6 to 42.3)	196.1 (92.3 to 359.4)	-4.2 (-37 to 46.1)
	Gallbladder and biliary diseases	Both	384 (257 to 558)	52.8 (36.3 to 74.4)	4 (2 to 7)	1 (0.7 to 1.8)	939 (685 to 1261)	55.8 (42 to 72.2)	16 (12 to 19)	1.4 (1.1 to 1.7)	144.5 (81.3 to 203.8)	5.7 (-19.9 to 30.3)	318.3 (114.1 to 611.5)	33.5 (-24.1 to 114.4)
		Female	251 (163 to 370)	67.5 (44.7 to 96.6)	2 (1 to 4)	1 (0.6 to 1.8)	615 (431 to 856)	69.5 (50.3 to 93)	8 (6 to 10)	1.4 (1 to 1.7)	144.7 (86 to 203.2)	2.8 (-20.7 to 27.4)	319.1 (105.4 to 706.8)	34.8 (-28.7 to 165.1)
		Male	133 (90 to 202)	38.5 (26.7 to 58.3)	2 (1 to 4)	1.1 (0.7 to 2)	325 (240 to 428)	41.9 (31.8 to 53.5)	8 (6 to 11)	1.4 (1 to 1.9)	144.3 (64.9 to 243.1)	8.7 (-25.9 to 53.5)	317.4 (103 to 740.4)	32.3 (-30 to 142.1)
	Pancreatitis	Both	123 (80 to 197)	19.2 (12.5 to 29.5)	4 (2 to 6)	0.8 (0.5 to 1.4)	262 (213 to 355)	16.1 (13.2 to 21.2)	9 (8 to 12)	0.7 (0.6 to 0.9)	112.4 (48.1 to 230.8)	-15.8 (-44.9 to 33.2)	151.7 (60.8 to 321.8)	-14.1 (-49.9 to 47.6)
		Female	53 (34 to 85)	17.5 (11 to 27.4)	2 (1 to 3)	0.8 (0.5 to 1.5)	131 (85 to 170)	16.7 (10.8 to 21.5)	5 (3 to 7)	0.8 (0.5 to 1.1)	146.8 (43 to 318.5)	-5 (-51.4 to 69.3)	200.1 (49.4 to 472.5)	-2.2 (-57 to 102.4)
		Male	70 (42 to 124)	20.4 (12.5 to 34.3)	2 (1 to 4)	0.8 (0.5 to 1.3)	131 (90 to 227)	15.6 (10.8 to 26.2)	4 (3 to 7)	0.6 (0.4 to 1)	86.4 (22.5 to 195.5)	-23.5 (-49.9 to 18)	110.9 (34 to 242.8)	-22 (-51.2 to 23.2)
	Other digestive diseases	Both	128 (92 to 206)	15.6 (11.5 to 22.6)	3 (2 to 4)	0.6 (0.4 to 1)	617 (253 to 766)	37.7 (16 to 46.1)	20 (8 to 25)	1.6 (0.7 to 1.9)	382.1 (36.8 to 643.9)	141.9 (-20.7 to 257.3)	639.8 (117.1 to 1052.3)	150.9 (-13.9 to 346.3)
		Female	62 (42 to 102)	15.2 (10.1 to 24.7)	1 (1 to 2)	0.6 (0.3 to 1.2)	284 (106 to 365)	34 (13.5 to 43)	9 (3 to 12)	1.4 (0.5 to 1.8)	360.1 (33.4 to 664.7)	123.9 (-26.6 to 264.6)	588.6 (85.9 to 1220.6)	126.9 (-23.9 to 429.9)
		Male	66 (45 to 116)	15.9 (11.2 to 23.2)	1 (1 to 2)	0.6 (0.4 to 1)	334 (147 to 437)	41.5 (18.7 to 53.9)	11 (5 to 15)	1.8 (0.8 to 2.3)	402.4 (41.3 to 722.9)	160.5 (-10.6 to 313.7)	686.9 (143.1 to 1213.2)	175.3 (-8.7 to 367.3)

Province	Cause	Sex	1990				2019				1990-2019 percent change (%)			
			DALYs		Death		DALYs		Death		DALYs		Death	
			Number	Rate	Number	Rate	Number	Rate	Number	Rate	Number	Rate	Number	Rate
	Neurological disorders	Both	14897 (7899 to 25246)	1497.1 (852.3 to 2474.7)	106 (61 to 205)	31.3 (13.1 to 73.3)	24457 (12578 to 41616)	1432.1 (797.1 to 2324.4)	338 (157 to 736)	32.7 (13.9 to 74.1)	64.2 (27.6 to 106.1)	-4.3 (-19 to 11.6)	219.1 (119.7 to 332.5)	4.4 (-8.4 to 40.4)
		Female	8160 (4076 to 14474)	1624.7 (867.9 to 2732)	55 (29 to 110)	30 (11.7 to 73)	13715 (6296 to 24634)	1565.9 (791.2 to 2677.4)	160 (68 to 366)	31.6 (12.2 to 75.8)	68.1 (30.5 to 107.8)	-3.6 (-18.9 to 12.4)	191.1 (94.6 to 333.4)	5.5 (-13.4 to 52.2)
		Male	6737 (3717 to 10898)	1375.2 (826.6 to 2145.8)	51 (30 to 96)	33.2 (14.4 to 75.8)	10742 (6080 to 17219)	1300.3 (781.3 to 2025.7)	178 (86 to 377)	34 (15.4 to 74.1)	59.4 (22.3 to 109.9)	-5.5 (-21.3 to 14.6)	249.5 (131.9 to 390.2)	2.2 (-15.1 to 37.9)
	Alzheimer's disease and other dementias	Both	1024 (449 to 2382)	365.8 (162 to 831.3)	53 (13 to 149)	24 (5.7 to 65.7)	3985 (1739 to 8739)	378.3 (167.5 to 834)	237 (57 to 639)	24.9 (6 to 66.8)	289.2 (244.2 to 391.8)	3.4 (-5.8 to 29.1)	345.6 (284.7 to 538.2)	3.6 (-8.3 to 47)
		Female	550 (241 to 1227)	366.9 (164.2 to 800.3)	31 (7 to 86)	23.9 (5.7 to 66.4)	2080 (910 to 4714)	391.5 (173.3 to 880.5)	121 (29 to 331)	25.7 (6.3 to 70.7)	278.6 (226.1 to 398)	6.7 (-7 to 39.4)	291.8 (222 to 495.7)	7.9 (-10.8 to 64.2)
		Male	474 (206 to 1123)	366.4 (160.2 to 835.6)	22 (5 to 65)	24.3 (5.6 to 65.8)	1904 (837 to 4251)	364.8 (160.6 to 825.3)	116 (27 to 316)	24 (5.7 to 64.2)	301.5 (230.9 to 409.5)	-0.4 (-13.8 to 23.5)	419.6 (315.9 to 639.6)	-1.1 (-17.6 to 38.1)
	Parkinson's disease	Both	287 (240 to 337)	74.2 (62.8 to 89)	13 (11 to 16)	4.3 (3.6 to 5.6)	1115 (956 to 1256)	92.4 (79.9 to 103.8)	60 (52 to 68)	5.6 (4.8 to 6.3)	288.7 (214.8 to 384.5)	24.6 (-1.2 to 52.8)	369 (270.6 to 498.8)	29.9 (0.3 to 62.9)
		Female	110 (87 to 139)	56.7 (45 to 76.5)	5 (4 to 7)	3.2 (2.5 to 4.9)	445 (378 to 517)	69.6 (59.6 to 81.4)	22 (19 to 26)	4 (3.4 to 4.8)	304.7 (207.2 to 432.7)	22.7 (-9.6 to 59.6)	350.4 (228.1 to 519.7)	25.4 (-9.8 to 68.7)
		Male	177 (138 to 219)	94.9 (75.7 to 115.9)	8 (6 to 10)	5.8 (4.5 to 7.2)	669 (520 to 786)	116.5 (91.5 to 136.9)	38 (29 to 45)	7.2 (5.5 to 8.6)	278.7 (180 to 424.8)	22.8 (-9.6 to 65)	380.7 (247 to 585.6)	24.8 (-12 to 74.5)
	Idiopathic epilepsy	Both	4630 (2511 to 7334)	282.2 (154.8 to 449.5)	34 (17 to 46)	2.4 (1.5 to 3)	3239 (1794 to 5530)	171.1 (93.9 to 291.6)	23 (17 to 27)	1.3 (0.9 to 1.5)	-30 (-64.4 to 43.2)	-39.4 (-70.1 to 23.3)	-32.9 (-52.1 to 11)	-46.9 (-59.8 to -18.5)
		Female	2262 (1137 to 3619)	278 (148.1 to 445.4)	17 (6 to 25)	2.4 (1.2 to 3.3)	1477 (779 to 2484)	156.5 (82.1 to 264.9)	10 (7 to 12)	1.1 (0.7 to 1.3)	-34.7 (-67.2 to 47.7)	-43.7 (-72.3 to 25.3)	-41.8 (-61.7 to 15.8)	-54.6 (-68.1 to -17.6)
		Male	2368 (1229 to 3755)	286.3 (153.6 to 461.2)	17 (9 to 23)	2.4 (1.5 to 3)	1762 (979 to 3072)	185.4 (101.8 to 323)	13 (10 to 16)	1.4 (1.1 to 1.7)	-25.6 (-62.8 to 53.1)	-35.2 (-67.7 to 34.6)	-24.1 (-48.7 to 33.3)	-39.3 (-55.7 to -1.3)
	Multiple sclerosis	Both	195 (123 to 368)	23.6 (14.6 to 45.2)	2 (1 to 6)	0.3 (0.1 to 0.9)	493 (328 to 862)	24.2 (16.2 to 41.8)	7 (4 to 17)	0.4 (0.2 to 0.9)	153.3 (98.1 to 220.7)	2.6 (-19.4 to 30.4)	203.3 (108.2 to 393.6)	15.2 (-22.7 to 94.3)
		Female	106 (65 to 199)	25.5 (15.8 to 47.8)	1 (0 to 3)	0.3 (0.1 to 0.9)	269 (183 to 467)	26.1 (17.8 to 44.6)	3 (2 to 8)	0.3 (0.2 to 0.8)	153.4 (85.6 to 242.2)	2.1 (-25 to 38.2)	208.1 (83.4 to 521.8)	13.5 (-28.9 to 137.6)
		Male	89 (51 to 190)	21.6 (12.3 to 47.7)	1 (0 to 4)	0.4 (0.1 to 1.1)	224 (138 to 426)	22.4 (14.1 to 42.1)	4 (2 to 9)	0.4 (0.2 to 1)	153.3 (73.1 to 238)	3.5 (-29.3 to 38)	199.3 (75.5 to 408.5)	18.2 (-29.7 to 101.7)
	Motor neuron disease	Both	21 (17 to 27)	2.3 (1.7 to 3)	0 (0 to 1)	0.1 (0 to 0.1)	46 (37 to 57)	2.4 (1.9 to 3)	1 (1 to 1)	0.1 (0 to 0.1)	114.4 (52.6 to 192.3)	6.1 (-27.5 to 49.4)	155.8 (53.9 to 310.4)	7 (-37.2 to 73.9)
		Female	11 (9 to 14)	2.1 (1.6 to 2.8)	0 (0 to 0)	0 (0 to 0.1)	21 (17 to 26)	2.1 (1.7 to 2.6)	0 (0 to 1)	0 (0 to 0.1)	87.8 (29.6 to 151.1)	0.6 (-33 to 39.1)	132.7 (31.5 to 264.8)	0 (-46.6 to 61.9)
		Male	10 (8 to 15)	2.4 (1.7 to 3.4)	0 (0 to 0)	0.1 (0 to 0.1)	25 (19 to 32)	2.7 (2.1 to 3.5)	1 (0 to 1)	0.1 (0 to 0.1)	143.5 (48.8 to 268.7)	13.9 (-32.4 to 78.3)	175 (40.4 to 412.2)	16.8 (-39.1 to 114.7)
	Headache disorders	Both	8080 (1684 to 17857)	706.1 (168.9 to 1512.8)	--	--	14557 (3530 to 30857)	708.4 (171.6 to 1509.4)	--	--	80.2 (64.6 to 118.5)	0.3 (-4.3 to 6.3)	--	--
		Female	4831 (931 to 10824)	857.1 (192.3 to 1883)	--	--	8980 (2010 to 19435)	872.8 (198.9 to 1893)	--	--	85.9 (69.5 to 118.5)	1.8 (-4.6 to 8.5)	--	--

Province	Cause	Sex	1990 DALYs Number	1990 DALYs Rate	1990 Death Number	1990 Death Rate	2019 DALYs Number	2019 DALYs Rate	2019 Death Number	2019 Death Rate	1990-2019 percent change (%) DALYs Number	1990-2019 percent change (%) DALYs Rate	1990-2019 percent change (%) Death Number	1990-2019 percent change (%) Death Rate
		Male	3249 (730 to 7049)	556.3 (148.3 to 1166.4)	--	--	5577 (1578 to 11662)	545.4 (151 to 1141.1)	--	--	71.7 (53.3 to 120.1)	-2 (-8.6 to 8.1)	--	--
	Other neurological disorders	Both	660 (308 to 1153)	42.9 (22.5 to 69.8)	3 (2 to 4)	0.3 (0.2 to 0.4)	1022 (651 to 1643)	55.2 (35.6 to 87.9)	10 (9 to 11)	0.6 (0.5 to 0.7)	54.8 (-20.9 to 250.5)	28.7 (-30 to 167.1)	226.7 (145.9 to 373.6)	98.7 (49.7 to 187.7)
		Female	290 (120 to 524)	38.2 (18.9 to 64.1)	1 (1 to 1)	0.2 (0.2 to 0.3)	442 (269 to 709)	47.3 (28.8 to 75.9)	4 (3 to 5)	0.4 (0.3 to 0.5)	52.7 (-26.4 to 287.6)	23.6 (-36.7 to 170.5)	251.5 (141.7 to 412.2)	90 (25 to 194.1)
		Male	371 (182 to 631)	47.4 (25.8 to 76.9)	2 (1 to 3)	0.4 (0.3 to 0.5)	580 (375 to 922)	63.1 (41.7 to 99)	6 (5 to 7)	0.8 (0.7 to 0.9)	56.5 (-16.9 to 243.1)	33.1 (-27.1 to 167.8)	213.9 (119.6 to 384.3)	105.4 (50.8 to 210.2)
	Mental disorders	Both	24057 (17688 to 31960)	2055 (1518.1 to 2709.6)	0 (0 to 0)	0 (0 to 0)	42410 (31133 to 55822)	2094.1 (1546.6 to 2767.6)	0 (0 to 0)	0 (0 to 0)	76.3 (66.9 to 86.2)	1.9 (-1 to 4.9)	203.1 (37.6 to 878.9)	33.6 (-39.5 to 355.6)
		Female	13177 (9651 to 17464)	2302.7 (1695.3 to 3042.3)	0 (0 to 0)	0 (0 to 0)	23734 (17322 to 31429)	2347.1 (1706.5 to 3105.1)	0 (0 to 0)	0 (0 to 0)	80.1 (68.9 to 91.5)	1.9 (-2 to 6.3)	158.7 (3.6 to 997.9)	15.6 (-54.2 to 421.5)
		Male	10880 (7955 to 14433)	1808.5 (1339.9 to 2379.4)	0 (0 to 0)	0 (0 to 0)	18676 (13736 to 24656)	1842.1 (1365.5 to 2429)	0 (0 to 0)	0 (0 to 0)	71.7 (61.5 to 82.3)	1.9 (-1.8 to 5.6)	530.3 (97.8 to 1974.8)	178.5 (-13.9 to 876.3)
	Schizophrenia	Both	1512 (1086 to 1959)	160.1 (116.2 to 205.4)	--	--	3516 (2557 to 4525)	161.2 (118.7 to 206.4)	--	--	132.5 (109.9 to 158.5)	0.7 (-7.8 to 10.6)	--	--
		Female	713 (502 to 925)	151.8 (107.8 to 195.9)	--	--	1663 (1203 to 2131)	152.8 (112.2 to 194.5)	--	--	133.2 (104.2 to 171.5)	0.7 (-11.2 to 15.7)	--	--
		Male	799 (575 to 1045)	168.3 (121.2 to 218.2)	--	--	1853 (1347 to 2400)	169.6 (123.7 to 218.6)	--	--	131.9 (103.1 to 165)	0.8 (-10.4 to 13.5)	--	--
	Depressive disorders	Both	6770 (4324 to 9896)	657.2 (432.7 to 957.8)	--	--	14234 (9271 to 21164)	693.9 (460.6 to 1008.3)	--	--	110.3 (88.8 to 133.9)	5.6 (0.2 to 10.9)	--	--
		Female	4254 (2722 to 6216)	822.8 (542.6 to 1202.9)	--	--	8737 (5689 to 12883)	853.1 (562.3 to 1241.9)	--	--	105.4 (81.9 to 133.7)	3.7 (-2.9 to 11.5)	--	--
		Male	2516 (1594 to 3709)	493.5 (322.9 to 712.5)	--	--	5497 (3593 to 8180)	535.2 (355.3 to 783.5)	--	--	118.5 (92.6 to 143.5)	8.5 (0.7 to 16.7)	--	--
	Bipolar disorder	Both	1859 (1127 to 2888)	172.3 (106.3 to 267.5)	--	--	3565 (2172 to 5577)	174.4 (106.3 to 268.9)	--	--	91.8 (73.8 to 111.6)	1.2 (-6.1 to 8.9)	--	--
		Female	1015 (608 to 1599)	185.7 (113.6 to 289.4)	--	--	1907 (1176 to 2940)	187.7 (115.5 to 288)	--	--	87.9 (65.3 to 113.6)	1.1 (-8.8 to 11.2)	--	--
		Male	843 (505 to 1316)	158.9 (97.9 to 244.1)	--	--	1658 (1004 to 2554)	161.4 (98.1 to 248.9)	--	--	96.6 (74.7 to 123.5)	1.6 (-8.6 to 12.3)	--	--
	Anxiety disorders	Both	8358 (5842 to 11539)	676.1 (471.7 to 932.3)	--	--	13889 (9704 to 19206)	697.6 (488.7 to 958.2)	--	--	66.2 (50.7 to 82.2)	3.2 (-3.1 to 9.1)	--	--
		Female	5014 (3512 to 6959)	827.6 (582.3 to 1134.7)	--	--	8497 (5875 to 11727)	854.6 (592.4 to 1176.3)	--	--	69.5 (51.2 to 88.1)	3.3 (-4.9 to 11.8)	--	--
		Male	3344 (2314 to 4664)	525.4 (366.3 to 731.5)	--	--	5391 (3734 to 7447)	542.1 (375.8 to 746.7)	--	--	61.2 (44.4 to 81.3)	3.2 (-5.2 to 12.1)	--	--
	Eating disorders	Both	591 (361 to 887)	44.1 (27.1 to 66.4)	0 (0 to 0)	0 (0 to 0)	1070 (653 to 1600)	50.7 (30.7 to 76.7)	0 (0 to 0)	0 (0 to 0)	81.1 (54.4 to 110.5)	14.8 (3.6 to 28.4)	203.1 (37.6 to 878.9)	33.6 (-39.5 to 355.6)

Province	Cause	Sex	1990				2019				1990-2019 percent change (%)			
			DALYs		Death		DALYs		Death		DALYs		Death	
			Number	Rate	Number	Rate	Number	Rate	Number	Rate	Number	Rate	Number	Rate
		Female	368 (224 to 550)	54.7 (33.2 to 81.3)	0 (0 to 0)	0 (0 to 0)	652 (404 to 973)	63.3 (39.3 to 96)	0 (0 to 0)	0 (0 to 0)	77.4 (49.6 to 111.5)	15.9 (1.9 to 34)	158.7 (3.6 to 997.9)	15.6 (-54.2 to 421.5)
		Male	223 (136 to 347)	33.4 (19.9 to 52.7)	0 (0 to 0)	0 (0 to 0)	417 (241 to 644)	38.4 (22.3 to 59.2)	0 (0 to 0)	0 (0 to 0)	87.2 (51.2 to 128)	14.9 (-2.9 to 34.2)	530.3 (97.8 to 1974.8)	178.5 (-13.9 to 876.3)
	Autism spectrum disorders	Both	649 (420 to 952)	44 (28.5 to 64.3)	--	--	877 (571 to 1289)	44.7 (29.2 to 65.1)	--	--	35.1 (20.5 to 51.1)	1.4 (-9.3 to 12.4)	--	--
		Female	164 (105 to 243)	22.4 (14.4 to 33.1)	--	--	220 (141 to 328)	22.6 (14.5 to 33.7)	--	--	34.2 (12.9 to 57.1)	1 (-14.1 to 17.1)	--	--
		Male	486 (318 to 712)	65.4 (42.5 to 96.3)	--	--	658 (424 to 962)	66.4 (43.2 to 97.4)	--	--	35.4 (18.2 to 54.7)	1.5 (-11.6 to 14.6)	--	--
	Attention-deficit/hyperactivity disorder	Both	538 (302 to 922)	32.3 (18.3 to 55)	--	--	652 (371 to 1106)	33.5 (19 to 57)	--	--	21.3 (3.4 to 41.3)	3.8 (-10.3 to 17.8)	--	--
		Female	165 (89 to 293)	20.1 (11.2 to 35.3)	--	--	200 (112 to 335)	20.9 (11.6 to 35.3)	--	--	21.6 (1.5 to 49.3)	4 (-12 to 25.3)	--	--
		Male	373 (208 to 632)	44.5 (25.1 to 74.2)	--	--	451 (258 to 780)	45.8 (26 to 79.8)	--	--	21.1 (-1.1 to 48.8)	3 (-14.6 to 23.2)	--	--
	Conduct disorder	Both	1545 (899 to 2438)	75.5 (43.7 to 118.6)	--	--	1311 (759 to 2099)	74.3 (43.1 to 118)	--	--	-15.1 (-22.9 to -6.4)	-1.6 (-10.3 to 8.7)	--	--
		Female	535 (292 to 886)	52.6 (28.8 to 87)	--	--	444 (237 to 732)	51 (27.2 to 83.8)	--	--	-17 (-28.8 to -2.3)	-3.1 (-17 to 14.2)	--	--
		Male	1010 (597 to 1583)	97.8 (57.5 to 152.7)	--	--	867 (507 to 1391)	96.7 (56.3 to 154.8)	--	--	-14.1 (-23.5 to -3.6)	-1.1 (-11.7 to 10.5)	--	--
	Idiopathic developmental intellectual disability	Both	1382 (747 to 2193)	92.9 (50.4 to 146.5)	--	--	1250 (633 to 2106)	63.2 (32.1 to 106.5)	--	--	-9.6 (-21.5 to -0.7)	-32 (-41 to -25.2)	--	--
		Female	606 (331 to 974)	82.6 (45.4 to 131.8)	--	--	570 (293 to 954)	58.3 (29.8 to 97.4)	--	--	-5.9 (-19.6 to 4.3)	-29.5 (-39.2 to -21.8)	--	--
		Male	776 (420 to 1228)	103.2 (55.8 to 161.8)	--	--	680 (347 to 1144)	68.1 (34.7 to 114.3)	--	--	-12.4 (-25.4 to -1.9)	-34 (-44.1 to -26)	--	--
	Other mental disorders	Both	853 (546 to 1313)	100.4 (64.3 to 152.2)	--	--	2047 (1304 to 3185)	100.5 (64.9 to 154.9)	--	--	139.9 (122.3 to 160)	0 (-5.1 to 6.1)	--	--
		Female	344 (214 to 524)	82.6 (52.3 to 125.1)	--	--	842 (541 to 1300)	82.8 (53.3 to 125.9)	--	--	145.3 (120.4 to 173.4)	0.2 (-8.1 to 9.3)	--	--
		Male	510 (324 to 775)	118.1 (75.5 to 178.5)	--	--	1204 (759 to 1847)	118.3 (75.2 to 179.8)	--	--	136.2 (114.5 to 159.1)	0.2 (-6.9 to 8)	--	--
	Substance use disorders	Both	2714 (2102 to 3386)	256.1 (202.6 to 314.8)	20 (15 to 25)	2.4 (1.9 to 3)	5968 (4670 to 7409)	281.4 (219.7 to 349.2)	42 (36 to 51)	2.1 (1.8 to 2.5)	119.9 (93.1 to 152.2)	9.9 (-4 to 25.6)	112.7 (58.7 to 192.9)	-11.8 (-32.5 to 20.9)
		Female	1002 (740 to 1304)	185.2 (138.6 to 235.2)	7 (5 to 10)	1.7 (1.2 to 2.2)	1772 (1338 to 2262)	168.4 (126.5 to 214)	10 (8 to 13)	1 (0.8 to 1.3)	76.8 (45.7 to 114.4)	-9 (-23.4 to 9.8)	42.5 (-7.1 to 126)	-38.6 (-57.7 to -3.2)
		Male	1712 (1340 to 2153)	327 (258.1 to 407.6)	13 (9 to 17)	3.2 (2.2 to 4.2)	4196 (3312 to 5206)	392.7 (310.7 to 483.9)	33 (26 to 40)	3.3 (2.7 to 4)	145.1 (110.1 to 194.4)	20.1 (2.8 to 43.3)	149.9 (78.7 to 268.5)	2.8 (-25.3 to 50.8)

Province	Cause	Sex	1990				2019				1990-2019 percent change (%)			
			DALYs		Death		DALYs		Death		DALYs		Death	
			Number	Rate	Number	Rate	Number	Rate	Number	Rate	Number	Rate	Number	Rate
	Alcohol use disorders	Both	648 (482 to 826)	66.6 (49.7 to 84.3)	6 (3 to 8)	0.8 (0.5 to 1)	873 (635 to 1157)	41 (30.1 to 53.7)	6 (5 to 8)	0.3 (0.2 to 0.4)	34.7 (11.2 to 71.8)	-38.5 (-49.6 to -20.8)	-7.7 (-35.3 to 78.1)	-61.7 (-72.6 to -29.5)
		Female	181 (130 to 237)	34.8 (25.4 to 45.2)	1 (1 to 2)	0.3 (0.2 to 0.4)	268 (185 to 369)	25.4 (17.8 to 34.7)	1 (1 to 1)	0.1 (0.1 to 0.1)	47.6 (16.6 to 79.9)	-27 (-41.4 to -11.6)	-22.6 (-46.6 to 14.4)	-65 (-75 to -48.3)
		Male	466 (327 to 603)	98.1 (69.5 to 126.8)	5 (2 to 7)	1.2 (0.6 to 1.7)	605 (443 to 800)	56.6 (41.9 to 74.1)	4 (4 to 6)	0.5 (0.4 to 0.7)	29.7 (2.3 to 79.8)	-42.3 (-54.7 to -19)	-3.3 (-36.7 to 120.7)	-60 (-73.2 to -13.7)
	Drug use disorders	Both	2066 (1589 to 2666)	189.4 (149.1 to 240)	14 (11 to 18)	1.6 (1.3 to 2.1)	5095 (3970 to 6408)	240.5 (186.9 to 300.3)	37 (31 to 45)	1.8 (1.6 to 2.2)	146.5 (113.1 to 188.9)	26.9 (10.5 to 47.8)	164.2 (91.8 to 273)	11.6 (-16.7 to 54.5)
		Female	821 (586 to 1099)	150.3 (110.8 to 197)	6 (4 to 8)	1.3 (0.9 to 1.8)	1504 (1112 to 1944)	143 (106 to 184.9)	9 (7 to 11)	0.9 (0.7 to 1.1)	83.2 (48.9 to 125.8)	-4.9 (-21 to 17.2)	58.2 (0.8 to 159)	-32 (-53.7 to 9.7)
		Male	1246 (943 to 1611)	228.9 (175.8 to 294.3)	8 (6 to 12)	2 (1.4 to 2.7)	3591 (2821 to 4499)	336.1 (264.4 to 418.7)	28 (22 to 35)	2.8 (2.2 to 3.4)	188.3 (140 to 249.8)	46.8 (22.5 to 76.1)	234.2 (116.7 to 410.8)	41.1 (-5.2 to 113.5)
	Diabetes and kidney diseases	Both	7879 (6590 to 9166)	1103.1 (923 to 1294.5)	146 (121 to 179)	31.1 (25.9 to 40.8)	25958 (21882 to 30202)	1639.3 (1402.2 to 1898.3)	577 (518 to 640)	44.5 (40 to 49.4)	229.5 (185.8 to 279)	48.6 (27.9 to 67.5)	296.1 (212.7 to 386.2)	43 (7.4 to 74.2)
		Female	3876 (3153 to 4721)	1101.8 (895 to 1382.1)	69 (54 to 92)	29.8 (22.9 to 44.1)	13330 (10964 to 15786)	1647.6 (1366.7 to 1941)	287 (246 to 335)	43.6 (37.4 to 50.5)	243.9 (183.2 to 308.6)	49.5 (20.2 to 75.9)	317.8 (189 to 450.9)	46.4 (-7.2 to 94.8)
		Male	4003 (3267 to 4776)	1102.5 (900.2 to 1327.8)	77 (60 to 97)	32.7 (25.5 to 42.5)	12628 (10496 to 14862)	1631.6 (1375.7 to 1908.6)	290 (246 to 337)	45.7 (38.6 to 53.5)	215.5 (162 to 279.1)	48 (22.7 to 78.4)	276.7 (180.5 to 419.5)	39.5 (-0.5 to 90.3)
	Diabetes mellitus	Both	2785 (2142 to 3563)	442.3 (340.1 to 568.8)	32 (25 to 47)	6.7 (5.3 to 10.9)	16877 (13677 to 20695)	1054.1 (858.2 to 1284.9)	291 (252 to 334)	21.8 (19.1 to 25)	506 (397.1 to 586.4)	138.3 (89.5 to 170.5)	809.9 (428.6 to 1124.3)	225.2 (76.3 to 339.6)
		Female	1385 (1045 to 1802)	453.1 (341.3 to 598.9)	16 (11 to 29)	6.8 (4.8 to 14.1)	8885 (6980 to 11012)	1084.2 (859.2 to 1343.1)	149 (120 to 180)	21.9 (18 to 26.4)	541.5 (362.4 to 651.3)	139.3 (66.5 to 181.8)	838 (262.9 to 1276.5)	222 (20.5 to 377.6)
		Male	1400 (1087 to 1800)	432.5 (334.3 to 554.9)	16 (12 to 21)	6.7 (5.2 to 8.6)	7992 (6373 to 9850)	1023.1 (820.3 to 1253.6)	142 (116 to 170)	21.9 (18 to 26.1)	470.9 (393.9 to 562.5)	136.5 (106.2 to 175.3)	782.2 (543.2 to 1159)	225.9 (143.6 to 364.4)
	Chronic kidney disease	Both	5081 (4227 to 5889)	659.9 (561.8 to 787.2)	113 (94 to 138)	24.4 (20.1 to 33.4)	8852 (7830 to 9924)	572.1 (508.9 to 639.4)	279 (246 to 314)	22.3 (19.6 to 25.2)	74.2 (47.3 to 110.5)	-13.3 (-28.5 to 2.1)	146.1 (95.3 to 203.7)	-8.8 (-36.9 to 14.2)
		Female	2487 (1986 to 3088)	648.1 (527.1 to 855.5)	53 (41 to 73)	23 (17.2 to 36.4)	4367 (3731 to 5050)	554.3 (475.6 to 635.8)	136 (114 to 160)	21.4 (18 to 25.1)	75.6 (39.6 to 117.9)	-14.5 (-38.3 to 5.7)	157.6 (69.4 to 237.3)	-6.9 (-47 to 26.6)
		Male	2594 (2047 to 3159)	668.8 (538.5 to 814.2)	61 (46 to 77)	26 (20 to 34.8)	4485 (3828 to 5234)	591.3 (504.6 to 693.8)	143 (118 to 170)	23.2 (19 to 27.7)	72.9 (38.4 to 122.9)	-11.6 (-32.1 to 13.9)	136.1 (72.2 to 225.9)	-10.7 (-38.9 to 23.1)
	Acute glomerulonephritis	Both	13 (4 to 138)	0.9 (0.3 to 8)	0 (0 to 2)	0 (0 to 0.2)	229 (48 to 308)	13.1 (2.9 to 17.5)	7 (1 to 9)	0.5 (0.1 to 0.6)	1654 (-69.7 to 7294.3)	1320.9 (-68.7 to 4542.5)	2973.7 (-46.4 to 11191.1)	1706.7 (-58.6 to 5127)
		Female	4 (1 to 32)	0.6 (0.2 to 4)	0 (0 to 0)	0 (0 to 0.1)	78 (18 to 109)	9 (2.2 to 12.4)	2 (1 to 3)	0.3 (0.1 to 0.5)	1774.5 (-53.3 to 6345.5)	1381.9 (-55.8 to 4321.1)	3353.2 (-15.9 to 11147.2)	1958.7 (-38.3 to 5965.3)
		Male	9 (1 to 105)	1.2 (0.3 to 12.3)	0 (0 to 2)	0 (0 to 0.3)	151 (18 to 224)	17.2 (2.2 to 25.4)	4 (0 to 6)	0.6 (0.1 to 0.9)	1598 (-80.4 to 14324.9)	1297.5 (-79.6 to 8585.5)	2795.4 (-67.5 to 21015.5)	1533.9 (-75.5 to 8872.2)

Province	Cause	Sex	1990				2019				1990-2019 percent change (%)			
			DALYs		Death		DALYs		Death		DALYs		Death	
			Number	Rate	Number	Rate	Number	Rate	Number	Rate	Number	Rate	Number	Rate
	Skin and subcutaneous diseases	Both	5828 (3881 to 8460)	416.1 (281.5 to 614.8)	2 (1 to 2)	0.4 (0.3 to 0.6)	8046 (5369 to 12009)	426.1 (284.9 to 636.2)	6 (3 to 8)	0.5 (0.3 to 0.7)	38.1 (32.6 to 42.8)	2.4 (-0.6 to 5.1)	291.3 (89.2 to 508.2)	28.6 (-40.1 to 120.9)
		Female	3076 (2049 to 4451)	441.1 (299.9 to 648.2)	1 (1 to 1)	0.4 (0.2 to 0.7)	4229 (2815 to 6251)	449.3 (300.3 to 665.9)	3 (1 to 4)	0.5 (0.2 to 0.7)	37.5 (31 to 43.4)	1.9 (-1.8 to 5.4)	251.5 (23.2 to 537.2)	15.8 (-64.2 to 148.8)
		Male	2752 (1822 to 3990)	391.5 (263.3 to 582)	1 (0 to 1)	0.4 (0.2 to 0.6)	3816 (2530 to 5707)	403.4 (267.6 to 606)	3 (2 to 4)	0.6 (0.3 to 0.7)	38.7 (32.4 to 44.6)	3 (-1.3 to 6.7)	335.5 (129.1 to 649.1)	45.7 (-34.5 to 184.6)
	Dermatitis	Both	1336 (785 to 2129)	93.6 (57.1 to 144.3)	--	--	1847 (1117 to 2841)	94.1 (56.8 to 144.6)	--	--	38.2 (27.5 to 50.5)	0.6 (-4 to 5.7)	--	--
		Female	737 (437 to 1172)	103.8 (62.5 to 159.5)	--	--	1019 (614 to 1575)	104.5 (62.5 to 161.9)	--	--	38.4 (26.1 to 52.3)	0.7 (-5.2 to 7.2)	--	--
		Male	600 (347 to 959)	83.5 (50.6 to 128.7)	--	--	827 (500 to 1270)	83.9 (51.7 to 128.7)	--	--	38 (23.8 to 53.6)	0.5 (-6 to 8.1)	--	--
	Psoriasis	Both	500 (347 to 664)	47 (33.3 to 62.2)	--	--	689 (492 to 919)	34.7 (24.8 to 46.3)	--	--	37.8 (22.4 to 54.5)	-26.2 (-33.6 to -18.5)	--	--
		Female	261 (180 to 346)	48.7 (34.2 to 64.5)	--	--	357 (253 to 484)	35.9 (25.6 to 48.5)	--	--	36.5 (14.9 to 62.2)	-26.2 (-36 to -14.8)	--	--
		Male	239 (165 to 327)	45.3 (31.9 to 61.1)	--	--	333 (233 to 451)	33.5 (23.5 to 45.3)	--	--	39.3 (16.7 to 65.5)	-26.1 (-36.1 to -15.5)	--	--
	Bacterial skin diseases	Both	107 (74 to 161)	10.5 (7.2 to 14.8)	1 (1 to 2)	0.3 (0.2 to 0.5)	173 (123 to 246)	10 (7.3 to 13.9)	4 (2 to 5)	0.3 (0.2 to 0.4)	61.9 (31.1 to 102.3)	-4.9 (-25.6 to 30.6)	161 (76.6 to 344.7)	-13.2 (-44.3 to 86)
		Female	50 (34 to 77)	10.3 (6.6 to 14.6)	1 (0 to 1)	0.3 (0.1 to 0.5)	82 (54 to 120)	9.5 (6.4 to 13.8)	2 (1 to 3)	0.3 (0.2 to 0.5)	62.7 (21.3 to 132.4)	-7.6 (-37.6 to 58.9)	158.1 (34.6 to 507.1)	-14.5 (-57.6 to 168.9)
		Male	57 (37 to 85)	10.7 (7.3 to 14.9)	1 (0 to 1)	0.3 (0.2 to 0.5)	91 (58 to 132)	10.5 (6.9 to 14.8)	2 (1 to 2)	0.3 (0.2 to 0.4)	61.3 (24.5 to 113)	-2.1 (-27.9 to 35.7)	164.1 (69.8 to 360.8)	-11.3 (-48 to 76.4)
	Scabies	Both	370 (200 to 591)	25.2 (13.8 to 40.4)	--	--	482 (265 to 778)	25.1 (13.7 to 40.4)	--	--	30.4 (21.6 to 40.9)	-0.7 (-4.8 to 3.9)	--	--
		Female	185 (102 to 295)	25.3 (14.1 to 40.6)	--	--	239 (130 to 383)	25.1 (13.7 to 40.3)	--	--	29.2 (17.9 to 42.2)	-0.9 (-6.9 to 6.2)	--	--
		Male	185 (101 to 299)	25.2 (13.9 to 40.6)	--	--	243 (133 to 396)	25 (13.7 to 40.2)	--	--	31.5 (20.7 to 43.1)	-0.5 (-6.3 to 5.8)	--	--
	Fungal skin diseases	Both	183 (74 to 384)	19.2 (7.8 to 40.8)	--	--	324 (130 to 702)	18.6 (7.5 to 39.6)	--	--	76.9 (65.1 to 89.1)	-3 (-5 to -1.1)	--	--
		Female	83 (33 to 180)	17.9 (7.3 to 39.1)	--	--	152 (61 to 328)	17.4 (7 to 37.6)	--	--	82.1 (68.4 to 95.9)	-2.8 (-5.6 to -0.3)	--	--
		Male	100 (40 to 209)	20.5 (8.3 to 43.3)	--	--	172 (69 to 370)	19.8 (8 to 42.4)	--	--	72.5 (60.2 to 85)	-3.3 (-5.8 to -0.9)	--	--
	Viral skin diseases	Both	933 (591 to 1403)	51.2 (32.6 to 76.5)	--	--	987 (622 to 1479)	51.4 (32.5 to 77.1)	--	--	5.8 (1 to 10.8)	0.3 (-3.8 to 4.6)	--	--
		Female	457 (287 to 689)	50.8 (32.1 to 75.9)	--	--	483 (306 to 724)	51 (32.4 to 76.5)	--	--	5.7 (-0.9 to 12.4)	0.3 (-5.5 to 6.3)	--	--
		Male	475 (301 to 704)	51.6 (33 to 76.5)	--	--	503 (318 to 741)	51.8 (32.8 to 76.2)	--	--	5.8 (-0.7 to 12.9)	0.2 (-5.4 to 6.2)	--	--
	Acne vulgaris	Both	869 (524 to 1362)	51.2 (30.9 to 79.8)	--	--	1220 (737 to 1934)	69.8 (42.1 to 111)	--	--	40.4 (29.1 to 52.7)	36.2 (25.6 to 46.9)	--	--
		Female	420 (254 to 666)	49.9 (30.2 to 78.7)	--	--	589 (359 to 927)	67.8 (41.1 to 107.2)	--	--	40 (24.5 to 58.9)	35.9 (21 to 52.8)	--	--

Province	Cause	Sex	1990				2019				1990-2019 percent change (%)			
			DALYs		Death		DALYs		Death		DALYs		Death	
			Number	Rate	Number	Rate	Number	Rate	Number	Rate	Number	Rate	Number	Rate
		Male	449 (265 to 697)	52.5 (31.3 to 81.9)	--	--	632 (372 to 1005)	71.6 (42 to 114.5)	--	--	40.8 (26.3 to 57.5)	36.4 (22.9 to 51.6)	--	--
	Alopecia areata	Both	73 (47 to 109)	6.4 (4.1 to 9.4)	--	--	133 (84 to 201)	6.4 (4 to 9.6)	--	--	81.9 (61.9 to 104.6)	0.2 (-9.9 to 10.5)	--	--
		Female	48 (30 to 71)	8.3 (5.3 to 12.3)	--	--	86 (54 to 129)	8.3 (5.2 to 12.3)	--	--	82 (57 to 109)	0.1 (-12.7 to 13.9)	--	--
		Male	26 (16 to 39)	4.5 (2.7 to 6.7)	--	--	47 (29 to 70)	4.5 (2.8 to 6.7)	--	--	81.8 (49.6 to 121.8)	0.2 (-16.4 to 18.8)	--	--
	Pruritus	Both	110 (51 to 200)	10.1 (4.8 to 18.3)	--	--	201 (96 to 366)	10.5 (5 to 18.8)	--	--	83.4 (68.7 to 99.8)	4.2 (-0.8 to 9.5)	--	--
		Female	59 (28 to 110)	11 (5.2 to 20.2)	--	--	110 (53 to 199)	11.5 (5.4 to 20.6)	--	--	84.6 (67 to 105.2)	3.7 (-3.5 to 11.5)	--	--
		Male	50 (23 to 91)	9.2 (4.3 to 16.5)	--	--	92 (44 to 165)	9.6 (4.6 to 17.2)	--	--	82 (64.9 to 101.2)	4.5 (-2.4 to 12.5)	--	--
	Urticaria	Both	919 (594 to 1345)	58.2 (38.1 to 83.8)	--	--	1140 (739 to 1659)	58.3 (37.7 to 84.6)	--	--	24 (14.2 to 35.3)	0.1 (-5.5 to 5.5)	--	--
		Female	562 (363 to 823)	70.9 (46.3 to 102.4)	--	--	686 (444 to 992)	71.1 (45.9 to 103.1)	--	--	22.2 (11.4 to 34.1)	0.3 (-6.4 to 7.7)	--	--
		Male	358 (229 to 525)	45.8 (29.5 to 66.1)	--	--	454 (295 to 664)	45.8 (29.8 to 67.1)	--	--	26.9 (12.6 to 43)	0.1 (-8.7 to 9.5)	--	--
	Decubitus ulcer	Both	5 (3 to 9)	1.2 (0.7 to 2.4)	0 (0 to 0)	0.1 (0 to 0.2)	45 (16 to 61)	3.6 (1.4 to 4.9)	2 (1 to 3)	0.2 (0.1 to 0.3)	795.2 (87.2 to 1431.6)	201.7 (-41.8 to 452.4)	1459.7 (76.7 to 4005.5)	250.7 (-57.3 to 830.8)
		Female	3 (2 to 5)	1.2 (0.7 to 2.7)	0 (0 to 0)	0.1 (0 to 0.2)	21 (7 to 31)	3.3 (1.1 to 5)	1 (0 to 2)	0.2 (0 to 0.3)	719.8 (38.4 to 1680.7)	165.2 (-59.7 to 519.3)	1022.4 (-11.9 to 3935.7)	188.6 (-75.6 to 914.9)
		Male	2 (2 to 5)	1.1 (0.7 to 2.4)	0 (0 to 0)	0 (0 to 0.2)	24 (8 to 33)	3.8 (1.3 to 5.4)	1 (0 to 2)	0.2 (0.1 to 0.3)	872.3 (123.4 to 1513.5)	251.4 (-25.8 to 506.1)	2301.1 (167.2 to 6318.2)	371.8 (-45 to 1244.2)
	Other skin and subcutaneous diseases	Both	422 (201 to 774)	42.2 (20.2 to 76.4)	0 (0 to 0)	0 (0 to 0)	804 (383 to 1452)	43.7 (21 to 79.2)	0 (0 to 0)	0 (0 to 0)	90.4 (85.2 to 96.6)	3.5 (1.2 to 6)	535.4 (191.2 to 1357.1)	34.9 (-38.8 to 263.4)
		Female	211 (100 to 390)	42.7 (20.5 to 78)	0 (0 to 0)	0 (0 to 0.1)	406 (193 to 747)	43.8 (21 to 80)	0 (0 to 0)	0 (0 to 0)	92.8 (85.8 to 100.8)	2.5 (-0.7 to 5.8)	49.8 (-55.8 to 456.4)	-63.8 (-89.4 to 51.6)
		Male	212 (101 to 388)	41.7 (19.9 to 75.1)	0 (0 to 0)	0 (0 to 0)	398 (192 to 720)	43.6 (21.2 to 79.3)	0 (0 to 0)	0 (0 to 0.1)	88 (80.6 to 96.6)	4.6 (1 to 8.6)	1245 (511.3 to 2878.9)	203.6 (32.8 to 717)
	Sense organ diseases	Both	6959 (4921 to 9427)	1042.3 (746.6 to 1389.2)	--	--	12410 (8747 to 17017)	812.4 (576.7 to 1102.3)	--	--	78.3 (68.1 to 87.8)	-22.1 (-25.1 to -18.8)	--	--
		Female	3397 (2389 to 4608)	1044 (753.8 to 1390.7)	--	--	6298 (4433 to 8602)	813.9 (581.9 to 1101)	--	--	85.4 (74.6 to 95.9)	-22 (-25.2 to -18.5)	--	--
		Male	3562 (2506 to 4855)	1039.2 (741.1 to 1396.3)	--	--	6112 (4290 to 8399)	810.9 (572.7 to 1107.7)	--	--	71.6 (60.4 to 81.7)	-22 (-25.3 to -18.3)	--	--
	Blindness and vision loss	Both	3245 (2291 to 4409)	512.6 (367.6 to 686.5)	--	--	5545 (3951 to 7517)	376.5 (269.2 to 506.9)	--	--	70.9 (63.9 to 78.1)	-26.6 (-29.5 to -23.1)	--	--
		Female	1664 (1178 to 2243)	539.6 (387.7 to 722.1)	--	--	2982 (2119 to 4046)	397.2 (284.9 to 536.6)	--	--	79.3 (70.7 to 88.6)	-26.4 (-29.7 to -22.6)	--	--

Province	Cause	Sex	1990				2019				1990-2019 percent change (%)			
			DALYs		Death		DALYs		Death		DALYs		Death	
			Number	Rate	Number	Rate	Number	Rate	Number	Rate	Number	Rate	Number	Rate
		Male	1581 (1112 to 2161)	486.6 (349.8 to 656.8)	--	--	2562 (1824 to 3477)	354.4 (253.6 to 477.5)	--	--	62 (54 to 70.8)	-27.2 (-30.6 to -23.3)	--	--
	Age-related and other hearing loss	Both	3353 (2318 to 4672)	488 (346.9 to 673.9)	--	--	6108 (4231 to 8576)	392.6 (274.6 to 546.7)	--	--	82.2 (63.6 to 100.3)	-19.5 (-23.3 to -15.8)	--	--
		Female	1542 (1065 to 2132)	459.3 (326.3 to 633)	--	--	2903 (2023 to 4096)	369.9 (258.9 to 515.6)	--	--	88.2 (67.7 to 108.8)	-19.5 (-23.8 to -14.9)	--	--
		Male	1811 (1246 to 2544)	514.4 (362.2 to 712)	--	--	3205 (2194 to 4491)	416.8 (290 to 578.1)	--	--	77 (58 to 94.4)	-19 (-23.7 to -14.3)	--	--
	Other sense organ diseases	Both	361 (218 to 548)	41.6 (24.8 to 64.5)	--	--	757 (442 to 1171)	43.3 (25.9 to 66.8)	--	--	109.9 (90 to 129.6)	3.9 (-1.4 to 9.1)	--	--
		Female	191 (114 to 290)	45.2 (26.8 to 70.2)	--	--	413 (241 to 642)	46.7 (27.5 to 71.9)	--	--	116.4 (92.5 to 141.4)	3.4 (-3.7 to 11.2)	--	--
		Male	170 (100 to 263)	38.2 (22.9 to 59.1)	--	--	344 (202 to 532)	39.7 (23.5 to 61.7)	--	--	102.7 (80.7 to 125.4)	3.9 (-2.7 to 12)	--	--
	Musculoskeletal disorders	Both	15459 (10999 to 20722)	1896.5 (1355.9 to 2512)	2 (2 to 5)	0.4 (0.3 to 0.7)	37878 (27067 to 50556)	1960.1 (1404 to 2595.7)	11 (9 to 14)	0.7 (0.6 to 0.9)	145 (133.8 to 157.2)	3.4 (0.3 to 6.7)	383.3 (156.1 to 666.3)	91.6 (-0.3 to 199)
		Female	8269 (5887 to 11083)	2063.5 (1477.7 to 2735.7)	2 (1 to 4)	0.5 (0.3 to 1.1)	21120 (15205 to 27829)	2172.6 (1563.5 to 2846.1)	8 (5 to 10)	0.9 (0.7 to 1.2)	155.4 (141.5 to 169.1)	5.3 (1.5 to 9.1)	397.8 (110 to 796.2)	99.3 (-22.8 to 273)
		Male	7190 (5054 to 9613)	1733 (1221.7 to 2298.9)	1 (1 to 2)	0.3 (0.2 to 0.5)	16758 (11868 to 22352)	1743.1 (1234.3 to 2308.6)	4 (3 to 4)	0.5 (0.4 to 0.6)	133.1 (119.7 to 148.2)	0.6 (-3.9 to 5.2)	355.2 (134.6 to 590.9)	80.2 (2.1 to 161.6)
	Rheumatoid arthritis	Both	93 (63 to 129)	11.5 (7.9 to 15.9)	0 (0 to 1)	0.1 (0 to 0.2)	257 (179 to 343)	13.2 (9.3 to 17.4)	1 (1 to 2)	0.1 (0.1 to 0.1)	175.7 (121.2 to 228.7)	15 (-9.5 to 36.3)	334.9 (31.4 to 948.3)	39 (-58.8 to 234.2)
		Female	65 (43 to 92)	16.2 (10.9 to 23.4)	0 (0 to 1)	0.1 (0 to 0.3)	182 (124 to 245)	18.4 (12.8 to 24.4)	1 (0 to 1)	0.1 (0.1 to 0.2)	178.7 (103.5 to 251.8)	13.2 (-19.3 to 42.6)	286.8 (-12.1 to 1140.6)	19.6 (-73.5 to 284.3)
		Male	28 (18 to 39)	6.9 (4.5 to 9.6)	0 (0 to 0)	0 (0 to 0)	75 (52 to 102)	7.9 (5.6 to 10.7)	0 (0 to 1)	0.1 (0 to 0.1)	168.8 (129 to 221.1)	15.3 (-1.7 to 37.8)	500.5 (253.6 to 976.5)	107.4 (23.8 to 273.6)
	Osteoarthritis	Both	1014 (514 to 1991)	177.4 (90.7 to 349.6)	--	--	3048 (1539 to 5954)	190.9 (96.4 to 373.5)	--	--	200.5 (189.9 to 212.2)	7.6 (4.1 to 11.4)	--	--
		Female	547 (276 to 1080)	201.3 (102 to 397.9)	--	--	1767 (892 to 3476)	216.3 (109 to 422.7)	--	--	223 (207.3 to 239.2)	7.5 (2.7 to 12.4)	--	--
		Male	467 (236 to 933)	154.9 (78.8 to 309.8)	--	--	1281 (644 to 2533)	164 (82.6 to 322)	--	--	174.2 (159.9 to 189.7)	5.8 (1 to 11.4)	--	--
	Low back pain	Both	8950 (6212 to 12031)	1028 (711.7 to 1382.6)	--	--	17857 (12514 to 24073)	924.2 (653.9 to 1233.3)	--	--	99.5 (86.5 to 112.8)	-10.1 (-13.9 to -6.1)	--	--
		Female	4220 (2946 to 5731)	977.9 (682.9 to 1314.4)	--	--	8822 (6149 to 11954)	916.5 (643.4 to 1222.8)	--	--	109.1 (91.8 to 127.9)	-6.3 (-11.8 to -0.7)	--	--
		Male	4730 (3260 to 6365)	1074.6 (743.2 to 1438.8)	--	--	9035 (6247 to 12180)	932.4 (660.6 to 1247.7)	--	--	91 (76.4 to 107)	-13.2 (-18.5 to -7.6)	--	--

Province	Cause	Sex	1990				2019				1990-2019 percent change (%)			
			DALYs		Death		DALYs		Death		DALYs		Death	
			Number	Rate	Number	Rate	Number	Rate	Number	Rate	Number	Rate	Number	Rate
	Neck pain	Both	3244 (2117 to 4736)	404 (267 to 583.9)	--	--	8134 (5307 to 11855)	406.9 (266.7 to 587.6)	--	--	150.8 (135.7 to 164)	0.7 (-1.4 to 2.9)	--	--
		Female	1943 (1276 to 2839)	493 (327.6 to 718.3)	--	--	4987 (3276 to 7368)	493.6 (325.5 to 720)	--	--	156.7 (139.7 to 171.4)	0.1 (-2.5 to 2.8)	--	--
		Male	1301 (843 to 1908)	318.2 (208.8 to 462.6)	--	--	3148 (2024 to 4673)	318.5 (207.3 to 465.5)	--	--	142 (125.4 to 157.3)	0.1 (-3.1 to 3.6)	--	--
	Gout	Both	90 (55 to 133)	14.4 (8.9 to 21.3)	--	--	260 (160 to 378)	15.2 (9.6 to 22.2)	--	--	188 (149 to 237.3)	5.9 (-6.8 to 22.2)	--	--
		Female	19 (11 to 27)	6.3 (3.8 to 9.2)	--	--	60 (37 to 88)	7 (4.3 to 10.2)	--	--	220.9 (150.3 to 309)	11.4 (-11.1 to 40.7)	--	--
		Male	72 (44 to 106)	22.4 (13.8 to 33.3)	--	--	200 (123 to 294)	23.9 (14.9 to 35.2)	--	--	179.5 (133.6 to 235)	6.8 (-8.4 to 24.5)	--	--
	Other musculoskeletal disorders	Both	2068 (1366 to 2929)	261.1 (172.6 to 369.4)	2 (1 to 4)	0.3 (0.2 to 0.6)	8322 (5700 to 11647)	409.6 (283.2 to 571.6)	10 (8 to 13)	0.6 (0.5 to 0.8)	302.5 (266.1 to 347.5)	56.9 (43.2 to 73.4)	389.7 (173.5 to 696.6)	101.7 (13.5 to 219.3)
		Female	1476 (1006 to 2045)	368.8 (251.5 to 512.8)	1 (1 to 3)	0.4 (0.2 to 0.8)	5302 (3708 to 7298)	520.8 (363.4 to 710)	7 (5 to 9)	0.8 (0.6 to 1.1)	259.3 (224.1 to 300.8)	41.2 (28.7 to 56.8)	415.5 (140.3 to 882.5)	119.2 (-0.7 to 332)
		Male	592 (353 to 916)	156 (92.6 to 238)	1 (1 to 2)	0.3 (0.2 to 0.5)	3020 (1992 to 4400)	296.5 (193.1 to 427.7)	3 (3 to 4)	0.5 (0.4 to 0.6)	410.4 (321.1 to 538.7)	90 (58.9 to 132.6)	342.9 (120 to 573.2)	77.4 (-2.8 to 159)
	Other non-communicable diseases	Both	91681 (61780 to 122623)	4968.3 (3499.8 to 6475.4)	883 (552 to 1233)	42.1 (26.7 to 58.5)	41611 (32308 to 52454)	2190.7 (1718.9 to 2739)	223 (174 to 278)	13.6 (10.5 to 16.9)	-54.6 (-69.2 to -27.8)	-55.9 (-68.5 to -35.7)	-74.7 (-84.2 to -53.5)	-67.7 (-79.9 to -41.7)
		Female	47878 (30447 to 64328)	5586.9 (3828.7 to 7256.4)	429 (244 to 608)	41.9 (24.6 to 58.7)	26518 (20279 to 34480)	2693.6 (2083 to 3444.1)	100 (78 to 125)	12.3 (9.6 to 15.3)	-44.6 (-62.6 to -10.4)	-51.8 (-64.9 to -30.2)	-76.7 (-85.6 to -54.9)	-70.6 (-82 to -45.2)
		Male	43802 (24311 to 62646)	4345.6 (2584.9 to 6043.2)	454 (233 to 663)	42.3 (22.5 to 61.3)	15094 (11937 to 19049)	1687.4 (1338.9 to 2135.6)	123 (91 to 157)	14.9 (10.8 to 18.8)	-65.5 (-78.1 to -31.5)	-61.2 (-74.3 to -28.2)	-72.9 (-84.3 to -40.5)	-64.8 (-79.8 to -26.3)
	Congenital birth defects	Both	72995 (44750 to 103210)	3373.9 (2086.2 to 4750.3)	811 (486 to 1152)	37.4 (22.6 to 53)	14195 (11052 to 18400)	811 (629 to 1059.5)	145 (113 to 192)	8.4 (6.4 to 11.2)	-80.6 (-87.9 to -62.4)	-76 (-85.1 to -53.6)	-82.1 (-89.1 to -64.5)	-77.7 (-86.4 to -55.7)
		Female	35260 (18538 to 51271)	3326.5 (1761 to 4825.8)	394 (204 to 575)	37.1 (19.5 to 54.1)	6267 (4757 to 8302)	730.2 (547.9 to 975)	65 (48 to 87)	7.6 (5.6 to 10.3)	-82.2 (-89.5 to -59)	-78 (-87.1 to -49.6)	-83.6 (-90.5 to -60.6)	-79.5 (-88.1 to -50.8)
		Male	37735 (18374 to 55429)	3417.7 (1682.8 to 5007.6)	417 (196 to 618)	37.7 (18 to 55.7)	7929 (6092 to 10638)	888.2 (678.5 to 1200.7)	80 (61 to 111)	9.1 (6.8 to 12.6)	-79 (-87.4 to -44.3)	-74 (-84.5 to -31.6)	-80.7 (-88.8 to -46.5)	-75.9 (-86.1 to -33)
	Urinary diseases and male infertility	Both	248 (175 to 352)	27.1 (18.5 to 39.5)	1 (1 to 3)	0.2 (0.1 to 0.8)	610 (449 to 808)	35.6 (26.9 to 46.4)	8 (6 to 9)	0.7 (0.5 to 0.8)	146 (81.7 to 186.3)	31.1 (-10.2 to 58.3)	555.5 (128 to 970.4)	223.1 (-25.5 to 559.9)
		Female	87 (64 to 121)	15.1 (10.5 to 27.3)	1 (0 to 2)	0.2 (0.1 to 0.9)	196 (149 to 243)	22.1 (17.3 to 26.9)	4 (3 to 5)	0.6 (0.5 to 0.8)	125.8 (46.2 to 193.1)	46.9 (-23.7 to 102.8)	451.5 (99.3 to 936.8)	161.2 (-20.7 to 525.3)

Province	Cause	Sex	1990				2019				1990-2019 percent change (%)			
			DALYs		Death		DALYs		Death		DALYs		Death	
			Number	Rate	Number	Rate	Number	Rate	Number	Rate	Number	Rate	Number	Rate
		Male	161 (107 to 244)	38.8 (25.4 to 57.9)	1 (0 to 1)	0.2 (0.1 to 0.7)	413 (291 to 589)	49.7 (35.9 to 68.6)	4 (3 to 5)	0.7 (0.5 to 0.9)	156.9 (95.6 to 201.7)	28.1 (-6.4 to 50.6)	694.8 (134.2 to 1181.8)	323.1 (-16.6 to 716.3)
	Gynecological diseases	Female	5429 (3687 to 7580)	1111 (761.5 to 1537.9)	1 (0 to 1)	0.2 (0 to 0.4)	11690 (8020 to 16315)	1052.1 (721.9 to 1462.3)	1 (0 to 1)	0.1 (0 to 0.1)	115.3 (101.8 to 127)	-5.3 (-8.5 to -2.3)	-4.7 (-52.9 to 137.9)	-63.1 (-82 to -9.2)
	Hemoglobinopathies and hemolytic anemias	Both	3766 (2839 to 4901)	235.2 (176.1 to 309.3)	24 (18 to 31)	1.4 (1.1 to 1.8)	1976 (1478 to 2740)	104 (78.8 to 142.2)	13 (12 to 15)	0.8 (0.7 to 0.9)	-47.5 (-59 to -31.9)	-55.8 (-64.1 to -44.7)	-43.8 (-58.4 to -21.4)	-46.8 (-58.9 to -30.2)
		Female	2259 (1581 to 3056)	298 (209.6 to 400.9)	13 (8 to 19)	1.6 (1.1 to 2.1)	1167 (808 to 1691)	121.9 (86 to 174.1)	6 (5 to 7)	0.7 (0.6 to 0.8)	-48.4 (-63 to -26.8)	-59.1 (-69.3 to -45.9)	-51.3 (-67.6 to -20.2)	-54.6 (-67.9 to -32.2)
		Male	1507 (1082 to 2017)	171.3 (126.5 to 229.1)	11 (8 to 15)	1.3 (1 to 1.7)	810 (617 to 1116)	86.1 (66 to 118.1)	7 (6 to 9)	0.8 (0.7 to 1)	-46.3 (-60.6 to -25)	-49.7 (-62 to -31.9)	-35 (-54.2 to -1.2)	-37.6 (-53.8 to -13.7)
	Endocrine, metabolic, blood, and immune disorders	Both	4179 (2946 to 6134)	344.4 (243 to 477.6)	18 (11 to 39)	1.6 (1.1 to 3.2)	7145 (5022 to 9435)	382.9 (268.6 to 501.7)	52 (24 to 69)	3.6 (1.7 to 4.7)	71 (5 to 120.3)	11.2 (-23.1 to 37.4)	190.5 (-21.6 to 464.4)	119.9 (-33 to 311.8)
		Female	2449 (1642 to 3648)	421.5 (291.9 to 588.1)	8 (4 to 19)	1.5 (0.9 to 3.7)	4243 (3021 to 5722)	445.6 (317.3 to 594.6)	23 (10 to 30)	3.1 (1.4 to 4.1)	73.2 (11.7 to 113.1)	5.7 (-23.4 to 26.8)	194.4 (-30.5 to 526.4)	100.8 (-46.5 to 325)
		Male	1730 (1183 to 2784)	268.7 (191 to 387.1)	10 (6 to 22)	1.7 (1.1 to 3.2)	2902 (1839 to 3873)	320.8 (203 to 424.1)	29 (11 to 42)	4.1 (1.5 to 6)	67.7 (-8.7 to 142)	19.4 (-25 to 63.8)	187.5 (-18.2 to 498)	136.3 (-20.3 to 357.5)
	Oral disorders	Both	2543 (1497 to 4019)	316.4 (186.9 to 494.2)	--	--	5671 (3360 to 9159)	312.6 (185.7 to 492.6)	--	--	123 (113 to 133.5)	-1.2 (-3.4 to 1.2)	--	--
		Female	1230 (722 to 1930)	305.5 (182 to 481.8)	--	--	2799 (1646 to 4570)	302.8 (179.7 to 480.9)	--	--	127.6 (115.4 to 139.8)	-0.9 (-4.1 to 2.1)	--	--
		Male	1313 (775 to 2094)	327.1 (195.4 to 511.8)	--	--	2872 (1707 to 4589)	323.2 (193 to 505.4)	--	--	118.7 (108.5 to 129.9)	-1.2 (-4.5 to 2)	--	--
	Sudden infant death syndrome	Both	2521 (406 to 8348)	115.7 (18.7 to 383.1)	29 (5 to 94)	1.3 (0.2 to 4.3)	324 (159 to 655)	19 (9.4 to 38.5)	4 (2 to 7)	0.2 (0.1 to 0.4)	-87.2 (-95.9 to -16.4)	-83.6 (-94.7 to 7)	-87.2 (-95.9 to -16.4)	-83.6 (-94.7 to 7)
		Female	1165 (199 to 4064)	109.2 (18.7 to 381)	13 (2 to 46)	1.2 (0.2 to 4.3)	155 (72 to 326)	18.7 (8.7 to 39.3)	2 (1 to 4)	0.2 (0.1 to 0.4)	-86.7 (-95.7 to -18.2)	-82.9 (-94.4 to 5.1)	-86.7 (-95.7 to -18.2)	-82.9 (-94.4 to 5.1)
		Male	1356 (179 to 4569)	121.9 (16.1 to 410.6)	15 (2 to 52)	1.4 (0.2 to 4.6)	168 (72 to 366)	19.3 (8.3 to 42)	2 (1 to 4)	0.2 (0.1 to 0.5)	-87.6 (-96 to -16.1)	-84.2 (-95 to 7.1)	-87.6 (-96.1 to -16.1)	-84.2 (-95 to 7.1)
	**Injuries**	Both	104633 (85495 to 124580)	7305.6 (6128.6 to 8556.7)	1455 (1192 to 1750)	121 (101.2 to 143.4)	70456 (63095 to 78843)	3651.7 (3271.9 to 4073.2)	1175 (1045 to 1345)	64.6 (57.2 to 74.1)	-32.7 (-45.7 to -14.1)	-50 (-59 to -38)	-19.2 (-35.6 to 5.5)	-46.6 (-57.2 to -31.6)
		Female	34961 (27625 to 44793)	4604.1 (3711.3 to 5803.2)	462 (367 to 598)	71.4 (57.4 to 90.2)	17348 (15418 to 19759)	1795.4 (1595.8 to 2029.4)	276 (242 to 324)	30.7 (26.9 to 35.7)	-50.4 (-62 to -34.4)	-61 (-70.1 to -50.7)	-40.1 (-56 to -20.1)	-57 (-67.4 to -43.5)
		Male	69672 (56096 to 83729)	9977.3 (8127.5 to 11921.2)	994 (791 to 1213)	170.3 (133.6 to 207.3)	53108 (46940 to 60334)	5484.7 (4848.2 to 6234.2)	899 (779 to 1053)	98.5 (85.1 to 116.5)	-23.8 (-39.1 to 0.5)	-45 (-55.5 to -29.5)	-9.5 (-29.2 to 24.4)	-42.1 (-54.8 to -20.5)
	Transport injuries	Both	57059 (44584 to 69411)	3974.3 (3150.3 to 4737.4)	855 (669 to 1035)	72.3 (55.6 to 87)	36420 (31853 to 41807)	1907.7 (1669.4 to 2187.8)	682 (586 to 805)	37.6 (32.3 to 44.7)	-36.2 (-49.6 to -12.7)	-52 (-61.5 to -35.7)	-20.2 (-37.5 to 12.2)	-47.9 (-58.9 to -27.7)

Province	Cause	Sex	1990				2019				1990-2019 percent change (%)			
			DALYs		Death		DALYs		Death		DALYs		Death	
			Number	Rate	Number	Rate	Number	Rate	Number	Rate	Number	Rate	Number	Rate
		Female	17336 (12984 to 22818)	2229.3 (1750.2 to 2844.4)	238 (182 to 312)	36.6 (28.7 to 46.4)	7410 (6321 to 8545)	762.3 (651.5 to 872.1)	139 (115 to 165)	15.1 (12.6 to 17.7)	-57.3 (-69.1 to -40.3)	-65.8 (-74.7 to -55.1)	-41.7 (-58.2 to -20.2)	-58.7 (-68.9 to -44.6)
		Male	39723 (29113 to 48587)	5700.2 (4228.6 to 6962.2)	617 (449 to 758)	107.7 (77.7 to 133.1)	29010 (24948 to 34098)	3035.5 (2611.8 to 3565.1)	543 (459 to 664)	60.2 (50.7 to 74.9)	-27 (-43.1 to 5.5)	-46.7 (-58.2 to -24.5)	-11.9 (-32.2 to 33)	-44.1 (-57.1 to -16.2)
	Road injuries	Both	55746 (43751 to 67763)	3875.4 (3064.2 to 4622.3)	836 (657 to 1011)	70.5 (54.6 to 84.8)	35042 (30522 to 40217)	1836.4 (1601 to 2106.2)	657 (564 to 777)	36.3 (31.2 to 43.2)	-37.1 (-50.6 to -13.5)	-52.6 (-62 to -36.4)	-21.3 (-38.3 to 10.6)	-48.6 (-59.4 to -28.6)
		Female	17029 (12762 to 22457)	2187.4 (1715.9 to 2796.7)	235 (179 to 307)	36 (28.2 to 45.4)	7161 (6088 to 8263)	736.6 (628.9 to 843.3)	135 (112 to 160)	14.7 (12.2 to 17.2)	-57.9 (-69.5 to -41.3)	-66.3 (-75 to -55.7)	-42.4 (-58.7 to -21.2)	-59.2 (-69.3 to -45.4)
		Male	38717 (28387 to 47338)	5545.3 (4112.7 to 6772.9)	601 (437 to 740)	104.8 (75.6 to 129.6)	27881 (23949 to 32734)	2919 (2508.9 to 3430.3)	522 (440 to 637)	57.8 (48.6 to 71.3)	-28 (-43.8 to 4.7)	-47.4 (-58.7 to -25.4)	-13.1 (-33.2 to 29.8)	-44.8 (-57.8 to -17.8)
	Other transport injuries	Both	1313 (891 to 1748)	98.9 (67.5 to 127.8)	19 (12 to 26)	1.8 (1.1 to 2.3)	1379 (1132 to 1720)	71.3 (58.3 to 88.4)	25 (20 to 32)	1.4 (1.1 to 1.8)	5 (-24.9 to 65.7)	-27.9 (-47.6 to 10.6)	29.2 (-9.3 to 112.6)	-21.6 (-44.9 to 30)
		Female	307 (202 to 460)	41.9 (29.4 to 58.9)	4 (2 to 6)	0.6 (0.4 to 0.9)	249 (200 to 305)	25.7 (20.8 to 31)	4 (3 to 5)	0.4 (0.3 to 0.5)	-18.8 (-47.3 to 27)	-38.6 (-58.1 to -9.6)	2.2 (-36.4 to 70.6)	-28.2 (-54.9 to 17.1)
		Male	1006 (625 to 1393)	154.9 (99.8 to 205.6)	15 (10 to 21)	2.9 (1.8 to 3.9)	1129 (890 to 1431)	116.5 (92 to 149.1)	21 (16 to 28)	2.3 (1.8 to 3.2)	12.2 (-24.9 to 85.9)	-24.8 (-48 to 19.8)	36 (-10.3 to 129.4)	-19.6 (-47.9 to 41)
	Unintentional injuries	Both	34812 (28064 to 43059)	2306.2 (1912.2 to 2734.9)	433 (351 to 535)	33.8 (28.3 to 40.6)	20544 (17930 to 23723)	1071.3 (932.4 to 1234.9)	286 (252 to 326)	16.5 (14.5 to 19)	-41 (-53.3 to -25.7)	-53.5 (-62.1 to -43.2)	-34 (-49.4 to -13.4)	-51.2 (-61.8 to -38.1)
		Female	12452 (9064 to 16380)	1576.7 (1209.5 to 2019.1)	148 (108 to 198)	22.1 (16.7 to 28.3)	5883 (4920 to 6978)	626 (524 to 740.8)	75 (62 to 91)	9.1 (7.5 to 11.3)	-52.8 (-65.9 to -34.2)	-60.3 (-70.4 to -47.7)	-49.5 (-65.4 to -25.1)	-58.8 (-71.1 to -42)
		Male	22360 (17621 to 28103)	3024.6 (2437 to 3705.5)	285 (222 to 362)	45.6 (35.8 to 57)	14661 (12709 to 16993)	1513.4 (1307.1 to 1758)	211 (183 to 247)	23.9 (20.6 to 27.8)	-34.4 (-48.6 to -16.2)	-50 (-59.5 to -37.5)	-25.9 (-44.4 to 0.2)	-47.5 (-60.5 to -30.5)
	Falls	Both	5006 (3814 to 6334)	440.6 (345.2 to 544.2)	52 (37 to 70)	5.9 (4.5 to 8.2)	5548 (4494 to 6853)	298.3 (242.7 to 366.6)	63 (53 to 80)	4.2 (3.5 to 5.2)	10.8 (-13.2 to 39.7)	-32.3 (-42.5 to -21.2)	21.1 (-10 to 69.9)	-29.7 (-44.2 to -5.3)
		Female	1369 (936 to 1962)	237.1 (185 to 305.5)	13 (9 to 20)	3.2 (2.2 to 5.3)	1324 (1027 to 1702)	151.4 (118.8 to 191.4)	13 (10 to 19)	2 (1.6 to 3)	-3.2 (-32.1 to 41.6)	-36.1 (-47.8 to -24.2)	-0.1 (-32.6 to 56.5)	-37.3 (-54.6 to -7.5)
		Male	3638 (2487 to 4795)	639.4 (485.1 to 800.6)	39 (24 to 55)	8.7 (6.3 to 11.5)	4224 (3419 to 5244)	446.4 (363.8 to 555.4)	50 (41 to 64)	6.3 (5.2 to 8)	16.1 (-13.2 to 62.9)	-30.2 (-42 to -12.6)	28.1 (-11.5 to 109)	-26.8 (-44.9 to 1.9)
	Drowning	Both	7224 (4449 to 10557)	369.1 (238.8 to 523.1)	92 (58 to 131)	5.1 (3.4 to 7)	1266 (1060 to 1571)	66.7 (55.4 to 84)	21 (18 to 26)	1.1 (1 to 1.4)	-82.5 (-88.8 to -67.7)	-81.9 (-88.2 to -67.2)	-77 (-84.9 to -58.3)	-77.8 (-84.9 to -60.9)
		Female	2058 (1254 to 3306)	199 (125.4 to 312)	25 (16 to 40)	2.5 (1.6 to 3.9)	194 (160 to 239)	20.3 (16.7 to 25.1)	3 (3 to 4)	0.3 (0.3 to 0.4)	-90.6 (-94.5 to -82.8)	-89.8 (-94 to -81.9)	-87.6 (-92.6 to -78.3)	-86.8 (-91.9 to -78)
		Male	5166 (2578 to 7857)	536.9 (279.9 to 785.2)	67 (35 to 100)	7.7 (4.2 to 10.8)	1072 (875 to 1373)	111.9 (90.6 to 143.8)	18 (15 to 23)	1.9 (1.6 to 2.5)	-79.3 (-87.3 to -53.1)	-79.2 (-86.7 to -54.3)	-73.1 (-83 to -41.2)	-74.9 (-83.6 to -48)
	Fire, heat, and hot substances	Both	5481 (3107 to 8060)	354.7 (219.3 to 500.7)	75 (45 to 109)	6.1 (4 to 8.2)	1720 (1160 to 2922)	88.9 (59.4 to 152.9)	33 (21 to 59)	1.9 (1.2 to 3.5)	-68.6 (-84.4 to -31.6)	-74.9 (-87.1 to -50.3)	-55.9 (-78.6 to -2)	-67.9 (-83.5 to -37.1)
		Female	2456 (675 to 4292)	325.5 (118.5 to 541)	33 (10 to 59)	5.5 (2.6 to 8.6)	823 (538 to 1290)	85.6 (54.7 to 135)	16 (9 to 27)	1.9 (1.1 to 3.3)	-66.5 (-86.6 to 36.1)	-73.7 (-89 to -18.1)	-53.2 (-82.5 to 91.1)	-66 (-86.3 to -12.9)
		Male	3026 (1263 to 4432)	382.8 (192.7 to 528.8)	42 (19 to 60)	6.6 (3.7 to 9.1)	897 (585 to 1788)	92 (59.4 to 186.2)	18 (11 to 36)	2 (1.3 to 4.2)	-70.4 (-85 to 21.9)	-76 (-87.1 to -19.7)	-58.1 (-78.6 to 62.9)	-69.4 (-83.6 to -11.7)
	Poisonings	Both	3078 (2064 to 4477)	173.2 (121.9 to 237.9)	40 (28 to 57)	2.7 (2 to 3.5)	942 (778 to 1260)	47 (38.9 to 63.3)	18 (15 to 23)	0.9 (0.8 to 1.2)	-69.4 (-79.7 to -50.5)	-72.8 (-81.3 to -58)	-55.3 (-69.4 to -31.6)	-64.4 (-74.5 to -48.8)
		Female	1358 (781 to 2244)	146.6 (92.3 to 227.2)	17 (10 to 28)	2.1 (1.4 to 3.1)	276 (226 to 344)	28 (23 to 34.4)	5 (4 to 6)	0.5 (0.5 to 0.7)	-79.6 (-88.1 to -58.3)	-80.9 (-88.3 to -64)	-70.4 (-82.1 to -43.6)	-74.2 (-83.3 to -55.8)

Province	Cause	Sex	1990				2019				1990-2019 percent change (%)			
			DALYs		Death		DALYs		Death		DALYs		Death	
			Number	Rate	Number	Rate	Number	Rate	Number	Rate	Number	Rate	Number	Rate
		Male	1720 (990 to 2749)	199.4 (124.5 to 297.7)	23 (14 to 36)	3.2 (2.1 to 4.6)	666 (514 to 937)	65.9 (51.2 to 94.4)	13 (10 to 18)	1.3 (1.1 to 1.8)	-61.3 (-77.5 to -30.4)	-67 (-79.4 to -43.8)	-44.1 (-65.4 to -3.4)	-57.9 (-72.7 to -30.4)
	Exposure to mechanical forces	Both	5156 (3514 to 8231)	389.5 (285.1 to 542.7)	65 (44 to 99)	5.6 (4.1 to 8)	5236 (3632 to 6515)	268.5 (182.8 to 337.3)	73 (46 to 94)	3.9 (2.4 to 5)	1.6 (-36.5 to 58.8)	-31.1 (-54.1 to 0.9)	12.6 (-32.8 to 91.2)	-30.4 (-57.7 to 10.8)
		Female	1835 (703 to 4014)	247 (121.5 to 458.6)	21 (6 to 46)	3.2 (1.4 to 5.6)	1361 (760 to 1809)	141.2 (79.3 to 187.6)	17 (6 to 23)	1.9 (0.8 to 2.5)	-25.8 (-63.8 to 37)	-42.8 (-68.2 to -8.5)	-19.6 (-60.1 to 59.3)	-41.9 (-66.3 to -5.3)
		Male	3320 (2160 to 5099)	530.5 (376.4 to 742.7)	44 (28 to 67)	8 (5.4 to 11.7)	3875 (2521 to 4905)	394.5 (251 to 507.9)	56 (33 to 74)	6 (3.4 to 7.9)	16.7 (-24.8 to 96.4)	-25.6 (-53.4 to 17.9)	27.9 (-28.9 to 136.4)	-25.7 (-62 to 31.3)
	Adverse effects of medical treatment	Both	1935 (1236 to 2643)	147.7 (97.9 to 207.4)	32 (21 to 45)	3.5 (2.4 to 5)	857 (698 to 1139)	47 (38.4 to 63.4)	21 (17 to 30)	1.4 (1.1 to 1.9)	-55.7 (-71.7 to -27)	-68.2 (-80.5 to -47.9)	-33.6 (-61 to 11.2)	-60.9 (-77.9 to -32.8)
		Female	724 (488 to 1040)	106.4 (73.8 to 146.8)	11 (8 to 16)	2.5 (1.6 to 4.4)	318 (248 to 463)	34.7 (27.2 to 50.8)	8 (6 to 12)	1 (0.7 to 1.6)	-56 (-71.9 to -31.5)	-67.3 (-79.1 to -48.5)	-31.4 (-58.3 to 9.1)	-59.3 (-77.5 to -31.3)
		Male	1211 (626 to 1865)	188.4 (103.6 to 297.8)	21 (12 to 32)	4.5 (2.6 to 7.1)	539 (415 to 729)	59.3 (46 to 81.3)	13 (10 to 19)	1.7 (1.3 to 2.6)	-55.5 (-74 to -17)	-68.5 (-82.6 to -36.6)	-34.8 (-64.5 to 32.6)	-61.7 (-80.1 to -21.8)
	Animal contact	Both	378 (253 to 552)	22.7 (16.1 to 32.2)	4 (3 to 6)	0.3 (0.2 to 0.4)	192 (144 to 277)	10 (7.6 to 14.6)	1 (1 to 3)	0.1 (0.1 to 0.2)	-49.3 (-65.2 to -21.6)	-56 (-67.6 to -38.9)	-63.5 (-76.9 to -34.5)	-66.9 (-77.3 to -48)
		Female	178 (95 to 265)	21.5 (13.4 to 30.4)	2 (1 to 3)	0.2 (0.1 to 0.4)	93 (69 to 129)	9.6 (7.2 to 13.4)	1 (0 to 1)	0.1 (0 to 0.1)	-47.9 (-68.7 to 3.3)	-55.2 (-70.7 to -26.7)	-70.2 (-83.5 to -27.6)	-71.8 (-83.8 to -44.4)
		Male	200 (116 to 337)	23.9 (15.4 to 37.9)	2 (1 to 4)	0.3 (0.2 to 0.6)	99 (75 to 157)	10.4 (7.9 to 16.8)	1 (1 to 2)	0.1 (0.1 to 0.3)	-50.5 (-68.5 to -14.4)	-56.6 (-70.2 to -34)	-58.2 (-76.7 to -22.3)	-63.2 (-77.7 to -39.3)
	Foreign body	Both	3384 (2234 to 5008)	173 (118.8 to 248.4)	38 (25 to 56)	2 (1.4 to 2.9)	1172 (961 to 1409)	62.5 (51.4 to 75.1)	14 (12 to 17)	0.8 (0.7 to 1)	-65.4 (-77.8 to -46.2)	-63.8 (-76.1 to -46.8)	-62.1 (-76.1 to -40.1)	-58.8 (-72.6 to -39.8)
		Female	1544 (910 to 2597)	157.6 (98.4 to 256.1)	17 (10 to 29)	1.8 (1.1 to 2.9)	404 (315 to 511)	43.9 (34.1 to 56.3)	4 (4 to 6)	0.5 (0.4 to 0.7)	-73.8 (-85.6 to -52.3)	-72.1 (-84.2 to -52.5)	-74.4 (-86 to -53.6)	-70.5 (-83.1 to -49.4)
		Male	1840 (967 to 2888)	188.2 (112 to 283.4)	21 (11 to 33)	2.3 (1.5 to 3.4)	768 (600 to 939)	80.8 (63 to 99.3)	10 (8 to 12)	1.1 (0.9 to 1.4)	-58.3 (-75 to -30)	-57.1 (-72.6 to -32.5)	-52 (-71.8 to -18.6)	-49.8 (-68.2 to -21.9)
	Environmental heat and cold exposure	Both	202 (146 to 307)	18.4 (13.3 to 27.3)	1 (1 to 3)	0.2 (0.1 to 0.4)	276 (207 to 365)	14.1 (10.6 to 18.6)	2 (1 to 2)	0.1 (0.1 to 0.1)	36.6 (-4.2 to 65.4)	-23.3 (-42.2 to -8.3)	29 (-32.2 to 141)	-39.4 (-67 to 11.9)
		Female	90 (62 to 136)	16.5 (11.5 to 23.3)	0 (0 to 1)	0.1 (0 to 0.3)	130 (93 to 177)	13.2 (9.5 to 18)	0 (0 to 1)	0.1 (0 to 0.1)	43.6 (-0.2 to 74.8)	-19.7 (-39.9 to -5.8)	34.4 (-42.7 to 238.3)	-31.4 (-73.9 to 74.9)
		Male	112 (80 to 181)	20.3 (14.6 to 33.3)	1 (1 to 2)	0.3 (0.1 to 0.6)	147 (107 to 194)	15 (11 to 19.7)	1 (1 to 2)	0.1 (0.1 to 0.2)	30.9 (-10.1 to 66)	-26.5 (-47.8 to -7.1)	27 (-33.1 to 131.6)	-43.4 (-71.1 to 2.7)
	Exposure to forces of nature	Both	54 (45 to 66)	4.3 (3.5 to 5.2)	0 (0 to 1)	0 (0 to 0)	696 (611 to 795)	35.2 (31 to 40)	10 (9 to 11)	0.5 (0.5 to 0.6)	1182.8 (1068.2 to 1283.4)	726.6 (642.2 to 809.4)	1943.2 (1943.2 to 1943.2)	1147.2 (1147.2 to 1147.2)
		Female	20 (16 to 24)	3 (2.5 to 3.7)	0 (0 to 0)	0 (0 to 0)	247 (217 to 285)	25.5 (22.5 to 29.1)	4 (3 to 4)	0.4 (0.4 to 0.5)	1159.4 (1053.5 to 1250.9)	747 (659.3 to 831.8)	1891.4 (1891.4 to 1891.4)	1139 (1139 to 1139)
		Male	35 (29 to 42)	5.5 (4.5 to 6.8)	0 (0 to 0)	0.1 (0 to 0.1)	448 (394 to 513)	44.7 (39.4 to 50.8)	6 (6 to 7)	0.7 (0.6 to 0.7)	1196.1 (1077.1 to 1305.1)	713.4 (631.3 to 797.6)	1974.5 (1974.5 to 1974.5)	1147.5 (1147.5 to 1147.5)
	Other unintentional injuries	Both	2914 (2117 to 4037)	213.1 (158.6 to 291.5)	32 (22 to 48)	2.4 (1.7 to 3.6)	2641 (2063 to 3311)	133.1 (104.1 to 167)	29 (22 to 36)	1.5 (1.1 to 1.8)	-9.4 (-39 to 27.2)	-37.5 (-57.6 to -15.1)	-10.9 (-48 to 43.5)	-39.6 (-66.6 to -3.4)
		Female	821 (569 to 1203)	116.5 (84.7 to 160.7)	7 (4 to 12)	1 (0.6 to 1.5)	712 (516 to 968)	72.5 (52.6 to 98.7)	4 (3 to 5)	0.4 (0.3 to 0.6)	-13.3 (-45 to 28.6)	-37.8 (-56.4 to -15.9)	-43.7 (-69.6 to 12.2)	-54.5 (-73.5 to -15.7)

Province	Cause	Sex	1990				2019				1990-2019 percent change (%)			
			DALYs		Death		DALYs		Death		DALYs		Death	
			Number	Rate	Number	Rate	Number	Rate	Number	Rate	Number	Rate	Number	Rate
		Male	2093 (1448 to 3083)	309.4 (221.3 to 453.1)	25 (16 to 40)	3.9 (2.5 to 6.3)	1929 (1460 to 2404)	192.6 (145.4 to 239.6)	25 (18 to 31)	2.5 (1.8 to 3.2)	-7.8 (-44.1 to 42.3)	-37.7 (-63.3 to -6.1)	-1.4 (-50.2 to 75.5)	-36.1 (-69.4 to 12.4)
	Self-harm and interpersonal violence	Both	12762 (10503 to 15449)	1025 (845.7 to 1230.6)	167 (135 to 211)	14.9 (11.8 to 18.6)	13492 (11730 to 15636)	672.7 (584.8 to 781)	207 (177 to 244)	10.5 (9 to 12.3)	5.7 (-14.6 to 32.5)	-34.4 (-46.5 to -18.3)	23.7 (-4.2 to 65.2)	-29.6 (-45.1 to -6.2)
		Female	5173 (3900 to 7089)	798.1 (608.1 to 1066.7)	75 (55 to 105)	12.6 (9.3 to 17.1)	4055 (3463 to 4769)	407.1 (346.7 to 478.5)	63 (52 to 77)	6.4 (5.4 to 7.8)	-21.6 (-44.7 to 9.2)	-49 (-63.3 to -29.7)	-16.8 (-43.1 to 21.5)	-49.2 (-64.1 to -27.1)
		Male	7589 (5991 to 9428)	1252.5 (981.6 to 1558.7)	92 (68 to 120)	17 (12.4 to 22.8)	9436 (7977 to 11210)	935.8 (790.3 to 1112.4)	144 (119 to 178)	14.4 (11.9 to 17.7)	24.3 (0.8 to 63.9)	-25.3 (-39.7 to -1.6)	56.9 (19 to 126.1)	-15 (-36.4 to 24.4)
	Self-harm	Both	6455 (4861 to 8474)	523.2 (400.2 to 687.6)	106 (80 to 141)	9.5 (7.4 to 12.6)	6368 (5337 to 7966)	318.2 (266.6 to 393.9)	119 (100 to 151)	6.1 (5.2 to 7.6)	-1.3 (-28.3 to 37)	-39.2 (-54.7 to -16)	12.1 (-18.7 to 59.2)	-36.1 (-52.1 to -11.5)
		Female	3989 (2850 to 5673)	620.6 (447.8 to 862)	63 (44 to 90)	10.6 (7.6 to 14.6)	2393 (1954 to 2951)	241.2 (195.9 to 295.3)	44 (35 to 54)	4.5 (3.7 to 5.5)	-40 (-60.1 to -8.6)	-61.1 (-73.4 to -41.8)	-30.8 (-53.3 to 6.8)	-57.8 (-70.7 to -36.9)
		Male	2466 (1627 to 3764)	424 (280.4 to 684.3)	43 (29 to 68)	8.4 (5.6 to 14)	3975 (3148 to 5451)	393.8 (309.9 to 529.9)	75 (60 to 104)	7.7 (6.1 to 10.6)	61.2 (15.3 to 148)	-7.1 (-34.6 to 42.2)	74.4 (24.7 to 169.3)	-8.4 (-35.2 to 42.6)
	Interpersonal violence	Both	2906 (2088 to 3678)	234 (171.3 to 294.8)	39 (26 to 52)	3.5 (2.3 to 4.7)	4541 (3823 to 5394)	227 (189.9 to 271.8)	64 (52 to 80)	3.2 (2.6 to 4)	56.3 (19.1 to 119.5)	-3 (-26.3 to 34)	63.2 (18 to 149.8)	-7.2 (-33.5 to 41)
		Female	1008 (707 to 1359)	149.9 (107.3 to 195.7)	11 (7 to 16)	1.8 (1.2 to 2.5)	1484 (1208 to 1828)	148 (120.3 to 180.9)	18 (13 to 23)	1.8 (1.4 to 2.3)	47.3 (7.8 to 113.6)	-1.3 (-26.1 to 37.4)	59 (5 to 170.4)	1 (-33.2 to 68.2)
		Male	1898 (1198 to 2594)	317.5 (205.9 to 435)	28 (16 to 40)	5.2 (2.9 to 7.5)	3057 (2444 to 3775)	304.5 (241.4 to 385.4)	46 (36 to 59)	4.7 (3.6 to 6)	61.1 (12.7 to 160.1)	-4.1 (-34 to 51.2)	64.8 (9.2 to 192.6)	-9.8 (-41.8 to 57.6)
	Conflict and terrorism	Both	2546 (1798 to 3671)	195.6 (136.5 to 281.4)	9 (8 to 10)	0.6 (0.6 to 0.7)	1198 (776 to 1805)	62.1 (40.6 to 93.2)	1 (1 to 1)	0 (0 to 0)	-53 (-59.3 to -46.7)	-68.2 (-72.5 to -64)	-91.5 (-91.5 to -91.5)	-93.7 (-93.7 to -93.7)
		Female	68 (41 to 113)	10.8 (6.6 to 17.6)	0 (0 to 0)	0 (0 to 0)	67 (43 to 111)	6.7 (4.3 to 11)	0 (0 to 0)	0 (0 to 0)	-1.5 (-20.5 to 25.6)	-37.3 (-49.4 to -20.2)	272 (272 to 272)	152.9 (152.9 to 152.9)
		Male	2478 (1750 to 3565)	382.8 (268 to 551.3)	9 (8 to 10)	1.2 (1.1 to 1.4)	1131 (729 to 1699)	118.7 (77.4 to 177)	1 (0 to 1)	0.1 (0.1 to 0.1)	-54.4 (-60.5 to -48.3)	-69 (-73.2 to -64.8)	-93.7 (-93.7 to -93.7)	-95.5 (-95.5 to -95.5)
	Executions and police conflict	Both	855 (608 to 1180)	72.2 (49.5 to 100.8)	13 (9 to 19)	1.2 (0.8 to 1.7)	1385 (745 to 2133)	65.4 (36.7 to 100.2)	23 (11 to 38)	1.1 (0.5 to 1.8)	61.9 (-2.2 to 176.9)	-9.4 (-44.6 to 53.3)	76.5 (-0.2 to 218.1)	-10.8 (-48.5 to 56.1)
		Female	108 (95 to 123)	16.8 (14.9 to 19)	1 (1 to 2)	0.3 (0.2 to 0.3)	111 (95 to 132)	11.1 (9.6 to 13.2)	1 (1 to 2)	0.2 (0.1 to 0.2)	2.7 (-3.2 to 10.3)	-33.8 (-37.5 to -29.3)	3.7 (1.9 to 5.6)	-43 (-43.8 to -42)
		Male	747 (500 to 1067)	128.1 (83.1 to 186.1)	12 (7 to 17)	2.2 (1.3 to 3.2)	1274 (633 to 2018)	118.8 (61.5 to 187.3)	22 (9 to 36)	2 (0.9 to 3.4)	70.5 (-3.1 to 204.7)	-7.3 (-46.2 to 64.7)	85.4 (-0.7 to 250.5)	-7.4 (-49.6 to 71.8)
**Hamadan**	**All causes**	Both	770003 (686149 to 861755)	48451.9 (43555.9 to 54040.7)	11035 (9823 to 12367)	1079.5 (963.3 to 1211.5)	477102 (415078 to 543670)	27040.5 (23682.3 to 30612.5)	10987 (9822 to 12173)	651.5 (584.1 to 720.1)	-38 (-45.8 to -29.9)	-44.2 (-50.6 to -37.9)	-0.4 (-15 to 16.4)	-39.6 (-48.7 to -29.1)
		Female	337375 (299723 to 378318)	43701.5 (38828 to 49120.4)	4485 (3922 to 5158)	952.2 (828.5 to 1112.9)	210897 (180162 to 244863)	24283 (20941 to 27924.3)	4516 (3968 to 5123)	570.2 (504.4 to 641.9)	-37.5 (-46.4 to -28.4)	-44.4 (-51.5 to -37.4)	0.7 (-16.9 to 20.7)	-40.1 (-50.7 to -28.4)
		Male	432628 (381368 to 492506)	52774.7 (45943.2 to 60622.2)	6550 (5613 to 7615)	1191 (1003.2 to 1417.4)	266205 (229980 to 305219)	29839.2 (25857.7 to 34123.8)	6471 (5546 to 7517)	741.6 (636.7 to 861.4)	-38.5 (-47.7 to -27.6)	-43.5 (-52.2 to -33.5)	-1.2 (-20.8 to 23.1)	-37.7 (-51.1 to -21.4)
	**Communicable, maternal, neonatal, and nutritional diseases**	Both	271467 (220122 to 329587)	11742.7 (9621.1 to 14150.9)	3060 (2481 to 3717)	147.5 (124.3 to 175)	38606 (33452 to 44398)	2684.1 (2307 to 3118.4)	540 (471 to 609)	36.6 (31.8 to 41.6)	-85.8 (-89 to -82)	-77.1 (-82.2 to -71.3)	-82.4 (-86.2 to -77.4)	-75.2 (-80.2 to -69.2)

Province	Cause	Sex	1990				2019				1990-2019 percent change (%)			
			DALYs		Death		DALYs		Death		DALYs		Death	
			Number	Rate	Number	Rate	Number	Rate	Number	Rate	Number	Rate	Number	Rate
		Female	127079 (102142 to 155469)	11310.1 (9207.3 to 13784)	1421 (1140 to 1744)	140.6 (116.5 to 169.7)	17391 (14884 to 20228)	2474.5 (2101.2 to 2902.3)	224 (189 to 262)	32.2 (27.4 to 37.5)	-86.3 (-89.5 to -82.4)	-78.1 (-83.1 to -72.3)	-84.2 (-87.9 to -79.2)	-77.1 (-82.1 to -70.9)
		Male	144388 (115737 to 177732)	12145.6 (9813.1 to 14882.3)	1639 (1304 to 2021)	153.8 (126.5 to 185.1)	21214 (18261 to 24451)	2887.7 (2457.1 to 3373.4)	315 (270 to 366)	41 (34.7 to 47.4)	-85.3 (-88.9 to -81.4)	-76.2 (-81.9 to -69.9)	-80.8 (-85.4 to -74.9)	-73.4 (-79.3 to -66.6)
	HIV/AIDS and sexually transmitted infections	Both	520 (271 to 994)	28.4 (17 to 48.8)	5 (2 to 10)	0.2 (0.1 to 0.5)	745 (544 to 1029)	40.1 (29.1 to 56.2)	10 (7 to 15)	0.5 (0.4 to 0.8)	43.3 (-18.1 to 156.8)	41 (-3.3 to 107.9)	116.6 (3.3 to 410.7)	123.5 (27.4 to 320.2)
		Female	258 (137 to 478)	30.4 (17.9 to 51.5)	2 (1 to 4)	0.2 (0.1 to 0.4)	292 (193 to 435)	33.1 (21.5 to 49.5)	3 (2 to 4)	0.3 (0.2 to 0.5)	13.3 (-31.4 to 84.3)	8.7 (-20.2 to 58)	41.5 (-31.2 to 263.8)	61.1 (-6.7 to 210.3)
		Male	262 (129 to 513)	26.4 (15.4 to 46.7)	3 (1 to 5)	0.3 (0.1 to 0.5)	453 (329 to 642)	46.9 (33.8 to 65.6)	7 (5 to 11)	0.7 (0.5 to 1.1)	72.8 (-8.4 to 245.9)	77.5 (11.3 to 187.5)	173.8 (28 to 558)	175 (45.8 to 451.3)
	HIV/AIDS	Both	39 (20 to 55)	2.9 (1.4 to 4.1)	1 (0 to 1)	0 (0 to 0.1)	399 (264 to 606)	19.6 (13.1 to 29.7)	7 (4 to 12)	0.3 (0.2 to 0.6)	919.9 (473.2 to 2164)	566.6 (274.2 to 1383.5)	1138.9 (518.3 to 3811.2)	626.4 (262.3 to 2463.9)
		Female	8 (4 to 11)	1 (0.5 to 1.4)	0 (0 to 0)	0 (0 to 0)	86 (49 to 156)	9.5 (5.3 to 17.1)	1 (1 to 3)	0.2 (0.1 to 0.3)	1025.8 (426.6 to 2583)	821.7 (330.6 to 2030.5)	1211.1 (424.1 to 3783.7)	811 (265.2 to 2737.3)
		Male	31 (16 to 44)	4.8 (2.2 to 6.8)	0 (0 to 1)	0.1 (0 to 0.1)	313 (209 to 485)	29.4 (19.7 to 44.9)	6 (4 to 9)	0.5 (0.3 to 0.9)	894.2 (472.2 to 2155.4)	508.7 (252.7 to 1324.3)	1122.2 (542.7 to 3925.6)	583.5 (253 to 2296.1)
	Sexually transmitted infections excluding HIV	Both	481 (234 to 951)	25.5 (14.3 to 45.5)	4 (1 to 9)	0.2 (0.1 to 0.4)	346 (217 to 539)	20.5 (12.2 to 33.6)	3 (1 to 4)	0.2 (0.1 to 0.3)	-28.1 (-53 to 13.2)	-19.6 (-37.2 to -0.4)	-32.2 (-61 to 54.5)	-2.4 (-36.8 to 64.5)
		Female	250 (129 to 470)	29.4 (16.9 to 50.4)	2 (1 to 4)	0.2 (0.1 to 0.4)	206 (127 to 329)	23.6 (14.2 to 38.1)	1 (1 to 2)	0.2 (0.1 to 0.3)	-17.6 (-47.7 to 23.2)	-19.8 (-36.4 to -4.5)	-27.2 (-60.3 to 57.2)	-2.8 (-37.6 to 54.7)
		Male	230 (98 to 477)	21.6 (10.5 to 41.5)	2 (1 to 5)	0.2 (0.1 to 0.4)	139 (83 to 217)	17.5 (9.9 to 29.4)	1 (0 to 2)	0.2 (0.1 to 0.3)	-39.5 (-60.3 to 3.5)	-18.8 (-41.2 to 11.8)	-36.6 (-69.2 to 57.7)	0.3 (-47.4 to 105.3)
	Respiratory infections and tuberculosis	Both	43630 (32288 to 60248)	2041.7 (1580.5 to 2711.8)	544 (419 to 729)	35.6 (29.9 to 43.2)	8142 (6944 to 9495)	494.6 (423.6 to 576.8)	258 (218 to 300)	15.6 (13.2 to 18.2)	-81.3 (-87.2 to -74.1)	-75.8 (-83 to -67.9)	-52.6 (-66.1 to -35.8)	-56.1 (-65.4 to -45.9)
		Female	21254 (14757 to 30865)	2000.1 (1453 to 2781.8)	258 (186 to 364)	33.6 (26.6 to 43.2)	3338 (2781 to 4034)	422.4 (354.7 to 511.8)	103 (84 to 130)	13.5 (11 to 17.2)	-84.3 (-89.8 to -76)	-78.9 (-85.9 to -69.5)	-60.2 (-73.1 to -41.1)	-59.9 (-70.4 to -48.2)
		Male	22375 (15918 to 31753)	2078.7 (1572.4 to 2834.5)	285 (210 to 391)	37.5 (29.8 to 47.4)	4804 (4015 to 5697)	567.7 (474.5 to 674.3)	155 (124 to 188)	17.9 (14.3 to 21.7)	-78.5 (-85.6 to -68.8)	-72.7 (-81.1 to -62)	-45.8 (-63.4 to -22)	-52.2 (-64.3 to -36.2)
	Tuberculosis	Both	1286 (1032 to 1535)	88.4 (70.5 to 105.8)	24 (19 to 29)	2.5 (1.9 to 3.1)	367 (313 to 424)	19.9 (17.1 to 23)	11 (9 to 13)	0.6 (0.5 to 0.8)	-71.5 (-77.4 to -61.8)	-77.5 (-82.4 to -69.3)	-54 (-64.6 to -34.4)	-74.3 (-80.6 to -61.1)
		Female	593 (356 to 802)	79.4 (46.6 to 109.3)	10 (5 to 14)	2.1 (1.1 to 3.1)	142 (116 to 180)	15.9 (13.1 to 20.1)	4 (3 to 6)	0.5 (0.4 to 0.7)	-76.1 (-83.6 to -52.1)	-79.9 (-86.4 to -59.7)	-57.8 (-72.6 to 0.9)	-75.9 (-84.7 to -41.6)
		Male	693 (555 to 855)	97.1 (77.7 to 120.8)	14 (11 to 18)	2.8 (2.2 to 3.6)	225 (188 to 269)	23.9 (19.8 to 28.5)	7 (6 to 8)	0.8 (0.6 to 0.9)	-67.5 (-75.1 to -57.8)	-75.4 (-81.2 to -68.1)	-51.2 (-64.4 to -35)	-72.8 (-80.6 to -63.4)
	Lower respiratory infections	Both	40132 (29209 to 56935)	1833.7 (1390 to 2506.1)	519 (395 to 706)	33.1 (27.5 to 40.7)	5813 (5018 to 6692)	359.7 (309.4 to 415.2)	246 (208 to 288)	15 (12.6 to 17.5)	-85.5 (-90.4 to -79.1)	-80.4 (-86.8 to -72.9)	-52.6 (-66.6 to -35)	-54.8 (-64.6 to -43.9)
		Female	19636 (13311 to 29136)	1806.1 (1274.3 to 2579.4)	248 (175 to 356)	31.5 (24.6 to 40.9)	2276 (1864 to 2794)	296.4 (243.4 to 362.7)	99 (80 to 126)	12.9 (10.5 to 16.6)	-88.4 (-92.8 to -81.6)	-83.6 (-89.5 to -75.1)	-60.3 (-73.7 to -40.3)	-58.9 (-70 to -46.2)
		Male	20496 (14252 to 29797)	1857.4 (1340.4 to 2602.5)	271 (197 to 377)	34.7 (27.3 to 44.3)	3538 (2925 to 4215)	424 (348.8 to 509)	148 (118 to 180)	17.1 (13.6 to 20.9)	-82.7 (-88.9 to -74)	-77.2 (-84.9 to -67)	-45.6 (-63.6 to -20.8)	-50.5 (-63.4 to -33.6)

Province	Cause	Sex	1990				2019				1990-2019 percent change (%)			
			DALYs		Death		DALYs		Death		DALYs		Death	
			Number	Rate	Number	Rate	Number	Rate	Number	Rate	Number	Rate	Number	Rate
	Upper respiratory infections	Both	1562 (928 to 2470)	84.9 (51.3 to 132.7)	0 (0 to 1)	0 (0 to 0)	1464 (884 to 2320)	85.5 (51.5 to 135.8)	0 (0 to 1)	0 (0 to 0)	-6.3 (-14.6 to 2.8)	0.6 (-4.7 to 6.5)	101.5 (-92.7 to 4682.8)	113.8 (-91.8 to 3591.3)
		Female	741 (435 to 1154)	83 (49.6 to 131.3)	0 (0 to 0)	0 (0 to 0)	698 (423 to 1104)	83.3 (50.6 to 131.9)	0 (0 to 0)	0 (0 to 0)	-5.7 (-13.7 to 4.3)	0.4 (-5.9 to 8.1)	69.9 (-95.4 to 5639.1)	98 (-94 to 4116.1)
		Male	821 (492 to 1305)	86.8 (52.3 to 136.1)	0 (0 to 1)	0 (0 to 0.1)	766 (458 to 1216)	87.6 (52.5 to 139.3)	0 (0 to 1)	0 (0 to 0.1)	-6.7 (-16.3 to 4)	0.9 (-6.8 to 8.4)	118.3 (-91.4 to 4738.4)	122.4 (-90.6 to 3617.2)
	Otitis media	Both	650 (370 to 1069)	34.6 (19.6 to 57.2)	0 (0 to 0)	0 (0 to 0)	498 (281 to 832)	29.5 (16.7 to 49.2)	0 (0 to 0)	0 (0 to 0)	-23.4 (-31.8 to -13.8)	-14.6 (-20.2 to -8.1)	-68.5 (-87.3 to -23.4)	-68.8 (-84.6 to -31.5)
		Female	284 (160 to 462)	31.7 (17.8 to 52.3)	0 (0 to 0)	0 (0 to 0)	222 (127 to 369)	26.8 (15.4 to 44.7)	0 (0 to 0)	0 (0 to 0)	-21.9 (-30.9 to -10.7)	-15.5 (-22.8 to -6.6)	36.3 (-75 to 368.1)	-28.6 (-77.3 to 129.4)
		Male	366 (209 to 607)	37.4 (21.2 to 61.2)	0 (0 to 0)	0 (0 to 0)	276 (154 to 470)	32.2 (18.1 to 53.9)	0 (0 to 0)	0 (0 to 0)	-24.6 (-33.9 to -14.2)	-13.8 (-21.3 to -5.8)	-85.3 (-93.9 to -61.7)	-81.9 (-91.8 to -55.7)
	Enteric infections	Both	16597 (11057 to 25293)	761.7 (529.1 to 1122.5)	181 (117 to 276)	10.8 (7.2 to 15.9)	4514 (3381 to 5768)	284.2 (212.3 to 362)	32 (23 to 48)	2.1 (1.5 to 3.1)	-72.8 (-83.2 to -58.1)	-62.7 (-76 to -44.6)	-82.3 (-89.7 to -71.1)	-81 (-87.2 to -71.8)
		Female	7559 (4687 to 11840)	717.1 (469.9 to 1085.8)	82 (48 to 133)	10.7 (6 to 17.8)	2132 (1580 to 2784)	273.4 (203.3 to 357)	14 (8 to 26)	2 (1.1 to 3.5)	-71.8 (-82.9 to -52.2)	-61.9 (-76.1 to -39.2)	-82.4 (-91.3 to -64.1)	-81.6 (-88.9 to -68.6)
		Male	9038 (5519 to 15274)	802.8 (518.3 to 1309.2)	99 (59 to 170)	10.8 (6.8 to 17)	2382 (1795 to 3084)	294.9 (221.2 to 377.9)	18 (12 to 29)	2.2 (1.5 to 3.5)	-73.6 (-85.2 to -55.5)	-63.3 (-78.7 to -40.7)	-82.2 (-91 to -65.9)	-79.8 (-88.3 to -67.7)
	Diarrheal diseases	Both	14891 (9508 to 23382)	686.1 (455.7 to 1032.8)	158 (96 to 249)	9.7 (6.2 to 14.4)	4101 (3019 to 5362)	256.6 (188.8 to 332.8)	26 (17 to 42)	1.7 (1.1 to 2.6)	-72.5 (-83.3 to -55.4)	-62.6 (-76.8 to -43.1)	-83.7 (-91.2 to -71.4)	-83 (-88.9 to -74)
		Female	6787 (3945 to 10981)	646 (402.7 to 1020.6)	72 (38 to 122)	9.7 (5.1 to 16.5)	1949 (1403 to 2596)	248.5 (180.1 to 330.5)	12 (5 to 23)	1.6 (0.8 to 3.1)	-71.3 (-83.4 to -48.1)	-61.5 (-76.9 to -36.1)	-83.8 (-93.2 to -63.7)	-83.4 (-91.1 to -69.8)
		Male	8105 (4671 to 14313)	723.3 (450.8 to 1223.6)	86 (47 to 155)	9.6 (5.9 to 15.9)	2152 (1573 to 2817)	264.8 (193 to 343.5)	14 (9 to 25)	1.7 (1.1 to 3)	-73.4 (-85.7 to -52.5)	-63.4 (-79.4 to -38.7)	-83.6 (-92.2 to -65.3)	-82 (-90.1 to -69.2)
	Typhoid and paratyphoid	Both	1299 (527 to 2604)	57.5 (24 to 113.8)	18 (7 to 35)	0.8 (0.3 to 1.6)	297 (120 to 593)	20 (8 to 39.3)	4 (2 to 9)	0.3 (0.1 to 0.6)	-77.2 (-82.7 to -71.1)	-65.3 (-73.5 to -57)	-75.4 (-81.1 to -68.6)	-65.6 (-73.1 to -58)
		Female	544 (218 to 1108)	50.5 (20.5 to 102.4)	7 (3 to 15)	0.7 (0.3 to 1.5)	132 (54 to 262)	18.1 (7.3 to 36)	2 (1 to 4)	0.3 (0.1 to 0.5)	-75.8 (-82.4 to -66.8)	-64.1 (-73.5 to -51.2)	-73.8 (-80.8 to -63.6)	-64.6 (-74 to -52.3)
		Male	755 (305 to 1531)	64 (25.9 to 126.4)	10 (4 to 21)	0.9 (0.4 to 1.7)	165 (65 to 333)	21.7 (8.4 to 43.8)	2 (1 to 5)	0.3 (0.1 to 0.6)	-78.2 (-84.3 to -71.3)	-66.1 (-75.3 to -55.9)	-76.5 (-83 to -68.9)	-66.2 (-75.2 to -56)
	Invasive Non-typhoidal Salmonella (iNTS)	Both	332 (181 to 564)	14.9 (8.2 to 24.7)	5 (2 to 8)	0.2 (0.1 to 0.4)	108 (55 to 188)	7.1 (3.5 to 12.5)	2 (1 to 3)	0.1 (0.1 to 0.2)	-67.4 (-78.9 to -55)	-52.5 (-68.7 to -36.5)	-58.6 (-71.1 to -44.5)	-50.4 (-63.2 to -37.1)
		Female	185 (97 to 315)	17 (9.1 to 29)	3 (1 to 4)	0.3 (0.1 to 0.4)	47 (24 to 84)	6.2 (3.1 to 11.3)	1 (0 to 1)	0.1 (0.1 to 0.2)	-74.4 (-84.2 to -63)	-63.3 (-76.1 to -48.3)	-65.7 (-77.5 to -51)	-59.7 (-71.2 to -45.5)
		Male	147 (79 to 254)	12.9 (6.9 to 22.1)	2 (1 to 4)	0.2 (0.1 to 0.4)	61 (30 to 108)	7.9 (3.8 to 14)	1 (1 to 2)	0.1 (0.1 to 0.2)	-58.6 (-74.4 to -41.6)	-39.2 (-61.2 to -16)	-49.9 (-66.9 to -30.5)	-39.1 (-57.3 to -19.4)
	Other intestinal infectious diseases	Both	74 (17 to 153)	3.1 (0.8 to 6.2)	1 (0 to 2)	0 (0 to 0.1)	8 (5 to 11)	0.5 (0.3 to 0.7)	0 (0 to 0)	0 (0 to 0)	-89.7 (-95.8 to -51.1)	-84 (-93.5 to -33.5)	-89.4 (-96 to -50.1)	-85.3 (-94.3 to -49)
		Female	43 (3 to 113)	3.6 (0.4 to 9.4)	0 (0 to 1)	0 (0 to 0.1)	4 (2 to 6)	0.5 (0.2 to 0.8)	0 (0 to 0)	0 (0 to 0)	-91.6 (-97.4 to 10.4)	-86.6 (-96 to 39.7)	-91.2 (-97.6 to 36)	-87.2 (-96.5 to -0.8)
		Male	31 (4 to 75)	2.6 (0.5 to 6.1)	0 (0 to 1)	0 (0 to 0.1)	4 (2 to 6)	0.5 (0.3 to 0.8)	0 (0 to 0)	0 (0 to 0)	-87.2 (-95.7 to 0.7)	-80.4 (-93.3 to 16.1)	-86.9 (-96.4 to 16.7)	-82.5 (-94.9 to -10.2)
	Neglected tropical diseases and malaria	Both	1877 (673 to 5974)	96.6 (34.9 to 310)	21 (4 to 85)	1.3 (0.3 to 5.1)	296 (214 to 415)	18 (12.9 to 25.6)	2 (1 to 3)	0.1 (0.1 to 0.2)	-84.2 (-95.4 to -56.6)	-81.4 (-94.5 to -49.8)	-90.9 (-97.8 to -50.4)	-91.1 (-97.8 to -56.2)

Province	Cause	Sex	1990				2019				1990-2019 percent change (%)			
			DALYs		Death		DALYs		Death		DALYs		Death	
			Number	Rate	Number	Rate	Number	Rate	Number	Rate	Number	Rate	Number	Rate
		Female	854 (294 to 2785)	93.8 (33.8 to 301.4)	9 (1 to 39)	1.1 (0.2 to 4.9)	146 (101 to 210)	18 (12.4 to 26)	1 (0 to 1)	0.1 (0.1 to 0.1)	-82.9 (-95.2 to -53.1)	-80.8 (-94.6 to -49)	-91.4 (-98.2 to -42.9)	-91.5 (-98.2 to -49.8)
		Male	1022 (329 to 3221)	99.1 (32.8 to 313.5)	12 (2 to 47)	1.4 (0.3 to 5.3)	150 (101 to 218)	17.9 (11.9 to 27.3)	1 (1 to 2)	0.1 (0.1 to 0.2)	-85.4 (-95.5 to -51.4)	-81.9 (-94.5 to -40.7)	-90.5 (-97.6 to -42.4)	-90.6 (-97.5 to -51)
	Malaria	Both	947 (22 to 4957)	50 (1.2 to 258.7)	15 (0 to 78)	0.9 (0 to 4.6)	20 (14 to 27)	1.1 (0.8 to 1.5)	0 (0 to 0)	0 (0 to 0)	-97.9 (-99.6 to -15.3)	-97.8 (-99.6 to -13.2)	-100 (-100 to -100)	-100 (-100 to -100)
		Female	412 (11 to 2336)	46.8 (1.3 to 261.6)	6 (0 to 37)	0.8 (0 to 4.6)	10 (7 to 14)	1.1 (0.8 to 1.6)	0 (0 to 0)	0 (0 to 0)	-97.6 (-99.6 to -14.6)	-97.6 (-99.6 to -17.4)	-100 (-100 to -100)	-100 (-100 to -100)
		Male	535 (10 to 2667)	53 (1.1 to 259.8)	8 (0 to 42)	1 (0 to 4.7)	10 (7 to 13)	1.1 (0.8 to 1.5)	0 (0 to 0)	0 (0 to 0)	-98.2 (-99.7 to -13.5)	-98 (-99.6 to -6.9)	-100 (-100 to -100)	-100 (-100 to -100)
	Leishmaniasis	Both	71 (0 to 765)	3.3 (0 to 33.8)	1 (0 to 10)	0.1 (0 to 0.5)	4 (0 to 38)	0.2 (0 to 2.4)	0 (0 to 1)	0 (0 to 0)	-94.6 (-95.5 to -68.9)	-92.7 (-94.2 to -61.7)	-93.6 (-94.6 to -89)	-92.6 (-94 to -90.8)
		Female	23 (0 to 230)	2.1 (0 to 20.7)	0 (0 to 3)	0 (0 to 0.3)	1 (0 to 11)	0.2 (0 to 1.4)	0 (0 to 0)	0 (0 to 0)	-94.8 (-95.8 to -50.1)	-92.9 (-94.7 to -44.5)	-93.7 (-94.9 to -88)	-92.9 (-94.5 to -90.3)
		Male	49 (0 to 533)	4.4 (0 to 45.7)	1 (0 to 7)	0.1 (0 to 0.7)	3 (0 to 26)	0.3 (0 to 3.3)	0 (0 to 0)	0 (0 to 0)	-94.5 (-95.4 to -79.3)	-92.5 (-94.2 to -72.1)	-93.6 (-94.6 to -88.7)	-92.5 (-94.2 to -90.1)
	Schistosomiasis	Both	0 (0 to 0)	0 (0 to 0)	0 (0 to 0)	0 (0 to 0)	0 (0 to 0)	0 (0 to 0)	0 (0 to 0)	0 (0 to 0)	0 (0 to 0)	0 (0 to 0)	0 (0 to 0)	0 (0 to 0)
		Female	0 (0 to 0)	0 (0 to 0)	0 (0 to 0)	0 (0 to 0)	0 (0 to 0)	0 (0 to 0)	0 (0 to 0)	0 (0 to 0)	0 (0 to 0)	0 (0 to 0)	0 (0 to 0)	0 (0 to 0)
		Male	0 (0 to 0)	0 (0 to 0)	0 (0 to 0)	0 (0 to 0)	0 (0 to 0)	0 (0 to 0)	0 (0 to 0)	0 (0 to 0)	0 (0 to 0)	0 (0 to 0)	0 (0 to 0)	0 (0 to 0)
	Cystic echinococcosis	Both	145 (74 to 237)	8.4 (5.2 to 12.1)	2 (1 to 3)	0.1 (0.1 to 0.2)	53 (38 to 76)	2.9 (2 to 4)	0 (0 to 1)	0 (0 to 0)	-63.2 (-78.6 to -27.4)	-66 (-78.1 to -43.9)	-75.5 (-88.9 to -44.7)	-81 (-91.2 to -63.8)
		Female	62 (24 to 116)	7.4 (3.8 to 12.1)	1 (0 to 1)	0.1 (0 to 0.2)	28 (18 to 41)	3 (2 to 4.4)	0 (0 to 0)	0 (0 to 0)	-55.4 (-78.8 to 12)	-59.8 (-78.3 to -19.5)	-73.9 (-92.4 to -5.2)	-79.3 (-93.7 to -41.5)
		Male	83 (32 to 155)	9.4 (4.8 to 14.8)	1 (0 to 2)	0.2 (0.1 to 0.3)	26 (17 to 37)	2.8 (1.8 to 3.9)	0 (0 to 0)	0 (0 to 0.1)	-68.9 (-84.4 to -21.2)	-70.5 (-83.5 to -39.4)	-76.5 (-92.5 to -26.7)	-81.8 (-93.5 to -53.6)
	Trachoma	Both	15 (9 to 26)	2.1 (1.2 to 3.5)	--	--	7 (3 to 11)	0.4 (0.2 to 0.7)	--	--	-57.4 (-68.7 to -44.2)	-81.4 (-86.2 to -76.1)	--	--
		Female	12 (7 to 20)	3.4 (1.9 to 5.6)	--	--	5 (2 to 8)	0.5 (0.3 to 0.9)	--	--	-60.9 (-72.8 to -45)	-83.9 (-88.7 to -78.1)	--	--
		Male	4 (2 to 7)	0.9 (0.5 to 1.7)	--	--	2 (1 to 4)	0.2 (0.1 to 0.4)	--	--	-46.1 (-64.3 to -17.4)	-74.5 (-82.8 to -63.9)	--	--
	Rabies	Both	17 (3 to 40)	1.1 (0.1 to 2.3)	0 (0 to 1)	0 (0 to 0)	6 (1 to 10)	0.3 (0 to 0.6)	0 (0 to 0)	0 (0 to 0)	-64.4 (-87.6 to -17.7)	-66.7 (-88.1 to -24.9)	-54.5 (-82.7 to -2.1)	-64.8 (-86 to -27.9)
		Female	0 (0 to 1)	0 (0 to 0.1)	0 (0 to 0)	0 (0 to 0)	0 (0 to 0)	0 (0 to 0)	0 (0 to 0)	0 (0 to 0)	-71.9 (-85.6 to -37.2)	-76.1 (-88.4 to -53.6)	-61.4 (-81.3 to -25.5)	-75.4 (-88.4 to -56.4)
		Male	17 (2 to 40)	2 (0.2 to 4.5)	0 (0 to 1)	0 (0 to 0.1)	6 (1 to 10)	0.7 (0.1 to 1.1)	0 (0 to 0)	0 (0 to 0)	-64.2 (-87.9 to -16.6)	-66.2 (-88.1 to -23.6)	-54.3 (-83.3 to -1.2)	-64 (-86.1 to -25.1)
	Intestinal nematode infections	Both	186 (73 to 349)	8.5 (3.8 to 14.9)	2 (0 to 3)	0.1 (0 to 0.1)	19 (10 to 32)	1.1 (0.6 to 1.9)	0 (0 to 0)	0 (0 to 0)	-89.9 (-95.7 to -74.7)	-86.6 (-93.8 to -71.2)	-95.6 (-98.6 to -80)	-93.6 (-97.8 to -76.3)
		Female	98 (33 to 210)	9.7 (3.9 to 18.6)	1 (0 to 2)	0.1 (0 to 0.2)	11 (6 to 20)	1.4 (0.7 to 2.4)	0 (0 to 0)	0 (0 to 0)	-88.4 (-95.7 to -65.9)	-85.6 (-94.1 to -64.8)	-95.9 (-99.1 to -24.5)	-93.9 (-98.7 to -43.6)
		Male	87 (22 to 198)	7.3 (2.3 to 15.6)	1 (0 to 2)	0.1 (0 to 0.2)	7 (4 to 13)	0.9 (0.4 to 1.6)	0 (0 to 0)	0 (0 to 0)	-91.5 (-97.2 to -64.7)	-87.8 (-95.7 to -58.5)	-95.4 (-98.8 to -51.8)	-93.3 (-98.2 to -60.6)

Province	Cause	Sex	1990				2019				1990-2019 percent change (%)			
			DALYs		Death		DALYs		Death		DALYs		Death	
			Number	Rate	Number	Rate	Number	Rate	Number	Rate	Number	Rate	Number	Rate
	Food-borne trematodiases	Both	6 (1 to 13)	0.5 (0.1 to 1)	--	--	19 (5 to 40)	1 (0.2 to 2.1)	--	--	221.6 (143.4 to 309.9)	107.8 (61.7 to 159.7)	--	--
		Female	2 (1 to 5)	0.4 (0.1 to 0.8)	--	--	8 (2 to 16)	0.8 (0.2 to 1.7)	--	--	218.6 (131.5 to 319.4)	100.9 (49.2 to 164.7)	--	--
		Male	4 (1 to 7)	0.6 (0.1 to 1.2)	--	--	12 (3 to 24)	1.2 (0.3 to 2.4)	--	--	223.6 (115.7 to 356.7)	113.4 (46 to 193.8)	--	--
	Leprosy	Both	0 (0 to 1)	0 (0 to 0.1)	--	--	1 (0 to 1)	0 (0 to 0.1)	--	--	42 (30.5 to 55.4)	-25.1 (-30.7 to -18.4)	--	--
		Female	0 (0 to 0)	0 (0 to 0.1)	--	--	0 (0 to 0)	0 (0 to 0)	--	--	34.7 (17.8 to 53.6)	-30.9 (-39.1 to -21.5)	--	--
		Male	0 (0 to 0)	0.1 (0 to 0.1)	--	--	0 (0 to 1)	0 (0 to 0.1)	--	--	45.7 (30.1 to 64.3)	-21 (-29.1 to -11.6)	--	--
	Other neglected tropical diseases	Both	488 (311 to 738)	22.7 (15.2 to 33.3)	2 (1 to 4)	0.1 (0.1 to 0.2)	167 (106 to 261)	10.8 (6.8 to 16.9)	1 (1 to 2)	0.1 (0 to 0.1)	-65.7 (-77.7 to -48.1)	-52.3 (-68.2 to -29.9)	-34.7 (-67.6 to 11.5)	-37 (-62.5 to 1.3)
		Female	245 (142 to 386)	24.1 (14.8 to 35.8)	1 (0 to 2)	0.1 (0.1 to 0.2)	83 (51 to 130)	11 (6.6 to 17.1)	1 (0 to 1)	0.1 (0 to 0.1)	-65.9 (-78.7 to -44.8)	-54.4 (-70.7 to -29.1)	-37.8 (-67.5 to 30.8)	-39.9 (-61.7 to 13.4)
		Male	243 (131 to 416)	21.4 (12.3 to 35)	1 (0 to 3)	0.1 (0.1 to 0.3)	84 (46 to 142)	10.7 (5.9 to 18.9)	1 (0 to 1)	0.1 (0 to 0.1)	-65.6 (-82 to -34.4)	-50.1 (-73.1 to -9.2)	-31.9 (-70.8 to 41.5)	-33.3 (-65.5 to 27.2)
	Other infectious diseases	Both	31338 (17939 to 54844)	1350.9 (811.5 to 2284.9)	383 (227 to 659)	19 (12.4 to 30.1)	2765 (2341 to 3398)	170.7 (142.2 to 216.6)	59 (49 to 69)	3.5 (2.9 to 4.2)	-91.2 (-94.9 to -84.3)	-87.4 (-92.6 to -78.2)	-84.6 (-90.9 to -73.4)	-81.4 (-88.1 to -70.5)
		Female	15957 (8916 to 28394)	1393.5 (811.7 to 2416.8)	191 (108 to 337)	19 (11.9 to 30.8)	1155 (933 to 1467)	149.8 (119 to 197.6)	22 (18 to 28)	2.8 (2.2 to 3.6)	-92.8 (-95.9 to -86.8)	-89.3 (-93.7 to -80.9)	-88.3 (-93.2 to -79.7)	-85.1 (-90.7 to -75.5)
		Male	15381 (8762 to 26851)	1309.2 (789.6 to 2209.5)	192 (113 to 325)	18.9 (12.1 to 29.4)	1610 (1316 to 1952)	191.7 (156.3 to 235)	37 (29 to 44)	4.2 (3.4 to 5.1)	-89.5 (-93.9 to -81.2)	-85.4 (-91.2 to -74.8)	-80.9 (-88.9 to -65.9)	-77.6 (-86 to -63.2)
	Meningitis	Both	7631 (5598 to 10307)	342.8 (259.7 to 450.5)	95 (72 to 126)	5.1 (4.1 to 6.4)	1006 (855 to 1192)	61.7 (52.1 to 73.3)	22 (19 to 27)	1.3 (1.1 to 1.6)	-86.8 (-90.7 to -81.2)	-82 (-87.1 to -74.5)	-76.6 (-83.1 to -66.6)	-74 (-80.5 to -64.2)
		Female	3726 (2308 to 5475)	340.5 (221 to 487.6)	46 (29 to 66)	5 (3.5 to 6.9)	422 (348 to 508)	53.7 (43.9 to 65.7)	9 (7 to 11)	1.1 (0.9 to 1.3)	-88.7 (-92.7 to -81.5)	-84.2 (-89.9 to -75.6)	-80.8 (-87.3 to -69.1)	-77.9 (-85 to -68)
		Male	3905 (2733 to 5588)	344.6 (246 to 478.3)	50 (35 to 68)	5.2 (3.9 to 6.8)	584 (467 to 733)	69.6 (55.3 to 87.4)	14 (11 to 17)	1.6 (1.2 to 2)	-85 (-90.1 to -76.1)	-79.8 (-86.4 to -68.2)	-72.8 (-81.9 to -55.5)	-70.1 (-79.3 to -52.7)
	Encephalitis	Both	514 (352 to 736)	24.9 (18.5 to 33.9)	6 (5 to 9)	0.4 (0.3 to 0.5)	303 (225 to 363)	19 (13.6 to 23.1)	6 (4 to 7)	0.3 (0.2 to 0.4)	-41 (-61.4 to -16.9)	-23.7 (-47.6 to 5.3)	-12.8 (-41.3 to 22.3)	-13.7 (-41.9 to 17.6)
		Female	296 (163 to 474)	28.8 (17.2 to 43.8)	4 (2 to 6)	0.4 (0.3 to 0.6)	153 (101 to 196)	19.8 (12.5 to 25.8)	3 (2 to 4)	0.3 (0.2 to 0.5)	-48.2 (-72.4 to -19.1)	-31.2 (-63.4 to 4.6)	-24.7 (-58.1 to 16.5)	-22.3 (-51.2 to 12.9)
		Male	218 (148 to 322)	21.1 (14.9 to 29.6)	3 (2 to 4)	0.4 (0.2 to 0.5)	150 (115 to 183)	18.2 (13.5 to 22.3)	3 (2 to 4)	0.3 (0.2 to 0.4)	-31.2 (-57.4 to 7.8)	-13.8 (-46.2 to 31.6)	2.3 (-38.3 to 64.1)	-1.8 (-42.8 to 52.8)
	Diphtheria	Both	109 (45 to 230)	4.5 (1.9 to 9.2)	1 (1 to 3)	0.1 (0 to 0.1)	1 (1 to 2)	0.1 (0.1 to 0.1)	0 (0 to 0)	0 (0 to 0)	-98.7 (-99.5 to -96.7)	-98 (-99.2 to -95)	-98.4 (-99.3 to -96)	-97.6 (-99 to -94.5)
		Female	51 (13 to 134)	4.3 (1.2 to 11.3)	1 (0 to 2)	0.1 (0 to 0.1)	0 (0 to 1)	0.1 (0 to 0.1)	0 (0 to 0)	0 (0 to 0)	-99 (-99.7 to -95.8)	-98.4 (-99.6 to -93.3)	-98.8 (-99.6 to -95.1)	-98.2 (-99.5 to -93.1)
		Male	58 (18 to 130)	4.7 (1.7 to 10.2)	1 (0 to 2)	0.1 (0 to 0.1)	1 (0 to 2)	0.1 (0.1 to 0.2)	0 (0 to 0)	0 (0 to 0)	-98.4 (-99.4 to -94.7)	-97.6 (-99.2 to -92.3)	-98 (-99.2 to -93.8)	-97.2 (-98.9 to -92.2)

Province	Cause	Sex	1990				2019				1990-2019 percent change (%)			
			DALYs		Death		DALYs		Death		DALYs		Death	
			Number	Rate	Number	Rate	Number	Rate	Number	Rate	Number	Rate	Number	Rate
	Whooping cough	Both	5927 (317 to 24486)	237.4 (12.7 to 986.1)	67 (3 to 282)	2.7 (0.1 to 11.4)	163 (11 to 695)	12.2 (0.8 to 52.4)	2 (0 to 8)	0.1 (0 to 0.6)	-97.3 (-99.9 to -39.4)	-94.9 (-99.7 to 13.8)	-97.3 (-99.9 to -20.6)	-95 (-99.8 to 47.5)
		Female	3365 (182 to 13935)	276.2 (14.9 to 1139.2)	38 (2 to 161)	3.1 (0.1 to 13.1)	88 (6 to 377)	13.6 (0.9 to 58.3)	1 (0 to 4)	0.2 (0 to 0.7)	-97.4 (-99.9 to -40.1)	-95.1 (-99.7 to 12)	-97.4 (-99.9 to -23.7)	-95.2 (-99.8 to 42.5)
		Male	2563 (135 to 10743)	200.3 (10.5 to 842)	29 (1 to 123)	2.3 (0.1 to 9.7)	75 (5 to 333)	10.9 (0.7 to 48.2)	1 (0 to 4)	0.1 (0 to 0.6)	-97.1 (-99.9 to -32.5)	-94.6 (-99.7 to 25.6)	-97.1 (-99.9 to -15.8)	-94.7 (-99.8 to 55.8)
	Tetanus	Both	363 (112 to 1207)	16 (5.5 to 53.1)	4 (2 to 15)	0.2 (0.1 to 0.7)	16 (12 to 27)	1 (0.8 to 1.6)	0 (0 to 1)	0 (0 to 0)	-95.6 (-98.8 to -85.1)	-93.7 (-98.2 to -79.8)	-93 (-98 to -79.8)	-91.7 (-97.3 to -78.7)
		Female	193 (42 to 779)	17.1 (4.4 to 66.7)	2 (1 to 9)	0.2 (0.1 to 0.8)	7 (5 to 10)	1 (0.7 to 1.3)	0 (0 to 0)	0 (0 to 0)	-96.2 (-99.1 to -82)	-94.4 (-98.7 to -76.3)	-93.9 (-98.5 to -76.4)	-92.6 (-97.9 to -76.3)
		Male	170 (50 to 595)	15 (4.6 to 51.4)	2 (1 to 7)	0.2 (0.1 to 0.7)	9 (6 to 18)	1.1 (0.8 to 2.2)	0 (0 to 0)	0 (0 to 0)	-95 (-98.7 to -81.9)	-92.8 (-98.1 to -76.5)	-92 (-97.9 to -74.1)	-90.8 (-97.4 to -71.5)
	Measles	Both	13694 (4466 to 29879)	540 (175.9 to 1179.5)	159 (52 to 348)	6.3 (2 to 13.7)	145 (43 to 341)	10.7 (3.2 to 25)	2 (0 to 4)	0.1 (0 to 0.3)	-98.9 (-99.6 to -98)	-98 (-99.2 to -96.2)	-99 (-99.6 to -98)	-98.1 (-99.2 to -96.3)
		Female	6831 (2190 to 15266)	553.3 (177.3 to 1242.1)	79 (25 to 177)	6.4 (2.1 to 14.4)	69 (21 to 162)	10.4 (3.1 to 24.6)	1 (0 to 2)	0.1 (0 to 0.3)	-99 (-99.6 to -98.1)	-98.1 (-99.2 to -96.4)	-99 (-99.6 to -98.1)	-98.2 (-99.2 to -96.5)
		Male	6863 (2213 to 14877)	527.3 (170.1 to 1140.8)	80 (26 to 173)	6.1 (2 to 13.3)	77 (23 to 181)	10.9 (3.2 to 25.8)	1 (0 to 2)	0.1 (0 to 0.3)	-98.9 (-99.5 to -97.8)	-97.9 (-99.1 to -95.8)	-98.9 (-99.5 to -97.8)	-98 (-99.1 to -95.9)
	Varicella and herpes zoster	Both	220 (126 to 374)	12 (8 to 18.3)	3 (2 to 5)	0.3 (0.2 to 0.4)	83 (59 to 115)	5 (3.6 to 7.1)	1 (1 to 2)	0.1 (0.1 to 0.1)	-62.2 (-80.6 to -30.3)	-58.3 (-75.6 to -34)	-55.3 (-77.2 to -15.8)	-71.1 (-83 to -52.3)
		Female	110 (54 to 213)	12.2 (7.2 to 20.8)	1 (1 to 3)	0.3 (0.2 to 0.4)	41 (28 to 59)	5 (3.3 to 7.3)	1 (0 to 1)	0.1 (0 to 0.1)	-63 (-82.6 to -21.5)	-59.3 (-78.3 to -24.3)	-59.2 (-81.5 to -7.2)	-71 (-85.2 to -42.5)
		Male	110 (53 to 202)	11.8 (7.1 to 19.2)	1 (1 to 3)	0.3 (0.2 to 0.5)	42 (28 to 61)	5 (3.2 to 7.4)	1 (0 to 1)	0.1 (0 to 0.1)	-61.5 (-81.5 to -20.1)	-57.4 (-77.2 to -25.6)	-51.5 (-79.3 to 7.2)	-71 (-86.2 to -40.2)
	Acute hepatitis	Both	2018 (1206 to 3019)	128 (76.4 to 189.5)	37 (21 to 55)	3.2 (1.8 to 4.9)	503 (409 to 611)	27.5 (22.5 to 33)	14 (11 to 17)	0.8 (0.6 to 0.9)	-75.1 (-84.5 to -55.3)	-78.5 (-86.4 to -62.1)	-63.2 (-77.8 to -32.2)	-76.4 (-86.1 to -56.4)
		Female	1004 (483 to 1658)	118.8 (57.1 to 182.9)	16 (7 to 25)	2.7 (1.2 to 4)	133 (106 to 163)	15.5 (12.4 to 19)	3 (2 to 4)	0.4 (0.3 to 0.5)	-86.7 (-92.3 to -71.5)	-87 (-91.9 to -71.4)	-80.3 (-87.8 to -52.5)	-86.5 (-91.5 to -65.2)
		Male	1014 (557 to 1783)	136.3 (77.3 to 239.4)	21 (11 to 37)	3.6 (2 to 6.4)	370 (283 to 469)	39.6 (30.4 to 49.9)	10 (8 to 13)	1.1 (0.8 to 1.5)	-63.5 (-81 to -30.4)	-70.9 (-84.8 to -44.8)	-49.7 (-75.3 to -1.4)	-68 (-84.6 to -38.1)
	Other unspecified infectious diseases	Both	861 (561 to 1801)	45.4 (30.5 to 87.7)	9 (5 to 21)	0.7 (0.4 to 1.4)	543 (380 to 675)	33.5 (23.3 to 41.9)	12 (7 to 15)	0.7 (0.4 to 0.9)	-36.9 (-75.8 to 6.4)	-26.2 (-68.6 to 19.9)	35.2 (-59.9 to 169.1)	1.9 (-60.4 to 98.6)
		Female	381 (244 to 849)	42.3 (28.2 to 82.1)	4 (2 to 9)	0.7 (0.4 to 1.4)	241 (162 to 313)	30.7 (20.6 to 40.1)	5 (3 to 7)	0.7 (0.4 to 0.9)	-36.7 (-77.1 to 6.6)	-27.3 (-70.2 to 18.2)	36.5 (-61.9 to 190.2)	-3.5 (-62.1 to 110.2)
		Male	480 (282 to 1109)	48.2 (30.2 to 103.3)	5 (3 to 13)	0.8 (0.5 to 1.6)	302 (203 to 391)	36.2 (24.5 to 47.7)	7 (4 to 9)	0.8 (0.4 to 1.1)	-37 (-76.1 to 18.8)	-24.9 (-69.1 to 32.6)	34.3 (-61.1 to 189.4)	7.8 (-61.3 to 112.5)
	Maternal and neonatal disorders	Both	169470 (135662 to 204462)	7055.7 (5649.3 to 8495.3)	1898 (1518 to 2293)	79.1 (63.3 to 95.2)	19528 (16164 to 23430)	1516.3 (1239.2 to 1826.3)	175 (139 to 218)	14.4 (11.4 to 18)	-88.5 (-91.3 to -85)	-78.5 (-83.9 to -71.9)	-90.8 (-93.3 to -87.7)	-81.7 (-86.7 to -75.7)
		Female	77152 (61237 to 94764)	6632.8 (5293.3 to 8139)	866 (689 to 1065)	74.6 (59.5 to 91.5)	8893 (7299 to 10692)	1401.6 (1145.7 to 1705.6)	79 (63 to 98)	13.3 (10.5 to 16.5)	-88.5 (-91.3 to -84.9)	-78.9 (-84.2 to -72.1)	-90.8 (-93.3 to -87.7)	-82.2 (-87 to -75.9)
		Male	92318 (72691 to 112106)	7454.8 (5867.9 to 9049.9)	1033 (813 to 1258)	83.3 (65.5 to 101.4)	10636 (8615 to 12806)	1624.4 (1301.8 to 1969.2)	96 (74 to 119)	15.5 (12 to 19.4)	-88.5 (-91.5 to -84.5)	-78.2 (-84 to -70.5)	-90.7 (-93.4 to -87.1)	-81.3 (-86.7 to -74)

Province	Cause	Sex	1990				2019				1990-2019 percent change (%)			
			DALYs		Death		DALYs		Death		DALYs		Death	
			Number	Rate	Number	Rate	Number	Rate	Number	Rate	Number	Rate	Number	Rate
	Maternal disorders	Female	1572 (1160 to 2097)	208.1 (153.8 to 277.9)	22 (15 to 30)	3 (2.1 to 4.2)	326 (252 to 417)	33.3 (26 to 42.4)	4 (3 to 5)	0.4 (0.3 to 0.5)	-79.2 (-85.6 to -69.7)	-84 (-88.9 to -76.5)	-82.1 (-88.5 to -70.6)	-87.4 (-91.9 to -79.7)
	Neonatal disorders	Both	167898 (134305 to 203003)	6953.4 (5565 to 8406.3)	1877 (1499 to 2272)	77.6 (62 to 93.9)	19202 (15782 to 23126)	1500 (1223.5 to 1809)	171 (135 to 214)	14.3 (11.2 to 17.8)	-88.6 (-91.4 to -85.1)	-78.4 (-83.9 to -71.7)	-90.9 (-93.4 to -87.8)	-81.6 (-86.7 to -75.4)
		Female	75579 (59566 to 93067)	6424.7 (5062.2 to 7909)	844 (664 to 1041)	71.6 (56.3 to 88.3)	8566 (6995 to 10346)	1368.3 (1113.1 to 1673.6)	76 (59 to 94)	12.9 (10.1 to 16.1)	-88.7 (-91.5 to -85)	-78.7 (-84.2 to -71.6)	-91 (-93.5 to -87.9)	-81.9 (-87 to -75.5)
		Male	92318 (72691 to 112106)	7454.8 (5867.9 to 9049.9)	1033 (813 to 1258)	83.3 (65.5 to 101.4)	10636 (8615 to 12806)	1624.4 (1301.8 to 1969.2)	96 (74 to 119)	15.5 (12 to 19.4)	-88.5 (-91.5 to -84.5)	-78.2 (-84 to -70.5)	-90.7 (-93.4 to -87.1)	-81.3 (-86.7 to -74)
	Nutritional deficiencies	Both	8036 (5677 to 11230)	407.8 (291.4 to 566)	28 (17 to 42)	1.5 (1 to 2.1)	2616 (1646 to 3911)	160.3 (100.8 to 239.7)	4 (3 to 4)	0.2 (0.2 to 0.3)	-67.4 (-76.5 to -56.4)	-60.7 (-70.8 to -47.4)	-86.2 (-91 to -76.8)	-84.4 (-89.1 to -74.8)
		Female	4045 (2727 to 5783)	442.4 (301.8 to 619.3)	12 (7 to 21)	1.4 (0.8 to 2.2)	1436 (895 to 2209)	176.3 (108.2 to 267.7)	1 (1 to 2)	0.2 (0.2 to 0.2)	-64.5 (-76.5 to -47.9)	-60.1 (-72.1 to -43.9)	-88 (-93.2 to -78)	-85 (-91 to -74.1)
		Male	3991 (2601 to 6031)	374.7 (248.5 to 556.8)	15 (8 to 24)	1.7 (1 to 2.4)	1180 (690 to 1903)	144.2 (83.1 to 235)	2 (2 to 3)	0.3 (0.2 to 0.3)	-70.4 (-81.4 to -53.6)	-61.5 (-75.6 to -40.8)	-84.8 (-90.6 to -71.2)	-84 (-89.3 to -70.9)
	Protein-energy malnutrition	Both	2748 (1879 to 4086)	121.3 (84.7 to 175.9)	27 (16 to 41)	1.4 (0.9 to 2)	529 (350 to 758)	32.9 (21.7 to 46.9)	3 (2 to 3)	0.2 (0.1 to 0.2)	-80.7 (-88.3 to -68.3)	-72.8 (-83.1 to -57.4)	-89.9 (-93.5 to -83.1)	-88.3 (-91.9 to -81.4)
		Female	1280 (818 to 2047)	114.9 (74.6 to 179.9)	12 (7 to 21)	1.3 (0.8 to 2.2)	248 (161 to 364)	31.6 (20.5 to 45.7)	1 (1 to 1)	0.2 (0.1 to 0.2)	-80.6 (-89.4 to -67.8)	-72.5 (-84.7 to -55.2)	-90.3 (-94.4 to -82.2)	-87.6 (-92.6 to -78.5)
		Male	1468 (912 to 2249)	127.3 (82.6 to 190.4)	14 (8 to 23)	1.5 (0.9 to 2.2)	281 (181 to 410)	34.3 (22.1 to 49.4)	1 (1 to 2)	0.2 (0.1 to 0.2)	-80.8 (-89.3 to -65.9)	-73.1 (-84.4 to -54.7)	-89.6 (-93.8 to -80)	-88.7 (-92.7 to -79.7)
	Iodine deficiency	Both	270 (166 to 395)	17.6 (11.4 to 25.6)	--	--	124 (71 to 200)	6.7 (3.9 to 10.8)	--	--	-54.1 (-66.3 to -41)	-61.9 (-71.4 to -52.7)	--	--
		Female	163 (99 to 241)	22 (13.9 to 31.9)	--	--	77 (43 to 126)	8.5 (4.7 to 13.6)	--	--	-52.9 (-67.6 to -37.1)	-61.4 (-72.7 to -49.8)	--	--
		Male	106 (66 to 159)	13.5 (8.6 to 19.9)	--	--	47 (27 to 75)	5 (2.9 to 7.9)	--	--	-55.9 (-68.2 to -41.9)	-62.9 (-72.6 to -52.7)	--	--
	Vitamin A deficiency	Both	307 (155 to 519)	11.9 (6.1 to 20.1)	--	--	11 (4 to 25)	0.8 (0.3 to 1.8)	--	--	-96.3 (-98.6 to -91.2)	-93.1 (-97.3 to -83.7)	--	--
		Female	111 (50 to 212)	8.9 (4 to 16.8)	--	--	5 (2 to 11)	0.7 (0.2 to 1.6)	--	--	-95.7 (-98.5 to -89.4)	-91.9 (-97.1 to -79.8)	--	--
		Male	195 (81 to 399)	14.7 (6.2 to 30)	--	--	6 (2 to 19)	0.9 (0.2 to 2.6)	--	--	-96.7 (-99.2 to -89)	-93.8 (-98.5 to -79.8)	--	--
	Dietary iron deficiency	Both	4495 (2651 to 7105)	244.5 (150.8 to 379.8)	--	--	1786 (1022 to 2900)	110.4 (63 to 182.4)	--	--	-60.3 (-73.6 to -42)	-54.9 (-69 to -36.3)	--	--
		Female	2412 (1356 to 3854)	287.1 (171.4 to 429.9)	--	--	1032 (565 to 1707)	127.1 (69.3 to 210.9)	--	--	-57.2 (-74.1 to -31)	-55.7 (-71.8 to -32.7)	--	--
		Male	2083 (1040 to 3799)	203.8 (109.2 to 347.7)	--	--	753 (352 to 1430)	93.6 (43.1 to 180.7)	--	--	-63.8 (-81.6 to -28.4)	-54.1 (-76.5 to -14.8)	--	--
	Other nutritional deficiencies	Both	217 (141 to 313)	12.5 (8.4 to 17.8)	1 (1 to 2)	0.1 (0.1 to 0.1)	166 (108 to 245)	9.4 (6.2 to 13.8)	1 (1 to 1)	0.1 (0.1 to 0.1)	-23.2 (-44.1 to 5.5)	-24.4 (-42.9 to 1.1)	-11.1 (-48.2 to 74.5)	-32.2 (-55.2 to 21.1)
		Female	79 (49 to 116)	9.5 (6 to 14.1)	0 (0 to 0)	0 (0 to 0.1)	74 (45 to 111)	8.5 (5.2 to 12.7)	0 (0 to 0)	0 (0 to 0.1)	-6.2 (-33.2 to 36.8)	-11.1 (-35.1 to 27.8)	14.7 (-40 to 156.9)	-8.4 (-51.7 to 105.9)
		Male	138 (87 to 208)	15.3 (10 to 21.9)	1 (0 to 2)	0.2 (0.1 to 0.2)	93 (60 to 138)	10.4 (6.8 to 15.3)	1 (1 to 1)	0.1 (0.1 to 0.1)	-32.9 (-56.2 to 5.9)	-32.2 (-53.7 to 2.2)	-17.8 (-55.3 to 77)	-40.1 (-63.3 to 17.7)

Province	Cause	Sex	1990				2019				1990-2019 percent change (%)			
			DALYs		Death		DALYs		Death		DALYs		Death	
			Number	Rate	Number	Rate	Number	Rate	Number	Rate	Number	Rate	Number	Rate
	**Non-communicable diseases**	Both	337606 (280316 to 393350)	27711.2 (23934.1 to 31585.9)	5750 (4892 to 6659)	790.1 (692.7 to 890.9)	369501 (317193 to 425551)	20609.8 (17747.1 to 23698.2)	9265 (8270 to 10253)	550.5 (492.8 to 607.5)	9.4 (-7.2 to 29.7)	-25.6 (-35.8 to -15.1)	61.1 (33.6 to 95)	-30.3 (-41.3 to -17.9)
		Female	158418 (129577 to 186634)	26744.8 (22670.8 to 30940.4)	2415 (1974 to 2898)	731 (623.1 to 863)	176402 (148718 to 208008)	19875.3 (16901.5 to 23319.4)	4027 (3529 to 4574)	507.2 (447 to 573)	11.4 (-6.7 to 32.9)	-25.7 (-36.3 to -14.1)	66.7 (32.9 to 112.7)	-30.6 (-43.7 to -15.1)
		Male	179188 (144212 to 215420)	28473.2 (23769.8 to 33729.3)	3335 (2721 to 4110)	837.6 (692.9 to 1013.7)	193099 (164461 to 223704)	21451.3 (18316.7 to 24779.9)	5238 (4460 to 6117)	603.3 (514.1 to 704.7)	7.8 (-13 to 35.1)	-24.7 (-38.2 to -8.4)	57.1 (18.5 to 108.4)	-28 (-44.6 to -6.8)
	Neoplasms	Both	29001 (23937 to 34636)	2577.8 (2142 to 3042.9)	801 (666 to 951)	95.5 (79.5 to 112.7)	42746 (36732 to 49059)	2368.1 (2036.9 to 2712.2)	1609 (1382 to 1842)	92.8 (79.6 to 105.8)	47.4 (16.9 to 88.6)	-8.1 (-26.6 to 17.6)	100.9 (59.5 to 156.7)	-2.8 (-21.7 to 24.2)
		Female	12321 (9492 to 15477)	2181.1 (1706.8 to 2731)	314 (247 to 393)	76.2 (60.3 to 96.2)	17608 (14927 to 20923)	1930.9 (1640.6 to 2295.3)	628 (533 to 744)	72.1 (61.4 to 85.2)	42.9 (8.5 to 94.8)	-11.5 (-32.5 to 18.6)	99.9 (50.1 to 167.8)	-5.4 (-28 to 26.2)
		Male	16680 (12578 to 21461)	2934.3 (2267.9 to 3757)	487 (374 to 629)	113.6 (88.7 to 144.4)	25137 (20419 to 30320)	2821.3 (2300 to 3398.4)	981 (803 to 1172)	113.8 (93 to 135.8)	50.7 (9 to 110)	-3.9 (-30.3 to 34.2)	101.5 (45.2 to 180.8)	0.2 (-26.4 to 39.6)
	Lip and oral cavity cancer	Both	148 (115 to 195)	15.8 (12.1 to 20.6)	5 (4 to 6)	0.6 (0.5 to 0.8)	280 (235 to 329)	15.2 (12.8 to 17.8)	11 (9 to 13)	0.6 (0.5 to 0.7)	88.5 (33.5 to 166.6)	-3.9 (-31.4 to 36.4)	125.4 (58.3 to 227.5)	-0.3 (-29.2 to 44.7)
		Female	50 (38 to 65)	11 (8.2 to 14.2)	2 (1 to 2)	0.4 (0.3 to 0.6)	120 (98 to 145)	13.1 (10.7 to 15.9)	5 (4 to 6)	0.6 (0.5 to 0.7)	139.6 (70.1 to 247)	19.5 (-14.1 to 71.5)	203.3 (115 to 345)	28.3 (-8.3 to 89.7)
		Male	99 (69 to 141)	20.3 (14.2 to 29)	3 (2 to 5)	0.8 (0.6 to 1.2)	160 (125 to 200)	17.3 (13.6 to 21.6)	6 (5 to 8)	0.7 (0.6 to 0.9)	62.5 (1.2 to 160.6)	-14.4 (-47.4 to 36.2)	88.3 (17.3 to 204.3)	-13.9 (-45.7 to 36.5)
	Nasopharynx cancer	Both	81 (63 to 103)	7.2 (5.7 to 9.1)	2 (2 to 3)	0.2 (0.2 to 0.3)	89 (74 to 106)	4.7 (4 to 5.7)	3 (2 to 3)	0.2 (0.1 to 0.2)	9.3 (-18.7 to 52)	-34.3 (-50.2 to -10)	29.8 (-1.9 to 76.7)	-34.5 (-50.2 to -11.6)
		Female	29 (20 to 39)	5.5 (3.9 to 7.3)	1 (1 to 1)	0.2 (0.1 to 0.3)	28 (23 to 35)	3.1 (2.5 to 3.9)	1 (1 to 1)	0.1 (0.1 to 0.1)	-3.7 (-31.7 to 55.9)	-43.1 (-59.5 to -9.9)	18.9 (-18.3 to 87.8)	-43.7 (-62 to -12.5)
		Male	52 (36 to 71)	8.8 (6.3 to 12)	1 (1 to 2)	0.3 (0.2 to 0.4)	60 (48 to 76)	6.4 (5.1 to 7.9)	2 (1 to 2)	0.2 (0.2 to 0.3)	16.6 (-21.4 to 81)	-27.9 (-51.3 to 10.3)	36 (-8 to 110)	-27 (-50 to 9.1)
	Other pharynx cancer	Both	57 (45 to 73)	6.2 (4.9 to 7.9)	2 (2 to 3)	0.3 (0.2 to 0.3)	92 (76 to 109)	5 (4.1 to 5.9)	4 (3 to 4)	0.2 (0.2 to 0.2)	61.8 (18.3 to 116.9)	-19.2 (-40.4 to 7.8)	87.2 (37.7 to 152.5)	-17 (-38.7 to 12.2)
		Female	25 (17 to 34)	5.7 (4 to 7.7)	1 (1 to 1)	0.2 (0.2 to 0.3)	36 (28 to 45)	3.9 (3.1 to 4.9)	1 (1 to 2)	0.2 (0.1 to 0.2)	42.6 (-6.8 to 120.7)	-31.6 (-54.9 to 5.9)	74.9 (14.9 to 170.5)	-27.6 (-51.8 to 10.2)
		Male	32 (22 to 45)	6.5 (4.7 to 9.2)	1 (1 to 2)	0.3 (0.2 to 0.4)	56 (43 to 72)	6.1 (4.7 to 7.9)	2 (2 to 3)	0.2 (0.2 to 0.3)	77.1 (10.3 to 170.6)	-6.6 (-41.2 to 41.5)	96.6 (23 to 197.5)	-4.5 (-39 to 42.4)
	Esophageal cancer	Both	702 (540 to 973)	81.1 (62.6 to 112.6)	26 (20 to 36)	3.6 (2.8 to 5)	1171 (943 to 1819)	65 (52.5 to 100.6)	51 (42 to 79)	3 (2.4 to 4.5)	66.9 (22.1 to 128.8)	-19.9 (-41 to 7.1)	96.4 (46.1 to 165.2)	-17 (-38.9 to 10.2)
		Female	271 (187 to 430)	67.2 (46.5 to 105.5)	10 (7 to 16)	3.1 (2.2 to 4.8)	393 (298 to 636)	43.8 (33.3 to 70.6)	18 (14 to 28)	2.1 (1.6 to 3.3)	44.9 (-1 to 118.4)	-34.8 (-54.7 to -3.2)	77.6 (24.2 to 161.7)	-30.9 (-52.8 to 0.7)
		Male	431 (302 to 619)	93.5 (66.3 to 135.8)	16 (11 to 23)	4 (2.9 to 5.6)	779 (596 to 1244)	86.6 (66.3 to 137.9)	33 (25 to 53)	3.8 (2.9 to 6.1)	80.7 (19.4 to 171.7)	-7.4 (-38.5 to 38.8)	108.3 (37.4 to 213.7)	-4.8 (-35.6 to 42)
	Stomach cancer	Both	5177 (4067 to 6389)	584.9 (463 to 721.2)	188 (149 to 233)	25.7 (20.5 to 31.5)	6696 (5618 to 7865)	372.7 (312.3 to 437.7)	292 (246 to 342)	17.1 (14.4 to 19.9)	29.3 (-1 to 73.7)	-36.3 (-50.8 to -15.2)	55.1 (19 to 107.3)	-33.5 (-48.2 to -13.1)
		Female	1795 (1319 to 2393)	421.8 (314.7 to 554.4)	63 (47 to 82)	18.5 (13.8 to 24.3)	2180 (1789 to 2650)	240.4 (197.8 to 290.6)	96 (80 to 116)	11.3 (9.4 to 13.7)	21.5 (-13.9 to 68.1)	-43 (-59.7 to -21.8)	53 (8.6 to 110.7)	-39 (-55.2 to -17.1)

Province	Cause	Sex	1990				2019				1990-2019 percent change (%)			
			DALYs		Death		DALYs		Death		DALYs		Death	
			Number	Rate	Number	Rate	Number	Rate	Number	Rate	Number	Rate	Number	Rate
		Male	3383 (2481 to 4478)	732.2 (534.6 to 963.6)	126 (92 to 165)	32.2 (23.9 to 41.8)	4515 (3562 to 5556)	510 (401.7 to 625)	196 (156 to 240)	23 (18.4 to 28.2)	33.5 (-6.5 to 95.2)	-30.3 (-50.9 to 1)	56.1 (9.7 to 125.7)	-28.6 (-49.1 to 3.4)
	Colon and rectum cancer	Both	1239 (966 to 1582)	136.2 (107.7 to 172.7)	42 (33 to 54)	5.6 (4.5 to 7.1)	3275 (2749 to 3802)	178.7 (150 to 207.8)	133 (112 to 155)	7.7 (6.4 to 8.9)	164.2 (90.5 to 265.7)	31.2 (-4.5 to 80.8)	215.8 (129 to 337.1)	35.7 (-0.9 to 86)
		Female	570 (416 to 773)	131.9 (96.6 to 176.9)	19 (14 to 26)	5.5 (4.1 to 7.3)	1435 (1164 to 1756)	156.8 (127.6 to 191.4)	60 (48 to 73)	7 (5.7 to 8.5)	151.9 (65.9 to 278.6)	18.9 (-20.1 to 77.7)	209.6 (109.5 to 372.2)	25.6 (-13.6 to 90.5)
		Male	670 (473 to 946)	139.6 (99.1 to 197.4)	23 (16 to 32)	5.7 (4.1 to 8)	1839 (1452 to 2260)	201.7 (160.3 to 250.4)	73 (58 to 91)	8.4 (6.7 to 10.5)	174.7 (75.1 to 327.4)	44.5 (-7.2 to 125.1)	221 (105.9 to 408.1)	47.7 (-3.2 to 131.5)
	Liver cancer	Both	860 (658 to 1114)	91.6 (70.4 to 117.9)	30 (23 to 39)	3.9 (3 to 4.9)	1526 (1246 to 1862)	84.9 (69.7 to 103.5)	63 (51 to 76)	3.6 (2.9 to 4.4)	77.5 (31.4 to 143.3)	-7.4 (-31.8 to 28.8)	110.4 (53 to 194)	-7.9 (-32.4 to 30.4)
		Female	353 (248 to 499)	79 (54.6 to 116.5)	12 (8 to 18)	3.5 (2.4 to 5.2)	563 (446 to 707)	62.4 (49.6 to 77.8)	23 (19 to 29)	2.7 (2.2 to 3.4)	59.5 (-0.2 to 160)	-20.9 (-51.8 to 30.5)	95.7 (18.7 to 224.3)	-21.4 (-53.9 to 30.7)
		Male	507 (356 to 728)	102.8 (72.5 to 146.3)	18 (12 to 25)	4.3 (3.1 to 6.1)	963 (730 to 1271)	108.1 (82.5 to 142.8)	39 (30 to 52)	4.6 (3.5 to 6)	90.1 (26.6 to 177.8)	5.1 (-29.5 to 54.2)	120.2 (48.4 to 223.7)	5.1 (-28.3 to 54.3)
	Gallbladder and biliary tract cancer	Both	243 (183 to 314)	28.4 (21.7 to 36.8)	9 (7 to 12)	1.3 (1 to 1.7)	464 (371 to 552)	25.7 (20.8 to 30.6)	20 (17 to 24)	1.2 (1 to 1.4)	90.7 (39.8 to 171.6)	-9.5 (-33.4 to 26.6)	121.4 (68.3 to 211.4)	-7.6 (-29.7 to 27.8)
		Female	122 (82 to 168)	30.6 (21.2 to 42.4)	5 (3 to 7)	1.4 (1 to 2)	221 (165 to 286)	24.6 (18.7 to 31.7)	10 (8 to 13)	1.2 (0.9 to 1.6)	81.5 (25.3 to 185.6)	-19.6 (-43.3 to 24.7)	116.4 (52.6 to 235.2)	-15.4 (-39.4 to 29.8)
		Male	122 (85 to 173)	26.4 (18.7 to 37.2)	4 (3 to 6)	1.1 (0.8 to 1.6)	243 (177 to 308)	26.9 (19.6 to 34.1)	10 (8 to 13)	1.2 (0.9 to 1.5)	99.8 (30.9 to 211)	1.6 (-33.3 to 55.6)	126.6 (50.8 to 244.5)	2.5 (-30.5 to 54.1)
	Pancreatic cancer	Both	399 (296 to 526)	44.9 (33.7 to 59.3)	15 (11 to 19)	1.9 (1.4 to 2.5)	1694 (1433 to 1964)	93.6 (79.1 to 108.5)	71 (60 to 82)	4.1 (3.5 to 4.7)	324.8 (200.4 to 504.4)	108.2 (48.5 to 191.2)	384.3 (246.2 to 584.6)	115.2 (55.2 to 202.8)
		Female	149 (110 to 204)	35.8 (26.6 to 48.6)	5 (4 to 8)	1.5 (1.2 to 2.1)	683 (554 to 833)	75.5 (61.2 to 91.7)	30 (24 to 36)	3.5 (2.8 to 4.2)	358.8 (197.6 to 563)	110.9 (38.3 to 205)	441.6 (248.2 to 684.5)	124.5 (45.3 to 225.3)
		Male	250 (162 to 369)	53.4 (35 to 77.6)	9 (6 to 13)	2.2 (1.5 to 3.3)	1011 (796 to 1265)	112 (88 to 140.9)	41 (33 to 51)	4.7 (3.8 to 5.9)	304.5 (150.3 to 583.5)	109.8 (30.2 to 250.8)	349.8 (178.3 to 659.7)	111.9 (33 to 247.1)
	Larynx cancer	Both	707 (538 to 910)	77.6 (59.6 to 100.1)	24 (19 to 32)	3.1 (2.4 to 3.9)	1025 (826 to 1238)	56 (45.2 to 67.6)	40 (33 to 48)	2.3 (1.9 to 2.8)	45 (3.4 to 107)	-27.9 (-48.1 to 2.1)	65.1 (18.5 to 133.4)	-25 (-44.9 to 4.7)
		Female	145 (103 to 190)	32.9 (23.8 to 43)	5 (3 to 6)	1.3 (0.9 to 1.7)	185 (149 to 230)	20.3 (16.4 to 25.1)	8 (6 to 9)	0.9 (0.7 to 1.1)	28 (-11.2 to 92.5)	-38.4 (-56.8 to -8)	58.8 (9.7 to 139.6)	-32.5 (-52.1 to 0.6)
		Male	562 (410 to 767)	118.8 (88 to 159.7)	20 (14 to 27)	4.7 (3.5 to 6.3)	840 (646 to 1048)	92.4 (71.3 to 116)	33 (25 to 40)	3.8 (2.9 to 4.7)	49.4 (1.9 to 125)	-22.2 (-46.7 to 16.1)	66.6 (12.5 to 148.2)	-20.7 (-45.5 to 17.4)
	Tracheal, bronchus, and lung cancer	Both	2453 (1858 to 3313)	265.4 (201 to 356.4)	86 (65 to 117)	10.8 (8.2 to 14.7)	5608 (4633 to 6616)	308.5 (255.4 to 363.9)	227 (189 to 268)	13.1 (10.8 to 15.4)	128.6 (51.5 to 229.8)	16.2 (-23.4 to 67.5)	163.4 (73.9 to 281.9)	20.6 (-20 to 71.6)
		Female	442 (326 to 629)	98.3 (73.3 to 138.6)	14 (11 to 21)	4 (3 to 5.6)	1405 (1143 to 1711)	153.4 (124.5 to 186.3)	58 (47 to 70)	6.7 (5.4 to 8.1)	217.8 (107.5 to 362.5)	56 (1.8 to 125.4)	299.2 (156.3 to 472.2)	66.3 (7 to 139)
		Male	2011 (1425 to 2803)	417.2 (296.5 to 580.5)	72 (51 to 102)	17.2 (12.2 to 23.8)	4203 (3320 to 5178)	468.4 (370.5 to 574.5)	170 (135 to 208)	19.7 (15.8 to 24.3)	109 (30.2 to 226.3)	12.3 (-29.7 to 74.3)	136.1 (49.6 to 267.8)	14.9 (-27.3 to 76.6)
	Malignant skin melanoma	Both	194 (97 to 286)	19.7 (10 to 28.4)	6 (3 to 9)	0.8 (0.4 to 1.2)	224 (167 to 290)	12.1 (8.9 to 15.6)	9 (6 to 11)	0.5 (0.3 to 0.6)	15.2 (-27.7 to 108.6)	-38.5 (-61 to 10)	43 (-6.7 to 145.4)	-41.2 (-60.7 to 1.4)
		Female	80 (34 to 128)	16.8 (7.6 to 25.8)	2 (1 to 4)	0.7 (0.3 to 1.1)	91 (57 to 126)	9.9 (6.3 to 13.7)	3 (2 to 5)	0.4 (0.2 to 0.6)	13.5 (-35.2 to 124.6)	-41.2 (-64.7 to 9)	42.1 (-11.4 to 150.4)	-43.8 (-63.3 to -6.9)

Province	Cause	Sex	1990				2019				1990-2019 percent change (%)			
			DALYs		Death		DALYs		Death		DALYs		Death	
			Number	Rate	Number	Rate	Number	Rate	Number	Rate	Number	Rate	Number	Rate
		Male	115 (51 to 175)	22.3 (9.7 to 34.3)	4 (2 to 6)	0.9 (0.4 to 1.5)	133 (89 to 197)	14.4 (9.5 to 20.9)	5 (3 to 7)	0.6 (0.3 to 0.8)	16.4 (-30.2 to 124.5)	-35.6 (-61.6 to 26.2)	43.6 (-11.2 to 171.8)	-38.2 (-62.9 to 22.2)
	Non-melanoma skin cancer	Both	50 (38 to 63)	6 (4.6 to 7.7)	2 (1 to 3)	0.3 (0.2 to 0.4)	155 (130 to 182)	8.6 (7.2 to 10.1)	8 (6 to 9)	0.5 (0.4 to 0.5)	211.7 (128.7 to 326.5)	43.2 (4.1 to 96)	294.4 (187.5 to 448.5)	48.3 (8.8 to 108.6)
		Female	8 (5 to 11)	1.9 (1.3 to 2.8)	0 (0 to 0)	0.1 (0.1 to 0.1)	29 (23 to 36)	3.3 (2.6 to 4)	1 (1 to 2)	0.2 (0.1 to 0.2)	288.1 (144.7 to 511.5)	71.9 (8.3 to 180.7)	401.8 (220.9 to 738.1)	78.3 (11 to 206.5)
		Male	42 (32 to 55)	9.8 (7.5 to 12.9)	2 (1 to 2)	0.5 (0.4 to 0.7)	125 (102 to 152)	13.9 (11.3 to 16.9)	6 (5 to 8)	0.7 (0.6 to 0.9)	197.9 (109.3 to 329.2)	41.2 (-0.2 to 100)	275.7 (162.8 to 438.7)	40.3 (0.6 to 99.9)
	Breast cancer	Both	1458 (1058 to 2050)	150.9 (109.1 to 215.1)	42 (30 to 60)	4.8 (3.4 to 7)	3324 (2694 to 4045)	168.8 (137.3 to 205.9)	100 (81 to 122)	5.4 (4.4 to 6.6)	128 (53.2 to 232.1)	11.9 (-25.2 to 63.5)	138.9 (56.9 to 257.2)	11.7 (-28.1 to 69.2)
		Female	1441 (1038 to 2034)	307.1 (220.6 to 441.8)	41 (29 to 60)	9.8 (6.8 to 14.4)	3298 (2670 to 4020)	335 (271.7 to 408.4)	99 (81 to 121)	10.6 (8.7 to 13)	128.9 (52.9 to 234.5)	9.1 (-27.5 to 60.5)	139.7 (56.5 to 259.9)	8.6 (-30.5 to 66)
		Male	17 (11 to 26)	3.3 (2.1 to 5.2)	1 (0 to 1)	0.1 (0.1 to 0.2)	26 (18 to 36)	2.8 (2 to 3.8)	1 (1 to 1)	0.1 (0.1 to 0.1)	54.9 (-12.7 to 158.6)	-15.5 (-51.9 to 40.9)	77.9 (0.5 to 193.8)	-15 (-51.7 to 40.9)
	Cervical cancer	Female	374 (250 to 508)	80.2 (55.1 to 109.4)	11 (8 to 16)	2.8 (2 to 3.9)	492 (373 to 622)	51.7 (39.1 to 65.2)	17 (13 to 22)	1.9 (1.5 to 2.4)	31.4 (-8.5 to 104.6)	-35.5 (-54.3 to -0.5)	53.6 (7.2 to 135.2)	-32.1 (-52.7 to 0.9)
	Uterine cancer	Female	62 (42 to 96)	14.2 (9.7 to 23.1)	2 (1 to 3)	0.6 (0.4 to 1)	175 (113 to 224)	18.9 (12.5 to 24)	6 (5 to 8)	0.7 (0.5 to 0.9)	184.4 (59 to 368.7)	33 (-27.1 to 117)	211 (75.5 to 404.7)	30.5 (-27.9 to 108.9)
	Ovarian cancer	Female	206 (121 to 389)	42 (24.8 to 79.6)	6 (3 to 11)	1.4 (0.8 to 2.6)	751 (529 to 941)	79.4 (56.7 to 99.3)	25 (19 to 31)	2.7 (2.1 to 3.3)	265.2 (62.6 to 555.9)	88.9 (-14.5 to 240.1)	318.6 (92 to 640.9)	98.2 (-9.7 to 253.4)
	Prostate cancer	Male	571 (404 to 836)	159.3 (113.4 to 236)	27 (19 to 40)	9.7 (6.7 to 14.8)	1602 (1243 to 2053)	192.9 (150.2 to 249.2)	95 (74 to 126)	11.4 (8.9 to 15.1)	180.5 (91.3 to 307.7)	21.1 (-16.5 to 76.5)	254.5 (143.6 to 419)	17.8 (-17.7 to 74.5)
	Testicular cancer	Male	15 (10 to 21)	1.5 (1.1 to 2.1)	0 (0 to 0)	0 (0 to 0)	106 (75 to 146)	11.5 (8.1 to 15.7)	2 (1 to 3)	0.2 (0.2 to 0.3)	611 (339.4 to 1090)	644.6 (359.6 to 1156.1)	822.5 (486.7 to 1432.2)	669.2 (398.4 to 1156.1)
	Kidney cancer	Both	383 (292 to 490)	28.7 (21.1 to 36.5)	9 (6 to 11)	1 (0.6 to 1.2)	728 (611 to 845)	40.8 (34.2 to 47.2)	26 (22 to 30)	1.5 (1.2 to 1.8)	89.9 (33.1 to 159.4)	42.4 (4.3 to 107.7)	195.7 (114.8 to 344.4)	57.2 (12.4 to 154.3)
		Female	184 (139 to 255)	27.3 (20.9 to 35.6)	4 (3 to 5)	0.9 (0.6 to 1.2)	302 (250 to 365)	34.6 (28.7 to 41.9)	10 (8 to 13)	1.2 (1 to 1.5)	64.5 (8.6 to 136)	26.8 (-10.9 to 72.7)	160.2 (80.2 to 277.1)	33.3 (-9.1 to 128.8)
		Male	200 (130 to 287)	29.9 (19.1 to 42.7)	5 (3 to 7)	1 (0.6 to 1.4)	426 (332 to 530)	47.3 (37.2 to 58.7)	16 (12 to 20)	1.8 (1.4 to 2.3)	113.2 (34.4 to 264.7)	58.3 (0.4 to 174.3)	224.7 (106 to 464.8)	81.1 (15.3 to 220.8)
	Bladder cancer	Both	396 (282 to 518)	48.9 (35.4 to 63.5)	16 (11 to 21)	2.4 (1.7 to 3.1)	909 (752 to 1095)	51.7 (42.8 to 62.2)	43 (35 to 52)	2.5 (2.1 to 3.1)	129.8 (61.1 to 245.6)	5.8 (-24.7 to 58.4)	172.3 (92.5 to 317)	5.7 (-23.8 to 58.1)
		Female	73 (50 to 101)	20 (13.9 to 27.5)	3 (2 to 4)	1.1 (0.7 to 1.5)	166 (133 to 206)	19.2 (15.4 to 23.9)	9 (7 to 11)	1.1 (0.9 to 1.3)	128.5 (48.1 to 263.5)	-4.3 (-36.3 to 50.9)	193 (89.5 to 367)	0.3 (-34.8 to 61.2)
		Male	323 (220 to 444)	75.5 (51.8 to 101)	13 (9 to 18)	3.7 (2.5 to 4.9)	743 (590 to 924)	85.2 (68 to 106.2)	34 (27 to 42)	4 (3.2 to 5)	130.1 (52.1 to 270.7)	13 (-23.4 to 83.2)	167.4 (76.2 to 334.1)	9.1 (-25.8 to 74.9)
	Brain and central nervous system cancer	Both	2529 (1437 to 3769)	158.6 (102.9 to 225.7)	47 (31 to 66)	3.9 (2.8 to 5.3)	2561 (1600 to 3178)	142.1 (89.8 to 175.7)	70 (44 to 87)	3.9 (2.5 to 4.9)	1.3 (-46.2 to 42.7)	-10.4 (-49.3 to 22.8)	49 (-17.4 to 103.6)	0.8 (-40.6 to 40)
		Female	1063 (437 to 1967)	135.1 (64.7 to 233.3)	19 (9 to 33)	3.3 (1.9 to 5.4)	1135 (690 to 1468)	128.7 (78.6 to 166.1)	32 (19 to 41)	3.6 (2.2 to 4.6)	6.8 (-52.4 to 125.7)	-4.7 (-53.7 to 81.1)	64.1 (-20.3 to 214.2)	9.7 (-42.6 to 94.2)
		Male	1466 (723 to 2460)	180.5 (97.8 to 291.9)	28 (16 to 45)	4.5 (2.8 to 6.7)	1426 (781 to 1941)	155.6 (86.3 to 210.8)	39 (21 to 52)	4.3 (2.3 to 5.8)	-2.7 (-52.3 to 61.7)	-13.8 (-54.9 to 40)	38.7 (-29.9 to 121.7)	-4.2 (-47.7 to 54)

Province	Cause	Sex	1990				2019				1990-2019 percent change (%)			
			DALYs		Death		DALYs		Death		DALYs		Death	
			Number	Rate	Number	Rate	Number	Rate	Number	Rate	Number	Rate	Number	Rate
	Thyroid cancer	Both	95 (75 to 121)	10 (7.9 to 12.9)	3 (2 to 4)	0.4 (0.3 to 0.5)	299 (177 to 367)	16.2 (9.7 to 19.7)	10 (7 to 12)	0.6 (0.4 to 0.7)	214.3 (61.3 to 354.7)	61.6 (-14.5 to 132)	235.9 (97.4 to 385.1)	46.2 (-16.7 to 111.2)
		Female	53 (38 to 73)	12 (8.8 to 16.9)	2 (1 to 2)	0.5 (0.4 to 0.8)	147 (89 to 188)	15.9 (9.8 to 20.2)	5 (3 to 6)	0.6 (0.4 to 0.7)	177.8 (45.5 to 329.8)	32.3 (-30.2 to 103.6)	196.5 (66.6 to 359.8)	19.1 (-35.9 to 87.2)
		Male	42 (30 to 58)	8.1 (5.8 to 11.3)	1 (1 to 2)	0.3 (0.2 to 0.4)	152 (84 to 197)	16.4 (9.2 to 21)	5 (3 to 6)	0.6 (0.3 to 0.7)	259.9 (87.5 to 469)	102.1 (4.3 to 218.8)	288 (109.7 to 517.6)	91.1 (0.4 to 202)
	Mesothelioma	Both	9 (5 to 16)	0.9 (0.6 to 1.6)	0 (0 to 0)	0 (0 to 0.1)	30 (24 to 37)	1.5 (1.2 to 1.9)	1 (1 to 1)	0 (0 to 0.1)	211.3 (78.2 to 477.6)	58 (-10.8 to 191.4)	244 (90.6 to 558.1)	59.4 (-10.8 to 197.3)
		Female	6 (3 to 9)	1.3 (0.8 to 1.9)	0 (0 to 0)	0 (0 to 0.1)	9 (6 to 12)	1 (0.7 to 1.3)	0 (0 to 0)	0 (0 to 0)	57.7 (-11.9 to 187.3)	-23.1 (-55.7 to 35.2)	71.7 (3.1 to 199.2)	-23 (-51.4 to 27)
		Male	3 (1 to 10)	0.7 (0.3 to 1.9)	0 (0 to 0)	0 (0 to 0.1)	20 (15 to 27)	2 (1.5 to 2.8)	1 (0 to 1)	0.1 (0 to 0.1)	474.8 (92.9 to 1693.4)	205.6 (-0.8 to 842.9)	557.2 (103.5 to 1959.1)	216.1 (-2.7 to 872.3)
	Hodgkin lymphoma	Both	219 (133 to 425)	15.6 (9.6 to 29.4)	5 (3 to 9)	0.4 (0.3 to 0.8)	291 (201 to 357)	15.8 (11 to 19.3)	7 (5 to 9)	0.4 (0.3 to 0.5)	32.9 (-31.1 to 141.1)	1.4 (-47.4 to 81)	53.2 (-18.5 to 172.1)	-6.7 (-51 to 67.3)
		Female	67 (36 to 138)	10.5 (5.7 to 21.5)	2 (1 to 3)	0.3 (0.2 to 0.6)	101 (69 to 133)	11.3 (7.6 to 14.8)	2 (2 to 3)	0.3 (0.2 to 0.4)	49.9 (-27 to 211.1)	7.4 (-48.7 to 127)	64.7 (-18.5 to 238.8)	-10.6 (-55.4 to 84.5)
		Male	152 (86 to 329)	20.4 (11.9 to 42.5)	3 (2 to 6)	0.5 (0.3 to 1)	190 (116 to 249)	20.2 (12.6 to 26.5)	5 (3 to 6)	0.5 (0.3 to 0.7)	25.4 (-39.6 to 144.1)	-0.7 (-51.5 to 89.3)	47.7 (-27 to 180.5)	-3.3 (-52.7 to 82.8)
	Non-Hodgkin lymphoma	Both	725 (554 to 918)	54.4 (43.2 to 67)	17 (13 to 20)	1.6 (1.3 to 2)	1205 (1021 to 1400)	66.1 (56 to 76.6)	36 (31 to 42)	2 (1.7 to 2.3)	66.1 (24.4 to 134)	21.5 (-7.8 to 64.9)	116.8 (66.5 to 190.7)	24.7 (-3.2 to 65)
		Female	251 (173 to 352)	40.1 (27.5 to 53.7)	6 (4 to 8)	1.2 (0.8 to 1.6)	441 (356 to 549)	49.2 (39.9 to 61)	14 (11 to 17)	1.5 (1.3 to 1.9)	75.3 (11 to 183.8)	22.6 (-18.8 to 99.5)	130.1 (57 to 285.4)	23.3 (-12.6 to 113.7)
		Male	474 (345 to 621)	67.8 (51.2 to 88.4)	11 (8 to 14)	2 (1.5 to 2.6)	764 (623 to 920)	82.9 (67.6 to 100.1)	22 (18 to 27)	2.5 (2 to 3)	61.2 (14.8 to 139.3)	22.2 (-12.7 to 74.9)	109.5 (48.8 to 196.7)	27.1 (-9.6 to 79.1)
	Multiple myeloma	Both	196 (148 to 277)	21.7 (16.3 to 30.7)	7 (5 to 10)	0.9 (0.7 to 1.3)	463 (322 to 574)	25.4 (17.8 to 31.3)	18 (13 to 22)	1 (0.8 to 1.2)	135.9 (34.4 to 257.8)	16.8 (-33.2 to 76.7)	153.8 (50.5 to 285.7)	15.4 (-30.9 to 75.3)
		Female	85 (59 to 115)	20.2 (14 to 27)	3 (2 to 4)	0.8 (0.6 to 1.2)	176 (116 to 230)	19.2 (12.8 to 24.9)	7 (5 to 9)	0.8 (0.6 to 1)	107.9 (30.2 to 232.8)	-4.8 (-39.5 to 54.8)	122.8 (48 to 260.3)	-6.9 (-37.2 to 51.7)
		Male	112 (74 to 184)	23.2 (15.6 to 38.7)	4 (3 to 7)	0.9 (0.6 to 1.6)	287 (190 to 380)	31.6 (21.2 to 42)	11 (8 to 14)	1.3 (0.9 to 1.7)	157.1 (27.4 to 341.8)	36.1 (-33.1 to 131)	178.3 (39.6 to 370.3)	36 (-31.5 to 129.6)
	Leukemia	Both	6989 (4324 to 9958)	399.5 (261.4 to 543.2)	119 (79 to 161)	9.4 (6.6 to 12.1)	4287 (2779 to 5370)	247.2 (159.9 to 308.8)	119 (82 to 146)	6.9 (4.7 to 8.4)	-38.7 (-57 to -10.1)	-38.1 (-54.9 to -11.9)	-0.1 (-27.6 to 38.3)	-27 (-44.7 to -1)
		Female	3416 (1310 to 5100)	388.3 (188.1 to 550.9)	55 (28 to 77)	8.6 (5.1 to 11.3)	1759 (961 to 2225)	205.7 (113.7 to 259.1)	48 (29 to 60)	5.5 (3.4 to 6.9)	-48.5 (-66.8 to -1.6)	-47 (-64.2 to -12.8)	-13.4 (-40.1 to 41.9)	-35.3 (-52.6 to -5.6)
		Male	3573 (1520 to 5470)	408.9 (202.9 to 594.2)	64 (33 to 93)	10.2 (6.3 to 14.3)	2528 (1458 to 3355)	288.6 (167.4 to 385.4)	72 (44 to 94)	8.2 (5 to 10.7)	-29.3 (-54 to 22.7)	-29.4 (-52.7 to 10.8)	11.3 (-25.6 to 81.6)	-19.5 (-44.5 to 18.1)
	Other malignant neoplasms	Both	2407 (1928 to 2934)	169.8 (135.3 to 207.3)	51 (41 to 63)	5.1 (4 to 6.4)	3147 (2170 to 3831)	180.6 (124.6 to 219.8)	100 (69 to 124)	5.8 (4 to 7.2)	30.7 (-11.1 to 72.7)	6.4 (-25.6 to 41.7)	94.5 (35.2 to 169.5)	13.1 (-19.5 to 64.3)
		Female	979 (779 to 1209)	139.5 (110.9 to 173.8)	20 (16 to 25)	4.1 (3.2 to 5.3)	1253 (1015 to 1507)	146.7 (119.5 to 175.6)	39 (31 to 47)	4.6 (3.7 to 5.6)	27.9 (-4.4 to 74.3)	5.1 (-20.8 to 45.4)	93.9 (44.6 to 176.5)	11.2 (-19.3 to 66.6)
		Male	1428 (957 to 1929)	197.6 (137.6 to 265.6)	31 (22 to 43)	6.1 (4.2 to 8.2)	1894 (1060 to 2507)	215.1 (121.6 to 284)	61 (33 to 81)	7.1 (3.8 to 9.4)	32.6 (-27.2 to 103.8)	8.8 (-36.9 to 70.4)	95 (9.2 to 205.2)	16.2 (-32.6 to 88)
	Other neoplasms	Both	58 (41 to 72)	5.4 (3.6 to 6.8)	1 (1 to 2)	0.2 (0.1 to 0.3)	80 (66 to 105)	4.6 (3.8 to 6)	3 (2 to 4)	0.2 (0.1 to 0.2)	37.3 (4.6 to 99.4)	-15.5 (-36.7 to 26.9)	82.7 (23.5 to 217.1)	-18.1 (-44.9 to 46.6)
		Female	26 (17 to 37)	5 (3 to 6.8)	1 (0 to 1)	0.2 (0.1 to 0.2)	35 (28 to 43)	4 (3.3 to 4.9)	1 (1 to 1)	0.1 (0.1 to 0.2)	32.7 (-10.5 to 112.9)	-20.4 (-45.2 to 39.9)	81.9 (13.8 to 291.6)	-24 (-53.5 to 86)

Province	Cause	Sex	1990				2019				1990-2019 percent change (%)			
			DALYs		Death		DALYs		Death		DALYs		Death	
			Number	Rate	Number	Rate	Number	Rate	Number	Rate	Number	Rate	Number	Rate
		Male	32 (21 to 42)	5.8 (3.8 to 7.7)	1 (1 to 1)	0.2 (0.1 to 0.3)	45 (34 to 68)	5.2 (4 to 7.9)	2 (1 to 3)	0.2 (0.1 to 0.3)	41 (-4.4 to 122)	-10.4 (-38.3 to 35.7)	83.3 (13.3 to 219.1)	-12.9 (-45.1 to 42.5)
	Cardiovascular diseases	Both	88005 (74898 to 102664)	10413.3 (8915.7 to 12102.3)	3183 (2709 to 3735)	523.5 (446.7 to 601.9)	108656 (95673 to 122088)	6210.5 (5480.4 to 6971.9)	5500 (4768 to 6178)	328.3 (285.1 to 368.5)	23.5 (2.7 to 50)	-40.4 (-50.4 to -28.2)	72.8 (41.6 to 110.1)	-37.3 (-47 to -25.4)
		Female	35069 (28805 to 43223)	9120.1 (7500.5 to 11048.2)	1322 (1075 to 1618)	496.7 (408.7 to 592.3)	44861 (39138 to 51789)	5311.4 (4637.1 to 6126.3)	2496 (2141 to 2877)	319.1 (272.8 to 366.2)	27.9 (-0.1 to 62.1)	-41.8 (-54.1 to -27.2)	88.8 (47.7 to 138)	-35.8 (-48.6 to -20.3)
		Male	52936 (42009 to 66010)	11538.3 (9282.4 to 14371.2)	1861 (1482 to 2341)	541.3 (437.4 to 665.8)	63795 (53617 to 75402)	7169 (6028.6 to 8474.9)	3003 (2494 to 3524)	345.7 (287.9 to 405.6)	20.5 (-9.6 to 60.8)	-37.9 (-53 to -17.6)	61.4 (20.2 to 117.2)	-36.1 (-51.3 to -16.4)
	Rheumatic heart disease	Both	2030 (1578 to 2599)	128.5 (102.4 to 165.5)	32 (25 to 42)	2.9 (2.2 to 4.1)	1051 (845 to 1312)	57.2 (46.2 to 71.1)	21 (17 to 25)	1.2 (1 to 1.4)	-48.2 (-62.1 to -32.4)	-55.5 (-67.8 to -42.7)	-33.5 (-57.4 to -7.7)	-58.1 (-74.7 to -39.6)
		Female	1093 (791 to 1452)	142.3 (107.5 to 183.9)	17 (12 to 23)	3.3 (2.3 to 4.9)	523 (406 to 669)	57.8 (45.3 to 73.3)	11 (8 to 14)	1.3 (1 to 1.6)	-52.1 (-65.7 to -29.6)	-59.4 (-70.9 to -41.3)	-36.6 (-59.2 to 2.2)	-60.8 (-77.3 to -36)
		Male	936 (617 to 1332)	115 (78.6 to 161.9)	15 (9 to 22)	2.5 (1.6 to 4)	528 (408 to 677)	56.7 (44.1 to 72.4)	10 (8 to 13)	1.2 (0.9 to 1.5)	-43.6 (-63.6 to -10.4)	-50.7 (-68.2 to -22.9)	-29.8 (-60.7 to 25.7)	-53.7 (-75 to -19.1)
	Ischemic heart disease	Both	51781 (43235 to 61967)	6456.8 (5486 to 7622.5)	2043 (1730 to 2424)	335.5 (287.7 to 389.5)	66990 (58321 to 75999)	3806.8 (3322.3 to 4313.2)	3547 (3063 to 3978)	211.3 (182.1 to 237.3)	29.4 (4.8 to 62.3)	-41 (-51.8 to -27.3)	73.6 (41.2 to 114.8)	-37 (-47.7 to -24.1)
		Female	18082 (14314 to 22900)	5122.3 (4119.4 to 6279.8)	777 (625 to 957)	297.6 (241.2 to 356.8)	25095 (21420 to 29201)	2980.9 (2543.2 to 3454.7)	1524 (1291 to 1756)	195.5 (165.5 to 224.7)	38.8 (5.7 to 83.6)	-41.8 (-54.7 to -25.1)	96 (52.2 to 152.6)	-34.3 (-47.5 to -17)
		Male	33699 (26415 to 42879)	7658.6 (6047.7 to 9637.9)	1265 (997 to 1601)	367.3 (296.7 to 455.2)	41896 (34754 to 49807)	4671 (3882.4 to 5550.8)	2023 (1675 to 2378)	232 (191.7 to 273.1)	24.3 (-9.4 to 69.8)	-39 (-54.6 to -17.3)	59.9 (18.3 to 116.6)	-36.8 (-52.1 to -16.7)
	Stroke	Both	22668 (18380 to 26813)	2718.4 (2187.3 to 3218.7)	795 (618 to 948)	135.6 (104.1 to 160.6)	25436 (22060 to 28577)	1463.3 (1266.4 to 1641.5)	1219 (1017 to 1379)	73.1 (60.7 to 82.6)	12.2 (-9.2 to 36.3)	-46.2 (-56.9 to -34.2)	53.4 (18.7 to 92)	-46.1 (-57.3 to -33.5)
		Female	10547 (8506 to 13065)	2746.3 (2204.6 to 3412.2)	376 (294 to 480)	142.7 (111.1 to 178.8)	12308 (10507 to 14368)	1443.6 (1234.7 to 1677.5)	610 (505 to 717)	77.4 (64 to 91.1)	16.7 (-9.5 to 48.8)	-47.4 (-59.5 to -32)	62.1 (20.3 to 120.1)	-45.8 (-59 to -26.5)
		Male	12121 (9022 to 15246)	2663.2 (1949.3 to 3348.6)	419 (292 to 540)	126.3 (85.8 to 160.7)	13128 (10692 to 15617)	1497.5 (1216.9 to 1782.8)	609 (475 to 728)	70.9 (55.1 to 84.7)	8.3 (-19.4 to 44.6)	-43.8 (-58.4 to -25)	45.5 (2.6 to 101.9)	-43.9 (-59.2 to -23.6)
	Hypertensive heart disease	Both	4165 (3202 to 5928)	579.1 (449.3 to 776.5)	178 (136 to 243)	33.2 (25.3 to 44.1)	7541 (6423 to 9067)	438 (373.6 to 524.2)	423 (352 to 499)	25.5 (21.2 to 30.1)	81.1 (36.8 to 144.1)	-24.4 (-43.1 to 0.3)	137.4 (73.7 to 221.2)	-23.2 (-44.6 to 2.4)
		Female	1952 (1388 to 2863)	599.2 (430.6 to 896.6)	89 (63 to 137)	36.5 (26 to 56.6)	3601 (2900 to 4486)	430.1 (345.9 to 529.4)	218 (171 to 265)	27.9 (21.8 to 33.8)	84.5 (13.9 to 172.4)	-28.2 (-58.5 to 4.6)	145.2 (37.4 to 269.8)	-23.5 (-59.2 to 14.7)
		Male	2213 (1552 to 3341)	554.3 (398.9 to 806.5)	89 (62 to 132)	29.3 (20.8 to 41.6)	3940 (3149 to 4955)	450.4 (360.1 to 568.2)	204 (162 to 258)	23.7 (18.9 to 29.9)	78.1 (21.5 to 161.6)	-18.7 (-42.9 to 16.1)	129.6 (59.3 to 236.6)	-19.2 (-41.9 to 16)
	Non-rheumatic valvular heart disease	Both	428 (330 to 550)	42.2 (33.8 to 51.7)	13 (10 to 16)	1.7 (1.4 to 2.1)	680 (570 to 790)	37.3 (31.4 to 43.4)	27 (23 to 32)	1.6 (1.3 to 1.8)	59 (17.2 to 117.3)	-11.6 (-32.5 to 17)	113.4 (61.8 to 178.6)	-10 (-29 to 14.2)
		Female	197 (137 to 279)	43 (31.6 to 57.2)	6 (5 to 8)	1.9 (1.5 to 2.5)	300 (247 to 367)	33.6 (27.9 to 41)	13 (11 to 16)	1.6 (1.3 to 1.9)	51.9 (0.2 to 136.7)	-21.9 (-45.7 to 15.3)	110.2 (47.9 to 204.5)	-17.9 (-42.5 to 13.1)
		Male	230 (160 to 325)	41.2 (29.8 to 56.9)	6 (5 to 9)	1.5 (1.1 to 2.1)	380 (294 to 472)	41.1 (32.1 to 51)	14 (11 to 17)	1.6 (1.2 to 2)	65 (5.8 to 154.4)	-0.2 (-35.6 to 49.7)	116.5 (41.1 to 222.3)	2.9 (-31.1 to 50.3)

Province	Cause	Sex	1990				2019				1990-2019 percent change (%)			
			DALYs		Death		DALYs		Death		DALYs		Death	
			Number	Rate	Number	Rate	Number	Rate	Number	Rate	Number	Rate	Number	Rate
	Cardiomyopathy and myocarditis	Both	1286 (833 to 2525)	81.1 (58 to 135.5)	24 (17 to 40)	2.5 (1.9 to 3.6)	868 (702 to 1147)	51.7 (41.7 to 68.9)	30 (23 to 41)	1.8 (1.4 to 2.4)	-32.5 (-66.2 to 11.1)	-36.3 (-62.4 to -5.7)	26.3 (-29 to 83.2)	-26.6 (-50.7 to 0)
		Female	550 (312 to 1199)	70.9 (45.5 to 125.2)	10 (7 to 18)	2.3 (1.5 to 3.8)	303 (228 to 414)	37.7 (27.7 to 51)	11 (8 to 15)	1.4 (1 to 1.9)	-44.9 (-75 to 6.1)	-46.8 (-70 to -10.3)	11.2 (-40 to 87)	-38.3 (-63.2 to 1.6)
		Male	735 (452 to 1442)	90.5 (60.4 to 159.7)	14 (9 to 24)	2.6 (1.8 to 4)	565 (422 to 796)	65.7 (49.5 to 92.5)	19 (14 to 29)	2.2 (1.6 to 3.3)	-23.2 (-62.7 to 32.2)	-27.4 (-59.7 to 14.5)	37.5 (-27.6 to 123.5)	-15 (-46.6 to 23.1)
	Atrial fibrillation and flutter	Both	415 (315 to 537)	71.2 (54 to 90.4)	10 (7 to 13)	2.8 (1.9 to 3.5)	1266 (997 to 1578)	75.5 (59.7 to 94.6)	51 (42 to 59)	3.2 (2.6 to 3.7)	205.2 (165.9 to 256.4)	6 (-7.7 to 25.5)	392.5 (274.1 to 589)	11.4 (-14.7 to 59.4)
		Female	221 (161 to 289)	83.4 (60.2 to 106.9)	7 (4 to 9)	3.8 (2.3 to 4.9)	685 (545 to 855)	84.3 (67.5 to 105)	29 (23 to 35)	3.9 (3.1 to 4.7)	209.2 (160.9 to 301.3)	1.1 (-15.6 to 34.8)	320.4 (204.4 to 657)	3 (-25.4 to 85.8)
		Male	193 (138 to 259)	58.6 (43.1 to 77.8)	4 (2 to 5)	1.8 (1.2 to 2.5)	581 (437 to 737)	67.6 (50.6 to 85.9)	23 (14 to 28)	2.6 (1.6 to 3.2)	200.7 (141.7 to 269)	15.4 (-5.2 to 38.2)	528.8 (275.2 to 873.3)	41.6 (-9.5 to 111.2)
	Aortic aneurysm	Both	175 (125 to 244)	20.1 (14.4 to 27.4)	7 (5 to 9)	1 (0.7 to 1.3)	306 (253 to 364)	17.1 (14.2 to 20.5)	14 (12 to 17)	0.8 (0.7 to 1)	74.4 (15.4 to 170.6)	-14.7 (-42.4 to 30)	120.5 (48.3 to 237.9)	-11.8 (-39.4 to 30.4)
		Female	42 (32 to 56)	10.9 (8.4 to 14.3)	2 (1 to 2)	0.6 (0.4 to 0.7)	79 (64 to 96)	8.8 (7.2 to 10.7)	4 (3 to 4)	0.4 (0.4 to 0.5)	87.2 (32 to 162)	-19.1 (-42 to 10.9)	118.6 (56.4 to 195.8)	-23 (-43.8 to 4.8)
		Male	133 (85 to 202)	28.6 (18.4 to 42.7)	5 (3 to 7)	1.3 (0.9 to 1.9)	227 (179 to 285)	25.5 (20 to 32)	11 (8 to 13)	1.2 (1 to 1.5)	70.4 (1.8 to 205.4)	-10.9 (-46.6 to 57.4)	121.1 (33.8 to 297.9)	-6.7 (-43.1 to 64.9)
	Peripheral artery disease	Both	40 (22 to 67)	6.4 (3.6 to 10.5)	0 (0 to 1)	0.1 (0.1 to 0.2)	374 (199 to 489)	22.3 (11.8 to 29.4)	19 (8 to 25)	1.2 (0.5 to 1.5)	830.5 (383.8 to 1516.8)	250.4 (74.7 to 503.5)	5141.4 (1502.8 to 8857.9)	1301.8 (279 to 2308.1)
		Female	22 (12 to 38)	7.5 (4.1 to 12.9)	0 (0 to 0)	0.1 (0 to 0.2)	189 (45 to 271)	23 (5.5 to 33)	10 (1 to 15)	1.2 (0.1 to 1.9)	752.3 (149.4 to 1581.2)	205.4 (-11.9 to 488.8)	5773.2 (234.5 to 14228.8)	1371.6 (-17.3 to 3689.7)
		Male	18 (10 to 29)	5.2 (3 to 8.4)	0 (0 to 0)	0.1 (0.1 to 0.1)	184 (139 to 236)	21.7 (16.2 to 28)	9 (7 to 12)	1.1 (0.8 to 1.4)	927.3 (537.2 to 1599.6)	312.6 (159.1 to 580.1)	4614.3 (2945.1 to 7735.8)	1270.4 (805.9 to 2103.1)
	Endocarditis	Both	419 (251 to 631)	26 (19.2 to 36.7)	8 (6 to 11)	0.7 (0.5 to 1.2)	287 (223 to 431)	16.1 (12.6 to 23.9)	10 (8 to 16)	0.6 (0.4 to 0.9)	-31.4 (-60.1 to 44.7)	-37.9 (-57.1 to -10.3)	32.1 (-9.9 to 95.5)	-21.7 (-38.2 to 1.8)
		Female	218 (97 to 374)	26.3 (17.3 to 40.2)	4 (3 to 6)	0.8 (0.5 to 1.3)	120 (88 to 198)	14.1 (10.3 to 22.5)	5 (3 to 8)	0.6 (0.4 to 1)	-44.7 (-72.7 to 59.1)	-46.2 (-67.7 to -10.5)	28 (-23.8 to 110.9)	-22.6 (-44.1 to 10.4)
		Male	201 (126 to 345)	25.7 (16.6 to 43.2)	4 (3 to 6)	0.7 (0.5 to 1.2)	167 (120 to 262)	18.2 (13.1 to 27.6)	5 (4 to 9)	0.6 (0.4 to 1)	-17 (-53.1 to 51.4)	-29.2 (-54.6 to 11.7)	36.1 (-12 to 114.9)	-18.2 (-42.3 to 16.4)
	Other cardiovascular and circulatory diseases	Both	4599 (3664 to 5769)	283.5 (234.6 to 346.3)	74 (60 to 92)	7.5 (6.1 to 10.2)	3857 (3324 to 4428)	225.2 (194.4 to 257)	137 (115 to 159)	8.2 (6.8 to 9.5)	-16.1 (-35 to 7.3)	-20.6 (-38.9 to -1.2)	85.9 (35.7 to 139.3)	8.6 (-26.9 to 41.6)
		Female	2144 (1564 to 2894)	267.8 (208.6 to 352.2)	33 (25 to 45)	7.1 (5.4 to 11.2)	1659 (1387 to 1968)	197.5 (166.2 to 233.1)	62 (50 to 74)	7.8 (6.3 to 9.4)	-22.6 (-45.9 to 8.2)	-26.3 (-46.9 to -2.8)	85.2 (19.1 to 159.5)	9.6 (-38.4 to 53)
		Male	2455 (1890 to 3307)	297.4 (234.4 to 399.7)	40 (31 to 55)	7.9 (6 to 11.3)	2198 (1826 to 2592)	253.6 (212.3 to 298.2)	75 (60 to 91)	8.7 (7 to 10.6)	-10.5 (-34.3 to 20.7)	-14.7 (-38.5 to 13.2)	86.5 (24.7 to 160.8)	10.5 (-28.3 to 55.8)
	Chronic respiratory diseases	Both	11396 (9422 to 14048)	1102.8 (922.7 to 1359.3)	253 (204 to 338)	40.1 (32.3 to 54.7)	14562 (12617 to 16706)	829.7 (718.9 to 951.6)	452 (377 to 547)	27 (22.5 to 32.7)	27.8 (7.1 to 49)	-24.8 (-37.2 to -11.8)	78.5 (40.4 to 127.1)	-32.7 (-47.3 to -14.9)
		Female	4685 (3608 to 6300)	943.2 (731.1 to 1320.1)	96 (65 to 154)	34.1 (23.2 to 55.1)	5741 (4892 to 7247)	666 (565.9 to 839.9)	166 (133 to 241)	20.8 (16.7 to 30.3)	22.5 (0 to 65.2)	-29.4 (-44.4 to -5.8)	72.9 (21.6 to 182.3)	-38.9 (-57.7 to -1.1)

Province	Cause	Sex	1990				2019				1990-2019 percent change (%)			
			DALYs		Death		DALYs		Death		DALYs		Death	
			Number	Rate	Number	Rate	Number	Rate	Number	Rate	Number	Rate	Number	Rate
		Male	6710 (5391 to 8313)	1244.4 (1005 to 1568)	158 (119 to 214)	45.5 (35 to 61.9)	8821 (7434 to 10262)	999 (841.8 to 1164.6)	287 (230 to 347)	33.4 (26.8 to 40.3)	31.5 (3.2 to 65.7)	-19.7 (-38.1 to 3.5)	81.9 (28.4 to 155.8)	-26.6 (-47.7 to 2.3)
	Chronic obstructive pulmonary disease	Both	4650 (3852 to 6181)	528.2 (435.6 to 733.9)	127 (98 to 195)	21.3 (16.4 to 34.5)	9482 (8284 to 11024)	537.3 (469.1 to 631)	338 (278 to 426)	20.2 (16.6 to 25.6)	103.9 (62.2 to 145.7)	1.7 (-19.1 to 23)	165.2 (97.4 to 246.1)	-5.3 (-31.7 to 23.3)
		Female	1864 (1339 to 2965)	450.2 (325.1 to 765.6)	48 (28 to 98)	18.4 (10.9 to 38.8)	3556 (3009 to 4797)	411.2 (346.2 to 556.6)	121 (93 to 190)	15.3 (11.8 to 24.3)	90.8 (43.6 to 185.8)	-8.7 (-32.5 to 35.3)	152.7 (69.3 to 370.5)	-16.4 (-44 to 45.6)
		Male	2786 (2207 to 3644)	596.9 (471.4 to 787.6)	79 (58 to 113)	24 (17.9 to 34.3)	5926 (4989 to 6931)	668.8 (565.1 to 782.4)	216 (173 to 263)	25.2 (20.1 to 30.7)	112.7 (56 to 175.3)	12 (-18 to 47.4)	172.8 (84.7 to 293.2)	5.3 (-28.5 to 51.2)
	Pneumoconiosis	Both	16 (10 to 22)	1.7 (1.1 to 2.4)	1 (0 to 1)	0.1 (0 to 0.1)	108 (85 to 137)	6 (4.8 to 7.6)	4 (3 to 5)	0.2 (0.2 to 0.3)	596.4 (359.3 to 1074)	255.3 (134.7 to 492.1)	685.7 (394.1 to 1355.1)	238.9 (117.6 to 519.1)
		Female	2 (2 to 3)	0.4 (0.3 to 0.6)	0 (0 to 0)	0 (0 to 0)	25 (18 to 34)	2.7 (2 to 3.7)	0 (0 to 1)	0.1 (0 to 0.1)	1058.8 (778.7 to 1444.7)	514.8 (369.1 to 693.7)	1145 (652 to 2063.6)	375.1 (181.2 to 711.6)
		Male	13 (8 to 20)	2.8 (1.7 to 4.2)	0 (0 to 1)	0.1 (0.1 to 0.2)	83 (63 to 110)	9.3 (7.1 to 12.3)	4 (3 to 5)	0.4 (0.3 to 0.5)	520.9 (283.4 to 1073.6)	228 (104.7 to 504.9)	654.6 (358.6 to 1368)	229.2 (103.2 to 543.3)
	Asthma	Both	6411 (5042 to 8018)	549.5 (428.9 to 675.2)	122 (90 to 158)	18.2 (12.9 to 24.7)	4214 (3302 to 5336)	244.3 (191 to 312.4)	96 (80 to 115)	5.7 (4.7 to 6.8)	-34.3 (-44.3 to -22.9)	-55.5 (-64.9 to -45.4)	-20.8 (-42.1 to 8.4)	-68.6 (-77.7 to -56.6)
		Female	2667 (1961 to 3506)	469.9 (335.9 to 635.6)	46 (27 to 69)	15.3 (8.2 to 24.5)	1832 (1367 to 2369)	214.8 (160.3 to 279.4)	39 (24 to 51)	4.8 (2.9 to 6.2)	-31.3 (-45.8 to -13.6)	-54.3 (-66.8 to -40.9)	-15.5 (-48.1 to 28.1)	-68.6 (-82.3 to -50.2)
		Male	3744 (2916 to 4782)	620.4 (480.6 to 795.6)	76 (54 to 104)	20.8 (14.9 to 28.9)	2382 (1831 to 3009)	274.1 (210.6 to 350.7)	57 (46 to 72)	6.7 (5.3 to 8.4)	-36.4 (-49.4 to -21)	-55.8 (-66.6 to -42)	-24 (-48.4 to 14.3)	-68 (-78.6 to -51.4)
	Interstitial lung disease and pulmonary sarcoidosis	Both	103 (77 to 143)	10.6 (7.5 to 15.3)	3 (2 to 4)	0.4 (0.2 to 0.7)	239 (179 to 295)	13.1 (9.9 to 16.2)	8 (6 to 10)	0.5 (0.3 to 0.6)	131.4 (58.9 to 223.5)	23.5 (-15.3 to 81.5)	209.9 (93.6 to 416)	22.2 (-26.2 to 121)
		Female	45 (32 to 66)	9.8 (6.5 to 16)	1 (1 to 2)	0.3 (0.2 to 0.7)	97 (71 to 138)	10.5 (7.8 to 15.2)	3 (2 to 5)	0.3 (0.2 to 0.6)	114.6 (62 to 190.5)	7.9 (-22.1 to 62.9)	163.5 (74.6 to 393.4)	-2 (-39.5 to 106.5)
		Male	58 (41 to 90)	11.4 (7.7 to 17.9)	1 (1 to 3)	0.4 (0.2 to 0.8)	143 (93 to 187)	15.7 (10.2 to 20.5)	5 (3 to 7)	0.6 (0.4 to 0.8)	144.4 (33.1 to 284.9)	37.6 (-24.7 to 128.5)	243.4 (73 to 558.6)	41.5 (-30.4 to 189.5)
	Other chronic respiratory diseases	Both	216 (144 to 449)	12.9 (9 to 24.3)	1 (1 to 5)	0.1 (0.1 to 0.3)	519 (372 to 652)	28.9 (20.6 to 36.3)	7 (2 to 11)	0.4 (0.1 to 0.6)	140.5 (-2.8 to 288.1)	124.9 (1.5 to 244.1)	357.4 (-33 to 1373.5)	192.3 (-43.8 to 704.9)
		Female	107 (71 to 241)	12.9 (9 to 25.4)	1 (0 to 3)	0.1 (0.1 to 0.3)	232 (171 to 288)	26.8 (19.5 to 33.4)	2 (1 to 4)	0.3 (0.1 to 0.5)	115.9 (-10.4 to 246.4)	107.8 (-3.1 to 216.3)	289.4 (-49.2 to 1378.2)	149.7 (-52.4 to 691.6)
		Male	108 (70 to 221)	12.9 (8.7 to 24.4)	1 (0 to 3)	0.1 (0.1 to 0.4)	287 (188 to 377)	31.1 (20.3 to 40.7)	4 (1 to 7)	0.5 (0.1 to 0.8)	164.9 (3.9 to 366.4)	141.6 (4.3 to 303.7)	406.8 (-25.8 to 1659.7)	222.3 (-42.3 to 932)
	Digestive diseases	Both	11164 (8978 to 13368)	922.2 (772.3 to 1084.7)	229 (191 to 275)	29.5 (23.7 to 37.1)	11504 (9611 to 13855)	634.8 (531.4 to 761.6)	341 (286 to 403)	20 (16.8 to 23.6)	3 (-20 to 34.8)	-31.2 (-43.7 to -14.2)	49.3 (16.5 to 97.8)	-32.3 (-48 to -11.6)
		Female	5000 (3965 to 6204)	845.8 (685.5 to 1040.6)	95 (76 to 120)	27.2 (20 to 37.6)	4809 (3920 to 5946)	542.8 (446.7 to 668.5)	136 (113 to 165)	16.9 (14.1 to 20.5)	-3.8 (-27.8 to 27.5)	-35.8 (-48.7 to -19.2)	42.9 (6 to 95.7)	-37.7 (-57 to -12.3)
		Male	6164 (4731 to 7766)	991.3 (787.1 to 1229.6)	133 (102 to 170)	31.4 (24.2 to 40.3)	6695 (5426 to 8203)	729.7 (592.9 to 891.7)	205 (162 to 253)	23.3 (18.6 to 28.9)	8.6 (-19.4 to 52.5)	-26.4 (-44.3 to 0.9)	53.9 (10.6 to 122.6)	-25.7 (-45.4 to 4.5)
	Cirrhosis and other chronic liver diseases	Both	4954 (3842 to 6191)	422.3 (344.6 to 513.2)	130 (106 to 160)	16.1 (12.6 to 20.7)	5233 (4260 to 6254)	289.2 (237 to 345.1)	202 (166 to 239)	11.7 (9.6 to 13.7)	5.6 (-22.8 to 52.6)	-31.5 (-48.3 to -6.1)	55.7 (17.6 to 111.6)	-27.5 (-46 to -2.9)

Province	Cause	Sex	1990				2019				1990-2019 percent change (%)			
			DALYs		Death		DALYs		Death		DALYs		Death	
			Number	Rate	Number	Rate	Number	Rate	Number	Rate	Number	Rate	Number	Rate
		Female	2096 (1488 to 2761)	358.2 (274.8 to 469.2)	53 (41 to 71)	14.3 (10.1 to 20.8)	1712 (1398 to 2106)	196.9 (161.3 to 241.7)	73 (59 to 90)	8.9 (7.2 to 10.9)	-18.3 (-44.6 to 27.7)	-45 (-60.1 to -21.6)	37.5 (-3.4 to 100.5)	-38.2 (-60.2 to -6.5)
		Male	2859 (2089 to 3765)	481.5 (365.5 to 624.8)	77 (58 to 100)	17.6 (13.3 to 23)	3521 (2748 to 4435)	382.9 (299.3 to 480.8)	129 (101 to 161)	14.6 (11.4 to 18.3)	23.2 (-18.8 to 92.1)	-20.5 (-46.4 to 17.5)	68.2 (14.2 to 145.9)	-17 (-41.8 to 19.2)
	Upper digestive system diseases	Both	2924 (2209 to 4006)	266.5 (203.5 to 350.3)	48 (37 to 61)	7.2 (5.3 to 9.6)	3111 (2161 to 4633)	164.1 (115.7 to 242)	48 (38 to 58)	2.9 (2.3 to 3.4)	6.4 (-15.5 to 25.2)	-38.4 (-50.4 to -26.8)	0.9 (-25.1 to 35.2)	-60.2 (-70.9 to -45)
		Female	1291 (927 to 1871)	244.6 (175.7 to 342.3)	18 (13 to 25)	6.3 (4.1 to 9.4)	1514 (990 to 2305)	161.5 (107.9 to 244.5)	20 (15 to 25)	2.5 (1.9 to 3.2)	17.2 (-7.1 to 38.3)	-34 (-48.1 to -21)	10.2 (-27.9 to 71.8)	-59.9 (-75.2 to -33.4)
		Male	1633 (1216 to 2191)	285.9 (213 to 373.5)	30 (22 to 40)	8 (5.7 to 11)	1597 (1146 to 2328)	167.5 (121.3 to 241.5)	29 (22 to 36)	3.3 (2.5 to 4.2)	-2.2 (-25 to 21.5)	-41.4 (-54.9 to -26.5)	-4.7 (-35 to 38)	-59 (-71.4 to -41.2)
	Appendicitis	Both	697 (381 to 1071)	43.3 (25.9 to 59.6)	12 (7 to 18)	1.3 (0.7 to 1.8)	277 (176 to 364)	16 (10.3 to 21)	8 (5 to 11)	0.5 (0.3 to 0.6)	-60.3 (-76.6 to -11.3)	-63.1 (-75.9 to -30.1)	-35.8 (-59.9 to 21.3)	-62.7 (-75.7 to -40.9)
		Female	288 (141 to 534)	37.6 (18.9 to 61.2)	5 (3 to 9)	1.2 (0.5 to 2)	116 (70 to 158)	14.1 (8.5 to 19.1)	4 (2 to 5)	0.4 (0.3 to 0.6)	-59.6 (-80 to -2.3)	-62.6 (-78.7 to -23.4)	-33.1 (-64.1 to 46.8)	-62.8 (-80.8 to -31.6)
		Male	409 (192 to 662)	48.6 (25.1 to 73)	7 (4 to 11)	1.3 (0.7 to 1.9)	160 (99 to 226)	17.9 (11.3 to 25)	5 (3 to 6)	0.5 (0.3 to 0.7)	-60.8 (-78.4 to 0.3)	-63.1 (-77.7 to -15)	-37.8 (-64.4 to 53.1)	-61.2 (-75.9 to -14.8)
	Paralytic ileus and intestinal obstruction	Both	1258 (724 to 2105)	66.2 (42.6 to 104.1)	18 (12 to 29)	1.7 (1.2 to 2.7)	529 (398 to 698)	35 (25.7 to 47.4)	19 (15 to 25)	1.2 (0.9 to 1.6)	-57.9 (-79.3 to -13.8)	-47.1 (-72.5 to -0.8)	5.7 (-38.1 to 79.2)	-28.9 (-55.6 to 8.1)
		Female	631 (327 to 1274)	68.6 (41.5 to 121.9)	9 (6 to 16)	1.8 (1.2 to 2.9)	230 (168 to 317)	31.7 (22.6 to 45.3)	8 (6 to 11)	1.1 (0.8 to 1.4)	-63.6 (-84.2 to -18.5)	-53.9 (-77.7 to -7.3)	-12.8 (-53.9 to 55.5)	-41.5 (-63.7 to -4.3)
		Male	627 (315 to 1134)	63.9 (36 to 109.9)	9 (5 to 16)	1.6 (1 to 2.8)	300 (201 to 416)	38.4 (25.3 to 54.4)	11 (8 to 15)	1.4 (0.9 to 1.8)	-52.2 (-78.5 to 13.5)	-39.9 (-72.3 to 29.8)	24.6 (-38 to 143.7)	-14.4 (-54.3 to 50)
	Inguinal, femoral, and abdominal hernia	Both	289 (178 to 452)	18.5 (12.4 to 26.1)	1 (1 to 3)	0.2 (0.1 to 0.3)	310 (214 to 428)	17.8 (12.3 to 24.5)	3 (2 to 4)	0.2 (0.1 to 0.2)	6.9 (-31.4 to 46.8)	-3.8 (-28.3 to 19)	95.5 (-21.9 to 307.2)	-16.3 (-47.8 to 39)
		Female	111 (61 to 227)	13.7 (8.6 to 23.6)	1 (0 to 2)	0.2 (0.1 to 0.3)	102 (68 to 148)	11.9 (8 to 17.5)	1 (0 to 1)	0.1 (0.1 to 0.2)	-8.7 (-58.9 to 46.9)	-12.9 (-52.6 to 23.3)	55.9 (-59.5 to 281.9)	-28 (-60.1 to 29.2)
		Male	178 (112 to 269)	23 (15.4 to 32.6)	1 (0 to 1)	0.2 (0.2 to 0.4)	208 (144 to 289)	23.6 (16.3 to 32.6)	2 (1 to 2)	0.2 (0.1 to 0.3)	16.7 (-16.1 to 58.9)	2.5 (-17.9 to 27.2)	123.4 (3.2 to 360.5)	-11.9 (-53 to 53.2)
	Inflammatory bowel disease	Both	101 (73 to 137)	9.4 (6.9 to 12.4)	1 (1 to 1)	0.1 (0.1 to 0.2)	209 (143 to 275)	10.8 (7.4 to 14.1)	3 (2 to 4)	0.2 (0.1 to 0.3)	107.1 (28.9 to 163.4)	15.2 (-20.2 to 43.8)	221.8 (26.4 to 399.9)	46.7 (-30.6 to 125.4)
		Female	49 (34 to 68)	9.3 (6.5 to 12.7)	0 (0 to 1)	0.1 (0.1 to 0.2)	96 (67 to 129)	10 (6.9 to 13.3)	1 (1 to 2)	0.2 (0.1 to 0.2)	97.9 (25.2 to 164.8)	8.1 (-21.6 to 42.4)	217 (27.8 to 481.7)	39.7 (-33.3 to 166.9)
		Male	52 (38 to 73)	9.5 (6.8 to 12.7)	1 (0 to 1)	0.1 (0.1 to 0.2)	113 (73 to 151)	11.7 (7.4 to 15.6)	2 (1 to 3)	0.2 (0.1 to 0.3)	115.6 (26.9 to 195.3)	22.5 (-20.8 to 66.2)	225.1 (15.9 to 471.3)	53.4 (-36.9 to 162.8)
	Vascular intestinal disorders	Both	71 (53 to 98)	9.6 (6.8 to 13.4)	3 (2 to 4)	0.5 (0.3 to 0.8)	135 (109 to 163)	7.9 (6.3 to 9.5)	7 (6 to 9)	0.4 (0.3 to 0.5)	90 (34.2 to 157.3)	-17.9 (-43.3 to 13.2)	138.2 (59.3 to 240.4)	-18.3 (-46.5 to 20.5)
		Female	35 (22 to 54)	10.6 (6 to 16.9)	2 (1 to 3)	0.6 (0.3 to 1)	66 (50 to 82)	7.7 (5.9 to 9.6)	4 (3 to 5)	0.4 (0.3 to 0.6)	87.5 (14.6 to 197.4)	-27.1 (-56.8 to 25.1)	121.3 (24.9 to 306.5)	-28.3 (-59.7 to 37.3)
		Male	36 (26 to 49)	8.6 (6.2 to 11.9)	1 (1 to 2)	0.4 (0.3 to 0.6)	70 (52 to 89)	7.9 (6 to 10.2)	4 (3 to 5)	0.4 (0.3 to 0.5)	92.4 (31.1 to 192.4)	-7.3 (-37 to 40.4)	157.8 (69.8 to 292)	-4.8 (-35.8 to 42.3)
	Gallbladder and biliary diseases	Both	549 (388 to 759)	54.9 (40.5 to 73.5)	6 (5 to 9)	1.1 (0.8 to 1.6)	1054 (752 to 1408)	57.5 (41.2 to 75.6)	25 (15 to 32)	1.5 (0.9 to 1.9)	92.1 (42.9 to 137.8)	4.8 (-20.9 to 29.5)	296.2 (104.5 to 552.9)	31 (-26.9 to 107.8)
		Female	357 (247 to 508)	72.6 (51.5 to 101.5)	3 (2 to 5)	1.3 (0.9 to 1.9)	681 (477 to 933)	74.6 (52.2 to 101.2)	14 (7 to 18)	1.8 (1 to 2.4)	90.6 (44.2 to 135.2)	2.6 (-21 to 24.8)	314.3 (100.4 to 615.9)	36.5 (-29 to 128.5)
		Male	191 (134 to 272)	37.8 (27.4 to 52.5)	3 (2 to 5)	1 (0.6 to 1.6)	372 (270 to 486)	41 (29.7 to 53.1)	11 (7 to 15)	1.3 (0.8 to 1.7)	94.8 (32 to 168)	8.4 (-25.4 to 50.3)	276.1 (85.7 to 608.7)	31.5 (-32.2 to 134.5)

Province	Cause	Sex	1990				2019				1990-2019 percent change (%)			
			DALYs		Death		DALYs		Death		DALYs		Death	
			Number	Rate	Number	Rate	Number	Rate	Number	Rate	Number	Rate	Number	Rate
	Pancreatitis	Both	166 (120 to 241)	18.2 (12.8 to 26.5)	5 (4 to 8)	0.8 (0.5 to 1.2)	266 (217 to 343)	14.6 (12 to 18.5)	11 (9 to 14)	0.6 (0.5 to 0.8)	60.1 (17.3 to 125.6)	-19.6 (-41.4 to 15.7)	115 (47.6 to 230.4)	-17.4 (-45.2 to 29.4)
		Female	69 (47 to 101)	16.9 (10.7 to 26.5)	2 (1 to 4)	0.8 (0.5 to 1.5)	119 (79 to 150)	13.7 (9 to 17.2)	6 (4 to 8)	0.7 (0.5 to 0.9)	72.3 (13.9 to 158.2)	-18.9 (-48.1 to 27.7)	146.5 (46.5 to 321.9)	-13.6 (-51.5 to 54.6)
		Male	97 (63 to 160)	19.3 (12.6 to 30.7)	3 (2 to 5)	0.7 (0.5 to 1.1)	147 (106 to 225)	15.7 (11.4 to 23.3)	5 (4 to 8)	0.6 (0.4 to 0.9)	51.5 (0.4 to 135.3)	-18.8 (-45.4 to 25.5)	88.6 (22.6 to 207.5)	-16.6 (-46.1 to 33.4)
	Other digestive diseases	Both	154 (113 to 234)	13.4 (10.6 to 18.3)	3 (2 to 5)	0.5 (0.4 to 0.8)	381 (248 to 471)	21.9 (14.6 to 27.2)	15 (10 to 18)	0.9 (0.6 to 1.1)	146.9 (7.6 to 280.9)	63.3 (-18.1 to 130.6)	343.7 (112.3 to 574.5)	70.6 (-16 to 151.5)
		Female	73 (52 to 115)	13.7 (10.1 to 19.4)	2 (1 to 2)	0.6 (0.3 to 1)	174 (105 to 225)	20.7 (12.8 to 26.9)	7 (4 to 9)	0.9 (0.5 to 1.1)	139.6 (9.4 to 282.3)	51.6 (-22.1 to 126.8)	322.9 (98.5 to 611.7)	55.8 (-25.9 to 175.8)
		Male	81 (55 to 125)	13.2 (9.5 to 19.1)	2 (1 to 3)	0.5 (0.3 to 0.7)	206 (140 to 261)	23.2 (16 to 29.5)	8 (5 to 10)	0.9 (0.6 to 1.1)	153.4 (11.8 to 337.1)	76.3 (-13.7 to 186)	363.3 (117.9 to 707.3)	92.8 (-4 to 225.3)
	Neurological disorders	Both	18777 (10012 to 31847)	1481.8 (857 to 2461.5)	153 (84 to 304)	32.3 (12.8 to 75.7)	25264 (13978 to 41952)	1413.7 (794.6 to 2335.4)	532 (219 to 1233)	32.5 (13.1 to 75.4)	34.5 (6.7 to 77)	-4.6 (-18.8 to 10.8)	247.9 (129.6 to 382.4)	0.7 (-15.2 to 37.2)
		Female	10080 (5108 to 17829)	1621.9 (886.8 to 2822)	73 (37 to 155)	31.5 (12 to 78.7)	13808 (7001 to 23936)	1569.1 (817.7 to 2714.4)	241 (93 to 568)	32.6 (12.1 to 77.3)	37 (7.8 to 78.4)	-3.3 (-19.6 to 12.3)	229.7 (103.9 to 416.6)	3.5 (-19.1 to 55.8)
		Male	8697 (5079 to 14144)	1347.7 (811 to 2168.8)	80 (44 to 157)	33.1 (13.4 to 75.8)	11456 (6789 to 18145)	1265.1 (764.6 to 1996.5)	291 (122 to 655)	32.7 (13.9 to 73.5)	31.7 (2 to 78.7)	-6.1 (-22.1 to 11.9)	264.7 (133.4 to 396.1)	-1.3 (-21.7 to 31.2)
	Alzheimer's disease and other dementias	Both	1700 (735 to 3924)	384.1 (167.6 to 879.9)	85 (20 to 240)	25.3 (6.1 to 68.8)	6448 (2826 to 14299)	391.6 (172.7 to 869.6)	417 (101 to 1122)	25.7 (6.2 to 68.8)	279.4 (227.8 to 372.5)	2 (-9.7 to 27)	388.9 (305.6 to 574.7)	1.6 (-13 to 43.6)
		Female	834 (366 to 1961)	387.8 (173.6 to 896.3)	43 (10 to 124)	25.3 (6 to 72.4)	3177 (1389 to 7103)	409.1 (180.1 to 903.4)	195 (47 to 522)	26.9 (6.5 to 71.9)	280.8 (215.3 to 407.7)	5.5 (-9.3 to 41.9)	355.6 (259.7 to 618)	6.3 (-13.5 to 68.6)
		Male	865 (370 to 2013)	380.6 (167.3 to 875.1)	43 (10 to 120)	25.2 (5.9 to 69.2)	3271 (1440 to 7043)	375.6 (167.1 to 812.2)	223 (54 to 595)	24.7 (5.9 to 66.5)	278 (201.8 to 377.5)	-1.3 (-17.2 to 20.8)	422.5 (302.6 to 620.4)	-2 (-21.7 to 31.3)
	Parkinson's disease	Both	417 (345 to 502)	69.2 (57.7 to 84.4)	19 (15 to 23)	4 (3.3 to 5.3)	1277 (1080 to 1467)	76.7 (64.6 to 88)	76 (62 to 88)	4.6 (3.8 to 5.4)	206.5 (143.5 to 287.2)	10.8 (-13.6 to 38.5)	306.6 (214 to 429.8)	15 (-13 to 49.2)
		Female	154 (122 to 197)	54.6 (43.3 to 74.4)	7 (5 to 10)	3.2 (2.4 to 5)	500 (421 to 595)	60.3 (50.8 to 72)	29 (24 to 37)	3.7 (3 to 4.7)	223.9 (148.6 to 332.3)	10.4 (-16.5 to 47.3)	315.6 (208.3 to 479.6)	14.6 (-15.6 to 59.2)
		Male	262 (200 to 336)	83.6 (64.5 to 105.4)	12 (9 to 15)	4.9 (3.6 to 6.3)	778 (604 to 931)	93.6 (72.1 to 112.3)	47 (35 to 57)	5.6 (4.1 to 6.8)	196.3 (115.4 to 311.1)	11.9 (-19.5 to 53.8)	301.2 (180.1 to 481.3)	14.6 (-20 to 62.7)
	Idiopathic epilepsy	Both	5162 (2895 to 7961)	256.5 (146.7 to 399.9)	40 (23 to 52)	2.2 (1.5 to 2.8)	2604 (1251 to 4394)	154.9 (75.1 to 262.4)	19 (16 to 23)	1.1 (0.9 to 1.3)	-49.6 (-76.3 to 8.4)	-39.6 (-72.4 to 28.4)	-52.8 (-66.9 to -17.2)	-51.8 (-64.6 to -21.9)
		Female	2510 (1204 to 3906)	255.4 (132.3 to 401)	20 (8 to 29)	2.2 (1.2 to 3.1)	1181 (568 to 2018)	144.7 (69.1 to 249.7)	8 (6 to 10)	1 (0.7 to 1.2)	-53 (-79 to 6.4)	-43.4 (-74.5 to 27.3)	-58.5 (-73.5 to -10.8)	-56.6 (-70.9 to -17.4)
		Male	2651 (1532 to 4048)	257.5 (150.5 to 402.1)	20 (12 to 27)	2.2 (1.4 to 2.8)	1423 (701 to 2378)	164.9 (79.4 to 277.2)	11 (9 to 13)	1.2 (0.9 to 1.4)	-46.3 (-75.5 to 13.2)	-36 (-71.3 to 37.3)	-47.2 (-64.1 to -7.1)	-46.8 (-62.4 to -11.3)
	Multiple sclerosis	Both	280 (194 to 462)	25.1 (17 to 42.4)	4 (2 to 8)	0.4 (0.2 to 0.9)	528 (385 to 789)	26.1 (19.1 to 38.6)	8 (5 to 15)	0.4 (0.3 to 0.8)	88.2 (45.3 to 142)	3.8 (-20 to 34.2)	125.5 (49.9 to 263.8)	12.7 (-25.9 to 90.5)
		Female	168 (120 to 242)	30.3 (21.3 to 43.8)	2 (1 to 4)	0.4 (0.2 to 0.8)	322 (253 to 404)	32.2 (25.4 to 40.2)	5 (3 to 6)	0.5 (0.4 to 0.7)	91.7 (25.1 to 161.2)	6.3 (-28.7 to 45.7)	131.1 (18.5 to 321.7)	14.2 (-39.4 to 122.9)

Province	Cause	Sex	1990				2019				1990-2019 percent change (%)			
			DALYs		Death		DALYs		Death		DALYs		Death	
			Number	Rate	Number	Rate	Number	Rate	Number	Rate	Number	Rate	Number	Rate
		Male	112 (63 to 255)	20.1 (11 to 46.8)	2 (1 to 5)	0.3 (0.1 to 1)	206 (115 to 423)	20 (11.4 to 40.5)	4 (2 to 9)	0.4 (0.2 to 0.9)	83 (25.6 to 157.1)	-0.5 (-32.5 to 35.4)	118.9 (22.7 to 253.7)	11.4 (-36.2 to 82.7)
	Motor neuron disease	Both	27 (22 to 34)	2.1 (1.7 to 2.8)	0 (0 to 1)	0 (0 to 0.1)	53 (44 to 64)	2.8 (2.4 to 3.4)	1 (1 to 2)	0.1 (0.1 to 0.1)	97 (44.6 to 157.8)	33.6 (-3.2 to 78.5)	156.3 (67 to 280.4)	46.8 (-5.9 to 119.9)
		Female	14 (11 to 18)	2.1 (1.6 to 2.9)	0 (0 to 0)	0 (0 to 0.1)	20 (17 to 24)	2.2 (1.9 to 2.6)	0 (0 to 1)	0 (0 to 0.1)	43.1 (4.2 to 87.7)	5 (-27.6 to 42.4)	82.5 (11.9 to 176.5)	9.4 (-36.5 to 70.9)
		Male	13 (10 to 17)	2.1 (1.5 to 2.9)	0 (0 to 0)	0 (0 to 0.1)	33 (26 to 41)	3.5 (2.7 to 4.3)	1 (1 to 1)	0.1 (0.1 to 0.1)	157.3 (70.4 to 275.8)	63.7 (6.7 to 145.3)	224.5 (92.7 to 450.9)	83 (8 to 213.8)
	Headache disorders	Both	10403 (2233 to 22888)	702.9 (170 to 1504.9)	--	--	13449 (3409 to 28549)	706.8 (172.9 to 1506.9)	--	--	29.3 (18.1 to 57.4)	0.5 (-4.2 to 6.9)	--	--
		Female	6088 (1224 to 13648)	856.6 (191.7 to 1877.7)	--	--	8223 (1892 to 17693)	873.3 (195.8 to 1895.1)	--	--	35.1 (22.5 to 59.2)	1.9 (-4.8 to 8.6)	--	--
		Male	4315 (994 to 9387)	555.4 (147.5 to 1172.9)	--	--	5226 (1532 to 10813)	545.7 (151.9 to 1141.1)	--	--	21.1 (6.6 to 59)	-1.8 (-8.3 to 8.1)	--	--
	Other neurological disorders	Both	789 (404 to 1338)	41.9 (24 to 67.3)	5 (3 to 6)	0.3 (0.2 to 0.4)	906 (584 to 1325)	54.7 (34.4 to 81.7)	11 (9 to 12)	0.6 (0.5 to 0.7)	14.9 (-37.6 to 147)	30.6 (-27.9 to 159.8)	124.3 (61.5 to 224.8)	80.2 (30.2 to 163.3)
		Female	311 (139 to 551)	35 (18.4 to 58.9)	1 (1 to 2)	0.2 (0.2 to 0.3)	386 (235 to 583)	47.3 (27.5 to 73.3)	4 (3 to 5)	0.5 (0.4 to 0.6)	23.9 (-37.5 to 191.2)	35 (-30.6 to 184.4)	208 (119.2 to 340.1)	105.4 (38.3 to 213.7)
		Male	478 (258 to 774)	48.4 (28.6 to 75.2)	3 (2 to 5)	0.4 (0.3 to 0.6)	521 (341 to 750)	62 (39.6 to 91.2)	7 (5 to 8)	0.7 (0.6 to 0.9)	9 (-38.7 to 117.1)	28.1 (-27 to 142.5)	91.8 (28.9 to 206.1)	69.7 (14.9 to 169.3)
	Mental disorders	Both	31557 (23047 to 41702)	2088 (1542.1 to 2754.3)	0 (0 to 0)	0 (0 to 0)	39436 (28987 to 52024)	2104.5 (1549.1 to 2773.7)	0 (0 to 0)	0 (0 to 0)	25 (17.8 to 32.4)	0.8 (-2.1 to 3.5)	170.1 (30.3 to 554.7)	73.1 (-15.9 to 346.3)
		Female	17077 (12478 to 22782)	2366.9 (1740.3 to 3144.7)	0 (0 to 0)	0 (0 to 0)	22090 (16195 to 29206)	2379.9 (1749.4 to 3144.9)	0 (0 to 0)	0 (0 to 0)	29.4 (21.7 to 37.4)	0.6 (-3.3 to 4.7)	118.9 (-4.1 to 494.8)	36.8 (-42.1 to 321.4)
		Male	14479 (10555 to 19182)	1822.6 (1333.2 to 2407)	0 (0 to 0)	0 (0 to 0)	17346 (12715 to 22858)	1836.9 (1338.7 to 2427.8)	0 (0 to 0)	0 (0 to 0)	19.8 (11.7 to 28.5)	0.8 (-3.1 to 4.8)	483.2 (61.5 to 1867.9)	358 (29.2 to 1508.7)
	Schizophrenia	Both	1980 (1453 to 2549)	159 (115.4 to 203.3)	--	--	3328 (2407 to 4263)	161.1 (117.5 to 205.2)	--	--	68.1 (52 to 85.7)	1.3 (-7 to 9.8)	--	--
		Female	921 (666 to 1196)	151.5 (109.7 to 195)	--	--	1559 (1096 to 2002)	153.1 (108.7 to 195)	--	--	69.3 (47.6 to 93.5)	1.1 (-10 to 14.4)	--	--
		Male	1060 (780 to 1374)	166.3 (121.6 to 212.7)	--	--	1769 (1280 to 2259)	168.9 (123.5 to 213.8)	--	--	67 (46.2 to 91.2)	1.5 (-9.6 to 14.4)	--	--
	Depressive disorders	Both	9246 (6048 to 13487)	678.8 (454.3 to 975.3)	--	--	13786 (9212 to 20011)	707.6 (471.2 to 1020)	--	--	49.1 (34.2 to 65.4)	4.3 (-0.9 to 9.6)	--	--
		Female	5852 (3810 to 8340)	876 (582.3 to 1253.5)	--	--	8500 (5678 to 12168)	886.9 (596.4 to 1263.8)	--	--	45.2 (31 to 62.1)	1.2 (-4.6 to 8.3)	--	--
		Male	3394 (2216 to 4990)	491.6 (330.7 to 708.6)	--	--	5286 (3461 to 7880)	533.5 (354.2 to 788)	--	--	55.8 (36.5 to 76.6)	8.5 (0.4 to 16.7)	--	--
	Bipolar disorder	Both	2446 (1477 to 3758)	172.3 (106.2 to 261.5)	--	--	3343 (2060 to 5115)	174.3 (107.8 to 268.1)	--	--	36.7 (23.5 to 49.9)	1.2 (-5.7 to 8.2)	--	--
		Female	1301 (778 to 2003)	186.4 (114 to 284.5)	--	--	1772 (1093 to 2721)	188.5 (116 to 286.2)	--	--	36.2 (20.6 to 54.5)	1.1 (-8 to 11.9)	--	--

Province	Cause	Sex	1990				2019				1990-2019 percent change (%)			
			DALYs		Death		DALYs		Death		DALYs		Death	
			Number	Rate	Number	Rate	Number	Rate	Number	Rate	Number	Rate	Number	Rate
		Male	1146 (694 to 1743)	158.9 (99.1 to 237.6)	--	--	1571 (968 to 2386)	160.8 (98.9 to 245.8)	--	--	37.2 (20.5 to 55.4)	1.1 (-8.8 to 12)	--	--
	Anxiety disorders	Both	10660 (7367 to 14837)	673 (470.8 to 923.5)	--	--	12647 (8793 to 17537)	694.7 (486.8 to 955.5)	--	--	18.6 (7.8 to 29.9)	3.2 (-2.3 to 9.6)	--	--
		Female	6255 (4306 to 8665)	827.3 (572 to 1132.4)	--	--	7697 (5300 to 10685)	853.3 (595.2 to 1181.6)	--	--	23.1 (10.3 to 36.1)	3.1 (-4.8 to 11.8)	--	--
		Male	4406 (3055 to 6178)	526.4 (368.9 to 729.1)	--	--	4949 (3429 to 6803)	541.3 (374.8 to 745.9)	--	--	12.3 (-0.6 to 27.4)	2.8 (-5.7 to 11.4)	--	--
	Eating disorders	Both	666 (401 to 1001)	38.7 (23.6 to 58)	0 (0 to 0)	0 (0 to 0)	950 (595 to 1427)	50.8 (31.6 to 76.2)	0 (0 to 0)	0 (0 to 0)	42.8 (22.8 to 68.7)	31.3 (17 to 46.8)	170.1 (30.3 to 554.7)	73.1 (-15.9 to 346.3)
		Female	404 (241 to 607)	48.3 (29.2 to 72.8)	0 (0 to 0)	0 (0 to 0)	579 (356 to 873)	63.8 (39 to 96.4)	0 (0 to 0)	0 (0 to 0)	43.3 (20.1 to 72.7)	32 (14.3 to 52.5)	118.9 (-4.1 to 494.8)	36.8 (-42.1 to 321.4)
		Male	262 (158 to 405)	29.6 (17.6 to 45.6)	0 (0 to 0)	0 (0 to 0)	372 (225 to 565)	38.4 (23.2 to 57.8)	0 (0 to 0)	0 (0 to 0)	42 (15.8 to 77.7)	29.8 (9.5 to 53.9)	483.2 (61.5 to 1867.9)	358 (29.2 to 1508.7)
	Autism spectrum disorders	Both	813 (531 to 1176)	44.7 (29.5 to 64.7)	--	--	770 (499 to 1115)	44.9 (29.2 to 64.9)	--	--	-5.3 (-15.1 to 5.1)	0.6 (-9.3 to 11.4)	--	--
		Female	196 (123 to 282)	22.3 (14 to 32)	--	--	190 (121 to 282)	22.6 (14.3 to 33.1)	--	--	-3.1 (-17.2 to 14.7)	1.2 (-13.5 to 18.9)	--	--
		Male	617 (400 to 896)	65.8 (42.5 to 94.5)	--	--	580 (377 to 837)	66.4 (43.2 to 95.6)	--	--	-6 (-17.2 to 7)	1 (-11 to 14.7)	--	--
	Attention-deficit/hyperactivity disorder	Both	671 (382 to 1167)	32.7 (18.5 to 55.8)	--	--	547 (312 to 930)	33.7 (18.9 to 57.2)	--	--	-18.5 (-30.6 to -3.4)	3 (-10.7 to 16.9)	--	--
		Female	197 (109 to 345)	20.1 (11.2 to 34.9)	--	--	166 (92 to 281)	20.9 (11.5 to 35.4)	--	--	-15.7 (-30 to 4.6)	4 (-13.4 to 24.9)	--	--
		Male	474 (267 to 804)	44.5 (25 to 75.4)	--	--	381 (216 to 646)	45.8 (26.2 to 78.1)	--	--	-19.7 (-34.4 to -1.1)	3 (-14.8 to 21.4)	--	--
	Conduct disorder	Both	1862 (1072 to 2893)	76.1 (43.7 to 117.9)	--	--	979 (567 to 1563)	74.5 (43.1 to 118.3)	--	--	-47.4 (-52.5 to -42.2)	-2.1 (-11.5 to 7.7)	--	--
		Female	615 (333 to 1011)	52.4 (28.4 to 86)	--	--	327 (170 to 537)	51.1 (26.5 to 84.1)	--	--	-46.8 (-54.5 to -38)	-2.5 (-16.6 to 14)	--	--
		Male	1247 (723 to 1933)	97.7 (56.6 to 151.6)	--	--	653 (383 to 1038)	96.6 (56.6 to 154.8)	--	--	-47.7 (-53.8 to -40.6)	-1.1 (-12.5 to 12.2)	--	--
	Idiopathic developmental intellectual disability	Both	2062 (1157 to 3291)	112.2 (63.3 to 177.6)	--	--	1063 (550 to 1774)	61.8 (31.8 to 103.5)	--	--	-48.4 (-56.8 to -42.7)	-44.9 (-53.9 to -38.6)	--	--
		Female	883 (485 to 1391)	99.9 (55.2 to 158.4)	--	--	479 (245 to 802)	56.7 (29 to 95.2)	--	--	-45.7 (-53.9 to -39.3)	-43.2 (-51.8 to -36.4)	--	--
		Male	1179 (672 to 1857)	123.7 (70.8 to 194.6)	--	--	584 (300 to 976)	66.7 (33.9 to 112.1)	--	--	-50.5 (-59.8 to -44.4)	-46.1 (-56.4 to -39.4)	--	--
	Other mental disorders	Both	1150 (738 to 1745)	100.7 (65.6 to 152.7)	--	--	2022 (1304 to 3101)	100.9 (65.5 to 153.6)	--	--	75.9 (62.6 to 89.8)	0.2 (-4.9 to 5.8)	--	--
		Female	454 (288 to 692)	82.6 (52.7 to 127)	--	--	821 (525 to 1262)	82.9 (53.3 to 126.5)	--	--	80.8 (62.5 to 98.9)	0.4 (-7.8 to 8.8)	--	--
		Male	695 (448 to 1041)	118.1 (75.8 to 175.1)	--	--	1201 (760 to 1834)	118.5 (75.6 to 180.2)	--	--	72.7 (57.3 to 89.1)	0.3 (-7 to 7.2)	--	--

Province	Cause	Sex	1990				2019				1990-2019 percent change (%)			
			DALYs		Death		DALYs		Death		DALYs		Death	
			Number	Rate	Number	Rate	Number	Rate	Number	Rate	Number	Rate	Number	Rate
	Substance use disorders	Both	4974 (3950 to 6147)	350.4 (280.9 to 427.9)	43 (34 to 54)	3.7 (2.9 to 4.6)	12079 (9966 to 14525)	604.3 (495.7 to 726.5)	131 (107 to 157)	6.5 (5.4 to 7.7)	142.8 (104.1 to 191)	72.5 (46.5 to 103.7)	201.5 (123 to 304.5)	75.5 (31.9 to 130.4)
		Female	1619 (1210 to 2090)	232.6 (176 to 296.6)	14 (10 to 20)	2.6 (1.8 to 3.5)	2486 (1912 to 3098)	258.3 (199.5 to 322.6)	20 (15 to 25)	2.1 (1.7 to 2.7)	53.5 (23.4 to 91.7)	11.1 (-10.2 to 36.7)	40.1 (-7.2 to 116.2)	-17.5 (-44.2 to 24)
		Male	3355 (2609 to 4199)	464.3 (361.9 to 574.7)	29 (21 to 39)	4.8 (3.5 to 6.4)	9593 (7778 to 11677)	936.8 (762.4 to 1144.6)	111 (87 to 136)	10.7 (8.5 to 13.2)	185.9 (133.2 to 251.5)	101.8 (64.7 to 145.4)	280.4 (162.4 to 448.1)	123.3 (56.2 to 221.3)
	Alcohol use disorders	Both	826 (611 to 1080)	61.7 (46.1 to 80.3)	6 (4 to 8)	0.5 (0.4 to 0.7)	1001 (769 to 1291)	49.3 (37.9 to 63.8)	8 (6 to 10)	0.4 (0.3 to 0.5)	21.1 (3.3 to 44.7)	-20.1 (-31.4 to -5.1)	43.9 (1.7 to 116.5)	-18.8 (-42.7 to 21.3)
		Female	207 (149 to 279)	30.4 (22.3 to 41)	1 (1 to 2)	0.2 (0.2 to 0.3)	257 (184 to 353)	26.5 (19.1 to 36)	1 (1 to 2)	0.1 (0.1 to 0.2)	24.3 (1.3 to 49.5)	-12.9 (-27.7 to 3.7)	3.1 (-29.6 to 51.7)	-38.1 (-56.4 to -10.9)
		Male	619 (452 to 813)	92.2 (68.2 to 121)	5 (3 to 6)	0.8 (0.5 to 1.1)	744 (569 to 951)	71.4 (54.7 to 91.2)	7 (5 to 9)	0.7 (0.5 to 0.9)	20.1 (-1.7 to 49.2)	-22.6 (-36.7 to -3.7)	54.9 (3.1 to 155)	-13 (-42.9 to 43.3)
	Drug use disorders	Both	4148 (3234 to 5186)	288.7 (229.9 to 356.9)	38 (29 to 48)	3.2 (2.5 to 4)	11078 (9032 to 13439)	555 (455.3 to 673)	122 (100 to 147)	6.1 (5 to 7.3)	167.1 (122.1 to 225.7)	92.3 (60 to 130.8)	225.5 (138.6 to 337)	90.6 (40 to 151)
		Female	1412 (1045 to 1849)	202.2 (151.2 to 261.3)	13 (9 to 18)	2.3 (1.7 to 3.2)	2229 (1716 to 2815)	231.8 (178.4 to 293.4)	19 (14 to 24)	2 (1.5 to 2.5)	57.8 (23.9 to 102.6)	14.7 (-9.4 to 44.1)	43.5 (-5.6 to 122.7)	-15.5 (-43.5 to 28.7)
		Male	2736 (2080 to 3492)	372.1 (285.2 to 465.2)	25 (18 to 33)	4 (2.9 to 5.4)	8849 (7148 to 10860)	865.4 (700.8 to 1063.4)	104 (82 to 128)	10.1 (8 to 12.4)	223.5 (158.9 to 304)	132.6 (85.8 to 188.1)	321.9 (182.6 to 520.9)	149.8 (70.7 to 267.5)
	Diabetes and kidney diseases	Both	9686 (8222 to 11278)	929.8 (796.9 to 1097.9)	193 (162 to 234)	27.2 (22.8 to 33.9)	21120 (17507 to 24817)	1184 (984 to 1387.5)	516 (444 to 589)	30.6 (26.3 to 34.9)	118.1 (85 to 155.9)	27.3 (10.2 to 44.8)	167.5 (113.3 to 229.9)	12.8 (-11 to 37.6)
		Female	4592 (3709 to 5634)	911.9 (757 to 1103.3)	86 (69 to 110)	26.1 (20.7 to 36.7)	10581 (8747 to 12676)	1195.2 (996.1 to 1421.2)	255 (216 to 303)	31.4 (26.6 to 37.6)	130.4 (88.4 to 177.6)	31.1 (9.2 to 54.1)	195.4 (124.6 to 283.1)	20.3 (-13.1 to 56.4)
		Male	5094 (4130 to 6155)	943 (777.3 to 1145.2)	107 (84 to 136)	27.9 (22.1 to 35.4)	10540 (8618 to 12428)	1174.8 (962.4 to 1391.4)	262 (212 to 316)	30.3 (24.5 to 36.6)	106.9 (64.6 to 156.7)	24.6 (1.1 to 50.5)	144.9 (79.8 to 235.6)	8.5 (-20 to 46.6)
	Diabetes mellitus	Both	2910 (2249 to 3757)	321.7 (249.9 to 420.4)	35 (28 to 58)	5 (4 to 9.3)	11581 (8939 to 14639)	641.2 (497.8 to 807.2)	168 (142 to 201)	10 (8.4 to 12)	297.9 (244.8 to 338.1)	99.3 (70.1 to 118.7)	374.9 (245 to 519.7)	98.1 (31.2 to 161.2)
		Female	1380 (1054 to 1914)	321.4 (243.4 to 450.2)	16 (12 to 38)	5.1 (3.6 to 13)	6111 (4732 to 7768)	675.8 (523.8 to 855.7)	90 (74 to 117)	10.9 (8.9 to 14.2)	342.9 (249.4 to 406.5)	110.3 (59.9 to 140.8)	451 (227.4 to 744.5)	114.3 (18.1 to 235.4)
		Male	1531 (1175 to 1944)	321.4 (246.2 to 407.6)	19 (15 to 25)	5 (3.9 to 6.5)	5470 (4250 to 6911)	606 (468.9 to 769.2)	78 (62 to 97)	9.2 (7.3 to 11.4)	257.4 (207.9 to 306.7)	88.6 (64.3 to 114.8)	309.8 (186.9 to 481.4)	84.9 (30.6 to 158.4)
	Chronic kidney disease	Both	6695 (5580 to 7857)	603.4 (525.4 to 696.1)	156 (131 to 186)	22 (18.5 to 27.3)	8518 (7435 to 9728)	485.2 (424.1 to 554.1)	312 (266 to 358)	18.6 (15.8 to 21.4)	27.2 (5.2 to 54.4)	-19.6 (-33 to -4.7)	99.7 (60.1 to 146.7)	-15.6 (-33.9 to 4)
		Female	3166 (2533 to 3913)	585.1 (483.1 to 711.9)	69 (55 to 87)	20.9 (16.3 to 28.2)	4017 (3454 to 4704)	465 (400.7 to 544.4)	147 (122 to 176)	18.4 (15.2 to 22)	26.9 (-1.2 to 60.9)	-20.5 (-36.2 to -2.6)	113 (60.8 to 177.9)	-12.2 (-37.8 to 16.8)
		Male	3529 (2766 to 4362)	617.7 (504.1 to 761.4)	87 (68 to 111)	22.8 (18 to 29.2)	4500 (3747 to 5335)	508.1 (423.6 to 600.1)	165 (133 to 199)	19.1 (15.4 to 23.1)	27.5 (-1.7 to 70.1)	-17.7 (-35.8 to 6.4)	89.1 (35.8 to 159.2)	-16.5 (-39.5 to 14.1)
	Acute glomerulonephritis	Both	80 (47 to 191)	4.6 (3.1 to 9)	1 (1 to 3)	0.1 (0.1 to 0.2)	1022 (42 to 1380)	57.6 (2.7 to 77.3)	36 (1 to 48)	2.1 (0.1 to 2.8)	1172 (-79 to 2376.8)	1138 (-69.6 to 2026.9)	2488.9 (-52.9 to 4281.8)	1580.8 (-58.2 to 2578.8)

Province	Cause	Sex	1990				2019				1990-2019 percent change (%)			
			DALYs		Death		DALYs		Death		DALYs		Death	
			Number	Rate	Number	Rate	Number	Rate	Number	Rate	Number	Rate	Number	Rate
		Female	46 (23 to 84)	5.4 (3 to 8.7)	1 (0 to 1)	0.1 (0.1 to 0.2)	453 (15 to 628)	54.3 (2 to 76.1)	17 (1 to 24)	2.2 (0.1 to 3)	882.9 (-67.5 to 2193)	907.4 (-56.7 to 2029.5)	2149.6 (-28 to 4574)	1433.3 (-40 to 2990.9)
		Male	34 (17 to 129)	3.9 (2.2 to 11.9)	1 (0 to 2)	0.1 (0.1 to 0.3)	569 (15 to 880)	60.7 (1.9 to 92.9)	19 (0 to 28)	2 (0.1 to 3.1)	1560.4 (-87.4 to 4645.8)	1444.8 (-81.5 to 3763.5)	2913.5 (-73.4 to 7582.4)	1796.6 (-75.7 to 4082.7)
	Skin and subcutaneous diseases	Both	7230 (4823 to 10537)	412.3 (276.1 to 604.6)	1 (1 to 2)	0.2 (0.2 to 0.4)	7096 (4708 to 10548)	421.5 (278.8 to 627.7)	5 (4 to 6)	0.3 (0.2 to 0.3)	-1.9 (-6 to 2.2)	2.2 (-0.9 to 5)	238.9 (99.9 to 400.7)	20.2 (-34.8 to 92.6)
		Female	3713 (2459 to 5377)	436.8 (292.1 to 637.6)	1 (0 to 1)	0.2 (0.1 to 0.5)	3682 (2451 to 5477)	444.4 (296.7 to 661.4)	2 (1 to 2)	0.2 (0.2 to 0.3)	-0.8 (-5.8 to 4)	1.7 (-2 to 4.9)	203.7 (49.4 to 408.6)	5.1 (-55.5 to 95.1)
		Male	3517 (2330 to 5159)	389.1 (260.9 to 573.1)	1 (1 to 1)	0.2 (0.1 to 0.5)	3414 (2256 to 5061)	399.4 (263.7 to 592.5)	3 (2 to 4)	0.3 (0.2 to 0.4)	-2.9 (-7.7 to 1.6)	2.7 (-1.5 to 6.3)	265.3 (117.9 to 485.4)	32 (-31.6 to 138.9)
	Dermatitis	Both	1652 (966 to 2628)	93.4 (55.7 to 144.3)	--	--	1630 (997 to 2472)	94.1 (57.2 to 145.2)	--	--	-1.3 (-9.6 to 8.7)	0.8 (-4.2 to 5.8)	--	--
		Female	890 (508 to 1394)	103.7 (61.4 to 159.9)	--	--	890 (538 to 1366)	104.6 (63.1 to 162.7)	--	--	0.1 (-9.6 to 11)	0.8 (-5.6 to 7.7)	--	--
		Male	762 (447 to 1207)	83.6 (50.2 to 130.2)	--	--	740 (454 to 1120)	84.1 (51.1 to 128.9)	--	--	-2.9 (-12.4 to 9)	0.6 (-6.7 to 8)	--	--
	Psoriasis	Both	664 (471 to 891)	48.1 (34 to 64.4)	--	--	652 (457 to 853)	34.5 (24.3 to 45.3)	--	--	-1.7 (-12.2 to 10.6)	-28.2 (-34.6 to -20.9)	--	--
		Female	339 (236 to 459)	49.9 (35.1 to 67.2)	--	--	333 (234 to 444)	35.9 (25.2 to 47.9)	--	--	-1.8 (-15.1 to 14.8)	-28.1 (-37.4 to -17.4)	--	--
		Male	325 (228 to 439)	46.4 (33 to 62.2)	--	--	319 (223 to 427)	33.2 (23.2 to 43.8)	--	--	-1.7 (-15.5 to 15.1)	-28.4 (-37.8 to -18.1)	--	--
	Bacterial skin diseases	Both	112 (75 to 179)	7.8 (5.5 to 11.5)	1 (1 to 2)	0.2 (0.1 to 0.3)	132 (95 to 195)	7.6 (5.5 to 11.3)	3 (2 to 4)	0.2 (0.1 to 0.2)	17.5 (0.4 to 39.8)	-3 (-19.9 to 16.3)	129.6 (70.6 to 240.3)	-12.3 (-37.6 to 49.4)
		Female	48 (31 to 79)	6.9 (4.7 to 10.8)	0 (0 to 1)	0.2 (0.1 to 0.3)	52 (36 to 83)	6.2 (4.2 to 9.9)	1 (1 to 1)	0.1 (0.1 to 0.2)	9.9 (-5.3 to 37.8)	-10.3 (-30.5 to 18.9)	97.5 (19.8 to 307.7)	-28.3 (-59.9 to 80.8)
		Male	64 (42 to 103)	8.6 (6 to 13.2)	1 (0 to 1)	0.2 (0.1 to 0.4)	79 (57 to 113)	8.9 (6.5 to 12.8)	2 (1 to 3)	0.2 (0.1 to 0.3)	23.1 (-4 to 55.4)	3 (-22.5 to 31)	152.3 (67.3 to 288.2)	-0.7 (-37 to 74)
	Scabies	Both	461 (251 to 747)	25.2 (13.8 to 40.8)	--	--	425 (234 to 685)	25.1 (13.7 to 40.3)	--	--	-7.9 (-14.9 to -0.1)	-0.5 (-4.8 to 4.1)	--	--
		Female	224 (121 to 360)	25.3 (13.9 to 40.8)	--	--	209 (114 to 336)	25.2 (13.8 to 40.5)	--	--	-6.6 (-15.5 to 2.4)	-0.4 (-6.7 to 6.2)	--	--
		Male	238 (129 to 384)	25.2 (13.8 to 40.4)	--	--	216 (118 to 348)	25 (13.6 to 40.2)	--	--	-9 (-16.6 to 0.3)	-0.7 (-7 to 5.6)	--	--
	Fungal skin diseases	Both	232 (93 to 492)	18.9 (7.6 to 40.1)	--	--	332 (134 to 712)	18.6 (7.5 to 39.6)	--	--	43 (33.1 to 52.8)	-1.6 (-3.4 to 0.1)	--	--
		Female	104 (41 to 219)	17.6 (7.1 to 38.1)	--	--	153 (62 to 332)	17.3 (6.9 to 37.9)	--	--	48.1 (37.7 to 58.4)	-1.5 (-4.2 to 0.9)	--	--
		Male	129 (51 to 276)	20.1 (8.1 to 42.7)	--	--	179 (72 to 386)	19.8 (8 to 41.9)	--	--	39 (28.5 to 49.2)	-1.5 (-3.9 to 0.9)	--	--
	Viral skin diseases	Both	1117 (703 to 1682)	51.2 (32.4 to 76.6)	--	--	801 (506 to 1197)	51.4 (32.5 to 76.8)	--	--	-28.3 (-31.6 to -24.7)	0.4 (-3.9 to 4.8)	--	--
		Female	535 (338 to 813)	50.8 (32.3 to 76.6)	--	--	389 (247 to 580)	51 (32.3 to 76.5)	--	--	-27.4 (-31.9 to -22.4)	0.5 (-5.3 to 6.9)	--	--

Province	Cause	Sex	1990				2019				1990-2019 percent change (%)			
			DALYs		Death		DALYs		Death		DALYs		Death	
			Number	Rate	Number	Rate	Number	Rate	Number	Rate	Number	Rate	Number	Rate
		Male	582 (366 to 872)	51.6 (32.7 to 77.3)	--	--	412 (261 to 614)	51.8 (32.7 to 77.4)	--	--	-29.1 (-33.4 to -24.2)	0.3 (-5.8 to 6.7)	--	--
	Acne vulgaris	Both	1098 (652 to 1752)	51.3 (30.7 to 81.5)	--	--	1017 (615 to 1629)	69.8 (42 to 111.3)	--	--	-7.4 (-14.3 to 0.9)	36.3 (26.2 to 47.5)	--	--
		Female	506 (302 to 817)	49.7 (29.7 to 80.3)	--	--	486 (292 to 766)	67.8 (41 to 107)	--	--	-4 (-13.9 to 7.9)	36.4 (22.8 to 52.5)	--	--
		Male	592 (353 to 947)	52.6 (31.5 to 84)	--	--	531 (318 to 854)	71.8 (43.1 to 115.3)	--	--	-10.3 (-19.8 to 0.3)	36.5 (23.2 to 52.2)	--	--
	Alopecia areata	Both	94 (59 to 139)	6.3 (4 to 9.5)	--	--	122 (77 to 185)	6.4 (4 to 9.6)	--	--	30.6 (16.1 to 45.3)	0.2 (-9.6 to 10.3)	--	--
		Female	60 (38 to 89)	8.3 (5.2 to 12.4)	--	--	79 (50 to 119)	8.3 (5.2 to 12.5)	--	--	32.2 (14.9 to 51.7)	0 (-12.3 to 13.4)	--	--
		Male	34 (21 to 53)	4.5 (2.8 to 6.9)	--	--	44 (27 to 66)	4.5 (2.7 to 6.8)	--	--	27.8 (5.6 to 55.8)	-0.1 (-16.9 to 18.8)	--	--
	Pruritus	Both	139 (67 to 249)	9.9 (4.7 to 17.6)	--	--	192 (92 to 349)	10.5 (5.1 to 19.1)	--	--	38.5 (27 to 52.9)	6.1 (1 to 11.6)	--	--
		Female	74 (35 to 131)	10.9 (5.2 to 19.3)	--	--	104 (49 to 192)	11.5 (5.5 to 20.7)	--	--	41.1 (27.4 to 56.6)	5.8 (-1 to 13.3)	--	--
		Male	65 (31 to 118)	9 (4.3 to 16.2)	--	--	88 (42 to 161)	9.6 (4.6 to 17.3)	--	--	35.5 (21.6 to 51.7)	6.1 (-1 to 14.6)	--	--
	Urticaria	Both	1113 (715 to 1643)	58 (37.5 to 83.9)	--	--	974 (636 to 1392)	58.3 (38.3 to 84.6)	--	--	-12.4 (-20.3 to -3.8)	0.5 (-4.8 to 6.4)	--	--
		Female	667 (434 to 978)	70.9 (46.4 to 102.8)	--	--	578 (380 to 833)	71.1 (46.8 to 102.8)	--	--	-13.3 (-21.3 to -4)	0.3 (-6.2 to 7.2)	--	--
		Male	445 (282 to 667)	45.8 (29.5 to 66.7)	--	--	396 (260 to 572)	46 (30 to 67)	--	--	-11.1 (-20.9 to 0.6)	0.4 (-8.4 to 9.7)	--	--
	Decubitus ulcer	Both	6 (4 to 12)	0.9 (0.6 to 2)	0 (0 to 0)	0 (0 to 0.1)	32 (20 to 39)	1.9 (1.2 to 2.3)	2 (1 to 2)	0.1 (0.1 to 0.1)	438.4 (103.6 to 688.9)	113.8 (-28.5 to 224.6)	1252 (148.5 to 2949.3)	197.9 (-45.7 to 570.2)
		Female	3 (2 to 6)	0.9 (0.5 to 2.2)	0 (0 to 0)	0 (0 to 0.2)	14 (8 to 18)	1.7 (1 to 2.2)	1 (0 to 1)	0.1 (0 to 0.1)	432.1 (78.9 to 785.6)	101.2 (-42.5 to 253)	1016.8 (71.1 to 3143.4)	169.3 (-58.1 to 649.6)
		Male	3 (2 to 7)	0.9 (0.6 to 2.2)	0 (0 to 0)	0 (0 to 0.2)	18 (11 to 24)	2.1 (1.2 to 2.7)	1 (0 to 1)	0.1 (0.1 to 0.2)	443.3 (99.3 to 724)	125.8 (-28.1 to 256.4)	1511.4 (183.2 to 4054.8)	229.6 (-40 to 777.3)
	Other skin and subcutaneous diseases	Both	542 (258 to 1001)	41.4 (19.9 to 75.4)	0 (0 to 0)	0 (0 to 0)	785 (381 to 1439)	43.3 (20.9 to 79.4)	0 (0 to 0)	0 (0 to 0)	44.8 (40.6 to 49.3)	4.8 (2.6 to 7.2)	287.6 (103.7 to 716.4)	-9.1 (-53.4 to 113.4)
		Female	265 (126 to 486)	41.9 (20.1 to 75.8)	0 (0 to 0)	0 (0 to 0)	394 (190 to 727)	43.8 (21 to 80.7)	0 (0 to 0)	0 (0 to 0)	48.7 (43.3 to 54.7)	4.6 (1.4 to 8.1)	134 (-8.8 to 697.1)	-44.2 (-78.9 to 109.6)
		Male	277 (131 to 512)	40.8 (19.6 to 74.7)	0 (0 to 0)	0 (0 to 0)	391 (188 to 712)	42.9 (20.6 to 78.2)	0 (0 to 0)	0 (0 to 0)	41 (36 to 46.9)	4.9 (1.7 to 8.5)	461.5 (198.3 to 1073.7)	37 (-27 to 213.6)
	Sense organ diseases	Both	9787 (6958 to 13150)	1039.7 (742.9 to 1389.6)	--	--	14280 (10110 to 19498)	814.9 (578.6 to 1107.6)	--	--	45.9 (38.1 to 53.6)	-21.6 (-24.6 to -18.3)	--	--
		Female	4672 (3283 to 6278)	1043.7 (753.5 to 1386.8)	--	--	7102 (5072 to 9624)	815.5 (584.7 to 1099.9)	--	--	52 (43.4 to 60.6)	-21.9 (-25.2 to -18.3)	--	--

Province	Cause	Sex	1990				2019				1990-2019 percent change (%)			
			DALYs		Death		DALYs		Death		DALYs		Death	
			Number	Rate	Number	Rate	Number	Rate	Number	Rate	Number	Rate	Number	Rate
		Male	5116 (3656 to 6915)	1035.6 (745.7 to 1387.2)	--	--	7178 (5042 to 9830)	814.5 (573.4 to 1113.2)	--	--	40.3 (32.6 to 48.2)	-21.4 (-24.7 to -17.9)	--	--
	Blindness and vision loss	Both	4571 (3277 to 6186)	505.5 (363 to 677.9)	--	--	6452 (4598 to 8711)	375.1 (267.7 to 505.4)	--	--	41.2 (35.6 to 47.3)	-25.8 (-28.6 to -22.6)	--	--
		Female	2292 (1643 to 3117)	533.6 (385.2 to 713)	--	--	3399 (2423 to 4608)	395.9 (282.8 to 535.2)	--	--	48.3 (41.6 to 55.8)	-25.8 (-28.8 to -22.2)	--	--
		Male	2278 (1622 to 3102)	479.7 (344.9 to 640.5)	--	--	3053 (2181 to 4112)	353.6 (252.3 to 476.4)	--	--	34 (27.3 to 41.4)	-26.3 (-29.6 to -22.6)	--	--
	Age-related and other hearing loss	Both	4737 (3263 to 6546)	492.9 (351.5 to 684.5)	--	--	7046 (4881 to 9946)	396.6 (275.7 to 556.3)	--	--	48.7 (34.3 to 63.5)	-19.5 (-23.8 to -15)	--	--
		Female	2130 (1472 to 2945)	465.1 (329.3 to 638.7)	--	--	3282 (2265 to 4622)	372.9 (259.1 to 519.5)	--	--	54.1 (37 to 70.8)	-19.8 (-25 to -14.8)	--	--
		Male	2607 (1826 to 3633)	518 (368 to 723.4)	--	--	3764 (2616 to 5302)	421.1 (292.7 to 591.2)	--	--	44.4 (29.2 to 58.7)	-18.7 (-23.4 to -13.7)	--	--
	Other sense organ diseases	Both	479 (282 to 743)	41.3 (24.2 to 64.1)	--	--	782 (456 to 1201)	43.3 (25.6 to 67.1)	--	--	63.1 (49.5 to 78.3)	4.8 (-0.4 to 10.3)	--	--
		Female	250 (147 to 387)	44.9 (26.4 to 69.5)	--	--	421 (247 to 651)	46.8 (27.5 to 71.8)	--	--	68.8 (52.1 to 87.5)	4.2 (-3.3 to 12)	--	--
		Male	230 (136 to 358)	37.9 (22 to 58.9)	--	--	361 (213 to 557)	39.8 (23.5 to 61.7)	--	--	57 (40.7 to 75.3)	4.9 (-2.4 to 13)	--	--
	Musculoskeletal disorders	Both	20346 (14354 to 27190)	1824.3 (1290.1 to 2432.5)	4 (3 to 6)	0.4 (0.3 to 0.7)	37464 (26608 to 49702)	1932.8 (1374.3 to 2550.8)	14 (10 to 17)	0.8 (0.6 to 0.9)	84.1 (74.9 to 94.3)	5.9 (2.3 to 10.4)	267.2 (108.1 to 430.5)	81.5 (6.2 to 162.8)
		Female	10693 (7571 to 14099)	1984.4 (1413.4 to 2636.6)	2 (2 to 4)	0.6 (0.4 to 1.1)	20615 (14765 to 27383)	2143.3 (1536.2 to 2843.2)	9 (5 to 11)	1.1 (0.6 to 1.3)	92.8 (83 to 104.4)	8 (3.8 to 12.8)	279 (81.3 to 534)	89.1 (-15.1 to 214.3)
		Male	9653 (6799 to 13006)	1672.8 (1178.2 to 2230.5)	1 (1 to 2)	0.3 (0.2 to 0.5)	16849 (11855 to 22468)	1724.6 (1221 to 2300.3)	5 (4 to 6)	0.6 (0.4 to 0.7)	74.5 (63.7 to 87)	3.1 (-1.7 to 8.7)	247.7 (62.3 to 404.6)	72.6 (-9.3 to 146.7)
	Rheumatoid arthritis	Both	126 (89 to 172)	11.5 (8.1 to 15.5)	0 (0 to 1)	0.1 (0 to 0.1)	256 (180 to 344)	13.1 (9.4 to 17.5)	1 (1 to 2)	0.1 (0 to 0.1)	102.7 (66.3 to 137.9)	14.3 (-7.9 to 35)	244.7 (4.9 to 616.5)	36.6 (-59.1 to 193.1)
		Female	87 (60 to 122)	16.3 (11.3 to 22.6)	0 (0 to 1)	0.1 (0 to 0.3)	181 (127 to 247)	18.6 (13 to 25.3)	1 (0 to 2)	0.1 (0 to 0.2)	106.9 (55.7 to 158.4)	14.2 (-17 to 43.4)	237.6 (-27.2 to 816.5)	28.2 (-72.5 to 266.3)
		Male	39 (26 to 54)	6.9 (4.7 to 9.5)	0 (0 to 0)	0 (0 to 0)	75 (51 to 102)	7.7 (5.3 to 10.5)	0 (0 to 0)	0 (0 to 0.1)	93.2 (63 to 128.2)	11.4 (-6.5 to 31.2)	268.7 (126.1 to 572.6)	62 (2.2 to 195.6)
	Osteoarthritis	Both	1487 (749 to 2907)	176.1 (89.1 to 346.4)	--	--	3453 (1741 to 6756)	191.3 (96.3 to 374.1)	--	--	132.3 (124.9 to 140.4)	8.7 (5.3 to 12.2)	--	--
		Female	801 (404 to 1566)	200.2 (100.8 to 394.8)	--	--	1973 (995 to 3840)	217.3 (109.6 to 424)	--	--	146.2 (135.9 to 158.5)	8.5 (4.4 to 13.7)	--	--
		Male	685 (342 to 1378)	153.8 (77.1 to 309.9)	--	--	1481 (747 to 2915)	164.8 (83 to 327.5)	--	--	116.1 (105 to 127.1)	7.1 (1.9 to 12.2)	--	--
	Low back pain	Both	12019 (8429 to 16181)	1024.2 (723.7 to 1372.5)	--	--	17740 (12222 to 24027)	922.9 (643.3 to 1251.2)	--	--	47.6 (37.6 to 58.1)	-9.9 (-13.9 to -5.9)	--	--
		Female	5538 (3899 to 7423)	973.5 (690.3 to 1297.5)	--	--	8660 (5948 to 11766)	914.8 (637.9 to 1232.7)	--	--	56.4 (44.5 to 70.2)	-6 (-11.7 to -0.2)	--	--

Province	Cause	Sex	1990				2019				1990-2019 percent change (%)			
			DALYs		Death		DALYs		Death		DALYs		Death	
			Number	Rate	Number	Rate	Number	Rate	Number	Rate	Number	Rate	Number	Rate
		Male	6480 (4516 to 8730)	1072.2 (755.1 to 1445.9)	--	--	9080 (6268 to 12331)	931.4 (648.9 to 1256.1)	--	--	40.1 (29.4 to 51.9)	-13.1 (-18.3 to -7.5)	--	--
	Neck pain	Both	4393 (2837 to 6409)	403.4 (265.8 to 580.7)	--	--	8090 (5336 to 11806)	406.3 (266.1 to 585.5)	--	--	84.1 (72.4 to 94.5)	0.7 (-1.4 to 2.9)	--	--
		Female	2608 (1706 to 3791)	492.8 (326.1 to 717.8)	--	--	4901 (3229 to 7186)	494.4 (328.6 to 717.4)	--	--	88 (75.7 to 98.7)	0.3 (-2.3 to 2.9)	--	--
		Male	1786 (1155 to 2609)	318 (209.1 to 462.1)	--	--	3189 (2070 to 4677)	319.2 (208.1 to 462.4)	--	--	78.6 (65 to 90.5)	0.4 (-2.7 to 3.6)	--	--
	Gout	Both	126 (77 to 182)	14.1 (8.8 to 20.3)	--	--	285 (180 to 412)	15.3 (9.6 to 22.2)	--	--	126.8 (96.4 to 165.1)	8.6 (-4.7 to 25.1)	--	--
		Female	26 (16 to 38)	6.1 (3.7 to 8.9)	--	--	65 (39 to 97)	7 (4.2 to 10.4)	--	--	150.6 (95.6 to 226.1)	14.6 (-9.8 to 46.2)	--	--
		Male	100 (61 to 145)	21.6 (13.5 to 31.1)	--	--	220 (140 to 317)	23.6 (14.8 to 34.5)	--	--	120.7 (86.4 to 165.9)	9.4 (-6.2 to 28.3)	--	--
	Other musculoskeletal disorders	Both	2196 (1321 to 3237)	195.1 (115.4 to 290.9)	3 (3 to 5)	0.4 (0.3 to 0.6)	7640 (5100 to 10846)	383.9 (258.2 to 533.7)	13 (9 to 15)	0.7 (0.5 to 0.9)	247.9 (202.9 to 324.8)	96.7 (70.8 to 146.1)	269.9 (115.1 to 432.7)	88.8 (15.8 to 177.4)
		Female	1632 (1071 to 2344)	295.5 (192.5 to 421.9)	2 (1 to 4)	0.5 (0.3 to 0.8)	4835 (3305 to 6707)	491.2 (335.6 to 680.4)	8 (5 to 10)	0.9 (0.6 to 1.2)	196.2 (161.2 to 243.2)	66.2 (46.2 to 93.5)	285.3 (103.1 to 545.3)	102.2 (3.1 to 249.1)
		Male	563 (225 to 979)	100.2 (40.1 to 173)	1 (1 to 2)	0.3 (0.2 to 0.5)	2804 (1754 to 4095)	277.9 (176 to 406.2)	5 (4 to 6)	0.5 (0.4 to 0.6)	397.8 (266.8 to 786.2)	177.2 (103.9 to 400.8)	246.2 (56.7 to 404.5)	73.4 (-12.6 to 150.8)
	Other non-communicable diseases	Both	95683 (61019 to 133030)	4568.7 (3083.2 to 6128.6)	889 (503 to 1320)	37.7 (21.6 to 55.5)	35294 (27265 to 45095)	2091 (1645 to 2638)	164 (132 to 203)	11.7 (9.3 to 14.7)	-63.1 (-75.7 to -38.1)	-54.2 (-68.1 to -28.8)	-81.6 (-88.6 to -62.7)	-68.9 (-80.7 to -37.8)
		Female	48896 (31794 to 69549)	5056.5 (3511.7 to 6852.2)	411 (224 to 646)	35.9 (19.9 to 56.2)	23019 (17412 to 29964)	2618.4 (2005.3 to 3393.8)	74 (58 to 92)	10.9 (8.4 to 13.8)	-52.9 (-69.3 to -24)	-48.2 (-63.7 to -25)	-82 (-89.7 to -62.8)	-69.7 (-82.3 to -37.7)
		Male	46787 (26074 to 68377)	4089.8 (2395.5 to 5880.4)	478 (244 to 729)	39.4 (20.5 to 59.8)	12275 (9608 to 15596)	1580.2 (1246.3 to 1986.5)	90 (70 to 114)	12.5 (9.6 to 16.2)	-73.8 (-83.5 to -48.3)	-61.4 (-75 to -27.2)	-81.1 (-88.7 to -57.9)	-68.3 (-81 to -29.6)
	Congenital birth defects	Both	73556 (40270 to 110441)	3043.7 (1675 to 4552.8)	816 (435 to 1240)	33.7 (18.1 to 51.1)	10231 (7886 to 13216)	778.8 (592.3 to 1015.1)	103 (78 to 136)	8 (6 to 10.7)	-86.1 (-91.6 to -70.2)	-74.4 (-84.6 to -44.3)	-87.3 (-92.6 to -71.6)	-76.3 (-86.2 to -46.1)
		Female	33950 (17661 to 54578)	2880.3 (1509.7 to 4613)	378 (192 to 613)	32 (16.4 to 51.9)	4543 (3370 to 6062)	710.4 (517.3 to 958.6)	46 (33 to 63)	7.4 (5.2 to 10.2)	-86.6 (-92.4 to -68.5)	-75.3 (-86.2 to -41.6)	-87.8 (-93.3 to -69.8)	-77 (-87.5 to -43.4)
		Male	39606 (18926 to 61499)	3198.7 (1544.4 to 4950.7)	438 (201 to 689)	35.3 (16.3 to 55.5)	5688 (4367 to 7698)	843.7 (639.8 to 1160.6)	57 (43 to 80)	8.6 (6.4 to 12.2)	-85.6 (-91.4 to -61.6)	-73.6 (-84.3 to -29.3)	-87 (-92.4 to -62.9)	-75.7 (-86 to -31.2)
	Urinary diseases and male infertility	Both	352 (257 to 488)	28.3 (19.7 to 40)	2 (1 to 4)	0.2 (0.1 to 0.7)	626 (467 to 828)	34.7 (25.9 to 45.5)	10 (8 to 12)	0.6 (0.5 to 0.7)	77.6 (41.2 to 102.5)	22.6 (-10.8 to 42.7)	430.6 (125.8 to 660)	179.7 (-19.6 to 372.3)
		Female	111 (83 to 151)	15.4 (11.2 to 25)	1 (1 to 2)	0.3 (0.1 to 0.9)	180 (141 to 223)	20.6 (16.3 to 25.3)	5 (4 to 6)	0.6 (0.5 to 0.7)	62.3 (16.4 to 102.4)	33.7 (-24.7 to 71.8)	388.3 (84.5 to 690)	136.8 (-29.6 to 356)
		Male	241 (165 to 354)	40.2 (26.7 to 58.6)	1 (1 to 2)	0.2 (0.1 to 0.6)	446 (315 to 625)	49.1 (34.7 to 68.4)	6 (4 to 7)	0.6 (0.4 to 0.8)	84.6 (51 to 112.8)	22 (-6.7 to 40.5)	472.6 (103.4 to 746.5)	242.4 (-23.9 to 476.4)

Province	Cause	Sex	1990				2019				1990-2019 percent change (%)			
			DALYs		Death		DALYs		Death		DALYs		Death	
			Number	Rate	Number	Rate	Number	Rate	Number	Rate	Number	Rate	Number	Rate
	Gynecological diseases	Female	6887 (4700 to 9593)	1101.1 (753 to 1522.4)	0 (0 to 0)	0 (0 to 0)	10810 (7349 to 14979)	1048.4 (716.4 to 1459.1)	0 (0 to 0)	0 (0 to 0)	57 (47.9 to 65.9)	-4.8 (-8.1 to -1.8)	222.8 (-55.8 to 1217.3)	76.4 (-73.6 to 546.5)
	Hemoglobinopathies and hemolytic anemias	Both	3100 (2256 to 4210)	166.2 (120.5 to 228.6)	13 (10 to 17)	0.7 (0.6 to 0.8)	1287 (879 to 1904)	76 (52.2 to 110.4)	5 (5 to 6)	0.3 (0.3 to 0.4)	-58.5 (-68.3 to -47.1)	-54.3 (-63.9 to -43.5)	-58.7 (-69.3 to -41.8)	-52.3 (-62.8 to -36.8)
		Female	1764 (1169 to 2487)	207 (137 to 297.1)	5 (3 to 7)	0.6 (0.4 to 0.7)	779 (497 to 1243)	90.3 (58.6 to 138.7)	2 (2 to 2)	0.2 (0.2 to 0.3)	-55.8 (-69.1 to -38.4)	-56.4 (-68.3 to -40.3)	-60.2 (-74.7 to -34.9)	-55.7 (-69.4 to -33.6)
		Male	1336 (951 to 1819)	126.9 (91.8 to 172.4)	8 (6 to 11)	0.8 (0.6 to 1)	507 (347 to 744)	62 (41.7 to 91.4)	3 (3 to 4)	0.4 (0.3 to 0.5)	-62 (-72.6 to -46.7)	-51.1 (-64.4 to -31.9)	-57.9 (-70 to -36.6)	-49.8 (-62.7 to -28.9)
	Endocrine, metabolic, blood, and immune disorders	Both	5098 (3656 to 7322)	334.5 (238.2 to 459.2)	22 (15 to 45)	1.6 (1.1 to 2.9)	6212 (4500 to 8312)	351 (256.3 to 467.4)	42 (24 to 53)	2.5 (1.5 to 3.2)	21.9 (-19.1 to 47.7)	4.9 (-22.8 to 24.1)	88.5 (-35.6 to 217)	60 (-35.1 to 165.4)
		Female	2976 (2062 to 4238)	410.7 (287.5 to 568)	10 (6 to 22)	1.6 (1 to 3.2)	3744 (2663 to 5083)	424.1 (300.7 to 575)	19 (9 to 25)	2.4 (1.2 to 3.1)	25.8 (-14.7 to 51.3)	3.3 (-23.1 to 20.9)	97.8 (-45.1 to 276.2)	56.4 (-44.8 to 201.2)
		Male	2122 (1508 to 3301)	261.3 (190 to 374.4)	13 (8 to 27)	1.6 (1.1 to 3.1)	2468 (1766 to 3231)	280.7 (200.5 to 365.6)	23 (12 to 31)	2.7 (1.4 to 3.5)	16.3 (-28.4 to 54.2)	7.4 (-25.5 to 37.2)	81.3 (-30.1 to 235.1)	64.4 (-27.6 to 190.3)
	Oral disorders	Both	3480 (2068 to 5433)	319.6 (193.6 to 494.4)	--	--	5877 (3475 to 9332)	314.3 (185.8 to 493.3)	--	--	68.9 (61.4 to 77)	-1.7 (-4.3 to 0.9)	--	--
		Female	1657 (985 to 2608)	310.2 (186.8 to 479.8)	--	--	2841 (1668 to 4539)	304.6 (180.6 to 486.7)	--	--	71.4 (62.4 to 80.9)	-1.8 (-5.2 to 1.3)	--	--
		Male	1823 (1084 to 2867)	328.1 (198.2 to 513.4)	--	--	3036 (1790 to 4770)	324.3 (192 to 501)	--	--	66.6 (59 to 75.5)	-1.2 (-4.5 to 2.2)	--	--
	Sudden infant death syndrome	Both	3208 (463 to 11240)	133.2 (19.2 to 466.7)	36 (5 to 127)	1.5 (0.2 to 5.3)	251 (119 to 512)	20.2 (9.6 to 41.2)	3 (1 to 6)	0.2 (0.1 to 0.5)	-92.2 (-97.5 to -49.2)	-84.8 (-95.1 to -1.5)	-92.2 (-97.5 to -49.2)	-84.8 (-95.1 to -1.5)
		Female	1550 (238 to 5646)	131.8 (20.3 to 480.1)	18 (3 to 64)	1.5 (0.2 to 5.4)	121 (56 to 248)	20.1 (9.3 to 41.1)	1 (1 to 3)	0.2 (0.1 to 0.5)	-92.2 (-97.4 to -51.3)	-84.8 (-95 to -5.1)	-92.2 (-97.4 to -51.3)	-84.8 (-95 to -5.1)
		Male	1658 (188 to 6291)	134.5 (15.3 to 510.4)	19 (2 to 71)	1.5 (0.2 to 5.8)	130 (53 to 276)	20.4 (8.4 to 43.3)	1 (1 to 3)	0.2 (0.1 to 0.5)	-92.2 (-97.5 to -42.9)	-84.9 (-95.2 to 10.6)	-92.2 (-97.5 to -42.9)	-84.9 (-95.2 to 10.6)
	**Injuries**	Both	160929 (136252 to 188546)	8998 (7662.3 to 10498.1)	2225 (1852 to 2613)	141.9 (118.1 to 168.6)	68995 (60816 to 78276)	3746.7 (3315.1 to 4225.6)	1183 (1023 to 1364)	64.5 (56 to 74)	-57.1 (-64.4 to -48.4)	-58.4 (-65.3 to -50.4)	-46.8 (-57.4 to -34.6)	-54.6 (-63.7 to -43.9)
		Female	51878 (42206 to 63415)	5646.5 (4664.2 to 6825.1)	649 (521 to 798)	80.6 (64.8 to 99.3)	17103 (14688 to 19786)	1933.3 (1671.9 to 2227.5)	266 (224 to 316)	30.8 (26 to 36.4)	-67 (-74 to -57.1)	-65.8 (-72.5 to -56.7)	-59.1 (-69 to -44.6)	-61.8 (-70.7 to -49.8)
		Male	109051 (90002 to 128965)	12155.8 (10036.9 to 14501.2)	1576 (1283 to 1918)	199.7 (160.3 to 247.4)	51892 (44841 to 59620)	5500.2 (4771.9 to 6301.1)	918 (773 to 1091)	97.3 (81.9 to 115.3)	-52.4 (-61.7 to -41.7)	-54.8 (-63.5 to -44.2)	-41.8 (-54.8 to -24.5)	-51.3 (-62.9 to -36.3)
	Transport injuries	Both	88085 (71272 to 105540)	4822.1 (3935.9 to 5726.5)	1339 (1085 to 1598)	86 (68.1 to 103.7)	34204 (28949 to 39535)	1887.9 (1610 to 2179.1)	680 (567 to 798)	37.1 (31 to 43.5)	-61.2 (-69 to -50.7)	-60.8 (-68.7 to -50.2)	-49.2 (-59.7 to -34.8)	-56.8 (-66.2 to -43.8)
		Female	28669 (21952 to 36671)	2974.7 (2305.9 to 3715.9)	397 (304 to 501)	48.1 (37.9 to 59.5)	7743 (6566 to 9135)	889.8 (756.9 to 1043.2)	151 (125 to 181)	17.2 (14.3 to 20.4)	-73 (-80.3 to -63.1)	-70.1 (-77.6 to -60.1)	-61.9 (-71.7 to -48.3)	-64.3 (-72.9 to -53.1)
		Male	59416 (46755 to 73044)	6561.2 (5119 to 8075.6)	942 (728 to 1171)	121.6 (91.3 to 153.6)	26461 (21926 to 31407)	2852.1 (2367.7 to 3380.3)	529 (426 to 645)	56.6 (45.6 to 68.7)	-55.5 (-65.6 to -41.3)	-56.5 (-66.8 to -42)	-43.9 (-58.4 to -24.8)	-53.4 (-65.7 to -35.8)
	Road injuries	Both	85964 (69499 to 102948)	4699.1 (3834.5 to 5577.8)	1307 (1059 to 1561)	83.8 (66.4 to 101.3)	32336 (27264 to 37386)	1785.3 (1499.6 to 2063.4)	644 (531 to 757)	35.2 (29 to 41.3)	-62.4 (-70 to -51.9)	-62 (-69.8 to -51.5)	-50.7 (-60.8 to -36.6)	-58 (-67.1 to -45.4)

Province	Cause	Sex	1990				2019				1990-2019 percent change (%)			
			DALYs		Death		DALYs		Death		DALYs		Death	
			Number	Rate	Number	Rate	Number	Rate	Number	Rate	Number	Rate	Number	Rate
		Female	28105 (21551 to 36009)	2914 (2257.9 to 3654)	389 (298 to 490)	47.1 (37.1 to 58.4)	7344 (6212 to 8671)	843.7 (717.8 to 992.2)	144 (119 to 172)	16.3 (13.6 to 19.3)	-73.9 (-80.9 to -64.2)	-71 (-78.3 to -61.2)	-63.1 (-72.6 to -49.6)	-65.5 (-74 to -54.6)
		Male	57858 (45742 to 71061)	6379.2 (4960.5 to 7863.1)	918 (707 to 1141)	118.3 (88.7 to 149.5)	24991 (20610 to 29816)	2694.8 (2230.2 to 3207.8)	500 (401 to 610)	53.6 (43.1 to 65.1)	-56.8 (-66.8 to -43)	-57.8 (-67.7 to -43.5)	-45.5 (-59.5 to -26.6)	-54.7 (-66.8 to -37.6)
	Other transport injuries	Both	2121 (1626 to 2703)	123 (96.4 to 155.2)	32 (24 to 41)	2.2 (1.6 to 2.8)	1868 (1453 to 2355)	102.6 (79.7 to 129.2)	36 (27 to 46)	2 (1.5 to 2.5)	-11.9 (-36.7 to 25.8)	-16.6 (-38.7 to 17.3)	14 (-19.3 to 64.7)	-8.3 (-35.4 to 32.1)
		Female	564 (370 to 836)	60.7 (43 to 84.9)	7 (5 to 11)	0.9 (0.7 to 1.3)	399 (317 to 499)	46.1 (36.7 to 57.6)	8 (6 to 10)	0.9 (0.7 to 1.1)	-29.3 (-54 to 12.7)	-24.1 (-47.4 to 10.4)	3.6 (-32.7 to 60.8)	-4.3 (-33.1 to 39.9)
		Male	1558 (1117 to 2147)	181.9 (131.9 to 246.4)	24 (17 to 34)	3.3 (2.3 to 4.6)	1469 (1044 to 1945)	157.3 (111 to 209.6)	29 (20 to 38)	3 (2.1 to 4.1)	-5.7 (-38.1 to 47.5)	-13.5 (-42.2 to 34.7)	17.1 (-24.9 to 80.6)	-8.3 (-40.6 to 45.5)
	Unintentional injuries	Both	45395 (37297 to 54097)	2453.6 (2045.3 to 2884.6)	581 (475 to 691)	36.6 (30.7 to 42.7)	19020 (16016 to 22324)	1042.5 (885.3 to 1213.5)	284 (244 to 330)	15.9 (13.7 to 18.4)	-58.1 (-66.1 to -47.5)	-57.5 (-64.6 to -48.8)	-51.1 (-61.7 to -36.1)	-56.6 (-65.3 to -44.7)
		Female	15555 (11784 to 20499)	1666.1 (1298.6 to 2122.4)	187 (139 to 250)	24.1 (18.5 to 30.5)	5321 (4356 to 6449)	613 (505.2 to 735.3)	75 (61 to 92)	9.1 (7.5 to 11.2)	-65.8 (-75.5 to -52.6)	-63.2 (-72.8 to -51.5)	-60.1 (-73.4 to -39.7)	-62.1 (-73.3 to -46)
		Male	29840 (23550 to 35737)	3198.3 (2573.4 to 3811.7)	394 (309 to 482)	48.3 (38.9 to 59.1)	13699 (11566 to 16287)	1459.8 (1238.6 to 1726.5)	209 (173 to 253)	22.5 (18.7 to 27.1)	-54.1 (-63.3 to -40.9)	-54.4 (-62.8 to -42.8)	-46.9 (-59.7 to -27.5)	-53.5 (-64.4 to -38)
	Falls	Both	8396 (6459 to 10917)	546.8 (435.9 to 677.7)	97 (73 to 127)	8.2 (6.4 to 9.9)	7030 (5614 to 8732)	379.4 (302.9 to 466.4)	98 (77 to 117)	5.6 (4.3 to 6.7)	-16.3 (-35.4 to 5.7)	-30.6 (-42.4 to -16.5)	0.7 (-24.6 to 39.4)	-31.5 (-47.2 to -8.1)
		Female	2447 (1362 to 3654)	326.1 (232.5 to 429.2)	26 (14 to 39)	5.2 (3.7 to 6.5)	1812 (1402 to 2318)	208.6 (161.8 to 263.8)	26 (18 to 32)	3.4 (2.4 to 4.1)	-25.9 (-52.6 to 23.6)	-36 (-52.7 to -16.9)	0.4 (-36.6 to 73.5)	-35.5 (-50.1 to -2.2)
		Male	5949 (3827 to 7846)	755.1 (548.2 to 953.6)	71 (43 to 96)	10.9 (7.8 to 14)	5218 (4120 to 6408)	547.4 (432.1 to 669.6)	72 (54 to 89)	7.8 (5.8 to 9.7)	-12.3 (-33.1 to 25.8)	-27.5 (-41.6 to -6.3)	0.8 (-29 to 66.2)	-28.2 (-48.3 to 3.5)
	Drowning	Both	6551 (4741 to 9261)	282.9 (206.3 to 389.9)	84 (62 to 117)	3.9 (2.9 to 5.2)	801 (637 to 1255)	48.7 (38.4 to 75.6)	14 (11 to 22)	0.8 (0.6 to 1.3)	-87.8 (-92.2 to -78.5)	-82.8 (-88.7 to -70.7)	-83.9 (-89.6 to -71.6)	-79.5 (-86.4 to -65.7)
		Female	1988 (1212 to 3289)	167.8 (105.4 to 272.4)	24 (15 to 39)	2.1 (1.4 to 3.3)	116 (93 to 142)	14.8 (11.6 to 18.5)	2 (1 to 2)	0.2 (0.2 to 0.3)	-94.2 (-96.6 to -89.5)	-91.2 (-94.8 to -84.4)	-92.6 (-95.8 to -87)	-89.5 (-93.7 to -82.3)
		Male	4563 (2958 to 6741)	390.3 (258.8 to 562.6)	60 (40 to 88)	5.5 (3.8 to 7.8)	685 (528 to 1126)	80.9 (62 to 133.3)	12 (9 to 20)	1.3 (1 to 2.3)	-85 (-91.1 to -71.3)	-79.3 (-87.3 to -61.2)	-80.5 (-88.2 to -62.9)	-75.6 (-84.6 to -55.8)
	Fire, heat, and hot substances	Both	8420 (4813 to 12917)	435.2 (269.3 to 639.4)	116 (68 to 175)	7.1 (4.7 to 10.1)	1975 (1512 to 2762)	109.1 (82.4 to 154.3)	39 (28 to 58)	2.1 (1.5 to 3.3)	-76.5 (-87.4 to -53.2)	-74.9 (-86.1 to -53.1)	-66.8 (-82.9 to -29.7)	-70 (-83.9 to -43.4)
		Female	3762 (912 to 7321)	404.9 (129.1 to 725.4)	51 (12 to 97)	6.6 (2.5 to 11)	885 (620 to 1309)	101.3 (70.1 to 150.1)	17 (11 to 29)	2 (1.2 to 3.4)	-76.5 (-90.6 to 14.6)	-75 (-89.5 to -6.9)	-66.5 (-88 to 78.2)	-69.6 (-87.9 to -0.9)
		Male	4658 (2066 to 6554)	464.1 (219.4 to 632.6)	65 (29 to 90)	7.7 (4.1 to 10.4)	1091 (813 to 1719)	116.4 (86.3 to 185.1)	22 (15 to 37)	2.3 (1.6 to 4)	-76.6 (-85.9 to -32.2)	-74.9 (-84.7 to -36.3)	-67.1 (-80.9 to -5.4)	-70.1 (-82.2 to -25.4)
	Poisonings	Both	5126 (3407 to 7419)	243.4 (166.9 to 338.1)	68 (46 to 95)	3.7 (2.6 to 5)	1208 (972 to 1456)	65.8 (52.7 to 79.2)	23 (18 to 28)	1.2 (1 to 1.5)	-76.4 (-84.2 to -64.7)	-72.9 (-81.2 to -60)	-66.6 (-77.5 to -51.9)	-66.7 (-76.6 to -53.5)
		Female	1789 (1047 to 2978)	164.9 (101.9 to 264.3)	22 (14 to 36)	2.3 (1.5 to 3.5)	306 (245 to 386)	35.6 (28.7 to 45.3)	5 (4 to 7)	0.6 (0.5 to 0.8)	-82.9 (-90.4 to -66.6)	-78.4 (-87.3 to -60.2)	-76.7 (-86.8 to -57.2)	-73.8 (-83.9 to -55.2)
		Male	3336 (2040 to 5098)	318.4 (199.3 to 468.4)	46 (29 to 68)	5 (3.3 to 7.1)	902 (658 to 1144)	94.9 (71 to 120.6)	18 (13 to 22)	1.8 (1.3 to 2.3)	-73 (-83.3 to -56.8)	-70.2 (-81 to -54)	-61.6 (-75.9 to -39.6)	-63.5 (-76.3 to -44.5)
	Exposure to mechanical forces	Both	5476 (4100 to 7510)	334.9 (253.2 to 456.2)	71 (50 to 106)	4.8 (3.4 to 7.2)	3532 (2834 to 4423)	190.3 (153.4 to 235)	48 (38 to 61)	2.6 (2.1 to 3.2)	-35.5 (-54.1 to -12.8)	-43.2 (-59.3 to -24.3)	-32.6 (-55.2 to -1.9)	-45.8 (-64.6 to -20.4)
		Female	1341 (810 to 1888)	158.2 (108.3 to 206.8)	15 (9 to 22)	2 (1.3 to 2.6)	750 (594 to 944)	85.1 (67.9 to 107.2)	8 (6 to 10)	0.9 (0.8 to 1.2)	-44.1 (-63 to -8.5)	-46.2 (-61.3 to -20)	-46 (-65.5 to 4.4)	-52 (-67 to -18.7)

Province	Cause	Sex	1990				2019				1990-2019 percent change (%)			
			DALYs		Death		DALYs		Death		DALYs		Death	
			Number	Rate	Number	Rate	Number	Rate	Number	Rate	Number	Rate	Number	Rate
		Male	4136 (2934 to 6240)	502.6 (366.1 to 748.2)	56 (38 to 91)	7.4 (5 to 12)	2782 (2200 to 3536)	292.2 (230.2 to 368)	40 (31 to 54)	4.2 (3.2 to 5.5)	-32.7 (-53.7 to -2)	-41.9 (-59.9 to -17.4)	-29 (-55.6 to 12.9)	-43.8 (-66 to -10.2)
	Adverse effects of medical treatment	Both	2857 (1749 to 3962)	168.3 (107.6 to 236.7)	48 (31 to 67)	3.8 (2.5 to 5.5)	893 (723 to 1084)	49.9 (40.9 to 60.6)	25 (20 to 31)	1.4 (1.1 to 1.8)	-68.7 (-78.7 to -48.2)	-70.3 (-80 to -52.2)	-48.8 (-67.8 to -15.4)	-63.6 (-77.2 to -41.1)
		Female	958 (595 to 1361)	112.4 (79.4 to 154.1)	15 (11 to 22)	2.5 (1.7 to 4.7)	297 (238 to 396)	34.7 (27.8 to 46.6)	8 (6 to 12)	1 (0.8 to 1.4)	-69 (-78.8 to -43.7)	-69.1 (-78.8 to -53.6)	-45.5 (-64.7 to -16.4)	-61.1 (-77.4 to -38.6)
		Male	1899 (1006 to 2972)	221.1 (115.1 to 346.6)	33 (17 to 51)	5 (2.6 to 7.7)	596 (434 to 763)	64.8 (48.1 to 82.1)	16 (12 to 21)	1.8 (1.3 to 2.3)	-68.6 (-81.1 to -42.2)	-70.7 (-82.5 to -43.8)	-50.3 (-71.9 to -1.4)	-64.4 (-79.5 to -29.1)
	Animal contact	Both	677 (408 to 1018)	32.2 (21.2 to 45.8)	8 (5 to 12)	0.4 (0.3 to 0.6)	178 (141 to 223)	10.3 (8.2 to 13)	2 (2 to 3)	0.1 (0.1 to 0.2)	-73.7 (-83.9 to -54.8)	-68 (-79.1 to -49.7)	-74.7 (-84.6 to -54.5)	-72 (-82.1 to -53.6)
		Female	279 (124 to 472)	27 (14 to 42.1)	3 (1 to 5)	0.3 (0.2 to 0.5)	81 (61 to 108)	9.6 (7.2 to 12.8)	1 (1 to 1)	0.1 (0.1 to 0.1)	-70.9 (-84.3 to -32.3)	-64.4 (-79.3 to -29.9)	-77.5 (-88.4 to -40.3)	-72.8 (-84.6 to -40.6)
		Male	398 (214 to 639)	37 (21.5 to 57.7)	5 (3 to 8)	0.5 (0.3 to 0.9)	97 (77 to 123)	11 (8.8 to 14.1)	1 (1 to 2)	0.2 (0.1 to 0.2)	-75.7 (-85.9 to -54)	-70.4 (-82.2 to -49)	-73 (-85.4 to -49.5)	-71.3 (-84.6 to -50.2)
	Foreign body	Both	3343 (2322 to 4707)	150.4 (108.7 to 205.7)	37 (26 to 53)	1.7 (1.3 to 2.4)	838 (694 to 1017)	50.7 (41.7 to 61)	10 (9 to 12)	0.6 (0.5 to 0.7)	-74.9 (-83.2 to -62.9)	-66.3 (-76.8 to -52.2)	-73 (-82.2 to -59)	-64.9 (-76 to -50.4)
		Female	1581 (1034 to 2488)	143.5 (96.6 to 220.9)	17 (11 to 28)	1.6 (1.1 to 2.5)	297 (237 to 368)	37.5 (29.6 to 46.6)	3 (3 to 4)	0.4 (0.3 to 0.5)	-81.2 (-89.2 to -68)	-73.8 (-84.7 to -56.5)	-81.8 (-89.1 to -68.2)	-74.3 (-84.5 to -56.8)
		Male	1762 (1168 to 2598)	156.9 (109.6 to 223.2)	20 (13 to 29)	1.8 (1.3 to 2.7)	541 (445 to 663)	63.3 (51.7 to 77.5)	7 (6 to 8)	0.8 (0.7 to 1)	-69.3 (-81.1 to -52.2)	-59.6 (-74.3 to -40.8)	-65.3 (-78.7 to -43.4)	-56.9 (-72.8 to -36.5)
	Environmental heat and cold exposure	Both	251 (183 to 385)	17.2 (12.6 to 25.5)	2 (1 to 4)	0.2 (0.1 to 0.4)	235 (171 to 317)	12.6 (9.2 to 16.8)	1 (1 to 2)	0.1 (0.1 to 0.1)	-6.4 (-34.2 to 11.8)	-26.9 (-45.4 to -14.9)	-25.1 (-54.2 to 15.9)	-53.1 (-69.4 to -26.9)
		Female	102 (71 to 154)	14.7 (10.4 to 20.9)	0 (0 to 1)	0.1 (0 to 0.2)	111 (78 to 153)	12 (8.6 to 16.5)	0 (0 to 1)	0 (0 to 0.1)	8.1 (-24.5 to 26.9)	-18.6 (-37.2 to -7.9)	-12.4 (-52.7 to 55.2)	-44.4 (-69 to 2)
		Male	148 (108 to 238)	19.6 (14.2 to 31.6)	1 (1 to 3)	0.2 (0.1 to 0.6)	124 (92 to 167)	13.1 (9.8 to 17.6)	1 (1 to 2)	0.1 (0.1 to 0.2)	-16.3 (-42.3 to 4.6)	-32.9 (-52.5 to -17.5)	-29 (-56.8 to 13.7)	-56 (-72.8 to -29.1)
	Exposure to forces of nature	Both	69 (58 to 82)	4.7 (3.9 to 5.7)	1 (1 to 1)	0 (0 to 0)	28 (21 to 38)	1.5 (1.1 to 1.9)	0 (0 to 0)	0 (0 to 0)	-58.9 (-64.7 to -52.4)	-68.9 (-73.4 to -64.3)	-83 (-83 to -83)	-85.5 (-85.5 to -85.5)
		Female	25 (21 to 30)	3.4 (2.8 to 4.3)	0 (0 to 0)	0 (0 to 0)	10 (7 to 14)	1 (0.8 to 1.4)	0 (0 to 0)	0 (0 to 0)	-59.7 (-66.1 to -52.2)	-69.7 (-74.4 to -64.7)	-83.4 (-83.4 to -83.4)	-85.9 (-85.9 to -85.9)
		Male	44 (37 to 53)	5.9 (4.8 to 7.1)	0 (0 to 0)	0 (0 to 0.1)	18 (14 to 24)	1.9 (1.4 to 2.5)	0 (0 to 0)	0 (0 to 0)	-58.5 (-64.2 to -52.1)	-68.2 (-72.7 to -63.3)	-82.7 (-82.7 to -82.7)	-85.3 (-85.3 to -85.3)
	Other unintentional injuries	Both	4230 (3276 to 5491)	237.6 (184.6 to 312.5)	48 (36 to 68)	2.8 (2.1 to 4)	2303 (1819 to 2887)	124.3 (98.9 to 155.1)	25 (19 to 32)	1.3 (1 to 1.7)	-45.6 (-61.9 to -26.9)	-47.7 (-62.8 to -31.6)	-47.9 (-68.1 to -21.9)	-51.4 (-70.8 to -28)
		Female	1283 (892 to 1761)	143.1 (102.8 to 189)	12 (8 to 18)	1.3 (0.9 to 1.9)	657 (481 to 891)	72.8 (53.5 to 98)	4 (3 to 5)	0.5 (0.4 to 0.6)	-48.8 (-67.2 to -22.6)	-49.2 (-65 to -29.4)	-68.2 (-81.5 to -40)	-65.9 (-79.5 to -40.9)
		Male	2947 (2133 to 4258)	327.3 (238.8 to 474.9)	36 (24 to 56)	4.1 (2.8 to 6.6)	1646 (1264 to 2066)	173.7 (133.9 to 217.1)	21 (15 to 28)	2.2 (1.6 to 2.9)	-44.2 (-66 to -18)	-46.9 (-66.9 to -24.3)	-40.9 (-68.9 to -2.6)	-46.6 (-72.2 to -11.2)
	Self-harm and interpersonal violence	Both	27449 (22223 to 33064)	1722.3 (1405.2 to 2071.8)	306 (234 to 367)	19.4 (14.9 to 23.5)	15771 (12312 to 18685)	816.2 (640 to 964.1)	219 (157 to 267)	11.4 (8.3 to 13.9)	-42.5 (-51.7 to -31)	-52.6 (-60.4 to -42.8)	-28.2 (-43.8 to -6.8)	-41 (-54 to -22.9)
		Female	7654 (5934 to 9901)	1005.7 (781.7 to 1303.1)	65 (49 to 86)	8.5 (6.4 to 11.2)	4039 (3236 to 5140)	430.4 (350.3 to 536.6)	40 (31 to 50)	4.5 (3.6 to 5.6)	-47.2 (-56.8 to -34.6)	-57.2 (-65.2 to -46.9)	-39 (-57.6 to -11.9)	-47.1 (-62.7 to -23.9)
		Male	19796 (15344 to 24029)	2396.4 (1869.3 to 2911.2)	240 (173 to 300)	29.7 (21.3 to 37.3)	11732 (8307 to 14277)	1188.3 (834.8 to 1440.9)	179 (116 to 227)	18.2 (11.7 to 22.7)	-40.7 (-52.3 to -24.7)	-50.4 (-60.3 to -36.7)	-25.3 (-44 to 1.1)	-38.9 (-54.6 to -15.5)
	Self-harm	Both	11148 (6782 to 14318)	680.4 (426 to 868.7)	185 (112 to 238)	12.4 (7.8 to 16)	8411 (5194 to 10564)	440.8 (272 to 552.1)	161 (98 to 201)	8.3 (5.1 to 10.4)	-24.6 (-44 to 6.1)	-35.2 (-51.4 to -8.6)	-13.3 (-36 to 24.6)	-32.8 (-49.8 to -4.2)

Province	Cause	Sex	1990				2019				1990-2019 percent change (%)			
			DALYs		Death		DALYs		Death		DALYs		Death	
			Number	Rate	Number	Rate	Number	Rate	Number	Rate	Number	Rate	Number	Rate
		Female	3406 (2462 to 4552)	414.5 (305.4 to 554.3)	53 (38 to 72)	7 (5.1 to 9.5)	1562 (1187 to 2005)	177.9 (135.8 to 229.5)	27 (21 to 35)	3.1 (2.4 to 3.9)	-54.1 (-69.5 to -29.7)	-57.1 (-71.3 to -35.5)	-48.6 (-66.1 to -20.4)	-55.9 (-70.1 to -32.5)
		Male	7742 (3631 to 10813)	934 (475.8 to 1282.3)	132 (64 to 182)	17.5 (9.2 to 23.9)	6848 (3581 to 8956)	693.7 (354.9 to 903.6)	133 (70 to 174)	13.4 (6.9 to 17.4)	-11.5 (-38.6 to 43.4)	-25.7 (-47.9 to 19)	1 (-30.1 to 63.7)	-23.7 (-46.5 to 22.4)
	Interpersonal violence	Both	3266 (2435 to 4220)	197.4 (150.4 to 248.4)	43 (30 to 57)	2.7 (1.9 to 3.6)	3409 (2838 to 4066)	185.4 (153.8 to 221.5)	46 (37 to 58)	2.5 (2 to 3.1)	4.4 (-19.5 to 39.8)	-6.1 (-26.7 to 23.5)	8.7 (-21.9 to 57.5)	-9.4 (-35.2 to 29.9)
		Female	1004 (726 to 1335)	119.9 (90 to 156.7)	10 (7 to 15)	1.2 (0.8 to 1.8)	1057 (841 to 1330)	119.4 (95 to 150.6)	11 (8 to 15)	1.2 (0.9 to 1.7)	5.2 (-25.8 to 47.9)	-0.4 (-27.4 to 36.6)	7.9 (-33.1 to 77.3)	0.4 (-37.2 to 65.9)
		Male	2262 (1561 to 3090)	270.8 (190 to 361)	33 (21 to 46)	4.2 (2.6 to 5.8)	2352 (1880 to 2964)	249 (198.2 to 315.2)	35 (27 to 46)	3.7 (2.8 to 4.9)	4 (-27.1 to 51)	-8.1 (-34.6 to 31.5)	9 (-28.3 to 71.4)	-11.5 (-41.6 to 39.4)
	Conflict and terrorism	Both	12319 (9459 to 16699)	798.5 (597.7 to 1083.7)	67 (61 to 74)	3.5 (3.2 to 3.9)	3171 (2076 to 4913)	149.7 (98.8 to 230.3)	1 (1 to 1)	0 (0 to 0)	-74.3 (-78.6 to -69.9)	-81.2 (-84.6 to -77.8)	-99 (-99 to -99)	-98.9 (-98.9 to -98.9)
		Female	3117 (1906 to 5232)	454.5 (285.7 to 736.5)	0 (0 to 0)	0 (0 to 0)	1315 (798 to 2273)	121.5 (74.2 to 207.6)	0 (0 to 0)	0 (0 to 0)	-57.8 (-63.5 to -52.3)	-73.3 (-78 to -68.3)	-3.9 (-3.9 to -3.9)	-13.1 (-13.1 to -13.1)
		Male	9202 (7457 to 11617)	1117.8 (891.3 to 1438.4)	67 (61 to 73)	6.8 (6.1 to 7.4)	1857 (1245 to 2733)	177.9 (121.1 to 261.4)	0 (0 to 1)	0.1 (0 to 0.1)	-79.8 (-83.6 to -75.9)	-84.1 (-86.9 to -81.1)	-99.3 (-99.3 to -99.3)	-99.2 (-99.2 to -99.2)
	Executions and police conflict	Both	716 (603 to 886)	45.9 (38.7 to 56.7)	11 (9 to 14)	0.7 (0.6 to 0.9)	780 (605 to 1094)	40.3 (31.7 to 56)	12 (9 to 17)	0.6 (0.5 to 0.9)	8.9 (-18.3 to 54.3)	-12.4 (-33.5 to 23.1)	7.8 (-22.8 to 62.8)	-19.2 (-41.1 to 21.3)
		Female	126 (111 to 144)	16.9 (14.7 to 19.6)	2 (2 to 2)	0.2 (0.2 to 0.3)	105 (89 to 126)	11.6 (10 to 13.7)	1 (1 to 1)	0.1 (0.1 to 0.2)	-16.9 (-22.5 to -8.8)	-31.3 (-36.2 to -25.8)	-18.7 (-20 to -17.1)	-37.4 (-38.3 to -36.3)
		Male	590 (487 to 757)	73.7 (60.7 to 95.9)	9 (8 to 12)	1.2 (1 to 1.6)	675 (508 to 983)	67.7 (51.7 to 98.4)	10 (7 to 16)	1 (0.8 to 1.5)	14.4 (-18.5 to 70.9)	-8.1 (-33.6 to 36.2)	12.6 (-23.3 to 78.4)	-15.8 (-42 to 33)
**Hormozgan**	**All causes**	Both	444229 (393655 to 498931)	48159.8 (43312.9 to 53357.7)	6231 (5570 to 7010)	1098.8 (985 to 1217.8)	415505 (358855 to 474863)	25895.3 (22573.1 to 29357.1)	6798 (6165 to 7499)	595.4 (543.2 to 652.9)	-6.5 (-18.7 to 8.2)	-46.2 (-52.1 to -39.5)	9.1 (-5.8 to 27.2)	-45.8 (-52.9 to -37.4)
		Female	196954 (172777 to 222476)	43300.7 (38121 to 49103.9)	2574 (2271 to 2911)	921.6 (810.4 to 1044.5)	182666 (153293 to 215668)	23508.3 (20168.3 to 27200.8)	2690 (2407 to 2939)	530.3 (477.9 to 573.4)	-7.3 (-20.3 to 8.8)	-45.7 (-52 to -38.6)	4.5 (-11.3 to 23.6)	-42.5 (-51 to -32.4)
		Male	247274 (217849 to 279235)	52556.2 (46130.4 to 59274.9)	3656 (3163 to 4212)	1268.1 (1074.9 to 1478.3)	232839 (201583 to 266406)	28334 (24540.5 to 32308.6)	4108 (3551 to 4755)	668.8 (577.2 to 776.2)	-5.8 (-19.6 to 10.4)	-46.1 (-53.9 to -36.5)	12.3 (-7.9 to 38.1)	-47.3 (-57.3 to -33.7)
	**Communicable, maternal, neonatal, and nutritional diseases**	Both	192294 (154849 to 256981)	13031.8 (10453 to 20536.1)	2226 (1767 to 3425)	181.4 (143.1 to 369)	51005 (44216 to 58580)	2793.5 (2436.9 to 3193)	592 (522 to 670)	39.7 (35.4 to 44.6)	-73.5 (-80.4 to -65.5)	-78.6 (-86.3 to -72.1)	-73.4 (-82.7 to -65.1)	-78.1 (-89.3 to -71.4)
		Female	88209 (71195 to 116028)	12267 (9939.2 to 18210.8)	1009 (801 to 1460)	164.7 (129.5 to 300.4)	22764 (19549 to 26342)	2564 (2213.4 to 2950)	252 (218 to 289)	35.8 (31.1 to 40.9)	-74.2 (-80.9 to -66.5)	-79.1 (-85.9 to -73.1)	-75 (-82.6 to -66.8)	-78.2 (-87.8 to -71.6)
		Male	104085 (82614 to 142609)	13742.9 (10918.9 to 22650.2)	1217 (952 to 1961)	197.9 (151.8 to 442.8)	28241 (24357 to 32712)	3015.6 (2614.2 to 3470.2)	340 (297 to 390)	43.8 (38 to 50)	-72.9 (-80.5 to -64.4)	-78.1 (-87 to -71)	-72.1 (-82.7 to -62.5)	-77.9 (-90.3 to -69.7)
	HIV/AIDS and sexually transmitted infections	Both	393 (227 to 703)	36.8 (24.1 to 56.4)	4 (2 to 7)	0.4 (0.2 to 0.6)	4978 (3795 to 6381)	240.1 (181.8 to 312.2)	81 (60 to 109)	4.1 (3 to 5.6)	1167.9 (590.8 to 2179.3)	552.3 (303.3 to 920)	1955.5 (947.3 to 4266.9)	1012.8 (516.2 to 2032.2)
		Female	180 (100 to 313)	35.1 (22.3 to 54.6)	2 (1 to 3)	0.3 (0.1 to 0.5)	2207 (1547 to 2953)	223 (155.2 to 300)	35 (23 to 50)	3.7 (2.5 to 5.5)	1126.5 (567.3 to 2168.2)	535.6 (275.5 to 994.1)	2129.2 (939.4 to 5288.9)	1309.8 (612.2 to 2773.7)

Province	Cause	Sex	1990				2019				1990-2019 percent change (%)			
			DALYs		Death		DALYs		Death		DALYs		Death	
			Number	Rate	Number	Rate	Number	Rate	Number	Rate	Number	Rate	Number	Rate
		Male	213 (121 to 377)	38.5 (24.7 to 58.6)	2 (1 to 4)	0.5 (0.2 to 0.7)	2771 (2214 to 3515)	254.9 (201.8 to 324.4)	46 (36 to 62)	4.4 (3.5 to 6)	1203 (610.4 to 2263.3)	562.2 (304.9 to 968.1)	1841.7 (936.6 to 3794.7)	849.6 (454.1 to 1778.7)
	HIV/AIDS	Both	98 (48 to 137)	12.2 (5.7 to 17.2)	1 (0 to 2)	0.2 (0.1 to 0.3)	4602 (3419 to 6004)	221.1 (164.3 to 290.1)	79 (57 to 107)	4 (2.9 to 5.5)	4614.8 (2775.6 to 9445.8)	1713.6 (993.6 to 3941.5)	5488.9 (3061.7 to 15006.9)	1878.8 (995.2 to 5761.8)
		Female	30 (15 to 44)	6.6 (3.2 to 9.4)	0 (0 to 1)	0.1 (0 to 0.2)	1993 (1352 to 2709)	201.2 (135.6 to 276.5)	34 (22 to 49)	3.6 (2.3 to 5.4)	6591.1 (3679.5 to 13301.4)	2953.1 (1591.5 to 6333.1)	8248.9 (4211.5 to 20307.7)	3315.1 (1585.2 to 9288.1)
		Male	68 (32 to 96)	17.4 (7.8 to 24.8)	1 (0 to 1)	0.3 (0.1 to 0.4)	2609 (2063 to 3347)	238.6 (188 to 307.8)	45 (35 to 61)	4.3 (3.3 to 5.8)	3747.1 (2348.2 to 7671.7)	1267.9 (764.8 to 3039)	4376.7 (2569.9 to 13532)	1375.7 (778.2 to 4670.2)
	Sexually transmitted infections excluding HIV	Both	295 (133 to 599)	24.6 (13.6 to 44.3)	3 (1 to 6)	0.2 (0.1 to 0.4)	376 (223 to 616)	19 (11.2 to 31.4)	2 (1 to 4)	0.1 (0.1 to 0.2)	27.6 (-14 to 101.3)	-22.8 (-39.8 to -5.5)	-13.6 (-40.1 to 42.8)	-19.2 (-45.9 to 34.7)
		Female	150 (73 to 284)	28.5 (16 to 48.6)	1 (0 to 3)	0.2 (0.1 to 0.3)	215 (127 to 343)	21.9 (12.9 to 35.5)	1 (0 to 2)	0.1 (0.1 to 0.2)	43 (-4.6 to 116.5)	-23.2 (-38.4 to -10)	-17.1 (-42.2 to 33.2)	-23.2 (-46.9 to 19.5)
		Male	145 (60 to 315)	21.1 (10.3 to 40.6)	1 (0 to 3)	0.2 (0.1 to 0.4)	162 (93 to 268)	16.4 (9.2 to 27.6)	1 (0 to 2)	0.1 (0.1 to 0.3)	11.6 (-25 to 87.4)	-22.3 (-43.3 to 4.7)	-10.7 (-41.5 to 62.2)	-14.6 (-53.2 to 62.9)
	Respiratory infections and tuberculosis	Both	36948 (26049 to 49776)	2704.5 (1994.9 to 3459.9)	463 (326 to 604)	49.8 (37.7 to 59.4)	8724 (7442 to 10304)	539.7 (471.5 to 625.3)	185 (162 to 210)	16.5 (14.3 to 19)	-76.4 (-83.6 to -64.8)	-80 (-85.2 to -71.4)	-60 (-70.3 to -42.9)	-66.9 (-73.3 to -56.2)
		Female	17991 (12039 to 24750)	2617.6 (1821 to 3477.3)	221 (149 to 298)	45.5 (33.4 to 56.3)	3685 (3036 to 4430)	474.6 (397.4 to 557.3)	78 (65 to 90)	15.3 (12.4 to 17.9)	-79.5 (-86.1 to -67.2)	-81.9 (-87.1 to -72.5)	-64.8 (-75.3 to -46.6)	-66.4 (-74.7 to -54.2)
		Male	18957 (13229 to 25834)	2785.5 (2039.4 to 3598.6)	242 (175 to 321)	54.4 (41.6 to 65.8)	5039 (4240 to 6006)	606 (507.8 to 714)	107 (89 to 129)	17.9 (14.7 to 21.8)	-73.4 (-81.9 to -59.9)	-78.2 (-84.2 to -69)	-55.7 (-68.8 to -34.2)	-67.1 (-74.7 to -55.5)
	Tuberculosis	Both	2045 (1237 to 2538)	259.9 (166.1 to 314.2)	40 (25 to 48)	7.8 (5.3 to 9.5)	693 (591 to 804)	43.4 (37.2 to 50.5)	18 (15 to 22)	1.5 (1.3 to 1.8)	-66.1 (-74.2 to -43.3)	-83.3 (-86.9 to -72.4)	-53.9 (-64.6 to -21.7)	-81 (-85.2 to -69.5)
		Female	775 (365 to 1064)	191.3 (89.6 to 256.5)	14 (6 to 19)	5.5 (2.3 to 7.5)	215 (174 to 283)	29 (23.7 to 39.2)	6 (5 to 8)	1.1 (0.8 to 1.5)	-72.3 (-81.5 to -25.5)	-84.9 (-89.4 to -57.1)	-58.7 (-71.9 to 30.3)	-81 (-87.5 to -35.6)
		Male	1270 (777 to 1620)	324.6 (210.1 to 406.6)	26 (17 to 32)	10.3 (7.4 to 13.2)	479 (400 to 570)	57.2 (47.7 to 68.3)	12 (10 to 15)	1.9 (1.6 to 2.3)	-62.3 (-72 to -36.8)	-82.4 (-86.7 to -71.7)	-51.4 (-64 to -20.3)	-81.5 (-86.2 to -72.9)
	Lower respiratory infections	Both	33675 (23429 to 46149)	2326.3 (1656 to 3082.4)	424 (300 to 564)	41.9 (31.9 to 50.9)	5779 (4981 to 6658)	381.7 (332.8 to 434)	167 (146 to 190)	15 (12.9 to 17.3)	-82.8 (-88.3 to -73.6)	-83.6 (-88.2 to -75.9)	-60.6 (-71.2 to -42.9)	-64.3 (-71.8 to -52.6)
		Female	16642 (10818 to 23198)	2313 (1570 to 3146.9)	207 (140 to 283)	39.9 (29.4 to 49.8)	2421 (1985 to 2889)	335.8 (276.3 to 393.9)	72 (59 to 84)	14.2 (11.5 to 16.8)	-85.5 (-90.5 to -76.2)	-85.5 (-90.2 to -77.6)	-65.2 (-76 to -46.7)	-64.4 (-73.6 to -50.7)
		Male	17033 (11569 to 23638)	2337.9 (1670.7 to 3132.3)	216 (154 to 293)	44.2 (33.8 to 54.6)	3358 (2755 to 3962)	429.5 (356.3 to 506.5)	95 (78 to 114)	16 (13.1 to 19.6)	-80.3 (-87.1 to -69.5)	-81.6 (-87.4 to -72.6)	-56.3 (-70 to -34.5)	-63.8 (-72.7 to -49.9)
	Upper respiratory infections	Both	872 (527 to 1394)	84.7 (50.8 to 132.2)	0 (0 to 1)	0 (0 to 0)	1680 (1009 to 2642)	85.4 (51.6 to 134.3)	0 (0 to 1)	0 (0 to 0)	92.6 (77.9 to 109.6)	0.8 (-5.1 to 7)	149.1 (-90.5 to 6575.8)	95.5 (-92.7 to 3250.4)
		Female	418 (250 to 659)	82.7 (49.8 to 129.8)	0 (0 to 0)	0 (0 to 0)	798 (477 to 1258)	83.3 (50.2 to 132)	0 (0 to 0)	0 (0 to 0)	91 (75.4 to 109.3)	0.7 (-6.3 to 8.8)	154.3 (-94.1 to 9834.8)	141.1 (-94.3 to 5164.9)
		Male	454 (272 to 712)	86.6 (52.1 to 134.9)	0 (0 to 0)	0 (0 to 0.1)	882 (530 to 1378)	87.4 (53 to 136.6)	0 (0 to 0)	0 (0 to 0.1)	94.1 (74.2 to 115)	0.9 (-7.3 to 9)	146.1 (-88.5 to 5798.5)	78 (-91.8 to 2870)
	Otitis media	Both	356 (203 to 586)	33.6 (18.9 to 55.3)	0 (0 to 0)	0 (0 to 0)	572 (324 to 958)	29.2 (16.7 to 48.5)	0 (0 to 0)	0 (0 to 0)	60.4 (46.3 to 76.5)	-12.9 (-18.8 to -6.5)	-55.7 (-79.2 to 19.5)	-71.1 (-86.1 to -25.2)

Province	Cause	Sex	1990				2019				1990-2019 percent change (%)			
			DALYs		Death		DALYs		Death		DALYs		Death	
			Number	Rate	Number	Rate	Number	Rate	Number	Rate	Number	Rate	Number	Rate
		Female	157 (87 to 257)	30.6 (17.1 to 50.3)	0 (0 to 0)	0 (0 to 0)	251 (142 to 426)	26.4 (15.1 to 44.7)	0 (0 to 0)	0 (0 to 0)	60.6 (42.9 to 80.6)	-13.7 (-21.7 to -5.1)	51.4 (-65.6 to 581.8)	-21.2 (-84.9 to 252.1)
		Male	200 (112 to 330)	36.4 (20.6 to 60.1)	0 (0 to 0)	0 (0 to 0)	321 (178 to 536)	31.9 (18 to 53.2)	0 (0 to 0)	0 (0 to 0)	60.3 (43.6 to 80.2)	-12.3 (-20.4 to -4.4)	-73.2 (-89.5 to -7.1)	-83.9 (-93 to -48.1)
	Enteric infections	Both	19127 (12415 to 28195)	1330.4 (892.8 to 1895.2)	220 (140 to 324)	20.8 (12.5 to 30.3)	5613 (4295 to 7189)	317.3 (243 to 404.6)	38 (25 to 52)	2.8 (1.8 to 3.8)	-70.7 (-81.7 to -53.3)	-76.2 (-84.4 to -64.1)	-82.6 (-89.5 to -71.7)	-86.7 (-90.8 to -80.6)
		Female	8023 (4523 to 12751)	1134 (663.2 to 1734.6)	91 (48 to 148)	17.6 (8.5 to 29.6)	2470 (1858 to 3218)	284.7 (215.8 to 371.2)	14 (9 to 22)	2.1 (1.2 to 3.7)	-69.2 (-82.6 to -42.7)	-74.9 (-85.3 to -55.5)	-84.8 (-92.3 to -69.4)	-87.9 (-92.9 to -77.1)
		Male	11103 (6807 to 17998)	1517.5 (945.6 to 2386.9)	128 (75 to 209)	24 (13.4 to 36.3)	3143 (2318 to 4068)	349 (259.3 to 448.7)	24 (14 to 34)	3.4 (1.8 to 4.7)	-71.7 (-83.7 to -50.6)	-77 (-85.9 to -62.3)	-81 (-90.6 to -66.1)	-85.8 (-91.7 to -76.8)
	Diarrheal diseases	Both	18423 (11929 to 27318)	1274.2 (842 to 1823.1)	210 (133 to 311)	20 (11.7 to 29.1)	5157 (3851 to 6793)	293.8 (220.5 to 383.7)	32 (19 to 44)	2.4 (1.4 to 3.5)	-72 (-82.8 to -54.6)	-76.9 (-85.1 to -64.5)	-84.9 (-91.4 to -75)	-87.9 (-91.9 to -82.1)
		Female	7665 (4277 to 12358)	1076.3 (624.3 to 1671.1)	86 (45 to 142)	16.7 (7.9 to 28.7)	2292 (1678 to 3043)	266 (196.3 to 353.5)	11 (6 to 19)	1.8 (1 to 3.4)	-70.1 (-83.5 to -43.3)	-75.3 (-85.8 to -55.5)	-87 (-93.9 to -72.3)	-88.9 (-93.9 to -78.2)
		Male	10757 (6461 to 17647)	1462.7 (889.3 to 2326.6)	124 (70 to 204)	23.2 (12.6 to 35.4)	2865 (2074 to 3746)	321.1 (234.7 to 419.7)	20 (10 to 30)	3 (1.4 to 4.3)	-73.4 (-84.9 to -52.9)	-78 (-86.8 to -63.5)	-83.5 (-92.6 to -69)	-87.1 (-93 to -78.3)
	Typhoid and paratyphoid	Both	502 (163 to 1026)	41 (13.6 to 80.7)	7 (2 to 14)	0.6 (0.2 to 1.2)	329 (133 to 631)	16.8 (6.8 to 32.8)	5 (2 to 9)	0.2 (0.1 to 0.5)	-34.5 (-55.9 to 64.9)	-58.9 (-72.2 to 4)	-31.3 (-53.3 to 77)	-60 (-71.8 to -0.4)
		Female	239 (71 to 505)	39.6 (12.3 to 81.1)	3 (1 to 7)	0.6 (0.2 to 1.2)	127 (54 to 251)	13.3 (5.6 to 25.9)	2 (1 to 4)	0.2 (0.1 to 0.4)	-46.6 (-66.4 to 48.7)	-66.5 (-78.2 to -3.6)	-43.6 (-63.9 to 66.6)	-67.1 (-78.1 to -6)
		Male	263 (89 to 536)	42.2 (14.5 to 83.5)	4 (1 to 7)	0.6 (0.2 to 1.2)	202 (80 to 406)	20.2 (8 to 41.5)	3 (1 to 6)	0.3 (0.1 to 0.6)	-23.4 (-47.9 to 74.7)	-52.1 (-67 to 8.6)	-20 (-44.6 to 87.7)	-53.5 (-67.2 to 3)
	Invasive Non-typhoidal Salmonella (iNTS)	Both	163 (74 to 288)	12.7 (5.7 to 22.5)	2 (1 to 4)	0.2 (0.1 to 0.4)	118 (59 to 212)	6.2 (3.1 to 10.9)	2 (1 to 3)	0.1 (0.1 to 0.2)	-27.3 (-57.4 to 43.7)	-51.4 (-69.3 to -3.2)	-17.5 (-48.2 to 66.1)	-51 (-65.6 to -11.2)
		Female	97 (41 to 173)	15 (6.5 to 26.4)	1 (1 to 2)	0.2 (0.1 to 0.4)	47 (23 to 83)	5 (2.5 to 8.8)	1 (0 to 1)	0.1 (0 to 0.2)	-51.7 (-72.9 to 4.8)	-66.8 (-80.2 to -30.3)	-41.5 (-65.1 to 24.1)	-63.2 (-75.3 to -29.7)
		Male	66 (31 to 119)	10.4 (5 to 18.3)	1 (0 to 2)	0.2 (0.1 to 0.3)	72 (35 to 129)	7.3 (3.5 to 13)	1 (1 to 2)	0.1 (0.1 to 0.2)	8.4 (-37.1 to 91.8)	-30.3 (-56.1 to 26.3)	15.8 (-26.8 to 110.2)	-35.1 (-54.9 to 11.1)
	Other intestinal infectious diseases	Both	40 (8 to 87)	2.6 (0.6 to 5.5)	0 (0 to 1)	0 (0 to 0.1)	9 (5 to 13)	0.5 (0.3 to 0.7)	0 (0 to 0)	0 (0 to 0)	-78.1 (-91.4 to 22.7)	-82.7 (-93 to -26.1)	-81.9 (-94 to 14)	-84.8 (-94.2 to -44.9)
		Female	23 (2 to 61)	3 (0.4 to 7.6)	0 (0 to 1)	0 (0 to 0.1)	4 (2 to 7)	0.4 (0.2 to 0.7)	0 (0 to 0)	0 (0 to 0)	-82.5 (-95.1 to 121.7)	-85.8 (-96 to 23.5)	-86 (-96.8 to 148.5)	-86.9 (-96.7 to -8.6)
		Male	17 (2 to 44)	2.2 (0.4 to 5.4)	0 (0 to 1)	0 (0 to 0.1)	5 (3 to 7)	0.5 (0.3 to 0.7)	0 (0 to 0)	0 (0 to 0)	-72 (-91.3 to 125.2)	-78.6 (-92.9 to 24.2)	-76.3 (-94.6 to 174.8)	-81.8 (-94.8 to -9.2)
	Neglected tropical diseases and malaria	Both	8851 (631 to 97147)	905 (56.9 to 10299.5)	139 (3 to 1668)	18.8 (0.4 to 232.4)	387 (260 to 561)	20.8 (14.1 to 29.7)	2 (1 to 4)	0.2 (0.1 to 0.3)	-95.6 (-99.6 to -41.8)	-97.7 (-99.8 to -65.2)	-98.2 (-99.9 to -22.9)	-99.1 (-99.9 to -55.2)
		Female	4373 (304 to 46353)	834.2 (60.3 to 9202.5)	61 (1 to 720)	14.6 (0.3 to 181.8)	187 (119 to 267)	20.9 (13.6 to 29.5)	1 (0 to 2)	0.1 (0.1 to 0.2)	-95.7 (-99.6 to -42)	-97.5 (-99.8 to -67.9)	-98.3 (-99.8 to -13.1)	-99 (-99.9 to -50.1)
		Male	4478 (288 to 50088)	965 (49.4 to 11281.1)	78 (2 to 955)	22.7 (0.4 to 285)	201 (127 to 316)	20.8 (13.4 to 31.9)	1 (1 to 3)	0.2 (0.1 to 0.3)	-95.5 (-99.6 to -30.4)	-97.8 (-99.8 to -58.6)	-98.2 (-99.8 to -14.3)	-99.2 (-99.9 to -52.5)
	Malaria	Both	8067 (107 to 96666)	837.8 (10.1 to 10250.4)	133 (0 to 1659)	18.2 (0 to 231.8)	24 (16 to 35)	1.2 (0.8 to 1.8)	0 (0 to 0)	0 (0 to 0)	-99.7 (-100 to -76.1)	-99.9 (-100 to -87)	-100 (-100 to -91.5)	-100 (-100 to -95.7)
		Female	4017 (66 to 46026)	768.6 (12.8 to 9146.1)	58 (0 to 719)	14.1 (0 to 181.5)	12 (8 to 18)	1.3 (0.9 to 1.9)	0 (0 to 0)	0 (0 to 0)	-99.7 (-100 to -79.8)	-99.8 (-100 to -89.3)	-100 (-100 to -92.5)	-100 (-100 to -96)
		Male	4050 (41 to 49693)	896.3 (7 to 11219.1)	74 (0 to 952)	21.9 (0 to 284.7)	12 (8 to 17)	1.2 (0.8 to 1.7)	0 (0 to 0)	0 (0 to 0)	-99.7 (-100 to -70.3)	-99.9 (-100 to -83.4)	-100 (-100 to -90.6)	-100 (-100 to -95.7)

Province	Cause	Sex	1990				2019				1990-2019 percent change (%)			
			DALYs		Death		DALYs		Death		DALYs		Death	
			Number	Rate	Number	Rate	Number	Rate	Number	Rate	Number	Rate	Number	Rate
	Leishmaniasis	Both	202 (1 to 2092)	16.2 (0.1 to 160.1)	3 (0 to 28)	0.3 (0 to 2.4)	14 (1 to 128)	0.7 (0 to 6.4)	0 (0 to 2)	0 (0 to 0.1)	-93 (-94.7 to 27.3)	-95.7 (-96.8 to -25.7)	-93.1 (-94.5 to -85.2)	-96.2 (-97.1 to -93.5)
		Female	66 (1 to 677)	10.7 (0.1 to 103.6)	1 (0 to 9)	0.2 (0 to 1.5)	5 (0 to 41)	0.5 (0 to 4.1)	0 (0 to 1)	0 (0 to 0.1)	-92.6 (-94.9 to 78.9)	-95.3 (-96.8 to -2.7)	-93.2 (-94.9 to -83.6)	-96.2 (-97.3 to -93.4)
		Male	136 (1 to 1455)	21.4 (0.1 to 217.7)	2 (0 to 19)	0.3 (0 to 3.2)	9 (0 to 89)	0.9 (0 to 8.6)	0 (0 to 1)	0 (0 to 0.1)	-93.3 (-94.8 to -7.1)	-95.9 (-96.9 to -42.6)	-93 (-94.6 to -85.7)	-96.2 (-97.2 to -93.5)
	Schistosomiasis	Both	0 (0 to 0)	0 (0 to 0)	0 (0 to 0)	0 (0 to 0)	0 (0 to 0)	0 (0 to 0)	0 (0 to 0)	0 (0 to 0)	0 (0 to 0)	0 (0 to 0)	0 (0 to 0)	0 (0 to 0)
		Female	0 (0 to 0)	0 (0 to 0)	0 (0 to 0)	0 (0 to 0)	0 (0 to 0)	0 (0 to 0)	0 (0 to 0)	0 (0 to 0)	0 (0 to 0)	0 (0 to 0)	0 (0 to 0)	0 (0 to 0)
		Male	0 (0 to 0)	0 (0 to 0)	0 (0 to 0)	0 (0 to 0)	0 (0 to 0)	0 (0 to 0)	0 (0 to 0)	0 (0 to 0)	0 (0 to 0)	0 (0 to 0)	0 (0 to 0)	0 (0 to 0)
	Cystic echinococcosis	Both	72 (33 to 126)	7.7 (4.5 to 11.4)	1 (0 to 2)	0.1 (0.1 to 0.2)	51 (35 to 72)	2.8 (2 to 3.9)	0 (0 to 0)	0 (0 to 0)	-28.9 (-61.7 to 50.7)	-63.3 (-77.4 to -34.6)	-64.9 (-84.2 to -10.9)	-80.6 (-90.3 to -57.9)
		Female	31 (11 to 60)	6.7 (3.5 to 10.9)	0 (0 to 1)	0.1 (0 to 0.2)	26 (17 to 39)	2.9 (1.9 to 4.3)	0 (0 to 0)	0 (0 to 0)	-14.1 (-60.9 to 129)	-56.2 (-76.3 to -14.1)	-64.9 (-88.8 to 39.2)	-78.6 (-92.8 to -33.3)
		Male	41 (15 to 80)	8.6 (4.3 to 13.9)	1 (0 to 1)	0.2 (0.1 to 0.3)	25 (16 to 35)	2.7 (1.7 to 3.8)	0 (0 to 0)	0 (0 to 0)	-39.9 (-72.7 to 74.6)	-68.5 (-83.8 to -31.7)	-64.9 (-88.2 to 23.1)	-81.7 (-93.5 to -51.6)
	Trachoma	Both	14 (8 to 23)	3.9 (2.2 to 6.5)	--	--	4 (2 to 7)	0.4 (0.2 to 0.7)	--	--	-67.7 (-74.8 to -59.6)	-89 (-91.6 to -86.1)	--	--
		Female	10 (6 to 17)	6.1 (3.5 to 10.2)	--	--	3 (2 to 5)	0.6 (0.3 to 1)	--	--	-69.6 (-77.3 to -59.4)	-90.3 (-92.8 to -87.2)	--	--
		Male	4 (2 to 7)	1.8 (0.9 to 3.2)	--	--	1 (1 to 2)	0.3 (0.1 to 0.5)	--	--	-62.2 (-74.5 to -44.1)	-86 (-90.8 to -79.8)	--	--
	Rabies	Both	8 (1 to 15)	1 (0.1 to 1.8)	0 (0 to 0)	0 (0 to 0)	5 (1 to 8)	0.3 (0 to 0.5)	0 (0 to 0)	0 (0 to 0)	-29 (-65.5 to 64.2)	-70.1 (-84 to -34)	-26.3 (-60.1 to 55.5)	-72.1 (-84.1 to -46.3)
		Female	2 (0 to 5)	0.9 (0 to 1.9)	0 (0 to 0)	0 (0 to 0.1)	2 (0 to 3)	0.2 (0 to 0.3)	0 (0 to 0)	0 (0 to 0)	-35.9 (-76.8 to 48.2)	-79.8 (-89.8 to -52.6)	-41.1 (-69.8 to 31.1)	-81.7 (-90.9 to -57.1)
		Male	5 (1 to 12)	1.1 (0.2 to 2.3)	0 (0 to 0)	0 (0 to 0)	4 (1 to 7)	0.4 (0.1 to 0.7)	0 (0 to 0)	0 (0 to 0)	-25.8 (-69.1 to 104.3)	-63.3 (-83.9 to -0.5)	-16.3 (-62.9 to 114.2)	-62.2 (-83.8 to -11.4)
	Intestinal nematode infections	Both	125 (46 to 237)	9.5 (4.3 to 16.8)	1 (0 to 2)	0.1 (0 to 0.2)	24 (13 to 39)	1.3 (0.7 to 2)	0 (0 to 0)	0 (0 to 0)	-80.7 (-91.6 to -48.8)	-86.7 (-93.7 to -71.2)	-91.8 (-97.2 to -59.4)	-92.1 (-97 to -72)
		Female	64 (21 to 139)	10.5 (4.4 to 20.4)	0 (0 to 1)	0.1 (0 to 0.2)	14 (7 to 24)	1.6 (0.8 to 2.6)	0 (0 to 0)	0 (0 to 0)	-77.2 (-92 to -30)	-85.1 (-94 to -63.9)	-92.5 (-98.4 to 31.3)	-92 (-98.2 to -36.4)
		Male	62 (13 to 145)	8.6 (2.5 to 18.5)	1 (0 to 2)	0.1 (0 to 0.2)	10 (5 to 17)	1 (0.5 to 1.7)	0 (0 to 0)	0 (0 to 0)	-84.2 (-94.8 to -27.1)	-88.4 (-95.8 to -60.6)	-91.3 (-98 to 20.3)	-92.1 (-97.9 to -52.5)
	Food-borne trematodiases	Both	3 (1 to 6)	0.5 (0.1 to 1)	--	--	18 (4 to 37)	1 (0.2 to 2.1)	--	--	480.3 (320.9 to 646.4)	107.2 (54.8 to 160.7)	--	--
		Female	1 (0 to 3)	0.4 (0.1 to 0.8)	--	--	7 (2 to 14)	0.8 (0.2 to 1.6)	--	--	465.7 (340.9 to 613.7)	99.7 (48.3 to 157.8)	--	--
		Male	2 (0 to 4)	0.6 (0.1 to 1.1)	--	--	11 (2 to 24)	1.2 (0.3 to 2.5)	--	--	489.9 (252 to 759.3)	114.2 (39.5 to 198.5)	--	--
	Leprosy	Both	1 (0 to 1)	0.1 (0.1 to 0.1)	--	--	1 (0 to 1)	0 (0 to 0.1)	--	--	19 (7.4 to 32)	-60.3 (-64.1 to -56)	--	--

Province	Cause	Sex	1990				2019				1990-2019 percent change (%)			
			DALYs		Death		DALYs		Death		DALYs		Death	
			Number	Rate	Number	Rate	Number	Rate	Number	Rate	Number	Rate	Number	Rate
		Female	0 (0 to 0)	0.1 (0 to 0.1)	--	--	0 (0 to 0)	0 (0 to 0)	--	--	14.3 (-4.7 to 35.4)	-63.2 (-68.7 to -56.9)	--	--
		Male	0 (0 to 1)	0.1 (0.1 to 0.2)	--	--	0 (0 to 1)	0.1 (0 to 0.1)	--	--	21.4 (5.9 to 36.7)	-58 (-63 to -52.9)	--	--
	Other neglected tropical diseases	Both	360 (231 to 521)	28.3 (18.9 to 40.3)	1 (1 to 2)	0.2 (0.1 to 0.2)	245 (148 to 371)	13 (7.9 to 19.6)	2 (1 to 2)	0.1 (0 to 0.2)	-31.8 (-57.4 to -1.4)	-54 (-69.9 to -35.8)	24.6 (-46.8 to 137.2)	-27.3 (-62.6 to 24.7)
		Female	181 (104 to 280)	30.1 (18.4 to 44.2)	1 (0 to 1)	0.1 (0.1 to 0.2)	117 (62 to 182)	13 (7.1 to 20)	1 (0 to 1)	0.1 (0 to 0.2)	-35.5 (-61.4 to 2.9)	-57 (-72.6 to -35.6)	19.8 (-47.6 to 169.1)	-29.3 (-63.7 to 47.9)
		Male	178 (103 to 291)	26.6 (16.1 to 41.8)	1 (0 to 1)	0.2 (0.1 to 0.3)	129 (69 to 219)	13.1 (7.1 to 21.8)	1 (0 to 1)	0.1 (0 to 0.2)	-28 (-62.1 to 34.1)	-50.6 (-72.2 to -15.3)	28.8 (-50 to 202)	-25 (-63.7 to 57.9)
	Other infectious diseases	Both	19350 (10227 to 36198)	1298.8 (724.1 to 2344.3)	232 (124 to 429)	18.3 (11 to 31)	2506 (2028 to 3353)	140 (114.7 to 183.9)	40 (32 to 50)	2.8 (2.2 to 3.3)	-87 (-93.1 to -74.4)	-89.2 (-94 to -80.1)	-82.9 (-90.7 to -66.8)	-84.8 (-90.8 to -73.4)
		Female	10215 (5170 to 20089)	1379.3 (743.8 to 2622.2)	121 (61 to 235)	18.7 (10.7 to 33)	1121 (872 to 1607)	127.8 (100.8 to 176.8)	16 (12 to 22)	2.3 (1.8 to 3)	-89 (-94.3 to -77.6)	-90.7 (-95 to -81.8)	-86.7 (-93 to -72.5)	-87.5 (-92.8 to -77.5)
		Male	9135 (4840 to 16845)	1218.3 (689.4 to 2131.2)	111 (60 to 200)	17.7 (10.6 to 29)	1385 (1131 to 1785)	152.3 (125.1 to 194.5)	24 (19 to 29)	3.2 (2.6 to 3.9)	-84.8 (-91.8 to -70)	-87.5 (-92.9 to -76.6)	-78.8 (-88.2 to -59)	-81.8 (-89 to -68.2)
	Meningitis	Both	3324 (2212 to 4562)	237.9 (158.9 to 315.3)	41 (27 to 55)	3.6 (2.5 to 4.7)	780 (646 to 935)	43.3 (36.2 to 51.6)	13 (11 to 15)	0.9 (0.7 to 1.1)	-76.5 (-84.3 to -62.9)	-81.8 (-87.4 to -72.1)	-68.5 (-78.8 to -50.5)	-75.6 (-82.5 to -63.1)
		Female	1700 (1053 to 2503)	246.9 (152.3 to 348)	21 (12 to 30)	3.7 (2.4 to 5.1)	357 (288 to 434)	41 (33.5 to 49.9)	5 (4 to 7)	0.8 (0.7 to 1)	-79 (-86.6 to -63.8)	-83.4 (-88.9 to -72.9)	-73.7 (-82.8 to -54.8)	-78 (-84.9 to -64.4)
		Male	1624 (1078 to 2319)	229.1 (153.8 to 312.5)	20 (13 to 28)	3.6 (2.5 to 4.8)	423 (336 to 535)	45.6 (36.4 to 58)	7 (6 to 10)	1 (0.8 to 1.3)	-74 (-83.8 to -56.7)	-80.1 (-87.2 to -67.2)	-63.3 (-77.1 to -37.9)	-72.8 (-82.2 to -55)
	Encephalitis	Both	284 (178 to 413)	22.8 (15.3 to 30.7)	3 (2 to 5)	0.4 (0.2 to 0.5)	323 (233 to 402)	17.5 (12.4 to 21.8)	5 (3 to 6)	0.3 (0.2 to 0.4)	13.8 (-28.1 to 84.6)	-23.2 (-47.8 to 17.5)	39.2 (-10.1 to 132.3)	-13.9 (-45.6 to 33.9)
		Female	161 (84 to 265)	25.5 (15.5 to 38.1)	2 (1 to 3)	0.4 (0.2 to 0.5)	153 (104 to 205)	17 (11.3 to 22.7)	2 (1 to 3)	0.3 (0.2 to 0.4)	-5.3 (-49.7 to 73.2)	-33.3 (-60.5 to 12.4)	13.8 (-36.5 to 113.4)	-21.6 (-52.4 to 27.6)
		Male	123 (72 to 190)	20.3 (12.9 to 29)	2 (1 to 2)	0.4 (0.2 to 0.5)	171 (125 to 215)	18.1 (13.2 to 22.7)	3 (2 to 3)	0.3 (0.2 to 0.4)	38.9 (-17.5 to 157)	-10.8 (-43.7 to 52.6)	69.9 (1.5 to 219.4)	-4.4 (-45.9 to 67.9)
	Diphtheria	Both	95 (37 to 199)	6.2 (2.6 to 12.4)	1 (0 to 2)	0.1 (0 to 0.2)	2 (1 to 3)	0.1 (0 to 0.1)	0 (0 to 0)	0 (0 to 0)	-98.3 (-99.4 to -94.6)	-98.7 (-99.5 to -96)	-98 (-99.2 to -93.9)	-98.5 (-99.4 to -95.8)
		Female	44 (11 to 116)	5.8 (1.7 to 14.6)	1 (0 to 1)	0.1 (0 to 0.2)	1 (0 to 1)	0.1 (0 to 0.1)	0 (0 to 0)	0 (0 to 0)	-98.7 (-99.7 to -93.8)	-99 (-99.7 to -95.3)	-98.5 (-99.6 to -93.2)	-98.8 (-99.6 to -95.2)
		Male	51 (14 to 121)	6.6 (2.1 to 14.9)	1 (0 to 1)	0.1 (0 to 0.2)	1 (0 to 2)	0.1 (0 to 0.2)	0 (0 to 0)	0 (0 to 0)	-98 (-99.4 to -91.2)	-98.5 (-99.5 to -94)	-97.6 (-99.2 to -90.3)	-98.2 (-99.4 to -94.1)
	Whooping cough	Both	4640 (228 to 21470)	282.7 (13.9 to 1309.1)	53 (2 to 248)	3.2 (0.1 to 15.1)	228 (16 to 1030)	11.6 (0.8 to 52.8)	3 (0 to 12)	0.1 (0 to 0.6)	-95.1 (-99.8 to 9.8)	-95.9 (-99.8 to -8.7)	-95.2 (-99.9 to 32.1)	-96 (-99.9 to 10.7)
		Female	2649 (131 to 11756)	329.2 (16.3 to 1462.2)	30 (1 to 136)	3.8 (0.1 to 17)	126 (9 to 560)	13.2 (0.9 to 58.7)	1 (0 to 6)	0.1 (0 to 0.7)	-95.2 (-99.8 to 7)	-96 (-99.8 to -9.6)	-95.3 (-99.9 to 32.5)	-96.1 (-99.9 to 11.6)
		Male	1991 (100 to 9040)	238.1 (11.9 to 1083.7)	23 (1 to 104)	2.7 (0.1 to 12.5)	102 (7 to 463)	10.1 (0.7 to 46.3)	1 (0 to 5)	0.1 (0 to 0.5)	-94.9 (-99.8 to 11.5)	-95.8 (-99.8 to -7.7)	-95 (-99.9 to 29)	-95.9 (-99.9 to 7)
	Tetanus	Both	1090 (308 to 2991)	71.3 (23.8 to 186.3)	13 (4 to 34)	1 (0.4 to 2.3)	34 (21 to 56)	1.9 (1.1 to 3)	1 (0 to 1)	0 (0 to 0.1)	-96.9 (-99.2 to -85.4)	-97.4 (-99.2 to -89.3)	-95.6 (-98.8 to -82.1)	-96.2 (-98.9 to -88.6)
		Female	636 (131 to 2073)	83.7 (20.7 to 261.9)	8 (2 to 24)	1.2 (0.4 to 3.2)	17 (9 to 31)	1.9 (1 to 3.5)	0 (0 to 0)	0 (0 to 0.1)	-97.4 (-99.3 to -81.5)	-97.7 (-99.4 to -88)	-96.2 (-99 to -80.5)	-96.5 (-99.1 to -87.5)
		Male	455 (112 to 1463)	59.3 (18.8 to 181.1)	6 (2 to 17)	0.9 (0.3 to 2.3)	17 (10 to 29)	1.9 (1 to 3.1)	0 (0 to 0)	0 (0 to 0.1)	-96.2 (-99.1 to -80.4)	-96.9 (-99.2 to -85.5)	-94.7 (-98.7 to -77)	-95.6 (-98.8 to -84.7)

Province	Cause	Sex	1990				2019				1990-2019 percent change (%)			
			DALYs		Death		DALYs		Death		DALYs		Death	
			Number	Rate	Number	Rate	Number	Rate	Number	Rate	Number	Rate	Number	Rate
	Measles	Both	8228 (2549 to 18570)	503.2 (156.2 to 1133.3)	96 (30 to 216)	5.9 (1.8 to 13.2)	166 (50 to 386)	8.3 (2.5 to 19.4)	2 (1 to 4)	0.1 (0 to 0.2)	-98 (-99.2 to -95.7)	-98.3 (-99.3 to -96.5)	-98 (-99.2 to -95.8)	-98.4 (-99.3 to -96.6)
		Female	4207 (1307 to 9613)	525 (162.9 to 1200.7)	49 (15 to 112)	6.1 (1.9 to 14)	81 (24 to 194)	8.4 (2.5 to 20)	1 (0 to 2)	0.1 (0 to 0.2)	-98.1 (-99.2 to -95.6)	-98.4 (-99.3 to -96.4)	-98.1 (-99.2 to -95.6)	-98.4 (-99.3 to -96.4)
		Male	4021 (1255 to 9158)	482.2 (150.1 to 1098.7)	47 (15 to 106)	5.6 (1.7 to 12.8)	85 (25 to 202)	8.3 (2.4 to 19.8)	1 (0 to 2)	0.1 (0 to 0.2)	-97.9 (-99.2 to -95.5)	-98.3 (-99.3 to -96.3)	-97.9 (-99.2 to -95.5)	-98.3 (-99.3 to -96.4)
	Varicella and herpes zoster	Both	198 (101 to 333)	17.6 (11.1 to 25.7)	3 (2 to 4)	0.5 (0.3 to 0.7)	88 (61 to 124)	5.2 (3.7 to 7)	1 (1 to 1)	0.1 (0.1 to 0.1)	-55.8 (-77.4 to -12.4)	-70.6 (-82.5 to -49.7)	-61.8 (-79.4 to -24.8)	-81.5 (-88.8 to -69.6)
		Female	100 (43 to 202)	17.9 (9.8 to 30.6)	1 (1 to 3)	0.5 (0.3 to 0.7)	43 (28 to 66)	5.2 (3.5 to 7.7)	0 (0 to 1)	0.1 (0.1 to 0.1)	-56.9 (-81.9 to 7.8)	-70.9 (-85.3 to -43.6)	-64.8 (-84 to -15.6)	-81.4 (-90.7 to -61.9)
		Male	98 (47 to 179)	17.2 (10.1 to 27.9)	1 (1 to 2)	0.5 (0.3 to 0.8)	44 (29 to 68)	5.1 (3.4 to 7.7)	1 (0 to 1)	0.1 (0.1 to 0.1)	-54.6 (-79.6 to 3.4)	-70.3 (-84.4 to -45.2)	-58.6 (-81.3 to -7)	-81.7 (-90.8 to -62.7)
	Acute hepatitis	Both	865 (451 to 1397)	101.9 (55.2 to 166.7)	16 (8 to 26)	2.7 (1.4 to 4.6)	355 (291 to 430)	21.6 (17.8 to 26.2)	7 (6 to 9)	0.6 (0.4 to 0.7)	-59 (-76.1 to -16.7)	-78.8 (-88 to -57.4)	-52.6 (-74.4 to 0.3)	-78.9 (-89 to -54.6)
		Female	433 (170 to 711)	93 (35.8 to 138.2)	7 (2 to 11)	2.2 (0.8 to 3.4)	100 (78 to 125)	12 (9.5 to 14.9)	2 (1 to 2)	0.3 (0.2 to 0.3)	-76.9 (-86.8 to -38.1)	-87.1 (-91.7 to -65.9)	-76.3 (-85.8 to -27.6)	-88.2 (-93.4 to -62.7)
		Male	433 (206 to 837)	107.9 (53 to 213.4)	9 (4 to 18)	3 (1.4 to 6.2)	255 (200 to 319)	31.1 (24.4 to 39.3)	6 (4 to 7)	0.9 (0.7 to 1.1)	-41.1 (-72 to 28.2)	-71.2 (-86.9 to -37.3)	-33.7 (-71.6 to 54)	-71 (-87.5 to -35.2)
	Other unspecified infectious diseases	Both	625 (408 to 1030)	55.1 (37.5 to 84.3)	7 (4 to 11)	0.9 (0.6 to 1.5)	531 (388 to 671)	30.5 (22 to 38.4)	8 (5 to 10)	0.7 (0.4 to 0.8)	-15 (-56.8 to 38.5)	-44.7 (-69.1 to -13.5)	29 (-45.8 to 153.5)	-31.1 (-63.8 to 28.7)
		Female	284 (168 to 462)	52.3 (33.8 to 76)	3 (2 to 5)	0.9 (0.5 to 1.5)	243 (162 to 323)	28.9 (19.3 to 38.1)	4 (2 to 5)	0.6 (0.3 to 0.8)	-14.3 (-60.8 to 42.3)	-44.7 (-71.2 to -14)	33.1 (-48.9 to 168.9)	-29.3 (-63.7 to 41.3)
		Male	341 (198 to 618)	57.6 (35.9 to 97.5)	4 (2 to 7)	1 (0.6 to 1.8)	288 (207 to 387)	32.1 (22.8 to 42.8)	5 (3 to 6)	0.7 (0.4 to 0.9)	-15.6 (-55.1 to 57.2)	-44.3 (-68.4 to -5.8)	26 (-44.9 to 175)	-31.1 (-64.6 to 32.4)
	Maternal and neonatal disorders	Both	99551 (81449 to 120001)	6110.1 (5020.6 to 7333.1)	1118 (915 to 1349)	68.9 (56.7 to 82.8)	25654 (21119 to 30889)	1363.2 (1118.3 to 1648.9)	240 (191 to 295)	12.9 (10.3 to 15.9)	-74.2 (-80.5 to -66.5)	-77.7 (-83.2 to -71)	-78.6 (-84.4 to -71.3)	-81.3 (-86.3 to -75)
		Female	43132 (34995 to 53493)	5541.1 (4505.5 to 6832.5)	487 (396 to 605)	63.3 (51.2 to 78.3)	11460 (9356 to 13865)	1243.1 (1011.6 to 1507.4)	106 (84 to 131)	11.6 (9.1 to 14.4)	-73.4 (-80.3 to -65)	-77.6 (-83.3 to -70.4)	-78.2 (-84.2 to -70.2)	-81.6 (-86.7 to -74.7)
		Male	56419 (44990 to 68332)	6649.5 (5300.4 to 8051.3)	631 (504 to 766)	74.2 (59.2 to 90)	14195 (11558 to 17371)	1476.9 (1199.4 to 1809.8)	134 (105 to 168)	14.1 (11.1 to 17.7)	-74.8 (-81.5 to -66.8)	-77.8 (-83.7 to -70.7)	-78.8 (-84.8 to -71.2)	-81 (-86.4 to -74.2)
	Maternal disorders	Female	1517 (514 to 2224)	378.8 (137.4 to 553.3)	23 (6 to 35)	6 (1.7 to 9)	602 (460 to 760)	52.3 (40.3 to 66)	8 (6 to 10)	0.7 (0.5 to 0.9)	-60.3 (-74.4 to 16.4)	-86.2 (-91.2 to -61.8)	-65.8 (-79.1 to 22.6)	-88.8 (-93.2 to -63.7)
	Neonatal disorders	Both	98034 (80181 to 118740)	5924.5 (4847.8 to 7167.8)	1096 (895 to 1329)	66 (53.9 to 80)	25053 (20481 to 30306)	1337.8 (1091.5 to 1623.4)	232 (184 to 287)	12.6 (10 to 15.5)	-74.4 (-80.7 to -66.6)	-77.4 (-83 to -70.5)	-78.8 (-84.6 to -71.6)	-81 (-86.1 to -74.5)
		Female	41616 (33630 to 52072)	5162.3 (4179.2 to 6452.1)	464 (374 to 582)	57.3 (46.1 to 71.8)	10858 (8762 to 13193)	1190.8 (960.2 to 1449.3)	98 (76 to 123)	10.9 (8.5 to 13.7)	-73.9 (-81 to -65.3)	-76.9 (-83.2 to -69.3)	-78.8 (-85 to -70.8)	-80.9 (-86.5 to -73.6)
		Male	56419 (44990 to 68332)	6649.5 (5300.4 to 8051.3)	631 (504 to 766)	74.2 (59.2 to 90)	14195 (11558 to 17371)	1476.9 (1199.4 to 1809.8)	134 (105 to 168)	14.1 (11.1 to 17.7)	-74.8 (-81.5 to -66.8)	-77.8 (-83.7 to -70.7)	-78.8 (-84.8 to -71.2)	-81 (-86.4 to -74.2)
	Nutritional deficiencies	Both	8074 (5756 to 11308)	646.2 (468.4 to 882)	50 (31 to 78)	4.5 (3.1 to 6.5)	3142 (2013 to 4710)	172.3 (112 to 253.5)	6 (5 to 7)	0.5 (0.5 to 0.6)	-61.1 (-74.3 to -43.3)	-73.3 (-81.2 to -63.2)	-87.7 (-92.4 to -79.6)	-88 (-91.9 to -81.8)

Province	Cause	Sex	1990				2019				1990-2019 percent change (%)			
			DALYs		Death		DALYs		Death		DALYs		Death	
			Number	Rate	Number	Rate	Number	Rate	Number	Rate	Number	Rate	Number	Rate
		Female	4295 (2700 to 6404)	725.7 (475.6 to 1028.2)	27 (13 to 46)	4.7 (2.4 to 7.5)	1634 (986 to 2515)	189.9 (116.6 to 288.5)	3 (2 to 3)	0.6 (0.5 to 0.7)	-62 (-76.8 to -36.9)	-73.8 (-83.3 to -60.8)	-89.7 (-94.2 to -77.9)	-88.3 (-92.6 to -76.2)
		Male	3779 (2597 to 5581)	568.6 (403.8 to 812.5)	23 (15 to 38)	4.4 (3 to 6.7)	1509 (892 to 2478)	155.6 (94.1 to 254)	3 (3 to 4)	0.5 (0.5 to 0.7)	-60.1 (-76.2 to -35.4)	-72.6 (-82.8 to -58.2)	-85.4 (-91.7 to -75.3)	-87.5 (-92.2 to -81.1)
	Protein-energy malnutrition	Both	4357 (2771 to 6720)	293.9 (190.9 to 439.2)	49 (30 to 76)	4.3 (2.9 to 6.3)	708 (502 to 963)	38.8 (28.1 to 51.7)	5 (4 to 5)	0.4 (0.4 to 0.5)	-83.8 (-90.4 to -71.9)	-86.8 (-92 to -78)	-90.4 (-94.1 to -84.1)	-90.4 (-93.5 to -85.5)
		Female	2355 (1182 to 4085)	320.5 (165 to 538.9)	27 (13 to 46)	4.6 (2.3 to 7.3)	333 (235 to 460)	38 (27.2 to 51.4)	2 (2 to 3)	0.5 (0.4 to 0.6)	-85.9 (-92.4 to -70.7)	-88.1 (-93.4 to -76)	-91.5 (-95.3 to -81.4)	-90.1 (-93.7 to -79.9)
		Male	2002 (1308 to 3262)	268.3 (179.7 to 426)	22 (14 to 37)	4.1 (2.8 to 6.3)	374 (262 to 514)	39.6 (27.9 to 53.6)	2 (2 to 3)	0.4 (0.3 to 0.5)	-81.3 (-90 to -67)	-85.2 (-91.7 to -75.1)	-89.1 (-93.8 to -81.3)	-90.5 (-94.2 to -85.4)
	Iodine deficiency	Both	129 (79 to 193)	16 (10.2 to 23.4)	--	--	125 (71 to 203)	6.7 (3.9 to 10.6)	--	--	-2.7 (-31.5 to 26.1)	-58.3 (-69.8 to -47.9)	--	--
		Female	78 (47 to 119)	20 (12.7 to 29.4)	--	--	78 (44 to 128)	8.4 (4.9 to 13.7)	--	--	-0.9 (-33 to 36)	-57.9 (-70.3 to -44)	--	--
		Male	50 (30 to 76)	12.3 (7.6 to 18.1)	--	--	47 (27 to 77)	5 (2.9 to 8)	--	--	-5.5 (-33.3 to 26.7)	-59.3 (-70.3 to -48)	--	--
	Vitamin A deficiency	Both	237 (124 to 413)	14.7 (7.8 to 25.4)	--	--	17 (7 to 36)	0.9 (0.4 to 1.8)	--	--	-92.8 (-96.9 to -84.7)	-94.1 (-97.4 to -87.6)	--	--
		Female	87 (43 to 150)	11.1 (5.5 to 19.1)	--	--	8 (2 to 17)	0.8 (0.3 to 1.8)	--	--	-91.2 (-96.8 to -80.3)	-92.8 (-97.3 to -84.1)	--	--
		Male	150 (63 to 297)	18.1 (7.6 to 36.3)	--	--	9 (3 to 26)	0.9 (0.3 to 2.5)	--	--	-93.7 (-98.3 to -80.3)	-94.9 (-98.5 to -84.1)	--	--
	Dietary iron deficiency	Both	3210 (1901 to 4932)	307.1 (189.9 to 453.9)	--	--	2099 (1184 to 3484)	115.4 (65.3 to 188.8)	--	--	-34.6 (-57.5 to -4.4)	-62.4 (-74.5 to -46.7)	--	--
		Female	1721 (948 to 2672)	362.8 (216.1 to 539.8)	--	--	1129 (590 to 1923)	133 (71.7 to 221.4)	--	--	-34.4 (-60.1 to 8.3)	-63.3 (-75.9 to -45.6)	--	--
		Male	1489 (747 to 2611)	252.4 (137.7 to 417.6)	--	--	970 (440 to 1898)	98.5 (44.9 to 188.7)	--	--	-34.8 (-67.5 to 21.5)	-61 (-79.3 to -31)	--	--
	Other nutritional deficiencies	Both	141 (94 to 204)	14.6 (10.1 to 19.9)	1 (1 to 2)	0.2 (0.1 to 0.3)	194 (134 to 275)	10.6 (7.6 to 14.8)	1 (1 to 2)	0.1 (0.1 to 0.2)	37.2 (-7.4 to 98.8)	-26.9 (-47.1 to -0.3)	21.9 (-26.3 to 152.2)	-31.4 (-56.1 to 30.6)
		Female	53 (33 to 81)	11.4 (7.1 to 17.5)	0 (0 to 1)	0.1 (0 to 0.3)	87 (57 to 131)	9.7 (6.5 to 14.4)	1 (0 to 1)	0.1 (0.1 to 0.1)	63.8 (-2 to 149.3)	-15.3 (-46.8 to 26.6)	40.2 (-38.3 to 279.1)	-18.7 (-68.3 to 164.9)
		Male	88 (54 to 139)	17.5 (11.5 to 24.5)	1 (0 to 1)	0.3 (0.1 to 0.4)	107 (72 to 156)	11.6 (7.9 to 16.3)	1 (1 to 1)	0.2 (0.1 to 0.2)	21.3 (-31.4 to 98)	-34.1 (-56.8 to -2.2)	13.7 (-39.3 to 159.2)	-39.4 (-62.9 to 33.4)
	**Non-communicable diseases**	Both	179949 (141854 to 213051)	27800 (22660.9 to 31920.9)	3008 (2190 to 3526)	795.4 (642.6 to 898.4)	297963 (249311 to 351937)	19649.7 (16890.3 to 22733.4)	5149 (4664 to 5674)	496.2 (452.7 to 544.4)	65.6 (38 to 111.5)	-29.3 (-38.1 to -12.9)	71.2 (41.7 to 136.3)	-37.6 (-46.8 to -21.3)
		Female	83796 (65607 to 101480)	26366 (21723.4 to 30773.1)	1244 (924 to 1484)	685.2 (572.1 to 789.4)	146431 (120635 to 176722)	19451 (16406.2 to 22911)	2240 (1994 to 2455)	469.1 (422.1 to 507.8)	74.7 (45.6 to 116.7)	-26.2 (-35.7 to -10.3)	80.1 (45.3 to 146.7)	-31.5 (-42.5 to -16.2)
		Male	96153 (74045 to 116387)	28997.6 (22971.2 to 33908.6)	1764 (1231 to 2174)	901.5 (705.8 to 1073.7)	151531 (127807 to 178019)	19989.5 (16994.2 to 23245.4)	2909 (2490 to 3393)	532.6 (455.6 to 620.4)	57.6 (25.8 to 109.6)	-31.1 (-43.2 to -11.7)	64.9 (25.4 to 140.6)	-40.9 (-53.7 to -19.5)
	Neoplasms	Both	10147 (5916 to 12838)	1843.8 (1209.2 to 2270.6)	287 (191 to 355)	70.5 (51.8 to 85.2)	22897 (20117 to 25966)	1650 (1453.5 to 1872.5)	755 (659 to 859)	65.7 (57.6 to 74.7)	125.7 (72.9 to 289.4)	-10.5 (-29.1 to 39.5)	162.7 (107.2 to 306.5)	-6.8 (-25.1 to 30.1)

Province	Cause	Sex	1990				2019				1990-2019 percent change (%)			
			DALYs		Death		DALYs		Death		DALYs		Death	
			Number	Rate	Number	Rate	Number	Rate	Number	Rate	Number	Rate	Number	Rate
		Female	4392 (2368 to 5825)	1604.8 (965 to 2071.4)	114 (71 to 146)	56.6 (40.9 to 71.5)	10383 (8819 to 12087)	1460.9 (1251.4 to 1692.3)	328 (279 to 380)	56.1 (48 to 64.3)	136.4 (68.6 to 344.4)	-9 (-32.7 to 50)	188.4 (113.9 to 356.1)	-1 (-24.4 to 37.5)
		Male	5754 (3441 to 7782)	2060.5 (1366.7 to 2686.9)	173 (114 to 226)	84.8 (59.6 to 108.7)	12515 (10481 to 14916)	1853.1 (1541.3 to 2211.5)	426 (351 to 511)	75.8 (62.3 to 91.1)	117.5 (53.6 to 270.1)	-10.1 (-35 to 43.3)	145.9 (76.8 to 295)	-10.7 (-33.8 to 36.8)
	Lip and oral cavity cancer	Both	68 (43 to 89)	15.5 (10.3 to 20.2)	2 (2 to 3)	0.7 (0.5 to 0.9)	200 (169 to 237)	15 (12.7 to 17.9)	7 (6 to 9)	0.6 (0.6 to 0.8)	194.7 (106.4 to 401.8)	-2.8 (-31.3 to 53.1)	200.3 (112.1 to 373)	-1.8 (-29.3 to 50.1)
		Female	21 (12 to 27)	10.1 (6.8 to 13)	1 (0 to 1)	0.4 (0.3 to 0.5)	81 (66 to 99)	12.5 (10.2 to 15.3)	3 (2 to 4)	0.6 (0.5 to 0.7)	293.6 (182.1 to 557.8)	24 (-9.2 to 91.2)	319.8 (208.6 to 535)	30.9 (-3.3 to 90.9)
		Male	47 (28 to 67)	20.2 (12.7 to 28)	2 (1 to 2)	0.9 (0.6 to 1.2)	119 (92 to 152)	17.7 (13.7 to 22.7)	4 (3 to 5)	0.7 (0.6 to 1)	151.5 (54.4 to 361.9)	-12.3 (-45.4 to 56)	149.9 (54.3 to 341.6)	-15.7 (-46.5 to 45.1)
	Nasopharynx cancer	Both	35 (19 to 45)	6.7 (4.1 to 8.5)	1 (1 to 1)	0.2 (0.2 to 0.3)	65 (55 to 78)	4.3 (3.6 to 5.1)	2 (2 to 2)	0.1 (0.1 to 0.2)	84.8 (34.8 to 242.3)	-36.3 (-52.8 to 8.1)	83.8 (35.6 to 203.7)	-38 (-53.5 to -2.9)
		Female	12 (6 to 17)	4.7 (2.7 to 6.5)	0 (0 to 0)	0.2 (0.1 to 0.2)	19 (15 to 24)	2.6 (2.1 to 3.2)	1 (0 to 1)	0.1 (0.1 to 0.1)	57.6 (4.3 to 220.2)	-44.5 (-62.3 to 2.5)	68.2 (13.5 to 201.3)	-43.5 (-63.1 to -6.2)
		Male	23 (13 to 32)	8.3 (5 to 11.4)	1 (0 to 1)	0.3 (0.2 to 0.4)	46 (36 to 57)	5.9 (4.7 to 7.4)	1 (1 to 2)	0.2 (0.2 to 0.2)	98.9 (31.3 to 250.4)	-29.2 (-52.4 to 19.5)	91.5 (28 to 224.5)	-32.1 (-53.7 to 9.9)
	Other pharynx cancer	Both	27 (17 to 35)	6.2 (4.2 to 7.9)	1 (1 to 1)	0.3 (0.2 to 0.3)	65 (55 to 78)	4.9 (4.1 to 5.9)	2 (2 to 3)	0.2 (0.2 to 0.2)	141.6 (73.5 to 302.3)	-20 (-41.6 to 24.4)	145.9 (80.9 to 281.5)	-18.2 (-38.7 to 19.5)
		Female	11 (6 to 15)	5.1 (3.3 to 7)	0 (0 to 0)	0.2 (0.1 to 0.3)	23 (18 to 29)	3.5 (2.8 to 4.3)	1 (1 to 1)	0.2 (0.1 to 0.2)	117.4 (38 to 294.5)	-31.7 (-55.1 to 13.7)	133.1 (54.3 to 280.9)	-26 (-50.3 to 16.2)
		Male	16 (10 to 23)	7 (4.5 to 9.7)	1 (0 to 1)	0.3 (0.2 to 0.4)	42 (33 to 54)	6.4 (5 to 8.2)	2 (1 to 2)	0.3 (0.2 to 0.3)	157.4 (64 to 339.6)	-8.2 (-41.1 to 51.3)	153.7 (64.9 to 318.6)	-9.7 (-40.3 to 43.5)
	Esophageal cancer	Both	187 (106 to 492)	45 (27.1 to 120.3)	7 (4 to 19)	2 (1.3 to 5.2)	387 (268 to 1079)	31.6 (22 to 87.6)	15 (11 to 43)	1.4 (1 to 3.9)	107.5 (47.4 to 213.6)	-29.7 (-49.4 to 2.4)	118.1 (59 to 218.1)	-27.4 (-46.7 to 0.5)
		Female	81 (46 to 201)	42.7 (25.7 to 105.1)	3 (2 to 8)	1.9 (1.2 to 4.8)	153 (106 to 412)	25.4 (17.6 to 67.7)	6 (4 to 17)	1.2 (0.8 to 3.2)	89 (26.4 to 214.7)	-40.6 (-60 to -6.8)	102.7 (37.9 to 208.1)	-36.1 (-56.6 to -5.7)
		Male	106 (55 to 307)	46.7 (24.6 to 137)	4 (2 to 12)	2.1 (1.1 to 6)	234 (146 to 714)	38.2 (23.9 to 115.1)	9 (6 to 28)	1.7 (1.1 to 5.1)	121.6 (40.7 to 289.7)	-18.2 (-47 to 34.6)	130.1 (50.2 to 283.7)	-17.7 (-44.7 to 29.4)
	Stomach cancer	Both	1234 (763 to 1560)	287.3 (190.8 to 359.4)	45 (30 to 56)	12.4 (9.2 to 15.4)	2057 (1758 to 2428)	161.4 (138.2 to 189.3)	79 (68 to 92)	7.3 (6.3 to 8.5)	66.6 (27.5 to 171.3)	-43.8 (-56.6 to -15.1)	76.7 (36.8 to 164.6)	-41.3 (-54 to -17.8)
		Female	460 (277 to 618)	224.7 (143.9 to 294.7)	16 (11 to 21)	9.5 (6.9 to 12.5)	773 (628 to 942)	119.9 (98.9 to 143.4)	30 (25 to 35)	5.6 (4.7 to 6.6)	68.2 (15.5 to 194)	-46.7 (-62.4 to -15.2)	85.4 (32.2 to 182.7)	-41.3 (-58.3 to -16.5)
		Male	775 (483 to 1059)	340.9 (223.5 to 456.8)	29 (19 to 39)	15.2 (10.4 to 19.7)	1284 (1028 to 1583)	205.3 (164.9 to 253.1)	49 (40 to 61)	9.1 (7.4 to 11.2)	65.7 (14.9 to 168)	-39.8 (-58 to -5.2)	71.8 (20.7 to 171)	-39.9 (-56.8 to -6.9)
	Colon and rectum cancer	Both	521 (337 to 681)	124.5 (86 to 160.8)	19 (13 to 25)	5.6 (4.1 to 7)	1869 (1582 to 2241)	148.8 (125 to 179.3)	74 (62 to 89)	6.9 (5.7 to 8.4)	258.8 (149.8 to 475.2)	19.5 (-16.7 to 82.3)	280.7 (167.8 to 477.8)	23.9 (-11.8 to 79.9)
		Female	222 (136 to 309)	114.5 (77.3 to 156.5)	8 (6 to 11)	5.1 (3.6 to 6.8)	823 (641 to 1015)	133.1 (104.7 to 164.3)	33 (26 to 41)	6.4 (5.1 to 8)	271.2 (132.9 to 538.8)	16.2 (-25.7 to 86.5)	304.1 (161 to 532.3)	26 (-16.6 to 88.4)

Province	Cause	Sex	1990				2019				1990-2019 percent change (%)			
			DALYs		Death		DALYs		Death		DALYs		Death	
			Number	Rate	Number	Rate	Number	Rate	Number	Rate	Number	Rate	Number	Rate
		Male	299 (185 to 424)	132.6 (87.2 to 185.2)	11 (7 to 16)	6 (4 to 8.3)	1046 (827 to 1316)	165.9 (131.1 to 211)	40 (32 to 51)	7.5 (5.9 to 9.6)	249.6 (117.9 to 497.5)	25.1 (-21 to 109.2)	263.5 (127.6 to 518)	24.8 (-19.9 to 101.2)
	Liver cancer	Both	279 (177 to 366)	63.6 (43.3 to 82.4)	10 (7 to 13)	2.9 (2 to 3.7)	689 (566 to 820)	54.9 (45.2 to 65.6)	26 (22 to 31)	2.4 (2 to 2.9)	146.6 (73.9 to 290.4)	-13.8 (-38.8 to 31)	153.4 (79.4 to 287.7)	-14.5 (-39.5 to 27.2)
		Female	91 (57 to 132)	43.5 (28.9 to 66.5)	3 (2 to 5)	2 (1.3 to 3)	211 (166 to 267)	33.1 (26.3 to 42)	8 (6 to 10)	1.5 (1.2 to 1.9)	132.3 (42.6 to 297.6)	-23.9 (-55 to 28.3)	144.3 (40.8 to 308.3)	-24 (-57.7 to 28.2)
		Male	188 (115 to 267)	80.9 (52.7 to 114.2)	7 (5 to 10)	3.7 (2.5 to 5.2)	478 (371 to 600)	77.7 (59.6 to 98.1)	18 (14 to 23)	3.4 (2.6 to 4.4)	153.5 (65.1 to 324.4)	-4 (-37.4 to 56.7)	157.6 (67.9 to 328)	-6.7 (-38.9 to 47)
	Gallbladder and biliary tract cancer	Both	91 (59 to 146)	22.3 (15.4 to 36.8)	4 (2 to 6)	1 (0.7 to 1.7)	199 (164 to 298)	16.1 (13.2 to 25)	8 (7 to 13)	0.7 (0.6 to 1.2)	119.4 (65.6 to 252.9)	-27.9 (-44.7 to 10.3)	125 (73.9 to 236.8)	-27.1 (-42.1 to 3.2)
		Female	43 (28 to 79)	23.4 (15.2 to 43)	2 (1 to 3)	1.1 (0.7 to 2)	96 (73 to 165)	16 (12.2 to 28.5)	4 (3 to 7)	0.8 (0.6 to 1.4)	121.1 (56.2 to 257.6)	-31.7 (-50.6 to 3.8)	131.6 (68.8 to 245.8)	-27.7 (-46.8 to 6.3)
		Male	47 (29 to 73)	21.4 (13.9 to 33.2)	2 (1 to 3)	1 (0.7 to 1.5)	103 (79 to 152)	16.2 (12.1 to 25.1)	4 (3 to 6)	0.7 (0.5 to 1.2)	117.9 (43.5 to 279.5)	-23.9 (-48.7 to 26.5)	118.8 (46.2 to 275.6)	-25.9 (-49.3 to 15.8)
	Pancreatic cancer	Both	173 (113 to 239)	41.9 (27.8 to 57)	7 (4 to 9)	1.9 (1.3 to 2.5)	980 (831 to 1143)	82.2 (69.8 to 95.1)	40 (34 to 47)	3.8 (3.2 to 4.4)	465.6 (290.8 to 813.2)	96 (37.2 to 200.1)	498.2 (317.8 to 819.2)	105 (44.7 to 207.4)
		Female	59 (38 to 85)	31.3 (21 to 45.9)	2 (2 to 3)	1.4 (1 to 2)	433 (354 to 528)	73.1 (60.1 to 88.7)	18 (15 to 22)	3.5 (2.9 to 4.2)	639.7 (367.2 to 1079.8)	133.3 (47.6 to 265.1)	685.6 (395 to 1116)	149.4 (53.8 to 280.9)
		Male	115 (67 to 174)	51.3 (30.9 to 76.8)	4 (3 to 7)	2.3 (1.4 to 3.4)	547 (433 to 686)	91.8 (73 to 115)	22 (18 to 28)	4.2 (3.3 to 5.3)	376.7 (175.2 to 770.1)	79 (4.7 to 214.5)	400.9 (191.8 to 796.5)	81.4 (7.5 to 211)
	Larynx cancer	Both	276 (179 to 354)	63 (42.2 to 80.3)	10 (6 to 12)	2.5 (1.7 to 3.2)	498 (420 to 598)	38.6 (32.7 to 46.3)	18 (15 to 22)	1.6 (1.4 to 1.9)	80.5 (34.4 to 205.2)	-38.7 (-54.1 to -0.5)	86.6 (38.4 to 199.8)	-35.9 (-51.6 to -2.5)
		Female	84 (48 to 113)	40.3 (25.4 to 53.8)	3 (2 to 4)	1.6 (1.1 to 2)	155 (122 to 190)	23.9 (19.2 to 29.6)	6 (5 to 7)	1 (0.8 to 1.3)	84.8 (27.1 to 225.9)	-40.8 (-58.2 to -2.4)	103.1 (44 to 230.1)	-33.9 (-52.5 to 3.6)
		Male	192 (122 to 263)	82.3 (52.7 to 112.2)	7 (4 to 9)	3.4 (2.2 to 4.6)	343 (271 to 438)	53.7 (42.5 to 68.2)	12 (10 to 16)	2.2 (1.7 to 2.8)	78.7 (26.7 to 223.3)	-34.7 (-53.7 to 16)	80 (27.4 to 221.2)	-34.8 (-53.5 to 7.1)
	Tracheal, bronchus, and lung cancer	Both	929 (580 to 1265)	212.8 (140.6 to 286.9)	34 (23 to 46)	8.9 (6.3 to 11.9)	2959 (2441 to 3643)	230.2 (190.2 to 281.4)	109 (91 to 133)	9.8 (8.1 to 12)	218.5 (109.2 to 433.4)	8.2 (-28.3 to 74.9)	224 (116.6 to 415.6)	10.7 (-24.8 to 69.2)
		Female	172 (101 to 258)	82.6 (54.7 to 124.5)	6 (4 to 9)	3.3 (2.4 to 4.8)	868 (687 to 1052)	132.7 (106.1 to 161.8)	32 (26 to 39)	5.8 (4.6 to 7)	403.7 (188.2 to 771.2)	60.8 (-9.1 to 154.7)	450.6 (209.8 to 751.4)	74.3 (2 to 158.1)
		Male	757 (468 to 1077)	325.8 (211.8 to 458.6)	28 (18 to 39)	14.1 (9.6 to 19.3)	2091 (1627 to 2694)	331.2 (257.1 to 430.2)	77 (60 to 100)	14.1 (10.9 to 18.2)	176.4 (69.4 to 385.7)	1.7 (-37.4 to 73.3)	177 (70.4 to 372.7)	-0.2 (-37.1 to 63.7)
	Malignant skin melanoma	Both	58 (32 to 108)	12.1 (7.5 to 19.7)	2 (1 to 3)	0.5 (0.4 to 0.7)	113 (69 to 215)	7.7 (4.8 to 14.4)	4 (2 to 7)	0.3 (0.2 to 0.6)	93.9 (7.8 to 254.4)	-36.5 (-63.3 to 7.7)	96.8 (14.9 to 229.2)	-39.9 (-63.8 to -2.4)
		Female	22 (11 to 44)	9.3 (5.5 to 16.8)	1 (0 to 1)	0.4 (0.3 to 0.7)	44 (25 to 92)	6.1 (3.6 to 12.6)	1 (1 to 3)	0.2 (0.2 to 0.5)	99.7 (0.2 to 302.3)	-34.8 (-64.9 to 13)	111.1 (16 to 254.9)	-35 (-61.2 to -2.3)
		Male	36 (19 to 68)	14.7 (8.7 to 24.1)	1 (1 to 2)	0.6 (0.4 to 0.9)	69 (41 to 139)	9.4 (5.7 to 18.4)	2 (1 to 4)	0.4 (0.2 to 0.7)	90.4 (0.1 to 272.6)	-36.2 (-65.9 to 21)	88.7 (1.6 to 255.4)	-42.7 (-69.1 to 9.8)

Province	Cause	Sex	1990				2019				1990-2019 percent change (%)			
			DALYs		Death		DALYs		Death		DALYs		Death	
			Number	Rate	Number	Rate	Number	Rate	Number	Rate	Number	Rate	Number	Rate
	Non-melanoma skin cancer	Both	24 (16 to 32)	6.3 (4.4 to 8.2)	1 (1 to 1)	0.3 (0.2 to 0.4)	82 (68 to 97)	6.6 (5.5 to 7.9)	4 (3 to 4)	0.4 (0.3 to 0.4)	234.4 (136.2 to 411.9)	6.1 (-24.3 to 58.5)	255.8 (152.6 to 438.7)	9.6 (-20.1 to 63.7)
		Female	3 (2 to 5)	1.7 (1.1 to 2.4)	0 (0 to 0)	0.1 (0.1 to 0.1)	11 (9 to 14)	1.9 (1.6 to 2.3)	1 (0 to 1)	0.1 (0.1 to 0.1)	260.4 (131.1 to 502.5)	13.2 (-26.2 to 89.6)	310.9 (168 to 593.5)	28.1 (-17.8 to 116.9)
		Male	21 (14 to 29)	10.6 (7.3 to 14)	1 (1 to 1)	0.6 (0.4 to 0.8)	70 (57 to 85)	11.4 (9.2 to 14)	3 (3 to 4)	0.6 (0.5 to 0.7)	230.5 (121.3 to 431.2)	7.9 (-25.8 to 65.7)	247.7 (139.3 to 442)	2.3 (-28.3 to 55.5)
	Breast cancer	Both	585 (341 to 831)	126 (78.3 to 179.7)	17 (11 to 24)	4.2 (2.7 to 6)	2419 (1958 to 2893)	155.8 (128 to 185.3)	68 (55 to 81)	5.1 (4.2 to 6.1)	313.5 (172 to 653)	23.7 (-17.7 to 110.7)	298.5 (163 to 558.7)	22.7 (-20.6 to 93.8)
		Female	566 (325 to 815)	260.8 (160.9 to 376.2)	16 (10 to 24)	8.4 (5.4 to 12.4)	2379 (1926 to 2848)	309 (253 to 368.1)	67 (54 to 80)	10.1 (8.3 to 12.1)	320.7 (172.6 to 670)	18.5 (-21.9 to 104.9)	306.2 (163.4 to 583.1)	19.3 (-23.8 to 90.3)
		Male	20 (11 to 30)	7.9 (4.6 to 11.8)	1 (0 to 1)	0.3 (0.2 to 0.4)	40 (28 to 54)	5.7 (4 to 7.8)	1 (1 to 2)	0.2 (0.1 to 0.3)	104 (14.1 to 306.8)	-28.7 (-59.2 to 38.2)	98.6 (11.9 to 291.9)	-30.7 (-60.9 to 31.8)
	Cervical cancer	Female	244 (121 to 341)	108.4 (57.3 to 149.1)	7 (4 to 10)	3.6 (2.1 to 5)	439 (282 to 560)	59.8 (38.7 to 74.8)	14 (9 to 17)	2.2 (1.5 to 2.7)	79.9 (23.5 to 245.4)	-44.9 (-60.9 to 0)	92.1 (36.5 to 239.2)	-39.4 (-56.3 to -0.7)
	Uterine cancer	Female	34 (20 to 49)	16.9 (10.6 to 24.6)	1 (1 to 2)	0.6 (0.4 to 1)	147 (74 to 195)	21.9 (12 to 28.6)	5 (3 to 6)	0.8 (0.5 to 1.1)	328.5 (108.9 to 696.1)	29.8 (-34.6 to 129.8)	319.8 (112.1 to 638.2)	30.2 (-32.1 to 120.2)
	Ovarian cancer	Female	79 (38 to 160)	36.2 (19 to 70.9)	2 (1 to 5)	1.2 (0.7 to 2.4)	506 (350 to 638)	72.6 (49.4 to 91.9)	16 (11 to 20)	2.6 (1.8 to 3.3)	540.6 (145.7 to 1265.8)	100.5 (-20.9 to 305.5)	569.9 (166.1 to 1263.8)	111.1 (-14 to 323.9)
	Prostate cancer	Male	275 (175 to 435)	161.8 (103.5 to 252.9)	13 (9 to 22)	10.1 (6.5 to 16)	798 (619 to 1107)	161.7 (123.8 to 231)	44 (33 to 63)	9.5 (7.1 to 13.9)	190.1 (109.8 to 358.1)	-0.1 (-26.7 to 53.2)	229.8 (135.2 to 415.8)	-6 (-31.1 to 40.8)
	Testicular cancer	Male	6 (3 to 8)	1.2 (0.6 to 1.6)	0 (0 to 0)	0 (0 to 0)	52 (37 to 71)	5.1 (3.7 to 6.9)	1 (1 to 1)	0.1 (0.1 to 0.1)	805 (422 to 1824)	334 (167.2 to 821.3)	822 (465.4 to 1817.7)	271.5 (132.5 to 579)
	Kidney cancer	Both	132 (77 to 191)	19.5 (12.2 to 26.9)	3 (2 to 4)	0.7 (0.4 to 0.9)	377 (313 to 447)	26.7 (22.1 to 31.8)	12 (10 to 14)	1 (0.8 to 1.2)	185 (80 to 404)	36.7 (-8.4 to 131.7)	272.3 (147.6 to 535.7)	46.9 (-0.3 to 167.7)
		Female	44 (24 to 68)	12.9 (8.2 to 18)	1 (1 to 1)	0.4 (0.3 to 0.6)	109 (87 to 132)	14.9 (12.1 to 17.8)	3 (3 to 4)	0.5 (0.4 to 0.6)	145 (41.2 to 355.9)	15.2 (-24.8 to 89.2)	216.2 (111.4 to 417)	19.6 (-18.6 to 111.9)
		Male	88 (49 to 137)	25.3 (14.6 to 37.7)	2 (1 to 3)	0.9 (0.5 to 1.3)	268 (207 to 336)	38.7 (30.1 to 49.1)	9 (7 to 11)	1.5 (1.1 to 1.9)	205.1 (76.6 to 498.6)	52.8 (-7.1 to 198.4)	297.5 (138.1 to 678.4)	63.7 (0.5 to 227.6)
	Bladder cancer	Both	143 (92 to 190)	36.6 (24.1 to 48.1)	6 (4 to 8)	1.8 (1.2 to 2.4)	377 (312 to 453)	32.8 (27.2 to 39.1)	16 (14 to 19)	1.6 (1.3 to 1.9)	164.3 (83.2 to 341.2)	-10.5 (-37.3 to 45.1)	175.7 (91.6 to 347.7)	-10.9 (-36.3 to 42.6)
		Female	32 (20 to 46)	17.8 (11.6 to 25)	1 (1 to 2)	0.9 (0.6 to 1.3)	89 (72 to 110)	16.1 (13 to 19.8)	4 (3 to 5)	0.9 (0.7 to 1.1)	182.6 (77.1 to 381.3)	-9.4 (-41.4 to 51.7)	213.7 (102.3 to 424.6)	-1.9 (-36.3 to 62.9)
		Male	111 (67 to 156)	53.6 (33.4 to 73.4)	5 (3 to 6)	2.7 (1.8 to 3.7)	288 (227 to 357)	50 (39.6 to 62.4)	12 (9 to 15)	2.3 (1.9 to 2.9)	159 (65.7 to 368.5)	-6.8 (-39.2 to 61.7)	164.5 (70 to 368.6)	-14 (-42.4 to 46.2)
		Both	973 (489 to 1718)	120.5 (66.1 to 192.9)	19 (11 to 31)	3.3 (2.1 to 4.8)	1922 (1411 to 2341)	115.5 (84.2 to 140.5)	46 (33 to 56)	3.3 (2.3 to 4.1)	97.5 (-10.6 to 296.4)	-4.1 (-51.3 to 76.9)	145.3 (17 to 351.2)	2.7 (-46.7 to 64.9)

Province	Cause	Sex	1990				2019				1990-2019 percent change (%)			
			DALYs		Death		DALYs		Death		DALYs		Death	
			Number	Rate	Number	Rate	Number	Rate	Number	Rate	Number	Rate	Number	Rate
	Brain and central nervous system cancer	Female	420 (170 to 868)	106.3 (49.8 to 197)	8 (4 to 15)	2.8 (1.6 to 4.8)	902 (584 to 1197)	111 (71.6 to 146.3)	22 (14 to 29)	3.3 (2.1 to 4.3)	114.9 (-26.9 to 450.7)	4.5 (-59.8 to 131.7)	182.2 (5.4 to 520.1)	16.6 (-49.5 to 127.7)
		Male	553 (267 to 1157)	132.8 (70 to 243)	11 (6 to 20)	3.6 (2.2 to 5.9)	1020 (685 to 1351)	119.8 (79.1 to 159.9)	24 (16 to 32)	3.4 (2.1 to 4.6)	84.3 (-9.8 to 304)	-9.7 (-53.8 to 79.3)	119 (8.3 to 329.5)	-6.4 (-51.3 to 71.9)
	Thyroid cancer	Both	36 (22 to 57)	7.9 (5.3 to 12.6)	1 (1 to 2)	0.3 (0.2 to 0.5)	142 (112 to 172)	10 (8.2 to 12.3)	4 (3 to 5)	0.4 (0.3 to 0.4)	291.9 (128.3 to 559.2)	26.5 (-22.9 to 96.8)	252.6 (125.9 to 444)	12.5 (-26.8 to 67)
		Female	19 (10 to 35)	8.9 (5.5 to 17.8)	1 (0 to 1)	0.4 (0.2 to 0.7)	60 (47 to 83)	8.9 (7 to 12.5)	2 (1 to 3)	0.3 (0.3 to 0.5)	225.7 (90.4 to 486)	-0.3 (-37.6 to 65.5)	195.4 (88.3 to 375.3)	-9.1 (-39.9 to 41)
		Male	18 (11 to 25)	7 (4.5 to 10)	1 (0 to 1)	0.3 (0.2 to 0.4)	82 (55 to 102)	11.2 (7.9 to 14.2)	2 (2 to 3)	0.4 (0.3 to 0.5)	361.2 (151.9 to 690.7)	59.9 (-9.7 to 172.4)	315.3 (136.4 to 612.7)	44.1 (-17.3 to 139.6)
	Mesothelioma	Both	5 (3 to 9)	1.2 (0.7 to 1.9)	0 (0 to 0)	0 (0 to 0.1)	20 (14 to 24)	1.3 (1 to 1.6)	1 (0 to 1)	0 (0 to 0.1)	263.1 (123.9 to 562.7)	14.9 (-29.7 to 95.8)	266.8 (124.1 to 507.3)	17.8 (-28.2 to 87.2)
		Female	3 (1 to 4)	1.2 (0.7 to 1.9)	0 (0 to 0)	0 (0 to 0.1)	6 (5 to 9)	0.9 (0.6 to 1.2)	0 (0 to 0)	0 (0 to 0)	146 (31.7 to 408.3)	-27.5 (-58.8 to 38.8)	147.2 (47 to 358.5)	-26.4 (-53 to 25)
		Male	3 (1 to 6)	1.1 (0.5 to 2.7)	0 (0 to 0)	0 (0 to 0.1)	13 (9 to 17)	1.8 (1.3 to 2.4)	0 (0 to 1)	0.1 (0 to 0.1)	374.2 (135 to 798.6)	59.9 (-22.1 to 208)	370.3 (121.9 to 793.7)	59.3 (-25.1 to 209.9)
	Hodgkin lymphoma	Both	86 (26 to 206)	13 (4.4 to 30.9)	2 (1 to 5)	0.4 (0.1 to 0.9)	153 (93 to 289)	8.4 (5.2 to 15.9)	3 (2 to 6)	0.2 (0.1 to 0.4)	76.6 (20.8 to 366.3)	-35 (-56 to 48.6)	69.2 (15.9 to 279.7)	-42.7 (-60.9 to 10.7)
		Female	28 (6 to 79)	9.1 (2.3 to 25.5)	1 (0 to 2)	0.3 (0.1 to 0.8)	45 (22 to 101)	5.2 (2.5 to 11.5)	1 (0 to 2)	0.1 (0.1 to 0.3)	60.2 (7 to 376.3)	-42.9 (-60.6 to 32.7)	57.7 (10.3 to 254.5)	-49.3 (-63.5 to -5.5)
		Male	58 (19 to 149)	16.4 (5.9 to 42.1)	1 (0 to 3)	0.5 (0.2 to 1.2)	107 (68 to 215)	11.6 (7.3 to 22.9)	2 (1 to 5)	0.3 (0.2 to 0.6)	84.6 (19.1 to 356.1)	-29.4 (-55.6 to 57.4)	74.9 (11.3 to 278.8)	-37.4 (-60.7 to 21.8)
	Non-Hodgkin lymphoma	Both	255 (139 to 336)	42.7 (26 to 54.8)	7 (4 to 8)	1.5 (1 to 1.8)	726 (608 to 857)	46.6 (39.4 to 54.7)	20 (17 to 23)	1.5 (1.3 to 1.8)	184.7 (96.8 to 457.8)	9.1 (-21.7 to 87.1)	194.7 (112.3 to 403.4)	4.6 (-21.6 to 60)
		Female	87 (41 to 129)	29.6 (17.1 to 41.8)	2 (1 to 3)	1 (0.6 to 1.3)	248 (196 to 306)	32.9 (26 to 40.6)	7 (6 to 9)	1.1 (0.9 to 1.4)	186.3 (66.8 to 527.1)	11.1 (-30.9 to 106)	225.3 (108.9 to 496.4)	13.3 (-22.6 to 105.2)
		Male	168 (93 to 225)	54.2 (32.1 to 71.5)	4 (3 to 6)	1.9 (1.3 to 2.4)	478 (388 to 580)	60.2 (49 to 72.9)	13 (10 to 15)	1.9 (1.6 to 2.3)	183.9 (94.2 to 441.4)	11 (-23 to 88.3)	179.9 (94.9 to 369)	2.5 (-27 to 60.4)
	Multiple myeloma	Both	80 (50 to 134)	18.9 (11.8 to 31.3)	3 (2 to 5)	0.8 (0.5 to 1.3)	213 (159 to 318)	16.9 (12.5 to 26.1)	8 (6 to 12)	0.7 (0.5 to 1.1)	165.1 (66.7 to 326.8)	-10.7 (-42.5 to 38.9)	157.6 (68 to 295.9)	-11.9 (-41 to 28.6)
		Female	33 (20 to 50)	17.1 (11.1 to 26.7)	1 (1 to 2)	0.7 (0.5 to 1.2)	87 (65 to 124)	13.9 (10.4 to 20.2)	3 (2 to 5)	0.6 (0.4 to 0.9)	168.2 (69.9 to 355.7)	-18.4 (-46.4 to 29.4)	158.1 (75.2 to 302.5)	-19.7 (-43.3 to 22.8)
		Male	48 (27 to 92)	20.5 (11.6 to 39.3)	2 (1 to 3)	0.9 (0.5 to 1.6)	126 (85 to 207)	19.9 (13.1 to 34.4)	5 (3 to 8)	0.8 (0.5 to 1.5)	163 (50.8 to 363.2)	-3 (-44.4 to 62.7)	157.3 (49.1 to 332)	-4.5 (-43.9 to 53.8)
	Leukemia	Both	2412 (1217 to 3534)	266.5 (152 to 354.9)	43 (25 to 57)	6.9 (4.5 to 8.8)	2788 (2194 to 3428)	166.8 (134.2 to 202.5)	65 (53 to 78)	4.8 (3.9 to 5.8)	15.6 (-24.1 to 142.3)	-37.4 (-56.2 to 16)	52.9 (6.8 to 185.9)	-30.7 (-47.7 to 16)
		Female	1148 (474 to 1748)	241.3 (122.4 to 338.8)	19 (10 to 27)	5.7 (3.5 to 7.6)	976 (747 to 1238)	118.9 (91.8 to 147)	23 (18 to 29)	3.5 (2.8 to 4.3)	-15 (-49.1 to 107.4)	-50.7 (-68 to 1.4)	24.1 (-19.3 to 152.5)	-39.2 (-57.1 to 1.9)
		Male	1265 (581 to 1954)	288.7 (157.8 to 405.8)	24 (13 to 34)	8.2 (5.2 to 11)	1812 (1305 to 2296)	214 (155 to 270.3)	42 (31 to 53)	6.1 (4.5 to 7.8)	43.3 (-13.6 to 198.3)	-25.9 (-51.9 to 35.1)	75.1 (13.3 to 224.6)	-24.8 (-49.1 to 27.8)
	Other malignant neoplasms	Both	872 (485 to 1229)	122.1 (75.3 to 164.4)	19 (12 to 26)	4 (2.8 to 5.3)	1599 (1373 to 1889)	102.5 (87.5 to 120.1)	42 (35 to 50)	3.3 (2.8 to 4)	83.4 (32.2 to 243.1)	-16.1 (-37.7 to 37.7)	116.6 (59.1 to 252.1)	-16.9 (-38.5 to 30.9)

Province	Cause	Sex	1990				2019				1990-2019 percent change (%)			
			DALYs		Death		DALYs		Death		DALYs		Death	
			Number	Rate	Number	Rate	Number	Rate	Number	Rate	Number	Rate	Number	Rate
		Female	368 (184 to 503)	100.6 (59.1 to 136.3)	8 (4 to 10)	3.1 (2.1 to 4.3)	674 (555 to 793)	87.7 (72.8 to 103.2)	18 (15 to 21)	2.9 (2.4 to 3.4)	83.1 (28.1 to 268)	-12.9 (-38.7 to 53)	131.9 (63.2 to 301.4)	-8.9 (-37 to 42.8)
		Male	504 (282 to 784)	141.4 (86.9 to 208.5)	12 (7 to 17)	4.9 (3 to 7)	925 (732 to 1212)	117.4 (92.5 to 152.9)	24 (19 to 32)	3.8 (2.9 to 5.1)	83.6 (21 to 230.6)	-17 (-43.3 to 46.4)	106.7 (37 to 266.3)	-21.5 (-46.9 to 33)
	Other neoplasms	Both	25 (17 to 31)	5 (3.3 to 6.4)	1 (0 to 1)	0.2 (0.1 to 0.3)	57 (48 to 74)	4.4 (3.7 to 5.8)	2 (1 to 2)	0.2 (0.1 to 0.2)	130 (79.4 to 229.5)	-11.1 (-32.9 to 33.3)	145.3 (73.3 to 314.7)	-16.7 (-41.5 to 41.9)
		Female	10 (6 to 14)	4 (2.3 to 5.5)	0 (0 to 0)	0.1 (0.1 to 0.2)	25 (20 to 31)	3.7 (3 to 4.6)	1 (1 to 1)	0.1 (0.1 to 0.2)	145.9 (67.1 to 297.2)	-7 (-36.8 to 69.4)	179.2 (72.8 to 564.7)	-10.6 (-44.4 to 138.6)
		Male	15 (10 to 20)	5.9 (3.7 to 7.9)	0 (0 to 1)	0.2 (0.1 to 0.3)	33 (25 to 47)	5.2 (4 to 7.5)	1 (1 to 2)	0.2 (0.1 to 0.3)	119.4 (58.5 to 229.5)	-11.3 (-37.3 to 31.2)	127.4 (50.2 to 278.9)	-19.3 (-45.8 to 27.3)
	Cardiovascular diseases	Both	46962 (32244 to 55277)	11202.9 (8387.2 to 13034.7)	1737 (1296 to 2031)	553 (445.8 to 632.5)	70475 (62663 to 79014)	5915.8 (5265.7 to 6599.3)	3041 (2671 to 3396)	306.8 (266.7 to 341.7)	50.1 (22.7 to 123.5)	-47.2 (-56.4 to -28.1)	75 (43.5 to 140.4)	-44.5 (-53.6 to -30.5)
		Female	18152 (12597 to 22030)	9295.9 (7206.4 to 11084.6)	694 (548 to 826)	474.7 (395.4 to 553.1)	27762 (24016 to 31321)	5064 (4402.2 to 5661.6)	1327 (1140 to 1490)	295.9 (252.6 to 330.1)	52.9 (20.6 to 119)	-45.5 (-56 to -29)	91.2 (54.1 to 145.8)	-37.7 (-48.6 to -24.1)
		Male	28810 (19514 to 35672)	12849.9 (9344.7 to 15844.4)	1043 (734 to 1293)	626.7 (478.4 to 757.2)	42713 (36047 to 50534)	6830.9 (5774.5 to 8076.8)	1714 (1442 to 2029)	325.2 (271.5 to 384.3)	48.3 (11.4 to 124.9)	-46.8 (-60 to -24)	64.3 (24.2 to 138.6)	-48.1 (-59.7 to -29.8)
	Rheumatic heart disease	Both	1270 (697 to 1807)	166.5 (102.6 to 242.9)	22 (12 to 34)	4.3 (2.7 to 7.2)	1313 (1065 to 1636)	77.8 (64.2 to 94.1)	24 (20 to 29)	2 (1.6 to 2.3)	3.4 (-30.9 to 87.5)	-53.3 (-71.2 to -23.8)	8.5 (-40.4 to 98.7)	-54.1 (-77.2 to -21.4)
		Female	694 (362 to 1008)	182.5 (105.3 to 271.6)	12 (6 to 18)	4.6 (2.6 to 7.7)	599 (467 to 767)	73.4 (58.4 to 92.3)	11 (9 to 14)	1.9 (1.5 to 2.5)	-13.7 (-44.5 to 66.2)	-59.8 (-75.1 to -24.5)	-6.3 (-50 to 110.6)	-57.7 (-79.9 to -12.1)
		Male	576 (327 to 894)	150.6 (89.6 to 244.9)	11 (6 to 18)	4.1 (2.2 to 7.5)	714 (553 to 920)	82.4 (63.8 to 105.2)	13 (10 to 17)	2.1 (1.5 to 2.7)	24 (-28.6 to 130.1)	-45.3 (-70.2 to -1.1)	24.9 (-41.5 to 162.5)	-49.2 (-78.4 to 2.7)
	Ischemic heart disease	Both	25491 (17167 to 30845)	6499.1 (4766.3 to 7708.6)	1029 (755 to 1223)	326.8 (260.3 to 380.8)	39719 (34618 to 45074)	3354.1 (2943.6 to 3781.2)	1813 (1584 to 2040)	181.4 (157.8 to 203.5)	55.8 (23.5 to 134.6)	-48.4 (-58.5 to -28.5)	76.1 (42.2 to 141.6)	-44.5 (-54 to -29.6)
		Female	8706 (6068 to 10946)	4945.3 (3796.7 to 6051.3)	380 (293 to 462)	264.8 (214.5 to 316.1)	14049 (11990 to 16121)	2642.3 (2272.4 to 2999.4)	748 (641 to 843)	167.4 (142.5 to 187.9)	61.4 (24 to 136.5)	-46.6 (-57.5 to -28.4)	96.7 (57.4 to 157.1)	-36.8 (-48.2 to -21.7)
		Male	16784 (10879 to 21825)	7857.1 (5518.1 to 9986.9)	649 (446 to 824)	385.9 (293.9 to 475.6)	25670 (21432 to 30755)	4102.9 (3434.9 to 4904.7)	1065 (887 to 1264)	199.7 (166 to 236.1)	52.9 (11.5 to 139.9)	-47.8 (-61.6 to -22.7)	64.1 (22 to 144.9)	-48.2 (-60.8 to -28.5)
	Stroke	Both	10938 (7671 to 13323)	2689.9 (2041.1 to 3271.3)	402 (298 to 494)	134.7 (104.3 to 162.5)	15771 (13936 to 17727)	1348.4 (1190.4 to 1509.6)	647 (556 to 735)	66.7 (56.7 to 75.8)	44.2 (13.9 to 107.4)	-49.9 (-60.2 to -32.5)	61.2 (25.3 to 121.7)	-50.4 (-60.9 to -35.3)
		Female	4699 (3369 to 5795)	2450.5 (1854.1 to 3006.8)	172 (127 to 214)	120.8 (91.4 to 147.9)	7216 (6148 to 8267)	1295.5 (1108.3 to 1480.6)	305 (254 to 351)	67.9 (56 to 77.7)	53.6 (18.4 to 117.2)	-47.1 (-58.4 to -28.6)	77 (36.4 to 145.8)	-43.8 (-55.4 to -23.6)
		Male	6238 (4177 to 8167)	2901.9 (2055.4 to 3744.7)	229 (154 to 303)	148.7 (105 to 191.7)	8555 (7166 to 10099)	1417.2 (1186.1 to 1683.2)	342 (280 to 411)	67.2 (54.3 to 80.8)	37.1 (-0.3 to 111.3)	-51.2 (-64.5 to -29)	49.2 (6.7 to 122.9)	-54.8 (-67.9 to -35.7)
	Hypertensive heart disease	Both	4401 (2737 to 5735)	1211.2 (774.3 to 1558.4)	193 (121 to 249)	66.5 (42 to 86.7)	7268 (5143 to 8469)	664.5 (454.4 to 770.7)	366 (238 to 425)	38.7 (24.5 to 44.9)	65.2 (22.7 to 160.5)	-45.1 (-59.3 to -16.7)	89.8 (37.2 to 191.9)	-41.8 (-57.8 to -16.6)
		Female	1873 (1122 to 2762)	1142 (703.3 to 1714.5)	89 (54 to 137)	65.9 (40.2 to 103.4)	3285 (2287 to 3946)	642 (433.2 to 768.2)	180 (116 to 217)	41.4 (25.9 to 50.2)	75.4 (4.5 to 184.1)	-43.8 (-67.7 to -12.4)	101.4 (10.1 to 217.4)	-37.2 (-66.8 to -3.8)

Province	Cause	Sex	1990				2019				1990-2019 percent change (%)			
			DALYs		Death		DALYs		Death		DALYs		Death	
			Number	Rate	Number	Rate	Number	Rate	Number	Rate	Number	Rate	Number	Rate
		Male	2528 (1385 to 3588)	1253.7 (723.9 to 1734.9)	103 (58 to 145)	65.6 (37.6 to 88.9)	3983 (2697 to 4962)	694.3 (452.6 to 867.7)	186 (112 to 233)	37 (21.9 to 46.1)	57.6 (4.7 to 179.5)	-44.6 (-61.8 to -9.4)	79.8 (21 to 208.7)	-43.6 (-60.9 to -9.8)
	Non-rheumatic valvular heart disease	Both	239 (125 to 312)	49.6 (31.5 to 62.6)	8 (5 to 9)	2.1 (1.6 to 2.6)	596 (494 to 707)	41.6 (34.8 to 48.5)	20 (17 to 24)	1.8 (1.5 to 2)	150 (72.9 to 389.3)	-16 (-38.2 to 37.7)	168.8 (99.3 to 331.4)	-16 (-35.7 to 16.4)
		Female	123 (65 to 178)	54.6 (34.5 to 75)	4 (3 to 5)	2.4 (1.7 to 3.1)	265 (214 to 323)	39.9 (32.9 to 47.8)	10 (8 to 12)	1.9 (1.6 to 2.3)	116.6 (39 to 305.1)	-26.9 (-49.7 to 20.9)	151 (75.1 to 301.3)	-19.3 (-42.3 to 19.8)
		Male	116 (62 to 169)	44.5 (26.5 to 64.1)	4 (2 to 5)	1.8 (1.2 to 2.5)	331 (238 to 429)	43.5 (32.5 to 56)	10 (8 to 13)	1.7 (1.2 to 2.1)	185.2 (68.6 to 480.2)	-2.2 (-40.3 to 77.1)	188.9 (75.9 to 430.7)	-7.4 (-41.7 to 51.8)
	Cardiomyopathy and myocarditis	Both	530 (301 to 1106)	65.2 (41.8 to 115.4)	11 (7 to 19)	2.3 (1.6 to 3.7)	574 (410 to 965)	38.2 (27.9 to 64)	16 (12 to 28)	1.4 (1 to 2.4)	8.4 (-41.2 to 126.5)	-41.5 (-62 to 0.3)	51.4 (-4.2 to 167.8)	-39.4 (-59.1 to -5.8)
		Female	257 (134 to 530)	61.4 (36.5 to 116.1)	5 (3 to 10)	2.2 (1.3 to 4.5)	208 (151 to 343)	29 (21.3 to 47.5)	6 (5 to 10)	1.2 (0.8 to 1.9)	-18.8 (-58.6 to 101.4)	-52.8 (-70.7 to -7.6)	23.4 (-26 to 146.5)	-47.4 (-68 to -11.7)
		Male	273 (146 to 643)	67.9 (40.3 to 136.1)	6 (3 to 11)	2.3 (1.5 to 4)	365 (241 to 670)	47.3 (31.4 to 88.6)	10 (7 to 19)	1.6 (1.1 to 3.2)	34 (-33.4 to 183.1)	-30.3 (-60.7 to 26.6)	76.2 (-3.7 to 222.6)	-30 (-57.8 to 13.7)
	Atrial fibrillation and flutter	Both	210 (161 to 270)	72 (55.7 to 91.2)	6 (4 to 7)	2.8 (1.9 to 3.5)	718 (563 to 909)	73.3 (57.7 to 92.5)	25 (21 to 29)	2.9 (2.4 to 3.4)	241.3 (202.6 to 296.8)	1.8 (-10.2 to 20.7)	349.2 (246.9 to 561.1)	4.5 (-19.5 to 57.1)
		Female	111 (82 to 144)	79.8 (58.5 to 102.1)	4 (2 to 5)	3.3 (2 to 4.3)	384 (303 to 483)	82.3 (65.3 to 102.6)	14 (12 to 18)	3.6 (2.9 to 4.5)	247.2 (195.8 to 342.4)	3.2 (-13.1 to 35.3)	303.2 (197 to 616.1)	11.7 (-18.1 to 102.8)
		Male	100 (73 to 133)	62.7 (46.6 to 83.1)	2 (1 to 3)	2.1 (1.3 to 2.9)	334 (246 to 432)	65.4 (48.6 to 84.2)	11 (7 to 13)	2.3 (1.5 to 2.9)	234.7 (172.8 to 303.6)	4.2 (-14.6 to 25.1)	429.9 (226.9 to 726.8)	8.3 (-29.4 to 64.5)
	Aortic aneurysm	Both	77 (50 to 108)	19.1 (13.1 to 25.9)	3 (2 to 4)	1 (0.7 to 1.3)	200 (163 to 243)	16.7 (13.6 to 20.2)	9 (7 to 11)	0.9 (0.7 to 1.1)	161.6 (70.9 to 334.9)	-12.3 (-41.3 to 39.5)	187 (91.8 to 355.3)	-8.1 (-37 to 38.9)
		Female	16 (10 to 21)	8.9 (6.6 to 11.4)	1 (1 to 1)	0.5 (0.4 to 0.6)	46 (37 to 55)	7.8 (6.4 to 9.4)	2 (2 to 3)	0.4 (0.4 to 0.5)	191.7 (104 to 346)	-12.3 (-35.6 to 25)	200.8 (123.8 to 312.1)	-11.2 (-33.5 to 19.6)
		Male	61 (36 to 90)	28.3 (16.9 to 40.9)	2 (1 to 4)	1.4 (0.9 to 2)	155 (119 to 197)	25.9 (19.8 to 32.8)	7 (5 to 9)	1.3 (1 to 1.7)	153.8 (49.2 to 381)	-8.4 (-44.7 to 68.7)	182.9 (71.2 to 419.9)	-6.8 (-43.3 to 67.1)
	Peripheral artery disease	Both	22 (12 to 37)	7.1 (4 to 11.9)	0 (0 to 0)	0.1 (0.1 to 0.2)	85 (57 to 127)	8.8 (5.8 to 13.2)	2 (2 to 3)	0.2 (0.2 to 0.3)	289.1 (218.3 to 407.9)	23.6 (2.5 to 58.2)	1040.6 (507.2 to 1791.1)	188.6 (44.1 to 366.9)
		Female	12 (6 to 21)	8.2 (4.4 to 14.3)	0 (0 to 0)	0.1 (0 to 0.2)	42 (24 to 66)	8.8 (5.2 to 14)	1 (0 to 1)	0.2 (0.1 to 0.3)	243.5 (173.9 to 343.4)	7.1 (-15.1 to 38.6)	900.4 (220.6 to 2424.9)	174 (-11.5 to 597.9)
		Male	10 (6 to 16)	6 (3.4 to 9.7)	0 (0 to 0)	0.1 (0.1 to 0.1)	44 (31 to 63)	8.8 (6.1 to 12.6)	1 (1 to 2)	0.3 (0.2 to 0.4)	345 (240.3 to 520)	47.4 (16.4 to 100.2)	1143.3 (649.9 to 2160.6)	205.8 (88.7 to 432)
	Endocarditis	Both	189 (107 to 279)	22.6 (14.7 to 31.9)	4 (2 to 5)	0.7 (0.5 to 1.2)	222 (171 to 328)	14.2 (10.9 to 21.7)	6 (5 to 10)	0.5 (0.4 to 0.9)	17.7 (-34.7 to 144.5)	-37.3 (-55.7 to -3.9)	74.8 (19.8 to 171.5)	-27.8 (-43.2 to -3.3)
		Female	93 (39 to 157)	21.2 (12.3 to 32.3)	2 (1 to 3)	0.7 (0.4 to 1.3)	86 (63 to 150)	12.1 (8.9 to 22)	3 (2 to 5)	0.5 (0.4 to 1)	-7.9 (-54.1 to 163.5)	-42.9 (-64.2 to -2.7)	65.3 (1.1 to 186.5)	-22.8 (-44.4 to 10.9)
		Male	96 (59 to 152)	23.8 (15.5 to 37.2)	2 (1 to 3)	0.8 (0.5 to 1.3)	137 (98 to 211)	16.3 (11.7 to 25.3)	4 (3 to 6)	0.5 (0.4 to 0.9)	42.4 (-25.6 to 167.5)	-31.6 (-55.1 to 7.3)	83.1 (17.1 to 187.9)	-29.4 (-50.1 to 5.7)
		Both	3597 (2529 to 4582)	400.7 (291.8 to 522)	60 (42 to 80)	11.8 (8.6 to 17.1)	4007 (3425 to 4670)	278.2 (238.3 to 321.7)	112 (92 to 129)	10.3 (8.4 to 12)	11.4 (-17.4 to 63.7)	-30.6 (-49.7 to -0.1)	84.7 (25.6 to 179.6)	-12.2 (-45.8 to 27)

Province	Cause	Sex	1990				2019				1990-2019 percent change (%)			
			DALYs		Death		DALYs		Death		DALYs		Death	
			Number	Rate	Number	Rate	Number	Rate	Number	Rate	Number	Rate	Number	Rate
	Other cardiovascular and circulatory diseases	Female	1569 (1014 to 2114)	341.6 (239.4 to 445.2)	25 (17 to 34)	9.6 (7.2 to 14.8)	1582 (1319 to 1865)	230.8 (193.2 to 269.6)	46 (37 to 54)	9.4 (7.4 to 11)	0.8 (-29.4 to 65.2)	-32.4 (-53.3 to -1.9)	84 (16.9 to 187.8)	-2 (-46.2 to 38.9)
		Male	2028 (1408 to 2691)	453.5 (323.8 to 599.6)	35 (24 to 48)	13.8 (9.7 to 20.5)	2425 (1962 to 2911)	326.9 (254.3 to 395.1)	65 (47 to 80)	11.4 (7.8 to 14.1)	19.6 (-14.6 to 76.4)	-27.9 (-49.9 to 7.3)	85.2 (21.6 to 193.1)	-17.5 (-51.1 to 33.6)
	Chronic respiratory diseases	Both	4712 (3598 to 6096)	862.4 (680.3 to 1126.9)	96 (68 to 139)	29.5 (22.2 to 43.9)	8305 (7019 to 9935)	585.9 (506.6 to 695)	169 (144 to 214)	16.9 (14.2 to 21.6)	76.2 (45.6 to 129.8)	-32.1 (-44.3 to -14.3)	76.7 (34.1 to 154.5)	-42.9 (-56.6 to -23.4)
		Female	2067 (1534 to 2661)	762.8 (579 to 1002)	38 (25 to 59)	24.2 (16.3 to 38)	3588 (2972 to 4351)	512.1 (433.4 to 614.4)	68 (55 to 95)	14.6 (11.8 to 20.2)	73.6 (40.3 to 132.9)	-32.9 (-46.3 to -10.9)	79.7 (27.7 to 192.1)	-39.6 (-58.1 to -6.3)
		Male	2646 (2001 to 3495)	954.8 (728 to 1341.3)	58 (39 to 94)	35.2 (25.6 to 57)	4717 (3916 to 5800)	663.1 (556.9 to 808.7)	101 (82 to 134)	19.4 (15.6 to 26.4)	78.3 (40.1 to 136)	-30.5 (-47.3 to -8.5)	74.7 (19.4 to 168.5)	-45 (-61.6 to -21.1)
	Chronic obstructive pulmonary disease	Both	1750 (1299 to 2460)	391.3 (300.9 to 569.2)	45 (30 to 76)	14.7 (10.3 to 25.7)	4291 (3708 to 5058)	338.2 (293.2 to 402.7)	113 (94 to 153)	11.6 (9.6 to 15.8)	145.2 (97.2 to 230)	-13.6 (-31.2 to 11.7)	154.6 (90.4 to 282.2)	-21 (-41.4 to 11)
		Female	685 (478 to 1002)	309.1 (218.9 to 491.9)	15 (8 to 30)	10.5 (6 to 22.3)	1692 (1428 to 2161)	272.4 (230.2 to 354)	42 (33 to 65)	9.4 (7.3 to 14.3)	147.2 (91.7 to 264.9)	-11.9 (-32.5 to 28)	177.5 (87.1 to 470.4)	-10.4 (-40.4 to 75.5)
		Male	1066 (779 to 1565)	468.9 (354.7 to 694.7)	29 (19 to 50)	19.2 (13 to 32.4)	2598 (2175 to 3160)	406.7 (340 to 503.4)	71 (57 to 98)	13.9 (11 to 19.8)	143.8 (81.8 to 236.5)	-13.3 (-36 to 18.9)	142.7 (62.7 to 285.1)	-27.6 (-50.9 to 10.1)
	Pneumoconiosis	Both	7 (4 to 11)	1.5 (0.9 to 2.4)	0 (0 to 0)	0.1 (0 to 0.1)	39 (31 to 49)	2.8 (2.2 to 3.5)	1 (1 to 1)	0.1 (0.1 to 0.1)	455.2 (252.1 to 904.4)	81.1 (16.5 to 222.9)	384.6 (191 to 930)	63.5 (1.3 to 230.4)
		Female	2 (1 to 2)	0.6 (0.4 to 0.8)	0 (0 to 0)	0 (0 to 0)	15 (11 to 21)	2 (1.5 to 2.6)	0 (0 to 0)	0 (0 to 0.1)	870.4 (639 to 1228.6)	223.6 (148.1 to 323.9)	879.9 (480 to 1760)	220.9 (83.9 to 472)
		Male	5 (3 to 9)	2.4 (1.2 to 4)	0 (0 to 0)	0.1 (0.1 to 0.2)	24 (18 to 32)	3.6 (2.7 to 4.9)	1 (1 to 1)	0.2 (0.1 to 0.2)	338.5 (152.7 to 852.8)	53.5 (-9.5 to 227.7)	334.7 (144.9 to 908)	45.8 (-15.6 to 236.8)
	Asthma	Both	2800 (2072 to 3638)	447.8 (335.4 to 575.6)	49 (33 to 69)	14.3 (9.4 to 20.7)	3300 (2477 to 4474)	205.7 (159.3 to 270.1)	45 (36 to 54)	4.3 (3.5 to 5.3)	17.9 (-5 to 52.4)	-54.1 (-65.5 to -39.8)	-9 (-38.7 to 38.7)	-69.7 (-81 to -55.4)
		Female	1304 (908 to 1720)	432.2 (304.8 to 587)	22 (13 to 33)	13.2 (7.6 to 21.3)	1560 (1141 to 2121)	200.2 (149.2 to 264.6)	21 (14 to 28)	4.4 (2.8 to 5.6)	19.6 (-8.2 to 56.9)	-53.7 (-66.4 to -38)	-2.3 (-41.1 to 55.1)	-66.9 (-81.4 to -48.2)
		Male	1496 (1082 to 2043)	460.9 (334.9 to 642.8)	27 (17 to 42)	15.3 (10.1 to 24.7)	1740 (1294 to 2366)	211.8 (162 to 281.2)	23 (18 to 33)	4.4 (3.3 to 6.3)	16.3 (-11.1 to 52.1)	-54 (-67.2 to -35.6)	-14.4 (-45.5 to 48.1)	-71.5 (-83.1 to -52.4)
	Interstitial lung disease and pulmonary sarcoidosis	Both	48 (33 to 71)	10 (6.6 to 15.7)	1 (1 to 2)	0.4 (0.2 to 0.7)	178 (134 to 222)	12.8 (9.9 to 15.7)	5 (3 to 6)	0.5 (0.3 to 0.6)	269.9 (139.6 to 459.8)	27.3 (-19.3 to 99.1)	307.6 (128.5 to 685.3)	26 (-28.7 to 151.2)
		Female	20 (13 to 30)	8.6 (5.5 to 15)	0 (0 to 1)	0.3 (0.1 to 0.6)	79 (55 to 103)	11.4 (7.9 to 15.1)	2 (1 to 3)	0.4 (0.2 to 0.6)	299.7 (173.6 to 513.9)	31.5 (-17 to 119.4)	347.2 (135.1 to 932.8)	34.3 (-30.5 to 224.5)
		Male	28 (19 to 46)	11.4 (7.2 to 19.3)	1 (0 to 1)	0.4 (0.2 to 0.9)	98 (70 to 128)	14.2 (10.3 to 18.5)	3 (2 to 4)	0.5 (0.3 to 0.8)	248.9 (95.8 to 466.8)	25 (-29.8 to 115.8)	283.3 (94.5 to 707.6)	19.8 (-38.4 to 163)
	Other chronic respiratory diseases	Both	107 (77 to 167)	11.8 (8.9 to 16.8)	1 (0 to 1)	0.1 (0.1 to 0.2)	498 (337 to 633)	26.4 (16.9 to 34)	5 (1 to 8)	0.4 (0.1 to 0.6)	365.1 (132.6 to 600.2)	124.3 (16.9 to 235.3)	675.6 (15.3 to 2292)	228.3 (-42.4 to 749.7)
		Female	57 (40 to 88)	12.3 (9.1 to 16.9)	0 (0 to 1)	0.1 (0.1 to 0.2)	241 (159 to 315)	26.1 (16.3 to 34.6)	2 (0 to 4)	0.4 (0.1 to 0.6)	326.3 (119.2 to 552.5)	113.2 (15 to 220.1)	644.2 (-10.5 to 2265.4)	240.4 (-49.6 to 841.5)

Province	Cause	Sex	1990				2019				1990-2019 percent change (%)			
			DALYs		Death		DALYs		Death		DALYs		Death	
			Number	Rate	Number	Rate	Number	Rate	Number	Rate	Number	Rate	Number	Rate
		Male	51 (34 to 83)	11.3 (8.1 to 17.3)	0 (0 to 1)	0.1 (0.1 to 0.3)	257 (172 to 335)	26.8 (16.9 to 35.8)	3 (1 to 5)	0.4 (0.1 to 0.7)	408.6 (144.1 to 717.6)	136.9 (17.2 to 277.4)	703.1 (35.4 to 2618.1)	219 (-40.6 to 880.8)
	Digestive diseases	Both	6339 (4286 to 8000)	983.2 (707.9 to 1168.1)	129 (87 to 156)	31.9 (23.4 to 40.3)	9464 (7874 to 11578)	624.7 (527.7 to 753.8)	218 (185 to 264)	19.5 (16.6 to 23.3)	49.3 (9.2 to 132.6)	-36.5 (-49.2 to -9.7)	69.1 (31.4 to 163.1)	-38.9 (-52 to -13.9)
		Female	2851 (1863 to 3776)	877.3 (636.2 to 1087.4)	53 (35 to 68)	27.5 (19.9 to 37.3)	3946 (3131 to 4907)	537.2 (438.7 to 650)	85 (69 to 103)	16.9 (13.8 to 20.4)	38.4 (-3.5 to 122)	-38.8 (-51.9 to -14.6)	59.9 (18.9 to 146)	-38.6 (-55.5 to -14)
		Male	3489 (2277 to 4449)	1069 (737.6 to 1321)	76 (49 to 96)	35.6 (24.9 to 45.4)	5518 (4469 to 6822)	714.6 (578.8 to 879.7)	133 (107 to 167)	22.5 (18.1 to 28.2)	58.2 (12.4 to 154.6)	-33.2 (-49.8 to -0.1)	75.7 (25.1 to 192.3)	-36.8 (-53.5 to -3.7)
	Cirrhosis and other chronic liver diseases	Both	2994 (1623 to 4032)	496.8 (319.2 to 617.9)	78 (50 to 97)	18.9 (13.3 to 24.2)	4349 (3609 to 5381)	303.7 (255.3 to 372.2)	142 (119 to 173)	12.2 (10.3 to 14.8)	45.2 (-1.3 to 174)	-38.9 (-54 to 0.4)	81.6 (35.7 to 199.9)	-35.4 (-50.7 to -3.2)
		Female	1252 (609 to 1865)	395.8 (246.4 to 524.2)	30 (19 to 40)	15.2 (10.1 to 21.3)	1388 (1108 to 1720)	207.8 (168.3 to 253)	50 (40 to 61)	9.4 (7.6 to 11.3)	10.9 (-32.5 to 137.4)	-47.5 (-63 to -13.3)	63.8 (15.8 to 173.6)	-38.1 (-57.8 to -5.8)
		Male	1743 (1034 to 2328)	581.2 (365.7 to 752.6)	48 (30 to 62)	22.1 (14.6 to 29.2)	2961 (2321 to 3813)	399.8 (313.8 to 511.4)	92 (72 to 118)	15.1 (11.9 to 19.2)	69.9 (13.3 to 212.2)	-31.2 (-52.5 to 16.1)	92.9 (35.1 to 227.6)	-31.6 (-51.6 to 10.7)
	Upper digestive system diseases	Both	1422 (994 to 2010)	253.5 (188.2 to 342.2)	22 (15 to 29)	6.7 (4.8 to 9)	2549 (1620 to 3992)	151.2 (101.5 to 228)	23 (19 to 27)	2.2 (1.8 to 2.7)	79.2 (30.2 to 130.7)	-40.4 (-53.1 to -25.9)	2.3 (-25.9 to 58.2)	-66.5 (-76.3 to -51.5)
		Female	681 (467 to 990)	249.3 (178.1 to 345.3)	10 (6 to 14)	6.3 (4.1 to 9.4)	1300 (819 to 2061)	158 (105.2 to 243.1)	10 (8 to 13)	2.3 (1.8 to 3)	91 (37.4 to 144.1)	-36.7 (-51.8 to -20.6)	7.9 (-32 to 79)	-62.9 (-76.9 to -37.7)
		Male	742 (529 to 1042)	255.3 (186.8 to 349.1)	13 (8 to 17)	7 (4.8 to 9.4)	1249 (813 to 1890)	145.4 (98.7 to 215.2)	12 (10 to 16)	2.2 (1.7 to 2.9)	68.4 (19 to 123.7)	-43 (-57.1 to -25.9)	-2 (-33.2 to 62.8)	-67.7 (-78.2 to -48.5)
	Appendicitis	Both	304 (141 to 528)	35.8 (20.2 to 53.3)	6 (3 to 8)	1.1 (0.7 to 1.6)	205 (159 to 277)	11.8 (9.4 to 15.8)	4 (3 to 6)	0.3 (0.3 to 0.4)	-32.4 (-62 to 75)	-66.9 (-79.3 to -28.5)	-27.5 (-56.8 to 70.4)	-70.2 (-81.3 to -45.3)
		Female	113 (43 to 221)	26.6 (13.7 to 46.1)	2 (1 to 4)	0.8 (0.5 to 1.5)	72 (51 to 100)	8.6 (6.2 to 11.9)	1 (1 to 2)	0.2 (0.2 to 0.4)	-36.3 (-69.8 to 94)	-67.6 (-83.9 to -23.2)	-35 (-70.3 to 71.3)	-70.5 (-86.3 to -40)
		Male	191 (83 to 365)	44.3 (21.8 to 69.7)	4 (2 to 6)	1.3 (0.7 to 1.9)	134 (96 to 190)	15 (10.8 to 21.6)	3 (2 to 4)	0.4 (0.3 to 0.6)	-30 (-65.1 to 105.6)	-66.1 (-80.6 to -18.4)	-23.2 (-58.6 to 104)	-69.4 (-81.7 to -34)
	Paralytic ileus and intestinal obstruction	Both	911 (486 to 1548)	71.9 (44.9 to 114.5)	13 (8 to 20)	1.8 (1.3 to 2.9)	524 (358 to 729)	34.6 (24.9 to 46.1)	13 (10 to 17)	1.2 (1 to 1.6)	-42.5 (-72.5 to 30.7)	-51.9 (-74.7 to -7.1)	6.7 (-41.1 to 102.1)	-33.6 (-60.3 to 6)
		Female	442 (211 to 851)	70.6 (39.3 to 122.8)	6 (3 to 11)	1.8 (1.1 to 2.9)	229 (157 to 337)	31 (22.3 to 43.3)	6 (4 to 8)	1.1 (0.9 to 1.5)	-48.2 (-77.8 to 27.8)	-56.1 (-78 to -7.6)	-5.8 (-52.3 to 95.3)	-38.6 (-62.1 to 12.3)
		Male	469 (218 to 873)	72.9 (40.5 to 126.1)	6 (3 to 11)	1.9 (1.2 to 3.2)	295 (184 to 446)	38.4 (24.9 to 55.2)	8 (5 to 11)	1.4 (0.9 to 1.8)	-37.2 (-74.5 to 72)	-47.4 (-76.7 to 19.4)	18.6 (-45.8 to 166.3)	-27.2 (-63.2 to 29.3)
	Inguinal, femoral, and abdominal hernia	Both	161 (95 to 269)	18.2 (11.9 to 26.2)	1 (0 to 2)	0.2 (0.1 to 0.3)	292 (194 to 423)	16.9 (11.6 to 24)	1 (1 to 2)	0.1 (0.1 to 0.2)	81 (4.7 to 162.4)	-7.1 (-34.5 to 18.3)	84.3 (-36.4 to 367.6)	-28.6 (-59.5 to 28.3)
		Female	62 (32 to 137)	13.1 (7.9 to 22.7)	0 (0 to 1)	0.1 (0.1 to 0.3)	102 (65 to 153)	11.9 (7.7 to 17.3)	1 (0 to 1)	0.1 (0.1 to 0.2)	65.2 (-30.2 to 180.4)	-9 (-51 to 28.4)	89.9 (-60.5 to 480.6)	-12.8 (-57.1 to 67.2)
		Male	99 (60 to 155)	22.9 (15.1 to 31.7)	1 (0 to 1)	0.3 (0.2 to 0.6)	189 (124 to 276)	21.9 (14.9 to 31)	1 (1 to 1)	0.2 (0.1 to 0.2)	90.8 (26.1 to 176.2)	-4.5 (-26.7 to 24.5)	80.8 (-24.9 to 350.1)	-40.4 (-73.4 to 19)
	Inflammatory bowel disease	Both	49 (35 to 73)	9.2 (6.7 to 12.5)	1 (0 to 1)	0.1 (0.1 to 0.2)	182 (122 to 243)	11.1 (7.3 to 14.6)	3 (1 to 3)	0.2 (0.1 to 0.3)	268.2 (95.3 to 399.9)	21.3 (-25.2 to 58.9)	364.7 (36.7 to 783)	53.2 (-46.2 to 179.2)
		Female	23 (15 to 34)	8.8 (6.1 to 12.5)	0 (0 to 0)	0.1 (0.1 to 0.2)	82 (56 to 111)	10.1 (6.7 to 13.3)	1 (0 to 1)	0.2 (0.1 to 0.3)	254.7 (101.6 to 394.3)	14.7 (-23.9 to 57.5)	384.8 (39.9 to 984.3)	62.4 (-35 to 239.2)
		Male	26 (18 to 40)	9.5 (6.6 to 13.4)	0 (0 to 1)	0.2 (0.1 to 0.3)	100 (62 to 137)	12.2 (7.3 to 16.4)	2 (1 to 2)	0.3 (0.1 to 0.4)	279.9 (82.9 to 459.9)	28.5 (-30 to 87.7)	353.1 (23.4 to 874.4)	49.6 (-54.1 to 209.2)

Province	Cause	Sex	1990				2019				1990-2019 percent change (%)			
			DALYs		Death		DALYs		Death		DALYs		Death	
			Number	Rate	Number	Rate	Number	Rate	Number	Rate	Number	Rate	Number	Rate
	Vascular intestinal disorders	Both	34 (24 to 46)	9.5 (6.7 to 13.1)	2 (1 to 2)	0.5 (0.4 to 0.8)	95 (77 to 117)	8.5 (6.8 to 10.4)	5 (4 to 6)	0.5 (0.4 to 0.6)	180.1 (104.7 to 316.8)	-11.2 (-37.3 to 28.6)	199.2 (108.4 to 347.2)	-13.1 (-40.9 to 29.2)
		Female	15 (10 to 23)	9.4 (5.6 to 14.5)	1 (0 to 1)	0.6 (0.3 to 0.9)	42 (32 to 54)	7.9 (6.1 to 10)	2 (2 to 3)	0.5 (0.4 to 0.6)	180.9 (90.1 to 342.6)	-15.6 (-45.7 to 38.1)	187.3 (76.1 to 388.7)	-15.8 (-49.9 to 49.5)
		Male	19 (12 to 27)	9.5 (6.3 to 13.3)	1 (0 to 1)	0.5 (0.3 to 0.7)	53 (40 to 69)	9.1 (6.8 to 11.9)	2 (2 to 3)	0.5 (0.4 to 0.6)	179.4 (83.2 to 370.8)	-4.1 (-36.4 to 55.9)	210.8 (104.8 to 424)	-6.3 (-37.2 to 47.7)
	Gallbladder and biliary diseases	Both	277 (194 to 386)	54.7 (39.9 to 74.3)	3 (2 to 5)	1.1 (0.8 to 1.6)	717 (513 to 985)	49.2 (36.6 to 64.9)	11 (8 to 13)	1.1 (0.8 to 1.4)	158.7 (97.3 to 216.3)	-10 (-29.4 to 8.4)	241 (92.9 to 466.2)	1.2 (-36.3 to 52.5)
		Female	177 (121 to 251)	71.6 (50.6 to 98.4)	2 (1 to 3)	1.2 (0.8 to 1.8)	475 (327 to 678)	65 (46.3 to 89.5)	6 (4 to 8)	1.3 (0.9 to 1.7)	168.8 (108.6 to 229.4)	-9.3 (-26.9 to 9.2)	252.1 (84.2 to 501)	12.3 (-31.5 to 82.6)
		Male	100 (70 to 145)	39.2 (28 to 56.3)	2 (1 to 3)	1 (0.7 to 1.8)	242 (179 to 320)	34.2 (25.6 to 43.8)	5 (3 to 7)	1 (0.7 to 1.4)	141.1 (69.9 to 222.8)	-12.7 (-37.9 to 17.2)	228.7 (73.8 to 513.7)	-4.6 (-46.3 to 63.5)
	Pancreatitis	Both	72 (40 to 138)	15.7 (9.4 to 31.4)	2 (1 to 5)	0.7 (0.4 to 1.5)	144 (100 to 249)	10.1 (7.2 to 17.4)	5 (3 to 8)	0.4 (0.3 to 0.8)	100.3 (43.9 to 218.9)	-35.8 (-55 to -3.1)	109.5 (41.8 to 225.3)	-36.9 (-58.7 to -2.6)
		Female	27 (15 to 56)	12.9 (7.4 to 27.8)	1 (0 to 2)	0.6 (0.3 to 1.6)	53 (37 to 94)	8.5 (6 to 15.2)	2 (1 to 4)	0.4 (0.3 to 0.8)	94.3 (25.2 to 205.8)	-34.6 (-59.1 to 3.7)	121.7 (29.4 to 272.2)	-30.9 (-62.3 to 19.2)
		Male	45 (24 to 91)	17.9 (10.1 to 35.2)	1 (1 to 3)	0.7 (0.4 to 1.4)	91 (58 to 170)	11.8 (7.8 to 21.5)	3 (2 to 5)	0.4 (0.3 to 0.8)	103.9 (32.8 to 241.6)	-34 (-56.5 to 6.3)	100.9 (25.2 to 234.2)	-36.7 (-59.9 to 1.6)
	Other digestive diseases	Both	114 (68 to 205)	17.7 (12 to 23.9)	2 (2 to 3)	0.7 (0.5 to 1.1)	407 (204 to 542)	27.4 (14.6 to 35.3)	12 (6 to 16)	1.1 (0.6 to 1.4)	255.8 (40.8 to 598.7)	54.6 (-32.2 to 153.4)	409.3 (103.1 to 799.6)	57.7 (-29.3 to 145.5)
		Female	60 (33 to 114)	19.1 (11.8 to 27.8)	1 (1 to 2)	0.8 (0.5 to 1.3)	203 (83 to 281)	28.4 (12.6 to 37.6)	6 (3 to 8)	1.3 (0.5 to 1.6)	238.6 (19.2 to 598.4)	49.1 (-26.1 to 164.8)	389.5 (115 to 859.4)	62.3 (-26.1 to 198.9)
		Male	55 (32 to 101)	16.2 (10.5 to 24.5)	1 (1 to 2)	0.6 (0.4 to 1)	204 (116 to 276)	26.8 (16.5 to 35.3)	6 (4 to 8)	1 (0.7 to 1.4)	274.7 (31.3 to 664.2)	65 (-31.5 to 192)	432.1 (97.3 to 946.4)	66.1 (-32.1 to 188)
	Neurological disorders	Both	9629 (4955 to 16420)	1433.5 (802.3 to 2350.7)	77 (40 to 153)	30.9 (12.7 to 73)	22250 (10628 to 38554)	1380 (752.2 to 2333.1)	280 (123 to 640)	31 (12.6 to 72.6)	131.1 (79.6 to 188.5)	-3.7 (-17.8 to 13.5)	265.9 (157 to 425.9)	0.5 (-14.3 to 37.7)
		Female	5130 (2443 to 9184)	1558.6 (817.6 to 2683.3)	39 (19 to 83)	29.8 (11.5 to 72.6)	12248 (5207 to 22576)	1531.3 (760.1 to 2653.9)	127 (51 to 295)	31.4 (11.4 to 74.8)	138.7 (84.8 to 192.1)	-1.7 (-16 to 14.1)	229.8 (131.2 to 387.2)	5.3 (-13 to 50.1)
		Male	4499 (2475 to 7445)	1320.3 (782.7 to 2092.4)	38 (21 to 74)	32.1 (13.9 to 76.2)	10003 (5375 to 16644)	1238.2 (724.9 to 1983.7)	153 (70 to 344)	30.9 (13.4 to 71.8)	122.4 (69.9 to 192.9)	-6.2 (-21.8 to 14)	302.6 (168.8 to 490.3)	-3.9 (-23.2 to 30.1)
	Alzheimer's disease and other dementias	Both	830 (372 to 1891)	366.5 (165 to 821)	44 (10 to 122)	24 (5.7 to 66.8)	3411 (1509 to 7605)	379.3 (169.9 to 850.3)	210 (51 to 572)	24.7 (6.1 to 66.5)	310.8 (256.4 to 421.2)	3.5 (-7.6 to 29.8)	376 (301.1 to 567.8)	2.7 (-10.9 to 45.9)
		Female	441 (197 to 974)	375.3 (168.7 to 820.8)	25 (6 to 70)	24.4 (5.8 to 67.3)	1689 (749 to 3710)	401.2 (179.6 to 890.5)	100 (24 to 269)	26.2 (6.3 to 70.3)	283.3 (231.4 to 389.9)	6.9 (-5.6 to 37.9)	298.8 (232.1 to 483.9)	7.3 (-9.6 to 57.9)
		Male	390 (171 to 899)	357.6 (160.2 to 822)	19 (4 to 54)	23.6 (5.5 to 67.9)	1722 (773 to 3832)	359.2 (162.8 to 807.9)	110 (27 to 305)	23.4 (5.8 to 64.7)	341.9 (259.6 to 473.9)	0.4 (-15.4 to 25.5)	477.4 (353.9 to 731.7)	-0.8 (-18.7 to 39.7)
	Parkinson's disease	Both	228 (174 to 267)	76 (60.7 to 89.5)	11 (8 to 13)	4.4 (3.6 to 5.4)	727 (595 to 845)	75.8 (62.7 to 87.9)	41 (33 to 47)	4.6 (3.7 to 5.3)	219.6 (153.1 to 322.9)	-0.2 (-21.6 to 30.1)	277.3 (192.2 to 409.4)	2.5 (-22.6 to 36)

Province	Cause	Sex	1990				2019				1990-2019 percent change (%)			
			DALYs		Death		DALYs		Death		DALYs		Death	
			Number	Rate	Number	Rate	Number	Rate	Number	Rate	Number	Rate	Number	Rate
		Female	84 (65 to 107)	58 (45.8 to 74.3)	4 (3 to 5)	3.3 (2.5 to 4.6)	297 (251 to 344)	62.7 (53.4 to 72.2)	16 (13 to 19)	3.8 (3.1 to 4.5)	253.6 (170.8 to 368)	8.1 (-18.1 to 41.7)	300.1 (195.6 to 439.9)	15.8 (-14.8 to 54.9)
		Male	144 (100 to 181)	96.1 (70.1 to 119)	7 (5 to 9)	5.9 (4.3 to 7.3)	430 (316 to 525)	89.4 (64.9 to 109.9)	24 (17 to 30)	5.3 (3.6 to 6.5)	199.7 (112.3 to 335)	-7 (-34 to 30.9)	263.7 (149.8 to 448.8)	-9.7 (-38.9 to 30.9)
	Idiopathic epilepsy	Both	2631 (1345 to 4215)	231.1 (119.3 to 373.2)	18 (9 to 25)	1.8 (1 to 2.3)	2771 (1279 to 4918)	146.3 (66.3 to 259.2)	15 (13 to 19)	0.9 (0.7 to 1.1)	5.3 (-55.2 to 144.9)	-36.7 (-73.3 to 46)	-14 (-42.4 to 87.2)	-50.6 (-64.3 to -5.1)
		Female	1195 (553 to 1958)	213.5 (102 to 349.3)	8 (3 to 12)	1.6 (0.8 to 2.2)	1168 (495 to 2129)	126.9 (53.1 to 233.2)	6 (4 to 7)	0.7 (0.5 to 0.8)	-2.3 (-61.1 to 133.7)	-40.5 (-77.3 to 41.7)	-31.7 (-60.5 to 100.3)	-59.1 (-73.1 to -2.8)
		Male	1436 (734 to 2317)	247.6 (127.2 to 402.5)	10 (5 to 14)	1.9 (1.1 to 2.5)	1602 (793 to 2830)	164.7 (80.2 to 295.3)	10 (8 to 12)	1.1 (0.9 to 1.4)	11.6 (-51.2 to 155.7)	-33.5 (-71.3 to 51.7)	0.5 (-35.9 to 124.4)	-43.8 (-60.1 to 12.5)
	Multiple sclerosis	Both	115 (76 to 186)	20.7 (13.6 to 34.7)	2 (1 to 3)	0.3 (0.2 to 0.7)	370 (266 to 547)	20.5 (14.9 to 30.6)	5 (4 to 10)	0.3 (0.2 to 0.6)	221.3 (144.3 to 355.3)	-1 (-25 to 34.7)	240.2 (123.8 to 551.1)	2.7 (-32.4 to 76.1)
		Female	60 (39 to 94)	22 (14.5 to 34.8)	1 (0 to 1)	0.3 (0.1 to 0.7)	191 (142 to 264)	21.6 (16 to 30.3)	2 (2 to 4)	0.3 (0.2 to 0.6)	219.1 (104.8 to 376.3)	-1.9 (-34.8 to 41.1)	241.1 (70.7 to 644.1)	1.4 (-46.1 to 102.8)
		Male	55 (33 to 109)	19.4 (11.2 to 38.8)	1 (0 to 2)	0.4 (0.2 to 0.9)	179 (116 to 308)	19.6 (12.9 to 33.9)	3 (2 to 6)	0.4 (0.2 to 0.8)	223.7 (107.6 to 383.3)	0.6 (-36 to 51.8)	239.4 (78.5 to 563.7)	5.9 (-44.1 to 91.7)
	Motor neuron disease	Both	12 (8 to 15)	1.8 (1.3 to 2.3)	0 (0 to 0)	0 (0 to 0.1)	31 (26 to 36)	1.9 (1.6 to 2.2)	1 (0 to 1)	0 (0 to 0.1)	161.7 (99.9 to 272.3)	4.9 (-23.6 to 50.5)	182.2 (84.9 to 363.4)	6.6 (-30.9 to 74.8)
		Female	6 (4 to 8)	1.7 (1.1 to 2.3)	0 (0 to 0)	0 (0 to 0.1)	16 (14 to 19)	2 (1.7 to 2.4)	0 (0 to 0)	0 (0 to 0.1)	172.3 (96.1 to 339.5)	17.2 (-19.3 to 82)	246.5 (111.1 to 572.7)	33.3 (-22.9 to 137.5)
		Male	6 (4 to 8)	1.8 (1.2 to 2.5)	0 (0 to 0)	0 (0 to 0.1)	14 (11 to 17)	1.8 (1.4 to 2.2)	0 (0 to 0)	0 (0 to 0.1)	150.4 (71.8 to 268.6)	-3.7 (-36.3 to 54)	132.1 (31.3 to 365.6)	-8.7 (-47.7 to 80.1)
	Headache disorders	Both	5406 (1165 to 11888)	699.1 (170.4 to 1490.2)	--	--	14005 (3349 to 29888)	704.5 (171 to 1502.6)	--	--	159.1 (139.6 to 200.8)	0.8 (-4.3 to 6.6)	--	--
		Female	3174 (626 to 7123)	855 (192 to 1886.4)	--	--	8492 (1853 to 18539)	872.3 (196.1 to 1890.9)	--	--	167.5 (144.9 to 204.9)	2 (-4.9 to 8.6)	--	--
		Male	2231 (521 to 4859)	554.4 (150.5 to 1171.1)	--	--	5513 (1506 to 11568)	544.9 (152.4 to 1137.7)	--	--	147.1 (121.9 to 203.4)	-1.7 (-8.2 to 7.5)	--	--
	Other neurological disorders	Both	407 (181 to 713)	38.3 (19.5 to 64.1)	2 (1 to 3)	0.3 (0.2 to 0.4)	936 (550 to 1549)	51.7 (31.7 to 82.9)	8 (7 to 9)	0.5 (0.5 to 0.6)	129.7 (9.7 to 474.1)	34.9 (-30.6 to 196)	316.5 (195.9 to 613.1)	100 (45.1 to 225)
		Female	170 (69 to 308)	33 (16.2 to 56.6)	1 (0 to 1)	0.2 (0.1 to 0.3)	393 (219 to 667)	44.5 (25.7 to 73.7)	3 (2 to 3)	0.4 (0.3 to 0.5)	131.5 (5.1 to 498.6)	34.9 (-32.9 to 196.5)	380.1 (239.2 to 791.5)	117 (44.8 to 260.7)
		Male	238 (109 to 414)	43.3 (22.4 to 71.7)	1 (1 to 2)	0.3 (0.2 to 0.5)	542 (325 to 868)	58.7 (36.6 to 91.4)	5 (4 to 6)	0.7 (0.5 to 0.8)	128.4 (12.1 to 449.5)	35.4 (-29.7 to 190.4)	287 (146.6 to 647.1)	94.3 (29.3 to 255.3)
	Mental disorders	Both	18068 (13297 to 23994)	2288.1 (1691 to 3011.8)	0 (0 to 0)	0 (0 to 0)	45714 (33579 to 60417)	2340.2 (1735.2 to 3082.3)	0 (0 to 0)	0 (0 to 0)	153 (140.8 to 164.1)	2.3 (-0.6 to 4.8)	488.2 (155.4 to 2105.8)	89.8 (-18.1 to 585.5)

Province	Cause	Sex	1990				2019				1990-2019 percent change (%)			
			DALYs		Death		DALYs		Death		DALYs		Death	
			Number	Rate	Number	Rate	Number	Rate	Number	Rate	Number	Rate	Number	Rate
		Female	9819 (7141 to 13010)	2597 (1917.5 to 3443.8)	0 (0 to 0)	0 (0 to 0)	25292 (18632 to 33545)	2649.8 (1958.2 to 3500)	0 (0 to 0)	0 (0 to 0)	157.6 (143.3 to 172.5)	2 (-1.8 to 6)	367.5 (91.9 to 1901.7)	53.7 (-40 to 570)
		Male	8249 (6092 to 10933)	1999.4 (1489.5 to 2621.2)	0 (0 to 0)	0 (0 to 0)	20421 (14948 to 26893)	2043.4 (1500.1 to 2692.4)	0 (0 to 0)	0 (0 to 0)	147.6 (134.2 to 160.3)	2.2 (-1.5 to 5.7)	1232.3 (234.4 to 5309.6)	359.7 (12 to 1837.7)
	Schizophrenia	Both	1030 (750 to 1322)	159.3 (115.8 to 201.8)	--	--	3306 (2366 to 4278)	161.2 (116.7 to 205.2)	--	--	220.9 (191.2 to 254.5)	1.1 (-6.6 to 10.8)	--	--
		Female	471 (337 to 607)	151.5 (110.4 to 193.5)	--	--	1515 (1099 to 1987)	152.4 (112 to 196.5)	--	--	221.4 (181.9 to 270)	0.6 (-10.6 to 12.8)	--	--
		Male	559 (400 to 721)	166.5 (118.7 to 213.1)	--	--	1791 (1263 to 2312)	169.5 (120.8 to 214.6)	--	--	220.4 (181.2 to 266.2)	1.8 (-9.4 to 15.2)	--	--
	Depressive disorders	Both	6340 (4227 to 8855)	887.3 (605.5 to 1234.4)	--	--	18334 (12436 to 25901)	945.4 (648.9 to 1314)	--	--	189.2 (168.4 to 209.5)	6.6 (1.8 to 11.2)	--	--
		Female	3887 (2579 to 5415)	1112.8 (756.8 to 1561.7)	--	--	10972 (7379 to 15425)	1159.7 (788.8 to 1611.7)	--	--	182.3 (159.4 to 206.9)	4.2 (-1.5 to 10.3)	--	--
		Male	2453 (1648 to 3455)	677.3 (461.8 to 950)	--	--	7361 (5019 to 10472)	740.2 (510.3 to 1037.6)	--	--	200.1 (175.5 to 225.8)	9.3 (2.5 to 15.7)	--	--
	Bipolar disorder	Both	1258 (766 to 1958)	171.7 (106.3 to 262.4)	--	--	3435 (2110 to 5292)	174.1 (108 to 264.5)	--	--	172.9 (148.5 to 196.7)	1.4 (-5.4 to 8.5)	--	--
		Female	672 (405 to 1049)	185.6 (114.7 to 286.9)	--	--	1812 (1105 to 2773)	187.8 (116 to 284.1)	--	--	169.6 (137.3 to 204)	1.2 (-8.8 to 11.4)	--	--
		Male	587 (355 to 916)	158.4 (96.7 to 240.7)	--	--	1623 (991 to 2498)	161.2 (100 to 244.2)	--	--	176.7 (144.2 to 212.1)	1.8 (-7.2 to 12.6)	--	--
	Anxiety disorders	Both	5595 (3852 to 7742)	670.9 (471.4 to 920.4)	--	--	13508 (9322 to 18896)	693 (483.4 to 958.9)	--	--	141.4 (121.5 to 163.6)	3.3 (-2.7 to 9.2)	--	--
		Female	3308 (2282 to 4629)	826.2 (573.5 to 1133.3)	--	--	8151 (5608 to 11359)	852.2 (590.9 to 1173.8)	--	--	146.4 (122.2 to 174.7)	3.2 (-4.6 to 11.3)	--	--
		Male	2287 (1582 to 3188)	525 (366.2 to 722.1)	--	--	5356 (3684 to 7522)	540.6 (374.8 to 748.9)	--	--	134.2 (109.8 to 161.7)	3 (-5.5 to 11.9)	--	--
	Eating disorders	Both	373 (231 to 561)	40.6 (24.8 to 61.1)	0 (0 to 0)	0 (0 to 0)	1164 (719 to 1749)	52 (32.4 to 78.1)	0 (0 to 0)	0 (0 to 0)	212 (170.2 to 258.6)	27.9 (14.8 to 42.1)	488.2 (155.4 to 2105.8)	89.8 (-18.1 to 585.5)
		Female	229 (140 to 348)	50.5 (31.1 to 76.8)	0 (0 to 0)	0 (0 to 0)	701 (442 to 1048)	65.1 (41 to 97)	0 (0 to 0)	0 (0 to 0)	206.7 (163.1 to 264.8)	28.9 (13.2 to 48.3)	367.5 (91.9 to 1901.7)	53.7 (-40 to 570)

Province	Cause	Sex	1990				2019				1990-2019 percent change (%)			
			DALYs		Death		DALYs		Death		DALYs		Death	
			Number	Rate	Number	Rate	Number	Rate	Number	Rate	Number	Rate	Number	Rate
		Male	144 (87 to 222)	31 (18.6 to 48.2)	0 (0 to 0)	0 (0 to 0)	463 (272 to 727)	39.6 (23.8 to 60.7)	0 (0 to 0)	0 (0 to 0)	220.5 (160.2 to 285)	27.8 (7.8 to 50.1)	1232.3 (234.4 to 5309.6)	359.7 (12 to 1837.7)
	Autism spectrum disorders	Both	448 (290 to 647)	44.3 (29 to 63.8)	--	--	876 (578 to 1284)	44.7 (29.5 to 65.6)	--	--	95.3 (74.4 to 117.9)	0.8 (-9.6 to 11.6)	--	--
		Female	110 (71 to 159)	22.1 (14.4 to 32.5)	--	--	213 (138 to 316)	22.4 (14.6 to 33.1)	--	--	94.1 (61 to 131.4)	1.1 (-14.5 to 19)	--	--
		Male	339 (220 to 491)	65.3 (42.6 to 94.4)	--	--	662 (433 to 983)	66 (43.2 to 98.5)	--	--	95.6 (71.6 to 124.1)	1.1 (-11.2 to 14.2)	--	--
	Attention-deficit/hyperactivity disorder	Both	361 (204 to 624)	32.5 (18.3 to 56.2)	--	--	676 (382 to 1135)	33.7 (18.8 to 56.8)	--	--	87.3 (61.5 to 118.7)	3.6 (-10.2 to 18.2)	--	--
		Female	109 (59 to 189)	20 (11.2 to 34.7)	--	--	204 (114 to 337)	20.9 (11.6 to 34.7)	--	--	87.4 (55.6 to 129.8)	4.1 (-13.7 to 25.1)	--	--
		Male	252 (141 to 433)	44.4 (24.9 to 75.9)	--	--	472 (266 to 811)	45.8 (25.7 to 78.3)	--	--	87.3 (54.2 to 126.6)	3 (-14.6 to 23)	--	--
	Conduct disorder	Both	994 (568 to 1563)	75.5 (43 to 118.8)	--	--	1344 (771 to 2139)	74.3 (42.2 to 118.4)	--	--	35.3 (22.1 to 48.5)	-1.5 (-11 to 8.1)	--	--
		Female	340 (181 to 554)	52.6 (27.9 to 86.2)	--	--	449 (237 to 753)	51 (26.8 to 85.4)	--	--	32.2 (13.9 to 55)	-2.9 (-16.4 to 13.7)	--	--
		Male	654 (388 to 1009)	97.5 (57.7 to 150.7)	--	--	895 (516 to 1421)	96.4 (55.7 to 152.2)	--	--	36.8 (21.2 to 53.8)	-1.1 (-12.5 to 11.1)	--	--
	Idiopathic developmental intellectual disability	Both	1079 (597 to 1703)	104.9 (58.8 to 165)	--	--	1206 (613 to 2020)	60.9 (31.1 to 102.2)	--	--	11.8 (-5.8 to 23.8)	-41.9 (-51.4 to -35.9)	--	--
		Female	467 (263 to 741)	93.1 (53.2 to 147.5)	--	--	536 (276 to 893)	55.7 (28.6 to 93)	--	--	14.9 (-3.3 to 29.1)	-40.2 (-49.5 to -32.9)	--	--
		Male	612 (344 to 973)	116.1 (66.1 to 183.5)	--	--	670 (335 to 1116)	65.9 (33.1 to 109.7)	--	--	9.4 (-9.6 to 23)	-43.2 (-53.3 to -36.3)	--	--
	Other mental disorders	Both	589 (374 to 889)	101.1 (64.6 to 153.1)	--	--	1867 (1183 to 2839)	100.8 (65 to 151.9)	--	--	216.7 (194.9 to 240.6)	-0.3 (-5.7 to 5.5)	--	--
		Female	228 (144 to 349)	82.6 (53 to 125.7)	--	--	739 (465 to 1144)	82.7 (52.9 to 126.7)	--	--	224.8 (191.8 to 262)	0.1 (-8.1 to 9.5)	--	--
		Male	362 (227 to 547)	118 (75 to 178.6)	--	--	1127 (718 to 1702)	118.2 (76.1 to 178.8)	--	--	211.7 (183.8 to 241.3)	0.2 (-6.9 to 7.6)	--	--
	Substance use disorders	Both	3054 (2233 to 4001)	396.5 (293.8 to 510.1)	15 (7 to 20)	2.7 (1.5 to 3.5)	10253 (7799 to 12940)	477.9 (364.1 to 602.7)	62 (50 to 75)	3.2 (2.6 to 3.9)	235.7 (191.4 to 297.9)	20.5 (5.3 to 42.5)	304.5 (184.9 to 792.2)	19.5 (-14.8 to 119.8)
		Female	956 (674 to 1266)	249.1 (180.1 to 320.1)	5 (2 to 7)	1.7 (0.9 to 2.3)	2514 (1831 to 3344)	242.6 (176.6 to 320)	9 (7 to 12)	1.1 (0.8 to 1.3)	163 (122.6 to 221.5)	-2.6 (-17.4 to 18)	95.2 (20.9 to 405.5)	-36.1 (-58 to 26.1)
		Male	2098 (1514 to 2794)	535.8 (398.3 to 697.8)	11 (5 to 15)	3.6 (2.1 to 5)	7739 (5970 to 9693)	701.2 (546.1 to 876.9)	53 (42 to 66)	5.3 (4.2 to 6.5)	268.9 (210.2 to 351.1)	30.9 (10.9 to 58.2)	395 (224.4 to 935.9)	45.5 (-3.5 to 158)

Province	Cause	Sex	1990				2019				1990-2019 percent change (%)			
			DALYs		Death		DALYs		Death		DALYs		Death	
			Number	Rate	Number	Rate	Number	Rate	Number	Rate	Number	Rate	Number	Rate
	Alcohol use disorders	Both	441 (309 to 578)	63.9 (46.1 to 82.8)	3 (2 to 4)	0.6 (0.3 to 0.8)	1105 (810 to 1436)	52.2 (38.8 to 66.8)	8 (5 to 11)	0.5 (0.3 to 0.6)	150.8 (109.8 to 224)	-18.4 (-31.7 to 4.1)	162.2 (69.6 to 447.9)	-21.4 (-47.7 to 40)
		Female	105 (72 to 143)	29.5 (20.8 to 39.6)	1 (0 to 1)	0.2 (0.1 to 0.3)	256 (173 to 356)	25.2 (17.5 to 34.7)	1 (1 to 1)	0.1 (0.1 to 0.1)	143.9 (95.7 to 213.3)	-14.6 (-29.4 to 6.6)	50.8 (0.3 to 292.2)	-45.4 (-62 to 3.3)
		Male	336 (233 to 444)	95.9 (67.4 to 125.2)	3 (1 to 4)	0.9 (0.5 to 1.3)	850 (615 to 1093)	77.7 (57 to 98.7)	8 (4 to 10)	0.8 (0.5 to 1)	153 (102.9 to 238.4)	-19 (-35 to 8.6)	186.7 (75.8 to 478.5)	-14.6 (-46.6 to 61.5)
	Drug use disorders	Both	2613 (1889 to 3534)	332.6 (245.9 to 436.4)	12 (5 to 16)	2.1 (1.1 to 2.8)	9148 (6871 to 11693)	425.7 (321.6 to 541.9)	54 (43 to 66)	2.8 (2.3 to 3.3)	250 (201.6 to 316.8)	28 (10.9 to 51.2)	342.3 (203.9 to 879.9)	30.7 (-8.6 to 142.7)
		Female	851 (585 to 1145)	219.6 (155.7 to 287.8)	4 (1 to 6)	1.5 (0.8 to 2.1)	2259 (1632 to 3061)	217.4 (159 to 292.8)	8 (6 to 11)	1 (0.8 to 1.2)	165.3 (122.7 to 228.1)	-1 (-17 to 21.1)	101.6 (22.1 to 422.8)	-34.7 (-57.5 to 28.5)
		Male	1762 (1256 to 2425)	439.9 (321.3 to 589.8)	8 (4 to 11)	2.7 (1.5 to 3.8)	6889 (5249 to 8720)	623.5 (475.7 to 786.6)	45 (35 to 57)	4.5 (3.6 to 5.6)	291 (222.2 to 381.5)	41.7 (17.9 to 72.7)	463 (259.9 to 1094.7)	65.7 (7.3 to 202.5)
	Diabetes and kidney diseases	Both	6654 (4926 to 7767)	1271.8 (1019.5 to 1524.1)	147 (103 to 181)	42.5 (33 to 56.4)	18258 (15368 to 21455)	1422.3 (1209.5 to 1659.1)	403 (352 to 450)	39.5 (34.3 to 44)	174.4 (129.8 to 271.8)	11.8 (-5.9 to 41.8)	172.9 (113.8 to 283.9)	-7 (-30.4 to 20.2)
		Female	3103 (2220 to 3832)	1210.2 (952.4 to 1526)	65 (46 to 90)	38.3 (28.9 to 59.4)	8816 (7367 to 10466)	1403.3 (1179.8 to 1661.1)	192 (162 to 220)	40.1 (34 to 45.8)	184.1 (127.4 to 300.7)	16 (-7.9 to 47.2)	194.2 (108.9 to 322.3)	4.6 (-32 to 42.4)
		Male	3551 (2613 to 4279)	1328.9 (1017.8 to 1626.3)	82 (56 to 105)	47.2 (34.8 to 62.1)	9441 (7877 to 11154)	1448.4 (1214.3 to 1698.9)	211 (174 to 250)	39.7 (32.7 to 47.2)	165.9 (111.3 to 261.1)	9 (-13.2 to 42.4)	156.1 (83.2 to 278.6)	-15.8 (-40.8 to 19.4)
	Diabetes mellitus	Both	1877 (1455 to 2368)	423 (331.6 to 536)	28 (20 to 38)	7.8 (6 to 11)	10144 (7974 to 12536)	798.8 (638.9 to 980.4)	146 (126 to 166)	14.2 (12.2 to 16.1)	440.5 (368.4 to 533.8)	88.9 (60.5 to 116)	427.1 (268.9 to 653.3)	81.7 (22.4 to 141.4)
		Female	841 (642 to 1089)	406.8 (313.2 to 534.7)	12 (9 to 19)	7.4 (5.4 to 12.6)	5014 (3950 to 6324)	799.4 (635 to 1001)	72 (59 to 85)	14.6 (12.1 to 17.3)	496 (370 to 610.6)	96.5 (53.2 to 132.8)	479.3 (244.2 to 762.1)	96.1 (8.8 to 183.2)
		Male	1035 (791 to 1306)	437.4 (337.4 to 548.3)	15 (11 to 20)	8.2 (6 to 10.5)	5130 (4027 to 6369)	800.6 (633.2 to 991.2)	74 (60 to 90)	14.1 (11.3 to 17)	395.5 (324.2 to 486.5)	83 (57.7 to 115.1)	384.7 (244 to 635.5)	71.4 (22.8 to 145)
	Chronic kidney disease	Both	4752 (3352 to 5615)	846.2 (632.6 to 1037.4)	119 (82 to 149)	34.6 (26.7 to 48.1)	8041 (7032 to 9101)	619 (546.1 to 697.7)	255 (221 to 288)	25.2 (21.6 to 28.6)	69.2 (37.3 to 141.6)	-26.8 (-41.2 to -0.6)	113.3 (63.3 to 204.3)	-27.3 (-48.7 to -4.2)
		Female	2249 (1481 to 2841)	800.7 (589.7 to 1084.9)	53 (36 to 77)	30.8 (22.7 to 52.1)	3767 (3206 to 4379)	599.3 (512.7 to 689.8)	119 (99 to 138)	25.3 (21 to 29.2)	67.5 (28 to 153.2)	-25.2 (-44.7 to 3.1)	126.2 (53.5 to 227.7)	-17.8 (-52.4 to 13.4)
		Male	2503 (1744 to 3113)	888.9 (638.5 to 1131)	67 (45 to 87)	38.9 (28.6 to 52.6)	4274 (3615 to 5040)	643.5 (537.9 to 765.2)	136 (110 to 165)	25.5 (20.6 to 31.1)	70.8 (29.9 to 148.6)	-27.6 (-45.7 to 2.8)	103.1 (43.9 to 205.6)	-34.5 (-55.7 to -5.8)
	Acute glomerulonephritis	Both	26 (11 to 110)	2.6 (1.3 to 9.7)	0 (0 to 2)	0.1 (0 to 0.3)	73 (38 to 101)	4.4 (2.4 to 6)	2 (1 to 3)	0.2 (0.1 to 0.2)	184.2 (-63.8 to 667.1)	69.4 (-74.4 to 286.4)	340.9 (-40.5 to 930.5)	106.7 (-66.9 to 326.1)
		Female	13 (5 to 30)	2.6 (1.2 to 5.2)	0 (0 to 0)	0.1 (0 to 0.1)	36 (13 to 53)	4.6 (1.8 to 6.6)	1 (0 to 1)	0.2 (0.1 to 0.3)	175.4 (-42 to 797.3)	77.4 (-57.4 to 396.4)	374.7 (-4.4 to 1266.9)	153.5 (-54.7 to 543.9)
		Male	13 (4 to 84)	2.6 (0.9 to 14.6)	0 (0 to 1)	0.1 (0 to 0.4)	37 (16 to 61)	4.3 (1.9 to 6.9)	1 (0 to 1)	0.1 (0.1 to 0.2)	193.1 (-78.8 to 1429.8)	63.5 (-84.6 to 606.5)	309.5 (-69.8 to 1688.6)	71.5 (-84.6 to 532)

Province	Cause	Sex	1990				2019				1990-2019 percent change (%)			
			DALYs		Death		DALYs		Death		DALYs		Death	
			Number	Rate	Number	Rate	Number	Rate	Number	Rate	Number	Rate	Number	Rate
	Skin and subcutaneous diseases	Both	3942 (2633 to 5756)	409.6 (272.5 to 596.6)	1 (0 to 1)	0.2 (0.2 to 0.5)	7833 (5171 to 11503)	419.8 (278.3 to 617.6)	3 (2 to 4)	0.3 (0.2 to 0.4)	98.7 (91 to 105.7)	2.5 (-0.7 to 5.2)	331.1 (78.7 to 637.9)	25.8 (-50.7 to 125.7)
		Female	2050 (1371 to 2969)	433.9 (291 to 636.9)	0 (0 to 1)	0.2 (0.1 to 0.6)	4046 (2658 to 5931)	442.3 (292.3 to 649.5)	1 (1 to 1)	0.3 (0.2 to 0.3)	97.4 (87.8 to 105.3)	1.9 (-2.1 to 5.4)	275.5 (34.6 to 614.7)	20.2 (-62.4 to 149.1)
		Male	1892 (1259 to 2789)	386.8 (257 to 562.2)	0 (0 to 1)	0.3 (0.2 to 0.7)	3787 (2479 to 5570)	398.3 (262.8 to 587)	2 (1 to 3)	0.4 (0.2 to 0.5)	100.1 (91.4 to 108.3)	3 (-1.2 to 6.5)	372.3 (94.4 to 851.4)	27.5 (-49.6 to 168.7)
	Dermatitis	Both	918 (541 to 1461)	93.3 (56.2 to 144.1)	--	--	1818 (1108 to 2836)	94.1 (57.1 to 145.2)	--	--	98 (84.4 to 114.7)	0.9 (-3.7 to 5.8)	--	--
		Female	499 (292 to 798)	103.6 (62.5 to 161.1)	--	--	988 (593 to 1544)	104.5 (63.2 to 162.7)	--	--	98 (81.7 to 118.1)	0.9 (-5.5 to 7.8)	--	--
		Male	419 (244 to 658)	83.5 (50.5 to 127)	--	--	830 (504 to 1291)	84.1 (51.2 to 129.5)	--	--	98 (80.2 to 119.4)	0.7 (-6.4 to 8.4)	--	--
	Psoriasis	Both	336 (236 to 457)	46.4 (32.8 to 61.8)	--	--	603 (418 to 810)	32.7 (23 to 43.6)	--	--	79.1 (57.6 to 102.7)	-29.4 (-36.1 to -21.8)	--	--
		Female	171 (120 to 233)	48.1 (33.9 to 64.4)	--	--	306 (213 to 422)	34 (23.7 to 46.4)	--	--	78.7 (49 to 113.6)	-29.4 (-39.7 to -17.4)	--	--
		Male	165 (114 to 224)	44.7 (31.6 to 59.9)	--	--	297 (200 to 401)	31.6 (21.6 to 42.2)	--	--	79.6 (50.3 to 112.9)	-29.4 (-39.1 to -18.3)	--	--
	Bacterial skin diseases	Both	60 (38 to 97)	7.9 (5.2 to 11.8)	1 (0 to 1)	0.2 (0.1 to 0.4)	131 (90 to 198)	7.8 (5.6 to 11.4)	2 (2 to 2)	0.2 (0.1 to 0.2)	119.9 (67 to 170.2)	-0.8 (-25.3 to 27.5)	194.8 (78.3 to 415.8)	-15.6 (-46 to 65.3)
		Female	26 (16 to 43)	6.9 (4.4 to 11.4)	0 (0 to 1)	0.2 (0.1 to 0.3)	52 (33 to 84)	6.3 (4.2 to 9.7)	1 (0 to 1)	0.1 (0.1 to 0.2)	99.7 (64.4 to 147.1)	-8.3 (-33.5 to 31.2)	152.3 (36.5 to 511.4)	-22.4 (-60.8 to 139.9)
		Male	34 (21 to 55)	8.8 (5.7 to 14.3)	0 (0 to 1)	0.2 (0.1 to 0.5)	79 (54 to 117)	9.3 (6.6 to 13.3)	1 (1 to 2)	0.2 (0.2 to 0.3)	135.3 (62.5 to 210.9)	5.4 (-33.1 to 47.3)	224.7 (78.5 to 534.5)	-12.1 (-49.8 to 86.2)
	Scabies	Both	255 (139 to 411)	25.3 (13.9 to 40.8)	--	--	489 (267 to 799)	25.1 (13.8 to 40.7)	--	--	91.9 (78.3 to 106.8)	-0.9 (-5.4 to 3.6)	--	--
		Female	125 (69 to 203)	25.4 (13.9 to 40.8)	--	--	239 (130 to 387)	25.2 (13.9 to 40.8)	--	--	90.7 (74.7 to 108.3)	-0.7 (-7 to 5.8)	--	--
		Male	129 (71 to 209)	25.3 (14.1 to 40.7)	--	--	249 (135 to 412)	25 (13.7 to 41.3)	--	--	93 (75.8 to 112.2)	-1.1 (-7.1 to 5.2)	--	--
	Fungal skin diseases	Both	129 (52 to 272)	19.3 (7.8 to 41.2)	--	--	299 (120 to 640)	18.6 (7.5 to 39.4)	--	--	131.2 (113.9 to 146.5)	-3.5 (-5.6 to -1.6)	--	--
		Female	58 (23 to 122)	18 (7.3 to 38.6)	--	--	136 (55 to 295)	17.4 (7 to 37.9)	--	--	135.7 (117.5 to 152.8)	-3.4 (-6.3 to -0.7)	--	--
		Male	72 (28 to 153)	20.6 (8.3 to 43.8)	--	--	163 (65 to 351)	19.8 (8 to 42.2)	--	--	127.6 (109.9 to 143.2)	-3.7 (-6.4 to -1.1)	--	--
	Viral skin diseases	Both	643 (406 to 967)	51.2 (32.8 to 76.9)	--	--	1005 (643 to 1504)	51.4 (32.9 to 76.8)	--	--	56.1 (49.5 to 63.7)	0.4 (-3.6 to 4.5)	--	--
		Female	312 (198 to 473)	50.8 (32.5 to 76.8)	--	--	486 (307 to 729)	51 (32.3 to 76.5)	--	--	55.5 (46.2 to 66.5)	0.4 (-5.2 to 6.9)	--	--
		Male	331 (210 to 494)	51.6 (32.5 to 77.1)	--	--	519 (327 to 776)	51.8 (32.8 to 77.4)	--	--	56.7 (47 to 67.2)	0.4 (-5.3 to 6.4)	--	--

Province	Cause	Sex	1990				2019				1990-2019 percent change (%)			
			DALYs		Death		DALYs		Death		DALYs		Death	
			Number	Rate	Number	Rate	Number	Rate	Number	Rate	Number	Rate	Number	Rate
	Acne vulgaris	Both	547 (328 to 867)	49.5 (29.7 to 78.4)	--	--	1284 (765 to 2044)	69.2 (41 to 110.6)	--	--	134.8 (115.6 to 155)	39.9 (28.5 to 51.5)	--	--
		Female	260 (155 to 417)	48 (28.9 to 76.7)	--	--	610 (361 to 962)	67.2 (39.6 to 106.4)	--	--	134.4 (107.6 to 165.7)	40 (23.6 to 58.3)	--	--
		Male	287 (169 to 453)	50.9 (30.2 to 80.5)	--	--	674 (400 to 1086)	71.2 (42.1 to 115)	--	--	135.2 (107.5 to 164.9)	39.8 (24 to 57.1)	--	--
	Alopecia areata	Both	50 (32 to 75)	6.3 (4 to 9.3)	--	--	130 (82 to 196)	6.4 (4 to 9.6)	--	--	160.3 (130.9 to 191.6)	1.1 (-9.1 to 12.1)	--	--
		Female	32 (20 to 48)	8.3 (5.3 to 12.3)	--	--	83 (53 to 125)	8.3 (5.3 to 12.5)	--	--	161 (125.4 to 200.9)	0.7 (-12.2 to 14.7)	--	--
		Male	18 (11 to 28)	4.5 (2.8 to 6.7)	--	--	47 (29 to 72)	4.5 (2.8 to 6.8)	--	--	159.2 (110.9 to 221.2)	0.6 (-15.7 to 21.3)	--	--
	Pruritus	Both	75 (35 to 134)	10 (4.8 to 18)	--	--	188 (88 to 339)	10.5 (5 to 18.8)	--	--	151.9 (133.7 to 173.9)	5.6 (0.2 to 11.1)	--	--
		Female	40 (18 to 73)	11 (5.2 to 20)	--	--	100 (47 to 182)	11.5 (5.5 to 21)	--	--	153.7 (131.5 to 180.9)	5 (-2.2 to 12.3)	--	--
		Male	35 (17 to 63)	9.1 (4.3 to 16.2)	--	--	88 (41 to 156)	9.6 (4.5 to 17.1)	--	--	149.9 (126.7 to 176.5)	5.7 (-1.9 to 13.5)	--	--
	Urticaria	Both	637 (416 to 933)	58 (37.9 to 83.6)	--	--	1138 (744 to 1656)	58.2 (38.4 to 84.2)	--	--	78.5 (64.5 to 94)	0.3 (-5.1 to 6.2)	--	--
		Female	385 (251 to 562)	70.9 (46.9 to 102.5)	--	--	677 (442 to 983)	71 (46.5 to 103.2)	--	--	75.7 (59 to 93.1)	0.2 (-6.8 to 7.6)	--	--
		Male	252 (161 to 372)	45.8 (29.2 to 66)	--	--	461 (298 to 666)	45.9 (29.7 to 66.4)	--	--	82.7 (64.1 to 103.8)	0.3 (-8.1 to 9.3)	--	--
	Decubitus ulcer	Both	3 (2 to 6)	0.9 (0.6 to 2.2)	0 (0 to 0)	0 (0 to 0.1)	26 (12 to 33)	2.4 (1.2 to 3)	1 (0 to 2)	0.1 (0.1 to 0.2)	733.6 (105.2 to 1245.4)	166.1 (-39.7 to 345.5)	1635 (70.1 to 4750.8)	285.8 (-59.6 to 1001.7)
		Female	1 (1 to 3)	0.8 (0.5 to 2.3)	0 (0 to 0)	0 (0 to 0.2)	9 (5 to 13)	2 (1 to 2.6)	0 (0 to 1)	0.1 (0 to 0.2)	604.6 (73.5 to 1220.8)	136.6 (-49.5 to 365.3)	1191 (20.8 to 5048.3)	253.4 (-66.9 to 1301.9)
		Male	2 (1 to 4)	1 (0.6 to 2.5)	0 (0 to 0)	0 (0 to 0.2)	16 (6 to 22)	2.8 (1.1 to 3.7)	1 (0 to 1)	0.2 (0 to 0.2)	832.5 (111 to 1426)	189.8 (-40.9 to 394)	2127.8 (111.6 to 6582.4)	300.4 (-59 to 1130.9)
	Other skin and subcutaneous diseases	Both	288 (138 to 531)	41.7 (20.1 to 76.1)	0 (0 to 0)	0 (0 to 0)	724 (344 to 1309)	43.4 (20.8 to 78.2)	0 (0 to 0)	0 (0 to 0)	150.9 (143.4 to 158.6)	4.1 (1.6 to 6.4)	150.9 (11.1 to 583.7)	-44 (-75.6 to 72.3)
		Female	140 (67 to 261)	42.2 (20.3 to 77.8)	0 (0 to 0)	0 (0 to 0)	360 (171 to 655)	43.9 (20.9 to 79.7)	0 (0 to 0)	0 (0 to 0)	156.8 (146.7 to 167.3)	4.2 (0.6 to 7.6)	174 (-15.9 to 1126.5)	-30.4 (-78.3 to 235.6)

Province	Cause	Sex	1990				2019				1990-2019 percent change (%)			
			DALYs		Death		DALYs		Death		DALYs		Death	
			Number	Rate	Number	Rate	Number	Rate	Number	Rate	Number	Rate	Number	Rate
		Male	148 (70 to 271)	41.2 (19.8 to 74.9)	0 (0 to 0)	0 (0 to 0)	364 (171 to 662)	42.8 (20.4 to 77.5)	0 (0 to 0)	0 (0 to 0)	145.4 (135.4 to 155.8)	3.9 (0.4 to 7.2)	125.8 (9.6 to 457.2)	-54.6 (-79.2 to 19)
	Sense organ diseases	Both	5128 (3630 to 6935)	1073.4 (775.9 to 1428.8)	--	--	10691 (7450 to 14708)	818.6 (580.3 to 1111.6)	--	--	108.5 (97 to 120.3)	-23.7 (-26.9 to -20.2)	--	--
		Female	2433 (1736 to 3262)	1076.4 (780.8 to 1422.1)	--	--	5252 (3680 to 7218)	819.8 (583.5 to 1108.2)	--	--	115.8 (103.4 to 128.7)	-23.8 (-27.2 to -20.2)	--	--
		Male	2694 (1912 to 3628)	1069.2 (766.7 to 1443.3)	--	--	5439 (3798 to 7480)	817.6 (578.3 to 1114.8)	--	--	101.9 (89.7 to 113.9)	-23.5 (-27.1 to -19.7)	--	--
	Blindness and vision loss	Both	2433 (1734 to 3297)	532.6 (380.2 to 709)	--	--	4732 (3349 to 6411)	378.3 (268.7 to 511.2)	--	--	94.5 (85.4 to 103.6)	-29 (-32.1 to -25.5)	--	--
		Female	1215 (867 to 1637)	561.8 (402.1 to 750.8)	--	--	2469 (1739 to 3355)	399.6 (285.4 to 537.9)	--	--	103.2 (91.9 to 113.5)	-28.9 (-32.4 to -25)	--	--
		Male	1218 (865 to 1650)	506.1 (363.5 to 675.5)	--	--	2264 (1601 to 3080)	356.6 (253.2 to 480.5)	--	--	85.9 (75.6 to 97)	-29.5 (-33.2 to -25.6)	--	--
	Age-related and other hearing loss	Both	2446 (1695 to 3379)	499.6 (356 to 690)	--	--	5291 (3655 to 7397)	397.1 (277.1 to 557.4)	--	--	116.3 (96.9 to 135.9)	-20.5 (-24.4 to -16.5)	--	--
		Female	1090 (750 to 1506)	469.8 (333.2 to 650.3)	--	--	2428 (1677 to 3398)	373.5 (261 to 520.7)	--	--	122.8 (98.9 to 145.7)	-20.5 (-25.4 to -15.5)	--	--
		Male	1356 (940 to 1876)	525.2 (373.1 to 722.2)	--	--	2863 (1965 to 4011)	421.2 (291 to 589.4)	--	--	111.1 (89.6 to 130.5)	-19.8 (-24.6 to -15)	--	--
	Other sense organ diseases	Both	249 (150 to 382)	41.2 (24.5 to 64.3)	--	--	667 (400 to 1027)	43.2 (25.8 to 66.4)	--	--	167.7 (145 to 191.4)	4.9 (0 to 10.4)	--	--
		Female	129 (77 to 198)	44.8 (26.4 to 70)	--	--	355 (213 to 545)	46.7 (27.8 to 72.1)	--	--	175.8 (148.2 to 206.9)	4.2 (-2.4 to 11.7)	--	--
		Male	121 (71 to 186)	37.9 (22.2 to 58.2)	--	--	313 (186 to 477)	39.7 (23.8 to 61.1)	--	--	159 (130.6 to 190.9)	4.9 (-2.1 to 13.1)	--	--
	Musculoskeletal disorders	Both	10361 (7390 to 13898)	1852.2 (1330.2 to 2451.3)	3 (2 to 4)	0.7 (0.4 to 0.9)	33255 (23762 to 44109)	1950.6 (1409 to 2570.5)	15 (8 to 19)	1.2 (0.5 to 1.4)	221 (207.2 to 236.3)	5.3 (1.7 to 9)	431.2 (208.6 to 866.6)	69.5 (-0.1 to 150.9)
		Female	5352 (3837 to 7133)	2021.2 (1450.6 to 2660.9)	2 (1 to 3)	0.8 (0.4 to 1.1)	18027 (13055 to 23788)	2169.3 (1580.3 to 2869.2)	9 (4 to 12)	1.4 (0.5 to 1.9)	236.8 (219.6 to 254.2)	7.3 (3.3 to 11.4)	444.7 (164.1 to 986.1)	82.2 (-17.9 to 226.8)
		Male	5009 (3535 to 6727)	1699.6 (1212.3 to 2277.5)	1 (1 to 1)	0.6 (0.4 to 0.7)	15228 (10644 to 20322)	1737.9 (1239.3 to 2301)	6 (3 to 7)	0.9 (0.4 to 1.1)	204 (186.7 to 223.8)	2.3 (-2.4 to 7.1)	410.5 (138 to 743.5)	54.7 (-22.1 to 132.1)
	Rheumatoid arthritis	Both	61 (43 to 83)	11.1 (7.8 to 14.9)	0 (0 to 0)	0.1 (0 to 0.1)	233 (167 to 314)	14 (10 to 18.6)	1 (1 to 2)	0.1 (0.1 to 0.2)	281.2 (216.6 to 347)	25.9 (2.3 to 50.3)	496.7 (81.5 to 1189)	80.9 (-44.1 to 307.8)
		Female	42 (28 to 57)	15.9 (10.9 to 21.9)	0 (0 to 0)	0.1 (0 to 0.2)	161 (110 to 221)	19.4 (13.2 to 26.4)	1 (0 to 1)	0.2 (0 to 0.2)	286.1 (195.3 to 387.2)	22 (-9.8 to 55.7)	426.6 (-27.9 to 1586.4)	58.2 (-77.6 to 418.8)
		Male	19 (13 to 27)	6.8 (4.7 to 9.4)	0 (0 to 0)	0 (0 to 0.1)	72 (52 to 95)	8.7 (6.4 to 11.4)	0 (0 to 1)	0.1 (0.1 to 0.1)	270.8 (212.1 to 349.3)	27.9 (7.3 to 57.1)	683.4 (367.8 to 1371.2)	147.4 (45.3 to 343.4)

Province	Cause	Sex	1990				2019				1990-2019 percent change (%)			
			DALYs		Death		DALYs		Death		DALYs		Death	
			Number	Rate	Number	Rate	Number	Rate	Number	Rate	Number	Rate	Number	Rate
	Osteoarthritis	Both	697 (352 to 1383)	174.2 (87.8 to 344.9)	--	--	2325 (1178 to 4583)	190.1 (96.4 to 377.9)	--	--	233.7 (221.7 to 246.7)	9.2 (5.7 to 12.8)	--	--
		Female	361 (181 to 705)	198.5 (99.9 to 388.9)	--	--	1318 (666 to 2619)	216.1 (109.6 to 427.2)	--	--	264.9 (248 to 282.8)	8.9 (4.2 to 13.6)	--	--
		Male	336 (168 to 651)	152.8 (76.7 to 297.9)	--	--	1007 (504 to 1984)	163.8 (81.9 to 328.4)	--	--	200 (181.6 to 219.7)	7.2 (2 to 13.1)	--	--
	Low back pain	Both	6048 (4233 to 8099)	1016 (713.1 to 1368.2)	--	--	15984 (10981 to 21426)	915.6 (644.5 to 1223.3)	--	--	164.3 (147.8 to 182.9)	-9.9 (-13.7 to -5.6)	--	--
		Female	2745 (1915 to 3717)	963.2 (679.8 to 1294.6)	--	--	7665 (5262 to 10317)	907.7 (640.5 to 1211.6)	--	--	179.2 (158.2 to 201.6)	-5.8 (-11.6 to 0.3)	--	--
		Male	3303 (2314 to 4454)	1061.5 (736.1 to 1438.8)	--	--	8319 (5626 to 11297)	923.6 (639.3 to 1236.8)	--	--	151.9 (132.5 to 173)	-13 (-18.1 to -7.1)	--	--
	Neck pain	Both	2183 (1425 to 3191)	400.4 (264.2 to 579.9)	--	--	7061 (4657 to 10374)	404.8 (268.6 to 586.1)	--	--	223.4 (203.6 to 242.9)	1.1 (-1.2 to 3.2)	--	--
		Female	1266 (830 to 1855)	492.5 (326.6 to 719.6)	--	--	4222 (2778 to 6142)	493.9 (327.7 to 715)	--	--	233.6 (213.5 to 252.3)	0.3 (-2.4 to 2.9)	--	--
		Male	918 (598 to 1337)	318 (208.1 to 463.6)	--	--	2839 (1825 to 4156)	318.7 (208.5 to 465.5)	--	--	209.4 (185.7 to 233.7)	0.2 (-3.3 to 3.7)	--	--
	Gout	Both	63 (39 to 93)	14.3 (9 to 21.1)	--	--	213 (132 to 311)	15.3 (9.6 to 22.2)	--	--	239.3 (189.6 to 296.4)	6.9 (-6.9 to 21.5)	--	--
		Female	12 (7 to 19)	6.1 (3.7 to 9.2)	--	--	47 (29 to 70)	7 (4.3 to 10.2)	--	--	286.2 (203.4 to 388)	14.1 (-10.2 to 41.2)	--	--
		Male	51 (31 to 76)	21.8 (13.5 to 32.1)	--	--	166 (102 to 241)	23.6 (14.7 to 33.9)	--	--	227.8 (171.1 to 295.7)	8.3 (-7.8 to 25.7)	--	--
	Other musculoskeletal disorders	Both	1309 (865 to 1842)	236.3 (156.6 to 339.6)	3 (1 to 4)	0.6 (0.4 to 0.8)	7438 (5179 to 10350)	410.7 (282.2 to 562.5)	14 (7 to 17)	1 (0.5 to 1.3)	468.1 (406.9 to 547.3)	73.8 (57.1 to 97.4)	425.7 (209.2 to 868.5)	68.2 (1.8 to 146.9)
		Female	926 (631 to 1281)	344.9 (235.4 to 474.8)	2 (1 to 2)	0.7 (0.4 to 1)	4614 (3242 to 6392)	525.2 (369.7 to 714.5)	9 (4 to 11)	1.3 (0.5 to 1.7)	398.4 (341.1 to 466.9)	52.3 (36.3 to 70.9)	446.6 (189.8 to 1064.8)	85.8 (-3.2 to 233.1)
		Male	383 (221 to 589)	138.7 (81.2 to 212.8)	1 (1 to 1)	0.5 (0.4 to 0.7)	2824 (1867 to 4037)	299.5 (194.3 to 429.2)	5 (2 to 7)	0.8 (0.3 to 1)	636.7 (497.5 to 894.2)	115.9 (77.9 to 180.1)	395 (115.7 to 724.3)	48.9 (-30.2 to 123.5)
	Other non-communicable diseases	Both	54953 (34145 to 76718)	4182.5 (2792.7 to 5614.6)	515 (286 to 774)	33.5 (19.5 to 49.3)	38567 (29866 to 48890)	2064.2 (1600.7 to 2597.6)	203 (158 to 257)	12 (9.4 to 15)	-29.8 (-53.8 to 20.4)	-50.6 (-65 to -24.9)	-60.7 (-76.1 to -17.8)	-64.1 (-78.2 to -29.1)

Province	Cause	Sex	1990				2019				1990-2019 percent change (%)			
			DALYs		Death		DALYs		Death		DALYs		Death	
			Number	Rate	Number	Rate	Number	Rate	Number	Rate	Number	Rate	Number	Rate
		Female	27491 (17469 to 38935)	4678.8 (3244.3 to 6237.5)	234 (126 to 360)	31.5 (17.8 to 47.9)	24557 (18526 to 31739)	2618.3 (1984.7 to 3399.8)	92 (70 to 120)	11.5 (8.7 to 14.6)	-10.7 (-42.4 to 46.5)	-44 (-60 to -20.7)	-60.6 (-77.9 to -15.9)	-63.6 (-79.9 to -26.9)
		Male	27463 (15265 to 40406)	3723.4 (2228.6 to 5274.7)	281 (144 to 423)	35.4 (18.8 to 53.1)	14010 (10875 to 18004)	1542.9 (1196 to 1974.3)	110 (84 to 146)	12.6 (9.6 to 16.3)	-49 (-67.9 to 5.3)	-58.6 (-72.7 to -23.1)	-60.8 (-77 to -8.1)	-64.4 (-79 to -21.9)
	Congenital birth defects	Both	40945 (21897 to 63313)	2525.1 (1368.8 to 3882.9)	451 (235 to 706)	27.7 (14.4 to 43.5)	13289 (9987 to 17794)	700.5 (526.1 to 942.1)	133 (97 to 183)	7 (5.1 to 9.7)	-67.5 (-81.3 to -28.6)	-72.3 (-83.9 to -39.5)	-70.6 (-83.4 to -31.3)	-74.6 (-85.7 to -41.1)
		Female	18328 (9578 to 29835)	2314.8 (1217.6 to 3743)	203 (102 to 331)	25.5 (12.9 to 41.4)	6005 (4395 to 8217)	650.1 (475.7 to 891.4)	61 (43 to 86)	6.6 (4.6 to 9.3)	-67.2 (-81.8 to -24.1)	-71.9 (-84.3 to -35.3)	-70.1 (-84.1 to -26.2)	-74.1 (-86.3 to -36.4)
		Male	22617 (10977 to 35563)	2725.2 (1330.6 to 4258.7)	248 (117 to 395)	29.7 (14 to 47.1)	7284 (5400 to 10436)	748.4 (553.6 to 1073.7)	72 (51 to 107)	7.4 (5.3 to 11.1)	-67.8 (-81.4 to -12.9)	-72.5 (-84.2 to -26.4)	-71 (-83.9 to -15.1)	-75 (-86.1 to -28)
	Urinary diseases and male infertility	Both	194 (141 to 265)	30 (21.2 to 41.8)	1 (1 to 3)	0.3 (0.2 to 0.9)	595 (404 to 801)	39.7 (27.1 to 51.3)	9 (4 to 11)	0.9 (0.4 to 1.1)	207.1 (116.8 to 269.4)	32.6 (-14.9 to 61)	593.1 (114.9 to 1151.2)	175.7 (-30.3 to 379.2)
		Female	67 (50 to 88)	17.7 (12.7 to 27.5)	1 (0 to 1)	0.4 (0.2 to 0.8)	188 (117 to 235)	25.2 (15.8 to 31)	4 (2 to 5)	0.9 (0.5 to 1.1)	179.7 (73.2 to 280.6)	42.6 (-28.6 to 100.2)	459.4 (96.8 to 959.6)	130.8 (-26.3 to 342.8)
		Male	127 (87 to 184)	41 (27.6 to 59.5)	1 (0 to 1)	0.3 (0.2 to 0.7)	407 (277 to 576)	54.5 (37.3 to 74.7)	5 (2 to 7)	0.9 (0.4 to 1.2)	221.7 (130.5 to 280.9)	32.8 (-11.6 to 59.2)	760.2 (89.8 to 1381.5)	241.6 (-42.6 to 507.8)
	Gynecological diseases	Female	3613 (2484 to 5063)	1115.9 (763.4 to 1545.4)	0 (0 to 0)	0 (0 to 0.1)	10793 (7411 to 15083)	1052.1 (722.8 to 1456)	1 (0 to 1)	0.1 (0 to 0.2)	198.7 (180.5 to 214.8)	-5.7 (-9.1 to -2.5)	535.5 (-40 to 2131.6)	120.7 (-77.1 to 622.3)
	Hemoglobinopathies and hemolytic anemias	Both	3680 (2578 to 4799)	315.4 (224.4 to 405.6)	28 (14 to 39)	2.4 (1.3 to 3.1)	2552 (1983 to 3318)	130.9 (102.2 to 169.8)	22 (18 to 26)	1.2 (1 to 1.4)	-30.7 (-48 to -0.4)	-58.5 (-67.3 to -43.1)	-24 (-48.1 to 52.2)	-50.4 (-63.9 to -6.3)
		Female	2102 (1381 to 2866)	386.9 (263.2 to 519.6)	15 (7 to 22)	2.5 (1.2 to 3.6)	1373 (982 to 1914)	143.7 (104.9 to 195.5)	9 (7 to 11)	1 (0.9 to 1.3)	-34.7 (-56.5 to -1.9)	-62.8 (-73.1 to -47.5)	-38.6 (-64.2 to 38.9)	-58.5 (-73.5 to -11.8)
		Male	1578 (1072 to 2097)	246.2 (173 to 319.5)	14 (8 to 19)	2.3 (1.3 to 3)	1179 (929 to 1508)	118.8 (93.8 to 153.1)	13 (10 to 15)	1.3 (1.1 to 1.6)	-25.3 (-46.4 to 13.9)	-51.7 (-64.5 to -30.2)	-8.4 (-38.4 to 74.2)	-41.5 (-57.9 to 4.8)
	Endocrine, metabolic, blood, and immune disorders	Both	3058 (2279 to 4064)	353.2 (259.6 to 462.4)	16 (10 to 24)	2 (1.4 to 2.9)	6086 (4276 to 8110)	352.2 (247.4 to 467.2)	35 (17 to 46)	2.7 (1.3 to 3.5)	99 (42.3 to 146.2)	-0.3 (-21.7 to 16.1)	123.8 (-14.1 to 291.9)	37.1 (-40.5 to 133.2)
		Female	1779 (1251 to 2408)	439.7 (312.2 to 589.2)	7 (4 to 11)	2 (1.3 to 3.3)	3666 (2543 to 5017)	427.5 (301.5 to 580.3)	16 (6 to 22)	2.7 (1 to 3.6)	106 (51.4 to 151.9)	-2.8 (-22 to 13.5)	118.4 (-30.1 to 337.8)	33.4 (-50.1 to 162.3)
		Male	1279 (882 to 1785)	273.8 (201.7 to 360.2)	8 (5 to 14)	1.9 (1.3 to 2.8)	2420 (1657 to 3215)	280.7 (189.3 to 370.3)	19 (8 to 26)	2.7 (1.2 to 3.8)	89.2 (23.8 to 157.2)	2.5 (-24.5 to 27.6)	128.7 (-8.1 to 309.5)	42.3 (-33.8 to 132)
	Oral disorders	Both	1764 (1045 to 2753)	318 (190.7 to 495.4)	--	--	4926 (2869 to 7947)	312.6 (185.9 to 488.9)	--	--	179.2 (167.5 to 190.4)	-1.7 (-4.1 to 0.5)	--	--
		Female	825 (485 to 1292)	307.5 (182.1 to 481.3)	--	--	2375 (1376 to 3817)	302.5 (180 to 475.8)	--	--	187.7 (173.6 to 200.9)	-1.6 (-4.8 to 1.4)	--	--
		Male	939 (559 to 1471)	327.4 (196.6 to 508)	--	--	2550 (1498 to 4116)	322.9 (193.4 to 502.5)	--	--	171.7 (158.6 to 185.9)	-1.3 (-4.5 to 1.6)	--	--
	Sudden infant death syndrome	Both	1699 (268 to 5887)	103.2 (16.3 to 357.7)	19 (3 to 67)	1.2 (0.2 to 4)	327 (163 to 675)	17.4 (8.7 to 35.8)	4 (2 to 8)	0.2 (0.1 to 0.4)	-80.7 (-93.7 to 20.2)	-83.2 (-94.5 to 5.2)	-80.8 (-93.7 to 20.2)	-83.2 (-94.5 to 5.2)

Province	Cause	Sex	1990				2019				1990-2019 percent change (%)			
			DALYs		Death		DALYs		Death		DALYs		Death	
			Number	Rate	Number	Rate	Number	Rate	Number	Rate	Number	Rate	Number	Rate
		Female	776 (125 to 2819)	96.4 (15.6 to 350.1)	9 (1 to 32)	1.1 (0.2 to 4)	157 (75 to 309)	17.1 (8.1 to 33.7)	2 (1 to 3)	0.2 (0.1 to 0.4)	-79.8 (-93.3 to 24.7)	-82.2 (-94.2 to 9.6)	-79.8 (-93.3 to 24.8)	-82.2 (-94.2 to 9.7)
		Male	923 (115 to 3301)	109.8 (13.6 to 392.7)	10 (1 to 37)	1.2 (0.2 to 4.4)	170 (76 to 373)	17.6 (7.8 to 38.6)	2 (1 to 4)	0.2 (0.1 to 0.4)	-81.6 (-94.4 to 26.1)	-84 (-95.1 to 9.8)	-81.6 (-94.4 to 26.1)	-84 (-95.1 to 9.8)
	**Injuries**	Both	71985 (39584 to 90263)	7328 (4277.6 to 8861.7)	997 (501 to 1243)	121.9 (68.6 to 148.8)	66537 (58457 to 74984)	3452.1 (3038.9 to 3904.3)	1056 (909 to 1208)	59.5 (51.2 to 68.2)	-7.6 (-27.6 to 72)	-52.9 (-61.8 to -16.4)	5.9 (-17.8 to 111.8)	-51.2 (-61.7 to -11.9)
		Female	24949 (12676 to 33441)	4667.7 (2556.2 to 5903.9)	321 (150 to 426)	71.6 (37.5 to 92.2)	13471 (11614 to 15419)	1493.3 (1292.7 to 1706.8)	198 (167 to 231)	25.3 (21.3 to 29.7)	-46 (-60.2 to 9)	-68 (-75.8 to -40.5)	-38.5 (-56.8 to 34.8)	-64.7 (-74.4 to -32.5)
		Male	47036 (26900 to 58323)	9815.7 (5843.4 to 11868.1)	676 (357 to 846)	168.8 (97 to 210)	53066 (45739 to 60696)	5329 (4579.7 to 6111.3)	859 (722 to 996)	92.5 (77.3 to 108.2)	12.8 (-10.9 to 106.3)	-45.7 (-56.8 to -4.7)	27.1 (-3.2 to 151.4)	-45.2 (-58.6 to -3.3)
	Transport injuries	Both	36729 (17697 to 47639)	3858.5 (2037.4 to 4848.1)	566 (280 to 722)	72.8 (40.1 to 92.1)	36918 (31290 to 42428)	1905.9 (1616.1 to 2192.2)	654 (542 to 764)	36.8 (30.5 to 42.8)	0.5 (-23.5 to 103)	-50.6 (-61.2 to -3.5)	15.5 (-10.7 to 137.5)	-49.5 (-60.9 to -6.3)
		Female	12472 (5763 to 17547)	2375.8 (1192.4 to 3164.2)	174 (80 to 238)	40.4 (20.7 to 53.7)	6444 (5491 to 7391)	716 (614 to 817.3)	115 (95 to 134)	14.4 (11.9 to 16.8)	-48.3 (-64.7 to 12.3)	-69.9 (-78.7 to -39.6)	-33.7 (-54.4 to 45.3)	-64.3 (-74.3 to -31)
		Male	24257 (12257 to 31131)	5238.8 (2802 to 6655.3)	393 (200 to 506)	102.9 (55.6 to 132.7)	30475 (25154 to 35656)	3044.2 (2517.3 to 3562.4)	539 (433 to 643)	58.3 (46.5 to 69.6)	25.6 (-4 to 160.2)	-41.9 (-55.9 to 11.4)	37.3 (3.2 to 176.4)	-43.3 (-57.5 to 1.9)
	Road injuries	Both	36161 (17337 to 46928)	3794 (1997.9 to 4770.7)	558 (276 to 711)	71.7 (39.5 to 90.7)	35784 (30319 to 41148)	1847.2 (1567.3 to 2123.4)	636 (526 to 743)	35.7 (29.7 to 41.7)	-1 (-24.6 to 99.3)	-51.3 (-61.7 to -4.6)	13.9 (-11.8 to 134.6)	-50.2 (-61.2 to -8.1)
		Female	12347 (5696 to 17387)	2349.2 (1175.4 to 3122.2)	172 (79 to 236)	40.1 (20.6 to 53.3)	6298 (5366 to 7218)	699.6 (599.1 to 800.7)	113 (94 to 132)	14.2 (11.7 to 16.6)	-49 (-65.3 to 11)	-70.2 (-79 to -40.2)	-34.2 (-54.7 to 44.1)	-64.6 (-74.5 to -31.5)
		Male	23814 (12012 to 30580)	5139.1 (2743.1 to 6553.1)	386 (195 to 496)	101 (54.2 to 130.5)	29487 (24279 to 34572)	2944.8 (2421.9 to 3462.9)	523 (418 to 622)	56.5 (45 to 67.6)	23.8 (-5.1 to 156)	-42.7 (-56.5 to 10.5)	35.4 (1.6 to 173.8)	-44.1 (-58.1 to 1.2)
	Other transport injuries	Both	568 (315 to 807)	64.5 (37.9 to 88.2)	8 (4 to 12)	1.1 (0.6 to 1.6)	1134 (917 to 1405)	58.8 (47.6 to 73.3)	18 (14 to 24)	1 (0.8 to 1.3)	99.7 (32.6 to 271.4)	-8.9 (-38.1 to 57.6)	126.8 (44 to 367.7)	-6.1 (-40.7 to 77.7)
		Female	125 (68 to 192)	26.6 (16.3 to 37.7)	1 (1 to 2)	0.3 (0.2 to 0.5)	146 (119 to 177)	16.3 (13.3 to 19.7)	2 (1 to 2)	0.2 (0.2 to 0.3)	17.4 (-23.6 to 116.1)	-38.5 (-57 to 0.6)	26.7 (-23.4 to 205.5)	-33 (-60.1 to 42.3)
		Male	443 (241 to 675)	99.6 (58 to 144.8)	7 (3 to 10)	1.8 (1 to 2.7)	988 (779 to 1284)	99.4 (78.7 to 128.4)	17 (13 to 22)	1.8 (1.4 to 2.4)	122.9 (37.6 to 327.7)	-0.2 (-36 to 78.5)	147.8 (47.4 to 418.1)	0.5 (-40.7 to 89.8)
	Unintentional injuries	Both	27250 (16834 to 35340)	2521.2 (1626.8 to 3073.1)	341 (194 to 437)	37.4 (22.3 to 45.5)	18116 (15488 to 21153)	970.7 (827 to 1137.1)	240 (205 to 285)	14.6 (12.5 to 17.4)	-33.5 (-50.1 to 13.5)	-61.5 (-69.5 to -38.2)	-29.6 (-48.7 to 32.2)	-61 (-70 to -33.4)
		Female	10478 (5756 to 14437)	1817.4 (1081.4 to 2396.4)	125 (62 to 175)	25.5 (14.7 to 33.6)	4795 (3989 to 5817)	552.1 (460.1 to 668.6)	56 (46 to 70)	8.1 (6.6 to 10.2)	-54.2 (-68.5 to -14.2)	-69.6 (-77.8 to -48.6)	-54.8 (-70.3 to -9.3)	-68.1 (-77.8 to -45)
		Male	16772 (10567 to 21840)	3174.6 (2076.2 to 3888.8)	216 (124 to 274)	48.4 (29 to 60.3)	13322 (11351 to 15659)	1373 (1162.3 to 1616.5)	183 (153 to 222)	20.9 (17.4 to 25.2)	-20.6 (-40.9 to 28.9)	-56.7 (-66.3 to -32.5)	-15 (-39.2 to 50.5)	-56.9 (-67.9 to -28.1)
	Falls	Both	3477 (2386 to 4568)	447.5 (338.6 to 558.4)	37 (20 to 50)	6.2 (4.3 to 7.9)	4934 (3945 to 6135)	282.8 (228.2 to 350.7)	54 (44 to 68)	3.9 (3.2 to 4.9)	41.9 (5.4 to 105.2)	-36.8 (-48.1 to -18.9)	46.6 (-1.3 to 165)	-37.4 (-53.2 to -3.1)
		Female	1029 (627 to 1543)	257.8 (192.3 to 330.7)	10 (6 to 16)	3.7 (2.6 to 5.3)	1171 (906 to 1511)	150.1 (118.3 to 190.1)	11 (9 to 16)	2.2 (1.8 to 3)	13.8 (-28.8 to 82.2)	-41.8 (-55.5 to -25.6)	10.5 (-33.2 to 111.3)	-41.6 (-56.2 to -7.5)
		Male	2448 (1579 to 3301)	620 (455.9 to 789.6)	26 (14 to 37)	8.4 (5.4 to 11.1)	3763 (2981 to 4675)	411.7 (327.9 to 508.3)	42 (34 to 56)	5.5 (4.4 to 7.3)	53.7 (9.3 to 129.4)	-33.6 (-47.7 to -12.1)	60.7 (2.7 to 209.3)	-34.2 (-53.8 to 6.9)

Province	Cause	Sex	1990				2019				1990-2019 percent change (%)			
			DALYs		Death		DALYs		Death		DALYs		Death	
			Number	Rate	Number	Rate	Number	Rate	Number	Rate	Number	Rate	Number	Rate
	Drowning	Both	7009 (3506 to 10609)	500.3 (250.4 to 724.9)	89 (44 to 131)	7 (3.6 to 9.7)	1754 (1432 to 2125)	88.9 (72.5 to 106.9)	28 (23 to 34)	1.5 (1.2 to 1.8)	-75 (-84.1 to -48.1)	-82.2 (-88.4 to -62.9)	-68.1 (-79.4 to -33.3)	-78.4 (-85.5 to -55.6)
		Female	2993 (1219 to 4872)	404.9 (165.1 to 648.6)	36 (15 to 59)	5.2 (2.2 to 8.1)	289 (228 to 372)	30.6 (24.3 to 38.9)	4 (4 to 6)	0.5 (0.4 to 0.6)	-90.3 (-94.6 to -75.2)	-92.4 (-95.6 to -80.5)	-87.8 (-92.9 to -68.6)	-90.2 (-94 to -76)
		Male	4016 (1612 to 6448)	591.2 (251.4 to 909.2)	52 (22 to 83)	8.6 (3.9 to 12.7)	1465 (1156 to 1820)	144.4 (114.2 to 179.7)	24 (19 to 30)	2.5 (1.9 to 3)	-63.5 (-78.2 to -16.7)	-75.6 (-84.9 to -47)	-54.4 (-72.6 to 1.3)	-71.4 (-81.8 to -40.8)
	Fire, heat, and hot substances	Both	3667 (1640 to 5668)	342.8 (165.6 to 487.9)	51 (22 to 76)	6 (3.2 to 8.2)	1565 (1030 to 2534)	82.4 (54.3 to 134.1)	29 (18 to 48)	1.8 (1.1 to 3.1)	-57.3 (-80 to 14.9)	-76 (-88 to -41)	-43.7 (-74.3 to 58.4)	-70 (-85.2 to -29.2)
		Female	1521 (420 to 2884)	293.7 (112 to 502)	21 (6 to 38)	5 (2.3 to 8)	650 (383 to 1145)	72.3 (42.5 to 129.5)	12 (6 to 23)	1.6 (0.9 to 3.3)	-57.3 (-85.1 to 116.1)	-75.4 (-90.6 to -10.4)	-42.5 (-81.2 to 184.8)	-66.9 (-87.7 to 0.9)
		Male	2146 (812 to 3172)	388.7 (172.6 to 544.9)	30 (12 to 44)	6.9 (3.3 to 9.7)	915 (607 to 1701)	92 (61.7 to 169.1)	17 (11 to 32)	1.9 (1.3 to 3.8)	-57.4 (-78.2 to 63.6)	-76.3 (-87.1 to -25.7)	-44.5 (-71.3 to 107.6)	-71.8 (-84.5 to -20.9)
	Poisonings	Both	1876 (1006 to 2802)	148.3 (80.2 to 214.1)	25 (13 to 36)	2.3 (1.3 to 3.2)	601 (458 to 1194)	30 (23.1 to 58.4)	10 (8 to 20)	0.6 (0.4 to 1.1)	-68 (-81.3 to -30)	-79.8 (-87.3 to -58.1)	-58 (-74.6 to -10.5)	-75.4 (-83.6 to -51.8)
		Female	796 (362 to 1363)	119.1 (57 to 188.6)	10 (5 to 17)	1.7 (0.8 to 2.5)	227 (177 to 292)	23.7 (18.9 to 30.4)	4 (3 to 5)	0.4 (0.3 to 0.5)	-71.5 (-84.6 to -34)	-80.1 (-88.6 to -57.3)	-63.4 (-79.3 to -16.8)	-75.1 (-84.3 to -48.5)
		Male	1081 (552 to 1794)	175.6 (94.8 to 275.8)	15 (8 to 24)	2.8 (1.5 to 4.4)	374 (257 to 947)	36.1 (25.1 to 89.7)	7 (5 to 17)	0.7 (0.5 to 1.7)	-65.4 (-82.8 to -15)	-79.5 (-88.8 to -52.8)	-54.3 (-75.9 to 5.2)	-75.3 (-86.1 to -46.4)
	Exposure to mechanical forces	Both	2636 (1505 to 3553)	313.7 (199.5 to 419.3)	33 (16 to 48)	4.5 (2.4 to 6.7)	3612 (2974 to 4400)	187.6 (153.8 to 227.9)	46 (37 to 56)	2.5 (2 to 3)	37.1 (-2.4 to 132.5)	-40.2 (-57 to -7.1)	37.6 (-9.5 to 187.4)	-44.5 (-64.8 to 6.7)
		Female	663 (375 to 991)	144.1 (95.3 to 194.1)	7 (3 to 11)	1.7 (0.9 to 2.4)	733 (565 to 947)	81.9 (63.2 to 105.3)	7 (5 to 9)	0.9 (0.7 to 1.2)	10.4 (-31.1 to 93.6)	-43.2 (-59.6 to -15.3)	-0.1 (-43.3 to 119.2)	-48.1 (-66.6 to -3.7)
		Male	1972 (1098 to 2796)	471.1 (295 to 669.5)	26 (12 to 41)	7 (3.6 to 11.2)	2880 (2345 to 3576)	289 (234 to 354.2)	39 (30 to 49)	4 (3.2 to 5)	46 (2.9 to 152.3)	-38.7 (-58.3 to -1.5)	47.6 (-6.7 to 205.1)	-42.8 (-65.6 to 11.2)
	Adverse effects of medical treatment	Both	1160 (653 to 1577)	125.8 (69.9 to 171.1)	20 (11 to 26)	3.1 (1.9 to 4.9)	682 (557 to 914)	39.2 (31.6 to 54)	15 (12 to 22)	1.1 (0.8 to 1.6)	-41.2 (-59 to 7.8)	-68.9 (-79.4 to -45.1)	-22.5 (-49.3 to 39.6)	-64.8 (-78.7 to -40)
		Female	455 (235 to 692)	96.3 (50.5 to 149.3)	7 (4 to 11)	2.3 (1.2 to 5.4)	228 (178 to 335)	27.7 (21.5 to 42.2)	5 (4 to 8)	0.8 (0.6 to 1.4)	-50 (-67.5 to -2.8)	-71.3 (-80.5 to -46.4)	-29.1 (-54.7 to 37)	-64.7 (-79.2 to -35.1)
		Male	705 (396 to 1020)	152.9 (84.1 to 224.1)	12 (7 to 18)	3.9 (2.1 to 6.7)	454 (356 to 604)	50.3 (38.7 to 68.7)	10 (8 to 14)	1.4 (1 to 2.1)	-35.5 (-59.6 to 17.8)	-67.1 (-80.1 to -38.9)	-18.6 (-51.4 to 50.6)	-64.6 (-79.9 to -32)
	Animal contact	Both	2943 (463 to 4668)	224.2 (43.5 to 338.3)	38 (5 to 59)	3.6 (0.6 to 5.1)	1000 (294 to 1306)	54.8 (16.2 to 71.1)	16 (3 to 21)	1.1 (0.2 to 1.4)	-66 (-79.5 to -18.7)	-75.6 (-84.5 to -49.4)	-56.5 (-73.1 to -2)	-70 (-80.1 to -42.5)
		Female	1249 (131 to 2466)	187.3 (28.4 to 344.1)	15 (1 to 30)	2.7 (0.3 to 4.8)	387 (108 to 563)	42.8 (12.1 to 61.1)	6 (1 to 8)	0.8 (0.1 to 1.1)	-69 (-85.3 to 6.4)	-77.1 (-88.3 to -37.4)	-62.9 (-81.5 to 24.9)	-72.3 (-84.4 to -31)
		Male	1693 (274 to 2788)	258.2 (48.8 to 405.2)	22 (3 to 36)	4.3 (0.7 to 6.8)	613 (178 to 803)	66.4 (19.2 to 86.1)	11 (2 to 14)	1.4 (0.3 to 1.8)	-63.8 (-79.5 to 3.7)	-74.3 (-84.8 to -39)	-52 (-72.4 to 25)	-68.4 (-81.2 to -33)
	Foreign body	Both	1552 (1060 to 2184)	113 (80.8 to 152.3)	17 (11 to 24)	1.3 (0.9 to 1.7)	774 (631 to 954)	41.4 (34 to 50.7)	8 (6 to 9)	0.5 (0.4 to 0.5)	-50.1 (-67.3 to -22.7)	-63.4 (-74.5 to -47)	-55.7 (-71.8 to -26.7)	-64.7 (-75.8 to -45.5)
		Female	712 (448 to 1093)	103.3 (68.7 to 151.2)	8 (5 to 12)	1.1 (0.7 to 1.7)	270 (211 to 341)	30.3 (24 to 37.8)	2 (2 to 3)	0.3 (0.3 to 0.4)	-62.1 (-77.9 to -34.6)	-70.7 (-81.7 to -53.4)	-70.3 (-83.2 to -45.8)	-73 (-83.7 to -54.1)
		Male	840 (540 to 1255)	122.2 (84.5 to 170.6)	9 (6 to 14)	1.4 (0.9 to 2)	504 (405 to 629)	52 (42 to 64.5)	5 (4 to 7)	0.6 (0.5 to 0.7)	-40 (-62.4 to -1.8)	-57.5 (-71.5 to -35.1)	-43.4 (-65.9 to 3.1)	-58.3 (-73 to -32.6)
	Environmental heat and cold exposure	Both	210 (153 to 272)	26.7 (19.6 to 35.4)	2 (1 to 3)	0.3 (0.2 to 0.5)	322 (235 to 422)	17.3 (12.8 to 22.8)	2 (1 to 3)	0.1 (0.1 to 0.2)	52.8 (12.7 to 94)	-35 (-51.2 to -19.3)	17 (-37.7 to 113.9)	-54.4 (-75.1 to -18.4)
		Female	88 (62 to 116)	22.9 (17.1 to 30)	0 (0 to 1)	0.2 (0.1 to 0.3)	143 (99 to 197)	16.1 (11.3 to 22.1)	1 (0 to 1)	0.1 (0 to 0.1)	63.1 (24.6 to 100)	-29.5 (-43.6 to -17.7)	16.6 (-45.8 to 122.1)	-44.5 (-76.7 to 2.9)

Province	Cause	Sex	1990				2019				1990-2019 percent change (%)			
			DALYs		Death		DALYs		Death		DALYs		Death	
			Number	Rate	Number	Rate	Number	Rate	Number	Rate	Number	Rate	Number	Rate
		Male	123 (84 to 172)	30.3 (21.2 to 43.7)	1 (1 to 2)	0.4 (0.2 to 0.8)	178 (125 to 231)	18.5 (13.4 to 23.8)	1 (1 to 2)	0.2 (0.1 to 0.2)	45.4 (2.1 to 95)	-38.9 (-57.5 to -18.5)	17.2 (-36.4 to 122.1)	-57.8 (-77.9 to -21.5)
	Exposure to forces of nature	Both	39 (32 to 48)	4.4 (3.6 to 5.5)	0 (0 to 0)	0 (0 to 0)	42 (31 to 59)	2.4 (1.7 to 3.3)	0 (0 to 0)	0 (0 to 0)	9.2 (-7.1 to 26.4)	-46.3 (-53.7 to -38.3)	-66.6 (-66.6 to -66.6)	-84.4 (-84.4 to -84.4)
		Female	14 (12 to 17)	3.2 (2.6 to 3.9)	0 (0 to 0)	0 (0 to 0)	15 (10 to 20)	1.7 (1.2 to 2.4)	0 (0 to 0)	0 (0 to 0)	5.3 (-12.1 to 25)	-46.6 (-54.7 to -37.7)	-67.4 (-67.4 to -67.4)	-84.3 (-84.3 to -84.3)
		Male	25 (20 to 31)	5.6 (4.6 to 7.1)	0 (0 to 0)	0.1 (0 to 0.1)	28 (20 to 38)	3 (2.2 to 4.1)	0 (0 to 0)	0 (0 to 0)	11.4 (-4.6 to 28.8)	-45.9 (-53.1 to -37.9)	-66.1 (-66.1 to -66.1)	-84.4 (-84.4 to -84.4)
	Other unintentional injuries	Both	2680 (1607 to 3627)	274.4 (173.9 to 361.4)	30 (15 to 43)	3.2 (1.6 to 4.5)	2830 (2215 to 3462)	143.9 (113.1 to 177.2)	32 (24 to 39)	1.6 (1.3 to 2)	5.6 (-27.5 to 75.6)	-47.6 (-63.6 to -16.9)	5.1 (-35.3 to 118.9)	-49.4 (-69.2 to 5.8)
		Female	958 (548 to 1432)	184.8 (116.5 to 255.3)	10 (4 to 15)	1.8 (0.8 to 2.7)	682 (510 to 908)	74.9 (55.9 to 100.1)	4 (3 to 5)	0.5 (0.4 to 0.6)	-28.8 (-57.1 to 27.5)	-59.4 (-72.8 to -37.6)	-54.8 (-75 to 7.5)	-71.3 (-83.1 to -39)
		Male	1722 (1008 to 2526)	358.7 (226.6 to 517.2)	21 (10 to 33)	4.6 (2.2 to 7.2)	2148 (1626 to 2623)	209.6 (159.2 to 256.7)	28 (20 to 35)	2.7 (2 to 3.4)	24.7 (-23.1 to 112.1)	-41.6 (-64.1 to -6.4)	32.9 (-28.9 to 175.9)	-40.8 (-69.2 to 21.8)
	Self-harm and interpersonal violence	Both	8006 (5213 to 9900)	948.3 (642.3 to 1163.4)	90 (45 to 113)	11.8 (6.4 to 15)	11502 (9715 to 13449)	575.5 (483.2 to 669.1)	162 (134 to 194)	8.2 (6.7 to 9.7)	43.7 (16.1 to 114.6)	-39.3 (-50.4 to -11.8)	80 (37.5 to 254.1)	-30.8 (-47 to 27.9)
		Female	2000 (1077 to 2673)	474.5 (273.3 to 617.8)	23 (8 to 32)	5.7 (2.4 to 7.9)	2232 (1849 to 2659)	225.2 (188.1 to 266.1)	26 (21 to 32)	2.8 (2.2 to 3.4)	11.6 (-17.6 to 107.1)	-52.5 (-64.4 to -18.7)	15.1 (-24.2 to 227.9)	-51.6 (-66.9 to 17.9)
		Male	6007 (4186 to 7459)	1402.4 (987.3 to 1748.6)	68 (37 to 87)	17.5 (10 to 23.1)	9270 (7654 to 11044)	911.7 (754.4 to 1080.3)	136 (110 to 166)	13.3 (10.7 to 16.2)	54.3 (23.4 to 119.6)	-35 (-47.9 to -8.7)	101.6 (51 to 260.9)	-23.8 (-43.4 to 32.8)
	Self-harm	Both	2814 (911 to 3805)	348 (133.5 to 463)	48 (16 to 64)	6.5 (2.8 to 8.8)	5379 (4232 to 6744)	259.8 (200.2 to 322.9)	95 (75 to 119)	4.8 (3.8 to 6)	91.1 (34.6 to 462.8)	-25.4 (-46.4 to 86.7)	100.4 (41.6 to 466.5)	-25.9 (-47.2 to 71.5)
		Female	987 (263 to 1475)	238.4 (79.3 to 343.8)	15 (4 to 23)	4 (1.4 to 5.8)	865 (666 to 1098)	87.1 (67.9 to 111)	15 (11 to 19)	1.6 (1.2 to 2)	-12.3 (-45.3 to 218.8)	-63.5 (-76.4 to 5.4)	-4.9 (-41.7 to 278.9)	-61.2 (-75.3 to 16.5)
		Male	1827 (640 to 2581)	450.6 (180.8 to 648)	32 (12 to 47)	8.9 (4 to 13)	4513 (3429 to 5827)	424.5 (318.7 to 543.8)	81 (61 to 103)	8 (6 to 10.2)	147 (69.8 to 588.6)	-5.8 (-35.2 to 122)	150.6 (74.2 to 533.5)	-10.1 (-38 to 92.2)
	Interpersonal violence	Both	1630 (865 to 2197)	193.8 (109 to 257.4)	21 (9 to 30)	2.8 (1.3 to 4)	3742 (3112 to 4520)	182.8 (152.1 to 220.4)	49 (39 to 62)	2.4 (1.9 to 3.1)	129.6 (65.5 to 330.9)	-5.7 (-31 to 64.4)	131.3 (53.6 to 447)	-12 (-41.4 to 86.3)
		Female	515 (317 to 692)	113.6 (74.6 to 149)	5 (2 to 7)	1.1 (0.5 to 1.7)	996 (785 to 1259)	100.2 (79.1 to 126.5)	9 (7 to 12)	1 (0.7 to 1.3)	93.2 (31.5 to 224.5)	-11.7 (-35.4 to 35.2)	86.1 (6.1 to 389.8)	-14.8 (-48.8 to 100)
		Male	1114 (541 to 1608)	268.3 (137.7 to 381.6)	16 (7 to 25)	4.3 (1.8 to 6.5)	2747 (2172 to 3419)	261.7 (210.3 to 328.7)	40 (30 to 51)	3.9 (2.9 to 5)	146.4 (63.2 to 401.3)	-2.5 (-35.2 to 89.4)	145 (51.9 to 503.5)	-9.9 (-44.3 to 115)
	Conflict and terrorism	Both	3124 (2312 to 4228)	352.8 (260.1 to 475.6)	15 (14 to 16)	1.6 (1.4 to 1.8)	1319 (865 to 1973)	82.5 (54.4 to 122.1)	1 (1 to 1)	0 (0 to 0)	-57.8 (-64 to -51.8)	-76.6 (-80.1 to -73.3)	-95.1 (-95.1 to -95.1)	-97.6 (-97.6 to -97.6)
		Female	423 (283 to 643)	104.5 (70.7 to 154.4)	1 (1 to 1)	0.3 (0.3 to 0.3)	241 (146 to 405)	24.4 (14.9 to 40.3)	0 (0 to 0)	0 (0 to 0)	-43 (-52.6 to -31.5)	-76.7 (-81.1 to -71.5)	-84.6 (-84.6 to -84.6)	-91.9 (-91.9 to -91.9)
		Male	2702 (2010 to 3631)	595.8 (441.9 to 799.5)	14 (12 to 15)	2.9 (2.6 to 3.2)	1079 (712 to 1597)	140.1 (92.8 to 207.5)	1 (0 to 1)	0.1 (0 to 0.1)	-60.1 (-66.1 to -54.4)	-76.5 (-80.2 to -73.2)	-96.1 (-96.1 to -96.1)	-98.2 (-98.2 to -98.2)
	Executions and police conflict	Both	439 (341 to 570)	53.7 (41.1 to 68.5)	6 (5 to 9)	0.9 (0.7 to 1.2)	1062 (696 to 1583)	50.4 (35 to 73.7)	17 (11 to 26)	0.8 (0.6 to 1.2)	142.1 (62.7 to 268.8)	-6.1 (-35.7 to 38.6)	166.8 (68.2 to 321.1)	-5.3 (-38.1 to 44.5)
		Female	75 (60 to 103)	18.1 (14 to 25.6)	1 (1 to 2)	0.3 (0.2 to 0.4)	130 (94 to 209)	13.5 (10.2 to 20.2)	2 (1 to 3)	0.2 (0.2 to 0.4)	74.2 (24.6 to 175)	-25.4 (-47.6 to 11.1)	96.1 (28.3 to 241.9)	-25 (-49.8 to 20.1)
		Male	364 (277 to 487)	87.6 (65.8 to 115.6)	5 (4 to 7)	1.4 (1.1 to 1.9)	932 (593 to 1447)	85.5 (56.9 to 131.3)	15 (9 to 24)	1.4 (0.9 to 2.2)	156.1 (62.5 to 308.1)	-2.4 (-36.8 to 52.1)	180 (66.5 to 365.4)	-1 (-39.9 to 59.6)

Province	Cause	Sex	1990				2019				1990-2019 percent change (%)			
			DALYs		Death		DALYs		Death		DALYs		Death	
			Number	Rate	Number	Rate	Number	Rate	Number	Rate	Number	Rate	Number	Rate
**Ilam**	**All causes**	Both	182972 (162779 to 204926)	43747.7 (39177.5 to 48907.1)	2503 (2251 to 2786)	1011.7 (910.5 to 1128.6)	143248 (124179 to 164227)	26575.7 (23373.6 to 30169)	2714 (2499 to 2932)	675.5 (626.8 to 724.5)	-21.7 (-31.7 to -10.6)	-39.3 (-45.2 to -33)	8.4 (-5 to 23.9)	-33.2 (-40.7 to -24.2)
		Female	82485 (72979 to 92620)	40258 (35756.2 to 45477.8)	1019 (899 to 1153)	859.9 (751.9 to 972.1)	65556 (55579 to 77618)	24725.5 (21357.1 to 28802)	1055 (952 to 1174)	577.1 (528.5 to 634.5)	-20.5 (-31.3 to -8.3)	-38.6 (-45.7 to -31)	3.5 (-11.9 to 21.6)	-32.9 (-42.8 to -20.7)
		Male	100487 (88546 to 113730)	46572.5 (40969.7 to 53536.2)	1483 (1297 to 1700)	1135.3 (971.7 to 1324.8)	77692 (67719 to 88293)	28261.4 (24805.4 to 31896.9)	1658 (1471 to 1866)	761 (678.3 to 851.5)	-22.7 (-33.3 to -10.8)	-39.3 (-47.4 to -30.7)	11.8 (-7 to 35)	-33 (-44.2 to -18.8)
	**Communicable, maternal, neonatal, and nutritional diseases**	Both	71183 (58052 to 86944)	10099.6 (8292.8 to 12693.6)	791 (640 to 991)	126 (104.1 to 170.2)	11156 (9265 to 13318)	2340.9 (1915.1 to 2822.3)	118 (99 to 144)	29.4 (24.7 to 36.1)	-84.3 (-88.3 to -80)	-76.8 (-82.8 to -70.3)	-85.1 (-88.7 to -80)	-76.7 (-83.1 to -69.5)
		Female	33425 (27221 to 41006)	9754.9 (8022.8 to 12212.4)	368 (298 to 463)	119.8 (98.1 to 171.6)	5097 (4182 to 6063)	2180.2 (1775 to 2602.9)	50 (41 to 61)	26.2 (21.3 to 32.9)	-84.8 (-88.5 to -80.5)	-77.7 (-83.7 to -71.3)	-86.5 (-90.1 to -81.8)	-78.1 (-84.8 to -71.2)
		Male	37758 (30715 to 47057)	10418.3 (8483.1 to 13006)	423 (340 to 533)	131.5 (106.8 to 171.5)	6059 (5007 to 7262)	2491.6 (2045.3 to 3023.4)	68 (57 to 85)	32.2 (26.9 to 41.5)	-84 (-88.2 to -79.3)	-76.1 (-82.4 to -68.9)	-83.9 (-88.1 to -77.5)	-75.6 (-82.4 to -67.2)
	HIV/AIDS and sexually transmitted infections	Both	134 (66 to 260)	25.3 (14.8 to 43.1)	1 (0 to 3)	0.2 (0.1 to 0.4)	152 (106 to 219)	24.8 (16.5 to 37.6)	1 (1 to 2)	0.2 (0.1 to 0.4)	13.5 (-29.3 to 93.8)	-2 (-24.5 to 23.9)	0.1 (-42.4 to 129.9)	19.2 (-21.7 to 106.2)
		Female	68 (35 to 126)	29 (17.1 to 48.2)	1 (0 to 1)	0.2 (0.1 to 0.4)	88 (59 to 132)	28.3 (18.9 to 42.7)	1 (0 to 1)	0.2 (0.1 to 0.4)	29.8 (-19.1 to 105.5)	-2.5 (-23.3 to 22.1)	11.6 (-38.2 to 166.7)	30.9 (-18.2 to 125.1)
		Male	66 (29 to 133)	21.7 (11.6 to 39.4)	1 (0 to 1)	0.2 (0.1 to 0.4)	64 (45 to 93)	21.3 (13.9 to 33.5)	1 (0 to 1)	0.2 (0.1 to 0.3)	-3.3 (-40.4 to 73.3)	-2.1 (-28.1 to 31.1)	-9.5 (-46.5 to 102.6)	8.7 (-27.7 to 87.1)
	HIV/AIDS	Both	5 (3 to 7)	1.4 (0.7 to 1.9)	0 (0 to 0)	0 (0 to 0)	40 (30 to 55)	5.8 (4.3 to 8)	1 (0 to 1)	0.1 (0.1 to 0.1)	726.4 (425.6 to 1498.2)	330.1 (170.8 to 748.3)	931.5 (474.4 to 2756.9)	366.6 (153.3 to 1385.7)
		Female	2 (1 to 3)	1.1 (0.6 to 1.5)	0 (0 to 0)	0 (0 to 0)	20 (14 to 29)	6.1 (4.2 to 8.7)	0 (0 to 0)	0.1 (0.1 to 0.1)	931.9 (515.9 to 2000.5)	474 (239.6 to 1066.5)	1186 (555.1 to 3478.2)	507.9 (205.2 to 1798)
		Male	3 (2 to 4)	1.6 (0.9 to 2.3)	0 (0 to 0)	0 (0 to 0)	20 (15 to 28)	5.6 (4.3 to 7.7)	0 (0 to 0)	0.1 (0.1 to 0.1)	591.1 (349.3 to 1208.9)	239.2 (118.3 to 559.6)	766.9 (404.8 to 2351.3)	275.3 (116 to 1114.6)
	Sexually transmitted infections excluding HIV	Both	129 (61 to 255)	23.9 (13.5 to 41.5)	1 (0 to 2)	0.2 (0.1 to 0.4)	112 (69 to 175)	18.9 (11.2 to 31.5)	1 (0 to 1)	0.1 (0.1 to 0.2)	-13.4 (-43.5 to 42)	-20.8 (-38 to -4.1)	-51.5 (-67.3 to -19.2)	-21.8 (-46.2 to 15)
		Female	66 (33 to 124)	28 (16 to 47.3)	0 (0 to 1)	0.2 (0.1 to 0.3)	68 (41 to 110)	22.2 (13.1 to 36)	0 (0 to 0)	0.1 (0.1 to 0.2)	3.4 (-34.6 to 61.6)	-20.5 (-35.6 to -6.1)	-44.1 (-64.6 to 3.4)	-13.2 (-42.7 to 30.2)
		Male	63 (26 to 130)	20.1 (10.1 to 37.7)	1 (0 to 1)	0.2 (0.1 to 0.4)	44 (26 to 73)	15.7 (8.5 to 28.2)	0 (0 to 1)	0.1 (0 to 0.2)	-31 (-55.7 to 17)	-21.9 (-42.5 to 2.3)	-57.7 (-71.7 to -34.5)	-29.5 (-52.6 to 5.5)
	Respiratory infections and tuberculosis	Both	7544 (5778 to 10017)	1222.6 (972.8 to 1535.2)	90 (70 to 117)	22.2 (18.3 to 30.6)	1908 (1563 to 2422)	378.3 (312.3 to 486)	42 (36 to 63)	11.4 (9.5 to 17.5)	-74.7 (-82.2 to -64.5)	-69.1 (-77 to -59.5)	-53.2 (-66.3 to -32.6)	-48.8 (-58.8 to -36.5)
		Female	4049 (2889 to 5733)	1302.3 (989.2 to 1732.7)	47 (34 to 66)	22.2 (17.6 to 30.9)	869 (690 to 1127)	359.5 (291.5 to 458.6)	18 (15 to 27)	10.7 (8.6 to 16.3)	-78.5 (-85.8 to -67.5)	-72.4 (-80.7 to -60.9)	-61.5 (-73.9 to -40.2)	-51.8 (-63.4 to -34.8)
		Male	3495 (2514 to 4883)	1142.7 (870.5 to 1516.8)	43 (32 to 59)	22.2 (17.7 to 35.3)	1039 (846 to 1386)	394.8 (324.4 to 531.5)	24 (20 to 40)	11.9 (9.6 to 20.5)	-70.3 (-79.8 to -57.4)	-65.5 (-75 to -54.2)	-44.1 (-61.6 to -18.5)	-46.4 (-58.8 to -30.6)
	Tuberculosis	Both	346 (253 to 438)	94 (67.7 to 120.6)	6 (4 to 8)	2.8 (2 to 3.7)	105 (90 to 121)	18.8 (16.4 to 21.5)	3 (2 to 3)	0.6 (0.6 to 0.8)	-69.8 (-77.4 to -55.4)	-80 (-85.1 to -70.3)	-55 (-67.3 to -29.5)	-76.8 (-83.4 to -64.4)

Province	Cause	Sex	1990				2019				1990-2019 percent change (%)			
			DALYs		Death		DALYs		Death		DALYs		Death	
			Number	Rate	Number	Rate	Number	Rate	Number	Rate	Number	Rate	Number	Rate
		Female	164 (85 to 251)	85.2 (44.1 to 135.1)	3 (1 to 4)	2.3 (1 to 4)	41 (34 to 52)	15.5 (12.8 to 19.7)	1 (1 to 1)	0.5 (0.4 to 0.7)	-74.7 (-84.6 to -41.7)	-81.9 (-89.6 to -57.1)	-59.6 (-78.5 to 14.9)	-78.1 (-88.8 to -35.2)
		Male	182 (145 to 233)	101.1 (80.5 to 132.1)	4 (3 to 5)	3.2 (2.4 to 4.3)	63 (53 to 75)	22 (18.6 to 26)	2 (1 to 2)	0.8 (0.6 to 0.9)	-65.4 (-75.2 to -53.7)	-78.2 (-84.5 to -70.3)	-51.6 (-66 to -32.4)	-75.8 (-83.1 to -65.8)
	Lower respiratory infections	Both	6594 (4887 to 9043)	1009.5 (778.8 to 1323.8)	84 (64 to 111)	19.5 (15.9 to 27.8)	1125 (956 to 1548)	244.6 (205.6 to 344.3)	39 (33 to 60)	10.7 (8.9 to 16.8)	-82.9 (-88.6 to -74.3)	-75.8 (-83.2 to -66.5)	-53.1 (-67.2 to -30.7)	-44.9 (-56.3 to -31.2)
		Female	3604 (2471 to 5255)	1102.7 (783 to 1550.6)	45 (32 to 64)	19.9 (15.5 to 28.3)	510 (419 to 709)	234.3 (191.4 to 327.7)	17 (14 to 26)	10.2 (8.1 to 15.8)	-85.8 (-91.2 to -76)	-78.8 (-86.3 to -67.4)	-61.6 (-74.9 to -38.8)	-48.8 (-62.2 to -30.7)
		Male	2990 (2061 to 4363)	917.9 (661.4 to 1288.3)	39 (28 to 55)	19 (14.8 to 32)	615 (510 to 907)	253 (208.3 to 383.6)	22 (18 to 38)	11.1 (8.9 to 19.7)	-79.4 (-87.1 to -67.8)	-72.4 (-81.8 to -60.4)	-43.5 (-62.2 to -15)	-41.5 (-55.3 to -23.4)
	Upper respiratory infections	Both	426 (255 to 674)	84.9 (51 to 131.6)	0 (0 to 0)	0 (0 to 0.1)	506 (306 to 794)	85.3 (51.6 to 133.9)	0 (0 to 0)	0 (0 to 0)	18.7 (7.3 to 30.4)	0.5 (-5.8 to 5.7)	30.7 (-94.6 to 3897.4)	53.1 (-93.6 to 3144.7)
		Female	204 (122 to 322)	82.9 (49.8 to 128.5)	0 (0 to 0)	0 (0 to 0)	240 (145 to 379)	82.9 (49.8 to 130.9)	0 (0 to 0)	0 (0 to 0)	18.1 (5.3 to 33.8)	0 (-7.6 to 7.1)	2.5 (-97 to 4464.4)	45 (-95.5 to 3284.7)
		Male	223 (134 to 356)	86.7 (52.4 to 134.6)	0 (0 to 0)	0 (0 to 0.1)	266 (157 to 417)	87.5 (52.4 to 138.5)	0 (0 to 0)	0 (0 to 0)	19.3 (7 to 32.8)	1 (-7 to 8.9)	48.2 (-93.1 to 4195.9)	59.2 (-92.5 to 3305.4)
	Otitis media	Both	178 (102 to 293)	34.3 (20 to 57)	0 (0 to 0)	0 (0 to 0)	172 (98 to 286)	29.6 (16.6 to 49.6)	0 (0 to 0)	0 (0 to 0)	-3.3 (-14.3 to 9.8)	-13.7 (-19.5 to -7.9)	26 (-83.4 to 286.9)	20.4 (-82.1 to 224.7)
		Female	78 (43 to 128)	31.4 (17.8 to 52.2)	0 (0 to 0)	0 (0 to 0)	77 (43 to 126)	26.8 (14.9 to 44.9)	0 (0 to 0)	0 (0 to 0)	-1.6 (-14.9 to 14.1)	-14.6 (-22.2 to -6.9)	422.1 (-75.2 to 1784.5)	154.7 (-80.5 to 869.1)
		Male	100 (57 to 166)	37 (21.6 to 61.1)	0 (0 to 0)	0 (0 to 0)	95 (53 to 162)	32.3 (18 to 53.8)	0 (0 to 0)	0 (0 to 0)	-4.6 (-16.7 to 10.3)	-12.9 (-20.8 to -4.2)	-81 (-92.5 to -41.3)	-79.9 (-91.5 to -42.2)
	Enteric infections	Both	5446 (3728 to 7849)	821.3 (590.9 to 1134.4)	58 (39 to 86)	11.4 (7.6 to 16)	1405 (1054 to 1823)	273.3 (204.6 to 354.9)	8 (6 to 11)	1.9 (1.4 to 2.9)	-74.2 (-83.3 to -60.9)	-66.7 (-77.3 to -52.3)	-86.7 (-92 to -77.7)	-83.1 (-89 to -74.5)
		Female	2601 (1592 to 4070)	800.4 (518 to 1219.7)	28 (16 to 45)	11.3 (6.4 to 18.8)	656 (483 to 857)	262 (195.4 to 341.5)	3 (2 to 5)	1.7 (1 to 3)	-74.8 (-84.8 to -57.1)	-67.3 (-79.5 to -48.4)	-88.6 (-94.4 to -76.5)	-85.1 (-91.7 to -72.1)
		Male	2844 (1782 to 4538)	839.3 (550.5 to 1287.6)	31 (18 to 50)	11.4 (7.3 to 16.9)	750 (552 to 980)	284 (210 to 369.2)	5 (3 to 7)	2.1 (1.5 to 3.4)	-73.6 (-84.3 to -55.9)	-66.2 (-78.9 to -47)	-84.9 (-91.7 to -71.4)	-81.2 (-88.4 to -70.5)
	Diarrheal diseases	Both	5070 (3440 to 7438)	762.2 (540 to 1076.8)	53 (35 to 80)	10.5 (6.8 to 15.1)	1290 (954 to 1711)	251.6 (186.9 to 331.2)	6 (4 to 9)	1.6 (1.1 to 2.6)	-74.6 (-84 to -60.5)	-67 (-78.1 to -51.7)	-88.7 (-93.6 to -80.3)	-84.8 (-90.4 to -76)
		Female	2416 (1429 to 3879)	741.3 (465.6 to 1157.5)	25 (14 to 42)	10.4 (5.5 to 17.8)	610 (444 to 813)	244.1 (178.8 to 324.3)	2 (1 to 5)	1.4 (0.7 to 2.7)	-74.8 (-85.3 to -55.4)	-67.1 (-79.8 to -46)	-90.3 (-95.9 to -77.4)	-86.4 (-93.1 to -72.7)
		Male	2654 (1594 to 4324)	780.3 (494.7 to 1220.4)	28 (16 to 47)	10.5 (6.5 to 16)	680 (487 to 904)	258.7 (189.5 to 343.8)	4 (2 to 6)	1.8 (1.1 to 3)	-74.4 (-85.1 to -55.2)	-66.9 (-79.8 to -46.4)	-87.3 (-93.5 to -73.9)	-83.3 (-90.2 to -72.4)
	Typhoid and paratyphoid	Both	277 (109 to 544)	43.8 (17.5 to 84.8)	4 (1 to 7)	0.6 (0.2 to 1.2)	85 (35 to 166)	15.9 (6.4 to 31.4)	1 (1 to 2)	0.2 (0.1 to 0.4)	-69.4 (-76.5 to -58.8)	-63.7 (-71.5 to -52.8)	-66 (-73.7 to -53.8)	-63.9 (-71.1 to -53.7)
		Female	126 (49 to 248)	40.8 (16.2 to 78.3)	2 (1 to 3)	0.6 (0.2 to 1.1)	33 (14 to 63)	12.8 (5.2 to 25.3)	0 (0 to 1)	0.2 (0.1 to 0.4)	-74 (-80.8 to -62.8)	-68.6 (-76.8 to -55.2)	-71.1 (-78.3 to -58.1)	-69 (-76.7 to -57.3)
		Male	151 (58 to 300)	46.7 (18.4 to 91.7)	2 (1 to 4)	0.7 (0.3 to 1.3)	52 (21 to 102)	18.7 (7.5 to 36.9)	1 (0 to 2)	0.3 (0.1 to 0.5)	-65.5 (-74.7 to -51.6)	-59.9 (-70.1 to -45.6)	-61.8 (-71.9 to -45.9)	-59.8 (-69.6 to -45.8)
	Invasive Non-typhoidal Salmonella (iNTS)	Both	80 (42 to 134)	12.5 (6.6 to 20.4)	1 (1 to 2)	0.2 (0.1 to 0.3)	29 (14 to 51)	5.4 (2.7 to 9.5)	0 (0 to 1)	0.1 (0 to 0.2)	-64.1 (-76.3 to -48.1)	-56.8 (-70.6 to -40)	-53.8 (-68.3 to -34.9)	-54.4 (-65.8 to -40.8)
		Female	48 (24 to 81)	15 (7.6 to 25)	1 (0 to 1)	0.2 (0.1 to 0.4)	12 (6 to 21)	4.6 (2.3 to 7.9)	0 (0 to 0)	0.1 (0 to 0.1)	-75.3 (-84.6 to -62)	-69.6 (-79.9 to -54.3)	-66.5 (-78.7 to -48.9)	-66.1 (-75.9 to -52.8)

Province	Cause	Sex	1990				2019				1990-2019 percent change (%)			
			DALYs		Death		DALYs		Death		DALYs		Death	
			Number	Rate	Number	Rate	Number	Rate	Number	Rate	Number	Rate	Number	Rate
		Male	32 (17 to 54)	10.1 (5.5 to 16.7)	0 (0 to 1)	0.2 (0.1 to 0.3)	17 (8 to 31)	6.2 (3 to 11.1)	0 (0 to 1)	0.1 (0.1 to 0.2)	-47.4 (-66.1 to -23)	-38.9 (-59.4 to -15.8)	-36 (-55.8 to -10.2)	-39.3 (-56.3 to -17.5)
	Other intestinal infectious diseases	Both	20 (4 to 43)	2.7 (0.6 to 5.8)	0 (0 to 0)	0 (0 to 0.1)	2 (2 to 3)	0.4 (0.3 to 0.7)	0 (0 to 0)	0 (0 to 0)	-88.2 (-95.2 to -32.1)	-83.6 (-93.2 to -25.9)	-89.2 (-96.2 to -32.4)	-84.9 (-94.1 to -42.7)
		Female	12 (1 to 32)	3.3 (0.3 to 8.6)	0 (0 to 0)	0 (0 to 0.1)	1 (1 to 2)	0.4 (0.2 to 0.7)	0 (0 to 0)	0 (0 to 0)	-90.6 (-97.1 to 29.8)	-86.4 (-95.9 to 34.9)	-91.6 (-97.9 to 76.9)	-87.1 (-96.6 to 7.8)
		Male	8 (1 to 21)	2.2 (0.4 to 5.5)	0 (0 to 0)	0 (0 to 0.1)	1 (1 to 2)	0.4 (0.2 to 0.7)	0 (0 to 0)	0 (0 to 0)	-84.6 (-94.9 to 32.7)	-79.5 (-93.1 to 22.8)	-85.6 (-96.1 to 67)	-81.8 (-94.7 to -8.2)
	Neglected tropical diseases and malaria	Both	991 (163 to 10480)	239.6 (31.9 to 2605.2)	15 (1 to 180)	4.8 (0.2 to 55.9)	93 (65 to 135)	17 (11.7 to 25.1)	1 (0 to 1)	0.1 (0.1 to 0.2)	-90.6 (-99.1 to -48)	-92.9 (-99.4 to -50.3)	-96.3 (-99.7 to -35.7)	-97.5 (-99.8 to -47.6)
		Female	478 (71 to 5039)	238.3 (30.6 to 2678.3)	7 (0 to 87)	4.8 (0.1 to 58.9)	41 (27 to 62)	15 (9.7 to 23.1)	0 (0 to 0)	0.1 (0 to 0.1)	-91.5 (-99.3 to -46.2)	-93.7 (-99.5 to -55.1)	-98.3 (-99.8 to -43.4)	-98.9 (-99.9 to -53.2)
		Male	513 (86 to 5189)	242.6 (31.1 to 2540.4)	8 (1 to 92)	4.9 (0.3 to 52.8)	53 (33 to 80)	18.9 (11.8 to 29.9)	0 (0 to 1)	0.2 (0.1 to 0.3)	-89.8 (-99 to -40.3)	-92.2 (-99.2 to -39.4)	-94.7 (-99.5 to -17.4)	-96.4 (-99.7 to -35.5)
	Malaria	Both	713 (5 to 10012)	190.1 (1.1 to 2555.4)	13 (0 to 177)	4.4 (0 to 54.8)	7 (5 to 9)	1.1 (0.8 to 1.5)	0 (0 to 0)	0 (0 to 0)	-99 (-99.9 to 17)	-99.4 (-100 to -8)	-100 (-100 to -100)	-100 (-100 to -100)
		Female	359 (3 to 4945)	191.9 (1.2 to 2643.2)	6 (0 to 86)	4.5 (0 to 58.6)	3 (2 to 5)	1.1 (0.8 to 1.5)	0 (0 to 0)	0 (0 to 0)	-99 (-99.9 to 18.3)	-99.4 (-100 to -11.2)	-100 (-100 to -100)	-100 (-100 to -100)
		Male	353 (3 to 5036)	189.9 (1 to 2493.1)	7 (0 to 91)	4.3 (0 to 52.4)	3 (2 to 5)	1.1 (0.8 to 1.5)	0 (0 to 0)	0 (0 to 0)	-99 (-99.9 to 18.2)	-99.4 (-100 to -3.5)	-100 (-100 to -100)	-100 (-100 to -100)
	Leishmaniasis	Both	62 (0 to 649)	10 (0.1 to 100)	1 (0 to 9)	0.2 (0 to 1.5)	4 (0 to 34)	0.7 (0 to 6.3)	0 (0 to 1)	0 (0 to 0.1)	-93.4 (-94.7 to 1)	-92.7 (-94.3 to -4.4)	-92.8 (-93.9 to -87.6)	-93.4 (-94.6 to -91.7)
		Female	21 (0 to 213)	6.8 (0.1 to 65.1)	0 (0 to 3)	0.1 (0 to 0.9)	1 (0 to 10)	0.5 (0.1 to 3.9)	0 (0 to 0)	0 (0 to 0.1)	-93.1 (-95.3 to 37.5)	-92.3 (-94.7 to 16.1)	-93.1 (-94.4 to -86.7)	-93.6 (-95 to -91.2)
		Male	41 (0 to 439)	13 (0.1 to 134.2)	1 (0 to 6)	0.2 (0 to 2)	3 (0 to 24)	0.9 (0 to 8.5)	0 (0 to 0)	0 (0 to 0.1)	-93.6 (-94.6 to -30.9)	-92.9 (-94.3 to -25.9)	-92.7 (-93.7 to -87.3)	-93.2 (-94.5 to -91.2)
	Schistosomiasis	Both	0 (0 to 0)	0 (0 to 0)	0 (0 to 0)	0 (0 to 0)	0 (0 to 0)	0 (0 to 0)	0 (0 to 0)	0 (0 to 0)	0 (0 to 0)	0 (0 to 0)	0 (0 to 0)	0 (0 to 0)
		Female	0 (0 to 0)	0 (0 to 0)	0 (0 to 0)	0 (0 to 0)	0 (0 to 0)	0 (0 to 0)	0 (0 to 0)	0 (0 to 0)	0 (0 to 0)	0 (0 to 0)	0 (0 to 0)	0 (0 to 0)
		Male	0 (0 to 0)	0 (0 to 0)	0 (0 to 0)	0 (0 to 0)	0 (0 to 0)	0 (0 to 0)	0 (0 to 0)	0 (0 to 0)	0 (0 to 0)	0 (0 to 0)	0 (0 to 0)	0 (0 to 0)
	Cystic echinococcosis	Both	30 (14 to 51)	6.9 (4.2 to 10)	0 (0 to 1)	0.1 (0.1 to 0.2)	17 (12 to 25)	2.8 (2 to 3.9)	0 (0 to 0)	0 (0 to 0)	-42.8 (-69 to 21.4)	-59.2 (-74.8 to -30.1)	-66.7 (-85.1 to -18.2)	-75.6 (-88.3 to -48.5)
		Female	13 (5 to 26)	6.2 (3.1 to 10.1)	0 (0 to 0)	0.1 (0 to 0.2)	9 (6 to 14)	3 (2 to 4.4)	0 (0 to 0)	0 (0 to 0)	-31.3 (-69.2 to 93.7)	-51.7 (-73.5 to -4.8)	-63.3 (-89.6 to 65)	-70.6 (-91.5 to -4.6)
		Male	17 (6 to 32)	7.4 (3.9 to 11.9)	0 (0 to 0)	0.1 (0.1 to 0.2)	8 (5 to 12)	2.6 (1.6 to 3.7)	0 (0 to 0)	0 (0 to 0.1)	-52.1 (-77.5 to 30.8)	-64.8 (-80.9 to -28.5)	-69 (-90.5 to 0.4)	-78.1 (-92.7 to -38.6)
	Trachoma	Both	4 (2 to 6)	2.7 (1.6 to 4.1)	--	--	1 (1 to 3)	0.3 (0.2 to 0.6)	--	--	-64.2 (-75.2 to -50.9)	-87.4 (-91.3 to -82.9)	--	--
		Female	3 (2 to 5)	4.9 (2.9 to 7.7)	--	--	1 (0 to 2)	0.5 (0.2 to 0.9)	--	--	-68.6 (-79.2 to -54.8)	-90.4 (-93.4 to -86.2)	--	--
		Male	1 (0 to 2)	1 (0.5 to 1.8)	--	--	0 (0 to 1)	0.2 (0.1 to 0.4)	--	--	-48.9 (-65.5 to -23)	-79.5 (-86.3 to -70.2)	--	--
	Rabies	Both	1 (0 to 2)	0.1 (0 to 0.5)	0 (0 to 0)	0 (0 to 0)	0 (0 to 1)	0 (0 to 0.1)	0 (0 to 0)	0 (0 to 0)	-57.8 (-86.4 to 22.3)	-70.3 (-90.7 to -24)	-46.3 (-82.8 to 36)	-69 (-89.9 to -32.6)

Province	Cause	Sex	1990				2019				1990-2019 percent change (%)			
			DALYs		Death		DALYs		Death		DALYs		Death	
			Number	Rate	Number	Rate	Number	Rate	Number	Rate	Number	Rate	Number	Rate
		Female	0 (0 to 0)	0 (0 to 0.1)	0 (0 to 0)	0 (0 to 0)	0 (0 to 0)	0 (0 to 0)	0 (0 to 0)	0 (0 to 0)	-53.2 (-79.7 to 42.7)	-70.2 (-83.2 to -38)	-36.4 (-65.3 to 44.1)	-69.2 (-84.5 to -44.4)
		Male	1 (0 to 2)	0.2 (0.1 to 0.9)	0 (0 to 0)	0 (0 to 0)	0 (0 to 1)	0.1 (0 to 0.2)	0 (0 to 0)	0 (0 to 0)	-58.1 (-87.7 to 28)	-69.7 (-91.4 to -15.5)	-47.2 (-84.9 to 40.2)	-67.7 (-90.6 to -19.8)
	Intestinal nematode infections	Both	45 (18 to 83)	7.4 (3.6 to 13)	0 (0 to 1)	0 (0 to 0.1)	6 (3 to 9)	1 (0.5 to 1.7)	0 (0 to 0)	0 (0 to 0)	-87.6 (-94.6 to -68.8)	-86.9 (-93.6 to -72.9)	-94.3 (-98.3 to -54.6)	-90.8 (-96.9 to -56.1)
		Female	25 (8 to 54)	8.8 (3.7 to 17.1)	0 (0 to 0)	0 (0 to 0.1)	3 (2 to 6)	1.2 (0.6 to 2.1)	0 (0 to 0)	0 (0 to 0)	-86.2 (-94.7 to -60.8)	-86.6 (-94.1 to -68.5)	-94.7 (-98.9 to 49.7)	-91 (-98.1 to -4.5)
		Male	20 (6 to 45)	6.1 (2.1 to 12.1)	0 (0 to 0)	0 (0 to 0.1)	2 (1 to 4)	0.8 (0.4 to 1.4)	0 (0 to 0)	0 (0 to 0)	-89.2 (-96.2 to -61.7)	-87.3 (-95 to -62.7)	-93.9 (-98.7 to 6.6)	-90.6 (-97.7 to -39.7)
	Food-borne trematodiases	Both	1 (0 to 3)	0.5 (0.1 to 1)	--	--	6 (1 to 13)	1 (0.2 to 2.1)	--	--	339.9 (225.3 to 460.3)	107.8 (61.7 to 157.8)	--	--
		Female	1 (0 to 1)	0.4 (0.1 to 0.8)	--	--	2 (1 to 5)	0.8 (0.2 to 1.7)	--	--	341.2 (218.2 to 478.8)	101.7 (49.1 to 162.7)	--	--
		Male	1 (0 to 2)	0.6 (0.1 to 1.2)	--	--	4 (1 to 8)	1.2 (0.3 to 2.6)	--	--	339.1 (182.2 to 516.6)	115.2 (48.7 to 193.8)	--	--
	Leprosy	Both	0 (0 to 0)	0.1 (0 to 0.1)	--	--	0 (0 to 0)	0 (0 to 0)	--	--	53.2 (40.4 to 65.7)	-41.1 (-45.6 to -36.6)	--	--
		Female	0 (0 to 0)	0 (0 to 0.1)	--	--	0 (0 to 0)	0 (0 to 0)	--	--	54.3 (32.6 to 76.6)	-44.2 (-51.1 to -37.4)	--	--
		Male	0 (0 to 0)	0.1 (0 to 0.1)	--	--	0 (0 to 0)	0 (0 to 0.1)	--	--	52.7 (37.8 to 69.8)	-37 (-42.9 to -30.3)	--	--
	Other neglected tropical diseases	Both	136 (86 to 205)	21.9 (14.4 to 31.9)	0 (0 to 1)	0.1 (0.1 to 0.2)	52 (32 to 79)	10 (6.3 to 15.4)	0 (0 to 0)	0.1 (0 to 0.1)	-62.1 (-75 to -38.9)	-54.3 (-69 to -30.2)	-24.3 (-62.9 to 58.7)	-32.8 (-59.9 to 27.9)
		Female	56 (29 to 97)	19.2 (10.6 to 32)	0 (0 to 0)	0 (0 to 0.2)	20 (10 to 36)	7.9 (3.9 to 14.2)	0 (0 to 0)	0 (0 to 0.1)	-64.7 (-80.8 to -37.8)	-59 (-76.5 to -33.2)	-42 (-64.3 to 36.4)	-51.3 (-68 to 5.9)
		Male	80 (45 to 129)	24.4 (14.2 to 38)	0 (0 to 1)	0.2 (0.1 to 0.3)	32 (16 to 53)	12 (6.1 to 20.1)	0 (0 to 0)	0.1 (0 to 0.2)	-60.2 (-78.9 to -28.4)	-50.9 (-72.6 to -15.5)	-21.5 (-64.4 to 68.9)	-28.8 (-58.8 to 34.5)
	Other infectious diseases	Both	9030 (4640 to 15840)	1275.5 (703.5 to 2149.4)	107 (56 to 187)	17.7 (10.5 to 28.1)	651 (540 to 835)	123.2 (101.1 to 163.9)	12 (10 to 14)	2.5 (2.2 to 3.1)	-92.8 (-95.8 to -86)	-90.3 (-94.3 to -82.3)	-89 (-93.7 to -78.9)	-85.6 (-90.9 to -75.3)
		Female	4894 (2404 to 8581)	1382.5 (731.2 to 2354.5)	57 (28 to 100)	18.1 (10.3 to 29.5)	268 (211 to 369)	106 (82.2 to 153.5)	4 (3 to 5)	1.8 (1.5 to 2.4)	-94.5 (-96.9 to -88.8)	-92.3 (-95.6 to -85.1)	-93.1 (-96.1 to -86)	-89.9 (-94 to -81.3)
		Male	4136 (2140 to 7374)	1167.3 (646.8 to 1993.3)	50 (27 to 87)	17 (10.1 to 27.6)	383 (318 to 482)	140 (114.9 to 181.3)	8 (7 to 10)	3.2 (2.7 to 3.9)	-90.7 (-94.8 to -82.1)	-88 (-93 to -78.5)	-84.4 (-91 to -71)	-81.1 (-88.5 to -67.9)
	Meningitis	Both	936 (678 to 1329)	142.9 (107.6 to 196.4)	11 (8 to 16)	2.1 (1.6 to 2.8)	170 (141 to 205)	31.5 (25.9 to 38.5)	3 (2 to 3)	0.6 (0.5 to 0.7)	-81.8 (-87.2 to -73.3)	-78 (-84 to -68.3)	-73.8 (-81.7 to -60.7)	-69.9 (-77.4 to -56.1)
		Female	421 (284 to 687)	130 (92 to 201.2)	5 (3 to 8)	1.8 (1.2 to 2.7)	71 (56 to 93)	26.9 (21.1 to 36.6)	1 (1 to 1)	0.4 (0.3 to 0.6)	-83.1 (-89.4 to -73.5)	-79.3 (-86.4 to -69.2)	-80.5 (-88.1 to -69.4)	-75.5 (-84.2 to -64.5)
		Male	515 (359 to 751)	154.6 (112.5 to 212.6)	6 (4 to 9)	2.4 (1.7 to 3.1)	99 (80 to 123)	35.7 (28.9 to 44.7)	2 (2 to 3)	0.8 (0.6 to 1)	-80.8 (-87.4 to -69.5)	-76.9 (-84.4 to -64.1)	-68.7 (-79.7 to -48.6)	-66 (-76.4 to -45.4)
	Encephalitis	Both	99 (70 to 146)	17.1 (12.7 to 25.5)	1 (1 to 2)	0.3 (0.2 to 0.4)	67 (55 to 87)	12.4 (10.2 to 15.8)	1 (1 to 1)	0.2 (0.2 to 0.3)	-31.5 (-53.7 to -2.2)	-27.5 (-48.5 to 2)	-6.7 (-38.9 to 39.3)	-13.5 (-43.2 to 29.3)

Province	Cause	Sex	1990				2019				1990-2019 percent change (%)			
			DALYs		Death		DALYs		Death		DALYs		Death	
			Number	Rate	Number	Rate	Number	Rate	Number	Rate	Number	Rate	Number	Rate
		Female	47 (30 to 73)	16.6 (11.1 to 25.4)	0 (0 to 1)	0.2 (0.1 to 0.4)	28 (21 to 40)	10.3 (7.7 to 14.8)	0 (0 to 1)	0.2 (0.1 to 0.2)	-41.2 (-64.1 to -8.8)	-37.8 (-60 to -7.3)	-30.1 (-58.7 to 15.3)	-31 (-55.6 to 11)
		Male	52 (34 to 80)	17.6 (12.3 to 25.7)	1 (0 to 1)	0.3 (0.2 to 0.5)	40 (32 to 50)	14.4 (11.5 to 18.2)	1 (1 to 1)	0.3 (0.2 to 0.3)	-22.7 (-52.5 to 23.5)	-18.3 (-47 to 23.8)	12.3 (-30.5 to 87.7)	-0.5 (-40.6 to 61.3)
	Diphtheria	Both	23 (9 to 48)	3.1 (1.3 to 6.3)	0 (0 to 1)	0 (0 to 0.1)	0 (0 to 1)	0.1 (0 to 0.1)	0 (0 to 0)	0 (0 to 0)	-98.2 (-99.3 to -94.8)	-97.5 (-99 to -93.3)	-97.7 (-99 to -93.8)	-97 (-98.7 to -92.9)
		Female	11 (3 to 28)	3.1 (0.8 to 7.6)	0 (0 to 0)	0 (0 to 0.1)	0 (0 to 0)	0.1 (0 to 0.1)	0 (0 to 0)	0 (0 to 0)	-98.6 (-99.6 to -92.5)	-97.9 (-99.5 to -89.6)	-98.2 (-99.5 to -91)	-97.6 (-99.3 to -89.4)
		Male	11 (3 to 27)	3 (0.9 to 7)	0 (0 to 0)	0 (0 to 0.1)	0 (0 to 0)	0.1 (0 to 0.2)	0 (0 to 0)	0 (0 to 0)	-97.8 (-99.2 to -91)	-97 (-99 to -89.8)	-97.1 (-98.9 to -89.5)	-96.5 (-98.7 to -89)
	Whooping cough	Both	1474 (72 to 6320)	191.3 (9.4 to 817.9)	17 (1 to 73)	2.2 (0.1 to 9.4)	45 (3 to 207)	10 (0.7 to 45.8)	1 (0 to 2)	0.1 (0 to 0.5)	-96.9 (-99.8 to -29.3)	-94.8 (-99.7 to 20.9)	-97 (-99.9 to 4.8)	-94.9 (-99.9 to 77.8)
		Female	862 (42 to 3668)	228.7 (11.1 to 974.5)	10 (0 to 42)	2.6 (0.1 to 11.2)	25 (2 to 116)	11.6 (0.8 to 53.1)	0 (0 to 1)	0.1 (0 to 0.6)	-97 (-99.8 to -27.5)	-94.9 (-99.7 to 23)	-97.1 (-99.9 to -3.1)	-95.1 (-99.9 to 63.8)
		Male	612 (31 to 2736)	155.6 (7.8 to 689.2)	7 (0 to 32)	1.8 (0.1 to 8)	20 (1 to 90)	8.4 (0.6 to 38.4)	0 (0 to 1)	0.1 (0 to 0.4)	-96.8 (-99.8 to -22.4)	-94.6 (-99.7 to 31.8)	-96.8 (-99.9 to 16.7)	-94.7 (-99.9 to 94.8)
	Tetanus	Both	520 (113 to 2006)	72.3 (19.3 to 268.3)	6 (2 to 23)	1 (0.3 to 3.3)	8 (5 to 13)	1.6 (1 to 2.7)	0 (0 to 0)	0 (0 to 0.1)	-98.5 (-99.7 to -92)	-97.8 (-99.5 to -89.9)	-97.6 (-99.5 to -89.6)	-96.7 (-99.2 to -88.9)
		Female	455 (82 to 1956)	128.7 (28.2 to 535.3)	5 (1 to 22)	1.7 (0.5 to 6.1)	5 (3 to 9)	2.2 (1.1 to 4)	0 (0 to 0)	0 (0 to 0.1)	-98.9 (-99.8 to -92.5)	-98.3 (-99.7 to -90)	-98.2 (-99.7 to -89.6)	-97.3 (-99.5 to -89.5)
		Male	65 (16 to 220)	18.7 (4.8 to 60.5)	1 (0 to 3)	0.3 (0.1 to 0.9)	3 (2 to 5)	1 (0.7 to 1.9)	0 (0 to 0)	0 (0 to 0)	-95.8 (-98.8 to -81.2)	-94.6 (-98.5 to -77.1)	-93.5 (-98.2 to -74.2)	-92.9 (-98 to -73.2)
	Measles	Both	5199 (1799 to 11159)	668.9 (231.4 to 1440)	60 (21 to 130)	7.8 (2.7 to 16.8)	42 (13 to 104)	9.2 (2.7 to 22.6)	0 (0 to 1)	0.1 (0 to 0.3)	-99.2 (-99.6 to -98.4)	-98.6 (-99.4 to -97.3)	-99.2 (-99.7 to -98.4)	-98.7 (-99.4 to -97.3)
		Female	2747 (913 to 5895)	724.2 (239.7 to 1562.8)	32 (11 to 69)	8.4 (2.8 to 18.2)	21 (6 to 53)	9.5 (2.9 to 23.6)	0 (0 to 1)	0.1 (0 to 0.3)	-99.2 (-99.7 to -98.4)	-98.7 (-99.4 to -97.4)	-99.2 (-99.7 to -98.5)	-98.7 (-99.5 to -97.4)
		Male	2451 (829 to 5253)	616.1 (207.8 to 1317.3)	28 (10 to 61)	7.2 (2.4 to 15.3)	21 (6 to 51)	8.9 (2.7 to 21.7)	0 (0 to 1)	0.1 (0 to 0.2)	-99.1 (-99.6 to -98.3)	-98.6 (-99.4 to -97.1)	-99.2 (-99.6 to -98.3)	-98.6 (-99.4 to -97.2)
	Varicella and herpes zoster	Both	57 (34 to 88)	10.8 (7.5 to 15.2)	1 (0 to 1)	0.2 (0.2 to 0.3)	24 (17 to 34)	4.6 (3.2 to 6.3)	0 (0 to 0)	0.1 (0 to 0.1)	-57.6 (-76.1 to -25)	-57.6 (-72.8 to -33.6)	-66.7 (-81.1 to -40.8)	-72.1 (-83 to -54.5)
		Female	29 (13 to 54)	11.1 (6.6 to 17.8)	0 (0 to 1)	0.2 (0.1 to 0.4)	12 (8 to 17)	4.6 (3.1 to 6.7)	0 (0 to 0)	0.1 (0 to 0.1)	-58.6 (-80.4 to -5.2)	-58.2 (-77.5 to -25.3)	-68.6 (-85 to -29)	-71.4 (-85.9 to -43.2)
		Male	28 (14 to 51)	10.5 (6.7 to 16.7)	0 (0 to 1)	0.2 (0.1 to 0.4)	12 (8 to 17)	4.5 (3.1 to 6.5)	0 (0 to 0)	0.1 (0 to 0.1)	-56.5 (-78.8 to -11.2)	-57 (-75.6 to -27.6)	-64.8 (-83 to -27)	-72.8 (-86.5 to -47.1)
	Acute hepatitis	Both	530 (284 to 906)	133.3 (74.7 to 225.6)	9 (5 to 16)	3.6 (2 to 6.5)	157 (130 to 190)	27.6 (23.1 to 33.2)	4 (3 to 5)	0.8 (0.6 to 1)	-70.4 (-84.1 to -39.7)	-79.3 (-88.6 to -60.1)	-57.2 (-77.4 to -14)	-77.5 (-88.8 to -55.7)
		Female	228 (91 to 389)	103.1 (40.5 to 163.5)	3 (1 to 6)	2.5 (0.9 to 4.3)	36 (29 to 45)	13.3 (10.6 to 16.4)	1 (1 to 1)	0.3 (0.2 to 0.4)	-84.1 (-91.5 to -56)	-87.1 (-92.5 to -64.4)	-79.1 (-88.6 to -35.6)	-87.7 (-93.6 to -61)
		Male	302 (150 to 640)	155.9 (82.9 to 314.9)	6 (3 to 12)	4.3 (2.3 to 9.1)	121 (96 to 151)	41.8 (33.1 to 52.6)	3 (2 to 4)	1.3 (1 to 1.6)	-60 (-82.3 to -14.5)	-73.2 (-87.6 to -46.1)	-44.4 (-75.7 to 18.3)	-70.4 (-87.1 to -38.6)
	Other unspecified infectious diseases	Both	193 (115 to 449)	35.9 (21.8 to 75)	2 (1 to 5)	0.5 (0.3 to 1.3)	137 (106 to 174)	26.3 (20.5 to 33.8)	2 (2 to 3)	0.6 (0.4 to 0.7)	-29.3 (-70.2 to 23.3)	-26.7 (-66.2 to 21.7)	44.8 (-53.5 to 243.9)	6.1 (-56.8 to 147.9)
		Female	93 (55 to 210)	37 (22.8 to 69.7)	1 (0 to 2)	0.6 (0.3 to 1.5)	69 (49 to 90)	27.5 (19 to 36.4)	1 (1 to 2)	0.6 (0.4 to 0.7)	-25.8 (-73.2 to 32.9)	-25.7 (-67.7 to 29.8)	52 (-58.3 to 284.1)	0.5 (-66.4 to 172.2)
		Male	101 (52 to 266)	35.2 (19.8 to 85.4)	1 (0 to 3)	0.5 (0.2 to 1.5)	68 (50 to 104)	25.1 (18.2 to 39)	1 (1 to 2)	0.6 (0.4 to 0.8)	-32.6 (-71.2 to 25.9)	-28.6 (-66.9 to 24.5)	38.6 (-52.8 to 248.7)	10 (-55.8 to 149.3)

Province	Cause	Sex	1990				2019				1990-2019 percent change (%)			
			DALYs		Death		DALYs		Death		DALYs		Death	
			Number	Rate	Number	Rate	Number	Rate	Number	Rate	Number	Rate	Number	Rate
	Maternal and neonatal disorders	Both	45541 (36858 to 54758)	6082.8 (4939.7 to 7298.7)	508 (410 to 612)	67.7 (54.7 to 81.5)	6121 (4838 to 7593)	1371.6 (1063.6 to 1717.8)	53 (39 to 69)	12.8 (9.5 to 16.6)	-86.6 (-90.1 to -82.5)	-77.5 (-83.5 to -70.5)	-89.5 (-92.7 to -85.7)	-81.1 (-86.9 to -74.1)
		Female	20167 (16155 to 24420)	5556.8 (4457.6 to 6725.4)	225 (180 to 273)	61.8 (49.4 to 75.1)	2738 (2144 to 3389)	1244.6 (950.2 to 1564.2)	23 (17 to 31)	11.5 (8.5 to 15.1)	-86.4 (-89.9 to -81.6)	-77.6 (-83.6 to -69.6)	-89.6 (-92.6 to -85.5)	-81.4 (-86.9 to -74.1)
		Male	25374 (20179 to 30779)	6581.5 (5238.1 to 7976.9)	283 (225 to 345)	73.3 (58.1 to 89.2)	3383 (2639 to 4228)	1491.5 (1143 to 1885.3)	30 (22 to 39)	14 (10.3 to 18.6)	-86.7 (-90.4 to -82.2)	-77.3 (-83.9 to -69.5)	-89.5 (-92.8 to -85.4)	-80.8 (-86.9 to -73.4)
	Maternal disorders	Female	251 (174 to 337)	130.8 (90.1 to 178.2)	3 (2 to 4)	1.6 (1 to 2.4)	79 (61 to 102)	20.8 (16.1 to 26.8)	1 (1 to 1)	0.2 (0.2 to 0.3)	-68.4 (-77.1 to -54.2)	-84.1 (-88.7 to -76.3)	-71.3 (-81.9 to -49.1)	-87.3 (-91.9 to -77.5)
	Neonatal disorders	Both	45290 (36538 to 54485)	6017.8 (4861.2 to 7229.7)	505 (406 to 610)	66.9 (53.8 to 80.7)	6042 (4759 to 7511)	1361.4 (1054.7 to 1709.7)	52 (39 to 68)	12.7 (9.4 to 16.5)	-86.7 (-90.1 to -82.6)	-77.4 (-83.5 to -70.3)	-89.6 (-92.8 to -85.8)	-81 (-86.8 to -74)
		Female	19916 (15877 to 24177)	5425.9 (4335.1 to 6582.3)	222 (177 to 270)	60.2 (48.1 to 73.3)	2658 (2057 to 3305)	1223.8 (931.9 to 1543.9)	23 (17 to 30)	11.3 (8.2 to 14.9)	-86.7 (-90.2 to -81.8)	-77.4 (-83.6 to -69.3)	-89.8 (-92.9 to -85.8)	-81.3 (-86.9 to -73.8)
		Male	25374 (20179 to 30779)	6581.5 (5238.1 to 7976.9)	283 (225 to 345)	73.3 (58.1 to 89.2)	3383 (2639 to 4228)	1491.5 (1143 to 1885.3)	30 (22 to 39)	14 (10.3 to 18.6)	-86.7 (-90.4 to -82.2)	-77.3 (-83.9 to -69.5)	-89.5 (-92.8 to -85.4)	-80.8 (-86.9 to -73.4)
	Nutritional deficiencies	Both	2497 (1792 to 3388)	432.5 (313.1 to 584.8)	11 (7 to 15)	2 (1.4 to 2.7)	826 (533 to 1247)	152.8 (99 to 232.9)	1 (1 to 1)	0.4 (0.3 to 0.4)	-66.9 (-76.5 to -54.3)	-64.7 (-73.8 to -53)	-88.1 (-92 to -81.3)	-82.4 (-87.1 to -73.8)
		Female	1168 (778 to 1687)	445.7 (306.4 to 637.7)	4 (2 to 7)	1.5 (0.9 to 2.3)	438 (262 to 687)	164.8 (101 to 260.8)	0 (0 to 0)	0.2 (0.2 to 0.3)	-62.5 (-75.9 to -43.5)	-63 (-74.4 to -49.1)	-91.7 (-95.2 to -85.5)	-85.6 (-91.5 to -75.7)
		Male	1329 (897 to 1961)	423.2 (292 to 605.2)	7 (4 to 10)	2.6 (1.6 to 3.5)	388 (233 to 641)	141.2 (84.1 to 235.8)	1 (1 to 1)	0.5 (0.4 to 0.6)	-70.8 (-81.3 to -55.2)	-66.6 (-78 to -48.3)	-85.9 (-91.3 to -74.6)	-81.2 (-87.1 to -67.8)
	Protein-energy malnutrition	Both	973 (685 to 1359)	140.4 (101.1 to 192.2)	10 (6 to 14)	1.7 (1.2 to 2.3)	176 (113 to 261)	32.5 (21.3 to 47.4)	1 (0 to 1)	0.1 (0.1 to 0.2)	-82 (-89.3 to -70.7)	-76.9 (-85.7 to -64.2)	-94.6 (-96.5 to -91.3)	-91.2 (-93.8 to -87)
		Female	399 (251 to 624)	116 (74.9 to 176.8)	4 (2 to 6)	1.2 (0.7 to 2)	80 (48 to 125)	30.1 (18.4 to 45.8)	0 (0 to 0)	0.1 (0.1 to 0.1)	-79.9 (-89.2 to -64.2)	-74.1 (-85.8 to -55.7)	-96.8 (-98.2 to -94.1)	-93.8 (-96.4 to -89)
		Male	574 (364 to 868)	163.2 (108.7 to 242.3)	6 (3 to 9)	2.1 (1.3 to 3.1)	96 (62 to 142)	34.7 (23.1 to 50.3)	0 (0 to 1)	0.2 (0.2 to 0.3)	-83.3 (-90.7 to -70.4)	-78.7 (-87.4 to -63.5)	-93.2 (-95.9 to -87.9)	-90.2 (-93.6 to -83.6)
	Iodine deficiency	Both	65 (40 to 97)	16.7 (10.6 to 24.6)	--	--	43 (24 to 69)	6.8 (4 to 11)	--	--	-34.9 (-54.2 to -14.6)	-59.3 (-69.8 to -48.7)	--	--
		Female	40 (24 to 59)	20.9 (13.4 to 30.7)	--	--	27 (15 to 44)	8.6 (4.9 to 14.1)	--	--	-33.2 (-54.8 to -8.1)	-58.9 (-71.1 to -45.6)	--	--
		Male	26 (15 to 39)	12.9 (8.1 to 19.1)	--	--	16 (9 to 26)	5.1 (3 to 8.2)	--	--	-37.4 (-55.8 to -15.7)	-60.5 (-71 to -49.6)	--	--
	Vitamin A deficiency	Both	120 (61 to 206)	15.4 (7.8 to 26.2)	--	--	3 (1 to 7)	0.7 (0.3 to 1.5)	--	--	-97.4 (-99 to -94.1)	-95.6 (-98.2 to -90.1)	--	--
		Female	44 (21 to 83)	11.5 (5.6 to 21.6)	--	--	1 (0 to 3)	0.6 (0.2 to 1.4)	--	--	-96.9 (-99 to -92.7)	-94.8 (-98.2 to -87.6)	--	--
		Male	77 (31 to 147)	19 (7.8 to 36.2)	--	--	2 (0 to 6)	0.7 (0.2 to 2.3)	--	--	-97.7 (-99.4 to -92.5)	-96.1 (-99 to -87.4)	--	--
	Dietary iron deficiency	Both	1233 (696 to 1953)	239.2 (147 to 360)	--	--	534 (306 to 906)	100.4 (56.7 to 170.3)	--	--	-56.7 (-73.1 to -31)	-58 (-72 to -36.8)	--	--
		Female	642 (344 to 1054)	280.3 (166.3 to 430.2)	--	--	300 (157 to 521)	114.9 (62.2 to 199.7)	--	--	-53.3 (-73.3 to -20.2)	-59 (-74.9 to -37.2)	--	--
		Male	591 (290 to 1112)	204 (107.7 to 350.5)	--	--	234 (112 to 439)	86.7 (40.5 to 167.8)	--	--	-60.4 (-81.1 to -17.6)	-57.5 (-78.3 to -17.1)	--	--

Province	Cause	Sex	1990				2019				1990-2019 percent change (%)			
			DALYs		Death		DALYs		Death		DALYs		Death	
			Number	Rate	Number	Rate	Number	Rate	Number	Rate	Number	Rate	Number	Rate
	Other nutritional deficiencies	Both	105 (69 to 158)	20.7 (14.8 to 29.3)	1 (1 to 2)	0.3 (0.2 to 0.4)	70 (49 to 98)	12.4 (9 to 17)	1 (1 to 1)	0.2 (0.2 to 0.3)	-33.1 (-57.4 to 2.6)	-40.1 (-57.7 to -16)	-27.9 (-56.4 to 36.2)	-36.2 (-55.5 to 12.3)
		Female	43 (26 to 71)	16.9 (10.8 to 26.1)	0 (0 to 1)	0.2 (0.1 to 0.4)	30 (18 to 45)	10.7 (7 to 15.6)	0 (0 to 0)	0.1 (0.1 to 0.2)	-30.4 (-60.8 to 17.1)	-37 (-60.5 to -2.3)	-40.7 (-69.3 to 20.4)	-38.9 (-67.5 to 24.2)
		Male	62 (36 to 101)	24 (15.9 to 36.1)	1 (0 to 1)	0.4 (0.2 to 0.6)	40 (28 to 56)	14 (10.1 to 19)	1 (0 to 1)	0.3 (0.2 to 0.3)	-35.1 (-62.5 to 13.3)	-41.7 (-62.2 to -8)	-20.8 (-57.3 to 78.6)	-36.1 (-59.2 to 24.2)
	**Non-communicable diseases**	Both	79984 (66275 to 93486)	26825.3 (23195.5 to 30548.4)	1265 (1077 to 1453)	768.7 (676.5 to 862.4)	109207 (92729 to 127908)	20643.3 (17860.1 to 23779.4)	2207 (2028 to 2387)	581.2 (538.7 to 623)	36.5 (17.5 to 60.6)	-23 (-31.3 to -13)	74.5 (49.5 to 110.9)	-24.4 (-33.2 to -12.8)
		Female	37392 (30613 to 44081)	25776.5 (21823.6 to 29853.3)	497 (414 to 579)	664.7 (567.2 to 758.9)	54681 (45652 to 65566)	20627.3 (17627.8 to 24201.8)	910 (816 to 1018)	516.6 (472.4 to 569.8)	46.2 (24.6 to 72.4)	-20 (-29.4 to -9.1)	83.2 (50.9 to 129.7)	-22.3 (-34.3 to -5.7)
		Male	42592 (34640 to 50487)	27420.8 (23253.8 to 31893.8)	768 (631 to 930)	850.5 (716.3 to 1012.3)	54526 (46448 to 63228)	20575.6 (17714.4 to 23572.5)	1297 (1142 to 1462)	634.8 (562.6 to 710.3)	28 (6 to 57.8)	-25 (-36.4 to -11.6)	68.9 (34.6 to 114.5)	-25.4 (-38.8 to -7.8)
	Neoplasms	Both	6371 (4947 to 7505)	2763.7 (2205.8 to 3272.3)	185 (148 to 218)	111.2 (89.5 to 132.1)	13874 (11806 to 15610)	2684 (2296.9 to 3013.1)	497 (429 to 558)	113.7 (97.9 to 127.8)	117.8 (79.1 to 191.1)	-2.9 (-19.5 to 29)	169.1 (123.1 to 256.3)	2.2 (-14.9 to 33.5)
		Female	2562 (1841 to 3197)	2318.8 (1739 to 2922.4)	67 (50 to 84)	88.1 (65.9 to 111.9)	6143 (5244 to 7059)	2315.9 (1976.3 to 2664.9)	204 (174 to 236)	91.4 (78 to 104.9)	139.8 (83.7 to 239.6)	-0.1 (-25 to 37.3)	202.5 (127.2 to 320.6)	3.7 (-23.5 to 39.9)
		Male	3810 (2827 to 4875)	3087.1 (2298.6 to 3921.6)	117 (87 to 149)	129.3 (97.3 to 163.4)	7732 (6277 to 9048)	3016.4 (2468.3 to 3518.9)	293 (244 to 342)	132.5 (110.4 to 154.1)	103 (52.4 to 195.6)	-2.3 (-25.6 to 40.9)	149.9 (90.5 to 260.1)	2.5 (-20.6 to 45.8)
	Lip and oral cavity cancer	Both	28 (20 to 36)	14.6 (10.4 to 19.1)	1 (1 to 1)	0.6 (0.4 to 0.8)	82 (70 to 95)	15.8 (13.6 to 18.3)	3 (3 to 4)	0.7 (0.6 to 0.8)	193.1 (109.5 to 354.5)	8 (-22.5 to 65.4)	215.1 (125.4 to 388.6)	13.1 (-18.1 to 75)
		Female	9 (6 to 11)	10 (7 to 13.3)	0 (0 to 0)	0.4 (0.3 to 0.6)	37 (31 to 44)	14.6 (12.2 to 17.3)	1 (1 to 2)	0.6 (0.5 to 0.8)	326.9 (197.3 to 563.5)	45.3 (0.6 to 126.9)	374.6 (228.2 to 639.5)	53.8 (5.8 to 135.6)
		Male	19 (12 to 27)	18 (11.9 to 25.3)	1 (0 to 1)	0.8 (0.5 to 1.1)	44 (36 to 56)	16.9 (13.6 to 20.9)	2 (1 to 2)	0.7 (0.6 to 0.9)	132 (45.6 to 293.4)	-6.3 (-40.2 to 57.2)	147.9 (56.7 to 324.7)	-3.4 (-36.8 to 62.4)
	Nasopharynx cancer	Both	26 (19 to 32)	10.8 (8.1 to 13.2)	1 (1 to 1)	0.4 (0.3 to 0.5)	41 (35 to 48)	7.4 (6.3 to 8.5)	1 (1 to 1)	0.3 (0.2 to 0.3)	60.6 (21.5 to 135.2)	-31.8 (-47.5 to -1.1)	77.4 (36.9 to 155.7)	-32.1 (-49.2 to -5.8)
		Female	12 (7 to 16)	11.2 (7.3 to 15.6)	0 (0 to 0)	0.4 (0.3 to 0.6)	19 (15 to 23)	7.1 (5.7 to 8.6)	1 (0 to 1)	0.2 (0.2 to 0.3)	56.7 (8.2 to 165.5)	-37.3 (-58.4 to 0)	76.5 (16.1 to 179.3)	-38.1 (-62.5 to -1.3)
		Male	14 (9 to 18)	10.4 (7.5 to 14.1)	0 (0 to 1)	0.4 (0.3 to 0.5)	22 (18 to 27)	7.7 (6.2 to 9.5)	1 (1 to 1)	0.3 (0.2 to 0.3)	64.2 (11.1 to 158.7)	-26.4 (-49.9 to 13.2)	78.2 (22 to 174)	-26.2 (-48.7 to 10.8)
	Other pharynx cancer	Both	11 (8 to 14)	5.7 (4.2 to 7.3)	0 (0 to 0)	0.2 (0.2 to 0.3)	25 (21 to 30)	4.8 (4.1 to 5.7)	1 (1 to 1)	0.2 (0.2 to 0.3)	137 (72.3 to 255.4)	-15 (-37.6 to 25.8)	152.2 (84.5 to 270.6)	-10.9 (-34.1 to 30.1)
		Female	4 (3 to 6)	5 (3.3 to 6.9)	0 (0 to 0)	0.2 (0.1 to 0.3)	10 (8 to 12)	3.7 (3 to 4.5)	0 (0 to 0)	0.2 (0.1 to 0.2)	127.9 (58.8 to 275.9)	-26.5 (-51.5 to 20.8)	143.3 (59.7 to 298.7)	-23.7 (-53.1 to 28.4)
		Male	6 (4 to 9)	6.2 (4.2 to 8.6)	0 (0 to 0)	0.3 (0.2 to 0.4)	16 (12 to 20)	6 (4.7 to 7.6)	1 (0 to 1)	0.3 (0.2 to 0.3)	143 (56.6 to 301.5)	-2.6 (-36.7 to 57.7)	157.6 (65.7 to 312.8)	1.4 (-33.4 to 60.4)
	Esophageal cancer	Both	381 (151 to 520)	220.5 (87.5 to 306.5)	14 (6 to 20)	10 (3.9 to 14.7)	873 (411 to 1058)	178.1 (83.2 to 216.3)	36 (16 to 43)	8.6 (3.9 to 10.4)	129.4 (68.9 to 236.2)	-19.2 (-40.4 to 16.5)	147.4 (80.7 to 256.8)	-14.4 (-40.1 to 19.7)

Province	Cause	Sex	1990				2019				1990-2019 percent change (%)			
			DALYs		Death		DALYs		Death		DALYs		Death	
			Number	Rate	Number	Rate	Number	Rate	Number	Rate	Number	Rate	Number	Rate
		Female	153 (56 to 237)	205.8 (75.9 to 338.3)	6 (2 to 10)	9.3 (3.4 to 16.8)	340 (151 to 433)	139.6 (61.7 to 176.6)	13 (6 to 17)	6.6 (2.8 to 8.3)	122.4 (46.6 to 259.4)	-32.2 (-57.9 to 7.3)	134.2 (44 to 268.8)	-29.6 (-60.1 to 11.2)
		Male	228 (86 to 328)	231.1 (86.3 to 327.8)	9 (3 to 12)	10.5 (3.9 to 14.7)	533 (237 to 682)	214.1 (95.7 to 273.5)	22 (10 to 29)	10.3 (4.5 to 13.2)	134.1 (59.8 to 289.7)	-7.3 (-35.8 to 49.1)	156.1 (79.1 to 309.5)	-2.1 (-30.4 to 52.5)
	Stomach cancer	Both	1418 (1045 to 1742)	790.9 (604.4 to 961.1)	52 (40 to 63)	35.1 (27.5 to 42.8)	2565 (2221 to 2937)	515.4 (445.1 to 586.3)	104 (90 to 118)	24.5 (21.4 to 27.7)	80.9 (41.1 to 160.5)	-34.8 (-47.9 to -9.5)	99.3 (58.6 to 176.8)	-30.1 (-44.2 to -8.5)
		Female	513 (354 to 694)	624.1 (455.5 to 849.9)	18 (13 to 24)	26.9 (19.8 to 39.8)	1056 (857 to 1255)	418 (341.6 to 497.2)	40 (33 to 48)	19.1 (15.8 to 22.7)	105.8 (39.1 to 209.1)	-33 (-54.3 to -3.2)	127.8 (56.1 to 225.8)	-28.9 (-53.5 to -0.4)
		Male	905 (596 to 1199)	908.7 (613.2 to 1179.7)	34 (23 to 45)	41.2 (28.6 to 52.4)	1509 (1240 to 1836)	606.6 (502.5 to 731.5)	64 (53 to 76)	29.2 (24.4 to 34.7)	66.8 (19.9 to 158.7)	-33.2 (-51.1 to 1.3)	84.8 (35.9 to 184.6)	-29.2 (-46.9 to 9.1)
	Colon and rectum cancer	Both	245 (177 to 323)	132.8 (96.9 to 172.5)	9 (6 to 11)	5.7 (4.2 to 7.4)	1076 (920 to 1248)	208.2 (177.1 to 241.7)	40 (34 to 47)	9.2 (7.9 to 10.8)	338.5 (209.7 to 549.4)	56.8 (11.4 to 130.2)	369.4 (234.5 to 584.9)	62.6 (15.3 to 137.6)
		Female	99 (65 to 140)	121.3 (81.3 to 168.1)	3 (2 to 5)	5.2 (3.6 to 7)	481 (379 to 578)	188.5 (150.4 to 227.8)	18 (14 to 21)	8.4 (6.7 to 10.2)	385.3 (205.4 to 691.7)	55.4 (-3.2 to 151.9)	423.6 (226.5 to 745.4)	61.3 (2.5 to 153.2)
		Male	146 (95 to 207)	140.7 (91.9 to 197.5)	5 (3 to 7)	6 (4 to 8.4)	595 (487 to 727)	226.5 (185.5 to 277.5)	22 (19 to 27)	10 (8.2 to 12.2)	306.8 (162 to 573)	61 (5.4 to 161.8)	334.1 (184.9 to 605.9)	65.4 (10.3 to 165.3)
	Liver cancer	Both	286 (210 to 365)	147.3 (105.6 to 192.8)	10 (7 to 13)	6.5 (4.6 to 8.6)	564 (476 to 663)	111.8 (95.5 to 130.5)	22 (18 to 25)	5 (4.3 to 5.8)	97.3 (44.2 to 193.4)	-24.1 (-45.9 to 15.6)	120.2 (57.6 to 234.1)	-23 (-45.9 to 16.3)
		Female	116 (77 to 179)	133.5 (86.3 to 222.3)	4 (2 to 6)	5.9 (3.7 to 10.3)	226 (181 to 278)	88.2 (71.3 to 107.9)	8 (7 to 10)	3.8 (3 to 4.6)	94.7 (11.4 to 223.4)	-33.9 (-64.7 to 11.7)	113.5 (15.1 to 262.3)	-36.6 (-67.8 to 12)
		Male	170 (118 to 229)	156.3 (110.3 to 209)	6 (4 to 8)	6.9 (4.9 to 9.2)	338 (269 to 421)	134.2 (107.9 to 166)	13 (11 to 17)	6.1 (5 to 7.4)	99.1 (34.2 to 213.6)	-14.1 (-41.2 to 33.5)	124.5 (52.6 to 257)	-11.5 (-39 to 33.4)
	Gallbladder and biliary tract cancer	Both	47 (35 to 59)	27.6 (21 to 35.1)	2 (1 to 2)	1.3 (1 to 1.6)	160 (103 to 193)	32.6 (21.6 to 38.8)	7 (4 to 8)	1.5 (1.1 to 1.8)	244.7 (116.4 to 433)	18.2 (-23.2 to 71.7)	259.2 (135.8 to 421.2)	21.5 (-18.8 to 66.6)
		Female	21 (14 to 30)	29.7 (20.8 to 43.3)	1 (1 to 1)	1.4 (1 to 2.1)	76 (49 to 95)	31.5 (20.9 to 39)	3 (2 to 4)	1.5 (1 to 1.8)	263.6 (126.2 to 490.6)	6.1 (-33.6 to 64.4)	268 (130.1 to 464.3)	7.3 (-32.1 to 61.4)
		Male	26 (17 to 35)	26 (18.1 to 35.1)	1 (1 to 1)	1.2 (0.9 to 1.6)	84 (46 to 108)	33.3 (18.9 to 42.5)	3 (2 to 4)	1.6 (0.9 to 2)	229.2 (88.9 to 449.1)	27.9 (-25.7 to 107.2)	251.8 (109.1 to 464.3)	33.1 (-21.3 to 111)
	Pancreatic cancer	Both	66 (43 to 93)	38 (24.9 to 52.4)	3 (2 to 3)	1.7 (1.1 to 2.3)	548 (476 to 633)	110.3 (96 to 126.6)	22 (19 to 25)	5 (4.4 to 5.7)	724.4 (457.6 to 1248.6)	190.3 (97.2 to 367)	767.4 (486.5 to 1307)	200.6 (104.1 to 385.8)
		Female	21 (13 to 33)	28.2 (18.2 to 44.1)	1 (1 to 1)	1.2 (0.8 to 2)	233 (184 to 285)	95.6 (76.6 to 115.4)	9 (7 to 11)	4.4 (3.6 to 5.3)	1017.4 (522.7 to 1856.4)	239.4 (89.9 to 483.9)	1078.5 (556.5 to 1924.1)	253.6 (95.6 to 488.2)
		Male	46 (27 to 68)	45.4 (27.4 to 67.6)	2 (1 to 3)	2 (1.2 to 2.9)	315 (258 to 387)	124.1 (101.9 to 151.9)	12 (10 to 15)	5.6 (4.6 to 6.8)	590.3 (324.4 to 1137.7)	173.4 (70.3 to 391.7)	625.5 (350.1 to 1226.1)	178.3 (75.2 to 404.3)
	Larynx cancer	Both	154 (115 to 195)	84.9 (63.2 to 105.5)	5 (4 to 7)	3.5 (2.7 to 4.3)	293 (241 to 354)	57.7 (48.3 to 69.4)	11 (9 to 13)	2.5 (2.1 to 3)	90.1 (41.9 to 184.1)	-32 (-48.4 to 0.9)	102.6 (53.1 to 201.7)	-28.1 (-44.8 to 4.8)
		Female	35 (24 to 47)	41.9 (29.5 to 54.7)	1 (1 to 1)	1.6 (1.1 to 2.1)	74 (60 to 92)	28.7 (23.2 to 35.2)	3 (2 to 3)	1.2 (1 to 1.5)	110.1 (48.6 to 235)	-31.6 (-50.8 to 4.6)	131.4 (64.7 to 259.4)	-26.3 (-46.5 to 15.3)

Province	Cause	Sex	1990				2019				1990-2019 percent change (%)			
			DALYs		Death		DALYs		Death		DALYs		Death	
			Number	Rate	Number	Rate	Number	Rate	Number	Rate	Number	Rate	Number	Rate
		Male	119 (80 to 157)	116.9 (81.1 to 151.6)	4 (3 to 6)	4.9 (3.6 to 6.3)	219 (171 to 273)	86.2 (68.3 to 107.4)	8 (7 to 10)	3.8 (3 to 4.6)	84.2 (27.9 to 194.7)	-26.3 (-48.1 to 16.6)	95 (37.5 to 205)	-24.2 (-45.9 to 16.6)
	Tracheal, bronchus, and lung cancer	Both	468 (338 to 623)	255.7 (187.2 to 342.8)	17 (12 to 22)	11 (8.1 to 14.6)	1549 (1315 to 1808)	305.9 (261.9 to 351.5)	60 (52 to 69)	13.8 (12 to 16)	231.2 (128.1 to 393.7)	19.6 (-17.3 to 75.7)	256 (145.4 to 424)	25.8 (-12.4 to 82.5)
		Female	90 (59 to 136)	105 (68 to 158.5)	3 (2 to 4)	4.3 (2.9 to 6.7)	522 (419 to 628)	201.2 (160.9 to 240.2)	19 (15 to 23)	8.7 (6.9 to 10.2)	483.2 (219.2 to 893.6)	91.6 (5.4 to 213.6)	543.9 (253.5 to 953)	100.8 (10.1 to 219.4)
		Male	378 (259 to 518)	368.5 (256.6 to 502.7)	14 (10 to 19)	16.1 (11.6 to 21.8)	1027 (841 to 1252)	407.5 (334.4 to 496.7)	41 (34 to 49)	18.6 (15.4 to 22.3)	171.6 (77.5 to 335.5)	10.6 (-27.6 to 74.4)	195.5 (95.2 to 361.3)	15 (-23.6 to 76.8)
	Malignant skin melanoma	Both	52 (20 to 82)	25.4 (9.5 to 39.6)	2 (1 to 3)	1.1 (0.4 to 1.8)	80 (48 to 103)	15 (8.6 to 19.5)	3 (2 to 4)	0.6 (0.3 to 0.9)	52.8 (0 to 198.7)	-41.1 (-59.6 to 11.3)	64.7 (15.5 to 206)	-42.7 (-59 to 4)
		Female	22 (7 to 37)	22.4 (7.3 to 36.3)	1 (0 to 1)	1 (0.3 to 1.6)	35 (17 to 48)	12.8 (6.3 to 17.8)	1 (1 to 2)	0.5 (0.2 to 0.7)	58.3 (3 to 222.9)	-42.7 (-60.1 to 7)	70.4 (20 to 214.5)	-45.1 (-62.3 to -6)
		Male	30 (11 to 49)	27.7 (9.6 to 45.9)	1 (0 to 2)	1.3 (0.4 to 2.2)	45 (24 to 60)	16.9 (8.7 to 22.8)	2 (1 to 2)	0.8 (0.3 to 1)	48.8 (-11.1 to 206.5)	-38.9 (-63.5 to 27.1)	61.1 (-0.7 to 224.2)	-41.2 (-63.2 to 25.6)
	Non-melanoma skin cancer	Both	10 (7 to 13)	5.8 (4.1 to 7.5)	0 (0 to 1)	0.3 (0.2 to 0.4)	45 (39 to 52)	9.5 (8.2 to 10.8)	2 (2 to 2)	0.5 (0.5 to 0.6)	368.4 (242.3 to 593.6)	62.8 (20 to 143)	409 (272.1 to 670.2)	77.6 (32 to 173.4)
		Female	1 (1 to 2)	1.8 (1 to 2.6)	0 (0 to 0)	0.1 (0.1 to 0.1)	8 (7 to 10)	3.5 (2.8 to 4.2)	0 (0 to 0)	0.2 (0.1 to 0.2)	533.5 (300.8 to 1033.3)	96.8 (21.6 to 275.2)	531.5 (287.4 to 1143)	98 (19.1 to 311.9)
		Male	8 (6 to 11)	9 (6.4 to 11.9)	0 (0 to 0)	0.5 (0.3 to 0.6)	37 (31 to 43)	15 (12.5 to 17.7)	2 (1 to 2)	0.8 (0.7 to 1)	342.2 (213.1 to 557.1)	67.6 (20.6 to 151.5)	389.9 (252.4 to 638)	77.2 (28 to 166.6)
	Breast cancer	Both	225 (144 to 329)	111.9 (71.7 to 167.2)	6 (4 to 10)	3.7 (2.4 to 5.8)	1036 (860 to 1245)	174.4 (143.7 to 211.2)	30 (24 to 36)	5.7 (4.7 to 6.9)	361.1 (189.5 to 645.1)	55.9 (-4.5 to 154.6)	357.4 (173.6 to 658.1)	53.1 (-12.7 to 161.5)
		Female	220 (140 to 324)	246.4 (154.9 to 373.2)	6 (4 to 10)	8.3 (5.1 to 13.2)	1023 (847 to 1234)	344.5 (284 to 417.6)	29 (24 to 36)	11.3 (9.3 to 13.7)	364.8 (188.1 to 657.6)	39.8 (-15.7 to 130.6)	361.4 (173.2 to 671.7)	37.1 (-22.8 to 137.1)
		Male	5 (3 to 7)	4.1 (2.5 to 6.4)	0 (0 to 0)	0.2 (0.1 to 0.2)	13 (9 to 18)	4.7 (3.4 to 6.4)	0 (0 to 1)	0.2 (0.1 to 0.2)	180.3 (59.9 to 417.2)	13.2 (-34.9 to 110)	186.8 (61.3 to 428.4)	11.4 (-35.9 to 104.5)
	Cervical cancer	Female	79 (47 to 109)	84.4 (54.5 to 119.1)	2 (1 to 3)	3 (2 to 4.4)	166 (121 to 208)	58.9 (43.4 to 73.1)	5 (4 to 7)	2.2 (1.6 to 2.7)	110.8 (45.1 to 258)	-30.2 (-53.9 to 11.4)	133.1 (52.8 to 270.6)	-25.6 (-53.7 to 12.8)
	Uterine cancer	Female	10 (6 to 17)	12.6 (7.7 to 23)	0 (0 to 1)	0.5 (0.3 to 1)	50 (36 to 63)	18.8 (14.2 to 24)	2 (1 to 2)	0.7 (0.5 to 0.9)	390.5 (169.1 to 754.8)	48.5 (-20.9 to 159)	382.9 (162.4 to 748.2)	42.1 (-26.3 to 148.7)
	Ovarian cancer	Female	32 (17 to 63)	34.3 (18.1 to 66.8)	1 (0 to 2)	1.2 (0.6 to 2.2)	230 (167 to 286)	82.1 (60.2 to 102.3)	7 (5 to 9)	2.9 (2.2 to 3.7)	613 (203.8 to 1369)	139 (2.8 to 393.1)	690.6 (234.3 to 1534.3)	152.6 (8 to 416.9)
	Prostate cancer	Male	125 (83 to 176)	165.8 (110.7 to 228.7)	6 (4 to 9)	10.2 (6.8 to 14.1)	459 (345 to 554)	220.5 (168.3 to 265.9)	26 (20 to 31)	13.8 (10.6 to 16.9)	266.3 (156.1 to 473.4)	33 (-5.6 to 105.3)	307.3 (188.2 to 530.1)	35.1 (-2.8 to 110)
	Testicular cancer	Male	2 (1 to 3)	0.8 (0.5 to 1)	0 (0 to 0)	0 (0 to 0)	32 (23 to 42)	9.7 (7.1 to 12.7)	1 (0 to 1)	0.2 (0.1 to 0.3)	1479.3 (875.9 to 2501.9)	1194.8 (723.7 to 1979)	1846.2 (1208.9 to 2959.5)	1234.7 (816 to 2025.7)

Province	Cause	Sex	1990				2019				1990-2019 percent change (%)			
			DALYs		Death		DALYs		Death		DALYs		Death	
			Number	Rate	Number	Rate	Number	Rate	Number	Rate	Number	Rate	Number	Rate
	Kidney cancer	Both	82 (53 to 109)	25.6 (15.4 to 33.4)	2 (1 to 2)	0.9 (0.5 to 1.2)	211 (175 to 246)	40.9 (33.9 to 47.4)	7 (6 to 9)	1.6 (1.3 to 1.9)	158.6 (74 to 336.6)	59.5 (11.5 to 191)	301.1 (177.4 to 635.2)	79.4 (24.8 to 263.1)
		Female	34 (23 to 48)	21.1 (12.3 to 28)	1 (0 to 1)	0.7 (0.4 to 1)	71 (58 to 85)	27.5 (22.6 to 32.7)	2 (2 to 3)	1 (0.8 to 1.2)	108.3 (32.7 to 240.4)	30.2 (-10.1 to 139.4)	220.9 (118.5 to 492.7)	33.8 (-11 to 220.2)
[truncated: 736,280 more chars]
